# Single-cell transcriptome analysis reveals cellular heterogeneity in the ascending aortas of normal and high-fat diet-fed mice

**DOI:** 10.1038/s12276-021-00671-2

**Published:** 2021-09-21

**Authors:** Hao Kan, Ka Zhang, Aiqin Mao, Li Geng, Mengru Gao, Lei Feng, Qingjun You, Xin Ma

**Affiliations:** 1 Wuxi School of Medicine, Jiangnan University, Wuxi, Jiangsu China; 2 Department of Thoracic Surgery, The Affiliated Hospital of Jiangnan University, Wuxi, Jiangsu China

**Keywords:** Transcriptomics, Aortic diseases

## Abstract

The aorta contains numerous cell types that contribute to vascular inflammation and thus the progression of aortic diseases. However, the heterogeneity and cellular composition of the ascending aorta in the setting of a high-fat diet (HFD) have not been fully assessed. We performed single-cell RNA sequencing on ascending aortas from mice fed a normal diet and mice fed a HFD. Unsupervised cluster analysis of the transcriptional profiles from 24,001 aortic cells identified 27 clusters representing 10 cell types: endothelial cells (ECs), fibroblasts, vascular smooth muscle cells (SMCs), immune cells (B cells, T cells, macrophages, and dendritic cells), mesothelial cells, pericytes, and neural cells. After HFD intake, subpopulations of endothelial cells with lipid transport and angiogenesis capacity and extensive expression of contractile genes were defined. In the HFD group, three major SMC subpopulations showed increased expression of extracellular matrix-degradation genes, and a synthetic SMC subcluster was proportionally increased. This increase was accompanied by upregulation of proinflammatory genes. Under HFD conditions, aortic-resident macrophage numbers were increased, and blood-derived macrophages showed the strongest expression of proinflammatory cytokines. Our study elucidates the nature and range of the cellular composition of the ascending aorta and increases understanding of the development and progression of aortic inflammatory disease.

## Cardiovascular disease: High-fat diets cause changes to aorta cells

RNA sequencing of aorta cells reveals how key changes in cell populations and gene expression caused by a high-fat diet can increase risk of cardiovascular diseases. Xin Ma and co-workers at Jiangnan University in Wuxi, China, performed single-cell sequencing on over 24,000 cells from the ascending aorta of mice fed either high-fat or normal diets. The resulting dataset of around 1,700 genes and 4,000 unique molecular identifiers reveals numerous fat-induced differences across the ten main aortic cell types. The high-fat diet extended specific sub-populations of endothelial cells that enhance lipid transport, while muscle cells showed increased expression of genes that degrade the extracellular matrix. Other fat-induced differences, including increased populations of immune cells known as macrophages and a sub-cluster of muscle cells that up-regulate pro-inflammatory genes, may act to promote excessive immune responses in obese individuals.

## Introduction

Consumption of high-fat diets (HFDs) like the Western diet is one of the important factors leading to a high rate of obesity^1^. In association with insulin resistance, hypertension, and dyslipidemia, obesity contributes to metabolic syndrome, a hallmark of cardiovascular risk^2^. Adipose tissue volume is increased in the aortas of obese mice and humans, and this increase is accompanied by an increase in inflammatory cytokines and oxidative stress. Under adverse conditions, increased aortic stiffness, plaque formation, and vascular dysfunction lead to aortic disease^3^. Although the main pathological features of aortic disease include extracellular matrix (ECM) degradation^4^, smooth muscle cell (SMC) loss^5^, and immune cell infiltration and activation^6^, the molecular and cellular processes that lead to aortic disease in HFD-induced obesity remain poorly understood.

The main cell types in the entire aorta are well known, and their heterogeneity is critical for aortic wall function^7,8^. However, the heterogeneity and relative contributions of different vascular cells in healthy and HFD aortas are poorly understood. Recently, the application and progress of scRNA-seq has provided a powerful tool for characterizing gene expression in individual cells. The transcriptional landscapes of ECs^7^, SMCs^9^, macrophages^8^, lymphocytes^10^, and adventitial cells^11^ in the aorta have been depicted by scRNA-seq data. Most studies have isolated individual cells from the thoracic aorta or the entire aorta and demonstrated the existence of complex cell populations and regulatory relationships among genes.

In this study, we characterized the cellular heterogeneity and diverse functional states within the wall of the ascending aorta in healthy and diseased mice using scRNA-seq to better understand the etiology and progression of aortic disease in HFD-induced obesity. Here, we describe integrative and differential analyses of lineage heterogeneity, functional status, and transcriptomic profiles of vascular cells from the ascending aortic wall in healthy and HFD-fed mice. By cluster analysis, we identified 27 clusters and 10 distinct cell types. Importantly, compared with healthy aortas, HFD-fed mouse aortas showed changes in cellular subpopulations, transcriptome characteristics, and biological functions.

## Materials and methods

### Animals

All animal experiments were conducted using protocols approved by the Animal Care Committee of Jiangnan University. Adult male C57BL/6 J mice at 6 weeks of age (Shanghai Laboratory Animal Co., Ltd., China) were fed a 60% fat diet (TP 2330055 A, Nantong Trofe Feed Technology Co., Ltd., China) or standard chow (11% fat) for 12 weeks^12,13^. Food and water were provided *ad libitum*. The mice were housed under specific pathogen-free conditions at 22–24 °C and on a 12-h/12-h light/dark cycle.

### Aortic dissociation and single cell preparation

Mice were euthanized by CO_2_ inhalation. The ascending aorta was collected after left ventricular perfusion with 10 mL of phosphate-buffered saline (PBS) and quickly transferred to cold PBS. A single-cell suspension of ascending aortic cells was prepared with a method consistent with a previous protocol^7,14^. In brief, after removing the perivascular adipose tissue, ascending aortas from the two groups were cut into ~1 mm pieces and digested with an enzyme solution (0.2% type I collagenase [Worthington Biochemical Corp., Lakewood, NJ] and 200 U/ml DNase [Sigma, USA]) for 20 min at 37 °C. The cell suspension was strained through a 40-μm filter (STEMCELL Technologies China Co., Ltd., Shanghai) and washed twice with PBS. The cells were resuspended in PBS with 0.04% bovine serum albumin, and their viability was > 80%. The resuspended cells were then used for sequencing.

### Single-cell RNA sequencing

Qualified aortic cell suspensions were loaded onto a Single-Cell Instrument (10x Genomics GemCode Technology, CA) to generate single-cell Gel beads in EMulsion (GEMs, Single Cell 3′ Library and Gel Bead Kit V2 [10x Genomics]). The sequencing protocols have been described in a previous report^15^. Briefly, the single-cell suspension, reagents, gel beads, and partitioning oil were loaded onto 10x Chromium Chip B. Single-cell RNA was barcoded through reverse transcription in individual GEMs. Next, all cDNAs were pooled, and a general library was constructed. Finally, the library was sent for sequencing. The accession number for the RNA-seq data reported here is Single Cell Portal number SCP1361.

### Single-cell RNA data analysis

The raw scRNA-seq data were processed using Cell Ranger software 3.0 (10x Genomics). The FASTQ data were produced using the FASTX-Toolkit, and then the reads were mapped to the prebuilt mouse mm10 transcriptome. The scRNA-seq data were filtered by the R package Seurat v3.0 with the following settings: a gene count per cell > 200 and < 5000, a percentage of mitochondrial genes < 10%, and no hemoglobin subunit beta gene detected in the cell^16^. The data were then normalized and programmed as scalar data and were subjected to principal component analysis, variable gene searching, cluster analysis, and t-distributed stochastic neighbor embedding (t-SNE) dimensional reduction. Gene expression was visualized with violin plots, dot plots, heatmaps, and t-SNE plots created with the Suerat functions VlnPlot, DotPlot, DoHeatmap, and FeaturePlot. Signature markers for each specific cluster were revealed by FindAllMarkers. Differentially expressed genes (*P* < 0.05) between two entities were obtained by FindMarkers in Seurat. Gene Ontology (GO) enrichment analysis was performed for the enriched genes found by the FindMarkers function with average log(fold change) values > 0.5 with the R package clusterProfiler.

### Ligand-receptor cellular communication analysis

Cell-cell communication was determined with the CellChat^17^ framework. Briefly, differentially expressed signaling genes (*P* < 0.05) were first identified across cell clusters in the scRNA-seq dataset to infer specific cellular communications. Next, signaling sources, influencers, targets, mediators, and high-order information were obtained by social network analysis. To evaluate ligand-receptor pairs, we used alluvial plots to show the associations of latent patterns with cell clusters and ligand-receptor pairs or signaling pathways. In addition, signaling networks for CXC chemokine ligands and CC chemokine ligands among ECs, SMCs, and macrophages were displayed with circle plots.

### Statistical analysis

After data normalization, dimensionality reduction, and clustering, the signature markers for each cluster were identified using the Wilcoxon rank-sum test in Seurat to determine whether the expression of a specific class of genes was altered in HFD aortas. GO enrichment analysis was performed with the enrichGO function, and the *P* values were computed with a hypergeometric test and adjusted for multiple hypothesis testing with the Benjamini-Hochberg procedure. The mouse body weights, serum lipid levels, macrophage infiltration in the aortic wall, and mRNA expression determined by RT–PCR are presented as the mean ± SEM. Statistical analysis was performed using GraphPad Prism 8.0. One-way variance analysis and Student’s t tests were used to analyze differences in data between different groups. *P* values of less than 0.05 were considered to indicate statistical significance.

## Results

### Single-cell RNA sequencing analysis of mouse ascending aortic walls

**Fig. 1:**
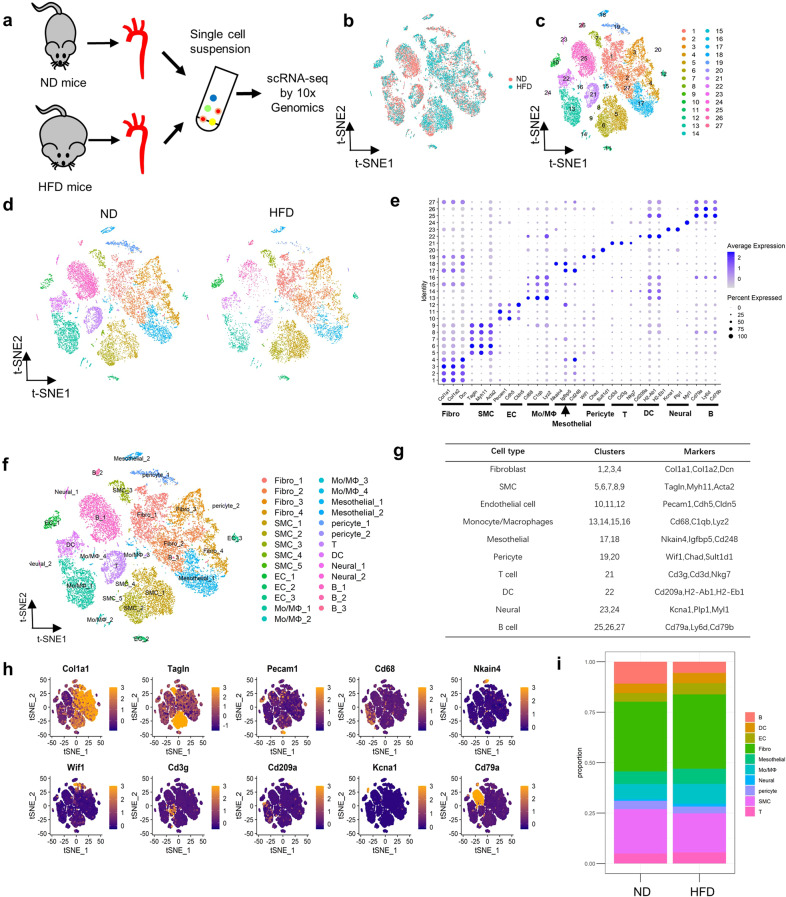
Identification of cell clusters in mouse ascending aortas by scRNA-seq. **a** Schematic diagram of the scRNA-seq program. ND, normal diet; HFD, high-fat diet. **b** t-SNE plot of aggregate cells from ND and HFD mice (after quality control, there were 14,663 cells in the ND group and 9338 in the HFD group). **c** t-SNE visualization of clustering revealing 27 cell clusters. **d** t-SNE plots of cell clusters across the indicated conditions. **e** Dot plot of conserved marker genes per cluster. Fibro, fibroblasts; SMC, smooth muscle cells; EC, endothelial cells; Mo/MΦ, monocytes/macrophages; T, T cells; DC, dendritic cells; Neural, neural cells; B, B cells. **f** t-SNE plot of cell clusters. **g** Major cell types and corresponding marker genes. **h** Relative expression of several marker genes in all cells from all samples. The cells are projected onto t-SNE plots. **i** Bar chart of the relative proportions of cell types from aggregated ascending aortas.

We profiled 24,001 ascending aortic cells from mice on a normal diet (ND) or a HFD (Fig. 1a and Supplementary Fig. S1). Overall, the sequencing yielded 1669 genes (median), and a 70.4% mean transcriptome mapping rate per cell was obtained with Cell Ranger. The median number of unique molecular identifiers per cell was 4063. After implementing quality control and filtering cells based on the number of reported genes (see methods), the individual transcriptional profiles of 14,663 cells for ND mice and 9,338 for HFD mice were included in the individual analysis (Supplementary Fig. 2). Subsequently, we applied unbiased clustering on 24,001 cells and identified 27 subpopulations, as shown by t-SNE (Fig. 1b–d and Supplementary Fig. 3).

After examining the known conserved marker genes in each cluster (Fig. 1e and h), we merged clusters with similar gene expression profiles and identified 10 major cell types from the integrated data. The 10 main cell types comprised (i) fibroblasts (Clusters 1–4), which were enriched with the expression of collagen proteins and small leucine-rich proteoglycan proteins, collagen type 1α1 (Col1a1), Col1a2, and Decorin; (ii) SMCs (Clusters 5–9), which strongly expressed the canonical SMC markers Tagln, Myh11, and Acta2; (iii) ECs (Clusters 10–12), which featured the expression of Pecam1, Cdh5, and Cldn5; (iv) monocytes and macrophages (Mo/MΦ cells, Clusters 13–16), which were marked by the expression of Cd68, C1qb, and Lyz2; (v) mesothelial cells (Clusters 17 and 18), which strongly expressed Nkain4, Igfpb5, and Cd248; (vi) pericytes (Clusters 19 and 20), which showed high levels of Wif1, Chad, and Sult1d1; (vii) T cells (Cluster 21), which strongly expressed Cd3g, Cd3d, and Nkg7; (viii) dendritic cells (DCs, Cluster 22), which demonstrated strong expression of Cd209a, H2-Ab1, and H2-Eb1; (ix) neural cells (Clusters 23 and 24), which strongly expressed Kcan1, Plp1, and Mly1; and (x) B cells (Clusters 25–27), which showed high levels of Cd79a, Ly6d, and Cd79b (Fig. 1f and g). These cells were also singled out in HFD-fed mouse aortic cells. Quantitatively, fibroblasts and SMCs were the largest subpopulations. Importantly, the proportion of monocytes and macrophages was increased in HFD aortas (Fig. 1i and Supplementary Table 1). A list of signature genes was identified for each cluster (Supplementary Table 2), and the top 5 marker genes for a cluster relative to all other clusters were defined (Supplementary Fig. 4).

### Three EC subpopulations exhibit gene expression profiles indicative of increased contractile gene expression in HFD mouse ascending aortas

**Fig. 2:**
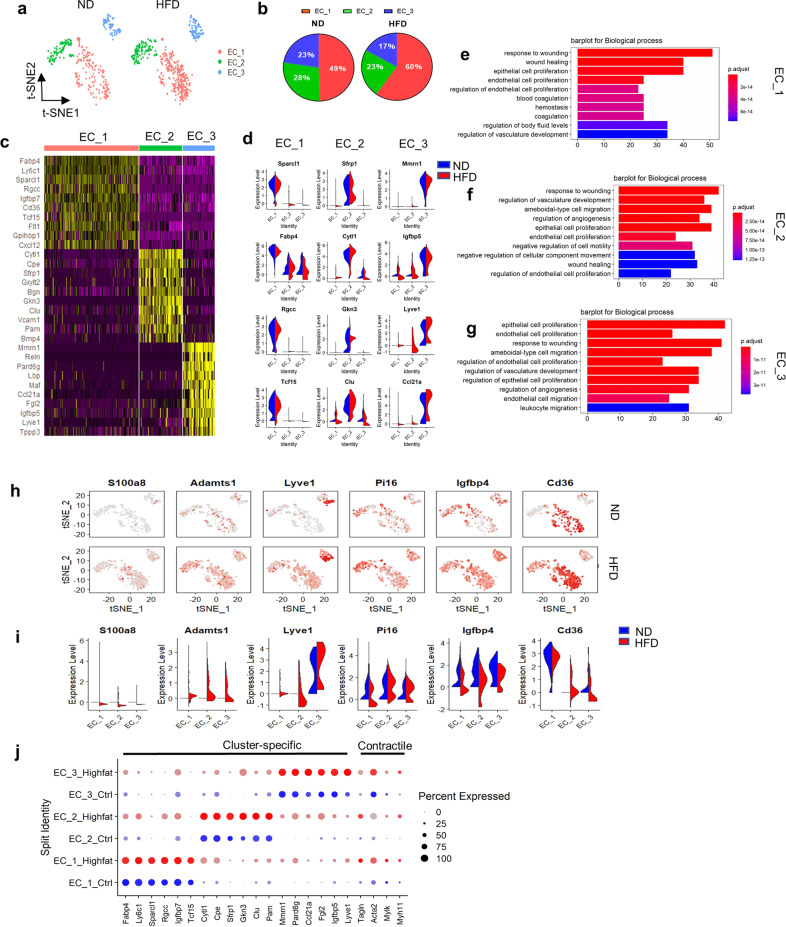
Comparison of EC subpopulations in ascending aortas from ND and HFD mice. **a** t-SNE plot of the EC subpopulations (EC_1, EC_2, and EC_3) from the ND (336 cells) and HFD (521 cells) groups. **b** Percentages of the EC subpopulations in the ND (EC_1, 49%; EC_2, 28%; and EC_3, 23%) and HFD (EC_1, 60%; EC_2, 23%; and EC_3, 17%) groups. **c** Heatmap of the top 10 marker genes per subpopulation. **d** Violin plots of signature genes confirming the subpopulation identities. **e**–**g** Top 10 pathways associated with the EC_1 (**e**), EC_2 (**f**), and EC_3 (**g**) clusters. **h** Feature plots of the expression of selected marker genes for the EC subpopulations from the ND and HFD groups. **i** Violin plots of the expression of selected marker genes for the EC subpopulations from the ND and HFD groups. **j** Expression of cluster-specific and contractile genes for EC subpopulations as visualized by dot plot.

After examining the cell populations determined by the conserved marker genes, we examined the subpopulations of cell types. Clustering analysis of all cells in both the ND and HFD mouse ascending aorta groups identified 3 distinct subpopulations (Cluster 10, EC_1; Cluster 11, EC_2; and Cluster 12, EC_3) of ECs (Fig. 2a). In the ND group, EC_1 accounted for the largest proportion (49%) of the EC population, and the other two clusters accounted for 51% (EC_2, 28%; EC_3, 23%). Of note, EC_1 was significantly increased in the HFD group, and EC_2 and EC_3 were both reduced by 5% (Fig. 2b).

To characterize these subpopulations, we identified the marker genes that differentiated each cluster (Fig. 2c, d and Supplementary Table 3). The largest population (EC_1) expressed genes involved in lipid transport (Fapb4, Cd36, and Gpihbp1), cell adhesion (Igfbp7 and Cxcl12), and an angiogenesis marker (Flt1). The second EC population (EC_2) was defined by strong expression of canonical markers (Vcam1 and Pecam1) with other genes involved in regulating EC function (Sfrp1, Clu, and Gxylt2). The EC_3 population strongly expressed Mmrn1, lymphatic vessel endothelial hyaluronic acid receptor 1 (Lyve1), and Ccl21a (log_2_FC > 3 *versus* the other subpopulations, *P* < 0.001), and these genes characterize markers of the lymphatic endothelium^7^. GO analysis of the strongly expressed genes in EC_1 (75 genes with log_2_FC > 1 *versus* the other subpopulations, *P* < 0.05), EC_2 (72 genes with the same criteria), and EC_3 (190 genes with the same criteria) showed enrichment of expected biological processes. The three clusters also displayed some similar functions, such as the response to wounding, EC proliferation, and regulation of vasculature development (Fig. 2e–g and Supplementary Table 4). Interestingly, GO analyses suggested highly specialized functional features of different clusters. For example, EC_1 responded to the regulation of body fluid levels and hemostasis (Fig. 2e), EC_2 expressed several genes that regulate cell migration and angiogenesis (Fig. 2f), and EC_3 showed unique enrichment in leukocyte migration (Fig. 2g).

To further determine the effect of the HFD on endothelial subpopulation markers, we next performed differential analysis on the 3 ECs and identified the differentially expressed genes between the ND and HFD groups (Fig. 2h, i and Supplementary Table 5). Notably, S100a8, a calcium-binding protein that plays an important role in the regulation of the immune response and inflammatory processes, was upregulated in EC_1 in the setting of HFD (Fig. 2h, i). Consistent with this finding, previous reports have indicated that EC-derived S100a8/9 may contribute to the amplification of inflammatory processes by enhancing leukocyte shape changes and transmigration in the microcirculation^18,19^. In addition, EC_1 in the HFD group strongly expressed a disintegrin and metalloproteinase with thrombospondin motifs 1 and Lyve1. Markers of cell migration, including insulin-like growth factor-binding protein 4^20^, which plays an important role in postdevelopmental adipose tissue expansion, and peptidase inhibitor 16^21^, were decreased in EC_1 in the HFD group. This evidence revealed that the cells in EC_1, which is also called the ‘activated’ EC cluster, may proliferate and play a role in adhesion via proinflammatory cytokines under HFD feeding. We further identified the expression of contractile genes^7^ such as Acta2, myosin light chain kinase (Mylk), and Myh11 and found that the expression of these genes was upregulated in all EC subpopulations after HFD feeding (Fig. 2j). In addition, RT–PCR was used to confirm the significant increases in the expression of Acta2 and Myh11 under HFD conditions (Supplementary Fig. 5a).

Finally, we compared our EC subpopulations with endothelial cells from a recent study involving single-cell analysis of mouse aortas^7^. Three EC subpopulations showed significant differences in gene expression, including Cd36^high^ ECs, Vcam1^high^ ECs, and lymphatic endothelial cells; this classification was similar to the classification of our endothelial cell subpopulations (Supplementary Fig. 6).

### Vascular SMC transcriptomes display heterogeneity of phenotype and function in HFD mouse ascending aortas

**Fig. 3:**
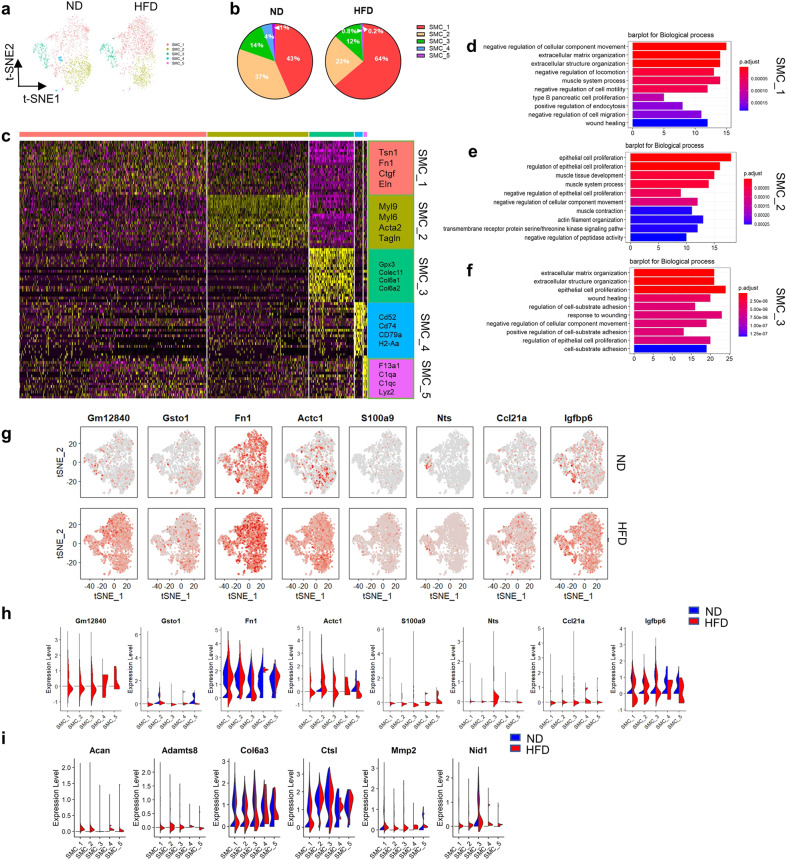
Comparison of SMC subpopulations in ascending aortas from ND and HFD mice. **a**, t-SNE plots of the SMC subpopulations (SMC_1, SMC_2, SMC_3, SMC_4, and SMC_5) from the ND (2,601 cells) and HFD (2,370 cells) groups. **b**, Percentages of the SMC subpopulations from the ND (SMC_1, 43%; SMC_2, 37%; SMC_3, 14%; SMC_4, 4%; and SMC_5, 1%) and HFD (SMC_1, 64%; SMC_2, 23%; SMC_3, 12%; SMC_4, 0.8%; and SMC_5, 0.2%) groups. **c**, Heatmap of the top 20 marker genes per subpopulation. **d**–**f**, Top 10 pathways associated with the SMC_1 (**d**), SMC_2 (**e**), and SMC_3 (**f**) clusters. **g**, Feature plots of the expression of selected marker genes for the SMC subpopulations from the ND and HFD groups. **h**, Violin plots of the expression of selected marker genes for the SMC subpopulations from the ND and HFD groups. **i**, Expression of extracellular matrix (ECM) degradation-associated genes for SMC subpopulations as shown by violin plots.

Five SMC clusters were identified in the ascending aortas of ND and HFD mice (Fig. 3a). SMC_1, SMC_2, and SMC_3 accounted for 95% of the SMC population in the ND group, whereas SMC_4 accounted for 4%, and SMC_5 accounted for only 1% (Fig. 3b). Interestingly, the proportion of SMC_1 increased from 43% in the ND group to 64% in the HFD group, while the proportions of the other subpopulations decreased to different degrees in the HFD group compared with the ND group (Fig. 3b), suggesting a special feature or origin of SMC_1.

Although all five subpopulations were considered SMCs, they had various transcriptional characteristics (Figs. 1e, 3c, and Supplementary Table 6). SMC_1 expressed genes associated with proliferation and migration (Fn1, Ctgf, Eln, and Tns1) (Fig. 3c), and their potential functions were also shown through GO analysis of the significantly changed genes (Fig. 3d). SMC_2 displayed contractile markers (Acta2, Myl9, Myl6, and Tagln) (Fig. 3c) and expressed low levels of cytokine and collagen genes (Col4a2, Col6a1, and Ccl4) (Supplementary Table 6). This cluster also expressed genes (Vim and Egr1) enriched in biological processes (GO analysis results in Supplementary Table 7) implicated in the transmembrane receptor protein serine/threonine kinase signaling pathway (Fig. 3e). Gm12840, which acts as a sponge for miR-677-5p to mediate the fibroblast activation induced by TGF-β1 *via* the WISP1/PKB (Akt) signaling pathway^22^, was strongly expressed in SMC_1 and the four other clusters during HFD feeding (Fig. 3g, h). Gsto1, which presumably modulates the severity and expansion of atherosclerosis, was weakly expressed in SMC_1 in the HFD group (Fig. 3g, h). In addition, similar gene expression patterns were displayed after HFD feeding, including upregulation of proinflammatory genes (Jchain and Rgs5) and migration-related genes (Fn1 and Clu) in SMC_1 and SMC_2 (Supplementary Table 8).

Although SMC_3, SMC_4 and SMC_5 displayed the contractile markers Tagln, Myh11, and Acta2, they displayed different transcriptome profiles than SMC_1 and SMC_2 (Fig. 3c). SMC_3 showed strong expression of collagen and oxidation-reduction genes (Col6a1, Col6a2, Gpx3, and Colec11) (Fig. 3c) and expressed genes (Vtn and Fbln1) that respond to extracellular matrix organization, as shown by GO analysis (Fig. 3f). In addition, SMC_4 and SMC_5 both strongly expressed immune-related genes, such as Cd79a, C1qa, and Lyz2, as well as migration-associated genes (Klf2 and Ctss) (Fig. 3c and Supplementary Table 6). In response to the HFD, SMC_3 showed strengthened expression of proinflammatory factors (Ccl21a, Ccl1, and Cxcl1) and contractile factors (Nts and Actc1) (Fig. 3g, h and Supplementary Table 8). Thus, SMC_3 showed a proinflammatory feature, and the cells therein were hence termed inflammatory-like SMCs. Notably, Igfbp6, which regulates vascular SMC proliferation^23^, was strongly expressed in SMC_4 and SMC_5 in the ND group, but the expression was weaker in the HFD group (Fig. 3g, h). Furthermore, SMC_1–3 displayed enhanced expression of ECM degradation genes, including Acan, Adamts8, Col6a3, Ctsl, Mmp2, and Nid1, in response to HFD feeding (Fig. 3i). The expression of Acan, Adamts8, Mmp2, and Nid1 was significantly increased in VSMCs under HFD conditions, as determined by RT–PCR (Supplementary Fig. 5b).

### Gene expression heterogeneity in monocyte/macrophage populations in HFD mouse ascending aortas

**Fig. 4:**
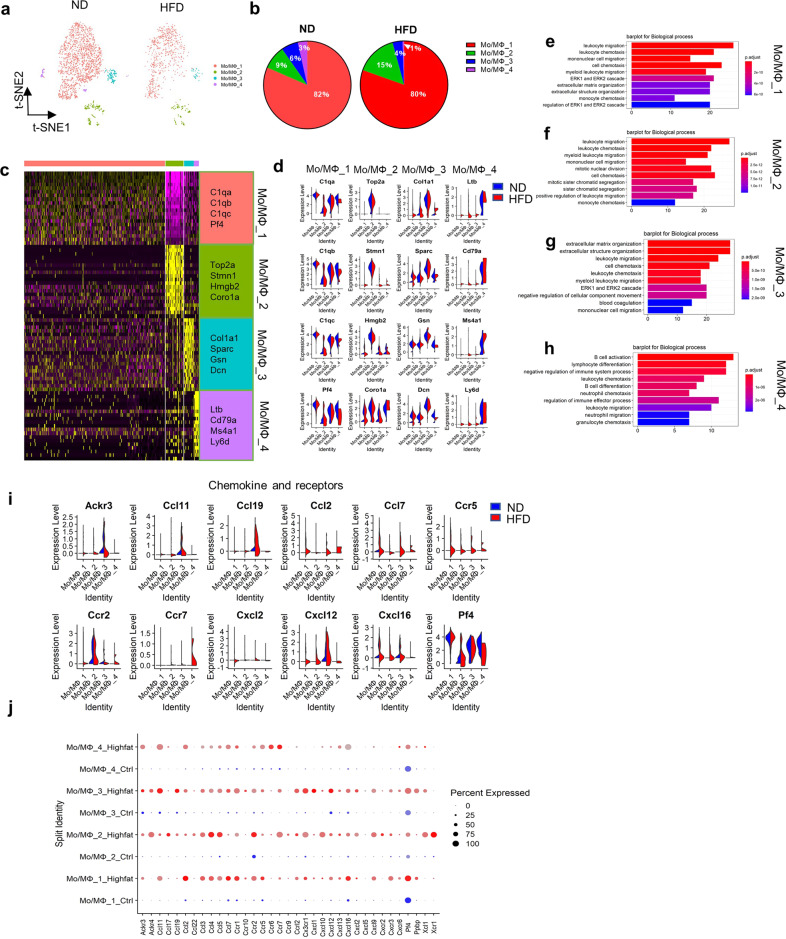
Comparison of monocyte/macrophage (Mo/MΦ) subpopulations in ascending aortas from ND and HFD mice. **a** t-SNE plots of the Mo/MΦ subpopulations (Mo/MΦ_1, Mo/MΦ_2, Mo/MΦ_3, Mo/MΦ_4) from the ND (1991 cells) and HFD (731 cells) groups. **b** Percentages of Mo/MΦ subpopulations from the ND (Mo/MΦ_1, 82%; Mo/MΦ_2, 9%; Mo/MΦ_3, 6%; and Mo/MΦ_4, 3%) and HFD (Mo/MΦ_1, 80%; Mo/MΦ_2, 15%; Mo/MΦ_3, 4%; and Mo/MΦ_4, 1%) groups. **c** Heatmap of the top 20 marker genes per subpopulation. **d** Violin plots of signature genes confirming the subpopulation identities. **e**–**h** Top 10 pathways associated with the Mo/MΦ_1 (**e**), Mo/MΦ_2 (**f**), Mo/MΦ_3 (**g**), and Mo/MΦ_4 (**h**) clusters. **i** Violin plots of the differential expression of chemokine- and receptor-associated genes by Mo/MΦ subpopulation. **j** Expression of chemokine- and receptor-associated genes for Mo/MΦ subpopulations as visualized by dot plot.

Four monocyte/macrophage clusters (Mo/MΦ_1–4) were identified among the ascending aortic cells of ND and HFD mice in the previous cluster analysis (Figs. 1f and 4a). In the ND group, 82% of Mo/MΦ cells corresponded to Mo/MΦ_1, and the remaining three clusters accounted for only 18% of cells (9%, 6%, and 3%) (Fig. 4b). Notably, although four Mo/MΦ clusters expressed recognized macrophage markers (Cd68, Cd14, and Adgre1), they differentially expressed multiple genes (Fig. 4c, d and S3). For example, Pf4, which was the first CXC class chemokine to be described and is an abundant platelet protein^24^, and C1 complement genes (C1qa, C1ab, and C1qc) were prominently expressed by Mo/MΦ_1. Mo/MΦ_2 strongly expressed cell chemotaxis genes, including Coro1a, Ccr2, and Hmgb2 (Fig. 4c, d and Supplementary Table 9), and expressed genes involved in nuclear division (Top2a and Stmn1) (Fig. 4c, d). In Mo/MΦ_3, Col1a1 (encoding type I collagen) and Sparc (regulating cell growth) were strongly expressed (Fig. 4c, d). The smallest cluster, Mo/MΦ_4, exhibited the strongest expression of B cell-like markers, including Cd79a, Ms4a1, and Ly6d (Fig. 4c, d). Moreover, an increased proportion of Mo/MΦ_2 cells and decreased proportions of Mo/MΦ_1, Mo/MΦ_3, and Mo/MΦ_4 cells were observed among all macrophage clusters in the ascending aortas after HFD intake (Fig. 4b). The invasion of aortic macrophage subpopulations was validated by immunostaining in HFD mouse ascending aortas (Supplementary Fig. 7).

To further characterize the 4 subpopulations, we next examined the biological processes differentially enriched per population as well as the enriched pathway genes (Fig. 4e–h and Supplementary Table 10). Both Mo/MΦ_1 and Mo/MΦ_2 showed enrichment for leukocyte migration and leukocyte chemotaxis, suggesting that cellular activation, recruitment, and immune cell interactions drove their phenotype (Fig. 4e, f). Of note, the biological processes of mitotic nuclear division and sister chromatid segregation were enriched in Mo/MΦ_2, which may have led to the increased proportion of Mo/MΦ_2 cells under HFD conditions. Similar to findings in previous reports^25^, Ccl8, Pf4, F13a1, Wfdc17, and Lyve1, considered to be markers of aortic-resident macrophages, were strongly expressed in Mo/MΦ_1 (Supplementary Table 9). In addition, Mo/MΦ_3 was enriched for extracellular matrix organization (Fig. 4g), strongly expressed proteolysis genes (Mmp2, Adamts5, and Ctsl) (Supplementary Table 9) and strongly expressed proinflammatory cytokines (Ccl2, Ccl11, Ccr1, Cx3cr1, Cxcl12, and Pf4) (Fig. 4j), thus resembling the subcluster of blood-derived macrophages. In addition, the expression of chemokines and receptors, including Ackr3, Ccl11, Ccl19, Ccl7, Cxcl12, and Cxcl16, was enhanced in Mo/MΦ_3 during HFD intake (Fig. 4i, j). Interestingly, Mo/MΦ_4 corresponded to lymphocyte differentiation and B cell activation, and the most strongly expressed genes included Ly6d, Ltb, and Cd79a, which are known markers of B cells (Fig. 4c, d, h). Furthermore, RT–PCR was used to show that the expression of most inflammatory chemokines and receptors increased in the setting of HFD feeding (Supplementary Fig. 5c).

### Intercellular communication drives inflammation in the ascending aortas of HFD mice

**Fig. 5:**
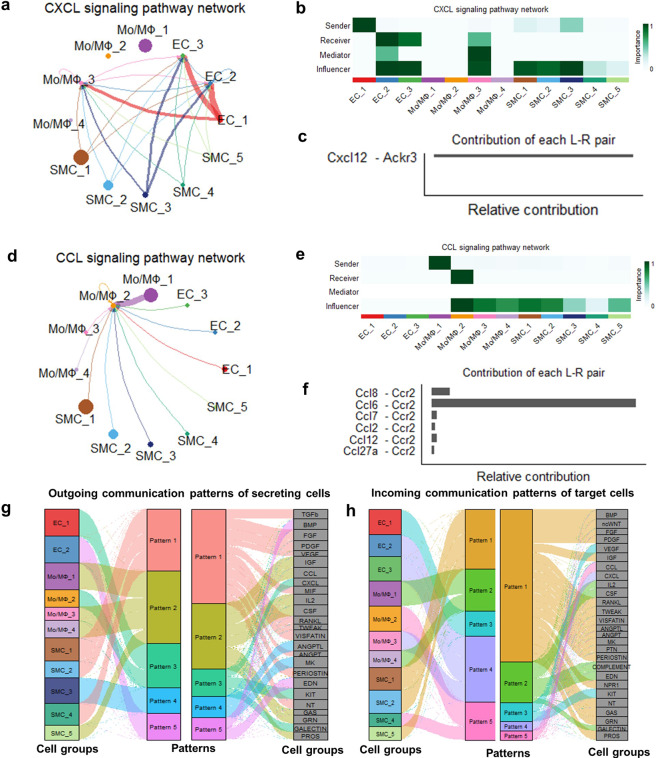
Ligand–receptor interaction analyses to assess intracellular communication in ascending aortas from ND and HFD mice. **a** An inferred intercellular communication network for CXCL signaling among endothelial cells, smooth muscle cells, and monocytes/macrophages is shown in the circle plot (the number of cells in each cluster is proportional to the circle size; the line thickness represents the strength of signaling). **b** The importance of each cluster based on the four network centrality measures of CXCL signaling is shown in the heatmap. **c** Relative contribution of each ligand-receptor pair to the overall communication network of the CXCL signaling pathway. **d** An inferred intercellular communication network for CCL signaling among endothelial cells, smooth muscle cells, and monocytes/macrophages is shown in the circle plot. **e** The importance of each cluster based on the four network centrality measures of CCL signaling is shown in the heatmap. **f** Relative contribution of each ligand-receptor pair to the overall communication network of the CCL signaling pathway. **g**, **h** Outgoing communication patterns of secreting cells (**g**) and incoming communication patterns of target cells (**h**) are shown in the alluvial plot.

In a previous report, to predict intercellular communication, potential ligand-receptor interactions between cell types were examined, and macrophages, ECs, and SMCs were found to have increased numbers of interactions^10^. In our study, 30 signaling pathways, including the TGFβ, BMP, PDGF, VEGF, CXCL, CCL, IL2, and MIF pathways, were detected among the 12 cell subclusters (Fig. 5g, h). EC and SMC populations are the sources for CXCL ligands that act on Mo/MΦ cells, as was verified by analysis of the CXCL signaling network (Fig. 5a, b). Interestingly, EC_2 was also an important mediator and played the role of a gatekeeper of intercellular communication. These findings demonstrate that ECs play critical roles in initiating inflammation during aortic disease and in driving the activation of aorta-resident macrophages *via* CXCL signaling^26^. Furthermore, our data showed that the major mode of CXCL interaction was paracrine signaling in HFD-fed aortic cells, with only one EC population (EC_2) and one macrophage population (Mo/MΦ_3) showing evident autocrine signaling (Fig. 5a). Notably, aortic CXCL signaling was dominated by the Cxcl12 ligand and its receptor Ackr3 among all ligand-receptor pairs (Fig. 5c).

Compared with the CXCL signaling network, our analysis of the CCL signaling network revealed a major difference: a simple structure with only one receptor (Ccr2) and only one population of macrophages (Mo/MΦ_2) displaying a greater extent of macrophage-to-macrophage signaling and less EC-to-macrophage and SMC-to-macrophage signaling (Fig. 5d–f). Prominent influencers, SMCs, were shown by network centrality analysis to control communications (Fig. 2e). Importantly, increased expression of Ccr2 in macrophages and its role in aortic disease have been reported^27–30^.

## Discussion

The scRNA-seq technique has been used to show the heterogeneity of vascular cells, including ECs^7^, vascular SMCs^9^, macrophages^8,10^, monocytes^31^, and fibroblasts^32^, in healthy and diseased arteries. However, the cellular heterogeneity and transcriptional features associated with arterial disease remain largely unknown. In this study, we revealed the comprehensive cellular composition of the mouse ascending aorta and obtained novel insights into how gene expression profiles are altered in a multitude of multifunctional HFD-induced aortic cells. Our data suggest that extensive expression of contractile genes in all ECs, ECM degradation in inflammatory SMCs, and enhanced expression of proinflammatory cytokines in blood-derived monocytes/macrophages occur in the ascending aorta after mice are fed a HFD. In addition, the results of cluster analyses of other cell types in both the ND and HFD mouse ascending aortas are displayed by t-SNE plots (Supplementary Fig. 8).

In agreement with previous studies on EC heterogeneity in the aorta conducted using scRNA-seq approaches^7^, our cellular composition study revealed 3 subpopulations of ECs in the ascending aortas of HFD mice and displayed their unique transcriptome profiles, which suggested corresponding functional signatures. EC heterogeneity has been identified among different organs, among different levels of the vascular tree, and, more recently, within a single vessel^33–35^. Previous work seeking to define EC heterogeneity has focused on variations in individual markers such as CD31, CD34, and vWF^35,36^. We identified 3 distinct profiles of ECs in the ascending aorta, including one cluster of adhesion/transport ECs (EC_1) characterized by the expression of lipid transport- and cell adhesion-related markers such as Fapb4, Cd36, Igfbp7, and Cxcl12. In addition, EC_1 exhibited reduced expression of cell migration genes, increased expression of genes related to inflammation and contraction, and an increased percentage of cells in the setting of HFD feeding. These differences suggest functional specialization of the EC_1 subpopulation in lipid handling and inflammation. Based on the differential gene expression patterns, we found that EC_2 strongly expressed genes that contribute to EC proliferation and the regulation of angiogenesis. A novel leukocyte-like EC subpopulation (EC_3) with strong expression of molecules related to leukocyte migration was also described. Importantly, the finding of upregulation of contractile gene expression in ECs after HFD feeding is particularly interesting given that endothelial-mesenchymal transition is a known common final pathway in EC dysfunction^7^. Accordingly, our data from aortic ECs from HFD mice showed upregulation of contractile genes and will serve as a reference for comprehensive characterization of vascular EC heterogeneity in healthy and diseased tissue.

Our study showed that there are 5 subtypes of vascular SMCs in the ascending aorta: synthetic (SMC_1), contractile (SMC_2), fibroblast-like (SMC_3), and inflammatory (SMC_4 and SMC_5) SMCs. The presence of synthetic, contractile, and fibroblast-like subpopulations is consistent with previous reports^15,16,37^. SMC_1 accounted for the highest proportion and expressed genes involved in cell proliferation and migration, but it expressed low levels of contractile genes. The increase in SMC_1 with low expression of contractile genes is an important factor promoting the pathological progression of aortic diseases due to the phenotypic transformation of smooth muscle cells under high-fat diet conditions^38^. Unlike the SMC_1 subpopulation, SMC_2 demonstrated the strongest expression of contractile transcription factors and the weakest expression of genes associated with ECM organization, even upon HFD feeding. These cells may play a pivotal role in the function of the aorta, and both good contractile function and good proliferative function are necessary for vascular regeneration^39^. Additionally, a novel fibroblast-like subpopulation (SMC_3) with the strongest expression of collagen and oxidation-reduction genes along with weak expression of contractile genes was described. The two subpopulations with the lowest proportions, SMC_4 and SMC_5, both showed increased expression of characteristic leukocyte genes associated with inflammation and ECM remodeling. Consistent with previous studies^40,41^, HFD intake induced upregulation of proinflammatory cytokines and proteinases, including Ccl21a, Ccl1, Cxcl1, Adamts1, and Il-6. Additionally, Bmp4, whose function in vascular restenosis and atherosclerosis has been well characterized^42^, was highly expressed only in SMC_3, especially in HFD SMCs. Thus, our scRNA-seq data from the ascending aortas of ND and HFD mice display the phenotypic diversity of vascular SMCs and provide unprecedented depth of information for characterization of SMC heterogeneity in healthy and diseased blood vessels.

Many studies have investigated the roles of macrophages in the pathophysiology of vascular inflammatory conditions, including hyperlipidemia associated with obesity, which contributes to atherosclerosis and aortic aneurysms^8,43–45^. In the process of vascular inflammation, one significant change in the adventitia is macrophage infiltration, which leads to amplification of the local inflammatory response through secretion of proinflammatory chemokines and cytokines as well as production of reactive oxygen species and proteases^6,46,47^. Considerable scRNA-seq data and numerous lineage tracing experiments have shown the heterogeneity of macrophages in HFD-induced aortic disease^8,25,48^. Based on scRNA-seq data, we have now uncovered 4 major Mo/MΦ subpopulations in the ascending aorta, including aortic-resident clusters (Mo/MΦ_1 and Mo/MΦ_2), a blood-derived cluster (Mo/MΦ_3), and a B cell-like cluster (Mo/MΦ_4). Although both aortic-resident macrophage clusters strongly expressed proinflammatory cytokines, the different genetic characteristics of the two subpopulations further displayed their unique functions. Mo/MΦ_1, which accounted for the larger proportion of the aortic-resident subpopulation, likely plays roles in antigen presentation and regulation of the ERK1 and ERK2 cascades. However, Mo/MΦ_2 was identified as containing proliferative aortic-resident macrophages, in keeping with the strong self-differentiation properties of the cells. In the setting of HFD intake, Mo/MΦ_2 cells continued to proliferate and express chemokines, increasing the likelihood that this subpopulation can be programmed to aggravate inflammation. Beyond the aortic-resident macrophage clusters, we also discovered another macrophage cluster of so-called blood-derived macrophages, Mo/MΦ_3, which strongly expressed proinflammatory genes, such as Ccl19, Ccl11, and Cxcl12, as well as secreted proteases, such as Mmp2, resulting in ECM degradation and aortic disease^4^. The Mo/MΦ_4 cluster also expressed inflammatory genes, such as Ccr7, which is associated with regulation of the immune effector process. Thus, our data provide an in-depth characterization of diverse macrophages in HFD-induced vascular inflammatory progression.

Among the inflammatory cell populations infiltrating aortic tissue, macrophages compose the main population, and their role in the pathogenesis of HFD-induced vascular injury is well described in mice^6,14^. Furthermore, consistent with previous studies^8,25^, the presence of B cells, T cells, DCs, and natural killer cells in HFD-induced vascular disease was characterized at the single-cell level. Of note, ECs, SMCs, and macrophages engaged in intensive communication by secreting cytokines in the setting of HFD feeding. By demonstrating the major pathway of intercellular communication, our study suggests that the binding of chemokines to ECs, SMCs, and macrophages triggers complex pathways of intracellular communication that directly or indirectly participate in regulating the development of vascular inflammation.

In summary, our study provides a comprehensive transcriptome profile for HFD mouse ascending aortas. Based on the RNA expression of individual cells, we uncovered, among other findings, the presence of proinflammatory, synthetic, and vascular SMC populations; multiple different sources of macrophages; functionally distinct EC populations; and their interactions. All of these factors can be considered influencers of aortic disease. These findings provide a valuable means of understanding metabolic disorders in the aorta and may contribute to the development of new methods of diagnosis and intervention.

## Supplementary information

Supplemental Information

## Data Availability

All data have been deposited into the Single Cell Portal: https://singlecell.broadinstitute.org/single_cell/study/SCP1361/single-cell-transcriptome-analysis-reveals-cellular-heterogeneity-in-the-ascending-aorta-of-normal-and-high-fat-diet-mice.

## References

[1] Grandl G, Wolfrum C (2018). Hemostasis, endothelial stress, inflammation, and the metabolic syndrome. Semin. Immunopathol..

[2] Spiegelman BM, Flier JS (2001). Obesity and the regulation of energy balance. Cell.

[3] Schäfer N (2013). Endothelial mineralocorticoid receptor activation mediates endothelial dysfunction in diet-induced obesity. Eur. Heart J..

[4] Rabkin SW (2017). The role matrix metalloproteinases in the production of aortic aneurysm. Prog. Mol. Biol. Transl. Sci..

[5] Zhao G (2017). Unspliced XBP1 confers VSMC homeostasis and prevents aortic aneurysm formation via FoxO4 interaction. Circ. Res..

[6] Raffort J (2017). Monocytes and macrophages in abdominal aortic aneurysm. Nat. Rev. Cardiol..

[7] Kalluri AS (2019). Single-cell analysis of the normal mouse aorta reveals functionally distinct endothelial cell populations. Circulation.

[8] Cochain C (2018). Single-cell RNA-seq reveals the transcriptional landscape and heterogeneity of aortic macrophages in murine atherosclerosis. Circ. Res..

[9] Pedroza AJ (2020). Single-cell transcriptomic profiling of vascular smooth muscle cell phenotype modulation in Marfan syndrome aortic aneurysm. Arterioscler. Thromb. Vasc. Biol..

[10] Depuydt MAC (2020). Microanatomy of the human atherosclerotic plaque by single-cell transcriptomics. Circ. Res..

[11] Gu W (2019). Adventitial cell atlas of wt (Wild Type) and ApoE (Apolipoprotein E)-deficient mice defined by single-cell RNA sequencing. Arterioscler. Thromb. Vasc. Biol..

[12] Fleury Curado T (2018). Sleep-disordered breathing in C57BL/6J mice with diet-induced obesity. Sleep.

[13] Berger S (2019). Intranasal leptin relieves sleep-disordered breathing in mice with diet-induced obesity. Am. J. Respir. Crit. Care Med..

[14] Zhao G (2020). Single cell RNA sequencing reveals the cellular heterogeneity of aneurysmal infrarenal abdominal aorta. Cardiovasc. Res..

[15] He D (2020). Aortic heterogeneity across segments and under high fat/salt/glucose conditions at the single-cell level. Natl Sci. Rev..

[16] Li Y (2020). Single-cell transcriptome analysis reveals dynamic cell populations and differential gene expression patterns in control and aneurysmal human aortic tissue. Circulation.

[17] Jin S (2021). Inference and analysis of cell-cell communication using CellChat. Nat. Commun..

[18] Ganta VC, Choi M, Farber CR, Annex BH (2019). Antiangiogenic VEGF(165)b regulates macrophage polarization via S100A8/S100A9 in peripheral artery disease. Circulation.

[19] Acharyya S (2012). A CXCL1 paracrine network links cancer chemoresistance and metastasis. Cell.

[20] Gealekman O (2014). Control of adipose tissue expandability in response to high fat diet by the insulin-like growth factor-binding protein-4. J. Biol. Chem..

[21] Singhmar P (2020). The fibroblast-derived protein PI16 controls neuropathic pain. Proc. Natl Acad. Sci. USA.

[22] Chen H (2020). LncRNA Gm12840 mediates WISP1 to regulate ischemia-reperfusion-induced renal fibrosis by sponging miR-677-5p. Epigenomics.

[23] Wang Z (2020). IGFBP6 regulates vascular smooth muscle cell proliferation and morphology via cyclin E-CDK2. J. Cell. Physiol..

[24] Shi G (2013). Platelet factor 4 mediates vascular smooth muscle cell injury responses. Blood.

[25] Zernecke A (2020). Meta-analysis of leukocyte diversity in atherosclerotic mouse aortas. Circ. Res..

[26] Rousselle A (2013). CXCL5 limits macrophage foam cell formation in atherosclerosis. J. Clin. Invest..

[27] Veillard NR (2005). Differential influence of chemokine receptors CCR2 and CXCR3 in development of atherosclerosis in vivo. Circulation.

[28] Ishibashi M (2004). Critical role of monocyte chemoattractant protein-1 receptor CCR2 on monocytes in hypertension-induced vascular inflammation and remodeling. Circ. Res..

[29] Boring L, Gosling J, Cleary M, Charo IF (1998). Decreased lesion formation in CCR2-/- mice reveals a role for chemokines in the initiation of atherosclerosis. Nature.

[30] Wenzel P (2015). Heme oxygenase-1 suppresses a pro-inflammatory phenotype in monocytes and determines endothelial function and arterial hypertension in mice and humans. Eur. Heart J..

[31] Roberts ME (2020). Deep phenotyping by mass cytometry and single-cell RNA-sequencing reveals LYN-regulated signaling profiles underlying monocyte subset heterogeneity and lifespan. Circ. Res..

[32] Muhl L, Genové G (2020). Single-cell analysis uncovers fibroblast heterogeneity and criteria for fibroblast and mural cell identification and discrimination. Nat. Commun..

[33] Augustin HG, Koh GY (2017). Organotypic vasculature: from descriptive heterogeneity to functional pathophysiology. Science.

[34] Potente M, Mäkinen T (2017). Vascular heterogeneity and specialization in development and disease. Nat. Rev. Mol. Cell Biol..

[35] Yuan L (2016). A role of stochastic phenotype switching in generating mosaic endothelial cell heterogeneity. Nat. Commun..

[36] Pusztaszeri MP, Seelentag W, Bosman FT (2006). Immunohistochemical expression of endothelial markers CD31, CD34, von Willebrand factor, and Fli-1 in normal human tissues. J. Histochem. Cytochem..

[37] Shanahan CM, Weissberg PL (1998). Smooth muscle cell heterogeneity: patterns of gene expression in vascular smooth muscle cells in vitro and in vivo. Arterioscler. Thromb. Vasc. Biol..

[38] Alencar GF (2020). Stem cell pluripotency genes Klf4 and Oct4 regulate complex SMC phenotypic changes critical in late-stage atherosclerotic lesion pathogenesis. Circulation.

[39] Wirka RC, Wagh D, Paik DT (2019). Atheroprotective roles of smooth muscle cell phenotypic modulation and the TCF21 disease gene as revealed by single-cell analysis. Nat. Med..

[40] Canesi F (2019). A thioredoxin-mimetic peptide exerts potent anti-inflammatory, antioxidant, and atheroprotective effects in ApoE2.Ki mice fed high fat diet. Cardiovasc. Res..

[41] Kelley EE (2014). Fatty acid nitroalkenes ameliorate glucose intolerance and pulmonary hypertension in high-fat diet-induced obesity. Cardiovasc. Res..

[42] Vendrov AE, Madamanchi NR, Hakim ZS, Rojas M, Runge MS (2006). Thrombin and NAD(P)H oxidase-mediated regulation of CD44 and BMP4-Id pathway in VSMC, restenosis, and atherosclerosis. Circ. Res..

[43] Li Q (2017). Regulation of macrophage apoptosis and atherosclerosis by lipid-induced PKCδ isoform activation. Circ. Res..

[44] Wei Y (2018). Dicer in macrophages prevents atherosclerosis by promoting mitochondrial oxidative metabolism. Circulation.

[45] Puca AA (2020). Single systemic transfer of a human gene associated with exceptional longevity halts the progression of atherosclerosis and inflammation in ApoE knockout mice through a CXCR4-mediated mechanism. Eur. Heart J..

[46] Rizas KD, Ippagunta N, Tilson MD (2009). Immune cells and molecular mediators in the pathogenesis of the abdominal aortic aneurysm. Cardiol. Rev..

[47] Shimizu K, Mitchell RN, Libby P (2006). Inflammation and cellular immune responses in abdominal aortic aneurysms. Arterioscler. Thromb. Vasc. Biol..

[48] Winkels H (2018). Atlas of the immune cell repertoire in mouse atherosclerosis defined by single-cell RNA-sequencing and mass cytometry. Circ. Res..

