# Supplementary material for: Single-cell transcriptome analysis reveals cellular heterogeneity in the ascending aortas of normal and high-fat diet-fed mice
Source: Exp Mol Med. 2021 Sep 21;53(9):1379–89. doi: 10.1038/s12276-021-00671-2 (PMC8492660; doi:10.1038/s12276-021-00671-2)
Supplement: Supplementary file 1 — Supplemental Information [file 12276_2021_671_MOESM1_ESM.pdf]

Supplemental information

Single-cell transcriptome analysis reveals cellular heterogeneity in the ascending aortas of normal and high-fat diet-fed mice

Hao Kan[1#], Ka Zhang[1#], Aiqin Mao[1#], Li Geng[1], Mengru Gao[1], Lei Feng[1], Qingjun You[2], Xin Ma[1*]

1. Wuxi School of Medicine, Jiangnan University, Wuxi, Jiangsu, China.

2. Department of Thoracic Surgery, The Affiliated Hospital of Jiangnan University, Wuxi, Jiangsu, China.

#Hao Kan, Ka Zhang, and Aiqin Mao contributed equally to this work.

*Corresponding author, name: Xin Ma, email: maxin@jiangnan.edu.cn, phone number: 008618951581116

Supplemental Materials and Methods

Lipid measurements, body weight

Blood was taken form experimental animals every 4 weeks and serum lipids analyzed for total cholesterol, triglycerides, low-density lipoprotein, and high-density lipoprotein using the corresponding reagent kit (Sangon Biotech, China). Body weight was measured by laboratory balance.

Endothelial cell preparation and culture

Primary mouse aortic endothelial cells (MAECs) were isolated from ND and HFD mice and used for RT-qPCR. Briefly, aortas were dissection in PBS and incubated with type IA collagenase (2 mg/ml, sigma, USA) for 15min at 37°C. Detached endothelial cells were collected by centrifugation, resuspended in endothelial cell growth medium (EGM) supplemented with bovine brain extract (Lonza, USA) and culture in a flask at 37°C with 5% CO_2. Non-adherent cells were removed 2 h later. The endothelial cells were cultured in EGM at 37°C with 5% CO_2 for 1-2 days[1].

Smooth muscle cell preparation and culture

Primary mouse VSMCs were isolated according to previous methods[2]. After attachment, the VSMCs were cultured in smooth muscle cell medium (Sciencell, USA), containing streptomycin, penicillin, L-Glutamine, and 10% FBS (Gibco, USA).

Macrophage preparation and culture

Primary mouse macrophages were separated as described previously[3]. Briefly, to prepare bone marrow-derived macrophages, the marrow was flushed with RPMI-1640 (Gibco, USA) medium. The flushed cells were collected through a strainer to remove debris and then centrifuged to collect the precipitate. The cells were resuspended in RPMI-1640 containing 20% FBS (Gibco, USA), and cultured for 3-4 days.

RNA extraction and RT-PCR

The total RNA was extracted from the mice in ND and HFD groups using the Trizol reagent (CWBIO, China). The concentration of RNA was measured by an ultraviolet spectrophotometer (NanoDrop one, USA). Reverse transcription was performed in a total volume of 20 μL. The total RNA (500 ng/μL, 2 μL) was mixed gDNA wiper Mix (4 μL, Vazyme, China) and RNase-free ddH_2O (10 μL), watered bath at 42°C for 2 min, and then added the reverse transcriptase mix (5x HiScript III qRT SuperMix, 4μL, Vazyme). Reverse transcription reaction was performed at 37°C for 15min, 85°C for 5s and 4°C for 5min. Quantitative PCR for cDNAs was conducted using the SYBR Mix reagent (Vazyme, China) and 7500 Real time PCR system, as previously described[4]. Primer sequences (GENEWIZ, China) for related genes were used (Table. S11).

Immunofluorescence

Slides of ascending aortic tissue were fixed in 4% paraformaldehyde at room

temperature for 30 min. The samples were incubated with fetal bovine serum (VisTech) and permeabilized with 1% PBS-Triton at 37°C for 1 h and then with primary antibodies anti-Emr1 (sc-377009, Santa Cruz, USA), anti-Top2a (SAB#41506, Signalway Antibody, USA), anti-C1qa (SAB#29251, Signalway Antibody, USA), anti-Col1a1 (SAB#28307, Signalway Antibody, USA), and anti-Cd79a (SAB#29288, Signalway Antibody, USA). Then the slides were incubated with the secondary antibody AF568 donkey anti-rabbit (Invitrogen, USA) and AF647 doney anti-mouse (Invitrogen, USA). Immunofluorescence was assessed by confocal laser-scanning microscopy (Zeiss LSM 880, Germany). The fluorescence values were analyzed using ImageJ software (developed at the National Institutes of Health). At least 100 cells were analyzed for each segment[5].

Supplemental references

1 Wong, S. L. *et al.* Cyclooxygenase-2-derived prostaglandin F2alpha mediates endothelium-dependent contractions in the aortae of hamsters with increased impact during aging. *Circ. Res.* **104**, 228-235 (2009).

2 Farina, F. M. *et al.* miR-128-3p Is a Novel Regulator of Vascular Smooth Muscle Cell Phenotypic Switch and Vascular Diseases. *Circ. Res.* **126**, e120-e135 (2020).

3 Liao, X. *et al.* Macrophage autophagy plays a protective role in advanced atherosclerosis. *Cell Metab.* **15**, 545-553 (2012).

4 Feng, B. *et al.* The endoplasmic reticulum is the site of cholesterol-induced cytotoxicity in macrophages. *Nat. Cell Biol.* **5**, 781-792 (2003).

5 He, D. *et al.* Aortic heterogeneity across segments and under high fat/salt/glucose conditions at the single-cell level. *Natl. Sci. Rev.* **7**, 881-896 (2020).

Supplemental figures and figure legends

Supplementary Figure 1

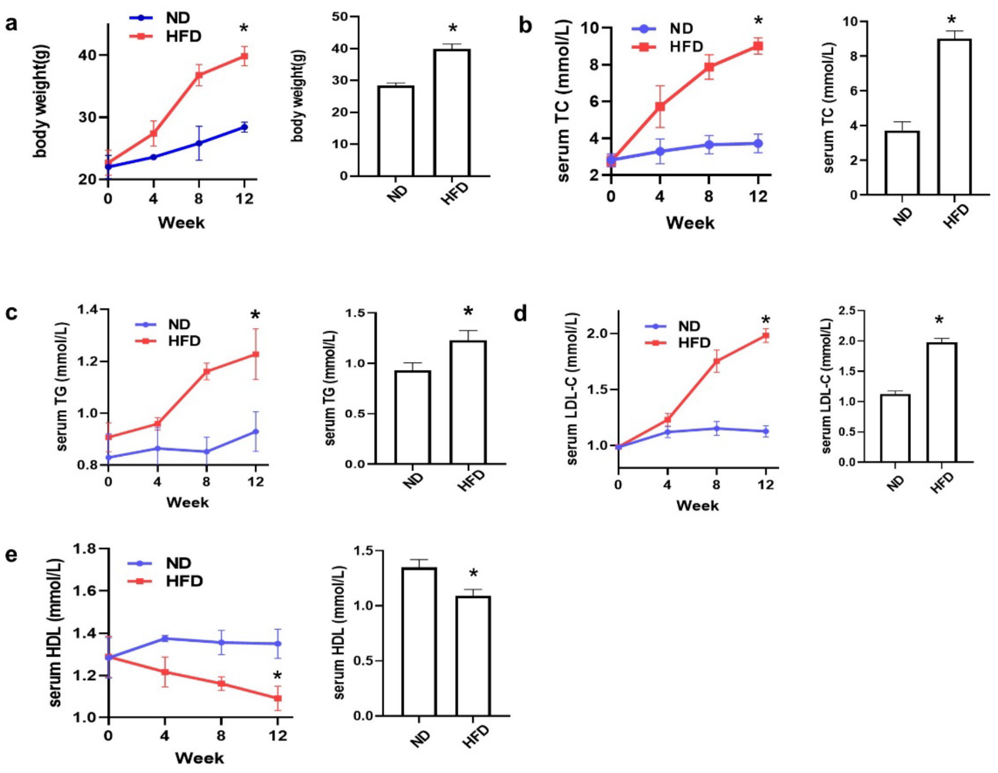

Supplementary Figure 1. Assessment of mouse body weight and lipids under HFD condition. a, body weight; b, serum cholesterol; c, serum triglycerides; d, serum low-density lipoprotein; e, serum high-density lipoprotein during 12 weeks in mice on high-fat diet. N=10 for each analysis. **P*<0.05 by Student's t-test.

Supplementary Figure 2

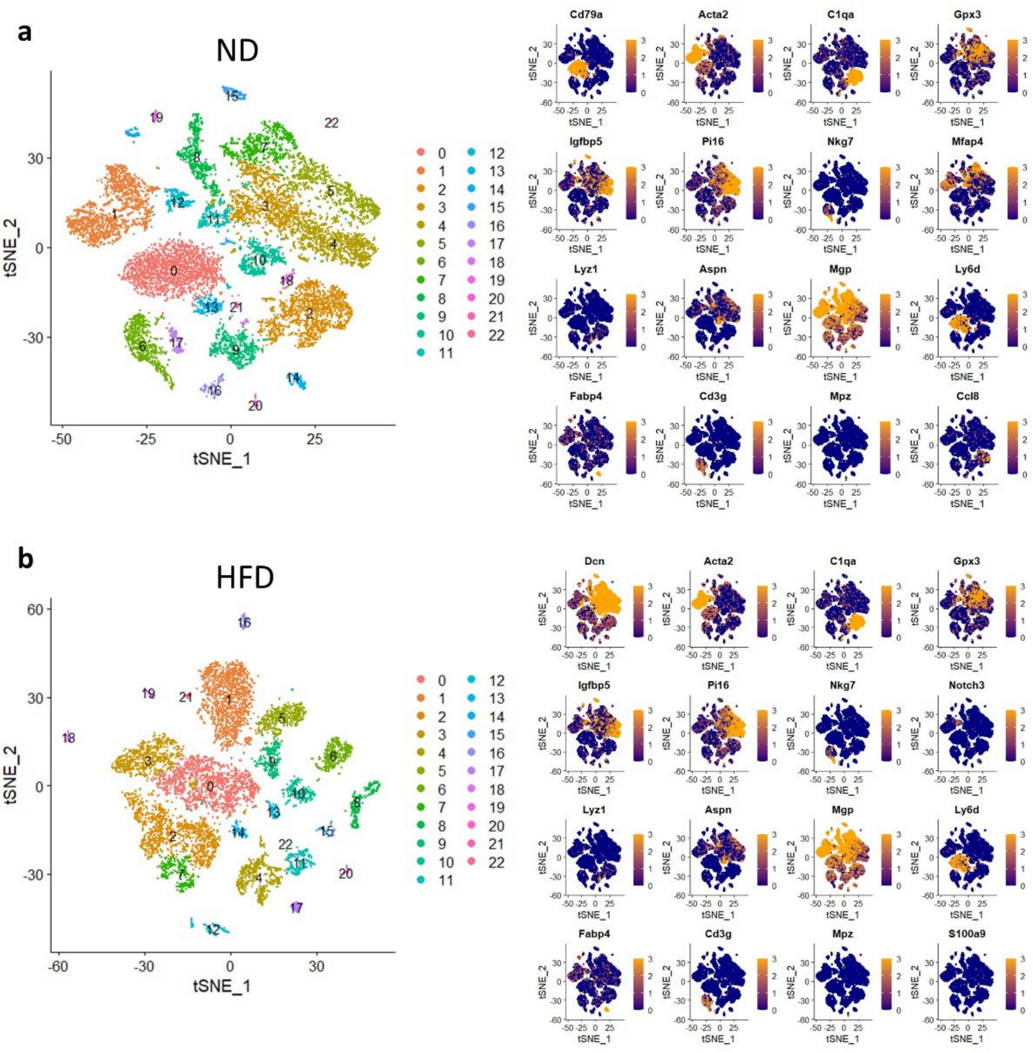

Supplementary Figure 2. Separate analysis of each scRNA-seq group. a, ND; b, HFD. In each panel, a t-SNE plot colored according to clusters is shown on the left, and the expression of select marker genes projected onto a t-SNE plot is shown on the right.

Supplementary Figure 3

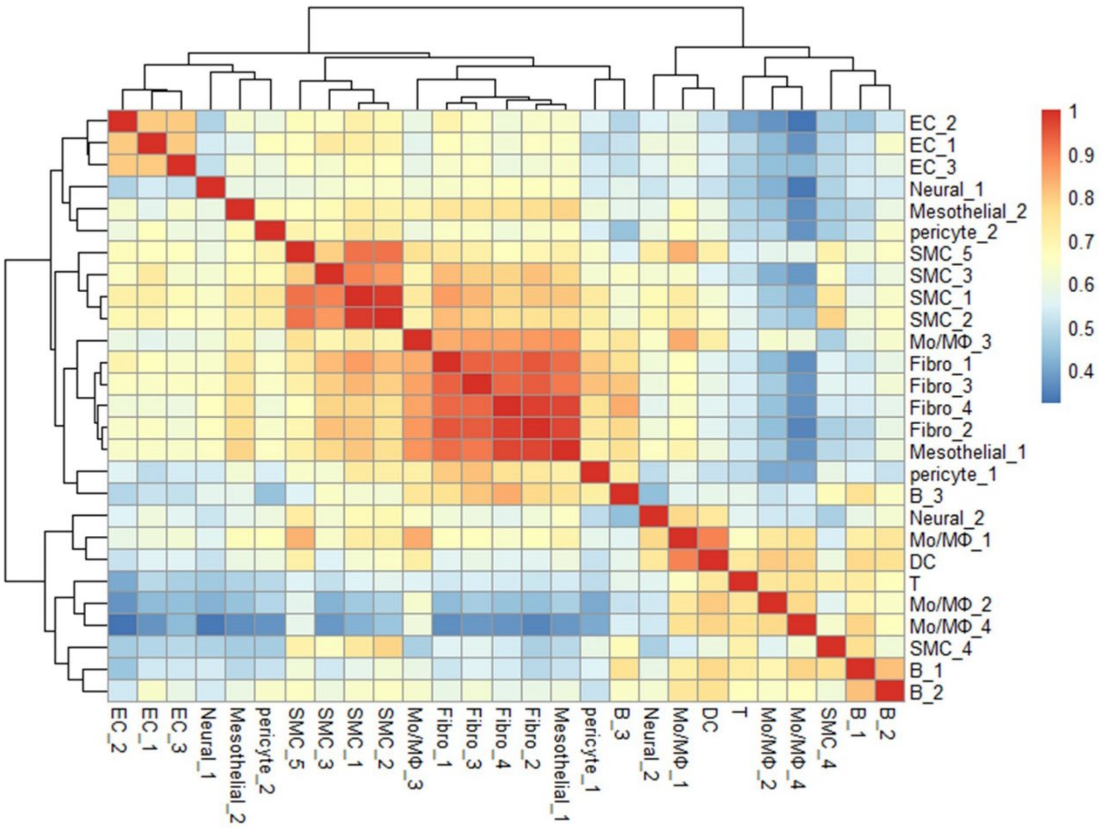

Supplementary Figure 3. Correlations between subpopulations of ascending aortic cells. The correlations between clusters were calculated based on the top 10 marker genes across cells. The correlation coefficients are represented by color (key on right).

Supplementary Figure 4

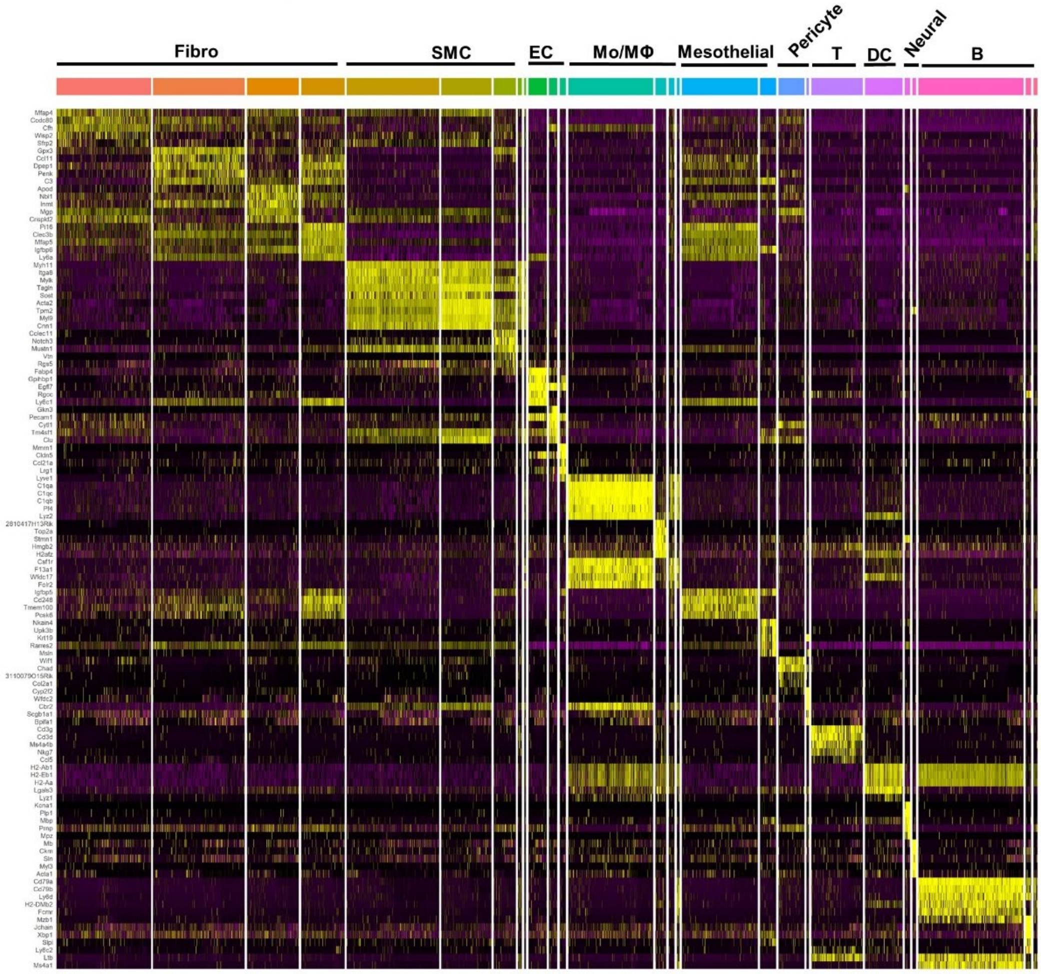

Supplementary Figure 4. Heatmap of top 5 marker genes per cluster.

Supplementary Figure 5

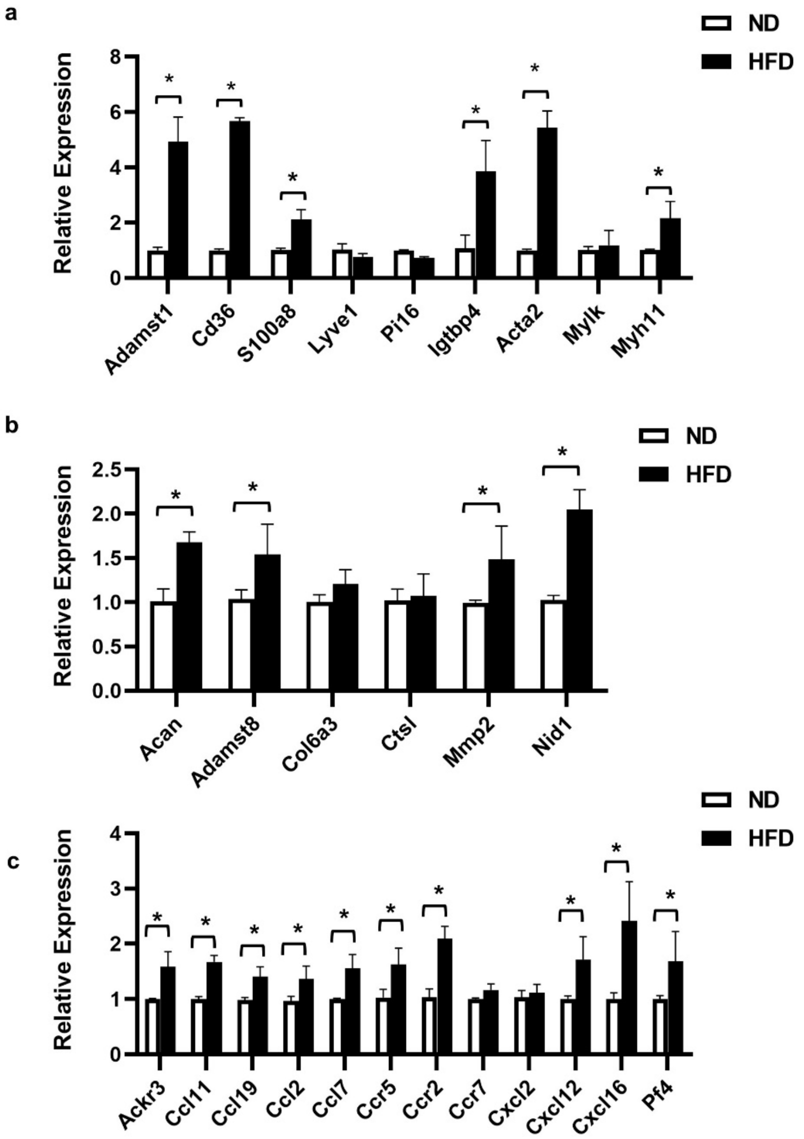

Supplementary Figure 5. The expression of mRNA in ND and HFD mice. a-c, the expression of mRNA with ECs (a), SMCs (b), macrophages (c) in ND and HFD mice. *P<0.05 by Student's t-test.

Supplementary Figure 6

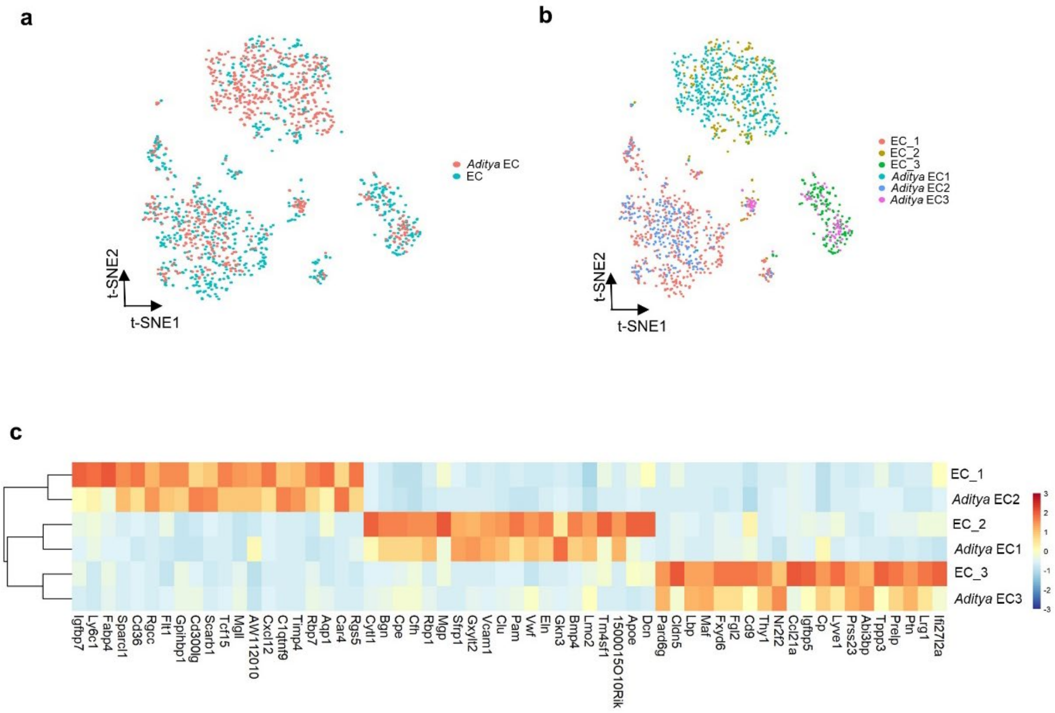

Supplementary Figure 6. Integration of ECs subpopulation from different scRNA-seq data. a and b, t-SNE plot of mouse aortic ECs from *Aditya*'s paper and our study. c, Heatmap showing overlap genes of EC clusters with EC clusters of *Aditya*'s scRNA-seq paper.

Supplementary Figure 7

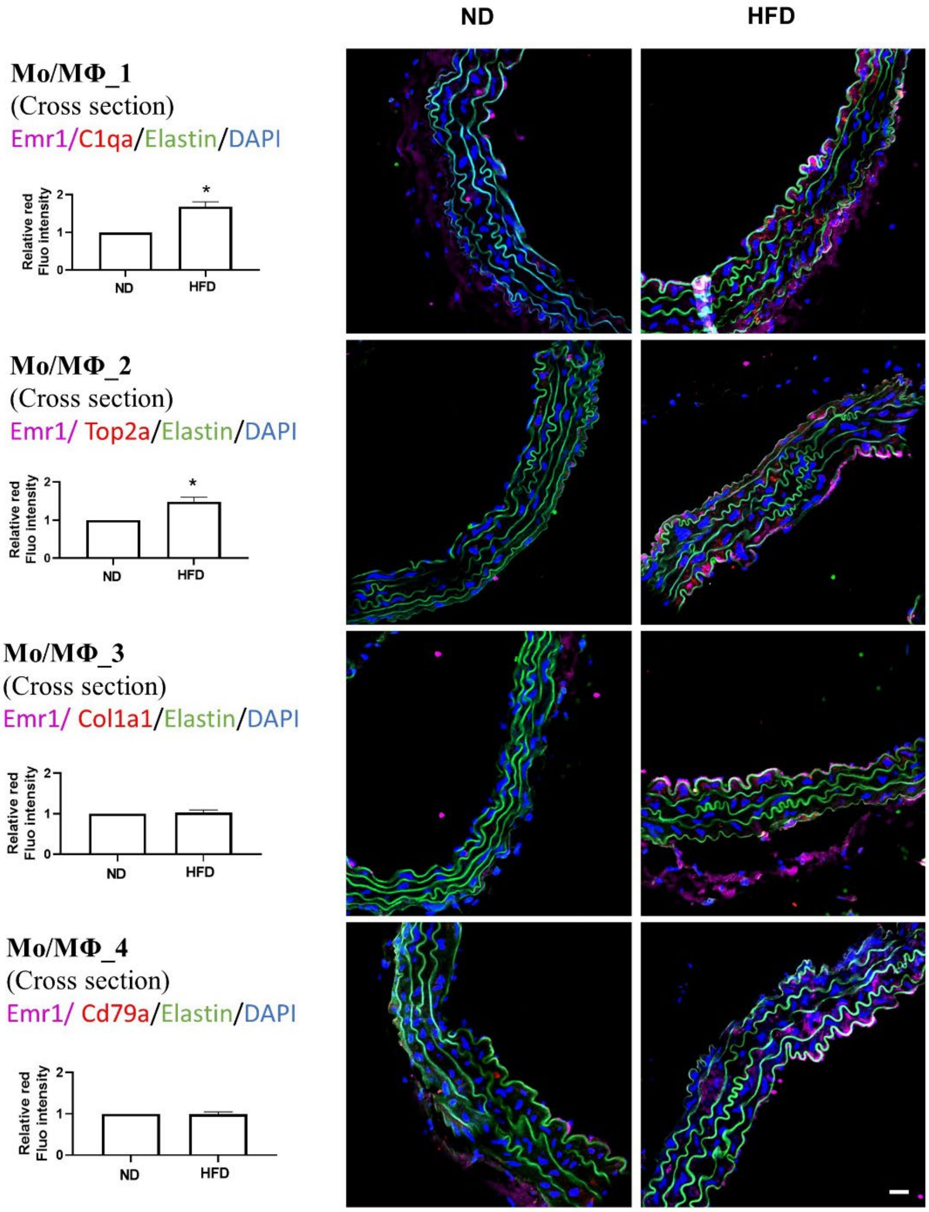

Supplementary Figure 7. Immunofluorescence validation of spatial heterogeneity of aortic macrophages cells. For each macrophage subpopulations, specific antibody displayed the number of macrophages invasion of aortic subintimal and media. The statistical analysis of relative immunofluorescence values are shown in the bar graph, where the value for control aorta was normalized to 1. **P*<0.05 vs control by one-way ANOVA. Scale bar, 50 µm.

Supplementary Figure 8

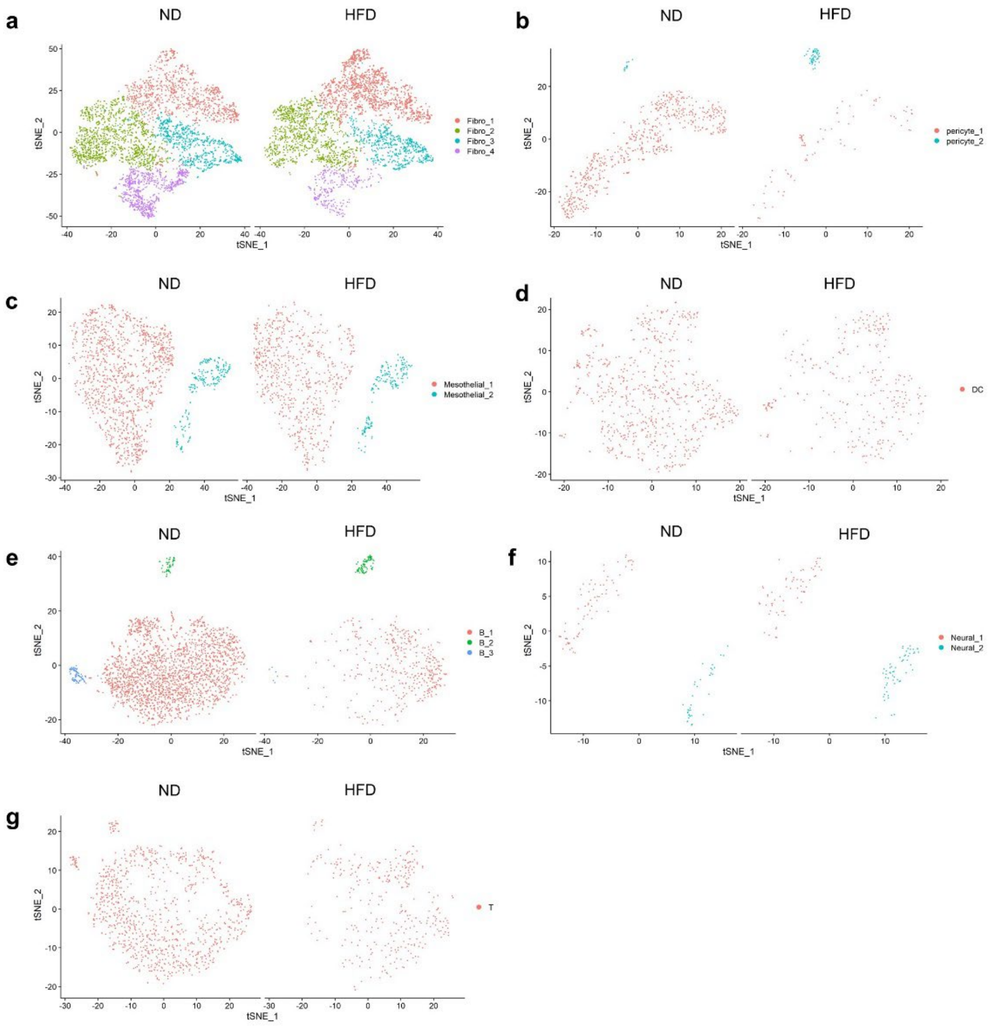

Supplementary Figure 8. Comparison of aortic cell subpopulations from ND and HFD. a-g, t-SNE plot of fibroblasts (a), pericytes (b), mesothelial cells (c), dendritic Cells (d), B cells (e), neural cells (f), and T cells (g) from ND and HFD.

Supplementary Figure 9

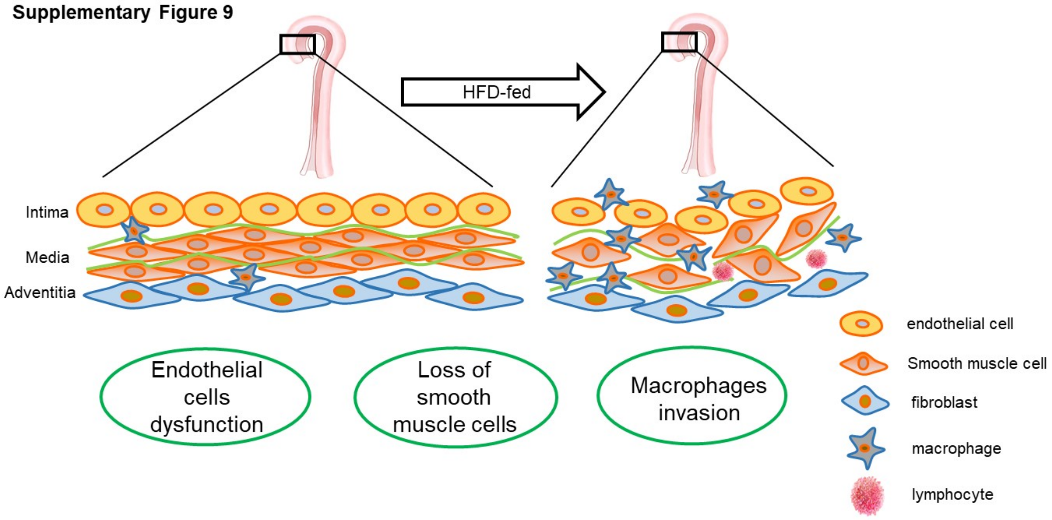

Supplementary Figure 9. Graphical abstract.

Supplemental tables

Supplementary Table 1_cellratio

cell_clusters	groups	proportion (%)
Fibro	ND	34.4952602
SMC	ND	22.0557867
EC	ND	4.291482
Mo/MΦ	ND	7.5783946
Mesothelial	ND	6.2843893
pericyte	ND	4.1260315
T	ND	4.9153652
DC	ND	4.6920821
Neural	ND	0.7433677
B	ND	10.8178408
Fibro	HFD	36.8815592
SMC	HFD	19.3801671
EC	HFD	5.5793532
Mo/MΦ	HFD	9.8282287
Mesothelial	HFD	7.5095309
pericyte	HFD	3.4242879
T	HFD	5.4804027
DC	HFD	5.0841722
Neural	HFD	1.3171985
B	HFD	5.5150996

Supplementary Table 2_marker genes of all clusters

	p_val	avg_logFC	pct.1	pct.2	p_val_adj	cluster	gene
Mfap4	0	1.322861173	0.889	0.498	0	Fibro_1	Mfap4
Ccdc80	0	1.24988746	0.9	0.524	0	Fibro_1	Ccdc80
Cfh	0	1.249231788	0.94	0.565	0	Fibro_1	Cfh
Lum	0	1.233017696	0.976	0.54	0	Fibro_1	Lum
Cygb	0	1.216616872	0.856	0.365	0	Fibro_1	Cygb
Smoc2	0	1.147684567	0.762	0.332	0	Fibro_1	Smoc2
Gxylt2	0	1.043077698	0.703	0.277	0	Fibro_1	Gxylt2
Igf1	0	1.028477336	0.82	0.447	0	Fibro_1	Igf1
Col1a1	0	0.953641577	0.989	0.709	0	Fibro_1	Col1a1
Timp3	0	0.950786594	0.943	0.672	0	Fibro_1	Timp3
Crispld2	0	0.944479659	0.888	0.416	0	Fibro_1	Crispld2
Fbln1	0	0.943806454	0.911	0.473	0	Fibro_1	Fbln1
Col3a1	0	0.923935252	0.993	0.792	0	Fibro_1	Col3a1
Col1a2	0	0.921692094	0.997	0.774	0	Fibro_1	Col1a2
Bgn	0	0.913572247	0.998	0.743	0	Fibro_1	Bgn
Cst3	0	0.909127086	0.997	0.952	0	Fibro_1	Cst3
Rbp1	0	0.892143352	0.929	0.604	0	Fibro_1	Rbp1
Serpinf1	0	0.883268822	0.924	0.554	0	Fibro_1	Serpinf1
Mgp	0	0.882646433	0.997	0.953	0	Fibro_1	Mgp
Dcn	0	0.875945155	0.999	0.843	0	Fibro_1	Dcn
Itgbl1	0	0.862087132	0.77	0.329	0	Fibro_1	Itgbl1
Col15a1	0	0.852297709	0.817	0.39	0	Fibro_1	Col15a1
Fmod	0	0.844189164	0.673	0.263	0	Fibro_1	Fmod
Ndufa4l2	0	0.824734467	0.685	0.167	0	Fibro_1	Ndufa4l2
Tgfbi	0	0.78390741	0.83	0.423	0	Fibro_1	Tgfbi
Pdgfrl	0	0.757010966	0.777	0.33	0	Fibro_1	Pdgfrl
Gsn	0	0.67999433	0.999	0.933	0	Fibro_1	Gsn
Bhlhe41	0	0.676772743	0.714	0.321	0	Fibro_1	Bhlhe41
Pdgfra	0	0.672507066	0.85	0.434	0	Fibro_1	Pdgfra
Dpt	0	0.67073195	0.982	0.582	0	Fibro_1	Dpt
Angptl7	0	0.670379289	0.588	0.187	0	Fibro_1	Angptl7
Mmp2	0	0.649817016	0.913	0.558	0	Fibro_1	Mmp2
Mdk	0	0.604869186	0.706	0.31	0	Fibro_1	Mdk
Clec11a	0	0.547971424	0.696	0.285	0	Fibro_1	Clec11a
Ltbp2	0	0.465604872	0.65	0.179	0	Fibro_1	Ltbp2
Bace2	0	0.456388066	0.68	0.288	0	Fibro_1	Bace2
AI507597	0	0.360256543	0.507	0.072	0	Fibro_1	AI507597
Hhip	0	0.294355997	0.52	0.143	0	Fibro_1	Hhip
Wisp2	8.47E-303	1.317490702	0.626	0.238	1.69E-299	Fibro_1	Wisp2
Abca8a	2.51E-294	0.769136998	0.772	0.369	5.02E-291	Fibro_1	Abca8a
Fibin	4.62E-294	0.760735485	0.666	0.283	9.25E-291	Fibro_1	Fibin
Prss12	1.73E-293	0.337777653	0.564	0.217	3.47E-290	Fibro_1	Prss12
Sparc	5.95E-293	0.711505453	0.998	0.859	1.19E-289	Fibro_1	Sparc
Paqr6	1.38E-290	0.308084444	0.62	0.247	2.75E-287	Fibro_1	Paqr6
Pam	3.98E-288	0.823382721	0.894	0.619	7.96E-285	Fibro_1	Pam
F3	6.33E-287	0.881409526	0.676	0.336	1.27E-283	Fibro_1	F3
Dbp	3.20E-286	0.576678522	0.838	0.566	6.40E-283	Fibro_1	Dbp
Fbln7	8.40E-283	0.33997895	0.585	0.223	1.68E-279	Fibro_1	Fbln7
Prelp	7.95E-282	0.824731828	0.832	0.517	1.59E-278	Fibro_1	Prelp
Igfbp7	1.50E-281	0.534505688	0.984	0.783	3.01E-278	Fibro_1	Igfbp7
Epyc	1.39E-272	0.282069152	0.528	0.175	2.79E-269	Fibro_1	Epyc
Matn2	5.07E-270	0.501452431	0.601	0.237	1.01E-266	Fibro_1	Matn2
Id3	9.83E-262	0.655837283	0.926	0.664	1.97E-258	Fibro_1	Id3
Nupr1	1.62E-259	0.818769068	0.901	0.657	3.24E-256	Fibro_1	Nupr1
Thbs1	6.55E-257	0.335402757	0.517	0.171	1.31E-253	Fibro_1	Thbs1
Gdf10	2.30E-253	0.529069454	0.685	0.35	4.61E-250	Fibro_1	Gdf10
Abi3bp	7.11E-251	0.660742709	0.768	0.423	1.42E-247	Fibro_1	Abi3bp
Timp1	1.51E-241	0.6968621	0.594	0.213	3.03E-238	Fibro_1	Timp1
Dkk3	2.58E-241	0.904082563	0.676	0.322	5.15E-238	Fibro_1	Dkk3
Pcolce	3.27E-236	0.553979438	0.871	0.526	6.55E-233	Fibro_1	Pcolce
Ccl19	5.53E-236	1.00951351	0.618	0.233	1.11E-232	Fibro_1	Ccl19
Islr	2.55E-229	0.436152438	0.862	0.555	5.10E-226	Fibro_1	Islr
Rbp4	3.68E-229	1.130905969	0.618	0.249	7.36E-226	Fibro_1	Rbp4
Gas1	1.16E-226	0.711744711	0.735	0.391	2.32E-223	Fibro_1	Gas1
Tmem119	5.24E-225	0.488922389	0.649	0.316	1.05E-221	Fibro_1	Tmem119
Fxyd6	9.50E-224	0.879156969	0.641	0.281	1.90E-220	Fibro_1	Fxyd6
Meg3	3.88E-223	0.463919088	0.914	0.577	7.76E-220	Fibro_1	Meg3
Fbln5	1.28E-217	0.702840303	0.841	0.523	2.55E-214	Fibro_1	Fbln5
Comp	4.37E-214	0.608150207	0.52	0.212	8.74E-211	Fibro_1	Comp
Col6a2	1.11E-213	0.471025764	0.889	0.582	2.22E-210	Fibro_1	Col6a2
Gas6	5.08E-213	0.662735487	0.845	0.536	1.02E-209	Fibro_1	Gas6
Mfap2	7.38E-210	0.426469966	0.743	0.437	1.48E-206	Fibro_1	Mfap2
Emp1	2.92E-209	0.70279673	0.7	0.383	5.84E-206	Fibro_1	Emp1
Col5a2	5.98E-208	0.471970567	0.818	0.507	1.20E-204	Fibro_1	Col5a2
Tspan11	5.01E-197	0.571098859	0.538	0.197	1.00E-193	Fibro_1	Tspan11
Kcnj8	6.33E-197	0.337807586	0.588	0.264	1.27E-193	Fibro_1	Kcnj8
Entpd2	1.04E-189	0.599309046	0.725	0.413	2.07E-186	Fibro_1	Entpd2
Lamc3	1.69E-189	0.275067659	0.518	0.204	3.37E-186	Fibro_1	Lamc3
Mfap5	1.98E-185	0.445946885	0.9	0.667	3.97E-182	Fibro_1	Mfap5
Nr2f2	9.62E-184	0.610520369	0.65	0.322	1.92E-180	Fibro_1	Nr2f2
Serpinh1	1.53E-181	0.546973326	0.894	0.648	3.06E-178	Fibro_1	Serpinh1
G0s2	1.93E-181	0.436016311	0.608	0.285	3.87E-178	Fibro_1	G0s2
Malat1	3.03E-178	0.361064647	1	0.999	6.05E-175	Fibro_1	Malat1
Cpxm1	3.10E-178	0.355492031	0.657	0.35	6.19E-175	Fibro_1	Cpxm1
Pmp22	4.39E-178	0.519472693	0.799	0.582	8.78E-175	Fibro_1	Pmp22
Igfbp4	7.62E-178	0.49805702	0.943	0.733	1.52E-174	Fibro_1	Igfbp4
Bok	2.17E-177	0.626042684	0.531	0.198	4.34E-174	Fibro_1	Bok
Gpm6b	1.25E-174	0.43048666	0.59	0.252	2.49E-171	Fibro_1	Gpm6b
Fstl1	7.22E-172	0.441235459	0.895	0.612	1.44E-168	Fibro_1	Fstl1
Col6a1	2.21E-171	0.407825933	0.896	0.602	4.41E-168	Fibro_1	Col6a1
Smoc1	5.66E-166	0.511074026	0.59	0.281	1.13E-162	Fibro_1	Smoc1
Plpp1	3.44E-154	0.428338826	0.668	0.366	6.87E-151	Fibro_1	Plpp1
Ctsl	6.47E-154	0.560248922	0.899	0.67	1.29E-150	Fibro_1	Ctsl
Frzb	7.34E-151	0.444448016	0.489	0.186	1.47E-147	Fibro_1	Frzb
Sod3	1.19E-150	0.352181025	0.881	0.597	2.38E-147	Fibro_1	Sod3
Olfml3	1.13E-148	0.650452992	0.611	0.306	2.26E-145	Fibro_1	Olfml3
Sfrp2	3.52E-148	1.319583971	0.69	0.419	7.04E-145	Fibro_1	Sfrp2
Svep1	4.22E-148	0.300814103	0.597	0.31	8.44E-145	Fibro_1	Svep1
Serping1	4.97E-146	0.346762183	0.902	0.643	9.94E-143	Fibro_1	Serping1
F2r	2.95E-136	0.557884643	0.537	0.248	5.89E-133	Fibro_1	F2r
Col6a3	3.17E-134	0.322942891	0.788	0.497	6.35E-131	Fibro_1	Col6a3
Pcolce2	1.68E-132	0.300253057	0.833	0.55	3.36E-129	Fibro_1	Pcolce2
Cd34	1.56E-130	0.308268666	0.803	0.474	3.11E-127	Fibro_1	Cd34
Fbln2	3.69E-130	0.40551855	0.658	0.351	7.39E-127	Fibro_1	Fbln2

Aldh1a1	3.85E-129	0.490503916	0.566	0.276	7.71E-126	Fibro 1	Aldh1a1
Cpxm2	4.31E-126	0.40472704	0.522	0.245	8.63E-123	Fibro 1	Cpxm2
Cilp	8.70E-125	0.731151447	0.507	0.235	1.74E-121	Fibro 1	Cilp
Ptgfr	1.30E-119	0.255364724	0.533	0.253	2.60E-116	Fibro 1	Ptgfr
Atf5	6.96E-119	0.583283505	0.663	0.415	1.39E-115	Fibro 1	Atf5
Gm13305	1.37E-116	0.363285324	0.468	0.174	2.74E-113	Fibro 1	Gm13305
Lox	9.13E-114	0.321376653	0.603	0.325	1.83E-110	Fibro 1	Lox
Cd9	2.03E-109	0.430733334	0.844	0.628	4.06E-106	Fibro 1	Cd9
Meox1	1.75E-104	0.891660564	0.43	0.101	3.49E-101	Fibro 1	Meox1
Cd200	1.78E-101	0.377115026	0.771	0.513	3.55E-98	Fibro 1	Cd200
Akap12	5.62E-100	0.627920459	0.583	0.319	1.12E-96	Fibro 1	Akap12
Col8a1	4.44E-98	0.452713816	0.514	0.274	8.89E-95	Fibro 1	Col8a1
Cped1	5.42E-97	0.375133873	0.666	0.383	1.08E-93	Fibro 1	Cped1
1500015O10Rik	4.50E-96	0.287483137	0.489	0.249	9.00E-93	Fibro 1	1500015O10Rik
Eln	8.52E-92	0.549231585	0.785	0.58	1.70E-88	Fibro 1	Eln
Tmem204	2.90E-90	0.543716029	0.498	0.213	5.81E-87	Fibro 1	Tmem204
Bmp4	1.94E-87	0.410037701	0.503	0.232	3.88E-84	Fibro 1	Bmp4
Adamtsl2	5.30E-86	0.71177137	0.419	0.082	1.06E-82	Fibro 1	Adamtsl2
Osr1	7.53E-84	0.253992098	0.684	0.421	1.51E-80	Fibro 1	Osr1
Tuba1a	1.74E-80	0.387091619	0.773	0.617	3.48E-77	Fibro 1	Tuba1a
Dio2	1.07E-78	0.749328387	0.423	0.109	2.14E-75	Fibro 1	Dio2
Fgl2	4.22E-78	0.333854503	0.632	0.38	8.43E-75	Fibro 1	Fgl2
Gng11	7.54E-75	0.25006658	0.662	0.424	1.51E-71	Fibro 1	Gng11
Hand2	4.61E-74	0.314946243	0.417	0.151	9.21E-71	Fibro 1	Hand2
Papss2	6.09E-70	0.423850439	0.472	0.214	1.22E-66	Fibro 1	Papss2
Anxa2	1.25E-68	0.270703945	0.836	0.649	2.51E-65	Fibro 1	Anxa2
Fmo2	9.02E-59	0.436704906	0.612	0.386	1.80E-55	Fibro 1	Fmo2
Figf	2.10E-53	0.268975697	0.514	0.288	4.19E-50	Fibro 1	Figf
Abcc9	2.94E-49	0.585280933	0.515	0.289	5.88E-46	Fibro 1	Abcc9
Prg4	3.93E-45	0.351877722	0.398	0.17	7.86E-42	Fibro 1	Prg4
Dclk1	5.86E-45	0.302619086	0.443	0.212	1.17E-41	Fibro 1	Dclk1
Prrx2	4.23E-38	0.256894371	0.444	0.23	8.46E-35	Fibro 1	Prrx2
Phlda1	4.03E-25	0.376674024	0.546	0.381	8.07E-22	Fibro 1	Phlda1
Cp	3.27E-20	0.338178686	0.474	0.278	6.54E-17	Fibro 1	Cp
Crlf1	8.13E-17	0.252614695	0.352	0.158	1.63E-13	Fibro 1	Crlf1
Hes1	5.02E-16	0.340856878	0.461	0.293	1.00E-12	Fibro 1	Hes1
Wif1	1.73E-15	0.357683771	0.472	0.338	3.45E-12	Fibro 1	Wif1
Serpina3n	1.07E-12	0.260961162	0.358	0.162	2.13E-09	Fibro 1	Serpina3n
Scn7a	1.85E-12	0.271129302	0.447	0.259	3.70E-09	Fibro 1	Scn7a
Vcan	2.29E-12	0.293116724	0.441	0.284	4.57E-09	Fibro 1	Vcan
Heyl	2.37E-08	0.284228366	0.345	0.255	4.73E-05	Fibro 1	Heyl
Ctgf	9.23E-08	0.423498239	0.385	0.213	0.000184597	Fibro 1	Ctgf
Dbi	2.45E-06	0.285067252	0.767	0.733	0.004890666	Fibro 1	Dbi
Tbx20	3.14E-06	0.62667369	0.324	0.12	0.006287085	Fibro 1	Tbx20
Vcam1	0.000177978	0.286145428	0.409	0.262	0.355955797	Fibro 1	Vcam1
Gm2564	0.009391053	0.423290229	0.332	0.128	1	Fibro 1	Gm2564
Gpx3	0	2.032090762	0.953	0.503	0	Fibro 2	Gpx3
Ccl11	0	1.482577868	0.787	0.358	0	Fibro 2	Ccl11
Dpep1	0	1.402289957	0.877	0.318	0	Fibro 2	Dpep1
Penk	0	1.330403776	0.719	0.267	0	Fibro 2	Penk
C3	0	1.324092905	0.956	0.486	0	Fibro 2	C3
Inmt	0	1.310519544	0.844	0.422	0	Fibro 2	Inmt
Gsn1	0	1.273850217	1	0.933	0	Fibro 2	Gsn
Sfrp1	0	1.272806552	0.702	0.236	0	Fibro 2	Sfrp1
Lpl	0	1.19911551	0.819	0.436	0	Fibro 2	Lpl
Abca8a1	0	1.140835415	0.906	0.355	0	Fibro 2	Abca8a
Pcolce1	0	1.110229724	0.921	0.522	0	Fibro 2	Pcolce
Pdgfra1	0	1.062385145	0.888	0.431	0	Fibro 2	Pdgfra
Scara5	0	1.036482019	0.871	0.411	0	Fibro 2	Scara5
Clec3b	0	1.029532491	0.982	0.546	0	Fibro 2	Clec3b
Fbln11	0	1.005857548	0.914	0.475	0	Fibro 2	Fbln1
Serping11	0	0.975972366	0.977	0.636	0	Fibro 2	Serping1
C1s1	0	0.971673069	0.889	0.439	0	Fibro 2	C1s1
Cygb1	0	0.970956256	0.861	0.366	0	Fibro 2	Cygb
Srpx	0	0.968184495	0.652	0.293	0	Fibro 2	Srpx
Igfbp41	0	0.966181179	0.983	0.73	0	Fibro 2	Igfbp4
Dcn1	0	0.958882756	1	0.844	0	Fibro 2	Dcn
Smoc21	0	0.944907365	0.759	0.334	0	Fibro 2	Smoc2
Col3a11	0	0.935320054	0.995	0.792	0	Fibro 2	Col3a1
Gas11	0	0.931846979	0.794	0.386	0	Fibro 2	Gas1
Igf11	0	0.906724219	0.792	0.451	0	Fibro 2	Igf1
Nid1	0	0.905699864	0.905	0.482	0	Fibro 2	Nid1
Mgst1	0	0.904277485	0.862	0.507	0	Fibro 2	Mgst1
Gdf101	0	0.893785958	0.709	0.348	0	Fibro 2	Gdf10
Entpd21	0	0.88263337	0.816	0.404	0	Fibro 2	Entpd2
Svep11	0	0.876526911	0.718	0.298	0	Fibro 2	Svep1
Ly6a	0	0.863610411	0.949	0.63	0	Fibro 2	Ly6a
Plac8	0	0.849644542	0.764	0.393	0	Fibro 2	Plac8
Dpt1	0	0.831241308	0.99	0.582	0	Fibro 2	Dpt
Col1a21	0	0.830233864	0.998	0.774	0	Fibro 2	Col1a2
Scn7a1	0	0.822246065	0.68	0.234	0	Fibro 2	Scn7a
Lum1	0	0.817219251	0.933	0.546	0	Fibro 2	Lum
Gpc3	0	0.782596133	0.746	0.405	0	Fibro 2	Gpc3
Serpinf11	0	0.77862228	0.898	0.558	0	Fibro 2	Serpinf1
Col1a11	0	0.765783723	0.979	0.711	0	Fibro 2	Col1a1
C1ra	0	0.741217142	0.768	0.431	0	Fibro 2	C1ra
Sult5a1	0	0.712971765	0.634	0.248	0	Fibro 2	Sult5a1
Adm	0	0.710890112	0.627	0.269	0	Fibro 2	Adm
Olfml31	0	0.687722103	0.686	0.299	0	Fibro 2	Olfml3
Mmp21	0	0.663165188	0.887	0.562	0	Fibro 2	Mmp2
Col6a11	0	0.65663866	0.91	0.602	0	Fibro 2	Col6a1
Tmem100	0	0.654173234	0.7	0.294	0	Fibro 2	Tmem100
Cpxm11	0	0.640658515	0.681	0.348	0	Fibro 2	Cpxm1
Meg31	0	0.637892074	0.941	0.575	0	Fibro 2	Meg3
Plpp3	0	0.630032758	0.909	0.587	0	Fibro 2	Plpp3
Pcolce21	0	0.629472239	0.891	0.545	0	Fibro 2	Pcolce2
Cd341	0	0.60680417	0.87	0.468	0	Fibro 2	Cd34
Rarres2	0	0.580707837	0.943	0.661	0	Fibro 2	Rarres2
Ly6c1	0	0.540469256	0.784	0.382	0	Fibro 2	Ly6c1
Ftl1	0	0.369428101	1	0.992	0	Fibro 2	Ftl1
Ccdc801	3.89E-275	0.668453713	0.826	0.533	7.79E-272	Fibro 2	Ccdc80
Itm2a	9.73E-270	0.63423767	0.71	0.446	1.95E-266	Fibro 2	Itm2a
Steap4	7.57E-262	0.758796963	0.531	0.205	1.51E-258	Fibro 2	Steap4
Serpinh11	2.97E-261	0.606964635	0.913	0.647	5.94E-258	Fibro 2	Serpinh1
Gstm1	1.34E-237	0.510501652	0.94	0.71	2.67E-234	Fibro 2	Gstm1
Col14a1	2.96E-233	0.475082073	0.846	0.527	5.93E-230	Fibro 2	Col14a1
Hmcn2	1.55E-230	0.348358201	0.525	0.258	3.11E-227	Fibro 2	Hmcn2
Pcsk6	1.44E-228	0.441166068	0.677	0.397	2.88E-225	Fibro 2	Pcsk6
Fst	1.38E-226	0.58494915	0.57	0.26	2.76E-223	Fibro 2	Fst
Lbp	3.85E-225	0.307731088	0.551	0.282	7.71E-222	Fibro 2	Lbp
Fndc1	3.49E-224	0.439080404	0.726	0.444	6.98E-221	Fibro 2	Fndc1
Cd248	7.18E-224	0.31638099	0.76	0.465	1.44E-220	Fibro 2	Cd248
Adh1	7.08E-222	0.400247428	0.55	0.254	1.42E-218	Fibro 2	Adh1
Lrrn4cl	2.57E-221	0.310907413	0.62	0.345	5.14E-218	Fibro 2	Lrrn4cl
Angptl1	3.08E-214	0.302190788	0.542	0.29	6.15E-211	Fibro 2	Angptl1
Fbln21	5.59E-214	0.619086248	0.681	0.349	1.12E-210	Fibro 2	Fbln2
Cfb	9.21E-209	0.250676556	0.499	0.236	1.84E-205	Fibro 2	Cfb
Cxcl12	2.25E-205	0.959722812	0.645	0.367	4.49E-202	Fibro 2	Cxcl12
Pi16	4.46E-205	0.25619364	0.882	0.663	8.92E-202	Fibro 2	Pi16
Aebp1	9.78E-199	0.484959649	0.905	0.619	1.96E-195	Fibro 2	Aebp1
Cp1	3.56E-191	0.710507498	0.573	0.268	7.12E-188	Fibro 2	Cp
Ramp2	5.09E-190	0.385530542	0.68	0.366	1.02E-186	Fibro 2	Ramp2
Col6a21	7.65E-190	0.494701407	0.847	0.587	1.53E-186	Fibro 2	Col6a2
Lamb1	5.16E-188	0.381281606	0.572	0.302	1.03E-184	Fibro 2	Lamb1
G0s21	3.65E-187	0.498737943	0.551	0.293	7.29E-184	Fibro 2	G0s2
Ptgfr1	1.94E-185	0.315507935	0.528	0.254	3.88E-182	Fibro 2	Ptgfr
Adamts5	6.82E-185	0.395457368	0.7	0.402	1.36E-181	Fibro 2	Adamts5
Fn1	5.86E-179	0.477084801	0.876	0.623	1.17E-175	Fibro 2	Fn1
Mfap51	2.55E-175	0.310529185	0.903	0.668	5.10E-172	Fibro 2	Mfap5

Bdh2	3.00E-164	0.284076437	0.521	0.272	6.00E-161	Fibro 2	Bdh2
Plat	3.55E-164	0.278466671	0.645	0.396	7.10E-161	Fibro 2	Plat
Ctsl1	5.58E-161	0.512073129	0.884	0.672	1.12E-157	Fibro 2	Ctsl
Mfap21	1.02E-159	0.391321748	0.684	0.445	2.04E-156	Fibro 2	Mfap2
Mt1	8.29E-159	0.510668662	0.919	0.74	1.66E-155	Fibro 2	Mt1
C4b	3.11E-156	0.623639427	0.598	0.316	6.23E-153	Fibro 2	C4b
Col5a21	1.32E-153	0.424351256	0.766	0.514	2.64E-150	Fibro 2	Col5a2
Qpct	2.21E-149	0.384464987	0.664	0.441	4.42E-146	Fibro 2	Qpct
Tmsb10	9.51E-149	0.279816528	0.998	0.945	1.90E-145	Fibro 2	Tmsb10
Islr1	1.97E-146	0.461434459	0.781	0.565	3.94E-143	Fibro 2	Islr
Phlda11	1.89E-145	0.776674053	0.612	0.374	3.78E-142	Fibro 2	Phlda1
Col6a31	5.67E-142	0.368256019	0.743	0.503	1.13E-138	Fibro 2	Col6a3
Cfh1	6.90E-141	0.416729037	0.816	0.58	1.38E-137	Fibro 2	Cfh
Slfn5	6.38E-139	0.273746499	0.42	0.173	1.28E-135	Fibro 2	Slfn5
Thbd	1.39E-133	0.382615571	0.606	0.344	2.78E-130	Fibro 2	Thbd
Serpina3n1	1.78E-133	0.46920592	0.43	0.155	3.56E-130	Fibro 2	Serpina3n
Fstl11	7.58E-127	0.314451814	0.888	0.614	1.52E-123	Fibro 2	Fstl1
Htra1	1.65E-126	0.363014062	0.757	0.524	3.29E-123	Fibro 2	Htra1
Pdpn	1.63E-121	0.305330851	0.513	0.266	3.26E-118	Fibro 2	Pdpn
Rbp11	2.54E-120	0.319692214	0.85	0.614	5.09E-117	Fibro 2	Rbp1
Ifi205	3.21E-119	0.641398967	0.45	0.185	6.43E-116	Fibro 2	Ifi205
AW112010	3.75E-116	0.413478609	0.61	0.385	7.49E-113	Fibro 2	AW112010
Akap121	2.12E-115	0.454783472	0.577	0.321	4.25E-112	Fibro 2	Akap12
Cdkn1c	1.13E-113	0.270668394	0.61	0.381	2.25E-110	Fibro 2	Cdkn1c
Apod	5.52E-113	0.565926865	0.522	0.3	1.10E-109	Fibro 2	Apod
Ugdh	2.86E-110	0.287374004	0.621	0.393	5.71E-107	Fibro 2	Ugdh
Gfpt2	2.22E-101	0.443189348	0.518	0.262	4.44E-98	Fibro 2	Gfpt2
Enpp2	1.73E-92	0.303136766	0.473	0.284	3.45E-89	Fibro 2	Enpp2
Ifi27l2a	9.14E-87	0.28189797	0.892	0.756	1.83E-83	Fibro 2	Ifi27l2a
Ccl191	2.38E-86	0.689857864	0.482	0.25	4.75E-83	Fibro 2	Ccl19
AI607873	1.49E-85	0.392066322	0.409	0.173	2.98E-82	Fibro 2	AI607873
Osr11	3.90E-85	0.443793133	0.618	0.429	7.79E-82	Fibro 2	Osr1
Smim1	2.15E-84	0.271778339	0.528	0.327	4.30E-81	Fibro 2	Smim1
Slc1a3	2.65E-79	0.291056781	0.383	0.154	5.31E-76	Fibro 2	Slc1a3
Tnfsf13b	6.56E-76	0.293076387	0.384	0.169	1.31E-72	Fibro 2	Tnfsf13b
F31	2.96E-72	0.327869597	0.519	0.355	5.92E-69	Fibro 2	F3
Ifi204	7.96E-72	0.391312945	0.423	0.197	1.59E-68	Fibro 2	Ifi204
Vcam11	1.71E-71	0.282267957	0.45	0.258	3.42E-68	Fibro 2	Vcam1
Ntrk2	2.07E-71	0.291304041	0.512	0.339	4.13E-68	Fibro 2	Ntrk2
S1pr3	2.24E-71	0.370514321	0.378	0.175	4.49E-68	Fibro 2	S1pr3
Gpm6b1	9.67E-69	0.299039423	0.467	0.267	1.93E-65	Fibro 2	Gpm6b
Sfrp4	8.66E-68	0.361942521	0.535	0.391	1.73E-64	Fibro 2	Sfrp4
Ecm1	1.03E-65	0.360080668	0.771	0.614	2.06E-62	Fibro 2	Ecm1
Errfi1	2.75E-65	0.449318901	0.462	0.262	5.50E-62	Fibro 2	Errfi1
Col15a11	1.77E-64	0.35219929	0.596	0.416	3.53E-61	Fibro 2	Col15a1
Hbb-bs	1.87E-61	0.436729648	0.839	0.706	3.75E-58	Fibro 2	Hbb-bs
Hba-a2	1.14E-59	0.418600566	0.691	0.499	2.28E-56	Fibro 2	Hba-a2
Adgrd1	2.86E-57	0.373012764	0.502	0.334	5.73E-54	Fibro 2	Adgrd1
Figf1	1.62E-55	0.35352563	0.475	0.293	3.25E-52	Fibro 2	Figf
Mt2	5.80E-55	0.447822905	0.614	0.455	1.16E-51	Fibro 2	Mt2
Socs3	1.19E-54	0.410420546	0.38	0.229	2.38E-51	Fibro 2	Socs3
Abi3bp1	8.87E-54	0.279855038	0.604	0.442	1.77E-50	Fibro 2	Abi3bp
Sned1	1.12E-49	0.436372928	0.405	0.239	2.24E-46	Fibro 2	Sned1
Sult1e1	1.59E-40	0.658315348	0.323	0.126	3.19E-37	Fibro 2	Sult1e1
Hba-a1	2.04E-40	0.433008974	0.704	0.545	4.09E-37	Fibro 2	Hba-a1
Cxcl14	1.29E-39	0.898900869	0.298	0.115	2.58E-36	Fibro 2	Cxcl14
Jun	3.20E-39	0.586858731	0.611	0.516	6.39E-36	Fibro 2	Jun
Abcc91	8.91E-38	0.276012933	0.453	0.296	1.78E-34	Fibro 2	Abcc9
Egr1	1.00E-32	0.500093926	0.513	0.411	2.01E-29	Fibro 2	Egr1
Il33	4.07E-32	0.411662164	0.426	0.241	8.14E-29	Fibro 2	Il33
Ier2	3.51E-24	0.512585411	0.617	0.554	7.03E-21	Fibro 2	Ier2
Dclk11	1.25E-23	0.260168037	0.373	0.22	2.50E-20	Fibro 2	Dclk1
Sox9	8.74E-22	0.258440346	0.364	0.234	1.75E-18	Fibro 2	Sox9
Gas61	4.31E-21	0.275966064	0.65	0.559	8.61E-18	Fibro 2	Gas6
Junb	6.80E-21	0.636603388	0.663	0.622	1.36E-17	Fibro 2	Junb
Epyc1	2.98E-15	0.290826219	0.35	0.196	5.95E-12	Fibro 2	Epyc
Igfbp3	3.33E-10	0.389633296	0.342	0.242	6.66E-07	Fibro 2	Igfbp3
Mnda	0.000635577	0.296057882	0.29	0.177	1	Fibro 2	Mnda
Apod1	0	1.765377402	0.829	0.292	0	Fibro 3	Apod
Nbl1	0	1.725145906	0.965	0.547	0	Fibro 3	Nbl1
Inmt1	0	1.724066111	0.9	0.438	0	Fibro 3	Inmt
Mgp1	0	1.545579455	0.999	0.955	0	Fibro 3	Mgp
Crispld21	0	1.250825362	0.868	0.44	0	Fibro 3	Crispld2
Col1a12	0	1.235420034	0.995	0.722	0	Fibro 3	Col1a1
Mmp22	0	1.221362131	0.954	0.573	0	Fibro 3	Mmp2
Serpinf12	0	1.178244395	0.925	0.572	0	Fibro 3	Serpinf1
Sparcl1	0	1.128024751	0.824	0.392	0	Fibro 3	Sparcl1
Col3a12	0	1.083716823	1	0.801	0	Fibro 3	Col3a1
Sparc1	0	1.078017376	0.997	0.866	0	Fibro 3	Sparc
Adh7	0	1.051943648	0.648	0.19	0	Fibro 3	Adh7
Plpp31	0	1.022227297	0.953	0.599	0	Fibro 3	Plpp3
Gsn2	0	1.014032959	1	0.936	0	Fibro 3	Gsn
Ptgfr2	0	0.98433453	0.754	0.253	0	Fibro 3	Ptgfr
Col1a22	0	0.98021844	0.997	0.784	0	Fibro 3	Col1a2
Igfbp6	0	0.961684392	0.963	0.556	0	Fibro 3	Igfbp6
Col6a32	0	0.940913551	0.881	0.505	0	Fibro 3	Col6a3
Adh11	0	0.92585613	0.72	0.258	0	Fibro 3	Adh1
Ntrk21	0	0.912382743	0.739	0.333	0	Fibro 3	Ntrk2
Col6a12	0	0.898609687	0.966	0.612	0	Fibro 3	Col6a1
Col6a22	0	0.885842486	0.942	0.593	0	Fibro 3	Col6a2
Dpt2	0	0.85541166	0.99	0.601	0	Fibro 3	Dpt
Ces1f	0	0.332186127	0.405	0.05	0	Fibro 3	Ces1f
Fbln12	2.92E-304	0.907090627	0.926	0.494	5.83E-301	Fibro 3	Fbln1
Serping12	3.17E-299	0.870767333	0.978	0.651	6.35E-296	Fibro 3	Serping1
Ctsk	4.05E-295	0.98731654	0.768	0.43	8.10E-292	Fibro 3	Ctsk
Aldh3a1	6.85E-295	0.650136348	0.582	0.206	1.37E-291	Fibro 3	Aldh3a1
C1qtnf3	5.75E-282	0.606972236	0.514	0.156	1.15E-278	Fibro 3	C1qtnf3
Bgn1	1.13E-277	0.768940182	0.993	0.755	2.27E-274	Fibro 3	Bgn
Col23a1	5.65E-274	1.222590665	0.609	0.229	1.13E-270	Fibro 3	Col23a1
Pdgfrl1	2.33E-272	0.787185178	0.721	0.355	4.66E-269	Fibro 3	Pdgfrl
Mfap22	4.82E-263	0.811407767	0.807	0.448	9.64E-260	Fibro 3	Mfap2
Osr2	5.18E-257	0.517874819	0.601	0.257	1.04E-253	Fibro 3	Osr2
Clec3b1	2.62E-255	0.675660333	0.975	0.567	5.24E-252	Fibro 3	Clec3b
Ppic	2.83E-247	0.804552064	0.835	0.512	5.65E-244	Fibro 3	Ppic
Hhip1	2.67E-242	0.535599497	0.508	0.162	5.34E-239	Fibro 3	Hhip
Pdgfra2	7.25E-238	0.662816012	0.863	0.453	1.45E-234	Fibro 3	Pdgfra
Mfap41	1.69E-234	0.959599217	0.876	0.518	3.38E-231	Fibro 3	Mfap4
Pappa2	5.39E-229	0.38091584	0.45	0.081	1.08E-225	Fibro 3	Pappa2
Dcn2	1.67E-226	0.931111986	0.993	0.851	3.33E-223	Fibro 3	Dcn
Abi3bp2	8.71E-220	0.670781742	0.816	0.437	1.74E-216	Fibro 3	Abi3bp
Ces1d	1.93E-217	0.909795331	0.582	0.254	3.86E-214	Fibro 3	Ces1d
Abca8a2	1.59E-212	0.608290066	0.825	0.385	3.17E-209	Fibro 3	Abca8a
Gdf102	3.94E-207	0.725665896	0.7	0.365	7.87E-204	Fibro 3	Gdf10
Pcolce3	8.55E-207	0.728693927	0.889	0.542	1.71E-203	Fibro 3	Pcolce
Serpinh12	2.02E-206	0.64837506	0.934	0.658	4.04E-203	Fibro 3	Serpinh1
C1s11	6.27E-204	0.54373298	0.853	0.462	1.25E-200	Fibro 3	C1s1
Olfml32	7.51E-197	0.706001637	0.686	0.316	1.50E-193	Fibro 3	Olfml3
Col5a22	1.81E-196	0.6763678	0.836	0.521	3.62E-193	Fibro 3	Col5a2
Dlk1	1.62E-190	0.539984755	0.472	0.184	3.23E-187	Fibro 3	Dlk1
Myoc	1.04E-181	0.59005607	0.576	0.303	2.09E-178	Fibro 3	Myoc
Faim2	5.13E-180	0.714833053	0.538	0.201	1.03E-176	Fibro 3	Faim2
Gpm6b2	5.96E-180	0.540445746	0.639	0.265	1.19E-176	Fibro 3	Gpm6b
Clec11a1	8.28E-178	0.586845344	0.641	0.308	1.66E-174	Fibro 3	Clec11a
Bmp41	6.70E-177	0.390967129	0.58	0.24	1.34E-173	Fibro 3	Bmp4
Itm2a1	2.22E-174	0.626716836	0.734	0.457	4.44E-171	Fibro 3	Itm2a
Fmo21	9.85E-171	0.650640596	0.768	0.387	1.97E-167	Fibro 3	Fmo2
Aqp1	1.58E-170	1.131236283	0.655	0.342	3.16E-167	Fibro 3	Aqp1
Fxyd61	6.32E-170	0.668496528	0.683	0.296	1.26E-166	Fibro 3	Fxyd6
Col15a12	7.20E-170	0.59668041	0.76	0.414	1.44E-166	Fibro 3	Col15a1

Cdh4	2.72E-169	0.505376322	0.48	0.151	5.44E-166	Fibro	3	Cdh4
Timp31	1.35E-167	0.774339042	0.933	0.686	2.70E-164	Fibro	3	Timp3
Dkk2	1.45E-160	0.500197721	0.555	0.301	2.89E-157	Fibro	3	Dkk2
Cdkn1c1	4.27E-152	0.493449887	0.711	0.385	8.55E-149	Fibro	3	Cdkn1c
Tmem1191	4.32E-150	0.523974764	0.64	0.333	8.63E-147	Fibro	3	Tmem119
Cygb2	1.16E-149	0.639477589	0.765	0.394	2.32E-146	Fibro	3	Cygb
Gpc31	2.06E-148	0.473791909	0.732	0.421	4.12E-145	Fibro	3	Gpc3
C31	1.35E-147	0.385064261	0.856	0.514	2.69E-144	Fibro	3	C3
Rbp12	4.47E-147	0.498427132	0.911	0.621	8.95E-144	Fibro	3	Rbp1
Mgst11	9.03E-145	0.472309904	0.845	0.525	1.81E-141	Fibro	3	Mgst1
S100a6	9.51E-144	0.385646788	1	0.901	1.90E-140	Fibro	3	S100a6
Tmem2041	1.63E-142	0.444567684	0.548	0.224	3.26E-139	Fibro	3	Tmem204
Ctsl2	6.71E-141	0.627859967	0.895	0.681	1.34E-137	Fibro	3	Ctsl
Figf2	1.47E-140	0.545409015	0.61	0.294	2.93E-137	Fibro	3	Figf
C1ra1	1.60E-139	0.465747921	0.74	0.448	3.20E-136	Fibro	3	C1ra
Ednra	6.08E-135	0.429730979	0.52	0.223	1.22E-131	Fibro	3	Ednra
Spon2	1.37E-134	0.919055794	0.513	0.179	2.74E-131	Fibro	3	Spon2
P2ry14	4.47E-134	0.269196215	0.486	0.232	8.94E-131	Fibro	3	P2ry14
Aebp11	1.93E-133	0.484203655	0.921	0.631	3.86E-130	Fibro	3	Aebp1
Itgbl11	8.44E-133	0.344329078	0.666	0.357	1.69E-129	Fibro	3	Itgbl1
Cd342	3.06E-131	0.376043417	0.87	0.486	6.12E-128	Fibro	3	Cd34
Cpxm12	1.23E-130	0.478711218	0.649	0.365	2.45E-127	Fibro	3	Cpxm1
Igfbp42	1.27E-129	0.381637484	0.977	0.741	2.55E-126	Fibro	3	Igfbp4
Hmcn1	2.25E-129	0.407530024	0.49	0.192	4.50E-126	Fibro	3	Hmcn1
Igfbp71	6.90E-129	0.326426335	0.992	0.793	1.38E-125	Fibro	3	Igfbp7
Meg32	1.45E-127	0.529186461	0.903	0.594	2.90E-124	Fibro	3	Meg3
Cyp2e1	2.71E-126	0.536472992	0.447	0.186	5.41E-123	Fibro	3	Cyp2e1
Cxcl121	7.30E-126	0.731427299	0.691	0.377	1.46E-122	Fibro	3	Cxcl12
Eln1	2.12E-122	0.277934941	0.886	0.584	4.24E-119	Fibro	3	Eln
Fez1	2.13E-118	0.293624091	0.619	0.369	4.26E-115	Fibro	3	Fez1
Gpnmb	1.45E-115	0.372034261	0.5	0.231	2.89E-112	Fibro	3	Gpnmb
Emp11	2.02E-115	0.520496107	0.704	0.398	4.04E-112	Fibro	3	Emp1
Pdpn1	2.92E-114	0.531116025	0.587	0.273	5.84E-111	Fibro	3	Pdpn
Cdh11	5.20E-114	0.362561362	0.486	0.202	1.04E-110	Fibro	3	Cdh11
Col14a11	5.81E-112	0.325981951	0.827	0.542	1.16E-108	Fibro	3	Col14a1
Scara51	5.43E-111	0.332267265	0.751	0.44	1.09E-107	Fibro	3	Scara5
Aldh1a11	9.65E-110	0.349359053	0.568	0.29	1.93E-106	Fibro	3	Aldh1a1
Rspo1	2.06E-108	0.326308023	0.441	0.146	4.12E-105	Fibro	3	Rspo1
Igfbp31	4.08E-107	0.568198614	0.478	0.239	8.17E-104	Fibro	3	Igfbp3
Svep12	2.38E-106	0.288877788	0.582	0.325	4.76E-103	Fibro	3	Svep1
Spon1	2.60E-106	0.862151649	0.493	0.2	5.20E-103	Fibro	3	Spon1
S100a4	5.11E-103	0.515782165	0.791	0.562	1.02E-99	Fibro	3	S100a4
Tspan111	1.88E-101	0.441249445	0.5	0.216	3.77E-98	Fibro	3	Tspan11
Selenbp1	1.65E-100	0.289994996	0.65	0.399	3.30E-97	Fibro	3	Selenbp1
Gas12	3.16E-99	0.438335233	0.728	0.408	6.32E-96	Fibro	3	Gas1
Lamb11	8.40E-99	0.417261586	0.564	0.315	1.68E-95	Fibro	3	Lamb1
Lum2	3.09E-98	0.425895043	0.849	0.569	6.17E-95	Fibro	3	Lum
Ccdc802	1.27E-97	0.298058242	0.818	0.547	2.55E-94	Fibro	3	Ccdc80
Loxl2	3.15E-97	0.320294583	0.588	0.341	6.31E-94	Fibro	3	Loxl2
Islr2	5.88E-95	0.322447782	0.807	0.574	1.18E-91	Fibro	3	Islr
Col4a6	2.37E-93	0.319766231	0.551	0.297	4.74E-90	Fibro	3	Col4a6
Ptn	6.05E-91	0.330955719	0.505	0.286	1.21E-87	Fibro	3	Ptn
Angptl11	2.38E-89	0.311375971	0.534	0.302	4.77E-86	Fibro	3	Angptl1
Lox1	6.21E-89	0.621156757	0.588	0.34	1.24E-85	Fibro	3	Lox
Atf51	7.24E-88	0.408364908	0.695	0.425	1.45E-84	Fibro	3	Atf5
Efemp1	2.80E-87	0.256265733	0.62	0.366	5.61E-84	Fibro	3	Efemp1
Cdo1	4.37E-87	0.281027073	0.464	0.206	8.74E-84	Fibro	3	Cdo1
Sod31	5.55E-87	0.36568779	0.877	0.611	1.11E-83	Fibro	3	Sod3
Abcc92	1.34E-85	0.279120975	0.553	0.298	2.67E-82	Fibro	3	Abcc9
Stc1	6.30E-84	0.409075452	0.448	0.208	1.26E-80	Fibro	3	Stc1
Aspn	1.14E-83	0.361062039	0.67	0.418	2.27E-80	Fibro	3	Aspn
Vcan1	2.12E-83	0.382785331	0.546	0.285	4.25E-80	Fibro	3	Vcan
Serpina3n2	4.06E-78	0.453070641	0.451	0.166	8.12E-75	Fibro	3	Serpina3n
Fbln22	3.43E-76	0.281459904	0.652	0.366	6.86E-73	Fibro	3	Fbln2
Pmp221	1.23E-75	0.325448222	0.808	0.592	2.47E-72	Fibro	3	Pmp22
Aqp2	8.08E-75	0.31706027	0.365	0.126	1.62E-71	Fibro	3	Aqp2
Gas62	1.59E-74	0.305409796	0.819	0.553	3.19E-71	Fibro	3	Gas6
Cd91	1.14E-73	0.326855101	0.86	0.637	2.28E-70	Fibro	3	Cd9
Nupr11	1.50E-72	0.317189858	0.89	0.67	3.01E-69	Fibro	3	Nupr1
Sned11	1.06E-69	0.371496604	0.46	0.243	2.12E-66	Fibro	3	Sned1
Phlda12	5.71E-68	0.498377613	0.613	0.385	1.14E-64	Fibro	3	Phlda1
Ism1	3.51E-67	0.502498948	0.427	0.174	7.01E-64	Fibro	3	Ism1
Plpp11	2.47E-66	0.279634952	0.609	0.385	4.95E-63	Fibro	3	Plpp1
Hsd11b1	1.31E-64	0.736561506	0.467	0.261	2.62E-61	Fibro	3	Hsd11b1
Egr11	1.94E-62	0.480760093	0.629	0.408	3.88E-59	Fibro	3	Egr1
Nr4a3	3.09E-62	0.314006842	0.345	0.133	6.18E-59	Fibro	3	Nr4a3
Cpxm21	7.12E-61	0.347704182	0.469	0.261	1.42E-57	Fibro	3	Cpxm2
Hmgcs2	7.72E-61	0.289379232	0.454	0.222	1.54E-57	Fibro	3	Hmgcs2
1-Jun	1.24E-59	0.432222374	0.715	0.514	2.47E-56	Fibro	3	Jun
C4b1	2.05E-59	0.476040911	0.569	0.33	4.10E-56	Fibro	3	C4b
Gem	3.62E-58	0.472164656	0.575	0.37	7.25E-55	Fibro	3	Gem
Dusp1	6.54E-54	0.469988195	0.679	0.487	1.31E-50	Fibro	3	Dusp1
Tuba1a1	2.06E-52	0.278028958	0.804	0.623	4.12E-49	Fibro	3	Tuba1a
Cilp1	7.26E-50	0.431418893	0.434	0.253	1.45E-46	Fibro	3	Cilp
Prrx21	1.15E-49	0.43016759	0.461	0.239	2.31E-46	Fibro	3	Prrx2
Nrep	8.73E-49	0.299747606	0.545	0.392	1.75E-45	Fibro	3	Nrep
Txndc5	2.80E-48	0.264684218	0.63	0.445	5.61E-45	Fibro	3	Txndc5
Id31	4.01E-48	0.314028076	0.879	0.679	8.02E-45	Fibro	3	Id3
Ifitm1	1.73E-47	0.579208941	0.345	0.135	3.46E-44	Fibro	3	Ifitm1
Adm1	1.89E-47	0.282307448	0.48	0.294	3.78E-44	Fibro	3	Adm
Wisp1	9.26E-46	0.461143925	0.418	0.2	1.85E-42	Fibro	3	Wisp1
Fos	1.58E-44	0.281687512	0.677	0.498	3.16E-41	Fibro	3	Fos
Ier21	9.96E-41	0.460259613	0.697	0.552	1.99E-37	Fibro	3	Ier2
Cox6b2	1.35E-39	0.288068632	0.44	0.279	2.70E-36	Fibro	3	Cox6b2
Mme	5.97E-39	0.28251844	0.38	0.186	1.19E-35	Fibro	3	Mme
Vcam12	4.03E-36	0.389027962	0.455	0.266	8.05E-33	Fibro	3	Vcam1
Bdh21	4.55E-35	0.267688419	0.458	0.287	9.11E-32	Fibro	3	Bdh2
Il331	9.95E-32	0.37280773	0.45	0.248	1.99E-28	Fibro	3	Il33
Junb1	1.12E-30	0.348474334	0.752	0.619	2.25E-27	Fibro	3	Junb
Pamr1	9.33E-26	0.251292503	0.488	0.36	1.87E-22	Fibro	3	Pamr1
Mmp3	1.18E-25	0.626589425	0.311	0.133	2.36E-22	Fibro	3	Mmp3
Tnfsf13b1	9.77E-23	0.354194353	0.356	0.181	1.95E-19	Fibro	3	Tnfsf13b
4-Sep	8.00E-22	0.25533493	0.375	0.23	1.60E-18	Fibro	3	4-Sep
Cgnl1	3.16E-19	0.299559286	0.387	0.238	6.33E-16	Fibro	3	Cgnl1
F2r1	3.19E-11	0.267263811	0.377	0.272	6.39E-08	Fibro	3	F2r
Pi161	0	2.151122753	0.957	0.672	0	Fibro	4	Pi16
Clec3b2	0	1.701412864	0.999	0.569	0	Fibro	4	Clec3b
Mfap52	0	1.627095457	0.996	0.676	0	Fibro	4	Mfap5
Igfbp61	0	1.560240349	0.971	0.56	0	Fibro	4	Igfbp6
Ly6a1	0	1.490141065	0.986	0.646	0	Fibro	4	Ly6a
Gsn3	0	1.476672671	1	0.936	0	Fibro	4	Gsn
Serping13	0	1.472938601	1	0.654	0	Fibro	4	Serping1
Anxa1	0	1.462501856	0.972	0.59	0	Fibro	4	Anxa1
Dcn3	0	1.422816162	1	0.852	0	Fibro	4	Dcn
Pcolce22	0	1.409770128	0.972	0.56	0	Fibro	4	Pcolce2
Ly6c11	0	1.359458876	0.92	0.397	0	Fibro	4	Ly6c1
Serpinf13	0	1.354709613	0.997	0.571	0	Fibro	4	Serpinf1
Pcolce4	0	1.312878913	0.955	0.542	0	Fibro	4	Pcolce
Cd2481	0	1.307282726	0.914	0.474	0	Fibro	4	Cd248
Dpep11	0	1.25671662	0.907	0.347	0	Fibro	4	Dpep1
Ecm11	0	1.25220591	0.982	0.612	0	Fibro	4	Ecm1
Efemp11	0	1.243818833	0.859	0.356	0	Fibro	4	Efemp1
Qpct1	0	1.237202002	0.897	0.442	0	Fibro	4	Qpct
Ctsl3	0	1.231283251	0.996	0.678	0	Fibro	4	Ctsl
C32	0	1.190210589	0.938	0.513	0	Fibro	4	C3
Cd343	0	1.185743306	0.985	0.485	0	Fibro	4	Cd34
Anxa3	0	1.169495124	0.88	0.46	0	Fibro	4	Anxa3
Islr3	0	1.164595674	0.958	0.568	0	Fibro	4	Islr
Fbn1	0	1.11103509	0.955	0.587	0	Fibro	4	Fbn1

Plpp32	0	1.094578864	0.962	0.602	0	Fibro	4	Plpp3
Tmem1001	0	1.075380538	0.866	0.308	0	Fibro	4	Tmem100
Dpt3	0	1.056243985	1	0.604	0	Fibro	4	Dpt
Fn11	0	1.03332684	0.978	0.632	0	Fibro	4	Fn1
C1ra2	0	1.024514739	0.905	0.442	0	Fibro	4	C1ra
Gstm11	0	1.020160334	0.995	0.72	0	Fibro	4	Gstm1
Nid11	0	1.003962541	0.96	0.502	0	Fibro	4	Nid1
Plat1	0	0.976529313	0.844	0.4	0	Fibro	4	Plat
Tuba1a2	0	0.955842639	0.96	0.617	0	Fibro	4	Tuba1a
Mgst12	0	0.913967944	0.955	0.522	0	Fibro	4	Mgst1
Entpd22	0	0.898696463	0.889	0.423	0	Fibro	4	Entpd2
S100a61	0	0.839184597	1	0.902	0	Fibro	4	S100a6
Rarres21	0	0.812482454	0.996	0.673	0	Fibro	4	Rarres2
Lrrn4cl1	7.93E-306	0.86017941	0.776	0.352	1.59E-302	Fibro	4	Lrrn4cl
C1s12	9.37E-304	0.857225187	0.923	0.462	1.87E-300	Fibro	4	C1s1
Tppp3	4.04E-296	1.016453459	0.943	0.56	8.08E-293	Fibro	4	Tppp3
Gfpt21	4.70E-294	0.9749187	0.761	0.264	9.40E-291	Fibro	4	Gfpt2
Aspn1	4.56E-288	1.269891339	0.844	0.412	9.12E-285	Fibro	4	Aspn
Scara52	2.53E-285	0.836716576	0.891	0.436	5.06E-282	Fibro	4	Scara5
Fndc11	2.75E-283	0.873310589	0.84	0.454	5.49E-280	Fibro	4	Fndc1
Anxa21	1.32E-280	0.883971634	0.955	0.654	2.65E-277	Fibro	4	Anxa2
Pcsk61	1.37E-274	0.895413323	0.806	0.406	2.73E-271	Fibro	4	Pcsk6
Pla1a	5.63E-271	1.405540536	0.687	0.321	1.13E-267	Fibro	4	Pla1a
Serpinh13	7.22E-263	0.755132728	0.981	0.658	1.44E-259	Fibro	4	Serpinh1
Aebp12	2.72E-262	0.834677465	0.974	0.631	5.44E-259	Fibro	4	Aebp1
Lum3	3.20E-258	0.894529026	0.958	0.566	6.40E-255	Fibro	4	Lum
Col14a12	2.22E-247	0.895960882	0.897	0.542	4.43E-244	Fibro	4	Col14a1
Adamts51	8.93E-244	0.835468877	0.828	0.412	1.79E-240	Fibro	4	Adamts5
Ramp21	5.34E-243	0.708549359	0.844	0.375	1.07E-239	Fibro	4	Ramp2
Procr	2.79E-242	1.143204567	0.699	0.353	5.58E-239	Fibro	4	Procr
Gpc32	1.10E-239	0.807002434	0.803	0.421	2.20E-236	Fibro	4	Gpc3
Sparc2	2.60E-239	0.724904835	0.999	0.867	5.19E-236	Fibro	4	Sparc
Metrnl	3.22E-238	0.904063353	0.769	0.389	6.43E-235	Fibro	4	Metrnl
Fstl12	2.06E-234	0.725019357	0.975	0.625	4.13E-231	Fibro	4	Fstl1
Cadm3	6.55E-229	0.658048828	0.615	0.258	1.31E-225	Fibro	4	Cadm3
Fez11	3.72E-228	0.744219887	0.721	0.366	7.45E-225	Fibro	4	Fez1
Igfbp43	1.26E-224	0.704685966	0.989	0.743	2.52E-221	Fibro	4	Igfbp4
Nbl11	1.11E-223	0.70013062	0.931	0.553	2.22E-220	Fibro	4	Nbl1
Ugdh1	6.30E-222	0.830990966	0.782	0.397	1.26E-218	Fibro	4	Ugdh
Sfrp41	3.73E-221	1.486073243	0.686	0.392	7.46E-218	Fibro	4	Sfrp4
Emilin2	5.03E-216	0.718346562	0.707	0.337	1.01E-212	Fibro	4	Emilin2
Fbln23	1.74E-209	0.779805223	0.784	0.362	3.48E-206	Fibro	4	Fbln2
Sema3c	5.43E-201	0.735730498	0.753	0.393	1.09E-197	Fibro	4	Sema3c
Itih5	7.20E-201	0.742766945	0.73	0.415	1.44E-197	Fibro	4	Itih5
Lamb12	1.95E-192	0.627402543	0.661	0.313	3.89E-189	Fibro	4	Lamb1
Ackr3	7.24E-189	0.737544073	0.688	0.334	1.45E-185	Fibro	4	Ackr3
Itm2a2	8.15E-188	0.705411788	0.748	0.459	1.63E-184	Fibro	4	Itm2a
Col3a13	9.00E-188	0.609024167	0.997	0.803	1.80E-184	Fibro	4	Col3a1
Prss23	9.76E-186	0.883988444	0.8	0.477	1.95E-182	Fibro	4	Prss23
Mmp23	3.32E-185	0.577335231	0.905	0.579	6.63E-182	Fibro	4	Mmp2
Ccl111	1.28E-184	0.980278965	0.694	0.386	2.56E-181	Fibro	4	Ccl11
Lbp1	4.87E-184	0.616254394	0.596	0.294	9.73E-181	Fibro	4	Lbp
Col1a23	2.91E-178	0.48822434	0.999	0.786	5.81E-175	Fibro	4	Col1a2
Adgrd11	1.72E-177	0.650032805	0.687	0.334	3.45E-174	Fibro	4	Adgrd1
Fbln13	5.24E-176	0.661369713	0.882	0.5	1.05E-172	Fibro	4	Fbln1
Uap1	4.64E-171	0.719394881	0.714	0.37	9.28E-168	Fibro	4	Uap1
Htra11	3.55E-168	0.624937222	0.844	0.532	7.09E-165	Fibro	4	Htra1
Meg33	9.31E-167	0.628571469	0.953	0.594	1.86E-163	Fibro	4	Meg3
Pamr11	3.75E-166	0.615260261	0.646	0.353	7.49E-163	Fibro	4	Pamr1
Ctsk1	8.52E-165	0.575369132	0.706	0.437	1.70E-161	Fibro	4	Ctsk
Dpp4	2.19E-160	0.584358994	0.609	0.307	4.38E-157	Fibro	4	Dpp4
Efhd1	3.09E-160	0.632108207	0.675	0.357	6.18E-157	Fibro	4	Efhd1
Pdgfra3	2.19E-159	0.509776081	0.83	0.459	4.38E-156	Fibro	4	Pdgfra
Penk1	7.74E-158	0.708036048	0.632	0.297	1.55E-154	Fibro	4	Penk
Ptgs1	6.06E-156	0.513710888	0.582	0.287	1.21E-152	Fibro	4	Ptgs1
Pmp222	1.74E-153	0.539157847	0.885	0.59	3.48E-150	Fibro	4	Pmp22
Col1a13	1.15E-152	0.399909983	0.989	0.725	2.29E-149	Fibro	4	Col1a1
Bdh22	1.62E-152	0.498929125	0.573	0.283	3.25E-149	Fibro	4	Bdh2
Col6a13	7.46E-152	0.502390285	0.935	0.617	1.49E-148	Fibro	4	Col6a1
Igfbp5	3.85E-151	1.011851284	0.782	0.526	7.70E-148	Fibro	4	Igfbp5
Hsp90b1	1.45E-149	0.526565864	0.913	0.661	2.90E-146	Fibro	4	Hsp90b1
Loxl21	9.90E-146	0.520803274	0.622	0.341	1.98E-142	Fibro	4	Loxl2
Thy1	2.06E-143	0.590824224	0.692	0.421	4.12E-140	Fibro	4	Thy1
Fst1	1.54E-142	0.449342286	0.58	0.277	3.08E-139	Fibro	4	Fst
Cd55	7.67E-135	0.647915675	0.664	0.383	1.53E-131	Fibro	4	Cd55
Plac81	1.58E-133	0.650296323	0.721	0.416	3.16E-130	Fibro	4	Plac8
Wnt2	1.66E-130	0.263361714	0.368	0.119	3.33E-127	Fibro	4	Wnt2
Klf4	4.86E-130	0.561477847	0.743	0.425	9.73E-127	Fibro	4	Klf4
Il332	1.41E-128	0.522890331	0.579	0.244	2.83E-125	Fibro	4	Il33
Prdx1	1.89E-128	0.431958434	0.973	0.789	3.77E-125	Fibro	4	Prdx1
Cpxm13	3.20E-128	0.559225786	0.621	0.369	6.40E-125	Fibro	4	Cpxm1
Dact2	1.24E-126	0.622308805	0.508	0.254	2.48E-123	Fibro	4	Dact2
Mfap23	1.56E-126	0.45924358	0.729	0.455	3.13E-123	Fibro	4	Mfap2
Lpl1	1.65E-126	0.546908339	0.752	0.46	3.31E-123	Fibro	4	Lpl
Selenbp11	2.63E-126	0.552188634	0.676	0.401	5.25E-123	Fibro	4	Selenbp1
Ifi27l2a1	1.56E-120	0.464634596	0.964	0.76	3.11E-117	Fibro	4	Ifi27l2a
Ppic1	1.79E-119	0.440925371	0.776	0.518	3.58E-116	Fibro	4	Ppic
Tek	5.43E-119	0.406421262	0.556	0.302	1.09E-115	Fibro	4	Tek
Ifi2041	1.74E-118	0.41278575	0.508	0.206	3.47E-115	Fibro	4	Ifi204
Car8	1.23E-115	0.422556227	0.48	0.235	2.45E-112	Fibro	4	Car8
Col6a23	4.47E-114	0.450816654	0.895	0.599	8.94E-111	Fibro	4	Col6a2
Sult5a11	7.99E-113	0.489564717	0.523	0.275	1.60E-109	Fibro	4	Sult5a1
Ccdc803	1.02E-112	0.405614418	0.846	0.548	2.05E-109	Fibro	4	Ccdc80
Akap122	2.70E-112	0.503039198	0.638	0.332	5.40E-109	Fibro	4	Akap12
Thbd1	1.35E-111	0.611505169	0.635	0.357	2.70E-108	Fibro	4	Thbd
Capg	5.30E-111	0.446889743	0.793	0.535	1.06E-107	Fibro	4	Capg
Abca8a3	7.46E-108	0.361058737	0.742	0.393	1.49E-104	Fibro	4	Abca8a
Aldh1a3	9.47E-103	0.49607966	0.466	0.227	1.89E-99	Fibro	4	Aldh1a3
Spon21	6.11E-101	0.613171637	0.431	0.186	1.22E-97	Fibro	4	Spon2
Uchl1	9.28E-101	0.35356905	0.482	0.254	1.86E-97	Fibro	4	Uchl1
Figf3	4.00E-100	0.48080348	0.549	0.3	8.01E-97	Fibro	4	Figf
Adm2	1.93E-96	0.473614553	0.514	0.294	3.87E-93	Fibro	4	Adm
Fbln51	1.13E-93	0.356592208	0.815	0.543	2.26E-90	Fibro	4	Fbln5
Col5a23	5.95E-93	0.398973392	0.771	0.527	1.19E-89	Fibro	4	Col5a2
Prnp	3.87E-92	0.294508653	0.63	0.372	7.74E-89	Fibro	4	Prnp
Slc25a4	3.35E-90	0.30256198	0.962	0.758	6.71E-87	Fibro	4	Slc25a4
Ecscr	6.20E-90	0.35704525	0.445	0.241	1.24E-86	Fibro	4	Ecscr
Hspa5	6.42E-90	0.4189539	0.836	0.582	1.28E-86	Fibro	4	Hspa5
Stmn4	1.18E-89	0.266677501	0.383	0.184	2.35E-86	Fibro	4	Stmn4
Krtdap	6.03E-88	0.421396924	0.304	0.112	1.21E-84	Fibro	4	Krtdap
Cd92	1.86E-85	0.402193175	0.878	0.639	3.72E-82	Fibro	4	Cd9
Ifi2051	8.11E-79	0.495080319	0.43	0.201	1.62E-75	Fibro	4	Ifi205
Dclk12	3.90E-78	0.294141049	0.434	0.225	7.80E-75	Fibro	4	Dclk1
Cdkn1c2	1.40E-76	0.438446219	0.612	0.393	2.79E-73	Fibro	4	Cdkn1c
Smoc22	7.86E-76	0.484282685	0.592	0.366	1.57E-72	Fibro	4	Smoc2
Abi3bp3	1.44E-73	0.329474548	0.705	0.446	2.89E-70	Fibro	4	Abi3bp
Ckb	3.41E-73	0.371125109	0.766	0.519	6.82E-70	Fibro	4	Ckb
Pdpn2	1.27E-71	0.30574604	0.509	0.28	2.54E-68	Fibro	4	Pdpn
Scn7a2	1.01E-70	0.260440901	0.522	0.267	2.01E-67	Fibro	4	Scn7a
Chst1	1.45E-70	0.339756378	0.387	0.199	2.91E-67	Fibro	4	Chst1
Srpx1	2.67E-70	0.398253438	0.517	0.319	5.35E-67	Fibro	4	Srpx
Gap43	1.54E-68	0.275039904	0.393	0.215	3.07E-65	Fibro	4	Gap43
Angptl12	9.31E-66	0.60912997	0.476	0.307	1.86E-62	Fibro	4	Angptl1
Mnda1	1.17E-63	0.305982641	0.37	0.179	2.35E-60	Fibro	4	Mnda
Ren1	1.47E-63	0.415404226	0.263	0.102	2.94E-60	Fibro	4	Ren1
Inmt2	7.47E-63	0.610859783	0.632	0.456	1.49E-59	Fibro	4	Inmt
Lgals1	1.79E-62	0.276095982	0.996	0.891	3.58E-59	Fibro	4	Lgals1
Tuba1b	3.68E-62	0.297136761	0.854	0.653	7.37E-59	Fibro	4	Tuba1b
Phlda13	1.92E-60	0.364693294	0.6	0.388	3.84E-57	Fibro	4	Phlda1

Smpd3	2.55E-60	0.537753761	0.44	0.278	5.09E-57	Fibro	4	Smpd3
Gas13	2.95E-59	0.31644278	0.662	0.415	5.90E-56	Fibro	4	Gas1
Akr1c18	6.89E-59	0.251346235	0.277	0.128	1.38E-55	Fibro	4	Akr1c18
Gpx31	2.09E-58	0.299546702	0.743	0.538	4.19E-55	Fibro	4	Gpx3
Dpysl3	1.14E-57	0.260421787	0.637	0.432	2.27E-54	Fibro	4	Dpysl3
Flrt2	1.85E-57	0.303395914	0.494	0.33	3.71E-54	Fibro	4	Flrt2
Errfi11	2.22E-56	0.259508639	0.473	0.272	4.44E-53	Fibro	4	Errfi1
Adh12	1.69E-52	0.506301745	0.454	0.275	3.38E-49	Fibro	4	Adh1
Col12a1	5.22E-52	0.408766759	0.447	0.322	1.04E-48	Fibro	4	Col12a1
Emp12	2.54E-51	0.291766074	0.601	0.406	5.09E-48	Fibro	4	Emp1
Sfrp11	3.22E-50	0.276603496	0.465	0.273	6.45E-47	Fibro	4	Sfrp1
Vcan2	1.05E-49	0.258646324	0.48	0.291	2.09E-46	Fibro	4	Vcan
Ackr4	2.18E-49	0.4333852	0.393	0.23	4.36E-46	Fibro	4	Ackr4
Mt21	1.29E-47	0.282766485	0.668	0.461	2.59E-44	Fibro	4	Mt2
Osr12	2.29E-47	0.320628079	0.629	0.439	4.59E-44	Fibro	4	Osr1
Tubb2b	6.19E-47	0.298142527	0.387	0.245	1.24E-43	Fibro	4	Tubb2b
Ppa1	3.35E-42	0.257511636	0.396	0.263	6.70E-39	Fibro	4	Ppa1
Tnfaip6	5.48E-39	0.366867293	0.385	0.247	1.10E-35	Fibro	4	Tnfaip6
Hmcn21	8.77E-39	0.387189929	0.393	0.279	1.75E-35	Fibro	4	Hmcn2
Ntrk22	1.97E-37	0.268306983	0.473	0.351	3.95E-34	Fibro	4	Ntrk2
Dkk21	2.59E-37	0.414368153	0.41	0.31	5.18E-34	Fibro	4	Dkk2
Postn	5.87E-35	0.379710272	0.615	0.467	1.17E-31	Fibro	4	Postn
Fibin1	6.77E-34	0.259219109	0.435	0.317	1.35E-30	Fibro	4	Fibin
Sbsn	7.20E-32	0.638194463	0.397	0.301	1.44E-28	Fibro	4	Sbsn
Enpp21	2.05E-31	0.308516751	0.4	0.298	4.10E-28	Fibro	4	Enpp2
Svep13	2.66E-31	0.265460067	0.443	0.334	5.32E-28	Fibro	4	Svep1
Col8a11	7.14E-19	0.259853395	0.364	0.296	1.43E-15	Fibro	4	Col8a1
Thbs4	8.95E-17	0.65879461	0.347	0.281	1.79E-13	Fibro	4	Thbs4
Cilp2	4.46E-11	0.473626832	0.281	0.262	8.91E-08	Fibro	4	Cilp
Myh11	0	1.981354028	0.995	0.327	0	SMC	1	Myh11
Itga8	0	1.844607021	0.984	0.292	0	SMC	1	Itga8
Mylk	0	1.769203268	0.991	0.31	0	SMC	1	Mylk
Tagln	0	1.75174329	0.999	0.411	0	SMC	1	Tagln
Sost	0	1.708792377	0.85	0.22	0	SMC	1	Sost
Npnt	0	1.688814306	0.95	0.255	0	SMC	1	Npnt
Flna	0	1.676556797	0.998	0.585	0	SMC	1	Flna
Myl9	0	1.637073714	0.998	0.571	0	SMC	1	Myl9
Lpp	0	1.630014323	0.985	0.418	0	SMC	1	Lpp
Fblim1	0	1.623376312	0.976	0.274	0	SMC	1	Fblim1
Dstn	0	1.622770918	1	0.755	0	SMC	1	Dstn
Actn1	0	1.59798688	0.991	0.348	0	SMC	1	Actn1
Eln2	0	1.584298839	0.983	0.558	0	SMC	1	Eln
Csrp1	0	1.583558934	0.996	0.457	0	SMC	1	Csrp1
Tns1	0	1.561665203	0.987	0.544	0	SMC	1	Tns1
Cald1	0	1.55508043	0.996	0.538	0	SMC	1	Cald1
Acta2	0	1.546035124	1	0.725	0	SMC	1	Acta2
Cnn1	0	1.515421805	0.984	0.289	0	SMC	1	Cnn1
Synpo2	0	1.478525564	0.917	0.212	0	SMC	1	Synpo2
Mustn1	0	1.439434617	0.963	0.427	0	SMC	1	Mustn1
Lmod1	0	1.430016671	0.975	0.254	0	SMC	1	Lmod1
Tpm1	0	1.421003719	0.997	0.56	0	SMC	1	Tpm1
Cpe	0	1.365529184	0.994	0.532	0	SMC	1	Cpe
Ppp1r14a	0	1.358434405	0.976	0.325	0	SMC	1	Ppp1r14a
Myl6	0	1.354025415	1	0.972	0	SMC	1	Myl6
Smtn	0	1.351633726	0.974	0.296	0	SMC	1	Smtn
Col18a1	0	1.344821567	0.926	0.285	0	SMC	1	Col18a1
Pcdh7	0	1.318298547	0.896	0.231	0	SMC	1	Pcdh7
Nov	0	1.315991727	0.843	0.27	0	SMC	1	Nov
Csrp2	0	1.292402157	0.985	0.614	0	SMC	1	Csrp2
Dmpk	0	1.281063078	0.954	0.359	0	SMC	1	Dmpk
Mir143hg	0	1.2554492	0.852	0.179	0	SMC	1	Mir143hg
Map1b	0	1.247019691	0.935	0.301	0	SMC	1	Map1b
Enah	0	1.242376437	0.857	0.237	0	SMC	1	Enah
Sh3bgr	0	1.230704925	0.94	0.211	0	SMC	1	Sh3bgr
Ncam1	0	1.223013324	0.821	0.241	0	SMC	1	Ncam1
Tpm2	0	1.219980376	0.994	0.533	0	SMC	1	Tpm2
Pls3	0	1.208354517	0.943	0.393	0	SMC	1	Pls3
Optc	0	1.17413093	0.883	0.199	0	SMC	1	Optc
Rbpms	0	1.17397274	0.965	0.361	0	SMC	1	Rbpms
Fhl1	0	1.150883371	0.984	0.512	0	SMC	1	Fhl1
Mcam	0	1.146460512	0.872	0.21	0	SMC	1	Mcam
Serpine2	0	1.140914922	0.952	0.364	0	SMC	1	Serpine2
Cav1	0	1.133547623	0.955	0.358	0	SMC	1	Cav1
Hspb6	0	1.132832936	0.909	0.247	0	SMC	1	Hspb6
Hspb7	0	1.131137362	0.899	0.196	0	SMC	1	Hspb7
Pbxip1	0	1.130965074	0.969	0.43	0	SMC	1	Pbxip1
Vcl	0	1.120308617	0.913	0.294	0	SMC	1	Vcl
Slmap	0	1.113002162	0.892	0.295	0	SMC	1	Slmap
Pcp4l1	0	1.111901216	0.862	0.191	0	SMC	1	Pcp4l1
Actn4	0	1.110764252	0.938	0.444	0	SMC	1	Actn4
Kcnmb1	0	1.10066288	0.867	0.177	0	SMC	1	Kcnmb1
Cd2001	0	1.098349514	0.966	0.492	0	SMC	1	Cd200
Nexn	0	1.097368561	0.835	0.207	0	SMC	1	Nexn
Adcy5	0	1.092406522	0.833	0.18	0	SMC	1	Adcy5
Dst	0	1.084897223	0.924	0.374	0	SMC	1	Dst
Msrb1	0	1.074083263	0.952	0.45	0	SMC	1	Msrb1
Nudt4	0	1.069781976	0.86	0.311	0	SMC	1	Nudt4
Cped11	0	1.063742542	0.893	0.359	0	SMC	1	Cped1
Ppp1r12b	0	1.062107223	0.884	0.273	0	SMC	1	Ppp1r12b
Susd5	0	1.041882011	0.848	0.241	0	SMC	1	Susd5
1190005I06Rik	0	1.039817225	0.832	0.19	0	SMC	1	1190005I06Rik
Tcap	0	1.037516179	0.894	0.32	0	SMC	1	Tcap
Gucy1a3	0	1.030816777	0.836	0.191	0	SMC	1	Gucy1a3
Hacd1	0	0.993447451	0.893	0.284	0	SMC	1	Hacd1
Itgb1	0	0.986099315	0.951	0.523	0	SMC	1	Itgb1
Rcan2	0	0.974719318	0.888	0.466	0	SMC	1	Rcan2
Postn1	0	0.971295902	0.92	0.424	0	SMC	1	Postn
Pkp4	0	0.961525308	0.823	0.262	0	SMC	1	Pkp4
Zak	0	0.951085782	0.869	0.266	0	SMC	1	Zak
Ramp1	0	0.945097504	0.879	0.379	0	SMC	1	Ramp1
Lmcd1	0	0.940835259	0.896	0.253	0	SMC	1	Lmcd1
Lgr6	0	0.935866099	0.85	0.224	0	SMC	1	Lgr6
Jph2	0	0.929851668	0.829	0.174	0	SMC	1	Jph2
E030013I19Rik	0	0.927480255	0.658	0.165	0	SMC	1	E030013I19Rik
Ppp1r12a	0	0.922938815	0.884	0.325	0	SMC	1	Ppp1r12a
Pde3a	0	0.92197524	0.785	0.228	0	SMC	1	Pde3a
Pdlim7	0	0.919281546	0.929	0.361	0	SMC	1	Pdlim7
Actg2	0	0.909508878	0.855	0.197	0	SMC	1	Actg2
Fbxl22	0	0.907608675	0.735	0.169	0	SMC	1	Fbxl22
Bcam	0	0.906986281	0.916	0.266	0	SMC	1	Bcam
Sod32	0	0.906015383	0.988	0.586	0	SMC	1	Sod3
Plcb4	0	0.901575575	0.764	0.224	0	SMC	1	Plcb4
Mrvi1	0	0.895724021	0.836	0.213	0	SMC	1	Mrvi1
Bgn2	0	0.89090045	1	0.743	0	SMC	1	Bgn
Samd4	0	0.883580938	0.773	0.277	0	SMC	1	Samd4
St5	0	0.88199223	0.784	0.238	0	SMC	1	St5
Itga9	0	0.875003183	0.85	0.292	0	SMC	1	Itga9
Mical2	0	0.874373977	0.76	0.201	0	SMC	1	Mical2
Ccdc3	0	0.870028509	0.735	0.219	0	SMC	1	Ccdc3
Dmd	0	0.860805295	0.723	0.226	0	SMC	1	Dmd
Cdh13	0	0.857062385	0.865	0.237	0	SMC	1	Cdh13
Rbpms2	0	0.854694456	0.823	0.195	0	SMC	1	Rbpms2
Heyl1	0	0.851539169	0.845	0.2	0	SMC	1	Heyl
Col4a2	0	0.833157367	0.894	0.354	0	SMC	1	Col4a2
Rassf3	0	0.832089562	0.717	0.232	0	SMC	1	Rassf3
Tm4sf1	0	0.820081917	0.968	0.469	0	SMC	1	Tm4sf1
Tspan2	0	0.810729392	0.796	0.244	0	SMC	1	Tspan2
Rasl12	0	0.806831948	0.783	0.177	0	SMC	1	Rasl12
Mfge8	0	0.804957772	0.948	0.48	0	SMC	1	Mfge8

Aoc3	0	0.796371746	0.865	0.279	0	SMC	1	Aoc3
Cspg4	0	0.787607084	0.762	0.209	0	SMC	1	Cspg4
Plin4	0	0.78629236	0.68	0.181	0	SMC	1	Plin4
Pi15	0	0.783909271	0.833	0.292	0	SMC	1	Pi15
Thsd4	0	0.778404424	0.771	0.209	0	SMC	1	Thsd4
Pam1	0	0.777507086	0.954	0.613	0	SMC	1	Pam
Tgfb1i1	0	0.771827398	0.903	0.365	0	SMC	1	Tgfb1i1
Pdlim3	0	0.768643567	0.808	0.233	0	SMC	1	Pdlim3
Fbxo30	0	0.767647515	0.803	0.287	0	SMC	1	Fbxo30
Kank1	0	0.767370852	0.761	0.277	0	SMC	1	Kank1
Lrrfip1	0	0.765766484	0.84	0.29	0	SMC	1	Lrrfip1
Ptgis	0	0.763547036	0.961	0.515	0	SMC	1	Ptgis
Vim	0	0.763480439	0.999	0.881	0	SMC	1	Vim
Igfbp72	0	0.759628778	0.998	0.782	0	SMC	1	Igfbp7
Hspb2	0	0.758118523	0.807	0.222	0	SMC	1	Hspb2
Nav2	0	0.748802855	0.756	0.206	0	SMC	1	Nav2
Cyb5r3	0	0.741326115	0.873	0.458	0	SMC	1	Cyb5r3
Mtus2	0	0.739276608	0.626	0.153	0	SMC	1	Mtus2
Serpine1	0	0.735437388	0.648	0.192	0	SMC	1	Serpine1
Filip1l	0	0.731128694	0.791	0.328	0	SMC	1	Filip1l
Scube3	0	0.723686031	0.78	0.167	0	SMC	1	Scube3
Gucy1b3	0	0.719035488	0.748	0.203	0	SMC	1	Gucy1b3
Fgl21	0	0.718663231	0.884	0.353	0	SMC	1	Fgl2
Tgm2	0	0.717550019	0.803	0.26	0	SMC	1	Tgm2
Sorbs2	0	0.71487707	0.618	0.146	0	SMC	1	Sorbs2
Smoc11	0	0.713623465	0.805	0.258	0	SMC	1	Smoc1
Kcnab1	0	0.704078912	0.778	0.167	0	SMC	1	Kcnab1
Cdc42ep3	0	0.702914952	0.803	0.341	0	SMC	1	Cdc42ep3
Cryab	0	0.698427965	0.931	0.49	0	SMC	1	Cryab
Fbxo32	0	0.696083821	0.766	0.255	0	SMC	1	Fbxo32
Dsp	0	0.689941854	0.758	0.171	0	SMC	1	Dsp
Hspb1	0	0.689477259	0.867	0.449	0	SMC	1	Hspb1
Id2	0	0.682454945	0.934	0.472	0	SMC	1	Id2
Rrad	0	0.667483109	0.82	0.256	0	SMC	1	Rrad
Smarcd3	0	0.664006606	0.762	0.236	0	SMC	1	Smarcd3
Sgcg	0	0.652382179	0.774	0.171	0	SMC	1	Sgcg
Itih4	0	0.647526255	0.749	0.188	0	SMC	1	Itih4
2200002D01Rik	0	0.64681657	0.663	0.182	0	SMC	1	2200002D01Rik
Ndrg2	0	0.644019874	0.921	0.349	0	SMC	1	Ndrg2
Nupr12	0	0.642304944	0.943	0.653	0	SMC	1	Nupr1
Tinagl1	0	0.626553412	0.771	0.204	0	SMC	1	Tinagl1
Nrtn	0	0.616850113	0.616	0.135	0	SMC	1	Nrtn
Dpysl31	0	0.614395756	0.882	0.392	0	SMC	1	Dpysl3
Col4a61	0	0.606962871	0.748	0.262	0	SMC	1	Col4a6
Tsc22d1	0	0.597118584	0.931	0.505	0	SMC	1	Tsc22d1
Btg2	0	0.578986465	0.862	0.46	0	SMC	1	Btg2
Efhd11	0	0.56262797	0.761	0.328	0	SMC	1	Efhd1
Wfdc1	0	0.552750087	0.84	0.325	0	SMC	1	Wfdc1
Perp	0	0.513510073	0.616	0.143	0	SMC	1	Perp
Atcayos	0	0.481142789	0.742	0.125	0	SMC	1	Atcayos
Clu	0	0.47338173	0.887	0.327	0	SMC	1	Clu
Dkk31	0	0.471741769	0.81	0.308	0	SMC	1	Dkk3
Igsf9	0	0.465008504	0.714	0.217	0	SMC	1	Igsf9
Tmsb4x	0	0.428701142	1	1	0	SMC	1	Tmsb4x
Ccdc42	0	0.411663877	0.698	0.235	0	SMC	1	Ccdc42
Smim5	0	0.398158669	0.772	0.311	0	SMC	1	Smim5
Ano1	0	0.391502097	0.657	0.202	0	SMC	1	Ano1
Pdlim4	0	0.390232107	0.824	0.391	0	SMC	1	Pdlim4
Parm1	0	0.390221461	0.669	0.163	0	SMC	1	Parm1
Bex4	0	0.389670454	0.593	0.14	0	SMC	1	Bex4
Nr4a2	0	0.388283015	0.69	0.24	0	SMC	1	Nr4a2
Crispld22	0	0.360619238	0.884	0.418	0	SMC	1	Crispld2
Col15a13	0	0.353151503	0.86	0.386	0	SMC	1	Col15a1
Lox2	0	0.352502511	0.746	0.31	0	SMC	1	Lox
Fgf1	0	0.313527641	0.635	0.216	0	SMC	1	Fgf1
Slc22a1	0	0.300698531	0.561	0.143	0	SMC	1	Slc22a1
Ctxn1	0	0.288773949	0.674	0.19	0	SMC	1	Ctxn1
Fgfr2	0	0.265795275	0.636	0.172	0	SMC	1	Fgfr2
Nr4a1	0	0.260326255	0.746	0.344	0	SMC	1	Nr4a1
Osr13	4.54E-300	0.394240479	0.845	0.404	9.07E-297	SMC	1	Osr1
Dusp11	1.70E-297	0.507540999	0.879	0.455	3.41E-294	SMC	1	Dusp1
Fbln52	3.24E-294	0.491771162	0.914	0.516	6.49E-291	SMC	1	Fbln5
Gm13889	4.77E-294	0.850300824	0.649	0.154	9.54E-291	SMC	1	Gm13889
Gja1	3.95E-292	0.355062049	0.645	0.258	7.91E-289	SMC	1	Gja1
Limch1	4.13E-292	0.80810245	0.713	0.303	8.25E-289	SMC	1	Limch1
Hbegf	6.16E-288	0.29662888	0.55	0.147	1.23E-284	SMC	1	Hbegf
Asb2	2.47E-287	0.395248449	0.601	0.171	4.94E-284	SMC	1	Asb2
Slc25a41	2.52E-286	0.463606026	0.984	0.743	5.04E-283	SMC	1	Slc25a4
Fos1	1.63E-284	0.503812009	0.837	0.471	3.26E-281	SMC	1	Fos
Prelp1	1.34E-281	0.496416043	0.902	0.51	2.67E-278	SMC	1	Prelp
Pdgfa	2.63E-277	0.659100243	0.697	0.287	5.26E-274	SMC	1	Pdgfa
Mylk4	1.60E-274	0.696319333	0.614	0.187	3.21E-271	SMC	1	Mylk4
Lgals11	1.54E-255	0.419290684	1	0.884	3.07E-252	SMC	1	Lgals1
Inhba	7.69E-246	0.450906151	0.604	0.201	1.54E-242	SMC	1	Inhba
Sparcl11	1.83E-229	0.551133639	0.776	0.376	3.67E-226	SMC	1	Sparcl1
Fmo22	6.28E-229	0.60051629	0.766	0.369	1.26E-225	SMC	1	Fmo2
Spint2	1.41E-226	0.552949865	0.675	0.252	2.83E-223	SMC	1	Spint2
Prss231	1.15E-225	0.327543652	0.831	0.455	2.30E-222	SMC	1	Prss23
Timp32	2.88E-220	0.276253956	0.969	0.67	5.76E-217	SMC	1	Timp3
Tes	1.06E-216	0.418356555	0.582	0.199	2.12E-213	SMC	1	Tes
Nes	3.71E-205	0.455668257	0.583	0.183	7.42E-202	SMC	1	Nes
Myom1	1.58E-198	0.677624398	0.602	0.183	3.17E-195	SMC	1	Myom1
Htra12	2.14E-194	0.280120142	0.874	0.51	4.27E-191	SMC	1	Htra1
Actb	1.05E-193	0.444675518	1	0.994	2.10E-190	SMC	1	Actb
Ctgf1	1.11E-188	0.55752641	0.597	0.189	2.23E-185	SMC	1	Ctgf
Tnfrsf12a	9.65E-178	0.51497749	0.566	0.176	1.93E-174	SMC	1	Tnfrsf12a
Fstl13	5.81E-172	0.286257019	0.956	0.606	1.16E-168	SMC	1	Fstl1
Ckb1	7.32E-168	0.345861539	0.866	0.494	1.46E-164	SMC	1	Ckb
Nox4	4.41E-167	0.649790733	0.635	0.265	8.83E-164	SMC	1	Nox4
Kcnk3	1.13E-160	0.365482566	0.509	0.143	2.25E-157	SMC	1	Kcnk3
Rasl11a	2.49E-103	0.30325305	0.48	0.149	4.97E-100	SMC	1	Rasl11a
Net1	1.89E-102	0.590541474	0.526	0.208	3.78E-99	SMC	1	Net1
Rgs5	1.79E-91	1.15274183	0.506	0.194	3.58E-88	SMC	1	Rgs5
Map3k7cl	1.71E-88	0.478854151	0.48	0.101	3.43E-85	SMC	1	Map3k7cl
Stbd1	1.02E-78	0.467995035	0.51	0.201	2.03E-75	SMC	1	Stbd1
Npy1r	1.64E-67	0.581218578	0.48	0.141	3.28E-64	SMC	1	Npy1r
Vwc2	1.51E-64	0.452050264	0.466	0.16	3.03E-61	SMC	1	Vwc2
Des	8.11E-60	0.63410229	0.463	0.156	1.62E-56	SMC	1	Des
Slc25a34	1.86E-56	0.327509928	0.419	0.116	3.72E-53	SMC	1	Slc25a34
Cdo11	2.80E-53	0.438941091	0.468	0.193	5.61E-50	SMC	1	Cdo1
Gadd45g	1.48E-52	0.270924349	0.52	0.263	2.96E-49	SMC	1	Gadd45g
Itga5	7.63E-43	0.505495774	0.482	0.216	1.53E-39	SMC	1	Itga5
2210407C18Rik	2.80E-40	0.389141425	0.436	0.144	5.61E-37	SMC	1	2210407C18Rik
Fmo3	7.28E-39	0.351219978	0.404	0.105	1.46E-35	SMC	1	Fmo3
Palld	5.98E-36	0.382095557	0.455	0.202	1.20E-32	SMC	1	Palld
Rgs4	1.25E-31	0.51921782	0.43	0.133	2.51E-28	SMC	1	Rgs4
Sncg	1.16E-23	0.801735094	0.399	0.137	2.33E-20	SMC	1	Sncg
Actc1	8.97E-23	0.306845657	0.401	0.196	1.79E-19	SMC	1	Actc1
Adamts1	1.38E-17	0.403724769	0.464	0.235	2.75E-14	SMC	1	Adamts1
Cd59a	1.29E-16	0.341861243	0.451	0.217	2.58E-13	SMC	1	Cd59a
Gm13861	9.42E-09	0.310472376	0.314	0.101	1.88E-05	SMC	1	Gm13861
Crim1	7.85E-08	0.315179362	0.446	0.269	0.000156997	SMC	1	Crim1
Tnnt2	5.92E-05	0.349496535	0.421	0.234	0.118462897	SMC	1	Tnnt2
Acta21	0	2.708067291	1	0.739	0	SMC	2	Acta2
Tpm21	0	2.393050633	1	0.555	0	SMC	2	Tpm2
Tagln1	0	2.356656219	1	0.439	0	SMC	2	Tagln
Myl91	0	2.237842366	1	0.592	0	SMC	2	Myl9
Cnn11	0	1.891457533	1	0.322	0	SMC	2	Cnn1
Actg21	0	1.80775819	0.925	0.225	0	SMC	2	Actg2

Csrp21	0	1.770347741	1	0.632	0	SMC	2	Csrp2
Myh111	0	1.704569828	1	0.36	0	SMC	2	Myh11
Dstn1	0	1.648117274	1	0.767	0	SMC	2	Dstn
Lmod11	0	1.644088987	0.998	0.288	0	SMC	2	Lmod1
Cpe1	0	1.641191805	1	0.554	0	SMC	2	Cpe
Flna1	0	1.598097273	1	0.605	0	SMC	2	Flna
Lmcd11	0	1.566898819	0.977	0.28	0	SMC	2	Lmcd1
Sost1	0	1.524343852	0.955	0.244	0	SMC	2	Sost
Ppp1r14a1	0	1.523236714	0.998	0.355	0	SMC	2	Ppp1r14a
Actn11	0	1.523197494	0.997	0.379	0	SMC	2	Actn1
Csrp11	0	1.516854072	0.999	0.483	0	SMC	2	Csrp1
Myl61	0	1.513134241	1	0.973	0	SMC	2	Myl6
Fblim11	0	1.498342916	0.998	0.307	0	SMC	2	Fblim1
Cald11	0	1.498311118	1	0.56	0	SMC	2	Cald1
Itga81	0	1.497379219	0.995	0.325	0	SMC	2	Itga8
Clu1	0	1.488529297	0.994	0.348	0	SMC	2	Clu
Sh3bgr1	0	1.487730018	0.99	0.244	0	SMC	2	Sh3bgr
Serpine21	0	1.446830483	0.994	0.39	0	SMC	2	Serpine2
Mylk1	0	1.441350875	0.999	0.342	0	SMC	2	Mylk
Itih41	0	1.431769758	0.829	0.21	0	SMC	2	Itih4
Igfbp73	0	1.415616758	1	0.793	0	SMC	2	Igfbp7
Tm4sf11	0	1.393942055	0.996	0.492	0	SMC	2	Tm4sf1
Smtn1	0	1.387161592	0.997	0.328	0	SMC	2	Smtn
Hspb11	0	1.386709868	0.995	0.462	0	SMC	2	Hspb1
Tcap1	0	1.383258	0.94	0.345	0	SMC	2	Tcap
Tpm11	0	1.37547835	1	0.581	0	SMC	2	Tpm1
Pbxip11	0	1.371246308	0.995	0.455	0	SMC	2	Pbxip1
Fbxl221	0	1.335974213	0.918	0.186	0	SMC	2	Fbxl22
Sod33	0	1.333091357	1	0.605	0	SMC	2	Sod3
Ramp11	0	1.316213278	0.991	0.397	0	SMC	2	Ramp1
Npnt1	0	1.297702005	0.982	0.287	0	SMC	2	Npnt
Hspb61	0	1.293194266	0.964	0.276	0	SMC	2	Hspb6
Bcam1	0	1.290491038	0.965	0.295	0	SMC	2	Bcam
Rbpms1	0	1.285429057	0.992	0.389	0	SMC	2	Rbpms
Ptgis1	0	1.277532017	1	0.535	0	SMC	2	Ptgis
Map1b1	0	1.268934935	0.988	0.328	0	SMC	2	Map1b
Col18a11	0	1.261227659	0.967	0.314	0	SMC	2	Col18a1
Sgcg1	0	1.241321059	0.946	0.19	0	SMC	2	Sgcg
Mfge81	0	1.220285304	0.995	0.5	0	SMC	2	Mfge8
Eln3	0	1.218993276	0.996	0.578	0	SMC	2	Eln
Msrb11	0	1.194264037	0.986	0.472	0	SMC	2	Msrb1
Cd93	0	1.193304959	1	0.63	0	SMC	2	Cd9
Pdlim31	0	1.18406232	0.942	0.253	0	SMC	2	Pdlim3
Synpo21	0	1.167702024	0.962	0.244	0	SMC	2	Synpo2
Pdlim71	0	1.162602241	0.988	0.385	0	SMC	2	Pdlim7
Pls31	0	1.152844173	0.985	0.418	0	SMC	2	Pls3
1-Nov	0	1.149702931	0.913	0.294	0	SMC	2	Nov
Vim1	0	1.139196833	1	0.887	0	SMC	2	Vim
Lpp1	0	1.129380182	0.994	0.445	0	SMC	2	Lpp
Ppp1r12a1	0	1.122219092	0.972	0.347	0	SMC	2	Ppp1r12a
Ncam11	0	1.118663945	0.952	0.261	0	SMC	2	Ncam1
Tns11	0	1.101718541	0.998	0.565	0	SMC	2	Tns1
St51	0	1.086337329	0.92	0.257	0	SMC	2	St5
Cdh131	0	1.079999006	0.914	0.264	0	SMC	2	Cdh13
Fhl11	0	1.076282989	0.997	0.534	0	SMC	2	Fhl1
Postn2	0	1.041736584	0.965	0.446	0	SMC	2	Postn
Dpysl32	0	0.976213285	0.962	0.411	0	SMC	2	Dpysl3
Mfap42	0	0.964165647	0.978	0.513	0	SMC	2	Mfap4
Dmpk1	0	0.961719236	0.963	0.387	0	SMC	2	Dmpk
Mcam1	0	0.961673379	0.883	0.242	0	SMC	2	Mcam
Nexn1	0	0.960494981	0.921	0.233	0	SMC	2	Nexn
Pcdh71	0	0.960100077	0.935	0.261	0	SMC	2	Pcdh7
Tgfb1i11	0	0.959048201	0.976	0.387	0	SMC	2	Tgfb1i1
Optc1	0	0.956737131	0.896	0.231	0	SMC	2	Optc
Pdlim41	0	0.951329054	0.932	0.406	0	SMC	2	Pdlim4
Rrad1	0	0.950368914	0.877	0.28	0	SMC	2	Rrad
Spint21	0	0.93814142	0.923	0.258	0	SMC	2	Spint2
Kcnmb11	0	0.927915373	0.899	0.209	0	SMC	2	Kcnmb1
Hspb71	0	0.927737394	0.903	0.23	0	SMC	2	Hspb7
Hacd11	0	0.922302834	0.934	0.311	0	SMC	2	Hacd1
Wfdc11	0	0.900749739	0.943	0.344	0	SMC	2	Wfdc1
Itgb11	0	0.893406478	0.992	0.542	0	SMC	2	Itgb1
Cyb5r31	0	0.890051982	0.967	0.473	0	SMC	2	Cyb5r3
Ckb2	0	0.884073151	0.933	0.508	0	SMC	2	Ckb
1190005I06Rik1	0	0.881934192	0.877	0.218	0	SMC	2	1190005I06Rik
Lrrfip11	0	0.877279062	0.921	0.312	0	SMC	2	Lrrfip1
Ndrg21	0	0.861811482	0.965	0.375	0	SMC	2	Ndrg2
Gapdh	0	0.856778998	0.998	0.8	0	SMC	2	Gapdh
Slmap1	0	0.850825223	0.916	0.323	0	SMC	2	Slmap
Cav11	0	0.846689447	0.975	0.386	0	SMC	2	Cav1
Smarcd31	0	0.834910543	0.87	0.255	0	SMC	2	Smarcd3
Enah1	0	0.834119332	0.883	0.265	0	SMC	2	Enah
Mir143hg1	0	0.830543812	0.87	0.21	0	SMC	2	Mir143hg
Bgn3	0	0.829023193	1	0.755	0	SMC	2	Bgn
Mustn11	0	0.825768554	0.949	0.454	0	SMC	2	Mustn1
Ldhb	0	0.821502731	0.884	0.292	0	SMC	2	Ldhb
Rcan21	0	0.817949524	0.929	0.484	0	SMC	2	Rcan2
Pcp4l11	0	0.812698823	0.869	0.223	0	SMC	2	Pcp4l1
Nupr13	0	0.805200243	0.995	0.664	0	SMC	2	Nupr1
Cped12	0	0.804195589	0.942	0.382	0	SMC	2	Cped1
Lgals12	0	0.792867465	1	0.89	0	SMC	2	Lgals1
Adcy51	0	0.783160195	0.844	0.211	0	SMC	2	Adcy5
Vcl1	0	0.782003509	0.926	0.323	0	SMC	2	Vcl
Tinagl11	0	0.781289032	0.835	0.228	0	SMC	2	Tinagl1
Cd2002	0	0.76866175	0.973	0.514	0	SMC	2	Cd200
Itga91	0	0.759648701	0.888	0.317	0	SMC	2	Itga9
Plcb41	0	0.755041675	0.849	0.245	0	SMC	2	Plcb4
Susd51	0	0.754076926	0.837	0.272	0	SMC	2	Susd5
Fbln53	0	0.731993072	0.966	0.532	0	SMC	2	Fbln5
E030013I19Rik1	0	0.731800693	0.727	0.185	0	SMC	2	E030013I19Rik
Hspb21	0	0.731478287	0.837	0.249	0	SMC	2	Hspb2
Lgr61	0	0.729215341	0.832	0.255	0	SMC	2	Lgr6
Pkp41	0	0.725464248	0.819	0.289	0	SMC	2	Pkp4
Nudt41	0	0.722399317	0.9	0.335	0	SMC	2	Nudt4
Rasl121	0	0.703483952	0.791	0.206	0	SMC	2	Rasl12
Mylk41	0	0.701014396	0.712	0.202	0	SMC	2	Mylk4
Dst1	0	0.694566064	0.922	0.4	0	SMC	2	Dst
Slc25a42	0	0.69380332	1	0.754	0	SMC	2	Slc25a4
Ppp1r12b1	0	0.690730297	0.886	0.303	0	SMC	2	Ppp1r12b
Aoc31	0	0.687176573	0.861	0.308	0	SMC	2	Aoc3
Jph21	0	0.677955259	0.807	0.207	0	SMC	2	Jph2
Rbpms21	0	0.670943998	0.799	0.227	0	SMC	2	Rbpms2
Cdc42ep31	0	0.670224295	0.908	0.357	0	SMC	2	Cdc42ep3
Dmd1	0	0.66826029	0.784	0.246	0	SMC	2	Dmd
Tspan21	0	0.667569676	0.801	0.27	0	SMC	2	Tspan2
Gucy1b31	0	0.664393945	0.776	0.228	0	SMC	2	Gucy1b3
Kank11	0	0.661946377	0.813	0.297	0	SMC	2	Kank1
Ccdc31	0	0.655997156	0.745	0.243	0	SMC	2	Ccdc3
Fgl22	0	0.654423821	0.912	0.377	0	SMC	2	Fgl2
Plin41	0	0.651691491	0.73	0.202	0	SMC	2	Plin4
Scube31	0	0.630765503	0.741	0.199	0	SMC	2	Scube3
Zak1	0	0.630491098	0.84	0.297	0	SMC	2	Zak
Dsp1	0	0.625676257	0.712	0.202	0	SMC	2	Dsp
Cbr2	0	0.623083359	0.97	0.37	0	SMC	2	Cbr2
Mrvi11	0	0.619223758	0.772	0.246	0	SMC	2	Mrvi1
Asb21	0	0.605451133	0.694	0.186	0	SMC	2	Asb2
Kcnab11	0	0.597709284	0.748	0.198	0	SMC	2	Kcnab1
Thsd41	0	0.589574398	0.765	0.237	0	SMC	2	Thsd4
Heyl2	0	0.539702202	0.799	0.234	0	SMC	2	Heyl

Casq1	0	0.415854563	0.603	0.23	0	SMC	2	Casq1
Atcayos1	0	0.389469607	0.643	0.161	0	SMC	2	Atcayos
Filip1l1	5.00E-308	0.740523708	0.876	0.346	9.99E-305	SMC	2	Filip1l
Limch11	6.33E-308	0.684402116	0.832	0.316	1.27E-304	SMC	2	Limch1
Rbp13	7.10E-308	0.683234277	0.991	0.617	1.42E-304	SMC	2	Rbp1
Serpini1	8.34E-308	0.49785133	0.629	0.188	1.67E-304	SMC	2	Serpini1
Nav21	2.79E-306	0.516456715	0.728	0.234	5.58E-303	SMC	2	Nav2
Smoc12	4.90E-306	0.499681081	0.76	0.287	9.81E-303	SMC	2	Smoc1
Myom11	1.13E-301	0.61777791	0.713	0.197	2.25E-298	SMC	2	Myom1
Fbxo321	1.21E-298	0.615217981	0.765	0.28	2.43E-295	SMC	2	Fbxo32
Efhd12	2.13E-298	0.626593255	0.816	0.346	4.27E-295	SMC	2	Efhd1
Ccdc421	4.61E-296	0.519411183	0.66	0.26	9.22E-293	SMC	2	Ccdc42
Fmo23	1.38E-292	0.743369543	0.904	0.38	2.76E-289	SMC	2	Fmo2
2210407C18Rik:	4.34E-291	0.741497808	0.586	0.15	8.69E-288	SMC	2	2210407C18Rik
Gucy1a31	2.55E-290	0.63581407	0.786	0.225	5.09E-287	SMC	2	Gucy1a3
Cryab1	4.90E-289	0.598506729	0.969	0.509	9.79E-286	SMC	2	Cryab
Mical21	2.89E-286	0.591553256	0.762	0.228	5.79E-283	SMC	2	Mical2
Fbxo301	4.06E-282	0.583034012	0.753	0.315	8.11E-279	SMC	2	Fbxo30
Htra13	6.85E-281	0.67527386	0.937	0.524	1.37E-277	SMC	2	Htra1
Gstm12	1.38E-279	0.625662193	0.999	0.717	2.76E-276	SMC	2	Gstm1
Aldoa	2.90E-278	0.528825048	0.992	0.755	5.80E-275	SMC	2	Aldoa
Uchl11	6.36E-277	0.641444023	0.688	0.24	1.27E-273	SMC	2	Uchl1
Rassf31	5.10E-276	0.624112619	0.775	0.252	1.02E-272	SMC	2	Rassf3
Sfrp21	2.57E-273	0.414843016	0.827	0.425	5.15E-270	SMC	2	Sfrp2
Cd59a1	3.57E-272	0.474472356	0.671	0.215	7.15E-269	SMC	2	Cd59a
Mgll	1.27E-271	0.449088526	0.844	0.378	2.53E-268	SMC	2	Mgll
Mgp2	1.28E-270	0.487567197	1	0.955	2.57E-267	SMC	2	Mgp
Cspg41	1.00E-265	0.565986741	0.7	0.24	2.01E-262	SMC	2	Cspg4
Tsc22d11	1.50E-263	0.531183263	0.943	0.525	2.99E-260	SMC	2	Tsc22d1
Pygm	3.34E-256	0.315770431	0.624	0.276	6.69E-253	SMC	2	Pygm
Smim51	1.23E-250	0.424004163	0.701	0.338	2.45E-247	SMC	2	Smim5
Pde3a1	1.40E-248	0.599898567	0.755	0.256	2.81E-245	SMC	2	Pde3a
Pi151	2.47E-244	0.466437742	0.771	0.322	4.94E-241	SMC	2	Pi15
Mdh1	9.08E-244	0.558245381	0.921	0.503	1.82E-240	SMC	2	Mdh1
Perp1	1.67E-241	0.500089047	0.623	0.166	3.34E-238	SMC	2	Perp
Id21	6.68E-239	0.583919987	0.934	0.494	1.34E-235	SMC	2	Id2
Actn41	2.52E-236	0.579128659	0.895	0.47	5.03E-233	SMC	2	Actn4
Tgm21	4.68E-234	0.504103511	0.797	0.287	9.36E-231	SMC	2	Tgm2
Gm138891	5.24E-233	0.651764427	0.673	0.177	1.05E-229	SMC	2	Gm13889
Col4a21	1.28E-232	0.465632058	0.877	0.381	2.56E-229	SMC	2	Col4a2
Fabp3	3.07E-232	0.324683672	0.598	0.167	6.14E-229	SMC	2	Fabp3
Dkk32	1.37E-226	0.356865173	0.829	0.332	2.74E-223	SMC	2	Dkk3
Prdx11	1.84E-226	0.470444964	0.995	0.787	3.69E-223	SMC	2	Prdx1
Ecm12	6.16E-226	0.519416671	0.967	0.61	1.23E-222	SMC	2	Ecm1
2200002D01Rik:	2.41E-224	0.504209598	0.613	0.208	4.83E-221	SMC	2	2200002D01Rik
Nrtn1	6.99E-222	0.572504719	0.604	0.159	1.40E-218	SMC	2	Nrtn
Col15a14	2.38E-221	0.342578338	0.868	0.409	4.76E-218	SMC	2	Col15a1
Plpp12	9.62E-220	0.351406347	0.807	0.374	1.92E-216	SMC	2	Plpp1
Nes1	4.72E-219	0.508748704	0.629	0.2	9.45E-216	SMC	2	Nes
Osr14	6.51E-218	0.447359925	0.873	0.423	1.30E-214	SMC	2	Osr1
Samd41	1.03E-214	0.5563288	0.765	0.302	2.05E-211	SMC	2	Samd4
Cst31	3.65E-214	0.29933387	1	0.955	7.29E-211	SMC	2	Cst3
Rarres22	5.85E-211	0.327418589	0.996	0.671	1.17E-207	SMC	2	Rarres2
Mtus21	2.13E-206	0.564442241	0.626	0.176	4.27E-203	SMC	2	Mtus2
Tubb4b	9.76E-204	0.389822483	0.814	0.386	1.95E-200	SMC	2	Tubb4b
Rasl11a1	1.16E-203	0.551892145	0.596	0.158	2.32E-200	SMC	2	Rasl11a
Tuba1c	5.51E-202	0.30070923	0.663	0.312	1.10E-198	SMC	2	Tuba1c
Cdo12	1.80E-200	0.504217299	0.623	0.197	3.61E-197	SMC	2	Cdo1
Pdgfa1	4.45E-197	0.412801793	0.738	0.305	8.91E-194	SMC	2	Pdgfa
Slc22a11	5.29E-197	0.494307283	0.532	0.165	1.06E-193	SMC	2	Slc22a1
Pam2	1.02E-196	0.456071534	0.99	0.628	2.03E-193	SMC	2	Pam
Tes1	1.15E-194	0.404494869	0.602	0.217	2.31E-191	SMC	2	Tes
Aebp13	5.20E-183	0.51191487	0.982	0.628	1.04E-179	SMC	2	Aebp1
Col4a62	1.18E-182	0.429590219	0.682	0.29	2.35E-179	SMC	2	Col4a6
Ano11	1.82E-182	0.275587499	0.577	0.228	3.65E-179	SMC	2	Ano1
Sparc3	7.65E-182	0.320707478	1	0.866	1.53E-178	SMC	2	Sparc
Nox41	1.82E-176	0.442464077	0.706	0.279	3.65E-173	SMC	2	Nox4
Actc11	1.87E-176	1.06509516	0.537	0.198	3.73E-173	SMC	2	Actc1
Prelp2	1.10E-175	0.387628346	0.924	0.528	2.21E-172	SMC	2	Prelp
Paqr61	6.77E-175	0.388842438	0.607	0.266	1.35E-171	SMC	2	Paqr6
Bex41	5.84E-171	0.417187552	0.516	0.166	1.17E-167	SMC	2	Bex4
Slc25a341	2.01E-167	0.36062783	0.476	0.127	4.01E-164	SMC	2	Slc25a34
Tmsb4x1	2.43E-162	0.29646258	1	1	4.86E-159	SMC	2	Tmsb4x
Stbd11	1.51E-160	0.440764324	0.608	0.21	3.03E-157	SMC	2	Stbd1
S100a41	3.16E-159	0.3457398	0.905	0.556	6.31E-156	SMC	2	S100a4
Tnfrsf12a1	3.12E-158	0.499497628	0.583	0.194	6.23E-155	SMC	2	Tnfrsf12a
Emb	1.79E-157	0.347219879	0.584	0.245	3.58E-154	SMC	2	Emb
Actb1	1.26E-143	0.346072888	1	0.994	2.51E-140	SMC	2	Actb
Btg21	5.46E-143	0.353354935	0.837	0.481	1.09E-139	SMC	2	Btg2
Sparcl12	1.17E-135	0.578813572	0.745	0.397	2.34E-132	SMC	2	Sparcl1
Inhba1	4.70E-135	0.444698917	0.571	0.223	9.41E-132	SMC	2	Inhba
Npy1r1	1.12E-133	0.419377095	0.527	0.155	2.25E-130	SMC	2	Npy1r
Bmp3	1.90E-133	0.473787241	0.481	0.15	3.80E-130	SMC	2	Bmp3
Ctgf2	2.94E-132	0.286577062	0.587	0.21	5.88E-129	SMC	2	Ctgf
Gadd45g1	4.04E-131	0.265884531	0.617	0.27	8.07E-128	SMC	2	Gadd45g
Dusp12	4.10E-130	0.324623221	0.856	0.477	8.20E-127	SMC	2	Dusp1
Prss232	4.97E-128	0.311635594	0.82	0.474	9.95E-125	SMC	2	Prss23
Hmgcs21	1.23E-119	0.346206596	0.554	0.216	2.47E-116	SMC	2	Hmgcs2
Itga51	3.38E-113	0.386338126	0.555	0.224	6.75E-110	SMC	2	Itga5
Map3k7cl1	7.48E-110	0.339874212	0.479	0.119	1.50E-106	SMC	2	Map3k7cl
Sorbs21	1.55E-108	0.31912717	0.505	0.175	3.11E-105	SMC	2	Sorbs2
Vwc21	5.25E-107	0.391132009	0.504	0.173	1.05E-103	SMC	2	Vwc2
Hbegf1	8.66E-102	0.259380582	0.441	0.173	1.73E-98	SMC	2	Hbegf
Igsf91	8.37E-101	0.348789809	0.525	0.252	1.67E-97	SMC	2	Igsf9
Tuba1b1	2.22E-96	0.274752846	0.939	0.647	4.45E-93	SMC	2	Tuba1b
Hspa51	1.37E-92	0.308082471	0.912	0.575	2.74E-89	SMC	2	Hspa5
Rgs41	7.65E-92	0.405113285	0.484	0.144	1.53E-88	SMC	2	Rgs4
Rbp41	5.46E-87	0.385666276	0.579	0.27	1.09E-83	SMC	2	Rbp4
Net11	2.86E-86	0.365770457	0.523	0.224	5.72E-83	SMC	2	Net1
Sncg1	6.75E-80	0.436753625	0.41	0.149	1.35E-76	SMC	2	Sncg
Rgs51	7.26E-73	0.680874512	0.498	0.21	1.45E-69	SMC	2	Rgs5
Ptprz1	1.82E-58	0.418743279	0.436	0.157	3.64E-55	SMC	2	Ptprz1
Des1	3.06E-57	0.511761832	0.416	0.173	6.12E-54	SMC	2	Des
Fmo31	6.35E-54	0.361985481	0.382	0.121	1.27E-50	SMC	2	Fmo3
Palld1	3.16E-52	0.280668117	0.452	0.215	6.32E-49	SMC	2	Palld
Serpine11	1.35E-48	0.448381166	0.45	0.226	2.69E-45	SMC	2	Serpine1
Igfbp2	2.66E-39	0.587242848	0.311	0.131	5.32E-36	SMC	2	Igfbp2
Tnnt21	4.84E-23	0.358732831	0.412	0.244	9.68E-20	SMC	2	Tnnt2
Colec11	0	1.799682111	0.809	0.153	0	SMC	3	Colec11
Notch3	0	1.605455947	0.872	0.181	0	SMC	3	Notch3
Mustn12	4.37E-248	1.744234497	0.956	0.469	8.74E-245	SMC	3	Mustn1
Ppp1r14a2	1.46E-240	1.406254125	0.955	0.376	2.92E-237	SMC	3	Ppp1r14a
Sdc1	2.50E-239	1.227707099	0.82	0.297	5.00E-236	SMC	3	Sdc1
Higd1b	1.22E-231	1.179944647	0.614	0.061	2.45E-228	SMC	3	Higd1b
Kcnk31	4.74E-219	1.162489234	0.731	0.166	9.47E-216	SMC	3	Kcnk3
Tinagl12	1.05E-218	1.320960408	0.825	0.247	2.09E-215	SMC	3	Tinagl1
Parm11	3.00E-216	0.816221024	0.745	0.201	6.00E-213	SMC	3	Parm1
Gucy1a32	1.12E-213	1.229028433	0.848	0.241	2.25E-210	SMC	3	Gucy1a3
Vtn	2.85E-211	1.487178644	0.656	0.158	5.71E-208	SMC	3	Vtn
Ndrg22	1.76E-203	1.106821712	0.921	0.394	3.52E-200	SMC	3	Ndrg2
Myl92	4.07E-202	1.156797288	0.993	0.605	8.14E-199	SMC	3	Myl9
Slc11a1	8.91E-198	1.074744684	0.755	0.254	1.78E-194	SMC	3	Slc11a1
Gpx32	5.95E-196	1.292231427	0.969	0.537	1.19E-192	SMC	3	Gpx3
Tagln2	8.22E-193	1.304939915	0.942	0.458	1.64E-189	SMC	3	Tagln
Ephx3	3.03E-189	0.445937684	0.446	0.048	6.06E-186	SMC	3	Ephx3
Igfbp74	2.53E-175	0.952070308	1	0.799	5.06E-172	SMC	3	Igfbp7
Nr2f21	2.66E-174	1.101323804	0.837	0.344	5.32E-171	SMC	3	Nr2f2
Mylk2	5.97E-173	1.112699537	0.913	0.365	1.19E-169	SMC	3	Mylk
Acta22	1.39E-172	0.910391456	0.981	0.747	2.78E-169	SMC	3	Acta2

Tpm22	1.90E-172	1.124543905	0.944	0.571	3.80E-169	SMC	3	Tpm2
Tpm12	3.66E-171	0.922080972	0.965	0.595	7.33E-168	SMC	3	Tpm1
Cald12	9.79E-166	0.910512165	0.965	0.575	1.96E-162	SMC	3	Cald1
Cdc42ep32	1.93E-152	1.075966216	0.844	0.376	3.86E-149	SMC	3	Cdc42ep3
Actg22	9.38E-150	1.360180787	0.774	0.251	1.88E-146	SMC	3	Actg2
Gm138892	1.31E-148	1.379460051	0.729	0.191	2.62E-145	SMC	3	Gm13889
Ndufa4l21	3.71E-148	1.367494325	0.701	0.208	7.41E-145	SMC	3	Ndufa4l2
Csrp12	1.29E-146	0.920883737	0.928	0.5	2.58E-143	SMC	3	Csrp1
Rgs52	2.94E-146	1.927075035	0.722	0.214	5.87E-143	SMC	3	Rgs5
Hes11	3.13E-144	0.91436559	0.778	0.299	6.26E-141	SMC	3	Hes1
Postn3	2.22E-140	1.130512418	0.874	0.464	4.43E-137	SMC	3	Postn
Tnc	1.10E-139	0.381576592	0.586	0.158	2.19E-136	SMC	3	Tnc
Fhl2	5.18E-139	0.571159781	0.622	0.182	1.04E-135	SMC	3	Fhl2
Cpxm22	2.58E-135	0.903978174	0.684	0.263	5.16E-132	SMC	3	Cpxm2
Prss233	6.43E-135	0.979286382	0.874	0.483	1.29E-131	SMC	3	Prss23
Fhl12	2.71E-134	0.915433833	0.928	0.55	5.42E-131	SMC	3	Fhl1
Filip1l2	8.30E-134	0.991464916	0.806	0.364	1.66E-130	SMC	3	Filip1l
Hmcn22	5.42E-133	0.455463915	0.666	0.275	1.08E-129	SMC	3	Hmcn2
Myh112	1.35E-131	0.747472779	0.888	0.382	2.69E-128	SMC	3	Myh11
Aspn2	2.00E-130	0.849526294	0.85	0.422	4.00E-127	SMC	3	Aspn
Heyl3	3.97E-121	0.751469367	0.724	0.253	7.94E-118	SMC	3	Heyl
Ednra1	1.02E-119	0.823960464	0.657	0.229	2.03E-116	SMC	3	Ednra
Ctxn11	1.52E-114	0.501126574	0.605	0.229	3.04E-111	SMC	3	Ctxn1
Epas1	2.28E-114	0.849594292	0.691	0.234	4.55E-111	SMC	3	Epas1
Rbpms3	4.52E-113	0.651170513	0.86	0.411	9.03E-110	SMC	3	Rbpms
Chrdl1	2.26E-112	0.849515354	0.619	0.195	4.52E-109	SMC	3	Chrdl1
Crip1	5.21E-112	0.568345553	0.997	0.967	1.04E-108	SMC	3	Crip1
Aoc32	4.22E-110	0.847426112	0.738	0.328	8.45E-107	SMC	3	Aoc3
Mfge82	1.26E-107	0.916771765	0.89	0.518	2.51E-104	SMC	3	Mfge8
Lmod12	2.48E-107	0.766709591	0.801	0.315	4.97E-104	SMC	3	Lmod1
Errfi12	1.28E-103	0.975151136	0.696	0.271	2.56E-100	SMC	3	Errfi1
Esam	1.21E-102	0.358587647	0.572	0.193	2.42E-99	SMC	3	Esam
Gstm13	2.81E-102	0.84651759	0.979	0.727	5.62E-99	SMC	3	Gstm1
Myl62	8.59E-101	0.534458144	0.997	0.974	1.72E-97	SMC	3	Myl6
Kcnj81	5.16E-100	1.133101506	0.656	0.289	1.03E-96	SMC	3	Kcnj8
Nts	2.16E-98	0.642657853	0.495	0.108	4.31E-95	SMC	3	Nts
Map1b2	6.96E-97	0.854787113	0.776	0.354	1.39E-93	SMC	3	Map1b
Tgfb1i12	1.73E-96	0.680220861	0.804	0.409	3.46E-93	SMC	3	Tgfb1i1
Gng111	2.52E-95	0.80784749	0.804	0.44	5.05E-92	SMC	3	Gng11
Cav12	3.08E-95	0.551613802	0.848	0.407	6.15E-92	SMC	3	Cav1
Mir143hg2	1.79E-94	0.782388977	0.68	0.236	3.57E-91	SMC	3	Mir143hg
Pdlim32	2.35E-92	0.607454617	0.71	0.28	4.70E-89	SMC	3	Pdlim3
C1s13	5.34E-92	0.607414593	0.872	0.475	1.07E-88	SMC	3	C1s1
Cnn12	1.18E-91	0.676845135	0.783	0.348	2.36E-88	SMC	3	Cnn1
Igfbp51	1.67E-91	0.36529712	0.895	0.529	3.33E-88	SMC	3	Igfbp5
Palld2	3.95E-90	0.756939083	0.612	0.218	7.91E-87	SMC	3	Palld
Apoe	6.35E-90	0.696737035	0.951	0.841	1.27E-86	SMC	3	Apoe
Cryab2	2.61E-88	0.580608569	0.858	0.526	5.22E-85	SMC	3	Cryab
Cox4i2	6.89E-88	0.842376983	0.582	0.202	1.38E-84	SMC	3	Cox4i2
Flna2	3.20E-86	0.461020838	0.92	0.619	6.41E-83	SMC	3	Flna
Bcam2	1.12E-85	0.464443253	0.74	0.321	2.24E-82	SMC	3	Bcam
Tns12	1.32E-85	0.511530382	0.92	0.58	2.64E-82	SMC	3	Tns1
Fbln71	2.42E-85	0.460471048	0.601	0.252	4.83E-82	SMC	3	Fbln7
Dstn2	2.83E-85	0.408801311	0.955	0.775	5.66E-82	SMC	3	Dstn
Figf4	4.75E-85	0.490732337	0.685	0.302	9.50E-82	SMC	3	Figf
Ppp1r1a	7.03E-85	0.297945339	0.509	0.16	1.41E-81	SMC	3	Ppp1r1a
Igfbp32	8.78E-84	1.107265884	0.596	0.244	1.76E-80	SMC	3	Igfbp3
Rasl122	1.94E-82	0.611005857	0.631	0.228	3.89E-79	SMC	3	Rasl12
Pde3a2	6.06E-82	0.60034558	0.68	0.274	1.21E-78	SMC	3	Pde3a
Tmem1192	1.87E-81	0.439449722	0.687	0.342	3.73E-78	SMC	3	Tmem119
Gadd45g2	9.00E-80	0.90150275	0.659	0.28	1.80E-76	SMC	3	Gadd45g
Actn42	1.46E-79	0.630671854	0.813	0.485	2.92E-76	SMC	3	Actn4
Ccl192	2.54E-79	0.716437382	0.668	0.263	5.07E-76	SMC	3	Ccl19
Ano12	2.95E-77	0.415759125	0.582	0.239	5.91E-74	SMC	3	Ano1
Gucy1b32	8.28E-76	0.745427117	0.617	0.249	1.66E-72	SMC	3	Gucy1b3
Stbd12	1.64E-72	0.755981542	0.608	0.222	3.27E-69	SMC	3	Stbd1
Tmem176b	3.63E-72	0.571615229	0.804	0.43	7.26E-69	SMC	3	Tmem176b
Cpe2	5.12E-72	0.34658563	0.927	0.57	1.02E-68	SMC	3	Cpe
Abcc93	3.63E-71	0.741653795	0.668	0.303	7.26E-68	SMC	3	Abcc9
Il34	5.09E-71	0.486942158	0.465	0.126	1.02E-67	SMC	3	Il34
Rarres23	1.62E-69	0.340153862	0.97	0.682	3.23E-66	SMC	3	Rarres2
Pbxip12	3.36E-69	0.451775241	0.837	0.476	6.73E-66	SMC	3	Pbxip1
Lpp2	2.56E-67	0.439578016	0.818	0.466	5.13E-64	SMC	3	Lpp
Nexn2	6.13E-67	0.626303605	0.663	0.26	1.23E-63	SMC	3	Nexn
Thbs11	2.86E-66	0.391391266	0.53	0.198	5.72E-63	SMC	3	Thbs1
Ifitm11	2.09E-64	1.242251312	0.474	0.139	4.19E-61	SMC	3	Ifitm1
Nes2	2.60E-64	0.330696081	0.542	0.215	5.21E-61	SMC	3	Nes
Lmcd12	1.07E-62	0.393360526	0.692	0.308	2.14E-59	SMC	3	Lmcd1
Ckb3	4.04E-61	0.579744301	0.816	0.524	8.07E-58	SMC	3	Ckb
Stc11	8.85E-61	0.257635769	0.516	0.215	1.77E-57	SMC	3	Stc1
Spint22	1.62E-60	0.429840428	0.649	0.285	3.24E-57	SMC	3	Spint2
Gem1	2.09E-60	0.386390633	0.678	0.375	4.17E-57	SMC	3	Gem
Vcl2	8.36E-60	0.435298812	0.698	0.347	1.67E-56	SMC	3	Vcl
Cyr61	1.08E-59	0.874812829	0.61	0.281	2.15E-56	SMC	3	Cyr61
Junb2	1.14E-58	0.73558945	0.871	0.62	2.28E-55	SMC	3	Junb
Cxcl122	5.24E-58	0.907396772	0.717	0.386	1.05E-54	SMC	3	Cxcl12
Egr12	7.13E-56	0.408878103	0.753	0.413	1.43E-52	SMC	3	Egr1
Malat11	9.62E-55	0.367464434	1	0.999	1.92E-51	SMC	3	Malat1
Synpo22	1.29E-54	0.422127806	0.673	0.274	2.59E-51	SMC	3	Synpo2
Fos2	8.16E-54	0.771055025	0.79	0.501	1.63E-50	SMC	3	Fos
Sod34	1.09E-53	0.375664724	0.902	0.62	2.19E-50	SMC	3	Sod3
Hspb22	6.31E-53	0.447429176	0.601	0.273	1.26E-49	SMC	3	Hspb2
Rasl11a2	7.68E-53	0.726700151	0.514	0.174	1.54E-49	SMC	3	Rasl11a
Slc25a43	1.40E-52	0.361903171	0.946	0.763	2.81E-49	SMC	3	Slc25a4
Pdlim72	1.91E-52	0.254588846	0.759	0.41	3.81E-49	SMC	3	Pdlim7
Mrvi12	2.28E-52	0.366472941	0.586	0.267	4.56E-49	SMC	3	Mrvi1
Sep-41	2.97E-52	0.877776557	0.528	0.231	5.94E-49	SMC	3	4-Sep
Des2	2.97E-52	0.614907233	0.523	0.178	5.95E-49	SMC	3	Des
Pi152	5.37E-52	0.494117411	0.654	0.339	1.07E-48	SMC	3	Pi15
Ppp1r12a2	7.32E-52	0.42589928	0.703	0.373	1.46E-48	SMC	3	Ppp1r12a
Rgs17	8.36E-52	0.449502789	0.439	0.074	1.67E-48	SMC	3	Rgs17
Msrb12	5.59E-51	0.412278272	0.795	0.493	1.12E-47	SMC	3	Msrb1
Meg34	1.60E-50	0.376261849	0.916	0.604	3.21E-47	SMC	3	Meg3
Tmem215	1.09E-49	0.304342911	0.4	0.053	2.17E-46	SMC	3	Tmem215
Dmpk2	3.19E-49	0.277542009	0.705	0.412	6.38E-46	SMC	3	Dmpk
Pdgfa2	3.51E-48	0.58378069	0.614	0.321	7.03E-45	SMC	3	Pdgfa
Aldh1a12	1.08E-47	0.330796171	0.601	0.299	2.17E-44	SMC	3	Aldh1a1
Ramp12	1.18E-46	0.395306039	0.724	0.422	2.35E-43	SMC	3	Ramp1
Sox4	1.63E-46	0.28424681	0.586	0.308	3.26E-43	SMC	3	Sox4
Lgals13	8.86E-46	0.367389812	0.998	0.893	1.77E-42	SMC	3	Lgals1
Matn21	9.90E-46	0.343633334	0.551	0.268	1.98E-42	SMC	3	Matn2
Tppp31	3.53E-45	0.473775228	0.823	0.572	7.07E-42	SMC	3	Tppp3
Btg22	5.55E-45	0.493282925	0.778	0.494	1.11E-41	SMC	3	Btg2
Cbfa2t3	2.82E-42	0.437388221	0.524	0.245	5.64E-39	SMC	3	Cbfa2t3
Id32	6.11E-42	0.459968558	0.893	0.686	1.22E-38	SMC	3	Id3
Mdk1	1.34E-41	0.427483634	0.593	0.345	2.67E-38	SMC	3	Mdk
Zfp36	1.40E-41	0.291449763	0.724	0.463	2.80E-38	SMC	3	Zfp36
Col6a14	3.44E-41	0.327753414	0.893	0.626	6.89E-38	SMC	3	Col6a1
Col6a24	3.69E-41	0.353854049	0.876	0.607	7.39E-38	SMC	3	Col6a2
Igfbp44	3.12E-40	0.333130375	0.958	0.75	6.25E-37	SMC	3	Igfbp4
Gja4	8.29E-40	0.595188694	0.432	0.15	1.66E-36	SMC	3	Gja4
Cyb5r32	3.22E-38	0.298326488	0.787	0.492	6.44E-35	SMC	3	Cyb5r3
Fabp4	4.94E-38	0.362908724	0.654	0.435	9.88E-35	SMC	3	Fabp4
Bok1	1.34E-36	0.340191039	0.497	0.226	2.68E-33	SMC	3	Bok
Hspa1a	1.95E-36	0.34413968	0.657	0.392	3.89E-33	SMC	3	Hspa1a
Tsc22d12	3.15E-36	0.324671329	0.778	0.543	6.31E-33	SMC	3	Tsc22d1
Wisp21	8.13E-36	0.2938579	0.552	0.271	1.63E-32	SMC	3	Wisp2
Pcdh72	6.05E-34	0.326568555	0.596	0.29	1.21E-30	SMC	3	Pcdh7
Prnp1	2.55E-33	0.254719975	0.642	0.378	5.10E-30	SMC	3	Prnp

Timp33	4.35E-33	0.535980949	0.906	0.695	8.69E-30	SMC	3	Timp3
Map3k7cl2	1.02E-32	0.407693753	0.439	0.131	2.04E-29	SMC	3	Map3k7cl
Anxa11	1.78E-32	0.297875521	0.837	0.602	3.57E-29	SMC	3	Anxa1
Adamts11	2.97E-32	0.595931359	0.531	0.251	5.95E-29	SMC	3	Adamts1
Zak2	3.04E-32	0.358200325	0.593	0.32	6.09E-29	SMC	3	Zak
Aldoa1	5.03E-29	0.282079836	0.907	0.765	1.01E-25	SMC	3	Aldoa
Dusp13	7.51E-29	0.341705999	0.74	0.492	1.50E-25	SMC	3	Dusp1
Tmem176a	8.77E-29	0.379918773	0.589	0.34	1.75E-25	SMC	3	Tmem176a
Itgb12	2.80E-28	0.272516891	0.804	0.56	5.60E-25	SMC	3	Itgb1
2-Nov	3.44E-28	0.37720664	0.568	0.322	6.88E-25	SMC	3	Nov
Col14a13	1.02E-27	0.351546485	0.766	0.553	2.05E-24	SMC	3	Col14a1
Sdpr	2.65E-27	0.322977747	0.57	0.308	5.30E-24	SMC	3	Sdpr
2-Jun	6.39E-27	0.350434626	0.75	0.52	1.28E-23	SMC	3	Jun
Ppp1r12b2	1.31E-26	0.263626043	0.587	0.328	2.63E-23	SMC	3	Ppp1r12b
G0s22	6.09E-26	0.412726803	0.542	0.313	1.22E-22	SMC	3	G0s2
H2-M9	5.88E-25	0.291989077	0.259	0.041	1.18E-21	SMC	3	H2-M9
Id4	1.05E-23	0.641985691	0.446	0.162	2.10E-20	SMC	3	Id4
Fmo24	1.20E-23	0.419078586	0.654	0.403	2.40E-20	SMC	3	Fmo2
Col4a22	2.01E-23	0.29840141	0.649	0.402	4.01E-20	SMC	3	Col4a2
Adra2a	6.51E-19	0.267837869	0.292	0.102	1.30E-15	SMC	3	Adra2a
Cxcl1	1.66E-18	0.277158689	0.39	0.186	3.32E-15	SMC	3	Cxcl1
Sncg2	9.28E-17	0.565931887	0.383	0.158	1.86E-13	SMC	3	Sncg
Myo1b	4.03E-15	0.428472543	0.358	0.124	8.05E-12	SMC	3	Myo1b
CR974586.5	7.15E-15	0.339505409	0.383	0.138	1.43E-11	SMC	3	CR974586.5
Rgs42	1.83E-14	0.492857269	0.364	0.158	3.66E-11	SMC	3	Rgs4
Ccl112	3.96E-14	0.295334507	0.554	0.397	7.93E-11	SMC	3	Ccl11
Cp2	6.12E-14	0.258037181	0.502	0.293	1.22E-10	SMC	3	Cp
Dmd2	1.34E-13	0.259423262	0.497	0.27	2.69E-10	SMC	3	Dmd
Ptprf	2.62E-13	0.255035077	0.418	0.219	5.23E-10	SMC	3	Ptprf
Ankrd1	5.50E-13	0.337851419	0.278	0.087	1.10E-09	SMC	3	Ankrd1
St52	2.12E-12	0.28873636	0.502	0.288	4.23E-09	SMC	3	St5
Crim11	2.24E-10	0.259379622	0.444	0.283	4.49E-07	SMC	3	Crim1
Cyp4b1	8.90E-09	0.579895098	0.316	0.105	1.78E-05	SMC	3	Cyp4b1
Sparcl13	1.83E-08	0.411702522	0.554	0.413	3.66E-05	SMC	3	Sparcl1
Tnnt22	1.94E-08	0.65198108	0.371	0.25	3.87E-05	SMC	3	Tnnt2
Npy1r2	1.19E-07	0.386230384	0.36	0.17	0.000238075	SMC	3	Npy1r
Gm138611	3.99E-05	0.305757186	0.315	0.118	0.079831985	SMC	3	Gm13861
Steap41	0.008201883	0.890138789	0.336	0.235	1	SMC	3	Steap4
Myh113	1.74E-47	1.321514908	1	0.392	3.47E-44	SMC	4	Myh11
Itga82	2.17E-47	1.22664539	0.98	0.358	4.34E-44	SMC	4	Itga8
Lmod13	9.42E-47	1.145086786	0.99	0.324	1.88E-43	SMC	4	Lmod1
Cnn13	1.50E-46	1.202903187	0.99	0.356	2.99E-43	SMC	4	Cnn1
Flna3	5.66E-45	1.226112717	1	0.625	1.13E-41	SMC	4	Flna
Npnt2	3.13E-44	1.141204651	0.941	0.322	6.27E-41	SMC	4	Npnt
Acta23	3.34E-44	1.438476687	1	0.752	6.68E-41	SMC	4	Acta2
Mylk3	3.45E-44	1.225352574	0.98	0.375	6.90E-41	SMC	4	Mylk
Myl93	5.68E-44	1.333842663	1	0.612	1.14E-40	SMC	4	Myl9
Tagln3	7.61E-44	1.357925556	0.99	0.467	1.52E-40	SMC	4	Tagln
Fblim12	7.70E-44	1.067489563	0.98	0.342	1.54E-40	SMC	4	Fblim1
Actn12	1.39E-41	1.04688724	0.99	0.41	2.77E-38	SMC	4	Actn1
Sost2	2.94E-41	1.180616675	0.891	0.28	5.87E-38	SMC	4	Sost
Myl63	1.45E-40	0.990749742	1	0.974	2.90E-37	SMC	4	Myl6
Sh3bgr2	2.70E-40	0.927579678	0.911	0.281	5.39E-37	SMC	4	Sh3bgr
Tpm23	1.81E-39	1.225349963	0.99	0.578	3.62E-36	SMC	4	Tpm2
Dstn3	1.85E-39	1.066115669	1	0.779	3.70E-36	SMC	4	Dstn
Smtn2	2.61E-39	0.898205254	0.95	0.362	5.22E-36	SMC	4	Smtn
Map1b3	2.75E-39	0.984352419	0.96	0.362	5.50E-36	SMC	4	Map1b
Csrp13	4.50E-39	1.051286625	0.99	0.509	8.99E-36	SMC	4	Csrp1
Col18a12	8.59E-39	1.034673448	0.901	0.347	1.72E-35	SMC	4	Col18a1
Tpm13	3.52E-38	0.908007812	1	0.602	7.03E-35	SMC	4	Tpm1
Cpe3	1.68E-37	1.053185128	0.99	0.577	3.36E-34	SMC	4	Cpe
Pbxip13	1.96E-37	0.935500075	0.97	0.482	3.92E-34	SMC	4	Pbxip1
Ppp1r14a3	6.80E-37	0.971178183	0.941	0.388	1.36E-33	SMC	4	Ppp1r14a
Dmpk3	1.12E-36	0.827109999	0.931	0.417	2.23E-33	SMC	4	Dmpk
Cald13	1.66E-36	0.978258282	0.99	0.582	3.32E-33	SMC	4	Cald1
Tns13	3.29E-36	0.966440998	0.98	0.587	6.57E-33	SMC	4	Tns1
Synpo23	3.86E-36	0.866601586	0.891	0.28	7.73E-33	SMC	4	Synpo2
Lpp3	4.57E-36	0.880691067	0.96	0.473	9.15E-33	SMC	4	Lpp
Csrp22	1.11E-35	1.074810908	0.99	0.65	2.23E-32	SMC	4	Csrp2
Pcdh73	3.32E-34	0.835993769	0.871	0.295	6.63E-31	SMC	4	Pcdh7
Rbpms4	6.27E-34	0.880960896	0.931	0.419	1.25E-30	SMC	4	Rbpms
Ncam12	7.24E-34	0.910671406	0.832	0.297	1.45E-30	SMC	4	Ncam1
Ppp1r12b3	1.04E-33	0.749876967	0.881	0.332	2.08E-30	SMC	4	Ppp1r12b
Eln4	1.32E-33	1.157562109	0.95	0.599	2.64E-30	SMC	4	Eln
Kcnmb12	1.50E-33	0.761172852	0.772	0.244	2.99E-30	SMC	4	Kcnmb1
Hspb72	5.94E-33	0.838892348	0.792	0.264	1.19E-29	SMC	4	Hspb7
Fbxl222	6.05E-33	0.83044568	0.772	0.224	1.21E-29	SMC	4	Fbxl22
Hspb62	6.20E-33	0.749646351	0.851	0.311	1.24E-29	SMC	4	Hspb6
Nexn3	2.12E-32	0.732169852	0.842	0.268	4.23E-29	SMC	4	Nexn
Serpine22	2.28E-32	0.886827238	0.941	0.42	4.56E-29	SMC	4	Serpine2
3-Nov	3.16E-32	0.95009685	0.851	0.325	6.31E-29	SMC	4	Nov
Mir143hg3	3.70E-32	0.790056017	0.802	0.244	7.40E-29	SMC	4	Mir143hg
Enah2	4.81E-32	0.800905239	0.851	0.297	9.62E-29	SMC	4	Enah
Adcy52	1.20E-31	0.760185189	0.772	0.243	2.40E-28	SMC	4	Adcy5
Lmcd13	2.18E-31	0.900173285	0.851	0.315	4.37E-28	SMC	4	Lmcd1
Pls32	7.51E-31	0.729779455	0.95	0.446	1.50E-27	SMC	4	Pls3
Actg23	5.77E-30	0.870304384	0.782	0.261	1.15E-26	SMC	4	Actg2
Ramp13	5.91E-30	0.82361172	0.901	0.427	1.18E-26	SMC	4	Ramp1
St53	3.92E-29	0.687446159	0.812	0.291	7.85E-26	SMC	4	St5
1190005I06Rik2	4.09E-29	0.64794801	0.713	0.252	8.18E-26	SMC	4	1190005I06Rik
Sgcg2	4.67E-29	0.761316173	0.673	0.229	9.34E-26	SMC	4	Sgcg
Optc2	1.50E-28	0.72125309	0.713	0.266	2.99E-25	SMC	4	Optc
Jph22	1.59E-28	0.626462751	0.713	0.238	3.18E-25	SMC	4	Jph2
Pcp4l12	1.71E-28	0.692218799	0.733	0.256	3.43E-25	SMC	4	Pcp4l1
Bcam3	1.87E-28	0.683668768	0.842	0.329	3.74E-25	SMC	4	Bcam
Myom12	1.71E-27	0.631290974	0.673	0.223	3.42E-24	SMC	4	Myom1
Slmap2	1.74E-27	0.593161786	0.891	0.353	3.48E-24	SMC	4	Slmap
Msrb13	2.10E-27	0.751621308	0.901	0.499	4.21E-24	SMC	4	Msrb1
Mcam2	3.83E-27	0.657226649	0.733	0.275	7.66E-24	SMC	4	Mcam
Cdh132	4.48E-27	0.692652456	0.743	0.298	8.97E-24	SMC	4	Cdh13
Thsd42	2.08E-26	0.553096392	0.693	0.264	4.16E-23	SMC	4	Thsd4
Aoc33	3.51E-26	0.657659483	0.762	0.336	7.02E-23	SMC	4	Aoc3
Rrad2	3.86E-26	0.663696943	0.743	0.31	7.71E-23	SMC	4	Rrad
Pdlim33	3.86E-26	0.610263038	0.792	0.288	7.73E-23	SMC	4	Pdlim3
Itga92	4.47E-26	0.654534896	0.792	0.346	8.93E-23	SMC	4	Itga9
Pdlim73	9.11E-26	0.697380937	0.861	0.416	1.82E-22	SMC	4	Pdlim7
Nudt42	1.48E-25	0.679734527	0.851	0.364	2.95E-22	SMC	4	Nudt4
Fhl13	2.32E-25	0.696410885	0.97	0.557	4.65E-22	SMC	4	Fhl1
Rcan22	2.40E-25	0.699649574	0.871	0.506	4.79E-22	SMC	4	Rcan2
Cd2003	3.13E-25	0.72503492	0.941	0.538	6.26E-22	SMC	4	Cd200
Pkp42	4.02E-25	0.584327987	0.762	0.316	8.04E-22	SMC	4	Pkp4
Itgb13	4.89E-25	0.670718267	0.95	0.565	9.77E-22	SMC	4	Itgb1
Igfbp75	6.23E-25	0.805840477	1	0.803	1.25E-21	SMC	4	Igfbp7
Gucy1a33	7.18E-25	0.607355898	0.713	0.253	1.44E-21	SMC	4	Gucy1a3
Ppp1r12a3	7.50E-25	0.620558441	0.871	0.379	1.50E-21	SMC	4	Ppp1r12a
Mrvi13	1.40E-24	0.586060468	0.693	0.273	2.79E-21	SMC	4	Mrvi1
Samd42	1.77E-24	0.626843513	0.762	0.325	3.54E-21	SMC	4	Samd4
Lrrfip12	3.47E-24	0.606401475	0.792	0.344	6.94E-21	SMC	4	Lrrfip1
Susd52	5.89E-24	0.64715319	0.683	0.301	1.18E-20	SMC	4	Susd5
Dmd3	6.29E-24	0.55333868	0.723	0.274	1.26E-20	SMC	4	Dmd
Sod35	9.99E-24	0.710762839	0.98	0.625	2.00E-20	SMC	4	Sod3
Vcl3	1.28E-23	0.631961328	0.782	0.354	2.57E-20	SMC	4	Vcl
Tgfb1i13	1.33E-23	0.590056001	0.871	0.417	2.65E-20	SMC	4	Tgfb1i1
Cped13	1.63E-23	0.668771422	0.861	0.41	3.26E-20	SMC	4	Cped1
Rassf32	1.90E-23	0.600787671	0.703	0.278	3.81E-20	SMC	4	Rassf3
Mical22	2.49E-23	0.564007453	0.693	0.255	4.99E-20	SMC	4	Mical2
Spint23	2.89E-23	0.605488	0.733	0.292	5.78E-20	SMC	4	Spint2
Gm138893	3.99E-23	0.602634001	0.624	0.202	7.98E-20	SMC	4	Gm13889
Ptgis2	6.01E-23	0.669951648	0.941	0.559	1.20E-19	SMC	4	Ptgis

Rasl123	6.59E-23	0.591011707	0.634	0.236	1.32E-19	SMC	4	Rasl12
Dst2	9.12E-23	0.599714189	0.871	0.427	1.82E-19	SMC	4	Dst
Mustn13	1.16E-22	0.861050112	0.911	0.479	2.32E-19	SMC	4	Mustn1
Rbpms22	1.47E-22	0.553775785	0.634	0.257	2.94E-19	SMC	4	Rbpms2
Fgl23	1.64E-22	0.495241335	0.851	0.404	3.28E-19	SMC	4	Fgl2
E030013I19Rik2	1.83E-22	0.668951963	0.614	0.212	3.66E-19	SMC	4	E030013I19Rik
Lgr62	2.21E-22	0.590052453	0.644	0.285	4.42E-19	SMC	4	Lgr6
Mfge83	2.32E-22	0.639615959	0.891	0.526	4.65E-19	SMC	4	Mfge8
Hspb12	2.57E-22	0.729175941	0.861	0.489	5.14E-19	SMC	4	Hspb1
Cav13	2.96E-22	0.56206552	0.891	0.416	5.93E-19	SMC	4	Cav1
Tm4sf12	4.28E-22	0.693737793	0.921	0.518	8.55E-19	SMC	4	Tm4sf1
Clu2	5.80E-22	0.60630597	0.802	0.382	1.16E-18	SMC	4	Clu
Actn43	6.57E-22	0.616331011	0.891	0.492	1.31E-18	SMC	4	Actn4
Tspan22	1.85E-21	0.563310533	0.653	0.298	3.70E-18	SMC	4	Tspan2
Vim2	2.64E-21	0.601729125	1	0.893	5.29E-18	SMC	4	Vim
Mtus22	5.31E-21	0.524731358	0.564	0.198	1.06E-17	SMC	4	Mtus2
Mylk42	2.82E-20	0.595193562	0.554	0.229	5.63E-17	SMC	4	Mylk4
Plin42	4.52E-20	0.503835306	0.584	0.229	9.05E-17	SMC	4	Plin4
Postn4	5.10E-20	0.585275794	0.842	0.472	1.02E-16	SMC	4	Postn
Cyb5r33	5.14E-20	0.571914824	0.921	0.498	1.03E-16	SMC	4	Cyb5r3
Zak3	5.82E-20	0.526079431	0.713	0.324	1.16E-16	SMC	4	Zak
Col4a23	9.66E-20	0.516823111	0.851	0.406	1.93E-16	SMC	4	Col4a2
Ccdc32	1.03E-19	0.55485563	0.614	0.269	2.05E-16	SMC	4	Ccdc3
Smarcd32	1.31E-19	0.4735773	0.653	0.287	2.62E-16	SMC	4	Smarcd3
Pde3a3	1.68E-19	0.457033131	0.663	0.282	3.36E-16	SMC	4	Pde3a
Gucy1b33	3.27E-19	0.463094771	0.604	0.256	6.54E-16	SMC	4	Gucy1b3
Cdc42ep33	7.02E-19	0.402121526	0.822	0.385	1.40E-15	SMC	4	Cdc42ep3
Hacd12	7.02E-19	0.485595604	0.733	0.343	1.40E-15	SMC	4	Hacd1
Cd79b	9.76E-19	0.290373245	0.782	0.459	1.95E-15	SMC	4	Cd79b
Kcnab12	1.09E-18	0.492951735	0.535	0.227	2.18E-15	SMC	4	Kcnab1
Cryab3	1.13E-18	0.448234108	0.931	0.532	2.27E-15	SMC	4	Cryab
Tinagl13	1.31E-18	0.434535884	0.634	0.259	2.62E-15	SMC	4	Tinagl1
Dpysl33	1.40E-18	0.548305425	0.792	0.44	2.80E-15	SMC	4	Dpysl3
Smoc13	2.72E-18	0.507220084	0.634	0.311	5.45E-15	SMC	4	Smoc1
Plcb42	3.75E-18	0.475790473	0.653	0.277	7.51E-15	SMC	4	Plcb4
Filip1l3	6.86E-18	0.5171623	0.772	0.373	1.37E-14	SMC	4	Filip1l
Cspg42	2.33E-17	0.443976013	0.564	0.263	4.65E-14	SMC	4	Cspg4
Wfdc12	2.59E-17	0.503548916	0.703	0.375	5.17E-14	SMC	4	Wfdc1
Heyl4	2.80E-17	0.424490815	0.604	0.263	5.60E-14	SMC	4	Heyl
Tcap2	1.00E-16	0.75518985	0.683	0.376	2.01E-13	SMC	4	Tcap
Fbxo322	1.36E-16	0.484471087	0.604	0.305	2.72E-13	SMC	4	Fbxo32
Hspb23	2.81E-16	0.387468321	0.604	0.279	5.62E-13	SMC	4	Hspb2
2200002D01Rik2	2.99E-16	0.516813256	0.505	0.229	5.99E-13	SMC	4	2200002D01Rik
Limch12	3.82E-16	0.527321357	0.683	0.343	7.65E-13	SMC	4	Limch1
Tgm22	6.43E-16	0.403687561	0.703	0.313	1.29E-12	SMC	4	Tgm2
Ndrg23	7.03E-16	0.504424183	0.762	0.405	1.41E-12	SMC	4	Ndrg2
Bgn4	1.57E-15	0.490353422	0.99	0.768	3.14E-12	SMC	4	Bgn
Nrtn2	1.17E-14	0.35971418	0.416	0.182	2.35E-11	SMC	4	Nrtn
Atcayos2	2.17E-14	0.424189539	0.436	0.186	4.34E-11	SMC	4	Atcayos
H2-DMb2	2.67E-14	0.252874393	0.673	0.39	5.34E-11	SMC	4	H2-DMb2
Nav22	2.81E-14	0.443256157	0.515	0.26	5.63E-11	SMC	4	Nav2
Tes2	5.28E-14	0.382030956	0.485	0.236	1.06E-10	SMC	4	Tes
Mfap43	6.71E-14	0.31190096	0.901	0.536	1.34E-10	SMC	4	Mfap4
Efhd13	3.53E-13	0.38262223	0.653	0.371	7.05E-10	SMC	4	Efhd1
Nox42	6.43E-13	0.388500038	0.584	0.301	1.29E-09	SMC	4	Nox4
Fmo25	8.73E-13	0.453400798	0.752	0.407	1.75E-09	SMC	4	Fmo2
Tmsb4x2	9.51E-13	0.275002724	1	1	1.90E-09	SMC	4	Tmsb4x
Sorbs22	1.32E-12	0.294306711	0.416	0.192	2.63E-09	SMC	4	Sorbs2
Pdgfa3	2.10E-12	0.365317383	0.614	0.327	4.19E-09	SMC	4	Pdgfa
Itga52	5.25E-12	0.419883002	0.465	0.241	1.05E-08	SMC	4	Itga5
Scube32	8.36E-12	0.372485694	0.436	0.228	1.67E-08	SMC	4	Scube3
Cd59a2	1.12E-11	0.307558657	0.455	0.239	2.25E-08	SMC	4	Cd59a
Col4a63	1.24E-11	0.397254382	0.535	0.31	2.48E-08	SMC	4	Col4a6
Tnfrsf12a2	1.70E-11	0.350045472	0.436	0.214	3.41E-08	SMC	4	Tnfrsf12a
Fbxo302	2.22E-11	0.408248737	0.554	0.338	4.44E-08	SMC	4	Fbxo30
Dsp2	2.23E-11	0.384730514	0.436	0.229	4.46E-08	SMC	4	Dsp
Itih42	2.95E-11	0.574026383	0.446	0.243	5.91E-08	SMC	4	Itih4
Ldhb1	3.46E-11	0.388392549	0.545	0.323	6.93E-08	SMC	4	Ldhb
Asb22	4.53E-11	0.343263304	0.396	0.213	9.07E-08	SMC	4	Asb2
Kank12	7.45E-11	0.317434586	0.594	0.324	1.49E-07	SMC	4	Kank1
Pdlim42	9.60E-11	0.41916985	0.653	0.433	1.92E-07	SMC	4	Pdlim4
Inhba2	1.03E-10	0.334659445	0.446	0.241	2.07E-07	SMC	4	Inhba
Net12	1.53E-10	0.285912653	0.446	0.239	3.05E-07	SMC	4	Net1
Stbd13	1.57E-10	0.304542788	0.436	0.231	3.14E-07	SMC	4	Stbd1
Ccdc422	2.65E-10	0.491630531	0.455	0.281	5.30E-07	SMC	4	Ccdc42
Nupr14	3.26E-10	0.33007785	0.931	0.681	6.52E-07	SMC	4	Nupr1
Perp2	1.11E-09	0.292399735	0.356	0.19	2.22E-06	SMC	4	Perp
Ptprz11	1.46E-09	0.291593903	0.356	0.172	2.92E-06	SMC	4	Ptprz1
Slc22a12	1.52E-09	0.477573605	0.337	0.184	3.04E-06	SMC	4	Slc22a1
Cd94	2.44E-09	0.348692999	0.891	0.649	4.88E-06	SMC	4	Cd9
Fbln54	2.63E-09	0.308809322	0.832	0.555	5.27E-06	SMC	4	Fbln5
Htra14	4.27E-09	0.250073145	0.802	0.546	8.54E-06	SMC	4	Htra1
Btg23	1.39E-08	0.30976912	0.743	0.5	2.79E-05	SMC	4	Btg2
Rgs53	2.94E-08	0.46175553	0.376	0.225	5.89E-05	SMC	4	Rgs5
Sparcl14	6.63E-08	0.262963346	0.663	0.415	0.000132653	SMC	4	Sparcl1
Sncg3	8.16E-08	0.379001452	0.267	0.163	0.00016313	SMC	4	Sncg
Pou2f2	1.22E-07	0.270272882	0.515	0.348	0.000243906	SMC	4	Pou2f2
2210407C18Rik2	1.31E-07	0.373550064	0.297	0.173	0.000261141	SMC	4	2210407C18Rik
Emb1	5.12E-07	0.272187659	0.376	0.263	0.001024106	SMC	4	Emb
Igsf92	2.84E-06	0.310665493	0.366	0.266	0.005676205	SMC	4	Igsf9
Smim52	7.52E-06	0.386395462	0.446	0.357	0.015036706	SMC	4	Smim5
Uchl12	1.75E-05	0.250199705	0.356	0.264	0.035072656	SMC	4	Uchl1
Ckb4	3.81E-05	0.278159413	0.693	0.53	0.076240556	SMC	4	Ckb
Bcl11a	5.89E-05	0.269129973	0.366	0.264	0.117844547	SMC	4	Bcl11a
Myh114	4.12E-25	1.456604896	1	0.393	8.24E-22	SMC	5	Myh11
Npnt3	5.73E-25	1.288577914	0.979	0.324	1.15E-21	SMC	5	Npnt
Actn13	1.45E-23	1.188067509	1	0.412	2.89E-20	SMC	5	Actn1
Mcam3	1.94E-23	1.056234257	0.917	0.275	3.89E-20	SMC	5	Mcam
Cnn14	1.98E-23	1.217733802	1	0.357	3.96E-20	SMC	5	Cnn1
Mylk5	2.05E-23	1.274130589	1	0.377	4.10E-20	SMC	5	Mylk
Acta24	2.12E-23	1.580154245	1	0.752	4.24E-20	SMC	5	Acta2
Flna4	3.10E-23	1.278269976	1	0.625	6.20E-20	SMC	5	Flna
Lpp4	5.12E-23	1.150140747	1	0.474	1.02E-19	SMC	5	Lpp
Tagln4	5.72E-23	1.446630471	1	0.469	1.14E-19	SMC	5	Tagln
Fblim13	6.97E-23	1.080116017	1	0.343	1.39E-19	SMC	5	Fblim1
Sh3bgr3	1.14E-22	1.145310972	0.938	0.283	2.27E-19	SMC	5	Sh3bgr
Smtn3	1.43E-22	1.064316415	0.979	0.363	2.86E-19	SMC	5	Smtn
Itga83	2.10E-22	1.196768666	0.979	0.36	4.20E-19	SMC	5	Itga8
Tpm24	2.15E-22	1.351516392	1	0.579	4.31E-19	SMC	5	Tpm2
Synpo24	2.18E-22	0.985100682	0.979	0.282	4.37E-19	SMC	5	Synpo2
Col18a13	3.27E-22	1.090740272	0.958	0.348	6.54E-19	SMC	5	Col18a1
Myl94	4.83E-22	1.375473217	1	0.613	9.66E-19	SMC	5	Myl9
1190005I06Rik3	5.20E-22	0.858068366	0.875	0.253	1.04E-18	SMC	5	1190005I06Rik
Ppp1r12a4	5.98E-22	1.014900497	0.979	0.38	1.20E-18	SMC	5	Ppp1r12a
Ppp1r12b4	6.40E-22	0.935448385	0.958	0.333	1.28E-18	SMC	5	Ppp1r12b
Pkp43	1.20E-21	0.856869256	0.938	0.317	2.39E-18	SMC	5	Pkp4
Tpm14	1.20E-21	1.076375861	1	0.603	2.39E-18	SMC	5	Tpm1
Lmod14	1.22E-21	1.16096207	0.979	0.325	2.44E-18	SMC	5	Lmod1
Mrc1	1.82E-21	0.713372246	0.938	0.292	3.64E-18	SMC	5	Mrc1
F13a1	2.97E-21	0.721044403	0.938	0.267	5.94E-18	SMC	5	F13a1
Myl64	3.87E-21	1.054212524	1	0.974	7.74E-18	SMC	5	Myl6
Dstn4	5.58E-21	1.163034041	1	0.779	1.12E-17	SMC	5	Dstn
Cpe4	6.40E-21	1.134645874	1	0.578	1.28E-17	SMC	5	Cpe
Tns14	7.68E-21	1.032201244	1	0.587	1.54E-17	SMC	5	Tns1
Pls33	1.02E-20	0.918777585	1	0.447	2.04E-17	SMC	5	Pls3
Pbxip14	1.40E-20	1.02322923	0.979	0.483	2.81E-17	SMC	5	Pbxip1
Map1b4	1.80E-20	1.05453701	0.979	0.363	3.60E-17	SMC	5	Map1b
Pdlim74	2.11E-20	1.018654249	0.958	0.417	4.22E-17	SMC	5	Pdlim7
Bcam4	2.66E-20	0.976378323	0.938	0.33	5.32E-17	SMC	5	Bcam

Cald14	3.28E-20	1.083110103	1	0.583	6.56E-17	SMC	5	Cald1
Kcnmb13	5.03E-20	0.713801648	0.875	0.245	1.01E-16	SMC	5	Kcnmb1
Csrp14	6.66E-20	1.031603124	1	0.51	1.33E-16	SMC	5	Csrp1
Rasl124	1.21E-19	0.777013325	0.833	0.237	2.42E-16	SMC	5	Rasl12
Ppp1r14a4	1.33E-19	0.924930395	1	0.389	2.65E-16	SMC	5	Ppp1r14a
Enah3	1.44E-19	0.86852504	0.938	0.298	2.88E-16	SMC	5	Enah
Pcp4l13	1.46E-19	0.785444984	0.875	0.257	2.91E-16	SMC	5	Pcp4l1
Sgcg3	2.87E-19	0.611968698	0.833	0.23	5.73E-16	SMC	5	Sgcg
Ramp14	4.05E-19	0.871152376	1	0.428	8.09E-16	SMC	5	Ramp1
4-Nov	6.14E-19	1.016483994	0.938	0.326	1.23E-15	SMC	5	Nov
Pcdh74	6.62E-19	0.772709324	0.938	0.296	1.32E-15	SMC	5	Pcdh7
Nexn4	7.37E-19	0.771263131	0.938	0.269	1.47E-15	SMC	5	Nexn
Serpine23	7.67E-19	0.951959314	0.979	0.421	1.53E-15	SMC	5	Serpine2
Rbpms5	7.88E-19	0.864674912	0.979	0.42	1.58E-15	SMC	5	Rbpms
Hspb73	8.28E-19	0.906733321	0.854	0.265	1.66E-15	SMC	5	Hspb7
Susd53	1.45E-18	0.743560516	0.833	0.301	2.90E-15	SMC	5	Susd5
Kcnab13	1.66E-18	0.545162913	0.771	0.227	3.31E-15	SMC	5	Kcnab1
Cd36	2.50E-18	0.662599103	0.896	0.256	5.01E-15	SMC	5	Cd36
Atcayos3	3.04E-18	0.428967997	0.688	0.186	6.09E-15	SMC	5	Atcayos
Smarcd33	4.13E-18	0.720716464	0.854	0.288	8.26E-15	SMC	5	Smarcd3
Adgre1	4.46E-18	0.449446983	0.771	0.21	8.92E-15	SMC	5	Adgre1
Optc3	5.67E-18	0.691614366	0.812	0.266	1.13E-14	SMC	5	Optc
Dmpk4	7.60E-18	0.862984023	0.938	0.418	1.52E-14	SMC	5	Dmpk
Mir143hg4	1.09E-17	0.765262571	0.896	0.245	2.17E-14	SMC	5	Mir143hg
Dsp3	1.40E-17	0.44547576	0.771	0.229	2.80E-14	SMC	5	Dsp
Scube33	1.58E-17	0.522769911	0.75	0.227	3.16E-14	SMC	5	Scube3
Ctsc	1.88E-17	0.667323872	0.958	0.345	3.77E-14	SMC	5	Ctsc
Ncam13	2.12E-17	0.946784625	0.833	0.298	4.25E-14	SMC	5	Ncam1
Csrp23	2.76E-17	0.94010633	1	0.651	5.53E-14	SMC	5	Csrp2
Csf1r	3.08E-17	0.652496642	0.896	0.28	6.16E-14	SMC	5	Csf1r
Hacd13	3.13E-17	0.871762795	0.875	0.344	6.27E-14	SMC	5	Hacd1
Pdlim34	3.24E-17	0.73762301	0.896	0.289	6.47E-14	SMC	5	Pdlim3
Thsd43	5.26E-17	0.809994725	0.771	0.264	1.05E-13	SMC	5	Thsd4
C1qc	6.87E-17	0.325667973	0.938	0.391	1.37E-13	SMC	5	C1qc
Cdh133	8.14E-17	0.611159071	0.854	0.299	1.63E-13	SMC	5	Cdh13
Plin43	9.06E-17	0.778500954	0.75	0.23	1.81E-13	SMC	5	Plin4
Sost3	9.17E-17	1.173412351	0.812	0.282	1.83E-13	SMC	5	Sost
Zak4	2.00E-16	0.795503843	0.833	0.325	4.00E-13	SMC	5	Zak
Spint24	2.48E-16	0.711183755	0.875	0.293	4.95E-13	SMC	5	Spint2
Vim3	2.59E-16	0.825071189	1	0.893	5.18E-13	SMC	5	Vim
Pde3a4	2.85E-16	0.579174697	0.854	0.282	5.70E-13	SMC	5	Pde3a
Itgb14	4.13E-16	0.786989662	0.979	0.565	8.25E-13	SMC	5	Itgb1
C5ar1	6.01E-16	0.499633728	0.708	0.179	1.20E-12	SMC	5	C5ar1
Jph23	6.65E-16	0.596902822	0.812	0.239	1.33E-12	SMC	5	Jph2
Rbpms23	6.83E-16	0.61902501	0.75	0.257	1.37E-12	SMC	5	Rbpms2
Cped14	8.36E-16	0.66318896	1	0.411	1.67E-12	SMC	5	Cped1
Clu3	1.01E-15	0.721418043	0.938	0.382	2.02E-12	SMC	5	Clu
Nav23	1.38E-15	0.513745402	0.792	0.26	2.76E-12	SMC	5	Nav2
Dst3	1.40E-15	0.774258193	0.938	0.428	2.79E-12	SMC	5	Dst
Myom13	2.97E-15	0.774975235	0.771	0.224	5.95E-12	SMC	5	Myom1
Mical23	3.51E-15	0.614959259	0.812	0.256	7.01E-12	SMC	5	Mical2
Eln5	5.26E-15	1.007315917	0.979	0.6	1.05E-11	SMC	5	Eln
Lmcd14	7.63E-15	0.631216682	0.896	0.316	1.53E-11	SMC	5	Lmcd1
Cbr21	8.56E-15	0.439471389	0.979	0.401	1.71E-11	SMC	5	Cbr2
Actg24	1.11E-14	0.989435489	0.792	0.262	2.23E-11	SMC	5	Actg2
Itih43	1.31E-14	0.583532065	0.729	0.243	2.62E-11	SMC	5	Itih4
Dab2	1.60E-14	0.642782513	0.896	0.392	3.19E-11	SMC	5	Dab2
Adcy53	1.62E-14	0.610627816	0.833	0.244	3.24E-11	SMC	5	Adcy5
Ccdc33	2.22E-14	0.509137642	0.771	0.269	4.43E-11	SMC	5	Ccdc3
Vcl4	2.49E-14	0.664567624	0.875	0.355	4.98E-11	SMC	5	Vcl
Tes3	3.17E-14	0.465350522	0.708	0.237	6.35E-11	SMC	5	Tes
Slmap3	3.92E-14	0.64778273	0.917	0.354	7.84E-11	SMC	5	Slmap
Mfge84	4.76E-14	0.788113471	0.938	0.526	9.52E-11	SMC	5	Mfge8
Hspb63	6.49E-14	0.738754163	0.812	0.312	1.30E-10	SMC	5	Hspb6
Lgr63	9.02E-14	0.677312801	0.708	0.286	1.80E-10	SMC	5	Lgr6
Hspb24	9.63E-14	0.623629315	0.75	0.28	1.93E-10	SMC	5	Hspb2
Stab1	1.01E-13	0.61223231	0.792	0.226	2.03E-10	SMC	5	Stab1
Smim53	1.13E-13	0.524518669	0.75	0.357	2.27E-10	SMC	5	Smim5
Postn5	1.14E-13	0.672369614	0.938	0.473	2.29E-10	SMC	5	Postn
Tcap3	1.20E-13	0.910163357	0.833	0.376	2.40E-10	SMC	5	Tcap
Sepp1	1.43E-13	0.480592061	1	0.823	2.86E-10	SMC	5	Sepp1
Fbxl223	1.89E-13	0.85678258	0.771	0.225	3.78E-10	SMC	5	Fbxl22
Sorbs23	2.96E-13	0.536489056	0.667	0.192	5.91E-10	SMC	5	Sorbs2
2200002D01Rik	3.05E-13	0.498399657	0.708	0.229	6.11E-10	SMC	5	2200002D01Rik
Ndrg24	3.74E-13	0.445103875	1	0.406	7.48E-10	SMC	5	Ndrg2
St54	5.28E-13	0.786838249	0.812	0.292	1.06E-09	SMC	5	St5
Bex42	5.45E-13	0.289599143	0.583	0.184	1.09E-09	SMC	5	Bex4
Tgm23	9.03E-13	0.505474311	0.896	0.313	1.81E-09	SMC	5	Tgm2
Fgl24	1.01E-12	0.557057784	0.896	0.405	2.02E-09	SMC	5	Fgl2
Bmp31	1.17E-12	0.424734309	0.583	0.167	2.34E-09	SMC	5	Bmp3
Efhd14	1.25E-12	0.437540022	0.854	0.371	2.51E-09	SMC	5	Efhd1
Heyl5	1.30E-12	0.659857123	0.708	0.263	2.59E-09	SMC	5	Heyl
Actn44	1.59E-12	0.71403302	0.896	0.492	3.17E-09	SMC	5	Actn4
Tm4sf13	1.63E-12	0.699609112	0.979	0.518	3.26E-09	SMC	5	Tm4sf1
Asb23	1.82E-12	0.379116523	0.646	0.213	3.64E-09	SMC	5	Asb2
Mylk43	2.07E-12	0.724896178	0.708	0.229	4.14E-09	SMC	5	Mylk4
Rcan23	2.09E-12	0.644486762	0.917	0.507	4.17E-09	SMC	5	Rcan2
Sod36	2.13E-12	0.756020663	1	0.626	4.25E-09	SMC	5	Sod3
Tgfb1i14	2.50E-12	0.712593187	0.875	0.418	5.01E-09	SMC	5	Tgfb1i1
Slc22a13	2.51E-12	0.514804843	0.542	0.184	5.01E-09	SMC	5	Slc22a1
Lrrfip13	2.63E-12	0.68659288	0.833	0.345	5.27E-09	SMC	5	Lrrfip1
Cd2004	2.64E-12	0.568787368	0.979	0.539	5.29E-09	SMC	5	Cd200
Nes3	3.09E-12	0.355628035	0.708	0.222	6.18E-09	SMC	5	Nes
Mustn14	3.98E-12	0.819628154	0.917	0.48	7.95E-09	SMC	5	Mustn1
Maf	4.05E-12	0.556250221	0.896	0.474	8.10E-09	SMC	5	Maf
Msrb14	4.25E-12	0.675882377	0.938	0.499	8.51E-09	SMC	5	Msrb1
Cyb5r34	4.58E-12	0.716202546	0.958	0.498	9.16E-09	SMC	5	Cyb5r3
Mrvi14	4.63E-12	0.79902508	0.75	0.274	9.26E-09	SMC	5	Mrvi1
Ldhb2	4.74E-12	0.431968218	0.792	0.323	9.48E-09	SMC	5	Ldhb
Fhl14	6.63E-12	0.6024516	0.979	0.558	1.33E-08	SMC	5	Fhl1
Aoc34	8.17E-12	0.487442444	0.792	0.337	1.63E-08	SMC	5	Aoc3
Hspb13	8.30E-12	0.721586372	0.896	0.49	1.66E-08	SMC	5	Hspb1
Tuba1c1	8.75E-12	0.422458903	0.708	0.33	1.75E-08	SMC	5	Tuba1c
Casq11	9.01E-12	0.271672097	0.583	0.25	1.80E-08	SMC	5	Casq1
Dpysl34	9.18E-12	0.543996825	0.875	0.44	1.84E-08	SMC	5	Dpysl3
Tspan23	9.56E-12	0.435574751	0.729	0.298	1.91E-08	SMC	5	Tspan2
Cav14	9.93E-12	0.589416585	0.938	0.417	1.99E-08	SMC	5	Cav1
Pdgfa4	9.97E-12	0.453989209	0.792	0.327	1.99E-08	SMC	5	Pdgfa
Igfbp76	1.41E-11	0.643229957	1	0.803	2.81E-08	SMC	5	Igfbp7
Itga93	1.43E-11	0.456274944	0.812	0.347	2.87E-08	SMC	5	Itga9
Cybb	1.55E-11	0.287246662	0.792	0.314	3.09E-08	SMC	5	Cybb
Wfdc13	2.04E-11	0.490997711	0.812	0.376	4.07E-08	SMC	5	Wfdc1
Rassf33	2.13E-11	0.471901157	0.75	0.279	4.25E-08	SMC	5	Rassf3
Ednrb	2.52E-11	0.387842842	0.625	0.207	5.04E-08	SMC	5	Ednrb
Ptgis3	4.12E-11	0.628445554	0.979	0.559	8.25E-08	SMC	5	Ptgis
Tinagl14	4.13E-11	0.470283308	0.729	0.26	8.26E-08	SMC	5	Tinagl1
Gm138894	5.92E-11	0.627850135	0.708	0.203	1.18E-07	SMC	5	Gm13889
Fabp31	6.37E-11	0.251986017	0.625	0.19	1.27E-07	SMC	5	Fabp3
Filip1l4	7.65E-11	0.526743404	0.896	0.374	1.53E-07	SMC	5	Filip1l
Col4a24	1.10E-10	0.482324929	0.875	0.407	2.20E-07	SMC	5	Col4a2
Nrtn3	1.92E-10	0.29292349	0.542	0.182	3.84E-07	SMC	5	Nrtn
Ninj1	2.43E-10	0.411973178	0.854	0.417	4.87E-07	SMC	5	Ninj1
Cd163	2.72E-10	0.507537887	0.646	0.226	5.45E-07	SMC	5	Cd163
E030013I19Rik3	2.82E-10	0.664228271	0.688	0.213	5.63E-07	SMC	5	E030013I19Rik
Plcb43	3.28E-10	0.698503462	0.708	0.277	6.56E-07	SMC	5	Plcb4
Nudt43	3.42E-10	0.480996437	0.875	0.365	6.83E-07	SMC	5	Nudt4
Fbxo323	4.33E-10	0.45181533	0.708	0.305	8.66E-07	SMC	5	Fbxo32
Prelp3	4.35E-10	0.525203093	0.938	0.548	8.71E-07	SMC	5	Prelp
Crim12	6.12E-10	0.442565139	0.646	0.286	1.22E-06	SMC	5	Crim1

Fos3	7.70E-10	0.678607288	0.875	0.507	1.54E-06	SMC	5	Fos
Fbxo303	9.29E-10	0.512694511	0.729	0.338	1.86E-06	SMC	5	Fbxo30
Gucy1b34	1.09E-09	0.502312146	0.646	0.257	2.17E-06	SMC	5	Gucy1b3
Cryab4	1.39E-09	0.425015956	1	0.533	2.79E-06	SMC	5	Cryab
Ano13	1.40E-09	0.309485865	0.604	0.247	2.81E-06	SMC	5	Ano1
Cd209f	1.66E-09	0.298411287	0.583	0.217	3.32E-06	SMC	5	Cd209f
Pdlim43	2.12E-09	0.456590252	0.792	0.433	4.25E-06	SMC	5	Pdlim4
Trf	2.14E-09	0.346806088	0.792	0.332	4.27E-06	SMC	5	Trf
Pi153	2.15E-09	0.348591523	0.792	0.345	4.31E-06	SMC	5	Pi15
Cdc42ep34	3.72E-09	0.481013873	0.833	0.386	7.44E-06	SMC	5	Cdc42ep3
Ccdc423	4.37E-09	0.370929751	0.604	0.281	8.74E-06	SMC	5	Ccdc42
Nupr15	4.43E-09	0.4928221	0.979	0.682	8.85E-06	SMC	5	Nupr1
Atf3	5.27E-09	0.432233911	0.583	0.281	1.05E-05	SMC	5	Atf3
Actb2	6.28E-09	0.525052604	1	0.994	1.26E-05	SMC	5	Actb
Cd95	6.36E-09	0.420772777	1	0.649	1.27E-05	SMC	5	Cd9
Fmo26	8.32E-09	0.548130182	0.854	0.408	1.66E-05	SMC	5	Fmo2
Kctd12	8.65E-09	0.257833041	0.75	0.361	1.73E-05	SMC	5	Kctd12
Samd43	8.79E-09	0.522101715	0.792	0.326	1.76E-05	SMC	5	Samd4
Zfp361	9.04E-09	0.427368414	0.833	0.468	1.81E-05	SMC	5	Zfp36
Hspa1b	1.02E-08	0.260701421	0.646	0.305	2.04E-05	SMC	5	Hspa1b
Clec10a	1.33E-08	0.472214758	0.604	0.18	2.66E-05	SMC	5	Clec10a
Smoc14	1.33E-08	0.309183494	0.667	0.312	2.67E-05	SMC	5	Smoc1
Mmp9	1.85E-08	0.455862016	0.562	0.216	3.70E-05	SMC	5	Mmp9
Lyve1	2.05E-08	0.344656519	0.604	0.237	4.09E-05	SMC	5	Lyve1
Rrad3	2.17E-08	0.331184017	0.688	0.311	4.33E-05	SMC	5	Rrad
Id22	2.62E-08	0.389980098	0.917	0.517	5.23E-05	SMC	5	Id2
Pam3	4.06E-08	0.470362952	0.979	0.647	8.12E-05	SMC	5	Pam
Fcgr3	5.99E-08	0.349241675	0.667	0.249	0.000119838	SMC	5	Fcgr3
Ctgf3	8.24E-08	0.417227476	0.625	0.229	0.000164833	SMC	5	Ctgf
Emb2	9.52E-08	0.253974687	0.583	0.263	0.000190483	SMC	5	Emb
Bgn5	9.58E-08	0.482787175	0.979	0.768	0.000191573	SMC	5	Bgn
Slc25a44	1.64E-07	0.385414891	0.979	0.767	0.000327944	SMC	5	Slc25a4
Kcnj15	1.73E-07	0.381614541	0.479	0.163	0.000345066	SMC	5	Kcnj15
Tmsb4x3	2.77E-07	0.29315198	1	1	0.000554781	SMC	5	Tmsb4x
Gucy1a34	2.90E-07	0.522180146	0.667	0.254	0.000580562	SMC	5	Gucy1a3
Gas63	3.62E-07	0.281048683	0.958	0.567	0.000724754	SMC	5	Gas6
Limch13	5.96E-07	0.405542047	0.708	0.344	0.001191175	SMC	5	Limch1
Mafb	6.94E-07	0.281392876	0.688	0.334	0.001388058	SMC	5	Mafb
Ckb5	7.80E-07	0.511311762	0.854	0.53	0.001559438	SMC	5	Ckb
Fcgr2b	1.14E-06	0.398040672	0.583	0.25	0.00228521	SMC	5	Fcgr2b
AI6078731	1.19E-06	0.285394719	0.562	0.196	0.002375578	SMC	5	AI607873
Mdh11	1.32E-06	0.354402043	0.854	0.525	0.002640546	SMC	5	Mdh1
Sparcl15	1.34E-06	0.594117136	0.792	0.415	0.002670434	SMC	5	Sparcl1
Kank13	1.57E-06	0.442610546	0.667	0.325	0.003148274	SMC	5	Kank1
Nr4a11	1.72E-06	0.253047065	0.625	0.384	0.003433908	SMC	5	Nr4a1
Ms4a6c	1.85E-06	0.2983206	0.604	0.199	0.003709771	SMC	5	Ms4a6c
Npy1r3	1.88E-06	0.380160931	0.521	0.174	0.003763878	SMC	5	Npy1r
Tsc22d13	2.47E-06	0.293272979	0.875	0.548	0.004949974	SMC	5	Tsc22d1
Lgmn	2.76E-06	0.351739296	0.771	0.464	0.005521951	SMC	5	Lgmn
Hspa1a1	2.97E-06	0.37146123	0.667	0.397	0.005932483	SMC	5	Hspa1a
Pltp	6.98E-06	0.317080027	0.75	0.407	0.013968105	SMC	5	Pltp
Mtus23	1.44E-05	0.59676612	0.583	0.199	0.028705046	SMC	5	Mtus2
Stard8	1.49E-05	0.250994759	0.562	0.298	0.029847988	SMC	5	Stard8
Dmd4	1.69E-05	0.411048657	0.646	0.275	0.033744105	SMC	5	Dmd
Ehd4	2.69E-05	0.352391858	0.708	0.388	0.053884292	SMC	5	Ehd4
Lgals14	4.64E-05	0.308641604	1	0.896	0.092887663	SMC	5	Lgals1
Aldoa2	5.96E-05	0.303325534	0.979	0.768	0.119179071	SMC	5	Aldoa
Slc25a342	6.75E-05	0.360152175	0.417	0.146	0.134905984	SMC	5	Slc25a34
Igfbp21	8.07E-05	0.276381981	0.375	0.141	0.161322634	SMC	5	Igfbp2
Nox43	0.000311924	0.274817293	0.625	0.301	0.623847155	SMC	5	Nox4
Dusp14	0.000531914	0.310196949	0.75	0.497	1	SMC	5	Dusp1
Tuba1b2	0.000661411	0.269706771	0.833	0.662	1	SMC	5	Tuba1b
Btg24	0.00093703	0.29170545	0.729	0.5	1	SMC	5	Btg2
Rasl11a3	0.001227966	0.279406332	0.5	0.181	1	SMC	5	Rasl11a
Col4a64	0.002534923	0.266532685	0.583	0.31	1	SMC	5	Col4a6
Fabp41	0	4.205197623	0.987	0.429	0	EC	1	Fabp4
Gpihbp1	0	2.72276984	0.941	0.074	0	EC	1	Gpihbp1
Egfl7	0	2.489323355	0.983	0.142	0	EC	1	Egfl7
Rgcc	0	2.433110519	0.939	0.2	0	EC	1	Rgcc
Flt1	0	2.258970762	0.933	0.128	0	EC	1	Flt1
Emcn	0	1.953642269	0.902	0.054	0	EC	1	Emcn
Esam1	0	1.913521238	0.927	0.187	0	EC	1	Esam
Tmem88	0	1.561900002	0.858	0.096	0	EC	1	Tmem88
Mmrn2	0	1.40591043	0.86	0.11	0	EC	1	Mmrn2
Ushbp1	0	1.338904783	0.812	0.076	0	EC	1	Ushbp1
Tie1	0	1.259507628	0.833	0.103	0	EC	1	Tie1
Sox17	0	1.256728796	0.789	0.026	0	EC	1	Sox17
Aplnr	0	1.154675627	0.826	0.032	0	EC	1	Aplnr
Gm12002	0	1.144722467	0.734	0.036	0	EC	1	Gm12002
Grrp1	0	1.14326967	0.757	0.042	0	EC	1	Grrp1
C130074G19Rik	0	1.007550409	0.822	0.147	0	EC	1	C130074G19Rik
Robo4	0	0.94013471	0.751	0.032	0	EC	1	Robo4
Pcdh17	0	0.748028677	0.745	0.058	0	EC	1	Pcdh17
Fam167b	0	0.688638883	0.711	0.064	0	EC	1	Fam167b
Esm1	0	0.562303228	0.678	0.03	0	EC	1	Esm1
Cyp1a1	0	0.467745882	0.615	0.03	0	EC	1	Cyp1a1
Exoc3l2	0	0.412469396	0.644	0.015	0	EC	1	Exoc3l2
Tmem252	0	0.411132587	0.628	0.026	0	EC	1	Tmem252
Arhgef15	8.41E-307	1.094369153	0.768	0.072	1.68E-303	EC	1	Arhgef15
Tcf15	1.50E-306	2.164064803	0.872	0.079	3.00E-303	EC	1	Tcf15
Cdh5	1.87E-303	2.019126328	0.906	0.134	3.74E-300	EC	1	Cdh5
Cd361	2.03E-300	2.151202262	0.967	0.243	4.06E-297	EC	1	Cd36
Ly6c12	1.06E-297	2.396201794	1	0.41	2.12E-294	EC	1	Ly6c1
Cd300lg	2.41E-291	1.950937328	0.9	0.153	4.82E-288	EC	1	Cd300lg
Tspan13	8.97E-285	2.026089315	0.939	0.269	1.79E-281	EC	1	Tspan13
Myct1	6.03E-277	1.376032829	0.822	0.102	1.21E-273	EC	1	Myct1
Scarb1	2.07E-275	1.744908587	0.887	0.229	4.13E-272	EC	1	Scarb1
Nos3	9.37E-273	0.35509583	0.676	0.084	1.87E-269	EC	1	Nos3
Cd931	1.61E-268	1.660012528	0.893	0.191	3.22E-265	EC	1	Cd93
Mgll1	3.72E-264	1.922323187	0.956	0.392	7.44E-261	EC	1	Mgll
Ptprb	4.74E-262	1.710638922	0.881	0.165	9.49E-259	EC	1	Ptprb
Slc9a3r2	3.13E-257	1.846767702	0.914	0.256	6.25E-254	EC	1	Slc9a3r2
Apold1	7.02E-257	0.689205978	0.69	0.084	1.40E-253	EC	1	Apold1
Slc26a10	4.39E-255	0.503975593	0.582	0.043	8.79E-252	EC	1	Slc26a10
Sdpr1	1.25E-250	1.882300792	0.933	0.301	2.50E-247	EC	1	Sdpr
Rasip1	7.62E-246	1.141454189	0.755	0.062	1.52E-242	EC	1	Rasip1
C1qtnf9	1.42E-245	1.751478197	0.797	0.081	2.85E-242	EC	1	C1qtnf9
Ctla2a	2.46E-242	1.982918967	0.877	0.18	4.93E-239	EC	1	Ctla2a
Gng112	2.98E-238	1.789263446	0.944	0.439	5.96E-235	EC	1	Gng11
Id1	2.59E-230	1.787952284	0.925	0.281	5.18E-227	EC	1	Id1
Arhgap31	1.04E-227	1.597204805	0.908	0.308	2.09E-224	EC	1	Arhgap31
Adgrf5	9.10E-226	1.740078521	0.847	0.123	1.82E-222	EC	1	Adgrf5
Ramp22	1.14E-222	1.637312425	0.962	0.386	2.28E-219	EC	1	Ramp2
Cav15	3.07E-217	1.5606652	0.946	0.407	6.13E-214	EC	1	Cav1
Eng	3.62E-216	1.541526254	0.908	0.325	7.25E-213	EC	1	Eng
Fam101b	2.74E-214	1.688810318	0.841	0.172	5.49E-211	EC	1	Fam101b
Kdr	4.04E-213	1.880693731	0.824	0.156	8.09E-210	EC	1	Kdr
Pecam1	2.36E-212	1.587259959	0.887	0.277	4.72E-209	EC	1	Pecam1
Adgrl4	1.64E-207	1.209553463	0.732	0.043	3.29E-204	EC	1	Adgrl4
Tspan7	8.20E-195	1.504459554	0.843	0.297	1.64E-191	EC	1	Tspan7
AW1120101	9.79E-195	1.464708364	0.941	0.397	1.96E-191	EC	1	AW112010
Epas11	1.12E-194	1.504012485	0.864	0.233	2.24E-191	EC	1	Epas1
Sparcl16	9.86E-189	1.375973792	0.937	0.406	1.97E-185	EC	1	Sparcl1
Unc45b	3.23E-184	1.283617961	0.766	0.139	6.47E-181	EC	1	Unc45b
Cd2005	2.64E-181	1.480972166	0.971	0.531	5.28E-178	EC	1	Cd200
Gimap6	7.87E-179	1.198562834	0.912	0.413	1.57E-175	EC	1	Gimap6
Ecscr1	5.18E-178	1.242150927	0.824	0.239	1.04E-174	EC	1	Ecscr
Gja41	1.10E-176	0.273075492	0.697	0.145	2.20E-173	EC	1	Gja4
Sep-42	2.00E-176	1.176892827	0.791	0.227	4.01E-173	EC	1	4-Sep

BC028528	6.24E-171	0.852621135	0.839	0.301	1.25E-167	EC	1	BC028528
Cyyr1	3.01E-170	1.330269443	0.707	0.048	6.03E-167	EC	1	Cyyr1
Pdzd2	4.58E-169	1.345983541	0.82	0.255	9.15E-166	EC	1	Pdzd2
Hspb14	7.60E-167	1.084009869	0.946	0.481	1.52E-163	EC	1	Hspb1
Aqp11	7.89E-165	2.354227306	0.858	0.35	1.58E-161	EC	1	Aqp1
Plvap	1.39E-164	1.649785095	0.78	0.296	2.79E-161	EC	1	Plvap
Cxcl123	1.07E-158	1.523560619	0.885	0.384	2.13E-155	EC	1	Cxcl12
Cldn5	6.59E-158	1.791908338	0.751	0.119	1.32E-154	EC	1	Cldn5
Ly6e	5.26E-154	1.033389104	0.998	0.791	1.05E-150	EC	1	Ly6e
Plk2	1.25E-152	0.828261947	0.709	0.181	2.51E-149	EC	1	Plk2
Tinagl15	5.99E-152	1.332383456	0.822	0.249	1.20E-148	EC	1	Tinagl1
Flt4	7.55E-152	0.267355334	0.605	0.104	1.51E-148	EC	1	Flt4
Vwf	9.47E-152	0.492724468	0.674	0.167	1.89E-148	EC	1	Vwf
Smagp	1.07E-151	0.701364487	0.795	0.227	2.14E-148	EC	1	Smagp
Ece1	4.31E-150	0.862675167	0.851	0.299	8.62E-147	EC	1	Ece1
Kank3	1.76E-148	1.311026222	0.768	0.218	3.52E-145	EC	1	Kank3
Jam2	1.83E-147	1.187891286	0.766	0.215	3.66E-144	EC	1	Jam2
Higd1b1	2.76E-147	0.4271086	0.596	0.064	5.52E-144	EC	1	Higd1b
Clec14a	2.92E-147	0.742050308	0.728	0.23	5.83E-144	EC	1	Clec14a
Ssu2	5.34E-147	0.330260487	0.464	0.021	1.07E-143	EC	1	Ssu2
Nrarp	2.89E-146	0.624786655	0.626	0.112	5.78E-143	EC	1	Nrarp
Lrg1	1.87E-145	0.722078572	0.63	0.071	3.75E-142	EC	1	Lrg1
Crim13	4.88E-139	0.794859275	0.799	0.277	9.76E-136	EC	1	Crim1
Ly6a2	9.83E-137	1.020305309	0.99	0.655	1.97E-133	EC	1	Ly6a
Sncg4	7.04E-131	0.56449655	0.718	0.152	1.41E-127	EC	1	Sncg
Cd38	1.27E-126	0.768802093	0.728	0.198	2.54E-123	EC	1	Cd38
Srgn	1.39E-126	0.922690685	0.921	0.49	2.78E-123	EC	1	Srgn
1810011O10Rik	2.35E-126	1.297476042	0.703	0.169	4.70E-123	EC	1	1810011O10Rik
Fabp5	6.70E-126	1.625429405	0.755	0.216	1.34E-122	EC	1	Fabp5
Tm4sf14	1.18E-124	0.986191602	0.937	0.511	2.35E-121	EC	1	Tm4sf1
Bst2	2.53E-124	1.165787727	0.902	0.488	5.06E-121	EC	1	Bst2
Rsad2	1.02E-123	1.314818117	0.678	0.16	2.05E-120	EC	1	Rsad2
Bcam5	1.11E-123	0.862337297	0.858	0.32	2.22E-120	EC	1	Bcam
Timp4	2.70E-121	1.664301552	0.697	0.095	5.41E-118	EC	1	Timp4
Col4a25	2.83E-119	1.042265301	0.854	0.399	5.66E-116	EC	1	Col4a2
H2-Q7	3.53E-119	0.638915322	0.833	0.371	7.07E-116	EC	1	H2-Q7
Igfbp33	1.41E-118	0.914295532	0.699	0.243	2.83E-115	EC	1	Igfbp3
Id33	1.36E-116	0.939816133	0.975	0.685	2.71E-113	EC	1	Id3
Art3	1.32E-114	0.305784271	0.676	0.23	2.65E-111	EC	1	Art3
S1pr1	5.82E-114	1.22055198	0.766	0.287	1.16E-110	EC	1	S1pr1
B2m	6.40E-114	0.624889112	1	0.983	1.28E-110	EC	1	B2m
Pcp4l14	4.57E-113	0.531671281	0.768	0.248	9.14E-110	EC	1	Pcp4l1
Iigp1	6.92E-112	0.84087253	0.736	0.294	1.38E-108	EC	1	Iigp1
Ifi44	1.51E-111	0.425903921	0.536	0.061	3.02E-108	EC	1	Ifi44
Ednrb1	1.55E-110	0.348560893	0.682	0.198	3.09E-107	EC	1	Ednrb
Podxl	1.23E-109	1.302199242	0.686	0.108	2.45E-106	EC	1	Podxl
Igfbp77	6.67E-107	0.955892187	0.996	0.8	1.33E-103	EC	1	Igfbp7
Meox11	1.02E-106	0.449925217	0.626	0.125	2.04E-103	EC	1	Meox1
Itga6	5.09E-103	1.12283633	0.684	0.182	1.02E-99	EC	1	Itga6
Rbp7	7.24E-100	1.824184668	0.63	0.041	1.45E-96	EC	1	Rbp7
Mall	2.32E-99	0.524156571	0.571	0.083	4.65E-96	EC	1	Mall
Tspan6	2.73E-96	0.496515007	0.705	0.297	5.46E-93	EC	1	Tspan6
Mcf2l	5.25E-96	1.124460461	0.651	0.146	1.05E-92	EC	1	Mcf2l
H2-Q6	1.07E-95	0.45764361	0.701	0.271	2.15E-92	EC	1	H2-Q6
Cxcl9	2.21E-95	0.436713522	0.561	0.138	4.42E-92	EC	1	Cxcl9
Kitl	1.34E-93	1.002573478	0.688	0.206	2.67E-90	EC	1	Kitl
Gimap1	1.05E-92	0.545414938	0.789	0.371	2.09E-89	EC	1	Gimap1
H2-D1	2.03E-91	0.737956496	0.985	0.889	4.07E-88	EC	1	H2-D1
Pitpnc1	6.04E-90	0.868646712	0.701	0.234	1.21E-86	EC	1	Pitpnc1
Sema3g	4.62E-89	0.40701926	0.462	0.017	9.23E-86	EC	1	Sema3g
Irf7	1.39E-88	0.431127374	0.669	0.262	2.78E-85	EC	1	Irf7
Plpp13	5.26E-86	0.928057412	0.801	0.389	1.05E-82	EC	1	Plpp1
Thrsp	2.84E-85	1.30012317	0.672	0.198	5.68E-82	EC	1	Thrsp
Klf2	2.88E-85	0.957852932	0.883	0.559	5.76E-82	EC	1	Klf2
Oasl2	4.49E-85	0.25217617	0.676	0.244	8.98E-82	EC	1	Oasl2
Nfkbia	1.48E-84	0.830387219	0.837	0.467	2.95E-81	EC	1	Nfkbia
Aqp7	1.19E-82	1.212951105	0.6	0.052	2.37E-79	EC	1	Aqp7
Cldn15	2.84E-82	0.37490739	0.559	0.121	5.69E-79	EC	1	Cldn15
Gngt2	5.23E-81	1.090327534	0.728	0.304	1.05E-77	EC	1	Gngt2
8430408G22Rik	1.53E-79	1.120691838	0.594	0.054	3.06E-76	EC	1	8430408G22Rik
Sorbs24	8.12E-78	0.299301715	0.632	0.184	1.62E-74	EC	1	Sorbs2
Rtp4	2.42E-77	0.622737173	0.659	0.231	4.84E-74	EC	1	Rtp4
H2-K1	2.68E-77	0.705106356	0.975	0.799	5.36E-74	EC	1	H2-K1
Clic5	2.74E-77	0.838861862	0.588	0.109	5.47E-74	EC	1	Clic5
Slc3a2	4.77E-75	0.749688907	0.814	0.432	9.55E-72	EC	1	Slc3a2
Icam2	7.62E-74	0.989680979	0.661	0.187	1.52E-70	EC	1	Icam2
Car4	1.41E-73	1.361645054	0.586	0.055	2.81E-70	EC	1	Car4
Nes4	2.27E-73	0.527888791	0.653	0.214	4.54E-70	EC	1	Nes
Cdkn1c3	1.64E-72	0.658485492	0.803	0.395	3.28E-69	EC	1	Cdkn1c
Csf2rb	6.51E-71	0.366321171	0.59	0.174	1.30E-67	EC	1	Csf2rb
Hes12	3.87E-69	0.740894667	0.724	0.302	7.73E-66	EC	1	Hes1
Tmsb4x4	1.10E-68	0.387379311	1	1	2.20E-65	EC	1	Tmsb4x
Ablim3	1.09E-67	0.982399732	0.594	0.117	2.18E-64	EC	1	Ablim3
Actn45	1.14E-66	0.592554043	0.864	0.486	2.27E-63	EC	1	Actn4
Tmem2042	2.95E-66	0.892010337	0.659	0.234	5.89E-63	EC	1	Tmem204
Cfd	4.61E-66	0.375798135	0.64	0.337	9.23E-63	EC	1	Cfd
Cd344	6.72E-65	0.580694858	0.923	0.5	1.34E-61	EC	1	Cd34
Mndal	3.35E-62	0.699565985	0.724	0.345	6.70E-59	EC	1	Mndal
Cyp4b11	6.10E-61	0.810123927	0.554	0.101	1.22E-57	EC	1	Cyp4b1
Tsc22d14	1.11E-60	0.745690782	0.847	0.542	2.21E-57	EC	1	Tsc22d1
Ier3	1.66E-60	0.488734397	0.663	0.315	3.32E-57	EC	1	Ier3
Igtp	5.74E-59	0.266189608	0.63	0.271	1.15E-55	EC	1	Igtp
Tgm24	3.14E-58	0.684663234	0.709	0.307	6.28E-55	EC	1	Tgm2
Btg25	1.04E-57	0.739385679	0.833	0.494	2.08E-54	EC	1	Btg2
Pltp1	9.12E-57	0.284216412	0.808	0.4	1.82E-53	EC	1	Pltp
Ifi47	1.25E-56	0.280409772	0.615	0.265	2.50E-53	EC	1	Ifi47
Fscn1	2.24E-56	0.731904316	0.615	0.237	4.49E-53	EC	1	Fscn1
Ehd41	3.07E-56	0.716723941	0.753	0.381	6.14E-53	EC	1	Ehd4
Ctla2b	8.52E-56	0.376359151	0.561	0.158	1.70E-52	EC	1	Ctla2b
Lpl2	1.90E-55	0.697003965	0.768	0.468	3.81E-52	EC	1	Lpl
F11r	3.19E-55	0.87751162	0.613	0.209	6.38E-52	EC	1	F11r
Sdc3	6.97E-55	0.482619296	0.642	0.277	1.39E-51	EC	1	Sdc3
Ptprr	2.13E-54	0.407811321	0.456	0.027	4.26E-51	EC	1	Ptprr
Gimap4	2.03E-53	0.637400376	0.674	0.313	4.06E-50	EC	1	Gimap4
Nrp2	3.16E-52	0.586847014	0.598	0.222	6.32E-49	EC	1	Nrp2
Limch14	6.95E-52	0.286178764	0.736	0.336	1.39E-48	EC	1	Limch1
Pdgfb	8.01E-52	0.76264331	0.565	0.156	1.60E-48	EC	1	Pdgfb
Thbd2	6.63E-51	0.434142956	0.751	0.362	1.33E-47	EC	1	Thbd
Sox41	1.22E-45	0.411453305	0.659	0.307	2.43E-42	EC	1	Sox4
Clec1a	1.85E-40	0.797793481	0.51	0.097	3.71E-37	EC	1	Clec1a
Zfp362	1.85E-39	0.305893993	0.782	0.462	3.70E-36	EC	1	Zfp36
Cdc42ep35	1.36E-37	0.302004575	0.736	0.38	2.72E-34	EC	1	Cdc42ep3
Tmem176a1	4.47E-37	0.258128633	0.705	0.339	8.94E-34	EC	1	Tmem176a
Psmb8	1.90E-35	0.424616546	0.82	0.533	3.79E-32	EC	1	Psmb8
Adamts12	2.37E-35	0.283693958	0.598	0.251	4.74E-32	EC	1	Adamts1
Plpp33	3.40E-35	0.460013383	0.889	0.613	6.79E-32	EC	1	Plpp3
Fxyd5	4.84E-34	0.345822485	0.81	0.452	9.67E-31	EC	1	Fxyd5
Ccdc85a	1.63E-33	0.704908795	0.513	0.126	3.25E-30	EC	1	Ccdc85a
Tmem176b1	3.07E-32	0.272396107	0.803	0.431	6.14E-29	EC	1	Tmem176b
Gda	8.58E-32	0.399288828	0.617	0.263	1.72E-28	EC	1	Gda
Ppic2	1.01E-31	0.300133939	0.828	0.524	2.02E-28	EC	1	Ppic
Sgk1	1.75E-29	0.874202236	0.563	0.202	3.49E-26	EC	1	Sgk1
Cdkn1a	1.89E-27	0.421456461	0.588	0.259	3.79E-24	EC	1	Cdkn1a
3-Jun	8.67E-27	0.536628954	0.776	0.52	1.73E-23	EC	1	Jun
Isg15	5.75E-26	0.68815611	0.536	0.22	1.15E-22	EC	1	Isg15
Ier22	6.70E-26	0.271704161	0.839	0.554	1.34E-22	EC	1	Ier2
Cdh134	4.93E-24	0.620085085	0.594	0.294	9.87E-21	EC	1	Cdh13
Plcb44	1.06E-23	0.611972578	0.594	0.272	2.13E-20	EC	1	Plcb4
Papss21	1.02E-22	0.294880944	0.538	0.235	2.04E-19	EC	1	Papss2

Cmpk2	2.89E-22	0.348887772	0.45	0.137	5.78E-19	EC	1	Cmpk2
Psmb9	1.46E-20	0.360626364	0.657	0.43	2.92E-17	EC	1	Psmb9
Btnl9	1.75E-19	1.032872987	0.473	0.087	3.50E-16	EC	1	Btnl9
Cd59a3	9.71E-17	0.389056464	0.521	0.234	1.94E-13	EC	1	Cd59a
Palmd	2.68E-16	0.718773437	0.464	0.091	5.35E-13	EC	1	Palmd
Pde2a	5.04E-15	0.72174649	0.508	0.169	1.01E-11	EC	1	Pde2a
Myo10	2.50E-14	0.841337329	0.494	0.191	5.00E-11	EC	1	Myo10
Trp53i11	5.81E-14	0.716013006	0.504	0.235	1.16E-10	EC	1	Trp53i11
Dusp15	1.01E-13	0.292804555	0.69	0.494	2.02E-10	EC	1	Dusp1
Ifit3	1.80E-13	0.3746294	0.494	0.251	3.59E-10	EC	1	Ifit3
Ndrg1	6.69E-13	0.482716169	0.542	0.287	1.34E-09	EC	1	Ndrg1
Gbp2	1.61E-12	0.44175233	0.542	0.317	3.23E-09	EC	1	Gbp2
Ifi203	4.11E-12	0.451131049	0.6	0.384	8.23E-09	EC	1	Ifi203
Tek1	8.09E-11	0.583424867	0.536	0.309	1.62E-07	EC	1	Tek
Gm42418	8.27E-11	0.331811838	0.872	0.824	1.65E-07	EC	1	Gm42418
Gja11	4.69E-10	0.394668275	0.339	0.296	9.39E-07	EC	1	Gja1
Ifit1	3.88E-09	0.415314076	0.293	0.219	7.77E-06	EC	1	Ifit1
Ddit4	2.14E-08	0.583636146	0.481	0.215	4.28E-05	EC	1	Ddit4
Myo1b1	3.34E-08	0.533282911	0.314	0.125	6.69E-05	EC	1	Myo1b
Rgs54	1.27E-07	0.739321014	0.462	0.221	0.000253763	EC	1	Rgs5
Car81	1.57E-06	0.44987416	0.458	0.242	0.003142789	EC	1	Car8
Abcb1b	2.26E-06	0.403178366	0.421	0.19	0.004524294	EC	1	Abcb1b
Calcrl	8.33E-06	0.427816706	0.454	0.266	0.01666325	EC	1	Calcrl
Slfn51	0.000525975	0.511509038	0.433	0.193	1	EC	1	Slfn5
Klf41	0.001054803	0.306729789	0.573	0.437	1	EC	1	Klf4
Galnt18	0.001391689	0.437563357	0.362	0.25	1	EC	1	Galnt18
Cyp26b1	0.00144021	0.547138991	0.333	0.206	1	EC	1	Cyp26b1
Pkp44	0.004553696	0.359634485	0.494	0.315	1	EC	1	Pkp4
Stmn2	0.005183251	0.62308586	0.356	0.073	1	EC	1	Stmn2
Cbfa2t31	0.007258844	0.360508699	0.45	0.248	1	EC	1	Cbfa2t3
Selp	0	0.732819524	0.665	0.031	0	EC	2	Selp
Serpina1b	0	0.250430083	0.553	0.018	0	EC	2	Serpina1b
Gja5	3.87E-259	1.193626442	0.758	0.043	7.74E-256	EC	2	Gja5
Bmx	4.98E-230	1.577884974	0.847	0.052	9.95E-227	EC	2	Bmx
Gkn3	3.24E-207	2.457449149	0.823	0.113	6.48E-204	EC	2	Gkn3
Sox171	5.98E-202	0.851570416	0.726	0.035	1.20E-198	EC	2	Sox17
Nos31	2.43E-181	1.133540136	0.763	0.089	4.86E-178	EC	2	Nos3
Mmrn21	1.90E-178	1.817396735	0.935	0.118	3.80E-175	EC	2	Mmrn2
AU021092	2.25E-165	1.077723015	0.758	0.028	4.50E-162	EC	2	AU021092
Clec1a1	1.93E-163	0.819898464	0.749	0.099	3.86E-160	EC	2	Clec1a
Klk10	1.38E-162	0.903156232	0.586	0.02	2.76E-159	EC	2	Klk10
Myct11	2.21E-157	1.280142521	0.856	0.11	4.42E-154	EC	2	Myct1
Pecam11	6.07E-156	2.72567019	0.991	0.283	1.21E-152	EC	2	Pecam1
Dhh	3.89E-152	0.545906138	0.563	0.017	7.79E-149	EC	2	Dhh
Egfl71	6.61E-147	1.948559115	0.963	0.151	1.32E-143	EC	2	Egfl7
Ptprb1	4.43E-146	2.244046551	0.935	0.172	8.86E-143	EC	2	Ptprb
Slco2a1	2.11E-136	1.233225048	0.837	0.188	4.23E-133	EC	2	Slco2a1
Cytl1	2.85E-130	2.91658372	0.972	0.417	5.70E-127	EC	2	Cytl1
Ptprr1	3.41E-129	0.620857806	0.591	0.031	6.82E-126	EC	2	Ptprr
Vwf1	7.41E-128	2.199047625	0.874	0.17	1.48E-124	EC	2	Vwf
Icam21	3.71E-123	1.884240083	0.87	0.19	7.41E-120	EC	2	Icam2
Epas12	5.19E-122	1.633186883	0.935	0.239	1.04E-118	EC	2	Epas1
Tm4sf15	1.60E-121	2.298483875	0.986	0.515	3.21E-118	EC	2	Tm4sf1
Ramp23	1.48E-116	1.872529301	0.981	0.392	2.95E-113	EC	2	Ramp2
Pla2g5	2.50E-116	0.31807431	0.614	0.118	5.00E-113	EC	2	Pla2g5
Cd932	9.57E-114	1.639547937	0.865	0.199	1.91E-110	EC	2	Cd93
Klk8	4.15E-113	1.089484938	0.753	0.157	8.29E-110	EC	2	Klk8
Gja42	6.93E-107	1.182560641	0.758	0.151	1.39E-103	EC	2	Gja4
Ecscr2	2.03E-106	1.44619919	0.865	0.245	4.05E-103	EC	2	Ecscr
Clu4	6.26E-105	2.39618075	0.949	0.378	1.25E-101	EC	2	Clu
Car82	6.94E-104	1.34544209	0.823	0.241	1.39E-100	EC	2	Car8
Bmp42	5.84E-102	1.908927741	0.842	0.254	1.17E-98	EC	2	Bmp4
Cav16	5.71E-100	1.37302106	0.972	0.413	1.14E-96	EC	2	Cav1
Prr15	1.68E-96	0.441607005	0.651	0.148	3.36E-93	EC	2	Prr15
Sdpr2	2.07E-94	1.428284916	0.893	0.309	4.15E-91	EC	2	Sdpr
Lmo2	3.39E-94	1.770162973	0.912	0.421	6.78E-91	EC	2	Lmo2
Ephx2	8.96E-92	1.1954662	0.74	0.213	1.79E-88	EC	2	Ephx2
Slc9a3r21	1.48E-91	1.376496861	0.837	0.264	2.96E-88	EC	2	Slc9a3r2
Vcam13	3.08E-91	1.831396043	0.851	0.272	6.16E-88	EC	2	Vcam1
Sfrp12	8.69E-87	1.695396452	0.86	0.277	1.74E-83	EC	2	Sfrp1
Arhgap311	1.12E-86	1.239254357	0.87	0.315	2.23E-83	EC	2	Arhgap31
Tek2	1.31E-85	1.294458789	0.842	0.309	2.62E-82	EC	2	Tek
Ece11	4.10E-84	1.462962183	0.856	0.305	8.20E-81	EC	2	Ece1
Cpe5	4.44E-84	1.336905656	0.981	0.575	8.88E-81	EC	2	Cpe
Spon11	4.04E-83	1.131976897	0.791	0.211	8.08E-80	EC	2	Spon1
Stab11	1.97E-82	0.966024048	0.856	0.221	3.95E-79	EC	2	Stab1
Kcnn4	7.10E-82	0.450698353	0.679	0.162	1.42E-78	EC	2	Kcnn4
Tgm25	2.84E-81	1.224185974	0.879	0.31	5.67E-78	EC	2	Tgm2
Scx	6.39E-79	0.2754928	0.619	0.119	1.28E-75	EC	2	Scx
Plvap1	2.01E-77	1.601140072	0.791	0.301	4.01E-74	EC	2	Plvap
H2-Q61	2.15E-76	0.875973866	0.763	0.275	4.29E-73	EC	2	H2-Q6
Adh71	8.77E-76	0.982783482	0.702	0.211	1.75E-72	EC	2	Adh7
Cd24a	1.04E-75	1.348692728	0.814	0.331	2.09E-72	EC	2	Cd24a
Cgnl11	5.34E-75	1.386548908	0.8	0.242	1.07E-71	EC	2	Cgnl1
Gxylt21	2.36E-74	1.328402627	0.865	0.316	4.72E-71	EC	2	Gxylt2
Gata3	3.20E-73	0.29355992	0.633	0.163	6.40E-70	EC	2	Gata3
Clic51	6.87E-72	0.58945662	0.614	0.114	1.37E-68	EC	2	Clic5
Mall1	3.02E-71	0.831121192	0.642	0.088	6.04E-68	EC	2	Mall
Ehd42	2.66E-70	1.184007049	0.86	0.384	5.32E-67	EC	2	Ehd4
Adh13	2.60E-69	0.963530341	0.786	0.279	5.20E-66	EC	2	Adh1
Cfh2	4.94E-67	1.234443685	0.94	0.6	9.88E-64	EC	2	Cfh
Tinagl16	9.25E-67	1.321370767	0.8	0.256	1.85E-63	EC	2	Tinagl1
Clec14a1	2.46E-66	1.114874672	0.74	0.236	4.92E-63	EC	2	Clec14a
Procr1	8.49E-66	1.127595583	0.791	0.366	1.70E-62	EC	2	Procr
Cdh51	2.91E-65	1.220146486	0.735	0.144	5.82E-62	EC	2	Cdh5
1500015O10Rik	1.09E-64	1.161896015	0.712	0.269	2.17E-61	EC	2	1500015O10Rik
Tmem158	1.92E-62	1.174446444	0.702	0.212	3.84E-59	EC	2	Tmem158
H2-K11	1.95E-60	1.045632064	0.981	0.801	3.90E-57	EC	2	H2-K1
Cbfa2t32	1.58E-59	0.972902392	0.758	0.247	3.15E-56	EC	2	Cbfa2t3
Sncg5	3.12E-59	0.616732691	0.702	0.158	6.24E-56	EC	2	Sncg
Itgb4	1.19E-58	0.638496712	0.591	0.1	2.39E-55	EC	2	Itgb4
Gng113	6.77E-58	0.823414279	0.898	0.445	1.35E-54	EC	2	Gng11
Tnfaip2	1.49E-57	0.605957421	0.684	0.224	2.97E-54	EC	2	Tnfaip2
Ndrg11	8.37E-57	0.6709637	0.8	0.287	1.67E-53	EC	2	Ndrg1
Parm12	6.41E-56	0.266634505	0.647	0.21	1.28E-52	EC	2	Parm1
Aqp12	8.63E-56	1.13785547	0.809	0.356	1.73E-52	EC	2	Aqp1
Ass1	1.53E-55	0.608722159	0.693	0.257	3.06E-52	EC	2	Ass1
Esam2	1.15E-54	1.181324202	0.712	0.197	2.29E-51	EC	2	Esam
H2-D11	1.65E-54	0.935542521	0.986	0.89	3.30E-51	EC	2	H2-D1
Pam4	4.28E-54	1.376365808	0.94	0.645	8.56E-51	EC	2	Pam
Mical24	4.11E-53	0.594029237	0.767	0.252	8.21E-50	EC	2	Mical2
H2-Q71	4.21E-52	0.707893253	0.791	0.377	8.42E-49	EC	2	H2-Q7
Cdc42ep36	1.13E-51	0.933387283	0.837	0.383	2.26E-48	EC	2	Cdc42ep3
Perp3	1.31E-51	0.441896696	0.656	0.187	2.62E-48	EC	2	Perp
Cldn51	2.40E-50	1.496554591	0.642	0.127	4.80E-47	EC	2	Cldn5
Rassf9	1.08E-49	0.431615024	0.567	0.149	2.15E-46	EC	2	Rassf9
Klf21	1.36E-48	1.078113645	0.898	0.562	2.72E-45	EC	2	Klf2
Srgn1	2.02E-47	1.000830214	0.851	0.496	4.05E-44	EC	2	Srgn
Eng1	2.05E-47	1.249161811	0.767	0.333	4.10E-44	EC	2	Eng
Aldh3a11	8.38E-47	0.305447477	0.6	0.224	1.68E-43	EC	2	Aldh3a1
Lmcd15	2.33E-46	0.547872826	0.805	0.313	4.66E-43	EC	2	Lmcd1
Atp1b1	9.00E-45	0.497329582	0.721	0.343	1.80E-41	EC	2	Atp1b1
Rbp14	2.82E-44	0.895313766	0.926	0.635	5.64E-41	EC	2	Rbp1
Apoe1	1.49E-43	0.688840089	0.953	0.843	2.98E-40	EC	2	Apoe
Smoc15	4.13E-43	0.730399945	0.73	0.309	8.26E-40	EC	2	Smoc1
Anxa31	1.93E-42	0.837753277	0.847	0.477	3.87E-39	EC	2	Anxa3
Ncam14	4.24E-42	0.315075017	0.753	0.295	8.48E-39	EC	2	Ncam1
Iigp11	9.23E-42	0.513870996	0.674	0.299	1.85E-38	EC	2	Iigp1
Id34	4.16E-40	0.660117609	0.935	0.688	8.33E-37	EC	2	Id3

Podxl1	7.03E-39	1.040690398	0.609	0.116	1.41E-35	EC	2	Podxl
Thbs12	4.14E-38	0.979754337	0.591	0.203	8.27E-35	EC	2	Thbs1
Cd2006	3.49E-37	0.62203049	0.926	0.536	6.98E-34	EC	2	Cd200
Cthrc1	3.89E-37	0.324064401	0.563	0.197	7.78E-34	EC	2	Cthrc1
Cp3	1.27E-36	0.699046275	0.707	0.294	2.54E-33	EC	2	Cp
H19	4.53E-35	0.394625232	0.079	0.264	9.07E-32	EC	2	H19
Gimap41	6.67E-35	0.329560515	0.679	0.317	1.33E-31	EC	2	Gimap4
Kctd121	8.93E-35	0.445188311	0.73	0.358	1.79E-31	EC	2	Kctd12
Fxyd51	1.00E-34	0.858242343	0.814	0.456	2.01E-31	EC	2	Fxyd5
Eln6	1.14E-34	0.655772615	0.93	0.598	2.29E-31	EC	2	Eln
Stmn21	2.28E-34	1.146658482	0.535	0.074	4.57E-31	EC	2	Stmn2
Isg151	4.91E-34	0.284546508	0.591	0.223	9.82E-31	EC	2	Isg15
Fxyd62	6.26E-34	0.515094698	0.712	0.315	1.25E-30	EC	2	Fxyd6
Ptgs11	4.68E-33	0.954987825	0.674	0.297	9.36E-30	EC	2	Ptgs1
Bace21	2.30E-32	0.564346607	0.665	0.325	4.60E-29	EC	2	Bace2
Pde3a5	2.80E-32	0.783067053	0.702	0.28	5.60E-29	EC	2	Pde3a
Cd96	1.03E-31	0.764913389	0.888	0.648	2.06E-28	EC	2	Cd9
Cyyr11	2.83E-29	0.531550384	0.488	0.057	5.66E-26	EC	2	Cyyr1
G0s23	4.21E-29	0.354595358	0.66	0.315	8.41E-26	EC	2	G0s2
Cdh135	4.92E-29	1.024051706	0.688	0.296	9.84E-26	EC	2	Cdh13
Emcn1	6.03E-29	1.267848065	0.553	0.067	1.21E-25	EC	2	Emcn
Actn46	3.23E-28	0.544236549	0.828	0.49	6.46E-25	EC	2	Actn4
Crim14	8.18E-28	0.916170535	0.66	0.284	1.64E-24	EC	2	Crim1
Pdlim35	4.59E-27	0.372399511	0.66	0.287	9.17E-24	EC	2	Pdlim3
Ctla2a1	1.23E-26	1.345328084	0.609	0.19	2.45E-23	EC	2	Ctla2a
Tspan71	4.71E-25	1.034073671	0.628	0.305	9.43E-22	EC	2	Tspan7
Dbp1	1.57E-24	0.404441032	0.874	0.591	3.14E-21	EC	2	Dbp
Itgb15	1.68E-24	0.507843824	0.842	0.564	3.35E-21	EC	2	Itgb1
Klf42	1.68E-23	0.528450433	0.786	0.437	3.36E-20	EC	2	Klf4
Lox3	2.26E-23	0.39899952	0.665	0.351	4.53E-20	EC	2	Lox
Tie11	2.90E-23	0.86806599	0.558	0.114	5.80E-20	EC	2	Tie1
Ier31	4.24E-23	0.292226268	0.651	0.319	8.48E-20	EC	2	Ier3
Fabp42	6.95E-23	0.424844756	0.693	0.438	1.39E-19	EC	2	Fabp4
Bcam6	2.69E-22	0.626138741	0.684	0.328	5.38E-19	EC	2	Bcam
Bst21	3.57E-22	0.508999736	0.772	0.494	7.14E-19	EC	2	Bst2
Fbln55	4.97E-22	0.783022987	0.828	0.553	9.93E-19	EC	2	Fbln5
B2m1	1.09E-21	0.420169303	0.991	0.983	2.18E-18	EC	2	B2m
Serpinb6b	2.04E-21	0.796616229	0.54	0.167	4.09E-18	EC	2	Serpinb6b
Ly6e1	3.39E-21	0.410246833	0.972	0.794	6.77E-18	EC	2	Ly6e
Bgn6	9.90E-21	0.42459304	0.963	0.767	1.98E-17	EC	2	Bgn
Mfge85	1.34E-20	0.400502603	0.828	0.524	2.68E-17	EC	2	Mfge8
Spint25	2.88E-19	0.296401581	0.633	0.291	5.76E-16	EC	2	Spint2
Ifit31	7.65E-19	0.288504524	0.563	0.253	1.53E-15	EC	2	Ifit3
Adamts13	1.01E-18	0.377133264	0.558	0.255	2.02E-15	EC	2	Adamts1
Adgrf51	1.26E-18	0.765141761	0.53	0.134	2.51E-15	EC	2	Adgrf5
Nfkbia1	1.43E-18	0.467603753	0.749	0.472	2.86E-15	EC	2	Nfkbia
S1pr11	4.40E-18	0.672957337	0.609	0.294	8.81E-15	EC	2	S1pr1
Jam21	1.14E-17	0.772692992	0.572	0.223	2.29E-14	EC	2	Jam2
Htra15	1.66E-17	0.357259202	0.823	0.544	3.31E-14	EC	2	Htra1
Hspb15	2.35E-17	0.406445959	0.726	0.488	4.70E-14	EC	2	Hspb1
Id11	4.85E-16	0.823485019	0.595	0.291	9.70E-13	EC	2	Id1
Vim4	5.19E-16	0.297935631	0.991	0.892	1.04E-12	EC	2	Vim
Cst32	8.00E-16	0.253659424	0.995	0.957	1.60E-12	EC	2	Cst3
Tmem2	8.78E-16	0.778056663	0.549	0.236	1.76E-12	EC	2	Tmem2
Thbd3	5.57E-15	0.476942769	0.642	0.367	1.11E-11	EC	2	Thbd
Sdc11	1.42E-14	0.409826773	0.544	0.307	2.83E-11	EC	2	Sdc1
Ahsg	4.39E-14	0.701279281	0.419	0.061	8.78E-11	EC	2	Ahsg
Psmb81	4.61E-14	0.263879908	0.8	0.537	9.22E-11	EC	2	Psmb8
Calcrl1	6.40E-14	0.981263326	0.558	0.267	1.28E-10	EC	2	Calcrl
Cyb5r35	6.89E-14	0.395440934	0.744	0.497	1.38E-10	EC	2	Cyb5r3
Palmd1	8.20E-13	0.807970492	0.47	0.095	1.64E-09	EC	2	Palmd
Fbln24	1.53E-12	0.374669782	0.693	0.379	3.06E-09	EC	2	Fbln2
Rasip11	7.51E-12	0.664831672	0.465	0.072	1.50E-08	EC	2	Rasip1
Timp34	8.61E-12	0.297846738	0.921	0.698	1.72E-08	EC	2	Timp3
Fabp51	1.55E-11	0.661978987	0.484	0.224	3.09E-08	EC	2	Fabp5
Slc3a21	1.89E-11	0.565080675	0.623	0.438	3.78E-08	EC	2	Slc3a2
F11r1	2.29E-11	0.716653772	0.516	0.215	4.58E-08	EC	2	F11r
Tspan131	3.44E-11	0.506211892	0.558	0.28	6.87E-08	EC	2	Tspan13
Flt11	4.20E-11	0.475450126	0.47	0.141	8.41E-08	EC	2	Flt1
Net13	4.34E-11	0.478276067	0.516	0.238	8.68E-08	EC	2	Net1
Tmem176b2	6.85E-11	0.310098454	0.707	0.436	1.37E-07	EC	2	Tmem176b
Gas64	8.37E-11	0.270408348	0.8	0.566	1.67E-07	EC	2	Gas6
Ccrl2	8.79E-11	0.293825442	0.507	0.265	1.76E-07	EC	2	Ccrl2
Kank31	1.04E-10	0.400775792	0.474	0.227	2.09E-07	EC	2	Kank3
Acer2	7.36E-10	0.777473761	0.456	0.103	1.47E-06	EC	2	Acer2
Ly6c13	1.63E-09	0.566622097	0.674	0.419	3.26E-06	EC	2	Ly6c1
Tspan61	5.21E-08	0.767137921	0.516	0.303	0.000104191	EC	2	Tspan6
Sorbs25	1.41E-07	0.39126539	0.456	0.191	0.000282761	EC	2	Sorbs2
Mal	5.77E-06	0.435893759	0.27	0.082	0.011540209	EC	2	Mal
Cyp26b11	5.94E-06	0.467001856	0.251	0.208	0.011874428	EC	2	Cyp26b1
Dst4	8.41E-06	0.308527397	0.623	0.427	0.016820825	EC	2	Dst
Cd59a4	1.25E-05	0.541315019	0.46	0.238	0.02502056	EC	2	Cd59a
Plpp14	2.16E-05	0.580540058	0.535	0.396	0.043105147	EC	2	Plpp1
Tuba1b3	2.23E-05	0.263286604	0.772	0.662	0.044587305	EC	2	Tuba1b
Pkp45	5.44E-05	0.332426864	0.493	0.317	0.108768853	EC	2	Pkp4
F2r2	0.000178088	0.342814693	0.47	0.276	0.356175092	EC	2	F2r
Fgfr3	0.0006055	0.348204899	0.284	0.198	1	EC	2	Fgfr3
Ttc9	0.001334239	0.31394172	0.34	0.07	1	EC	2	Ttc9
Pthlh	0.00344897	0.521856489	0.321	0.249	1	EC	2	Pthlh
Sgk11	0.00395033	0.469557903	0.414	0.208	1	EC	2	Sgk1
Lsr	0.009909505	0.547618857	0.377	0.126	1	EC	2	Lsr
Klhl4	0	0.998090757	0.762	0.024	0	EC	3	Klhl4
Sh3gl3	0	0.930984703	0.72	0.029	0	EC	3	Sh3gl3
Duoxa2	0	0.612613817	0.604	0.013	0	EC	3	Duoxa2
Adgrg3	7.18E-228	1.013612867	0.701	0.026	1.44E-224	EC	3	Adgrg3
Pkhd1l1	1.12E-188	0.56480353	0.677	0.036	2.24E-185	EC	3	Pkhd1l1
Reln	7.25E-178	1.97413509	0.805	0.097	1.45E-174	EC	3	Reln
Hoxd9	2.97E-177	1.015988565	0.768	0.043	5.94E-174	EC	3	Hoxd9
Flt41	8.08E-172	1.718075777	0.872	0.108	1.62E-168	EC	3	Flt4
Mmrn1	5.17E-151	2.952666691	0.963	0.152	1.03E-147	EC	3	Mmrn1
Grrp11	6.36E-138	0.669677777	0.683	0.052	1.27E-134	EC	3	Grrp1
Cldn52	2.21E-133	3.506815976	0.994	0.125	4.42E-130	EC	3	Cldn5
Ecscr3	2.76E-123	2.126032664	0.982	0.246	5.53E-120	EC	3	Ecscr
Tbx1	6.52E-123	1.027256749	0.799	0.131	1.30E-119	EC	3	Tbx1
Pard6g	9.02E-123	2.141019692	0.939	0.26	1.80E-119	EC	3	Pard6g
Mall2	1.15E-116	1.051499231	0.787	0.088	2.31E-113	EC	3	Mall
Egfl72	6.99E-112	1.706965105	0.957	0.153	1.40E-108	EC	3	Egfl7
Sdpr3	5.25E-111	2.15557029	0.988	0.309	1.05E-107	EC	3	Sdpr
Dtx1	1.93E-110	1.428077691	0.854	0.201	3.86E-107	EC	3	Dtx1
Lbp2	1.31E-107	1.957314704	0.933	0.304	2.63E-104	EC	3	Lbp
Gng114	1.65E-105	2.180674823	0.988	0.445	3.29E-102	EC	3	Gng11
F11r2	3.94E-101	1.296152207	0.89	0.213	7.88E-98	EC	3	F11r
Rasip12	9.66E-100	0.971164875	0.756	0.071	1.93E-96	EC	3	Rasip1
Fxyd63	3.43E-99	2.027699744	0.957	0.314	6.85E-96	EC	3	Fxyd6
Cyp4b12	1.63E-96	1.178573597	0.762	0.106	3.27E-93	EC	3	Cyp4b1
Cldn11	1.26E-94	0.726672075	0.652	0.136	2.52E-91	EC	3	Cldn11
Gngt21	2.32E-93	1.609160048	0.939	0.308	4.65E-90	EC	3	Gngt2
Ccl21a	1.11E-91	5.400941763	0.854	0.187	2.22E-88	EC	3	Ccl21a
Stmn22	4.34E-87	1.321387306	0.701	0.074	8.69E-84	EC	3	Stmn2
Ramp24	7.98E-87	1.62583905	0.976	0.394	1.60E-83	EC	3	Ramp2
Nts1	8.63E-85	1.906316903	0.726	0.113	1.73E-81	EC	3	Nts
Cp4	1.19E-83	1.661152885	0.915	0.294	2.37E-80	EC	3	Cp
Cd97	2.71E-83	1.63636889	1	0.648	5.41E-80	EC	3	Cd9
Ctla2a2	5.64E-83	1.476374031	0.835	0.19	1.13E-79	EC	3	Ctla2a
Ndrg12	2.66E-80	1.538413411	0.896	0.288	5.33E-77	EC	3	Ndrg1
Lcn2	5.09E-80	1.756785292	0.787	0.061	1.02E-76	EC	3	Lcn2
Sbspon	1.16E-76	0.259856794	0.591	0.107	2.32E-73	EC	3	Sbspon
Lrg11	2.04E-74	2.19352812	0.811	0.077	4.08E-71	EC	3	Lrg1
Fgl25	5.91E-73	1.801021382	0.884	0.403	1.18E-69	EC	3	Fgl2

Lyve11	5.40E-71	2.523811811	0.805	0.234	1.08E-67	EC	3	Lyve1
8430408G22Rik	8.87E-71	0.506150245	0.64	0.061	1.77E-67	EC	3	8430408G22Rik
Pecam12	5.37E-70	1.209045538	0.896	0.285	1.07E-66	EC	3	Pecam1
Nr2f22	6.21E-68	1.253655499	0.896	0.352	1.24E-64	EC	3	Nr2f2
Nrp21	6.74E-68	1.131104595	0.793	0.226	1.35E-64	EC	3	Nrp2
Slc9a3r22	8.57E-68	1.128754505	0.829	0.265	1.71E-64	EC	3	Slc9a3r2
Gpm6a	1.18E-66	1.132593183	0.768	0.152	2.36E-63	EC	3	Gpm6a
Thy11	1.20E-64	1.346218648	0.884	0.431	2.40E-61	EC	3	Thy1
Ece12	5.61E-62	1.368314504	0.835	0.307	1.12E-58	EC	3	Ece1
Kank32	7.70E-62	1.233898492	0.799	0.225	1.54E-58	EC	3	Kank3
Ptn1	6.69E-60	1.321623198	0.787	0.295	1.34E-56	EC	3	Ptn
Ctla2b1	4.06E-58	0.490106946	0.659	0.162	8.12E-55	EC	3	Ctla2b
AA467197	8.45E-57	0.531937356	0.598	0.168	1.69E-53	EC	3	AA467197
Gimap42	4.59E-56	0.79020601	0.829	0.316	9.18E-53	EC	3	Gimap4
Tspan132	1.68E-55	0.858216743	0.793	0.279	3.36E-52	EC	3	Tspan13
Kdr1	8.11E-55	1.260687376	0.762	0.165	1.62E-51	EC	3	Kdr
Maf1	1.27E-54	1.092463932	0.921	0.472	2.54E-51	EC	3	Maf
Cyb5r36	1.01E-53	1.24665681	0.909	0.497	2.01E-50	EC	3	Cyb5r3
Plpp15	1.53E-52	1.191548232	0.86	0.394	3.06E-49	EC	3	Plpp1
Prox1	8.90E-52	1.307510196	0.634	0.092	1.78E-48	EC	3	Prox1
Gap431	2.99E-50	0.461095908	0.622	0.221	5.99E-47	EC	3	Gap43
Timp35	6.26E-50	0.89554605	0.988	0.698	1.25E-46	EC	3	Timp3
Clca3a1	3.50E-49	1.282204494	0.598	0.024	7.00E-46	EC	3	Clca3a1
Duox2	8.23E-49	0.439619758	0.256	0.009	1.65E-45	EC	3	Duox2
Tgm26	1.04E-48	1.210334392	0.841	0.311	2.08E-45	EC	3	Tgm2
Fscn11	1.31E-48	0.4954744	0.665	0.241	2.63E-45	EC	3	Fscn1
Arhgef151	1.70E-47	0.250207575	0.549	0.083	3.40E-44	EC	3	Arhgef15
Pgf	3.42E-47	0.355929226	0.591	0.146	6.83E-44	EC	3	Pgf
Icam22	4.74E-47	0.906714578	0.72	0.193	9.49E-44	EC	3	Icam2
Ank3	2.30E-46	0.445719205	0.591	0.195	4.60E-43	EC	3	Ank3
Emcn2	2.57E-46	1.379615442	0.683	0.067	5.14E-43	EC	3	Emcn
Sepp11	2.97E-46	0.76819532	0.988	0.822	5.93E-43	EC	3	Sepp1
Cav17	1.93E-45	0.849539692	0.896	0.414	3.87E-42	EC	3	Cav1
Igfbp52	3.69E-44	1.205780647	0.927	0.535	7.37E-41	EC	3	Igfbp5
Col23a11	7.79E-44	0.352025464	0.646	0.248	1.56E-40	EC	3	Col23a1
Tnc1	9.49E-44	0.468719567	0.598	0.166	1.90E-40	EC	3	Tnc
Stc12	1.99E-43	0.472915889	0.604	0.219	3.98E-40	EC	3	Stc1
Trp53i111	1.99E-43	0.467822773	0.701	0.237	3.99E-40	EC	3	Trp53i11
Ptprf1	1.71E-42	0.455138202	0.634	0.221	3.41E-39	EC	3	Ptprf
Rasgrp3	1.50E-41	0.318272588	0.652	0.249	3.01E-38	EC	3	Rasgrp3
Adrb2	1.89E-41	0.36325625	0.646	0.247	3.78E-38	EC	3	Adrb2
Enho	3.77E-41	0.254182295	0.616	0.175	7.54E-38	EC	3	Enho
Wipf3	2.31E-40	0.919003971	0.579	0.058	4.61E-37	EC	3	Wipf3
Gimap61	3.29E-40	0.69738993	0.848	0.42	6.57E-37	EC	3	Gimap6
Igfbp34	3.97E-40	0.566671202	0.646	0.249	7.95E-37	EC	3	Igfbp3
Prdx12	1.77E-39	0.700799276	0.976	0.797	3.54E-36	EC	3	Prdx1
Ackr41	2.18E-39	0.823967113	0.665	0.235	4.35E-36	EC	3	Ackr4
Calcrl2	7.59E-39	0.980004461	0.695	0.267	1.52E-35	EC	3	Calcrl
Tie12	1.58E-38	1.126252691	0.64	0.114	3.17E-35	EC	3	Tie1
Gpihbp11	1.99E-38	0.997929139	0.671	0.087	3.98E-35	EC	3	Gpihbp1
Prss234	1.15E-37	0.964865065	0.872	0.49	2.30E-34	EC	3	Prss23
Cdh52	1.38E-37	1.024441104	0.695	0.145	2.76E-34	EC	3	Cdh5
Cd24a1	1.75E-37	0.431662936	0.75	0.333	3.50E-34	EC	3	Cd24a
Nmb	1.73E-36	0.324934048	0.604	0.255	3.46E-33	EC	3	Nmb
Kctd122	1.39E-35	0.632100772	0.768	0.359	2.78E-32	EC	3	Kctd12
Gimap11	3.81E-35	0.443188325	0.774	0.377	7.62E-32	EC	3	Gimap1
Sep-43	7.67E-35	0.297064563	0.634	0.236	1.53E-31	EC	3	4-Sep
Pthlh1	3.15E-31	0.282409589	0.567	0.247	6.29E-28	EC	3	Pthlh
Tmem2043	3.19E-31	0.320854821	0.646	0.239	6.39E-28	EC	3	Tmem204
Tm4sf16	4.76E-31	0.988670729	0.896	0.517	9.51E-28	EC	3	Tm4sf1
Col18a14	9.63E-31	0.510868306	0.787	0.347	1.93E-27	EC	3	Col18a1
Stab12	2.01E-30	1.037286852	0.72	0.224	4.02E-27	EC	3	Stab1
Itga94	2.66E-30	0.453529796	0.774	0.345	5.32E-27	EC	3	Itga9
Anxa22	4.57E-30	0.639781801	0.927	0.666	9.15E-27	EC	3	Anxa2
Pcp4l15	3.31E-29	0.678841909	0.683	0.255	6.61E-26	EC	3	Pcp4l1
Lgmn1	2.46E-28	0.609097538	0.835	0.462	4.91E-25	EC	3	Lgmn
Tppp32	4.84E-28	1.346770918	0.841	0.576	9.67E-25	EC	3	Tppp3
Ddit41	1.22E-27	0.712359608	0.622	0.218	2.45E-24	EC	3	Ddit4
Sned12	2.36E-26	0.468946689	0.622	0.253	4.72E-23	EC	3	Sned1
Ucp2	3.02E-26	0.576456999	0.835	0.438	6.05E-23	EC	3	Ucp2
Mmrn22	1.39E-25	1.051824951	0.616	0.121	2.78E-22	EC	3	Mmrn2
Abi3bp4	1.39E-25	0.788435001	0.799	0.456	2.79E-22	EC	3	Abi3bp
Aqp13	7.49E-25	0.519516614	0.695	0.357	1.50E-21	EC	3	Aqp1
Cd2	9.97E-25	0.409877799	0.622	0.344	1.99E-21	EC	3	Cd2
Vim5	2.35E-24	0.501775396	0.982	0.892	4.71E-21	EC	3	Vim
Eng2	2.58E-24	0.770311092	0.713	0.334	5.16E-21	EC	3	Eng
Fbln72	3.21E-24	0.296811291	0.579	0.258	6.42E-21	EC	3	Fbln7
Galnt181	4.39E-23	1.113202129	0.61	0.25	8.78E-20	EC	3	Galnt18
Tnfaip21	5.14E-23	0.429867859	0.567	0.226	1.03E-19	EC	3	Tnfaip2
Procr2	1.48E-21	0.371054095	0.72	0.367	2.97E-18	EC	3	Procr
Ly6e2	7.46E-21	0.445515826	0.963	0.794	1.49E-17	EC	3	Ly6e
Gas65	7.77E-21	0.513217397	0.878	0.566	1.55E-17	EC	3	Gas6
S1pr12	8.65E-20	0.672488549	0.646	0.294	1.73E-16	EC	3	S1pr1
Edn1	1.20E-19	0.310304139	0.159	0.274	2.41E-16	EC	3	Edn1
H2-D12	2.84E-19	0.479129012	0.988	0.89	5.68E-16	EC	3	H2-D1
Apold11	2.36E-18	0.904179528	0.482	0.093	4.73E-15	EC	3	Apold1
Prelp4	2.47E-18	0.564796232	0.823	0.547	4.94E-15	EC	3	Prelp
Adgrf52	2.79E-18	0.512337331	0.549	0.134	5.59E-15	EC	3	Adgrf5
Serpine24	4.68E-18	0.364496012	0.787	0.42	9.36E-15	EC	3	Serpine2
Smagp1	7.60E-18	0.736046251	0.646	0.235	1.52E-14	EC	3	Smagp
Gmfg	1.01E-17	0.274838792	0.738	0.399	2.01E-14	EC	3	Gmfg
Podxl2	2.49E-17	0.963893215	0.543	0.117	4.99E-14	EC	3	Podxl
Tuba1b4	2.28E-16	0.415790407	0.902	0.661	4.57E-13	EC	3	Tuba1b
Nfkbia2	2.67E-16	0.538992859	0.768	0.473	5.35E-13	EC	3	Nfkbia
Esam3	4.21E-16	0.334758307	0.524	0.2	8.42E-13	EC	3	Esam
Bst22	7.32E-16	0.642945208	0.756	0.494	1.46E-12	EC	3	Bst2
Meox12	6.14E-15	0.711017594	0.518	0.133	1.23E-11	EC	3	Meox1
Lmo21	9.59E-15	0.614833314	0.689	0.423	1.92E-11	EC	3	Lmo2
Gpr182	7.48E-14	0.921892703	0.488	0.042	1.50E-10	EC	3	Gpr182
Fndc12	1.05E-13	0.343871327	0.72	0.47	2.09E-10	EC	3	Fndc1
Aldoa3	1.75E-13	0.333454018	0.939	0.767	3.51E-10	EC	3	Aldoa
Btnl91	8.80E-13	0.806838066	0.5	0.092	1.76E-09	EC	3	Btnl9
Pitpnc11	9.95E-13	0.670654998	0.518	0.242	1.99E-09	EC	3	Pitpnc1
Tuba1a3	3.76E-11	0.372387984	0.848	0.632	7.53E-08	EC	3	Tuba1a
Hsp90b11	3.90E-11	0.417730699	0.823	0.672	7.80E-08	EC	3	Hsp90b1
Pdpn3	6.86E-11	0.616507865	0.579	0.289	1.37E-07	EC	3	Pdpn
Tinagl17	1.38E-10	0.461299884	0.549	0.259	2.77E-07	EC	3	Tinagl1
Nos32	2.09E-09	0.41658052	0.421	0.093	4.18E-06	EC	3	Nos3
Hes13	9.21E-09	0.639342757	0.561	0.308	1.84E-05	EC	3	Hes1
Gja12	2.19E-08	0.687017479	0.524	0.295	4.38E-05	EC	3	Gja1
Slc3a22	3.05E-08	0.523489921	0.652	0.439	6.11E-05	EC	3	Slc3a2
Tubb5	3.88E-07	0.277371297	0.835	0.732	0.000775329	EC	3	Tubb5
Myct12	4.53E-07	0.783805194	0.463	0.114	0.000906667	EC	3	Myct1
Mgll2	1.57E-06	0.54898179	0.567	0.403	0.003144224	EC	3	Mgll
Emp13	1.85E-06	0.425718741	0.598	0.414	0.003703172	EC	3	Emp1
Sox42	2.52E-06	0.353472419	0.53	0.313	0.005047682	EC	3	Sox4
Nudt44	4.05E-06	0.306643534	0.628	0.364	0.008107888	EC	3	Nudt4
Htr2b	4.66E-06	0.582122909	0.293	0.048	0.009329109	EC	3	Htr2b
Gm424181	5.18E-06	0.85492852	0.927	0.824	0.010369187	EC	3	Gm42418
Tspan72	6.06E-06	0.416878532	0.293	0.308	0.012119317	EC	3	Tspan7
Pdlim44	1.17E-05	0.318281271	0.591	0.433	0.02333117	EC	3	Pdlim4
Tsc22d15	1.88E-05	0.688458698	0.701	0.547	0.037625231	EC	3	Tsc22d1
Fabp43	2.29E-05	0.478805768	0.616	0.439	0.045765938	EC	3	Fabp4
Slfn2	5.24E-05	0.394202632	0.494	0.298	0.104706218	EC	3	Slfn2
Thbd4	8.31E-05	0.449626127	0.561	0.368	0.166176517	EC	3	Thbd
Sdc12	8.39E-05	0.295242048	0.287	0.309	0.167706525	EC	3	Sdc1
Gbp21	0.000111795	0.318717448	0.512	0.32	0.22358956	EC	3	Gbp2
Iigp12	0.000196644	0.300127736	0.293	0.303	0.393287904	EC	3	Iigp1
Rbp71	0.000639463	0.657062908	0.348	0.051	1	EC	3	Rbp7

Cd300lg1	0.000782496	0.471254127	0.421	0.166	1	EC	3	Cd300lg
Bok2	0.000992241	0.418763521	0.421	0.231	1	EC	3	Bok
Arhgap312	0.001148588	0.460431138	0.524	0.319	1	EC	3	Arhgap31
Cd933	0.00129487	0.313235937	0.293	0.205	1	EC	3	Cd93
Crim15	0.004876288	0.361877858	0.476	0.286	1	EC	3	Crim1
Tubb4b1	0.005337833	0.305867813	0.549	0.408	1	EC	3	Tubb4b
Limch15	0.006068031	0.29353017	0.47	0.343	1	EC	3	Limch1
Pglyrp1	0.009042535	0.591798853	0.421	0.226	1	EC	3	Pglyrp1
C1qa	0	3.386641428	1	0.401	0	Mo/MΦ	1	C1qa
C1qc1	0	3.303070962	1	0.33	0	Mo/MΦ	1	C1qc
C1qb	0	3.29804854	0.999	0.35	0	Mo/MΦ	1	C1qb
Pf4	0	3.182543411	0.988	0.376	0	Mo/MΦ	1	Pf4
Lyz2	0	3.04949603	1	0.613	0	Mo/MΦ	1	Lyz2
Retnla	0	2.559727284	0.861	0.622	0	Mo/MΦ	1	Retnla
F13a11	0	2.53357343	0.925	0.202	0	Mo/MΦ	1	F13a1
Wfdc17	0	2.504523569	0.943	0.299	0	Mo/MΦ	1	Wfdc17
Folr2	0	2.433596056	0.895	0.191	0	Mo/MΦ	1	Folr2
Ccl6	0	2.396462755	0.943	0.317	0	Mo/MΦ	1	Ccl6
Fcer1g	0	2.306668762	0.996	0.319	0	Mo/MΦ	1	Fcer1g
Apoe2	0	2.293047681	0.995	0.828	0	Mo/MΦ	1	Apoe
Csf1r1	0	2.266702929	0.97	0.212	0	Mo/MΦ	1	Csf1r
Fxyd2	0	2.220015582	0.877	0.217	0	Mo/MΦ	1	Fxyd2
Tyrobp	0	2.202883831	0.993	0.309	0	Mo/MΦ	1	Tyrobp
Mrc11	0	2.07560066	0.905	0.232	0	Mo/MΦ	1	Mrc1
Sepp12	0	2.063877236	0.996	0.806	0	Mo/MΦ	1	Sepp1
Ccl9	0	2.034845036	0.929	0.225	0	Mo/MΦ	1	Ccl9
Dab21	0	2.003868514	0.917	0.34	0	Mo/MΦ	1	Dab2
Ccl24	0	1.987834875	0.802	0.179	0	Mo/MΦ	1	Ccl24
Fcgr31	0	1.909625019	0.901	0.184	0	Mo/MΦ	1	Fcgr3
Ninj11	0	1.899612003	0.893	0.37	0	Mo/MΦ	1	Ninj1
Ctsc1	0	1.865596805	0.926	0.287	0	Mo/MΦ	1	Ctsc
Lyve12	0	1.846380979	0.785	0.182	0	Mo/MΦ	1	Lyve1
Ltc4s	0	1.844385629	0.801	0.171	0	Mo/MΦ	1	Ltc4s
Ccl8	0	1.838443035	0.586	0.18	0	Mo/MΦ	1	Ccl8
Cd209f1	0	1.826893512	0.56	0.183	0	Mo/MΦ	1	Cd209f
Maf2	0	1.813318678	0.919	0.43	0	Mo/MΦ	1	Maf
Cd1631	0	1.778409205	0.719	0.177	0	Mo/MΦ	1	Cd163
Pltp2	0	1.767077543	0.889	0.359	0	Mo/MΦ	1	Pltp
Cbr22	0	1.758148927	0.921	0.35	0	Mo/MΦ	1	Cbr2
Mgl2	0	1.746570518	0.821	0.194	0	Mo/MΦ	1	Mgl2
Stab13	0	1.738702978	0.793	0.17	0	Mo/MΦ	1	Stab1
Trf1	0	1.732824918	0.909	0.274	0	Mo/MΦ	1	Trf
Aif1	0	1.700939297	0.835	0.216	0	Mo/MΦ	1	Aif1
Rgs10	0	1.621862883	0.919	0.378	0	Mo/MΦ	1	Rgs10
Fcna	0	1.620277947	0.67	0.224	0	Mo/MΦ	1	Fcna
Lst1	0	1.55952321	0.784	0.279	0	Mo/MΦ	1	Lst1
Cfp	0	1.548098682	0.795	0.197	0	Mo/MΦ	1	Cfp
Alox5ap	0	1.514736956	0.865	0.224	0	Mo/MΦ	1	Alox5ap
Mafb1	0	1.509014022	0.821	0.285	0	Mo/MΦ	1	Mafb
Ptpn18	0	1.477263048	0.957	0.399	0	Mo/MΦ	1	Ptpn18
C5ar11	0	1.469470532	0.714	0.126	0	Mo/MΦ	1	C5ar1
Fcgr2b1	0	1.450522453	0.799	0.195	0	Mo/MΦ	1	Fcgr2b
Cd362	0	1.426782164	0.819	0.201	0	Mo/MΦ	1	Cd36
Ctss	0	1.406059479	0.906	0.368	0	Mo/MΦ	1	Ctss
Ms4a6c1	0	1.402337537	0.754	0.144	0	Mo/MΦ	1	Ms4a6c
P2ry6	0	1.391302203	0.768	0.244	0	Mo/MΦ	1	P2ry6
Lgmn2	0	1.380769171	0.844	0.426	0	Mo/MΦ	1	Lgmn
Cd68	0	1.362296221	0.728	0.198	0	Mo/MΦ	1	Cd68
Clec10a1	0	1.332324228	0.645	0.134	0	Mo/MΦ	1	Clec10a
Adgre11	0	1.302955798	0.705	0.161	0	Mo/MΦ	1	Adgre1
Unc93b1	0	1.300081598	0.869	0.386	0	Mo/MΦ	1	Unc93b1
Cybb1	0	1.296463502	0.776	0.269	0	Mo/MΦ	1	Cybb
Cd209g	0	1.293970603	0.517	0.137	0	Mo/MΦ	1	Cd209g
Ucp21	0	1.289150522	0.924	0.392	0	Mo/MΦ	1	Ucp2
Ftl11	0	1.280271104	1	0.992	0	Mo/MΦ	1	Ftl1
Ptafr	0	1.274376944	0.692	0.22	0	Mo/MΦ	1	Ptafr
Fcrls	0	1.272689797	0.67	0.17	0	Mo/MΦ	1	Fcrls
C3ar1	0	1.269796849	0.692	0.123	0	Mo/MΦ	1	C3ar1
Ifi27l2a2	0	1.259577397	0.982	0.748	0	Mo/MΦ	1	Ifi27l2a
Pld4	0	1.256568915	0.767	0.244	0	Mo/MΦ	1	Pld4
Spi1	0	1.213494483	0.795	0.249	0	Mo/MΦ	1	Spi1
Ednrb2	0	1.190811242	0.613	0.166	0	Mo/MΦ	1	Ednrb
H2-Eb1	0	1.190156919	0.856	0.578	0	Mo/MΦ	1	H2-Eb1
Ms4a7	0	1.183387346	0.594	0.114	0	Mo/MΦ	1	Ms4a7
Laptm5	0	1.18325807	0.889	0.382	0	Mo/MΦ	1	Laptm5
H2-Aa	0	1.169695396	0.896	0.662	0	Mo/MΦ	1	H2-Aa
Marcksl1	0	1.159125877	0.712	0.321	0	Mo/MΦ	1	Marcksl1
Clec4a1	0	1.157370629	0.675	0.169	0	Mo/MΦ	1	Clec4a1
Hpgd	0	1.148785674	0.63	0.252	0	Mo/MΦ	1	Hpgd
Stard81	0	1.14492626	0.667	0.261	0	Mo/MΦ	1	Stard8
Tmem176b3	0	1.130281871	0.865	0.396	0	Mo/MΦ	1	Tmem176b
Ehd43	0	1.109526974	0.784	0.348	0	Mo/MΦ	1	Ehd4
Cd14	0	1.076515416	0.533	0.17	0	Mo/MΦ	1	Cd14
Lcp1	0	1.075015154	0.82	0.389	0	Mo/MΦ	1	Lcp1
Cd74	0	1.06651074	0.973	0.85	0	Mo/MΦ	1	Cd74
Smagp2	0	1.06466168	0.682	0.193	0	Mo/MΦ	1	Smagp
Mmp91	0	1.030801608	0.529	0.185	0	Mo/MΦ	1	Mmp9
H2-Ab1	0	1.022221348	0.915	0.668	0	Mo/MΦ	1	H2-Ab1
Gmfg1	0	1.014695958	0.849	0.355	0	Mo/MΦ	1	Gmfg
Sh3bgrl3	0	1.014387569	0.99	0.794	0	Mo/MΦ	1	Sh3bgrl3
C4b2	0	0.997187425	0.655	0.312	0	Mo/MΦ	1	C4b
Evi2a	0	0.994761228	0.674	0.334	0	Mo/MΦ	1	Evi2a
Kctd123	0	0.982376794	0.709	0.326	0	Mo/MΦ	1	Kctd12
Tgfbi1	0	0.966778928	0.814	0.43	0	Mo/MΦ	1	Tgfbi
Pla2g2d	0	0.949617772	0.495	0.12	0	Mo/MΦ	1	Pla2g2d
Fyb	0	0.948988052	0.645	0.226	0	Mo/MΦ	1	Fyb
Pla2g7	0	0.943477198	0.498	0.127	0	Mo/MΦ	1	Pla2g7
Clec4a2	0	0.942539533	0.582	0.174	0	Mo/MΦ	1	Clec4a2
Clec4a3	0	0.939345972	0.582	0.198	0	Mo/MΦ	1	Clec4a3
Ms4a6b	0	0.920398011	0.578	0.156	0	Mo/MΦ	1	Ms4a6b
Cotl1	0	0.906808338	0.845	0.473	0	Mo/MΦ	1	Cotl1
Ly86	0	0.903231762	0.679	0.341	0	Mo/MΦ	1	Ly86
Cd33	0	0.898678232	0.562	0.152	0	Mo/MΦ	1	Cd33
Tmem37	0	0.87335973	0.513	0.155	0	Mo/MΦ	1	Tmem37
Rassf4	0	0.868011797	0.59	0.27	0	Mo/MΦ	1	Rassf4
Timd4	0	0.860186456	0.517	0.157	0	Mo/MΦ	1	Timd4
Tmem176a2	0	0.853678172	0.72	0.308	0	Mo/MΦ	1	Tmem176a
Pirb	0	0.847888097	0.555	0.182	0	Mo/MΦ	1	Pirb
Lair1	0	0.844748784	0.537	0.096	0	Mo/MΦ	1	Lair1
Cd300ld	0	0.841660869	0.52	0.128	0	Mo/MΦ	1	Cd300ld
Ly6e3	0	0.839765602	0.926	0.782	0	Mo/MΦ	1	Ly6e
Dok2	0	0.839622419	0.545	0.216	0	Mo/MΦ	1	Dok2
Itgam	0	0.826177698	0.492	0.127	0	Mo/MΦ	1	Itgam
Ms4a6d	0	0.807601507	0.433	0.075	0	Mo/MΦ	1	Ms4a6d
F630028O10Rik	0	0.777698098	0.508	0.2	0	Mo/MΦ	1	F630028O10Rik
Trem2	0	0.760860444	0.45	0.09	0	Mo/MΦ	1	Trem2
Sdc31	0	0.740249468	0.556	0.257	0	Mo/MΦ	1	Sdc3
Hpgds	0	0.737828713	0.475	0.142	0	Mo/MΦ	1	Hpgds
Gas66	0	0.707167997	0.827	0.541	0	Mo/MΦ	1	Gas6
Cd48	0	0.706529456	0.605	0.302	0	Mo/MΦ	1	Cd48
Clec12a	0	0.705930497	0.487	0.189	0	Mo/MΦ	1	Clec12a
Tmsb4x5	0	0.66545778	1	1	0	Mo/MΦ	1	Tmsb4x
H2-D13	0	0.617057966	0.978	0.882	0	Mo/MΦ	1	H2-D1
B2m2	0	0.58725473	0.999	0.982	0	Mo/MΦ	1	B2m
Lilr4b	0	0.55410163	0.397	0.078	0	Mo/MΦ	1	Lilr4b
Cst33	0	0.530123909	1	0.953	0	Mo/MΦ	1	Cst3
Ifitm6	1.46E-307	0.439936617	0.424	0.132	2.92E-304	Mo/MΦ	1	Ifitm6
Cd53	1.11E-306	0.668851947	0.73	0.417	2.23E-303	Mo/MΦ	1	Cd53
AI6078732	6.36E-303	0.734872772	0.507	0.165	1.27E-299	Mo/MΦ	1	AI607873

Tnfrsf17	5.48E-300	0.325417197	0.339	0.065	1.10E-296	Mo/MΦ	1	Tnfrsf17
Cebpa	2.55E-299	0.714965704	0.523	0.244	5.11E-296	Mo/MΦ	1	Cebpa
Prune2	1.03E-296	0.395930361	0.374	0.106	2.05E-293	Mo/MΦ	1	Prune2
Cd381	1.08E-293	0.603001503	0.471	0.181	2.16E-290	Mo/MΦ	1	Cd38
Lilrb4a	3.18E-281	0.743163818	0.464	0.169	6.35E-278	Mo/MΦ	1	Lilrb4a
Ccl2	6.38E-277	0.83085613	0.416	0.165	1.28E-273	Mo/MΦ	1	Ccl2
Ccl12	2.88E-275	1.266814425	0.378	0.106	5.76E-272	Mo/MΦ	1	Ccl12
Hmox1	3.25E-257	0.913428331	0.528	0.253	6.51E-254	Mo/MΦ	1	Hmox1
Igf12	2.42E-255	0.471704968	0.768	0.456	4.84E-252	Mo/MΦ	1	Igf1
Ccl7	1.44E-250	0.97143179	0.398	0.143	2.88E-247	Mo/MΦ	1	Ccl7
Slamf9	3.54E-242	0.676354152	0.477	0.244	7.08E-239	Mo/MΦ	1	Slamf9
Arhgap30	4.45E-241	0.611286723	0.53	0.276	8.89E-238	Mo/MΦ	1	Arhgap30
Crip11	6.87E-239	0.441608096	0.993	0.965	1.37E-235	Mo/MΦ	1	Crip1
Fcgr1	1.57E-226	0.640689999	0.399	0.148	3.14E-223	Mo/MΦ	1	Fcgr1
Batf3	9.37E-225	0.528622387	0.457	0.245	1.87E-221	Mo/MΦ	1	Batf3
Hbb-bs1	4.47E-223	0.653446007	0.839	0.707	8.94E-220	Mo/MΦ	1	Hbb-bs
Arhgdib	1.70E-222	0.431369456	0.808	0.533	3.40E-219	Mo/MΦ	1	Arhgdib
Vsig4	6.75E-222	0.477205177	0.27	0.045	1.35E-218	Mo/MΦ	1	Vsig4
Epsti1	8.02E-217	0.654494559	0.518	0.277	1.60E-213	Mo/MΦ	1	Epsti1
Gatm	3.13E-211	0.530133975	0.407	0.152	6.27E-208	Mo/MΦ	1	Gatm
Nrp22	1.19E-208	0.477112612	0.427	0.209	2.39E-205	Mo/MΦ	1	Nrp2
Mpeg1	3.05E-205	0.591510854	0.454	0.235	6.10E-202	Mo/MΦ	1	Mpeg1
Gda1	2.16E-203	0.612270374	0.497	0.247	4.32E-200	Mo/MΦ	1	Gda
H2-DMa	1.48E-202	0.665409863	0.65	0.396	2.96E-199	Mo/MΦ	1	H2-DMa
Actb3	1.13E-200	0.446784434	1	0.994	2.26E-197	Mo/MΦ	1	Actb
Csf2ra	8.43E-200	0.612582588	0.489	0.268	1.69E-196	Mo/MΦ	1	Csf2ra
Siglech	6.52E-194	0.40863733	0.316	0.076	1.30E-190	Mo/MΦ	1	Siglech
H2-K12	6.05E-191	0.413350889	0.909	0.792	1.21E-187	Mo/MΦ	1	H2-K1
Slc6a6	2.06E-190	0.612854482	0.648	0.46	4.11E-187	Mo/MΦ	1	Slc6a6
Rasgrp31	4.87E-178	0.369414078	0.427	0.234	9.74E-175	Mo/MΦ	1	Rasgrp3
Clec4b1	1.15E-174	0.416410605	0.394	0.21	2.30E-171	Mo/MΦ	1	Clec4b1
Ccnd1	2.30E-174	0.44964659	0.457	0.272	4.60E-171	Mo/MΦ	1	Ccnd1
Irf8	1.36E-170	0.330200201	0.534	0.32	2.73E-167	Mo/MΦ	1	Irf8
H2-DMb1	2.15E-169	0.718342558	0.559	0.35	4.29E-166	Mo/MΦ	1	H2-DMb1
Selplg	1.26E-164	0.394760249	0.488	0.278	2.53E-161	Mo/MΦ	1	Selplg
Ifi2042	7.18E-164	0.562136265	0.438	0.198	1.44E-160	Mo/MΦ	1	Ifi204
Lyz1	1.55E-163	0.643664795	0.373	0.199	3.09E-160	Mo/MΦ	1	Lyz1
Plin2	9.30E-162	0.683402708	0.5	0.293	1.86E-158	Mo/MΦ	1	Plin2
Csf2rb1	3.27E-158	0.298695436	0.347	0.166	6.55E-155	Mo/MΦ	1	Csf2rb
Clec4n	1.28E-156	0.429259244	0.31	0.103	2.56E-153	Mo/MΦ	1	Clec4n
Gpr34	1.91E-156	0.660911145	0.416	0.204	3.82E-153	Mo/MΦ	1	Gpr34
Lmo22	9.97E-156	0.444458202	0.599	0.408	1.99E-152	Mo/MΦ	1	Lmo2
Ccr1	1.63E-155	0.510564536	0.376	0.175	3.26E-152	Mo/MΦ	1	Ccr1
Hba-a11	1.16E-153	0.504648333	0.731	0.543	2.32E-150	Mo/MΦ	1	Hba-a1
Ccdc109b	2.04E-151	0.482972463	0.422	0.23	4.08E-148	Mo/MΦ	1	Ccdc109b
Lgals3	3.33E-151	0.54602728	0.536	0.324	6.67E-148	Mo/MΦ	1	Lgals3
Bst23	1.55E-150	0.50096097	0.669	0.478	3.10E-147	Mo/MΦ	1	Bst2
Cd84	1.63E-150	0.51735226	0.473	0.287	3.25E-147	Mo/MΦ	1	Cd84
Sdf2l1	3.36E-149	0.465130165	0.477	0.263	6.72E-146	Mo/MΦ	1	Sdf2l1
Pou2f21	2.73E-146	0.427274322	0.51	0.332	5.47E-143	Mo/MΦ	1	Pou2f2
Mt11	6.13E-146	0.400439752	0.917	0.742	1.23E-142	Mo/MΦ	1	Mt1
Ctla2b2	4.13E-140	0.483153846	0.34	0.148	8.27E-137	Mo/MΦ	1	Ctla2b
H2afz	8.55E-140	0.330670286	0.84	0.698	1.71E-136	Mo/MΦ	1	H2afz
Pycard	3.22E-137	0.424556387	0.541	0.365	6.43E-134	Mo/MΦ	1	Pycard
Cfh3	6.24E-136	0.309579194	0.811	0.582	1.25E-132	Mo/MΦ	1	Cfh
Slfn21	7.01E-135	0.523121937	0.463	0.282	1.40E-131	Mo/MΦ	1	Slfn2
Slc11a11	2.08E-133	0.546293854	0.422	0.25	4.16E-130	Mo/MΦ	1	Slc11a1
Arhgap313	2.41E-130	0.394113717	0.483	0.304	4.82E-127	Mo/MΦ	1	Arhgap31
Adrb21	8.99E-130	0.556763373	0.404	0.234	1.80E-126	Mo/MΦ	1	Adrb2
Iqgap2	9.67E-130	0.450889026	0.393	0.234	1.93E-126	Mo/MΦ	1	Iqgap2
Hba-a21	7.41E-128	0.4800806	0.672	0.502	1.48E-124	Mo/MΦ	1	Hba-a2
Klf22	1.43E-127	0.495803723	0.717	0.55	2.86E-124	Mo/MΦ	1	Klf2
Tifab	1.13E-122	0.266909729	0.317	0.137	2.26E-119	Mo/MΦ	1	Tifab
Cxcl16	2.81E-120	0.530330005	0.456	0.295	5.63E-117	Mo/MΦ	1	Cxcl16
Gm4951	3.03E-108	0.440501656	0.349	0.195	6.07E-105	Mo/MΦ	1	Gm4951
Hbb-bt	3.43E-108	0.399516187	0.573	0.414	6.86E-105	Mo/MΦ	1	Hbb-bt
Plek	3.04E-101	0.50919492	0.378	0.227	6.08E-98	Mo/MΦ	1	Plek
Fxyd52	2.29E-99	0.403922831	0.609	0.444	4.57E-96	Mo/MΦ	1	Fxyd5
AF251705	3.28E-99	0.467763113	0.325	0.151	6.57E-96	Mo/MΦ	1	AF251705
Ptprc	1.31E-92	0.399048853	0.559	0.425	2.61E-89	Mo/MΦ	1	Ptprc
Cd209d	1.07E-91	0.740522574	0.267	0.103	2.13E-88	Mo/MΦ	1	Cd209d
Dpep2	1.45E-89	0.258358245	0.313	0.162	2.89E-86	Mo/MΦ	1	Dpep2
Rtp41	1.05E-88	0.390217031	0.357	0.228	2.09E-85	Mo/MΦ	1	Rtp4
Slc40a1	1.23E-86	0.377482388	0.328	0.217	2.46E-83	Mo/MΦ	1	Slc40a1
Itgb2	1.38E-85	0.498253033	0.396	0.244	2.76E-82	Mo/MΦ	1	Itgb2
Ifi2031	1.21E-76	0.413964476	0.51	0.376	2.43E-73	Mo/MΦ	1	Ifi203
Ccr2	1.38E-75	0.426588336	0.33	0.225	2.76E-72	Mo/MΦ	1	Ccr2
Ccr5	4.68E-75	0.566442532	0.331	0.16	9.36E-72	Mo/MΦ	1	Ccr5
Cd300a	1.03E-65	0.436409426	0.29	0.127	2.05E-62	Mo/MΦ	1	Cd300a
Irf71	4.25E-58	0.281955209	0.334	0.263	8.50E-55	Mo/MΦ	1	Irf7
Cmah	8.16E-57	0.313358493	0.436	0.367	1.63E-53	Mo/MΦ	1	Cmah
AI662270	4.80E-55	0.350362596	0.384	0.29	9.61E-52	Mo/MΦ	1	AI662270
Bcl2a1b	3.05E-51	0.345235479	0.316	0.211	6.11E-48	Mo/MΦ	1	Bcl2a1b
Metrnl1	5.57E-51	0.37896836	0.488	0.398	1.11E-47	Mo/MΦ	1	Metrnl
Ccl4	6.72E-51	0.307216001	0.255	0.165	1.34E-47	Mo/MΦ	1	Ccl4
Ifi30	7.32E-48	0.39094078	0.483	0.413	1.46E-44	Mo/MΦ	1	Ifi30
Xlr	1.28E-47	0.271342755	0.26	0.175	2.55E-44	Mo/MΦ	1	Xlr
Mndal1	2.81E-45	0.292828423	0.426	0.346	5.61E-42	Mo/MΦ	1	Mndal
Shtn1	2.64E-40	0.362346732	0.286	0.181	5.29E-37	Mo/MΦ	1	Shtn1
Ccl3	1.69E-36	0.474882223	0.264	0.157	3.37E-33	Mo/MΦ	1	Ccl3
Slc3a23	1.45E-34	0.359190277	0.482	0.436	2.91E-31	Mo/MΦ	1	Slc3a2
Oasl21	7.19E-24	0.327977836	0.296	0.248	1.44E-20	Mo/MΦ	1	Oasl2
Selenbp12	2.05E-23	0.301464394	0.44	0.411	4.11E-20	Mo/MΦ	1	Selenbp1
Hspa1a2	2.96E-23	0.573204895	0.426	0.395	5.91E-20	Mo/MΦ	1	Hspa1a
Rgs2	1.87E-20	0.287479414	0.377	0.342	3.75E-17	Mo/MΦ	1	Rgs2
Nfkbiz	1.00E-16	0.380589908	0.305	0.287	2.00E-13	Mo/MΦ	1	Nfkbiz
Ier32	6.90E-12	0.368538988	0.329	0.322	1.38E-08	Mo/MΦ	1	Ier3
Cd83	4.03E-06	0.445992335	0.323	0.322	0.008054945	Mo/MΦ	1	Cd83
Zfp363	4.76E-05	0.486659638	0.438	0.472	0.095136105	Mo/MΦ	1	Zfp36
Fos4	0.000337099	0.396792485	0.472	0.511	0.674198719	Mo/MΦ	1	Fos
Neil3	0	0.747823033	0.622	0.025	0	Mo/MΦ	2	Neil3
Mki67	5.89E-284	1.423359141	0.836	0.069	1.18E-280	Mo/MΦ	2	Mki67
Cdca3	1.59E-274	1.230789331	0.794	0.08	3.18E-271	Mo/MΦ	2	Cdca3
2810417H13Rik	7.44E-241	1.963357177	0.871	0.102	1.49E-237	Mo/MΦ	2	2810417H13Rik
Top2a	2.40E-240	1.838626322	0.902	0.111	4.79E-237	Mo/MΦ	2	Top2a
Stmn1	1.29E-208	2.622081443	0.979	0.202	2.58E-205	Mo/MΦ	2	Stmn1
Cenpm	1.96E-206	0.824839853	0.696	0.082	3.92E-203	Mo/MΦ	2	Cenpm
Mxd3	7.52E-206	0.662871098	0.601	0.045	1.50E-202	Mo/MΦ	2	Mxd3
Birc5	1.29E-201	1.75298109	0.899	0.185	2.57E-198	Mo/MΦ	2	Birc5
Ccna2	3.29E-201	0.952918744	0.696	0.094	6.57E-198	Mo/MΦ	2	Ccna2
Hmgb2	3.23E-197	2.697335193	1	0.379	6.45E-194	Mo/MΦ	2	Hmgb2
Depdc1a	6.13E-185	0.301151706	0.406	0.019	1.23E-181	Mo/MΦ	2	Depdc1a
Cenpf	2.01E-182	1.370774532	0.689	0.056	4.02E-179	Mo/MΦ	2	Cenpf
H2afz1	9.46E-179	2.149403166	1	0.708	1.89E-175	Mo/MΦ	2	H2afz
Fam64a	2.45E-175	0.690609888	0.51	0.02	4.90E-172	Mo/MΦ	2	Fam64a
Cdkn3	3.95E-170	0.779924488	0.577	0.042	7.90E-167	Mo/MΦ	2	Cdkn3
Nuf2	4.19E-167	0.58075206	0.524	0.044	8.39E-164	Mo/MΦ	2	Nuf2
Ccnb2	2.84E-163	1.040354372	0.664	0.088	5.68E-160	Mo/MΦ	2	Ccnb2
Casc5	3.32E-162	0.635948169	0.5	0.023	6.64E-159	Mo/MΦ	2	Casc5
Hist1h2bm	8.68E-157	0.33166961	0.497	0.065	1.74E-153	Mo/MΦ	2	Hist1h2bm
1700097N02Rik	1.57E-152	0.262325875	0.43	0.038	3.14E-149	Mo/MΦ	2	1700097N02Rik
Nusap1	1.12E-151	1.092544658	0.71	0.123	2.25E-148	Mo/MΦ	2	Nusap1
Ube2c	1.10E-149	1.818627392	0.776	0.126	2.21E-146	Mo/MΦ	2	Ube2c
Aspm	5.92E-148	0.445438884	0.406	0.014	1.18E-144	Mo/MΦ	2	Aspm
Cdca8	1.92E-146	1.076677744	0.713	0.065	3.83E-143	Mo/MΦ	2	Cdca8
Tubb51	3.70E-142	1.736406189	0.986	0.73	7.41E-139	Mo/MΦ	2	Tubb5
Tuba1b5	4.11E-138	1.618057702	0.976	0.659	8.22E-135	Mo/MΦ	2	Tuba1b
Cks1b	4.48E-136	1.306253786	0.892	0.276	8.96E-133	Mo/MΦ	2	Cks1b
Cks2	6.58E-134	1.677244399	0.818	0.208	1.32E-130	Mo/MΦ	2	Cks2

Asf1b	6.69E-134	0.719864595	0.636	0.068	1.34E-130	Mo/MΦ	2	Asf1b
Lmnb1	3.58E-132	1.040739579	0.815	0.246	7.15E-129	Mo/MΦ	2	Lmnb1
Cdc20	5.43E-124	0.939750197	0.598	0.107	1.09E-120	Mo/MΦ	2	Cdc20
Hmmr	1.01E-122	0.587558057	0.42	0.015	2.01E-119	Mo/MΦ	2	Hmmr
Uhrf1	1.25E-119	0.577512771	0.577	0.081	2.49E-116	Mo/MΦ	2	Uhrf1
Hmgn2	2.79E-118	1.447883274	0.927	0.474	5.58E-115	Mo/MΦ	2	Hmgn2
Racgap1	1.30E-116	0.784200066	0.657	0.125	2.60E-113	Mo/MΦ	2	Racgap1
Cenpa	2.79E-114	1.360949094	0.79	0.28	5.59E-111	Mo/MΦ	2	Cenpa
Spc24	7.34E-114	0.990383836	0.72	0.13	1.47E-110	Mo/MΦ	2	Spc24
Ckap2l	5.70E-111	0.646968614	0.517	0.058	1.14E-107	Mo/MΦ	2	Ckap2l
Tk1	6.04E-110	0.880406229	0.654	0.131	1.21E-106	Mo/MΦ	2	Tk1
H2afx	2.22E-109	1.419450783	0.801	0.256	4.43E-106	Mo/MΦ	2	H2afx
Cdk1	5.77E-109	0.937341746	0.619	0.089	1.15E-105	Mo/MΦ	2	Cdk1
Knstrn	7.58E-109	0.812516124	0.65	0.122	1.52E-105	Mo/MΦ	2	Knstrn
Kif11	1.19E-108	0.523259485	0.49	0.045	2.38E-105	Mo/MΦ	2	Kif11
Prc1	3.85E-108	0.971266498	0.598	0.082	7.70E-105	Mo/MΦ	2	Prc1
Lig1	7.04E-107	0.952180041	0.752	0.252	1.41E-103	Mo/MΦ	2	Lig1
Aurkb	1.25E-104	0.62614739	0.549	0.071	2.49E-101	Mo/MΦ	2	Aurkb
Lockd	4.08E-103	1.369240004	0.741	0.256	8.16E-100	Mo/MΦ	2	Lockd
Smc2	3.81E-102	1.004005928	0.741	0.173	7.63E-99	Mo/MΦ	2	Smc2
Kif20a	1.20E-100	0.425068136	0.465	0.038	2.40E-97	Mo/MΦ	2	Kif20a
Smc4	2.52E-99	1.140869478	0.804	0.307	5.04E-96	Mo/MΦ	2	Smc4
Gmnn	4.58E-98	0.798592966	0.706	0.186	9.16E-95	Mo/MΦ	2	Gmnn
Sh2d1a	2.38E-95	0.339893748	0.423	0.061	4.76E-92	Mo/MΦ	2	Sh2d1a
Cenpe	9.40E-92	0.775571346	0.538	0.078	1.88E-88	Mo/MΦ	2	Cenpe
Kif23	1.31E-86	0.711364439	0.528	0.067	2.62E-83	Mo/MΦ	2	Kif23
Tpx2	4.30E-86	0.798168143	0.591	0.122	8.60E-83	Mo/MΦ	2	Tpx2
Arl6ip1	1.53E-82	1.13479585	0.934	0.521	3.06E-79	Mo/MΦ	2	Arl6ip1
Bcl2a1d	9.48E-82	0.488064351	0.591	0.145	1.90E-78	Mo/MΦ	2	Bcl2a1d
Spc25	3.38E-81	0.808217433	0.552	0.085	6.76E-78	Mo/MΦ	2	Spc25
Xcr1	3.66E-81	0.276912489	0.455	0.103	7.32E-78	Mo/MΦ	2	Xcr1
Dut	4.31E-80	0.963345847	0.78	0.344	8.61E-77	Mo/MΦ	2	Dut
Esco2	1.92E-79	0.405704055	0.371	0.011	3.84E-76	Mo/MΦ	2	Esco2
Sgol1	2.17E-79	0.583134985	0.507	0.057	4.34E-76	Mo/MΦ	2	Sgol1
AI6622701	1.37E-76	0.598272808	0.738	0.294	2.75E-73	Mo/MΦ	2	AI662270
Ucp22	2.50E-75	0.85918278	0.92	0.435	4.99E-72	Mo/MΦ	2	Ucp2
Sh3bgrl31	8.76E-75	0.876827984	0.99	0.81	1.75E-71	Mo/MΦ	2	Sh3bgrl3
Alox5ap1	1.31E-72	0.825087165	0.773	0.277	2.62E-69	Mo/MΦ	2	Alox5ap
Ccr21	1.49E-71	1.015840116	0.664	0.229	2.97E-68	Mo/MΦ	2	Ccr2
Mcm5	1.68E-70	0.488006762	0.57	0.155	3.35E-67	Mo/MΦ	2	Mcm5
Selplg1	3.75E-67	0.626468319	0.762	0.292	7.50E-64	Mo/MΦ	2	Selplg
Spi11	1.06E-66	0.698465006	0.752	0.294	2.12E-63	Mo/MΦ	2	Spi1
Plbd1	7.03E-66	0.679533869	0.64	0.246	1.41E-62	Mo/MΦ	2	Plbd1
Cotl11	1.32E-65	0.803424074	0.895	0.503	2.64E-62	Mo/MΦ	2	Cotl1
Laptm51	1.34E-64	0.794278483	0.871	0.424	2.68E-61	Mo/MΦ	2	Laptm5
Pycard1	2.92E-63	0.708241873	0.822	0.376	5.83E-60	Mo/MΦ	2	Pycard
Tmsb4x6	5.88E-63	0.782033282	1	1	1.18E-59	Mo/MΦ	2	Tmsb4x
Mis18bp1	7.00E-63	0.450738224	0.43	0.045	1.40E-59	Mo/MΦ	2	Mis18bp1
Coro1a	6.71E-62	0.75587484	0.927	0.535	1.34E-58	Mo/MΦ	2	Coro1a
H2-DMb11	5.78E-61	1.029750115	0.745	0.364	1.16E-57	Mo/MΦ	2	H2-DMb1
Cit	1.92E-59	0.747980308	0.57	0.15	3.85E-56	Mo/MΦ	2	Cit
Hells	4.87E-59	0.509069471	0.507	0.082	9.73E-56	Mo/MΦ	2	Hells
Cd481	5.38E-59	0.571807105	0.738	0.325	1.08E-55	Mo/MΦ	2	Cd48
Gatm1	8.61E-59	0.300639626	0.542	0.171	1.72E-55	Mo/MΦ	2	Gatm
Actb4	2.49E-57	0.798126559	1	0.994	4.98E-54	Mo/MΦ	2	Actb
Klrk1	8.92E-57	0.39670932	0.455	0.093	1.78E-53	Mo/MΦ	2	Klrk1
Lcp11	2.07E-56	0.718015533	0.818	0.424	4.14E-53	Mo/MΦ	2	Lcp1
Psmb91	2.10E-56	0.724172018	0.78	0.43	4.21E-53	Mo/MΦ	2	Psmb9
Kif15	1.36E-55	0.373190676	0.385	0.019	2.73E-52	Mo/MΦ	2	Kif15
Tyrobp1	3.58E-54	0.633442984	0.822	0.367	7.15E-51	Mo/MΦ	2	Tyrobp
H2-DMa1	7.21E-54	0.771524831	0.794	0.415	1.44E-50	Mo/MΦ	2	H2-DMa
Sdf2l11	4.18E-53	0.598790809	0.724	0.277	8.37E-50	Mo/MΦ	2	Sdf2l1
Klrd1	4.56E-53	0.301252619	0.497	0.151	9.12E-50	Mo/MΦ	2	Klrd1
Cx3cr1	5.36E-53	0.425874206	0.5	0.169	1.07E-49	Mo/MΦ	2	Cx3cr1
Rrm2	2.08E-52	0.824861375	0.545	0.135	4.17E-49	Mo/MΦ	2	Rrm2
Hist1h1b	2.58E-52	0.905734154	0.573	0.151	5.17E-49	Mo/MΦ	2	Hist1h1b
Pbk	3.49E-52	0.626320439	0.462	0.071	6.99E-49	Mo/MΦ	2	Pbk
Cd52	7.77E-52	0.761773421	0.927	0.623	1.55E-48	Mo/MΦ	2	Cd52
Ptpn181	1.10E-51	0.529133785	0.902	0.445	2.20E-48	Mo/MΦ	2	Ptpn18
Xcl1	5.70E-51	0.288358534	0.402	0.115	1.14E-47	Mo/MΦ	2	Xcl1
Clec12a1	1.10E-50	0.48189651	0.563	0.213	2.19E-47	Mo/MΦ	2	Clec12a
Plek1	1.84E-50	0.292358838	0.573	0.237	3.68E-47	Mo/MΦ	2	Plek
Cdkn2c	8.24E-50	0.99194848	0.671	0.331	1.65E-46	Mo/MΦ	2	Cdkn2c
Fcer1g1	6.79E-49	0.821844093	0.797	0.376	1.36E-45	Mo/MΦ	2	Fcer1g
Lgals31	1.10E-48	1.004781542	0.717	0.339	2.20E-45	Mo/MΦ	2	Lgals3
Tuba1c2	5.10E-48	0.771777474	0.661	0.327	1.02E-44	Mo/MΦ	2	Tuba1c
Crip12	9.01E-48	0.750575345	0.997	0.968	1.80E-44	Mo/MΦ	2	Crip1
Pld41	8.71E-46	0.428173663	0.671	0.288	1.74E-42	Mo/MΦ	2	Pld4
Slamf91	1.70E-45	0.413794446	0.591	0.261	3.39E-42	Mo/MΦ	2	Slamf9
Psmb82	1.70E-45	0.710600385	0.857	0.535	3.39E-42	Mo/MΦ	2	Psmb8
Aif11	4.32E-44	0.578940347	0.622	0.269	8.64E-41	Mo/MΦ	2	Aif1
Clec4b11	1.23E-43	0.614106701	0.538	0.223	2.45E-40	Mo/MΦ	2	Clec4b1
Rac2	2.45E-43	0.557567984	0.85	0.497	4.89E-40	Mo/MΦ	2	Rac2
Ms4a6c2	5.42E-43	0.585570468	0.598	0.195	1.08E-39	Mo/MΦ	2	Ms4a6c
Sec11c	7.12E-43	0.528728167	0.801	0.426	1.42E-39	Mo/MΦ	2	Sec11c
Apobec3	7.38E-43	0.461370617	0.738	0.391	1.48E-39	Mo/MΦ	2	Apobec3
Fxyd53	1.02E-42	0.609577812	0.829	0.455	2.04E-39	Mo/MΦ	2	Fxyd5
Cd3e	4.88E-42	0.332293288	0.483	0.167	9.76E-39	Mo/MΦ	2	Cd3e
Lyz21	7.42E-42	0.362740987	0.93	0.645	1.48E-38	Mo/MΦ	2	Lyz2
Arhgdib1	8.41E-42	0.557209742	0.888	0.554	1.68E-38	Mo/MΦ	2	Arhgdib
Csf2ra1	1.64E-41	0.447346151	0.608	0.284	3.27E-38	Mo/MΦ	2	Csf2ra
P2ry61	1.07E-39	0.352528948	0.608	0.288	2.13E-36	Mo/MΦ	2	P2ry6
Gmfg2	9.39E-39	0.515600844	0.811	0.396	1.88E-35	Mo/MΦ	2	Gmfg
Tmsb101	1.00E-38	0.734191058	0.993	0.949	2.00E-35	Mo/MΦ	2	Tmsb10
H2-Eb11	1.33E-38	0.888011735	0.878	0.6	2.67E-35	Mo/MΦ	2	H2-Eb1
Fyb1	1.85E-38	0.359951456	0.608	0.26	3.69E-35	Mo/MΦ	2	Fyb
Samsn1	4.28E-38	0.325299137	0.531	0.218	8.56E-35	Mo/MΦ	2	Samsn1
H2-Ab11	1.51E-37	0.954912935	0.913	0.688	3.01E-34	Mo/MΦ	2	H2-Ab1
Ly861	3.02E-37	0.373624638	0.664	0.369	6.05E-34	Mo/MΦ	2	Ly86
Bcl2a1b1	3.23E-37	0.27197479	0.531	0.217	6.46E-34	Mo/MΦ	2	Bcl2a1b
Lsp1	7.62E-37	0.636904244	0.892	0.626	1.52E-33	Mo/MΦ	2	Lsp1
Map4k1	8.29E-37	0.270200224	0.549	0.27	1.66E-33	Mo/MΦ	2	Map4k1
Spink2	1.12E-36	0.259519209	0.402	0.127	2.25E-33	Mo/MΦ	2	Spink2
Lat	1.33E-36	0.482225744	0.503	0.237	2.65E-33	Mo/MΦ	2	Lat
Tubb4b2	5.42E-36	0.869783095	0.71	0.405	1.08E-32	Mo/MΦ	2	Tubb4b
H2-Aa1	1.64E-35	0.845518751	0.913	0.681	3.27E-32	Mo/MΦ	2	H2-Aa
Kif22	2.42E-35	0.554770726	0.479	0.15	4.84E-32	Mo/MΦ	2	Kif22
Cfp1	1.38E-34	0.458224021	0.594	0.248	2.76E-31	Mo/MΦ	2	Cfp
Ccnd11	2.55E-34	0.373538722	0.577	0.285	5.10E-31	Mo/MΦ	2	Ccnd1
Srgn2	5.06E-34	0.380248929	0.825	0.495	1.01E-30	Mo/MΦ	2	Srgn
Dlgap5	2.34E-33	0.393441303	0.346	0.025	4.68E-30	Mo/MΦ	2	Dlgap5
Plk1	2.98E-33	0.42614937	0.367	0.049	5.96E-30	Mo/MΦ	2	Plk1
C330027C09Rik	3.14E-33	0.360745618	0.392	0.062	6.29E-30	Mo/MΦ	2	C330027C09Rik
Cd741	8.01E-33	0.729437672	0.979	0.86	1.60E-29	Mo/MΦ	2	Cd74
Cbfa2t33	1.08E-32	0.519320123	0.57	0.248	2.15E-29	Mo/MΦ	2	Cbfa2t3
Bub1	1.99E-32	0.302854103	0.294	0.004	3.98E-29	Mo/MΦ	2	Bub1
Gapdh1	4.04E-32	0.420536775	0.951	0.809	8.08E-29	Mo/MΦ	2	Gapdh
Rgs101	9.01E-32	0.426442899	0.752	0.424	1.80E-28	Mo/MΦ	2	Rgs10
Hbb-bt1	2.32E-31	0.31074894	0.727	0.425	4.63E-28	Mo/MΦ	2	Hbb-bt
Mpeg11	2.72E-31	0.605842873	0.556	0.251	5.43E-28	Mo/MΦ	2	Mpeg1
Csf2rb2	8.28E-31	0.253222345	0.444	0.179	1.66E-27	Mo/MΦ	2	Csf2rb
Ptpn6	1.27E-30	0.333209373	0.668	0.387	2.55E-27	Mo/MΦ	2	Ptpn6
Ccl41	3.05E-30	0.293478391	0.413	0.17	6.10E-27	Mo/MΦ	2	Ccl4
Cd209a	5.25E-30	0.636205863	0.458	0.202	1.05E-26	Mo/MΦ	2	Cd209a
Ccnb1	2.32E-29	0.653752366	0.437	0.103	4.63E-26	Mo/MΦ	2	Ccnb1
Napsa	2.63E-29	0.36592137	0.587	0.297	5.25E-26	Mo/MΦ	2	Napsa
Rnase6	3.30E-29	0.429098861	0.552	0.267	6.61E-26	Mo/MΦ	2	Rnase6
Ctss1	7.28E-29	0.373763484	0.755	0.414	1.46E-25	Mo/MΦ	2	Ctss
Kif20b	5.70E-28	0.42214388	0.416	0.096	1.14E-24	Mo/MΦ	2	Kif20b

Ptprc1	9.28E-28	0.477204576	0.724	0.434	1.86E-24	Mo/MΦ	2	Ptprc
BC0285281	8.69E-27	0.366859194	0.58	0.309	1.74E-23	Mo/MΦ	2	BC028528
Ifi301	1.82E-26	0.51395421	0.668	0.416	3.64E-23	Mo/MΦ	2	Ifi30
Pim1	1.02E-25	0.311013433	0.563	0.265	2.05E-22	Mo/MΦ	2	Pim1
Cd3d	1.73E-25	0.372947366	0.486	0.27	3.45E-22	Mo/MΦ	2	Cd3d
Mgl21	2.04E-25	0.409615437	0.528	0.249	4.08E-22	Mo/MΦ	2	Mgl2
Lck	3.08E-25	0.497953493	0.5	0.247	6.16E-22	Mo/MΦ	2	Lck
Cep55	8.97E-25	0.330113172	0.367	0.05	1.79E-21	Mo/MΦ	2	Cep55
Dok21	1.62E-23	0.295475612	0.49	0.243	3.23E-20	Mo/MΦ	2	Dok2
H2-Q72	3.40E-23	0.432006433	0.626	0.377	6.80E-20	Mo/MΦ	2	H2-Q7
Ccl5	3.78E-23	0.961974245	0.465	0.255	7.56E-20	Mo/MΦ	2	Ccl5
Cybb2	1.75E-22	0.371090051	0.587	0.312	3.50E-19	Mo/MΦ	2	Cybb
Gzma	2.13E-22	0.267577881	0.259	0.083	4.26E-19	Mo/MΦ	2	Gzma
Rassf41	2.46E-22	0.636642721	0.563	0.296	4.92E-19	Mo/MΦ	2	Rassf4
Cd681	5.11E-22	0.402427124	0.521	0.243	1.02E-18	Mo/MΦ	2	Cd68
Hbb-bs2	5.39E-22	0.396678218	0.885	0.717	1.08E-18	Mo/MΦ	2	Hbb-bs
Ccl91	7.28E-22	0.525513859	0.559	0.287	1.46E-18	Mo/MΦ	2	Ccl9
Hist1h2ap	3.26E-21	0.380744778	0.29	0.027	6.51E-18	Mo/MΦ	2	Hist1h2ap
Ckap2	5.17E-21	0.489092333	0.381	0.082	1.03E-17	Mo/MΦ	2	Ckap2
S100a42	7.51E-21	0.781071703	0.766	0.573	1.50E-17	Mo/MΦ	2	S100a4
Ctsc2	4.54E-20	0.290948765	0.612	0.343	9.09E-17	Mo/MΦ	2	Ctsc
Slfn22	8.25E-20	0.346064348	0.552	0.296	1.65E-16	Mo/MΦ	2	Slfn2
Batf31	1.67E-19	0.446501602	0.503	0.262	3.34E-16	Mo/MΦ	2	Batf3
Tcf7	2.33E-19	0.280594825	0.385	0.156	4.67E-16	Mo/MΦ	2	Tcf7
Arhgap301	1.11E-18	0.297325812	0.573	0.296	2.22E-15	Mo/MΦ	2	Arhgap30
Clec4a21	1.64E-18	0.302613245	0.451	0.209	3.28E-15	Mo/MΦ	2	Clec4a2
Kpna2	3.81E-18	0.473598855	0.42	0.197	7.61E-15	Mo/MΦ	2	Kpna2
Hist1h1e	8.13E-18	0.594251677	0.469	0.216	1.63E-14	Mo/MΦ	2	Hist1h1e
Sgol2a	2.88E-17	0.334467557	0.357	0.08	5.75E-14	Mo/MΦ	2	Sgol2a
Ly6e4	4.62E-17	0.273835332	0.937	0.794	9.24E-14	Mo/MΦ	2	Ly6e
Ear2	6.35E-17	0.499546179	0.409	0.209	1.27E-13	Mo/MΦ	2	Ear2
Tifab1	1.54E-16	0.310066253	0.413	0.15	3.07E-13	Mo/MΦ	2	Tifab
Mrc12	2.54E-16	0.284954569	0.503	0.291	5.08E-13	Mo/MΦ	2	Mrc1
Ccdc109b1	5.36E-15	0.357521631	0.469	0.245	1.07E-11	Mo/MΦ	2	Ccdc109b
Gm2a	9.09E-15	0.719160111	0.685	0.558	1.82E-11	Mo/MΦ	2	Gm2a
Kctd124	7.92E-14	0.319285221	0.559	0.359	1.58E-10	Mo/MΦ	2	Kctd12
Phf11b	8.80E-14	0.354762102	0.49	0.293	1.76E-10	Mo/MΦ	2	Phf11b
Cd8b1	3.26E-13	0.411193785	0.434	0.261	6.52E-10	Mo/MΦ	2	Cd8b1
Ncapg	2.11E-12	0.296267889	0.29	0.009	4.23E-09	Mo/MΦ	2	Ncapg
Glipr1	5.62E-12	0.391011638	0.465	0.262	1.12E-08	Mo/MΦ	2	Glipr1
Naaa	1.14E-11	0.728233728	0.531	0.384	2.29E-08	Mo/MΦ	2	Naaa
Retnla1	5.72E-11	0.333712821	0.857	0.641	1.14E-07	Mo/MΦ	2	Retnla
H2-D14	7.42E-11	0.265428692	0.951	0.89	1.48E-07	Mo/MΦ	2	H2-D1
H2-K13	1.34E-10	0.256411699	0.93	0.801	2.67E-07	Mo/MΦ	2	H2-K1
Itgb21	1.65E-10	0.354650476	0.462	0.255	3.30E-07	Mo/MΦ	2	Itgb2
Ms4a4b	1.73E-10	0.377740762	0.444	0.303	3.46E-07	Mo/MΦ	2	Ms4a4b
Cd24a2	2.11E-10	0.532023711	0.49	0.334	4.22E-07	Mo/MΦ	2	Cd24a
Ifi27l2a3	7.30E-10	0.469461969	0.913	0.768	1.46E-06	Mo/MΦ	2	Ifi27l2a
Fcrls1	8.22E-10	0.263059283	0.36	0.214	1.64E-06	Mo/MΦ	2	Fcrls
Irf81	1.58E-08	0.705796838	0.469	0.338	3.17E-05	Mo/MΦ	2	Irf8
AW1120102	1.27E-06	0.565647506	0.556	0.406	0.002543662	Mo/MΦ	2	AW112010
Lyz11	8.91E-06	0.852482146	0.339	0.214	0.017819613	Mo/MΦ	2	Lyz1
Slamf8	1.15E-05	0.258789613	0.35	0.185	0.023088003	Mo/MΦ	2	Slamf8
Itga4	1.23E-05	0.300466976	0.42	0.292	0.024667395	Mo/MΦ	2	Itga4
Thy12	4.24E-05	0.329675016	0.552	0.432	0.084712372	Mo/MΦ	2	Thy1
Hist1h4d	0.000195266	0.327097676	0.294	0.147	0.390532109	Mo/MΦ	2	Hist1h4d
Tnni2	0.000337892	0.361111649	0.308	0.367	0.675784842	Mo/MΦ	2	Tnni2
Cd8a	0.000481403	0.422235746	0.308	0.148	0.962806344	Mo/MΦ	2	Cd8a
Nkg7	0.000699103	0.723811575	0.35	0.263	1	Mo/MΦ	2	Nkg7
Id23	0.000938891	0.367926298	0.608	0.517	1	Mo/MΦ	2	Id2
Hist1h2ae	0.001045741	0.295213803	0.262	0.088	1	Mo/MΦ	2	Hist1h2ae
Wdfy4	0.004605719	0.382111084	0.357	0.238	1	Mo/MΦ	2	Wdfy4
Lyz22	1.98E-56	1.002064896	0.98	0.647	3.96E-53	Mo/MΦ	3	Lyz2
Fcer1g2	4.36E-54	0.909327522	0.96	0.377	8.71E-51	Mo/MΦ	3	Fcer1g
C1qc2	1.99E-50	0.99839298	0.887	0.389	3.98E-47	Mo/MΦ	3	C1qc
Csf1r2	1.63E-47	0.953910782	0.788	0.278	3.25E-44	Mo/MΦ	3	Csf1r
Pf41	2.12E-47	0.882244312	0.901	0.43	4.24E-44	Mo/MΦ	3	Pf4
C1qa1	1.89E-46	0.943106814	0.887	0.453	3.78E-43	Mo/MΦ	3	C1qa
F13a12	3.24E-46	1.061284586	0.762	0.265	6.48E-43	Mo/MΦ	3	F13a1
C1qb1	6.87E-46	0.923069269	0.874	0.407	1.37E-42	Mo/MΦ	3	C1qb
Tyrobp2	6.04E-45	0.863750665	0.894	0.369	1.21E-41	Mo/MΦ	3	Tyrobp
Wfdc171	1.80E-43	1.184268136	0.828	0.355	3.61E-40	Mo/MΦ	3	Wfdc17
Dab22	2.13E-41	0.923924139	0.815	0.391	4.27E-38	Mo/MΦ	3	Dab2
Mrc13	1.56E-38	0.917483142	0.715	0.291	3.13E-35	Mo/MΦ	3	Mrc1
Fcgr32	4.00E-38	0.665611575	0.715	0.247	7.99E-35	Mo/MΦ	3	Fcgr3
Ccl61	3.68E-37	0.816420474	0.795	0.372	7.36E-34	Mo/MΦ	3	Ccl6
Sepp13	1.07E-35	0.786907178	0.98	0.822	2.14E-32	Mo/MΦ	3	Sepp1
Ctsc3	8.07E-35	0.723576632	0.775	0.343	1.61E-31	Mo/MΦ	3	Ctsc
Fxyd21	1.58E-34	0.806906746	0.675	0.275	3.16E-31	Mo/MΦ	3	Fxyd2
Alox5ap2	7.50E-34	0.698818167	0.728	0.28	1.50E-30	Mo/MΦ	3	Alox5ap
Ccl92	2.10E-33	0.654976655	0.742	0.287	4.19E-30	Mo/MΦ	3	Ccl9
Ftl12	2.17E-31	0.523619516	1	0.992	4.35E-28	Mo/MΦ	3	Ftl1
Stab14	5.55E-30	0.726061027	0.609	0.225	1.11E-26	Mo/MΦ	3	Stab1
Cd363	9.30E-30	0.548514344	0.669	0.255	1.86E-26	Mo/MΦ	3	Cd36
Apoe3	4.23E-29	0.674704733	0.98	0.843	8.46E-26	Mo/MΦ	3	Apoe
Folr21	6.77E-29	0.699614327	0.623	0.253	1.35E-25	Mo/MΦ	3	Folr2
Ms4a6c3	7.54E-29	0.697495191	0.57	0.198	1.51E-25	Mo/MΦ	3	Ms4a6c
Maf3	2.12E-28	0.813042595	0.775	0.473	4.24E-25	Mo/MΦ	3	Maf
Lyve13	3.32E-28	0.802313267	0.583	0.236	6.63E-25	Mo/MΦ	3	Lyve1
Mgl22	7.87E-28	0.681331651	0.589	0.25	1.57E-24	Mo/MΦ	3	Mgl2
Clec10a2	2.89E-27	0.73929908	0.523	0.178	5.78E-24	Mo/MΦ	3	Clec10a
Trf2	3.80E-26	0.545014329	0.715	0.33	7.61E-23	Mo/MΦ	3	Trf
Cybb3	4.54E-26	0.605973241	0.636	0.313	9.07E-23	Mo/MΦ	3	Cybb
Cd1632	2.95E-25	0.795100413	0.536	0.225	5.89E-22	Mo/MΦ	3	Cd163
Ms4a71	1.59E-24	0.451337798	0.43	0.157	3.19E-21	Mo/MΦ	3	Ms4a7
Pltp3	3.30E-24	0.720861999	0.709	0.406	6.59E-21	Mo/MΦ	3	Pltp
Ccl241	5.32E-23	0.529232709	0.536	0.235	1.06E-19	Mo/MΦ	3	Ccl24
Laptm52	8.79E-23	0.436001702	0.821	0.427	1.76E-19	Mo/MΦ	3	Laptm5
Ucp23	1.83E-22	0.427697819	0.834	0.439	3.66E-19	Mo/MΦ	3	Ucp2
Cfp2	2.37E-22	0.595943477	0.563	0.25	4.74E-19	Mo/MΦ	3	Cfp
Pld42	2.57E-22	0.527490602	0.603	0.29	5.14E-19	Mo/MΦ	3	Pld4
Mafb2	3.33E-22	0.553731246	0.649	0.332	6.65E-19	Mo/MΦ	3	Mafb
Fcgr2b2	7.08E-22	0.611898541	0.563	0.249	1.42E-18	Mo/MΦ	3	Fcgr2b
Itgam1	8.39E-22	0.442089557	0.437	0.159	1.68E-18	Mo/MΦ	3	Itgam
Lst11	1.95E-21	0.625818978	0.603	0.324	3.89E-18	Mo/MΦ	3	Lst1
Rgs102	2.26E-21	0.558618521	0.728	0.426	4.52E-18	Mo/MΦ	3	Rgs10
Tgfbi2	4.01E-21	0.581047021	0.762	0.463	8.02E-18	Mo/MΦ	3	Tgfbi
Fcna1	4.49E-21	0.679246788	0.517	0.264	8.99E-18	Mo/MΦ	3	Fcna
Ifi27l2a4	6.38E-21	0.583488455	0.967	0.768	1.28E-17	Mo/MΦ	3	Ifi27l2a
Ptpn182	9.19E-21	0.38206681	0.828	0.448	1.84E-17	Mo/MΦ	3	Ptpn18
C5ar12	1.29E-20	0.447990135	0.464	0.178	2.58E-17	Mo/MΦ	3	C5ar1
Spi12	2.60E-20	0.46405033	0.609	0.298	5.21E-17	Mo/MΦ	3	Spi1
Ccl81	2.94E-20	0.950511406	0.483	0.216	5.88E-17	Mo/MΦ	3	Ccl8
Ltc4s1	3.38E-20	0.678994455	0.523	0.227	6.76E-17	Mo/MΦ	3	Ltc4s
C3ar11	4.46E-20	0.503936985	0.43	0.174	8.92E-17	Mo/MΦ	3	C3ar1
Ctss2	6.04E-20	0.440164339	0.742	0.416	1.21E-16	Mo/MΦ	3	Ctss
Adgre12	7.91E-20	0.486472953	0.464	0.209	1.58E-16	Mo/MΦ	3	Adgre1
Cd209f2	8.76E-20	0.815029793	0.45	0.216	1.75E-16	Mo/MΦ	3	Cd209f
Fcrls2	8.93E-20	0.487134239	0.464	0.214	1.79E-16	Mo/MΦ	3	Fcrls
Cd209g1	1.68E-19	0.578658762	0.397	0.17	3.36E-16	Mo/MΦ	3	Cd209g
Cd682	3.22E-19	0.511882759	0.51	0.245	6.45E-16	Mo/MΦ	3	Cd68
Unc93b11	4.02E-19	0.484563609	0.709	0.429	8.05E-16	Mo/MΦ	3	Unc93b1
Clec4a11	1.12E-18	0.330541747	0.45	0.214	2.24E-15	Mo/MΦ	3	Clec4a1
Ninj12	2.94E-18	0.565165036	0.669	0.417	5.87E-15	Mo/MΦ	3	Ninj1
Ccl121	4.28E-18	0.687056365	0.325	0.13	8.56E-15	Mo/MΦ	3	Ccl12
Aif12	2.92E-17	0.520393124	0.53	0.272	5.83E-14	Mo/MΦ	3	Aif1
Cbr23	5.99E-17	0.396533859	0.675	0.401	1.20E-13	Mo/MΦ	3	Cbr2
Trem21	8.62E-17	0.324590771	0.318	0.122	1.72E-13	Mo/MΦ	3	Trem2
P2ry62	3.80E-16	0.462215884	0.543	0.291	7.61E-13	Mo/MΦ	3	P2ry6

Gmfg3	4.01E-16	0.262007065	0.715	0.399	8.01E-13	Mo/MΦ	3	Gmfg
Ednrb3	4.03E-16	0.434846479	0.417	0.206	8.06E-13	Mo/MΦ	3	Ednrb
C4b3	4.29E-16	0.537232471	0.603	0.342	8.58E-13	Mo/MΦ	3	C4b
Mpeg12	6.04E-16	0.380113045	0.464	0.254	1.21E-12	Mo/MΦ	3	Mpeg1
Timd41	8.25E-16	0.271478959	0.391	0.189	1.65E-12	Mo/MΦ	3	Timd4
Retnla2	8.95E-16	0.661928336	0.808	0.643	1.79E-12	Mo/MΦ	3	Retnla
AI6078733	1.18E-15	0.438089346	0.411	0.195	2.36E-12	Mo/MΦ	3	AI607873
Igfbp45	1.57E-15	0.329974103	0.974	0.753	3.14E-12	Mo/MΦ	3	Igfbp4
Tmem176b4	1.97E-15	0.46254106	0.728	0.437	3.93E-12	Mo/MΦ	3	Tmem176b
Clec4a22	2.65E-15	0.360590958	0.424	0.21	5.30E-12	Mo/MΦ	3	Clec4a2
Fyb2	2.81E-15	0.396700344	0.497	0.263	5.62E-12	Mo/MΦ	3	Fyb
Cd141	3.31E-15	0.349298354	0.391	0.203	6.63E-12	Mo/MΦ	3	Cd14
Stard82	3.65E-15	0.578960033	0.49	0.298	7.29E-12	Mo/MΦ	3	Stard8
Igf13	4.66E-15	0.423694072	0.762	0.483	9.33E-12	Mo/MΦ	3	Igf1
H2-Ab12	8.30E-15	0.294074055	0.881	0.69	1.66E-11	Mo/MΦ	3	H2-Ab1
Lair11	1.19E-14	0.324776091	0.344	0.135	2.38E-11	Mo/MΦ	3	Lair1
Ifi2043	1.27E-14	0.320293249	0.45	0.218	2.54E-11	Mo/MΦ	3	Ifi204
Gas67	1.54E-14	0.513883035	0.815	0.566	3.07E-11	Mo/MΦ	3	Gas6
Evi2a1	1.84E-14	0.38151371	0.556	0.364	3.69E-11	Mo/MΦ	3	Evi2a
Cfh4	2.50E-14	0.431478032	0.848	0.602	5.01E-11	Mo/MΦ	3	Cfh
Pla2g71	2.77E-14	0.291537761	0.358	0.16	5.54E-11	Mo/MΦ	3	Pla2g7
Ptafr1	7.54E-14	0.427203213	0.464	0.262	1.51E-10	Mo/MΦ	3	Ptafr
Kctd125	8.60E-14	0.346550921	0.589	0.36	1.72E-10	Mo/MΦ	3	Kctd12
Gda2	1.15E-13	0.272856668	0.51	0.269	2.31E-10	Mo/MΦ	3	Gda
Ms4a6b1	1.74E-13	0.311446329	0.391	0.194	3.49E-10	Mo/MΦ	3	Ms4a6b
Slc6a61	1.92E-13	0.438364229	0.669	0.476	3.85E-10	Mo/MΦ	3	Slc6a6
Dpt4	3.62E-13	0.294833615	0.934	0.621	7.25E-10	Mo/MΦ	3	Dpt
H2-Aa2	3.69E-13	0.298290855	0.854	0.683	7.39E-10	Mo/MΦ	3	H2-Aa
Tmem176a3	6.43E-13	0.387780527	0.589	0.345	1.29E-09	Mo/MΦ	3	Tmem176a
Rassf42	6.77E-13	0.328227834	0.49	0.298	1.35E-09	Mo/MΦ	3	Rassf4
Clec4a31	9.80E-13	0.312680963	0.404	0.232	1.96E-09	Mo/MΦ	3	Clec4a3
H2-Eb12	1.24E-12	0.282103654	0.808	0.602	2.47E-09	Mo/MΦ	3	H2-Eb1
Hpgd1	3.08E-12	0.446156925	0.457	0.286	6.15E-09	Mo/MΦ	3	Hpgd
Lgmn3	3.67E-12	0.514260834	0.629	0.464	7.33E-09	Mo/MΦ	3	Lgmn
Pirb1	5.25E-12	0.306906825	0.384	0.215	1.05E-08	Mo/MΦ	3	Pirb
Hbb-bs3	1.14E-11	0.459983726	0.868	0.718	2.29E-08	Mo/MΦ	3	Hbb-bs
Csf2ra2	1.90E-11	0.310852919	0.444	0.287	3.80E-08	Mo/MΦ	3	Csf2ra
Cst34	2.27E-11	0.255393006	0.993	0.957	4.54E-08	Mo/MΦ	3	Cst3
Sdc32	5.92E-11	0.355521871	0.444	0.284	1.18E-07	Mo/MΦ	3	Sdc3
Abca8a4	8.32E-11	0.37588833	0.642	0.408	1.66E-07	Mo/MΦ	3	Abca8a
Nrp23	9.65E-11	0.354928469	0.371	0.229	1.93E-07	Mo/MΦ	3	Nrp2
Lcp12	2.57E-10	0.266675607	0.623	0.427	5.14E-07	Mo/MΦ	3	Lcp1
Emp14	2.65E-10	0.286833219	0.662	0.414	5.29E-07	Mo/MΦ	3	Emp1
Cygb3	6.70E-10	0.305126391	0.629	0.414	1.34E-06	Mo/MΦ	3	Cygb
Cd331	8.31E-10	0.31881283	0.338	0.189	1.66E-06	Mo/MΦ	3	Cd33
Mmp24	1.12E-09	0.263731494	0.781	0.593	2.23E-06	Mo/MΦ	3	Mmp2
Pla2g2d1	4.58E-09	0.309081669	0.298	0.154	9.15E-06	Mo/MΦ	3	Pla2g2d
Cx3cr11	5.19E-09	0.467340424	0.285	0.172	1.04E-05	Mo/MΦ	3	Cx3cr1
Hba-a12	7.10E-09	0.43302934	0.742	0.56	1.42E-05	Mo/MΦ	3	Hba-a1
Ly862	1.06E-08	0.304877363	0.517	0.371	2.12E-05	Mo/MΦ	3	Ly86
Ccl71	1.17E-08	0.527560285	0.265	0.166	2.33E-05	Mo/MΦ	3	Ccl7
Bst24	1.44E-08	0.31787973	0.682	0.495	2.87E-05	Mo/MΦ	3	Bst2
Marcksl11	1.57E-08	0.321686476	0.483	0.356	3.14E-05	Mo/MΦ	3	Marcksl1
Igfbp53	3.29E-08	0.393273952	0.695	0.537	6.57E-05	Mo/MΦ	3	Igfbp5
Batf32	6.94E-08	0.271865683	0.371	0.264	0.000138733	Mo/MΦ	3	Batf3
Cd482	7.41E-08	0.285318238	0.457	0.329	0.000148257	Mo/MΦ	3	Cd48
Ccnd12	7.67E-08	0.250847759	0.404	0.288	0.000153463	Mo/MΦ	3	Ccnd1
Clec4b12	7.88E-08	0.258550217	0.318	0.226	0.000157642	Mo/MΦ	3	Clec4b1
Hba-a22	1.08E-07	0.434317234	0.669	0.517	0.000215355	Mo/MΦ	3	Hba-a2
Hpgds1	1.36E-07	0.383239226	0.305	0.171	0.000271333	Mo/MΦ	3	Hpgds
Hmox11	2.35E-07	0.337848793	0.377	0.277	0.000469334	Mo/MΦ	3	Hmox1
C1s14	2.92E-07	0.289463123	0.662	0.483	0.000584218	Mo/MΦ	3	C1s1
Lgals32	3.62E-07	0.341375169	0.47	0.343	0.000724446	Mo/MΦ	3	Lgals3
Smoc23	4.12E-07	0.271349253	0.497	0.375	0.000824005	Mo/MΦ	3	Smoc2
Ccdc109b2	4.54E-07	0.278380323	0.384	0.247	0.000907721	Mo/MΦ	3	Ccdc109b
Mt12	4.95E-07	0.260592807	0.907	0.757	0.000990696	Mo/MΦ	3	Mt1
Mmp92	5.72E-07	0.337140138	0.311	0.216	0.001144858	Mo/MΦ	3	Mmp9
Cebpa1	2.21E-06	0.274884139	0.351	0.269	0.004429348	Mo/MΦ	3	Cebpa
Klf43	3.18E-06	0.253177532	0.583	0.439	0.00635356	Mo/MΦ	3	Klf4
Actb5	5.83E-06	0.285792976	1	0.994	0.011653697	Mo/MΦ	3	Actb
Emilin21	2.11E-05	0.307502425	0.444	0.354	0.042259864	Mo/MΦ	3	Emilin2
Wisp22	3.23E-05	0.285482563	0.338	0.278	0.064674551	Mo/MΦ	3	Wisp2
Cxcl161	0.000188868	0.254757355	0.371	0.31	0.377736603	Mo/MΦ	3	Cxcl16
Oasl22	0.000511034	0.26579218	0.298	0.252	1	Mo/MΦ	3	Oasl2
Cxcl124	0.000688079	0.253089792	0.464	0.394	1	Mo/MΦ	3	Cxcl12
F13a13	4.03E-37	1.360515812	0.932	0.266	8.06E-34	Mo/MΦ	4	F13a1
C1qa2	6.99E-36	1.305949877	0.986	0.454	1.40E-32	Mo/MΦ	4	C1qa
Folr22	2.52E-34	1.310923249	0.892	0.254	5.04E-31	Mo/MΦ	4	Folr2
C1qc3	3.33E-34	1.234145705	0.973	0.39	6.66E-31	Mo/MΦ	4	C1qc
Pf42	1.90E-33	1.290972679	0.973	0.431	3.80E-30	Mo/MΦ	4	Pf4
Lyz23	3.56E-32	1.16870219	1	0.648	7.12E-29	Mo/MΦ	4	Lyz2
Mrc14	4.04E-31	1.112409271	0.865	0.292	8.08E-28	Mo/MΦ	4	Mrc1
Dab23	2.94E-30	1.168023823	0.905	0.392	5.87E-27	Mo/MΦ	4	Dab2
Stab15	1.52E-29	1.133784044	0.811	0.225	3.05E-26	Mo/MΦ	4	Stab1
Ctsc4	4.32E-29	1.091149272	0.878	0.344	8.65E-26	Mo/MΦ	4	Ctsc
C1qb2	7.31E-29	1.165846944	0.919	0.408	1.46E-25	Mo/MΦ	4	C1qb
Fcer1g3	1.02E-27	0.8740251	0.959	0.379	2.05E-24	Mo/MΦ	4	Fcer1g
Pltp4	4.01E-27	1.090281392	0.878	0.407	8.02E-24	Mo/MΦ	4	Pltp
Csf1r3	1.47E-26	1.233995818	0.811	0.28	2.93E-23	Mo/MΦ	4	Csf1r
Wfdc172	1.28E-25	1.151704158	0.865	0.357	2.55E-22	Mo/MΦ	4	Wfdc17
Apoe4	1.63E-25	1.085653455	0.986	0.843	3.27E-22	Mo/MΦ	4	Apoe
Ptpn183	4.01E-25	0.95288172	0.932	0.449	8.02E-22	Mo/MΦ	4	Ptpn18
Trf3	4.17E-25	0.899334246	0.865	0.331	8.35E-22	Mo/MΦ	4	Trf
Sepp14	4.92E-25	1.019044997	0.973	0.823	9.84E-22	Mo/MΦ	4	Sepp1
Ccl93	3.08E-24	0.966960132	0.811	0.288	6.16E-21	Mo/MΦ	4	Ccl9
Ctss3	3.23E-24	0.882435267	0.919	0.416	6.46E-21	Mo/MΦ	4	Ctss
Ptprcap	5.11E-24	0.710740364	0.905	0.452	1.02E-20	Mo/MΦ	4	Ptprcap
Ucp24	1.16E-23	0.923366461	0.919	0.44	2.32E-20	Mo/MΦ	4	Ucp2
Cd1633	1.91E-23	1.173890249	0.689	0.226	3.81E-20	Mo/MΦ	4	Cd163
Lcp13	2.13E-23	0.873106361	0.878	0.427	4.27E-20	Mo/MΦ	4	Lcp1
Cd521	8.42E-23	0.825150905	1	0.625	1.68E-19	Mo/MΦ	4	Cd52
Cd37	1.30E-22	0.894306429	0.878	0.487	2.61E-19	Mo/MΦ	4	Cd37
Cbr24	1.74E-22	0.754737614	0.878	0.401	3.48E-19	Mo/MΦ	4	Cbr2
Fxyd22	2.19E-22	1.143687637	0.73	0.276	4.38E-19	Mo/MΦ	4	Fxyd2
Ccl62	3.69E-22	0.977053331	0.824	0.373	7.38E-19	Mo/MΦ	4	Ccl6
Laptm53	4.53E-22	0.892563636	0.878	0.428	9.06E-19	Mo/MΦ	4	Laptm5
Maf4	6.99E-22	0.951806247	0.851	0.474	1.40E-18	Mo/MΦ	4	Maf
Lyve14	8.86E-22	1.026479062	0.676	0.236	1.77E-18	Mo/MΦ	4	Lyve1
Tyrobp3	2.52E-21	0.843837423	0.851	0.371	5.04E-18	Mo/MΦ	4	Tyrobp
Fcgr33	3.03E-21	0.852326924	0.703	0.249	6.07E-18	Mo/MΦ	4	Fcgr3
Ltc4s2	5.55E-21	0.893802414	0.662	0.228	1.11E-17	Mo/MΦ	4	Ltc4s
Mgl23	5.84E-21	0.888770033	0.689	0.251	1.17E-17	Mo/MΦ	4	Mgl2
Cd364	1.28E-20	0.779318377	0.73	0.256	2.57E-17	Mo/MΦ	4	Cd36
Fcgr2b3	1.37E-20	0.899919032	0.689	0.25	2.74E-17	Mo/MΦ	4	Fcgr2b
H2-Aa3	4.04E-20	0.852528061	0.959	0.683	8.09E-17	Mo/MΦ	4	H2-Aa
Cd742	6.29E-20	0.753372898	1	0.861	1.26E-16	Mo/MΦ	4	Cd74
Cfp3	3.12E-19	0.768219002	0.689	0.251	6.25E-16	Mo/MΦ	4	Cfp
Rac21	2.27E-18	0.701034331	0.878	0.5	4.53E-15	Mo/MΦ	4	Rac2
Coro1a1	3.20E-18	0.722925025	0.919	0.539	6.41E-15	Mo/MΦ	4	Coro1a
Ccl242	4.16E-18	0.967814108	0.608	0.235	8.31E-15	Mo/MΦ	4	Ccl24
Ninj13	8.27E-18	0.728374732	0.784	0.417	1.65E-14	Mo/MΦ	4	Ninj1
H2-Eb13	8.31E-18	0.735738033	0.932	0.602	1.66E-14	Mo/MΦ	4	H2-Eb1
Sh3bgrl32	1.21E-17	0.687127869	0.973	0.812	2.42E-14	Mo/MΦ	4	Sh3bgrl3
H2-Ab13	1.81E-17	0.651539202	0.959	0.69	3.61E-14	Mo/MΦ	4	H2-Ab1
Unc93b12	3.20E-17	0.898693479	0.77	0.43	6.40E-14	Mo/MΦ	4	Unc93b1
Gmfg4	6.95E-17	0.694509228	0.797	0.4	1.39E-13	Mo/MΦ	4	Gmfg
Rgs103	1.42E-16	0.749610334	0.757	0.427	2.84E-13	Mo/MΦ	4	Rgs10
Cotl12	2.23E-16	0.691273994	0.851	0.506	4.46E-13	Mo/MΦ	4	Cotl1
Limd2	6.39E-16	0.685261154	0.811	0.505	1.28E-12	Mo/MΦ	4	Limd2

Ly6e5	7.14E-16	0.629528393	0.986	0.795	1.43E-12	Mo/MΦ_4	Ly6e
Pld43	9.28E-16	0.730921242	0.649	0.291	1.86E-12	Mo/MΦ_4	Pld4
Arhgdib2	1.11E-15	0.628581505	0.892	0.557	2.23E-12	Mo/MΦ_4	Arhgdib
Clec10a3	2.01E-15	0.825692695	0.486	0.18	4.02E-12	Mo/MΦ_4	Clec10a
Alox5ap3	3.46E-15	0.579925354	0.662	0.282	6.91E-12	Mo/MΦ_4	Alox5ap
Fcna2	3.57E-15	0.915534934	0.568	0.264	7.14E-12	Mo/MΦ_4	Fcna
C5ar13	4.04E-15	0.621774633	0.527	0.179	8.09E-12	Mo/MΦ_4	C5ar1
Ltb	4.05E-15	0.684025084	0.757	0.442	8.11E-12	Mo/MΦ_4	Ltb
Cd531	4.54E-15	0.674243538	0.77	0.445	9.08E-12	Mo/MΦ_4	Cd53
Cd79a	1.51E-14	0.791479164	0.77	0.5	3.03E-11	Mo/MΦ_4	Cd79a
Lgmn4	2.14E-14	0.751165706	0.73	0.464	4.27E-11	Mo/MΦ_4	Lgmn
Pou2f22	3.42E-14	0.806863935	0.649	0.348	6.84E-11	Mo/MΦ_4	Pou2f2
P2ry63	5.79E-14	0.742327039	0.608	0.291	1.16E-10	Mo/MΦ_4	P2ry6
B2m3	7.23E-14	0.365836649	1	0.983	1.45E-10	Mo/MΦ_4	B2m
Ly6d	9.01E-14	0.805892056	0.703	0.418	1.80E-10	Mo/MΦ_4	Ly6d
Ms4a1	1.72E-13	0.719458715	0.662	0.37	3.44E-10	Mo/MΦ_4	Ms4a1
Cybb4	1.97E-13	0.726047745	0.622	0.314	3.94E-10	Mo/MΦ_4	Cybb
H2-K14	2.34E-13	0.481677247	0.973	0.802	4.68E-10	Mo/MΦ_4	H2-K1
Ptprc2	2.34E-13	0.784129322	0.703	0.437	4.69E-10	Mo/MΦ_4	Ptprc
Fyb3	2.48E-13	0.630218101	0.568	0.263	4.95E-10	Mo/MΦ_4	Fyb
Ednrb4	5.04E-13	0.595219466	0.473	0.207	1.01E-09	Mo/MΦ_4	Ednrb
H2-D15	5.30E-13	0.442008218	1	0.89	1.06E-09	Mo/MΦ_4	H2-D1
Ccl82	9.66E-13	0.650587069	0.486	0.217	1.93E-09	Mo/MΦ_4	Ccl8
Ms4a6c4	1.08E-12	0.584160038	0.514	0.199	2.15E-09	Mo/MΦ_4	Ms4a6c
Spi13	1.15E-12	0.632992942	0.608	0.299	2.29E-09	Mo/MΦ_4	Spi1
Aif13	1.87E-12	0.572504537	0.581	0.272	3.75E-09	Mo/MΦ_4	Aif1
C4b4	2.00E-12	0.586093524	0.649	0.343	4.01E-09	Mo/MΦ_4	C4b
Mafb3	2.11E-12	0.642964804	0.622	0.333	4.22E-09	Mo/MΦ_4	Mafb
H2-DMb21	2.68E-12	0.661019845	0.662	0.39	5.36E-09	Mo/MΦ_4	H2-DMb2
Pla2g72	6.52E-12	0.56131765	0.405	0.161	1.30E-08	Mo/MΦ_4	Pla2g7
Cd79b1	6.62E-12	0.633053289	0.703	0.459	1.32E-08	Mo/MΦ_4	Cd79b
Ptpn61	8.00E-12	0.624931617	0.649	0.389	1.60E-08	Mo/MΦ_4	Ptpn6
H2-DMa2	9.54E-12	0.477201934	0.73	0.418	1.91E-08	Mo/MΦ_4	H2-DMa
Ptafr2	1.07E-11	0.536120009	0.527	0.262	2.15E-08	Mo/MΦ_4	Ptafr
Stard83	1.37E-11	0.735981083	0.541	0.298	2.75E-08	Mo/MΦ_4	Stard8
Cd209f3	1.99E-11	0.87858272	0.446	0.217	3.98E-08	Mo/MΦ_4	Cd209f
Clec4a12	2.09E-11	0.469629091	0.459	0.215	4.17E-08	Mo/MΦ_4	Clec4a1
Fcrls3	3.04E-11	0.437555128	0.459	0.215	6.08E-08	Mo/MΦ_4	Fcrls
Ehd44	3.45E-11	0.585832881	0.649	0.387	6.90E-08	Mo/MΦ_4	Ehd4
Pirb2	4.28E-11	0.594409517	0.459	0.215	8.57E-08	Mo/MΦ_4	Pirb
Lair12	4.77E-11	0.492456238	0.365	0.136	9.54E-08	Mo/MΦ_4	Lair1
Mndal2	5.25E-11	0.54152509	0.622	0.352	1.05E-07	Mo/MΦ_4	Mndal
Tmsb4x7	6.47E-11	0.296543238	1	1	1.29E-07	Mo/MΦ_4	Tmsb4x
Ms4a6d1	6.80E-11	0.412023056	0.297	0.107	1.36E-07	Mo/MΦ_4	Ms4a6d
Ms4a72	1.30E-10	0.485086785	0.378	0.158	2.59E-07	Mo/MΦ_4	Ms4a7
Retnla3	1.35E-09	1.123129303	0.757	0.644	2.70E-06	Mo/MΦ_4	Retnla
Apobec31	1.76E-09	0.610901927	0.608	0.395	3.53E-06	Mo/MΦ_4	Apobec3
Klf23	2.02E-09	0.404990056	0.838	0.564	4.03E-06	Mo/MΦ_4	Klf2
Mzb1	3.09E-09	0.346359016	0.473	0.263	6.19E-06	Mo/MΦ_4	Mzb1
Cd683	3.14E-09	0.506232187	0.446	0.246	6.28E-06	Mo/MΦ_4	Cd68
Gimap12	3.21E-09	0.537507492	0.595	0.379	6.41E-06	Mo/MΦ_4	Gimap1
Ms4a6b2	3.82E-09	0.423677189	0.405	0.194	7.65E-06	Mo/MΦ_4	Ms4a6b
Cd483	4.41E-09	0.549150658	0.541	0.33	8.83E-06	Mo/MΦ_4	Cd48
Adgre13	4.53E-09	0.584912064	0.419	0.21	9.06E-06	Mo/MΦ_4	Adgre1
Itgam2	6.78E-09	0.458697231	0.351	0.16	1.36E-05	Mo/MΦ_4	Itgam
C3ar12	6.88E-09	0.446455488	0.378	0.175	1.38E-05	Mo/MΦ_4	C3ar1
Fcmr	7.25E-09	0.671664966	0.595	0.41	1.45E-05	Mo/MΦ_4	Fcmr
Selplg2	1.06E-08	0.472825521	0.5	0.296	2.11E-05	Mo/MΦ_4	Selplg
Cd382	1.11E-08	0.389184626	0.378	0.208	2.22E-05	Mo/MΦ_4	Cd38
Hmox12	1.13E-08	0.533212454	0.459	0.278	2.27E-05	Mo/MΦ_4	Hmox1
Smagp3	1.79E-08	0.438799267	0.419	0.238	3.58E-05	Mo/MΦ_4	Smagp
Bank1	3.18E-08	0.666751506	0.527	0.351	6.35E-05	Mo/MΦ_4	Bank1
Pla2g2d2	3.56E-08	0.657210007	0.351	0.154	7.13E-05	Mo/MΦ_4	Pla2g2d
Cd142	4.70E-08	0.483413349	0.351	0.203	9.41E-05	Mo/MΦ_4	Cd14
S1pr13	5.23E-08	0.500431563	0.473	0.296	0.000104643	Mo/MΦ_4	S1pr1
Marcksl12	7.59E-08	0.498412966	0.541	0.356	0.000151894	Mo/MΦ_4	Marcksl1
Irf82	1.12E-07	0.454199057	0.514	0.339	0.000223535	Mo/MΦ_4	Irf8
Srgn3	1.15E-07	0.303788052	0.716	0.498	0.000230608	Mo/MΦ_4	Srgn
Tgfbi3	1.41E-07	0.360993969	0.689	0.464	0.000282341	Mo/MΦ_4	Tgfbi
Arhgap302	1.59E-07	0.523417463	0.486	0.299	0.000318019	Mo/MΦ_4	Arhgap30
Cd841	1.71E-07	0.553057964	0.473	0.304	0.000341561	Mo/MΦ_4	Cd84
Tmem176b5	2.16E-07	0.458064499	0.649	0.438	0.000432535	Mo/MΦ_4	Tmem176b
Tmem176a4	2.72E-07	0.362472029	0.554	0.346	0.000543554	Mo/MΦ_4	Tmem176a
Hpgd2	2.86E-07	0.474446921	0.459	0.286	0.000571073	Mo/MΦ_4	Hpgd
Cd332	2.91E-07	0.482056479	0.365	0.19	0.000582633	Mo/MΦ_4	Cd33
Gm424182	3.67E-07	0.32858491	0.973	0.824	0.000734195	Mo/MΦ_4	Gm42418
Timd42	3.95E-07	0.430351018	0.338	0.19	0.000789945	Mo/MΦ_4	Timd4
Ly863	4.39E-07	0.495797885	0.554	0.372	0.000877532	Mo/MΦ_4	Ly86
Gas68	4.49E-07	0.335465512	0.784	0.567	0.000897328	Mo/MΦ_4	Gas6
Gda3	5.10E-07	0.379115918	0.432	0.27	0.001019234	Mo/MΦ_4	Gda
Mmp93	8.19E-07	0.704778547	0.338	0.216	0.001637305	Mo/MΦ_4	Mmp9
Kctd126	9.13E-07	0.481211372	0.527	0.361	0.001825695	Mo/MΦ_4	Kctd12
AI6078734	1.08E-06	0.277429867	0.311	0.196	0.002150236	Mo/MΦ_4	AI607873
Lilrb4a1	1.10E-06	0.516342686	0.365	0.195	0.002195958	Mo/MΦ_4	Lilrb4a
Il2rg	1.22E-06	0.430470981	0.527	0.376	0.00244898	Mo/MΦ_4	Il2rg
Epsti11	1.28E-06	0.405161827	0.446	0.299	0.002564152	Mo/MΦ_4	Epsti1
Gimap62	1.54E-06	0.340512316	0.581	0.422	0.003078537	Mo/MΦ_4	Gimap6
Tnfrsf13b	1.64E-06	0.466611182	0.432	0.278	0.003274657	Mo/MΦ_4	Tnfrsf13b
Gimap43	1.93E-06	0.421223909	0.459	0.32	0.003868774	Mo/MΦ_4	Gimap4
Hpgds2	2.00E-06	0.438335395	0.311	0.172	0.003997315	Mo/MΦ_4	Hpgds
Ifi2032	2.01E-06	0.439912794	0.527	0.388	0.004018157	Mo/MΦ_4	Ifi203
H2-Ob	2.03E-06	0.68587402	0.541	0.418	0.004067841	Mo/MΦ_4	H2-Ob
Arl6ip11	3.49E-06	0.354109775	0.662	0.526	0.006980253	Mo/MΦ_4	Arl6ip1
Tmem371	5.51E-06	0.408379451	0.297	0.188	0.01102293	Mo/MΦ_4	Tmem37
Psmb83	6.07E-06	0.260800203	0.73	0.538	0.012131625	Mo/MΦ_4	Psmb8
Clec4a32	6.44E-06	0.384414209	0.351	0.233	0.012889676	Mo/MΦ_4	Clec4a3
Bcl2a1b2	6.56E-06	0.362305446	0.365	0.22	0.013126781	Mo/MΦ_4	Bcl2a1b
Ralgps2	6.61E-06	0.447383785	0.459	0.337	0.013219259	Mo/MΦ_4	Ralgps2
Slfn23	8.79E-06	0.419528937	0.419	0.299	0.017577033	Mo/MΦ_4	Slfn2
Plek2	9.88E-06	0.430506927	0.365	0.24	0.019760589	Mo/MΦ_4	Plek
Cd72	1.01E-05	0.483588652	0.392	0.275	0.02019958	Mo/MΦ_4	Cd72
Cd209g2	1.39E-05	0.434755174	0.27	0.171	0.027718517	Mo/MΦ_4	Cd209g
Dok22	2.20E-05	0.356689228	0.365	0.246	0.043995099	Mo/MΦ_4	Dok2
H2-Q73	2.26E-05	0.490994831	0.514	0.38	0.045118804	Mo/MΦ_4	H2-Q7
Lst12	2.91E-05	0.556228517	0.432	0.325	0.058172245	Mo/MΦ_4	Lst1
Hcst	4.31E-05	0.589044283	0.446	0.347	0.08618323	Mo/MΦ_4	Hcst
Lmo23	5.09E-05	0.457923349	0.527	0.425	0.101704049	Mo/MΦ_4	Lmo2
Pyhin1	6.01E-05	0.406677817	0.338	0.231	0.120213292	Mo/MΦ_4	Pyhin1
Gm43291	7.68E-05	0.359279925	0.27	0.146	0.153551536	Mo/MΦ_4	Gm43291
Siglecg	0.000122406	0.408779012	0.392	0.29	0.244812478	Mo/MΦ_4	Siglecg
Sell	0.000150422	0.449199396	0.446	0.357	0.300843718	Mo/MΦ_4	Sell
H2-Oa	0.000196909	0.575604441	0.459	0.374	0.393818887	Mo/MΦ_4	H2-Oa
Csf2ra3	0.000261237	0.286637943	0.351	0.288	0.522473928	Mo/MΦ_4	Csf2ra
Clec4a23	0.000266093	0.250231467	0.27	0.211	0.532185996	Mo/MΦ_4	Clec4a2
Acp5	0.00030801	0.364389641	0.338	0.267	0.616019781	Mo/MΦ_4	Acp5
Iqgap21	0.000322998	0.327029578	0.311	0.249	0.645996967	Mo/MΦ_4	Iqgap2
Cebpa2	0.000331244	0.352108766	0.338	0.269	0.662488921	Mo/MΦ_4	Cebpa
Cd831	0.000371782	0.410830368	0.378	0.322	0.743564374	Mo/MΦ_4	Cd83
Cd3g	0.000381187	0.48341422	0.27	0.216	0.762374329	Mo/MΦ_4	Cd3g
Rassf43	0.000391101	0.394208874	0.392	0.299	0.78220118	Mo/MΦ_4	Rassf4
Evi2a2	0.000743238	0.42276864	0.432	0.365	1	Mo/MΦ_4	Evi2a
Rasgrp32	0.000766118	0.324913064	0.324	0.252	1	Mo/MΦ_4	Rasgrp3
Clec12a2	0.000939635	0.343240269	0.284	0.217	1	Mo/MΦ_4	Clec12a
H2-DMb12	0.001056872	0.328421028	0.419	0.369	1	Mo/MΦ_4	H2-DMb1
Cd300ld1	0.001064288	0.366211602	0.257	0.164	1	Mo/MΦ_4	Cd300ld
Capg1	0.001084573	0.284762347	0.608	0.547	1	Mo/MΦ_4	Capg
Gm43603	0.001160075	0.417049565	0.351	0.265	1	Mo/MΦ_4	Gm43603
Cmah1	0.002716232	0.502468261	0.419	0.373	1	Mo/MΦ_4	Cmah
Cd24a3	0.00382639	0.306935095	0.365	0.335	1	Mo/MΦ_4	Cd24a

Itgb7	0.004935294	0.287317228	0.297	0.259	1	Mo/MΦ 4	Itgb7
Hvcn1	0.005631753	0.503158842	0.419	0.393	1	Mo/MΦ 4	Hvcn1
Serpinb1a	0.006285053	0.27986964	0.338	0.325	1	Mo/MΦ 4	Serpinb1a
Ifi302	0.006779218	0.298882778	0.446	0.419	1	Mo/MΦ 4	Ifi30
Gimap3	0.007446683	0.320337761	0.284	0.238	1	Mo/MΦ 4	Gimap3
Tnfrsf13c	0.00842546	0.310240233	0.338	0.261	1	Mo/MΦ 4	Tnfrsf13c
Igfbp54	0	1.982556582	0.885	0.507	0	Mesotheli	Igfbp5
Pi162	0	1.669024466	0.971	0.659	0	Mesotheli	Pi16
Cd2482	0	1.541424826	0.943	0.454	0	Mesotheli	Cd248
Tmem1002	0	1.50297539	0.903	0.283	0	Mesotheli	Tmem100
Pcsk62	0	1.454388413	0.867	0.385	0	Mesotheli	Pcsk6
Ugdh2	0	1.389239691	0.845	0.377	0	Mesotheli	Ugdh
Sema3c1	0	1.364698717	0.86	0.37	0	Mesotheli	Sema3c
Nid12	0	1.338084406	0.964	0.484	0	Mesotheli	Nid1
Col14a14	0	1.322251773	0.936	0.525	0	Mesotheli	Col14a1
Adamts52	0	1.320587449	0.87	0.392	0	Mesotheli	Adamts5
Fbn11	0	1.28987378	0.928	0.575	0	Mesotheli	Fbn1
Fn12	0	1.283203527	0.988	0.618	0	Mesotheli	Fn1
Igfbp62	0	1.214870504	0.96	0.545	0	Mesotheli	Igfbp6
Scara53	0	1.173558143	0.917	0.416	0	Mesotheli	Scara5
Clec3b3	0	1.172854462	0.999	0.553	0	Mesotheli	Clec3b
Lrrn4cl2	0	1.141630219	0.804	0.333	0	Mesotheli	Lrrn4cl
Gfpt22	0	1.134590256	0.754	0.246	0	Mesotheli	Gfpt2
Cd345	0	1.114943632	0.971	0.467	0	Mesotheli	Cd34
Ackr31	0	1.106309706	0.786	0.312	0	Mesotheli	Ackr3
Pcolce23	0	1.103658713	0.969	0.544	0	Mesotheli	Pcolce2
Ly6a3	0	1.094392167	0.99	0.632	0	Mesotheli	Ly6a
Fndc13	0	1.093150346	0.861	0.437	0	Mesotheli	Fndc1
Fstl14	0	1.080304184	0.977	0.611	0	Mesotheli	Fstl1
Klf44	0	1.076046139	0.819	0.406	0	Mesotheli	Klf4
Meg35	0	1.063144614	0.967	0.579	0	Mesotheli	Meg3
Mfap53	0	1.058808558	0.984	0.665	0	Mesotheli	Mfap5
Adgrd12	0	1.055920926	0.76	0.314	0	Mesotheli	Adgrd1
Dact21	0	1.049626693	0.654	0.231	0	Mesotheli	Dact2
Emilin22	0	1.036767254	0.781	0.317	0	Mesotheli	Emilin2
Thbd5	0	1.036750784	0.734	0.337	0	Mesotheli	Thbd
Ly6c14	0	1.035176481	0.936	0.375	0	Mesotheli	Ly6c1
Tppp33	0	1.023259883	0.912	0.548	0	Mesotheli	Tppp3
Anxa32	0	1.01251321	0.834	0.448	0	Mesotheli	Anxa3
Dpt5	0	0.996358994	0.994	0.589	0	Mesotheli	Dpt
Procr3	0	0.966171156	0.703	0.34	0	Mesotheli	Procr
Pla1a1	0	0.96497346	0.66	0.309	0	Mesotheli	Pla1a
Efemp12	0	0.960537496	0.825	0.34	0	Mesotheli	Efemp1
Plpp34	0	0.932328688	0.952	0.589	0	Mesotheli	Plpp3
Dpp41	0	0.885753019	0.685	0.288	0	Mesotheli	Dpp4
Thy13	0	0.860961749	0.762	0.404	0	Mesotheli	Thy1
Ifi27l2a5	0	0.854438433	0.979	0.751	0	Mesotheli	Ifi27l2a
Itih51	0	0.83041726	0.745	0.401	0	Mesotheli	Itih5
Metrnl2	0	0.80682583	0.744	0.376	0	Mesotheli	Metrnl
Fbln25	0	0.789309292	0.765	0.348	0	Mesotheli	Fbln2
Cadm31	0	0.776455517	0.632	0.243	0	Mesotheli	Cadm3
Islr4	0	0.771665637	0.87	0.561	0	Mesotheli	Islr
Efhd15	0	0.766714772	0.687	0.343	0	Mesotheli	Efhd1
Ptgs12	0	0.75303638	0.636	0.27	0	Mesotheli	Ptgs1
Aldh1a31	0	0.702530755	0.555	0.21	0	Mesotheli	Aldh1a3
S100a62	0	0.69111944	0.999	0.899	0	Mesotheli	S100a6
Igfbp46	0	0.686895399	0.978	0.735	0	Mesotheli	Igfbp4
C33	0	0.611211539	0.918	0.499	0	Mesotheli	C3
Nbl12	0	0.608796304	0.913	0.54	0	Mesotheli	Nbl1
Stmn41	0	0.521828121	0.481	0.168	0	Mesotheli	Stmn4
Col1a24	0	0.509395962	0.997	0.778	0	Mesotheli	Col1a2
Ackr1	0	0.483334837	0.493	0.179	0	Mesotheli	Ackr1
Ftl13	0	0.3984157	1	0.992	0	Mesotheli	Ftl1
Ildr2	1.03E-307	0.491904239	0.485	0.151	2.06E-304	Mesotheli	Ildr2
Gap432	4.19E-307	0.434960091	0.496	0.199	8.39E-304	Mesotheli	Gap43
Smpd31	1.08E-306	1.016292224	0.581	0.259	2.16E-303	Mesotheli	Smpd3
Tmsb102	8.96E-306	0.527525497	0.999	0.945	1.79E-302	Mesotheli	Tmsb10
Plat2	3.56E-300	0.702476232	0.715	0.394	7.12E-297	Mesotheli	Plat
Col3a14	2.93E-291	0.520940388	0.996	0.796	5.86E-288	Mesotheli	Col3a1
Col1a14	1.99E-279	0.46210809	0.984	0.715	3.98E-276	Mesotheli	Col1a1
Chst11	6.61E-275	0.601637808	0.492	0.182	1.32E-271	Mesotheli	Chst1
Anxa12	5.79E-274	0.632956621	0.876	0.584	1.16E-270	Mesotheli	Anxa1
Hbb-bs4	4.57E-267	0.555930523	0.924	0.701	9.14E-264	Mesotheli	Hbb-bs
Il333	2.22E-265	0.698775389	0.598	0.229	4.44E-262	Mesotheli	Il33
Car83	1.26E-264	0.48210528	0.507	0.223	2.52E-261	Mesotheli	Car8
Fst2	7.13E-262	0.50467998	0.584	0.265	1.43E-258	Mesotheli	Fst
Prss235	4.21E-254	0.751128278	0.763	0.468	8.42E-251	Mesotheli	Prss23
Plac82	9.00E-244	0.641412772	0.734	0.403	1.80E-240	Mesotheli	Plac8
Dpep12	1.62E-241	0.55216381	0.726	0.342	3.24E-238	Mesotheli	Dpep1
Serping14	2.68E-239	0.325385163	0.958	0.644	5.35E-236	Mesotheli	Serping1
Aebp14	2.85E-228	0.494390181	0.94	0.621	5.70E-225	Mesotheli	Aebp1
Flrt21	6.53E-224	0.667188423	0.598	0.315	1.31E-220	Mesotheli	Flrt2
Tek3	1.13E-220	0.61062172	0.564	0.291	2.26E-217	Mesotheli	Tek
Pamr12	6.09E-218	0.50219358	0.602	0.346	1.22E-214	Mesotheli	Pamr1
Col12a11	1.17E-214	0.617971872	0.539	0.309	2.34E-211	Mesotheli	Col12a1
Akap123	5.42E-213	0.563475025	0.645	0.319	1.08E-209	Mesotheli	Akap12
Lamb13	1.77E-210	0.466558077	0.569	0.308	3.54E-207	Mesotheli	Lamb1
Cd551	1.68E-204	0.671510136	0.644	0.374	3.35E-201	Mesotheli	Cd55
Dbp2	4.68E-199	0.504210007	0.786	0.576	9.36E-196	Mesotheli	Dbp
Ramp25	5.90E-198	0.438826626	0.7	0.37	1.18E-194	Mesotheli	Ramp2
C1s15	6.15E-191	0.474304729	0.763	0.459	1.23E-187	Mesotheli	C1s1
Ecscr4	2.79E-189	0.377141614	0.484	0.23	5.59E-186	Mesotheli	Ecscr
Edn11	7.45E-189	0.47263158	0.489	0.254	1.49E-185	Mesotheli	Edn1
Mt22	5.70E-187	0.61868845	0.734	0.447	1.14E-183	Mesotheli	Mt2
Mmp25	7.92E-183	0.358599927	0.841	0.572	1.58E-179	Mesotheli	Mmp2
Limch16	2.25E-182	0.363181715	0.611	0.32	4.49E-179	Mesotheli	Limch1
Gpc33	8.78E-181	0.39549578	0.673	0.418	1.76E-177	Mesotheli	Gpc3
Hba-a13	1.52E-177	0.47027294	0.819	0.538	3.04E-174	Mesotheli	Hba-a1
Qpct2	9.89E-176	0.480290335	0.674	0.445	1.98E-172	Mesotheli	Qpct
Col6a15	5.02E-173	0.44580875	0.886	0.61	1.00E-169	Mesotheli	Col6a1
Ecm13	1.58E-170	0.451187182	0.87	0.608	3.16E-167	Mesotheli	Ecm1
Hba-a23	8.53E-169	0.470836999	0.768	0.496	1.71E-165	Mesotheli	Hba-a2
Uchl13	1.21E-167	0.267041009	0.468	0.246	2.43E-164	Mesotheli	Uchl1
D630033O11Rik	9.03E-167	0.334986795	0.427	0.218	1.81E-163	Mesotheli	D630033O11Rik
Crip13	2.00E-166	0.32281968	0.997	0.965	3.99E-163	Mesotheli	Crip1
Ccl113	2.82E-163	0.681058837	0.611	0.382	5.64E-160	Mesotheli	Ccl11
Entpd23	8.93E-160	0.339192307	0.701	0.422	1.79E-156	Mesotheli	Entpd2
Abca8a5	1.76E-157	0.304925337	0.706	0.383	3.52E-154	Mesotheli	Abca8a
Aspn3	3.90E-156	0.510266466	0.686	0.409	7.80E-153	Mesotheli	Aspn
Tubb2b1	5.20E-152	0.306191778	0.446	0.234	1.04E-148	Mesotheli	Tubb2b
Sult5a12	2.24E-149	0.342437926	0.495	0.268	4.48E-146	Mesotheli	Sult5a1
Dclk13	3.51E-147	0.359285147	0.464	0.215	7.03E-144	Mesotheli	Dclk1
Cdkn1c4	1.25E-144	0.438811162	0.61	0.385	2.50E-141	Mesotheli	Cdkn1c
Pdgfra4	9.44E-144	0.356987794	0.735	0.453	1.89E-140	Mesotheli	Pdgfra
Sfrp42	1.43E-142	0.422295485	0.566	0.391	2.86E-139	Mesotheli	Sfrp4
Uap11	8.82E-142	0.650803189	0.596	0.367	1.76E-138	Mesotheli	Uap1
Hbb-bt2	8.85E-142	0.353763269	0.663	0.407	1.77E-138	Mesotheli	Hbb-bt
Dkk22	3.49E-141	0.775544615	0.501	0.298	6.98E-138	Mesotheli	Dkk2
Lsp11	2.83E-139	0.37510776	0.842	0.611	5.66E-136	Mesotheli	Lsp1
Tmem1581	4.48E-131	0.25284504	0.394	0.2	8.96E-128	Mesotheli	Tmem158
Loxl22	5.79E-128	0.454119581	0.542	0.338	1.16E-124	Mesotheli	Loxl2
Tuba1a4	2.12E-126	0.395947278	0.835	0.615	4.24E-123	Mesotheli	Tuba1a
Gstm14	5.97E-123	0.281682599	0.947	0.713	1.19E-119	Mesotheli	Gstm1
Sbsn1	1.59E-122	0.726788737	0.469	0.291	3.17E-119	Mesotheli	Sbsn
Timp36	9.62E-122	0.274718099	0.938	0.678	1.92E-118	Mesotheli	Timp3
Ifi2044	4.47E-119	0.379143162	0.446	0.199	8.94E-116	Mesotheli	Ifi204
Tnfaip22	8.21E-111	0.406301289	0.42	0.211	1.64E-107	Mesotheli	Tnfaip2
Mt13	4.30E-109	0.297510531	0.923	0.743	8.61E-106	Mesotheli	Mt1
Col6a25	2.63E-105	0.307498602	0.828	0.594	5.26E-102	Mesotheli	Col6a2

Smim11	6.41E-104	0.381424183	0.528	0.331	1.28E-100	Mesotheli	Smim1
Vcan3	1.58E-103	0.416003963	0.506	0.282	3.16E-100	Mesotheli	Vcan
Col5a24	1.49E-97	0.313909101	0.732	0.521	2.97E-94	Mesotheli	Col5a2
Scn7a3	8.23E-97	0.358373499	0.505	0.258	1.65E-93	Mesotheli	Scn7a
Mgst13	1.70E-96	0.286423233	0.753	0.524	3.40E-93	Mesotheli	Mgst1
Cmah2	4.69E-87	0.366353871	0.499	0.362	9.39E-84	Mesotheli	Cmah
Pmp223	5.57E-77	0.285708507	0.76	0.59	1.11E-73	Mesotheli	Pmp22
Fez12	7.70E-73	0.422667696	0.522	0.371	1.54E-69	Mesotheli	Fez1
Pdpn4	2.57E-72	0.422884325	0.466	0.275	5.14E-69	Mesotheli	Pdpn
Krtdap1	8.74E-69	0.506574536	0.3	0.105	1.75E-65	Mesotheli	Krtdap
Emp15	7.07E-65	0.307456662	0.58	0.4	1.41E-61	Mesotheli	Emp1
Basp1	3.05E-57	0.314182661	0.329	0.162	6.10E-54	Mesotheli	Basp1
Gda4	1.12E-28	0.273497566	0.389	0.26	2.24E-25	Mesotheli	Gda
Gm12840	5.49E-14	0.264560789	0.269	0.203	1.10E-10	Mesotheli	Gm12840
Lrrn4	0	1.700989273	0.803	0.057	0	Mesotheli	Lrrn4
Lgals2	0	0.68770683	0.578	0.032	0	Mesotheli	Lgals2
Bnc1	0	0.644472255	0.59	0.025	0	Mesotheli	Bnc1
Cldn151	1.19E-293	1.553540822	0.808	0.118	2.37E-290	Mesotheli	Cldn15
Cxadr	1.90E-292	0.927914577	0.688	0.079	3.80E-289	Mesotheli	Cxadr
Rpp25	5.24E-286	0.60377803	0.662	0.073	1.05E-282	Mesotheli	Rpp25
Upk1b	7.96E-275	1.617555128	0.791	0.153	1.59E-271	Mesotheli	Upk1b
Pcdh15	8.69E-269	0.302825489	0.408	0.007	1.74E-265	Mesotheli	Pcdh15
Slc26a3	1.07E-246	0.671660643	0.48	0.041	2.15E-243	Mesotheli	Slc26a3
Lrp2	2.49E-240	0.597801266	0.535	0.032	4.99E-237	Mesotheli	Lrp2
Chst4	8.21E-235	1.147904615	0.626	0.078	1.64E-231	Mesotheli	Chst4
Wt1	6.51E-230	1.205893774	0.741	0.113	1.30E-226	Mesotheli	Wt1
Tmem151a	1.93E-229	1.192932672	0.77	0.167	3.85E-226	Mesotheli	Tmem151a
Nkain4	7.09E-225	2.549138505	0.849	0.206	1.42E-221	Mesotheli	Nkain4
Gm128401	7.14E-221	2.230003767	0.873	0.196	1.43E-217	Mesotheli	Gm12840
Lgals7	1.60E-217	2.108745489	0.808	0.182	3.20E-214	Mesotheli	Lgals7
Myrf	5.34E-208	1.056463392	0.729	0.139	1.07E-204	Mesotheli	Myrf
Upk3b	6.29E-208	2.875220435	0.82	0.156	1.26E-204	Mesotheli	Upk3b
Lsr1	1.03E-206	0.922274514	0.698	0.118	2.06E-203	Mesotheli	Lsr
Crym	9.66E-191	0.684041712	0.607	0.082	1.93E-187	Mesotheli	Crym
Tspan8	5.10E-189	0.701285151	0.518	0.053	1.02E-185	Mesotheli	Tspan8
Krt19	1.18E-187	2.325098192	0.777	0.12	2.37E-184	Mesotheli	Krt19
Fras1	3.98E-185	0.635604137	0.619	0.138	7.95E-182	Mesotheli	Fras1
Plet1	1.62E-184	0.760366394	0.513	0.039	3.23E-181	Mesotheli	Plet1
Aldh1a2	1.39E-183	1.218037857	0.765	0.23	2.77E-180	Mesotheli	Aldh1a2
C34	5.05E-182	2.054342371	0.945	0.526	1.01E-178	Mesotheli	C3
Cyp2s1	1.39E-178	0.821641669	0.652	0.128	2.78E-175	Mesotheli	Cyp2s1
Fabp52	4.81E-178	1.579685027	0.82	0.216	9.62E-175	Mesotheli	Fabp5
Slc9a3r1	5.17E-176	1.420789301	0.837	0.31	1.03E-172	Mesotheli	Slc9a3r1
Gsta3	1.63E-173	1.204946571	0.741	0.143	3.26E-170	Mesotheli	Gsta3
Gsta4	2.56E-172	0.941523356	0.727	0.169	5.12E-169	Mesotheli	Gsta4
C2	2.64E-169	1.736794233	0.803	0.292	5.29E-166	Mesotheli	C2
Rspo11	8.15E-168	1.575984506	0.775	0.152	1.63E-164	Mesotheli	Rspo1
Trf4	2.67E-163	1.444535189	0.885	0.323	5.35E-160	Mesotheli	Trf
Gpm6a1	6.92E-160	2.284156849	0.751	0.146	1.38E-156	Mesotheli	Gpm6a
Bace22	4.38E-159	1.382281131	0.815	0.319	8.75E-156	Mesotheli	Bace2
Cldn10	1.19E-143	1.296049104	0.724	0.252	2.38E-140	Mesotheli	Cldn10
Tmem176b6	5.53E-142	1.283290946	0.921	0.43	1.11E-138	Mesotheli	Tmem176b
Fcor	1.53E-141	0.635323571	0.669	0.208	3.07E-138	Mesotheli	Fcor
Rarres24	2.23E-141	2.358311303	0.947	0.684	4.46E-138	Mesotheli	Rarres2
Igfbp63	5.83E-140	2.103148301	0.916	0.573	1.17E-136	Mesotheli	Igfbp6
Msln	2.80E-139	2.544813441	0.719	0.16	5.60E-136	Mesotheli	Msln
Smim12	6.99E-138	1.236830652	0.808	0.339	1.40E-134	Mesotheli	Smim1
Clu5	1.61E-137	1.403393163	0.868	0.375	3.23E-134	Mesotheli	Clu
Aqp14	3.95E-137	1.477360756	0.839	0.351	7.91E-134	Mesotheli	Aqp1
Fmod1	4.03E-134	0.868029979	0.758	0.297	8.06E-131	Mesotheli	Fmod
Fxyd3	5.84E-134	1.546267807	0.741	0.277	1.17E-130	Mesotheli	Fxyd3
Cybrd1	9.08E-131	0.834048954	0.53	0.103	1.82E-127	Mesotheli	Cybrd1
Tst	6.42E-128	0.851323836	0.758	0.303	1.28E-124	Mesotheli	Tst
F11r3	3.10E-127	0.794972562	0.693	0.209	6.21E-124	Mesotheli	F11r
Muc16	6.40E-121	1.022624134	0.513	0.052	1.28E-117	Mesotheli	Muc16
Ezr	3.10E-120	1.326144816	0.775	0.3	6.21E-117	Mesotheli	Ezr
Gjb5	3.02E-119	0.734672332	0.552	0.09	6.03E-116	Mesotheli	Gjb5
Mgst14	7.80E-119	1.097834079	0.926	0.536	1.56E-115	Mesotheli	Mgst1
Hspb16	8.20E-118	1.242923333	0.878	0.484	1.64E-114	Mesotheli	Hspb1
Atp1b11	2.57E-114	1.690648895	0.755	0.339	5.14E-111	Mesotheli	Atp1b1
Krt8	2.10E-113	1.114623031	0.597	0.079	4.19E-110	Mesotheli	Krt8
Tmem1193	3.91E-113	1.089827324	0.77	0.343	7.83E-110	Mesotheli	Tmem119
Dmkn	7.34E-113	1.706289419	0.621	0.13	1.47E-109	Mesotheli	Dmkn
Ptprf2	3.12E-112	1.028425773	0.693	0.216	6.25E-109	Mesotheli	Ptprf
Efemp13	7.76E-112	1.617528888	0.796	0.373	1.55E-108	Mesotheli	Efemp1
Tgm27	1.22E-111	0.911464842	0.801	0.306	2.45E-108	Mesotheli	Tgm2
Pkhd1l11	3.50E-111	0.941466328	0.506	0.032	6.99E-108	Mesotheli	Pkhd1l1
Aebp15	3.58E-110	1.255825121	0.909	0.643	7.16E-107	Mesotheli	Aebp1
Col6a5	4.43E-110	0.299934107	0.489	0.11	8.86E-107	Mesotheli	Col6a5
Igfbp55	2.43E-106	1.851924233	0.868	0.532	4.86E-103	Mesotheli	Igfbp5
Tmem176a5	4.63E-105	0.883636702	0.825	0.338	9.26E-102	Mesotheli	Tmem176a
Ppm1l	3.54E-104	0.433051562	0.554	0.15	7.08E-101	Mesotheli	Ppm1l
Krt18	6.98E-104	1.461360008	0.664	0.198	1.40E-100	Mesotheli	Krt18
Slc39a8	1.40E-102	0.983154303	0.595	0.166	2.80E-99	Mesotheli	Slc39a8
Ptgds	1.73E-102	0.393955129	0.463	0.123	3.47E-99	Mesotheli	Ptgds
Flrt22	6.71E-102	1.084712278	0.748	0.331	1.34E-98	Mesotheli	Flrt2
Krt7	1.15E-99	1.236728544	0.537	0.078	2.30E-96	Mesotheli	Krt7
H2-K15	2.06E-99	1.174043281	0.933	0.801	4.11E-96	Mesotheli	H2-K1
Gas14	2.29E-99	1.090729588	0.842	0.419	4.58E-96	Mesotheli	Gas1
Il341	3.12E-99	0.316482338	0.489	0.128	6.23E-96	Mesotheli	Il34
Pdpn5	1.70E-97	0.940191956	0.746	0.283	3.39E-94	Mesotheli	Pdpn
Cav18	9.32E-97	0.788448988	0.894	0.409	1.86E-93	Mesotheli	Cav1
Gas69	2.87E-93	1.013359581	0.918	0.562	5.75E-90	Mesotheli	Gas6
C1s16	1.51E-92	0.841243887	0.873	0.477	3.02E-89	Mesotheli	C1s1
Ildr21	4.77E-92	0.739597307	0.619	0.171	9.54E-89	Mesotheli	Ildr2
Crip14	8.95E-91	1.235985734	0.993	0.967	1.79E-87	Mesotheli	Crip1
Fgf11	4.73E-87	0.630996747	0.612	0.252	9.46E-84	Mesotheli	Fgf1
Palmd2	2.44E-86	0.383910919	0.499	0.092	4.89E-83	Mesotheli	Palmd
Gpc34	2.74E-84	1.454889908	0.775	0.433	5.48E-81	Mesotheli	Gpc3
Dpp42	2.78E-84	0.766613815	0.698	0.314	5.57E-81	Mesotheli	Dpp4
Prlr	4.01E-84	0.370049033	0.458	0.099	8.03E-81	Mesotheli	Prlr
Sbsn2	4.45E-83	0.944262227	0.674	0.299	8.90E-80	Mesotheli	Sbsn
Slco2a11	1.93E-78	0.820875107	0.595	0.187	3.87E-75	Mesotheli	Slco2a1
Sdpr4	4.30E-78	1.072516362	0.741	0.306	8.60E-75	Mesotheli	Sdpr
H2-D16	1.01E-77	0.848678613	0.969	0.889	2.02E-74	Mesotheli	H2-D1
AI661453	8.38E-76	0.704858779	0.566	0.183	1.68E-72	Mesotheli	AI661453
Fbln14	1.52E-73	0.799076148	0.89	0.512	3.04E-70	Mesotheli	Fbln1
Oasl23	9.66E-73	0.829801259	0.647	0.246	1.93E-69	Mesotheli	Oasl2
B2m4	1.66E-72	0.642153658	0.986	0.983	3.33E-69	Mesotheli	B2m
Anxa13	3.77E-72	0.708834354	0.904	0.603	7.53E-69	Mesotheli	Anxa1
Il334	1.70E-70	0.56551391	0.7	0.252	3.40E-67	Mesotheli	Il33
C1ra3	2.03E-70	0.873724557	0.777	0.459	4.07E-67	Mesotheli	C1ra
Ptgis4	2.30E-70	0.676186002	0.904	0.554	4.60E-67	Mesotheli	Ptgis
Lmo7	5.67E-70	0.480771696	0.475	0.094	1.13E-66	Mesotheli	Lmo7
Abcb1b1	1.19E-69	0.784703836	0.585	0.187	2.38E-66	Mesotheli	Abcb1b
Ldhb3	2.29E-69	0.768161081	0.695	0.318	4.58E-66	Mesotheli	Ldhb
Gbp22	2.40E-69	0.854147382	0.679	0.315	4.80E-66	Mesotheli	Gbp2
Aldh1a13	4.90E-69	0.851485894	0.695	0.299	9.81E-66	Mesotheli	Aldh1a1
Ces1d1	2.67E-68	1.026137722	0.628	0.266	5.35E-65	Mesotheli	Ces1d
Anxa23	4.51E-67	0.817813285	0.93	0.663	9.02E-64	Mesotheli	Anxa2
Serping15	1.42E-65	0.683171725	0.938	0.665	2.84E-62	Mesotheli	Serping1
Dbi1	2.02E-63	0.71995538	0.938	0.733	4.03E-60	Mesotheli	Dbi
Hdc	3.95E-62	0.459292534	0.446	0.115	7.90E-59	Mesotheli	Hdc
Pcolce5	7.72E-62	0.532374081	0.894	0.556	1.54E-58	Mesotheli	Pcolce
Bcam7	2.92E-61	0.90112467	0.717	0.324	5.85E-58	Mesotheli	Bcam
Dcn4	1.12E-60	0.589248742	0.978	0.857	2.23E-57	Mesotheli	Dcn
Serpinb6b1	1.24E-59	0.702009887	0.552	0.164	2.48E-56	Mesotheli	Serpinb6b
Cmah3	3.92E-59	0.523300811	0.676	0.368	7.84E-56	Mesotheli	Cmah
Esam4	1.32E-58	0.746924219	0.595	0.195	2.64E-55	Mesotheli	Esam

1500015O10Rik	2.21E-57	0.454844313	0.597	0.267	4.41E-54	Mesotheli	1500015O10Rik
Gm20186	4.00E-57	1.267069293	0.568	0.166	8.00E-54	Mesotheli	Gm20186
Spint26	1.42E-56	0.549330107	0.671	0.287	2.84E-53	Mesotheli	Spint2
Perp4	4.33E-56	0.487279895	0.52	0.185	8.66E-53	Mesotheli	Perp
Nbl13	1.23E-55	0.574692049	0.868	0.566	2.45E-52	Mesotheli	Nbl1
Tm4sf17	1.43E-55	0.925616697	0.806	0.514	2.86E-52	Mesotheli	Tm4sf1
Ctsl4	4.01E-53	0.585824233	0.928	0.689	8.02E-50	Mesotheli	Ctsl
Prss236	5.61E-53	0.890905779	0.758	0.488	1.12E-49	Mesotheli	Prss23
Anxa8	1.85E-51	0.513295068	0.429	0.106	3.70E-48	Mesotheli	Anxa8
Ano14	3.08E-50	0.816712252	0.595	0.241	6.16E-47	Mesotheli	Ano1
Aplp1	6.67E-50	0.629731272	0.513	0.197	1.33E-46	Mesotheli	Aplp1
Gng13	1.10E-49	0.325462953	0.374	0.067	2.19E-46	Mesotheli	Gng13
Cdh111	1.23E-49	0.661694936	0.571	0.212	2.47E-46	Mesotheli	Cdh11
C4b5	1.41E-49	0.795430683	0.679	0.338	2.81E-46	Mesotheli	C4b
Anxa33	3.37E-49	0.457261206	0.813	0.474	6.73E-46	Mesotheli	Anxa3
Slpi	3.64E-49	2.138935372	0.516	0.198	7.27E-46	Mesotheli	Slpi
Ly6e6	3.96E-49	0.498468089	0.957	0.792	7.93E-46	Mesotheli	Ly6e
Smpd32	1.79E-48	0.730277253	0.607	0.28	3.58E-45	Mesotheli	Smpd3
Fam180a	7.43E-46	0.387447543	0.472	0.155	1.49E-42	Mesotheli	Fam180a
Mbp	8.09E-46	0.277717892	0.53	0.228	1.62E-42	Mesotheli	Mbp
Xbp1	9.77E-46	0.432027711	0.782	0.45	1.95E-42	Mesotheli	Xbp1
Ptgs13	2.78E-44	0.567616155	0.607	0.295	5.56E-41	Mesotheli	Ptgs1
Cryab5	3.96E-44	0.484526605	0.827	0.529	7.93E-41	Mesotheli	Cryab
Lgals33	4.23E-44	0.442998386	0.676	0.338	8.46E-41	Mesotheli	Lgals3
Tnfrsf11b	1.40E-43	0.378061661	0.463	0.172	2.79E-40	Mesotheli	Tnfrsf11b
Htra16	4.89E-43	0.495719272	0.813	0.542	9.78E-40	Mesotheli	Htra1
Slc16a1	5.63E-43	0.844186708	0.542	0.202	1.13E-39	Mesotheli	Slc16a1
Mdk2	1.51E-42	0.319816725	0.602	0.346	3.02E-39	Mesotheli	Mdk
Slurp1	2.54E-42	0.66579695	0.312	0.051	5.09E-39	Mesotheli	Slurp1
Prr15l	3.14E-42	0.392439496	0.362	0.02	6.28E-39	Mesotheli	Prr15l
Enpp22	5.14E-42	1.345720576	0.552	0.298	1.03E-38	Mesotheli	Enpp2
Cox6b21	7.00E-42	0.505568152	0.554	0.284	1.40E-38	Mesotheli	Cox6b2
Ndrg13	1.54E-40	0.31561969	0.609	0.286	3.08E-37	Mesotheli	Ndrg1
Rasl11a4	9.26E-38	0.297994458	0.47	0.177	1.85E-34	Mesotheli	Rasl11a
Sema3c2	1.07E-37	0.440176585	0.71	0.405	2.15E-34	Mesotheli	Sema3c
Cd346	1.63E-37	0.285381726	0.875	0.502	3.27E-34	Mesotheli	Cd34
Tm4sf5	1.76E-37	0.895424887	0.417	0.051	3.52E-34	Mesotheli	Tm4sf5
Clic3	4.92E-37	0.887519523	0.393	0.024	9.85E-34	Mesotheli	Clic3
Tubb2b2	6.32E-37	0.309140534	0.499	0.247	1.26E-33	Mesotheli	Tubb2b
Cyb5r37	1.02E-36	0.425940403	0.813	0.494	2.05E-33	Mesotheli	Cyb5r3
Fbln26	1.46E-36	0.265308903	0.703	0.376	2.91E-33	Mesotheli	Fbln2
Itm2a3	1.86E-36	0.421380724	0.722	0.468	3.71E-33	Mesotheli	Itm2a
Aldoa4	5.10E-36	0.346809861	0.933	0.765	1.02E-32	Mesotheli	Aldoa
Fabp32	1.06E-35	0.351681399	0.482	0.185	2.12E-32	Mesotheli	Fabp3
Csrp24	3.06E-35	0.470619196	0.873	0.648	6.12E-32	Mesotheli	Csrp2
Adipoq	9.83E-35	0.456604843	0.424	0.199	1.97E-31	Mesotheli	Adipoq
Spink21	2.04E-33	0.379240882	0.41	0.125	4.07E-30	Mesotheli	Spink2
Cgnl12	5.74E-33	0.390013472	0.523	0.242	1.15E-29	Mesotheli	Cgnl1
Wfdc14	8.05E-33	0.639870373	0.626	0.372	1.61E-29	Mesotheli	Wfdc1
Fndc14	1.04E-32	0.396534971	0.693	0.468	2.07E-29	Mesotheli	Fndc1
Unc45b1	3.52E-32	0.642219689	0.463	0.146	7.04E-29	Mesotheli	Unc45b
Bst25	1.00E-31	0.37301526	0.758	0.491	2.00E-28	Mesotheli	Bst2
Cd2007	4.36E-31	0.429052534	0.787	0.535	8.73E-28	Mesotheli	Cd200
Prdx13	4.80E-31	0.34227767	0.935	0.796	9.60E-28	Mesotheli	Prdx1
Cxcl162	9.12E-31	0.321822793	0.568	0.305	1.82E-27	Mesotheli	Cxcl16
Cldn3	1.49E-29	0.459493308	0.453	0.195	2.99E-26	Mesotheli	Cldn3
Sox43	1.74E-29	0.573868835	0.564	0.31	3.48E-26	Mesotheli	Sox4
Ifi27l2a6	2.67E-29	0.273707442	0.918	0.767	5.34E-26	Mesotheli	Ifi27l2a
Stmn11	5.32E-29	0.341353974	0.501	0.206	1.06E-25	Mesotheli	Stmn1
Hmgn21	1.68E-28	0.355067241	0.7	0.475	3.35E-25	Mesotheli	Hmgn2
Slc23a3	2.07E-28	0.281205461	0.314	0.077	4.14E-25	Mesotheli	Slc23a3
Ifit32	8.75E-28	0.459955426	0.494	0.252	1.75E-24	Mesotheli	Ifit3
Chrdl11	1.76E-27	0.299855643	0.451	0.201	3.53E-24	Mesotheli	Chrdl1
Tubb4b3	7.58E-27	0.518793746	0.647	0.405	1.52E-23	Mesotheli	Tubb4b
H2-Q74	8.68E-26	0.343363001	0.585	0.377	1.74E-22	Mesotheli	H2-Q7
S100a63	3.62E-25	0.384824868	0.993	0.905	7.24E-22	Mesotheli	S100a6
Crim16	1.98E-24	0.5797598	0.525	0.283	3.96E-21	Mesotheli	Crim1
Arxes2	3.74E-24	0.31109435	0.432	0.18	7.49E-21	Mesotheli	Arxes2
Ccdc109b3	1.24E-23	0.263734566	0.482	0.244	2.49E-20	Mesotheli	Ccdc109b
Prr151	1.38E-23	0.351773658	0.4	0.148	2.76E-20	Mesotheli	Prr15
Hspa52	1.55E-23	0.405359878	0.787	0.59	3.11E-20	Mesotheli	Hspa5
Pdlim45	2.32E-23	0.303225721	0.671	0.43	4.64E-20	Mesotheli	Pdlim4
Tuba1b6	4.72E-23	0.331627979	0.842	0.659	9.45E-20	Mesotheli	Tuba1b
Kdr2	7.84E-23	0.54319932	0.434	0.165	1.57E-19	Mesotheli	Kdr
Rbp15	8.20E-23	0.281652156	0.856	0.633	1.64E-19	Mesotheli	Rbp1
Serpinh14	1.12E-22	0.262506174	0.911	0.669	2.25E-19	Mesotheli	Serpinh1
Cmbl	1.20E-22	0.344446474	0.451	0.25	2.39E-19	Mesotheli	Cmbl
Bok3	2.91E-22	0.254529191	0.463	0.228	5.81E-19	Mesotheli	Bok
Krt14	4.05E-22	0.411237396	0.278	0.039	8.10E-19	Mesotheli	Krt14
Osr15	1.34E-21	0.412162459	0.669	0.444	2.67E-18	Mesotheli	Osr1
AU0210921	2.32E-21	0.572570436	0.333	0.03	4.64E-18	Mesotheli	AU021092
Plpp16	4.99E-21	0.290355569	0.616	0.393	9.98E-18	Mesotheli	Plpp1
Isg152	2.76E-20	0.443402359	0.441	0.222	5.51E-17	Mesotheli	Isg15
Gstm15	8.31E-20	0.369919968	0.928	0.729	1.66E-16	Mesotheli	Gstm1
Tspan73	2.17E-19	0.26752438	0.499	0.305	4.35E-16	Mesotheli	Tspan7
Myl7	4.68E-19	0.514713842	0.544	0.331	9.36E-16	Mesotheli	Myl7
Bst1	1.18E-18	0.55026998	0.386	0.095	2.35E-15	Mesotheli	Bst1
Gatm2	4.73E-16	0.253622624	0.381	0.172	9.46E-13	Mesotheli	Gatm
Hsp90b12	5.83E-16	0.254489688	0.835	0.67	1.17E-12	Mesotheli	Hsp90b1
Adgrd13	5.75E-15	0.320436814	0.54	0.348	1.15E-11	Mesotheli	Adgrd1
Scd1	1.41E-14	0.360727277	0.676	0.486	2.82E-11	Mesotheli	Scd1
Pdia4	1.86E-14	0.288761326	0.566	0.361	3.72E-11	Mesotheli	Pdia4
Hmcn11	2.01E-14	0.418041665	0.403	0.206	4.02E-11	Mesotheli	Hmcn1
Wfdc2	4.07E-14	0.59541518	0.338	0.083	8.14E-11	Mesotheli	Wfdc2
Bex43	1.79E-13	0.424743118	0.403	0.181	3.59E-10	Mesotheli	Bex4
Ablim31	2.25E-13	0.259802057	0.343	0.122	4.50E-10	Mesotheli	Ablim3
Npr3	2.37E-13	0.292945571	0.312	0.12	4.74E-10	Mesotheli	Npr3
Lgals15	2.55E-12	0.688320718	0.945	0.895	5.09E-09	Mesotheli	Lgals1
Id12	1.60E-11	0.308445141	0.501	0.29	3.21E-08	Mesotheli	Id1
Basp11	1.95E-11	0.267735166	0.365	0.173	3.91E-08	Mesotheli	Basp1
Wnt4	1.75E-10	0.464265719	0.309	0.058	3.50E-07	Mesotheli	Wnt4
Selenbp13	3.77E-10	0.269988642	0.568	0.411	7.55E-07	Mesotheli	Selenbp1
Des3	6.45E-10	0.320877927	0.341	0.184	1.29E-06	Mesotheli	Des
Serpinb1a1	7.45E-10	0.589909485	0.482	0.322	1.49E-06	Mesotheli	Serpinb1a
Prss121	2.82E-08	0.252115045	0.412	0.25	5.63E-05	Mesotheli	Prss12
Hba-a24	5.25E-08	0.322340074	0.7	0.515	0.000105082	Mesotheli	Hba-a2
Il18r1	8.85E-08	0.342275233	0.314	0.082	0.000176912	Mesotheli	Il18r1
Hpgd3	2.24E-07	0.790963752	0.444	0.284	0.000447938	Mesotheli	Hpgd
Cxcl13	1.55E-05	1.77933919	0.252	0.138	0.030999547	Mesotheli	Cxcl13
Car3	6.60E-05	1.59587412	0.465	0.359	0.13190215	Mesotheli	Car3
Nrgn	0.000276521	0.65193298	0.4	0.279	0.553042249	Mesotheli	Nrgn
Hba-a14	0.001258609	0.311060521	0.664	0.559	1	Mesotheli	Hba-a1
Hbb-bs5	0.002108837	0.36133746	0.801	0.718	1	Mesotheli	Hbb-bs
Mt23	0.007211	0.261250755	0.559	0.47	1	Mesotheli	Mt2
Wif11	0	2.514409777	0.829	0.338	0	pericyte 1	Wif1
Chad	1.62E-280	2.883453575	0.804	0.386	3.24E-277	pericyte 1	Chad
Mgp3	1.74E-268	1.88549056	0.999	0.956	3.47E-265	pericyte 1	Mgp
Tsc22d16	9.69E-263	1.77038743	0.941	0.537	1.94E-259	pericyte 1	Tsc22d1
1500015O10Rik	9.97E-256	2.052899533	0.742	0.26	1.99E-252	pericyte 1	1500015O10Rik
Mia	2.47E-230	1.861818976	0.669	0.239	4.94E-227	pericyte 1	Mia
Cemip	1.44E-225	1.514108997	0.585	0.182	2.88E-222	pericyte 1	Cemip
S100b	1.27E-207	2.026628893	0.711	0.329	2.54E-204	pericyte 1	S100b
Id35	9.26E-192	1.250738257	0.939	0.683	1.85E-188	pericyte 1	Id3
Sparc4	5.87E-179	1.093597164	1	0.87	1.17E-175	pericyte 1	Sparc
Cdc42ep37	8.64E-178	1.138099977	0.809	0.375	1.73E-174	pericyte 1	Cdc42ep3
Col11a1	2.93E-176	1.733487669	0.626	0.283	5.85E-173	pericyte 1	Col11a1
Col8a12	3.40E-176	1.701626311	0.66	0.288	6.81E-173	pericyte 1	Col8a1
Cytl11	9.18E-173	3.547534129	0.746	0.412	1.84E-169	pericyte 1	Cytl1
Cilp21	1.78E-156	1.244083004	0.534	0.202	3.57E-153	pericyte 1	Cilp2

Cyr611	1.32E-154	1.822480362	0.65	0.278	2.64E-151	pericyte	1	Cyr61
Col8a2	5.60E-150	0.96346457	0.524	0.188	1.12E-146	pericyte	1	Col8a2
Fzd9	6.88E-149	1.163979809	0.49	0.17	1.38E-145	pericyte	1	Fzd9
Cst35	2.65E-148	0.820555009	1	0.956	5.30E-145	pericyte	1	Cst3
Col25a1	6.80E-145	0.666570626	0.307	0.063	1.36E-141	pericyte	1	Col25a1
Sox91	1.30E-144	1.294842517	0.585	0.237	2.61E-141	pericyte	1	Sox9
Id13	2.00E-143	1.111651217	0.666	0.283	4.00E-140	pericyte	1	Id1
Tmem213	8.52E-141	0.752414008	0.28	0.049	1.70E-137	pericyte	1	Tmem213
Pantr1	9.41E-139	0.804724587	0.381	0.102	1.88E-135	pericyte	1	Pantr1
Cdkn1c5	6.97E-138	1.104241237	0.723	0.394	1.39E-134	pericyte	1	Cdkn1c
Ppic3	9.68E-136	0.94720107	0.792	0.523	1.94E-132	pericyte	1	Ppic
Myoc1	3.92E-134	2.184075734	0.602	0.31	7.83E-131	pericyte	1	Myoc
Cadm1	5.00E-134	1.043415885	0.559	0.249	1.00E-130	pericyte	1	Cadm1
Dkk33	1.73E-131	0.950670324	0.697	0.349	3.46E-128	pericyte	1	Dkk3
Clu6	2.03E-129	1.276386657	0.743	0.373	4.05E-126	pericyte	1	Clu
Itga10	4.73E-129	1.079044294	0.553	0.255	9.45E-126	pericyte	1	Itga10
Col9a2	1.45E-126	1.597280055	0.506	0.213	2.89E-123	pericyte	1	Col9a2
Prg41	5.13E-126	1.396010011	0.491	0.185	1.03E-122	pericyte	1	Prg4
5033426O07Rik	5.89E-124	0.415610492	0.263	0.051	1.18E-120	pericyte	1	5033426O07Rik
Dhx58os	1.14E-122	1.355889982	0.512	0.223	2.28E-119	pericyte	1	Dhx58os
Meg36	1.73E-120	0.885057062	0.895	0.603	3.46E-117	pericyte	1	Meg3
Clec11a2	1.31E-117	0.870282273	0.611	0.319	2.62E-114	pericyte	1	Clec11a
Omd	3.50E-114	1.363518136	0.564	0.294	7.00E-111	pericyte	1	Omd
Fmod2	7.17E-113	1.75642829	0.568	0.298	1.43E-109	pericyte	1	Fmod
Mpped2	2.50E-112	1.096706525	0.545	0.279	5.01E-109	pericyte	1	Mpped2
Bgn7	3.82E-112	0.625935719	0.99	0.762	7.64E-109	pericyte	1	Bgn
4-Jun	7.13E-106	1.178290141	0.758	0.518	1.43E-102	pericyte	1	Jun
Nrep1	4.86E-100	1.025359606	0.611	0.395	9.72E-97	pericyte	1	Nrep
Rspo3	1.93E-98	1.084700955	0.516	0.246	3.86E-95	pericyte	1	Rspo3
S100a43	4.56E-98	0.881945092	0.818	0.568	9.12E-95	pericyte	1	S100a4
Lum4	5.98E-98	1.021277999	0.835	0.577	1.20E-94	pericyte	1	Lum
Ccdc804	7.74E-96	0.758570351	0.846	0.554	1.55E-92	pericyte	1	Ccdc80
Cd98	2.34E-95	0.732824481	0.902	0.643	4.68E-92	pericyte	1	Cd9
Col11a2	2.51E-95	1.320130398	0.424	0.179	5.03E-92	pericyte	1	Col11a2
Prelp5	1.33E-94	0.794263204	0.791	0.542	2.67E-91	pericyte	1	Prelp
Cpe6	2.01E-94	0.651923103	0.874	0.57	4.01E-91	pericyte	1	Cpe
Serpine25	1.61E-93	0.746550485	0.754	0.413	3.22E-90	pericyte	1	Serpine2
Rbp42	2.33E-93	0.775909613	0.576	0.279	4.67E-90	pericyte	1	Rbp4
Ctgf4	3.74E-91	0.94411167	0.485	0.223	7.49E-88	pericyte	1	Ctgf
Cdkn1a1	8.97E-90	1.010706015	0.521	0.258	1.79E-86	pericyte	1	Cdkn1a
Abi3bp5	7.31E-89	0.692883944	0.718	0.451	1.46E-85	pericyte	1	Abi3bp
Fos5	5.76E-87	1.274131948	0.718	0.502	1.15E-83	pericyte	1	Fos
Acan	3.18E-86	1.316431174	0.444	0.211	6.37E-83	pericyte	1	Acan
Mafb4	5.81E-85	1.034922201	0.58	0.327	1.16E-81	pericyte	1	Mafb
Ncmap	2.62E-84	0.754202181	0.374	0.148	5.23E-81	pericyte	1	Ncmap
Col9a3	3.64E-81	1.313347876	0.414	0.19	7.28E-78	pericyte	1	Col9a3
Sdk2	7.28E-78	0.634339937	0.371	0.158	1.46E-74	pericyte	1	Sdk2
Lmo71	7.85E-77	0.411647976	0.28	0.096	1.57E-73	pericyte	1	Lmo7
Gpm6b3	3.90E-76	0.661666121	0.512	0.28	7.80E-73	pericyte	1	Gpm6b
Fam101a	4.90E-75	0.673656817	0.347	0.137	9.80E-72	pericyte	1	Fam101a
Prnp2	1.17E-74	0.474286763	0.614	0.377	2.34E-71	pericyte	1	Prnp
Comp1	6.72E-73	1.279065859	0.43	0.238	1.34E-69	pericyte	1	Comp
Col27a1	2.79E-72	1.21864931	0.478	0.292	5.58E-69	pericyte	1	Col27a1
Lect1	2.85E-72	1.233060944	0.387	0.18	5.71E-69	pericyte	1	Lect1
Ucma	3.21E-72	2.157407611	0.453	0.251	6.42E-69	pericyte	1	Ucma
Tspan133	8.62E-72	0.79341161	0.484	0.276	1.72E-68	pericyte	1	Tspan13
Fxyd64	6.72E-71	0.574147783	0.565	0.311	1.34E-67	pericyte	1	Fxyd6
Prrx22	1.09E-68	0.729105982	0.448	0.246	2.17E-65	pericyte	1	Prrx2
3110079O15Rik	2.70E-66	2.480821427	0.451	0.267	5.40E-63	pericyte	1	3110079O15Rik
Pdzrn4	1.03E-65	1.032193074	0.369	0.17	2.07E-62	pericyte	1	Pdzrn4
Thbs13	1.89E-65	0.941253274	0.374	0.201	3.78E-62	pericyte	1	Thbs1
Pam5	2.58E-64	0.532399745	0.892	0.64	5.15E-61	pericyte	1	Pam
Mmp26	3.03E-64	0.515515073	0.788	0.589	6.06E-61	pericyte	1	Mmp2
Cpxm23	9.32E-64	0.699827888	0.447	0.268	1.86E-60	pericyte	1	Cpxm2
Tnn	3.99E-63	0.585185014	0.26	0.082	7.99E-60	pericyte	1	Tnn
Col9a1	1.23E-62	1.227323538	0.39	0.199	2.46E-59	pericyte	1	Col9a1
Hapln1	9.64E-62	0.881395394	0.387	0.196	1.93E-58	pericyte	1	Hapln1
Ppp1r1b	1.02E-61	0.705259007	0.297	0.128	2.05E-58	pericyte	1	Ppp1r1b
Crispld1	1.76E-58	0.86908763	0.463	0.293	3.53E-55	pericyte	1	Crispld1
Pla2g51	5.98E-58	0.687440587	0.297	0.118	1.20E-54	pericyte	1	Pla2g5
Cdh112	2.58E-57	0.69051553	0.378	0.213	5.15E-54	pericyte	1	Cdh11
Enho1	4.63E-57	0.701271041	0.326	0.173	9.26E-54	pericyte	1	Enho
Dbi2	1.06E-56	0.80436169	0.826	0.734	2.12E-53	pericyte	1	Dbi
Pdgfa5	2.44E-56	0.573355358	0.509	0.323	4.88E-53	pericyte	1	Pdgfa
Aqp15	3.52E-55	0.741517067	0.53	0.355	7.05E-52	pericyte	1	Aqp1
Id24	3.65E-55	0.719983803	0.721	0.512	7.30E-52	pericyte	1	Id2
Hspb17	2.14E-54	0.648359124	0.694	0.485	4.28E-51	pericyte	1	Hspb1
Ndrg14	3.30E-54	0.465920815	0.467	0.287	6.61E-51	pericyte	1	Ndrg1
Npnt4	1.32E-52	0.304589549	0.547	0.319	2.63E-49	pericyte	1	Npnt
Tmem176b7	1.51E-52	0.365917534	0.665	0.432	3.03E-49	pericyte	1	Tmem176b
Btg26	1.45E-51	1.1820083	0.638	0.497	2.90E-48	pericyte	1	Btg2
Mdk3	2.09E-50	0.685525577	0.488	0.347	4.18E-47	pericyte	1	Mdk
Hspa1a3	2.26E-50	1.036077374	0.543	0.394	4.52E-47	pericyte	1	Hspa1a
Notum	4.60E-50	0.700661257	0.298	0.133	9.20E-47	pericyte	1	Notum
Thsd44	4.69E-50	0.604092853	0.412	0.261	9.38E-47	pericyte	1	Thsd4
Aspn4	1.11E-49	0.701638864	0.599	0.427	2.22E-46	pericyte	1	Aspn
Phlda14	2.89E-49	0.586823747	0.577	0.393	5.77E-46	pericyte	1	Phlda1
Serpinh15	1.99E-48	0.390464232	0.889	0.667	3.97E-45	pericyte	1	Serpinh1
Egr13	9.46E-48	0.825343254	0.585	0.416	1.89E-44	pericyte	1	Egr1
Fibin2	4.02E-47	0.541095925	0.469	0.318	8.05E-44	pericyte	1	Fibin
Ier23	4.91E-47	1.072477848	0.662	0.557	9.81E-44	pericyte	1	Ier2
Fn13	6.79E-46	0.734400043	0.794	0.644	1.36E-42	pericyte	1	Fn1
Dusp16	4.08E-45	0.913031493	0.629	0.494	8.16E-42	pericyte	1	Dusp1
Fgfr21	8.27E-45	0.641265451	0.358	0.214	1.65E-41	pericyte	1	Fgfr2
Bmp2	2.05E-43	0.727246759	0.364	0.23	4.09E-40	pericyte	1	Bmp2
Mfi2	9.45E-41	0.667358639	0.272	0.128	1.89E-37	pericyte	1	Mfi2
Nr4a12	2.40E-39	0.779243437	0.504	0.381	4.81E-36	pericyte	1	Nr4a1
Itm2a4	2.42E-39	0.602334684	0.57	0.47	4.84E-36	pericyte	1	Itm2a
Fxyd31	3.45E-39	1.654394511	0.377	0.282	6.90E-36	pericyte	1	Fxyd3
Hs3st3a1	3.75E-39	0.433433963	0.283	0.149	7.50E-36	pericyte	1	Hs3st3a1
Gdf5	4.44E-39	0.779746156	0.282	0.123	8.88E-36	pericyte	1	Gdf5
A230065H16Rik	1.95E-38	0.550868905	0.264	0.128	3.89E-35	pericyte	1	A230065H16Rik
Mfap24	1.53E-37	0.732335638	0.568	0.465	3.06E-34	pericyte	1	Mfap2
Spp1	9.62E-36	1.214518978	0.306	0.171	1.92E-32	pericyte	1	Spp1
Fhl21	7.42E-34	0.308685436	0.266	0.19	1.48E-30	pericyte	1	Fhl2
Col2a1	1.14E-33	2.351162379	0.408	0.306	2.28E-30	pericyte	1	Col2a1
Col6a26	1.61E-33	0.358069259	0.76	0.609	3.21E-30	pericyte	1	Col6a2
Malat12	2.73E-33	0.321207755	1	0.999	5.46E-30	pericyte	1	Malat1
Hmcn12	2.76E-33	0.490149719	0.307	0.206	5.52E-30	pericyte	1	Hmcn1
G0s24	5.26E-33	0.756253684	0.411	0.316	1.05E-29	pericyte	1	G0s2
Fam180a1	9.39E-33	0.364456692	0.273	0.158	1.88E-29	pericyte	1	Fam180a
Tmem21	4.79E-32	0.481797905	0.318	0.237	9.57E-29	pericyte	1	Tmem2
Col6a16	6.60E-32	0.307558659	0.788	0.628	1.32E-28	pericyte	1	Col6a1
Col6a33	2.22E-31	0.426988384	0.628	0.524	4.43E-28	pericyte	1	Col6a3
Dcn5	3.25E-31	0.479709605	0.969	0.856	6.50E-28	pericyte	1	Dcn
Fxyd54	6.96E-31	0.319272704	0.605	0.455	1.39E-27	pericyte	1	Fxyd5
Col5a25	8.15E-30	0.411014001	0.645	0.536	1.63E-26	pericyte	1	Col5a2
Crim17	1.95E-29	0.431977536	0.372	0.284	3.90E-26	pericyte	1	Crim1
Slc6a62	1.39E-28	0.517262913	0.556	0.475	2.77E-25	pericyte	1	Slc6a6
Kank14	6.72E-28	0.370326132	0.429	0.322	1.34E-24	pericyte	1	Kank1
Junb3	7.37E-28	0.803484657	0.675	0.625	1.47E-24	pericyte	1	Junb
Hspa1b1	2.91E-27	0.528478591	0.381	0.303	5.83E-24	pericyte	1	Hspa1b
Cdo13	3.39E-26	0.418221633	0.295	0.218	6.78E-23	pericyte	1	Cdo1
Fgfr31	6.32E-26	0.492361407	0.276	0.197	1.26E-22	pericyte	1	Fgfr3
Wisp11	1.76E-25	0.567356749	0.303	0.209	3.52E-22	pericyte	1	Wisp1
Cyp26b12	4.46E-25	0.279643009	0.257	0.207	8.92E-22	pericyte	1	Cyp26b1
Tspan62	6.27E-25	0.579052407	0.366	0.304	1.25E-21	pericyte	1	Tspan6
Pdgfrl2	1.54E-24	0.457988904	0.444	0.374	3.08E-21	pericyte	1	Pdgfrl
Pitpnc12	3.86E-24	0.355898605	0.295	0.242	7.72E-21	pericyte	1	Pitpnc1

Dapl1	4.14E-24	0.839212625	0.263	0.171	8.29E-21	pericyte	1	Dapl1
Nbl14	6.70E-24	0.357422559	0.69	0.568	1.34E-20	pericyte	1	Nbl1
Sox44	4.74E-23	0.430629934	0.375	0.313	9.49E-20	pericyte	1	Sox4
Zfp364	5.04E-23	0.683265073	0.524	0.467	1.01E-19	pericyte	1	Zfp36
Ddit4l	2.99E-22	0.524655548	0.325	0.255	5.98E-19	pericyte	1	Ddit4l
Susd54	9.85E-22	0.330875225	0.366	0.3	1.97E-18	pericyte	1	Susd5
Lox4	7.26E-21	0.346498876	0.423	0.352	1.45E-17	pericyte	1	Lox
Tgfb2	7.41E-21	0.770685975	0.383	0.335	1.48E-17	pericyte	1	Tgfb2
Ptn2	9.38E-21	0.831328199	0.358	0.296	1.88E-17	pericyte	1	Ptn
Ppa11	7.32E-20	1.073792006	0.286	0.268	1.46E-16	pericyte	1	Ppa1
Cd2008	1.24E-19	0.280168994	0.647	0.536	2.49E-16	pericyte	1	Cd200
Apod2	1.75E-19	0.344762272	0.362	0.321	3.51E-16	pericyte	1	Apod
Col1a15	2.24E-18	0.439143543	0.767	0.737	4.49E-15	pericyte	1	Col1a1
Nrp24	3.43E-18	0.396620636	0.264	0.228	6.86E-15	pericyte	1	Nrp2
Papss22	9.93E-18	0.311716127	0.277	0.24	1.99E-14	pericyte	1	Papss2
Ctsk2	3.39E-17	0.476254039	0.475	0.449	6.78E-14	pericyte	1	Ctsk
Serpinf14	5.20E-17	0.264781534	0.669	0.589	1.04E-13	pericyte	1	Serpinf1
Nr4a21	1.09E-16	0.525881565	0.322	0.284	2.18E-13	pericyte	1	Nr4a2
Sfrp22	1.61E-16	0.287135071	0.212	0.454	3.22E-13	pericyte	1	Sfrp2
Col12a12	1.34E-15	0.555145519	0.362	0.327	2.68E-12	pericyte	1	Col12a1
Sdc13	2.02E-15	0.288264754	0.335	0.309	4.04E-12	pericyte	1	Sdc1
Angptl13	2.80E-15	0.649591986	0.372	0.313	5.61E-12	pericyte	1	Angptl1
Flrt23	4.67E-13	0.294787368	0.362	0.337	9.34E-10	pericyte	1	Flrt2
Gdf103	8.31E-13	0.555536417	0.404	0.384	1.66E-09	pericyte	1	Gdf10
Prdx6	1.46E-12	0.274199515	0.576	0.57	2.92E-09	pericyte	1	Prdx6
Enpp23	4.34E-12	0.412474003	0.316	0.302	8.68E-09	pericyte	1	Enpp2
Gpha23	4.58E-12	1.498824399	0.282	0.211	9.17E-09	pericyte	1	Gpha2
Klf24	9.88E-11	0.482296901	0.589	0.564	1.98E-07	pericyte	1	Klf2
H191	9.04E-10	0.918841442	0.273	0.262	1.81E-06	pericyte	1	H19
Thbs41	6.48E-09	0.652258525	0.171	0.287	1.30E-05	pericyte	1	Thbs4
Itih52	6.07E-07	0.291833401	0.255	0.434	0.00121357	pericyte	1	Itih5
Ptgfr3	8.17E-07	0.307740101	0.276	0.282	0.001634484	pericyte	1	Ptgfr
Gadd45a	1.75E-06	0.735384196	0.273	0.292	0.003506237	pericyte	1	Gadd45a
Dbp3	5.91E-05	0.334763126	0.545	0.595	0.118289164	pericyte	1	Dbp
Rgs21	0.000295946	0.423851096	0.295	0.347	0.591891847	pericyte	1	Rgs2
Ntrk23	0.000719687	0.314115171	0.313	0.358	1	pericyte	1	Ntrk2
Ier33	0.001226379	0.340004277	0.28	0.323	1	pericyte	1	Ier3
Gem2	0.001635608	0.428232403	0.344	0.383	1	pericyte	1	Gem
4631405K08Rik	0	1.381182707	0.906	0.004	0	pericyte	2	4631405K08Rik
BC048546	0	1.200117647	0.922	0.006	0	pericyte	2	BC048546
Sox21	0	0.985754609	0.906	0.004	0	pericyte	2	Sox21
Gm16136	0	0.813106864	0.922	0.007	0	pericyte	2	Gm16136
Cyp4a12b	0	0.593246679	0.812	0.015	0	pericyte	2	Cyp4a12b
Ugt2b34	0	0.483934125	0.859	0	0	pericyte	2	Ugt2b34
Gm29538	0	0.400532903	0.859	0.004	0	pericyte	2	Gm29538
Rsph4a	0	0.336756901	0.844	0.007	0	pericyte	2	Rsph4a
Tekt4	0	0.333473976	0.844	0.004	0	pericyte	2	Tekt4
Ccdc129	0	0.332419127	0.844	0.006	0	pericyte	2	Ccdc129
Prom2	0	0.300747119	0.844	0	0	pericyte	2	Prom2
Cdh26	1.31E-296	0.343301778	0.844	0.005	2.62E-293	pericyte	2	Cdh26
Elf3	9.35E-268	0.753875849	0.875	0.009	1.87E-264	pericyte	2	Elf3
Krt17	6.23E-248	1.094472393	0.891	0.003	1.25E-244	pericyte	2	Krt17
1700007K13Rik	1.71E-247	0.414676064	0.828	0.015	3.42E-244	pericyte	2	1700007K13Rik
Oit1	8.43E-238	1.014346843	0.906	0.019	1.69E-234	pericyte	2	Oit1
Fam183b	9.95E-236	0.655170715	0.844	0.024	1.99E-232	pericyte	2	Fam183b
AU040972	1.28E-221	0.647070596	0.844	0.021	2.56E-218	pericyte	2	AU040972
Aqp4	7.19E-207	1.215794093	0.781	0.006	1.44E-203	pericyte	2	Aqp4
1700016K19Rik	2.63E-205	0.572949374	0.844	0.034	5.27E-202	pericyte	2	1700016K19Rik
Cfap77	3.68E-202	0.260783805	0.828	0.029	7.36E-199	pericyte	2	Cfap77
Pifo	6.25E-187	0.285842502	0.844	0.038	1.25E-183	pericyte	2	Pifo
Hcar2	1.27E-173	0.289511195	0.812	0.012	2.55E-170	pericyte	2	Hcar2
Lad1	1.32E-166	0.346741176	0.828	0.024	2.65E-163	pericyte	2	Lad1
Erich2	2.60E-166	0.317448412	0.828	0.036	5.21E-163	pericyte	2	Erich2
Tmem212	3.89E-166	0.765390729	0.859	0.043	7.79E-163	pericyte	2	Tmem212
Ccdc153	1.26E-163	0.838680736	0.828	0.021	2.53E-160	pericyte	2	Ccdc153
Kcnj13	2.64E-162	0.342777675	0.844	0.045	5.28E-159	pericyte	2	Kcnj13
Myh14	1.11E-161	1.532757309	0.969	0.032	2.23E-158	pericyte	2	Myh14
Gjb3	1.29E-157	0.384587775	0.828	0.029	2.57E-154	pericyte	2	Gjb3
Arhgdig	7.48E-149	0.400050562	0.859	0.032	1.50E-145	pericyte	2	Arhgdig
Cdh1	4.61E-143	0.970757225	0.859	0.028	9.22E-140	pericyte	2	Cdh1
Sox2	2.79E-134	1.050856104	0.797	0.014	5.59E-131	pericyte	2	Sox2
Dnah5	1.16E-125	0.438954784	0.844	0.034	2.32E-122	pericyte	2	Dnah5
Dynlrb2	1.29E-117	0.73988348	0.828	0.046	2.57E-114	pericyte	2	Dynlrb2
Sult1d1	4.83E-114	2.831280942	1	0.039	9.66E-111	pericyte	2	Sult1d1
Aqp3	4.93E-113	2.250354464	0.953	0.044	9.86E-110	pericyte	2	Aqp3
Sftpa1	2.98E-111	0.575745692	0.844	0.022	5.95E-108	pericyte	2	Sftpa1
Fgfbp1	2.01E-110	0.581621598	0.828	0.04	4.02E-107	pericyte	2	Fgfbp1
Smim22	8.80E-107	1.076922692	0.938	0.038	1.76E-103	pericyte	2	Smim22
Trp63	5.44E-106	1.203564199	0.875	0.067	1.09E-102	pericyte	2	Trp63
Gdpd2	1.20E-104	1.00802885	0.906	0.06	2.40E-101	pericyte	2	Gdpd2
Gpx2	4.18E-97	1.473107635	0.891	0.025	8.37E-94	pericyte	2	Gpx2
Scnn1a	4.00E-95	1.049245759	0.906	0.097	8.00E-92	pericyte	2	Scnn1a
Defb1	6.14E-94	1.131940724	0.812	0.03	1.23E-90	pericyte	2	Defb1
Clic31	9.77E-94	0.336770413	0.812	0.028	1.95E-90	pericyte	2	Clic3
Cyp2b10	4.05E-91	0.638174818	0.875	0.084	8.10E-88	pericyte	2	Cyp2b10
Cyp2a5	1.58E-90	1.580675084	0.875	0.057	3.16E-87	pericyte	2	Cyp2a5
Tspan81	1.19E-89	0.6076964	0.859	0.059	2.39E-86	pericyte	2	Tspan8
Klf5	1.43E-86	1.332638179	0.953	0.118	2.85E-83	pericyte	2	Klf5
1110017D15Rik	3.59E-84	0.58541066	0.797	0.026	7.19E-81	pericyte	2	1110017D15Rik
Plet11	2.02E-75	0.381044694	0.828	0.046	4.03E-72	pericyte	2	Plet1
Chil1	5.82E-71	0.266394889	0.828	0.084	1.16E-67	pericyte	2	Chil1
Capsl	8.45E-71	0.356126244	0.844	0.075	1.69E-67	pericyte	2	Capsl
Dapl11	5.51E-70	2.29665596	0.969	0.172	1.10E-66	pericyte	2	Dapl1
Krt81	2.18E-66	1.875544551	1	0.085	4.37E-63	pericyte	2	Krt8
Nkx2-1	3.22E-64	0.735014142	0.594	0.008	6.43E-61	pericyte	2	Nkx2-1
Adig	3.58E-64	0.358309662	0.844	0.153	7.17E-61	pericyte	2	Adig
Anxa81	2.24E-63	1.033576225	0.922	0.109	4.47E-60	pericyte	2	Anxa8
Kcnk1	1.23E-62	0.284435768	0.75	0.052	2.46E-59	pericyte	2	Kcnk1
Lrrc26	1.08E-59	0.328743955	0.281	0.001	2.16E-56	pericyte	2	Lrrc26
Foxa1	4.68E-59	0.95768226	0.688	0.006	9.36E-56	pericyte	2	Foxa1
Krt181	3.81E-57	1.736145551	0.984	0.204	7.62E-54	pericyte	2	Krt18
Lypd2	4.07E-57	2.591116752	0.984	0.077	8.13E-54	pericyte	2	Lypd2
Aqp5	3.84E-55	1.38671366	0.844	0.054	7.67E-52	pericyte	2	Aqp5
Cyp2f2	4.28E-55	4.252623023	1	0.145	8.56E-52	pericyte	2	Cyp2f2
Wfdc21	6.27E-55	3.583786742	1	0.085	1.25E-51	pericyte	2	Wfdc2
Fgfr32	7.89E-55	1.090893878	0.938	0.197	1.58E-51	pericyte	2	Fgfr3
8430408G22Rik	3.14E-54	1.663102134	0.953	0.062	6.27E-51	pericyte	2	8430408G22Rik
Fxyd32	3.02E-52	2.434883905	1	0.283	6.03E-49	pericyte	2	Fxyd3
Upk1b1	4.86E-52	0.749532578	0.875	0.162	9.71E-49	pericyte	2	Upk1b
Gsta31	5.74E-52	1.795006202	0.984	0.151	1.15E-48	pericyte	2	Gsta3
Gsto1	1.21E-50	3.017259915	1	0.266	2.43E-47	pericyte	2	Gsto1
Gsta41	1.28E-49	1.938571409	0.969	0.177	2.55E-46	pericyte	2	Gsta4
Rassf91	1.95E-49	1.12300131	0.906	0.15	3.89E-46	pericyte	2	Rassf9
Epas13	1.79E-47	2.216978818	0.984	0.243	3.58E-44	pericyte	2	Epas1
Reg3g	3.44E-47	2.934402985	0.922	0.08	6.89E-44	pericyte	2	Reg3g
Cbr25	8.18E-47	3.680043359	1	0.401	1.64E-43	pericyte	2	Cbr2
Fmo32	6.57E-45	1.080746693	0.922	0.133	1.31E-41	pericyte	2	Fmo3
Tnfrsf19	8.77E-45	0.276820391	0.812	0.151	1.75E-41	pericyte	2	Tnfrsf19
Apoc1	1.38E-44	0.736490998	0.844	0.181	2.76E-41	pericyte	2	Apoc1
Krt15	2.57E-43	2.404382428	0.875	0.103	5.14E-40	pericyte	2	Krt15
Mal2	4.86E-43	0.728273587	0.656	0.01	9.71E-40	pericyte	2	Mal2
Spint27	1.40E-42	1.712850346	0.984	0.292	2.80E-39	pericyte	2	Spint2
Ank31	2.60E-42	0.304160807	0.859	0.196	5.19E-39	pericyte	2	Ank3
Prkar2b	1.79E-41	0.276292424	0.859	0.148	3.58E-38	pericyte	2	Prkar2b
Ppm1l1	1.54E-40	0.42719774	0.844	0.155	3.09E-37	pericyte	2	Ppm1l
Tst1	2.35E-40	1.390228418	0.953	0.309	4.71E-37	pericyte	2	Tst
Prdx61	2.52E-40	2.1206199	0.984	0.569	5.05E-37	pericyte	2	Prdx6
F32	3.53E-40	1.974142895	0.969	0.37	7.05E-37	pericyte	2	F3
Pglyrp11	5.54E-40	1.513810639	0.922	0.226	1.11E-36	pericyte	2	Pglyrp1

Aldh3a12	1.48E-39	0.747828785	0.875	0.225	2.96E-36	pericyte 2	Aldh3a1
Tmem108	5.96E-39	0.26723148	0.844	0.152	1.19E-35	pericyte 2	Tmem108
Krt191	6.96E-39	1.851048619	0.922	0.129	1.39E-35	pericyte 2	Krt19
Adh72	1.39E-38	1.289873691	0.906	0.214	2.78E-35	pericyte 2	Adh7
Cyp4b13	7.42E-38	1.472670863	0.844	0.108	1.48E-34	pericyte 2	Cyp4b1
Kpna21	3.52E-37	0.282171624	0.859	0.197	7.04E-34	pericyte 2	Kpna2
Emb3	4.41E-37	1.20534183	0.969	0.262	8.82E-34	pericyte 2	Emb
Gstm16	7.93E-37	1.710215377	1	0.732	1.59E-33	pericyte 2	Gstm1
S100a14	1.50E-36	0.696804468	0.641	0.017	2.99E-33	pericyte 2	S100a14
Ifitm12	1.66E-36	2.121658028	0.906	0.145	3.32E-33	pericyte 2	Ifitm1
Ces1d2	1.23E-34	1.492815328	0.906	0.271	2.47E-31	pericyte 2	Ces1d
Tmem176a6	1.35E-34	1.796938592	0.984	0.345	2.71E-31	pericyte 2	Tmem176a
5330417C22Rik	1.41E-34	0.816319129	0.641	0.03	2.83E-31	pericyte 2	5330417C22Rik
Crlf11	1.61E-34	1.001901421	0.844	0.176	3.22E-31	pericyte 2	Crlf1
Scgb3a2	3.11E-34	3.543018026	0.891	0.113	6.22E-31	pericyte 2	Scgb3a2
Selenbp14	5.00E-34	1.973734633	0.938	0.412	1.00E-30	pericyte 2	Selenbp1
Epcam	6.27E-34	0.806623683	0.734	0.07	1.25E-30	pericyte 2	Epcam
Mlf1	1.45E-33	0.260269768	0.812	0.144	2.90E-30	pericyte 2	Mlf1
Msln1	1.62E-33	0.361588372	0.828	0.168	3.24E-30	pericyte 2	Msln
Ifit11	7.72E-33	0.326184446	0.875	0.219	1.54E-29	pericyte 2	Ifit1
Lgr64	1.00E-32	0.948330425	0.922	0.285	2.01E-29	pericyte 2	Lgr6
2200002D01Rik4	5.62E-32	1.015298008	0.922	0.228	1.12E-28	pericyte 2	2200002D01Rik
Fgf12	7.52E-31	0.310970767	0.859	0.257	1.50E-27	pericyte 2	Fgf1
Scgb1a1	2.75E-30	4.758591529	0.922	0.382	5.51E-27	pericyte 2	Scgb1a1
Prom1	3.64E-29	0.309954542	0.297	0.008	7.28E-26	pericyte 2	Prom1
Ptn3	5.55E-29	0.542829849	0.891	0.297	1.11E-25	pericyte 2	Ptn
Rtp42	4.46E-28	0.492635662	0.891	0.238	8.92E-25	pericyte 2	Rtp4
Cd24a4	7.59E-28	1.070928367	0.938	0.334	1.52E-24	pericyte 2	Cd24a
Oasl24	9.73E-28	0.420949048	0.891	0.251	1.95E-24	pericyte 2	Oasl2
Hpgd4	1.06E-27	0.336504291	0.875	0.285	2.12E-24	pericyte 2	Hpgd
Dsp4	1.81E-27	0.663462827	0.875	0.228	3.63E-24	pericyte 2	Dsp
Bpifa1	8.16E-27	4.229389322	0.844	0.153	1.63E-23	pericyte 2	Bpifa1
Isg153	2.54E-26	0.42600655	0.859	0.224	5.08E-23	pericyte 2	Isg15
Cebpa3	1.18E-25	0.482771775	0.875	0.268	2.36E-22	pericyte 2	Cebpa
Sec14l3	8.60E-25	1.394477819	0.578	0.013	1.72E-21	pericyte 2	Sec14l3
Col17a1	1.89E-24	0.365418346	0.297	0.049	3.77E-21	pericyte 2	Col17a1
Aldh1a14	5.09E-23	1.675031409	0.859	0.304	1.02E-19	pericyte 2	Aldh1a1
Chchd10	1.57E-22	1.187653882	0.922	0.314	3.14E-19	pericyte 2	Chchd10
Perp5	7.27E-22	1.180251109	0.812	0.189	1.45E-18	pericyte 2	Perp
Dnajb1	3.21E-21	0.311550385	0.859	0.314	6.42E-18	pericyte 2	Dnajb1
Ptprf3	7.65E-21	0.924681719	0.797	0.223	1.53E-17	pericyte 2	Ptprf
Tmem176b8	1.59E-20	1.158023343	0.938	0.437	3.17E-17	pericyte 2	Tmem176b
Abi3bp6	4.59E-19	1.18766407	0.938	0.457	9.18E-16	pericyte 2	Abi3bp
AI6614531	9.27E-19	0.342513632	0.703	0.188	1.85E-15	pericyte 2	AI661453
Krt71	1.12E-18	1.059134952	0.719	0.084	2.25E-15	pericyte 2	Krt7
Krt5	2.40E-18	1.273075838	0.609	0.033	4.81E-15	pericyte 2	Krt5
Car84	6.48E-16	0.759206807	0.75	0.245	1.30E-12	pericyte 2	Car8
Atp1b12	6.78E-16	0.574738189	0.812	0.345	1.36E-12	pericyte 2	Atp1b1
Cyp2s11	3.61E-15	0.441231139	0.672	0.136	7.21E-12	pericyte 2	Cyp2s1
Agr2	8.80E-15	0.564890386	0.297	0.025	1.76E-11	pericyte 2	Agr2
Hes14	1.88E-12	0.930888996	0.781	0.309	3.75E-09	pericyte 2	Hes1
Tspan1	1.99E-12	0.864014816	0.578	0.009	3.99E-09	pericyte 2	Tspan1
Hspb18	2.28E-12	0.478616946	0.922	0.489	4.56E-09	pericyte 2	Hspb1
Pdpn6	8.10E-12	0.818083157	0.75	0.29	1.62E-08	pericyte 2	Pdpn
Slc6a63	9.10E-12	0.779285642	0.812	0.476	1.82E-08	pericyte 2	Slc6a6
Sfn	2.20E-11	0.900075466	0.672	0.236	4.40E-08	pericyte 2	Sfn
Cldn7	2.87E-11	0.907768673	0.609	0.137	5.73E-08	pericyte 2	Cldn7
Ece13	3.21E-11	0.640145668	0.766	0.309	6.43E-08	pericyte 2	Ece1
B3galt2	5.03E-11	1.050348505	0.578	0.024	1.01E-07	pericyte 2	B3galt2
Emp16	5.19E-11	0.287942067	0.906	0.414	1.04E-07	pericyte 2	Emp1
Ccnd13	4.84E-10	0.825805814	0.719	0.288	9.68E-07	pericyte 2	Ccnd1
Sdc14	5.98E-10	0.91800651	0.688	0.308	1.20E-06	pericyte 2	Sdc1
Ezr1	1.05E-09	0.676175445	0.734	0.307	2.10E-06	pericyte 2	Ezr
Ly6e7	1.90E-09	0.435753212	1	0.795	3.80E-06	pericyte 2	Ly6e
Pigr	5.42E-09	1.010196189	0.297	0.064	1.08E-05	pericyte 2	Pigr
Anxa24	2.57E-08	0.477723766	0.969	0.667	5.15E-05	pericyte 2	Anxa2
Aox3	3.33E-08	0.828267654	0.578	0.141	6.66E-05	pericyte 2	Aox3
Gclc	4.36E-08	0.45393792	0.672	0.242	8.72E-05	pericyte 2	Gclc
Mgst15	3.72E-07	0.514038046	0.922	0.542	0.00074326	pericyte 2	Mgst1
Cd99	6.15E-07	0.501994274	0.906	0.649	0.001230992	pericyte 2	Cd9
Wnt41	9.87E-07	0.539972718	0.562	0.061	0.001973666	pericyte 2	Wnt4
Scgb3a1	1.73E-06	3.582916426	0.609	0.125	0.003454645	pericyte 2	Scgb3a1
Lgals34	2.48E-06	0.767691131	0.719	0.342	0.00495088	pericyte 2	Lgals3
Tubb4b4	5.58E-06	0.538830687	0.75	0.408	0.011162223	pericyte 2	Tubb4b
Ltf	6.71E-06	1.497900012	0.266	0.077	0.01342774	pericyte 2	Ltf
Hmgb21	9.01E-06	0.285991733	0.734	0.386	0.018019475	pericyte 2	Hmgb2
Prdx14	2.06E-05	0.450995586	0.953	0.798	0.041105245	pericyte 2	Prdx1
Tgm28	2.56E-05	0.463695015	0.703	0.314	0.051274048	pericyte 2	Tgm2
F11r4	3.47E-05	0.455131721	0.609	0.216	0.069349576	pericyte 2	F11r
Itgb41	5.86E-05	0.546076245	0.547	0.103	0.117250632	pericyte 2	Itgb4
Cxadr1	7.50E-05	0.496571285	0.531	0.089	0.150081778	pericyte 2	Cxadr
Muc1	9.20E-05	0.553881999	0.531	0.095	0.184091627	pericyte 2	Muc1
Net14	0.000339556	0.512697719	0.609	0.239	0.679112759	pericyte 2	Net1
Rab25	0.000537014	0.54508824	0.469	0.006	1	pericyte 2	Rab25
Anxa14	0.00055378	0.251961155	0.812	0.607	1	pericyte 2	Anxa1
Cxcl17	0.001123529	0.65839918	0.344	0.018	1	pericyte 2	Cxcl17
Galnt182	0.003189324	0.277629429	0.359	0.252	1	pericyte 2	Galnt18
Bpifb1	0.003316313	1.616993159	0.344	0.081	1	pericyte 2	Bpifb1
Hp	0.003741732	0.33255591	0.328	0.18	1	pericyte 2	Hp
Bcam8	0.006349763	0.275448053	0.656	0.33	1	pericyte 2	Bcam
Cd3g1	0	2.064347123	0.809	0.181	0	T	Cd3g
Cd3d1	0	2.044817548	0.854	0.239	0	T	Cd3d
Ms4a4b1	0	1.775037285	0.753	0.278	0	T	Ms4a4b
Hcst1	0	1.686784346	0.807	0.32	0	T	Hcst
Lck1	0	1.514632931	0.765	0.219	0	T	Lck
Cd3e1	0	1.412986766	0.674	0.14	0	T	Cd3e
Lat1	0	1.394925368	0.698	0.213	0	T	Lat
Rac22	0	1.394222462	0.932	0.475	0	T	Rac2
Ptprcap1	0	1.343888842	0.87	0.429	0	T	Ptprcap
Limd21	0	1.326775222	0.881	0.484	0	T	Limd2
Gimap31	0	1.311738292	0.627	0.215	0	T	Gimap3
Selplg3	0	1.275763929	0.695	0.274	0	T	Selplg
Skap1	0	1.259978476	0.647	0.15	0	T	Skap1
Cd522	0	1.215642597	0.984	0.605	0	T	Cd52
Ltb1	0	1.195003877	0.814	0.421	0	T	Ltb
Coro1a2	0	1.065022569	0.936	0.517	0	T	Coro1a
Tmsb103	0	1.051771656	1	0.947	0	T	Tmsb10
Ptpn184	0	1.051230266	0.922	0.422	0	T	Ptpn18
Fyb4	0	1.048678905	0.668	0.24	0	T	Fyb
Lef1	0	1.029464683	0.55	0.162	0	T	Lef1
Cd247	0	0.983969151	0.522	0.12	0	T	Cd247
Fxyd55	0	0.961635954	0.829	0.438	0	T	Fxyd5
Txk	0	0.951045706	0.514	0.123	0	T	Txk
Arhgdib3	0	0.946250206	0.906	0.537	0	T	Arhgdib
H2-K16	0	0.868748534	0.97	0.793	0	T	H2-K1
Sh2d1a1	0	0.487481246	0.361	0.048	0	T	Sh2d1a
Ms4a6b3	2.43E-287	0.993563767	0.594	0.171	4.87E-284	T	Ms4a6b
Klk81	1.22E-285	0.885091347	0.514	0.142	2.45E-282	T	Klk8
H2-Q75	2.48E-279	1.156305963	0.715	0.361	4.97E-276	T	H2-Q7
Gimap44	1.55E-276	1.093987226	0.653	0.3	3.09E-273	T	Gimap4
B2m5	2.45E-274	0.558788018	0.998	0.982	4.90E-271	T	B2m
Tcf71	5.99E-269	0.95260149	0.503	0.138	1.20E-265	T	Tcf7
Cd8a1	2.02E-268	0.837123472	0.448	0.132	4.04E-265	T	Cd8a
Thy14	4.35E-258	1.182436915	0.721	0.417	8.69E-255	T	Thy1
Nsg2	1.04E-250	0.445661882	0.367	0.076	2.08E-247	T	Nsg2
Gimap13	3.06E-246	0.994332756	0.695	0.361	6.12E-243	T	Gimap1
Ptprc3	1.06E-243	0.968029627	0.735	0.42	2.12E-240	T	Ptprc
AW1120103	9.75E-243	1.344475115	0.709	0.39	1.95E-239	T	AW112010
Il2rg1	1.29E-239	0.940892766	0.66	0.36	2.59E-236	T	Il2rg

Gmfg5	2.75E-239	0.792346887	0.759	0.38	5.50E-236	T	Gmfg
Ctsw	9.32E-234	0.854028635	0.456	0.158	1.86E-230	T	Ctsw
Cd7	2.28E-224	0.751899077	0.386	0.107	4.55E-221	T	Cd7
Psmb84	6.66E-214	0.771675507	0.806	0.523	1.33E-210	T	Psmb8
Cotl13	6.64E-209	0.751559257	0.788	0.491	1.33E-205	T	Cotl1
Nkg71	7.95E-206	1.891713874	0.538	0.248	1.59E-202	T	Nkg7
Cd8b11	6.16E-200	1.462212021	0.514	0.248	1.23E-196	T	Cd8b1
Laptm54	3.25E-199	0.702722552	0.752	0.41	6.50E-196	T	Laptm5
Izumo1r	2.53E-190	0.557449246	0.297	0.058	5.05E-187	T	Izumo1r
Sh3bgrl33	7.70E-190	0.558439347	0.968	0.803	1.54E-186	T	Sh3bgrl3
Tnfrsf18	8.88E-182	0.636523319	0.371	0.097	1.78E-178	T	Tnfrsf18
Cd21	1.82E-181	0.873000086	0.588	0.332	3.64E-178	T	Cd2
Gimap63	5.14E-181	0.856550524	0.666	0.409	1.03E-177	T	Gimap6
Il7r	3.17E-176	1.042886661	0.437	0.129	6.33E-173	T	Il7r
Itgb71	5.34E-172	0.831029698	0.5	0.245	1.07E-168	T	Itgb7
Cd532	3.89E-168	0.752212224	0.702	0.431	7.78E-165	T	Cd53
H2-D17	4.27E-163	0.521908702	0.97	0.886	8.54E-160	T	H2-D1
Ly6c2	9.35E-162	1.121704869	0.404	0.178	1.87E-158	T	Ly6c2
Klrd11	1.97E-154	0.661587775	0.391	0.141	3.94E-151	T	Klrd1
Cd28	2.05E-154	0.72526951	0.431	0.18	4.10E-151	T	Cd28
S1pr14	7.48E-153	0.708294944	0.535	0.282	1.50E-149	T	S1pr1
Dapl12	3.66E-144	0.828383755	0.404	0.16	7.33E-141	T	Dapl1
Tmsb4x8	2.09E-130	0.292848774	1	1	4.17E-127	T	Tmsb4x
Il2rb	4.16E-129	0.572536494	0.342	0.116	8.32E-126	T	Il2rb
Epsti12	8.83E-113	0.625531426	0.478	0.289	1.77E-109	T	Epsti1
Bcl11b	8.83E-110	0.831822461	0.396	0.13	1.77E-106	T	Bcl11b
Srgn4	5.23E-109	0.599196172	0.68	0.488	1.05E-105	T	Srgn
Acp51	1.24E-106	0.516058716	0.435	0.257	2.48E-103	T	Acp5
Cytip	3.25E-106	0.639243054	0.482	0.271	6.51E-103	T	Cytip
Ccl51	2.57E-103	2.672183985	0.406	0.248	5.14E-100	T	Ccl5
Rgs104	1.07E-101	0.39379901	0.631	0.416	2.14E-98	T	Rgs10
Ucp25	7.59E-92	0.398266714	0.654	0.428	1.52E-88	T	Ucp2
Ccr7	2.60E-82	0.549512477	0.456	0.307	5.20E-79	T	Ccr7
Itgb22	3.18E-80	0.488140586	0.4	0.249	6.37E-77	T	Itgb2
Smc41	2.10E-77	0.577262489	0.448	0.305	4.21E-74	T	Smc4
Pycard2	4.15E-76	0.49549121	0.521	0.373	8.30E-73	T	Pycard
B930036N10Rik	4.25E-74	0.694825017	0.423	0.295	8.51E-71	T	B930036N10Rik
Slamf6	6.06E-71	0.464886267	0.392	0.232	1.21E-67	T	Slamf6
Gimap7	2.86E-68	0.485674754	0.343	0.185	5.72E-65	T	Gimap7
Emb4	8.89E-68	0.615993682	0.401	0.256	1.78E-64	T	Emb
Sell1	9.41E-66	0.603107756	0.484	0.349	1.88E-62	T	Sell
Cxcr6	1.41E-65	0.954614714	0.301	0.117	2.81E-62	T	Cxcr6
Slc9a3r11	5.68E-64	0.453772536	0.43	0.313	1.14E-60	T	Slc9a3r1
Slfn24	1.63E-62	0.567426988	0.426	0.291	3.27E-59	T	Slfn2
Pitpnc13	1.29E-56	0.337022403	0.339	0.238	2.59E-53	T	Pitpnc1
Lcp14	9.65E-56	0.421793408	0.558	0.421	1.93E-52	T	Lcp1
Ctla2a3	2.67E-53	0.478740147	0.299	0.188	5.34E-50	T	Ctla2a
Pim11	4.01E-53	0.563278948	0.376	0.262	8.02E-50	T	Pim1
Hmgb22	9.72E-53	0.434303939	0.481	0.381	1.94E-49	T	Hmgb2
Kcnn41	1.44E-48	0.445597463	0.305	0.158	2.87E-45	T	Kcnn4
Ass11	9.88E-47	0.289473933	0.338	0.256	1.98E-43	T	Ass1
Tspan134	1.18E-45	0.284828899	0.386	0.276	2.35E-42	T	Tspan13
Cd484	2.78E-43	0.401709621	0.418	0.325	5.56E-40	T	Cd48
Tesc	1.53E-41	0.301313011	0.288	0.188	3.07E-38	T	Tesc
Cd4	1.75E-37	0.359749346	0.269	0.119	3.49E-34	T	Cd4
Ezr2	2.40E-35	0.281249985	0.385	0.304	4.81E-32	T	Ezr
Klf25	1.15E-32	0.278402156	0.653	0.56	2.29E-29	T	Klf2
H2-Q62	2.15E-30	0.448531444	0.362	0.275	4.30E-27	T	H2-Q6
Tes4	3.78E-28	0.291694948	0.3	0.234	7.56E-25	T	Tes
Ikzf2	1.08E-27	0.570516067	0.258	0.205	2.17E-24	T	Ikzf2
Gata31	5.81E-27	0.589930385	0.269	0.161	1.16E-23	T	Gata3
Hsd11b11	9.56E-20	0.261829806	0.32	0.27	1.91E-16	T	Hsd11b1
Ccr22	2.95E-17	0.563058068	0.285	0.231	5.90E-14	T	Ccr2
Apobec32	1.78E-16	0.333291911	0.421	0.394	3.56E-13	T	Apobec3
Rgcc1	2.95E-14	0.259732584	0.261	0.212	5.90E-11	T	Rgcc
Ramp15	5.82E-12	0.28304672	0.441	0.428	1.16E-08	T	Ramp1
Psmb92	7.16E-10	0.313692644	0.429	0.435	1.43E-06	T	Psmb9
Ifi2033	1.07E-09	0.265625228	0.402	0.387	2.14E-06	T	Ifi203
Cd842	2.23E-09	0.428395454	0.345	0.302	4.45E-06	T	Cd84
Traf1	7.95E-09	0.384907429	0.258	0.199	1.59E-05	T	Traf1
Itga41	8.19E-09	0.339517279	0.296	0.293	1.64E-05	T	Itga4
Glipr11	5.95E-08	0.262863967	0.264	0.264	0.000118939	T	Glipr1
Prdx62	5.15E-07	0.266623866	0.531	0.572	0.00102999	T	Prdx6
Arhgap303	1.15E-06	0.399569553	0.321	0.298	0.002304256	T	Arhgap30
Id25	8.48E-06	0.487853533	0.459	0.522	0.016965674	T	Id2
Tmem176a7	0.003882496	0.334419469	0.273	0.351	1	T	Tmem176a
H2-Ab14	0	2.072043611	0.963	0.679	0	DC	H2-Ab1
H2-Eb14	0	1.918185026	0.959	0.588	0	DC	H2-Eb1
H2-Aa4	0	1.894106402	0.953	0.672	0	DC	H2-Aa
Lgals35	0	1.85200985	0.903	0.32	0	DC	Lgals3
Cd743	0	1.782792382	0.985	0.856	0	DC	Cd74
H2-DMb13	0	1.744581801	0.873	0.347	0	DC	H2-DMb1
Tyrobp4	0	1.66422945	0.985	0.347	0	DC	Tyrobp
Alox5ap4	0	1.605839117	0.935	0.255	0	DC	Alox5ap
H2-DMa3	0	1.583069807	0.939	0.397	0	DC	H2-DMa
Ccr23	0	1.547307516	0.808	0.21	0	DC	Ccr2
Fcer1g4	0	1.510274311	0.97	0.356	0	DC	Fcer1g
Gm2a1	0	1.507251714	0.945	0.544	0	DC	Gm2a
Lsp12	0	1.462824472	0.975	0.615	0	DC	Lsp1
Cd523	0	1.403727708	0.994	0.611	0	DC	Cd52
Plbd11	0	1.375007845	0.775	0.228	0	DC	Plbd1
Lyz24	0	1.347377913	0.972	0.635	0	DC	Lyz2
Fxyd56	0	1.345489093	0.932	0.439	0	DC	Fxyd5
Ctss4	0	1.225669839	0.94	0.396	0	DC	Ctss
Ucp26	0	1.190448774	0.952	0.419	0	DC	Ucp2
Laptm55	0	1.18404735	0.954	0.407	0	DC	Laptm5
Tmsb4x9	0	1.180773478	1	1	0	DC	Tmsb4x
Sh3bgrl34	0	1.131447443	0.999	0.804	0	DC	Sh3bgrl3
Bcl2a1d1	0	1.130672607	0.619	0.13	0	DC	Bcl2a1d
Spi14	0	1.120344559	0.857	0.276	0	DC	Spi1
Coro1a3	0	1.049240396	0.959	0.522	0	DC	Coro1a
Rnase61	6.28E-303	1.022689001	0.716	0.252	1.26E-299	DC	Rnase6
Mpeg13	4.62E-302	1.187066529	0.699	0.236	9.24E-299	DC	Mpeg1
Cotl14	1.31E-299	1.077778919	0.923	0.49	2.61E-296	DC	Cotl1
Ifitm61	1.53E-299	1.158697155	0.62	0.14	3.05E-296	DC	Ifitm6
Napsa1	3.32E-299	1.081372203	0.772	0.281	6.63E-296	DC	Napsa
Ccl94	3.26E-272	1.223577986	0.78	0.269	6.52E-269	DC	Ccl9
H2afz2	1.34E-271	1.010823713	0.919	0.703	2.69E-268	DC	H2afz
Pld44	2.84E-270	0.812800816	0.763	0.272	5.67E-267	DC	Pld4
Ccl63	4.10E-264	1.267881023	0.838	0.355	8.20E-261	DC	Ccl6
Tifab2	5.30E-261	0.606156002	0.539	0.137	1.06E-257	DC	Tifab
Actb6	6.32E-249	0.853390808	0.999	0.994	1.26E-245	DC	Actb
Cbfa2t34	1.84E-247	0.950225293	0.68	0.234	3.68E-244	DC	Cbfa2t3
Csf2ra4	1.36E-245	0.897419715	0.691	0.271	2.73E-242	DC	Csf2ra
Wfdc173	3.50E-238	1.078653082	0.797	0.34	7.01E-235	DC	Wfdc17
S100a44	5.40E-237	1.333263917	0.873	0.562	1.08E-233	DC	S100a4
Selplg4	3.78E-236	0.822734795	0.726	0.279	7.56E-233	DC	Selplg
Crip15	5.44E-234	0.944172291	0.984	0.967	1.09E-230	DC	Crip1
Psmb85	1.29E-232	0.912987059	0.894	0.524	2.57E-229	DC	Psmb8
Batf33	2.93E-231	0.825352145	0.641	0.249	5.86E-228	DC	Batf3
Cfp4	2.84E-229	0.86999382	0.706	0.233	5.68E-226	DC	Cfp
Clec4b13	3.78E-224	1.098517422	0.623	0.21	7.56E-221	DC	Clec4b1
Rassf44	2.54E-216	0.957077043	0.706	0.282	5.09E-213	DC	Rassf4
Tnip3	2.75E-216	0.551585706	0.401	0.044	5.50E-213	DC	Tnip3
Ptprc4	6.92E-209	0.780987177	0.811	0.422	1.38E-205	DC	Ptprc
Arhgdib4	4.79E-208	0.723744954	0.93	0.542	9.58E-205	DC	Arhgdib
AF2517051	2.92E-206	0.628957871	0.516	0.152	5.84E-203	DC	AF251705
Bcl2a1a	6.05E-204	0.665070787	0.451	0.117	1.21E-200	DC	Bcl2a1a
Ccdc109b4	2.57E-203	0.847151129	0.634	0.231	5.14E-200	DC	Ccdc109b

Evi2a3	3.48E-199	0.749909814	0.705	0.351	6.95E-196	DC	Evi2a
Ly864	4.32E-199	0.688972944	0.721	0.358	8.64E-196	DC	Ly86
Srgn5	1.03E-197	0.935641237	0.844	0.484	2.06E-194	DC	Srgn
Cd684	2.04E-195	0.589943397	0.641	0.23	4.08E-192	DC	Cd68
Ear21	5.05E-194	1.562579798	0.547	0.197	1.01E-190	DC	Ear2
Dpep21	5.18E-194	0.689044091	0.506	0.162	1.04E-190	DC	Dpep2
Aif14	2.73E-185	0.618512048	0.635	0.258	5.46E-182	DC	Aif1
Hepacam2	7.95E-185	0.39931431	0.388	0.039	1.59E-181	DC	Hepacam2
Slamf92	4.21E-183	0.785160429	0.601	0.251	8.42E-180	DC	Slamf9
Gmfg6	4.46E-183	0.613122307	0.833	0.383	8.92E-180	DC	Gmfg
BC0285282	2.80E-180	0.815456669	0.641	0.298	5.60E-177	DC	BC028528
Ifi303	1.18E-175	0.957032667	0.734	0.406	2.37E-172	DC	Ifi30
Pycard3	1.88E-175	0.783440361	0.73	0.367	3.76E-172	DC	Pycard
Clec4a24	3.55E-166	0.617905292	0.536	0.198	7.11E-163	DC	Clec4a2
Ms4a6c5	8.81E-166	0.995195842	0.591	0.183	1.76E-162	DC	Ms4a6c
Upb1	1.14E-164	0.535649478	0.482	0.181	2.28E-161	DC	Upb1
Cst36	2.16E-164	1.134520729	0.997	0.955	4.32E-161	DC	Cst3
Klrd12	8.39E-161	0.798719672	0.481	0.141	1.68E-157	DC	Klrd1
Rac23	3.60E-158	0.493420891	0.853	0.486	7.20E-155	DC	Rac2
Cd485	1.07E-157	0.620635981	0.659	0.316	2.14E-154	DC	Cd48
Kctd127	6.07E-157	0.768606088	0.688	0.348	1.21E-153	DC	Kctd12
Lcp15	2.64E-155	0.651894014	0.781	0.413	5.29E-152	DC	Lcp1
F630028O10Rik	3.23E-152	0.566704353	0.52	0.216	6.46E-149	DC	F630028O10Rik
Rgs22	2.35E-151	0.786251841	0.65	0.332	4.71E-148	DC	Rgs2
Ptpn185	1.35E-148	0.471419317	0.857	0.433	2.70E-145	DC	Ptpn18
Itgb72	1.68E-148	0.545641797	0.548	0.247	3.36E-145	DC	Itgb7
Tmem176a8	1.63E-145	0.75109803	0.689	0.332	3.25E-142	DC	Tmem176a
Cd209a1	5.93E-145	1.681534075	0.522	0.192	1.19E-141	DC	Cd209a
Trf5	2.99E-142	0.47161865	0.681	0.318	5.98E-139	DC	Trf
Slamf81	1.24E-141	0.539668997	0.473	0.175	2.49E-138	DC	Slamf8
Cybb5	1.62E-141	0.709208707	0.636	0.302	3.24E-138	DC	Cybb
Clec9a	6.49E-136	0.367576318	0.342	0.063	1.30E-132	DC	Clec9a
Dok23	2.62E-133	0.580393908	0.526	0.234	5.24E-130	DC	Dok2
H2-D18	3.77E-132	0.506395677	0.989	0.886	7.54E-129	DC	H2-D1
Cytip1	4.28E-131	0.607344761	0.579	0.27	8.55E-128	DC	Cytip
Unc93b13	6.89E-131	0.524413746	0.745	0.417	1.38E-127	DC	Unc93b1
Bcl2a1b3	3.08E-130	0.900899853	0.493	0.209	6.16E-127	DC	Bcl2a1b
Tmem176b9	1.99E-129	0.728686987	0.749	0.426	3.98E-126	DC	Tmem176b
Sdf2l12	4.85E-128	0.554348104	0.615	0.268	9.70E-125	DC	Sdf2l1
Phf11b1	5.37E-125	0.610875957	0.547	0.285	1.07E-121	DC	Phf11b
Tmsb104	1.43E-124	0.578642564	0.998	0.948	2.85E-121	DC	Tmsb10
Arhgap304	8.31E-119	0.579908666	0.597	0.287	1.66E-115	DC	Arhgap30
Fcgr2b4	1.09E-118	0.594826686	0.578	0.237	2.18E-115	DC	Fcgr2b
Psmb93	1.97E-117	0.697645605	0.67	0.424	3.95E-114	DC	Psmb9
Naaa1	3.78E-116	1.043203414	0.633	0.375	7.57E-113	DC	Naaa
Glipr12	9.31E-114	0.543533916	0.533	0.253	1.86E-110	DC	Glipr1
Fcrls4	1.76E-113	0.595245149	0.463	0.205	3.52E-110	DC	Fcrls
Anxa25	5.46E-113	0.610930725	0.874	0.659	1.09E-109	DC	Anxa2
Mcemp1	1.27E-112	0.551370639	0.338	0.067	2.54E-109	DC	Mcemp1
Itgax	1.02E-107	0.366354196	0.369	0.107	2.03E-104	DC	Itgax
Itgb23	3.64E-106	0.591217969	0.539	0.246	7.28E-103	DC	Itgb2
Cd533	2.87E-103	0.469870955	0.732	0.434	5.74E-100	DC	Cd53
Slamf7	4.41E-103	0.550250916	0.451	0.155	8.81E-100	DC	Slamf7
Clec4a13	8.72E-103	0.328166077	0.457	0.205	1.74E-99	DC	Clec4a1
Pirb3	2.25E-99	0.522599254	0.483	0.205	4.50E-96	DC	Pirb
Ccnd14	2.76E-99	0.770395167	0.541	0.278	5.51E-96	DC	Ccnd1
Wdfy41	1.11E-98	0.572047905	0.483	0.23	2.23E-95	DC	Wdfy4
Amica1	1.46E-97	0.523780232	0.38	0.085	2.92E-94	DC	Amica1
Clec12a3	2.51E-97	0.609723379	0.487	0.205	5.03E-94	DC	Clec12a
Cd300a1	3.33E-96	0.453852857	0.412	0.13	6.66E-93	DC	Cd300a
Clec10a4	9.75E-95	0.566613172	0.449	0.169	1.95E-91	DC	Clec10a
Xcr11	1.22E-93	0.318602355	0.318	0.098	2.45E-90	DC	Xcr1
Mgl24	1.07E-91	0.772459685	0.485	0.242	2.15E-88	DC	Mgl2
H2-DMb22	7.78E-91	0.373827292	0.615	0.381	1.56E-87	DC	H2-DMb2
Lst13	1.13E-90	0.706957263	0.538	0.317	2.25E-87	DC	Lst1
P2ry64	1.95E-90	0.31016487	0.531	0.282	3.89E-87	DC	P2ry6
Lyz12	4.30E-90	2.005493525	0.429	0.206	8.59E-87	DC	Lyz1
Csf2rb3	2.49E-87	0.414217085	0.408	0.173	4.98E-84	DC	Csf2rb
Rgs105	4.24E-85	0.33010716	0.69	0.417	8.47E-82	DC	Rgs10
Pkib	1.57E-84	0.38477724	0.439	0.213	3.14E-81	DC	Pkib
Adam8	3.21E-82	0.401027392	0.37	0.121	6.42E-79	DC	Adam8
Fcgr34	6.83E-82	0.272040485	0.491	0.24	1.37E-78	DC	Fcgr3
Clec4a33	2.13E-81	0.523939877	0.458	0.223	4.25E-78	DC	Clec4a3
Cd24a5	3.43E-81	0.593183746	0.544	0.327	6.85E-78	DC	Cd24a
Ltb4r1	1.89E-80	0.607812849	0.426	0.143	3.77E-77	DC	Ltb4r1
Sec11c1	8.38E-74	0.349241906	0.685	0.419	1.68E-70	DC	Sec11c
Slfn25	2.20E-72	0.503817243	0.531	0.289	4.40E-69	DC	Slfn2
Gapdh2	2.85E-72	0.33149441	0.934	0.806	5.70E-69	DC	Gapdh
H2-Oa1	6.16E-72	0.443082043	0.547	0.367	1.23E-68	DC	H2-Oa
AI6622702	1.51E-69	0.499960774	0.514	0.29	3.03E-66	DC	AI662270
Itgam3	1.06E-68	0.520166889	0.414	0.15	2.12E-65	DC	Itgam
Emb5	3.28E-67	0.517029315	0.465	0.255	6.55E-64	DC	Emb
Ptpn62	1.32E-66	0.350611633	0.591	0.382	2.63E-63	DC	Ptpn6
Sdc33	1.61E-65	0.419663782	0.488	0.276	3.23E-62	DC	Sdc3
Pilra	5.18E-65	0.443240051	0.358	0.135	1.04E-61	DC	Pilra
Ckb6	9.00E-65	0.514073177	0.72	0.523	1.80E-61	DC	Ckb
Ptafr3	9.75E-64	0.377026736	0.448	0.255	1.95E-60	DC	Ptafr
Klrk11	2.91E-61	0.355255509	0.343	0.087	5.82E-58	DC	Klrk1
Fyb5	2.56E-58	0.43225473	0.457	0.256	5.12E-55	DC	Fyb
Pafah1b3	9.40E-56	0.313012118	0.458	0.298	1.88E-52	DC	Pafah1b3
Retnla4	2.29E-52	1.714258245	0.804	0.637	4.59E-49	DC	Retnla
Map4k11	9.37E-51	0.396776541	0.452	0.265	1.87E-47	DC	Map4k1
Itga42	1.84E-50	0.43734078	0.462	0.286	3.68E-47	DC	Itga4
Gsto11	5.47E-50	0.301171639	0.436	0.261	1.09E-46	DC	Gsto1
Plin21	2.47E-49	0.362682948	0.498	0.304	4.94E-46	DC	Plin2
Skint3	1.27E-46	0.423976176	0.303	0.058	2.54E-43	DC	Skint3
B2m6	3.05E-45	0.262163852	0.993	0.983	6.11E-42	DC	B2m
Lilrb4a2	1.69E-44	0.303583899	0.358	0.189	3.38E-41	DC	Lilrb4a
Shtn11	2.28E-44	0.44398069	0.371	0.183	4.56E-41	DC	Shtn1
Klrb1b	2.93E-43	0.319962709	0.272	0.042	5.87E-40	DC	Klrb1b
Hbb-bs6	1.66E-39	0.555907537	0.823	0.715	3.33E-36	DC	Hbb-bs
Cx3cr12	1.56E-36	0.256511702	0.307	0.167	3.12E-33	DC	Cx3cr1
Gm15987	1.11E-34	0.306687394	0.31	0.113	2.22E-31	DC	Gm15987
Limd22	1.37E-33	0.284578342	0.609	0.502	2.75E-30	DC	Limd2
Hba-a15	1.20E-32	0.599298218	0.7	0.555	2.40E-29	DC	Hba-a1
Gngt22	3.79E-32	0.497985325	0.419	0.308	7.58E-29	DC	Gngt2
Adssl1	8.61E-32	0.313217799	0.371	0.23	1.72E-28	DC	Adssl1
Epsti13	2.06E-30	0.489047069	0.432	0.294	4.12E-27	DC	Epsti1
Tnni21	2.06E-29	0.41731779	0.447	0.363	4.12E-26	DC	Tnni2
Pltp5	2.00E-28	0.367952494	0.536	0.403	4.00E-25	DC	Pltp
Irf83	1.01E-26	1.016620052	0.424	0.336	2.02E-23	DC	Irf8
Gda5	4.17E-26	0.341231574	0.391	0.265	8.35E-23	DC	Gda
Hba-a25	1.37E-25	0.497762952	0.645	0.513	2.74E-22	DC	Hba-a2
Samsn11	3.65E-25	0.614298288	0.364	0.216	7.31E-22	DC	Samsn1
Traf11	5.52E-25	0.421504404	0.338	0.196	1.10E-21	DC	Traf1
Marcksl13	8.91E-21	0.519823446	0.431	0.354	1.78E-17	DC	Marcksl1
Ccl52	1.36E-19	0.949682139	0.31	0.255	2.72E-16	DC	Ccl5
Plek3	6.79E-18	0.340275895	0.338	0.237	1.36E-14	DC	Plek
Pim12	3.14E-15	0.281661835	0.363	0.265	6.27E-12	DC	Pim1
Cd843	1.05E-13	0.297504364	0.383	0.301	2.10E-10	DC	Cd84
Ncf4	1.35E-13	0.303880052	0.41	0.336	2.71E-10	DC	Ncf4
Capg2	1.74E-11	0.345695024	0.591	0.545	3.49E-08	DC	Capg
Tbc1d4	7.56E-10	0.280705217	0.253	0.208	1.51E-06	DC	Tbc1d4
Id26	3.76E-09	0.28915336	0.57	0.516	7.52E-06	DC	Id2
Kcnn42	2.53E-07	0.294783436	0.278	0.162	0.000505291	DC	Kcnn4
Iqgap22	4.85E-07	0.269861854	0.309	0.247	0.000969424	DC	Iqgap2
Ms4a4c	3.52E-05	0.378294928	0.35	0.285	0.07047451	DC	Ms4a4c
Lpl3	0.00629373	0.377546468	0.392	0.477	1	DC	Lpl
Sox10	0	1.92245369	0.892	0.038	0	Neural 1	Sox10

Vmn1r181	0	0.507978346	0.518	0	0	Neural	1	Vmn1r181
Mmd2	3.18E-294	0.459438902	0.532	0.024	6.36E-291	Neural	1	Mmd2
Kcna1	1.52E-279	3.094636563	0.935	0.051	3.04E-276	Neural	1	Kcna1
Sostdc1	2.80E-226	2.219790256	0.813	0.048	5.60E-223	Neural	1	Sostdc1
Gpr37l1	9.00E-223	2.034842833	0.863	0.045	1.80E-219	Neural	1	Gpr37l1
Chl1	2.95E-201	1.614786365	0.712	0.022	5.90E-198	Neural	1	Chl1
Cldn19	2.60E-194	0.683156965	0.482	0.013	5.21E-191	Neural	1	Cldn19
Moxd1	4.85E-194	0.962280401	0.626	0.048	9.70E-191	Neural	1	Moxd1
Fabp7	4.67E-188	1.57010816	0.734	0.027	9.35E-185	Neural	1	Fabp7
Cadm2	2.16E-184	0.914844636	0.54	0.022	4.32E-181	Neural	1	Cadm2
Gfra3	5.35E-172	2.505451263	0.799	0.054	1.07E-168	Neural	1	Gfra3
Mal1	1.42E-145	1.779373337	0.777	0.08	2.85E-142	Neural	1	Mal
Cadm4	1.18E-120	1.606733747	0.777	0.088	2.36E-117	Neural	1	Cadm4
Plp1	5.68E-116	2.847419247	0.957	0.216	1.14E-112	Neural	1	Plp1
Sfrp5	1.14E-109	1.58800966	0.712	0.056	2.28E-106	Neural	1	Sfrp5
Kcna2	4.82E-109	2.186448896	0.878	0.156	9.63E-106	Neural	1	Kcna2
Nrn1	6.13E-105	2.342797933	0.878	0.197	1.23E-101	Neural	1	Nrn1
Kcna6	2.36E-101	1.290830044	0.727	0.132	4.72E-98	Neural	1	Kcna6
Art31	8.36E-101	2.415582991	0.935	0.234	1.67E-97	Neural	1	Art3
Vwa1	2.35E-97	2.65622349	0.942	0.258	4.71E-94	Neural	1	Vwa1
Cd59a5	2.38E-97	2.423817073	0.95	0.236	4.75E-94	Neural	1	Cd59a
Gpm6b4	8.10E-93	2.341703843	0.964	0.282	1.62E-89	Neural	1	Gpm6b
Mbp1	9.15E-93	3.370456047	0.914	0.229	1.83E-89	Neural	1	Mbp
Gatm3	1.09E-90	2.126217187	0.827	0.172	2.19E-87	Neural	1	Gatm
Snca	1.08E-86	1.149608352	0.676	0.108	2.16E-83	Neural	1	Snca
S100b1	1.33E-86	2.294779593	0.914	0.336	2.66E-83	Neural	1	S100b
Dbi3	1.91E-85	2.488730698	0.993	0.735	3.82E-82	Neural	1	Dbi
Prnp3	3.70E-83	3.15911649	0.942	0.381	7.40E-80	Neural	1	Prnp
Cadm11	1.80E-81	1.990613887	0.849	0.254	3.59E-78	Neural	1	Cadm1
Scn7a4	6.44E-80	2.230687147	0.921	0.275	1.29E-76	Neural	1	Scn7a
Itgb42	4.03E-76	0.798724397	0.655	0.101	8.06E-73	Neural	1	Itgb4
Cryab6	1.45E-74	2.111079772	0.95	0.531	2.89E-71	Neural	1	Cryab
Ptn4	2.45E-72	1.95880363	0.871	0.295	4.89E-69	Neural	1	Ptn
Cd910	1.79E-70	1.798677035	0.993	0.648	3.57E-67	Neural	1	Cd9
Cdh19	6.99E-70	1.504713393	0.662	0.092	1.40E-66	Neural	1	Cdh19
Ank32	1.51E-69	1.750848106	0.784	0.195	3.02E-66	Neural	1	Ank3
Stmn12	2.23E-67	1.525454177	0.842	0.208	4.47E-64	Neural	1	Stmn1
Acsbg1	5.73E-67	0.902549252	0.583	0.102	1.15E-63	Neural	1	Acsbg1
Wnt6	3.58E-64	0.983738737	0.604	0.078	7.16E-61	Neural	1	Wnt6
Mpz	2.18E-63	3.685681399	0.712	0.213	4.36E-60	Neural	1	Mpz
Ndrg15	3.58E-62	1.67763166	0.871	0.288	7.17E-59	Neural	1	Ndrg1
Mt3	1.10E-61	1.386200382	0.547	0.053	2.21E-58	Neural	1	Mt3
Rgs16	8.68E-60	0.273276514	0.518	0.102	1.74E-56	Neural	1	Rgs16
Pllp	1.33E-58	0.842264074	0.453	0.061	2.65E-55	Neural	1	Pllp
Egfl8	2.44E-58	1.550664946	0.705	0.187	4.88E-55	Neural	1	Egfl8
Scg3	7.76E-58	0.339618944	0.532	0.11	1.55E-54	Neural	1	Scg3
Kcnk11	1.97E-56	0.336450147	0.41	0.051	3.93E-53	Neural	1	Kcnk1
Matn22	3.22E-55	2.074220891	0.791	0.271	6.44E-52	Neural	1	Matn2
Klk82	7.55E-54	1.030682329	0.647	0.16	1.51E-50	Neural	1	Klk8
Tuba1a5	3.58E-53	1.470182097	0.964	0.631	7.15E-50	Neural	1	Tuba1a
Fabp53	4.59E-49	1.077252611	0.755	0.223	9.19E-46	Neural	1	Fabp5
Hmgcs22	7.04E-49	1.333993628	0.77	0.232	1.41E-45	Neural	1	Hmgcs2
Sfrp13	1.91E-48	1.585708662	0.799	0.279	3.82E-45	Neural	1	Sfrp1
Sparc5	8.00E-48	1.254631419	0.993	0.873	1.60E-44	Neural	1	Sparc
Diaph3	1.11E-47	0.277767127	0.525	0.106	2.22E-44	Neural	1	Diaph3
Il342	2.41E-47	0.535072866	0.561	0.132	4.82E-44	Neural	1	Il34
Apoe5	2.44E-46	1.461813761	0.964	0.843	4.88E-43	Neural	1	Apoe
Pmp224	6.54E-46	2.048030537	0.906	0.603	1.31E-42	Neural	1	Pmp22
Pdgfa6	2.60E-44	1.421977639	0.791	0.326	5.20E-41	Neural	1	Pdgfa
Kif21a	1.17E-43	0.612423962	0.576	0.156	2.35E-40	Neural	1	Kif21a
Ncmap1	1.63E-42	1.318494761	0.547	0.152	3.27E-39	Neural	1	Ncmap
Paqr62	1.93E-42	1.09802574	0.712	0.282	3.87E-39	Neural	1	Paqr6
Ngfr	2.00E-41	0.566951784	0.504	0.088	3.99E-38	Neural	1	Ngfr
Enho2	4.46E-41	0.635756778	0.59	0.175	8.91E-38	Neural	1	Enho
Ldhb4	7.86E-41	1.121606446	0.777	0.322	1.57E-37	Neural	1	Ldhb
S100a64	8.45E-38	0.739736936	1	0.906	1.69E-34	Neural	1	S100a6
Ptprz12	1.54E-37	0.803454132	0.604	0.17	3.08E-34	Neural	1	Ptprz1
Ephx21	1.98E-37	0.694796258	0.612	0.215	3.95E-34	Neural	1	Ephx2
Nkain41	4.05E-37	0.535335817	0.612	0.215	8.09E-34	Neural	1	Nkain4
Itih53	1.68E-36	1.313634871	0.777	0.427	3.36E-33	Neural	1	Itih5
Cdkn2c1	2.38E-35	0.889757267	0.712	0.333	4.76E-32	Neural	1	Cdkn2c
S100a45	5.33E-35	1.214399607	0.885	0.573	1.07E-31	Neural	1	S100a4
Dusp151	6.18E-35	0.386878827	0.36	0.06	1.24E-31	Neural	1	Dusp15
Gap433	1.03E-34	0.62600287	0.576	0.222	2.05E-31	Neural	1	Gap43
Apod3	1.05E-34	2.224655356	0.719	0.32	2.10E-31	Neural	1	Apod
C4b6	2.30E-33	1.159716313	0.763	0.341	4.59E-30	Neural	1	C4b
Mcam4	1.55E-32	0.795627461	0.698	0.274	3.10E-29	Neural	1	Mcam
Pdgfb1	5.13E-32	0.318758459	0.532	0.162	1.03E-28	Neural	1	Pdgfb
Slc9a3r23	8.51E-32	0.726989823	0.655	0.267	1.70E-28	Neural	1	Slc9a3r2
F33	1.28E-31	1.231818167	0.712	0.369	2.57E-28	Neural	1	F3
Tst2	9.47E-31	0.699384523	0.662	0.309	1.89E-27	Neural	1	Tst
Limch17	1.32E-30	1.060964045	0.748	0.342	2.64E-27	Neural	1	Limch1
Fscn12	2.51E-30	0.405758994	0.583	0.242	5.03E-27	Neural	1	Fscn1
Cadm32	2.75E-30	0.67479879	0.633	0.273	5.50E-27	Neural	1	Cadm3
Fam178b	3.10E-30	0.818748411	0.525	0.182	6.19E-27	Neural	1	Fam178b
Fcgr2b5	1.02E-29	0.815633161	0.64	0.249	2.04E-26	Neural	1	Fcgr2b
Tmem176a9	3.98E-28	0.86535863	0.748	0.344	7.97E-25	Neural	1	Tmem176a
Tspan74	5.72E-28	0.726944533	0.64	0.306	1.14E-24	Neural	1	Tspan7
Rarres25	1.45E-27	0.842110753	0.921	0.687	2.89E-24	Neural	1	Rarres2
Pdlim46	5.27E-27	1.250857999	0.719	0.433	1.05E-23	Neural	1	Pdlim4
Csrp15	4.35E-26	0.753497548	0.871	0.509	8.70E-23	Neural	1	Csrp1
Tnfaip61	1.13E-25	0.336936309	0.576	0.252	2.26E-22	Neural	1	Tnfaip6
Tmem176b10	1.40E-24	0.766561121	0.799	0.437	2.81E-21	Neural	1	Tmem176b
Aldh1a32	1.61E-24	0.32769925	0.554	0.236	3.22E-21	Neural	1	Aldh1a3
Fdps	1.61E-24	0.468228529	0.597	0.263	3.22E-21	Neural	1	Fdps
Malat13	3.27E-24	0.516314652	1	0.999	6.53E-21	Neural	1	Malat1
Arhgap314	5.64E-24	0.614895016	0.683	0.318	1.13E-20	Neural	1	Arhgap31
Socs31	7.05E-24	0.812231501	0.576	0.242	1.41E-20	Neural	1	Socs3
Sorbs26	1.33E-23	0.65142146	0.554	0.191	2.66E-20	Neural	1	Sorbs2
Nav24	2.90E-23	0.471872443	0.597	0.259	5.79E-20	Neural	1	Nav2
Reln1	5.34E-23	0.514750824	0.453	0.1	1.07E-19	Neural	1	Reln
Itm2a5	5.75E-23	1.000512178	0.741	0.471	1.15E-19	Neural	1	Itm2a
Ntng1	4.29E-22	0.666650774	0.504	0.172	8.58E-19	Neural	1	Ntng1
Aldh1a15	8.35E-22	1.026004971	0.633	0.304	1.67E-18	Neural	1	Aldh1a1
Igfbp78	8.87E-22	0.880812628	0.906	0.803	1.77E-18	Neural	1	Igfbp7
Fez13	1.44E-21	0.713214405	0.676	0.381	2.88E-18	Neural	1	Fez1
Tppp34	7.25E-21	1.013082857	0.849	0.577	1.45E-17	Neural	1	Tppp3
H2-D19	1.20E-20	0.769086286	0.957	0.89	2.40E-17	Neural	1	H2-D1
Itga61	1.56E-20	0.471928499	0.504	0.19	3.12E-17	Neural	1	Itga6
Nrp25	1.65E-20	0.397059373	0.54	0.228	3.30E-17	Neural	1	Nrp2
Ttyh2	2.17E-20	0.45864769	0.525	0.229	4.35E-17	Neural	1	Ttyh2
Anxa26	3.28E-20	0.633123319	0.906	0.667	6.56E-17	Neural	1	Anxa2
Ier34	3.30E-20	0.442302594	0.604	0.321	6.60E-17	Neural	1	Ier3
Rtp43	8.19E-20	0.40139201	0.547	0.238	1.64E-16	Neural	1	Rtp4
Prdx15	1.80E-19	0.629606505	0.921	0.797	3.61E-16	Neural	1	Prdx1
Dst5	3.91E-19	0.763661666	0.719	0.427	7.83E-16	Neural	1	Dst
B2m7	1.46E-18	0.494047518	0.993	0.983	2.93E-15	Neural	1	B2m
Prss237	6.07E-18	0.789260643	0.748	0.491	1.21E-14	Neural	1	Prss23
Gfra1	6.27E-18	0.316916674	0.432	0.14	1.25E-14	Neural	1	Gfra1
Mgll3	2.01E-17	0.510000952	0.698	0.402	4.02E-14	Neural	1	Mgll
Hspb25	2.59E-17	0.288823046	0.59	0.279	5.19E-14	Neural	1	Hspb2
Cox7a1	4.30E-17	0.404222803	0.511	0.21	8.61E-14	Neural	1	Cox7a1
Ckb7	1.49E-16	0.732900618	0.77	0.53	2.99E-13	Neural	1	Ckb
Atf31	1.56E-16	0.504881771	0.532	0.28	3.11E-13	Neural	1	Atf3
Plin22	1.78E-16	0.540734733	0.604	0.31	3.56E-13	Neural	1	Plin2
Col27a11	2.78E-16	0.635243586	0.583	0.296	5.57E-13	Neural	1	Col27a1
Capg3	2.02E-15	0.563634976	0.799	0.546	4.03E-12	Neural	1	Capg
Eng3	2.74E-15	0.670139589	0.619	0.335	5.48E-12	Neural	1	Eng
Prdx63	3.08E-15	0.754713784	0.777	0.569	6.16E-12	Neural	1	Prdx6

Pdzd21	5.92E-15	0.763315097	0.576	0.264	1.18E-11	Neural	1	Pdzd2
Vim6	6.73E-15	0.378604368	1	0.892	1.35E-11	Neural	1	Vim
Plpp17	7.11E-15	0.500909247	0.662	0.396	1.42E-11	Neural	1	Plpp1
Ndrg25	7.86E-15	0.734625028	0.655	0.405	1.57E-11	Neural	1	Ndrg2
Postn6	3.04E-14	1.015968451	0.705	0.473	6.08E-11	Neural	1	Postn
Uchl14	6.15E-14	0.428669133	0.532	0.263	1.23E-10	Neural	1	Uchl1
Ctsl5	1.20E-13	0.508471385	0.871	0.692	2.40E-10	Neural	1	Ctsl
Phgdh	4.60E-13	0.463942584	0.532	0.316	9.19E-10	Neural	1	Phgdh
Col28a1	7.59E-13	0.381488914	0.317	0.011	1.52E-09	Neural	1	Col28a1
Fos6	6.31E-12	0.937222947	0.727	0.506	1.26E-08	Neural	1	Fos
Junb4	6.99E-12	0.782653035	0.784	0.625	1.40E-08	Neural	1	Junb
Pappa	9.62E-12	0.332148739	0.417	0.152	1.92E-08	Neural	1	Pappa
Dmd5	1.72E-11	0.631753256	0.525	0.274	3.45E-08	Neural	1	Dmd
Timp37	1.88E-11	0.256320156	0.921	0.699	3.75E-08	Neural	1	Timp3
Ncam15	5.79E-11	0.828398051	0.583	0.297	1.16E-07	Neural	1	Ncam1
G0s25	6.37E-11	0.570214881	0.554	0.317	1.27E-07	Neural	1	G0s2
Ddit42	8.49E-11	0.403689503	0.439	0.219	1.70E-07	Neural	1	Ddit4
Lgmn5	1.49E-10	0.473840956	0.676	0.463	2.99E-07	Neural	1	Lgmn
Tubb52	3.70E-10	0.466072352	0.863	0.732	7.40E-07	Neural	1	Tubb5
Cdkn1a2	4.26E-10	0.856752907	0.532	0.264	8.52E-07	Neural	1	Cdkn1a
5-Jun	7.00E-10	0.715988215	0.705	0.524	1.40E-06	Neural	1	Jun
Serpine26	8.79E-10	0.456667107	0.655	0.421	1.76E-06	Neural	1	Serpine2
Iqgap23	1.60E-09	0.85600392	0.482	0.248	3.20E-06	Neural	1	Iqgap2
Abca8a6	2.83E-09	0.598839103	0.626	0.409	5.65E-06	Neural	1	Abca8a
Mdh12	4.77E-09	0.452628255	0.691	0.525	9.53E-06	Neural	1	Mdh1
Ccnd15	1.47E-07	0.503011952	0.496	0.287	0.000293115	Neural	1	Ccnd1
Fndc15	5.49E-07	0.388892906	0.655	0.471	0.001098687	Neural	1	Fndc1
Naaa2	7.28E-07	0.281093384	0.568	0.385	0.001456591	Neural	1	Naaa
Npy	7.33E-07	0.339887132	0.281	0.022	0.001465067	Neural	1	Npy
Lgals36	9.37E-07	0.766793937	0.518	0.342	0.001874395	Neural	1	Lgals3
Anxa15	1.06E-06	0.544902366	0.727	0.607	0.002122664	Neural	1	Anxa1
Zfp365	1.88E-06	0.282320114	0.619	0.468	0.003750581	Neural	1	Zfp36
Col18a15	3.97E-06	0.544520486	0.54	0.349	0.007939197	Neural	1	Col18a1
Ifit33	4.84E-06	0.388350685	0.201	0.257	0.009681686	Neural	1	Ifit3
Gm424183	6.21E-06	0.650753584	0.813	0.825	0.012422959	Neural	1	Gm42418
Entpd24	7.43E-06	0.396599004	0.612	0.444	0.014866803	Neural	1	Entpd2
Lamb14	2.53E-05	0.305334563	0.489	0.328	0.050657834	Neural	1	Lamb1
Ifit12	2.65E-05	0.423649509	0.388	0.22	0.052913105	Neural	1	Ifit1
Rgcc2	4.35E-05	0.419026691	0.41	0.214	0.086963642	Neural	1	Rgcc
Fbln56	5.86E-05	0.251316063	0.719	0.555	0.117115302	Neural	1	Fbln5
Fgl26	0.000126692	1.183027064	0.518	0.405	0.253384932	Neural	1	Fgl2
Sox45	0.000549683	0.420346446	0.46	0.314	1	Neural	1	Sox4
Efhd16	0.000815536	0.458277699	0.518	0.371	1	Neural	1	Efhd1
Tuba1b7	0.000931166	0.380175058	0.676	0.662	1	Neural	1	Tuba1b
Hes15	0.000985138	0.683828441	0.468	0.309	1	Neural	1	Hes1
Rassf45	0.003467543	0.371601096	0.432	0.299	1	Neural	1	Rassf4
Phlda15	0.005385056	0.329688721	0.525	0.397	1	Neural	1	Phlda1
Slc25a45	0.009678706	0.287070394	0.835	0.767	1	Neural	1	Slc25a4
Murc	0	0.507202591	0.731	0.007	0	Neural	2	Murc
Tmod4	1.22E-174	0.416350781	0.72	0.024	2.44E-171	Neural	2	Tmod4
Jsrp1	5.85E-165	1.426587257	0.828	0.055	1.17E-161	Neural	2	Jsrp1
Rpl3l	6.05E-140	1.344637173	0.817	0.043	1.21E-136	Neural	2	Rpl3l
Art1	1.10E-124	0.666044902	0.763	0.065	2.21E-121	Neural	2	Art1
Pvalb	3.58E-120	1.820912547	0.817	0.104	7.15E-117	Neural	2	Pvalb
Myh1	8.80E-120	0.838942606	0.753	0.093	1.76E-116	Neural	2	Myh1
Klhl41	7.24E-113	1.378099852	0.839	0.104	1.45E-109	Neural	2	Klhl41
Lmod2	9.95E-111	1.497701555	0.882	0.043	1.99E-107	Neural	2	Lmod2
Myoz1	7.96E-110	2.242375294	0.839	0.13	1.59E-106	Neural	2	Myoz1
Mybpc2	1.19E-108	0.654276432	0.731	0.097	2.37E-105	Neural	2	Mybpc2
Itgb1bp2	1.90E-97	1.021696071	0.796	0.084	3.81E-94	Neural	2	Itgb1bp2
Neb	4.93E-90	0.544863514	0.731	0.074	9.86E-87	Neural	2	Neb
Trdn	1.96E-89	1.840121632	0.925	0.084	3.93E-86	Neural	2	Trdn
Fitm1	7.75E-85	1.10856642	0.839	0.087	1.55E-81	Neural	2	Fitm1
Mlf11	5.88E-78	2.087932345	0.978	0.143	1.18E-74	Neural	2	Mlf1
Apobec2	9.33E-78	2.552392004	0.946	0.182	1.87E-74	Neural	2	Apobec2
Myl1	2.42E-77	4.192185878	1	0.198	4.84E-74	Neural	2	Myl1
Csrp3	1.52E-76	3.35499883	0.989	0.119	3.03E-73	Neural	2	Csrp3
Mb	1.49E-75	4.232791749	1	0.159	2.99E-72	Neural	2	Mb
Actn3	1.29E-74	1.901203762	0.828	0.181	2.58E-71	Neural	2	Actn3
Eno3	1.94E-73	3.160829807	0.978	0.188	3.87E-70	Neural	2	Eno3
Cox8b	2.24E-73	2.875253416	0.989	0.175	4.49E-70	Neural	2	Cox8b
Trim54	2.94E-73	1.316153977	0.806	0.045	5.89E-70	Neural	2	Trim54
Pgam2	4.05E-73	2.75802916	0.978	0.133	8.10E-70	Neural	2	Pgam2
Cox6a2	5.34E-73	3.321307618	0.989	0.157	1.07E-69	Neural	2	Cox6a2
Ckm	6.06E-73	4.547403	0.978	0.218	1.21E-69	Neural	2	Ckm
Cox7a11	2.94E-72	3.002030274	0.989	0.208	5.89E-69	Neural	2	Cox7a1
Smpx	9.61E-72	2.539513643	0.946	0.1	1.92E-68	Neural	2	Smpx
Sln	2.56E-67	4.266303627	0.978	0.244	5.12E-64	Neural	2	Sln
Tbx11	1.39E-66	0.877041813	0.774	0.133	2.77E-63	Neural	2	Tbx1
Myl3	2.27E-66	4.284844594	0.914	0.263	4.54E-63	Neural	2	Myl3
Tcap4	3.19E-66	3.05210068	1	0.375	6.37E-63	Neural	2	Tcap
Des4	1.01E-64	3.094366734	0.957	0.184	2.02E-61	Neural	2	Des
Chchd101	2.20E-64	2.511963623	0.978	0.313	4.40E-61	Neural	2	Chchd10
Adssl11	5.24E-64	1.647641623	0.892	0.233	1.05E-60	Neural	2	Adssl1
Actc12	8.93E-64	2.680806704	0.946	0.214	1.79E-60	Neural	2	Actc1
Tpm15	9.15E-64	3.204450976	1	0.602	1.83E-60	Neural	2	Tpm1
Prox11	3.73E-63	0.277288238	0.677	0.094	7.45E-60	Neural	2	Prox1
Slc25a46	3.15E-62	2.520704564	1	0.766	6.30E-59	Neural	2	Slc25a4
Aldoa5	9.92E-61	3.110916336	1	0.767	1.98E-57	Neural	2	Aldoa
Gapdh3	1.11E-60	2.917356867	1	0.81	2.21E-57	Neural	2	Gapdh
Cryab7	1.86E-60	2.113178463	1	0.532	3.71E-57	Neural	2	Cryab
Pygm1	2.02E-60	2.050566953	0.903	0.293	4.04E-57	Neural	2	Pygm
Fabp33	3.48E-58	2.344784656	0.892	0.188	6.95E-55	Neural	2	Fabp3
Ppp1r1a1	3.61E-57	0.758740172	0.774	0.166	7.22E-54	Neural	2	Ppp1r1a
H192	5.23E-57	1.808649729	0.839	0.26	1.05E-53	Neural	2	H19
Atcayos4	3.94E-56	1.355090791	0.828	0.184	7.88E-53	Neural	2	Atcayos
Hspb64	2.91E-54	1.881723841	0.957	0.311	5.81E-51	Neural	2	Hspb6
Tnnt3	6.44E-52	3.641539922	0.839	0.304	1.29E-48	Neural	2	Tnnt3
Tnni22	6.57E-52	3.619798737	0.86	0.364	1.31E-48	Neural	2	Tnni2
Myoz2	2.74E-51	2.076574124	0.86	0.176	5.48E-48	Neural	2	Myoz2
Acta1	1.29E-50	5.212163318	0.882	0.456	2.58E-47	Neural	2	Acta1
Mylpf	7.10E-50	3.801970047	0.839	0.354	1.42E-46	Neural	2	Mylpf
Car31	1.65E-49	2.439047151	0.839	0.359	3.29E-46	Neural	2	Car3
Tnnc2	1.72E-49	4.147681148	0.849	0.391	3.44E-46	Neural	2	Tnnc2
Tnni3	4.29E-47	2.869996925	0.806	0.217	8.59E-44	Neural	2	Tnni3
Pln	7.88E-45	1.976609849	0.774	0.15	1.58E-41	Neural	2	Pln
Tnnc1	6.87E-43	1.955391667	0.753	0.125	1.37E-39	Neural	2	Tnnc1
Myl2	1.05E-42	1.451023701	0.634	0.113	2.11E-39	Neural	2	Myl2
Cmbl1	3.43E-42	0.809283679	0.774	0.251	6.86E-39	Neural	2	Cmbl
Ddit4l1	3.46E-42	0.612999029	0.753	0.255	6.92E-39	Neural	2	Ddit4l
Tnnt23	6.75E-42	2.467868813	0.806	0.251	1.35E-38	Neural	2	Tnnt2
Myl4	1.66E-41	3.423737222	0.806	0.308	3.31E-38	Neural	2	Myl4
Asb24	3.92E-40	0.939760079	0.806	0.212	7.84E-37	Neural	2	Asb2
Ndrg26	8.69E-40	1.45886352	0.957	0.405	1.74E-36	Neural	2	Ndrg2
Tnnt1	2.67E-38	0.341509955	0.71	0.185	5.35E-35	Neural	2	Tnnt1
Casq12	3.11E-38	0.34433862	0.72	0.249	6.22E-35	Neural	2	Casq1
Hspb19	2.17E-37	1.330469529	0.914	0.489	4.34E-34	Neural	2	Hspb1
Myl71	9.35E-37	3.590391497	0.785	0.333	1.87E-33	Neural	2	Myl7
Hspb26	4.98E-34	1.025608235	0.86	0.278	9.95E-31	Neural	2	Hspb2
Mdh13	3.20E-30	1.302985803	0.892	0.524	6.39E-27	Neural	2	Mdh1
Tpm25	5.33E-28	1.809204121	0.882	0.578	1.07E-24	Neural	2	Tpm2
Gm15543	1.97E-27	1.111927904	0.602	0.062	3.94E-24	Neural	2	Gm15543
Mybphl	2.13E-27	1.186915871	0.645	0.117	4.26E-24	Neural	2	Mybphl
Sgcg4	2.38E-27	0.427373929	0.753	0.229	4.75E-24	Neural	2	Sgcg
Sh3bgr4	1.53E-23	0.583389303	0.839	0.282	3.07E-20	Neural	2	Sh3bgr
Myom14	1.98E-23	0.25030957	0.71	0.223	3.95E-20	Neural	2	Myom1
Myh6	9.11E-23	1.236665775	0.645	0.113	1.82E-19	Neural	2	Myh6
Ldhb5	1.54E-21	1.017848327	0.753	0.323	3.07E-18	Neural	2	Ldhb
Pdlim75	3.94E-21	0.871303481	0.871	0.416	7.88E-18	Neural	2	Pdlim7

Pdlim36	1.27E-20	0.625152156	0.785	0.289	2.54E-17	Neural	2	Pdlim3
Fbxo324	2.97E-20	0.323895641	0.731	0.305	5.93E-17	Neural	2	Fbxo32
Hspb74	5.94E-20	1.194277683	0.742	0.265	1.19E-16	Neural	2	Hspb7
Fabp3-ps1	4.42E-19	0.968039821	0.538	0.072	8.83E-16	Neural	2	Fabp3-ps1
Smarcd34	4.35E-18	0.331097205	0.72	0.287	8.70E-15	Neural	2	Smarcd3
Tubb4b5	1.06E-17	0.755806424	0.839	0.407	2.12E-14	Neural	2	Tubb4b
Cdkn1a3	1.47E-17	0.395478588	0.742	0.263	2.94E-14	Neural	2	Cdkn1a
Nppa	2.83E-14	2.388088257	0.581	0.213	5.66E-11	Neural	2	Nppa
Fhl22	4.30E-13	1.168212419	0.602	0.191	8.59E-10	Neural	2	Fhl2
Actn2	1.40E-12	1.208899828	0.613	0.087	2.80E-09	Neural	2	Actn2
Pitpnc14	3.13E-12	0.299199358	0.634	0.242	6.26E-09	Neural	2	Pitpnc1
Adprhl1	3.59E-12	0.876493466	0.505	0.056	7.19E-09	Neural	2	Adprhl1
Rrad4	4.14E-12	0.799451582	0.667	0.311	8.28E-09	Neural	2	Rrad
Ankrd11	4.15E-12	1.409883281	0.538	0.09	8.30E-09	Neural	2	Ankrd1
Eef1a2	1.16E-11	0.394572889	0.452	0.017	2.32E-08	Neural	2	Eef1a2
Selenbp15	2.92E-11	0.450039991	0.763	0.412	5.84E-08	Neural	2	Selenbp1
Ckmt2	3.92E-09	0.929220556	0.505	0.076	7.84E-06	Neural	2	Ckmt2
Atf52	5.93E-09	0.374785786	0.806	0.439	1.19E-05	Neural	2	Atf5
Tppp35	1.80E-08	0.498195611	0.892	0.577	3.59E-05	Neural	2	Tppp3
Prdx64	1.62E-06	0.358036648	0.828	0.569	0.003230038	Neural	2	Prdx6
Nexn5	2.82E-06	0.510998812	0.591	0.269	0.005630475	Neural	2	Nexn
Mlip	3.80E-06	0.585701906	0.258	0.075	0.007603876	Neural	2	Mlip
Ucp27	2.24E-05	0.566363472	0.677	0.44	0.044789289	Neural	2	Ucp2
Msrb15	0.000143339	0.417708759	0.667	0.5	0.286677052	Neural	2	Msrb1
Fhl15	0.000317265	0.314109605	0.828	0.558	0.634529292	Neural	2	Fhl1
Fndc5	0.002001303	0.65398157	0.43	0.13	1	Neural	2	Fndc5
Ephx22	0.002127908	0.310762029	0.29	0.217	1	Neural	2	Ephx2
Art32	0.003905707	0.439228052	0.29	0.238	1	Neural	2	Art3
C130080G10Rik	0.004835905	0.514151523	0.398	0.038	1	Neural	2	C130080G10Rik
Doc2g	0.005182485	0.879067944	0.409	0.093	1	Neural	2	Doc2g
Pcp4l16	0.005698858	0.275038087	0.505	0.257	1	Neural	2	Pcp4l1
Ehd45	0.009621886	0.514850691	0.548	0.388	1	Neural	2	Ehd4
Cd79a1	0	3.040138848	0.995	0.437	0	B	1	Cd79a
Cd79b2	0	2.618999816	0.964	0.395	0	B	1	Cd79b
Ly6d1	0	2.606932109	0.923	0.354	0	B	1	Ly6d
H2-DMb23	0	2.09272475	0.878	0.328	0	B	1	H2-DMb2
Fcmr1	0	2.057827735	0.789	0.362	0	B	1	Fcmr
Ms4a11	0	2.02406988	0.816	0.314	0	B	1	Ms4a1
Cd371	0	2.007311885	0.912	0.434	0	B	1	Cd37
Cd744	0	1.836621736	0.999	0.844	0	B	1	Cd74
Ltb2	0	1.789062537	0.835	0.392	0	B	1	Ltb
Cd524	0	1.775982686	0.991	0.58	0	B	1	Cd52
H2-Ob1	0	1.719907286	0.73	0.378	0	B	1	H2-Ob
Ptprcap2	0	1.673084384	0.847	0.402	0	B	1	Ptprcap
Coro1a4	0	1.625956374	0.933	0.489	0	B	1	Coro1a
Vpreb3	0	1.565918975	0.574	0.345	0	B	1	Vpreb3
H2-Aa5	0	1.543685552	0.996	0.643	0	B	1	H2-Aa
H2-Oa2	0	1.518389903	0.681	0.334	0	B	1	H2-Oa
Rac24	0	1.459787008	0.873	0.453	0	B	1	Rac2
Napsa2	0	1.458482279	0.669	0.253	0	B	1	Napsa
H2-Eb15	0	1.457748049	0.992	0.553	0	B	1	H2-Eb1
Hvcn11	0	1.438665414	0.63	0.363	0	B	1	Hvcn1
Siglecg1	0	1.432084956	0.611	0.248	0	B	1	Siglecg
Fcer2a	0	1.421797695	0.569	0.318	0	B	1	Fcer2a
H2-Ab15	0	1.41082815	0.993	0.652	0	B	1	H2-Ab1
Bank11	0	1.383070814	0.605	0.319	0	B	1	Bank1
Gimap14	0	1.375745021	0.683	0.341	0	B	1	Gimap1
Gm436031	0	1.302933163	0.539	0.23	0	B	1	Gm43603
Tnfrsf13c1	0	1.279028417	0.536	0.226	0	B	1	Tnfrsf13c
Gm432911	0	1.27677327	0.442	0.109	0	B	1	Gm43291
Arhgdib5	0	1.26843651	0.892	0.515	0	B	1	Arhgdib
Cd24a6	0	1.247119964	0.559	0.307	0	B	1	Cd24a
Ptpn63	0	1.237362748	0.616	0.361	0	B	1	Ptpn6
Gimap64	0	1.229934467	0.664	0.392	0	B	1	Gimap6
Pou2f23	0	1.22780302	0.589	0.318	0	B	1	Pou2f2
Limd23	0	1.197429671	0.71	0.48	0	B	1	Limd2
Mzb11	0	1.194092646	0.564	0.225	0	B	1	Mzb1
Apobec33	0	1.187563462	0.638	0.364	0	B	1	Apobec3
H2-DMa4	0	1.178131626	0.791	0.371	0	B	1	H2-DMa
Cd534	0	1.142890072	0.711	0.411	0	B	1	Cd53
Ly6e8	0	1.113888441	0.984	0.771	0	B	1	Ly6e
Cd721	0	1.095703327	0.485	0.248	0	B	1	Cd72
Il2rg2	0	1.068689912	0.562	0.353	0	B	1	Il2rg
Chchd102	0	1.055282621	0.561	0.284	0	B	1	Chchd10
Srgn6	0	1.035026446	0.727	0.47	0	B	1	Srgn
Irf84	0	0.994755422	0.566	0.31	0	B	1	Irf8
Sh3bgrl35	0	0.964082536	0.976	0.791	0	B	1	Sh3bgrl3
Gmfg7	0	0.84474443	0.643	0.37	0	B	1	Gmfg
Ctss5	0	0.733600664	0.684	0.383	0	B	1	Ctss
H2-K17	0	0.623448727	0.887	0.792	0	B	1	H2-K1
Tmsb105	0	0.484540188	0.999	0.944	0	B	1	Tmsb10
H2-D110	0	0.480797485	0.94	0.884	0	B	1	H2-D1
Ptpn186	3.37E-280	0.573868779	0.688	0.42	6.75E-277	B	1	Ptpn18
Ralgps21	1.45E-268	1.154995046	0.501	0.317	2.91E-265	B	1	Ralgps2
Fcrla	4.40E-268	1.085490931	0.484	0.286	8.80E-265	B	1	Fcrla
Lcp16	2.97E-261	0.826303033	0.594	0.407	5.94E-258	B	1	Lcp1
Psmb86	5.82E-254	0.685406394	0.674	0.522	1.16E-250	B	1	Psmb8
Cd19	1.05E-243	1.03571677	0.455	0.267	2.10E-240	B	1	Cd19
Tnfrsf13b1	4.02E-215	0.933832563	0.421	0.26	8.04E-212	B	1	Tnfrsf13b
Serpinb1a2	2.03E-213	0.902709437	0.449	0.309	4.06E-210	B	1	Serpinb1a
Cotl15	1.14E-208	0.626686736	0.639	0.49	2.28E-205	B	1	Cotl1
Bcl11a1	3.91E-206	1.006926833	0.426	0.244	7.82E-203	B	1	Bcl11a
Klf26	1.59E-203	0.645413583	0.67	0.552	3.18E-200	B	1	Klf2
Sell2	7.29E-200	1.052484391	0.483	0.341	1.46E-196	B	1	Sell
Gimap45	4.93E-194	0.888198106	0.461	0.302	9.85E-191	B	1	Gimap4
Lsp13	1.67E-183	0.457025238	0.718	0.618	3.34E-180	B	1	Lsp1
Laptm56	3.50E-183	0.482058587	0.581	0.409	6.99E-180	B	1	Laptm5
Blk	9.73E-171	0.679968723	0.31	0.138	1.95E-167	B	1	Blk
Spib	2.85E-166	0.915147177	0.39	0.229	5.69E-163	B	1	Spib
Rnase62	2.14E-164	0.743237883	0.389	0.255	4.28E-161	B	1	Rnase6
Ms4a4c1	1.65E-161	1.006552039	0.42	0.271	3.30E-158	B	1	Ms4a4c
Ptprc5	2.67E-159	0.804643597	0.529	0.426	5.35E-156	B	1	Ptprc
Ezr3	5.78E-146	0.753461381	0.404	0.296	1.16E-142	B	1	Ezr
Ucp28	8.92E-141	0.336280053	0.576	0.424	1.78E-137	B	1	Ucp2
Cr2	5.62E-125	0.650423364	0.289	0.144	1.12E-121	B	1	Cr2
Capg4	3.70E-114	0.646595102	0.573	0.544	7.41E-111	B	1	Capg
Unc93b14	2.96E-113	0.56715805	0.498	0.422	5.93E-110	B	1	Unc93b1
Cd832	2.07E-112	0.920679479	0.393	0.313	4.13E-109	B	1	Cd83
Cd23	2.56E-109	0.866148343	0.419	0.336	5.12E-106	B	1	Cd2
Cd552	2.85E-108	0.653623244	0.45	0.389	5.69E-105	B	1	Cd55
Plac83	3.61E-106	0.60052839	0.512	0.419	7.22E-103	B	1	Plac8
Lmo24	2.09E-103	0.609773891	0.474	0.419	4.17E-100	B	1	Lmo2
Cd383	7.16E-101	0.458817104	0.272	0.2	1.43E-97	B	1	Cd38
Acp52	1.34E-94	0.67426128	0.328	0.26	2.69E-91	B	1	Acp5
Il4i1	5.13E-93	0.600186987	0.287	0.193	1.03E-89	B	1	Il4i1
Pou2af1	1.37E-92	0.797526955	0.328	0.207	2.74E-89	B	1	Pou2af1
Ly865	5.43E-89	0.71952273	0.442	0.363	1.09E-85	B	1	Ly86
H2-Q76	8.88E-77	0.628689376	0.422	0.375	1.78E-73	B	1	H2-Q7
Cytip2	1.31E-74	0.64211936	0.345	0.275	2.62E-71	B	1	Cytip
Ncf41	5.05E-73	0.789045191	0.386	0.333	1.01E-69	B	1	Ncf4
Cd22	3.91E-67	0.736571865	0.31	0.21	7.81E-64	B	1	Cd22
Trp53i112	7.82E-62	0.44296015	0.261	0.238	1.56E-58	B	1	Trp53i11
Map4k12	2.22E-59	0.634935407	0.322	0.267	4.45E-56	B	1	Map4k1
Mndal3	8.29E-58	0.435313279	0.373	0.35	1.66E-54	B	1	Mndal
Ifi304	4.95E-52	0.676645682	0.417	0.419	9.90E-49	B	1	Ifi30
Nfkbid	7.53E-48	0.446422519	0.262	0.217	1.51E-44	B	1	Nfkbid
Ccr71	1.84E-43	0.748234683	0.349	0.311	3.68E-40	B	1	Ccr7
Ifi2034	6.39E-41	0.447525758	0.388	0.388	1.28E-37	B	1	Ifi203
Slamf61	8.08E-40	0.687911163	0.311	0.232	1.62E-36	B	1	Slamf6

Gimap32	3.67E-38	0.491916148	0.282	0.232	7.33E-35	B	1	Gimap3
Nfkbia3	4.90E-36	0.314953038	0.454	0.477	9.80E-33	B	1	Nfkbia
Scd11	1.46E-32	0.567227009	0.445	0.495	2.91E-29	B	1	Scd1
Dnajc7	6.44E-30	0.544077528	0.319	0.352	1.29E-26	B	1	Dnajc7
Cd844	4.80E-29	0.493531268	0.311	0.303	9.61E-26	B	1	Cd84
Slfn26	4.07E-25	0.342542173	0.274	0.302	8.15E-22	B	1	Slfn2
Gngt23	4.94E-25	0.3362681	0.296	0.315	9.88E-22	B	1	Gngt2
Marcksl14	2.52E-23	0.338803847	0.32	0.361	5.05E-20	B	1	Marcksl1
S1pr15	1.72E-19	0.458905876	0.283	0.298	3.44E-16	B	1	S1pr1
Rasgrp33	2.47E-18	0.396429456	0.246	0.253	4.95E-15	B	1	Rasgrp3
H2-Q63	2.06E-16	0.53236536	0.277	0.28	4.12E-13	B	1	H2-Q6
Arhgap305	2.23E-14	0.416860412	0.272	0.303	4.45E-11	B	1	Arhgap30
Cd486	5.60E-14	0.398847311	0.294	0.335	1.12E-10	B	1	Cd48
Cmah4	1.43E-11	0.327166948	0.23	0.392	2.86E-08	B	1	Cmah
a	0	0.351896427	0.725	0.006	0	B	2	a
Prg2	4.28E-207	1.990792729	0.913	0.077	8.56E-204	B	2	Prg2
Derl3	9.12E-177	1.812276334	0.935	0.055	1.82E-173	B	2	Derl3
Cacna1s	5.98E-158	1.237508414	0.819	0.029	1.20E-154	B	2	Cacna1s
Gm432912	3.21E-115	2.321638079	0.848	0.143	6.41E-112	B	2	Gm43291
Reln2	1.41E-113	0.253851264	0.717	0.098	2.82E-110	B	2	Reln
Mzb12	9.07E-109	3.332317257	1	0.259	1.81E-105	B	2	Mzb1
Pou2af11	6.83E-108	1.482981981	0.957	0.217	1.37E-104	B	2	Pou2af1
Jchain	5.61E-105	6.387881933	1	0.295	1.12E-101	B	2	Jchain
Kcnn43	2.04E-103	1.079899554	0.913	0.162	4.09E-100	B	2	Kcnn4
Eaf2	5.53E-98	1.53849168	0.855	0.094	1.11E-94	B	2	Eaf2
Sec11c2	1.69E-94	2.279257921	1	0.427	3.38E-91	B	2	Sec11c
Edem1	1.83E-94	1.951809107	0.993	0.323	3.65E-91	B	2	Edem1
Xbp11	2.40E-94	2.391842947	1	0.452	4.79E-91	B	2	Xbp1
Txndc51	1.70E-92	2.358980589	0.993	0.452	3.40E-89	B	2	Txndc5
Edem2	2.89E-89	1.697583755	0.964	0.274	5.78E-86	B	2	Edem2
Pafah1b31	6.75E-87	1.421056777	0.957	0.301	1.35E-83	B	2	Pafah1b3
B2m8	3.38E-86	1.795333299	1	0.983	6.77E-83	B	2	B2m
Serpina3g	6.59E-85	1.362055566	0.848	0.182	1.32E-81	B	2	Serpina3g
Ikzf3	1.17E-83	0.517916711	0.761	0.125	2.34E-80	B	2	Ikzf3
Slpi1	8.51E-74	3.313689226	0.848	0.2	1.70E-70	B	2	Slpi
Tspan135	1.13E-72	1.07237076	0.949	0.278	2.26E-69	B	2	Tspan13
Pim13	5.02E-72	1.562610002	0.928	0.265	1.00E-68	B	2	Pim1
Hsp90b13	1.39E-71	1.646437954	0.993	0.671	2.77E-68	B	2	Hsp90b1
Creld2	2.34E-69	1.355367826	0.92	0.212	4.69E-66	B	2	Creld2
Cytip3	1.02E-68	0.995075246	0.884	0.279	2.05E-65	B	2	Cytip
Pdia41	2.86E-68	1.705379091	0.92	0.362	5.72E-65	B	2	Pdia4
Srgn7	3.49E-67	1.428882998	0.993	0.496	6.97E-64	B	2	Srgn
Fcrla1	2.67E-64	1.039481026	0.826	0.306	5.34E-61	B	2	Fcrla
Sdf2l13	1.52E-63	1.50077971	0.92	0.279	3.04E-60	B	2	Sdf2l1
Ly6d2	5.20E-63	1.617733956	0.949	0.416	1.04E-59	B	2	Ly6d
Sfn1	2.07E-61	0.443780776	0.761	0.234	4.15E-58	B	2	Sfn
Cd79a2	4.11E-60	0.946289	1	0.498	8.21E-57	B	2	Cd79a
Sdc15	8.37E-60	1.16160975	0.862	0.306	1.67E-56	B	2	Sdc1
Trp53inp1	4.03E-59	1.3496308	0.87	0.249	8.06E-56	B	2	Trp53inp1
Samsn12	6.80E-59	0.400532295	0.768	0.219	1.36E-55	B	2	Samsn1
Chchd103	6.59E-56	0.926454386	0.92	0.312	1.32E-52	B	2	Chchd10
Gimap71	1.37E-55	0.411282087	0.717	0.191	2.75E-52	B	2	Gimap7
Slamf71	6.74E-55	1.095433112	0.761	0.163	1.35E-51	B	2	Slamf7
H2-Q77	5.10E-54	1.151560742	0.891	0.377	1.02E-50	B	2	H2-Q7
AI6622703	1.26E-53	1.202520989	0.841	0.296	2.52E-50	B	2	AI662270
Cd79b3	1.33E-53	0.839225408	0.957	0.457	2.65E-50	B	2	Cd79b
Gm5547	2.35E-53	0.92461234	0.71	0.121	4.69E-50	B	2	Gm5547
Ly6k	7.57E-51	0.638807652	0.63	0.118	1.51E-47	B	2	Ly6k
Selplg5	2.28E-50	0.591321266	0.877	0.294	4.57E-47	B	2	Selplg
Rasgrp34	6.58E-50	0.464800422	0.775	0.249	1.32E-46	B	2	Rasgrp3
Tnfrsf171	5.35E-49	1.141943435	0.703	0.086	1.07E-45	B	2	Tnfrsf17
Ly6e9	5.99E-48	0.967630729	1	0.794	1.20E-44	B	2	Ly6e
Ctss6	3.07E-47	1.073933998	0.935	0.415	6.13E-44	B	2	Ctss
Pycard4	5.19E-47	1.018735496	0.899	0.379	1.04E-43	B	2	Pycard
Adssl12	1.21E-46	0.278175907	0.725	0.233	2.41E-43	B	2	Adssl1
Cd24a7	5.56E-46	0.748355697	0.855	0.333	1.11E-42	B	2	Cd24a
Gimap15	1.17E-45	0.613132037	0.891	0.377	2.34E-42	B	2	Gimap1
Cd525	1.58E-45	1.485945646	1	0.624	3.17E-42	B	2	Cd52
Cybb6	8.35E-45	0.641851698	0.848	0.312	1.67E-41	B	2	Cybb
B930036N10Rik	1.11E-43	0.642575727	0.804	0.3	2.22E-40	B	2	B930036N10Rik
H2-K18	4.45E-43	1.119499711	0.986	0.802	8.90E-40	B	2	H2-K1
Oosp1	1.64E-42	0.682666739	0.522	0.01	3.27E-39	B	2	Oosp1
Dnajc71	8.04E-42	0.926866383	0.848	0.345	1.61E-38	B	2	Dnajc7
Ptprcap3	1.83E-41	0.794023174	0.906	0.451	3.65E-38	B	2	Ptprcap
Fdps1	8.41E-40	0.368840809	0.732	0.262	1.68E-36	B	2	Fdps
Ly6c21	9.69E-40	2.707089194	0.768	0.187	1.94E-36	B	2	Ly6c2
Spint28	1.41E-39	0.924264679	0.855	0.291	2.81E-36	B	2	Spint2
Bcl2l11	4.63E-39	0.518290202	0.681	0.249	9.27E-36	B	2	Bcl2l11
Rnase63	3.33E-38	0.966873849	0.768	0.268	6.67E-35	B	2	Rnase6
Itgb24	4.35E-38	0.307716914	0.739	0.255	8.71E-35	B	2	Itgb2
Apobec34	3.34E-37	0.720391033	0.87	0.392	6.67E-34	B	2	Apobec3
Phgdh1	1.88E-36	0.759234438	0.761	0.315	3.75E-33	B	2	Phgdh
Evi2a4	7.56E-36	0.520372679	0.804	0.363	1.51E-32	B	2	Evi2a
Vpreb31	6.44E-35	0.262213558	0.739	0.369	1.29E-31	B	2	Vpreb3
Ccr10	9.83E-35	0.388078726	0.507	0.045	1.97E-31	B	2	Ccr10
Cd745	2.21E-33	0.544963401	1	0.861	4.43E-30	B	2	Cd74
Tnfrsf13b2	2.94E-33	0.694795404	0.754	0.276	5.88E-30	B	2	Tnfrsf13b
Cd535	3.35E-33	0.577565066	0.906	0.443	6.71E-30	B	2	Cd53
Rgcc3	1.23E-32	0.975414861	0.746	0.212	2.45E-29	B	2	Rgcc
Trf6	1.42E-32	0.444718965	0.884	0.33	2.83E-29	B	2	Trf
Slc3a24	3.01E-30	1.080932735	0.819	0.438	6.01E-27	B	2	Slc3a2
Unc93b15	4.49E-29	0.551502057	0.855	0.428	8.98E-26	B	2	Unc93b1
Glipr13	7.17E-28	1.152299783	0.725	0.262	1.43E-24	B	2	Glipr1
Ly6a4	1.81E-27	0.66174525	0.978	0.66	3.63E-24	B	2	Ly6a
Ptpn64	3.04E-27	0.342875497	0.783	0.388	6.07E-24	B	2	Ptpn6
Limd24	2.20E-26	0.397354991	0.87	0.504	4.40E-23	B	2	Limd2
Cd24	2.46E-26	0.295887095	0.717	0.344	4.91E-23	B	2	Cd2
Hba-a26	1.16E-25	0.616898372	0.862	0.516	2.33E-22	B	2	Hba-a2
Psmb87	3.73E-24	0.615916364	0.891	0.537	7.46E-21	B	2	Psmb8
Cd281	5.36E-24	0.64887273	0.638	0.191	1.07E-20	B	2	Cd28
Psmb94	1.28E-22	0.600187498	0.797	0.432	2.56E-19	B	2	Psmb9
Hba-a16	4.38E-22	0.575473198	0.877	0.559	8.76E-19	B	2	Hba-a1
Hbb-bs7	6.97E-22	0.625551531	0.928	0.718	1.39E-18	B	2	Hbb-bs
Hbb-bt3	3.87E-21	0.462252285	0.797	0.426	7.73E-18	B	2	Hbb-bt
Plac84	9.01E-21	0.407118381	0.87	0.428	1.80E-17	B	2	Plac8
Hspa53	1.12E-20	0.638033983	0.891	0.592	2.24E-17	B	2	Hspa5
Fkbp11	1.30E-20	1.08216621	0.667	0.264	2.59E-17	B	2	Fkbp11
H2-D111	2.31E-18	0.594610399	0.978	0.89	4.62E-15	B	2	H2-D1
Cmah5	2.55E-18	0.576457791	0.717	0.371	5.10E-15	B	2	Cmah
Myl41	2.44E-15	0.272953565	0.688	0.308	4.89E-12	B	2	Myl4
Nfkbia4	1.10E-14	0.482291101	0.812	0.473	2.21E-11	B	2	Nfkbia
Tmem176a10	7.82E-14	0.686525759	0.739	0.344	1.56E-10	B	2	Tmem176a
Bst26	1.09E-12	0.538586014	0.833	0.494	2.17E-09	B	2	Bst2
Ralgps22	3.81E-10	0.374831639	0.616	0.336	7.61E-07	B	2	Ralgps2
Gapdh4	5.87E-10	0.29624795	0.964	0.81	1.17E-06	B	2	Gapdh
Tmem176b11	5.16E-09	0.400987046	0.746	0.437	1.03E-05	B	2	Tmem176b
Mdh14	3.54E-07	0.382698444	0.732	0.525	0.000708453	B	2	Mdh1
Ppa12	4.47E-06	0.366692148	0.529	0.267	0.008930498	B	2	Ppa1
Map4k13	0.000187203	0.29037749	0.355	0.273	0.374406474	B	2	Map4k1
Prlr1	0.000307872	0.279156792	0.319	0.104	0.615743128	B	2	Prlr
Cd69	0.00113971	0.297645827	0.297	0.126	1	B	2	Cd69
Cd79a3	2.45E-38	0.86303063	0.942	0.499	4.90E-35	B	3	Cd79a
Ly6d3	3.72E-33	0.778269568	0.874	0.417	7.44E-30	B	3	Ly6d
Cd79b4	3.02E-31	0.636378777	0.883	0.458	6.04E-28	B	3	Cd79b
H2-DMb24	7.57E-25	0.543068878	0.786	0.389	1.51E-21	B	3	H2-DMb2
Ltb3	8.67E-23	0.651210593	0.796	0.441	1.73E-19	B	3	Ltb
Cd746	2.41E-22	0.289485809	1	0.861	4.82E-19	B	3	Cd74
Ms4a12	5.98E-21	0.624534158	0.709	0.37	1.20E-17	B	3	Ms4a1
Cd526	1.55E-20	0.373168989	0.961	0.625	3.11E-17	B	3	Cd52

Cd372	1.58E-17	0.418467543	0.796	0.487	3.16E-14	B 3	Cd37
Fcmr2	3.21E-16	0.544693544	0.67	0.41	6.42E-13	B 3	Fcmr
Rac25	5.91E-15	0.385109011	0.806	0.499	1.18E-11	B 3	Rac2
Ptprcap4	2.21E-14	0.330217672	0.728	0.452	4.42E-11	B 3	Ptprcap
Coro1a5	2.87E-14	0.321384354	0.845	0.539	5.74E-11	B 3	Coro1a
Dcn6	4.69E-14	0.33973131	1	0.858	9.39E-11	B 3	Dcn
Clec3b4	9.11E-14	0.388865247	0.942	0.588	1.82E-10	B 3	Clec3b
Mzb13	4.37E-13	0.424722509	0.495	0.262	8.73E-10	B 3	Mzb1
Siglecg2	4.81E-13	0.480378794	0.534	0.289	9.62E-10	B 3	Siglecg
Bank12	8.67E-13	0.507286432	0.573	0.351	1.73E-09	B 3	Bank1
Gsn4	1.45E-12	0.302443044	1	0.939	2.89E-09	B 3	Gsn
Nbl15	1.46E-12	0.374450135	0.864	0.57	2.92E-09	B 3	Nbl1
H2-Ob2	2.63E-12	0.490591646	0.621	0.417	5.26E-09	B 3	H2-Ob
Nid13	4.04E-12	0.467471547	0.796	0.523	8.07E-09	B 3	Nid1
Igfbp64	5.04E-12	0.28381578	0.854	0.578	1.01E-08	B 3	Igfbp6
Napsa3	1.04E-11	0.348009854	0.544	0.3	2.08E-08	B 3	Napsa
Serping16	1.85E-11	0.359594732	0.942	0.669	3.69E-08	B 3	Serping1
Ly6a5	2.92E-11	0.409975933	0.903	0.661	5.84E-08	B 3	Ly6a
Gm432913	2.96E-11	0.45479854	0.369	0.146	5.92E-08	B 3	Gm43291
Plac85	1.44E-10	0.385751953	0.67	0.429	2.89E-07	B 3	Plac8
Srgn8	6.51E-10	0.269444774	0.738	0.498	1.30E-06	B 3	Srgn
Gimap16	8.18E-10	0.341945175	0.583	0.379	1.64E-06	B 3	Gimap1
Pi163	8.76E-10	0.440637642	0.864	0.684	1.75E-06	B 3	Pi16
Vpreb32	1.48E-09	0.43563217	0.534	0.37	2.95E-06	B 3	Vpreb3
Scara54	1.52E-09	0.34406182	0.699	0.456	3.04E-06	B 3	Scara5
Col14a15	2.92E-09	0.400660346	0.757	0.558	5.83E-06	B 3	Col14a1
Cd2483	3.30E-09	0.365455194	0.68	0.494	6.60E-06	B 3	Cd248
Fbln27	3.63E-09	0.378625886	0.592	0.381	7.26E-06	B 3	Fbln2
Cd347	6.01E-09	0.256843524	0.786	0.507	1.20E-05	B 3	Cd34
Dpep13	8.45E-09	0.401408532	0.583	0.373	1.69E-05	B 3	Dpep1
Pdgfra5	1.96E-08	0.304245382	0.689	0.476	3.92E-05	B 3	Pdgfra
Plpp35	3.13E-08	0.312448073	0.825	0.618	6.27E-05	B 3	Plpp3
Ptpn65	6.14E-08	0.338665694	0.534	0.389	0.000122877	B 3	Ptpn6
Pou2f24	7.01E-08	0.398805668	0.505	0.348	0.000140182	B 3	Pou2f2
C35	1.12E-07	0.281854295	0.718	0.532	0.000224647	B 3	C3
Fbn12	2.19E-07	0.452071267	0.738	0.604	0.000438232	B 3	Fbn1
Gfpt23	2.30E-07	0.324817183	0.417	0.287	0.000460821	B 3	Gfpt2
Cd553	3.08E-07	0.403172104	0.534	0.396	0.000616967	B 3	Cd55
Hvcn12	3.85E-07	0.436956181	0.505	0.393	0.00077013	B 3	Hvcn1
Tmem1003	4.19E-07	0.32340045	0.495	0.333	0.000838736	B 3	Tmem100
Ptprc6	7.47E-07	0.304673404	0.583	0.437	0.00149413	B 3	Ptprc
Entpd25	2.34E-06	0.250720502	0.583	0.444	0.004679478	B 3	Entpd2
Cygb4	4.33E-06	0.318421933	0.592	0.414	0.008660147	B 3	Cygb
Gimap65	5.55E-06	0.269176729	0.544	0.422	0.011097154	B 3	Gimap6
Fbln15	6.26E-06	0.294345366	0.728	0.517	0.012528052	B 3	Fbln1
Thbd6	7.37E-06	0.292262575	0.505	0.369	0.014740861	B 3	Thbd
Sell3	8.42E-06	0.296175261	0.456	0.356	0.016841711	B 3	Sell
Mfap54	9.45E-06	0.27333369	0.883	0.69	0.018906536	B 3	Mfap5
Adamts53	1.10E-05	0.324887908	0.563	0.431	0.021990152	B 3	Adamts5
Gm436032	1.18E-05	0.323334794	0.388	0.265	0.023676899	B 3	Gm43603
Bcl11a2	1.19E-05	0.363401095	0.369	0.264	0.023785431	B 3	Bcl11a
Spib1	2.42E-05	0.272944198	0.359	0.247	0.048397021	B 3	Spib
H2-Oa3	3.98E-05	0.367693491	0.466	0.374	0.079690762	B 3	H2-Oa
Gpx33	6.52E-05	0.284828855	0.718	0.547	0.130443738	B 3	Gpx3
Ralgps23	9.82E-05	0.327642017	0.398	0.338	0.19631868	B 3	Ralgps2
Fcer2a1	0.000126914	0.390382909	0.427	0.346	0.253828298	B 3	Fcer2a
Fndc16	0.000325789	0.266660064	0.534	0.472	0.651577828	B 3	Fndc1
Plat3	0.00058739	0.310829366	0.466	0.42	1	B 3	Plat
Cd833	0.000801338	0.368045133	0.35	0.322	1	B 3	Cd83
Phlda16	0.001374565	0.277787723	0.466	0.398	1	B 3	Phlda1
Gas15	0.001763962	0.257257873	0.505	0.426	1	B 3	Gas1
Cd722	0.002276489	0.255329816	0.311	0.275	1	B 3	Cd72
Cadm33	0.002917257	0.292821134	0.301	0.275	1	B 3	Cadm3
Pdia42	0.006901805	0.252479225	0.379	0.365	1	B 3	Pdia4
Pcsk63	0.007018625	0.260982704	0.437	0.425	1	B 3	Pcsk6
Cd221	0.00747387	0.376248311	0.272	0.221	1	B 3	Cd22
Igfbp56	0.007534525	0.344513545	0.573	0.538	1	B 3	Igfbp5
Gem3	0.007864206	0.264692545	0.388	0.382	1	B 3	Gem

Supplementary Table 3_EC.markers

	p_val	avg_logFC	pct.1	pct.2	p_val_adj	cluster	gene
Fabp4	2.17E-124	2.921672066	0.987	0.66	4.33E-121	EC 1	Fabp4
Ly6c1	1.26E-119	2.074865241	1	0.485	2.52E-116	EC 1	Ly6c1
Sparcl1	1.16E-116	2.158062898	0.937	0.161	2.31E-113	EC 1	Sparcl1
Rgcc	4.64E-116	2.464121238	0.939	0.317	9.27E-113	EC 1	Rgcc
Igfbp7	1.22E-114	2.124948865	0.996	0.821	2.45E-111	EC 1	Igfbp7
Cd36	4.21E-112	1.927845592	0.967	0.427	8.42E-109	EC 1	Cd36
Tcf15	2.52E-104	2.137015994	0.872	0.05	5.05E-101	EC 1	Tcf15
Flt1	3.95E-103	1.8298884	0.933	0.29	7.90E-100	EC 1	Flt1
Aplnr	3.09E-101	1.137798167	0.826	0.084	6.18E-98	EC 1	Aplnr
Gpihbp1	2.88E-99	1.956376874	0.941	0.412	5.75E-96	EC 1	Gpihbp1
Mgll	1.57E-91	1.518419213	0.956	0.467	3.13E-88	EC 1	Mgll
Cd300lg	1.81E-91	1.631134894	0.9	0.23	3.62E-88	EC 1	Cd300lg
Esm1	1.97E-90	0.574912858	0.678	0.013	3.94E-87	EC 1	Esm1
Scarb1	2.22E-90	1.634653698	0.887	0.417	4.44E-87	EC 1	Scarb1
AW112010	1.13E-89	1.757473394	0.941	0.641	2.26E-86	EC 1	AW112010
Tmem88	1.68E-88	1.344183061	0.858	0.182	3.36E-85	EC 1	Tmem88
Ly6a	1.52E-85	1.31462647	0.99	0.565	3.04E-82	EC 1	Ly6a
C1qtnf9	2.89E-81	1.707899834	0.797	0.04	5.79E-78	EC 1	C1qtnf9
Fam167b	1.42E-78	0.674488174	0.711	0.119	2.83E-75	EC 1	Fam167b
Cxcl12	8.15E-78	1.921714241	0.885	0.464	1.63E-74	EC 1	Cxcl12
Fam101b	1.00E-75	1.622599729	0.841	0.277	2.00E-72	EC 1	Fam101b
Tmsb4x	1.97E-75	0.691619317	1	1	3.93E-72	EC 1	Tmsb4x
Atf3	4.06E-75	0.294923235	0.68	0.098	8.11E-72	EC 1	Atf3
Rgs4	9.83E-74	0.334103657	0.628	0.063	1.97E-70	EC 1	Rgs4
Gm12002	1.12E-71	1.143481253	0.734	0.003	2.24E-68	EC 1	Gm12002
BC028528	1.28E-71	0.868338328	0.839	0.343	2.56E-68	EC 1	BC028528
Cox4i2	6.94E-70	0.352933781	0.613	0.053	1.39E-66	EC 1	Cox4i2
Col15a1	4.53E-69	0.645095296	0.772	0.232	9.06E-66	EC 1	Col15a1
Steap4	2.46E-67	0.428745883	0.628	0.074	4.92E-64	EC 1	Steap4
Pdzd2	6.14E-67	1.268600464	0.82	0.235	1.23E-63	EC 1	Pdzd2
Pcdh17	1.40E-64	0.651254291	0.745	0.274	2.80E-61	EC 1	Pcdh17
Sparc	1.73E-64	0.984427056	1	0.91	3.47E-61	EC 1	Sparc
Tspan13	1.79E-64	1.236598342	0.939	0.66	3.58E-61	EC 1	Tspan13
Col4a2	1.90E-63	1.169158259	0.854	0.327	3.80E-60	EC 1	Col4a2
Irf7	2.19E-60	0.45634317	0.669	0.103	4.38E-57	EC 1	Irf7
Robo4	9.39E-60	0.709889078	0.751	0.153	1.88E-56	EC 1	Robo4
Tmem252	3.72E-58	0.319673075	0.628	0.045	7.44E-55	EC 1	Tmem252
Ushbp1	2.83E-57	0.985106395	0.812	0.261	5.67E-54	EC 1	Ushbp1
C130074G19Rik	1.04E-56	0.714255953	0.822	0.274	2.08E-53	EC 1	C130074G19Rik
Id1	1.37E-56	1.162838646	0.925	0.499	2.73E-53	EC 1	Id1
Rsad2	3.48E-53	1.305261287	0.678	0.15	6.96E-50	EC 1	Rsad2
Abcc9	2.06E-50	0.317877744	0.575	0.058	4.12E-47	EC 1	Abcc9
Adgrf5	1.54E-49	0.995280555	0.847	0.538	3.09E-46	EC 1	Adgrf5
Btg2	2.25E-48	1.208128704	0.833	0.385	4.50E-45	EC 1	Btg2
Prnp	3.69E-47	0.521932112	0.747	0.274	7.38E-44	EC 1	Prnp
Cdh5	8.36E-47	0.774389869	0.906	0.718	1.67E-43	EC 1	Cdh5
Unc45b	1.04E-46	0.875965029	0.766	0.296	2.08E-43	EC 1	Unc45b
Higd1b	4.62E-46	0.472536687	0.596	0.011	9.23E-43	EC 1	Higd1b
1810011O10Rik	6.30E-46	1.284685664	0.703	0.164	1.26E-42	EC 1	1810011O10Rik
Esam	1.35E-45	0.922894947	0.927	0.631	2.71E-42	EC 1	Esam
Hspb1	1.10E-44	0.802409682	0.946	0.71	2.20E-41	EC 1	Hspb1
Cd200	1.33E-44	1.029856948	0.971	0.876	2.66E-41	EC 1	Cd200
Timp4	1.81E-44	1.597863201	0.697	0.161	3.61E-41	EC 1	Timp4
Cd38	4.03E-43	0.79626673	0.728	0.383	8.05E-40	EC 1	Cd38
Adgrl4	7.07E-43	0.954702143	0.732	0.206	1.41E-39	EC 1	Adgrl4
Kdr	2.04E-42	1.000923035	0.824	0.525	4.09E-39	EC 1	Kdr
Slc26a10	1.27E-40	0.504848679	0.582	0.005	2.55E-37	EC 1	Slc26a10
Notch3	1.51E-40	0.407416165	0.437	0.026	3.02E-37	EC 1	Notch3

Mcf2l	1.04E-39	1.044739317	0.651	0.108	2.08E-36	EC	1	Mcf2l
Plpp3	1.16E-39	1.070129212	0.889	0.549	2.33E-36	EC	1	Plpp3
Plk2	1.44E-38	0.478866837	0.709	0.24	2.88E-35	EC	1	Plk2
Exoc3l2	6.02E-38	0.364229179	0.644	0.28	1.20E-34	EC	1	Exoc3l2
Socs3	1.03E-37	0.354832856	0.607	0.211	2.06E-34	EC	1	Socs3
Art3	1.40E-37	0.417055308	0.676	0.391	2.80E-34	EC	1	Art3
Nid1	3.55E-36	0.468241074	0.772	0.293	7.10E-33	EC	1	Nid1
Ly6e	5.10E-36	0.576525462	0.998	0.968	1.02E-32	EC	1	Ly6e
Mndal	6.68E-36	0.998262621	0.724	0.472	1.34E-32	EC	1	Mndal
Csf2rb	1.51E-35	0.402209523	0.59	0.129	3.03E-32	EC	1	Csf2rb
Lpl	2.10E-35	1.09657763	0.768	0.517	4.21E-32	EC	1	Lpl
Pltp	6.73E-35	0.687308668	0.808	0.422	1.35E-31	EC	1	Pltp
Ablim3	1.52E-34	0.926490485	0.594	0.079	3.05E-31	EC	1	Ablim3
4-Sep	3.18E-34	0.937631285	0.791	0.609	6.37E-31	EC	1	4-Sep
Fabp5	5.36E-34	1.137182373	0.755	0.356	1.07E-30	EC	1	Fabp5
Thrsp	6.88E-34	1.321702278	0.672	0.208	1.38E-30	EC	1	Thrsp
Cd34	1.12E-33	0.815483827	0.923	0.691	2.25E-30	EC	1	Cd34
Itga6	3.65E-32	0.841090827	0.684	0.256	7.30E-29	EC	1	Itga6
Gda	7.14E-32	0.587341779	0.617	0.169	1.43E-28	EC	1	Gda
Rtp4	3.77E-31	0.621873756	0.659	0.301	7.53E-28	EC	1	Rtp4
Gimap6	8.73E-31	0.765546511	0.912	0.739	1.75E-27	EC	1	Gimap6
Cdkn1c	3.63E-30	0.713744147	0.803	0.443	7.27E-27	EC	1	Cdkn1c
Mcam	8.94E-30	0.341984715	0.684	0.338	1.79E-26	EC	1	Mcam
Id3	3.69E-29	0.657025479	0.975	0.786	7.38E-26	EC	1	Id3
Kitl	5.20E-29	0.879894311	0.688	0.38	1.04E-25	EC	1	Kitl
Nrarp	1.52E-28	0.480835843	0.626	0.338	3.04E-25	EC	1	Nrarp
Ifi44	2.13E-28	0.268609357	0.536	0.145	4.27E-25	EC	1	Ifi44
Rbp7	2.25E-28	1.406866628	0.63	0.182	4.50E-25	EC	1	Rbp7
Cyp1a1	6.49E-28	0.418432522	0.615	0.388	1.30E-24	EC	1	Cyp1a1
Tspan7	8.28E-28	0.637644417	0.843	0.483	1.66E-24	EC	1	Tspan7
Nes	2.47E-27	0.599440555	0.653	0.401	4.95E-24	EC	1	Nes
Arhgef15	2.90E-27	0.598556446	0.768	0.454	5.81E-24	EC	1	Arhgef15
Pdgfb	9.71E-27	0.689321329	0.565	0.179	1.94E-23	EC	1	Pdgfb
Fos	1.46E-26	0.733313227	0.768	0.47	2.92E-23	EC	1	Fos
Egfl7	4.82E-26	0.483434047	0.983	0.96	9.64E-23	EC	1	Egfl7
Pcp4l1	5.62E-25	0.254484455	0.768	0.38	1.12E-21	EC	1	Pcp4l1
Jam2	7.12E-25	0.670957177	0.766	0.546	1.42E-21	EC	1	Jam2
Grrp1	4.49E-24	0.683187876	0.757	0.515	8.98E-21	EC	1	Grrp1
B2m	1.49E-23	0.347394198	1	0.989	2.98E-20	EC	1	B2m
Sox17	1.81E-23	0.665401715	0.789	0.644	3.61E-20	EC	1	Sox17
Car4	2.90E-23	1.359177838	0.586	0.018	5.81E-20	EC	1	Car4
Ssu2	9.16E-23	0.323063894	0.464	0.029	1.83E-19	EC	1	Ssu2
Cfd	9.24E-23	0.425974917	0.64	0.42	1.85E-19	EC	1	Cfd
Igfbp3	1.47E-22	0.735945123	0.699	0.467	2.94E-19	EC	1	Igfbp3
Cyyr1	3.44E-22	0.799390917	0.707	0.417	6.89E-19	EC	1	Cyyr1
Arhgap31	3.84E-22	0.566261529	0.908	0.72	7.67E-19	EC	1	Arhgap31
Junb	8.86E-22	0.747819236	0.851	0.446	1.77E-18	EC	1	Junb
Aqp1	9.61E-22	1.279838191	0.858	0.76	1.92E-18	EC	1	Aqp1
Aqp7	1.38E-21	1.201587222	0.6	0.005	2.76E-18	EC	1	Aqp7
Dusp1	1.60E-20	0.676925925	0.69	0.422	3.20E-17	EC	1	Dusp1
Tmem204	2.15E-19	0.744413239	0.659	0.383	4.30E-16	EC	1	Tmem204
Srgn	2.56E-19	0.312431698	0.921	0.591	5.13E-16	EC	1	Srgn
Chchd10	6.94E-19	0.408141231	0.523	0.156	1.39E-15	EC	1	Chchd10
Slc9a3r2	7.28E-18	0.490676063	0.914	0.834	1.46E-14	EC	1	Slc9a3r2
Emcn	4.80E-17	0.543508579	0.902	0.609	9.61E-14	EC	1	Emcn
Zfp36	1.42E-16	0.52819376	0.782	0.628	2.85E-13	EC	1	Zfp36
Cxcl9	1.92E-16	0.435666109	0.561	0.259	3.83E-13	EC	1	Cxcl9
Ppic	2.29E-16	0.409365502	0.828	0.517	4.58E-13	EC	1	Ppic
Ndufa4l2	2.89E-16	0.51329434	0.393	0.079	5.78E-13	EC	1	Ndufa4l2
Gm13889	9.45E-16	0.276976418	0.469	0.158	1.89E-12	EC	1	Gm13889
Bcam	1.85E-15	0.469546854	0.858	0.541	3.70E-12	EC	1	Bcam
Nrep	3.98E-15	0.276705139	0.726	0.612	7.96E-12	EC	1	Nrep
Myo10	1.14E-14	0.881698871	0.494	0.053	2.28E-11	EC	1	Myo10
Ccdc85a	1.53E-14	0.694161498	0.513	0.026	3.05E-11	EC	1	Ccdc85a
Bst2	1.67E-14	0.559599007	0.902	0.765	3.33E-11	EC	1	Bst2
S1pr1	1.82E-14	0.508998682	0.766	0.625	3.63E-11	EC	1	S1pr1
Eng	3.50E-14	0.419173551	0.908	0.744	6.99E-11	EC	1	Eng
Ifi203	3.64E-14	0.802376072	0.6	0.332	7.29E-11	EC	1	Ifi203
Cd93	2.82E-13	0.352265365	0.893	0.617	5.65E-10	EC	1	Cd93
Col1a1	6.17E-13	0.273069162	0.68	0.414	1.23E-09	EC	1	Col1a1
Cav1	1.42E-12	0.33015824	0.946	0.939	2.84E-09	EC	1	Cav1
Pitpnc1	2.75E-12	0.459113712	0.701	0.478	5.50E-09	EC	1	Pitpnc1
Kank3	3.15E-11	0.423333078	0.768	0.615	6.30E-08	EC	1	Kank3
Cdkn1a	3.68E-11	0.473492843	0.588	0.311	7.36E-08	EC	1	Cdkn1a
Plcb4	4.80E-11	0.518304896	0.594	0.351	9.60E-08	EC	1	Plcb4
Tmem176a	6.05E-11	0.328960916	0.705	0.422	1.21E-07	EC	1	Tmem176a
8430408G22Rik	6.57E-11	0.798137676	0.594	0.467	1.31E-07	EC	1	8430408G22Rik
Egr1	7.89E-11	0.393328079	0.718	0.586	1.58E-07	EC	1	Egr1
Ier2	1.06E-10	0.430868662	0.839	0.62	2.12E-07	EC	1	Ier2
Ctla2a	4.19E-10	0.485249624	0.877	0.707	8.37E-07	EC	1	Ctla2a
Fscn1	6.64E-10	0.506321352	0.615	0.327	1.33E-06	EC	1	Fscn1
Ier3	9.55E-10	0.260749049	0.663	0.47	1.91E-06	EC	1	Ier3
Sema3g	9.92E-10	0.322191997	0.462	0.285	1.98E-06	EC	1	Sema3g
Jun	1.19E-09	0.675848556	0.776	0.575	2.38E-06	EC	1	Jun
Iigp1	6.68E-09	0.392651864	0.736	0.509	1.34E-05	EC	1	Iigp1
Myl4	7.16E-09	0.554331698	0.454	0.314	1.43E-05	EC	1	Myl4
Sdc3	1.78E-08	0.417490361	0.642	0.538	3.57E-05	EC	1	Sdc3
Rasip1	8.21E-08	0.300673625	0.755	0.591	0.000164158	EC	1	Rasip1
Rgs5	2.08E-07	1.231367722	0.462	0.214	0.000416895	EC	1	Rgs5
Nfkbia	2.15E-07	0.310936333	0.837	0.757	0.000430733	EC	1	Nfkbia
Tsc22d1	3.02E-07	0.277260251	0.847	0.76	0.000603572	EC	1	Tsc22d1
Psmb8	3.55E-07	0.301714586	0.82	0.66	0.00070931	EC	1	Psmb8
Pde2a	7.19E-07	0.535554697	0.508	0.235	0.001438822	EC	1	Pde2a
Tinagl1	1.68E-06	0.26343719	0.822	0.691	0.003368838	EC	1	Tinagl1
Abcb1b	1.92E-06	0.47269962	0.421	0.185	0.003838858	EC	1	Abcb1b
Slc6a6	2.51E-06	0.974488528	0.483	0.261	0.005012961	EC	1	Slc6a6
C3	1.08E-05	0.307406881	0.54	0.385	0.021698773	EC	1	C3
Cygb	2.06E-05	0.332213877	0.502	0.354	0.041109758	EC	1	Cygb
Cmpk2	2.27E-05	0.275579821	0.45	0.319	0.045418829	EC	1	Cmpk2
Gimap1	3.84E-05	0.262722511	0.789	0.599	0.076841965	EC	1	Gimap1
Tpm1	7.00E-05	0.309094304	0.743	0.668	0.139981426	EC	1	Tpm1
Clic5	0.000189599	0.297923512	0.588	0.475	0.379198885	EC	1	Clic5
Myl7	0.000193414	0.396544161	0.485	0.354	0.386828228	EC	1	Myl7
Apobec3	0.00026943	0.267258805	0.515	0.361	0.538859559	EC	1	Apobec3
Cldn15	0.00055272	0.28662838	0.559	0.512	1	EC	1	Cldn15
Lmo7	0.00058859	0.363471664	0.345	0.111	1	EC	1	Lmo7
Car3	0.000590143	0.293365454	0.331	0.259	1	EC	1	Car3
Podxl	0.000741871	0.256164169	0.686	0.58	1	EC	1	Podxl
Mylk	0.001775674	0.293948261	0.383	0.317	1	EC	1	Mylk
Filip1l	0.00214373	0.298575024	0.395	0.272	1	EC	1	Filip1l
Hspa1a	0.002566762	0.531745162	0.351	0.364	1	EC	1	Hspa1a
Sgk1	0.003439924	0.347460561	0.563	0.404	1	EC	1	Sgk1
Vwa1	0.003787045	0.538735297	0.452	0.298	1	EC	1	Vwa1
Psmb9	0.004870171	0.25783628	0.657	0.509	1	EC	1	Psmb9
Btnl9	0.007768252	0.580686341	0.473	0.385	1	EC	1	Btnl9
Cytl1	1.49E-94	3.988999449	0.972	0.614	2.97E-91	EC	2	Cytl1
Cpe	1.17E-87	2.060841414	0.981	0.692	2.34E-84	EC	2	Cpe
Sfrp1	3.04E-83	2.151518542	0.86	0.146	6.08E-80	EC	2	Sfrp1
Gxylt2	9.98E-81	1.78760467	0.865	0.176	2.00E-77	EC	2	Gxylt2
Bgn	3.66E-80	1.962706714	0.963	0.632	7.31E-77	EC	2	Bgn
Gkn3	5.99E-80	2.44497638	0.823	0.268	1.20E-76	EC	2	Gkn3
Mgp	9.65E-79	1.660902822	1	0.975	1.93E-75	EC	2	Mgp
Clu	1.86E-78	2.523700184	0.949	0.422	3.72E-75	EC	2	Clu
Rbp1	5.23E-72	1.776244904	0.926	0.435	1.05E-68	EC	2	Rbp1
Vcam1	4.01E-71	1.991057171	0.851	0.246	8.02E-68	EC	2	Vcam1
Cfh	3.57E-70	1.768351868	0.94	0.436	7.14E-67	EC	2	Cfh
Pecam1	9.49E-70	1.165193368	0.991	0.889	1.90E-66	EC	2	Pecam1
Bmx	2.49E-66	1.393934376	0.847	0.299	4.98E-63	EC	2	Bmx
AU021092	5.45E-66	0.983718269	0.758	0.202	1.09E-62	EC	2	AU021092

Pam	1.34E-65	2.031765267	0.94	0.57	2.68E-62	EC	2	Pam
Adh7	6.28E-65	1.12207498	0.702	0.168	1.26E-61	EC	2	Adh7
Itgbl1	1.06E-62	0.5262354	0.665	0.067	2.13E-59	EC	2	Itgbl1
Ncam1	9.78E-62	0.813412905	0.753	0.232	1.96E-58	EC	2	Ncam1
Klk8	4.85E-59	1.038633821	0.753	0.223	9.71E-56	EC	2	Klk8
Klk10	1.91E-58	0.906717168	0.586	0.033	3.81E-55	EC	2	Klk10
Perp	1.16E-57	0.601002087	0.656	0.126	2.31E-54	EC	2	Perp
Eln	2.16E-57	1.75559553	0.93	0.606	4.33E-54	EC	2	Eln
Htra1	6.91E-57	1.070679445	0.823	0.338	1.38E-53	EC	2	Htra1
Spon1	7.36E-57	1.289158697	0.791	0.355	1.47E-53	EC	2	Spon1
Ptgis	9.61E-57	1.11925613	0.823	0.243	1.92E-53	EC	2	Ptgis
Vwf	1.74E-56	1.717546438	0.874	0.587	3.47E-53	EC	2	Vwf
Bmp4	1.76E-56	1.864612512	0.842	0.517	3.52E-53	EC	2	Bmp4
Scx	3.38E-56	0.313747476	0.619	0.109	6.76E-53	EC	2	Scx
Gja5	4.59E-56	1.135153611	0.758	0.299	9.18E-53	EC	2	Gja5
Lox	1.13E-55	0.783874269	0.665	0.145	2.25E-52	EC	2	Lox
Prr15	2.15E-54	0.451717806	0.651	0.173	4.31E-51	EC	2	Prr15
Tm4sf1	5.65E-52	1.261854368	0.986	0.927	1.13E-48	EC	2	Tm4sf1
Lmo2	6.33E-52	1.468646599	0.912	0.674	1.27E-48	EC	2	Lmo2
1500015O10Rik	2.86E-51	1.49065066	0.712	0.333	5.72E-48	EC	2	1500015O10Rik
Cgnl1	1.53E-48	1.244411011	0.8	0.254	3.07E-45	EC	2	Cgnl1
Selp	1.26E-47	0.723085074	0.665	0.218	2.52E-44	EC	2	Selp
Kcnn4	1.61E-47	0.529190368	0.679	0.251	3.21E-44	EC	2	Kcnn4
Fez1	9.58E-47	0.447017393	0.684	0.283	1.92E-43	EC	2	Fez1
Dkk3	1.89E-46	0.292608817	0.586	0.117	3.78E-43	EC	2	Dkk3
Fbln5	1.20E-45	1.271348451	0.828	0.301	2.40E-42	EC	2	Fbln5
Col5a2	1.41E-45	0.669972257	0.781	0.394	2.81E-42	EC	2	Col5a2
Sod3	2.50E-45	1.103088013	0.795	0.327	4.99E-42	EC	2	Sod3
Gata3	2.81E-45	0.326211448	0.633	0.237	5.62E-42	EC	2	Gata3
Ephx2	2.90E-45	1.172184016	0.74	0.46	5.81E-42	EC	2	Ephx2
Slco2a1	2.87E-44	0.934553541	0.837	0.54	5.74E-41	EC	2	Slco2a1
Fstl1	4.24E-43	1.01023464	0.879	0.512	8.47E-40	EC	2	Fstl1
Fblim1	2.28E-42	0.788534671	0.772	0.285	4.57E-39	EC	2	Fblim1
Cthrc1	7.66E-42	0.337479321	0.563	0.047	1.53E-38	EC	2	Cthrc1
Apoe	6.35E-40	1.338292453	0.953	0.919	1.27E-36	EC	2	Apoe
Mical2	1.10E-39	0.683421213	0.767	0.346	2.19E-36	EC	2	Mical2
Smoc1	4.43E-39	1.045014586	0.73	0.445	8.86E-36	EC	2	Smoc1
Adh1	3.19E-38	0.86553685	0.786	0.408	6.39E-35	EC	2	Adh1
Fhl1	4.77E-37	0.842574747	0.828	0.389	9.53E-34	EC	2	Fhl1
Krt18	3.22E-36	0.346486011	0.54	0.109	6.44E-33	EC	2	Krt18
Cdh11	1.42E-35	0.257851798	0.572	0.252	2.84E-32	EC	2	Cdh11
Car8	4.86E-35	0.939369525	0.823	0.495	9.71E-32	EC	2	Car8
Sfrp2	6.44E-35	0.511187205	0.633	0.324	1.29E-31	EC	2	Sfrp2
Bace2	1.47E-34	0.748877576	0.665	0.308	2.93E-31	EC	2	Bace2
Nupr1	3.62E-34	0.881049418	0.837	0.439	7.23E-31	EC	2	Nupr1
Pde3a	5.81E-34	1.063587084	0.702	0.249	1.16E-30	EC	2	Pde3a
Tmem158	7.39E-34	1.064600631	0.702	0.371	1.48E-30	EC	2	Tmem158
Cxcl1	1.22E-33	0.266636264	0.595	0.336	2.43E-30	EC	2	Cxcl1
Atp1b1	1.70E-33	0.692852458	0.721	0.519	3.41E-30	EC	2	Atp1b1
Stab1	1.89E-33	0.674598155	0.856	0.419	3.77E-30	EC	2	Stab1
Procr	4.56E-33	1.135562722	0.791	0.693	9.11E-30	EC	2	Procr
Selenbp1	3.44E-32	0.343897787	0.591	0.24	6.88E-29	EC	2	Selenbp1
Thbs1	1.91E-31	1.084287895	0.591	0.252	3.83E-28	EC	2	Thbs1
Pcolce2	2.43E-31	0.76524824	0.707	0.458	4.85E-28	EC	2	Pcolce2
Cd24a	2.82E-31	1.159308173	0.814	0.695	5.64E-28	EC	2	Cd24a
Pdlim3	1.26E-30	0.590050702	0.66	0.302	2.52E-27	EC	2	Pdlim3
Ccdc80	1.07E-28	0.846747541	0.698	0.265	2.13E-25	EC	2	Ccdc80
Tek	2.42E-28	0.734223416	0.842	0.494	4.84E-25	EC	2	Tek
Mfap5	3.33E-28	0.744969634	0.805	0.449	6.65E-25	EC	2	Mfap5
Mmp2	5.87E-28	0.278303061	0.693	0.4	1.17E-24	EC	2	Mmp2
Ass1	8.91E-28	0.580729862	0.693	0.417	1.78E-24	EC	2	Ass1
Gm2a	5.81E-27	0.356232358	0.721	0.405	1.16E-23	EC	2	Gm2a
Pla2g5	2.71E-26	0.34203559	0.614	0.416	5.41E-23	EC	2	Pla2g5
Cdc42ep3	6.00E-26	0.775951012	0.837	0.589	1.20E-22	EC	2	Cdc42ep3
Metrnl	6.12E-26	0.365751258	0.656	0.441	1.22E-22	EC	2	Metrnl
Icam2	1.26E-25	0.893843937	0.87	0.676	2.53E-22	EC	2	Icam2
Meg3	2.20E-25	0.395654322	0.605	0.24	4.41E-22	EC	2	Meg3
G0s2	2.72E-25	0.456960758	0.66	0.358	5.44E-22	EC	2	G0s2
Fn1	6.30E-25	0.834027586	0.805	0.492	1.26E-21	EC	2	Fn1
Ehd4	6.85E-25	0.611902063	0.86	0.731	1.37E-21	EC	2	Ehd4
Anxa3	1.74E-24	0.75402898	0.847	0.555	3.47E-21	EC	2	Anxa3
Emb	2.70E-24	0.30993347	0.484	0.136	5.39E-21	EC	2	Emb
Gja4	7.90E-24	0.814830835	0.758	0.606	1.58E-20	EC	2	Gja4
Ptprb	1.07E-23	0.741337039	0.935	0.765	2.15E-20	EC	2	Ptprb
Dbp	1.60E-23	0.481699082	0.874	0.751	3.20E-20	EC	2	Dbp
Gstm1	6.12E-23	0.611782312	0.893	0.673	1.22E-19	EC	2	Gstm1
Nos3	4.51E-22	0.750813961	0.763	0.611	9.03E-19	EC	2	Nos3
H2-Q6	1.86E-21	0.503632704	0.763	0.555	3.71E-18	EC	2	H2-Q6
Pdgfa	4.62E-21	0.326941174	0.66	0.417	9.24E-18	EC	2	Pdgfa
Smim5	8.43E-21	0.254931406	0.633	0.503	1.69E-17	EC	2	Smim5
Cbfa2t3	1.24E-20	0.594264265	0.758	0.438	2.49E-17	EC	2	Cbfa2t3
Lmcd1	1.95E-20	0.497622621	0.805	0.486	3.91E-17	EC	2	Lmcd1
Vim	1.92E-19	0.443624728	0.991	0.925	3.84E-16	EC	2	Vim
Aldh3a1	5.06E-19	0.387299341	0.6	0.467	1.01E-15	EC	2	Aldh3a1
Mt1	2.96E-18	0.787918657	0.716	0.463	5.91E-15	EC	2	Mt1
Pi16	2.22E-17	0.35394726	0.795	0.519	4.43E-14	EC	2	Pi16
Mfge8	3.23E-17	0.495797001	0.828	0.595	6.47E-14	EC	2	Mfge8
Ptgs1	4.50E-17	0.894393427	0.674	0.522	8.99E-14	EC	2	Ptgs1
Itgb1	1.42E-16	0.476675072	0.842	0.701	2.84E-13	EC	2	Itgb1
Hmgn2	2.79E-16	0.354665783	0.744	0.6	5.58E-13	EC	2	Hmgn2
Arhgdib	1.05E-15	0.407484731	0.735	0.506	2.11E-12	EC	2	Arhgdib
Spint2	2.49E-15	0.370670525	0.633	0.386	4.98E-12	EC	2	Spint2
Actn1	2.50E-15	0.529375084	0.679	0.435	5.00E-12	EC	2	Actn1
Itga5	1.19E-14	0.320581596	0.437	0.237	2.39E-11	EC	2	Itga5
H2-DMb1	1.20E-14	0.40890397	0.647	0.586	2.39E-11	EC	2	H2-DMb1
Tnfaip2	1.52E-14	0.449743159	0.684	0.598	3.03E-11	EC	2	Tnfaip2
Mmrn2	7.27E-14	0.495814395	0.935	0.798	1.45E-10	EC	2	Mmrn2
H2-K1	8.16E-14	0.442990181	0.981	0.935	1.63E-10	EC	2	H2-K1
Serpinb6b	1.05E-13	0.763749338	0.54	0.298	2.10E-10	EC	2	Serpinb6b
Dhh	1.57E-13	0.515108965	0.563	0.417	3.15E-10	EC	2	Dhh
Itgb4	9.82E-13	0.487477842	0.591	0.397	1.96E-09	EC	2	Itgb4
Ece1	1.72E-12	0.439944698	0.856	0.847	3.44E-09	EC	2	Ece1
Cst3	2.17E-12	0.455121543	0.995	0.998	4.33E-09	EC	2	Cst3
Epas1	3.58E-12	0.366033028	0.935	0.724	7.15E-09	EC	2	Epas1
Fxyd5	5.20E-12	0.552581978	0.814	0.754	1.04E-08	EC	2	Fxyd5
Ahsg	7.03E-12	0.658403876	0.419	0.039	1.41E-08	EC	2	Ahsg
Sdc1	1.68E-10	0.406627348	0.544	0.393	3.37E-07	EC	2	Sdc1
Clec14a	6.13E-10	0.497438389	0.74	0.699	1.23E-06	EC	2	Clec14a
Fbln2	7.51E-10	0.430558976	0.693	0.428	1.50E-06	EC	2	Fbln2
Tgm2	2.06E-09	0.373681301	0.879	0.743	4.12E-06	EC	2	Tgm2
F2r	8.79E-09	0.365946149	0.47	0.249	1.76E-05	EC	2	F2r
Dcn	1.10E-08	0.755903787	0.86	0.776	2.20E-05	EC	2	Dcn
Ptprr	1.38E-08	0.305381666	0.591	0.477	2.76E-05	EC	2	Ptprr
H2-Eb1	1.79E-08	0.270818525	0.702	0.539	3.58E-05	EC	2	H2-Eb1
Retnla	4.09E-08	0.282749971	0.684	0.54	8.19E-05	EC	2	Retnla
Mt2	1.17E-07	0.519268768	0.386	0.248	0.000233071	EC	2	Mt2
Cdh13	4.20E-07	0.513400353	0.688	0.516	0.000839821	EC	2	Cdh13
Ninj1	1.02E-06	0.340097519	0.642	0.519	0.00203491	EC	2	Ninj1
Hspa5	1.04E-06	0.363591548	0.679	0.592	0.002081096	EC	2	Hspa5
Mgst1	1.80E-06	0.434860247	0.633	0.519	0.003591961	EC	2	Mgst1
Acer2	2.86E-06	0.459223521	0.456	0.231	0.005713649	EC	2	Acer2
Hsp90b1	3.16E-06	0.263228595	0.767	0.712	0.006314366	EC	2	Hsp90b1
Mal	1.04E-05	0.461853098	0.27	0.202	0.020845881	EC	2	Mal
Klf2	1.29E-05	0.277326754	0.898	0.832	0.025792325	EC	2	Klf2
Cd74	1.87E-05	0.27935668	0.902	0.824	0.037370394	EC	2	Cd74
Dst	3.12E-05	0.27702244	0.623	0.544	0.062431043	EC	2	Dst
Srgn1	3.42E-05	0.304421523	0.851	0.749	0.068362134	EC	2	Srgn
H2-D1	7.01E-05	0.26168625	0.986	0.986	0.140199865	EC	2	H2-D1
Ecm1	7.54E-05	0.422937031	0.516	0.46	0.150840246	EC	2	Ecm1

Ramp2	0.000112599	0.255895144	0.981	0.966	0.225198288	EC	2	Ramp2
Kank1	0.000135563	0.376094662	0.451	0.259	0.271126493	EC	2	Kank1
Crip1	0.00015752	0.302961157	1	0.98	0.315039288	EC	2	Crip1
Tmem2	0.000514283	0.595916074	0.549	0.528	1	EC	2	Tmem2
Id2	0.000980254	0.574149311	0.614	0.648	1	EC	2	Id2
Stmn2	0.002625529	0.292620187	0.535	0.444	1	EC	2	Stmn2
Txndc5	0.002967889	0.316389689	0.465	0.382	1	EC	2	Txndc5
Dtx1	1.89E-95	1.466026683	0.854	0.144	3.78E-92	EC	3	Dtx1
Mmrn1	1.06E-87	2.94888501	0.963	0.325	2.12E-84	EC	3	Mmrn1
Reln	3.15E-82	1.983491536	0.805	0.166	6.30E-79	EC	3	Reln
Pard6g	3.83E-76	2.173233605	0.939	0.524	7.65E-73	EC	3	Pard6g
Lbp	5.20E-72	1.986163828	0.933	0.466	1.04E-68	EC	3	Lbp
Maf	3.22E-71	1.886136379	0.921	0.352	6.45E-68	EC	3	Maf
Thy1	2.70E-66	1.818662758	0.884	0.351	5.39E-63	EC	3	Thy1
Ccl21a	3.92E-66	4.670766305	0.854	0.24	7.84E-63	EC	3	Ccl21a
Cldn5	3.46E-65	1.676313595	0.994	0.717	6.91E-62	EC	3	Cldn5
Fxyd6	8.93E-65	1.868606941	0.957	0.651	1.79E-61	EC	3	Fxyd6
Fgl2	1.76E-63	2.172517034	0.884	0.365	3.51E-60	EC	3	Fgl2
Cd9	3.20E-61	1.456612768	1	0.739	6.40E-58	EC	3	Cd9
Nr2f2	3.56E-60	1.409669976	0.896	0.266	7.12E-57	EC	3	Nr2f2
Igfbp5	1.42E-59	2.535824642	0.927	0.404	2.84E-56	EC	3	Igfbp5
Sbspon	4.33E-58	0.281464736	0.591	0.029	8.66E-55	EC	3	Sbspon
Hoxd9	7.10E-57	0.964451934	0.768	0.221	1.42E-53	EC	3	Hoxd9
Cp	1.66E-55	1.5231879	0.915	0.478	3.31E-52	EC	3	Cp
Cldn11	3.72E-52	0.700026645	0.652	0.143	7.45E-49	EC	3	Cldn11
Lcn2	4.92E-51	1.717346841	0.787	0.094	9.85E-48	EC	3	Lcn2
Ptn	5.95E-51	1.544406826	0.787	0.289	1.19E-47	EC	3	Ptn
Lyve1	1.58E-50	2.736972715	0.805	0.359	3.15E-47	EC	3	Lyve1
Flt4	2.59E-50	1.455312778	0.872	0.531	5.19E-47	EC	3	Flt4
Prss23	1.41E-49	1.510783327	0.872	0.481	2.81E-46	EC	3	Prss23
Nts	8.25E-49	1.792688963	0.726	0.156	1.65E-45	EC	3	Nts
Gmfg	5.05E-48	1.014873374	0.738	0.202	1.01E-44	EC	3	Gmfg
Sepp1	3.51E-47	1.056533202	0.988	0.912	7.02E-44	EC	3	Sepp1
Pkhd1l1	2.25E-46	0.471426623	0.677	0.123	4.49E-43	EC	3	Pkhd1l1
Gap43	7.71E-46	0.535487353	0.622	0.172	1.54E-42	EC	3	Gap43
Lst1	1.72E-45	0.388110862	0.659	0.17	3.43E-42	EC	3	Lst1
Abi3bp	6.28E-44	1.435541903	0.799	0.462	1.26E-40	EC	3	Abi3bp
Tbx1	2.23E-43	0.968999012	0.799	0.41	4.45E-40	EC	3	Tbx1
Ptprf	4.07E-42	0.560198541	0.634	0.231	8.13E-39	EC	3	Ptprf
Col18a1	4.45E-42	0.940820776	0.787	0.294	8.90E-39	EC	3	Col18a1
Tppp3	3.47E-41	1.978041828	0.841	0.444	6.94E-38	EC	3	Tppp3
Thsd4	4.45E-41	0.257493807	0.585	0.163	8.91E-38	EC	3	Thsd4
Fndc1	1.24E-40	0.814035973	0.72	0.254	2.48E-37	EC	3	Fndc1
Duoxa2	1.95E-40	0.613513137	0.604	0.098	3.90E-37	EC	3	Duoxa2
Tnc	3.20E-39	0.528610759	0.598	0.18	6.41E-36	EC	3	Tnc
Gpm6a	1.80E-38	1.175020821	0.768	0.346	3.61E-35	EC	3	Gpm6a
Prelp	1.02E-37	1.246749311	0.823	0.39	2.04E-34	EC	3	Prelp
Serpine2	4.60E-37	1.055143324	0.787	0.332	9.20E-34	EC	3	Serpine2
Sh3gl3	9.89E-37	0.915170443	0.72	0.44	1.98E-33	EC	3	Sh3gl3
Serping1	1.85E-36	0.874254835	0.89	0.403	3.70E-33	EC	3	Serping1
Fbln7	5.80E-36	0.462041794	0.579	0.247	1.16E-32	EC	3	Fbln7
Mrc1	5.95E-36	0.451636087	0.652	0.281	1.19E-32	EC	3	Mrc1
Itga9	1.02E-35	0.571994294	0.774	0.297	2.04E-32	EC	3	Itga9
Ecscr	1.96E-35	0.807818186	0.982	0.837	3.92E-32	EC	3	Ecscr
Enho	3.54E-33	0.251159445	0.616	0.215	7.08E-30	EC	3	Enho
Ndrg1	2.78E-32	0.979692334	0.896	0.622	5.55E-29	EC	3	Ndrg1
S100a6	7.49E-32	0.7211943	0.982	0.899	1.50E-28	EC	3	S100a6
Klhl4	2.69E-31	0.862342797	0.762	0.42	5.37E-28	EC	3	Klhl4
Lgmn	6.20E-31	0.826860782	0.835	0.414	1.24E-27	EC	3	Lgmn
Col23a1	7.35E-31	0.409443795	0.646	0.28	1.47E-27	EC	3	Col23a1
Adrb2	1.66E-30	0.359127439	0.646	0.341	3.31E-27	EC	3	Adrb2
Lrg1	1.10E-29	1.378236773	0.811	0.494	2.19E-26	EC	3	Lrg1
Vim1	1.40E-29	0.688707626	0.982	0.932	2.80E-26	EC	3	Vim
Cd2	1.53E-29	0.71953416	0.622	0.356	3.07E-26	EC	3	Cd2
Timp3	2.87E-29	0.67137644	0.988	0.913	5.75E-26	EC	3	Timp3
Cyb5r3	3.35E-29	0.952490897	0.909	0.665	6.70E-26	EC	3	Cyb5r3
Adgrg3	4.31E-29	0.977600978	0.701	0.521	8.63E-26	EC	3	Adgrg3
Spp1	1.80E-28	0.283748889	0.555	0.167	3.61E-25	EC	3	Spp1
Gngt2	1.48E-27	0.701290649	0.939	0.632	2.96E-24	EC	3	Gngt2
Prdx1	1.12E-26	0.611372451	0.976	0.856	2.23E-23	EC	3	Prdx1
AA467197	2.09E-26	0.526483533	0.598	0.348	4.17E-23	EC	3	AA467197
Ank3	9.88E-26	0.486463559	0.591	0.322	1.98E-22	EC	3	Ank3
Ifi27l2a	2.47E-25	1.135663941	0.835	0.671	4.95E-22	EC	3	Ifi27l2a
Mfap4	5.30E-25	0.262469693	0.689	0.294	1.06E-21	EC	3	Mfap4
Pcsk6	1.73E-24	0.677661091	0.72	0.595	3.45E-21	EC	3	Pcsk6
Anxa2	4.00E-24	0.64343228	0.927	0.742	8.00E-21	EC	3	Anxa2
Gng11	8.93E-24	0.63456407	0.988	0.929	1.79E-20	EC	3	Gng11
Ptpn18	7.56E-23	0.844775157	0.866	0.586	1.51E-19	EC	3	Ptpn18
Nrp2	6.51E-22	0.684669038	0.793	0.479	1.30E-18	EC	3	Nrp2
Loxl2	2.97E-20	0.299552103	0.634	0.489	5.94E-17	EC	3	Loxl2
Tgfbi	4.67E-20	1.088579984	0.561	0.19	9.34E-17	EC	3	Tgfbi
Dstn	2.73E-19	0.504712781	0.957	0.843	5.46E-16	EC	3	Dstn
Samsn1	6.27E-19	0.344364659	0.646	0.519	1.25E-15	EC	3	Samsn1
Stc1	2.82E-18	0.404593199	0.604	0.481	5.63E-15	EC	3	Stc1
Cfb	3.98E-18	0.253945251	0.591	0.443	7.95E-15	EC	3	Cfb
Clca3a1	6.62E-18	1.226728936	0.598	0.189	1.32E-14	EC	3	Clca3a1
F11r	2.23E-17	0.470361876	0.89	0.583	4.45E-14	EC	3	F11r
Tmsb10	6.70E-17	0.47196029	0.994	0.971	1.34E-13	EC	3	Tmsb10
Stmn21	8.70E-17	0.504144412	0.701	0.411	1.74E-13	EC	3	Stmn2
Nmb	2.72E-16	0.34436989	0.604	0.532	5.43E-13	EC	3	Nmb
Stab11	4.64E-16	0.713060937	0.72	0.483	9.28E-13	EC	3	Stab1
Ucp2	6.39E-16	0.687769607	0.835	0.633	1.28E-12	EC	3	Ucp2
Sdpr	3.31E-15	0.430091941	0.988	0.921	6.62E-12	EC	3	Sdpr
Tuba1a	3.32E-15	0.551404096	0.848	0.6	6.64E-12	EC	3	Tuba1a
Wipf3	4.41E-15	0.827347805	0.579	0.188	8.82E-12	EC	3	Wipf3
Slc25a4	9.67E-15	0.377900815	0.89	0.792	1.93E-11	EC	3	Slc25a4
Prox1	1.51E-14	1.249519033	0.634	0.478	3.02E-11	EC	3	Prox1
Mall	1.90E-14	0.421854446	0.787	0.593	3.81E-11	EC	3	Mall
Cald1	2.10E-14	0.526395703	0.805	0.685	4.21E-11	EC	3	Cald1
Flrt2	5.31E-14	0.27362355	0.591	0.329	1.06E-10	EC	3	Flrt2
Galnt18	1.12E-13	0.751205432	0.61	0.326	2.24E-10	EC	3	Galnt18
Hsp90b11	1.16E-13	0.506667131	0.823	0.703	2.31E-10	EC	3	Hsp90b1
Pgf	1.51E-13	0.303659187	0.591	0.541	3.02E-10	EC	3	Pgf
Pdpn	2.01E-13	0.931184237	0.579	0.351	4.02E-10	EC	3	Pdpn
Ackr4	3.55E-13	0.837166189	0.665	0.342	7.10E-10	EC	3	Ackr4
Cyp4b1	6.02E-13	0.497004608	0.762	0.481	1.20E-09	EC	3	Cyp4b1
Csrp2	1.16E-12	0.474401938	0.738	0.551	2.33E-09	EC	3	Csrp2
Tuba1b	2.62E-12	0.395395577	0.902	0.697	5.23E-09	EC	3	Tuba1b
Aldoa	8.16E-11	0.388533263	0.939	0.786	1.63E-07	EC	3	Aldoa
Lrrfip1	1.18E-10	0.277512225	0.671	0.431	2.36E-07	EC	3	Lrrfip1
Gas6	2.38E-10	0.458255331	0.878	0.747	4.77E-07	EC	3	Gas6
Dpysl3	2.49E-10	0.386883791	0.695	0.518	4.97E-07	EC	3	Dpysl3
Dab2	6.72E-10	0.690255071	0.585	0.28	1.34E-06	EC	3	Dab2
Calcrl	1.33E-09	0.346838601	0.695	0.486	2.65E-06	EC	3	Calcrl
Ctsl	1.60E-09	0.338524784	0.957	0.872	3.19E-06	EC	3	Ctsl
Akap12	3.23E-09	0.293814675	0.36	0.254	6.46E-06	EC	3	Akap12
Sned1	5.00E-09	0.47350495	0.622	0.46	1.00E-05	EC	3	Sned1
Igfbp4	7.16E-09	0.286839451	0.744	0.561	1.43E-05	EC	3	Igfbp4
Gja1	1.25E-08	0.436771468	0.524	0.352	2.49E-05	EC	3	Gja1
Aebp1	1.84E-08	0.437692227	0.683	0.44	3.67E-05	EC	3	Aebp1
Capg	2.02E-08	0.281368831	0.695	0.567	4.05E-05	EC	3	Capg
Gdf10	2.52E-08	0.252023995	0.53	0.25	5.05E-05	EC	3	Gdf10
Plpp1	3.17E-08	0.367860968	0.86	0.719	6.34E-05	EC	3	Plpp1
Cd55	4.95E-08	0.591927169	0.61	0.455	9.89E-05	EC	3	Cd55
Duox2	7.52E-08	0.436929347	0.256	0.206	0.000150465	EC	3	Duox2
Gapdh	7.86E-08	0.279442643	0.939	0.814	0.00015715	EC	3	Gapdh
Lrrn4cl	1.10E-07	0.372128463	0.451	0.32	0.000219292	EC	3	Lrrn4cl
Slfn2	4.07E-07	0.421484527	0.494	0.273	0.000814405	EC	3	Slfn2
Nudt4	1.08E-06	0.42816077	0.628	0.42	0.002165972	EC	3	Nudt4

Il2rg	1.84E-06	0.259144442	0.293	0.525	0.003680511	EC_3		Il2rg
Nov	2.09E-06	0.360750474	2.99	0.209	0.004180668	EC_3		Nov
Tgm21	1.22E-05	0.329530604	0.841	0.762	0.024459493	EC_3		Tgm2
Il33	1.49E-05	0.66346531	1.415	0.167	0.029793124	EC_3		Il33
Ece11	2.26E-05	0.286496995	0.835	0.853	0.045104087	EC_3		Ece1
Kctd12	4.60E-05	0.442816975	0.768	0.675	0.092051333	EC_3		Kctd12
Emp1	4.92E-05	0.461592452	0.598	0.567	0.098386856	EC_3		Emp1
Pcp4l11	5.67E-05	0.331646872	0.683	0.576	0.113470269	EC_3		Pcp4l1
Igfbp6	7.75E-05	0.329350963	0.555	0.4	0.154970942	EC_3		Igfbp6
Gpr182	0.000151824	0.698037956	0.488	0.388	0.303647124	EC_3		Gpr182
Pdlim4	0.000195003	0.360582364	0.591	0.579	0.3900068	EC_3		Pdlim4
Ninj11	0.000322271	0.374214942	0.598	0.538	0.644542527	EC_3		Ninj1
Gucy1b3	0.001661202	0.419177639	0.366	0.139	1	EC_3		Gucy1b3
Bok	0.003193124	0.277262032	0.421	0.372	1	EC_3		Bok
Arhgdib1	0.004064735	0.358575766	0.604	0.554	1	EC_3		Arhgdib
Meox1	0.005394762	0.391397197	0.518	0.478	1	EC_3		Meox1
Trf	0.009194422	0.34820314	0.396	0.629	1	EC_3		Trf

Supplementary Table 4_EC.GO Analysis

EC_1 GO:BP

	ID	Description	GeneRatio	BgRatio	pvalue	p.adjust	qvalue	geneID	Count
GO:0009611	GO:0009611	response to wounding	51/385	492/23328	6.48E-26	2.71E-22	1.63E-22	Cd36/Cd9	51
GO:0042060	GO:0042060	wound healing	40/385	349/23328	4.31E-22	9.02E-19	5.42E-19	Cd36/Cd9	40
GO:0050673	GO:0050673	epithelial cell proliferation	40/385	438/23328	1.67E-18	2.33E-15	1.40E-15	Rgcc/Flt1/	40
GO:0001935	GO:0001935	endothelial cell proliferation	25/385	149/23328	3.20E-18	3.35E-15	2.01E-15	Rgcc/Flt1/	25
GO:0001936	GO:0001936	regulation of endothelial cell proliferation	23/385	134/23328	4.32E-17	3.24E-14	1.95E-14	Rgcc/Flt1/	23
GO:0007596	GO:0007596	blood coagulation	25/385	166/23328	4.65E-17	3.24E-14	1.95E-14	Cd36/Cd9	25
GO:0007599	GO:0007599	hemostasis	25/385	168/23328	6.23E-17	3.73E-14	2.24E-14	Cd36/Cd9	25
GO:0050817	GO:0050817	coagulation	25/385	169/23328	7.20E-17	3.77E-14	2.26E-14	Cd36/Cd9	25
GO:0050878	GO:0050878	regulation of body fluid levels	34/385	354/23328	1.52E-16	7.08E-14	4.25E-14	Cd36/Aplr	34
GO:1901342	GO:1901342	regulation of vasculature development	34/385	356/23328	1.81E-16	7.56E-14	4.54E-14	Rgcc/Cd3	34
GO:0030198	GO:0030198	extracellular matrix organization	31/385	302/23328	5.45E-16	2.07E-13	1.25E-13	Rgcc/Tcf1	31
GO:0043062	GO:0043062	extracellular structure organization	31/385	303/23328	5.98E-16	2.08E-13	1.25E-13	Rgcc/Tcf1	31
GO:0050678	GO:0050678	regulation of epithelial cell proliferation	34/385	372/23328	6.82E-16	2.20E-13	1.32E-13	Rgcc/Flt1/	34
GO:0050900	GO:0050900	leukocyte migration	33/385	360/23328	1.70E-15	5.09E-13	3.06E-13	Flt1/Cxcl1	33
GO:0045765	GO:0045765	regulation of angiogenesis	31/385	320/23328	2.75E-15	7.67E-13	4.61E-13	Rgcc/Cd3	31
GO:0051271	GO:0051271	negative regulation of cellular component movement	31/385	326/23328	4.60E-15	1.20E-12	7.23E-13	Rgcc/Cxcl	31
GO:0031589	GO:0031589	cell-substrate adhesion	32/385	351/23328	5.42E-15	1.33E-12	8.01E-13	Cd36/Thy	32
GO:0001667	GO:0001667	ameboidal-type cell migration	35/385	427/23328	6.97E-15	1.58E-12	9.50E-13	Rgcc/Scar	35
GO:1903034	GO:1903034	regulation of response to wounding	24/385	186/23328	7.18E-15	1.58E-12	9.50E-13	Cd36/Cd9	24
GO:2000146	GO:2000146	negative regulation of cell motility	28/385	291/23328	7.54E-14	1.58E-11	9.48E-12	Rgcc/Cxcl	28
GO:0040013	GO:0040013	negative regulation of locomotion	29/385	326/23328	2.00E-13	3.99E-11	2.40E-11	Rgcc/Cxcl	29
GO:1900046	GO:1900046	regulation of hemostasis	16/385	83/23328	4.20E-13	7.65E-11	4.59E-11	Cd36/Cd9	16
GO:0061041	GO:0061041	regulation of wound healing	20/385	146/23328	4.20E-13	7.65E-11	4.59E-11	Cd36/Cd9	20
GO:0050818	GO:0050818	regulation of coagulation	16/385	86/23328	7.51E-13	1.31E-10	7.87E-11	Cd36/Cd9	16
GO:0030336	GO:0030336	negative regulation of cell migration	26/385	278/23328	1.16E-12	1.94E-10	1.17E-10	Rgcc/Cxcl	26
GO:0030193	GO:0030193	regulation of blood coagulation	15/385	82/23328	5.11E-12	8.23E-10	4.94E-10	Cd36/Cd9	15
GO:1904018	GO:1904018	positive regulation of vasculature development	22/385	208/23328	5.74E-12	8.89E-10	5.34E-10	Flt1/Aplnr/	22
GO:0060326	GO:0060326	cell chemotaxis	26/385	303/23328	8.28E-12	1.21E-09	7.29E-10	Flt1/Cxcl1	26
GO:0043542	GO:0043542	endothelial cell migration	22/385	212/23328	8.40E-12	1.21E-09	7.29E-10	Rgcc/Scar	22
GO:0003158	GO:0003158	endothelium development	17/385	122/23328	1.86E-11	2.60E-09	1.56E-09	Col15a1/F	17
GO:0007517	GO:0007517	muscle organ development	30/385	420/23328	2.08E-11	2.80E-09	1.68E-09	Tcf15/Atf3	30
GO:0001938	GO:0001938	positive regulation of endothelial cell proliferation	15/385	91/23328	2.46E-11	3.18E-09	1.91E-09	Aplnr/Cxc	15
GO:0003018	GO:0003018	vascular process in circulatory system	22/385	224/23328	2.51E-11	3.18E-09	1.91E-09	Cd36/Mgll	22
GO:0010631	GO:0010631	epithelial cell migration	25/385	295/23328	2.76E-11	3.40E-09	2.04E-09	Rgcc/Scar	25
GO:0045446	GO:0045446	endothelial cell differentiation	16/385	108/23328	2.84E-11	3.40E-09	2.04E-09	Col15a1/F	16
GO:0050727	GO:0050727	regulation of inflammatory response	28/385	372/23328	2.92E-11	3.40E-09	2.04E-09	Fabp4/Mg	28
GO:0090132	GO:0090132	epithelium migration	25/385	297/23328	3.20E-11	3.61E-09	2.17E-09	Rgcc/Scar	25
GO:0090130	GO:0090130	tissue migration	25/385	299/23328	3.69E-11	4.07E-09	2.44E-09	Rgcc/Scar	25
GO:0045766	GO:0045766	positive regulation of angiogenesis	20/385	188/23328	4.81E-11	5.16E-09	3.10E-09	Flt1/Aplnr/	20
GO:0045785	GO:0045785	positive regulation of cell adhesion	30/385	435/23328	4.93E-11	5.16E-09	3.10E-09	Cd36/Cxc	30
GO:0060537	GO:0060537	muscle tissue development	31/385	470/23328	7.12E-11	7.27E-09	4.37E-09	Atf3/Rgs4	31
GO:0019932	GO:0019932	second-messenger-mediated signaling	30/385	445/23328	8.59E-11	8.56E-09	5.14E-09	Cd36/Aplr	30
GO:0032103	GO:0032103	positive regulation of response to external stimulus	29/385	418/23328	9.09E-11	8.85E-09	5.32E-09	Fabp4/Cx	29
GO:0001763	GO:0001763	morphogenesis of a branching structure	22/385	241/23328	1.05E-10	9.98E-09	6.00E-09	Flt1/Cxcl1	22
GO:0007162	GO:0007162	negative regulation of cell adhesion	24/385	290/23328	1.13E-10	1.06E-08	6.34E-09	Rgcc/Dtx1	24
GO:0061138	GO:0061138	morphogenesis of a branching epithelium	21/385	222/23328	1.45E-10	1.32E-08	7.92E-09	Flt1/Cxcl1	21
GO:0070371	GO:0070371	ERK1 and ERK2 cascade	25/385	325/23328	2.19E-10	1.95E-08	1.17E-08	Cd36/Atf3	25
GO:0001666	GO:0001666	response to hypoxia	20/385	208/23328	2.99E-10	2.55E-08	1.53E-08	Rgcc/Flt1/	20
GO:0050679	GO:0050679	positive regulation of epithelial cell proliferation	20/385	208/23328	2.99E-10	2.55E-08	1.53E-08	Aplnr/Cxc	20
GO:0010594	GO:0010594	regulation of endothelial cell migration	18/385	166/23328	3.30E-10	2.76E-08	1.66E-08	Rgcc/Spa	18
GO:0010811	GO:0010811	positive regulation of cell-substrate adhesion	16/385	128/23328	3.86E-10	3.14E-08	1.88E-08	Cd36/Thy	16
GO:0070372	GO:0070372	regulation of ERK1 and ERK2 cascade	24/385	308/23328	3.90E-10	3.14E-08	1.88E-08	Cd36/Atf3	24
GO:1903036	GO:1903036	positive regulation of response to wounding	13/385	78/23328	4.61E-10	3.64E-08	2.19E-08	Cd36/Selp	13
GO:0007160	GO:0007160	cell-matrix adhesion	20/385	214/23328	4.95E-10	3.77E-08	2.27E-08	Cd36/Thy	20
GO:0010810	GO:0010810	regulation of cell-substrate adhesion	20/385	214/23328	4.95E-10	3.77E-08	2.27E-08	Cd36/Thy	20
GO:0002685	GO:0002685	regulation of leukocyte migration	20/385	217/23328	6.34E-10	4.74E-08	2.85E-08	Cxcl12/Cc	20
GO:1902903	GO:1902903	regulation of supramolecular fiber organization	26/385	370/23328	6.69E-10	4.91E-08	2.95E-08	Rgcc/Tms	26
GO:0010595	GO:0010595	positive regulation of endothelial cell migration	14/385	103/23328	1.60E-09	1.15E-07	6.94E-08	Sparc/Hsp	14
GO:0050866	GO:0050866	negative regulation of cell activation	19/385	210/23328	2.33E-09	1.65E-07	9.93E-08	Dtx1/Fgl2/	19
GO:0036293	GO:0036293	response to decreased oxygen levels	20/385	234/23328	2.37E-09	1.66E-07	9.95E-08	Rgcc/Flt1/	20
GO:0010632	GO:0010632	regulation of epithelial cell migration	20/385	235/23328	2.56E-09	1.75E-07	1.05E-07	Rgcc/Spa	20
GO:0050680	GO:0050680	negative regulation of epithelial cell proliferation	16/385	148/23328	3.35E-09	2.26E-07	1.36E-07	Rgcc/Flt1/	16
GO:0090303	GO:0090303	positive regulation of wound healing	11/385	60/23328	3.56E-09	2.37E-07	1.42E-07	Cd36/Selp	11
GO:0010634	GO:0010634	positive regulation of epithelial cell migration	16/385	150/23328	4.08E-09	2.67E-07	1.60E-07	Sparc/Hsp	16
GO:0032970	GO:0032970	regulation of actin filament-based process	26/385	410/23328	5.79E-09	3.73E-07	2.24E-07	Rgcc/Tms	26
GO:0042326	GO:0042326	negative regulation of phosphorylation	28/385	471/23328	6.12E-09	3.86E-07	2.32E-07	Fabp4/Atf	28
GO:1900047	GO:1900047	negative regulation of hemostasis	10/385	49/23328	6.18E-09	3.86E-07	2.32E-07	Cd9/Cd34	10
GO:0071900	GO:0071900	regulation of protein serine/threonine kinase activity	27/385	442/23328	6.49E-09	4.00E-07	2.40E-07	Rgcc/Flt1/	27
GO:0032102	GO:0032102	negative regulation of response to external stimulus	25/385	384/23328	6.77E-09	4.10E-07	2.47E-07	Fgl2/Cd9/	25
GO:0001570	GO:0001570	vasculogenesis	13/385	97/23328	7.34E-09	4.39E-07	2.64E-07	Aplnr/Kdr/	13
GO:0050819	GO:0050819	negative regulation of coagulation	10/385	50/23328	7.62E-09	4.49E-07	2.70E-07	Cd9/Cd34	10
GO:0048608	GO:0048608	reproductive structure development	28/385	477/23328	8.06E-09	4.68E-07	2.81E-07	Sfrp1/Nup	28
GO:0014706	GO:0014706	striated muscle tissue development	27/385	447/23328	8.24E-09	4.72E-07	2.84E-07	Atf3/Rgs4	27
GO:0061458	GO:0061458	reproductive system development	28/385	481/23328	9.65E-09	5.46E-07	3.28E-07	Sfrp1/Nup	28
GO:1901343	GO:1901343	negative regulation of vasculature development	15/385	139/23328	1.07E-08	5.95E-07	3.58E-07	Rgcc/Cd3	15
GO:0051961	GO:0051961	negative regulation of nervous system development	24/385	365/23328	1.13E-08	6.21E-07	3.73E-07	Dtx1/Thy1	24
GO:1903706	GO:1903706	regulation of hemopoiesis	26/385	424/23328	1.15E-08	6.24E-07	3.75E-07	Flt1/Dtx1/I	26
GO:0030168	GO:0030168	platelet activation	12/385	83/23328	1.16E-08	6.24E-07	3.75E-07	Cd9/Cfh/S	12
GO:0007178	GO:0007178	transmembrane receptor protein serine/threonine kinas	24/385	368/23328	1.32E-08	6.99E-07	4.20E-07	Vim/Id1/S	24
GO:0042493	GO:0042493	response to drug	20/385	260/23328	1.44E-08	7.53E-07	4.53E-07	Sfrp1/Prnp	20
GO:0070482	GO:0070482	response to oxygen levels	21/385	287/23328	1.50E-08	7.77E-07	4.67E-07	Rgcc/Flt1/	21
GO:0048660	GO:0048660	regulation of smooth muscle cell proliferation	16/385	165/23328	1.62E-08	8.26E-07	4.96E-07	Notch3/Igf	16
GO:0003073	GO:0003073	regulation of systemic arterial blood pressure	14/385	124/23328	1.85E-08	9.32E-07	5.60E-07	Cd36/Pdg	14
GO:0018108	GO:0018108	peptidyl-tyrosine phosphorylation	22/385	322/23328	2.35E-08	1.17E-06	7.03E-07	Cd36/Flt1/	22
GO:0048659	GO:0048659	smooth muscle cell proliferation	16/385	170/23328	2.48E-08	1.22E-06	7.33E-07	Notch3/Igf	16
GO:0060840	GO:0060840	artery development	13/385	108/23328	2.76E-08	1.30E-06	7.83E-07	Adgrf5/Tb	13
GO:0018212	GO:0018212	peptidyl-tyrosine modification	22/385	325/23328	2.77E-08	1.30E-06	7.83E-07	Cd36/Flt1/	22
GO:0030595	GO:0030595	leukocyte chemotaxis	18/385	219/23328	2.77E-08	1.30E-06	7.83E-07	Flt1/Cxcl1	18
GO:0097529	GO:0097529	myeloid leukocyte migration	18/385	219/23328	2.77E-08	1.30E-06	7.83E-07	Flt1/Cxcl1	18
GO:0048771	GO:0048771	tissue remodeling	17/385	196/23328	3.06E-08	1.42E-06	8.56E-07	Sfrp1/Cd3	17
GO:0010721	GO:0010721	negative regulation of cell development	24/385	387/23328	3.45E-08	1.58E-06	9.47E-07	Dtx1/Rgs4	24
GO:0010038	GO:0010038	response to metal ion	20/385	274/23328	3.46E-08	1.58E-06	9.47E-07	Fabp4/Cp	20
GO:0035296	GO:0035296	regulation of tube diameter	16/385	176/23328	4.05E-08	1.80E-06	1.08E-06	Mgll/Nts/C	16
GO:0097746	GO:0097746	regulation of blood vessel diameter	16/385	176/23328	4.05E-08	1.80E-06	1.08E-06	Mgll/Nts/C	16
GO:0032956	GO:0032956	regulation of actin cytoskeleton organization	23/385	361/23328	4.09E-08	1.80E-06	1.08E-06	Rgcc/Tms	23
GO:0001569	GO:0001569	branching involved in blood vessel morphogenesis	9/385	45/23328	4.34E-08	1.89E-06	1.14E-06	Flt1/Cxcl1	9
GO:0035150	GO:0035150	regulation of tube size	16/385	177/23328	4.38E-08	1.89E-06	1.14E-06	Mgll/Nts/C	16
GO:0090287	GO:0090287	regulation of cellular response to growth factor stimulu	20/385	281/23328	5.26E-08	2.25E-06	1.35E-06	Sfrp1/Cdh	20
GO:0048844	GO:0048844	artery morphogenesis	11/385	77/23328	5.41E-08	2.29E-06	1.37E-06	Adgrf5/Tb	11
GO:0002683	GO:0002683	negative regulation of immune system process	26/385	462/23328	6.48E-08	2.71E-06	1.63E-06	Dtx1/Fgl2/	26
GO:0046890	GO:0046890	regulation of lipid biosynthetic process	16/385	184/23328	7.55E-08	3.13E-06	1.88E-06	Igfbp7/Sca	16
GO:0030195	GO:0030195	negative regulation of blood coagulation	9/385	48/23328	7.86E-08	3.20E-06	1.92E-06	Cd9/Cd34	9
GO:0008217	GO:0008217	regulation of blood pressure	17/385	209/23328	7.86E-08	3.20E-06	1.92E-06	Cd36/Cd3	17
GO:0007015	GO:0007015	actin filament organization	25/385	437/23328	8.53E-08	3.43E-06	2.06E-06	Rgcc/Tms	25
GO:0001558	GO:0001558	regulation of cell growth	25/385	438/23328	8.91E-08	3.55E-06	2.13E-06	Igfbp7/Mg	25
GO:0048015	GO:0048015	phosphatidylinositol-mediated signaling	15/385	164/23328	9.93E-08	3.92E-06	2.36E-06	Flt1/Reln/	15
GO:0003094	GO:0003094	glomerular filtration	7/385	25/23328	1.18E-07	4.60E-06	2.76E-06	Adgrf5/Pd	7
GO:0016525	GO:0016525	negative regulation of angiogenesis	13/385	122/23328	1.19E-07	4.63E-06	2.78E-06	Rgcc/Cd3	13

GO:0048017	GO:0048017	inositol lipid-mediated signaling	15/385	167/23328	1.26E-07	4.84E-06	2.91E-06	Flt1/Reln/:	15
GO:0022408	GO:0022408	negative regulation of cell-cell adhesion	16/385	192/23328	1.36E-07	5.18E-06	3.11E-06	Rgcc/Dtx1	16
GO:0007263	GO:0007263	nitric oxide mediated signal transduction	7/385	26/23328	1.59E-07	5.92E-06	3.56E-06	Cd36/Thb	7
GO:0097205	GO:0097205	renal filtration	7/385	26/23328	1.59E-07	5.92E-06	3.56E-06	Adgrf5/Pd	7
GO:2000181	GO:2000181	negative regulation of blood vessel morphogenesis	13/385	126/23328	1.75E-07	6.48E-06	3.89E-06	Rgcc/Cd3	13
GO:0050921	GO:0050921	positive regulation of chemotaxis	14/385	149/23328	1.90E-07	6.94E-06	4.17E-06	Cxcl12/Fn	14
GO:0048146	GO:0048146	positive regulation of fibroblast proliferation	10/385	69/23328	1.91E-07	6.94E-06	4.17E-06	Fn1/Pdgfb	10
GO:0019216	GO:0019216	regulation of lipid metabolic process	22/385	363/23328	1.93E-07	6.95E-06	4.18E-06	Igfbp7/Cd	22
GO:0001933	GO:0001933	negative regulation of protein phosphorylation	24/385	425/23328	1.97E-07	7.05E-06	4.24E-06	Fabp4/Atf	24
GO:0034329	GO:0034329	cell junction assembly	24/385	426/23328	2.06E-07	7.27E-06	4.37E-06	Reln/Cd9/	24
GO:0031099	GO:0031099	regeneration	14/385	150/23328	2.07E-07	7.27E-06	4.37E-06	Cxcl12/Cc	14
GO:0048145	GO:0048145	regulation of fibroblast proliferation	12/385	107/23328	2.08E-07	7.27E-06	4.37E-06	Sfrp1/Nup	12
GO:0018149	GO:0018149	peptide cross-linking	7/385	27/23328	2.11E-07	7.30E-06	4.38E-06	Fn1/Bgn/T	7
GO:0022407	GO:0022407	regulation of cell-cell adhesion	24/385	427/23328	2.15E-07	7.37E-06	4.43E-06	Rgcc/Dtx1	24
GO:0014065	GO:0014065	phosphatidylinositol 3-kinase signaling	13/385	129/23328	2.31E-07	7.82E-06	4.70E-06	Flt1/Reln/:	13
GO:0050768	GO:0050768	negative regulation of neurogenesis	21/385	337/23328	2.32E-07	7.82E-06	4.70E-06	Dtx1/Thy1	21
GO:0042692	GO:0042692	muscle cell differentiation	23/385	399/23328	2.47E-07	8.23E-06	4.94E-06	Cxcl12/Tn	23
GO:0051149	GO:0051149	positive regulation of muscle cell differentiation	11/385	89/23328	2.48E-07	8.23E-06	4.94E-06	Cxcl12/Tn	11
GO:0001885	GO:0001885	endothelial cell development	10/385	71/23328	2.52E-07	8.29E-06	4.98E-06	Col15a1/F	10
GO:0048144	GO:0048144	fibroblast proliferation	12/385	109/23328	2.56E-07	8.37E-06	5.03E-06	Sfrp1/Nup	12
GO:0010712	GO:0010712	regulation of collagen metabolic process	9/385	55/23328	2.69E-07	8.67E-06	5.21E-06	Rgcc/Vim/	9
GO:2000351	GO:2000351	regulation of endothelial cell apoptotic process	9/385	55/23328	2.69E-07	8.67E-06	5.21E-06	Rgcc/Cdh	9
GO:1903035	GO:1903035	negative regulation of response to wounding	11/385	90/23328	2.78E-07	8.88E-06	5.34E-06	Cd9/Cd34	11
GO:0048644	GO:0048644	muscle organ morphogenesis	11/385	91/23328	3.12E-07	9.88E-06	5.94E-06	Tcf15/Tbx	11
GO:0002687	GO:0002687	positive regulation of leukocyte migration	14/385	156/23328	3.36E-07	1.06E-05	6.34E-06	Cxcl12/Th	14
GO:0043254	GO:0043254	regulation of protein-containing complex assembly	24/385	438/23328	3.41E-07	1.06E-05	6.39E-06	Cd36/Tms	24
GO:0045665	GO:0045665	negative regulation of neuron differentiation	18/385	259/23328	3.49E-07	1.08E-05	6.50E-06	Dtx1/Thy1	18
GO:0032964	GO:0032964	collagen biosynthetic process	9/385	57/23328	3.70E-07	1.14E-05	6.84E-06	Rgcc/Vim/	9
GO:0031345	GO:0031345	negative regulation of cell projection organization	16/385	207/23328	3.80E-07	1.15E-05	6.93E-06	Thy1/Vim/	16
GO:0003014	GO:0003014	renal system process	12/385	113/23328	3.80E-07	1.15E-05	6.93E-06	Adgrf5/Lg	12
GO:0071560	GO:0071560	cellular response to transforming growth factor beta sti	16/385	208/23328	4.06E-07	1.22E-05	7.34E-06	Col4a2/Cc	16
GO:0072577	GO:0072577	endothelial cell apoptotic process	9/385	58/23328	4.32E-07	1.29E-05	7.75E-06	Rgcc/Cdh	9
GO:1904035	GO:1904035	regulation of epithelial cell apoptotic process	11/385	94/23328	4.35E-07	1.29E-05	7.76E-06	Rgcc/Nup	11
GO:0033002	GO:0033002	muscle cell proliferation	17/385	236/23328	4.48E-07	1.31E-05	7.88E-06	Notch3/Igf	17
GO:0010876	GO:0010876	lipid localization	23/385	413/23328	4.53E-07	1.31E-05	7.88E-06	Fabp4/Cd	23
GO:2001233	GO:2001233	regulation of apoptotic signaling pathway	23/385	413/23328	4.53E-07	1.31E-05	7.88E-06	Cxcl12/At	23
GO:0072593	GO:0072593	reactive oxygen species metabolic process	19/385	292/23328	4.54E-07	1.31E-05	7.88E-06	Cd36/Fbln	19
GO:0001945	GO:0001945	lymph vessel development	7/385	30/23328	4.63E-07	1.33E-05	7.98E-06	Tbx1/Kdr/	7
GO:0030099	GO:0030099	myeloid cell differentiation	23/385	414/23328	4.73E-07	1.35E-05	8.08E-06	Sfrp1/Adg	23
GO:0007229	GO:0007229	integrin-mediated signaling pathway	11/385	95/23328	4.85E-07	1.37E-05	8.24E-06	Thy1/Fn1/	11
GO:0009636	GO:0009636	response to toxic substance	12/385	116/23328	5.06E-07	1.42E-05	8.55E-06	Cd36/Nup	12
GO:0071559	GO:0071559	response to transforming growth factor beta	16/385	212/23328	5.24E-07	1.46E-05	8.79E-06	Col4a2/Cc	16
GO:0001819	GO:0001819	positive regulation of cytokine production	24/385	449/23328	5.32E-07	1.48E-05	8.86E-06	Rgcc/Cd3	24
GO:0006898	GO:0006898	receptor-mediated endocytosis	17/385	240/23328	5.67E-07	1.56E-05	9.38E-06	Cd36/Cd9	17
GO:1902904	GO:1902904	negative regulation of supramolecular fiber organizatio	14/385	163/23328	5.74E-07	1.57E-05	9.43E-06	Tmsb4x/C	14
GO:0001937	GO:0001937	negative regulation of endothelial cell proliferation	8/385	45/23328	6.47E-07	1.76E-05	1.06E-05	Rgcc/Flt1/	8
GO:0043534	GO:0043534	blood vessel endothelial cell migration	12/385	119/23328	6.68E-07	1.80E-05	1.08E-05	Rgcc/Scar	12
GO:0070374	GO:0070374	positive regulation of ERK1 and ERK2 cascade	16/385	216/23328	6.74E-07	1.81E-05	1.09E-05	Cd36/Ccl2	16
GO:0014066	GO:0014066	regulation of phosphatidylinositol 3-kinase signaling	11/385	99/23328	7.38E-07	1.97E-05	1.18E-05	Flt1/Reln/:	11
GO:0001503	GO:0001503	ossification	22/385	394/23328	7.73E-07	2.04E-05	1.23E-05	Id1/Rsad2	22
GO:0031102	GO:0031102	neuron projection regeneration	9/385	62/23328	7.76E-07	2.04E-05	1.23E-05	Thy1/Gap	9
GO:0010959	GO:0010959	regulation of metal ion transport	23/385	427/23328	8.07E-07	2.11E-05	1.27E-05	Aplnr/Cxc	23
GO:0006869	GO:0006869	lipid transport	21/385	364/23328	8.17E-07	2.12E-05	1.28E-05	Fabp4/Cd	21
GO:0007179	GO:0007179	transforming growth factor beta receptor signaling path	14/385	169/23328	8.89E-07	2.30E-05	1.38E-05	Cdh5/Htra	14
GO:0003007	GO:0003007	heart morphogenesis	18/385	277/23328	9.30E-07	2.39E-05	1.44E-05	Cpe/Tbx1/	18
GO:0002040	GO:0002040	sprouting angiogenesis	12/385	123/23328	9.55E-07	2.44E-05	1.46E-05	Flt1/Aplnr/	12
GO:0042063	GO:0042063	gliogenesis	20/385	338/23328	1.01E-06	2.57E-05	1.54E-05	Reln/Dtx1	20
GO:0007043	GO:0007043	cell-cell junction assembly	13/385	147/23328	1.04E-06	2.63E-05	1.58E-05	Cd9/Cdh5	13
GO:0032965	GO:0032965	regulation of collagen biosynthetic process	8/385	48/23328	1.09E-06	2.70E-05	1.62E-05	Rgcc/Vim/	8
GO:0048010	GO:0048010	vascular endothelial growth factor receptor signaling pa	8/385	48/23328	1.09E-06	2.70E-05	1.62E-05	Flt1/Hspb1	8
GO:0050920	GO:0050920	regulation of chemotaxis	16/385	224/23328	1.09E-06	2.70E-05	1.63E-05	Cxcl12/Fn	16
GO:0060415	GO:0060415	muscle tissue morphogenesis	10/385	83/23328	1.11E-06	2.74E-05	1.65E-05	Tbx1/Ly6e	10
GO:0019934	GO:0019934	cGMP-mediated signaling	7/385	34/23328	1.16E-06	2.83E-05	1.70E-05	Cd36/Pde	7
GO:1905954	GO:1905954	positive regulation of lipid localization	11/385	105/23328	1.34E-06	3.25E-05	1.95E-05	Cd36/Sca	11
GO:0003012	GO:0003012	muscle system process	22/385	408/23328	1.38E-06	3.33E-05	2.00E-05	Rgs4/Cd3	22
GO:0007159	GO:0007159	leukocyte cell-cell adhesion	20/385	345/23328	1.39E-06	3.34E-05	2.01E-05	Dtx1/Fgl2/	20
GO:0045216	GO:0045216	cell-cell junction organization	15/385	205/23328	1.75E-06	4.19E-05	2.52E-05	Cd9/Perp/	15
GO:0048661	GO:0048661	positive regulation of smooth muscle cell proliferation	11/385	108/23328	1.77E-06	4.21E-05	2.53E-05	Notch3/Igf	11
GO:0097756	GO:0097756	negative regulation of blood vessel diameter	11/385	109/23328	1.94E-06	4.59E-05	2.76E-05	Mgll/Cd38	11
GO:0050730	GO:0050730	regulation of peptidyl-tyrosine phosphorylation	17/385	263/23328	2.01E-06	4.72E-05	2.83E-05	Cd36/Reln	17
GO:0071248	GO:0071248	cellular response to metal ion	13/385	156/23328	2.04E-06	4.78E-05	2.87E-05	Fabp4/Fn1	13
GO:0032963	GO:0032963	collagen metabolic process	11/385	111/23328	2.32E-06	5.40E-05	3.24E-05	Rgcc/Vim/	11
GO:0010543	GO:0010543	regulation of platelet activation	7/385	38/23328	2.56E-06	5.93E-05	3.56E-05	Cd9/Selp/	7
GO:1903524	GO:1903524	positive regulation of blood circulation	10/385	91/23328	2.61E-06	6.01E-05	3.61E-05	Rgs4/Cd3	10
GO:0046688	GO:0046688	response to copper ion	6/385	25/23328	2.64E-06	6.04E-05	3.63E-05	Cp/Prnp/A	6
GO:0061045	GO:0061045	negative regulation of wound healing	9/385	72/23328	2.82E-06	6.40E-05	3.85E-05	Cd9/Cd34	9
GO:0030278	GO:0030278	regulation of ossification	16/385	241/23328	2.84E-06	6.43E-05	3.86E-05	Id1/Rsad2	16
GO:0043405	GO:0043405	regulation of MAP kinase activity	18/385	301/23328	3.03E-06	6.83E-05	4.10E-05	Flt1/Sfrp1/	18
GO:0031214	GO:0031214	biomineral tissue development	13/385	162/23328	3.11E-06	6.92E-05	4.16E-05	Mgp/Tbx1	13
GO:0110148	GO:0110148	biomineralization	13/385	162/23328	3.11E-06	6.92E-05	4.16E-05	Mgp/Tbx1	13
GO:1902043	GO:1902043	positive regulation of extrinsic apoptotic signaling path	5/385	15/23328	3.13E-06	6.92E-05	4.16E-05	Atf3/Sfrp1	5
GO:0014068	GO:0014068	positive regulation of phosphatidylinositol 3-kinase sig	9/385	73/23328	3.16E-06	6.93E-05	4.17E-05	Flt1/Reln/:	9
GO:0051155	GO:0051155	positive regulation of striated muscle cell differentiatio	9/385	73/23328	3.16E-06	6.93E-05	4.17E-05	Cxcl12/Tb	9
GO:0033622	GO:0033622	integrin activation	6/385	26/23328	3.38E-06	7.34E-05	4.41E-05	Cxcl12/Se	6
GO:0072012	GO:0072012	glomerulus vasculature development	6/385	26/23328	3.38E-06	7.34E-05	4.41E-05	Notch3/Pc	6
GO:0030199	GO:0030199	collagen fibril organization	8/385	56/23328	3.64E-06	7.81E-05	4.70E-05	Col5a2/Lc	8
GO:0045620	GO:0045620	negative regulation of lymphocyte differentiation	8/385	56/23328	3.64E-06	7.81E-05	4.70E-05	Dtx1/Fgl2/	8
GO:0051147	GO:0051147	regulation of muscle cell differentiation	13/385	165/23328	3.81E-06	8.10E-05	4.87E-05	Cxcl12/Tn	13
GO:1905952	GO:1905952	regulation of lipid localization	13/385	165/23328	3.81E-06	8.10E-05	4.87E-05	Cd36/Sca	13
GO:0034109	GO:0034109	homotypic cell-cell adhesion	9/385	75/23328	3.98E-06	8.41E-05	5.05E-05	Cd9/Cfh/F	9
GO:0031349	GO:0031349	positive regulation of defense response	17/385	278/23328	4.24E-06	8.80E-05	5.29E-05	Fabp4/Irf7	17
GO:1904019	GO:1904019	epithelial cell apoptotic process	11/385	118/23328	4.24E-06	8.80E-05	5.29E-05	Rgcc/Nup	11
GO:0030194	GO:0030194	positive regulation of blood coagulation	6/385	27/23328	4.29E-06	8.80E-05	5.29E-05	Cd36/Selp	6
GO:0061437	GO:0061437	renal system vasculature development	6/385	27/23328	4.29E-06	8.80E-05	5.29E-05	Notch3/Pc	6
GO:0061440	GO:0061440	kidney vasculature development	6/385	27/23328	4.29E-06	8.80E-05	5.29E-05	Notch3/Pc	6
GO:1900048	GO:1900048	positive regulation of hemostasis	6/385	27/23328	4.29E-06	8.80E-05	5.29E-05	Cd36/Selp	6
GO:0045860	GO:0045860	positive regulation of protein kinase activity	22/385	440/23328	4.68E-06	9.56E-05	5.74E-05	Rgcc/Flt1/	22
GO:0019935	GO:0019935	cyclic-nucleotide-mediated signaling	15/385	223/23328	4.95E-06	0.0001	6.04E-05	Cd36/Aplr	15
GO:0042445	GO:0042445	hormone metabolic process	14/385	196/23328	5.13E-06	0.0001	6.21E-05	Adh7/Reln	14
GO:0006979	GO:0006979	response to oxidative stress	21/385	409/23328	5.13E-06	0.0001	6.21E-05	Cd36/Prnp	21
GO:0007272	GO:0007272	ensheathment of neurons	13/385	170/23328	5.30E-06	0.00011	6.35E-05	Cd9/Clu/N	13
GO:0008366	GO:0008366	axon ensheathment	13/385	170/23328	5.30E-06	0.00011	6.35E-05	Cd9/Clu/N	13
GO:0070167	GO:0070167	regulation of biomineral tissue development	10/385	99/23328	5.64E-06	0.00011	6.69E-05	Mgp/Ptn/S	10
GO:0110149	GO:0110149	regulation of biomineralization	10/385	99/23328	5.64E-06	0.00011	6.69E-05	Mgp/Ptn/S	10
GO:0045807	GO:0045807	positive regulation of endocytosis	11/385	122/23328	5.86E-06	0.00012	6.92E-05	Cd36/Clu/	11
GO:0032271	GO:0032271	regulation of protein polymerization	15/385	227/23328	6.14E-06	0.00012	7.21E-05	Tmsb4x/C	15
GO:0051146	GO:0051146	striated muscle cell differentiation	18/385	317/23328	6.23E-06	0.00012	7.27E-05	Cxcl12/Rg	18
GO:0003206	GO:0003206	cardiac chamber morphogenesis	12/385	147/23328	6.24E-06	0.00012	7.27E-05	Cpe/Tbx1/	12
GO:0002064	GO:0002064	epithelial cell development	16/385	257/23328	6.46E-06	0.00012	7.47E-05	Col15a1/V	16
GO:0031348	GO:0031348	negative regulation of defense response	15/385	228/23328	6.47E-06	0.00012	7.47E-05	Fgl2/Cdh5	15
GO:0001516	GO:0001516	prostaglandin biosynthetic process	6/385	29/23328	6.70E-06	0.00013	7.65E-05	Fabp5/Ptg	6
GO:0046457	GO:0046457	prostanoid biosynthetic process	6/385	29/23328	6.70E-06	0.00013	7.65E-05	Fabp5/Ptg	6
GO:0016485	GO:0016485	protein processing	15/385	229/23328	6.83E-06	0.00013	7.74E-05	Perp/Cpe/	15
GO:0001890	GO:0001890	placenta development	13/385	174/23328	6.83E-06	0.00013	7.74E-05	Htra1/Soc	13
GO:0051235	GO:0051235	maintenance of location	18/385	320/23328	7.09E-06	0.00013	7.99E-05	Cd36/Aplr	18
GO:0043393	GO:0043393	regulation of protein binding	15/385	231/23328	7.58E-06	0.00014	8.51E-05	Id1/Spon1	15
GO:0009612	GO:0009612	response to mechanical stimulus	13/385	176/23328	7.74E-06	0.00014	8.61E-05	Cd36/Cxc	13
GO:0010977	GO:0010977	negative regulation of neuron projection development	13/385	176/23328	7.74E-06	0.00014	8.61E-05	Thy1/Vim/	13
GO:0032835	GO:0032835	glomerulus development	8/385	62/23328	7.95E-06	0.00015	8.81E-05	Cfh/Notch	8
GO:0052547	GO:0052547	regulation of peptidase activity	21/385	421/23328	7.99E-06	0.00015	8.81E-05	Perp/Timp	21
GO:0042310	GO:0042310	vasoconstriction	10/385	103/23328	8.05E-06	0.00015	8.84E-05	Cd38/Pdg	10
GO:0050820	GO:0050820	positive regulation of coagulation	6/385	30/23328	8.25E-06	0.00015	9.02E-05	Cd36/Selp	6
GO:0097191	GO:0097191	extrinsic apoptotic signaling pathway	15/385	233/23328	8.41E-06	0.00015	9.15E-05	Atf3/Sfrp1	15
GO:0001649	GO:0001649	osteoblast differentiation	14/385	205/23328	8.59E-06	0.00015	9.31E-05	Id1/Sfrp1/	14
GO:0050729	GO:0050729	positive regulation of inflammatory response	12/385	152/23328	8.80E-06	0.00016	9.50E-05	Fabp4/Nu	12
GO:0032272	GO:0032272	negative regulation of protein polymerization	9/385	83/23328	9.27E-06	0.00017	9.96E-05	Tmsb4x/C	9
GO:0032967	GO:0032967	positive regulation of collagen biosynthetic process	6/385	31/23328	1.01E-05	0.00018	0.00011	Rgcc/Vim/	6
GO:0007611	GO:0007611	learning or memory	17/385	299/23328	1.11E-05	0.0002	0.00012	Reln/Nts/E	17
GO:0043277	GO:0043277	apoptotic cell clearance	7/385	47/23328	1.12E-05	0.0002	0.00012	Cd36/Sca	7

GO:0050728	GO:0050728	negative regulation of inflammatory response	12/385	156/23328	1.15E-05	0.0002	0.00012	Cdh5/Cd2	12
GO:0010544	GO:0010544	negative regulation of platelet activation	5/385	19/23328	1.15E-05	0.0002	0.00012	Cd9/Pdgfb	5
GO:0043116	GO:0043116	negative regulation of vascular permeability	5/385	19/23328	1.15E-05	0.0002	0.00012	Pde3a/Clc	5
GO:1903707	GO:1903707	negative regulation of hemopoiesis	12/385	157/23328	1.22E-05	0.00021	0.00013	Dtx1/Fgl2/	12
GO:0034754	GO:0034754	cellular hormone metabolic process	10/385	108/23328	1.23E-05	0.00021	0.00013	Adh7/Sca	10
GO:0043112	GO:0043112	receptor metabolic process	14/385	212/23328	1.26E-05	0.00022	0.00013	Cd36/Cd9	14
GO:0048754	GO:0048754	branching morphogenesis of an epithelial tube	13/385	185/23328	1.32E-05	0.00023	0.00014	Flt1/Cxcl1	13
GO:0110053	GO:0110053	regulation of actin filament organization	16/385	273/23328	1.38E-05	0.00024	0.00014	Rgcc/Tms	16
GO:1903038	GO:1903038	negative regulation of leukocyte cell-cell adhesion	11/385	134/23328	1.44E-05	0.00024	0.00015	Dtx1/Fgl2/	11
GO:0002262	GO:0002262	myeloid cell homeostasis	13/385	187/23328	1.49E-05	0.00025	0.00015	Cfh/Adgrf5	13
GO:0006692	GO:0006692	prostanoid metabolic process	7/385	49/23328	1.49E-05	0.00025	0.00015	Fabp5/Pd	7
GO:0006693	GO:0006693	prostaglandin metabolic process	7/385	49/23328	1.49E-05	0.00025	0.00015	Fabp5/Pd	7
GO:0061028	GO:0061028	establishment of endothelial barrier	7/385	49/23328	1.49E-05	0.00025	0.00015	Robo4/Cd	7
GO:1902041	GO:1902041	regulation of extrinsic apoptotic signaling pathway via d	7/385	49/23328	1.49E-05	0.00025	0.00015	Atf3/Sfrp1	7
GO:0071674	GO:0071674	mononuclear cell migration	9/385	89/23328	1.64E-05	0.00027	0.00016	Flt1/Cxcl1	9
GO:0002695	GO:0002695	negative regulation of leukocyte activation	13/385	189/23328	1.66E-05	0.00028	0.00017	Dtx1/Fgl2/	13
GO:0090092	GO:0090092	regulation of transmembrane receptor protein serine/th	15/385	247/23328	1.68E-05	0.00028	0.00017	Sfrp1/Cdh	15
GO:1902105	GO:1902105	regulation of leukocyte differentiation	17/385	309/23328	1.69E-05	0.00028	0.00017	Dtx1/Fgl2/	17
GO:1903522	GO:1903522	regulation of blood circulation	15/385	248/23328	1.76E-05	0.00029	0.00017	Rgs4/Abc	15
GO:0050808	GO:0050808	synapse organization	22/385	479/23328	1.76E-05	0.00029	0.00017	Sparcl1/R	22
GO:0010714	GO:0010714	positive regulation of collagen metabolic process	6/385	34/23328	1.77E-05	0.00029	0.00017	Rgcc/Vim/	6
GO:0033627	GO:0033627	cell adhesion mediated by integrin	8/385	69/23328	1.78E-05	0.00029	0.00017	Plpp3/Itgb	8
GO:0010001	GO:0010001	glial cell differentiation	15/385	249/23328	1.85E-05	0.0003	0.00018	Reln/Dtx1	15
GO:0045862	GO:0045862	positive regulation of proteolysis	18/385	344/23328	1.88E-05	0.0003	0.00018	Perp/Nupr	18
GO:0019430	GO:0019430	removal of superoxide radicals	5/385	21/23328	1.95E-05	0.00031	0.00019	Cd36/Fblr	5
GO:0098754	GO:0098754	detoxification	7/385	51/23328	1.96E-05	0.00031	0.00019	Cd36/Fblr	7
GO:0060485	GO:0060485	mesenchyme development	16/385	281/23328	1.97E-05	0.00031	0.00019	Rgcc/Tcf1	16
GO:0006939	GO:0006939	smooth muscle contraction	10/385	114/23328	1.98E-05	0.00031	0.00019	Cd38/Cav	10
GO:2001236	GO:2001236	regulation of extrinsic apoptotic signaling pathway	12/385	165/23328	2.02E-05	0.00032	0.00019	Atf3/Sfrp1	12
GO:0052548	GO:0052548	regulation of endopeptidase activity	18/385	346/23328	2.03E-05	0.00032	0.00019	Perp/Timp	18
GO:0060348	GO:0060348	bone development	14/385	222/23328	2.11E-05	0.00033	0.0002	Sparc/Kdr	14
GO:0003205	GO:0003205	cardiac chamber development	13/385	194/23328	2.19E-05	0.00034	0.00021	Cpe/Tbx1/	13
GO:0030509	GO:0030509	BMP signaling pathway	12/385	167/23328	2.27E-05	0.00035	0.00021	Id1/Sfrp1/	12
GO:0042552	GO:0042552	myelination	12/385	167/23328	2.27E-05	0.00035	0.00021	Cd9/Clu/N	12
GO:0002792	GO:0002792	negative regulation of peptide secretion	10/385	116/23328	2.30E-05	0.00035	0.00021	Rgcc/Rsa	10
GO:0071901	GO:0071901	negative regulation of protein serine/threonine kinase a	10/385	117/23328	2.48E-05	0.00038	0.00023	Sfrp1/Cdk	10
GO:2000353	GO:2000353	positive regulation of endothelial cell apoptotic process	5/385	22/23328	2.49E-05	0.00038	0.00023	Rgcc/Col1	5
GO:2001026	GO:2001026	regulation of endothelial cell chemotaxis	5/385	22/23328	2.49E-05	0.00038	0.00023	Hspb1/Kd	5
GO:0031103	GO:0031103	axon regeneration	7/385	53/23328	2.53E-05	0.00038	0.00023	Gap43/Tn	7
GO:0043535	GO:0043535	regulation of blood vessel endothelial cell migration	9/385	94/23328	2.56E-05	0.00039	0.00023	Rgcc/Hsp	9
GO:0051017	GO:0051017	actin filament bundle assembly	12/385	169/23328	2.56E-05	0.00039	0.00023	Rgcc/Id1/	12
GO:0060562	GO:0060562	epithelial tube morphogenesis	19/385	387/23328	2.68E-05	0.0004	0.00024	Flt1/Cxcl1	19
GO:0071241	GO:0071241	cellular response to inorganic substance	14/385	227/23328	2.70E-05	0.0004	0.00024	Fabp4/Fn1	14
GO:0150063	GO:0150063	visual system development	20/385	422/23328	2.73E-05	0.00041	0.00024	Flt1/Thy1/	20
GO:0055074	GO:0055074	calcium ion homeostasis	22/385	493/23328	2.73E-05	0.00041	0.00024	Cd36/Aplr	22
GO:0061077	GO:0061077	chaperone-mediated protein folding	7/385	54/23328	2.87E-05	0.00042	0.00025	Unc45b/H	7
GO:0002237	GO:0002237	response to molecule of bacterial origin	19/385	389/23328	2.87E-05	0.00042	0.00025	Cd36/Sca	19
GO:0051494	GO:0051494	negative regulation of cytoskeleton organization	12/385	171/23328	2.88E-05	0.00042	0.00025	Tmsb4x/C	12
GO:0071902	GO:0071902	positive regulation of protein serine/threonine kinase a	16/385	291/23328	3.01E-05	0.00044	0.00026	Rgcc/Flt1/	16
GO:0061572	GO:0061572	actin filament bundle organization	12/385	172/23328	3.05E-05	0.00044	0.00027	Rgcc/Id1/	12
GO:0048880	GO:0048880	sensory system development	20/385	426/23328	3.12E-05	0.00045	0.00027	Flt1/Thy1/	20
GO:0071450	GO:0071450	cellular response to oxygen radical	5/385	23/23328	3.14E-05	0.00045	0.00027	Cd36/Fblr	5
GO:0071451	GO:0071451	cellular response to superoxide	5/385	23/23328	3.14E-05	0.00045	0.00027	Cd36/Fblr	5
GO:0051604	GO:0051604	protein maturation	16/385	294/23328	3.40E-05	0.00049	0.00029	Perp/Cpe/	16
GO:0003231	GO:0003231	cardiac ventricle development	11/385	147/23328	3.42E-05	0.00049	0.00029	Cpe/Ly6e/	11
GO:0030510	GO:0030510	regulation of BMP signaling pathway	9/385	98/23328	3.57E-05	0.00051	0.00031	Sfrp1/Cdh	9
GO:0045926	GO:0045926	negative regulation of growth	15/385	264/23328	3.65E-05	0.00052	0.00031	Rgs4/Sfrp	15
GO:0035924	GO:0035924	cellular response to vascular endothelial growth factor	7/385	56/23328	3.65E-05	0.00052	0.00031	Flt1/Hspb1	7
GO:0007204	GO:0007204	positive regulation of cytosolic calcium ion concentratio	17/385	330/23328	3.89E-05	0.00055	0.00033	Cd36/Aplr	17
GO:0055001	GO:0055001	muscle cell development	13/385	205/23328	3.91E-05	0.00055	0.00033	Rgs4/Lox/	13
GO:0002523	GO:0002523	leukocyte migration involved in inflammatory response	5/385	24/23328	3.92E-05	0.00055	0.00033	Cd24a/Ptr	5
GO:0098869	GO:0098869	cellular oxidant detoxification	5/385	24/23328	3.92E-05	0.00055	0.00033	Cd36/Fblr	5
GO:0006936	GO:0006936	muscle contraction	16/385	298/23328	4.00E-05	0.00056	0.00034	Cd38/Cav	16
GO:0051153	GO:0051153	regulation of striated muscle cell differentiation	10/385	124/23328	4.10E-05	0.00057	0.00034	Cxcl12/Rg	10
GO:0002548	GO:0002548	monocyte chemotaxis	7/385	57/23328	4.10E-05	0.00057	0.00034	Flt1/Cxcl1	7
GO:0043536	GO:0043536	positive regulation of blood vessel endothelial cell migr	7/385	57/23328	4.10E-05	0.00057	0.00034	Hspb1/Kd	7
GO:0034614	GO:0034614	cellular response to reactive oxygen species	11/385	150/23328	4.12E-05	0.00057	0.00034	Cd36/Fblr	11
GO:0071772	GO:0071772	response to BMP	12/385	178/23328	4.27E-05	0.00058	0.00035	Id1/Sfrp1/	12
GO:0071773	GO:0071773	cellular response to BMP stimulus	12/385	178/23328	4.27E-05	0.00058	0.00035	Id1/Sfrp1/	12
GO:0006469	GO:0006469	negative regulation of protein kinase activity	13/385	207/23328	4.32E-05	0.00059	0.00035	Fabp4/Thy	13
GO:0019229	GO:0019229	regulation of vasoconstriction	8/385	78/23328	4.38E-05	0.0006	0.00036	Cd38/Pdg	8
GO:0043270	GO:0043270	positive regulation of ion transport	17/385	334/23328	4.53E-05	0.00061	0.00037	Aplnr/Reln	17
GO:0032092	GO:0032092	positive regulation of protein binding	9/385	101/23328	4.54E-05	0.00061	0.00037	Spon1/Plk	9
GO:0032496	GO:0032496	response to lipopolysaccharide	18/385	368/23328	4.56E-05	0.00061	0.00037	Cd36/Sca	18
GO:0003093	GO:0003093	regulation of glomerular filtration	4/385	13/23328	4.64E-05	0.00062	0.00037	Pdgfb/Gas	4
GO:0071621	GO:0071621	granulocyte chemotaxis	10/385	126/23328	4.70E-05	0.00063	0.00038	Ccl21a/Itg	10
GO:0034368	GO:0034368	protein-lipid complex remodeling	5/385	25/23328	4.83E-05	0.00064	0.00039	Gpihbp1/S	5
GO:0034369	GO:0034369	plasma lipoprotein particle remodeling	5/385	25/23328	4.83E-05	0.00064	0.00039	Gpihbp1/S	5
GO:0050890	GO:0050890	cognition	17/385	336/23328	4.87E-05	0.00065	0.00039	Reln/Nts/E	17
GO:0006874	GO:0006874	cellular calcium ion homeostasis	21/385	476/23328	4.90E-05	0.00065	0.00039	Cd36/Aplr	21
GO:0008277	GO:0008277	regulation of G protein-coupled receptor signaling path	10/385	127/23328	5.03E-05	0.00066	0.0004	Aplnr/Mgll	10
GO:0002791	GO:0002791	regulation of peptide secretion	19/385	406/23328	5.12E-05	0.00067	0.0004	Rgcc/Cd3	19
GO:0022612	GO:0022612	gland morphogenesis	11/385	154/23328	5.25E-05	0.00069	0.00041	Sfrp1/Tnc/	11
GO:0006631	GO:0006631	fatty acid metabolic process	19/385	408/23328	5.47E-05	0.00071	0.00043	Fabp4/Cd	19
GO:0097530	GO:0097530	granulocyte migration	11/385	155/23328	5.56E-05	0.00072	0.00043	Ccl21a/Itg	11
GO:1902106	GO:1902106	negative regulation of leukocyte differentiation	9/385	104/23328	5.72E-05	0.00074	0.00045	Dtx1/Fgl2/	9
GO:2001238	GO:2001238	positive regulation of extrinsic apoptotic signaling path	7/385	60/23328	5.75E-05	0.00074	0.00045	Atf3/Sfrp1	7
GO:2001235	GO:2001235	positive regulation of apoptotic signaling pathway	12/385	184/23328	5.89E-05	0.00075	0.00045	Atf3/Sfrp1	12
GO:0000303	GO:0000303	response to superoxide	5/385	26/23328	5.90E-05	0.00075	0.00045	Cd36/Fblr	5
GO:0042573	GO:0042573	retinoic acid metabolic process	5/385	26/23328	5.90E-05	0.00075	0.00045	Adh7/Rbp	5
GO:0044342	GO:0044342	type B pancreatic cell proliferation	5/385	26/23328	5.90E-05	0.00075	0.00045	Sfrp1/Nup	5
GO:0055094	GO:0055094	response to lipoprotein particle	5/385	26/23328	5.90E-05	0.00075	0.00045	Cd36/Cd9	5
GO:0070168	GO:0070168	negative regulation of biomineral tissue development	5/385	26/23328	5.90E-05	0.00075	0.00045	Srgn/Gas6	5
GO:0110150	GO:0110150	negative regulation of biomineralization	5/385	26/23328	5.90E-05	0.00075	0.00045	Srgn/Gas6	5
GO:0090257	GO:0090257	regulation of muscle system process	14/385	244/23328	5.94E-05	0.00075	0.00045	Rgs4/Igfbp	14
GO:0019915	GO:0019915	lipid storage	8/385	82/23328	6.29E-05	0.00079	0.00047	Cd36/Sca	8
GO:0030500	GO:0030500	regulation of bone mineralization	8/385	82/23328	6.29E-05	0.00079	0.00047	Mgp/Ptn/S	8
GO:0048286	GO:0048286	lung alveolus development	7/385	61/23328	6.40E-05	0.0008	0.00048	Kdr/Igfbp5	7
GO:0032535	GO:0032535	regulation of cellular component size	19/385	413/23328	6.43E-05	0.0008	0.00048	Mgll/C1qtn	19
GO:0051250	GO:0051250	negative regulation of lymphocyte activation	11/385	158/23328	6.62E-05	0.00082	0.00049	Dtx1/Fgl2/	11
GO:0090596	GO:0090596	sensory organ morphogenesis	16/385	311/23328	6.65E-05	0.00082	0.00049	Flt1/Thy1/	16
GO:0051090	GO:0051090	regulation of DNA-binding transcription factor activity	19/385	415/23328	6.85E-05	0.00084	0.00051	Rgcc/Cd3	19
GO:0051899	GO:0051899	membrane depolarization	8/385	83/23328	6.86E-05	0.00084	0.00051	Kdr/Fhl1/E	8
GO:0071496	GO:0071496	cellular response to external stimulus	15/385	280/23328	7.12E-05	0.00087	0.00052	Atf3/Lpl/Itg	15
GO:0000305	GO:0000305	response to oxygen radical	5/385	27/23328	7.14E-05	0.00087	0.00052	Cd36/Fblr	5
GO:0034367	GO:0034367	protein-containing complex remodeling	5/385	27/23328	7.14E-05	0.00087	0.00052	Gpihbp1/S	5
GO:0048745	GO:0048745	smooth muscle tissue development	5/385	27/23328	7.14E-05	0.00087	0.00052	Eng/Bmp4	5
GO:0050709	GO:0050709	negative regulation of protein secretion	9/385	107/23328	7.15E-05	0.00087	0.00052	Rgcc/Rsa	9
GO:0015850	GO:0015850	organic hydroxy compound transport	14/385	249/23328	7.39E-05	0.00089	0.00054	Cd36/Sca	14
GO:0003151	GO:0003151	outflow tract morphogenesis	8/385	84/23328	7.48E-05	0.0009	0.00054	Tbx1/Sox1	8
GO:0001654	GO:0001654	eye development	19/385	418/23328	7.53E-05	0.00091	0.00054	Flt1/Thy1/	19
GO:0006816	GO:0006816	calcium ion transport	19/385	420/23328	8.02E-05	0.00096	0.00058	Aplnr/Cxcl	19
GO:0007568	GO:0007568	aging	14/385	251/23328	8.04E-05	0.00096	0.00058	Cfh/Prnp/I	14
GO:0043114	GO:0043114	regulation of vascular permeability	6/385	44/23328	8.08E-05	0.00096	0.00058	Cdh5/Pde	6
GO:1901800	GO:1901800	positive regulation of proteasomal protein catabolic pro	9/385	109/23328	8.26E-05	0.00098	0.00059	Nupr1/Plk	9
GO:0071402	GO:0071402	cellular response to lipoprotein particle stimulus	5/385	28/23328	8.58E-05	0.00102	0.00061	Cd36/Cd9	5
GO:0030449	GO:0030449	regulation of complement activation	4/385	15/23328	8.63E-05	0.00102	0.00061	Cfh/Serpir	4
GO:0071280	GO:0071280	cellular response to copper ion	4/385	15/23328	8.63E-05	0.00102	0.00061	Prnp/Aqp1	4
GO:0035904	GO:0035904	aorta development	7/385	64/23328	8.74E-05	0.00103	0.00062	Tbx1/Lox/	7
GO:0030324	GO:0030324	lung development	13/385	222/23328	8.83E-05	0.00103	0.00062	Sparc/Id1/	13
GO:0097581	GO:0097581	lamellipodium organization	8/385	86/23328	8.85E-05	0.00103	0.00062	Ablim3/Pc	8
GO:0120192	GO:0120192	tight junction assembly	8/385	86/23328	8.85E-05	0.00103	0.00062	Cdh5/Esa	8
GO:0045619	GO:0045619	regulation of lymphocyte differentiation	12/385	192/23328	8.86E-05	0.00103	0.00062	Dtx1/Fgl2/	12
GO:0017015	GO:0017015	regulation of transforming growth factor beta receptor s	9/385	110/23328	8.86E-05	0.00103	0.00062	Htra1/Lox/	9
GO:0051048	GO:0051048	negative regulation of secretion	13/385	223/23328	9.24E-05	0.00107	0.00064	Rgcc/Rsa	13
GO:0043433	GO:0043433	negative regulation of DNA-binding transcription factor	11/385	164/23328	9.26E-05	0.00107	0.00064	Id1/Nupr1	11
GO:1903531	GO:1903531	negative regulation of secretion by cell	12/385	193/23328	9.31E-05	0.00107	0.00064	Rgcc/Rsa	12
GO:0030323	GO:0030323	respiratory tube development	13/385	225/23328	0.000101065	0.00115	0.00069	Sparc/Id1/	13
GO:0048762	GO:0048762	mesenchymal cell differentiation	13/385	225/23328	0.000101065	0.00115	0.00069	Rgcc/Sfrp	13

GO:0051495	GO:0051495	positive regulation of cytoskeleton organization	13/385	225/23328	0.000101065	0.00115	0.00069	Rgcc/Id1/(	13
GO:1903844	GO:1903844	regulation of cellular response to transforming growth f	9/385	112/23328	0.000101896	0.00116	0.0007	Htra1/Lox/	9
GO:0006636	GO:0006636	unsaturated fatty acid biosynthetic process	6/385	46/23328	0.000104225	0.00118	0.00071	Fabp5/Ptg	6
GO:0043406	GO:0043406	positive regulation of MAP kinase activity	13/385	226/23328	0.000105663	0.0012	0.00072	Flt1/Kitl/P	13
GO:0097006	GO:0097006	regulation of plasma lipoprotein particle levels	7/385	66/23328	0.00010647	0.0012	0.00072	Cd36/Gpil	7
GO:0048738	GO:0048738	cardiac muscle tissue development	14/385	258/23328	0.000107685	0.00121	0.00073	Rgs4/Ly6e	14
GO:0007519	GO:0007519	skeletal muscle tissue development	12/385	196/23328	0.000107771	0.00121	0.00073	Atf3/Nupr1	12
GO:0045834	GO:0045834	positive regulation of lipid metabolic process	11/385	167/23328	0.000108882	0.00122	0.00073	Cd36/Flt1/	11
GO:0033673	GO:0033673	negative regulation of kinase activity	13/385	227/23328	0.000110441	0.00123	0.00074	Fabp4/Thy	13
GO:0001977	GO:0001977	renal system process involved in regulation of blood vo	4/385	16/23328	0.000113598	0.00126	0.00076	Pdgfb/Gas	4
GO:0071404	GO:0071404	cellular response to low-density lipoprotein particle stin	4/385	16/23328	0.000113598	0.00126	0.00076	Cd36/Cd9	4
GO:1902430	GO:1902430	negative regulation of amyloid-beta formation	4/385	16/23328	0.000113598	0.00126	0.00076	Spon1/Prn	4
GO:0051348	GO:0051348	negative regulation of transferase activity	14/385	260/23328	0.000116808	0.00129	0.00078	Fabp4/Thy	14
GO:0010718	GO:0010718	positive regulation of epithelial to mesenchymal transit	6/385	47/23328	0.000117832	0.00129	0.00078	Rgcc/Pdp	6
GO:0045581	GO:0045581	negative regulation of T cell differentiation	6/385	47/23328	0.000117832	0.00129	0.00078	Dtx1/Fgl2/	6
GO:0051258	GO:0051258	protein polymerization	15/385	293/23328	0.000117946	0.00129	0.00078	Tmsb4x/C	15
GO:0035767	GO:0035767	endothelial cell chemotaxis	5/385	30/23328	0.000121016	0.00132	0.00079	Hspb1/Kd	5
GO:0001894	GO:0001894	tissue homeostasis	14/385	261/23328	0.000121616	0.00133	0.0008	Adgrf5/Cd	14
GO:0048732	GO:0048732	gland development	20/385	471/23328	0.000123688	0.00134	0.00081	Sfrp1/Tbx	20
GO:0042632	GO:0042632	cholesterol homeostasis	8/385	91/23328	0.00013194	0.00143	0.00086	Fabp4/Gp	8
GO:0120193	GO:0120193	tight junction organization	8/385	91/23328	0.00013194	0.00143	0.00086	Cdh5/Esa	8
GO:0048872	GO:0048872	homeostasis of number of cells	16/385	330/23328	0.000132444	0.00143	0.00086	Cfh/Adgrf5	16
GO:0045907	GO:0045907	positive regulation of vasoconstriction	6/385	48/23328	0.00013281	0.00143	0.00086	Cd38/Pdg	6
GO:0070527	GO:0070527	platelet aggregation	6/385	48/23328	0.00013281	0.00143	0.00086	Cd9/Cfh/F	6
GO:0048678	GO:0048678	response to axon injury	7/385	69/23328	0.000141388	0.0015	0.0009	Gap43/Tn	7
GO:0062197	GO:0062197	cellular response to chemical stress	16/385	332/23328	0.000141933	0.0015	0.0009	Cd36/Hsp	16
GO:0002828	GO:0002828	regulation of type 2 immune response	5/385	31/23328	0.000142347	0.0015	0.0009	Rsad2/Il3	5
GO:0032373	GO:0032373	positive regulation of sterol transport	5/385	31/23328	0.000142347	0.0015	0.0009	Pltp/Anxa	5
GO:0032376	GO:0032376	positive regulation of cholesterol transport	5/385	31/23328	0.000142347	0.0015	0.0009	Pltp/Anxa	5
GO:0050901	GO:0050901	leukocyte tethering or rolling	5/385	31/23328	0.000142347	0.0015	0.0009	Cxcl12/Se	5
GO:0055092	GO:0055092	sterol homeostasis	8/385	92/23328	0.000142457	0.0015	0.0009	Fabp4/Gp	8
GO:0001886	GO:0001886	endothelial cell morphogenesis	4/385	17/23328	0.000146622	0.00153	0.00092	Col15a1/I	4
GO:0033623	GO:0033623	regulation of integrin activation	4/385	17/23328	0.000146622	0.00153	0.00092	Selp/Cd24	4
GO:2001028	GO:2001028	positive regulation of endothelial cell chemotaxis	4/385	17/23328	0.000146622	0.00153	0.00092	Hspb1/Kd	4
GO:0031346	GO:0031346	positive regulation of cell projection organization	20/385	478/23328	0.000150523	0.00157	0.00094	Reln/Cxcl	20
GO:0019221	GO:0019221	cytokine-mediated signaling pathway	17/385	369/23328	0.00015209	0.00158	0.00095	Cxcl12/Irf	17
GO:1903364	GO:1903364	positive regulation of cellular protein catabolic process	10/385	145/23328	0.000152681	0.00159	0.00095	Nupr1/Plk	10
GO:0055008	GO:0055008	cardiac muscle tissue morphogenesis	7/385	70/23328	0.000154899	0.0016	0.00096	Ly6e/S1pr	7
GO:0042476	GO:0042476	odontogenesis	9/385	119/23328	0.000162156	0.00168	0.00101	Perp/Tbx1	9
GO:0010717	GO:0010717	regulation of epithelial to mesenchymal transition	8/385	94/23328	0.00016557	0.0017	0.00102	Rgcc/Sfrp	8
GO:0015908	GO:0015908	fatty acid transport	8/385	94/23328	0.00016557	0.0017	0.00102	Fabp4/Cd	8
GO:0001893	GO:0001893	maternal placenta development	5/385	32/23328	0.000166439	0.0017	0.00102	Ptgis/Junt	5
GO:0060669	GO:0060669	embryonic placenta morphogenesis	5/385	32/23328	0.000166439	0.0017	0.00102	Socs3/Cd	5
GO:0007492	GO:0007492	endoderm development	7/385	71/23328	0.000169436	0.00173	0.00104	Fn1/Col5a	7
GO:0031333	GO:0031333	negative regulation of protein-containing complex asse	10/385	147/23328	0.000170839	0.00174	0.00105	Tmsb4x/C	10
GO:0060538	GO:0060538	skeletal muscle organ development	12/385	206/23328	0.000171971	0.00174	0.00105	Atf3/Nupr1	12
GO:0030282	GO:0030282	bone mineralization	9/385	120/23328	0.000172796	0.00174	0.00105	Mgp/Lox/F	9
GO:0033559	GO:0033559	unsaturated fatty acid metabolic process	9/385	120/23328	0.000172796	0.00174	0.00105	Mgll/Fabp	9
GO:0030098	GO:0030098	lymphocyte differentiation	18/385	409/23328	0.000172924	0.00174	0.00105	Dtx1/Fgl2/	18
GO:0044403	GO:0044403	symbiotic process	17/385	374/23328	0.000178373	0.00179	0.00108	Scarb1/Rs	17
GO:0031032	GO:0031032	actomyosin structure organization	12/385	207/23328	0.000179895	0.0018	0.00108	Rgcc/Cgn	12
GO:0035265	GO:0035265	organ growth	12/385	207/23328	0.000179895	0.0018	0.00108	Rgs4/Kdr/	12
GO:2000377	GO:2000377	regulation of reactive oxygen species metabolic proces	12/385	207/23328	0.000179895	0.0018	0.00108	Cd36/Fbln	12
GO:0002688	GO:0002688	regulation of leukocyte chemotaxis	9/385	121/23328	0.000184012	0.00183	0.0011	Cxcl12/Lg	9
GO:0051480	GO:0051480	regulation of cytosolic calcium ion concentration	17/385	375/23328	0.000184081	0.00183	0.0011	Cd36/Aplr	17
GO:0045123	GO:0045123	cellular extravasation	7/385	72/23328	0.000185057	0.00184	0.0011	Cxcl12/Th	7
GO:1902992	GO:1902992	negative regulation of amyloid precursor protein catabo	4/385	18/23328	0.000186068	0.00184	0.0011	Spon1/Prn	4
GO:1990000	GO:1990000	amyloid fibril formation	4/385	18/23328	0.000186068	0.00184	0.0011	Cd36/Clu/	4
GO:0034101	GO:0034101	erythrocyte homeostasis	10/385	149/23328	0.000190782	0.00188	0.00113	Adgrf5/Lm	10
GO:0002690	GO:0002690	positive regulation of leukocyte chemotaxis	8/385	96/23328	0.000191682	0.00188	0.00113	Cxcl12/Lg	8
GO:0050999	GO:0050999	regulation of nitric-oxide synthase activity	5/385	33/23328	0.000193524	0.0019	0.00114	Scarb1/Er	5
GO:0050868	GO:0050868	negative regulation of T cell activation	9/385	122/23328	0.000195827	0.00191	0.00115	Dtx1/Fgl2/	9
GO:0034765	GO:0034765	regulation of ion transmembrane transport	20/385	489/23328	0.000203107	0.00198	0.00119	Aplnr/Reln	20
GO:0043588	GO:0043588	skin development	14/385	275/23328	0.000209292	0.00203	0.00122	Tcf15/Col5	14
GO:0051924	GO:0051924	regulation of calcium ion transport	14/385	275/23328	0.000209292	0.00203	0.00122	Aplnr/Cxc	14
GO:1903037	GO:1903037	regulation of leukocyte cell-cell adhesion	15/385	309/23328	0.000210536	0.00204	0.00123	Dtx1/Fgl2/	15
GO:0034599	GO:0034599	cellular response to oxidative stress	14/385	276/23328	0.000217241	0.00209	0.00126	Cd36/Hsp	14
GO:0061448	GO:0061448	connective tissue development	14/385	276/23328	0.000217241	0.00209	0.00126	Mgp/Lox/C	14
GO:0007266	GO:0007266	Rho protein signal transduction	11/385	181/23328	0.000220938	0.00212	0.00127	Mcf2l/Pdp	11
GO:1903052	GO:1903052	positive regulation of proteolysis involved in cellular pr	9/385	124/23328	0.000221356	0.00212	0.00127	Nupr1/Plk	9
GO:1990266	GO:1990266	neutrophil migration	9/385	124/23328	0.000221356	0.00212	0.00127	Ccl21a/Itg	9
GO:0098868	GO:0098868	bone growth	5/385	34/23328	0.000223841	0.00214	0.00129	Kdr/Fbln5/	5
GO:0048592	GO:0048592	eye morphogenesis	11/385	182/23328	0.000231744	0.00221	0.00132	Flt1/Thy1/	11
GO:0048662	GO:0048662	negative regulation of smooth muscle cell proliferation	6/385	53/23328	0.00023183	0.00221	0.00132	Igfbp5/Igfl	6
GO:0071071	GO:0071071	regulation of phospholipid biosynthetic process	4/385	19/23328	0.000232629	0.00221	0.00133	Adgrf5/Pd	4
GO:0051651	GO:0051651	maintenance of location in cell	12/385	213/23328	0.000234291	0.00222	0.00133	Aplnr/Tms	12
GO:1903409	GO:1903409	reactive oxygen species biosynthetic process	9/385	125/23328	0.000235122	0.00222	0.00133	Cd36/Duo	9
GO:0030593	GO:0030593	neutrophil chemotaxis	8/385	99/23328	0.000237117	0.00223	0.00134	Ccl21a/Itg	8
GO:0051145	GO:0051145	smooth muscle cell differentiation	7/385	75/23328	0.000239026	0.00224	0.00135	Tmsb4x/P	7
GO:1903725	GO:1903725	regulation of phospholipid metabolic process	7/385	75/23328	0.000239026	0.00224	0.00135	Flt1/Scarb	7
GO:0008016	GO:0008016	regulation of heart contraction	11/385	183/23328	0.000242993	0.00228	0.00137	Rgs4/Abc	11
GO:0019722	GO:0019722	calcium-mediated signaling	12/385	214/23328	0.000244596	0.00228	0.00137	Selp/Ackr	12
GO:0034113	GO:0034113	heterotypic cell-cell adhesion	6/385	54/23328	0.000257223	0.00238	0.00143	Thy1/Perp	6
GO:0046456	GO:0046456	icosanoid biosynthetic process	6/385	54/23328	0.000257223	0.00238	0.00143	Fabp5/Ptg	6
GO:1904705	GO:1904705	regulation of vascular associated smooth muscle cell p	6/385	54/23328	0.000257223	0.00238	0.00143	Igfbp5/Pd	6
GO:1990874	GO:1990874	vascular associated smooth muscle cell proliferation	6/385	54/23328	0.000257223	0.00238	0.00143	Igfbp5/Pd	6
GO:0046686	GO:0046686	response to cadmium ion	5/385	35/23328	0.000257641	0.00238	0.00143	Prnp/Fos/	5
GO:0006457	GO:0006457	protein folding	10/385	155/23328	0.000262721	0.00242	0.00146	Unc45b/H	10
GO:0090090	GO:0090090	negative regulation of canonical Wnt signaling pathway	9/385	127/23328	0.000264788	0.00244	0.00146	Tmem88/S	9
GO:0031644	GO:0031644	regulation of nervous system process	11/385	185/23328	0.00026688	0.00245	0.00147	Mgll/Reln/	11
GO:0006809	GO:0006809	nitric oxide biosynthetic process	7/385	77/23328	0.000281563	0.00258	0.00155	Cd36/Cd3	7
GO:0003044	GO:0003044	regulation of systemic arterial blood pressure mediated	6/385	55/23328	0.000284751	0.0026	0.00156	Ece1/Gja5	6
GO:0006826	GO:0006826	iron ion transport	6/385	55/23328	0.000284751	0.0026	0.00156	Cp/Steap	6
GO:0002577	GO:0002577	regulation of antigen processing and presentation	4/385	20/23328	0.000287016	0.0026	0.00156	Fgl2/Thbs	4
GO:0010875	GO:0010875	positive regulation of cholesterol efflux	4/385	20/23328	0.000287016	0.0026	0.00156	Pltp/Cav1	4
GO:0035584	GO:0035584	calcium-mediated signaling using intracellular calcium	4/385	20/23328	0.000287016	0.0026	0.00156	Selp/Prnp	4
GO:0048545	GO:0048545	response to steroid hormone	12/385	218/23328	0.000289763	0.00262	0.00157	Sfrp1/Lox	12
GO:0060070	GO:0060070	canonical Wnt signaling pathway	14/385	284/23328	0.000290743	0.00262	0.00157	Tmem88/S	14
GO:0051098	GO:0051098	regulation of binding	17/385	390/23328	0.000290954	0.00262	0.00157	Id1/Spon1	17
GO:0034381	GO:0034381	plasma lipoprotein particle clearance	5/385	36/23328	0.000295179	0.00265	0.00159	Cd36/Gpil	5
GO:0035909	GO:0035909	aorta morphogenesis	5/385	36/23328	0.000295179	0.00265	0.00159	Tbx1/Eng/	5
GO:0003208	GO:0003208	cardiac ventricle morphogenesis	7/385	78/23328	0.000305001	0.00272	0.00164	Cpe/Ly6e/	7
GO:0008625	GO:0008625	extrinsic apoptotic signaling pathway via death domain	7/385	78/23328	0.000305001	0.00272	0.00164	Atf3/Sfrp1	7
GO:0070838	GO:0070838	divalent metal ion transport	19/385	467/23328	0.000311019	0.00277	0.00166	Aplnr/Cxc	19
GO:0050732	GO:0050732	negative regulation of peptidyl-tyrosine phosphorylatio	6/385	56/23328	0.00031454	0.00279	0.00168	Thy1/Sfrp	6
GO:0032368	GO:0032368	regulation of lipid transport	9/385	130/23328	0.000315055	0.00279	0.00168	Pltp/Igfbp	9
GO:0060047	GO:0060047	heart contraction	12/385	221/23328	0.000328117	0.0029	0.00174	Rgs4/Abc	12
GO:0062012	GO:0062012	regulation of small molecule metabolic process	16/385	358/23328	0.000331119	0.00292	0.00176	Nupr1/Fab	16
GO:0060541	GO:0060541	respiratory system development	13/385	254/23328	0.000332154	0.00293	0.00176	Sparc/Id1/	13
GO:0031623	GO:0031623	receptor internalization	9/385	131/23328	0.000333467	0.00293	0.00176	Cd36/Cd9	9
GO:0055002	GO:0055002	striated muscle cell development	11/385	190/23328	0.000335414	0.00294	0.00177	Rgs4/Lox/	11
GO:0072511	GO:0072511	divalent inorganic cation transport	19/385	470/23328	0.000336677	0.00294	0.00177	Aplnr/Cxc	19
GO:1990748	GO:1990748	cellular detoxification	5/385	37/23328	0.00033672	0.00294	0.00177	Cd36/Fbln	5
GO:0030100	GO:0030100	regulation of endocytosis	12/385	222/23328	0.000341821	0.00297	0.00179	Cd36/Clu/	12
GO:0045732	GO:0045732	positive regulation of protein catabolic process	12/385	222/23328	0.000341821	0.00297	0.00179	Nupr1/Plk	12
GO:0002043	GO:0002043	blood vessel endothelial cell proliferation involved in s	4/385	21/23328	0.000349955	0.00303	0.00182	Aplnr/Nrar	4
GO:0046697	GO:0046697	decidualization	4/385	21/23328	0.000349955	0.00303	0.00182	Ptgis/Junt	4
GO:1904062	GO:1904062	regulation of cation transmembrane transport	16/385	361/23328	0.000362973	0.00314	0.00189	Aplnr/Reln	16
GO:0045637	GO:0045637	regulation of myeloid cell differentiation	12/385	224/23328	0.000370688	0.0032	0.00192	Sfrp1/Lmc	12
GO:0014812	GO:0014812	muscle cell migration	8/385	106/23328	0.000378052	0.00326	0.00196	Tmsb4x/Ig	8
GO:0010831	GO:0010831	positive regulation of myotube differentiation	5/385	38/23328	0.000382537	0.00327	0.00197	Cxcl12/Tb	5
GO:0038084	GO:0038084	vascular endothelial growth factor signaling pathway	5/385	38/23328	0.000382537	0.00327	0.00197	Hspb1/Kd	5
GO:1904037	GO:1904037	positive regulation of epithelial cell apoptotic process	5/385	38/23328	0.000382537	0.00327	0.00197	Rgcc/Col1	5
GO:0007264	GO:0007264	small GTPase mediated signal transduction	18/385	437/23328	0.000384471	0.00327	0.00197	Reln/Mcf2	18
GO:0045165	GO:0045165	cell fate commitment	14/385	292/23328	0.000384634	0.00327	0.00197	Gap43/Sfr	14
GO:0046209	GO:0046209	nitric oxide metabolic process	7/385	81/23328	0.000384853	0.00327	0.00197	Cd36/Cd3	7
GO:1901652	GO:1901652	response to peptide	17/385	400/23328	0.000389065	0.0033	0.00198	Cd36/C1q	17
GO:0110020	GO:0110020	regulation of actomyosin structure organization	8/385	107/23328	0.000402826	0.00341	0.00205	Rgcc/Cgn	8
GO:0050708	GO:0050708	regulation of protein secretion	16/385	365/23328	0.000409532	0.00346	0.00208	Rgcc/Cd3	16

GO:0051224	GO:0051224	negative regulation of protein transport	10/385	164/23328	0.000412278	0.00348	0.00209	Rgcc/Cd3	10
GO:0032370	GO:0032370	positive regulation of lipid transport	7/385	82/23328	0.000414899	0.00349	0.0021	Pltp/Anxa	7
GO:0001818	GO:0001818	negative regulation of cytokine production	13/385	260/23328	0.000414959	0.00349	0.0021	Rgcc/Prnp	13
GO:0050731	GO:0050731	positive regulation of peptidyl-tyrosine phosphorylation	11/385	195/23328	0.000418188	0.00351	0.00211	Cd36/Reln	11
GO:0002504	GO:0002504	antigen processing and presentation of peptide or poly	4/385	22/23328	0.000422184	0.00353	0.00212	H2-DMb1/	4
GO:0072215	GO:0072215	regulation of metanephros development	4/385	22/23328	0.000422184	0.00353	0.00212	Pdgfb/Egr	4
GO:0043266	GO:0043266	regulation of potassium ion transport	8/385	108/23328	0.000428905	0.00358	0.00215	Rgs4/Prnp	8
GO:0071827	GO:0071827	plasma lipoprotein particle organization	5/385	39/23328	0.000432909	0.0036	0.00216	Gpihbp1/S	5
GO:0072330	GO:0072330	monocarboxylic acid biosynthetic process	11/385	196/23328	0.000436645	0.00363	0.00218	Mgll/Lpl/F	11
GO:0051928	GO:0051928	positive regulation of calcium ion transport	9/385	136/23328	0.000439373	0.00364	0.00219	Aplnr/Cxc	9
GO:0048260	GO:0048260	positive regulation of receptor-mediated endocytosis	6/385	60/23328	0.000458905	0.0038	0.00228	Clu/B2m/	6
GO:0043010	GO:0043010	camera-type eye development	16/385	370/23328	0.000474889	0.00392	0.00236	Flt1/Thy1/	16
GO:0070830	GO:0070830	bicellular tight junction assembly	7/385	84/23328	0.000480608	0.00396	0.00238	Cdh5/Esa	7
GO:0032612	GO:0032612	interleukin-1 production	8/385	110/23328	0.000485178	0.00399	0.0024	Cd36/Hsp	8
GO:1901653	GO:1901653	cellular response to peptide	14/385	299/23328	0.000486958	0.00399	0.0024	Cd36/C1q	14
GO:0042092	GO:0042092	type 2 immune response	5/385	40/23328	0.000488122	0.00399	0.0024	Rsad2/Il3	5
GO:0003015	GO:0003015	heart process	12/385	231/23328	0.00048851	0.00399	0.0024	Rgs4/Abc	12
GO:0035023	GO:0035023	regulation of Rho protein signal transduction	9/385	138/23328	0.000488828	0.00399	0.0024	Mcf2l/Arh	9
GO:0034370	GO:0034370	triglyceride-rich lipoprotein particle remodeling	3/385	10/23328	0.000491036	0.004	0.0024	Gpihbp1/L	3
GO:0030308	GO:0030308	negative regulation of cell growth	11/385	199/23328	0.000496162	0.00403	0.00242	Rgs4/Sfrp	11
GO:0022409	GO:0022409	positive regulation of cell-cell adhesion	13/385	265/23328	0.000496775	0.00403	0.00242	Thy1/Selp	13
GO:0048638	GO:0048638	regulation of developmental growth	17/385	409/23328	0.000500621	0.00405	0.00244	Mgll/Cxcl1	17
GO:0010976	GO:0010976	positive regulation of neuron projection development	16/385	372/23328	0.000503436	0.00406	0.00244	Reln/Cxcl	16
GO:0042572	GO:0042572	retinol metabolic process	4/385	23/23328	0.00050445	0.00406	0.00244	Adh7/Rbp	4
GO:0071276	GO:0071276	cellular response to cadmium ion	4/385	23/23328	0.00050445	0.00406	0.00244	Fos/Mt1/J	4
GO:0030301	GO:0030301	cholesterol transport	7/385	85/23328	0.000516434	0.00414	0.00249	Cd36/Sca	7
GO:0051216	GO:0051216	cartilage development	11/385	200/23328	0.000517449	0.00414	0.00249	Mgp/Cytl1	11
GO:1902905	GO:1902905	positive regulation of supramolecular fiber organizatior	11/385	200/23328	0.000517449	0.00414	0.00249	Rgcc/Id1/	11
GO:1904950	GO:1904950	negative regulation of establishment of protein localiza	10/385	169/23328	0.000522217	0.00417	0.00251	Rgcc/Cd3	10
GO:0060828	GO:0060828	regulation of canonical Wnt signaling pathway	12/385	234/23328	0.000547894	0.00436	0.00262	Tmem88/S	12
GO:0098801	GO:0098801	regulation of renal system process	5/385	41/23328	0.000548468	0.00436	0.00262	Pdgfb/Gas	5
GO:0015918	GO:0015918	sterol transport	7/385	86/23328	0.000554355	0.00439	0.00264	Cd36/Sca	7
GO:2001057	GO:2001057	reactive nitrogen species metabolic process	7/385	86/23328	0.000554355	0.00439	0.00264	Cd36/Cd3	7
GO:0000302	GO:0000302	response to reactive oxygen species	11/385	202/23328	0.000562318	0.00443	0.00266	Cd36/Fbln	11
GO:0048736	GO:0048736	appendage development	11/385	202/23328	0.000562318	0.00443	0.00266	Smoc1/Ho	11
GO:0060173	GO:0060173	limb development	11/385	202/23328	0.000562318	0.00443	0.00266	Smoc1/Ho	11
GO:0034103	GO:0034103	regulation of tissue remodeling	7/385	87/23328	0.000594457	0.00468	0.00281	Sfrp1/Cd3	7
GO:1900077	GO:1900077	negative regulation of cellular response to insulin stimu	5/385	42/23328	0.000614247	0.00482	0.00289	Socs3/Lpl	5
GO:0001892	GO:0001892	embryonic placenta development	8/385	114/23328	0.000615686	0.00482	0.00289	Socs3/Cd	8
GO:0010927	GO:0010927	cellular component assembly involved in morphogenes	8/385	114/23328	0.000615686	0.00482	0.00289	Cd9/Csrp	8
GO:0043409	GO:0043409	negative regulation of MAPK cascade	10/385	173/23328	0.000626747	0.00489	0.00294	Atf3/Sfrp1	10
GO:0032436	GO:0032436	positive regulation of proteasomal ubiquitin-dependent	7/385	88/23328	0.000636828	0.00495	0.00298	Plk2/Clu/I	7
GO:0035914	GO:0035914	skeletal muscle cell differentiation	7/385	88/23328	0.000636828	0.00495	0.00298	Atf3/Nupr	7
GO:0015909	GO:0015909	long-chain fatty acid transport	6/385	64/23328	0.000650318	0.00505	0.00303	Fabp4/Cd	6
GO:0072089	GO:0072089	stem cell proliferation	9/385	144/23328	0.000665304	0.00511	0.00307	Cd34/Kitl/	9
GO:0002468	GO:0002468	dendritic cell antigen processing and presentation	3/385	11/23328	0.000666929	0.00511	0.00307	Fgl2/Thbs	3
GO:0043568	GO:0043568	positive regulation of insulin-like growth factor receptor	3/385	11/23328	0.000666929	0.00511	0.00307	Igfbp5/Igf	3
GO:0097084	GO:0097084	vascular associated smooth muscle cell development	3/385	11/23328	0.000666929	0.00511	0.00307	Eng/Adm/	3
GO:0097278	GO:0097278	complement-dependent cytotoxicity	3/385	11/23328	0.000666929	0.00511	0.00307	Cfh/C3/Cc	3
GO:0150104	GO:0150104	transport across blood-brain barrier	3/385	11/23328	0.000666929	0.00511	0.00307	Cd36/Fab	3
GO:2001214	GO:2001214	positive regulation of vasculogenesis	3/385	11/23328	0.000666929	0.00511	0.00307	Kdr/Cd34/	3
GO:0010639	GO:0010639	negative regulation of organelle organization	17/385	420/23328	0.000673484	0.00515	0.0031	Rgcc/Tms	17
GO:0001656	GO:0001656	metanephros development	7/385	89/23328	0.000681561	0.0052	0.00313	Id3/Pdgfb	7
GO:0006813	GO:0006813	potassium ion transport	12/385	240/23328	0.000684996	0.00521	0.00313	Rgs4/Abc	12
GO:0071825	GO:0071825	protein-lipid complex subunit organization	5/385	43/23328	0.000685764	0.00521	0.00313	Gpihbp1/S	5
GO:1903053	GO:1903053	regulation of extracellular matrix organization	5/385	43/23328	0.000685764	0.00521	0.00313	Rgcc/Tcf1	5
GO:0019882	GO:0019882	antigen processing and presentation	8/385	116/23328	0.00069082	0.00524	0.00315	Fgl2/B2m	8
GO:0010884	GO:0010884	positive regulation of lipid storage	4/385	25/23328	0.000702123	0.0053	0.00319	Cd36/Sca	4
GO:1902230	GO:1902230	negative regulation of intrinsic apoptotic signaling path	4/385	25/23328	0.000702123	0.0053	0.00319	Cxcl12/Cl	4
GO:0042246	GO:0042246	tissue regeneration	6/385	65/23328	0.000706635	0.00533	0.0032	Cd9/Gap4	6
GO:0060249	GO:0060249	anatomical structure homeostasis	17/385	422/23328	0.000709871	0.00534	0.00321	Adgrf5/Cd	17
GO:0019233	GO:0019233	sensory perception of pain	9/385	146/23328	0.000734549	0.00552	0.00332	Mgll/Cxcl1	9
GO:0098742	GO:0098742	cell-cell adhesion via plasma-membrane adhesion mol	11/385	209/23328	0.000745746	0.00559	0.00336	Sparcl1/P	11
GO:0035051	GO:0035051	cardiocyte differentiation	10/385	177/23328	0.000747992	0.0056	0.00336	Rgs4/Pdg	10
GO:0015711	GO:0015711	organic anion transport	18/385	463/23328	0.000753762	0.00563	0.00338	Fabp4/Cd	18
GO:0032768	GO:0032768	regulation of monooxygenase activity	5/385	44/23328	0.000763328	0.00566	0.0034	Scarb1/Er	5
GO:0071385	GO:0071385	cellular response to glucocorticoid stimulus	5/385	44/23328	0.000763328	0.00566	0.0034	Aqp1/Zfp3	5
GO:0097237	GO:0097237	cellular response to toxic substance	5/385	44/23328	0.000763328	0.00566	0.0034	Cd36/Fbln	5
GO:1902895	GO:1902895	positive regulation of pri-miRNA transcription by RNA p	5/385	44/23328	0.000763328	0.00566	0.0034	Pdgfb/Fos	5
GO:0021782	GO:0021782	glial cell development	8/385	118/23328	0.000773172	0.00572	0.00344	Vim/Clu/P	8
GO:0007265	GO:0007265	Ras protein signal transduction	15/385	350/23328	0.000774832	0.00572	0.00344	Mcf2l/Plk2	15
GO:2000027	GO:2000027	regulation of animal organ morphogenesis	11/385	210/23328	0.000775605	0.00572	0.00344	Sfrp1/Tbx	11
GO:0014033	GO:0014033	neural crest cell differentiation	7/385	91/23328	0.00077848	0.00573	0.00344	Sfrp1/Tbx	7
GO:0072091	GO:0072091	regulation of stem cell proliferation	7/385	91/23328	0.00077848	0.00573	0.00344	Kitl/Cd24a	7
GO:0010769	GO:0010769	regulation of cell morphogenesis involved in differentia	15/385	351/23328	0.000797547	0.00586	0.00352	Mgll/Reln/	15
GO:0050927	GO:0050927	positive regulation of positive chemotaxis	4/385	26/23328	0.00081906	0.006	0.00361	Cxcl12/Cc	4
GO:0006940	GO:0006940	regulation of smooth muscle contraction	6/385	67/23328	0.000830529	0.00605	0.00363	Cav1/Calc	6
GO:0046847	GO:0046847	filopodium assembly	6/385	67/23328	0.000830529	0.00605	0.00363	Gap43/Itg	6
GO:1901570	GO:1901570	fatty acid derivative biosynthetic process	6/385	67/23328	0.000830529	0.00605	0.00363	Fabp5/Ptg	6
GO:0014909	GO:0014909	smooth muscle cell migration	7/385	92/23328	0.000830859	0.00605	0.00363	Tmsb4x/Ig	7
GO:0010464	GO:0010464	regulation of mesenchymal cell proliferation	5/385	45/23328	0.000847257	0.00614	0.00369	Tbx1/Kdr/	5
GO:0061756	GO:0061756	leukocyte adhesion to vascular endothelial cell	5/385	45/23328	0.000847257	0.00614	0.00369	Cxcl12/Se	5
GO:0007613	GO:0007613	memory	9/385	149/23328	0.000849341	0.00614	0.00369	Reln/Plk2	9
GO:0010466	GO:0010466	negative regulation of peptidase activity	12/385	246/23328	0.000849744	0.00614	0.00369	Timp4/Aq	12
GO:0032680	GO:0032680	regulation of tumor necrosis factor production	8/385	120/23328	0.000863238	0.00623	0.00374	Cd36/Cd3	8
GO:0003157	GO:0003157	endocardium development	3/385	12/23328	0.000878386	0.00624	0.00375	Kdr/Sox17	3
GO:0006957	GO:0006957	complement activation, alternative pathway	3/385	12/23328	0.000878386	0.00624	0.00375	Cfh/Cfd/C	3
GO:0010232	GO:0010232	vascular transport	3/385	12/23328	0.000878386	0.00624	0.00375	Cd36/Fab	3
GO:0030949	GO:0030949	positive regulation of vascular endothelial growth facto	3/385	12/23328	0.000878386	0.00624	0.00375	Kdr/Tek/B	3
GO:0031392	GO:0031392	regulation of prostaglandin biosynthetic process	3/385	12/23328	0.000878386	0.00624	0.00375	Fabp5/Cd	3
GO:0034375	GO:0034375	high-density lipoprotein particle remodeling	3/385	12/23328	0.000878386	0.00624	0.00375	Scarb1/Pl	3
GO:0072124	GO:0072124	regulation of glomerular mesangial cell proliferation	3/385	12/23328	0.000878386	0.00624	0.00375	Pdgfb/Egr	3
GO:1900272	GO:1900272	negative regulation of long-term synaptic potentiation	3/385	12/23328	0.000878386	0.00624	0.00375	Prnp/Ptn/	3
GO:1902947	GO:1902947	regulation of tau-protein kinase activity	3/385	12/23328	0.000878386	0.00624	0.00375	Clu/Egr1/	3
GO:0043297	GO:0043297	apical junction assembly	7/385	93/23328	0.00088598	0.00628	0.00378	Cdh5/Esa	7
GO:0030032	GO:0030032	lamellipodium assembly	6/385	68/23328	0.000898434	0.00634	0.00381	Ablim3/Ac	6
GO:0030837	GO:0030837	negative regulation of actin filament polymerization	6/385	68/23328	0.000898434	0.00634	0.00381	Tmsb4x/P	6
GO:0032507	GO:0032507	maintenance of protein location in cell	6/385	68/23328	0.000898434	0.00634	0.00381	Tmsb4x/S	6
GO:0010951	GO:0010951	negative regulation of endopeptidase activity	10/385	182/23328	0.000926081	0.00652	0.00392	Timp4/Aq	10
GO:0003254	GO:0003254	regulation of membrane depolarization	5/385	46/23328	0.000937871	0.00655	0.00394	Kdr/Fhl1/	5
GO:0031952	GO:0031952	regulation of protein autophosphorylation	5/385	46/23328	0.000937871	0.00655	0.00394	Pdgfb/Eng	5
GO:0033344	GO:0033344	cholesterol efflux	5/385	46/23328	0.000937871	0.00655	0.00394	Scarb1/Pl	5
GO:0071384	GO:0071384	cellular response to corticosteroid stimulus	5/385	46/23328	0.000937871	0.00655	0.00394	Aqp1/Zfp3	5
GO:0045666	GO:0045666	positive regulation of neuron differentiation	18/385	472/23328	0.00093836	0.00655	0.00394	Reln/Cxcl	18
GO:0008154	GO:0008154	actin polymerization or depolymerization	11/385	215/23328	0.000940194	0.00655	0.00394	Tmsb4x/E	11
GO:0002478	GO:0002478	antigen processing and presentation of exogenous pep	4/385	27/23328	0.000949087	0.00655	0.00394	B2m/H2-D	4
GO:0003071	GO:0003071	renal system process involved in regulation of systemi	4/385	27/23328	0.000949087	0.00655	0.00394	Pdgfb/Gas	4
GO:0050926	GO:0050926	regulation of positive chemotaxis	4/385	27/23328	0.000949087	0.00655	0.00394	Cxcl12/Cc	4
GO:0060713	GO:0060713	labyrinthine layer morphogenesis	4/385	27/23328	0.000949087	0.00655	0.00394	Socs3/Vc	4
GO:1900120	GO:1900120	regulation of receptor binding	4/385	27/23328	0.000949087	0.00655	0.00394	Lox/B2m/	4
GO:1905809	GO:1905809	negative regulation of synapse organization	4/385	27/23328	0.000949087	0.00655	0.00394	Prnp/Arhg	4
GO:0042176	GO:0042176	regulation of protein catabolic process	16/385	395/23328	0.000953051	0.00657	0.00395	Nupr1/Tim	16
GO:1903555	GO:1903555	regulation of tumor necrosis factor superfamily cytokin	8/385	122/23328	0.000961535	0.00662	0.00398	Cd36/Cd3	8
GO:0006801	GO:0006801	superoxide metabolic process	6/385	69/23328	0.000970533	0.00666	0.004	Cd36/Fbln	6
GO:0045428	GO:0045428	regulation of nitric oxide biosynthetic process	6/385	69/23328	0.000970533	0.00666	0.004	Cd36/Cd3	6
GO:0050863	GO:0050863	regulation of T cell activation	14/385	321/23328	0.000971995	0.00666	0.004	Dtx1/Fgl2	14
GO:0055088	GO:0055088	lipid homeostasis	9/385	153/23328	0.001024671	0.00701	0.00421	Fabp4/Gp	9
GO:0001990	GO:0001990	regulation of systemic arterial blood pressure by horm	5/385	47/23328	0.001035495	0.00706	0.00424	Ece1/Gja	5
GO:0060135	GO:0060135	maternal process involved in female pregnancy	5/385	47/23328	0.001035495	0.00706	0.00424	Ptgis/Junb	5
GO:1990868	GO:1990868	response to chemokine	6/385	70/23328	0.001046999	0.00711	0.00427	Cxcl12/Cc	6
GO:1990869	GO:1990869	cellular response to chemokine	6/385	70/23328	0.001046999	0.00711	0.00427	Cxcl12/Cc	6
GO:0010460	GO:0010460	positive regulation of heart rate	4/385	28/23328	0.001092977	0.00738	0.00443	Rgs4/Adm	4
GO:0045992	GO:0045992	negative regulation of embryonic development	4/385	28/23328	0.001092977	0.00738	0.00443	Col5a2/Nr	4
GO:0060037	GO:0060037	pharyngeal system development	4/385	28/23328	0.001092977	0.00738	0.00443	Adgrf5/Tb	4
GO:2000178	GO:2000178	negative regulation of neural precursor cell proliferatio	4/385	28/23328	0.001092977	0.00738	0.00443	Btg2/Cd24	4
GO:0002697	GO:0002697	regulation of immune effector process	17/385	440/23328	0.001120338	0.00752	0.00452	Cd36/Fgl2	17
GO:0031953	GO:0031953	negative regulation of protein autophosphorylation	3/385	13/23328	0.001127977	0.00752	0.00452	Eng/Cav1	3
GO:0032060	GO:0032060	bleb assembly	3/385	13/23328	0.001127977	0.00752	0.00452	Pmp22/My	3
GO:0043589	GO:0043589	skin morphogenesis	3/385	13/23328	0.001127977	0.00752	0.00452	Itga6/Col1	3

GO:0060670	GO:0060670	branching involved in labyrinthine layer morphogenesis	3/385	13/23328	0.001127977	0.00752	0.00452	Socs3/Spi	3
GO:0072110	GO:0072110	glomerular mesangial cell proliferation	3/385	13/23328	0.001127977	0.00752	0.00452	Pdgfb/Egr	3
GO:0072216	GO:0072216	positive regulation of metanephros development	3/385	13/23328	0.001127977	0.00752	0.00452	Pdgfb/Egr	3
GO:0043407	GO:0043407	negative regulation of MAP kinase activity	6/385	71/23328	0.001128005	0.00752	0.00452	Sfrp1/Dus	6
GO:0002573	GO:0002573	myeloid leukocyte differentiation	11/385	220/23328	0.001132669	0.00753	0.00452	Sfrp1/Rbp	11
GO:0010660	GO:0010660	regulation of muscle cell apoptotic process	7/385	97/23328	0.001135921	0.00753	0.00452	Fndc1/Nu	7
GO:0120162	GO:0120162	positive regulation of cold-induced thermogenesis	7/385	97/23328	0.001135921	0.00753	0.00452	Fabp4/Cd	7
GO:0003382	GO:0003382	epithelial cell morphogenesis	5/385	48/23328	0.001140458	0.00753	0.00452	Col15a1/Ie	5
GO:0010883	GO:0010883	regulation of lipid storage	5/385	48/23328	0.001140458	0.00753	0.00452	Cd36/Sca	5
GO:1903727	GO:1903727	positive regulation of phospholipid metabolic process	5/385	48/23328	0.001140458	0.00753	0.00452	Flt1/Adgrf	5
GO:0032675	GO:0032675	regulation of interleukin-6 production	9/385	156/23328	0.001174529	0.00774	0.00465	Cd36/Cd2	9
GO:0008064	GO:0008064	regulation of actin polymerization or depolymerization	10/385	188/23328	0.001184252	0.00779	0.00468	Tmsb4x/E	10
GO:0032640	GO:0032640	tumor necrosis factor production	8/385	126/23328	0.001184977	0.00779	0.00468	Cd36/Cd3	8
GO:0030279	GO:0030279	negative regulation of ossification	7/385	98/23328	0.001206293	0.0079	0.00475	Id1/Sfrp1/	7
GO:0032652	GO:0032652	regulation of interleukin-1 production	7/385	98/23328	0.001206293	0.0079	0.00475	Hspb1/Mn	7
GO:0061136	GO:0061136	regulation of proteasomal protein catabolic process	10/385	189/23328	0.001232499	0.00806	0.00484	Nupr1/Plk	10
GO:0051346	GO:0051346	negative regulation of hydrolase activity	16/385	405/23328	0.001235586	0.00807	0.00485	Timp4/Aqp	16
GO:0003416	GO:0003416	endochondral bone growth	4/385	29/23328	0.001251497	0.00814	0.00489	Kdr/Thbs1	4
GO:0007157	GO:0007157	heterophilic cell-cell adhesion via plasma membrane c	5/385	49/23328	0.001253094	0.00814	0.00489	Selp/Mcar	5
GO:0090207	GO:0090207	regulation of triglyceride metabolic process	5/385	49/23328	0.001253094	0.00814	0.00489	Cd36/Sca	5
GO:0006959	GO:0006959	humoral immune response	16/385	406/23328	0.001267376	0.00822	0.00494	Rgcc/Cxcl	16
GO:0045185	GO:0045185	maintenance of protein location	7/385	99/23328	0.001280035	0.00829	0.00498	Tmsb4x/S	7
GO:0071706	GO:0071706	tumor necrosis factor superfamily cytokine production	8/385	128/23328	0.001311244	0.00848	0.0051	Cd36/Cd3	8
GO:0030832	GO:0030832	regulation of actin filament length	10/385	191/23328	0.001333806	0.00861	0.00518	Tmsb4x/E	10
GO:1990845	GO:1990845	adaptive thermogenesis	9/385	159/23328	0.001341617	0.00865	0.0052	Fabp4/Cd	9
GO:0042116	GO:0042116	macrophage activation	7/385	100/23328	0.001357256	0.00873	0.00524	Adgrf5/Cd	7
GO:0046889	GO:0046889	positive regulation of lipid biosynthetic process	7/385	100/23328	0.001357256	0.00873	0.00524	Scarb1/Ac	7
GO:0006690	GO:0006690	icosanoid metabolic process	8/385	129/23328	0.001378269	0.00883	0.00531	Mgll/Fabp	8
GO:0061008	GO:0061008	hepaticobiliary system development	8/385	129/23328	0.001378269	0.00883	0.00531	Sox17/Ptn	8
GO:0015672	GO:0015672	monovalent inorganic cation transport	18/385	489/23328	0.001393755	0.00892	0.00536	Rgs4/Abc	18
GO:0014820	GO:0014820	tonic smooth muscle contraction	3/385	14/23328	0.001418116	0.00898	0.0054	Cd38/Mylk	3
GO:0035791	GO:0035791	platelet-derived growth factor receptor-beta signaling p	3/385	14/23328	0.001418116	0.00898	0.0054	Lox/Pdgfb	3
GO:0070365	GO:0070365	hepatocyte differentiation	3/385	14/23328	0.001418116	0.00898	0.0054	Bmp4/Pro	3
GO:0072224	GO:0072224	metanephric glomerulus development	3/385	14/23328	0.001418116	0.00898	0.0054	Pdgfb/Aqp	3
GO:2001279	GO:2001279	regulation of unsaturated fatty acid biosynthetic proces	3/385	14/23328	0.001418116	0.00898	0.0054	Fabp5/Cd	3
GO:0010874	GO:0010874	regulation of cholesterol efflux	4/385	30/23328	0.001425418	0.00898	0.0054	Pltp/Cav1	4
GO:0030947	GO:0030947	regulation of vascular endothelial growth factor recepto	4/385	30/23328	0.001425418	0.00898	0.0054	Kdr/Tmem	4
GO:0051085	GO:0051085	chaperone cofactor-dependent protein refolding	4/385	30/23328	0.001425418	0.00898	0.0054	H2-DMb1/	4
GO:0071711	GO:0071711	basement membrane organization	4/385	30/23328	0.001425418	0.00898	0.0054	Cav1/Spin	4
GO:1903579	GO:1903579	negative regulation of ATP metabolic process	4/385	30/23328	0.001425418	0.00898	0.0054	Nupr1/Ier3	4
GO:2000060	GO:2000060	positive regulation of ubiquitin-dependent protein catab	7/385	101/23328	0.00143807	0.00905	0.00544	Plk2/Clu/Il	7
GO:0006694	GO:0006694	steroid biosynthetic process	9/385	161/23328	0.001463277	0.00918	0.00552	Igfbp7/Sca	9
GO:0030178	GO:0030178	negative regulation of Wnt signaling pathway	9/385	161/23328	0.001463277	0.00918	0.00552	Tmem88/S	9
GO:0046394	GO:0046394	carboxylic acid biosynthetic process	13/385	299/23328	0.001499551	0.00939	0.00565	Mgll/Lpl/F	13
GO:0048640	GO:0048640	negative regulation of developmental growth	8/385	131/23328	0.001520477	0.00951	0.00571	Rgs4/Sfrp	8
GO:0010657	GO:0010657	muscle cell apoptotic process	7/385	102/23328	0.00152259	0.00951	0.00571	Fndc1/Nu	7
GO:0016053	GO:0016053	organic acid biosynthetic process	13/385	300/23328	0.001544677	0.00963	0.00579	Mgll/Lpl/F	13
GO:0019933	GO:0019933	cAMP-mediated signaling	10/385	195/23328	0.001556778	0.0097	0.00583	Aplnr/Pde	10
GO:0001655	GO:0001655	urogenital system development	15/385	376/23328	0.001579476	0.00982	0.0059	Cfh/Sfrp1/	15
GO:0000079	GO:0000079	regulation of cyclin-dependent protein serine/threonine	6/385	76/23328	0.001607406	0.00996	0.00598	Rgcc/Cdk	6
GO:0015748	GO:0015748	organophosphate ester transport	7/385	103/23328	0.001610931	0.00996	0.00598	Scarb1/Kc	7
GO:0072676	GO:0072676	lymphocyte migration	7/385	103/23328	0.001610931	0.00996	0.00598	Cxcl12/Cc	7
GO:0002026	GO:0002026	regulation of the force of heart contraction	4/385	31/23328	0.001615502	0.00996	0.00598	Cav1/Myl4	4
GO:0002063	GO:0002063	chondrocyte development	4/385	31/23328	0.001615502	0.00996	0.00598	Pthlh/Com	4
GO:0033687	GO:0033687	osteoblast proliferation	4/385	31/23328	0.001615502	0.00996	0.00598	Sfrp1/Fbln	4
GO:1901214	GO:1901214	regulation of neuron death	15/385	377/23328	0.001620836	0.00998	0.00599	Btg2/Prnp	15
GO:1904036	GO:1904036	negative regulation of epithelial cell apoptotic process	5/385	52/23328	0.001640428	0.01008	0.00606	Nupr1/Cdl	5
GO:0009991	GO:0009991	response to extracellular stimulus	15/385	378/23328	0.001663098	0.01021	0.00613	Atf3/Fstl1/	15
GO:0009952	GO:0009952	anterior/posterior pattern specification	11/385	231/23328	0.0016718	0.01023	0.00615	Tcf15/Sfrp	11
GO:2001234	GO:2001234	negative regulation of apoptotic signaling pathway	11/385	231/23328	0.0016718	0.01023	0.00615	Cxcl12/Hs	11
GO:1901615	GO:1901615	organic hydroxy compound metabolic process	18/385	498/23328	0.001702864	0.01035	0.00622	Adh7/Reln	18
GO:0046849	GO:0046849	bone remodeling	7/385	104/23328	0.001703209	0.01035	0.00622	Sfrp1/Cd3	7
GO:0048525	GO:0048525	negative regulation of viral process	7/385	104/23328	0.001703209	0.01035	0.00622	Rsad2/Zfp	7
GO:0050886	GO:0050886	endocrine process	7/385	104/23328	0.001703209	0.01035	0.00622	Aqp1/Ece	7
GO:0060349	GO:0060349	bone morphogenesis	7/385	104/23328	0.001703209	0.01035	0.00622	Pthlh/Col1	7
GO:0003002	GO:0003002	regionalization	15/385	379/23328	0.001706278	0.01035	0.00622	Tcf15/Reln	15
GO:0061097	GO:0061097	regulation of protein tyrosine kinase activity	6/385	77/23328	0.001719449	0.01041	0.00626	Reln/Thy1	6
GO:0019730	GO:0019730	antimicrobial humoral response	9/385	165/23328	0.001733193	0.01048	0.0063	Cxcl12/Nt	9
GO:0030111	GO:0030111	regulation of Wnt signaling pathway	13/385	304/23328	0.001736602	0.01048	0.0063	Tmem88/S	13
GO:0030307	GO:0030307	positive regulation of cell growth	10/385	198/23328	0.001743113	0.01048	0.0063	Cxcl12/Sf	10
GO:0010885	GO:0010885	regulation of cholesterol storage	3/385	15/23328	0.001751065	0.01048	0.0063	Cd36/Sca	3
GO:0019755	GO:0019755	one-carbon compound transport	3/385	15/23328	0.001751065	0.01048	0.0063	Car4/Aqp1	3
GO:0031115	GO:0031115	negative regulation of microtubule polymerization	3/385	15/23328	0.001751065	0.01048	0.0063	Cdh5/Stm	3
GO:0043129	GO:0043129	surfactant homeostasis	3/385	15/23328	0.001751065	0.01048	0.0063	Adgrf5/Kd	3
GO:2000095	GO:2000095	regulation of Wnt signaling pathway, planar cell polarit	3/385	15/23328	0.001751065	0.01048	0.0063	Sfrp1/Dab	3
GO:0045995	GO:0045995	regulation of embryonic development	8/385	134/23328	0.001755344	0.01048	0.0063	Sfrp1/Col5	8
GO:0055076	GO:0055076	transition metal ion homeostasis	8/385	134/23328	0.001755344	0.01048	0.0063	Cp/Steap4	8
GO:0010596	GO:0010596	negative regulation of endothelial cell migration	5/385	53/23328	0.001787156	0.01056	0.00635	Rgcc/Nr2f	5
GO:0031113	GO:0031113	regulation of microtubule polymerization	5/385	53/23328	0.001787156	0.01056	0.00635	Cdh5/Gda	5
GO:0032371	GO:0032371	regulation of sterol transport	5/385	53/23328	0.001787156	0.01056	0.00635	Pltp/Anxa	5
GO:0032374	GO:0032374	regulation of cholesterol transport	5/385	53/23328	0.001787156	0.01056	0.00635	Pltp/Anxa	5
GO:0050435	GO:0050435	amyloid-beta metabolic process	5/385	53/23328	0.001787156	0.01056	0.00635	Spon1/Prn	5
GO:0055010	GO:0055010	ventricular cardiac muscle tissue morphogenesis	5/385	53/23328	0.001787156	0.01056	0.00635	Ly6e/Eng/	5
GO:0061383	GO:0061383	trabecula morphogenesis	5/385	53/23328	0.001787156	0.01056	0.00635	Sfrp1/S1p	5
GO:0010043	GO:0010043	response to zinc ion	4/385	32/23328	0.001822511	0.01073	0.00645	Pam/Mt1/I	4
GO:0051150	GO:0051150	regulation of smooth muscle cell differentiation	4/385	32/23328	0.001822511	0.01073	0.00645	Tmsb4x/P	4
GO:2000352	GO:2000352	negative regulation of endothelial cell apoptotic proces	4/385	32/23328	0.001822511	0.01073	0.00645	Cdh5/Kdr/	4
GO:0046785	GO:0046785	microtubule polymerization	6/385	78/23328	0.00183732	0.01077	0.00647	Cdh5/Gda	6
GO:0050810	GO:0050810	regulation of steroid biosynthetic process	6/385	78/23328	0.00183732	0.01077	0.00647	Igfbp7/Dh	6
GO:0070373	GO:0070373	negative regulation of ERK1 and ERK2 cascade	6/385	78/23328	0.00183732	0.01077	0.00647	Atf3/Dusp	6
GO:2000116	GO:2000116	regulation of cysteine-type endopeptidase activity	11/385	234/23328	0.001850552	0.01083	0.00651	Perp/Lgm	11
GO:0051099	GO:0051099	positive regulation of binding	10/385	200/23328	0.00187707	0.01096	0.00658	Spon1/Plk	10
GO:0099173	GO:0099173	postsynapse organization	10/385	200/23328	0.00187707	0.01096	0.00658	Reln/Gap4	10
GO:0032635	GO:0032635	interleukin-6 production	9/385	167/23328	0.001882351	0.01096	0.00658	Cd36/Cd2	9
GO:2001242	GO:2001242	regulation of intrinsic apoptotic signaling pathway	9/385	167/23328	0.001882351	0.01096	0.00658	Cxcl12/Nu	9
GO:0031532	GO:0031532	actin cytoskeleton reorganization	7/385	106/23328	0.001900053	0.01103	0.00663	Esam/Arh	7
GO:0034446	GO:0034446	substrate adhesion-dependent cell spreading	7/385	106/23328	0.001900053	0.01103	0.00663	Sfrp1/Fn1	7
GO:0009896	GO:0009896	positive regulation of catabolic process	17/385	463/23328	0.001924014	0.01115	0.0067	Nupr1/Btg	17
GO:0051056	GO:0051056	regulation of small GTPase mediated signal transducti	12/385	271/23328	0.001933409	0.01119	0.00672	Reln/Mcf2	12
GO:0014009	GO:0014009	glial cell proliferation	5/385	54/23328	0.001943272	0.0112	0.00673	Vim/Clu/P	5
GO:0042743	GO:0042743	hydrogen peroxide metabolic process	5/385	54/23328	0.001943272	0.0112	0.00673	Duoxa2/C	5
GO:1900271	GO:1900271	regulation of long-term synaptic potentiation	5/385	54/23328	0.001943272	0.0112	0.00673	Reln/Prnp	5
GO:0008406	GO:0008406	gonad development	11/385	236/23328	0.001978152	0.01139	0.00684	Sfrp1/Nup	11
GO:0008202	GO:0008202	steroid metabolic process	13/385	309/23328	0.00200385	0.01151	0.00692	Igfbp7/Sca	13
GO:1903426	GO:1903426	regulation of reactive oxygen species biosynthetic proc	7/385	107/23328	0.002004859	0.01151	0.00692	Cd36/Duo	7
GO:1904029	GO:1904029	regulation of cyclin-dependent protein kinase activity	6/385	80/23328	0.002091317	0.01186	0.00713	Rgcc/Cdk	6
GO:0002042	GO:0002042	cell migration involved in sprouting angiogenesis	5/385	55/23328	0.002109124	0.01186	0.00713	Kdr/Plk2/T	5
GO:0002720	GO:0002720	positive regulation of cytokine production involved in ir	5/385	55/23328	0.002109124	0.01186	0.00713	Cd36/Rsa	5
GO:0010665	GO:0010665	regulation of cardiac muscle cell apoptotic process	5/385	55/23328	0.002109124	0.01186	0.00713	Fndc1/Nu	5
GO:0006639	GO:0006639	acylglycerol metabolic process	8/385	138/23328	0.00211209	0.01186	0.00713	Cd36/Mgll	8
GO:0030218	GO:0030218	erythrocyte differentiation	8/385	138/23328	0.00211209	0.01186	0.00713	Adgrf5/Lm	8
GO:0006641	GO:0006641	triglyceride metabolic process	7/385	108/23328	0.002114083	0.01186	0.00713	Cd36/Sca	7
GO:0032091	GO:0032091	negative regulation of protein binding	7/385	108/23328	0.002114083	0.01186	0.00713	Id1/Pdgfb/	7
GO:0002830	GO:0002830	positive regulation of type 2 immune response	3/385	16/23328	0.002128942	0.01186	0.00713	Rsad2/Il33	3
GO:0015669	GO:0015669	gas transport	3/385	16/23328	0.002128942	0.01186	0.00713	Car4/Aqp1	3
GO:0019886	GO:0019886	antigen processing and presentation of exogenous pep	3/385	16/23328	0.002128942	0.01186	0.00713	H2-DMb1/	3
GO:0045820	GO:0045820	negative regulation of glycolytic process	3/385	16/23328	0.002128942	0.01186	0.00713	Nupr1/Ier3	3
GO:0060100	GO:0060100	positive regulation of phagocytosis, engulfment	3/385	16/23328	0.002128942	0.01186	0.00713	Cd36/C3/I	3
GO:0060856	GO:0060856	establishment of blood-brain barrier	3/385	16/23328	0.002128942	0.01186	0.00713	Eng/Lsr/A	3
GO:0070486	GO:0070486	leukocyte aggregation	3/385	16/23328	0.002128942	0.01186	0.00713	Jam2/S10	3
GO:0072109	GO:0072109	glomerular mesangium development	3/385	16/23328	0.002128942	0.01186	0.00713	Pdgfb/Egr	3
GO:0072537	GO:0072537	fibroblast activation	3/385	16/23328	0.002128942	0.01186	0.00713	Rgcc/Pdg	3
GO:0090177	GO:0090177	establishment of planar polarity involved in neural tube	3/385	16/23328	0.002128942	0.01186	0.00713	Sfrp1/Cthr	3
GO:1901722	GO:1901722	regulation of cell proliferation involved in kidney devel	3/385	16/23328	0.002128942	0.01186	0.00713	Pdgfb/Egr	3
GO:1905155	GO:1905155	positive regulation of membrane invagination	3/385	16/23328	0.002128942	0.01186	0.00713	Cd36/C3/I	3
GO:2000647	GO:2000647	negative regulation of stem cell proliferation	3/385	16/23328	0.002128942	0.01186	0.00713	Cd24a/Ptn	3
GO:2001212	GO:2001212	regulation of vasculogenesis	3/385	16/23328	0.002128942	0.01186	0.00713	Kdr/Cd34/	3
GO:0045927	GO:0045927	positive regulation of growth	13/385	312/23328	0.002179869	0.01211	0.00728	Cxcl12/Sf	13
GO:0045861	GO:0045861	negative regulation of proteolysis	14/385	350/23328	0.002181664	0.01211	0.00728	Timp4/Aqp	14

GO:0060560	GO:0060560	developmental growth involved in morphogenesis	12/385	275/23328	0.002182515	0.01211	0.00728	Mgll/Cxcl1	12
GO:0030833	GO:0030833	regulation of actin filament polymerization	9/385	171/23328	0.002211411	0.01226	0.00737	Tmsb4x/E	9
GO:0001959	GO:0001959	regulation of cytokine-mediated signaling pathway	7/385	109/23328	0.00222785	0.0123	0.00739	Irf7/Cd24a	7
GO:0002062	GO:0002062	chondrocyte differentiation	7/385	109/23328	0.00222785	0.0123	0.00739	Cytl1/Pthl	7
GO:0051897	GO:0051897	positive regulation of protein kinase B signaling	7/385	109/23328	0.00222785	0.0123	0.00739	Igfbp5/En	7
GO:0009306	GO:0009306	protein secretion	17/385	470/23328	0.002248492	0.0124	0.00745	Rgcc/Cd3	17
GO:0045137	GO:0045137	development of primary sexual characteristics	11/385	240/23328	0.002254851	0.01242	0.00746	Sfrp1/Nup	11
GO:0001523	GO:0001523	retinoid metabolic process	5/385	56/23328	0.002285064	0.01248	0.0075	Adh7/Rbp	5
GO:0001954	GO:0001954	positive regulation of cell-matrix adhesion	5/385	56/23328	0.002285064	0.01248	0.0075	Cd36/Thy	5
GO:0051930	GO:0051930	regulation of sensory perception of pain	5/385	56/23328	0.002285064	0.01248	0.0075	Mgll/Fabp	5
GO:1902893	GO:1902893	regulation of pri-miRNA transcription by RNA polymera	5/385	56/23328	0.002285064	0.01248	0.0075	Pdgfb/Fos	5
GO:0019884	GO:0019884	antigen processing and presentation of exogenous ant	4/385	34/23328	0.002290314	0.01248	0.0075	B2m/H2-D	4
GO:0032689	GO:0032689	negative regulation of interferon-gamma production	4/385	34/23328	0.002290314	0.01248	0.0075	Prnp/Il33/	4
GO:0071634	GO:0071634	regulation of transforming growth factor beta productio	4/385	34/23328	0.002290314	0.01248	0.0075	Cd200/Cd	4
GO:1904646	GO:1904646	cellular response to amyloid-beta	4/385	34/23328	0.002290314	0.01248	0.0075	Cd36/Prnp	4
GO:0035592	GO:0035592	establishment of protein localization to extracellular reg	17/385	471/23328	0.002298384	0.01251	0.00752	Rgcc/Cd3	17
GO:0006638	GO:0006638	neutral lipid metabolic process	8/385	140/23328	0.00231063	0.01256	0.00755	Cd36/Mgll	8
GO:0050807	GO:0050807	regulation of synapse organization	12/385	277/23328	0.002316571	0.01257	0.00756	Sparcl1/R	12
GO:0046777	GO:0046777	protein autophosphorylation	11/385	241/23328	0.002328715	0.01262	0.00759	Flt1/Thy1/	11
GO:0032231	GO:0032231	regulation of actin filament bundle assembly	7/385	110/23328	0.002346284	0.01269	0.00762	Rgcc/Id1/	7
GO:2000106	GO:2000106	regulation of leukocyte apoptotic process	7/385	110/23328	0.002346284	0.01269	0.00762	Cxcl12/Kit	7
GO:0015914	GO:0015914	phospholipid transport	6/385	82/23328	0.002370961	0.0128	0.00769	Scarb1/Kc	6
GO:0035966	GO:0035966	response to topologically incorrect protein	8/385	141/23328	0.002415238	0.01303	0.00783	Atf3/Hspb	8
GO:0008630	GO:0008630	intrinsic apoptotic signaling pathway in response to DN	7/385	111/23328	0.002469512	0.01324	0.00796	Cxcl12/Nu	7
GO:0019218	GO:0019218	regulation of steroid metabolic process	7/385	111/23328	0.002469512	0.01324	0.00796	Igfbp7/Dh	7
GO:0010463	GO:0010463	mesenchymal cell proliferation	5/385	57/23328	0.002471444	0.01324	0.00796	Tbx1/Kdr/	5
GO:0043030	GO:0043030	regulation of macrophage activation	5/385	57/23328	0.002471444	0.01324	0.00796	Adgrf5/Cd	5
GO:0051931	GO:0051931	regulation of sensory perception	5/385	57/23328	0.002471444	0.01324	0.00796	Mgll/Fabp	5
GO:0015718	GO:0015718	monocarboxylic acid transport	9/385	174/23328	0.002487019	0.01331	0.008	Fabp4/Cd	9
GO:0045844	GO:0045844	positive regulation of striated muscle tissue developme	6/385	83/23328	0.002520896	0.01338	0.00804	Tbx1/Ncar	6
GO:0048636	GO:0048636	positive regulation of muscle organ development	6/385	83/23328	0.002520896	0.01338	0.00804	Tbx1/Ncar	6
GO:0006458	GO:0006458	'de novo' protein folding	4/385	35/23328	0.002552594	0.01338	0.00804	H2-DMb1/	4
GO:0034110	GO:0034110	regulation of homotypic cell-cell adhesion	4/385	35/23328	0.002552594	0.01338	0.00804	Cd9/Pdpn	4
GO:0051084	GO:0051084	'de novo' posttranslational protein folding	4/385	35/23328	0.002552594	0.01338	0.00804	H2-DMb1/	4
GO:0055090	GO:0055090	acylglycerol homeostasis	4/385	35/23328	0.002552594	0.01338	0.00804	Gpihbp1/S	4
GO:0070328	GO:0070328	triglyceride homeostasis	4/385	35/23328	0.002552594	0.01338	0.00804	Gpihbp1/S	4
GO:1902003	GO:1902003	regulation of amyloid-beta formation	4/385	35/23328	0.002552594	0.01338	0.00804	Spon1/Prn	4
GO:0001921	GO:0001921	positive regulation of receptor recycling	3/385	17/23328	0.002553724	0.01338	0.00804	Ece1/Anx	3
GO:0003184	GO:0003184	pulmonary valve morphogenesis	3/385	17/23328	0.002553724	0.01338	0.00804	Bmp4/Gja	3
GO:0010878	GO:0010878	cholesterol storage	3/385	17/23328	0.002553724	0.01338	0.00804	Cd36/Sca	3
GO:0010934	GO:0010934	macrophage cytokine production	3/385	17/23328	0.002553724	0.01338	0.00804	Cd36/Gas	3
GO:0033194	GO:0033194	response to hydroperoxide	3/385	17/23328	0.002553724	0.01338	0.00804	Cd36/Cd3	3
GO:0048875	GO:0048875	chemical homeostasis within a tissue	3/385	17/23328	0.002553724	0.01338	0.00804	Adgrf5/Kd	3
GO:0060850	GO:0060850	regulation of transcription involved in cell fate commitm	3/385	17/23328	0.002553724	0.01338	0.00804	Sox17/Nr	3
GO:0071636	GO:0071636	positive regulation of transforming growth factor beta p	3/385	17/23328	0.002553724	0.01338	0.00804	Cd200/Cd	3
GO:0090192	GO:0090192	regulation of glomerulus development	3/385	17/23328	0.002553724	0.01338	0.00804	Pdgfb/Egr	3
GO:0001822	GO:0001822	kidney development	13/385	318/23328	0.002570162	0.01345	0.00808	Cfh/Sfrp1/	13
GO:0007612	GO:0007612	learning	9/385	175/23328	0.002584711	0.0135	0.00811	Reln/Nts/E	9
GO:0031331	GO:0031331	positive regulation of cellular catabolic process	15/385	396/23328	0.002596376	0.01352	0.00812	Nupr1/Btg	15
GO:0030038	GO:0030038	contractile actin filament bundle assembly	7/385	112/23328	0.00259766	0.01352	0.00812	Rgcc/Cgn	7
GO:0043149	GO:0043149	stress fiber assembly	7/385	112/23328	0.00259766	0.01352	0.00812	Rgcc/Cgn	7
GO:0001755	GO:0001755	neural crest cell migration	5/385	58/23328	0.002668616	0.01377	0.00827	Tbx1/Kitl/	5
GO:0010656	GO:0010656	negative regulation of muscle cell apoptotic process	5/385	58/23328	0.002668616	0.01377	0.00827	Nupr1/Slc	5
GO:0010662	GO:0010662	regulation of striated muscle cell apoptotic process	5/385	58/23328	0.002668616	0.01377	0.00827	Fndc1/Nu	5
GO:0016101	GO:0016101	diterpenoid metabolic process	5/385	58/23328	0.002668616	0.01377	0.00827	Adh7/Rbp	5
GO:0035690	GO:0035690	cellular response to drug	5/385	58/23328	0.002668616	0.01377	0.00827	Prnp/Gstn	5
GO:0061614	GO:0061614	pri-miRNA transcription by RNA polymerase II	5/385	58/23328	0.002668616	0.01377	0.00827	Pdgfb/Fos	5
GO:0071692	GO:0071692	protein localization to extracellular region	17/385	478/23328	0.002674209	0.01377	0.00827	Rgcc/Cd3	17
GO:0014032	GO:0014032	neural crest cell development	6/385	84/23328	0.002677841	0.01377	0.00827	Tbx1/Kitl/	6
GO:0051384	GO:0051384	response to glucocorticoid	6/385	84/23328	0.002677841	0.01377	0.00827	Aqp1/Zfp3	6
GO:1901863	GO:1901863	positive regulation of muscle tissue development	6/385	84/23328	0.002677841	0.01377	0.00827	Tbx1/Ncar	6
GO:0030217	GO:0030217	T cell differentiation	12/385	282/23328	0.002681335	0.01377	0.00827	Dtx1/Fgl2/	12
GO:0007369	GO:0007369	gastrulation	9/385	176/23328	0.002685419	0.01377	0.00828	Aplnr/Sfrp	9
GO:0043583	GO:0043583	ear development	11/385	246/23328	0.002728028	0.01397	0.0084	Igfbp7/Tcf	11
GO:0001837	GO:0001837	epithelial to mesenchymal transition	8/385	144/23328	0.002751467	0.01404	0.00844	Rgcc/Sfrp	8
GO:0106106	GO:0106106	cold-induced thermogenesis	8/385	144/23328	0.002751467	0.01404	0.00844	Fabp4/Cd	8
GO:0120161	GO:0120161	regulation of cold-induced thermogenesis	8/385	144/23328	0.002751467	0.01404	0.00844	Fabp4/Cd	8
GO:0046486	GO:0046486	glycerolipid metabolic process	14/385	360/23328	0.002815299	0.01435	0.00862	Cd36/Mgll	14
GO:0003401	GO:0003401	axis elongation	4/385	36/23328	0.002834771	0.01435	0.00862	Sfrp1/Tnc	4
GO:0035886	GO:0035886	vascular associated smooth muscle cell differentiation	4/385	36/23328	0.002834771	0.01435	0.00862	Pdgfb/Eng	4
GO:0070306	GO:0070306	lens fiber cell differentiation	4/385	36/23328	0.002834771	0.01435	0.00862	Vim/Cdkn	4
GO:0071604	GO:0071604	transforming growth factor beta production	4/385	36/23328	0.002834771	0.01435	0.00862	Cd200/Cd	4
GO:0097242	GO:0097242	amyloid-beta clearance	4/385	36/23328	0.002834771	0.01435	0.00862	Cd36/Clu/	4
GO:1904707	GO:1904707	positive regulation of vascular associated smooth mus	4/385	36/23328	0.002834771	0.01435	0.00862	Igfbp5/Pd	4
GO:0014910	GO:0014910	regulation of smooth muscle cell migration	6/385	85/23328	0.002841999	0.01436	0.00863	Tmsb4x/Id	6
GO:2000379	GO:2000379	positive regulation of reactive oxygen species metabol	7/385	114/23328	0.002869232	0.01448	0.0087	Cd36/Duo	7
GO:0006633	GO:0006633	fatty acid biosynthetic process	8/385	145/23328	0.002871306	0.01448	0.0087	Mgll/Lpl/F	8
GO:0010659	GO:0010659	cardiac muscle cell apoptotic process	5/385	59/23328	0.002876936	0.01449	0.00871	Fndc1/Nu	5
GO:0002819	GO:0002819	regulation of adaptive immune response	10/385	213/23328	0.00296411	0.01491	0.00896	Irf7/Rsad2	10
GO:0009615	GO:0009615	response to virus	12/385	286/23328	0.00300565	0.01494	0.00898	Fgl2/Irf7/F	12
GO:0048259	GO:0048259	regulation of receptor-mediated endocytosis	7/385	115/23328	0.003012917	0.01494	0.00898	Clu/B2m/	7
GO:0090100	GO:0090100	positive regulation of transmembrane receptor protein	7/385	115/23328	0.003012917	0.01494	0.00898	Cdh5/Kdr/	7
GO:0060411	GO:0060411	cardiac septum morphogenesis	6/385	86/23328	0.003013572	0.01494	0.00898	Tbx1/Eng	6
GO:1901379	GO:1901379	regulation of potassium ion transmembrane transport	6/385	86/23328	0.003013572	0.01494	0.00898	Rgs4/Prnp	6
GO:0021542	GO:0021542	dentate gyrus development	3/385	18/23328	0.003027256	0.01494	0.00898	Reln/Btg2	3
GO:0033631	GO:0033631	cell-cell adhesion mediated by integrin	3/385	18/23328	0.003027256	0.01494	0.00898	Plpp3/Cd2	3
GO:0042026	GO:0042026	protein refolding	3/385	18/23328	0.003027256	0.01494	0.00898	B2m/Hspa	3
GO:0042249	GO:0042249	establishment of planar polarity of embryonic epitheliu	3/385	18/23328	0.003027256	0.01494	0.00898	Sfrp1/Cth	3
GO:0045019	GO:0045019	negative regulation of nitric oxide biosynthetic process	3/385	18/23328	0.003027256	0.01494	0.00898	Cd34/Ptgi	3
GO:0045603	GO:0045603	positive regulation of endothelial cell differentiation	3/385	18/23328	0.003027256	0.01494	0.00898	Cdh5/Cldr	3
GO:0046184	GO:0046184	aldehyde biosynthetic process	3/385	18/23328	0.003027256	0.01494	0.00898	Adh1/Aldo	3
GO:0051000	GO:0051000	positive regulation of nitric-oxide synthase activity	3/385	18/23328	0.003027256	0.01494	0.00898	Scarb1/Ap	3
GO:0072148	GO:0072148	epithelial cell fate commitment	3/385	18/23328	0.003027256	0.01494	0.00898	Pdpn/Nr2	3
GO:1901881	GO:1901881	positive regulation of protein depolymerization	3/385	18/23328	0.003027256	0.01494	0.00898	Dstn/Nes/	3
GO:1904406	GO:1904406	negative regulation of nitric oxide metabolic process	3/385	18/23328	0.003027256	0.01494	0.00898	Cd34/Ptgi	3
GO:0010524	GO:0010524	positive regulation of calcium ion transport into cytosol	5/385	60/23328	0.003096757	0.01521	0.00914	Aplnr/Thy	5
GO:0060711	GO:0060711	labyrinthine layer development	5/385	60/23328	0.003096757	0.01521	0.00914	Socs3/Vc	5
GO:0072678	GO:0072678	T cell migration	5/385	60/23328	0.003096757	0.01521	0.00914	Cxcl12/Cc	5
GO:2000401	GO:2000401	regulation of lymphocyte migration	5/385	60/23328	0.003096757	0.01521	0.00914	Cxcl12/Cc	5
GO:1901861	GO:1901861	regulation of muscle tissue development	9/385	180/23328	0.003119733	0.01529	0.00919	Rgs4/Tbx	9
GO:0045667	GO:0045667	regulation of osteoblast differentiation	8/385	147/23328	0.003123168	0.01529	0.00919	Id1/Sfrp1/	8
GO:0002691	GO:0002691	regulation of cellular extravasation	4/385	37/23328	0.003137565	0.01529	0.00919	Cxcl12/Th	4
GO:0002920	GO:0002920	regulation of humoral immune response	4/385	37/23328	0.003137565	0.01529	0.00919	Cfh/Serpi	4
GO:0051482	GO:0051482	positive regulation of cytosolic calcium ion concentratio	4/385	37/23328	0.003137565	0.01529	0.00919	Tgm2/S1p	4
GO:0060071	GO:0060071	Wnt signaling pathway, planar cell polarity pathway	4/385	37/23328	0.003137565	0.01529	0.00919	Sfrp1/Dab	4
GO:1902229	GO:1902229	regulation of intrinsic apoptotic signaling pathway in re	4/385	37/23328	0.003137565	0.01529	0.00919	Cxcl12/Cl	4
GO:0070663	GO:0070663	regulation of leukocyte proliferation	11/385	251/23328	0.003180865	0.01548	0.0093	Prnp/Cd3	11
GO:0001659	GO:0001659	temperature homeostasis	9/385	181/23328	0.003236514	0.01573	0.00945	Fabp4/Cd	9
GO:0050803	GO:0050803	regulation of synapse structure or activity	12/385	289/23328	0.003269186	0.01587	0.00954	Sparcl1/R	12
GO:0045444	GO:0045444	fat cell differentiation	11/385	252/23328	0.003278293	0.0159	0.00955	Fabp4/Ste	11
GO:0006986	GO:0006986	response to unfolded protein	7/385	117/23328	0.003316741	0.01607	0.00965	Atf3/Hspb	7
GO:0010812	GO:0010812	negative regulation of cell-substrate adhesion	5/385	61/23328	0.003328434	0.0161	0.00968	Col1a1/Th	5
GO:0048705	GO:0048705	skeletal system morphogenesis	11/385	253/23328	0.003378102	0.01628	0.00978	Tcf15/Sfrp	11
GO:0031960	GO:0031960	response to corticosteroid	6/385	88/23328	0.003379786	0.01628	0.00978	Aqp1/Zfp3	6
GO:0032526	GO:0032526	response to retinoic acid	6/385	88/23328	0.003379786	0.01628	0.00978	Tbx1/Adh	6
GO:0046683	GO:0046683	response to organophosphorus	6/385	88/23328	0.003379786	0.01628	0.00978	Abcc9/Pd	6
GO:0031668	GO:0031668	cellular response to extracellular stimulus	10/385	217/23328	0.003384364	0.01628	0.00978	Atf3/Lpl/It	10
GO:0002831	GO:0002831	regulation of response to biotic stimulus	12/385	291/23328	0.003455036	0.0165	0.00991	Fgl2/Irf7/H	12
GO:0001706	GO:0001706	endoderm formation	4/385	38/23328	0.003461686	0.0165	0.00991	Fn1/Col5a	4
GO:0019432	GO:0019432	triglyceride biosynthetic process	4/385	38/23328	0.003461686	0.0165	0.00991	Scarb1/Lp	4
GO:0043267	GO:0043267	negative regulation of potassium ion transport	4/385	38/23328	0.003461686	0.0165	0.00991	Rgs4/Cav	4
GO:0045601	GO:0045601	regulation of endothelial cell differentiation	4/385	38/23328	0.003461686	0.0165	0.00991	Id1/Cdh5/	4
GO:0046627	GO:0046627	negative regulation of insulin receptor signaling pathwa	4/385	38/23328	0.003461686	0.0165	0.00991	Socs3/Ptp	4
GO:0072210	GO:0072210	metanephric nephron development	4/385	38/23328	0.003461686	0.0165	0.00991	Pdgfb/Aqp	4
GO:2000249	GO:2000249	regulation of actin cytoskeleton reorganization	4/385	38/23328	0.003461686	0.0165	0.00991	Esam/Arh	4
GO:1903362	GO:1903362	regulation of cellular protein catabolic process	11/385	254/23328	0.003480333	0.01657	0.00996	Nupr1/Plk	11
GO:0043903	GO:0043903	regulation of symbiotic process	10/385	218/23328	0.003496492	0.01663	0.00999	Rsad2/Zfp	10
GO:0042391	GO:0042391	regulation of membrane potential	16/385	450/23328	0.003534456	0.01675	0.01007	Cd36/Reln	16
GO:0016486	GO:0016486	peptide hormone processing	3/385	19/23328	0.003551248	0.01675	0.01007	Cpe/Ece1	3

GO:0030502	GO:0030502	negative regulation of bone mineralization	3/385	19/23328	0.003551248	0.01675	0.01007	Srgn/Ecm	3
GO:0034111	GO:0034111	negative regulation of homotypic cell-cell adhesion	3/385	19/23328	0.003551248	0.01675	0.01007	Cd9/Cd24	3
GO:0035994	GO:0035994	response to muscle stretch	3/385	19/23328	0.003551248	0.01675	0.01007	Fos/Jun/N	3
GO:0042730	GO:0042730	fibrinolysis	3/385	19/23328	0.003551248	0.01675	0.01007	Anxa2/Se	3
GO:0060099	GO:0060099	regulation of phagocytosis, engulfment	3/385	19/23328	0.003551248	0.01675	0.01007	Cd36/C3/I	3
GO:0003229	GO:0003229	ventricular cardiac muscle tissue development	5/385	62/23328	0.003572318	0.01675	0.01007	Ly6e/Eng/	5
GO:0010658	GO:0010658	striated muscle cell apoptotic process	5/385	62/23328	0.003572318	0.01675	0.01007	Fndc1/Nu	5
GO:0046850	GO:0046850	regulation of bone remodeling	5/385	62/23328	0.003572318	0.01675	0.01007	Sfrp1/Cd3	5
GO:0060350	GO:0060350	endochondral bone morphogenesis	5/385	62/23328	0.003572318	0.01675	0.01007	Pthlh/Col1	5
GO:1905207	GO:1905207	regulation of cardiocyte differentiation	5/385	62/23328	0.003572318	0.01675	0.01007	Rgs4/Sox	5
GO:0014031	GO:0014031	mesenchymal cell development	6/385	89/23328	0.003574838	0.01675	0.01007	Tbx1/Kitl/S	6
GO:0001952	GO:0001952	regulation of cell-matrix adhesion	7/385	119/23328	0.003643393	0.01704	0.01024	Cd36/Thy	7
GO:0060759	GO:0060759	regulation of response to cytokine stimulus	7/385	119/23328	0.003643393	0.01704	0.01024	Irf7/Cd24a	7
GO:0048593	GO:0048593	camera-type eye morphogenesis	8/385	151/23328	0.003678204	0.01716	0.01031	Flt1/Thy1/	8
GO:0072006	GO:0072006	nephron development	8/385	151/23328	0.003678204	0.01716	0.01031	Cfh/Notch	8
GO:0007044	GO:0007044	cell-substrate junction assembly	6/385	90/23328	0.00377813	0.01757	0.01056	Thy1/Fn1/	6
GO:0048864	GO:0048864	stem cell development	6/385	90/23328	0.00377813	0.01757	0.01056	Tbx1/Kitl/S	6
GO:1900076	GO:1900076	regulation of cellular response to insulin stimulus	6/385	90/23328	0.00377813	0.01757	0.01056	C1qtnf9/S	6
GO:0072001	GO:0072001	renal system development	13/385	333/23328	0.003801213	0.01763	0.01059	Cfh/Sfrp1/	13
GO:0030513	GO:0030513	positive regulation of BMP signaling pathway	4/385	39/23328	0.003807832	0.01763	0.01059	Cdh5/Kdr/	4
GO:0050715	GO:0050715	positive regulation of cytokine secretion	4/385	39/23328	0.003807832	0.01763	0.01059	Rgcc/Cd3	4
GO:0090049	GO:0090049	regulation of cell migration involved in sprouting angio	4/385	39/23328	0.003807832	0.01763	0.01059	Kdr/Plk2/T	4
GO:0090288	GO:0090288	negative regulation of cellular response to growth facto	8/385	152/23328	0.003828198	0.01765	0.0106	Sfrp1/Htra	8
GO:0006721	GO:0006721	terpenoid metabolic process	5/385	63/23328	0.003828765	0.01765	0.0106	Adh7/Rbp	5
GO:0010830	GO:0010830	regulation of myotube differentiation	5/385	63/23328	0.003828765	0.01765	0.0106	Cxcl12/Tb	5
GO:0071260	GO:0071260	cellular response to mechanical stimulus	5/385	63/23328	0.003828765	0.01765	0.0106	Aqp1/Col1	5
GO:1903050	GO:1903050	regulation of proteolysis involved in cellular protein cat	10/385	221/23328	0.003850671	0.01772	0.01065	Nupr1/Plk	10
GO:0097193	GO:0097193	intrinsic apoptotic signaling pathway	12/385	295/23328	0.003852333	0.01772	0.01065	Cxcl12/Pe	12
GO:0032611	GO:0032611	interleukin-1 beta production	6/385	91/23328	0.00398987	0.01833	0.01101	Cd36/Hsp	6
GO:0030041	GO:0030041	actin filament polymerization	9/385	187/23328	0.004011334	0.01839	0.01105	Tmsb4x/E	9
GO:0034767	GO:0034767	positive regulation of ion transmembrane transport	9/385	187/23328	0.004011334	0.01839	0.01105	Aplnr/Relr	9
GO:0008347	GO:0008347	glial cell migration	5/385	64/23328	0.004098125	0.01873	0.01125	Reln/Cd9/	5
GO:0050707	GO:0050707	regulation of cytokine secretion	5/385	64/23328	0.004098125	0.01873	0.01125	Rgcc/Cd3	5
GO:0007163	GO:0007163	establishment or maintenance of cell polarity	10/385	223/23328	0.00410216	0.01873	0.01125	Tcf15/Cdh	10
GO:0070997	GO:0070997	neuron death	15/385	416/23328	0.004103799	0.01873	0.01125	Btg2/Prnp	15
GO:0001946	GO:0001946	lymphangiogenesis	3/385	20/23328	0.004127289	0.01877	0.01128	Pdpn/Prox	3
GO:0140448	GO:0140448	signaling receptor ligand precursor processing	3/385	20/23328	0.004127289	0.01877	0.01128	Cpe/Ece1	3
GO:1905153	GO:1905153	regulation of membrane invagination	3/385	20/23328	0.004127289	0.01877	0.01128	Cd36/C3/I	3
GO:0008360	GO:0008360	regulation of cell shape	8/385	154/23328	0.004142332	0.0188	0.0113	Fn1/Kdr/P	8
GO:1901568	GO:1901568	fatty acid derivative metabolic process	8/385	154/23328	0.004142332	0.0188	0.0113	Mgll/Fabp	8
GO:0007616	GO:0007616	long-term memory	4/385	40/23328	0.004176688	0.01886	0.01133	Reln/Egr1	4
GO:0070570	GO:0070570	regulation of neuron projection regeneration	4/385	40/23328	0.004176688	0.01886	0.01133	Thy1/Ptn/I	4
GO:0090175	GO:0090175	regulation of establishment of planar polarity	4/385	40/23328	0.004176688	0.01886	0.01133	Sfrp1/Dab	4
GO:1903959	GO:1903959	regulation of anion transmembrane transport	4/385	40/23328	0.004176688	0.01886	0.01133	Rgs4/Prnp	4
GO:1904645	GO:1904645	response to amyloid-beta	4/385	40/23328	0.004176688	0.01886	0.01133	Cd36/Prnp	4
GO:0051261	GO:0051261	protein depolymerization	7/385	123/23328	0.004369497	0.01971	0.01184	Dstn/Nes/	7
GO:0034121	GO:0034121	regulation of toll-like receptor signaling pathway	5/385	65/23328	0.004380749	0.01971	0.01185	Cd36/Irf7/	5
GO:0034308	GO:0034308	primary alcohol metabolic process	5/385	65/23328	0.004380749	0.01971	0.01185	Adh7/Rbp	5
GO:0002028	GO:0002028	regulation of sodium ion transport	6/385	93/23328	0.004439527	0.01991	0.01197	Fxyd6/Ser	6
GO:0071774	GO:0071774	response to fibroblast growth factor	6/385	93/23328	0.004439527	0.01991	0.01197	Sfrp1/Tbx	6
GO:0150115	GO:0150115	cell-substrate junction organization	6/385	93/23328	0.004439527	0.01991	0.01197	Thy1/Fn1/	6
GO:0006937	GO:0006937	regulation of muscle contraction	8/385	156/23328	0.004475969	0.02006	0.01205	Cav1/Calc	8
GO:0002686	GO:0002686	negative regulation of leukocyte migration	4/385	41/23328	0.004568928	0.02038	0.01225	Cxcl12/Cc	4
GO:0034205	GO:0034205	amyloid-beta formation	4/385	41/23328	0.004568928	0.02038	0.01225	Spon1/Prr	4
GO:0046164	GO:0046164	alcohol catabolic process	4/385	41/23328	0.004568928	0.02038	0.01225	Adh7/Sca	4
GO:1902991	GO:1902991	regulation of amyloid precursor protein catabolic proce	4/385	41/23328	0.004568928	0.02038	0.01225	Spon1/Prr	4
GO:0042982	GO:0042982	amyloid precursor protein metabolic process	5/385	66/23328	0.004676986	0.02082	0.01251	Spon1/Prr	5
GO:0090559	GO:0090559	regulation of membrane permeability	5/385	66/23328	0.004676986	0.02082	0.01251	Slc25a4/C	5
GO:0002495	GO:0002495	antigen processing and presentation of peptide antiger	3/385	21/23328	0.004756845	0.02107	0.01266	H2-DMb1/	3
GO:0003177	GO:0003177	pulmonary valve development	3/385	21/23328	0.004756845	0.02107	0.01266	Bmp4/Gja	3
GO:0043117	GO:0043117	positive regulation of vascular permeability	3/385	21/23328	0.004756845	0.02107	0.01266	Pde3a/Ap	3
GO:0090050	GO:0090050	positive regulation of cell migration involved in sproutir	3/385	21/23328	0.004756845	0.02107	0.01266	Kdr/Plk2/A	3
GO:0043087	GO:0043087	regulation of GTPase activity	12/385	303/23328	0.004757136	0.02107	0.01266	Rgs4/Thy	12
GO:0051341	GO:0051341	regulation of oxidoreductase activity	6/385	95/23328	0.004925481	0.02174	0.01307	Scarb1/Er	6
GO:0051492	GO:0051492	regulation of stress fiber assembly	6/385	95/23328	0.004925481	0.02174	0.01307	Rgcc/Cgn	6
GO:0055013	GO:0055013	cardiac muscle cell development	6/385	95/23328	0.004925481	0.02174	0.01307	Rgs4/Slc2	6
GO:0070661	GO:0070661	leukocyte proliferation	13/385	344/23328	0.004977828	0.02185	0.01313	Cxcl12/Pr	13
GO:0001889	GO:0001889	liver development	7/385	126/23328	0.004982088	0.02185	0.01313	Ptn/Lsr/Ju	7
GO:0030048	GO:0030048	actin filament-based movement	7/385	126/23328	0.004982088	0.02185	0.01313	Pdpn/Cav	7
GO:1901216	GO:1901216	positive regulation of neuron death	7/385	126/23328	0.004982088	0.02185	0.01313	Prnp/Clu/I	7
GO:0001709	GO:0001709	cell fate determination	4/385	42/23328	0.004985211	0.02185	0.01313	Sox17/Bm	4
GO:0006509	GO:0006509	membrane protein ectodomain proteolysis	4/385	42/23328	0.004985211	0.02185	0.01313	Timp4/Tim	4
GO:0030239	GO:0030239	myofibril assembly	5/385	67/23328	0.004987184	0.02185	0.01313	Csrp2/Itgb	5
GO:0045580	GO:0045580	regulation of T cell differentiation	8/385	159/23328	0.005014695	0.02195	0.01319	Dtx1/Fgl2/	8
GO:0014902	GO:0014902	myotube differentiation	7/385	127/23328	0.005199978	0.02272	0.01365	Cxcl12/Cc	7
GO:0060048	GO:0060048	cardiac muscle contraction	7/385	127/23328	0.005199978	0.02272	0.01365	Cav1/Myl4	7
GO:2001244	GO:2001244	positive regulation of intrinsic apoptotic signaling pathw	5/385	68/23328	0.005311688	0.02318	0.01393	Nupr1/Clu	5
GO:0003279	GO:0003279	cardiac septum development	7/385	128/23328	0.005424946	0.02357	0.01416	Tbx1/Eng/	7
GO:0051592	GO:0051592	response to calcium ion	7/385	128/23328	0.005424946	0.02357	0.01416	Lgmn/Fos	7
GO:0043491	GO:0043491	protein kinase B signaling	9/385	196/23328	0.005437309	0.02357	0.01416	Kdr/Igfbp5	9
GO:0072111	GO:0072111	cell proliferation involved in kidney development	3/385	22/23328	0.005441265	0.02357	0.01416	Pdgfb/Egr	3
GO:1900543	GO:1900543	negative regulation of purine nucleotide metabolic proc	3/385	22/23328	0.005441265	0.02357	0.01416	Nupr1/Ier3	3
GO:1901741	GO:1901741	positive regulation of myoblast fusion	3/385	22/23328	0.005441265	0.02357	0.01416	Cxcl12/Cx	3
GO:1903055	GO:1903055	positive regulation of extracellular matrix organization	3/385	22/23328	0.005441265	0.02357	0.01416	Rgcc/Pdp	3
GO:0002027	GO:0002027	regulation of heart rate	6/385	97/23328	0.005449406	0.02358	0.01417	Rgs4/Cav	6
GO:0022898	GO:0022898	regulation of transmembrane transporter activity	11/385	270/23328	0.005480666	0.02367	0.01422	Reln/Fxyd	11
GO:0043523	GO:0043523	regulation of neuron apoptotic process	11/385	270/23328	0.005480666	0.02367	0.01422	Btg2/Prnp	11
GO:0016055	GO:0016055	Wnt signaling pathway	15/385	430/23328	0.005535053	0.02388	0.01435	Tmem88/S	15
GO:0016032	GO:0016032	viral process	12/385	309/23328	0.005541282	0.02388	0.01435	Rsad2/Vc	12
GO:0044706	GO:0044706	multi-multicellular organism process	9/385	197/23328	0.005617223	0.02419	0.01453	Igfbp5/Ptg	9
GO:0031638	GO:0031638	zymogen activation	5/385	69/23328	0.005650841	0.02426	0.01457	Perp/Lgm	5
GO:0032677	GO:0032677	regulation of interleukin-8 production	5/385	69/23328	0.005650841	0.02426	0.01457	Cd74/F2r/	5
GO:0045600	GO:0045600	positive regulation of fat cell differentiation	5/385	69/23328	0.005650841	0.02426	0.01457	Sfrp1/Lpl/	5
GO:0061844	GO:0061844	antimicrobial humoral immune response mediated by a	7/385	129/23328	0.005657132	0.02426	0.01458	Cxcl12/Nt	7
GO:0060395	GO:0060395	SMAD protein signal transduction	6/385	98/23328	0.005726131	0.02453	0.01474	Vim/Fos/J	6
GO:0198738	GO:0198738	cell-cell signaling by wnt	15/385	432/23328	0.005768984	0.02469	0.01483	Tmem88/S	15
GO:0006941	GO:0006941	striated muscle contraction	8/385	163/23328	0.005808578	0.02481	0.01491	Cav1/Aldo	8
GO:1903169	GO:1903169	regulation of calcium ion transmembrane transport	8/385	163/23328	0.005808578	0.02481	0.01491	Aplnr/Thy	8
GO:0045823	GO:0045823	positive regulation of heart contraction	4/385	44/23328	0.005892465	0.0251	0.01508	Rgs4/Adm	4
GO:1903672	GO:1903672	positive regulation of sprouting angiogenesis	4/385	44/23328	0.005892465	0.0251	0.01508	Aplnr/Kdr/	4
GO:0031334	GO:0031334	positive regulation of protein-containing complex asse	10/385	235/23328	0.005895726	0.0251	0.01508	Cd36/Cdh	10
GO:0150076	GO:0150076	neuroinflammatory response	5/385	70/23328	0.006004983	0.02551	0.01533	Nupr1/Cd	5
GO:2000107	GO:2000107	negative regulation of leukocyte apoptotic process	5/385	70/23328	0.006004983	0.02551	0.01533	Cxcl12/Kit	5
GO:0043502	GO:0043502	regulation of muscle adaptation	6/385	99/23328	0.006012976	0.02552	0.01534	Rgs4/Igfb	6
GO:0045017	GO:0045017	glycerolipid biosynthetic process	8/385	164/23328	0.006021211	0.02553	0.01534	Scarb1/Lp	8
GO:0032434	GO:0032434	regulation of proteasomal ubiquitin-dependent protein	7/385	131/23328	0.00614372	0.02597	0.01561	Plk2/Clu/I	7
GO:0010743	GO:0010743	regulation of macrophage derived foam cell differentiat	3/385	23/23328	0.006181787	0.02597	0.01561	Cd36/Lpl/	3
GO:0010866	GO:0010866	regulation of triglyceride biosynthetic process	3/385	23/23328	0.006181787	0.02597	0.01561	Scarb1/Th	3
GO:0031579	GO:0031579	membrane raft organization	3/385	23/23328	0.006181787	0.02597	0.01561	Anxa2/Ca	3
GO:0032288	GO:0032288	myelin assembly	3/385	23/23328	0.006181787	0.02597	0.01561	Cd9/Pmp2	3
GO:0034383	GO:0034383	low-density lipoprotein particle clearance	3/385	23/23328	0.006181787	0.02597	0.01561	Cd36/Sca	3
GO:0036303	GO:0036303	lymph vessel morphogenesis	3/385	23/23328	0.006181787	0.02597	0.01561	Pdpn/Prox	3
GO:0045980	GO:0045980	negative regulation of nucleotide metabolic process	3/385	23/23328	0.006181787	0.02597	0.01561	Nupr1/Ier3	3
GO:0051043	GO:0051043	regulation of membrane protein ectodomain proteolysis	3/385	23/23328	0.006181787	0.02597	0.01561	Timp4/Tim	3
GO:0007389	GO:0007389	pattern specification process	16/385	478/23328	0.00624414	0.02619	0.01574	Tcf15/Rel	16
GO:0048863	GO:0048863	stem cell differentiation	10/385	237/23328	0.00624636	0.02619	0.01574	Sfrp1/Tbx	10
GO:0050792	GO:0050792	regulation of viral process	9/385	201/23328	0.006383306	0.02664	0.01601	Rsad2/Zfp	9
GO:0050918	GO:0050918	positive chemotaxis	4/385	45/23328	0.00638468	0.02664	0.01601	Cxcl12/Cc	4
GO:0060421	GO:0060421	positive regulation of heart growth	4/385	45/23328	0.00638468	0.02664	0.01601	Ncam1/Sl	4
GO:0090184	GO:0090184	positive regulation of kidney development	4/385	45/23328	0.00638468	0.02664	0.01601	Pdgfb/Egr	4
GO:1902743	GO:1902743	regulation of lamellipodium organization	4/385	45/23328	0.00638468	0.02664	0.01601	Pdpn/Aqp	4
GO:0099175	GO:0099175	regulation of postsynapse organization	7/385	132/23328	0.006398403	0.02667	0.01603	Reln/Gap4	7
GO:0007565	GO:0007565	female pregnancy	8/385	166/23328	0.006464173	0.02692	0.01617	Igfbp5/Ptg	8
GO:0051607	GO:0051607	defense response to virus	10/385	239/23328	0.006613016	0.02745	0.01649	Fgl2/Irf7/F	10
GO:0032755	GO:0032755	positive regulation of interleukin-6 production	6/385	101/23328	0.00661786	0.02745	0.01649	Cd36/Lpl/	6
GO:0055006	GO:0055006	cardiac cell development	6/385	101/23328	0.00661786	0.02745	0.01649	Rgs4/Slc2	6
GO:0071456	GO:0071456	cellular response to hypoxia	6/385	101/23328	0.00661786	0.02745	0.01649	Rgcc/Fnd	6
GO:0051100	GO:0051100	negative regulation of binding	8/385	167/23328	0.006694704	0.02774	0.01667	Id1/Pdgfb/	8
GO:0010633	GO:0010633	negative regulation of epithelial cell migration	5/385	72/23328	0.006759579	0.02798	0.01681	Rgcc/Nr2f	5

GO:0046578	GO:0046578	regulation of Ras protein signal transduction	10/385	240/23328	0.006802501	0.02813	0.0169	Mcf2l/Kitl/	10
GO:0043618	GO:0043618	regulation of transcription from RNA polymerase II pro	4/385	46/23328	0.006903424	0.02846	0.0171	Cd36/Atf3	4
GO:0061098	GO:0061098	positive regulation of protein tyrosine kinase activity	4/385	46/23328	0.006903424	0.02846	0.0171	Reln/Prnp	4
GO:2000404	GO:2000404	regulation of T cell migration	4/385	46/23328	0.006903424	0.02846	0.0171	Cxcl12/Cc	4
GO:0001704	GO:0001704	formation of primary germ layer	6/385	102/23328	0.006936314	0.02857	0.01717	Fn1/Col5a	6
GO:0007205	GO:0007205	protein kinase C-activating G protein-coupled receptor	3/385	24/23328	0.006979542	0.02861	0.01719	Htr2b/F2r/	3
GO:0031639	GO:0031639	plasminogen activation	3/385	24/23328	0.006979542	0.02861	0.01719	Serpine2/	3
GO:0045736	GO:0045736	negative regulation of cyclin-dependent protein serine/	3/385	24/23328	0.006979542	0.02861	0.01719	Cdkn1c/N	3
GO:0070841	GO:0070841	inclusion body assembly	3/385	24/23328	0.006979542	0.02861	0.01719	Clu/Apoe/	3
GO:0090330	GO:0090330	regulation of platelet aggregation	3/385	24/23328	0.006979542	0.02861	0.01719	Cd9/Pdpn	3
GO:0032409	GO:0032409	regulation of transporter activity	11/385	280/23328	0.00713213	0.02921	0.01755	Reln/Fxyd	11
GO:0030177	GO:0030177	positive regulation of Wnt signaling pathway	7/385	135/23328	0.007209693	0.02949	0.01772	Sfrp1/Nra	7
GO:0071216	GO:0071216	cellular response to biotic stimulus	12/385	320/23328	0.007243311	0.0296	0.01779	Cd36/Vim	12
GO:0010522	GO:0010522	regulation of calcium ion transport into cytosol	6/385	103/23328	0.007265719	0.02964	0.01781	Aplnr/Thy	6
GO:0090263	GO:0090263	positive regulation of canonical Wnt signaling pathway	6/385	103/23328	0.007265719	0.02964	0.01781	Sfrp1/Nra	6
GO:0034764	GO:0034764	positive regulation of transmembrane transport	10/385	243/23328	0.007396308	0.03014	0.01811	Aplnr/Reln	10
GO:0003197	GO:0003197	endocardial cushion development	4/385	47/23328	0.007449278	0.03024	0.01817	Eng/Thbs	4
GO:0045214	GO:0045214	sarcomere organization	4/385	47/23328	0.007449278	0.03024	0.01817	Csrp2/Itgb	4
GO:0046460	GO:0046460	neutral lipid biosynthetic process	4/385	47/23328	0.007449278	0.03024	0.01817	Scarb1/Lp	4
GO:0046463	GO:0046463	acylglycerol biosynthetic process	4/385	47/23328	0.007449278	0.03024	0.01817	Scarb1/Lp	4
GO:0055123	GO:0055123	digestive system development	7/385	136/23328	0.007496339	0.0304	0.01827	Sfrp1/Cdk	7
GO:0014074	GO:0014074	response to purine-containing compound	6/385	104/23328	0.007606281	0.03079	0.0185	Abcc9/Pd	6
GO:0046916	GO:0046916	cellular transition metal ion homeostasis	6/385	104/23328	0.007606281	0.03079	0.0185	Cp/Prnp/L	6
GO:0006066	GO:0006066	alcohol metabolic process	12/385	323/23328	0.007772386	0.03143	0.01888	Adh7/Sca	12
GO:0010165	GO:0010165	response to X-ray	3/385	25/23328	0.007835553	0.03144	0.01889	Sfrp1/Sfrp	3
GO:0032703	GO:0032703	negative regulation of interleukin-2 production	3/385	25/23328	0.007835553	0.03144	0.01889	Prnp/Cd3	3
GO:0032878	GO:0032878	regulation of establishment or maintenance of cell pola	3/385	25/23328	0.007835553	0.03144	0.01889	Cdh5/Tek	3
GO:0035024	GO:0035024	negative regulation of Rho protein signal transduction	3/385	25/23328	0.007835553	0.03144	0.01889	Rasip1/Itg	3
GO:0043567	GO:0043567	regulation of insulin-like growth factor receptor signalin	3/385	25/23328	0.007835553	0.03144	0.01889	Igfbp5/Igf	3
GO:0051894	GO:0051894	positive regulation of focal adhesion assembly	3/385	25/23328	0.007835553	0.03144	0.01889	Thy1/Kdr/	3
GO:1901739	GO:1901739	regulation of myoblast fusion	3/385	25/23328	0.007835553	0.03144	0.01889	Cxcl12/Cx	3
GO:1902993	GO:1902993	positive regulation of amyloid precursor protein catabo	3/385	25/23328	0.007835553	0.03144	0.01889	Spon1/Clu	3
GO:0014743	GO:0014743	regulation of muscle hypertrophy	5/385	75/23328	0.00801222	0.03203	0.01924	Rgs4/Igfb	5
GO:0032637	GO:0032637	interleukin-8 production	5/385	75/23328	0.00801222	0.03203	0.01924	Cd74/F2r/	5
GO:0051057	GO:0051057	positive regulation of small GTPase mediated signal tr	5/385	75/23328	0.00801222	0.03203	0.01924	Reln/Mcf2	5
GO:1903670	GO:1903670	regulation of sprouting angiogenesis	5/385	75/23328	0.00801222	0.03203	0.01924	Aplnr/Kdr/	5
GO:0007187	GO:0007187	G protein-coupled receptor signaling pathway, coupled	10/385	246/23328	0.008029415	0.03206	0.01927	Adgrl4/Pt	10
GO:0071222	GO:0071222	cellular response to lipopolysaccharide	11/385	285/23328	0.008091865	0.03226	0.01939	Cd36/Vim	11
GO:0071887	GO:0071887	leukocyte apoptotic process	7/385	138/23328	0.008094798	0.03226	0.01939	Cxcl12/Kit	7
GO:0000041	GO:0000041	transition metal ion transport	6/385	106/23328	0.008321694	0.0331	0.01989	Cp/Steap	6
GO:0035282	GO:0035282	segmentation	6/385	106/23328	0.008321694	0.0331	0.01989	Tcf15/Sfrp	6
GO:0008584	GO:0008584	male gonad development	7/385	139/23328	0.008406891	0.03335	0.02004	Sfrp1/Nup	7
GO:0010565	GO:0010565	regulation of cellular ketone metabolic process	7/385	139/23328	0.008406891	0.03335	0.02004	Fabp5/Ca	7
GO:0055007	GO:0055007	cardiac muscle cell differentiation	7/385	139/23328	0.008406891	0.03335	0.02004	Rgs4/Slc2	7
GO:0050663	GO:0050663	cytokine secretion	5/385	76/23328	0.008463263	0.03354	0.02015	Rgcc/Cd3	5
GO:1905953	GO:1905953	negative regulation of lipid localization	4/385	49/23328	0.008624559	0.03412	0.0205	Igfbp3/Th	4
GO:2000725	GO:2000725	regulation of cardiac muscle cell differentiation	4/385	49/23328	0.008624559	0.03412	0.0205	Rgs4/Slc2	4
GO:0002367	GO:0002367	cytokine production involved in immune response	6/385	107/23328	0.008696951	0.03432	0.02062	Cd36/Rsa	6
GO:0014897	GO:0014897	striated muscle hypertrophy	6/385	107/23328	0.008696951	0.03432	0.02062	Rgs4/Igfb	6
GO:0046546	GO:0046546	development of primary male sexual characteristics	7/385	140/23328	0.008727747	0.03432	0.02062	Sfrp1/Nup	7
GO:0071453	GO:0071453	cellular response to oxygen levels	7/385	140/23328	0.008727747	0.03432	0.02062	Rgcc/Fnd	7
GO:0050867	GO:0050867	positive regulation of cell activation	16/385	496/23328	0.008737249	0.03432	0.02062	Thy1/Selp	16
GO:0034104	GO:0034104	negative regulation of tissue remodeling	3/385	26/23328	0.008750748	0.03432	0.02062	Sfrp1/Cd3	3
GO:0034123	GO:0034123	positive regulation of toll-like receptor signaling pathw	3/385	26/23328	0.008750748	0.03432	0.02062	Rsad2/Ca	3
GO:1904030	GO:1904030	negative regulation of cyclin-dependent protein kinase	3/385	26/23328	0.008750748	0.03432	0.02062	Cdkn1c/N	3
GO:2000050	GO:2000050	regulation of non-canonical Wnt signaling pathway	3/385	26/23328	0.008750748	0.03432	0.02062	Sfrp1/Dab	3
GO:0071277	GO:0071277	cellular response to calcium ion	5/385	77/23328	0.008931583	0.035	0.02103	Lgmn/Fos	5
GO:0002224	GO:0002224	toll-like receptor signaling pathway	6/385	108/23328	0.009084179	0.03553	0.02135	Cd36/Irf7/	6
GO:0008306	GO:0008306	associative learning	6/385	108/23328	0.009084179	0.03553	0.02135	Reln/Nts/	6
GO:0008361	GO:0008361	regulation of cell size	9/385	213/23328	0.009168848	0.03582	0.02153	Mgll/C1qt	9
GO:1901215	GO:1901215	negative regulation of neuron death	10/385	251/23328	0.009176405	0.03582	0.02153	Btg2/Cd2	10
GO:0007520	GO:0007520	myoblast fusion	4/385	50/23328	0.009255065	0.03606	0.02167	Cxcl12/Cc	4
GO:0045646	GO:0045646	regulation of erythrocyte differentiation	4/385	50/23328	0.009255065	0.03606	0.02167	Lmo2/B2m	4
GO:0016202	GO:0016202	regulation of striated muscle tissue development	8/385	177/23328	0.009356137	0.03642	0.02189	Rgs4/Tbx	8
GO:0060976	GO:0060976	coronary vasculature development	5/385	78/23328	0.009417492	0.03663	0.02201	Aplnr/Tbx	5
GO:0002702	GO:0002702	positive regulation of production of molecular mediator	6/385	109/23328	0.009483577	0.03678	0.0221	Cd36/Rsa	6
GO:0014896	GO:0014896	muscle hypertrophy	6/385	109/23328	0.009483577	0.03678	0.0221	Rgs4/Igfb	6
GO:0051668	GO:0051668	localization within membrane	6/385	109/23328	0.009483577	0.03678	0.0221	Reln/Thy1	6
GO:0046034	GO:0046034	ATP metabolic process	10/385	253/23328	0.009668772	0.03721	0.02236	Cox4i2/Cf	10
GO:0002253	GO:0002253	activation of immune response	14/385	416/23328	0.009723418	0.03721	0.02236	Rgcc/Thy	14
GO:0010742	GO:0010742	macrophage derived foam cell differentiation	3/385	27/23328	0.009725956	0.03721	0.02236	Cd36/Lpl/	3
GO:0014829	GO:0014829	vascular associated smooth muscle contraction	3/385	27/23328	0.009725956	0.03721	0.02236	Cd38/Con	3
GO:0032770	GO:0032770	positive regulation of monooxygenase activity	3/385	27/23328	0.009725956	0.03721	0.02236	Scarb1/Ap	3
GO:0033688	GO:0033688	regulation of osteoblast proliferation	3/385	27/23328	0.009725956	0.03721	0.02236	Sfrp1/Fbl	3
GO:0042044	GO:0042044	fluid transport	3/385	27/23328	0.009725956	0.03721	0.02236	Aqp1/Aqp	3
GO:0045730	GO:0045730	respiratory burst	3/385	27/23328	0.009725956	0.03721	0.02236	Cd24a/Lb	3
GO:0051767	GO:0051767	nitric-oxide synthase biosynthetic process	3/385	27/23328	0.009725956	0.03721	0.02236	Kdr/Il33/E	3
GO:0051769	GO:0051769	regulation of nitric-oxide synthase biosynthetic proces	3/385	27/23328	0.009725956	0.03721	0.02236	Kdr/Il33/E	3
GO:0060343	GO:0060343	trabecula formation	3/385	27/23328	0.009725956	0.03721	0.02236	Sfrp1/Col	3
GO:0071425	GO:0071425	hematopoietic stem cell proliferation	3/385	27/23328	0.009725956	0.03721	0.02236	Cd34/Kitl/	3
GO:0090023	GO:0090023	positive regulation of neutrophil chemotaxis	3/385	27/23328	0.009725956	0.03721	0.02236	Cd74/Lbp	3
GO:0098581	GO:0098581	detection of external biotic stimulus	3/385	27/23328	0.009725956	0.03721	0.02236	Scarb1/Lb	3
GO:2000727	GO:2000727	positive regulation of cardiac muscle cell differentiatio	3/385	27/23328	0.009725956	0.03721	0.02236	Slc25a4/E	3
GO:0045089	GO:0045089	positive regulation of innate immune response	6/385	110/23328	0.009895343	0.03776	0.02269	Irf7/Mnda	6
GO:0042987	GO:0042987	amyloid precursor protein catabolic process	4/385	51/23328	0.009914837	0.03776	0.02269	Spon1/Prr	4
GO:0043620	GO:0043620	regulation of DNA-templated transcription in response	4/385	51/23328	0.009914837	0.03776	0.02269	Cd36/Atf3	4
GO:0051489	GO:0051489	regulation of filopodium assembly	4/385	51/23328	0.009914837	0.03776	0.02269	Gap43/My	4
GO:0060412	GO:0060412	ventricular septum morphogenesis	4/385	51/23328	0.009914837	0.03776	0.02269	Bmp4/Pro	4
GO:0043624	GO:0043624	cellular protein complex disassembly	7/385	144/23328	0.01010158	0.03843	0.02309	Dstn/Nes/	7
GO:0007219	GO:0007219	Notch signaling pathway	8/385	180/23328	0.01029011	0.03911	0.0235	Dtx1/Perp	8
GO:0071219	GO:0071219	cellular response to molecule of bacterial origin	11/385	295/23328	0.010310081	0.03915	0.02353	Cd36/Vim	11
GO:0001843	GO:0001843	neural tube closure	6/385	111/23328	0.010319677	0.03915	0.02353	Sfrp1/Spir	6
GO:0032651	GO:0032651	regulation of interleukin-1 beta production	5/385	80/23328	0.010443293	0.03959	0.02379	Hspb1/Mn	5
GO:0001974	GO:0001974	blood vessel remodeling	4/385	52/23328	0.010604372	0.03999	0.02403	Tbx1/Tgm	4
GO:0032731	GO:0032731	positive regulation of interleukin-1 beta production	4/385	52/23328	0.010604372	0.03999	0.02403	Hspb1/Mn	4
GO:0032757	GO:0032757	positive regulation of interleukin-8 production	4/385	52/23328	0.010604372	0.03999	0.02403	Cd74/F2r/	4
GO:0045124	GO:0045124	regulation of bone resorption	4/385	52/23328	0.010604372	0.03999	0.02403	Cd38/S1p	4
GO:0051353	GO:0051353	positive regulation of oxidoreductase activity	4/385	52/23328	0.010604372	0.03999	0.02403	Scarb1/Ap	4
GO:0045088	GO:0045088	regulation of innate immune response	8/385	181/23328	0.010616288	0.03999	0.02403	Irf7/Mnda	8
GO:0048634	GO:0048634	regulation of muscle organ development	8/385	181/23328	0.010616288	0.03999	0.02403	Rgs4/Tbx	8
GO:0060606	GO:0060606	tube closure	6/385	112/23328	0.010756772	0.04014	0.02412	Sfrp1/Spir	6
GO:0002693	GO:0002693	positive regulation of cellular extravasation	3/385	28/23328	0.010761913	0.04014	0.02412	Thy1/Selp	3
GO:0030224	GO:0030224	monocyte differentiation	3/385	28/23328	0.010761913	0.04014	0.02412	Jun/Cd74	3
GO:0032801	GO:0032801	receptor catabolic process	3/385	28/23328	0.010761913	0.04014	0.02412	Lgmn/Anx	3
GO:0035987	GO:0035987	endodermal cell differentiation	3/385	28/23328	0.010761913	0.04014	0.02412	Fn1/Col5a	3
GO:0042744	GO:0042744	hydrogen peroxide catabolic process	3/385	28/23328	0.010761913	0.04014	0.02412	Prdx1/Gp	3
GO:0045737	GO:0045737	positive regulation of cyclin-dependent protein serine/t	3/385	28/23328	0.010761913	0.04014	0.02412	Rgcc/Pdg	3
GO:0060445	GO:0060445	branching involved in salivary gland morphogenesis	3/385	28/23328	0.010761913	0.04014	0.02412	Pdgfb/Tgn	3
GO:0090025	GO:0090025	regulation of monocyte chemotaxis	3/385	28/23328	0.010761913	0.04014	0.02412	Cxcl12/Lg	3
GO:0090077	GO:0090077	foam cell differentiation	3/385	28/23328	0.010761913	0.04014	0.02412	Cd36/Lpl/	3
GO:1903131	GO:1903131	mononuclear cell differentiation	3/385	28/23328	0.010761913	0.04014	0.02412	Jun/Cd74	3
GO:0043524	GO:0043524	negative regulation of neuron apoptotic process	8/385	182/23328	0.010950062	0.04081	0.02452	Btg2/Kdr/	8
GO:0051402	GO:0051402	neuron apoptotic process	11/385	298/23328	0.011059087	0.04103	0.02466	Btg2/Prnp	11
GO:0006707	GO:0006707	cholesterol catabolic process	2/385	10/23328	0.011199788	0.04103	0.02466	Scarb1/Ap	2
GO:0016127	GO:0016127	sterol catabolic process	2/385	10/23328	0.011199788	0.04103	0.02466	Scarb1/Ap	2
GO:0036462	GO:0036462	TRAIL-activated apoptotic signaling pathway	2/385	10/23328	0.011199788	0.04103	0.02466	Atf3/Timp	2
GO:0048251	GO:0048251	elastic fiber assembly	2/385	10/23328	0.011199788	0.04103	0.02466	Fbln5/Lox	2
GO:0051001	GO:0051001	negative regulation of nitric-oxide synthase activity	2/385	10/23328	0.011199788	0.04103	0.02466	Eng/Cav1	2
GO:0060297	GO:0060297	regulation of sarcomere organization	2/385	10/23328	0.011199788	0.04103	0.02466	Prox1/Edr	2
GO:0060907	GO:0060907	positive regulation of macrophage cytokine production	2/385	10/23328	0.011199788	0.04103	0.02466	Cd36/Cd7	2
GO:0061517	GO:0061517	macrophage proliferation	2/385	10/23328	0.011199788	0.04103	0.02466	Clu/Il33	2
GO:0061626	GO:0061626	pharyngeal arch artery morphogenesis	2/385	10/23328	0.011199788	0.04103	0.02466	Adgrf5/Bm	2
GO:0070391	GO:0070391	response to lipoteichoic acid	2/385	10/23328	0.011199788	0.04103	0.02466	Cd36/Lbp	2
GO:0071223	GO:0071223	cellular response to lipoteichoic acid	2/385	10/23328	0.011199788	0.04103	0.02466	Cd36/Lbp	2
GO:0072203	GO:0072203	cell proliferation involved in metanephros developmen	2/385	10/23328	0.011199788	0.04103	0.02466	Pdgfb/Egr	2
GO:0090091	GO:0090091	positive regulation of extracellular matrix disassembly	2/385	10/23328	0.011199788	0.04103	0.02466	Pdpn/Fsc	2
GO:0099541	GO:0099541	trans-synaptic signaling by lipid	2/385	10/23328	0.011199788	0.04103	0.02466	Fabp5/F2	2
GO:0099542	GO:0099542	trans-synaptic signaling by endocannabinoid	2/385	10/23328	0.011199788	0.04103	0.02466	Fabp5/F2	2
GO:1900122	GO:1900122	positive regulation of receptor binding	2/385	10/23328	0.011199788	0.04103	0.02466	B2m/Anxa	2

GO:1901724	GO:1901724	positive regulation of cell proliferation involved in kidne	2/385	10/23328	0.011199788	0.04103	0.02466	Pdgfb/Egr	2
GO:2000489	GO:2000489	regulation of hepatic stellate cell activation	2/385	10/23328	0.011199788	0.04103	0.02466	Pdgfb/Cyg	2
GO:0050764	GO:0050764	regulation of phagocytosis	6/385	113/23328	0.011206823	0.04103	0.02466	Cd36/Sca	6
GO:0032412	GO:0032412	regulation of ion transmembrane transporter activity	10/385	259/23328	0.011267414	0.04121	0.02476	Reln/Fxyd	10
GO:0000082	GO:0000082	G1/S transition of mitotic cell cycle	8/385	183/23328	0.011291532	0.04121	0.02476	Rgcc/Plk2	8
GO:0001960	GO:0001960	negative regulation of cytokine-mediated signaling patl	4/385	53/23328	0.011324144	0.04121	0.02476	Cav1/Gas	4
GO:0030514	GO:0030514	negative regulation of BMP signaling pathway	4/385	53/23328	0.011324144	0.04121	0.02476	Sfrp1/Htra	4
GO:0030850	GO:0030850	prostate gland development	4/385	53/23328	0.011324144	0.04121	0.02476	Sfrp1/Tnc	4
GO:0032720	GO:0032720	negative regulation of tumor necrosis factor production	4/385	53/23328	0.011324144	0.04121	0.02476	Cd34/Cd2	4
GO:0051055	GO:0051055	negative regulation of lipid biosynthetic process	4/385	53/23328	0.011324144	0.04121	0.02476	Pdgfb/Apo	4
GO:1905330	GO:1905330	regulation of morphogenesis of an epithelium	6/385	114/23328	0.011670023	0.04241	0.02548	Sfrp1/Dab	6
GO:0022411	GO:0022411	cellular component disassembly	13/385	383/23328	0.011674336	0.04241	0.02548	Dstn/Nes/	13
GO:0043547	GO:0043547	positive regulation of GTPase activity	9/385	222/23328	0.011798781	0.04264	0.02562	Rgs4/Thy	9
GO:0001919	GO:0001919	regulation of receptor recycling	3/385	29/23328	0.011859269	0.04264	0.02562	Ece1/Anx	3
GO:0002029	GO:0002029	desensitization of G protein-coupled receptor signaling	3/385	29/23328	0.011859269	0.04264	0.02562	Aplnr/Adm	3
GO:0003148	GO:0003148	outflow tract septum morphogenesis	3/385	29/23328	0.011859269	0.04264	0.02562	Tbx1/Eng/	3
GO:0022401	GO:0022401	negative adaptation of signaling pathway	3/385	29/23328	0.011859269	0.04264	0.02562	Aplnr/Adm	3
GO:0030511	GO:0030511	positive regulation of transforming growth factor beta r	3/385	29/23328	0.011859269	0.04264	0.02562	Thbs1/Dal	3
GO:0043032	GO:0043032	positive regulation of macrophage activation	3/385	29/23328	0.011859269	0.04264	0.02562	Il33/Thbs1	3
GO:0061050	GO:0061050	regulation of cell growth involved in cardiac muscle cel	3/385	29/23328	0.011859269	0.04264	0.02562	Rgs4/Slc2	3
GO:0071353	GO:0071353	cellular response to interleukin-4	3/385	29/23328	0.011859269	0.04264	0.02562	Mrc1/Tuba	3
GO:0071624	GO:0071624	positive regulation of granulocyte chemotaxis	3/385	29/23328	0.011859269	0.04264	0.02562	Cd74/Lbp	3
GO:0150117	GO:0150117	positive regulation of cell-substrate junction organizatio	3/385	29/23328	0.011859269	0.04264	0.02562	Thy1/Kdr/	3
GO:1903846	GO:1903846	positive regulation of cellular response to transforming	3/385	29/23328	0.011859269	0.04264	0.02562	Thbs1/Dal	3
GO:0010498	GO:0010498	proteasomal protein catabolic process	15/385	470/23328	0.01194464	0.04291	0.02578	Nupr1/Plk	15
GO:0048546	GO:0048546	digestive tract morphogenesis	4/385	54/23328	0.012074614	0.04323	0.02597	Sfrp1/Sox	4
GO:0055025	GO:0055025	positive regulation of cardiac muscle tissue developme	4/385	54/23328	0.012074614	0.04323	0.02597	Ncam1/Sl	4
GO:1900024	GO:1900024	regulation of substrate adhesion-dependent cell spread	4/385	54/23328	0.012074614	0.04323	0.02597	Pdpn/Dab	4
GO:1903556	GO:1903556	negative regulation of tumor necrosis factor superfamil	4/385	54/23328	0.012074614	0.04323	0.02597	Cd34/Cd2	4
GO:0001738	GO:0001738	morphogenesis of a polarized epithelium	5/385	83/23328	0.012121402	0.04332	0.02603	Tcf15/Sfrp	5
GO:1904427	GO:1904427	positive regulation of calcium ion transmembrane trans	5/385	83/23328	0.012121402	0.04332	0.02603	Aplnr/Thy	5
GO:0008585	GO:0008585	female gonad development	6/385	115/23328	0.012146561	0.04337	0.02606	Sfrp1/Nup	6
GO:2000045	GO:2000045	regulation of G1/S transition of mitotic cell cycle	6/385	116/23328	0.012636625	0.04508	0.02709	Rgcc/Fhl1	6
GO:0006720	GO:0006720	isoprenoid metabolic process	5/385	84/23328	0.012719095	0.04526	0.0272	Adh7/Rbp	5
GO:0050766	GO:0050766	positive regulation of phagocytosis	5/385	84/23328	0.012719095	0.04526	0.0272	Cd36/Gas	5
GO:0051817	GO:0051817	modulation of process of other organism involved in sy	5/385	84/23328	0.012719095	0.04526	0.0272	Aqp1/Anx	5
GO:0033619	GO:0033619	membrane protein proteolysis	4/385	55/23328	0.012856221	0.04567	0.02744	Timp4/Tim	4
GO:0050891	GO:0050891	multicellular organismal water homeostasis	4/385	55/23328	0.012856221	0.04567	0.02744	Aqp1/Aqp	4
GO:0002825	GO:0002825	regulation of T-helper 1 type immune response	3/385	30/23328	0.013018585	0.04586	0.02756	Il33/H2-Ab	3
GO:0006929	GO:0006929	substrate-dependent cell migration	3/385	30/23328	0.013018585	0.04586	0.02756	Fn1/Pdgfb	3
GO:0010955	GO:0010955	negative regulation of protein processing	3/385	30/23328	0.013018585	0.04586	0.02756	Serpine2/	3
GO:0035162	GO:0035162	embryonic hemopoiesis	3/385	30/23328	0.013018585	0.04586	0.02756	Kdr/Lmo2	3
GO:0043552	GO:0043552	positive regulation of phosphatidylinositol 3-kinase acti	3/385	30/23328	0.013018585	0.04586	0.02756	Flt1/Pdgfb	3
GO:0043951	GO:0043951	negative regulation of cAMP-mediated signaling	3/385	30/23328	0.013018585	0.04586	0.02756	Aplnr/Pde	3
GO:0060143	GO:0060143	positive regulation of syncytium formation by plasma m	3/385	30/23328	0.013018585	0.04586	0.02756	Cxcl12/Cx	3
GO:0061082	GO:0061082	myeloid leukocyte cytokine production	3/385	30/23328	0.013018585	0.04586	0.02756	Cd36/Gas	3
GO:0120178	GO:0120178	steroid hormone biosynthetic process	3/385	30/23328	0.013018585	0.04586	0.02756	Scarb1/Eg	3
GO:1903318	GO:1903318	negative regulation of protein maturation	3/385	30/23328	0.013018585	0.04586	0.02756	Serpine2/	3
GO:0070665	GO:0070665	positive regulation of leukocyte proliferation	7/385	152/23328	0.013311802	0.04675	0.02809	Cd38/Kitl/	7
GO:0002718	GO:0002718	regulation of cytokine production involved in immune r	5/385	85/23328	0.013336414	0.04675	0.02809	Cd36/Rsa	5
GO:0030203	GO:0030203	glycosaminoglycan metabolic process	5/385	85/23328	0.013336414	0.04675	0.02809	Bgn/Pdgft	5
GO:0001765	GO:0001765	membrane raft assembly	2/385	11/23328	0.013540423	0.04675	0.02809	Anxa2/Ca	2
GO:0002138	GO:0002138	retinoic acid biosynthetic process	2/385	11/23328	0.013540423	0.04675	0.02809	Rbp1/Cyp	2
GO:0002579	GO:0002579	positive regulation of antigen processing and presenta	2/385	11/23328	0.013540423	0.04675	0.02809	H2-Ab1/C	2
GO:0016102	GO:0016102	diterpenoid biosynthetic process	2/385	11/23328	0.013540423	0.04675	0.02809	Rbp1/Cyp	2
GO:0031077	GO:0031077	post-embryonic camera-type eye development	2/385	11/23328	0.013540423	0.04675	0.02809	Flt1/Kdr	2
GO:0031642	GO:0031642	negative regulation of myelination	2/385	11/23328	0.013540423	0.04675	0.02809	Jam2/Klk8	2
GO:0033625	GO:0033625	positive regulation of integrin activation	2/385	11/23328	0.013540423	0.04675	0.02809	Cd24a/Ra	2
GO:0045647	GO:0045647	negative regulation of erythrocyte differentiation	2/385	11/23328	0.013540423	0.04675	0.02809	Lmo2/Zfp	2
GO:0046689	GO:0046689	response to mercury ion	2/385	11/23328	0.013540423	0.04675	0.02809	Fn1/Aqp1	2
GO:0048563	GO:0048563	post-embryonic animal organ morphogenesis	2/385	11/23328	0.013540423	0.04675	0.02809	Flt1/Kdr	2
GO:0048845	GO:0048845	venous blood vessel morphogenesis	2/385	11/23328	0.013540423	0.04675	0.02809	Eng/Prox1	2
GO:0051601	GO:0051601	exocyst localization	2/385	11/23328	0.013540423	0.04675	0.02809	Exoc3l2/T	2
GO:0051901	GO:0051901	positive regulation of mitochondrial depolarization	2/385	11/23328	0.013540423	0.04675	0.02809	Kdr/Dcn	2
GO:0060020	GO:0060020	Bergmann glial cell differentiation	2/385	11/23328	0.013540423	0.04675	0.02809	Vim/Plpp3	2
GO:0060056	GO:0060056	mammary gland involution	2/385	11/23328	0.013540423	0.04675	0.02809	Igfbp5/Ca	2
GO:0060346	GO:0060346	bone trabecula formation	2/385	11/23328	0.013540423	0.04675	0.02809	Sfrp1/Col	2
GO:0071732	GO:0071732	cellular response to nitric oxide	2/385	11/23328	0.013540423	0.04675	0.02809	Aqp1/Thb	2
GO:0072102	GO:0072102	glomerulus morphogenesis	2/385	11/23328	0.013540423	0.04675	0.02809	Notch3/Br	2
GO:2000121	GO:2000121	regulation of removal of superoxide radicals	2/385	11/23328	0.013540423	0.04675	0.02809	Cd36/Fbln	2
GO:2000644	GO:2000644	regulation of receptor catabolic process	2/385	11/23328	0.013540423	0.04675	0.02809	Anxa2/Ap	2
GO:2001280	GO:2001280	positive regulation of unsaturated fatty acid biosyntheti	2/385	11/23328	0.013540423	0.04675	0.02809	Cd74/Anx	2
GO:0014020	GO:0014020	primary neural tube formation	6/385	118/23328	0.013658077	0.04693	0.0282	Sfrp1/Spir	6
GO:0046545	GO:0046545	development of primary female sexual characteristics	6/385	118/23328	0.013658077	0.04693	0.0282	Sfrp1/Nup	6
GO:0060419	GO:0060419	heart growth	6/385	118/23328	0.013658077	0.04693	0.0282	Rgs4/Nca	6
GO:2000177	GO:2000177	regulation of neural precursor cell proliferation	6/385	118/23328	0.013658077	0.04693	0.0282	Btg2/Cd24	6
GO:0045777	GO:0045777	positive regulation of blood pressure	4/385	56/23328	0.013669387	0.04693	0.0282	Eng/Nr2f2	4
GO:0050873	GO:0050873	brown fat cell differentiation	4/385	56/23328	0.013669387	0.04693	0.0282	Fabp4/Itg	4
GO:0071675	GO:0071675	regulation of mononuclear cell migration	4/385	56/23328	0.013669387	0.04693	0.0282	Cxcl12/Lg	4
GO:0120032	GO:0120032	regulation of plasma membrane bounded cell projectio	8/385	190/23328	0.01390578	0.0477	0.02866	Gap43/Aq	8
GO:0003281	GO:0003281	ventricular septum development	5/385	86/23328	0.013973626	0.04786	0.02876	Pde2a/Bm	5
GO:0048708	GO:0048708	astrocyte differentiation	5/385	86/23328	0.013973626	0.04786	0.02876	Vim/Plpp3	5
GO:0002821	GO:0002821	positive regulation of adaptive immune response	7/385	154/23328	0.01421702	0.04822	0.02897	Rsad2/Cd	7
GO:0002833	GO:0002833	positive regulation of response to biotic stimulus	7/385	154/23328	0.01421702	0.04822	0.02897	Irf7/Mndal	7
GO:0001958	GO:0001958	endochondral ossification	3/385	31/23328	0.014240344	0.04822	0.02897	Pthlh/Col1	3
GO:0022011	GO:0022011	myelination in peripheral nervous system	3/385	31/23328	0.014240344	0.04822	0.02897	Ndrg1/Pm	3
GO:0023058	GO:0023058	adaptation of signaling pathway	3/385	31/23328	0.014240344	0.04822	0.02897	Aplnr/Adm	3
GO:0032292	GO:0032292	peripheral nervous system axon ensheathment	3/385	31/23328	0.014240344	0.04822	0.02897	Ndrg1/Pm	3
GO:0036075	GO:0036075	replacement ossification	3/385	31/23328	0.014240344	0.04822	0.02897	Pthlh/Col1	3
GO:0042481	GO:0042481	regulation of odontogenesis	3/385	31/23328	0.014240344	0.04822	0.02897	Cd34/Bmp	3
GO:0051497	GO:0051497	negative regulation of stress fiber assembly	3/385	31/23328	0.014240344	0.04822	0.02897	Cgnl1/S1p	3
GO:0060795	GO:0060795	cell fate commitment involved in formation of primary g	3/385	31/23328	0.014240344	0.04822	0.02897	Sox17/Bm	3
GO:0070670	GO:0070670	response to interleukin-4	3/385	31/23328	0.014240344	0.04822	0.02897	Mrc1/Tuba	3
GO:0095500	GO:0095500	acetylcholine receptor signaling pathway	3/385	31/23328	0.014240344	0.04822	0.02897	Ly6c1/Ly6	3
GO:1904031	GO:1904031	positive regulation of cyclin-dependent protein kinase a	3/385	31/23328	0.014240344	0.04822	0.02897	Rgcc/Pdg	3
GO:1905314	GO:1905314	semi-lunar valve development	3/385	31/23328	0.014240344	0.04822	0.02897	Bmp4/Gja	3
GO:0003170	GO:0003170	heart valve development	4/385	57/23328	0.014514515	0.04895	0.02941	Pde2a/Bm	4
GO:0035567	GO:0035567	non-canonical Wnt signaling pathway	4/385	57/23328	0.014514515	0.04895	0.02941	Sfrp1/Dab	4
GO:0046622	GO:0046622	positive regulation of organ growth	4/385	57/23328	0.014514515	0.04895	0.02941	Ncam1/Sl	4
GO:0060761	GO:0060761	negative regulation of response to cytokine stimulus	4/385	57/23328	0.014514515	0.04895	0.02941	Cav1/Gas	4
GO:0071622	GO:0071622	regulation of granulocyte chemotaxis	4/385	57/23328	0.014514515	0.04895	0.02941	Thbs1/Cd	4
GO:0002793	GO:0002793	positive regulation of peptide secretion	9/385	230/23328	0.01457689	0.04912	0.02951	Rgcc/Cd3	9
GO:0060420	GO:0060420	regulation of heart growth	5/385	87/23328	0.014630996	0.04926	0.0296	Rgs4/Nca	5
GO:0060491	GO:0060491	regulation of cell projection assembly	8/385	192/23328	0.014727675	0.04955	0.02977	Gap43/Aq	8
GO:0050670	GO:0050670	regulation of lymphocyte proliferation	9/385	231/23328	0.014955387	0.05027	0.03021	Prnp/Cd38	9
GO:0030856	GO:0030856	regulation of epithelial cell differentiation	7/385	156/23328	0.015165408	0.05094	0.03061	Id1/Cdh5/	7
GO:0051047	GO:0051047	positive regulation of secretion	14/385	440/23328	0.015215101	0.05106	0.03068	Rgcc/Cd3	14
GO:0062207	GO:0062207	regulation of pattern recognition receptor signaling patl	5/385	88/23328	0.01530878	0.05133	0.03084	Cd36/Irf7/	5
GO:0001736	GO:0001736	establishment of planar polarity	4/385	58/23328	0.015391989	0.05133	0.03084	Sfrp1/Dab	4
GO:0001754	GO:0001754	eye photoreceptor cell differentiation	4/385	58/23328	0.015391989	0.05133	0.03084	Thy1/Cfh/	4
GO:0001825	GO:0001825	blastocyst formation	4/385	58/23328	0.015391989	0.05133	0.03084	Sox17/Jur	4
GO:0010761	GO:0010761	fibroblast migration	4/385	58/23328	0.015391989	0.05133	0.03084	Thbs1/Pm	4
GO:0032663	GO:0032663	regulation of interleukin-2 production	4/385	58/23328	0.015391989	0.05133	0.03084	Prnp/Cd3	4
GO:0035456	GO:0035456	response to interferon-beta	4/385	58/23328	0.015391989	0.05133	0.03084	Mndal/Bst	4
GO:0045071	GO:0045071	negative regulation of viral genome replication	4/385	58/23328	0.015391989	0.05133	0.03084	Rsad2/Bs	4
GO:0010939	GO:0010939	regulation of necrotic cell death	3/385	32/23328	0.015524948	0.05169	0.03106	Slc25a4/C	3
GO:0099560	GO:0099560	synaptic membrane adhesion	3/385	32/23328	0.015524948	0.05169	0.03106	Sparcl1/P	3
GO:0060402	GO:0060402	calcium ion transport into cytosol	7/385	157/23328	0.015656111	0.05208	0.0313	Aplnr/Thy	7
GO:0002699	GO:0002699	positive regulation of immune effector process	10/385	273/23328	0.015770334	0.05235	0.03145	Cd36/Rsa	10
GO:0031109	GO:0031109	microtubule polymerization or depolymerization	6/385	122/23328	0.015871345	0.05235	0.03145	Cdh5/Gda	6
GO:0090101	GO:0090101	negative regulation of transmembrane receptor protein	6/385	122/23328	0.015871345	0.05235	0.03145	Sfrp1/Htra	6
GO:0051702	GO:0051702	interaction with symbiont	5/385	89/23328	0.016007229	0.05235	0.03145	Fn1/Aqp1	5
GO:0010749	GO:0010749	regulation of nitric oxide mediated signal transduction	2/385	12/23328	0.016072831	0.05235	0.03145	Thbs1/Pd	2
GO:0010755	GO:0010755	regulation of plasminogen activation	2/385	12/23328	0.016072831	0.05235	0.03145	Serpine2/	2
GO:0014745	GO:0014745	negative regulation of muscle adaptation	2/385	12/23328	0.016072831	0.05235	0.03145	Igfbp5/No	2
GO:0014824	GO:0014824	artery smooth muscle contraction	2/385	12/23328	0.016072831	0.05235	0.03145	Cd38/Edn	2
GO:0016264	GO:0016264	gap junction assembly	2/385	12/23328	0.016072831	0.05235	0.03145	Cav1/Gja	2
GO:0032490	GO:0032490	detection of molecule of bacterial origin	2/385	12/23328	0.016072831	0.05235	0.03145	Scarb1/Lb	2
GO:0033275	GO:0033275	actin-myosin filament sliding	2/385	12/23328	0.016072831	0.05235	0.03145	Tpm1/Act	2

GO:0033632	GO:0033632	regulation of cell-cell adhesion mediated by integrin	2/385	12/23328	0.016072831	0.05235	0.03145	Cd24a/Po	2
GO:0035376	GO:0035376	sterol import	2/385	12/23328	0.016072831	0.05235	0.03145	Cd36/Sca	2
GO:0035733	GO:0035733	hepatic stellate cell activation	2/385	12/23328	0.016072831	0.05235	0.03145	Pdgfb/Cyc	2
GO:0038063	GO:0038063	collagen-activated tyrosine kinase receptor signaling p	2/385	12/23328	0.016072831	0.05235	0.03145	Col4a2/Cc	2
GO:0042989	GO:0042989	sequestering of actin monomers	2/385	12/23328	0.016072831	0.05235	0.03145	Tmsb4x/T	2
GO:0043587	GO:0043587	tongue morphogenesis	2/385	12/23328	0.016072831	0.05235	0.03145	Tbx1/Cyp:	2
GO:0044766	GO:0044766	multi-organism transport	2/385	12/23328	0.016072831	0.05235	0.03145	Bst2/Cav1	2
GO:0046794	GO:0046794	transport of virus	2/385	12/23328	0.016072831	0.05235	0.03145	Bst2/Cav1	2
GO:0048241	GO:0048241	epinephrine transport	2/385	12/23328	0.016072831	0.05235	0.03145	Ly6e/Ptgs	2
GO:0048934	GO:0048934	peripheral nervous system neuron differentiation	2/385	12/23328	0.016072831	0.05235	0.03145	Hoxd9/Pm	2
GO:0048935	GO:0048935	peripheral nervous system neuron development	2/385	12/23328	0.016072831	0.05235	0.03145	Hoxd9/Pm	2
GO:0060213	GO:0060213	positive regulation of nuclear-transcribed mRNA poly(A	2/385	12/23328	0.016072831	0.05235	0.03145	Btg2/Zfp3	2
GO:0070278	GO:0070278	extracellular matrix constituent secretion	2/385	12/23328	0.016072831	0.05235	0.03145	Rgcc/Eng	2
GO:0070508	GO:0070508	cholesterol import	2/385	12/23328	0.016072831	0.05235	0.03145	Cd36/Sca	2
GO:1902579	GO:1902579	multi-organism localization	2/385	12/23328	0.016072831	0.05235	0.03145	Bst2/Cav1	2
GO:2000343	GO:2000343	positive regulation of chemokine (C-X-C motif) ligand 2	2/385	12/23328	0.016072831	0.05235	0.03145	Lpl/Cd74	2
GO:0007548	GO:0007548	sex differentiation	11/385	315/23328	0.016118007	0.05245	0.03152	Sfrp1/Nup	11
GO:0032944	GO:0032944	regulation of mononuclear cell proliferation	9/385	234/23328	0.016134355	0.05246	0.03153	Prnp/Cd3	9
GO:0007164	GO:0007164	establishment of tissue polarity	4/385	59/23328	0.016302174	0.05281	0.03173	Sfrp1/Dab	4
GO:0007528	GO:0007528	neuromuscular junction development	4/385	59/23328	0.016302174	0.05281	0.03173	Tnc/Ptn/P	4
GO:0042551	GO:0042551	neuron maturation	4/385	59/23328	0.016302174	0.05281	0.03173	Cdkn1c/P	4
GO:0043462	GO:0043462	regulation of ATPase activity	4/385	59/23328	0.016302174	0.05281	0.03173	Myl4/Tpm	4
GO:0060443	GO:0060443	mammary gland morphogenesis	4/385	59/23328	0.016302174	0.05281	0.03173	Igfbp5/Pth	4
GO:0048565	GO:0048565	digestive tract development	6/385	123/23328	0.016461183	0.05328	0.03202	Sfrp1/Sox	6
GO:0032868	GO:0032868	response to insulin	9/385	235/23328	0.016542088	0.0535	0.03215	C1qtnf9/S	9
GO:0071383	GO:0071383	cellular response to steroid hormone stimulus	7/385	159/23328	0.016671166	0.05383	0.03235	Sfrp1/Aqp	7
GO:2000058	GO:2000058	regulation of ubiquitin-dependent protein catabolic proc	7/385	159/23328	0.016671166	0.05383	0.03235	Plk2/Clu/I	7
GO:0010667	GO:0010667	negative regulation of cardiac muscle cell apoptotic pro	3/385	33/23328	0.016872723	0.05411	0.03251	Nupr1/Slc	3
GO:0022617	GO:0022617	extracellular matrix disassembly	3/385	33/23328	0.016872723	0.05411	0.03251	Pdpn/Eng	3
GO:0031112	GO:0031112	positive regulation of microtubule polymerization or dep	3/385	33/23328	0.016872723	0.05411	0.03251	Gda/Stmn	3
GO:0032369	GO:0032369	negative regulation of lipid transport	3/385	33/23328	0.016872723	0.05411	0.03251	Igfbp3/Thl	3
GO:0050654	GO:0050654	chondroitin sulfate proteoglycan metabolic process	3/385	33/23328	0.016872723	0.05411	0.03251	Bgn/Cytl1	3
GO:0060706	GO:0060706	cell differentiation involved in embryonic placenta deve	3/385	33/23328	0.016872723	0.05411	0.03251	Socs3/Nr2	3
GO:0060740	GO:0060740	prostate gland epithelium morphogenesis	3/385	33/23328	0.016872723	0.05411	0.03251	Sfrp1/Tnc	3
GO:1905144	GO:1905144	response to acetylcholine	3/385	33/23328	0.016872723	0.05411	0.03251	Ly6c1/Ly6	3
GO:1905145	GO:1905145	cellular response to acetylcholine	3/385	33/23328	0.016872723	0.05411	0.03251	Ly6c1/Ly6	3
GO:0002822	GO:0002822	regulation of adaptive immune response based on som	8/385	197/23328	0.016936161	0.05427	0.03261	Rsad2/Cd	8
GO:0043500	GO:0043500	muscle adaptation	6/385	124/23328	0.017065973	0.05465	0.03284	Rgs4/Igfb	6
GO:0010950	GO:0010950	positive regulation of endopeptidase activity	7/385	160/23328	0.017195768	0.05493	0.033	Perp/Lgm	7
GO:0051302	GO:0051302	regulation of cell division	7/385	160/23328	0.017195768	0.05493	0.033	Plk2/Pdgf	7
GO:0045744	GO:0045744	negative regulation of G protein-coupled receptor signa	4/385	60/23328	0.017245418	0.05493	0.033	Aplnr/Rgs	4
GO:0070098	GO:0070098	chemokine-mediated signaling pathway	4/385	60/23328	0.017245418	0.05493	0.033	Cxcl12/Cc	4
GO:0090183	GO:0090183	regulation of kidney development	4/385	60/23328	0.017245418	0.05493	0.033	Pdgfb/Egr	4
GO:1901016	GO:1901016	regulation of potassium ion transmembrane transporte	4/385	60/23328	0.017245418	0.05493	0.033	Fhl1/Cav1	4
GO:1901616	GO:1901616	organic hydroxy compound catabolic process	4/385	60/23328	0.017245418	0.05493	0.033	Adh7/Sca	4
GO:0002088	GO:0002088	lens development in camera-type eye	5/385	91/23328	0.017467094	0.0555	0.03335	Vim/Cdkn	5
GO:0031110	GO:0031110	regulation of microtubule polymerization or depolymeri	5/385	91/23328	0.017467094	0.0555	0.03335	Cdh5/Gda	5
GO:0044344	GO:0044344	cellular response to fibroblast growth factor stimulus	5/385	91/23328	0.017467094	0.0555	0.03335	Sfrp1/Tbx	5
GO:0031641	GO:0031641	regulation of myelination	4/385	61/23328	0.018222049	0.05753	0.03457	Jam2/Ptn	4
GO:0032732	GO:0032732	positive regulation of interleukin-1 production	4/385	61/23328	0.018222049	0.05753	0.03457	Hspb1/Mn	4
GO:0042698	GO:0042698	ovulation cycle	4/385	61/23328	0.018222049	0.05753	0.03457	Ptn/Egr1/	4
GO:0045776	GO:0045776	negative regulation of blood pressure	4/385	61/23328	0.018222049	0.05753	0.03457	Cd36/Ier3	4
GO:0051851	GO:0051851	modulation by host of symbiont process	4/385	61/23328	0.018222049	0.05753	0.03457	Aqp1/Anx	4
GO:0055072	GO:0055072	iron ion homeostasis	5/385	92/23328	0.018228981	0.05753	0.03457	Cp/Steap	5
GO:0003209	GO:0003209	cardiac atrium morphogenesis	3/385	34/23328	0.018283924	0.05753	0.03457	Eng/Prox1	3
GO:0014044	GO:0014044	Schwann cell development	3/385	34/23328	0.018283924	0.05753	0.03457	Ndrg1/Pm	3
GO:0031338	GO:0031338	regulation of vesicle fusion	3/385	34/23328	0.018283924	0.05753	0.03457	Tbc1d4/A	3
GO:0043537	GO:0043537	negative regulation of blood vessel endothelial cell mig	3/385	34/23328	0.018283924	0.05753	0.03457	Rgcc/Thb	3
GO:0060512	GO:0060512	prostate gland morphogenesis	3/385	34/23328	0.018283924	0.05753	0.03457	Sfrp1/Tnc	3
GO:0090022	GO:0090022	regulation of neutrophil chemotaxis	3/385	34/23328	0.018283924	0.05753	0.03457	Cd74/Lbp	3
GO:0090218	GO:0090218	positive regulation of lipid kinase activity	3/385	34/23328	0.018283924	0.05753	0.03457	Flt1/Pdgfb	3
GO:0036294	GO:0036294	cellular response to decreased oxygen levels	6/385	126/23328	0.01832107	0.05761	0.03462	Rgcc/Fnd	6
GO:0001906	GO:0001906	cell killing	8/385	200/23328	0.018370493	0.05767	0.03466	Cxcl12/Cf	8
GO:0071805	GO:0071805	potassium ion transmembrane transport	8/385	200/23328	0.018370493	0.05767	0.03466	Rgs4/Abc	8
GO:0003091	GO:0003091	renal water homeostasis	2/385	13/23328	0.018790077	0.05821	0.03498	Aqp1/Aqp	2
GO:0009404	GO:0009404	toxin metabolic process	2/385	13/23328	0.018790077	0.05821	0.03498	Pam/Cyp1	2
GO:0010744	GO:0010744	positive regulation of macrophage derived foam cell di	2/385	13/23328	0.018790077	0.05821	0.03498	Cd36/Lpl	2
GO:0021683	GO:0021683	cerebellar granular layer morphogenesis	2/385	13/23328	0.018790077	0.05821	0.03498	Serpine2/	2
GO:0032799	GO:0032799	low-density lipoprotein receptor particle metabolic proc	2/385	13/23328	0.018790077	0.05821	0.03498	Anxa2/Ap	2
GO:0034350	GO:0034350	regulation of glial cell apoptotic process	2/385	13/23328	0.018790077	0.05821	0.03498	Gas6/Pmp	2
GO:0035627	GO:0035627	ceramide transport	2/385	13/23328	0.018790077	0.05821	0.03498	Pltp/Abcb	2
GO:0042482	GO:0042482	positive regulation of odontogenesis	2/385	13/23328	0.018790077	0.05821	0.03498	Cd34/Edn	2
GO:0043691	GO:0043691	reverse cholesterol transport	2/385	13/23328	0.018790077	0.05821	0.03498	Scarb1/Ap	2
GO:0071073	GO:0071073	positive regulation of phospholipid biosynthetic proces	2/385	13/23328	0.018790077	0.05821	0.03498	Adgrf5/Ht	2
GO:0071281	GO:0071281	cellular response to iron ion	2/385	13/23328	0.018790077	0.05821	0.03498	B2m/Trf	2
GO:0071294	GO:0071294	cellular response to zinc ion	2/385	13/23328	0.018790077	0.05821	0.03498	Mt1/Mt2	2
GO:0072376	GO:0072376	protein activation cascade	2/385	13/23328	0.018790077	0.05821	0.03498	Fn1/Fbln1	2
GO:0090193	GO:0090193	positive regulation of glomerulus development	2/385	13/23328	0.018790077	0.05821	0.03498	Pdgfb/Egr	2
GO:0097011	GO:0097011	cellular response to granulocyte macrophage colony-st	2/385	13/23328	0.018790077	0.05821	0.03498	Zfp36/Pde	2
GO:1900025	GO:1900025	negative regulation of substrate adhesion-dependent c	2/385	13/23328	0.018790077	0.05821	0.03498	Fbln1/Kan	2
GO:1902170	GO:1902170	cellular response to reactive nitrogen species	2/385	13/23328	0.018790077	0.05821	0.03498	Aqp1/Thb	2
GO:1904181	GO:1904181	positive regulation of membrane depolarization	2/385	13/23328	0.018790077	0.05821	0.03498	Kdr/Dcn	2
GO:0032869	GO:0032869	cellular response to insulin stimulus	8/385	201/23328	0.018867371	0.05836	0.03507	C1qtnf9/S	8
GO:0044843	GO:0044843	cell cycle G1/S phase transition	8/385	201/23328	0.018867371	0.05836	0.03507	Rgcc/Plk2	8
GO:0043506	GO:0043506	regulation of JUN kinase activity	5/385	93/23328	0.019012473	0.05872	0.03529	Sfrp1/Dab	5
GO:0045445	GO:0045445	myoblast differentiation	5/385	93/23328	0.019012473	0.05872	0.03529	Id3/Igfbp3	5
GO:0014911	GO:0014911	positive regulation of smooth muscle cell migration	4/385	62/23328	0.019232375	0.05931	0.03564	Tmsb4x/I	4
GO:0051058	GO:0051058	negative regulation of small GTPase mediated signal t	4/385	62/23328	0.019232375	0.05931	0.03564	Cgnl1/Ras	4
GO:0070588	GO:0070588	calcium ion transmembrane transport	10/385	282/23328	0.019297991	0.05947	0.03574	Aplnr/Thy	10
GO:0034249	GO:0034249	negative regulation of cellular amide metabolic proces	7/385	164/23328	0.019410844	0.05978	0.03592	Spon1/Btg	7
GO:0032147	GO:0032147	activation of protein kinase activity	9/385	242/23328	0.019610267	0.06034	0.03626	Prnp/Pdgf	9
GO:0002082	GO:0002082	regulation of oxidative phosphorylation	3/385	35/23328	0.019758736	0.06045	0.03632	Nupr1/Pde	3
GO:0035025	GO:0035025	positive regulation of Rho protein signal transduction	3/385	35/23328	0.019758736	0.06045	0.03632	Mcf2l/F2r	3
GO:0043243	GO:0043243	positive regulation of protein-containing complex disas	3/385	35/23328	0.019758736	0.06045	0.03632	Dstn/Nes/	3
GO:0045746	GO:0045746	negative regulation of Notch signaling pathway	3/385	35/23328	0.019758736	0.06045	0.03632	Nrarp/Egf	3
GO:0045987	GO:0045987	positive regulation of smooth muscle contraction	3/385	35/23328	0.019758736	0.06045	0.03632	F2r/Edn1/	3
GO:0060142	GO:0060142	regulation of syncytium formation by plasma membran	3/385	35/23328	0.019758736	0.06045	0.03632	Cxcl12/Cx	3
GO:0060292	GO:0060292	long-term synaptic depression	3/385	35/23328	0.019758736	0.06045	0.03632	Mgll/Cd38	3
GO:0071320	GO:0071320	cellular response to cAMP	3/385	35/23328	0.019758736	0.06045	0.03632	Igfbp5/Aq	3
GO:0070252	GO:0070252	actin-mediated cell contraction	5/385	94/23328	0.019817788	0.06054	0.03638	Pdpn/Cav	5
GO:0070664	GO:0070664	negative regulation of leukocyte proliferation	5/385	94/23328	0.019817788	0.06054	0.03638	Prnp/Cd2	5
GO:0006575	GO:0006575	cellular modified amino acid metabolic process	7/385	165/23328	0.019994375	0.06103	0.03667	Reln/Duox	7
GO:0030104	GO:0030104	water homeostasis	4/385	63/23328	0.020276688	0.06167	0.03706	Aqp1/Aqp	4
GO:0032760	GO:0032760	positive regulation of tumor necrosis factor production	4/385	63/23328	0.020276688	0.06167	0.03706	Cd36/Clu/	4
GO:0045661	GO:0045661	regulation of myoblast differentiation	4/385	63/23328	0.020276688	0.06167	0.03706	Id3/Igfbp3	4
GO:0071479	GO:0071479	cellular response to ionizing radiation	4/385	63/23328	0.020276688	0.06167	0.03706	Sfrp1/Cdk	4
GO:1903428	GO:1903428	positive regulation of reactive oxygen species biosynth	4/385	63/23328	0.020276688	0.06167	0.03706	Cd36/Duo	4
GO:0051896	GO:0051896	regulation of protein kinase B signaling	7/385	166/23328	0.020590045	0.06253	0.03758	Igfbp5/En	7
GO:0098739	GO:0098739	import across plasma membrane	7/385	166/23328	0.020590045	0.06253	0.03758	Rgs4/Abc	7
GO:2001243	GO:2001243	negative regulation of intrinsic apoptotic signaling path	5/385	95/23328	0.020645138	0.06265	0.03765	Cxcl12/Hs	5
GO:0006775	GO:0006775	fat-soluble vitamin metabolic process	3/385	36/23328	0.021297278	0.06382	0.03835	Pltp/Rbp1	3
GO:0010453	GO:0010453	regulation of cell fate commitment	3/385	36/23328	0.021297278	0.06382	0.03835	Sox17/Bm	3
GO:0010664	GO:0010664	negative regulation of striated muscle cell apoptotic pro	3/385	36/23328	0.021297278	0.06382	0.03835	Nupr1/Slc	3
GO:0032232	GO:0032232	negative regulation of actin filament bundle assembly	3/385	36/23328	0.021297278	0.06382	0.03835	Cgnl1/S1p	3
GO:0048009	GO:0048009	insulin-like growth factor receptor signaling pathway	3/385	36/23328	0.021297278	0.06382	0.03835	Igfbp5/Igf	3
GO:0048679	GO:0048679	regulation of axon regeneration	3/385	36/23328	0.021297278	0.06382	0.03835	Ptn/Klk8/F	3
GO:0060351	GO:0060351	cartilage development involved in endochondral bone	3/385	36/23328	0.021297278	0.06382	0.03835	Col1a1/Th	3
GO:0061384	GO:0061384	heart trabecula morphogenesis	3/385	36/23328	0.021297278	0.06382	0.03835	S1pr1/Eng	3
GO:1902624	GO:1902624	positive regulation of neutrophil migration	3/385	36/23328	0.021297278	0.06382	0.03835	Cd74/Lbp	3
GO:1905209	GO:1905209	positive regulation of cardiocyte differentiation	3/385	36/23328	0.021297278	0.06382	0.03835	Slc25a4/E	3
GO:2000171	GO:2000171	negative regulation of dendrite development	3/385	36/23328	0.021297278	0.06382	0.03835	Id1/Plk2/A	3
GO:0045668	GO:0045668	negative regulation of osteoblast differentiation	4/385	64/23328	0.021355259	0.06382	0.03835	Id1/Sfrp1/	4
GO:0048008	GO:0048008	platelet-derived growth factor receptor signaling pathw	4/385	64/23328	0.021355259	0.06382	0.03835	Lox/Pdgfb	4
GO:1903557	GO:1903557	positive regulation of tumor necrosis factor superfamily	4/385	64/23328	0.021355259	0.06382	0.03835	Cd36/Clu/	4
GO:1903578	GO:1903578	regulation of ATP metabolic process	5/385	96/23328	0.021494728	0.06382	0.03835	Nupr1/Ier3	5
GO:1902115	GO:1902115	regulation of organelle assembly	8/385	206/23328	0.021496608	0.06382	0.03835	Gap43/Nu	8
GO:0010644	GO:0010644	cell communication by electrical coupling	2/385	14/23328	0.021685404	0.06382	0.03835	Cav1/Gja5	2
GO:0010882	GO:0010882	regulation of cardiac muscle contraction by calcium ior	2/385	14/23328	0.021685404	0.06382	0.03835	Atp1b1/Tr	2
GO:0032769	GO:0032769	negative regulation of monooxygenase activity	2/385	14/23328	0.021685404	0.06382	0.03835	Eng/Cav1	2

GO:0035358	GO:0035358	regulation of peroxisome proliferator activated recepto	2/385	14/23328	0.021685404	0.06382	0.03835	Fabp5/Ptg	2
GO:0036296	GO:0036296	response to increased oxygen levels	2/385	14/23328	0.021685404	0.06382	0.03835	Pdpn/Cav	2
GO:0038065	GO:0038065	collagen-activated signaling pathway	2/385	14/23328	0.021685404	0.06382	0.03835	Col4a2/Co	2
GO:0042532	GO:0042532	negative regulation of tyrosine phosphorylation of STA	2/385	14/23328	0.021685404	0.06382	0.03835	Socs3/Ca	2
GO:0043508	GO:0043508	negative regulation of JUN kinase activity	2/385	14/23328	0.021685404	0.06382	0.03835	Sfrp1/Sfrp	2
GO:0044764	GO:0044764	multi-organism cellular process	2/385	14/23328	0.021685404	0.06382	0.03835	Cav1/Gas	2
GO:0046886	GO:0046886	positive regulation of hormone biosynthetic process	2/385	14/23328	0.021685404	0.06382	0.03835	Egr1/Dab	2
GO:0051152	GO:0051152	positive regulation of smooth muscle cell differentiation	2/385	14/23328	0.021685404	0.06382	0.03835	Tmsb4x/E	2
GO:0060211	GO:0060211	regulation of nuclear-transcribed mRNA poly(A) tail sh	2/385	14/23328	0.021685404	0.06382	0.03835	Btg2/Zfp3	2
GO:0060263	GO:0060263	regulation of respiratory burst	2/385	14/23328	0.021685404	0.06382	0.03835	Lbp/Jchai	2
GO:0061450	GO:0061450	trophoblast cell migration	2/385	14/23328	0.021685404	0.06382	0.03835	Arhgdib/Itg	2
GO:0061469	GO:0061469	regulation of type B pancreatic cell proliferation	2/385	14/23328	0.021685404	0.06382	0.03835	Sfrp1/Nup	2
GO:0071850	GO:0071850	mitotic cell cycle arrest	2/385	14/23328	0.021685404	0.06382	0.03835	Rgcc/Cdk	2
GO:0090179	GO:0090179	planar cell polarity pathway involved in neural tube clo	2/385	14/23328	0.021685404	0.06382	0.03835	Sfrp1/Sfrp	2
GO:0090331	GO:0090331	negative regulation of platelet aggregation	2/385	14/23328	0.021685404	0.06382	0.03835	Cd9/Serpi	2
GO:0097012	GO:0097012	response to granulocyte macrophage colony-stimulati	2/385	14/23328	0.021685404	0.06382	0.03835	Zfp36/Pde	2
GO:0097202	GO:0097202	activation of cysteine-type endopeptidase activity	2/385	14/23328	0.021685404	0.06382	0.03835	Perp/Lgm	2
GO:0099550	GO:0099550	trans-synaptic signaling, modulating synaptic transmis	2/385	14/23328	0.021685404	0.06382	0.03835	F2r/Plat	2
GO:1902950	GO:1902950	regulation of dendritic spine maintenance	2/385	14/23328	0.021685404	0.06382	0.03835	Prnp/Apoe	2
GO:1903236	GO:1903236	regulation of leukocyte tethering or rolling	2/385	14/23328	0.021685404	0.06382	0.03835	Cxcl12/Se	2
GO:1904948	GO:1904948	midbrain dopaminergic neuron differentiation	2/385	14/23328	0.021685404	0.06382	0.03835	Sfrp1/Sfrp	2
GO:1905063	GO:1905063	regulation of vascular associated smooth muscle cell	2/385	14/23328	0.021685404	0.06382	0.03835	Pdgfb/Eng	2
GO:2000035	GO:2000035	regulation of stem cell division	2/385	14/23328	0.021685404	0.06382	0.03835	Sox17/Sfr	2
GO:2000052	GO:2000052	positive regulation of non-canonical Wnt signaling path	2/385	14/23328	0.021685404	0.06382	0.03835	Sfrp1/Dab	2
GO:0002761	GO:0002761	regulation of myeloid leukocyte differentiation	6/385	131/2332	0.021731784	0.06391	0.0384	Sfrp1/Rbp	6
GO:0032233	GO:0032233	positive regulation of actin filament bundle assembly	4/385	65/23328	0.022468341	0.06599	0.03965	Rgcc/Id1/	4
GO:0090398	GO:0090398	cellular senescence	4/385	65/23328	0.022468341	0.06599	0.03965	Plk2/B2m	4
GO:0002053	GO:0002053	positive regulation of mesenchymal cell proliferation	3/385	37/23328	0.022899606	0.06702	0.04027	Tbx1/Kdr/	3
GO:0007435	GO:0007435	salivary gland morphogenesis	3/385	37/23328	0.022899606	0.06702	0.04027	Pdgfb/Tgr	3
GO:0009595	GO:0009595	detection of biotic stimulus	3/385	37/23328	0.022899606	0.06702	0.04027	Scarb1/Lb	3
GO:0033363	GO:0033363	secretory granule organization	3/385	37/23328	0.022899606	0.06702	0.04027	Aqp1/Srgr	3
GO:1903427	GO:1903427	negative regulation of reactive oxygen species biosynt	3/385	37/23328	0.022899606	0.06702	0.04027	Cd34/Ptgi	3
GO:0034976	GO:0034976	response to endoplasmic reticulum stress	9/385	249/2332	0.023070785	0.06747	0.04054	Atf3/Nupr	9
GO:1904064	GO:1904064	positive regulation of cation transmembrane transport	7/385	170/2332	0.023096355	0.0675	0.04056	Aplnr/Relr	7
GO:0046942	GO:0046942	carboxylic acid transport	11/385	333/2332	0.023185121	0.06764	0.04064	Fabp4/Cd	11
GO:0043281	GO:0043281	regulation of cysteine-type endopeptidase activity invo	8/385	209/2332	0.023193208	0.06764	0.04064	Aqp1/Thb	8
GO:0001841	GO:0001841	neural tube formation	6/385	133/2332	0.023208452	0.06764	0.04064	Sfrp1/Spir	6
GO:0044070	GO:0044070	regulation of anion transport	6/385	133/2332	0.023208452	0.06764	0.04064	Rgs4/Prnp	6
GO:0030258	GO:0030258	lipid modification	9/385	250/2332	0.023598583	0.06873	0.0413	Cd36/Adh	9
GO:0032623	GO:0032623	interleukin-2 production	4/385	66/23328	0.023616171	0.06873	0.0413	Prnp/Cd3	4
GO:0010952	GO:0010952	positive regulation of peptidase activity	7/385	171/2332	0.02375439	0.06904	0.04148	Perp/Lgm	7
GO:0046661	GO:0046661	male sex differentiation	7/385	171/2332	0.02375439	0.06904	0.04148	Sfrp1/Nup	7
GO:0002698	GO:0002698	negative regulation of immune effector process	6/385	134/2332	0.023971446	0.06957	0.0418	Fgl2/Htra1	6
GO:1902806	GO:1902806	regulation of cell cycle G1/S phase transition	6/385	134/2332	0.023971446	0.06957	0.0418	Rgcc/Fhl1	6
GO:0007200	GO:0007200	phospholipase C-activating G protein-coupled recepto	5/385	99/23328	0.024178884	0.06994	0.04202	Tgm2/S1p	5
GO:0062014	GO:0062014	negative regulation of small molecule metabolic proce	5/385	99/23328	0.024178884	0.06994	0.04202	Nupr1/Ier	5
GO:0042180	GO:0042180	cellular ketone metabolic process	8/385	211/2332	0.024375312	0.06994	0.04202	Fabp5/Ca	8
GO:0060041	GO:0060041	retina development in camera-type eye	7/385	172/2332	0.024425221	0.06994	0.04202	Thy1/Cfh/	7
GO:0043434	GO:0043434	response to peptide hormone	11/385	336/2332	0.024553704	0.06994	0.04202	C1qtnf9/S	11
GO:0014002	GO:0014002	astrocyte development	3/385	38/23328	0.024565713	0.06994	0.04202	Vim/S100	3
GO:0035335	GO:0035335	peptidyl-tyrosine dephosphorylation	3/385	38/23328	0.024565713	0.06994	0.04202	Dusp1/Ptp	3
GO:0048265	GO:0048265	response to pain	3/385	38/23328	0.024565713	0.06994	0.04202	Reln/Thbs	3
GO:0060219	GO:0060219	camera-type eye photoreceptor cell differentiation	3/385	38/23328	0.024565713	0.06994	0.04202	Thy1/Cfh/	3
GO:0060674	GO:0060674	placenta blood vessel development	3/385	38/23328	0.024565713	0.06994	0.04202	Socs3/Jur	3
GO:0062208	GO:0062208	positive regulation of pattern recognition receptor sign	3/385	38/23328	0.024565713	0.06994	0.04202	Rsad2/Ca	3
GO:0070528	GO:0070528	protein kinase C signaling	3/385	38/23328	0.024565713	0.06994	0.04202	Pdgfb/Htr	3
GO:0010561	GO:0010561	negative regulation of glycoprotein biosynthetic proces	2/385	15/23328	0.024752225	0.06994	0.04202	Bace2/Ac	2
GO:0010715	GO:0010715	regulation of extracellular matrix disassembly	2/385	15/23328	0.024752225	0.06994	0.04202	Pdpn/Fscr	2
GO:0010935	GO:0010935	regulation of macrophage cytokine production	2/385	15/23328	0.024752225	0.06994	0.04202	Cd36/Cd7	2
GO:0010958	GO:0010958	regulation of amino acid import across plasma membra	2/385	15/23328	0.024752225	0.06994	0.04202	Rgs4/Itgb	2
GO:0016114	GO:0016114	terpenoid biosynthetic process	2/385	15/23328	0.024752225	0.06994	0.04202	Rbp1/Cyp	2
GO:0031340	GO:0031340	positive regulation of vesicle fusion	2/385	15/23328	0.024752225	0.06994	0.04202	Anxa2/An	2
GO:0032530	GO:0032530	regulation of microvillus organization	2/385	15/23328	0.024752225	0.06994	0.04202	Fscn1/Po	2
GO:0033690	GO:0033690	positive regulation of osteoblast proliferation	2/385	15/23328	0.024752225	0.06994	0.04202	Fbln5/Cth	2
GO:0042178	GO:0042178	xenobiotic catabolic process	2/385	15/23328	0.024752225	0.06994	0.04202	Cyp1a1/G	2
GO:0042953	GO:0042953	lipoprotein transport	2/385	15/23328	0.024752225	0.06994	0.04202	Cd36/Lsr	2
GO:0043031	GO:0043031	negative regulation of macrophage activation	2/385	15/23328	0.024752225	0.06994	0.04202	Adgrf5/Cd	2
GO:0046541	GO:0046541	saliva secretion	2/385	15/23328	0.024752225	0.06994	0.04202	Kcnn4/Aq	2
GO:0050930	GO:0050930	induction of positive chemotaxis	2/385	15/23328	0.024752225	0.06994	0.04202	Cxcl12/Co	2
GO:0060442	GO:0060442	branching involved in prostate gland morphogenesis	2/385	15/23328	0.024752225	0.06994	0.04202	Sfrp1/Bmp	2
GO:0060546	GO:0060546	negative regulation of necroptotic process	2/385	15/23328	0.024752225	0.06994	0.04202	Slc25a4/C	2
GO:0061051	GO:0061051	positive regulation of cell growth involved in cardiac m	2/385	15/23328	0.024752225	0.06994	0.04202	Slc25a4/E	2
GO:0061430	GO:0061430	bone trabecula morphogenesis	2/385	15/23328	0.024752225	0.06994	0.04202	Sfrp1/Col	2
GO:0062099	GO:0062099	negative regulation of programmed necrotic cell death	2/385	15/23328	0.024752225	0.06994	0.04202	Slc25a4/C	2
GO:0070307	GO:0070307	lens fiber cell development	2/385	15/23328	0.024752225	0.06994	0.04202	Vim/Prox1	2
GO:0090178	GO:0090178	regulation of establishment of planar polarity involved i	2/385	15/23328	0.024752225	0.06994	0.04202	Sfrp1/Sfrp	2
GO:0090594	GO:0090594	inflammatory response to wounding	2/385	15/23328	0.024752225	0.06994	0.04202	Cd24a/F2	2
GO:0150105	GO:0150105	protein localization to cell-cell junction	2/385	15/23328	0.024752225	0.06994	0.04202	Cdh5/Lsr	2
GO:1900121	GO:1900121	negative regulation of receptor binding	2/385	15/23328	0.024752225	0.06994	0.04202	B2m/Ptprf	2
GO:1903587	GO:1903587	regulation of blood vessel endothelial cell proliferation	2/385	15/23328	0.024752225	0.06994	0.04202	Aplnr/Thb	2
GO:1903789	GO:1903789	regulation of amino acid transmembrane transport	2/385	15/23328	0.024752225	0.06994	0.04202	Rgs4/Itgb	2
GO:2000811	GO:2000811	negative regulation of anoikis	2/385	15/23328	0.024752225	0.06994	0.04202	Cav1/Itgb	2
GO:0000768	GO:0000768	syncytium formation by plasma membrane fusion	4/385	67/23328	0.024798964	0.06994	0.04202	Cxcl12/Co	4
GO:0060688	GO:0060688	regulation of morphogenesis of a branching structure	4/385	67/23328	0.024798964	0.06994	0.04202	Sfrp1/Cpe	4
GO:0070613	GO:0070613	regulation of protein processing	4/385	67/23328	0.024798964	0.06994	0.04202	Spon1/Se	4
GO:0140253	GO:0140253	cell-cell fusion	4/385	67/23328	0.024798964	0.06994	0.04202	Cxcl12/Co	4
GO:0071214	GO:0071214	cellular response to abiotic stimulus	10/385	294/2332	0.024860022	0.07001	0.04207	Sfrp1/Aqp	10
GO:0104004	GO:0104004	cellular response to environmental stimulus	10/385	294/2332	0.024860022	0.07001	0.04207	Sfrp1/Aqp	10
GO:0015849	GO:0015849	organic acid transport	11/385	337/2332	0.025022705	0.07042	0.04232	Fabp4/Cd	11
GO:0010771	GO:0010771	negative regulation of cell morphogenesis involved in	5/385	100/2332	0.025119344	0.07065	0.04245	Thy1/Id1/	5
GO:0071375	GO:0071375	cellular response to peptide hormone stimulus	9/385	253/2332	0.025233628	0.07092	0.04262	C1qtnf9/S	9
GO:0043271	GO:0043271	negative regulation of ion transport	7/385	174/2332	0.025805687	0.07248	0.04355	Rgs4/Serp	7
GO:0002763	GO:0002763	positive regulation of myeloid leukocyte differentiation	4/385	68/23328	0.026016918	0.07283	0.04376	Kitl/Fos/Ju	4
GO:0007566	GO:0007566	embryo implantation	4/385	68/23328	0.026016918	0.07283	0.04376	Ptgis/Arhg	4
GO:0043949	GO:0043949	regulation of cAMP-mediated signaling	4/385	68/23328	0.026016918	0.07283	0.04376	Aplnr/Pde	4
GO:0046579	GO:0046579	positive regulation of Ras protein signal transduction	4/385	68/23328	0.026016918	0.07283	0.04376	Mcf2l/Kitl/	4
GO:1901264	GO:1901264	carbohydrate derivative transport	4/385	68/23328	0.026016918	0.07283	0.04376	Scarb1/Pl	4
GO:0033555	GO:0033555	multicellular organismal response to stress	5/385	101/2332	0.026082963	0.07292	0.04382	Reln/Ncar	5
GO:2001237	GO:2001237	negative regulation of extrinsic apoptotic signaling path	5/385	101/2332	0.026082963	0.07292	0.04382	Itga6/Dab	5
GO:0003230	GO:0003230	cardiac atrium development	3/385	39/23328	0.026295536	0.07317	0.04397	Eng/Prox1	3
GO:0010613	GO:0010613	positive regulation of cardiac muscle hypertrophy	3/385	39/23328	0.026295536	0.07317	0.04397	Ece1/Slc2	3
GO:0032781	GO:0032781	positive regulation of ATPase activity	3/385	39/23328	0.026295536	0.07317	0.04397	Myl4/Tpm	3
GO:0060251	GO:0060251	regulation of glial cell proliferation	3/385	39/23328	0.026295536	0.07317	0.04397	Vim/Ptn/P	3
GO:1900087	GO:1900087	positive regulation of G1/S transition of mitotic cell cyc	3/385	39/23328	0.026295536	0.07317	0.04397	Rgcc/Cyp	3
GO:1902108	GO:1902108	regulation of mitochondrial membrane permeability inv	3/385	39/23328	0.026295536	0.07317	0.04397	Slc25a4/C	3
GO:2000403	GO:2000403	positive regulation of lymphocyte migration	3/385	39/23328	0.026295536	0.07317	0.04397	Cxcl12/Co	3
GO:0035821	GO:0035821	modulation of process of other organism	6/385	137/2332	0.026360691	0.0733	0.04405	Aqp1/Anx	6
GO:0048839	GO:0048839	inner ear development	8/385	215/2332	0.026864979	0.07465	0.04486	Igfbp7/Tb	8
GO:0048675	GO:0048675	axon extension	6/385	138/2332	0.027190959	0.07546	0.04534	Mgll/Cxcl1	6
GO:0050671	GO:0050671	positive regulation of lymphocyte proliferation	6/385	138/2332	0.027190959	0.07546	0.04534	Cd38/Cd2	6
GO:0006949	GO:0006949	syncytium formation	4/385	69/23328	0.027270215	0.07563	0.04545	Cxcl12/Co	4
GO:0051403	GO:0051403	stress-activated MAPK cascade	9/385	257/2332	0.027536664	0.07613	0.04575	Sfrp1/Dus	9
GO:0035148	GO:0035148	tube formation	7/385	177/2332	0.027974897	0.07613	0.04575	Sfrp1/Tgm	7
GO:0060401	GO:0060401	cytosolic calcium ion transport	7/385	177/2332	0.027974897	0.07613	0.04575	Aplnr/Thy	7
GO:0006825	GO:0006825	copper ion transport	2/385	16/23328	0.027984123	0.07613	0.04575	Cp/Steap	2
GO:0014067	GO:0014067	negative regulation of phosphatidylinositol 3-kinase sig	2/385	16/23328	0.027984123	0.07613	0.04575	Slc9a3r2/	2
GO:0016322	GO:0016322	neuron remodeling	2/385	16/23328	0.027984123	0.07613	0.04575	Pdgfb/C3	2
GO:0018119	GO:0018119	peptidyl-cysteine S-nitrosylation	2/385	16/23328	0.027984123	0.07613	0.04575	S100a8/S	2
GO:0030322	GO:0030322	stabilization of membrane potential	2/385	16/23328	0.027984123	0.07613	0.04575	Kcnn4/Ch	2
GO:0043217	GO:0043217	myelin maintenance	2/385	16/23328	0.027984123	0.07613	0.04575	Clu/Ndrg1	2
GO:0044872	GO:0044872	lipoprotein localization	2/385	16/23328	0.027984123	0.07613	0.04575	Cd36/Lsr	2
GO:0045655	GO:0045655	regulation of monocyte differentiation	2/385	16/23328	0.027984123	0.07613	0.04575	Jun/Cd74	2
GO:0060026	GO:0060026	convergent extension	2/385	16/23328	0.027984123	0.07613	0.04575	Sfrp1/Sfrp	2
GO:0060391	GO:0060391	positive regulation of SMAD protein signal transductio	2/385	16/23328	0.027984123	0.07613	0.04575	Dab2/Bmp	2
GO:0060766	GO:0060766	negative regulation of androgen receptor signaling pat	2/385	16/23328	0.027984123	0.07613	0.04575	Sfrp1/Dab	2
GO:0061000	GO:0061000	negative regulation of dendritic spine development	2/385	16/23328	0.027984123	0.07613	0.04575	Plk2/Apoe	2
GO:0071731	GO:0071731	response to nitric oxide	2/385	16/23328	0.027984123	0.07613	0.04575	Aqp1/Thb	2
GO:0072567	GO:0072567	chemokine (C-X-C motif) ligand 2 production	2/385	16/23328	0.027984123	0.07613	0.04575	Lpl/Cd74	2
GO:0072673	GO:0072673	lamellipodium morphogenesis	2/385	16/23328	0.027984123	0.07613	0.04575	Pdpn/Kan	2

GO:0090083	GO:0090083	regulation of inclusion body assembly	2/385	16/23328	0.027984123	0.07613	0.04575	Clu/Apoe	2
GO:0090128	GO:0090128	regulation of synapse maturation	2/385	16/23328	0.027984123	0.07613	0.04575	Reln/Arhg	2
GO:0090141	GO:0090141	positive regulation of mitochondrial fission	2/385	16/23328	0.027984123	0.07613	0.04575	Kdr/Dcn	2
GO:1901550	GO:1901550	regulation of endothelial cell development	2/385	16/23328	0.027984123	0.07613	0.04575	Cdh5/Cldr	2
GO:1903140	GO:1903140	regulation of establishment of endothelial barrier	2/385	16/23328	0.027984123	0.07613	0.04575	Cdh5/Cldr	2
GO:1903779	GO:1903779	regulation of cardiac conduction	2/385	16/23328	0.027984123	0.07613	0.04575	Cav1/Gja5	2
GO:2000341	GO:2000341	regulation of chemokine (C-X-C motif) ligand 2 product	2/385	16/23328	0.027984123	0.07613	0.04575	Lpl/Cd74	2
GO:0016125	GO:0016125	sterol metabolic process	6/385	139/23328	0.028038361	0.07613	0.04575	Scarb1/Cy	6
GO:0003300	GO:0003300	cardiac muscle hypertrophy	5/385	103/23328	0.028080321	0.07613	0.04575	Rgs4/Ece	5
GO:0055024	GO:0055024	regulation of cardiac muscle tissue development	5/385	103/23328	0.028080321	0.07613	0.04575	Rgs4/Nca	5
GO:0001881	GO:0001881	receptor recycling	3/385	40/23328	0.028088956	0.07613	0.04575	Ece1/Anxa	3
GO:0006730	GO:0006730	one-carbon metabolic process	3/385	40/23328	0.028088956	0.07613	0.04575	Car4/Car8	3
GO:0014742	GO:0014742	positive regulation of muscle hypertrophy	3/385	40/23328	0.028088956	0.07613	0.04575	Ece1/Slc2	3
GO:0043551	GO:0043551	regulation of phosphatidylinositol 3-kinase activity	3/385	40/23328	0.028088956	0.07613	0.04575	Flt1/Pdgfb	3
GO:0071542	GO:0071542	dopaminergic neuron differentiation	3/385	40/23328	0.028088956	0.07613	0.04575	Cxcl12/Sfi	3
GO:0085029	GO:0085029	extracellular matrix assembly	3/385	40/23328	0.028088956	0.07613	0.04575	Rgcc/Fbln	3
GO:0097009	GO:0097009	energy homeostasis	3/385	40/23328	0.028088956	0.07613	0.04575	Cd36/C1q	3
GO:0017038	GO:0017038	protein import	8/385	217/23328	0.028173814	0.07627	0.04583	Cd36/Gpil	8
GO:0048639	GO:0048639	positive regulation of developmental growth	8/385	217/23328	0.028173814	0.07627	0.04583	Cxcl12/Fn	8
GO:0030888	GO:0030888	regulation of B cell proliferation	4/385	70/23328	0.028559017	0.07711	0.04633	Cd38/Cd2	4
GO:0043954	GO:0043954	cellular component maintenance	4/385	70/23328	0.028559017	0.07711	0.04633	Prnp/Chch	4
GO:0055117	GO:0055117	regulation of cardiac muscle contraction	4/385	70/23328	0.028559017	0.07711	0.04633	Cav1/Gja5	4
GO:1903317	GO:1903317	regulation of protein maturation	4/385	70/23328	0.028559017	0.07711	0.04633	Spon1/Se	4
GO:0042594	GO:0042594	response to starvation	7/385	178/23328	0.028724624	0.07746	0.04654	Atf3/Fstl1/	7
GO:0050821	GO:0050821	protein stabilization	7/385	178/23328	0.028724624	0.07746	0.04654	Gpihbp1/T	7
GO:0007189	GO:0007189	adenylate cyclase-activating G protein-coupled recepto	6/385	140/23328	0.028903015	0.07779	0.04674	Adgrl4/Pth	6
GO:0032946	GO:0032946	positive regulation of mononuclear cell proliferation	6/385	140/23328	0.028903015	0.07779	0.04674	Cd38/Cd2	6
GO:0050905	GO:0050905	neuromuscular process	6/385	140/23328	0.028903015	0.07779	0.04674	Tcf15/Cfh	6
GO:0051091	GO:0051091	positive regulation of DNA-binding transcription factor	9/385	260/23328	0.029358404	0.07896	0.04745	Rgcc/Cd3	9
GO:0007188	GO:0007188	adenylate cyclase-modulating G protein-coupled recep	8/385	219/23328	0.029526134	0.07935	0.04768	Adgrl4/Pth	8
GO:0031667	GO:0031667	response to nutrient levels	11/385	346/23328	0.029540708	0.07935	0.04768	Atf3/Fstl1/	11
GO:0043113	GO:0043113	receptor clustering	4/385	71/23328	0.029883469	0.08013	0.04815	Reln/Thy1	4
GO:0006953	GO:0006953	acute-phase response	3/385	41/23328	0.029945801	0.08013	0.04815	Fn1/Lbp/A	3
GO:0007431	GO:0007431	salivary gland development	3/385	41/23328	0.029945801	0.08013	0.04815	Pdgfb/Tgr	3
GO:0014037	GO:0014037	Schwann cell differentiation	3/385	41/23328	0.029945801	0.08013	0.04815	Ndrg1/Pm	3
GO:0051281	GO:0051281	positive regulation of release of sequestered calcium i	3/385	41/23328	0.029945801	0.08013	0.04815	Aplnr/Thy1	3
GO:0086009	GO:0086009	membrane repolarization	3/385	41/23328	0.029945801	0.08013	0.04815	Cav1/Gja5	3
GO:0001708	GO:0001708	cell fate specification	5/385	105/23328	0.030172172	0.08068	0.04848	Tbx1/Sox1	5
GO:2001056	GO:2001056	positive regulation of cysteine-type endopeptidase acti	6/385	142/23328	0.030684541	0.082	0.04927	Perp/Lgm	6
GO:0019058	GO:0019058	viral life cycle	8/385	221/23328	0.030922532	0.08243	0.04953	Rsad2/Bs	8
GO:1903039	GO:1903039	positive regulation of leukocyte cell-cell adhesion	8/385	221/23328	0.030922532	0.08243	0.04953	Thy1/Selp	8
GO:0010611	GO:0010611	regulation of cardiac muscle hypertrophy	4/385	72/23328	0.031243698	0.08243	0.04953	Rgs4/Ece	4
GO:0048002	GO:0048002	antigen processing and presentation of peptide antigen	4/385	72/23328	0.031243698	0.08243	0.04953	B2m/H2-D	4
GO:0006022	GO:0006022	aminoglycan metabolic process	5/385	106/23328	0.031253878	0.08243	0.04953	Bgn/Pdgfb	5
GO:0006165	GO:0006165	nucleoside diphosphate phosphorylation	5/385	106/23328	0.031253878	0.08243	0.04953	Nupr1/Ald	5
GO:0110110	GO:0110110	positive regulation of animal organ morphogenesis	5/385	106/23328	0.031253878	0.08243	0.04953	Cd34/Ptn/	5
GO:0001710	GO:0001710	mesodermal cell fate commitment	2/385	17/23328	0.031374843	0.08243	0.04953	Bmp4/Sfrp	2
GO:0006968	GO:0006968	cellular defense response	2/385	17/23328	0.031374843	0.08243	0.04953	B2m/Il33	2
GO:0007096	GO:0007096	regulation of exit from mitosis	2/385	17/23328	0.031374843	0.08243	0.04953	Rgcc/Cdk	2
GO:0010713	GO:0010713	negative regulation of collagen metabolic process	2/385	17/23328	0.031374843	0.08243	0.04953	Fn1/Cygb	2
GO:0010752	GO:0010752	regulation of cGMP-mediated signaling	2/385	17/23328	0.031374843	0.08243	0.04953	Thbs1/Pde	2
GO:0017014	GO:0017014	protein nitrosylation	2/385	17/23328	0.031374843	0.08243	0.04953	S100a8/S	2
GO:0021681	GO:0021681	cerebellar granular layer development	2/385	17/23328	0.031374843	0.08243	0.04953	Serpine2/I	2
GO:0032966	GO:0032966	negative regulation of collagen biosynthetic process	2/385	17/23328	0.031374843	0.08243	0.04953	Fn1/Cygb	2
GO:0034349	GO:0034349	glial cell apoptotic process	2/385	17/23328	0.031374843	0.08243	0.04953	Gas6/Pmp	2
GO:0040015	GO:0040015	negative regulation of multicellular organism growth	2/385	17/23328	0.031374843	0.08243	0.04953	Lgmn/H19	2
GO:0050665	GO:0050665	hydrogen peroxide biosynthetic process	2/385	17/23328	0.031374843	0.08243	0.04953	Duoxa2/C	2
GO:0051014	GO:0051014	actin filament severing	2/385	17/23328	0.031374843	0.08243	0.04953	Dstn/Gmfg	2
GO:0060973	GO:0060973	cell migration involved in heart development	2/385	17/23328	0.031374843	0.08243	0.04953	Eng/Bmp4	2
GO:0070206	GO:0070206	protein trimerization	2/385	17/23328	0.031374843	0.08243	0.04953	Steap4/Co	2
GO:0070293	GO:0070293	renal absorption	2/385	17/23328	0.031374843	0.08243	0.04953	Aqp1/Aqp	2
GO:0072010	GO:0072010	glomerular epithelium development	2/385	17/23328	0.031374843	0.08243	0.04953	Pdgfb/Bm	2
GO:1900119	GO:1900119	positive regulation of execution phase of apoptosis	2/385	17/23328	0.031374843	0.08243	0.04953	Igfbp3/Ptg	2
GO:1900221	GO:1900221	regulation of amyloid-beta clearance	2/385	17/23328	0.031374843	0.08243	0.04953	Clu/Apoe	2
GO:1902894	GO:1902894	negative regulation of pri-miRNA transcription by RNA	2/385	17/23328	0.031374843	0.08243	0.04953	Pdgfb/Bm	2
GO:2000369	GO:2000369	regulation of clathrin-dependent endocytosis	2/385	17/23328	0.031374843	0.08243	0.04953	Dab2/Sh3	2
GO:0030010	GO:0030010	establishment of cell polarity	6/385	143/23328	0.031601632	0.08287	0.04979	Tcf15/Cdh	6
GO:0030326	GO:0030326	embryonic limb morphogenesis	6/385	143/23328	0.031601632	0.08287	0.04979	Hoxd9/Ec	6
GO:0035113	GO:0035113	embryonic appendage morphogenesis	6/385	143/23328	0.031601632	0.08287	0.04979	Hoxd9/Ec	6
GO:0010762	GO:0010762	regulation of fibroblast migration	3/385	42/23328	0.031865846	0.08345	0.05015	Thbs1/Itgb	3
GO:0055023	GO:0055023	positive regulation of cardiac muscle tissue growth	3/385	42/23328	0.031865846	0.08345	0.05015	Ncam1/Sl	3
GO:0042130	GO:0042130	negative regulation of T cell proliferation	4/385	73/23328	0.032639814	0.08537	0.0513	Prnp/Cd24	4
GO:0043627	GO:0043627	response to estrogen	4/385	73/23328	0.032639814	0.08537	0.0513	Sfrp1/Cd2	4
GO:1903708	GO:1903708	positive regulation of hemopoiesis	8/385	224/23328	0.033101015	0.08653	0.05199	Flt1/Kitl/Fo	8
GO:0006119	GO:0006119	oxidative phosphorylation	5/385	108/23328	0.033489485	0.08732	0.05247	Cox4i2/Nu	5
GO:0045639	GO:0045639	positive regulation of myeloid cell differentiation	5/385	108/23328	0.033489485	0.08732	0.05247	Kitl/Fos/Ju	5
GO:0046939	GO:0046939	nucleotide phosphorylation	5/385	108/23328	0.033489485	0.08732	0.05247	Nupr1/Ald	5
GO:0055017	GO:0055017	cardiac muscle tissue growth	5/385	108/23328	0.033489485	0.08732	0.05247	Rgs4/Nca	5
GO:0003298	GO:0003298	physiological muscle hypertrophy	3/385	43/23328	0.033848821	0.08782	0.05277	Rgs4/Slc2	3
GO:0003301	GO:0003301	physiological cardiac muscle hypertrophy	3/385	43/23328	0.033848821	0.08782	0.05277	Rgs4/Slc2	3
GO:0032008	GO:0032008	positive regulation of TOR signaling	3/385	43/23328	0.033848821	0.08782	0.05277	Reln/Kdr/C	3
GO:0038083	GO:0038083	peptidyl-tyrosine autophosphorylation	3/385	43/23328	0.033848821	0.08782	0.05277	Kdr/Bmx/C	3
GO:0042304	GO:0042304	regulation of fatty acid biosynthetic process	3/385	43/23328	0.033848821	0.08782	0.05277	Fabp5/Cd	3
GO:0042771	GO:0042771	intrinsic apoptotic signaling pathway in response to DN	3/385	43/23328	0.033848821	0.08782	0.05277	Nupr1/Cdk	3
GO:0061049	GO:0061049	cell growth involved in cardiac muscle cell developmer	3/385	43/23328	0.033848821	0.08782	0.05277	Rgs4/Slc2	3
GO:1903792	GO:1903792	negative regulation of anion transport	3/385	43/23328	0.033848821	0.08782	0.05277	Rgs4/Thbs	3
GO:0048588	GO:0048588	developmental cell growth	9/385	267/23328	0.033933912	0.08799	0.05287	Mgll/Cxcl1	9
GO:0007422	GO:0007422	peripheral nervous system development	4/385	74/23328	0.034071909	0.08824	0.05302	Hoxd9/Nd	4
GO:2001021	GO:2001021	negative regulation of response to DNA damage stimu	4/385	74/23328	0.034071909	0.08824	0.05302	Cxcl12/Clu	4
GO:0009895	GO:0009895	negative regulation of catabolic process	10/385	311/23328	0.03459321	0.08916	0.05358	Nupr1/Tim	10
GO:1900006	GO:1900006	positive regulation of dendrite development	5/385	109/23328	0.034643616	0.08916	0.05358	Reln/Ptn/A	5
GO:0000188	GO:0000188	inactivation of MAPK activity	2/385	18/23328	0.034918291	0.08916	0.05358	Dusp1/Ca	2
GO:0001991	GO:0001991	regulation of systemic arterial blood pressure by circula	2/385	18/23328	0.034918291	0.08916	0.05358	Gja5/F2r	2
GO:0002281	GO:0002281	macrophage activation involved in immune response	2/385	18/23328	0.034918291	0.08916	0.05358	Il33/Lbp	2
GO:0003214	GO:0003214	cardiac left ventricle morphogenesis	2/385	18/23328	0.034918291	0.08916	0.05358	Cpe/Sfrp2	2
GO:0006670	GO:0006670	sphingosine metabolic process	2/385	18/23328	0.034918291	0.08916	0.05358	Plpp3/Ace	2
GO:0006700	GO:0006700	C21-steroid hormone biosynthetic process	2/385	18/23328	0.034918291	0.08916	0.05358	Egr1/Dab2	2
GO:0006833	GO:0006833	water transport	2/385	18/23328	0.034918291	0.08916	0.05358	Aqp1/Aqp	2
GO:0009886	GO:0009886	post-embryonic animal morphogenesis	2/385	18/23328	0.034918291	0.08916	0.05358	Flt1/Kdr	2
GO:0030033	GO:0030033	microvillus assembly	2/385	18/23328	0.034918291	0.08916	0.05358	Fscn1/Poo	2
GO:0032793	GO:0032793	positive regulation of CREB transcription factor activity	2/385	18/23328	0.034918291	0.08916	0.05358	Reln/Cd20	2
GO:0035633	GO:0035633	maintenance of blood-brain barrier	2/385	18/23328	0.034918291	0.08916	0.05358	Cldn5/Ptg	2
GO:0042659	GO:0042659	regulation of cell fate specification	2/385	18/23328	0.034918291	0.08916	0.05358	Sox17/Sfr	2
GO:0044241	GO:0044241	lipid digestion	2/385	18/23328	0.034918291	0.08916	0.05358	Cd36/Aqp	2
GO:0044406	GO:0044406	adhesion of symbiont to host	2/385	18/23328	0.034918291	0.08916	0.05358	Scarb1/Ga	2
GO:0050710	GO:0050710	negative regulation of cytokine secretion	2/385	18/23328	0.034918291	0.08916	0.05358	Rgcc/Srgr	2
GO:0060572	GO:0060572	morphogenesis of an epithelial bud	2/385	18/23328	0.034918291	0.08916	0.05358	Pthlh/Bmp	2
GO:0060841	GO:0060841	venous blood vessel development	2/385	18/23328	0.034918291	0.08916	0.05358	Eng/Prox1	2
GO:0070571	GO:0070571	negative regulation of neuron projection regeneration	2/385	18/23328	0.034918291	0.08916	0.05358	Thy1/Klk8	2
GO:1900153	GO:1900153	positive regulation of nuclear-transcribed mRNA catab	2/385	18/23328	0.034918291	0.08916	0.05358	Btg2/Zfp3	2
GO:1903019	GO:1903019	negative regulation of glycoprotein metabolic process	2/385	18/23328	0.034918291	0.08916	0.05358	Bace2/Ac	2
GO:1904706	GO:1904706	negative regulation of vascular associated smooth mu	2/385	18/23328	0.034918291	0.08916	0.05358	Cdkn1a/R	2
GO:0002824	GO:0002824	positive regulation of adaptive immune response base	6/385	147/23328	0.035447923	0.0904	0.05432	Rsad2/Cd	6
GO:0035270	GO:0035270	endocrine system development	6/385	147/23328	0.035447923	0.0904	0.05432	Tbx1/Ly6e	6
GO:0032642	GO:0032642	regulation of chemokine production	4/385	75/23328	0.035540061	0.09042	0.05433	Lpl/Cd24a	4
GO:0046626	GO:0046626	regulation of insulin receptor signaling pathway	4/385	75/23328	0.035540061	0.09042	0.05433	Socs3/Ptp	4
GO:0048747	GO:0048747	muscle fiber development	4/385	75/23328	0.035540061	0.09042	0.05433	Lox/Pmp2	4
GO:0097755	GO:0097755	positive regulation of blood vessel diameter	4/385	75/23328	0.035540061	0.09042	0.05433	Kdr/Apoe/	4
GO:0002526	GO:0002526	acute inflammatory response	5/385	110/23328	0.035822105	0.09093	0.05464	Nupr1/Fn1	5
GO:0007156	GO:0007156	homophilic cell adhesion via plasma membrane adhes	5/385	110/23328	0.035822105	0.09093	0.05464	Cdh5/Esa	5
GO:0010972	GO:0010972	negative regulation of G2/M transition of mitotic cell cy	3/385	44/23328	0.035894408	0.09093	0.05464	Fhl1/Cdkn	3
GO:0032350	GO:0032350	regulation of hormone metabolic process	3/385	44/23328	0.035894408	0.09093	0.05464	Duoxa2/E	3
GO:0033628	GO:0033628	regulation of cell adhesion mediated by integrin	3/385	44/23328	0.035894408	0.09093	0.05464	Cd24a/Ac	3
GO:0042462	GO:0042462	eye photoreceptor cell development	3/385	44/23328	0.035894408	0.09093	0.05464	Thy1/Cfh/	3
GO:0048821	GO:0048821	erythrocyte development	3/385	44/23328	0.035894408	0.09093	0.05464	Adgrf5/Hb	3
GO:0006909	GO:0006909	phagocytosis	11/385	358/23328	0.036436283	0.09225	0.05543	Cd36/Sca	11
GO:0010770	GO:0010770	positive regulation of cell morphogenesis involved in d	7/385	188/23328	0.036977814	0.0935	0.05619	Reln/Cxcl	7
GO:0001676	GO:0001676	long-chain fatty acid metabolic process	5/385	111/23328	0.03702505	0.0935	0.05619	Cd36/Mgll	5
GO:0008637	GO:0008637	apoptotic mitochondrial changes	5/385	111/23328	0.03702505	0.0935	0.05619	Cd24a/Slc	5

GO:0030316	GO:0030316	osteoclast differentiation	5/385	111/23328	0.03702505	0.0935	0.05619	Sfrp1/Fos	5
GO:0006919	GO:0006919	activation of cysteine-type endopeptidase activity invol	4/385	76/23328	0.037044327	0.0935	0.05619	Acer2/F2r	4
GO:0002221	GO:0002221	pattern recognition receptor signaling pathway	6/385	149/23328	0.037479145	0.09446	0.05676	Cd36/Irf7/	6
GO:0046660	GO:0046660	female sex differentiation	6/385	149/23328	0.037479145	0.09446	0.05676	Sfrp1/Nup	6
GO:0031098	GO:0031098	stress-activated protein kinase signaling cascade	9/385	272/23328	0.037489039	0.09446	0.05676	Sfrp1/Dus	9
GO:0043161	GO:0043161	proteasome-mediated ubiquitin-dependent protein cata	12/385	405/23328	0.037630654	0.09476	0.05694	Plk2/Clu/H	12
GO:0045786	GO:0045786	negative regulation of cell cycle	13/385	451/23328	0.037756606	0.09493	0.05704	Rgcc/Nup	13
GO:0001961	GO:0001961	positive regulation of cytokine-mediated signaling path	3/385	45/23328	0.038002246	0.09493	0.05704	Irf7/Gas6/	3
GO:0030890	GO:0030890	positive regulation of B cell proliferation	3/385	45/23328	0.038002246	0.09493	0.05704	Cd38/Cdk	3
GO:0032873	GO:0032873	negative regulation of stress-activated MAPK cascade	3/385	45/23328	0.038002246	0.09493	0.05704	Sfrp1/Dus	3
GO:0042181	GO:0042181	ketone biosynthetic process	3/385	45/23328	0.038002246	0.09493	0.05704	Egr1/Aldo	3
GO:0050856	GO:0050856	regulation of T cell receptor signaling pathway	3/385	45/23328	0.038002246	0.09493	0.05704	Thy1/Prnp	3
GO:0070303	GO:0070303	negative regulation of stress-activated protein kinase s	3/385	45/23328	0.038002246	0.09493	0.05704	Sfrp1/Dus	3
GO:0071300	GO:0071300	cellular response to retinoic acid	3/385	45/23328	0.038002246	0.09493	0.05704	Tbx1/Aqp	3
GO:0089718	GO:0089718	amino acid import across plasma membrane	3/385	45/23328	0.038002246	0.09493	0.05704	Rgs4/Itgb	3
GO:2000826	GO:2000826	regulation of heart morphogenesis	3/385	45/23328	0.038002246	0.09493	0.05704	Tbx1/Eng	3
GO:0007589	GO:0007589	body fluid secretion	5/385	112/23328	0.038252539	0.09493	0.05704	Kcnn4/Aq	5
GO:0001756	GO:0001756	somitogenesis	4/385	77/23328	0.038584752	0.09493	0.05704	Tcf15/Sfrp	4
GO:0003407	GO:0003407	neural retina development	4/385	77/23328	0.038584752	0.09493	0.05704	Thy1/Cfh/	4
GO:0045453	GO:0045453	bone resorption	4/385	77/23328	0.038584752	0.09493	0.05704	Cd38/S1p	4
GO:0002274	GO:0002274	myeloid leukocyte activation	8/385	231/23328	0.038584944	0.09493	0.05704	Adgrf5/Cd	8
GO:0046651	GO:0046651	lymphocyte proliferation	10/385	317/23328	0.038588251	0.09493	0.05704	Cxcl12/Pr	10
GO:0002031	GO:0002031	G protein-coupled receptor internalization	2/385	19/23328	0.038608531	0.09493	0.05704	Adm/Htr2l	2
GO:0002726	GO:0002726	positive regulation of T cell cytokine production	2/385	19/23328	0.038608531	0.09493	0.05704	Rsad2/B2	2
GO:0003180	GO:0003180	aortic valve morphogenesis	2/385	19/23328	0.038608531	0.09493	0.05704	Bmp4/Nos	2
GO:0006027	GO:0006027	glycosaminoglycan catabolic process	2/385	19/23328	0.038608531	0.09493	0.05704	Lyve1/Pgl	2
GO:0006525	GO:0006525	arginine metabolic process	2/385	19/23328	0.038608531	0.09493	0.05704	Nos3/Ass	2
GO:0010566	GO:0010566	regulation of ketone biosynthetic process	2/385	19/23328	0.038608531	0.09493	0.05704	Egr1/Dab	2
GO:0032352	GO:0032352	positive regulation of hormone metabolic process	2/385	19/23328	0.038608531	0.09493	0.05704	Egr1/Dab	2
GO:0032823	GO:0032823	regulation of natural killer cell differentiation	2/385	19/23328	0.038608531	0.09493	0.05704	Gas6/Pgly	2
GO:0044539	GO:0044539	long-chain fatty acid import into cell	2/385	19/23328	0.038608531	0.09493	0.05704	Cd36/Thb	2
GO:0055003	GO:0055003	cardiac myofibril assembly	2/385	19/23328	0.038608531	0.09493	0.05704	Prox1/Act	2
GO:0060033	GO:0060033	anatomical structure regression	2/385	19/23328	0.038608531	0.09493	0.05704	Flt1/Ninj1	2
GO:0060221	GO:0060221	retinal rod cell differentiation	2/385	19/23328	0.038608531	0.09493	0.05704	Cfh/Ptn	2
GO:0060231	GO:0060231	mesenchymal to epithelial transition	2/385	19/23328	0.038608531	0.09493	0.05704	Tcf15/Bm	2
GO:0060544	GO:0060544	regulation of necroptotic process	2/385	19/23328	0.038608531	0.09493	0.05704	Slc25a4/C	2
GO:0060707	GO:0060707	trophoblast giant cell differentiation	2/385	19/23328	0.038608531	0.09493	0.05704	Socs3/Nr2	2
GO:0060712	GO:0060712	spongiotrophoblast layer development	2/385	19/23328	0.038608531	0.09493	0.05704	Socs3/Adı	2
GO:0061081	GO:0061081	positive regulation of myeloid leukocyte cytokine produ	2/385	19/23328	0.038608531	0.09493	0.05704	Cd36/Cd7	2
GO:0062098	GO:0062098	regulation of programmed necrotic cell death	2/385	19/23328	0.038608531	0.09493	0.05704	Slc25a4/C	2
GO:0072578	GO:0072578	neurotransmitter-gated ion channel clustering	2/385	19/23328	0.038608531	0.09493	0.05704	Reln/Apoe	2
GO:0140354	GO:0140354	lipid import into cell	2/385	19/23328	0.038608531	0.09493	0.05704	Cd36/Thb	2
GO:1903960	GO:1903960	negative regulation of anion transmembrane transport	2/385	19/23328	0.038608531	0.09493	0.05704	Rgs4/Thb	2
GO:1904754	GO:1904754	positive regulation of vascular associated smooth mus	2/385	19/23328	0.038608531	0.09493	0.05704	Igfbp5/Pd	2
GO:1905276	GO:1905276	regulation of epithelial tube formation	2/385	19/23328	0.038608531	0.09493	0.05704	Sfrp1/Sfrp	2
GO:2000269	GO:2000269	regulation of fibroblast apoptotic process	2/385	19/23328	0.038608531	0.09493	0.05704	Sfrp1/Gas	2
GO:0032872	GO:0032872	regulation of stress-activated MAPK cascade	8/385	232/23328	0.039415082	0.09686	0.0582	Sfrp1/Dus	8
GO:0030516	GO:0030516	regulation of axon extension	5/385	113/23328	0.039504656	0.09702	0.0583	Mgll/Cxcl	5
GO:0050830	GO:0050830	defense response to Gram-positive bacterium	6/385	151/23328	0.039583336	0.09716	0.05838	Cd36/B2m	6
GO:0051783	GO:0051783	regulation of nuclear division	7/385	191/23328	0.03972908	0.09746	0.05856	Rgcc/Pde	7
GO:0060042	GO:0060042	retina morphogenesis in camera-type eye	4/385	78/23328	0.04016136	0.09826	0.05904	Thy1/Cfh/	4
GO:0072332	GO:0072332	intrinsic apoptotic signaling pathway by p53 class med	4/385	78/23328	0.04016136	0.09826	0.05904	Perp/Nupr	4
GO:0010259	GO:0010259	multicellular organism aging	3/385	46/23328	0.040171931	0.09826	0.05904	Cfh/Pmp2	3
GO:0098926	GO:0098926	postsynaptic signal transduction	3/385	46/23328	0.040171931	0.09826	0.05904	Ly6c1/Ly6	3
GO:1905521	GO:1905521	regulation of macrophage migration	3/385	46/23328	0.040171931	0.09826	0.05904	Cd9/Cd20	3
GO:0032943	GO:0032943	mononuclear cell proliferation	10/385	320/23328	0.040700635	0.09949	0.05978	Cxcl12/Pr	10
GO:0032649	GO:0032649	regulation of interferon-gamma production	5/385	114/23328	0.040781474	0.09963	0.05987	Prnp/Il33/	5
GO:0048167	GO:0048167	regulation of synaptic plasticity	11/385	365/23328	0.040943796	0.09997	0.06007	Mgll/Reln/	11
GO:0032602	GO:0032602	chemokine production	4/385	79/23328	0.041774164	0.1017	0.06111	Lpl/Cd24a	4
GO:0051781	GO:0051781	positive regulation of cell division	4/385	79/23328	0.041774164	0.1017	0.06111	Pdgfb/Ptn	4
GO:0055021	GO:0055021	regulation of cardiac muscle tissue growth	4/385	79/23328	0.041774164	0.1017	0.06111	Rgs4/Nca	4
GO:0060191	GO:0060191	regulation of lipase activity	4/385	79/23328	0.041774164	0.1017	0.06111	Flt1/Gpihb	4
GO:1902117	GO:1902117	positive regulation of organelle assembly	4/385	79/23328	0.041774164	0.1017	0.06111	Fscn1/Pro	4
GO:0070302	GO:0070302	regulation of stress-activated protein kinase signaling	8/385	235/23328	0.041976839	0.10202	0.0613	Sfrp1/Dus	8
GO:0001776	GO:0001776	leukocyte homeostasis	5/385	115/23328	0.042083061	0.10202	0.0613	Cfh/Kitl/C	5
GO:0019079	GO:0019079	viral genome replication	5/385	115/23328	0.042083061	0.10202	0.0613	Rsad2/Bs	5
GO:0001941	GO:0001941	postsynaptic membrane organization	3/385	47/23328	0.042403021	0.10202	0.0613	Reln/Ptn/	3
GO:0003179	GO:0003179	heart valve morphogenesis	3/385	47/23328	0.042403021	0.10202	0.0613	Bmp4/Gja	3
GO:0035458	GO:0035458	cellular response to interferon-beta	3/385	47/23328	0.042403021	0.10202	0.0613	Mndal/Ifi2	3
GO:0042088	GO:0042088	T-helper 1 type immune response	3/385	47/23328	0.042403021	0.10202	0.0613	Il33/H2-Al	3
GO:0006883	GO:0006883	cellular sodium ion homeostasis	2/385	20/23328	0.04243978	0.10202	0.0613	Sgk1/Atp1	2
GO:0008209	GO:0008209	androgen metabolic process	2/385	20/23328	0.04243978	0.10202	0.0613	Scarb1/Ac	2
GO:0010763	GO:0010763	positive regulation of fibroblast migration	2/385	20/23328	0.04243978	0.10202	0.0613	Thbs1/Itgl	2
GO:0010984	GO:0010984	regulation of lipoprotein particle clearance	2/385	20/23328	0.04243978	0.10202	0.0613	Gpihbp1/	2
GO:0032495	GO:0032495	response to muramyl dipeptide	2/385	20/23328	0.04243978	0.10202	0.0613	Vim/Nfkbi	2
GO:0046514	GO:0046514	ceramide catabolic process	2/385	20/23328	0.04243978	0.10202	0.0613	Gm2a/Ace	2
GO:0046519	GO:0046519	sphingoid metabolic process	2/385	20/23328	0.04243978	0.10202	0.0613	Plpp3/Ace	2
GO:0046851	GO:0046851	negative regulation of bone remodeling	2/385	20/23328	0.04243978	0.10202	0.0613	Sfrp1/Cd3	2
GO:0060547	GO:0060547	negative regulation of necrotic cell death	2/385	20/23328	0.04243978	0.10202	0.0613	Slc25a4/C	2
GO:0072189	GO:0072189	ureter development	2/385	20/23328	0.04243978	0.10202	0.0613	Sox17/Bm	2
GO:0072243	GO:0072243	metanephric nephron epithelium development	2/385	20/23328	0.04243978	0.10202	0.0613	Pdgfb/Aqp	2
GO:1900151	GO:1900151	regulation of nuclear-transcribed mRNA catabolic proc	2/385	20/23328	0.04243978	0.10202	0.0613	Btg2/Zfp3	2
GO:1901028	GO:1901028	regulation of mitochondrial outer membrane permeabil	2/385	20/23328	0.04243978	0.10202	0.0613	Chchd10/	2
GO:2000114	GO:2000114	regulation of establishment of cell polarity	2/385	20/23328	0.04243978	0.10202	0.0613	Cdh5/Kan	2
GO:2000136	GO:2000136	regulation of cell proliferation involved in heart morpho	2/385	20/23328	0.04243978	0.10202	0.0613	Tbx1/Bmp	2
GO:0021915	GO:0021915	neural tube development	7/385	194/23328	0.042610823	0.10237	0.06151	Sfrp1/Sox	7
GO:0045930	GO:0045930	negative regulation of mitotic cell cycle	8/385	236/23328	0.042854717	0.10289	0.06183	Rgcc/Btg	8
GO:0014013	GO:0014013	regulation of gliogenesis	6/385	154/23328	0.042877758	0.10289	0.06183	Vim/Ptn/S	6
GO:0048041	GO:0048041	focal adhesion assembly	4/385	80/23328	0.043423157	0.10408	0.06254	Thy1/Kdr/	4
GO:1900542	GO:1900542	regulation of purine nucleotide metabolic process	4/385	80/23328	0.043423157	0.10408	0.06254	Nupr1/Ier	4
GO:0001838	GO:0001838	embryonic epithelial tube formation	6/385	155/23328	0.044013007	0.10543	0.06335	Sfrp1/Spir	6
GO:0001774	GO:0001774	microglial cell activation	3/385	48/23328	0.044695035	0.10646	0.06397	Clu/Il33/J	3
GO:0002269	GO:0002269	leukocyte activation involved in inflammatory response	3/385	48/23328	0.044695035	0.10646	0.06397	Clu/Il33/J	3
GO:0006110	GO:0006110	regulation of glycolytic process	3/385	48/23328	0.044695035	0.10646	0.06397	Nupr1/Ier	3
GO:0010862	GO:0010862	positive regulation of pathway-restricted SMAD protein	3/385	48/23328	0.044695035	0.10646	0.06397	Eng/Dab2	3
GO:0031111	GO:0031111	negative regulation of microtubule polymerization or de	3/385	48/23328	0.044695035	0.10646	0.06397	Cdh5/Stm	3
GO:0045429	GO:0045429	positive regulation of nitric oxide biosynthetic process	3/385	48/23328	0.044695035	0.10646	0.06397	Cd36/Clu/	3
GO:0050850	GO:0050850	positive regulation of calcium-mediated signaling	3/385	48/23328	0.044695035	0.10646	0.06397	Kdr/Cd24	3
GO:0090279	GO:0090279	regulation of calcium ion import	3/385	48/23328	0.044695035	0.10646	0.06397	Cxcl12/Pr	3
GO:1902622	GO:1902622	regulation of neutrophil migration	3/385	48/23328	0.044695035	0.10646	0.06397	Cd74/Lbp	3
GO:1903115	GO:1903115	regulation of actin filament-based movement	3/385	48/23328	0.044695035	0.10646	0.06397	Pdpn/Cav	3
GO:1901890	GO:1901890	positive regulation of cell junction assembly	5/385	117/23328	0.044760775	0.10656	0.06403	Thy1/Kdr/	5
GO:0006140	GO:0006140	regulation of nucleotide metabolic process	4/385	81/23328	0.045108319	0.10726	0.06445	Nupr1/Ier	4
GO:0043525	GO:0043525	positive regulation of neuron apoptotic process	4/385	81/23328	0.045108319	0.10726	0.06445	Prnp/Egr1	4
GO:0050773	GO:0050773	regulation of dendrite development	7/385	197/23328	0.045624674	0.10843	0.06515	Reln/Id1/F	7
GO:0021700	GO:0021700	developmental maturation	10/385	327/23328	0.045934863	0.1091	0.06556	Reln/Cdh5	10
GO:0002863	GO:0002863	positive regulation of inflammatory response to antiger	2/385	21/23328	0.046406405	0.10929	0.06567	Cd24a/C3	2
GO:0003417	GO:0003417	growth plate cartilage development	2/385	21/23328	0.046406405	0.10929	0.06567	Thbs1/Co	2
GO:0010039	GO:0010039	response to iron ion	2/385	21/23328	0.046406405	0.10929	0.06567	B2m/Trf	2
GO:0033630	GO:0033630	positive regulation of cell adhesion mediated by integri	2/385	21/23328	0.046406405	0.10929	0.06567	Cd24a/Po	2
GO:0035089	GO:0035089	establishment of apical/basal cell polarity	2/385	21/23328	0.046406405	0.10929	0.06567	Tcf15/Fsc	2
GO:0035357	GO:0035357	peroxisome proliferator activated receptor signaling pa	2/385	21/23328	0.046406405	0.10929	0.06567	Fabp5/Ptg	2
GO:0044346	GO:0044346	fibroblast apoptotic process	2/385	21/23328	0.046406405	0.10929	0.06567	Sfrp1/Gas	2
GO:0046716	GO:0046716	muscle cell cellular homeostasis	2/385	21/23328	0.046406405	0.10929	0.06567	Lox/Aldoa	2
GO:0048569	GO:0048569	post-embryonic animal organ development	2/385	21/23328	0.046406405	0.10929	0.06567	Flt1/Kdr	2
GO:0051900	GO:0051900	regulation of mitochondrial depolarization	2/385	21/23328	0.046406405	0.10929	0.06567	Kdr/Dcn	2
GO:0061323	GO:0061323	cell proliferation involved in heart morphogenesis	2/385	21/23328	0.046406405	0.10929	0.06567	Tbx1/Bmp	2
GO:0090026	GO:0090026	positive regulation of monocyte chemotaxis	2/385	21/23328	0.046406405	0.10929	0.06567	Cxcl12/Lg	2
GO:0097734	GO:0097734	extracellular exosome biogenesis	2/385	21/23328	0.046406405	0.10929	0.06567	Cd34/Sdc	2
GO:1902004	GO:1902004	positive regulation of amyloid-beta formation	2/385	21/23328	0.046406405	0.10929	0.06567	Clu/Apoe	2
GO:2000209	GO:2000209	regulation of anoikis	2/385	21/23328	0.046406405	0.10929	0.06567	Cav1/Itgb	2
GO:0048562	GO:0048562	embryonic organ morphogenesis	10/385	328/23328	0.046718053	0.10996	0.06608	Tbx1/Hox	10
GO:0050805	GO:0050805	negative regulation of synaptic transmission	4/385	82/23328	0.046829615	0.11016	0.0662	Mgll/Pcdh	4
GO:0043090	GO:0043090	amino acid import	3/385	49/23328	0.047047458	0.11043	0.06636	Rgs4/Itgb	3
GO:0043550	GO:0043550	regulation of lipid kinase activity	3/385	49/23328	0.047047458	0.11043	0.06636	Flt1/Pdgfb	3
GO:1902808	GO:1902808	positive regulation of cell cycle G1/S phase transition	3/385	49/23328	0.047047458	0.11043	0.06636	Rgcc/Cyp	3
GO:1904407	GO:1904407	positive regulation of nitric oxide metabolic process	3/385	49/23328	0.047047458	0.11043	0.06636	Cd36/Clu/	3
GO:0006644	GO:0006644	phospholipid metabolic process	11/385	374/23328	0.047288775	0.11093	0.06666	Flt1/Scarb	11
GO:0050777	GO:0050777	negative regulation of immune response	6/385	158/23328	0.047530902	0.11144	0.06696	Fgl2/Il33/	6

GO:0050714	GO:0050714	positive regulation of protein secretion	7/385	199/23328	0.047708023	0.11179	0.06717	Rgcc/Cd3	7
GO:1903901	GO:1903901	negative regulation of viral life cycle	4/385	83/23328	0.048586993	0.11379	0.06837	Rsad2/Bst	4
GO:0002700	GO:0002700	regulation of production of molecular mediator of immu	6/385	159/23328	0.048741088	0.11396	0.06848	Cd36/Rsa	6
GO:0007569	GO:0007569	cell aging	6/385	159/23328	0.048741088	0.11396	0.06848	Plk2/B2m	6
GO:0009266	GO:0009266	response to temperature stimulus	6/385	159/23328	0.048741088	0.11396	0.06848	Cxcl12/Cc	6
GO:0050870	GO:0050870	positive regulation of T cell activation	7/385	200/23328	0.048772078	0.11396	0.06848	Thy1/Cd24	7
GO:0010677	GO:0010677	negative regulation of cellular carbohydrate metabolic	3/385	50/23328	0.049459738	0.11544	0.06937	Nupr1/Ier3	3
GO:0032715	GO:0032715	negative regulation of interleukin-6 production	3/385	50/23328	0.049459738	0.11544	0.06937	Cd200/Cd	3
GO:0031647	GO:0031647	regulation of protein stability	9/385	287/23328	0.049656663	0.11584	0.06961	Gpihbp1/T	9
GO:0070507	GO:0070507	regulation of microtubule cytoskeleton organization	7/385	201/23328	0.049851114	0.11623	0.06984	Cdh5/Plk2	7
GO:0007088	GO:0007088	regulation of mitotic nuclear division	6/385	160/23328	0.049970126	0.11644	0.06997	Rgcc/Cdk	6
GO:0006942	GO:0006942	regulation of striated muscle contraction	4/385	84/23328	0.050380387	0.11677	0.07017	Cav1/Gja5	4
GO:0043154	GO:0043154	negative regulation of cysteine-type endopeptidase act	4/385	84/23328	0.050380387	0.11677	0.07017	Aqp1/Thbs	4
GO:0046530	GO:0046530	photoreceptor cell differentiation	4/385	84/23328	0.050380387	0.11677	0.07017	Thy1/Cfh/	4
GO:0061515	GO:0061515	myeloid cell development	4/385	84/23328	0.050380387	0.11677	0.07017	Adgrf5/An	4
GO:0046620	GO:0046620	regulation of organ growth	5/385	121/23328	0.050415592	0.11677	0.07017	Rgs4/Nca	5
GO:0050829	GO:0050829	defense response to Gram-negative bacterium	5/385	121/23328	0.050415592	0.11677	0.07017	B2m/Iigp1	5
GO:0010499	GO:0010499	proteasomal ubiquitin-independent protein catabolic pr	2/385	22/23328	0.050502919	0.11677	0.07017	Psmb8/Ps	2
GO:0010666	GO:0010666	positive regulation of cardiac muscle cell apoptotic pro	2/385	22/23328	0.050502919	0.11677	0.07017	Fndc1/Igfl	2
GO:0014912	GO:0014912	negative regulation of smooth muscle cell migration	2/385	22/23328	0.050502919	0.11677	0.07017	Igfbp5/Igfl	2
GO:0032148	GO:0032148	activation of protein kinase B activity	2/385	22/23328	0.050502919	0.11677	0.07017	Pdgfb/Gas	2
GO:0035634	GO:0035634	response to stilbenoid	2/385	22/23328	0.050502919	0.11677	0.07017	Cd36/Hba	2
GO:0050860	GO:0050860	negative regulation of T cell receptor signaling pathwa	2/385	22/23328	0.050502919	0.11677	0.07017	Thy1/Prnp	2
GO:0051770	GO:0051770	positive regulation of nitric-oxide synthase biosynthetic	2/385	22/23328	0.050502919	0.11677	0.07017	Kdr/Il33	2
GO:0060602	GO:0060602	branch elongation of an epithelium	2/385	22/23328	0.050502919	0.11677	0.07017	Tnc/Bmp4	2
GO:0006914	GO:0006914	autophagy	12/385	425/23328	0.050841438	0.11742	0.07056	Nupr1/Kdr	12
GO:0061919	GO:0061919	process utilizing autophagic mechanism	12/385	425/23328	0.050841438	0.11742	0.07056	Nupr1/Kdr	12
GO:0031648	GO:0031648	protein destabilization	3/385	51/23328	0.051931295	0.11961	0.07187	Id1/Prnp/S	3
GO:0031663	GO:0031663	lipopolysaccharide-mediated signaling pathway	3/385	51/23328	0.051931295	0.11961	0.07187	Nfkbia/No	3
GO:0032330	GO:0032330	regulation of chondrocyte differentiation	3/385	51/23328	0.051931295	0.11961	0.07187	Pthlh/Bmp	3
GO:0032722	GO:0032722	positive regulation of chemokine production	3/385	51/23328	0.051931295	0.11961	0.07187	Lpl/Cd74/I	3
GO:0060760	GO:0060760	positive regulation of response to cytokine stimulus	3/385	51/23328	0.051931295	0.11961	0.07187	Irf7/Gas6/	3
GO:0072175	GO:0072175	epithelial tube formation	6/385	162/23328	0.052484916	0.12082	0.0726	Sfrp1/Spin	6
GO:0009132	GO:0009132	nucleoside diphosphate metabolic process	5/385	123/23328	0.053393141	0.12284	0.07382	Nupr1/Ald	5
GO:0002244	GO:0002244	hematopoietic progenitor cell differentiation	6/385	163/23328	0.053770734	0.12364	0.0743	Flt1/Sfrp1	6
GO:0045933	GO:0045933	positive regulation of muscle contraction	3/385	52/23328	0.054461514	0.12481	0.075	F2r/Edn1/	3
GO:0140353	GO:0140353	lipid export from cell	3/385	52/23328	0.054461514	0.12481	0.075	Igfbp3/Da	3
GO:0002827	GO:0002827	positive regulation of T-helper 1 type immune response	2/385	23/23328	0.054723979	0.12481	0.075	H2-Ab1/A	2
GO:0003176	GO:0003176	aortic valve development	2/385	23/23328	0.054723979	0.12481	0.075	Bmp4/Nos	2
GO:0006907	GO:0006907	pinocytosis	2/385	23/23328	0.054723979	0.12481	0.075	Cav1/Dab	2
GO:0010663	GO:0010663	positive regulation of striated muscle cell apoptotic pro	2/385	23/23328	0.054723979	0.12481	0.075	Fndc1/Igfl	2
GO:0032331	GO:0032331	negative regulation of chondrocyte differentiation	2/385	23/23328	0.054723979	0.12481	0.075	Pthlh/Bmp	2
GO:0045056	GO:0045056	transcytosis	2/385	23/23328	0.054723979	0.12481	0.075	Gpihbp1/C	2
GO:0048333	GO:0048333	mesodermal cell differentiation	2/385	23/23328	0.054723979	0.12481	0.075	Bmp4/Sfrp	2
GO:0061162	GO:0061162	establishment of monopolar cell polarity	2/385	23/23328	0.054723979	0.12481	0.075	Tcf15/Fsc	2
GO:0072202	GO:0072202	cell differentiation involved in metanephros developme	2/385	23/23328	0.054723979	0.12481	0.075	Pdgfb/Bm	2
GO:0097062	GO:0097062	dendritic spine maintenance	2/385	23/23328	0.054723979	0.12481	0.075	Prnp/Apoe	2
GO:0097186	GO:0097186	amelogenesis	2/385	23/23328	0.054723979	0.12481	0.075	Perp/Tbx1	2
GO:0140112	GO:0140112	extracellular vesicle biogenesis	2/385	23/23328	0.054723979	0.12481	0.075	Cd34/Sdc	2
GO:1901626	GO:1901626	regulation of postsynaptic membrane organization	2/385	23/23328	0.054723979	0.12481	0.075	Ptn/Apoe	2
GO:0002696	GO:0002696	positive regulation of leukocyte activation	13/385	479/23328	0.055868385	0.12735	0.07652	Thy1/Cd3	13
GO:0010212	GO:0010212	response to ionizing radiation	5/385	125/23328	0.056470893	0.12865	0.07731	Sfrp1/Cdk	5
GO:0030522	GO:0030522	intracellular receptor signaling pathway	7/385	207/23328	0.056642088	0.12897	0.0775	Sfrp1/Tbx	7
GO:0046902	GO:0046902	regulation of mitochondrial membrane permeability	3/385	53/23328	0.057049757	0.12976	0.07797	Slc25a4/C	3
GO:0048247	GO:0048247	lymphocyte chemotaxis	3/385	53/23328	0.057049757	0.12976	0.07797	Cxcl12/Cc	3
GO:0098657	GO:0098657	import into cell	8/385	251/23328	0.057495177	0.1307	0.07854	Cd36/Rgs	8
GO:0050770	GO:0050770	regulation of axonogenesis	7/385	208/23328	0.057827022	0.13129	0.07889	Mgll/Cxcl1	7
GO:0008593	GO:0008593	regulation of Notch signaling pathway	4/385	88/23328	0.057912331	0.13129	0.07889	Dtx1/Nrarp	4
GO:0034620	GO:0034620	cellular response to unfolded protein	4/385	88/23328	0.057912331	0.13129	0.07889	Atf3/Hspa	4
GO:0042475	GO:0042475	odontogenesis of dentin-containing tooth	4/385	88/23328	0.057912331	0.13129	0.07889	Perp/Tbx1	4
GO:0050672	GO:0050672	negative regulation of lymphocyte proliferation	4/385	88/23328	0.057912331	0.13129	0.07889	Prnp/Cd24	4
GO:0046822	GO:0046822	regulation of nucleocytoplasmic transport	5/385	126/23328	0.058047332	0.13145	0.07899	Cd36/Gas	5
GO:0120254	GO:0120254	olefinic compound metabolic process	5/385	126/23328	0.058047332	0.13145	0.07899	Mgll/Egr1/	5
GO:0007409	GO:0007409	axonogenesis	13/385	482/23328	0.058107246	0.13152	0.07903	Mgll/Reln/	13
GO:1903532	GO:1903532	positive regulation of secretion by cell	11/385	388/23328	0.058437449	0.13219	0.07944	Rgcc/Cd3	11
GO:0015732	GO:0015732	prostaglandin transport	2/385	24/23328	0.059064383	0.13268	0.07973	Slco2a1/E	2
GO:0030728	GO:0030728	ovulation	2/385	24/23328	0.059064383	0.13268	0.07973	Nos3/Plat	2
GO:0031670	GO:0031670	cellular response to nutrient	2/385	24/23328	0.059064383	0.13268	0.07973	Lpl/Gas6	2
GO:0031954	GO:0031954	positive regulation of protein autophosphorylation	2/385	24/23328	0.059064383	0.13268	0.07973	Pdgfb/Pdg	2
GO:0034505	GO:0034505	tooth mineralization	2/385	24/23328	0.059064383	0.13268	0.07973	Tbx1/Col1	2
GO:0035455	GO:0035455	response to interferon-alpha	2/385	24/23328	0.059064383	0.13268	0.07973	Bst2/Gas6	2
GO:0042474	GO:0042474	middle ear morphogenesis	2/385	24/23328	0.059064383	0.13268	0.07973	Tbx1/Edn1	2
GO:0043586	GO:0043586	tongue development	2/385	24/23328	0.059064383	0.13268	0.07973	Tbx1/Cyp2	2
GO:0044331	GO:0044331	cell-cell adhesion mediated by cadherin	2/385	24/23328	0.059064383	0.13268	0.07973	Rgcc/Cdh	2
GO:0050687	GO:0050687	negative regulation of defense response to virus	2/385	24/23328	0.059064383	0.13268	0.07973	Fgl2/Htra1	2
GO:0051882	GO:0051882	mitochondrial depolarization	2/385	24/23328	0.059064383	0.13268	0.07973	Kdr/Dcn	2
GO:0061339	GO:0061339	establishment or maintenance of monopolar cell polari	2/385	24/23328	0.059064383	0.13268	0.07973	Tcf15/Fsc	2
GO:0072207	GO:0072207	metanephric epithelium development	2/385	24/23328	0.059064383	0.13268	0.07973	Pdgfb/Aqp	2
GO:0035272	GO:0035272	exocrine system development	3/385	54/23328	0.059695353	0.13388	0.08045	Pdgfb/Tgm	3
GO:0070542	GO:0070542	response to fatty acid	3/385	54/23328	0.059695353	0.13388	0.08045	Cd36/Lpl/	3
GO:2000649	GO:2000649	regulation of sodium ion transmembrane transporter ac	3/385	54/23328	0.059695353	0.13388	0.08045	Fxyd6/Atp	3
GO:0032945	GO:0032945	negative regulation of mononuclear cell proliferation	4/385	89/23328	0.059884335	0.13423	0.08066	Prnp/Cd24	4
GO:0032609	GO:0032609	interferon-gamma production	5/385	128/23328	0.06127525	0.13721	0.08245	Prnp/Il33/	5
GO:0061387	GO:0061387	regulation of extent of cell growth	5/385	128/23328	0.06127525	0.13721	0.08245	Mgll/Cxcl1	5
GO:0071356	GO:0071356	cellular response to tumor necrosis factor	6/385	169/23328	0.061885088	0.13844	0.08319	Fabp4/Sfr	6
GO:0009593	GO:0009593	detection of chemical stimulus	4/385	90/23328	0.061891672	0.13844	0.08319	Scarb1/Rt	4
GO:0009948	GO:0009948	anterior/posterior axis specification	3/385	55/23328	0.062397611	0.1389	0.08346	Nrarp/Bmp	3
GO:0031529	GO:0031529	ruffle organization	3/385	55/23328	0.062397611	0.1389	0.08346	Cav1/Tpm	3
GO:0045773	GO:0045773	positive regulation of axon extension	3/385	55/23328	0.062397611	0.1389	0.08346	Cxcl12/Fn	3
GO:0045912	GO:0045912	negative regulation of carbohydrate metabolic process	3/385	55/23328	0.062397611	0.1389	0.08346	Nupr1/Ier3	3
GO:0046580	GO:0046580	negative regulation of Ras protein signal transduction	3/385	55/23328	0.062397611	0.1389	0.08346	Rasip1/Itg	3
GO:0050775	GO:0050775	positive regulation of dendrite morphogenesis	3/385	55/23328	0.062397611	0.1389	0.08346	Reln/Sgk1	3
GO:0050922	GO:0050922	negative regulation of chemotaxis	3/385	55/23328	0.062397611	0.1389	0.08346	Dusp1/Th	3
GO:0051496	GO:0051496	positive regulation of stress fiber assembly	3/385	55/23328	0.062397611	0.1389	0.08346	Rgcc/Arhg	3
GO:1902750	GO:1902750	negative regulation of cell cycle G2/M phase transition	3/385	55/23328	0.062397611	0.1389	0.08346	Fhl1/Cdkn	3
GO:0030900	GO:0030900	forebrain development	11/385	393/23328	0.062807857	0.13974	0.08397	Reln/Cxcl	11
GO:0006706	GO:0006706	steroid catabolic process	2/385	25/23328	0.063519063	0.1405	0.08443	Scarb1/Ap	2
GO:0007095	GO:0007095	mitotic G2 DNA damage checkpoint	2/385	25/23328	0.063519063	0.1405	0.08443	Cdkn1a/Ie	2
GO:0010894	GO:0010894	negative regulation of steroid biosynthetic process	2/385	25/23328	0.063519063	0.1405	0.08443	Apoe/Prox	2
GO:0032897	GO:0032897	negative regulation of viral transcription	2/385	25/23328	0.063519063	0.1405	0.08443	Zfp36/Jun	2
GO:0036342	GO:0036342	post-anal tail morphogenesis	2/385	25/23328	0.063519063	0.1405	0.08443	Tcf15/Sfrp	2
GO:0045939	GO:0045939	negative regulation of steroid metabolic process	2/385	25/23328	0.063519063	0.1405	0.08443	Apoe/Prox	2
GO:0046426	GO:0046426	negative regulation of receptor signaling pathway via J	2/385	25/23328	0.063519063	0.1405	0.08443	Socs3/Ca	2
GO:0048266	GO:0048266	behavioral response to pain	2/385	25/23328	0.063519063	0.1405	0.08443	Thbs1/Vw	2
GO:0070633	GO:0070633	transepithelial transport	2/385	25/23328	0.063519063	0.1405	0.08443	Aqp1/Edn	2
GO:1904738	GO:1904738	vascular associated smooth muscle cell migration	2/385	25/23328	0.063519063	0.1405	0.08443	Igfbp5/Pd	2
GO:1904752	GO:1904752	regulation of vascular associated smooth muscle cell r	2/385	25/23328	0.063519063	0.1405	0.08443	Igfbp5/Pd	2
GO:0014015	GO:0014015	positive regulation of gliogenesis	4/385	91/23328	0.063934182	0.1412	0.08484	Vim/Ptn/S	4
GO:0032890	GO:0032890	regulation of organic acid transport	4/385	91/23328	0.063934182	0.1412	0.08484	Rgs4/Thbs	4
GO:2000117	GO:2000117	negative regulation of cysteine-type endopeptidase act	4/385	91/23328	0.063934182	0.1412	0.08484	Aqp1/Thbs	4
GO:0042098	GO:0042098	T cell proliferation	7/385	213/23328	0.063980822	0.14122	0.08486	Cxcl12/Pr	7
GO:0051222	GO:0051222	positive regulation of protein transport	10/385	348/23328	0.064304569	0.14186	0.08524	Rgcc/Cd3	10
GO:0008203	GO:0008203	cholesterol metabolic process	5/385	130/23328	0.064603028	0.14245	0.0856	Scarb1/Cy	5
GO:0045931	GO:0045931	positive regulation of mitotic cell cycle	6/385	171/23328	0.064742362	0.14268	0.08573	Rgcc/Lgm	6
GO:0021587	GO:0021587	cerebellum morphogenesis	3/385	56/23328	0.065155812	0.14306	0.08597	Serpine2/I	3
GO:0042446	GO:0042446	hormone biosynthetic process	3/385	56/23328	0.065155812	0.14306	0.08597	Scarb1/Eg	3
GO:0044060	GO:0044060	regulation of endocrine process	3/385	56/23328	0.065155812	0.14306	0.08597	Dab2/Gja5	3
GO:0061900	GO:0061900	glial cell activation	3/385	56/23328	0.065155812	0.14306	0.08597	Clu/Il33/Ju	3
GO:0070265	GO:0070265	necrotic cell death	3/385	56/23328	0.065155812	0.14306	0.08597	Slc25a4/C	3
GO:0071715	GO:0071715	icosanoid transport	3/385	56/23328	0.065155812	0.14306	0.08597	Slco2a1/E	3
GO:1901571	GO:1901571	fatty acid derivative transport	3/385	56/23328	0.065155812	0.14306	0.08597	Slco2a1/E	3
GO:0032984	GO:0032984	protein-containing complex disassembly	7/385	214/23328	0.065257532	0.14321	0.08605	Dstn/Nes/	7
GO:0006096	GO:0006096	glycolytic process	4/385	92/23328	0.066011691	0.14471	0.08696	Nupr1/Ald	4
GO:0043900	GO:0043900	regulation of multi-organism process	4/385	92/23328	0.066011691	0.14471	0.08696	Pde3a/Arl	4
GO:0002460	GO:0002460	adaptive immune response based on somatic recombi	12/385	445/23328	0.066910246	0.14661	0.0881	Fgl2/Irf7/F	12
GO:0006749	GO:0006749	glutathione metabolic process	3/385	57/23328	0.067969218	0.14749	0.08863	Gstm1/Mg	3
GO:0007585	GO:0007585	respiratory gaseous exchange by respiratory system	3/385	57/23328	0.067969218	0.14749	0.08863	Tcf15/Flt4	3
GO:0051591	GO:0051591	response to cAMP	3/385	57/23328	0.067969218	0.14749	0.08863	Igfbp5/Aq	3
GO:0055078	GO:0055078	sodium ion homeostasis	3/385	57/23328	0.067969218	0.14749	0.08863	Sgk1/Atp1	3

GO:0000289	GO:0000289	nuclear-transcribed mRNA poly(A) tail shortening	2/385	26/23328	0.06808309	0.14749	0.08863	Btg2/Zfp3	2
GO:0001780	GO:0001780	neutrophil homeostasis	2/385	26/23328	0.06808309	0.14749	0.08863	Cfh/Anxa1	2
GO:0003084	GO:0003084	positive regulation of systemic arterial blood pressure	2/385	26/23328	0.06808309	0.14749	0.08863	Eng/Nr2f2	2
GO:0010575	GO:0010575	positive regulation of vascular endothelial growth facto	2/385	26/23328	0.06808309	0.14749	0.08863	C3/Flt4	2
GO:0010592	GO:0010592	positive regulation of lamellipodium assembly	2/385	26/23328	0.06808309	0.14749	0.08863	Aqp1/Fscı	2
GO:0030204	GO:0030204	chondroitin sulfate metabolic process	2/385	26/23328	0.06808309	0.14749	0.08863	Bgn/Dcn	2
GO:0030325	GO:0030325	adrenal gland development	2/385	26/23328	0.06808309	0.14749	0.08863	Ly6e/Cdkı	2
GO:0043171	GO:0043171	peptide catabolic process	2/385	26/23328	0.06808309	0.14749	0.08863	Cpe/Ece1	2
GO:0045723	GO:0045723	positive regulation of fatty acid biosynthetic process	2/385	26/23328	0.06808309	0.14749	0.08863	Cd74/Anx	2
GO:0060765	GO:0060765	regulation of androgen receptor signaling pathway	2/385	26/23328	0.06808309	0.14749	0.08863	Sfrp1/Dab	2
GO:0071549	GO:0071549	cellular response to dexamethasone stimulus	2/385	26/23328	0.06808309	0.14749	0.08863	Aqp1/Bmp	2
GO:0090280	GO:0090280	positive regulation of calcium ion import	2/385	26/23328	0.06808309	0.14749	0.08863	Cxcl12/Pc	2
GO:0098901	GO:0098901	regulation of cardiac muscle cell action potential	2/385	26/23328	0.06808309	0.14749	0.08863	Cav1/Gja5	2
GO:1901380	GO:1901380	negative regulation of potassium ion transmembrane t	2/385	26/23328	0.06808309	0.14749	0.08863	Rgs4/Cav	2
GO:1904893	GO:1904893	negative regulation of receptor signaling pathway via S	2/385	26/23328	0.06808309	0.14749	0.08863	Socs3/Ca	2
GO:2000637	GO:2000637	positive regulation of gene silencing by miRNA	2/385	26/23328	0.06808309	0.14749	0.08863	Zfp36/Bm	2
GO:2000738	GO:2000738	positive regulation of stem cell differentiation	2/385	26/23328	0.06808309	0.14749	0.08863	Ptn/Bmp4	2
GO:0006757	GO:0006757	ATP generation from ADP	4/385	93/23328	0.068124015	0.14749	0.08863	Nupr1/Ald	4
GO:0009798	GO:0009798	axis specification	4/385	93/23328	0.068124015	0.14749	0.08863	Sfrp1/Nra	4
GO:0006091	GO:0006091	generation of precursor metabolites and energy	11/385	400/23328	0.06927763	0.14991	0.09008	Adh7/Cox	11
GO:0009314	GO:0009314	response to radiation	11/385	401/23328	0.070235613	0.1519	0.09128	Nts/Sfrp1/	11
GO:1901879	GO:1901879	regulation of protein depolymerization	4/385	94/23328	0.070270959	0.1519	0.09128	Dstn/Nes/	4
GO:0035107	GO:0035107	appendage morphogenesis	6/385	175/23328	0.07068569	0.15256	0.09167	Hoxd9/Ec	6
GO:0035108	GO:0035108	limb morphogenesis	6/385	175/23328	0.07068569	0.15256	0.09167	Hoxd9/Ec	6
GO:0042129	GO:0042129	regulation of T cell proliferation	6/385	175/23328	0.07068569	0.15256	0.09167	Prnp/Cd24	6
GO:0010517	GO:0010517	regulation of phospholipase activity	3/385	58/23328	0.070837066	0.15265	0.09173	Flt1/Htr2b	3
GO:0043470	GO:0043470	regulation of carbohydrate catabolic process	3/385	58/23328	0.070837066	0.15265	0.09173	Nupr1/Ier3	3
GO:0048016	GO:0048016	inositol phosphate-mediated signaling	3/385	58/23328	0.070837066	0.15265	0.09173	Prnp/Pdgf	3
GO:0006882	GO:0006882	cellular zinc ion homeostasis	2/385	27/23328	0.07275166	0.15566	0.09353	Mt1/Mt2	2
GO:0014821	GO:0014821	phasic smooth muscle contraction	2/385	27/23328	0.07275166	0.15566	0.09353	Htr2b/Edn	2
GO:0032528	GO:0032528	microvillus organization	2/385	27/23328	0.07275166	0.15566	0.09353	Fscn1/Poc	2
GO:0035809	GO:0035809	regulation of urine volume	2/385	27/23328	0.07275166	0.15566	0.09353	Adm/Edn1	2
GO:0045780	GO:0045780	positive regulation of bone resorption	2/385	27/23328	0.07275166	0.15566	0.09353	Trf/Ahsg	2
GO:0046852	GO:0046852	positive regulation of bone remodeling	2/385	27/23328	0.07275166	0.15566	0.09353	Trf/Ahsg	2
GO:0046885	GO:0046885	regulation of hormone biosynthetic process	2/385	27/23328	0.07275166	0.15566	0.09353	Egr1/Dab2	2
GO:0060148	GO:0060148	positive regulation of posttranscriptional gene silencing	2/385	27/23328	0.07275166	0.15566	0.09353	Zfp36/Bm	2
GO:0061298	GO:0061298	retina vasculature development in camera-type eye	2/385	27/23328	0.07275166	0.15566	0.09353	Arhgef15/	2
GO:0071677	GO:0071677	positive regulation of mononuclear cell migration	2/385	27/23328	0.07275166	0.15566	0.09353	Cxcl12/Lg	2
GO:0090103	GO:0090103	cochlea morphogenesis	2/385	27/23328	0.07275166	0.15566	0.09353	Tbx1/Cthr	2
GO:0150077	GO:0150077	regulation of neuroinflammatory response	2/385	27/23328	0.07275166	0.15566	0.09353	Nupr1/Cd2	2
GO:1903010	GO:1903010	regulation of bone development	2/385	27/23328	0.07275166	0.15566	0.09353	Kdr/Lox	2
GO:1904994	GO:1904994	regulation of leukocyte adhesion to vascular endothelia	2/385	27/23328	0.07275166	0.15566	0.09353	Cxcl12/Se	2
GO:0043467	GO:0043467	regulation of generation of precursor metabolites and e	5/385	135/23328	0.07335699	0.15679	0.09422	Nupr1/Ier3	5
GO:0072331	GO:0072331	signal transduction by p53 class mediator	5/385	135/23328	0.07335699	0.15679	0.09422	Perp/Nupı	5
GO:1901990	GO:1901990	regulation of mitotic cell cycle phase transition	8/385	265/23328	0.073717068	0.15741	0.09459	Rgcc/Fhl1	8
GO:0030042	GO:0030042	actin filament depolymerization	3/385	59/23328	0.073758578	0.15741	0.09459	Dstn/Mica	3
GO:0060193	GO:0060193	positive regulation of lipase activity	3/385	59/23328	0.073758578	0.15741	0.09459	Flt1/Gpihb	3
GO:0031016	GO:0031016	pancreas development	4/385	96/23328	0.074667861	0.15903	0.09556	Clu/Bmp4	4
GO:0045638	GO:0045638	negative regulation of myeloid cell differentiation	4/385	96/23328	0.074667861	0.15903	0.09556	Sfrp1/Lmc	4
GO:0061053	GO:0061053	somite development	4/385	96/23328	0.074667861	0.15903	0.09556	Tcf15/Sfrp	4
GO:0070555	GO:0070555	response to interleukin-1	4/385	96/23328	0.074667861	0.15903	0.09556	Fn1/Ccl21	4
GO:0097553	GO:0097553	calcium ion transmembrane import into cytosol	5/385	136/23328	0.075181782	0.16004	0.09617	Aplnr/Thy	5
GO:0021575	GO:0021575	hindbrain morphogenesis	3/385	60/23328	0.076732955	0.16286	0.09786	Serpine2/I	3
GO:0051893	GO:0051893	regulation of focal adhesion assembly	3/385	60/23328	0.076732955	0.16286	0.09786	Thy1/Kdr/	3
GO:0090109	GO:0090109	regulation of cell-substrate junction assembly	3/385	60/23328	0.076732955	0.16286	0.09786	Thy1/Kdr/	3
GO:0098900	GO:0098900	regulation of action potential	3/385	60/23328	0.076732955	0.16286	0.09786	Cd36/Cav	3
GO:1905517	GO:1905517	macrophage migration	3/385	60/23328	0.076732955	0.16286	0.09786	Cd9/Cd20	3
GO:0019217	GO:0019217	regulation of fatty acid metabolic process	4/385	97/23328	0.076917371	0.16286	0.09786	Fabp5/Ca	4
GO:0045069	GO:0045069	regulation of viral genome replication	4/385	97/23328	0.076917371	0.16286	0.09786	Rsad2/Bs	4
GO:0007050	GO:0007050	cell cycle arrest	5/385	137/23328	0.077031066	0.16286	0.09786	Rgcc/Cdk	5
GO:0034341	GO:0034341	response to interferon-gamma	5/385	137/23328	0.077031066	0.16286	0.09786	Vim/Ccl21	5
GO:0002089	GO:0002089	lens morphogenesis in camera-type eye	2/385	28/23328	0.077520103	0.16286	0.09786	Bmp4/Pro	2
GO:0006026	GO:0006026	aminoglycan catabolic process	2/385	28/23328	0.077520103	0.16286	0.09786	Lyve1/Pgl	2
GO:0010458	GO:0010458	exit from mitosis	2/385	28/23328	0.077520103	0.16286	0.09786	Rgcc/Cdk	2
GO:0019835	GO:0019835	cytolysis	2/385	28/23328	0.077520103	0.16286	0.09786	Fgl2/Lbp	2
GO:0034311	GO:0034311	diol metabolic process	2/385	28/23328	0.077520103	0.16286	0.09786	Plpp3/Ace	2
GO:0045663	GO:0045663	positive regulation of myoblast differentiation	2/385	28/23328	0.077520103	0.16286	0.09786	Igfbp3/Cx	2
GO:0055069	GO:0055069	zinc ion homeostasis	2/385	28/23328	0.077520103	0.16286	0.09786	Mt1/Mt2	2
GO:0060039	GO:0060039	pericardium development	2/385	28/23328	0.077520103	0.16286	0.09786	Pdpn/Vca	2
GO:0060384	GO:0060384	innervation	2/385	28/23328	0.077520103	0.16286	0.09786	Vcam1/Se	2
GO:0060390	GO:0060390	regulation of SMAD protein signal transduction	2/385	28/23328	0.077520103	0.16286	0.09786	Dab2/Bmp	2
GO:0061099	GO:0061099	negative regulation of protein tyrosine kinase activity	2/385	28/23328	0.077520103	0.16286	0.09786	Thy1/Cav	2
GO:0061436	GO:0061436	establishment of skin barrier	2/385	28/23328	0.077520103	0.16286	0.09786	Lsr/Cyp26	2
GO:0071480	GO:0071480	cellular response to gamma radiation	2/385	28/23328	0.077520103	0.16286	0.09786	Cdkn1a/E	2
GO:0086005	GO:0086005	ventricular cardiac muscle cell action potential	2/385	28/23328	0.077520103	0.16286	0.09786	Cav1/Gja5	2
GO:0098698	GO:0098698	postsynaptic specialization assembly	2/385	28/23328	0.077520103	0.16286	0.09786	Reln/Gap4	2
GO:1900273	GO:1900273	positive regulation of long-term synaptic potentiation	2/385	28/23328	0.077520103	0.16286	0.09786	Reln/Lgmı	2
GO:1901532	GO:1901532	regulation of hematopoietic progenitor cell differentiatio	2/385	28/23328	0.077520103	0.16286	0.09786	Flt1/Kdr	2
GO:1902107	GO:1902107	positive regulation of leukocyte differentiation	6/385	180/23328	0.078542567	0.16493	0.0991	Kitl/Fos/G	6
GO:0045833	GO:0045833	negative regulation of lipid metabolic process	4/385	98/23328	0.079200603	0.16623	0.09988	Pdgfb/Apc	4
GO:0007584	GO:0007584	response to nutrient	3/385	61/23328	0.079759381	0.16731	0.10054	Lpl/Rbp1/	3
GO:1904951	GO:1904951	positive regulation of establishment of protein localizat	10/385	364/23328	0.081091342	0.17002	0.10217	Rgcc/Cd3	10
GO:0046031	GO:0046031	ADP metabolic process	4/385	99/23328	0.081517307	0.17075	0.1026	Nupr1/Ald	4
GO:0097061	GO:0097061	dendritic spine organization	4/385	99/23328	0.081517307	0.17075	0.1026	Reln/Prnp	4
GO:0048813	GO:0048813	dendrite morphogenesis	6/385	182/23328	0.081817691	0.17094	0.10271	Reln/Id1/F	6
GO:0003085	GO:0003085	negative regulation of systemic arterial blood pressure	2/385	29/23328	0.082383868	0.17094	0.10271	Cd36/Ier3	2
GO:0030262	GO:0030262	apoptotic nuclear changes	2/385	29/23328	0.082383868	0.17094	0.10271	Cd24a/Igf	2
GO:0031116	GO:0031116	positive regulation of microtubule polymerization	2/385	29/23328	0.082383868	0.17094	0.10271	Gda/Cav1	2
GO:0031295	GO:0031295	T cell costimulation	2/385	29/23328	0.082383868	0.17094	0.10271	Cd24a/Ca	2
GO:0033198	GO:0033198	response to ATP	2/385	29/23328	0.082383868	0.17094	0.10271	Abcc9/Hs	2
GO:0033273	GO:0033273	response to vitamin	2/385	29/23328	0.082383868	0.17094	0.10271	Rbp1/Gas	2
GO:0034629	GO:0034629	cellular protein-containing complex localization	2/385	29/23328	0.082383868	0.17094	0.10271	Exoc3l2/T	2
GO:0042403	GO:0042403	thyroid hormone metabolic process	2/385	29/23328	0.082383868	0.17094	0.10271	Reln/Duox	2
GO:0048643	GO:0048643	positive regulation of skeletal muscle tissue developme	2/385	29/23328	0.082383868	0.17094	0.10271	Tbx1/Cyp	2
GO:0050858	GO:0050858	negative regulation of antigen receptor-mediated signa	2/385	29/23328	0.082383868	0.17094	0.10271	Thy1/Prnp	2
GO:0060571	GO:0060571	morphogenesis of an epithelial fold	2/385	29/23328	0.082383868	0.17094	0.10271	Pthlh/Bmp	2
GO:0061311	GO:0061311	cell surface receptor signaling pathway involved in hea	2/385	29/23328	0.082383868	0.17094	0.10271	Sox17/Bm	2
GO:0071548	GO:0071548	response to dexamethasone	2/385	29/23328	0.082383868	0.17094	0.10271	Aqp1/Bmp	2
GO:0090140	GO:0090140	regulation of mitochondrial fission	2/385	29/23328	0.082383868	0.17094	0.10271	Kdr/Dcn	2
GO:0090208	GO:0090208	positive regulation of triglyceride metabolic process	2/385	29/23328	0.082383868	0.17094	0.10271	Cd36/Sca	2
GO:0097345	GO:0097345	mitochondrial outer membrane permeabilization	2/385	29/23328	0.082383868	0.17094	0.10271	Chchd10/	2
GO:1900027	GO:1900027	regulation of ruffle assembly	2/385	29/23328	0.082383868	0.17094	0.10271	Cav1/Kan	2
GO:1901185	GO:1901185	negative regulation of ERBB signaling pathway	2/385	29/23328	0.082383868	0.17094	0.10271	Lgmn/Ptpı	2
GO:0006081	GO:0006081	cellular aldehyde metabolic process	3/385	62/23328	0.082837026	0.17128	0.10292	Adh1/Aldc	3
GO:0030330	GO:0030330	DNA damage response, signal transduction by p53 cla	3/385	62/23328	0.082837026	0.17128	0.10292	Cdkn1a/A	3
GO:0031018	GO:0031018	endocrine pancreas development	3/385	62/23328	0.082837026	0.17128	0.10292	Clu/Bmp4	3
GO:0031640	GO:0031640	killing of cells of other organism	3/385	62/23328	0.082837026	0.17128	0.10292	Cxcl12/Nt	3
GO:0050854	GO:0050854	regulation of antigen receptor-mediated signaling path	3/385	62/23328	0.082837026	0.17128	0.10292	Thy1/Prnp	3
GO:0060393	GO:0060393	regulation of pathway-restricted SMAD protein phosph	3/385	62/23328	0.082837026	0.17128	0.10292	Eng/Dab2	3
GO:0061337	GO:0061337	cardiac conduction	3/385	62/23328	0.082837026	0.17128	0.10292	Abcc9/Ca	3
GO:0016331	GO:0016331	morphogenesis of embryonic epithelium	6/385	183/23328	0.083483466	0.17253	0.10367	Sfrp1/Spir	6
GO:0071695	GO:0071695	anatomical structure maturation	8/385	273/23328	0.084114564	0.17375	0.10441	Cdh5/Pde	8
GO:0009799	GO:0009799	specification of symmetry	5/385	141/23328	0.084671287	0.17473	0.10499	Tbx1/Sox	5
GO:1902652	GO:1902652	secondary alcohol metabolic process	5/385	141/23328	0.084671287	0.17473	0.10499	Scarb1/Cy	5
GO:0006661	GO:0006661	phosphatidylinositol biosynthetic process	3/385	63/23328	0.085965043	0.17722	0.10649	Pdgfb/Htr	3
GO:0150116	GO:0150116	regulation of cell-substrate junction organization	3/385	63/23328	0.085965043	0.17722	0.10649	Thy1/Kdr/	3
GO:0010821	GO:0010821	regulation of mitochondrion organization	5/385	142/23328	0.086641606	0.17835	0.10717	Kdr/Chchc	5
GO:0016052	GO:0016052	carbohydrate catabolic process	5/385	142/23328	0.086641606	0.17835	0.10717	Nupr1/Ald	5
GO:0046488	GO:0046488	phosphatidylinositol metabolic process	5/385	142/23328	0.086641606	0.17835	0.10717	Socs3/Pd	5
GO:0016050	GO:0016050	vesicle organization	8/385	275/23328	0.086842247	0.17868	0.10737	Cd34/Aqp	8
GO:0002724	GO:0002724	regulation of T cell cytokine production	2/385	30/23328	0.087338531	0.17935	0.10777	Rsad2/B2	2
GO:0006921	GO:0006921	cellular component disassembly involved in execution	2/385	30/23328	0.087338531	0.17935	0.10777	Cd24a/Igf	2
GO:0010863	GO:0010863	positive regulation of phospholipase C activity	2/385	30/23328	0.087338531	0.17935	0.10777	Flt1/Htr2b	2
GO:0030149	GO:0030149	sphingolipid catabolic process	2/385	30/23328	0.087338531	0.17935	0.10777	Gm2a/Ace	2
GO:0046328	GO:0046328	regulation of JNK cascade	6/385	186/23328	0.088593078	0.18184	0.10926	Sfrp1/Dab	6
GO:0050848	GO:0050848	regulation of calcium-mediated signaling	4/385	102/23328	0.088665595	0.18189	0.1093	Prnp/Kdr/	4
GO:0022412	GO:0022412	cellular process involved in reproduction in multicellula	12/385	468/23328	0.089117085	0.18243	0.10962	Cxcl12/Cc	12
GO:0006879	GO:0006879	cellular iron ion homeostasis	3/385	64/23328	0.089142573	0.18243	0.10962	Cp/Lcn2/T	3
GO:0030858	GO:0030858	positive regulation of epithelial cell differentiation	3/385	64/23328	0.089142573	0.18243	0.10962	Cdh5/Cldı	3

GO:1902305	GO:1902305	regulation of sodium ion transmembrane transport	3/385	64/23328	0.089142573	0.18243	0.10962	Fxyd6/Atp	3
GO:1903510	GO:1903510	mucopolysaccharide metabolic process	3/385	64/23328	0.089142573	0.18243	0.10962	Bgn/Pdgft	3
GO:0006956	GO:0006956	complement activation	6/385	187/23328	0.090333531	0.18477	0.11103	Rgcc/Cfh/	6
GO:0010506	GO:0010506	regulation of autophagy	8/385	278/23328	0.091029705	0.18611	0.11183	Nupr1/Kdr	8
GO:0031669	GO:0031669	cellular response to nutrient levels	6/385	188/23328	0.092092511	0.18722	0.1125	Atf3/Lpl/G	6
GO:0034612	GO:0034612	response to tumor necrosis factor	6/385	188/23328	0.092092511	0.18722	0.1125	Fabp4/Sfr	6
GO:0042461	GO:0042461	photoreceptor cell development	3/385	65/23328	0.092368746	0.18722	0.1125	Thy1/Cfh/	3
GO:0045840	GO:0045840	positive regulation of mitotic nuclear division	3/385	65/23328	0.092368746	0.18722	0.1125	Rgcc/Pdg	3
GO:0046503	GO:0046503	glycerolipid catabolic process	3/385	65/23328	0.092368746	0.18722	0.1125	Mgll/Scarl	3
GO:0060389	GO:0060389	pathway-restricted SMAD protein phosphorylation	3/385	65/23328	0.092368746	0.18722	0.1125	Eng/Dab2	3
GO:0008299	GO:0008299	isoprenoid biosynthetic process	2/385	31/23328	0.092379785	0.18722	0.1125	Rbp1/Cyp	2
GO:0031294	GO:0031294	lymphocyte costimulation	2/385	31/23328	0.092379785	0.18722	0.1125	Cd24a/Ca	2
GO:0043276	GO:0043276	anoikis	2/385	31/23328	0.092379785	0.18722	0.1125	Cav1/Itgb	2
GO:0045662	GO:0045662	negative regulation of myoblast differentiation	2/385	31/23328	0.092379785	0.18722	0.1125	Id3/Bmp4	2
GO:0048535	GO:0048535	lymph node development	2/385	31/23328	0.092379785	0.18722	0.1125	Ccl21a/Pc	2
GO:0060074	GO:0060074	synapse maturation	2/385	31/23328	0.092379785	0.18722	0.1125	Reln/Arhg	2
GO:0061037	GO:0061037	negative regulation of cartilage development	2/385	31/23328	0.092379785	0.18722	0.1125	Pthlh/Bmp	2
GO:0072574	GO:0072574	hepatocyte proliferation	2/385	31/23328	0.092379785	0.18722	0.1125	Ptn/Prox1	2
GO:0072575	GO:0072575	epithelial cell proliferation involved in liver morphogene	2/385	31/23328	0.092379785	0.18722	0.1125	Ptn/Prox1	2
GO:1900117	GO:1900117	regulation of execution phase of apoptosis	2/385	31/23328	0.092379785	0.18722	0.1125	Igfbp3/Ptc	2
GO:1902042	GO:1902042	negative regulation of extrinsic apoptotic signaling path	2/385	31/23328	0.092379785	0.18722	0.1125	Sfrp2/Hm	2
GO:2001014	GO:2001014	regulation of skeletal muscle cell differentiation	2/385	31/23328	0.092379785	0.18722	0.1125	Tbx1/Cyp	2
GO:0009135	GO:0009135	purine nucleoside diphosphate metabolic process	4/385	104/23328	0.09359345	0.18922	0.1137	Nupr1/Ald	4
GO:0009179	GO:0009179	purine ribonucleoside diphosphate metabolic process	4/385	104/23328	0.09359345	0.18922	0.1137	Nupr1/Ald	4
GO:0019731	GO:0019731	antibacterial humoral response	4/385	104/23328	0.09359345	0.18922	0.1137	B2m/Adm	4
GO:0045778	GO:0045778	positive regulation of ossification	4/385	104/23328	0.09359345	0.18922	0.1137	Ptn/Bmp4	4
GO:1901222	GO:1901222	regulation of NIK/NF-kappaB signaling	4/385	104/23328	0.09359345	0.18922	0.1137	Adgrg3/Pr	4
GO:0001764	GO:0001764	neuron migration	6/385	189/23328	0.093869953	0.18969	0.11398	Reln/Cxcl	6
GO:0006814	GO:0006814	sodium ion transport	7/385	234/23328	0.094011136	0.18988	0.1141	Fxyd6/Ser	7
GO:0045598	GO:0045598	regulation of fat cell differentiation	5/385	146/23328	0.094760474	0.1913	0.11495	Sfrp1/Lpl/	5
GO:0044772	GO:0044772	mitotic cell cycle phase transition	10/385	376/23328	0.095299612	0.1923	0.11555	Rgcc/Plk2	10
GO:0061351	GO:0061351	neural precursor cell proliferation	6/385	191/23328	0.097479955	0.19561	0.11754	Btg2/Cd24	6
GO:0002902	GO:0002902	regulation of B cell apoptotic process	2/385	32/23328	0.097503441	0.19561	0.11754	Cd24a/Cd	2
GO:0010574	GO:0010574	regulation of vascular endothelial growth factor produc	2/385	32/23328	0.097503441	0.19561	0.11754	C3/Flt4	2
GO:0031645	GO:0031645	negative regulation of nervous system process	2/385	32/23328	0.097503441	0.19561	0.11754	Jam2/Klk8	2
GO:0040036	GO:0040036	regulation of fibroblast growth factor receptor signaling	2/385	32/23328	0.097503441	0.19561	0.11754	Pdgfb/Thb	2
GO:0043901	GO:0043901	negative regulation of multi-organism process	2/385	32/23328	0.097503441	0.19561	0.11754	Arhgdib/B	2
GO:0045577	GO:0045577	regulation of B cell differentiation	2/385	32/23328	0.097503441	0.19561	0.11754	Sfrp1/Cd2	2
GO:0048384	GO:0048384	retinoic acid receptor signaling pathway	2/385	32/23328	0.097503441	0.19561	0.11754	Tbx1/Cyp	2
GO:0051491	GO:0051491	positive regulation of filopodium assembly	2/385	32/23328	0.097503441	0.19561	0.11754	Fscn1/Gp	2
GO:0060306	GO:0060306	regulation of membrane repolarization	2/385	32/23328	0.097503441	0.19561	0.11754	Cav1/Gja5	2
GO:0090075	GO:0090075	relaxation of muscle	2/385	32/23328	0.097503441	0.19561	0.11754	Atp1b1/Sl	2
GO:1902110	GO:1902110	positive regulation of mitochondrial membrane permea	2/385	32/23328	0.097503441	0.19561	0.11754	Chchd10/	2
GO:0106027	GO:0106027	neuron projection organization	4/385	106/23328	0.098648372	0.19782	0.11887	Reln/Prnp	4
GO:0021695	GO:0021695	cerebellar cortex development	3/385	67/23328	0.098963477	0.19826	0.11913	Serpine2/	3
GO:0060425	GO:0060425	lung morphogenesis	3/385	67/23328	0.098963477	0.19826	0.11913	Id1/Tnc/B	3
GO:0009913	GO:0009913	epidermal cell differentiation	6/385	193/23328	0.101162961	0.20257	0.12172	Zfp36/Bm	6
GO:0019395	GO:0019395	fatty acid oxidation	4/385	107/23328	0.101222693	0.20259	0.12174	Cd36/Adh	4
GO:0046635	GO:0046635	positive regulation of alpha-beta T cell activation	3/385	68/23328	0.102330241	0.2038	0.12246	Cd24a/H2	3
GO:0050432	GO:0050432	catecholamine secretion	3/385	68/23328	0.102330241	0.2038	0.12246	Cxcl12/Ly	3
GO:0097194	GO:0097194	execution phase of apoptosis	3/385	68/23328	0.102330241	0.2038	0.12246	Cd24a/Igf	3
GO:2000378	GO:2000378	negative regulation of reactive oxygen species metabo	3/385	68/23328	0.102330241	0.2038	0.12246	Cd34/Ptgi	3
GO:0000186	GO:0000186	activation of MAPKK activity	2/385	33/23328	0.102705423	0.2038	0.12246	Bmp4/F2r	2
GO:0001779	GO:0001779	natural killer cell differentiation	2/385	33/23328	0.102705423	0.2038	0.12246	Gas6/Pgly	2
GO:0003081	GO:0003081	regulation of systemic arterial blood pressure by renin-	2/385	33/23328	0.102705423	0.2038	0.12246	Gja5/F2r	2
GO:0032891	GO:0032891	negative regulation of organic acid transport	2/385	33/23328	0.102705423	0.2038	0.12246	Rgs4/Thb	2
GO:0033561	GO:0033561	regulation of water loss via skin	2/385	33/23328	0.102705423	0.2038	0.12246	Lsr/Cyp26	2
GO:0044818	GO:0044818	mitotic G2/M transition checkpoint	2/385	33/23328	0.102705423	0.2038	0.12246	Cdkn1a/Ie	2
GO:0045652	GO:0045652	regulation of megakaryocyte differentiation	2/385	33/23328	0.102705423	0.2038	0.12246	Lox/Hmgb	2
GO:0045745	GO:0045745	positive regulation of G protein-coupled receptor signa	2/385	33/23328	0.102705423	0.2038	0.12246	Ece1/C3	2
GO:0048841	GO:0048841	regulation of axon extension involved in axon guidance	2/385	33/23328	0.102705423	0.2038	0.12246	Cxcl12/Se	2
GO:0051354	GO:0051354	negative regulation of oxidoreductase activity	2/385	33/23328	0.102705423	0.2038	0.12246	Eng/Cav1	2
GO:0060914	GO:0060914	heart formation	2/385	33/23328	0.102705423	0.2038	0.12246	Tbx1/Bmp	2
GO:0070266	GO:0070266	necroptotic process	2/385	33/23328	0.102705423	0.2038	0.12246	Slc25a4/C	2
GO:0072576	GO:0072576	liver morphogenesis	2/385	33/23328	0.102705423	0.2038	0.12246	Ptn/Prox1	2
GO:1902745	GO:1902745	positive regulation of lamellipodium organization	2/385	33/23328	0.102705423	0.2038	0.12246	Aqp1/Fscn	2
GO:0001824	GO:0001824	blastocyst development	5/385	150/23328	0.103253115	0.2047	0.123	Sox17/Jun	5
GO:0061025	GO:0061025	membrane fusion	5/385	150/23328	0.103253115	0.2047	0.123	Cd9/Tbc1	5
GO:0009185	GO:0009185	ribonucleoside diphosphate metabolic process	4/385	108/23328	0.103827816	0.20574	0.12363	Nupr1/Ald	4
GO:0002449	GO:0002449	lymphocyte mediated immunity	11/385	432/23328	0.104172129	0.20632	0.12398	Fgl2/Irf7/F	11
GO:0006109	GO:0006109	regulation of carbohydrate metabolic process	6/385	195/23328	0.104918343	0.2076	0.12475	Nupr1/Pd	6
GO:2000241	GO:2000241	regulation of reproductive process	6/385	195/23328	0.104918343	0.2076	0.12475	Sfrp1/Nup	6
GO:0008643	GO:0008643	carbohydrate transport	5/385	151/23328	0.105433587	0.20853	0.1253	Fabp5/Aq	5
GO:0008542	GO:0008542	visual learning	3/385	69/23328	0.10574206	0.20904	0.12561	Nts/Itgb1/	3
GO:0051209	GO:0051209	release of sequestered calcium ion into cytosol	4/385	109/23328	0.106463405	0.21036	0.12641	Aplnr/Thy	4
GO:0002021	GO:0002021	response to dietary excess	2/385	34/23328	0.107981767	0.21186	0.12731	Apoe/Clic	2
GO:0003203	GO:0003203	endocardial cushion morphogenesis	2/385	34/23328	0.107981767	0.21186	0.12731	Eng/Nos3	2
GO:0006884	GO:0006884	cell volume homeostasis	2/385	34/23328	0.107981767	0.21186	0.12731	Kcnn4/Aq	2
GO:0014741	GO:0014741	negative regulation of muscle hypertrophy	2/385	34/23328	0.107981767	0.21186	0.12731	Rgs4/Igfb	2
GO:0032728	GO:0032728	positive regulation of interferon-beta production	2/385	34/23328	0.107981767	0.21186	0.12731	Irf7/Hmgb	2
GO:0033032	GO:0033032	regulation of myeloid cell apoptotic process	2/385	34/23328	0.107981767	0.21186	0.12731	Kitl/Anxa1	2
GO:0046466	GO:0046466	membrane lipid catabolic process	2/385	34/23328	0.107981767	0.21186	0.12731	Gm2a/Ace	2
GO:0048048	GO:0048048	embryonic eye morphogenesis	2/385	34/23328	0.107981767	0.21186	0.12731	Mfap5/Pro	2
GO:0090181	GO:0090181	regulation of cholesterol metabolic process	2/385	34/23328	0.107981767	0.21186	0.12731	Apoe/Eph	2
GO:0099068	GO:0099068	postsynapse assembly	2/385	34/23328	0.107981767	0.21186	0.12731	Reln/Gap	2
GO:0120255	GO:0120255	olefinic compound biosynthetic process	2/385	34/23328	0.107981767	0.21186	0.12731	Egr1/Dab	2
GO:1900274	GO:1900274	regulation of phospholipase C activity	2/385	34/23328	0.107981767	0.21186	0.12731	Flt1/Htr2b	2
GO:1902686	GO:1902686	mitochondrial outer membrane permeabilization involv	2/385	34/23328	0.107981767	0.21186	0.12731	Chchd10/	2
GO:2000108	GO:2000108	positive regulation of leukocyte apoptotic process	2/385	34/23328	0.107981767	0.21186	0.12731	Cd24a/An	2
GO:2000406	GO:2000406	positive regulation of T cell migration	2/385	34/23328	0.107981767	0.21186	0.12731	Cxcl12/Cc	2
GO:0022600	GO:0022600	digestive system process	4/385	110/23328	0.109129114	0.21335	0.1282	Cd36/Sca	4
GO:0035967	GO:0035967	cellular response to topologically incorrect protein	4/385	110/23328	0.109129114	0.21335	0.1282	Atf3/Hspa	4
GO:0038061	GO:0038061	NIK/NF-kappaB signaling	4/385	110/23328	0.109129114	0.21335	0.1282	Adgrg3/Pr	4
GO:0051283	GO:0051283	negative regulation of sequestering of calcium ion	4/385	110/23328	0.109129114	0.21335	0.1282	Aplnr/Thy	4
GO:0001541	GO:0001541	ovarian follicle development	3/385	70/23328	0.109198018	0.21335	0.1282	Kdr/Kitl/B	3
GO:0006766	GO:0006766	vitamin metabolic process	3/385	70/23328	0.109198018	0.21335	0.1282	Pltp/Rbp1	3
GO:0030512	GO:0030512	negative regulation of transforming growth factor beta	3/385	70/23328	0.109198018	0.21335	0.1282	Htra1/Cav	3
GO:0032922	GO:0032922	circadian regulation of gene expression	3/385	70/23328	0.109198018	0.21335	0.1282	Id1/Id3/E	3
GO:0045669	GO:0045669	positive regulation of osteoblast differentiation	3/385	70/23328	0.109198018	0.21335	0.1282	Bmp4/Cth	3
GO:0030183	GO:0030183	B cell differentiation	5/385	153/23328	0.109862194	0.21445	0.12886	Sfrp1/Cd2	5
GO:0071478	GO:0071478	cellular response to radiation	5/385	153/23328	0.109862194	0.21445	0.12886	Sfrp1/Aqp	5
GO:1990138	GO:1990138	neuron projection extension	6/385	198/23328	0.110685629	0.21595	0.12976	Mgll/Cxcl1	6
GO:0002437	GO:0002437	inflammatory response to antigenic stimulus	3/385	71/23328	0.112697189	0.21937	0.13182	Cd24a/C3	3
GO:0042509	GO:0042509	regulation of tyrosine phosphorylation of STAT protein	3/385	71/23328	0.112697189	0.21937	0.13182	Socs3/Pe	3
GO:0042733	GO:0042733	embryonic digit morphogenesis	3/385	71/23328	0.112697189	0.21937	0.13182	Ece1/Bmp	3
GO:0055081	GO:0055081	anion homeostasis	3/385	71/23328	0.112697189	0.21937	0.13182	Pthlh/Apo	3
GO:0061035	GO:0061035	regulation of cartilage development	3/385	71/23328	0.112697189	0.21937	0.13182	Pthlh/Bmp	3
GO:0003016	GO:0003016	respiratory system process	2/385	35/23328	0.113328617	0.21957	0.13194	Tcf15/Flt4	2
GO:0010591	GO:0010591	regulation of lamellipodium assembly	2/385	35/23328	0.113328617	0.21957	0.13194	Aqp1/Fscn	2
GO:0010719	GO:0010719	negative regulation of epithelial to mesenchymal transi	2/385	35/23328	0.113328617	0.21957	0.13194	Sfrp1/Sfrp	2
GO:0016339	GO:0016339	calcium-dependent cell-cell adhesion via plasma mem	2/385	35/23328	0.113328617	0.21957	0.13194	Selp/Cdh5	2
GO:0034331	GO:0034331	cell junction maintenance	2/385	35/23328	0.113328617	0.21957	0.13194	Chchd10/	2
GO:0048147	GO:0048147	negative regulation of fibroblast proliferation	2/385	35/23328	0.113328617	0.21957	0.13194	Sfrp1/Nup	2
GO:0050869	GO:0050869	negative regulation of B cell activation	2/385	35/23328	0.113328617	0.21957	0.13194	Sfrp1/Cd2	2
GO:0055083	GO:0055083	monovalent inorganic anion homeostasis	2/385	35/23328	0.113328617	0.21957	0.13194	Pthlh/Ckb	2
GO:0060441	GO:0060441	epithelial tube branching involved in lung morphogene	2/385	35/23328	0.113328617	0.21957	0.13194	Tnc/Bmp4	2
GO:0086091	GO:0086091	regulation of heart rate by cardiac conduction	2/385	35/23328	0.113328617	0.21957	0.13194	Cav1/Gja5	2
GO:0007009	GO:0007009	plasma membrane organization	4/385	112/23328	0.114549484	0.22173	0.13324	Clu/Ndrg1	4
GO:0051282	GO:0051282	regulation of sequestering of calcium ion	4/385	112/23328	0.114549484	0.22173	0.13324	Aplnr/Thy	4
GO:0007254	GO:0007254	JNK cascade	6/385	200/23328	0.114618886	0.22177	0.13326	Sfrp1/Dab	6
GO:0051606	GO:0051606	detection of stimulus	7/385	247/23328	0.115937804	0.22421	0.13473	Scarb1/Cx	7
GO:0007193	GO:0007193	adenylate cyclase-inhibiting G protein-coupled recepto	3/385	72/23328	0.116238645	0.22459	0.13495	S1pr1/Pde	3
GO:1903845	GO:1903845	negative regulation of cellular response to transforming	3/385	72/23328	0.116238645	0.22459	0.13495	Htra1/Cav	3
GO:0009267	GO:0009267	cellular response to starvation	5/385	156/23328	0.116671714	0.22532	0.13539	Atf3/Gas6	5
GO:0072659	GO:0072659	protein localization to plasma membrane	8/385	295/23328	0.116906832	0.22567	0.1356	Tmem88/	8
GO:0051208	GO:0051208	sequestering of calcium ion	4/385	113/23328	0.117303423	0.22633	0.136	Aplnr/Thy	4
GO:0060291	GO:0060291	long-term synaptic potentiation	7/385	248/23328	0.117726861	0.22704	0.13643	Reln/Prnp	7
GO:0010573	GO:0010573	vascular endothelial growth factor production	2/385	36/23328	0.118742225	0.22827	0.13716	C3/Flt4	2
GO:0031572	GO:0031572	G2 DNA damage checkpoint	2/385	36/23328	0.118742225	0.22827	0.13716	Cdkn1a/Ie	2

GO:0034142	GO:0034142	toll-like receptor 4 signaling pathway	2/385	36/23328	0.118742225	0.22827	0.13716	Nfkbia/Lb	2
GO:0048169	GO:0048169	regulation of long-term neuronal synaptic plasticity	2/385	36/23328	0.118742225	0.22827	0.13716	Kdr/Egr1	2
GO:0071158	GO:0071158	positive regulation of cell cycle arrest	2/385	36/23328	0.118742225	0.22827	0.13716	Rgcc/Cdk	2
GO:0071398	GO:0071398	cellular response to fatty acid	2/385	36/23328	0.118742225	0.22827	0.13716	Lpl/Id3	2
GO:2000191	GO:2000191	regulation of fatty acid transport	2/385	36/23328	0.118742225	0.22827	0.13716	Thbs1/Ed	2
GO:0062013	GO:0062013	positive regulation of small molecule metabolic proces	5/385	157/2332	0.118985273	0.22863	0.13738	Pdgfb/Da	5
GO:0002711	GO:0002711	positive regulation of T cell mediated immunity	3/385	73/23328	0.119821452	0.2299	0.13814	Rsad2/Cd	3
GO:0007632	GO:0007632	visual behavior	3/385	73/23328	0.119821452	0.2299	0.13814	Nts/Itgb1/	3
GO:1903793	GO:1903793	positive regulation of anion transport	3/385	73/23328	0.119821452	0.2299	0.13814	Apoe/Itgb	3
GO:0007498	GO:0007498	mesoderm development	4/385	114/2332	0.120086039	0.2299	0.13814	Tcf15/Tbx	4
GO:0034440	GO:0034440	lipid oxidation	4/385	114/2332	0.120086039	0.2299	0.13814	Cd36/Adh	4
GO:0042100	GO:0042100	B cell proliferation	4/385	114/2332	0.120086039	0.2299	0.13814	Cd38/Cd2	4
GO:0043401	GO:0043401	steroid hormone mediated signaling pathway	4/385	114/2332	0.120086039	0.2299	0.13814	Sfrp1/Dab	4
GO:0071346	GO:0071346	cellular response to interferon-gamma	4/385	114/2332	0.120086039	0.2299	0.13814	Vim/Ccl21	4
GO:1901987	GO:1901987	regulation of cell cycle phase transition	8/385	297/2332	0.120187061	0.22999	0.1382	Rgcc/Fhl1	8
GO:0031330	GO:0031330	negative regulation of cellular catabolic process	7/385	250/2332	0.121347894	0.2321	0.13947	Nupr1/Tim	7
GO:0030216	GO:0030216	keratinocyte differentiation	4/385	115/2332	0.122896957	0.23496	0.14118	Zfp36/Anx	4
GO:0006029	GO:0006029	proteoglycan metabolic process	3/385	74/23328	0.123444672	0.23579	0.14168	Bgn/Cytl1	3
GO:0021766	GO:0021766	hippocampus development	3/385	74/23328	0.123444672	0.23579	0.14168	Reln/Btg2	3
GO:1903900	GO:1903900	regulation of viral life cycle	5/385	159/2332	0.123676854	0.23598	0.1418	Rsad2/Bs	5
GO:0002218	GO:0002218	activation of innate immune response	2/385	37/23328	0.124218946	0.23598	0.1418	Mndal/Ifi2	2
GO:0002931	GO:0002931	response to ischemia	2/385	37/23328	0.124218946	0.23598	0.1418	Cav1/Egr	2
GO:0009954	GO:0009954	proximal/distal pattern formation	2/385	37/23328	0.124218946	0.23598	0.1418	Hoxd9/Cy	2
GO:0033144	GO:0033144	negative regulation of intracellular steroid hormone rec	2/385	37/23328	0.124218946	0.23598	0.1418	Sfrp1/Dab	2
GO:0035794	GO:0035794	positive regulation of mitochondrial membrane permea	2/385	37/23328	0.124218946	0.23598	0.1418	Chchd10/	2
GO:0046329	GO:0046329	negative regulation of JNK cascade	2/385	37/23328	0.124218946	0.23598	0.1418	Sfrp1/Sfrp	2
GO:0048846	GO:0048846	axon extension involved in axon guidance	2/385	37/23328	0.124218946	0.23598	0.1418	Cxcl12/Se	2
GO:0071514	GO:0071514	genetic imprinting	2/385	37/23328	0.124218946	0.23598	0.1418	Cdkn1c/H	2
GO:0071709	GO:0071709	membrane assembly	2/385	37/23328	0.124218946	0.23598	0.1418	Anxa2/Ca	2
GO:1902284	GO:1902284	neuron projection extension involved in neuron project	2/385	37/23328	0.124218946	0.23598	0.1418	Cxcl12/Se	2
GO:2000273	GO:2000273	positive regulation of signaling receptor activity	2/385	37/23328	0.124218946	0.23598	0.1418	Reln/Itgb1	2
GO:0048469	GO:0048469	cell maturation	6/385	205/2332	0.124755546	0.23689	0.14234	Pde3a/Kd	6
GO:0021537	GO:0021537	telencephalon development	7/385	252/2332	0.125025696	0.23729	0.14259	Reln/Cxcl	7
GO:0051952	GO:0051952	regulation of amine transport	4/385	116/2332	0.125735796	0.23853	0.14333	Cxcl12/Rg	4
GO:0048511	GO:0048511	rhythmic process	8/385	301/2332	0.126892793	0.24062	0.14459	Id1/Id3/Pt	8
GO:0007260	GO:0007260	tyrosine phosphorylation of STAT protein	3/385	75/23328	0.127107364	0.2407	0.14463	Socs3/Pe	3
GO:0010507	GO:0010507	negative regulation of autophagy	3/385	75/23328	0.127107364	0.2407	0.14463	Nupr1/Ra	3
GO:0042306	GO:0042306	regulation of protein import into nucleus	3/385	75/23328	0.127107364	0.2407	0.14463	Cd36/Tek	3
GO:0048814	GO:0048814	regulation of dendrite morphogenesis	4/385	117/2332	0.128602169	0.24342	0.14627	Reln/Id1/S	4
GO:0001953	GO:0001953	negative regulation of cell-matrix adhesion	2/385	38/23328	0.129755237	0.24461	0.14698	Thbs1/Ac	2
GO:0002861	GO:0002861	regulation of inflammatory response to antigenic stimu	2/385	38/23328	0.129755237	0.24461	0.14698	Cd24a/C3	2
GO:0010661	GO:0010661	positive regulation of muscle cell apoptotic process	2/385	38/23328	0.129755237	0.24461	0.14698	Fndc1/Igf	2
GO:0030851	GO:0030851	granulocyte differentiation	2/385	38/23328	0.129755237	0.24461	0.14698	Rbp1/Cbf	2
GO:0045940	GO:0045940	positive regulation of steroid metabolic process	2/385	38/23328	0.129755237	0.24461	0.14698	Dab2/Apo	2
GO:0051953	GO:0051953	negative regulation of amine transport	2/385	38/23328	0.129755237	0.24461	0.14698	Rgs4/Ptgs	2
GO:0086065	GO:0086065	cell communication involved in cardiac conduction	2/385	38/23328	0.129755237	0.24461	0.14698	Cav1/Gja	2
GO:0090322	GO:0090322	regulation of superoxide metabolic process	2/385	38/23328	0.129755237	0.24461	0.14698	Cd36/Fbl	2
GO:2000463	GO:2000463	positive regulation of excitatory postsynaptic potential	2/385	38/23328	0.129755237	0.24461	0.14698	Reln/Rgs	2
GO:0010675	GO:0010675	regulation of cellular carbohydrate metabolic process	5/385	162/2332	0.130872463	0.2466	0.14818	Nupr1/Igf	5
GO:0000187	GO:0000187	activation of MAPK activity	4/385	119/2332	0.134415944	0.25284	0.15193	Thbs1/Da	4
GO:2000278	GO:2000278	regulation of DNA biosynthetic process	4/385	119/2332	0.134415944	0.25284	0.15193	Pdgfb/Du	4
GO:0006650	GO:0006650	glycerophospholipid metabolic process	7/385	257/2332	0.134464692	0.25284	0.15193	Scarb1/S	7
GO:0010822	GO:0010822	positive regulation of mitochondrion organization	3/385	77/23328	0.134547389	0.25284	0.15193	Kdr/Chch	3
GO:0030433	GO:0030433	ubiquitin-dependent ERAD pathway	3/385	77/23328	0.134547389	0.25284	0.15193	Hsp90b1/	3
GO:0043507	GO:0043507	positive regulation of JUN kinase activity	3/385	77/23328	0.134547389	0.25284	0.15193	Dab2/Trf/	3
GO:0007257	GO:0007257	activation of JUN kinase activity	2/385	39/23328	0.135347653	0.25366	0.15242	Dab2/Trf	2
GO:0033028	GO:0033028	myeloid cell apoptotic process	2/385	39/23328	0.135347653	0.25366	0.15242	Kitl/Anxa1	2
GO:0035850	GO:0035850	epithelial cell differentiation involved in kidney develop	2/385	39/23328	0.135347653	0.25366	0.15242	Pdgfb/Bm	2
GO:0050892	GO:0050892	intestinal absorption	2/385	39/23328	0.135347653	0.25366	0.15242	Cd36/Sca	2
GO:0072523	GO:0072523	purine-containing compound catabolic process	2/385	39/23328	0.135347653	0.25366	0.15242	Gda/Pde2	2
GO:1905710	GO:1905710	positive regulation of membrane permeability	2/385	39/23328	0.135347653	0.25366	0.15242	Chchd10/	2
GO:0016358	GO:0016358	dendrite development	8/385	306/2332	0.135543213	0.25392	0.15258	Reln/Id1/F	8
GO:0030879	GO:0030879	mammary gland development	5/385	164/2332	0.135772669	0.25423	0.15277	Hoxd9/Igf	5
GO:0032886	GO:0032886	regulation of microtubule-based process	7/385	258/2332	0.136393777	0.25528	0.1534	Cdh5/Plk2	7
GO:0006090	GO:0006090	pyruvate metabolic process	4/385	120/2332	0.137362549	0.25698	0.15442	Nupr1/Ald	4
GO:0006606	GO:0006606	protein import into nucleus	5/385	165/2332	0.138253085	0.25831	0.15522	Cd36/Cdk	5
GO:0032088	GO:0032088	negative regulation of NF-kappaB transcription factor a	3/385	78/23328	0.13832283	0.25831	0.15522	Cd200/Pt	3
GO:0046718	GO:0046718	viral entry into host cell	3/385	78/23328	0.13832283	0.25831	0.15522	Cav1/Gas	3
GO:0070228	GO:0070228	regulation of lymphocyte apoptotic process	3/385	78/23328	0.13832283	0.25831	0.15522	Cd24a/Cd	3
GO:0018209	GO:0018209	peptidyl-serine modification	9/385	357/2332	0.138415318	0.25837	0.15525	Bgn/Plk2/	9
GO:0045787	GO:0045787	positive regulation of cell cycle	9/385	358/2332	0.140063349	0.26133	0.15703	Rgcc/Nup	9
GO:0015837	GO:0015837	amine transport	4/385	121/2332	0.140335095	0.26143	0.15709	Cxcl12/Rg	4
GO:1901136	GO:1901136	carbohydrate derivative catabolic process	4/385	121/2332	0.140335095	0.26143	0.15709	Lyve1/Gm	4
GO:0000578	GO:0000578	embryonic axis specification	2/385	40/23328	0.140992848	0.26143	0.15709	Nrarp/Pcs	2
GO:0001562	GO:0001562	response to protozoan	2/385	40/23328	0.140992848	0.26143	0.15709	Ier3/Iigp1	2
GO:0015804	GO:0015804	neutral amino acid transport	2/385	40/23328	0.140992848	0.26143	0.15709	Rgs4/Slc6	2
GO:0030521	GO:0030521	androgen receptor signaling pathway	2/385	40/23328	0.140992848	0.26143	0.15709	Sfrp1/Dab	2
GO:0034105	GO:0034105	positive regulation of tissue remodeling	2/385	40/23328	0.140992848	0.26143	0.15709	Trf/Ahsg	2
GO:0040019	GO:0040019	positive regulation of embryonic development	2/385	40/23328	0.140992848	0.26143	0.15709	Pdgfb/Hs	2
GO:0046006	GO:0046006	regulation of activated T cell proliferation	2/385	40/23328	0.140992848	0.26143	0.15709	Prnp/Cd2	2
GO:0051205	GO:0051205	protein insertion into membrane	2/385	40/23328	0.140992848	0.26143	0.15709	Rtp4/Mal	2
GO:0060325	GO:0060325	face morphogenesis	2/385	40/23328	0.140992848	0.26143	0.15709	Tbx1/Col1	2
GO:0086004	GO:0086004	regulation of cardiac muscle cell contraction	2/385	40/23328	0.140992848	0.26143	0.15709	Cav1/Gja	2
GO:1901021	GO:1901021	positive regulation of calcium ion transmembrane trans	2/385	40/23328	0.140992848	0.26143	0.15709	Bmp4/Atp	2
GO:1902692	GO:1902692	regulation of neuroblast proliferation	2/385	40/23328	0.140992848	0.26143	0.15709	Cd24a/Ptr	2
GO:0051279	GO:0051279	regulation of release of sequestered calcium ion into c	3/385	79/23328	0.142133962	0.26343	0.15829	Aplnr/Thy	3
GO:0034248	GO:0034248	regulation of cellular amide metabolic process	10/385	410/2332	0.142994328	0.26491	0.15918	Vim/Spon	10
GO:0002440	GO:0002440	production of molecular mediator of immune response	9/385	361/2332	0.14506771	0.26863	0.16142	Cd36/Fgl2	9
GO:0050688	GO:0050688	regulation of defense response to virus	3/385	80/23328	0.145979837	0.26984	0.16215	Fgl2/Htra1	3
GO:1901862	GO:1901862	negative regulation of muscle tissue development	3/385	80/23328	0.145979837	0.26984	0.16215	Rgs4/Igfb	3
GO:1901888	GO:1901888	regulation of cell junction assembly	6/385	215/2332	0.146281032	0.26984	0.16215	Thy1/Gap	6
GO:0008286	GO:0008286	insulin receptor signaling pathway	4/385	123/2332	0.146356366	0.26984	0.16215	Socs3/Ptp	4
GO:0046474	GO:0046474	glycerophospholipid biosynthetic process	4/385	123/2332	0.146356366	0.26984	0.16215	Fabp5/Pd	4
GO:0002369	GO:0002369	T cell cytokine production	2/385	41/23328	0.146687568	0.26984	0.16215	Rsad2/B2	2
GO:0008207	GO:0008207	C21-steroid hormone metabolic process	2/385	41/23328	0.146687568	0.26984	0.16215	Egr1/Dab	2
GO:0010470	GO:0010470	regulation of gastrulation	2/385	41/23328	0.146687568	0.26984	0.16215	Col5a2/Sf	2
GO:0045806	GO:0045806	negative regulation of endocytosis	2/385	41/23328	0.146687568	0.26984	0.16215	Cav1/Sh3	2
GO:0046461	GO:0046461	neutral lipid catabolic process	2/385	41/23328	0.146687568	0.26984	0.16215	Mgll/Lpl	2
GO:0046464	GO:0046464	acylglycerol catabolic process	2/385	41/23328	0.146687568	0.26984	0.16215	Mgll/Lpl	2
GO:0071364	GO:0071364	cellular response to epidermal growth factor stimulus	2/385	41/23328	0.146687568	0.26984	0.16215	Zfp36/Da	2
GO:0072595	GO:0072595	maintenance of protein localization in organelle	2/385	41/23328	0.146687568	0.26984	0.16215	Chchd10/	2
GO:0097300	GO:0097300	programmed necrotic cell death	2/385	41/23328	0.146687568	0.26984	0.16215	Slc25a4/C	2
GO:0099084	GO:0099084	postsynaptic specialization organization	2/385	41/23328	0.146687568	0.26984	0.16215	Reln/Gap	2
GO:0042742	GO:0042742	defense response to bacterium	11/385	464/2332	0.147743197	0.27156	0.16318	Cd36/Sta	11
GO:0008544	GO:0008544	epidermis development	8/385	313/2332	0.148141957	0.27156	0.16318	Igfbp5/Zfp	8
GO:0051170	GO:0051170	import into nucleus	5/385	169/2332	0.148371763	0.27156	0.16318	Cd36/Cdk	5
GO:0071897	GO:0071897	DNA biosynthetic process	5/385	169/2332	0.148371763	0.27156	0.16318	Lox/Pdgfb	5
GO:1990778	GO:1990778	protein localization to cell periphery	9/385	363/2332	0.148453706	0.27156	0.16318	Tmem88/	9
GO:0032411	GO:0032411	positive regulation of transporter activity	4/385	124/2332	0.149404261	0.27156	0.16318	Reln/Slc2	4
GO:1905039	GO:1905039	carboxylic acid transmembrane transport	4/385	124/2332	0.149404261	0.27156	0.16318	Rgs4/Thb	4
GO:0010389	GO:0010389	regulation of G2/M transition of mitotic cell cycle	3/385	81/23328	0.149859511	0.27156	0.16318	Fhl1/Cdkn	3
GO:0021675	GO:0021675	nerve development	3/385	81/23328	0.149859511	0.27156	0.16318	Tbx1/Vca	3
GO:0051785	GO:0051785	positive regulation of nuclear division	3/385	81/23328	0.149859511	0.27156	0.16318	Rgcc/Pdg	3
GO:0071347	GO:0071347	cellular response to interleukin-1	3/385	81/23328	0.149859511	0.27156	0.16318	Fn1/Ccl21	3
GO:1904589	GO:1904589	regulation of protein import	3/385	81/23328	0.149859511	0.27156	0.16318	Cd36/Tek	3
GO:0046879	GO:0046879	hormone secretion	9/385	364/2332	0.150161484	0.27156	0.16318	Sfrp1/Cpe	9
GO:0016064	GO:0016064	immunoglobulin mediated immune response	7/385	265/2332	0.15027242	0.27156	0.16318	Fgl2/Irf7/S	7
GO:0044770	GO:0044770	cell cycle phase transition	10/385	415/2332	0.150901773	0.27156	0.16318	Rgcc/Plk2	10
GO:0009268	GO:0009268	response to pH	2/385	42/23328	0.152428655	0.27156	0.16318	Lgmn/Pan	2
GO:0010765	GO:0010765	positive regulation of sodium ion transport	2/385	42/23328	0.152428655	0.27156	0.16318	Sgk1/Atp1	2
GO:0030501	GO:0030501	positive regulation of bone mineralization	2/385	42/23328	0.152428655	0.27156	0.16318	Ptn/Bmp4	2
GO:0046839	GO:0046839	phospholipid dephosphorylation	2/385	42/23328	0.152428655	0.27156	0.16318	Plpp3/Eph	2
GO:0048873	GO:0048873	homeostasis of number of cells within a tissue	2/385	42/23328	0.152428655	0.27156	0.16318	F2r/Nos3	2
GO:0072348	GO:0072348	sulfur compound transport	2/385	42/23328	0.152428655	0.27156	0.16318	Slc26a10/	2
GO:0072583	GO:0072583	clathrin-dependent endocytosis	2/385	42/23328	0.152428655	0.27156	0.16318	Dab2/Sh3	2
GO:2000279	GO:2000279	negative regulation of DNA biosynthetic process	2/385	42/23328	0.152428655	0.27156	0.16318	Dusp1/Cd	2
GO:2000772	GO:2000772	regulation of cellular senescence	2/385	42/23328	0.152428655	0.27156	0.16318	Plk2/B2m	2
GO:0043280	GO:0043280	positive regulation of cysteine-type endopeptidase acti	4/385	125/2332	0.152476439	0.27156	0.16318	Acer2/F2r	4
GO:1902414	GO:1902414	protein localization to cell junction	4/385	125/2332	0.152476439	0.27156	0.16318	Reln/Cdh5	4

GO:1903825	GO:1903825	organic acid transmembrane transport	4/385	125/23328	0.152476439	0.27156	0.16318	Rgs4/Thbs	4
GO:0051251	GO:0051251	positive regulation of lymphocyte activation	10/385	416/23328	0.152509506	0.27156	0.16318	Thy1/Cd3	10
GO:0000050	GO:0000050	urea cycle	1/385	10/23328	0.153332435	0.27156	0.16318	Ass1	1
GO:0002934	GO:0002934	desmosome organization	1/385	10/23328	0.153332435	0.27156	0.16318	Perp	1
GO:0003149	GO:0003149	membranous septum morphogenesis	1/385	10/23328	0.153332435	0.27156	0.16318	Bmp4	1
GO:0003307	GO:0003307	regulation of Wnt signaling pathway involved in heart c	1/385	10/23328	0.153332435	0.27156	0.16318	Sox17	1
GO:0006000	GO:0006000	fructose metabolic process	1/385	10/23328	0.153332435	0.27156	0.16318	Aldoa	1
GO:0006527	GO:0006527	arginine catabolic process	1/385	10/23328	0.153332435	0.27156	0.16318	Nos3	1
GO:0006977	GO:0006977	DNA damage response, signal transduction by p53 cla	1/385	10/23328	0.153332435	0.27156	0.16318	Cdkn1a	1
GO:0009265	GO:0009265	2'-deoxyribonucleotide biosynthetic process	1/385	10/23328	0.153332435	0.27156	0.16318	Cmpk2	1
GO:0010745	GO:0010745	negative regulation of macrophage derived foam cell d	1/385	10/23328	0.153332435	0.27156	0.16318	Nfkbia	1
GO:0010940	GO:0010940	positive regulation of necrotic cell death	1/385	10/23328	0.153332435	0.27156	0.16318	Slc6a6	1
GO:0015865	GO:0015865	purine nucleotide transport	1/385	10/23328	0.153332435	0.27156	0.16318	Slc25a4	1
GO:0015911	GO:0015911	long-chain fatty acid import across plasma membrane	1/385	10/23328	0.153332435	0.27156	0.16318	Thbs1	1
GO:0030049	GO:0030049	muscle filament sliding	1/385	10/23328	0.153332435	0.27156	0.16318	Tpm1	1
GO:0030952	GO:0030952	establishment or maintenance of cytoskeleton polarity	1/385	10/23328	0.153332435	0.27156	0.16318	Aqp1	1
GO:0032815	GO:0032815	negative regulation of natural killer cell activation	1/385	10/23328	0.153332435	0.27156	0.16318	Pglyrp1	1
GO:0032836	GO:0032836	glomerular basement membrane development	1/385	10/23328	0.153332435	0.27156	0.16318	Nid1	1
GO:0033029	GO:0033029	regulation of neutrophil apoptotic process	1/385	10/23328	0.153332435	0.27156	0.16318	Anxa1	1
GO:0033085	GO:0033085	negative regulation of T cell differentiation in thymus	1/385	10/23328	0.153332435	0.27156	0.16318	Bmp4	1
GO:0033572	GO:0033572	transferrin transport	1/385	10/23328	0.153332435	0.27156	0.16318	Myo1b	1
GO:0033689	GO:0033689	negative regulation of osteoblast proliferation	1/385	10/23328	0.153332435	0.27156	0.16318	Sfrp1	1
GO:0034145	GO:0034145	positive regulation of toll-like receptor 4 signaling path	1/385	10/23328	0.153332435	0.27156	0.16318	Lbp	1
GO:0034380	GO:0034380	high-density lipoprotein particle assembly	1/385	10/23328	0.153332435	0.27156	0.16318	Apoe	1
GO:0035907	GO:0035907	dorsal aorta development	1/385	10/23328	0.153332435	0.27156	0.16318	Eng	1
GO:0045654	GO:0045654	positive regulation of megakaryocyte differentiation	1/385	10/23328	0.153332435	0.27156	0.16318	Hmgb2	1
GO:0045869	GO:0045869	negative regulation of single stranded viral RNA replic	1/385	10/23328	0.153332435	0.27156	0.16318	Apobec3	1
GO:0046385	GO:0046385	deoxyribose phosphate biosynthetic process	1/385	10/23328	0.153332435	0.27156	0.16318	Cmpk2	1
GO:0046598	GO:0046598	positive regulation of viral entry into host cell	1/385	10/23328	0.153332435	0.27156	0.16318	Cd74	1
GO:0048703	GO:0048703	embryonic viscerocranium morphogenesis	1/385	10/23328	0.153332435	0.27156	0.16318	Tbx1	1
GO:0048739	GO:0048739	cardiac muscle fiber development	1/385	10/23328	0.153332435	0.27156	0.16318	Bmp4	1
GO:0051006	GO:0051006	positive regulation of lipoprotein lipase activity	1/385	10/23328	0.153332435	0.27156	0.16318	Gpihbp1	1
GO:0051917	GO:0051917	regulation of fibrinolysis	1/385	10/23328	0.153332435	0.27156	0.16318	Thbs1	1
GO:0060513	GO:0060513	prostatic bud formation	1/385	10/23328	0.153332435	0.27156	0.16318	Bmp4	1
GO:0060710	GO:0060710	chorio-allantoic fusion	1/385	10/23328	0.153332435	0.27156	0.16318	Vcam1	1
GO:0060982	GO:0060982	coronary artery morphogenesis	1/385	10/23328	0.153332435	0.27156	0.16318	Tbx1	1
GO:0070244	GO:0070244	negative regulation of thymocyte apoptotic process	1/385	10/23328	0.153332435	0.27156	0.16318	Bmp4	1
GO:0070673	GO:0070673	response to interleukin-18	1/385	10/23328	0.153332435	0.27156	0.16318	Pdgfb	1
GO:0070857	GO:0070857	regulation of bile acid biosynthetic process	1/385	10/23328	0.153332435	0.27156	0.16318	Prox1	1
GO:0071635	GO:0071635	negative regulation of transforming growth factor beta	1/385	10/23328	0.153332435	0.27156	0.16318	Cd24a	1
GO:0072070	GO:0072070	loop of Henle development	1/385	10/23328	0.153332435	0.27156	0.16318	Aqp1	1
GO:0075294	GO:0075294	positive regulation by symbiont of entry into host	1/385	10/23328	0.153332435	0.27156	0.16318	Cd74	1
GO:0090129	GO:0090129	positive regulation of synapse maturation	1/385	10/23328	0.153332435	0.27156	0.16318	Reln	1
GO:0090269	GO:0090269	fibroblast growth factor production	1/385	10/23328	0.153332435	0.27156	0.16318	Rgcc	1
GO:0090270	GO:0090270	regulation of fibroblast growth factor production	1/385	10/23328	0.153332435	0.27156	0.16318	Rgcc	1
GO:0090713	GO:0090713	immunological memory process	1/385	10/23328	0.153332435	0.27156	0.16318	Fgl2	1
GO:0097067	GO:0097067	cellular response to thyroid hormone stimulus	1/385	10/23328	0.153332435	0.27156	0.16318	Lmo2	1
GO:0097119	GO:0097119	postsynaptic density protein 95 clustering	1/385	10/23328	0.153332435	0.27156	0.16318	Reln	1
GO:0097264	GO:0097264	self proteolysis	1/385	10/23328	0.153332435	0.27156	0.16318	Lgmn	1
GO:0140052	GO:0140052	cellular response to oxidised low-density lipoprotein pa	1/385	10/23328	0.153332435	0.27156	0.16318	Cd36	1
GO:1900038	GO:1900038	negative regulation of cellular response to hypoxia	1/385	10/23328	0.153332435	0.27156	0.16318	Cd34	1
GO:1902033	GO:1902033	regulation of hematopoietic stem cell proliferation	1/385	10/23328	0.153332435	0.27156	0.16318	Kitl	1
GO:1902746	GO:1902746	regulation of lens fiber cell differentiation	1/385	10/23328	0.153332435	0.27156	0.16318	Cdkn1c	1
GO:1903238	GO:1903238	positive regulation of leukocyte tethering or rolling	1/385	10/23328	0.153332435	0.27156	0.16318	Selp	1
GO:1905522	GO:1905522	negative regulation of macrophage migration	1/385	10/23328	0.153332435	0.27156	0.16318	Cd200	1
GO:1990535	GO:1990535	neuron projection maintenance	1/385	10/23328	0.153332435	0.27156	0.16318	Prnp	1
GO:2000192	GO:2000192	negative regulation of fatty acid transport	1/385	10/23328	0.153332435	0.27156	0.16318	Thbs1	1
GO:2000551	GO:2000551	regulation of T-helper 2 cell cytokine production	1/385	10/23328	0.153332435	0.27156	0.16318	Rsad2	1
GO:2000774	GO:2000774	positive regulation of cellular senescence	1/385	10/23328	0.153332435	0.27156	0.16318	B2m	1
GO:2000807	GO:2000807	regulation of synaptic vesicle clustering	1/385	10/23328	0.153332435	0.27156	0.16318	Pcdh17	1
GO:2001138	GO:2001138	regulation of phospholipid transport	1/385	10/23328	0.153332435	0.27156	0.16318	Apoe	1
GO:2001140	GO:2001140	positive regulation of phospholipid transport	1/385	10/23328	0.153332435	0.27156	0.16318	Apoe	1
GO:0044409	GO:0044409	entry into host	3/385	82/23328	0.153772039	0.27199	0.16344	Cav1/Gas	3
GO:0060113	GO:0060113	inner ear receptor cell differentiation	3/385	82/23328	0.153772039	0.27199	0.16344	Bmp4/Cth	3
GO:1901019	GO:1901019	regulation of calcium ion transmembrane transporter a	3/385	82/23328	0.153772039	0.27199	0.16344	Bmp4/Atp	3
GO:0044242	GO:0044242	cellular lipid catabolic process	6/385	219/23328	0.155336101	0.27464	0.16503	Mgll/Scarb	6
GO:0007586	GO:0007586	digestion	4/385	126/23328	0.155572475	0.27495	0.16521	Cd36/Sca	4
GO:0008654	GO:0008654	phospholipid biosynthetic process	5/385	172/23328	0.156161337	0.27587	0.16577	Adgrf5/Fa	5
GO:0003333	GO:0003333	amino acid transmembrane transport	3/385	83/23328	0.157716478	0.2785	0.16735	Rgs4/Itgb	3
GO:0001783	GO:0001783	B cell apoptotic process	2/385	43/23328	0.158213037	0.27902	0.16766	Cd24a/Cd	2
GO:0046782	GO:0046782	regulation of viral transcription	2/385	43/23328	0.158213037	0.27902	0.16766	Zfp36/Jun	2
GO:0060122	GO:0060122	inner ear receptor cell stereocilium organization	2/385	43/23328	0.158213037	0.27902	0.16766	Cthrc1/Cli	2
GO:0019724	GO:0019724	B cell mediated immunity	7/385	269/23328	0.158489659	0.27939	0.16789	Fgl2/Irf7/S	7
GO:0043244	GO:0043244	regulation of protein-containing complex disassembly	4/385	128/23328	0.161834419	0.28517	0.17136	Dstn/Nes/	4
GO:0010559	GO:0010559	regulation of glycoprotein biosynthetic process	2/385	44/23328	0.164037733	0.28603	0.17187	Bace2/Ac	2
GO:0034198	GO:0034198	cellular response to amino acid starvation	2/385	44/23328	0.164037733	0.28603	0.17187	Atf3/Cdkn	2
GO:0043403	GO:0043403	skeletal muscle tissue regeneration	2/385	44/23328	0.164037733	0.28603	0.17187	Cd9/Anxa	2
GO:0044091	GO:0044091	membrane biogenesis	2/385	44/23328	0.164037733	0.28603	0.17187	Anxa2/Ca	2
GO:0050919	GO:0050919	negative chemotaxis	2/385	44/23328	0.164037733	0.28603	0.17187	Sema3g/F	2
GO:0070849	GO:0070849	response to epidermal growth factor	2/385	44/23328	0.164037733	0.28603	0.17187	Zfp36/Dab	2
GO:0086002	GO:0086002	cardiac muscle cell action potential involved in contrac	2/385	44/23328	0.164037733	0.28603	0.17187	Cav1/Gja	2
GO:1903523	GO:1903523	negative regulation of blood circulation	2/385	44/23328	0.164037733	0.28603	0.17187	Adm/F2r	2
GO:0009914	GO:0009914	hormone transport	9/385	372/23328	0.16417158	0.28603	0.17187	Sfrp1/Cpe	9
GO:0032273	GO:0032273	positive regulation of protein polymerization	4/385	129/23328	0.164999466	0.28603	0.17187	Gda/Cav1	4
GO:0033138	GO:0033138	positive regulation of peptidyl-serine phosphorylation	4/385	129/23328	0.164999466	0.28603	0.17187	Cav1/Gas	4
GO:0048524	GO:0048524	positive regulation of viral process	3/385	85/23328	0.165697334	0.28603	0.17187	Jun/Apoe/	3
GO:0051937	GO:0051937	catecholamine transport	3/385	85/23328	0.165697334	0.28603	0.17187	Cxcl12/Ly	3
GO:0042593	GO:0042593	glucose homeostasis	7/385	273/23328	0.16690747	0.28603	0.17187	Cd36/Adg	7
GO:0001767	GO:0001767	establishment of lymphocyte polarity	1/385	11/23328	0.167311636	0.28603	0.17187	Cyp26b1	1
GO:0001768	GO:0001768	establishment of T cell polarity	1/385	11/23328	0.167311636	0.28603	0.17187	Cyp26b1	1
GO:0001867	GO:0001867	complement activation, lectin pathway	1/385	11/23328	0.167311636	0.28603	0.17187	Serping1	1
GO:0002679	GO:0002679	respiratory burst involved in defense response	1/385	11/23328	0.167311636	0.28603	0.17187	Lbp	1
GO:0002921	GO:0002921	negative regulation of humoral immune response	1/385	11/23328	0.167311636	0.28603	0.17187	Serping1	1
GO:0002923	GO:0002923	regulation of humoral immune response mediated by c	1/385	11/23328	0.167311636	0.28603	0.17187	Cd55	1
GO:0003139	GO:0003139	secondary heart field specification	1/385	11/23328	0.167311636	0.28603	0.17187	Bmp4	1
GO:0003266	GO:0003266	regulation of secondary heart field cardioblast prolifera	1/385	11/23328	0.167311636	0.28603	0.17187	Tbx1	1
GO:0008300	GO:0008300	isoprenoid catabolic process	1/385	11/23328	0.167311636	0.28603	0.17187	Cyp26b1	1
GO:0009219	GO:0009219	pyrimidine deoxyribonucleotide metabolic process	1/385	11/23328	0.167311636	0.28603	0.17187	Cmpk2	1
GO:0009812	GO:0009812	flavonoid metabolic process	1/385	11/23328	0.167311636	0.28603	0.17187	Cyp1a1	1
GO:0014010	GO:0014010	Schwann cell proliferation	1/385	11/23328	0.167311636	0.28603	0.17187	Pmp22	1
GO:0019227	GO:0019227	neuronal action potential propagation	1/385	11/23328	0.167311636	0.28603	0.17187	Pmp22	1
GO:0019627	GO:0019627	urea metabolic process	1/385	11/23328	0.167311636	0.28603	0.17187	Ass1	1
GO:0021684	GO:0021684	cerebellar granular layer formation	1/385	11/23328	0.167311636	0.28603	0.17187	Prox1	1
GO:0021707	GO:0021707	cerebellar granule cell differentiation	1/385	11/23328	0.167311636	0.28603	0.17187	Prox1	1
GO:0030644	GO:0030644	cellular chloride ion homeostasis	1/385	11/23328	0.167311636	0.28603	0.17187	Ckb	1
GO:0032342	GO:0032342	aldosterone biosynthetic process	1/385	11/23328	0.167311636	0.28603	0.17187	Dab2	1
GO:0032429	GO:0032429	regulation of phospholipase A2 activity	1/385	11/23328	0.167311636	0.28603	0.17187	Anxa1	1
GO:0033084	GO:0033084	regulation of immature T cell proliferation in thymus	1/385	11/23328	0.167311636	0.28603	0.17187	Bmp4	1
GO:0033327	GO:0033327	Leydig cell differentiation	1/385	11/23328	0.167311636	0.28603	0.17187	Dhh	1
GO:0033700	GO:0033700	phospholipid efflux	1/385	11/23328	0.167311636	0.28603	0.17187	Apoe	1
GO:0036005	GO:0036005	response to macrophage colony-stimulating factor	1/385	11/23328	0.167311636	0.28603	0.17187	Pde2a	1
GO:0036006	GO:0036006	cellular response to macrophage colony-stimulating fa	1/385	11/23328	0.167311636	0.28603	0.17187	Pde2a	1
GO:0043619	GO:0043619	regulation of transcription from RNA polymerase II pro	1/385	11/23328	0.167311636	0.28603	0.17187	Cd36	1
GO:0045602	GO:0045602	negative regulation of endothelial cell differentiation	1/385	11/23328	0.167311636	0.28603	0.17187	Id1	1
GO:0048149	GO:0048149	behavioral response to ethanol	1/385	11/23328	0.167311636	0.28603	0.17187	Adh1	1
GO:0048617	GO:0048617	embryonic foregut morphogenesis	1/385	11/23328	0.167311636	0.28603	0.17187	Sox17	1
GO:0051458	GO:0051458	corticotropin secretion	1/385	11/23328	0.167311636	0.28603	0.17187	Aqp1	1
GO:0051561	GO:0051561	positive regulation of mitochondrial calcium ion concer	1/385	11/23328	0.167311636	0.28603	0.17187	Tgm2	1
GO:0055015	GO:0055015	ventricular cardiac muscle cell development	1/385	11/23328	0.167311636	0.28603	0.17187	Prox1	1
GO:0060353	GO:0060353	regulation of cell adhesion molecule production	1/385	11/23328	0.167311636	0.28603	0.17187	Cav1	1
GO:0060693	GO:0060693	regulation of branching involved in salivary gland morp	1/385	11/23328	0.167311636	0.28603	0.17187	Pdgfa	1
GO:0060900	GO:0060900	embryonic camera-type eye formation	1/385	11/23328	0.167311636	0.28603	0.17187	Prox1	1
GO:0061299	GO:0061299	retina vasculature morphogenesis in camera-type eye	1/385	11/23328	0.167311636	0.28603	0.17187	Arhgef15	1
GO:0061309	GO:0061309	cardiac neural crest cell development involved in outfl	1/385	11/23328	0.167311636	0.28603	0.17187	Bmp4	1
GO:0061365	GO:0061365	positive regulation of triglyceride lipase activity	1/385	11/23328	0.167311636	0.28603	0.17187	Gpihbp1	1
GO:0070493	GO:0070493	thrombin-activated receptor signaling pathway	1/385	11/23328	0.167311636	0.28603	0.17187	F2r	1
GO:0071600	GO:0071600	otic vesicle morphogenesis	1/385	11/23328	0.167311636	0.28603	0.17187	Tbx1	1
GO:0072178	GO:0072178	nephric duct morphogenesis	1/385	11/23328	0.167311636	0.28603	0.17187	Bmp4	1
GO:0072431	GO:0072431	signal transduction involved in mitotic G1 DNA damag	1/385	11/23328	0.167311636	0.28603	0.17187	Cdkn1a	1

GO:0086016	GO:0086016	AV node cell action potential	1/385	11/23328	0.167311636	0.28603	0.17187	Gja5	1
GO:0086027	GO:0086027	AV node cell to bundle of His cell signaling	1/385	11/23328	0.167311636	0.28603	0.17187	Gja5	1
GO:0086103	GO:0086103	G protein-coupled receptor signaling pathway involved	1/385	11/23328	0.167311636	0.28603	0.17187	Cav1	1
GO:0090037	GO:0090037	positive regulation of protein kinase C signaling	1/385	11/23328	0.167311636	0.28603	0.17187	Flt4	1
GO:0098664	GO:0098664	G protein-coupled serotonin receptor signaling pathwa	1/385	11/23328	0.167311636	0.28603	0.17187	Htr2b	1
GO:0098870	GO:0098870	action potential propagation	1/385	11/23328	0.167311636	0.28603	0.17187	Pmp22	1
GO:0099612	GO:0099612	protein localization to axon	1/385	11/23328	0.167311636	0.28603	0.17187	Mal	1
GO:1901030	GO:1901030	positive regulation of mitochondrial outer membrane pe	1/385	11/23328	0.167311636	0.28603	0.17187	Chchd10	1
GO:1901142	GO:1901142	insulin metabolic process	1/385	11/23328	0.167311636	0.28603	0.17187	Cpe	1
GO:1901339	GO:1901339	regulation of store-operated calcium channel activity	1/385	11/23328	0.167311636	0.28603	0.17187	Bmp4	1
GO:1902166	GO:1902166	negative regulation of intrinsic apoptotic signaling path	1/385	11/23328	0.167311636	0.28603	0.17187	Cd74	1
GO:1902400	GO:1902400	intracellular signal transduction involved in G1 DNA da	1/385	11/23328	0.167311636	0.28603	0.17187	Cdkn1a	1
GO:1902548	GO:1902548	negative regulation of cellular response to vascular en	1/385	11/23328	0.167311636	0.28603	0.17187	Dcn	1
GO:1903365	GO:1903365	regulation of fear response	1/385	11/23328	0.167311636	0.28603	0.17187	Apoe	1
GO:1903867	GO:1903867	extraembryonic membrane development	1/385	11/23328	0.167311636	0.28603	0.17187	Htra1	1
GO:1903911	GO:1903911	positive regulation of receptor clustering	1/385	11/23328	0.167311636	0.28603	0.17187	Ptn	1
GO:1990440	GO:1990440	positive regulation of transcription from RNA polymera	1/385	11/23328	0.167311636	0.28603	0.17187	Atf3	1
GO:2000822	GO:2000822	regulation of behavioral fear response	1/385	11/23328	0.167311636	0.28603	0.17187	Apoe	1
GO:0042177	GO:0042177	negative regulation of protein catabolic process	4/385	130/2332(	0.168186655	0.28729	0.17263	Timp4/An:	4
GO:0050852	GO:0050852	T cell receptor signaling pathway	4/385	130/2332(	0.168186655	0.28729	0.17263	Thy1/Prnp	4
GO:0033500	GO:0033500	carbohydrate homeostasis	7/385	274/2332(	0.169042483	0.28863	0.17344	Cd36/Adg	7
GO:1901617	GO:1901617	organic hydroxy compound biosynthetic process	6/385	225/2332(	0.169367067	0.2888	0.17354	Pltp/Cyb5	6
GO:0031646	GO:0031646	positive regulation of nervous system process	3/385	86/23328	0.16973188	0.2888	0.17354	Reln/Rgs4	3
GO:0035050	GO:0035050	embryonic heart tube development	3/385	86/23328	0.16973188	0.2888	0.17354	Sox17/En	3
GO:1905897	GO:1905897	regulation of response to endoplasmic reticulum stress	3/385	86/23328	0.16973188	0.2888	0.17354	Nupr1/Clu	3
GO:0003156	GO:0003156	regulation of animal organ formation	2/385	45/23328	0.169899849	0.2888	0.17354	Tbx1/Bmp	2
GO:0021696	GO:0021696	cerebellar cortex morphogenesis	2/385	45/23328	0.169899849	0.2888	0.17354	Serpine2/I	2
GO:0044088	GO:0044088	regulation of vacuole organization	2/385	45/23328	0.169899849	0.2888	0.17354	Nupr1/Anx	2
GO:0045923	GO:0045923	positive regulation of fatty acid metabolic process	2/385	45/23328	0.169899849	0.2888	0.17354	Cd74/Anx	2
GO:0071526	GO:0071526	semaphorin-plexin signaling pathway	2/385	45/23328	0.169899849	0.2888	0.17354	Ncam1/Se	2
GO:0097178	GO:0097178	ruffle assembly	2/385	45/23328	0.169899849	0.2888	0.17354	Cav1/Kan	2
GO:1901031	GO:1901031	regulation of response to reactive oxygen species	2/385	45/23328	0.169899849	0.2888	0.17354	Cd36/Fblr	2
GO:0090407	GO:0090407	organophosphate biosynthetic process	10/385	427/2332(	0.170756559	0.29014	0.17434	Adgrf5/Fa	10
GO:0007368	GO:0007368	determination of left/right symmetry	4/385	131/2332(	0.171395549	0.29111	0.17492	Tbx1/Sox:	4
GO:0034763	GO:0034763	negative regulation of transmembrane transport	4/385	132/2332(	0.174625712	0.29647	0.17815	Rgs4/Fab	4
GO:0010518	GO:0010518	positive regulation of phospholipase activity	2/385	46/23328	0.175796572	0.29774	0.17891	Flt1/Htr2b	2
GO:0019083	GO:0019083	viral transcription	2/385	46/23328	0.175796572	0.29774	0.17891	Zfp36/Jun	2
GO:0043616	GO:0043616	keratinocyte proliferation	2/385	46/23328	0.175796572	0.29774	0.17891	Klk8/Zfp3(	2
GO:0045740	GO:0045740	positive regulation of DNA replication	2/385	46/23328	0.175796572	0.29774	0.17891	Pdgfb/Jun	2
GO:0060323	GO:0060323	head morphogenesis	2/385	46/23328	0.175796572	0.29774	0.17891	Tbx1/Col1	2
GO:1902667	GO:1902667	regulation of axon guidance	2/385	46/23328	0.175796572	0.29774	0.17891	Cxcl12/Se	2
GO:0010827	GO:0010827	regulation of glucose transmembrane transport	3/385	88/23328	0.177884559	0.29974	0.18011	Fabp5/C3	3
GO:1901992	GO:1901992	positive regulation of mitotic cell cycle phase transition	3/385	88/23328	0.177884559	0.29974	0.18011	Rgcc/Cyp	3
GO:0032386	GO:0032386	regulation of intracellular transport	8/385	329/2332(	0.178963953	0.29974	0.18011	Cd36/Prn	8
GO:0006790	GO:0006790	sulfur compound metabolic process	7/385	279/2332(	0.179895461	0.29974	0.18011	Bgn/Mical	7
GO:0002329	GO:0002329	pre-B cell differentiation	1/385	12/23328	0.181060618	0.29974	0.18011	Cd24a	1
GO:0003129	GO:0003129	heart induction	1/385	12/23328	0.181060618	0.29974	0.18011	Bmp4	1
GO:0003263	GO:0003263	cardioblast proliferation	1/385	12/23328	0.181060618	0.29974	0.18011	Tbx1	1
GO:0003264	GO:0003264	regulation of cardioblast proliferation	1/385	12/23328	0.181060618	0.29974	0.18011	Tbx1	1
GO:0003337	GO:0003337	mesenchymal to epithelial transition involved in metan	1/385	12/23328	0.181060618	0.29974	0.18011	Bmp4	1
GO:0006123	GO:0006123	mitochondrial electron transport, cytochrome c to oxyg	1/385	12/23328	0.181060618	0.29974	0.18011	Cox4i2	1
GO:0006705	GO:0006705	mineralocorticoid biosynthetic process	1/385	12/23328	0.181060618	0.29974	0.18011	Dab2	1
GO:0007210	GO:0007210	serotonin receptor signaling pathway	1/385	12/23328	0.181060618	0.29974	0.18011	Htr2b	1
GO:0007440	GO:0007440	foregut morphogenesis	1/385	12/23328	0.181060618	0.29974	0.18011	Sox17	1
GO:0007501	GO:0007501	mesodermal cell fate specification	1/385	12/23328	0.181060618	0.29974	0.18011	Sfrp2	1
GO:0010870	GO:0010870	positive regulation of receptor biosynthetic process	1/385	12/23328	0.181060618	0.29974	0.18011	Edn1	1
GO:0010872	GO:0010872	regulation of cholesterol esterification	1/385	12/23328	0.181060618	0.29974	0.18011	Apoe	1
GO:0019646	GO:0019646	aerobic electron transport chain	1/385	12/23328	0.181060618	0.29974	0.18011	Cox4i2	1
GO:0030388	GO:0030388	fructose 1,6-bisphosphate metabolic process	1/385	12/23328	0.181060618	0.29974	0.18011	Aldoa	1
GO:0032341	GO:0032341	aldosterone metabolic process	1/385	12/23328	0.181060618	0.29974	0.18011	Dab2	1
GO:0032754	GO:0032754	positive regulation of interleukin-5 production	1/385	12/23328	0.181060618	0.29974	0.18011	Il33	1
GO:0032825	GO:0032825	positive regulation of natural killer cell differentiation	1/385	12/23328	0.181060618	0.29974	0.18011	Gas6	1
GO:0033080	GO:0033080	immature T cell proliferation in thymus	1/385	12/23328	0.181060618	0.29974	0.18011	Bmp4	1
GO:0033603	GO:0033603	positive regulation of dopamine secretion	1/385	12/23328	0.181060618	0.29974	0.18011	Cxcl12	1
GO:0034392	GO:0034392	negative regulation of smooth muscle cell apoptotic pr	1/385	12/23328	0.181060618	0.29974	0.18011	Edn1	1
GO:0035437	GO:0035437	maintenance of protein localization in endoplasmic reti	1/385	12/23328	0.181060618	0.29974	0.18011	Hspa5	1
GO:0035457	GO:0035457	cellular response to interferon-alpha	1/385	12/23328	0.181060618	0.29974	0.18011	Gas6	1
GO:0036295	GO:0036295	cellular response to increased oxygen levels	1/385	12/23328	0.181060618	0.29974	0.18011	Cav1	1
GO:0042363	GO:0042363	fat-soluble vitamin catabolic process	1/385	12/23328	0.181060618	0.29974	0.18011	Cyp26b1	1
GO:0042447	GO:0042447	hormone catabolic process	1/385	12/23328	0.181060618	0.29974	0.18011	Ece1	1
GO:0043922	GO:0043922	negative regulation by host of viral transcription	1/385	12/23328	0.181060618	0.29974	0.18011	Jun	1
GO:0045091	GO:0045091	regulation of single stranded viral RNA replication via (	1/385	12/23328	0.181060618	0.29974	0.18011	Apobec3	1
GO:0045348	GO:0045348	positive regulation of MHC class II biosynthetic proces	1/385	12/23328	0.181060618	0.29974	0.18011	Il33	1
GO:0045625	GO:0045625	regulation of T-helper 1 cell differentiation	1/385	12/23328	0.181060618	0.29974	0.18011	Anxa1	1
GO:0046462	GO:0046462	monoacylglycerol metabolic process	1/385	12/23328	0.181060618	0.29974	0.18011	Mgll	1
GO:0051024	GO:0051024	positive regulation of immunoglobulin secretion	1/385	12/23328	0.181060618	0.29974	0.18011	Il33	1
GO:0060159	GO:0060159	regulation of dopamine receptor signaling pathway	1/385	12/23328	0.181060618	0.29974	0.18011	Rgs4	1
GO:0060433	GO:0060433	bronchus development	1/385	12/23328	0.181060618	0.29974	0.18011	Bmp4	1
GO:0060439	GO:0060439	trachea morphogenesis	1/385	12/23328	0.181060618	0.29974	0.18011	Bmp4	1
GO:0060502	GO:0060502	epithelial cell proliferation involved in lung morphogen	1/385	12/23328	0.181060618	0.29974	0.18011	Bmp4	1
GO:0060525	GO:0060525	prostate glandular acinus development	1/385	12/23328	0.181060618	0.29974	0.18011	Sfrp1	1
GO:0060872	GO:0060872	semicircular canal development	1/385	12/23328	0.181060618	0.29974	0.18011	Tbx1	1
GO:0061744	GO:0061744	motor behavior	1/385	12/23328	0.181060618	0.29974	0.18011	Pmp22	1
GO:0070444	GO:0070444	oligodendrocyte progenitor proliferation	1/385	12/23328	0.181060618	0.29974	0.18011	Trf	1
GO:0070445	GO:0070445	regulation of oligodendrocyte progenitor proliferation	1/385	12/23328	0.181060618	0.29974	0.18011	Trf	1
GO:0071318	GO:0071318	cellular response to ATP	1/385	12/23328	0.181060618	0.29974	0.18011	Hsp90b1	1
GO:0071941	GO:0071941	nitrogen cycle metabolic process	1/385	12/23328	0.181060618	0.29974	0.18011	Ass1	1
GO:0072044	GO:0072044	collecting duct development	1/385	12/23328	0.181060618	0.29974	0.18011	Bmp4	1
GO:0072176	GO:0072176	nephric duct development	1/385	12/23328	0.181060618	0.29974	0.18011	Bmp4	1
GO:0072584	GO:0072584	caveolin-mediated endocytosis	1/385	12/23328	0.181060618	0.29974	0.18011	Cav1	1
GO:0090209	GO:0090209	negative regulation of triglyceride metabolic process	1/385	12/23328	0.181060618	0.29974	0.18011	Apoe	1
GO:0090343	GO:0090343	positive regulation of cell aging	1/385	12/23328	0.181060618	0.29974	0.18011	B2m	1
GO:0097113	GO:0097113	AMPA glutamate receptor clustering	1/385	12/23328	0.181060618	0.29974	0.18011	Apoe	1
GO:0097709	GO:0097709	connective tissue replacement	1/385	12/23328	0.181060618	0.29974	0.18011	F2r	1
GO:0150079	GO:0150079	negative regulation of neuroinflammatory response	1/385	12/23328	0.181060618	0.29974	0.18011	Cd200	1
GO:1902510	GO:1902510	regulation of apoptotic DNA fragmentation	1/385	12/23328	0.181060618	0.29974	0.18011	Igfbp3	1
GO:1903800	GO:1903800	positive regulation of production of miRNAs involved ir	1/385	12/23328	0.181060618	0.29974	0.18011	Bmp4	1
GO:2000402	GO:2000402	negative regulation of lymphocyte migration	1/385	12/23328	0.181060618	0.29974	0.18011	Cd200	1
GO:0032814	GO:0032814	regulation of natural killer cell activation	2/385	47/23328	0.181725175	0.30024	0.18041	Gas6/Pgly	2
GO:0043902	GO:0043902	positive regulation of multi-organism process	2/385	47/23328	0.181725175	0.30024	0.18041	Pde3a/Pla	2
GO:0045332	GO:0045332	phospholipid translocation	2/385	47/23328	0.181725175	0.30024	0.18041	Kcnn4/Ab	2
GO:0050974	GO:0050974	detection of mechanical stimulus involved in sensory p	2/385	47/23328	0.181725175	0.30024	0.18041	Cxcl12/Se	2
GO:0060964	GO:0060964	regulation of gene silencing by miRNA	2/385	47/23328	0.181725175	0.30024	0.18041	Zfp36/Bm	2
GO:0042490	GO:0042490	mechanoreceptor differentiation	3/385	89/23328	0.182000846	0.30034	0.18047	Bmp4/Cth	3
GO:0042770	GO:0042770	signal transduction in response to DNA damage	3/385	89/23328	0.182000846	0.30034	0.18047	Cdkn1a/A	3
GO:0070301	GO:0070301	cellular response to hydrogen peroxide	3/385	89/23328	0.182000846	0.30034	0.18047	Lcn2/Aqp:	3
GO:0051701	GO:0051701	interaction with host	4/385	135/2332(	0.184439418	0.30425	0.18282	Cav1/Gas	4
GO:0006672	GO:0006672	ceramide metabolic process	3/385	90/23328	0.186142542	0.3067	0.18429	Plpp3/Gm	3
GO:0046888	GO:0046888	negative regulation of hormone secretion	3/385	90/23328	0.186142542	0.3067	0.18429	Sfrp1/Igfb	3
GO:0070509	GO:0070509	calcium ion import	3/385	90/23328	0.186142542	0.3067	0.18429	Cxcl12/Pr	3
GO:0000266	GO:0000266	mitochondrial fission	2/385	48/23328	0.187683007	0.30863	0.18545	Kdr/Dcn	2
GO:0038066	GO:0038066	p38MAPK cascade	2/385	48/23328	0.187683007	0.30863	0.18545	Dusp1/Zfp	2
GO:0050798	GO:0050798	activated T cell proliferation	2/385	48/23328	0.187683007	0.30863	0.18545	Prnp/Cd24	2
GO:1990573	GO:1990573	potassium ion import across plasma membrane	2/385	48/23328	0.187683007	0.30863	0.18545	Abcc9/Atp	2
GO:1990928	GO:1990928	response to amino acid starvation	2/385	48/23328	0.187683007	0.30863	0.18545	Atf3/Cdkn	2
GO:0030534	GO:0030534	adult behavior	5/385	184/2332(	0.188903157	0.30904	0.1857	Cxcl12/Pc	5
GO:0032388	GO:0032388	positive regulation of intracellular transport	5/385	184/2332(	0.188903157	0.30904	0.1857	Prnp/Anxa	5
GO:1900407	GO:1900407	regulation of cellular response to oxidative stress	3/385	91/23328	0.190308736	0.30904	0.1857	Cd36/Hsp	3
GO:0006695	GO:0006695	cholesterol biosynthetic process	2/385	49/23328	0.193667498	0.30904	0.1857	Cyb5r3/Ap	2
GO:0010332	GO:0010332	response to gamma radiation	2/385	49/23328	0.193667498	0.30904	0.1857	Cdkn1a/E	2
GO:0021532	GO:0021532	neural tube patterning	2/385	49/23328	0.193667498	0.30904	0.1857	Sox17/Bm	2
GO:0032941	GO:0032941	secretion by tissue	2/385	49/23328	0.193667498	0.30904	0.1857	Kcnn4/Aq	2
GO:0034204	GO:0034204	lipid translocation	2/385	49/23328	0.193667498	0.30904	0.1857	Kcnn4/Ab	2
GO:0042755	GO:0042755	eating behavior	2/385	49/23328	0.193667498	0.30904	0.1857	Cd36/Tcf1	2
GO:0090102	GO:0090102	cochlea development	2/385	49/23328	0.193667498	0.30904	0.1857	Tbx1/Cthr	2
GO:0090342	GO:0090342	regulation of cell aging	2/385	49/23328	0.193667498	0.30904	0.1857	Plk2/B2m	2
GO:1902653	GO:1902653	secondary alcohol biosynthetic process	2/385	49/23328	0.193667498	0.30904	0.1857	Cyb5r3/Ap	2
GO:1903018	GO:1903018	regulation of glycoprotein metabolic process	2/385	49/23328	0.193667498	0.30904	0.1857	Bace2/Ac	2
GO:0001508	GO:0001508	action potential	4/385	138/2332(	0.194428676	0.30904	0.1857	Cd36/Cav	4

GO:0042471	GO:0042471	ear morphogenesis	4/385	138/23328	0.194428676	0.30904	0.1857	Tbx1/Cthr	4
GO:0046165	GO:0046165	alcohol biosynthetic process	4/385	138/23328	0.194428676	0.30904	0.1857	Cyb5r3/Da	4
GO:0002832	GO:0002832	negative regulation of response to biotic stimulus	3/385	92/23328	0.194498524	0.30904	0.1857	Fgl2/Htra1	3
GO:0045685	GO:0045685	regulation of glial cell differentiation	3/385	92/23328	0.194498524	0.30904	0.1857	Ptn/Serpir	3
GO:0001542	GO:0001542	ovulation from ovarian follicle	1/385	13/23328	0.194583163	0.30904	0.1857	Nos3	1
GO:0001781	GO:0001781	neutrophil apoptotic process	1/385	13/23328	0.194583163	0.30904	0.1857	Anxa1	1
GO:0003174	GO:0003174	mitral valve development	1/385	13/23328	0.194583163	0.30904	0.1857	Gja5	1
GO:0003306	GO:0003306	Wnt signaling pathway involved in heart development	1/385	13/23328	0.194583163	0.30904	0.1857	Sox17	1
GO:0006983	GO:0006983	ER overload response	1/385	13/23328	0.194583163	0.30904	0.1857	Hspa5	1
GO:0007320	GO:0007320	insemination	1/385	13/23328	0.194583163	0.30904	0.1857	Serpine2	1
GO:0008212	GO:0008212	mineralocorticoid metabolic process	1/385	13/23328	0.194583163	0.30904	0.1857	Dab2	1
GO:0009148	GO:0009148	pyrimidine nucleoside triphosphate biosynthetic proces	1/385	13/23328	0.194583163	0.30904	0.1857	Cmpk2	1
GO:0009263	GO:0009263	deoxyribonucleotide biosynthetic process	1/385	13/23328	0.194583163	0.30904	0.1857	Cmpk2	1
GO:0009950	GO:0009950	dorsal/ventral axis specification	1/385	13/23328	0.194583163	0.30904	0.1857	Sfrp1	1
GO:0010359	GO:0010359	regulation of anion channel activity	1/385	13/23328	0.194583163	0.30904	0.1857	Prnp	1
GO:0010988	GO:0010988	regulation of low-density lipoprotein particle clearance	1/385	13/23328	0.194583163	0.30904	0.1857	Anxa2	1
GO:0015816	GO:0015816	glycine transport	1/385	13/23328	0.194583163	0.30904	0.1857	Rgs4	1
GO:0021670	GO:0021670	lateral ventricle development	1/385	13/23328	0.194583163	0.30904	0.1857	Aqp1	1
GO:0021794	GO:0021794	thalamus development	1/385	13/23328	0.194583163	0.30904	0.1857	Ncam1	1
GO:0030836	GO:0030836	positive regulation of actin filament depolymerization	1/385	13/23328	0.194583163	0.30904	0.1857	Dstn	1
GO:0032026	GO:0032026	response to magnesium ion	1/385	13/23328	0.194583163	0.30904	0.1857	Thbs1	1
GO:0032372	GO:0032372	negative regulation of sterol transport	1/385	13/23328	0.194583163	0.30904	0.1857	Apoe	1
GO:0032375	GO:0032375	negative regulation of cholesterol transport	1/385	13/23328	0.194583163	0.30904	0.1857	Apoe	1
GO:0032486	GO:0032486	Rap protein signal transduction	1/385	13/23328	0.194583163	0.30904	0.1857	Plk2	1
GO:0032488	GO:0032488	Cdc42 protein signal transduction	1/385	13/23328	0.194583163	0.30904	0.1857	Apoe	1
GO:0033083	GO:0033083	regulation of immature T cell proliferation	1/385	13/23328	0.194583163	0.30904	0.1857	Bmp4	1
GO:0034374	GO:0034374	low-density lipoprotein particle remodeling	1/385	13/23328	0.194583163	0.30904	0.1857	Apoe	1
GO:0035810	GO:0035810	positive regulation of urine volume	1/385	13/23328	0.194583163	0.30904	0.1857	Edn1	1
GO:0038166	GO:0038166	angiotensin-activated signaling pathway	1/385	13/23328	0.194583163	0.30904	0.1857	Cav1	1
GO:0039692	GO:0039692	single stranded viral RNA replication via double strand	1/385	13/23328	0.194583163	0.30904	0.1857	Apobec3	1
GO:0042693	GO:0042693	muscle cell fate commitment	1/385	13/23328	0.194583163	0.30904	0.1857	Tbx1	1
GO:0042994	GO:0042994	cytoplasmic sequestering of transcription factor	1/385	13/23328	0.194583163	0.30904	0.1857	Nfkbia	1
GO:0044090	GO:0044090	positive regulation of vacuole organization	1/385	13/23328	0.194583163	0.30904	0.1857	Anxa2	1
GO:0045161	GO:0045161	neuronal ion channel clustering	1/385	13/23328	0.194583163	0.30904	0.1857	Pmp22	1
GO:0045628	GO:0045628	regulation of T-helper 2 cell differentiation	1/385	13/23328	0.194583163	0.30904	0.1857	Anxa1	1
GO:0045906	GO:0045906	negative regulation of vasoconstriction	1/385	13/23328	0.194583163	0.30904	0.1857	Adm	1
GO:0046007	GO:0046007	negative regulation of activated T cell proliferation	1/385	13/23328	0.194583163	0.30904	0.1857	Prnp	1
GO:0046512	GO:0046512	sphingosine biosynthetic process	1/385	13/23328	0.194583163	0.30904	0.1857	Acer2	1
GO:0046520	GO:0046520	sphingoid biosynthetic process	1/385	13/23328	0.194583163	0.30904	0.1857	Acer2	1
GO:0048012	GO:0048012	hepatocyte growth factor receptor signaling pathway	1/385	13/23328	0.194583163	0.30904	0.1857	Esm1	1
GO:0048548	GO:0048548	regulation of pinocytosis	1/385	13/23328	0.194583163	0.30904	0.1857	Cav1	1
GO:0048711	GO:0048711	positive regulation of astrocyte differentiation	1/385	13/23328	0.194583163	0.30904	0.1857	Serpine2	1
GO:0051639	GO:0051639	actin filament network formation	1/385	13/23328	0.194583163	0.30904	0.1857	Actn1	1
GO:0055062	GO:0055062	phosphate ion homeostasis	1/385	13/23328	0.194583163	0.30904	0.1857	Pthlh	1
GO:0055119	GO:0055119	relaxation of cardiac muscle	1/385	13/23328	0.194583163	0.30904	0.1857	Atp1b1	1
GO:0060272	GO:0060272	embryonic skeletal joint morphogenesis	1/385	13/23328	0.194583163	0.30904	0.1857	Bmp4	1
GO:0060340	GO:0060340	positive regulation of type I interferon-mediated signali	1/385	13/23328	0.194583163	0.30904	0.1857	Irf7	1
GO:0060352	GO:0060352	cell adhesion molecule production	1/385	13/23328	0.194583163	0.30904	0.1857	Cav1	1
GO:0060394	GO:0060394	negative regulation of pathway-restricted SMAD protei	1/385	13/23328	0.194583163	0.30904	0.1857	Eng	1
GO:0060601	GO:0060601	lateral sprouting from an epithelium	1/385	13/23328	0.194583163	0.30904	0.1857	Bmp4	1
GO:0061052	GO:0061052	negative regulation of cell growth involved in cardiac m	1/385	13/23328	0.194583163	0.30904	0.1857	Rgs4	1
GO:0061418	GO:0061418	regulation of transcription from RNA polymerase II pro	1/385	13/23328	0.194583163	0.30904	0.1857	Egr1	1
GO:0070431	GO:0070431	nucleotide-binding oligomerization domain containing 2	1/385	13/23328	0.194583163	0.30904	0.1857	Nfkbia	1
GO:0071285	GO:0071285	cellular response to lithium ion	1/385	13/23328	0.194583163	0.30904	0.1857	Fabp4	1
GO:0071295	GO:0071295	cellular response to vitamin	1/385	13/23328	0.194583163	0.30904	0.1857	Gas6	1
GO:0071679	GO:0071679	commissural neuron axon guidance	1/385	13/23328	0.194583163	0.30904	0.1857	Ncam1	1
GO:0072505	GO:0072505	divalent inorganic anion homeostasis	1/385	13/23328	0.194583163	0.30904	0.1857	Pthlh	1
GO:0072506	GO:0072506	trivalent inorganic anion homeostasis	1/385	13/23328	0.194583163	0.30904	0.1857	Pthlh	1
GO:0086067	GO:0086067	AV node cell to bundle of His cell communication	1/385	13/23328	0.194583163	0.30904	0.1857	Gja5	1
GO:0097090	GO:0097090	presynaptic membrane organization	1/385	13/23328	0.194583163	0.30904	0.1857	Apoe	1
GO:0097201	GO:0097201	negative regulation of transcription from RNA polymera	1/385	13/23328	0.194583163	0.30904	0.1857	Jun	1
GO:0097577	GO:0097577	sequestering of iron ion	1/385	13/23328	0.194583163	0.30904	0.1857	Lcn2	1
GO:0097688	GO:0097688	glutamate receptor clustering	1/385	13/23328	0.194583163	0.30904	0.1857	Apoe	1
GO:0099558	GO:0099558	maintenance of synapse structure	1/385	13/23328	0.194583163	0.30904	0.1857	Chchd10	1
GO:0110154	GO:0110154	RNA decapping	1/385	13/23328	0.194583163	0.30904	0.1857	Zfp36	1
GO:0110156	GO:0110156	methylguanosine-cap decapping	1/385	13/23328	0.194583163	0.30904	0.1857	Zfp36	1
GO:1901163	GO:1901163	regulation of trophoblast cell migration	1/385	13/23328	0.194583163	0.30904	0.1857	Arhgdib	1
GO:1901201	GO:1901201	regulation of extracellular matrix assembly	1/385	13/23328	0.194583163	0.30904	0.1857	Rgcc	1
GO:1901678	GO:1901678	iron coordination entity transport	1/385	13/23328	0.194583163	0.30904	0.1857	Lcn2	1
GO:1903651	GO:1903651	positive regulation of cytoplasmic transport	1/385	13/23328	0.194583163	0.30904	0.1857	Dab2	1
GO:1903817	GO:1903817	negative regulation of voltage-gated potassium channe	1/385	13/23328	0.194583163	0.30904	0.1857	Cav1	1
GO:1903862	GO:1903862	positive regulation of oxidative phosphorylation	1/385	13/23328	0.194583163	0.30904	0.1857	Nupr1	1
GO:1904251	GO:1904251	regulation of bile acid metabolic process	1/385	13/23328	0.194583163	0.30904	0.1857	Prox1	1
GO:2000833	GO:2000833	positive regulation of steroid hormone secretion	1/385	13/23328	0.194583163	0.30904	0.1857	Dab2	1
GO:2001054	GO:2001054	negative regulation of mesenchymal cell apoptotic proc	1/385	13/23328	0.194583163	0.30904	0.1857	Tbx1	1
GO:2001223	GO:2001223	negative regulation of neuron migration	1/385	13/23328	0.194583163	0.30904	0.1857	Col3a1	1
GO:1901361	GO:1901361	organic cyclic compound catabolic process	10/385	442/23328	0.197217498	0.31311	0.18814	Scarb1/Vi	10
GO:0033077	GO:0033077	T cell differentiation in thymus	3/385	93/23328	0.198711008	0.31536	0.1895	B2m/Cd74	3
GO:0008038	GO:0008038	neuron recognition	2/385	50/23328	0.199676155	0.31558	0.18963	Gap43/Nc	2
GO:0018198	GO:0018198	peptidyl-cysteine modification	2/385	50/23328	0.199676155	0.31558	0.18963	S100a8/S	2
GO:0032309	GO:0032309	icosanoid secretion	2/385	50/23328	0.199676155	0.31558	0.18963	Edn1/Anx	2
GO:0043392	GO:0043392	negative regulation of DNA binding	2/385	50/23328	0.199676155	0.31558	0.18963	Id1/Jun	2
GO:0060147	GO:0060147	regulation of posttranscriptional gene silencing	2/385	50/23328	0.199676155	0.31558	0.18963	Zfp36/Bm	2
GO:0060966	GO:0060966	regulation of gene silencing by RNA	2/385	50/23328	0.199676155	0.31558	0.18963	Zfp36/Bm	2
GO:0061005	GO:0061005	cell differentiation involved in kidney development	2/385	50/23328	0.199676155	0.31558	0.18963	Pdgfb/Bm	2
GO:0070169	GO:0070169	positive regulation of biomineral tissue development	2/385	50/23328	0.199676155	0.31558	0.18963	Ptn/Bmp4	2
GO:0070229	GO:0070229	negative regulation of lymphocyte apoptotic process	2/385	50/23328	0.199676155	0.31558	0.18963	Cd74/Bmp	2
GO:0110151	GO:0110151	positive regulation of biomineralization	2/385	50/23328	0.199676155	0.31558	0.18963	Ptn/Bmp4	2
GO:1902475	GO:1902475	L-alpha-amino acid transmembrane transport	2/385	50/23328	0.199676155	0.31558	0.18963	Rgs4/Itgb	2
GO:0009855	GO:0009855	determination of bilateral symmetry	4/385	140/23328	0.201179793	0.31771	0.19091	Tbx1/Sox	4
GO:0046330	GO:0046330	positive regulation of JNK cascade	4/385	140/23328	0.201179793	0.31771	0.19091	Dab2/Trf/I	4
GO:0009408	GO:0009408	response to heat	3/385	94/23328	0.202945295	0.32038	0.19251	Cd34/Pmp	3
GO:0000288	GO:0000288	nuclear-transcribed mRNA catabolic process, deadeny	2/385	51/23328	0.205706557	0.32038	0.19252	Btg2/Zfp3	2
GO:0030901	GO:0030901	midbrain development	2/385	51/23328	0.205706557	0.32038	0.19252	Sfrp1/Sfrp	2
GO:0035088	GO:0035088	establishment or maintenance of apical/basal cell pola	2/385	51/23328	0.205706557	0.32038	0.19252	Tcf15/Fsc	2
GO:0035137	GO:0035137	hindlimb morphogenesis	2/385	51/23328	0.205706557	0.32038	0.19252	Hoxd9/Bm	2
GO:0046470	GO:0046470	phosphatidylcholine metabolic process	2/385	51/23328	0.205706557	0.32038	0.19252	Scarb1/Fa	2
GO:0061245	GO:0061245	establishment or maintenance of bipolar cell polarity	2/385	51/23328	0.205706557	0.32038	0.19252	Tcf15/Fsc	2
GO:0071470	GO:0071470	cellular response to osmotic stress	2/385	51/23328	0.205706557	0.32038	0.19252	Aqp1/Mylk	2
GO:0072132	GO:0072132	mesenchyme morphogenesis	2/385	51/23328	0.205706557	0.32038	0.19252	Eng/Nos3	2
GO:0002638	GO:0002638	negative regulation of immunoglobulin production	1/385	14/23328	0.207882991	0.32038	0.19252	Il33	1
GO:0002756	GO:0002756	MyD88-independent toll-like receptor signaling pathwa	1/385	14/23328	0.207882991	0.32038	0.19252	Irf7	1
GO:0002829	GO:0002829	negative regulation of type 2 immune response	1/385	14/23328	0.207882991	0.32038	0.19252	Anxa1	1
GO:0002885	GO:0002885	positive regulation of hypersensitivity	1/385	14/23328	0.207882991	0.32038	0.19252	C3	1
GO:0007351	GO:0007351	tripartite regional subdivision	1/385	14/23328	0.207882991	0.32038	0.19252	Pcsk6	1
GO:0008298	GO:0008298	intracellular mRNA localization	1/385	14/23328	0.207882991	0.32038	0.19252	Zfp36	1
GO:0008595	GO:0008595	anterior/posterior axis specification, embryo	1/385	14/23328	0.207882991	0.32038	0.19252	Pcsk6	1
GO:0009084	GO:0009084	glutamine family amino acid biosynthetic process	1/385	14/23328	0.207882991	0.32038	0.19252	Ass1	1
GO:0009111	GO:0009111	vitamin catabolic process	1/385	14/23328	0.207882991	0.32038	0.19252	Cyp26b1	1
GO:0010226	GO:0010226	response to lithium ion	1/385	14/23328	0.207882991	0.32038	0.19252	Fabp4	1
GO:0010804	GO:0010804	negative regulation of tumor necrosis factor-mediated :	1/385	14/23328	0.207882991	0.32038	0.19252	Gas6	1
GO:0010889	GO:0010889	regulation of sequestering of triglyceride	1/385	14/23328	0.207882991	0.32038	0.19252	Lpl	1
GO:0014831	GO:0014831	gastro-intestinal system smooth muscle contraction	1/385	14/23328	0.207882991	0.32038	0.19252	Htr2b	1
GO:0018158	GO:0018158	protein oxidation	1/385	14/23328	0.207882991	0.32038	0.19252	Lox	1
GO:0021903	GO:0021903	rostrocaudal neural tube patterning	1/385	14/23328	0.207882991	0.32038	0.19252	Sox17	1
GO:0030213	GO:0030213	hyaluronan biosynthetic process	1/385	14/23328	0.207882991	0.32038	0.19252	Pdgfb	1
GO:0032328	GO:0032328	alanine transport	1/385	14/23328	0.207882991	0.32038	0.19252	Slc6a6	1
GO:0032525	GO:0032525	somite rostral/caudal axis specification	1/385	14/23328	0.207882991	0.32038	0.19252	Nrarp	1
GO:0032736	GO:0032736	positive regulation of interleukin-13 production	1/385	14/23328	0.207882991	0.32038	0.19252	Il33	1
GO:0033079	GO:0033079	immature T cell proliferation	1/385	14/23328	0.207882991	0.32038	0.19252	Bmp4	1
GO:0034116	GO:0034116	positive regulation of heterotypic cell-cell adhesion	1/385	14/23328	0.207882991	0.32038	0.19252	Thy1	1
GO:0034309	GO:0034309	primary alcohol biosynthetic process	1/385	14/23328	0.207882991	0.32038	0.19252	Dab2	1
GO:0034638	GO:0034638	phosphatidylcholine catabolic process	1/385	14/23328	0.207882991	0.32038	0.19252	Scarb1	1
GO:0035815	GO:0035815	positive regulation of renal sodium excretion	1/385	14/23328	0.207882991	0.32038	0.19252	Edn1	1
GO:0036376	GO:0036376	sodium ion export across plasma membrane	1/385	14/23328	0.207882991	0.32038	0.19252	Atp1b1	1
GO:0036498	GO:0036498	IRE1-mediated unfolded protein response	1/385	14/23328	0.207882991	0.32038	0.19252	Hspa5	1
GO:0042362	GO:0042362	fat-soluble vitamin biosynthetic process	1/385	14/23328	0.207882991	0.32038	0.19252	Pltp	1
GO:0042983	GO:0042983	amyloid precursor protein biosynthetic process	1/385	14/23328	0.207882991	0.32038	0.19252	Bace2	1
GO:0042984	GO:0042984	regulation of amyloid precursor protein biosynthetic pro	1/385	14/23328	0.207882991	0.32038	0.19252	Bace2	1

GO:0045060	GO:0045060	negative thymic T cell selection	1/385	14/23328	0.207882991	0.32038	0.19252	Cd74	1
GO:0045792	GO:0045792	negative regulation of cell size	1/385	14/23328	0.207882991	0.32038	0.19252	C1qtnf9	1
GO:0046479	GO:0046479	glycosphingolipid catabolic process	1/385	14/23328	0.207882991	0.32038	0.19252	Gm2a	1
GO:0048505	GO:0048505	regulation of timing of cell differentiation	1/385	14/23328	0.207882991	0.32038	0.19252	Serpine2	1
GO:0048755	GO:0048755	branching morphogenesis of a nerve	1/385	14/23328	0.207882991	0.32038	0.19252	Cpe	1
GO:0051451	GO:0051451	myoblast migration	1/385	14/23328	0.207882991	0.32038	0.19252	Anxa1	1
GO:0060281	GO:0060281	regulation of oocyte development	1/385	14/23328	0.207882991	0.32038	0.19252	Pde3a	1
GO:0060911	GO:0060911	cardiac cell fate commitment	1/385	14/23328	0.207882991	0.32038	0.19252	Sox17	1
GO:0061308	GO:0061308	cardiac neural crest cell development involved in heart	1/385	14/23328	0.207882991	0.32038	0.19252	Bmp4	1
GO:0061615	GO:0061615	glycolytic process through fructose-6-phosphate	1/385	14/23328	0.207882991	0.32038	0.19252	Aldoa	1
GO:0070207	GO:0070207	protein homotrimerization	1/385	14/23328	0.207882991	0.32038	0.19252	Steap4	1
GO:0070423	GO:0070423	nucleotide-binding oligomerization domain containing s	1/385	14/23328	0.207882991	0.32038	0.19252	Nfkbia	1
GO:0071340	GO:0071340	skeletal muscle acetylcholine-gated channel clustering	1/385	14/23328	0.207882991	0.32038	0.19252	Ptn	1
GO:0071803	GO:0071803	positive regulation of podosome assembly	1/385	14/23328	0.207882991	0.32038	0.19252	Fscn1	1
GO:0072413	GO:0072413	signal transduction involved in mitotic cell cycle checkp	1/385	14/23328	0.207882991	0.32038	0.19252	Cdkn1a	1
GO:0086069	GO:0086069	bundle of His cell to Purkinje myocyte communication	1/385	14/23328	0.207882991	0.32038	0.19252	Gja5	1
GO:0097066	GO:0097066	response to thyroid hormone	1/385	14/23328	0.207882991	0.32038	0.19252	Lmo2	1
GO:0098911	GO:0098911	regulation of ventricular cardiac muscle cell action pote	1/385	14/23328	0.207882991	0.32038	0.19252	Cav1	1
GO:1902001	GO:1902001	fatty acid transmembrane transport	1/385	14/23328	0.207882991	0.32038	0.19252	Thbs1	1
GO:1902402	GO:1902402	signal transduction involved in mitotic DNA damage ch	1/385	14/23328	0.207882991	0.32038	0.19252	Cdkn1a	1
GO:1902403	GO:1902403	signal transduction involved in mitotic DNA integrity ch	1/385	14/23328	0.207882991	0.32038	0.19252	Cdkn1a	1
GO:1902902	GO:1902902	negative regulation of autophagosome assembly	1/385	14/23328	0.207882991	0.32038	0.19252	Nupr1	1
GO:1902931	GO:1902931	negative regulation of alcohol biosynthetic process	1/385	14/23328	0.207882991	0.32038	0.19252	Apoe	1
GO:1903624	GO:1903624	regulation of DNA catabolic process	1/385	14/23328	0.207882991	0.32038	0.19252	Igfbp3	1
GO:1904396	GO:1904396	regulation of neuromuscular junction development	1/385	14/23328	0.207882991	0.32038	0.19252	Ptn	1
GO:1905879	GO:1905879	regulation of oogenesis	1/385	14/23328	0.207882991	0.32038	0.19252	Pde3a	1
GO:2000347	GO:2000347	positive regulation of hepatocyte proliferation	1/385	14/23328	0.207882991	0.32038	0.19252	Ptn	1
GO:0048706	GO:0048706	embryonic skeletal system development	4/385	142/23328	0.208000049	0.32038	0.19252	Tbx1/Hox	4
GO:1901991	GO:1901991	negative regulation of mitotic cell cycle phase transition	4/385	142/23328	0.208000049	0.32038	0.19252	Rgcc/Fhl1	4
GO:0007416	GO:0007416	synapse assembly	5/385	191/23328	0.209049939	0.32188	0.19342	Reln/Pcdh	5
GO:0035725	GO:0035725	sodium ion transmembrane transport	4/385	143/23328	0.211434991	0.32498	0.19528	Fxyd6/Slc	4
GO:0060998	GO:0060998	regulation of dendritic spine development	3/385	96/23328	0.211475747	0.32498	0.19528	Reln/Plk2/	3
GO:0030857	GO:0030857	negative regulation of epithelial cell differentiation	2/385	52/23328	0.211756359	0.32498	0.19528	Id1/Cav1	2
GO:0032481	GO:0032481	positive regulation of type I interferon production	2/385	52/23328	0.211756359	0.32498	0.19528	Irf7/Hmgb	2
GO:0043268	GO:0043268	positive regulation of potassium ion transport	2/385	52/23328	0.211756359	0.32498	0.19528	Fhl1/Atp1	2
GO:0045104	GO:0045104	intermediate filament cytoskeleton organization	2/385	52/23328	0.211756359	0.32498	0.19528	Vim/Nes	2
GO:0048483	GO:0048483	autonomic nervous system development	2/385	52/23328	0.211756359	0.32498	0.19528	Tbx1/Vcar	2
GO:0061014	GO:0061014	positive regulation of mRNA catabolic process	2/385	52/23328	0.211756359	0.32498	0.19528	Btg2/Zfp3	2
GO:0034504	GO:0034504	protein localization to nucleus	7/385	293/23328	0.211758494	0.32498	0.19528	Cd36/Chc	7
GO:0002708	GO:0002708	positive regulation of lymphocyte mediated immunity	4/385	144/23328	0.214885883	0.32953	0.19801	Rsad2/Cd	4
GO:0015698	GO:0015698	inorganic anion transport	4/385	144/23328	0.214885883	0.32953	0.19801	Prnp/Slc2	4
GO:0002709	GO:0002709	regulation of T cell mediated immunity	3/385	97/23328	0.215770164	0.33065	0.19868	Rsad2/Cd	3
GO:1902749	GO:1902749	regulation of cell cycle G2/M phase transition	3/385	97/23328	0.215770164	0.33065	0.19868	Fhl1/Cdkn	3
GO:0032648	GO:0032648	regulation of interferon-beta production	2/385	53/23328	0.217823285	0.3318	0.19938	Irf7/Hmgb	2
GO:0034605	GO:0034605	cellular response to heat	2/385	53/23328	0.217823285	0.3318	0.19938	Cd34/Pmp	2
GO:0045103	GO:0045103	intermediate filament-based process	2/385	53/23328	0.217823285	0.3318	0.19938	Vim/Nes	2
GO:0045839	GO:0045839	negative regulation of mitotic nuclear division	2/385	53/23328	0.217823285	0.3318	0.19938	Rgcc/Bmp	2
GO:0048701	GO:0048701	embryonic cranial skeleton morphogenesis	2/385	53/23328	0.217823285	0.3318	0.19938	Tbx1/Bmp	2
GO:0048713	GO:0048713	regulation of oligodendrocyte differentiation	2/385	53/23328	0.217823285	0.3318	0.19938	Ptn/Bmp4	2
GO:0051955	GO:0051955	regulation of amino acid transport	2/385	53/23328	0.217823285	0.3318	0.19938	Rgs4/Itgb	2
GO:0052372	GO:0052372	modulation by symbiont of entry into host	2/385	53/23328	0.217823285	0.3318	0.19938	Cav1/Cd7	2
GO:0072073	GO:0072073	kidney epithelium development	4/385	145/23328	0.218352281	0.3318	0.19938	Sfrp1/Pdg	4
GO:0001711	GO:0001711	endodermal cell fate commitment	1/385	15/23328	0.22096376	0.3318	0.19938	Sox17	1
GO:0003188	GO:0003188	heart valve formation	1/385	15/23328	0.22096376	0.3318	0.19938	Gja5	1
GO:0006862	GO:0006862	nucleotide transport	1/385	15/23328	0.22096376	0.3318	0.19938	Slc25a4	1
GO:0006978	GO:0006978	DNA damage response, signal transduction by p53 cla	1/385	15/23328	0.22096376	0.3318	0.19938	Cdkn1a	1
GO:0010960	GO:0010960	magnesium ion homeostasis	1/385	15/23328	0.22096376	0.3318	0.19938	Pthlh	1
GO:0016553	GO:0016553	base conversion or substitution editing	1/385	15/23328	0.22096376	0.3318	0.19938	Apobec3	1
GO:0019377	GO:0019377	glycolipid catabolic process	1/385	15/23328	0.22096376	0.3318	0.19938	Gm2a	1
GO:0021819	GO:0021819	layer formation in cerebral cortex	1/385	15/23328	0.22096376	0.3318	0.19938	Reln	1
GO:0021932	GO:0021932	hindbrain radial glia guided cell migration	1/385	15/23328	0.22096376	0.3318	0.19938	Itgb1	1
GO:0023035	GO:0023035	CD40 signaling pathway	1/385	15/23328	0.22096376	0.3318	0.19938	Itgb1	1
GO:0030007	GO:0030007	cellular potassium ion homeostasis	1/385	15/23328	0.22096376	0.3318	0.19938	Atp1b1	1
GO:0030299	GO:0030299	intestinal cholesterol absorption	1/385	15/23328	0.22096376	0.3318	0.19938	Cd36	1
GO:0032308	GO:0032308	positive regulation of prostaglandin secretion	1/385	15/23328	0.22096376	0.3318	0.19938	Edn1	1
GO:0032717	GO:0032717	negative regulation of interleukin-8 production	1/385	15/23328	0.22096376	0.3318	0.19938	Anxa1	1
GO:0034162	GO:0034162	toll-like receptor 9 signaling pathway	1/385	15/23328	0.22096376	0.3318	0.19938	Rsad2	1
GO:0034433	GO:0034433	steroid esterification	1/385	15/23328	0.22096376	0.3318	0.19938	Apoe	1
GO:0034434	GO:0034434	sterol esterification	1/385	15/23328	0.22096376	0.3318	0.19938	Apoe	1
GO:0034435	GO:0034435	cholesterol esterification	1/385	15/23328	0.22096376	0.3318	0.19938	Apoe	1
GO:0035745	GO:0035745	T-helper 2 cell cytokine production	1/385	15/23328	0.22096376	0.3318	0.19938	Rsad2	1
GO:0035872	GO:0035872	nucleotide-binding domain, leucine rich repeat containi	1/385	15/23328	0.22096376	0.3318	0.19938	Nfkbia	1
GO:0042159	GO:0042159	lipoprotein catabolic process	1/385	15/23328	0.22096376	0.3318	0.19938	Apoe	1
GO:0043383	GO:0043383	negative T cell selection	1/385	15/23328	0.22096376	0.3318	0.19938	Cd74	1
GO:0046548	GO:0046548	retinal rod cell development	1/385	15/23328	0.22096376	0.3318	0.19938	Cfh	1
GO:0048070	GO:0048070	regulation of developmental pigmentation	1/385	15/23328	0.22096376	0.3318	0.19938	Kitl	1
GO:0048680	GO:0048680	positive regulation of axon regeneration	1/385	15/23328	0.22096376	0.3318	0.19938	Ptn	1
GO:0051764	GO:0051764	actin crosslink formation	1/385	15/23328	0.22096376	0.3318	0.19938	Actn1	1
GO:0060049	GO:0060049	regulation of protein glycosylation	1/385	15/23328	0.22096376	0.3318	0.19938	Acer2	1
GO:0060347	GO:0060347	heart trabecula formation	1/385	15/23328	0.22096376	0.3318	0.19938	Tek	1
GO:0061029	GO:0061029	eyelid development in camera-type eye	1/385	15/23328	0.22096376	0.3318	0.19938	Jun	1
GO:0061042	GO:0061042	vascular wound healing	1/385	15/23328	0.22096376	0.3318	0.19938	Kdr	1
GO:0061307	GO:0061307	cardiac neural crest cell differentiation involved in hear	1/385	15/23328	0.22096376	0.3318	0.19938	Bmp4	1
GO:0061318	GO:0061318	renal filtration cell differentiation	1/385	15/23328	0.22096376	0.3318	0.19938	Bmp4	1
GO:0072074	GO:0072074	kidney mesenchyme development	1/385	15/23328	0.22096376	0.3318	0.19938	Bmp4	1
GO:0072112	GO:0072112	glomerular visceral epithelial cell differentiation	1/385	15/23328	0.22096376	0.3318	0.19938	Bmp4	1
GO:0072283	GO:0072283	metanephric renal vesicle morphogenesis	1/385	15/23328	0.22096376	0.3318	0.19938	Bmp4	1
GO:0072311	GO:0072311	glomerular epithelial cell differentiation	1/385	15/23328	0.22096376	0.3318	0.19938	Bmp4	1
GO:0072350	GO:0072350	tricarboxylic acid metabolic process	1/385	15/23328	0.22096376	0.3318	0.19938	Ass1	1
GO:0086014	GO:0086014	atrial cardiac muscle cell action potential	1/385	15/23328	0.22096376	0.3318	0.19938	Gja5	1
GO:0086026	GO:0086026	atrial cardiac muscle cell to AV node cell signaling	1/385	15/23328	0.22096376	0.3318	0.19938	Gja5	1
GO:0086066	GO:0086066	atrial cardiac muscle cell to AV node cell communicatio	1/385	15/23328	0.22096376	0.3318	0.19938	Gja5	1
GO:0098712	GO:0098712	L-glutamate import across plasma membrane	1/385	15/23328	0.22096376	0.3318	0.19938	Itgb1	1
GO:0098917	GO:0098917	retrograde trans-synaptic signaling	1/385	15/23328	0.22096376	0.3318	0.19938	Fabp5	1
GO:1900225	GO:1900225	regulation of NLRP3 inflammasome complex assembly	1/385	15/23328	0.22096376	0.3318	0.19938	Cd36	1
GO:1902165	GO:1902165	regulation of intrinsic apoptotic signaling pathway in re	1/385	15/23328	0.22096376	0.3318	0.19938	Cd74	1
GO:1903543	GO:1903543	positive regulation of exosomal secretion	1/385	15/23328	0.22096376	0.3318	0.19938	Sdc1	1
GO:1903961	GO:1903961	positive regulation of anion transmembrane transport	1/385	15/23328	0.22096376	0.3318	0.19938	Itgb1	1
GO:1904294	GO:1904294	positive regulation of ERAD pathway	1/385	15/23328	0.22096376	0.3318	0.19938	Cav1	1
GO:0035637	GO:0035637	multicellular organismal signaling	4/385	146/23328	0.22183374	0.33299	0.20009	Abcc9/Ca	4
GO:0002706	GO:0002706	regulation of lymphocyte mediated immunity	5/385	196/23328	0.223850155	0.33514	0.20138	Rsad2/Cd	5
GO:0007019	GO:0007019	microtubule depolymerization	2/385	54/23328	0.223905128	0.33514	0.20138	Stmn2/Sg	2
GO:0014014	GO:0014014	negative regulation of gliogenesis	2/385	54/23328	0.223905128	0.33514	0.20138	Ptn/Bmp4	2
GO:0032355	GO:0032355	response to estradiol	2/385	54/23328	0.223905128	0.33514	0.20138	Nos3/Anx	2
GO:0045687	GO:0045687	positive regulation of glial cell differentiation	2/385	54/23328	0.223905128	0.33514	0.20138	Ptn/Serpir	2
GO:0051954	GO:0051954	positive regulation of amine transport	2/385	54/23328	0.223905128	0.33514	0.20138	Cxcl12/Itg	2
GO:1902930	GO:1902930	regulation of alcohol biosynthetic process	2/385	54/23328	0.223905128	0.33514	0.20138	Dab2/Apo	2
GO:2001222	GO:2001222	regulation of neuron migration	2/385	54/23328	0.223905128	0.33514	0.20138	Reln/Col3	2
GO:0006805	GO:0006805	xenobiotic metabolic process	3/385	99/23328	0.224413061	0.33542	0.20155	Cyp1a1/G	3
GO:0021761	GO:0021761	limbic system development	3/385	99/23328	0.224413061	0.33542	0.20155	Reln/Btg2	3
GO:0030101	GO:0030101	natural killer cell activation	3/385	99/23328	0.224413061	0.33542	0.20155	Gas6/Prdx	3
GO:0036503	GO:0036503	ERAD pathway	3/385	99/23328	0.224413061	0.33542	0.20155	Hsp90b1/	3
GO:0032006	GO:0032006	regulation of TOR signaling	3/385	100/23328	0.22875984	0.34118	0.20501	Reln/Kdr/	3
GO:0042102	GO:0042102	positive regulation of T cell proliferation	3/385	100/23328	0.22875984	0.34118	0.20501	Cd24a/Vc	3
GO:0050795	GO:0050795	regulation of behavior	3/385	100/23328	0.22875984	0.34118	0.20501	Reln/Apoe	3
GO:0098659	GO:0098659	inorganic cation import across plasma membrane	3/385	100/23328	0.22875984	0.34118	0.20501	Abcc9/Prr	3
GO:0099587	GO:0099587	inorganic ion import across plasma membrane	3/385	100/23328	0.22875984	0.34118	0.20501	Abcc9/Prr	3
GO:1902882	GO:1902882	regulation of response to oxidative stress	3/385	100/23328	0.22875984	0.34118	0.20501	Cd36/Hsp	3
GO:0016126	GO:0016126	sterol biosynthetic process	2/385	55/23328	0.229999752	0.3418	0.20539	Cyb5r3/Ap	2
GO:0030834	GO:0030834	regulation of actin filament depolymerization	2/385	55/23328	0.229999752	0.3418	0.20539	Dstn/Tpm	2
GO:0032608	GO:0032608	interferon-beta production	2/385	55/23328	0.229999752	0.3418	0.20539	Irf7/Hmgb	2
GO:0032892	GO:0032892	positive regulation of organic acid transport	2/385	55/23328	0.229999752	0.3418	0.20539	Itgb1/Edn	2
GO:0035036	GO:0035036	sperm-egg recognition	2/385	55/23328	0.229999752	0.3418	0.20539	Cd9/Aldoa	2
GO:0045604	GO:0045604	regulation of epidermal cell differentiation	2/385	55/23328	0.229999752	0.3418	0.20539	Zfp36/Bm	2
GO:0086001	GO:0086001	cardiac muscle cell action potential	2/385	55/23328	0.229999752	0.3418	0.20539	Cav1/Gja5	2
GO:0097035	GO:0097035	regulation of membrane lipid distribution	2/385	55/23328	0.229999752	0.3418	0.20539	Kcnn4/Ab	2
GO:0006906	GO:0006906	vesicle fusion	3/385	101/23328	0.233122383	0.3418	0.20539	Tbc1d4/A	3
GO:0002407	GO:0002407	dendritic cell chemotaxis	1/385	16/23328	0.233829069	0.3418	0.20539	Gas6	1
GO:0002866	GO:0002866	positive regulation of acute inflammatory response to a	1/385	16/23328	0.233829069	0.3418	0.20539	C3	1

GO:0003161	GO:0003161	cardiac conduction system development	1/385	16/23328	0.233829069	0.3418	0.20539	Gja5	1
GO:0003222	GO:0003222	ventricular trabecula myocardium morphogenesis	1/385	16/23328	0.233829069	0.3418	0.20539	Eng	1
GO:0006620	GO:0006620	posttranslational protein targeting to endoplasmic retic	1/385	16/23328	0.233829069	0.3418	0.20539	Hspa5	1
GO:0006878	GO:0006878	cellular copper ion homeostasis	1/385	16/23328	0.233829069	0.3418	0.20539	Prnp	1
GO:0007512	GO:0007512	adult heart development	1/385	16/23328	0.233829069	0.3418	0.20539	Aplnr	1
GO:0009147	GO:0009147	pyrimidine nucleoside triphosphate metabolic process	1/385	16/23328	0.233829069	0.3418	0.20539	Cmpk2	1
GO:0010224	GO:0010224	response to UV-B	1/385	16/23328	0.233829069	0.3418	0.20539	Cdkn1a	1
GO:0010454	GO:0010454	negative regulation of cell fate commitment	1/385	16/23328	0.233829069	0.3418	0.20539	Sfrp2	1
GO:0014733	GO:0014733	regulation of skeletal muscle adaptation	1/385	16/23328	0.233829069	0.3418	0.20539	Igfbp5	1
GO:0021978	GO:0021978	telencephalon regionalization	1/385	16/23328	0.233829069	0.3418	0.20539	Bmp4	1
GO:0030238	GO:0030238	male sex determination	1/385	16/23328	0.233829069	0.3418	0.20539	Dhh	1
GO:0035461	GO:0035461	vitamin transmembrane transport	1/385	16/23328	0.233829069	0.3418	0.20539	Scarb1	1
GO:0035641	GO:0035641	locomotory exploration behavior	1/385	16/23328	0.233829069	0.3418	0.20539	Apoe	1
GO:0040034	GO:0040034	regulation of development, heterochronic	1/385	16/23328	0.233829069	0.3418	0.20539	Serpine2	1
GO:0042308	GO:0042308	negative regulation of protein import into nucleus	1/385	16/23328	0.233829069	0.3418	0.20539	Cd36	1
GO:0042772	GO:0042772	DNA damage response, signal transduction resulting i	1/385	16/23328	0.233829069	0.3418	0.20539	Cdkn1a	1
GO:0043518	GO:0043518	negative regulation of DNA damage response, signal t	1/385	16/23328	0.233829069	0.3418	0.20539	Cd74	1
GO:0043923	GO:0043923	positive regulation by host of viral transcription	1/385	16/23328	0.233829069	0.3418	0.20539	Jun	1
GO:0046549	GO:0046549	retinal cone cell development	1/385	16/23328	0.233829069	0.3418	0.20539	Thy1	1
GO:0048385	GO:0048385	regulation of retinoic acid receptor signaling pathway	1/385	16/23328	0.233829069	0.3418	0.20539	Cyp26b1	1
GO:0048681	GO:0048681	negative regulation of axon regeneration	1/385	16/23328	0.233829069	0.3418	0.20539	Klk8	1
GO:0048715	GO:0048715	negative regulation of oligodendrocyte differentiation	1/385	16/23328	0.233829069	0.3418	0.20539	Bmp4	1
GO:0050862	GO:0050862	positive regulation of T cell receptor signaling pathway	1/385	16/23328	0.233829069	0.3418	0.20539	Kcnn4	1
GO:0051044	GO:0051044	positive regulation of membrane protein ectodomain pr	1/385	16/23328	0.233829069	0.3418	0.20539	Apoe	1
GO:0051151	GO:0051151	negative regulation of smooth muscle cell differentiatic	1/385	16/23328	0.233829069	0.3418	0.20539	Pdgfb	1
GO:0051386	GO:0051386	regulation of neurotrophin TRK receptor signaling path	1/385	16/23328	0.233829069	0.3418	0.20539	Ptprf	1
GO:0051938	GO:0051938	L-glutamate import	1/385	16/23328	0.233829069	0.3418	0.20539	Itgb1	1
GO:0055089	GO:0055089	fatty acid homeostasis	1/385	16/23328	0.233829069	0.3418	0.20539	Apoe	1
GO:0070166	GO:0070166	enamel mineralization	1/385	16/23328	0.233829069	0.3418	0.20539	Tbx1	1
GO:0070572	GO:0070572	positive regulation of neuron projection regeneration	1/385	16/23328	0.233829069	0.3418	0.20539	Ptn	1
GO:0070885	GO:0070885	negative regulation of calcineurin-NFAT signaling casc	1/385	16/23328	0.233829069	0.3418	0.20539	Prnp	1
GO:0071498	GO:0071498	cellular response to fluid shear stress	1/385	16/23328	0.233829069	0.3418	0.20539	Ass1	1
GO:0071599	GO:0071599	otic vesicle development	1/385	16/23328	0.233829069	0.3418	0.20539	Tbx1	1
GO:0072498	GO:0072498	embryonic skeletal joint development	1/385	16/23328	0.233829069	0.3418	0.20539	Bmp4	1
GO:0090051	GO:0090051	negative regulation of cell migration involved in sprouti	1/385	16/23328	0.233829069	0.3418	0.20539	Thbs1	1
GO:0097094	GO:0097094	craniofacial suture morphogenesis	1/385	16/23328	0.233829069	0.3418	0.20539	Bmp4	1
GO:0106057	GO:0106057	negative regulation of calcineurin-mediated signaling	1/385	16/23328	0.233829069	0.3418	0.20539	Prnp	1
GO:1900037	GO:1900037	regulation of cellular response to hypoxia	1/385	16/23328	0.233829069	0.3418	0.20539	Cd34	1
GO:1901077	GO:1901077	regulation of relaxation of muscle	1/385	16/23328	0.233829069	0.3418	0.20539	Sln	1
GO:1903541	GO:1903541	regulation of exosomal secretion	1/385	16/23328	0.233829069	0.3418	0.20539	Sdc1	1
GO:1903729	GO:1903729	regulation of plasma membrane organization	1/385	16/23328	0.233829069	0.3418	0.20539	S100a9	1
GO:1904590	GO:1904590	negative regulation of protein import	1/385	16/23328	0.233829069	0.3418	0.20539	Cd36	1
GO:1905048	GO:1905048	regulation of metallopeptidase activity	1/385	16/23328	0.233829069	0.3418	0.20539	Timp3	1
GO:2000194	GO:2000194	regulation of female gonad development	1/385	16/23328	0.233829069	0.3418	0.20539	Nupr1	1
GO:2000651	GO:2000651	positive regulation of sodium ion transmembrane trans	1/385	16/23328	0.233829069	0.3418	0.20539	Atp1b1	1
GO:2001053	GO:2001053	regulation of mesenchymal cell apoptotic process	1/385	16/23328	0.233829069	0.3418	0.20539	Tbx1	1
GO:0050806	GO:0050806	positive regulation of synaptic transmission	8/385	355/23328	0.234338502	0.34243	0.20576	Reln/Rgs4	8
GO:0007411	GO:0007411	axon guidance	6/385	251/23328	0.235578814	0.34405	0.20673	Reln/Cxcl	6
GO:0007618	GO:0007618	mating	2/385	56/23328	0.236105083	0.34405	0.20673	Serpine2/I	2
GO:0017145	GO:0017145	stem cell division	2/385	56/23328	0.236105083	0.34405	0.20673	Sox17/Sfr	2
GO:0030219	GO:0030219	megakaryocyte differentiation	2/385	56/23328	0.236105083	0.34405	0.20673	Lox/Hmgb	2
GO:0098815	GO:0098815	modulation of excitatory postsynaptic potential	2/385	56/23328	0.236105083	0.34405	0.20673	Reln/Rgs4	2
GO:1900449	GO:1900449	regulation of glutamate receptor signaling pathway	2/385	56/23328	0.236105083	0.34405	0.20673	Reln/Prnp	2
GO:1903350	GO:1903350	response to dopamine	2/385	56/23328	0.236105083	0.34405	0.20673	Rgs4/Htr2	2
GO:1903351	GO:1903351	cellular response to dopamine	2/385	56/23328	0.236105083	0.34405	0.20673	Rgs4/Htr2	2
GO:0007006	GO:0007006	mitochondrial membrane organization	3/385	102/23328	0.237499862	0.34572	0.20774	Slc25a4/C	3
GO:0070227	GO:0070227	lymphocyte apoptotic process	3/385	102/23328	0.237499862	0.34572	0.20774	Cd24a/Cd	3
GO:1901989	GO:1901989	positive regulation of cell cycle phase transition	3/385	102/23328	0.237499862	0.34572	0.20774	Rgcc/Cyp	3
GO:0097485	GO:0097485	neuron projection guidance	6/385	252/23328	0.238275231	0.34673	0.20834	Reln/Cxcl	6
GO:0050954	GO:0050954	sensory perception of mechanical stimulus	7/385	305/23328	0.240599439	0.34999	0.2103	Cxcl12/Tb	7
GO:0006821	GO:0006821	chloride transport	3/385	103/23328	0.241891453	0.35038	0.21054	Prnp/Clic5	3
GO:0050772	GO:0050772	positive regulation of axonogenesis	3/385	103/23328	0.241891453	0.35038	0.21054	Cxcl12/Fn	3
GO:0071466	GO:0071466	cellular response to xenobiotic stimulus	3/385	103/23328	0.241891453	0.35038	0.21054	Cyp1a1/G	3
GO:0002067	GO:0002067	glandular epithelial cell differentiation	2/385	57/23328	0.242219114	0.35038	0.21054	Bmp4/Pro	2
GO:0019748	GO:0019748	secondary metabolic process	2/385	57/23328	0.242219114	0.35038	0.21054	Pam/Cyp1	2
GO:0050879	GO:0050879	multicellular organismal movement	2/385	57/23328	0.242219114	0.35038	0.21054	Comp/Tnr	2
GO:0050881	GO:0050881	musculoskeletal movement	2/385	57/23328	0.242219114	0.35038	0.21054	Comp/Tnr	2
GO:0140115	GO:0140115	export across plasma membrane	2/385	57/23328	0.242219114	0.35038	0.21054	Kcnn4/Atp	2
GO:2000648	GO:2000648	positive regulation of stem cell proliferation	2/385	57/23328	0.242219114	0.35038	0.21054	Kitl/Bmp4	2
GO:0007281	GO:0007281	germ cell development	8/385	359/23328	0.243353856	0.35038	0.21054	Cxcl12/Pc	8
GO:0090068	GO:0090068	positive regulation of cell cycle process	6/385	254/23328	0.243696082	0.35038	0.21054	Rgcc/Cyp	6
GO:0009743	GO:0009743	response to carbohydrate	5/385	203/23328	0.245072347	0.35038	0.21054	Vcam1/Th	5
GO:0015844	GO:0015844	monoamine transport	3/385	104/23328	0.246296344	0.35038	0.21054	Cxcl12/Ly	3
GO:0034766	GO:0034766	negative regulation of ion transmembrane transport	3/385	104/23328	0.246296344	0.35038	0.21054	Rgs4/Thb	3
GO:0052126	GO:0052126	movement in host environment	3/385	104/23328	0.246296344	0.35038	0.21054	Cav1/Gas	3
GO:0001821	GO:0001821	histamine secretion	1/385	17/23328	0.246482458	0.35038	0.21054	Edn1	1
GO:0002024	GO:0002024	diet induced thermogenesis	1/385	17/23328	0.246482458	0.35038	0.21054	Clic5	1
GO:0002327	GO:0002327	immature B cell differentiation	1/385	17/23328	0.246482458	0.35038	0.21054	Cd24a	1
GO:0002689	GO:0002689	negative regulation of leukocyte chemotaxis	1/385	17/23328	0.246482458	0.35038	0.21054	Dusp1	1
GO:0003159	GO:0003159	morphogenesis of an endothelium	1/385	17/23328	0.246482458	0.35038	0.21054	Bmp4	1
GO:0003198	GO:0003198	epithelial to mesenchymal transition involved in endoc	1/385	17/23328	0.246482458	0.35038	0.21054	Eng	1
GO:0008272	GO:0008272	sulfate transport	1/385	17/23328	0.246482458	0.35038	0.21054	Slc26a10	1
GO:0010310	GO:0010310	regulation of hydrogen peroxide metabolic process	1/385	17/23328	0.246482458	0.35038	0.21054	Duoxa2	1
GO:0015701	GO:0015701	bicarbonate transport	1/385	17/23328	0.246482458	0.35038	0.21054	Car4	1
GO:0019048	GO:0019048	modulation by virus of host process	1/385	17/23328	0.246482458	0.35038	0.21054	Fbln1	1
GO:0030730	GO:0030730	sequestering of triglyceride	1/385	17/23328	0.246482458	0.35038	0.21054	Lpl	1
GO:0032306	GO:0032306	regulation of prostaglandin secretion	1/385	17/23328	0.246482458	0.35038	0.21054	Edn1	1
GO:0032570	GO:0032570	response to progesterone	1/385	17/23328	0.246482458	0.35038	0.21054	Cav1	1
GO:0032682	GO:0032682	negative regulation of chemokine production	1/385	17/23328	0.246482458	0.35038	0.21054	Cd24a	1
GO:0032780	GO:0032780	negative regulation of ATPase activity	1/385	17/23328	0.246482458	0.35038	0.21054	Tnni3	1
GO:0034755	GO:0034755	iron ion transmembrane transport	1/385	17/23328	0.246482458	0.35038	0.21054	Steap4	1
GO:0035729	GO:0035729	cellular response to hepatocyte growth factor stimulus	1/385	17/23328	0.246482458	0.35038	0.21054	Lgmn	1
GO:0036035	GO:0036035	osteoclast development	1/385	17/23328	0.246482458	0.35038	0.21054	Anxa2	1
GO:0042074	GO:0042074	cell migration involved in gastrulation	1/385	17/23328	0.246482458	0.35038	0.21054	Sox17	1
GO:0042415	GO:0042415	norepinephrine metabolic process	1/385	17/23328	0.246482458	0.35038	0.21054	Ly6e	1
GO:0042670	GO:0042670	retinal cone cell differentiation	1/385	17/23328	0.246482458	0.35038	0.21054	Thy1	1
GO:0044546	GO:0044546	NLRP3 inflammasome complex assembly	1/385	17/23328	0.246482458	0.35038	0.21054	Cd36	1
GO:0044794	GO:0044794	positive regulation by host of viral process	1/385	17/23328	0.246482458	0.35038	0.21054	Apoe	1
GO:0045059	GO:0045059	positive thymic T cell selection	1/385	17/23328	0.246482458	0.35038	0.21054	Cd74	1
GO:0045064	GO:0045064	T-helper 2 cell differentiation	1/385	17/23328	0.246482458	0.35038	0.21054	Anxa1	1
GO:0045198	GO:0045198	establishment of epithelial cell apical/basal polarity	1/385	17/23328	0.246482458	0.35038	0.21054	Tcf15	1
GO:0045346	GO:0045346	regulation of MHC class II biosynthetic process	1/385	17/23328	0.246482458	0.35038	0.21054	Il33	1
GO:0045793	GO:0045793	positive regulation of cell size	1/385	17/23328	0.246482458	0.35038	0.21054	Edn1	1
GO:0051004	GO:0051004	regulation of lipoprotein lipase activity	1/385	17/23328	0.246482458	0.35038	0.21054	Gpihbp1	1
GO:0055012	GO:0055012	ventricular cardiac muscle cell differentiation	1/385	17/23328	0.246482458	0.35038	0.21054	Prox1	1
GO:0055070	GO:0055070	copper ion homeostasis	1/385	17/23328	0.246482458	0.35038	0.21054	Prnp	1
GO:0060253	GO:0060253	negative regulation of glial cell proliferation	1/385	17/23328	0.246482458	0.35038	0.21054	Ptn	1
GO:0060413	GO:0060413	atrial septum morphogenesis	1/385	17/23328	0.246482458	0.35038	0.21054	Gja5	1
GO:0060788	GO:0060788	ectodermal placode formation	1/385	17/23328	0.246482458	0.35038	0.21054	Prox1	1
GO:0061154	GO:0061154	endothelial tube morphogenesis	1/385	17/23328	0.246482458	0.35038	0.21054	Bmp4	1
GO:0070243	GO:0070243	regulation of thymocyte apoptotic process	1/385	17/23328	0.246482458	0.35038	0.21054	Bmp4	1
GO:0070262	GO:0070262	peptidyl-serine dephosphorylation	1/385	17/23328	0.246482458	0.35038	0.21054	Dusp1	1
GO:0071360	GO:0071360	cellular response to exogenous dsRNA	1/385	17/23328	0.246482458	0.35038	0.21054	Cav1	1
GO:0071697	GO:0071697	ectodermal placode morphogenesis	1/385	17/23328	0.246482458	0.35038	0.21054	Prox1	1
GO:0072234	GO:0072234	metanephric nephron tubule development	1/385	17/23328	0.246482458	0.35038	0.21054	Aqp1	1
GO:0072672	GO:0072672	neutrophil extravasation	1/385	17/23328	0.246482458	0.35038	0.21054	Pecam1	1
GO:0086012	GO:0086012	membrane depolarization during cardiac muscle cell ac	1/385	17/23328	0.246482458	0.35038	0.21054	Gja5	1
GO:0090185	GO:0090185	negative regulation of kidney development	1/385	17/23328	0.246482458	0.35038	0.21054	Bmp4	1
GO:0090336	GO:0090336	positive regulation of brown fat cell differentiation	1/385	17/23328	0.246482458	0.35038	0.21054	Metrnl	1
GO:0097152	GO:0097152	mesenchymal cell apoptotic process	1/385	17/23328	0.246482458	0.35038	0.21054	Tbx1	1
GO:0098814	GO:0098814	spontaneous synaptic transmission	1/385	17/23328	0.246482458	0.35038	0.21054	Itgb1	1
GO:0099640	GO:0099640	axo-dendritic protein transport	1/385	17/23328	0.246482458	0.35038	0.21054	Hspb1	1
GO:1902176	GO:1902176	negative regulation of oxidative stress-induced intrinsic	1/385	17/23328	0.246482458	0.35038	0.21054	Hspb1	1
GO:1905331	GO:1905331	negative regulation of morphogenesis of an epithelium	1/385	17/23328	0.246482458	0.35038	0.21054	Bmp4	1
GO:2000047	GO:2000047	regulation of cell-cell adhesion mediated by cadherin	1/385	17/23328	0.246482458	0.35038	0.21054	Rgcc	1
GO:2000380	GO:2000380	regulation of mesoderm development	1/385	17/23328	0.246482458	0.35038	0.21054	Sfrp2	1
GO:2001044	GO:2001044	regulation of integrin-mediated signaling pathway	1/385	17/23328	0.246482458	0.35038	0.21054	Dab2	1
GO:0006913	GO:0006913	nucleocytoplasmic transport	7/385	308/23328	0.248001832	0.3523	0.2117	Cd36/Gas	7
GO:0051169	GO:0051169	nuclear transport	7/385	308/23328	0.248001832	0.3523	0.2117	Cd36/Gas	7

GO:0032655	GO:0032655	regulation of interleukin-12 production	2/385	58/23328	0.248339902	0.35254	0.21184	Cd36/Thb	2
GO:0043330	GO:0043330	response to exogenous dsRNA	2/385	58/23328	0.248339902	0.35254	0.21184	Cav1/Nfkb	2
GO:0009953	GO:0009953	dorsal/ventral pattern formation	3/385	105/2332	0.250713731	0.35567	0.21372	Sfrp1/Bmp	3
GO:0046634	GO:0046634	regulation of alpha-beta T cell activation	3/385	105/2332	0.250713731	0.35567	0.21372	Cd24a/H2	3
GO:0007292	GO:0007292	female gamete generation	4/385	155/2332	0.253771912	0.35989	0.21626	Pde3a/Ptr	4
GO:0007588	GO:0007588	excretion	2/385	59/23328	0.254465562	0.36051	0.21663	Dab2/Edn	2
GO:0044773	GO:0044773	mitotic DNA damage checkpoint	2/385	59/23328	0.254465562	0.36051	0.21663	Cdkn1a/Ie	2
GO:0071156	GO:0071156	regulation of cell cycle arrest	2/385	59/23328	0.254465562	0.36051	0.21663	Rgcc/Cdk	2
GO:0007626	GO:0007626	locomotory behavior	6/385	258/2332	0.254644618	0.36064	0.21671	Reln/Cxcl	6
GO:0048285	GO:0048285	organelle fission	10/385	472/2332	0.254908066	0.36089	0.21686	Rgcc/Pde	10
GO:0002076	GO:0002076	osteoblast development	1/385	18/23328	0.258927409	0.36193	0.21748	Pthlh	1
GO:0002836	GO:0002836	positive regulation of response to tumor cell	1/385	18/23328	0.258927409	0.36193	0.21748	Cd24a	1
GO:0002839	GO:0002839	positive regulation of immune response to tumor cell	1/385	18/23328	0.258927409	0.36193	0.21748	Cd24a	1
GO:0002883	GO:0002883	regulation of hypersensitivity	1/385	18/23328	0.258927409	0.36193	0.21748	C3	1
GO:0003128	GO:0003128	heart field specification	1/385	18/23328	0.258927409	0.36193	0.21748	Bmp4	1
GO:0006390	GO:0006390	mitochondrial transcription	1/385	18/23328	0.258927409	0.36193	0.21748	Chchd10	1
GO:0007350	GO:0007350	blastoderm segmentation	1/385	18/23328	0.258927409	0.36193	0.21748	Pcsk6	1
GO:0010867	GO:0010867	positive regulation of triglyceride biosynthetic process	1/385	18/23328	0.258927409	0.36193	0.21748	Scarb1	1
GO:0021783	GO:0021783	preganglionic parasympathetic fiber development	1/385	18/23328	0.258927409	0.36193	0.21748	Tbx1	1
GO:0031281	GO:0031281	positive regulation of cyclase activity	1/385	18/23328	0.258927409	0.36193	0.21748	Nos3	1
GO:0031571	GO:0031571	mitotic G1 DNA damage checkpoint	1/385	18/23328	0.258927409	0.36193	0.21748	Cdkn1a	1
GO:0032674	GO:0032674	regulation of interleukin-5 production	1/385	18/23328	0.258927409	0.36193	0.21748	Il33	1
GO:0032968	GO:0032968	positive regulation of transcription elongation from RN	1/385	18/23328	0.258927409	0.36193	0.21748	Dab2	1
GO:0033574	GO:0033574	response to testosterone	1/385	18/23328	0.258927409	0.36193	0.21748	Adh1	1
GO:0033604	GO:0033604	negative regulation of catecholamine secretion	1/385	18/23328	0.258927409	0.36193	0.21748	Ptgs1	1
GO:0042407	GO:0042407	cristae formation	1/385	18/23328	0.258927409	0.36193	0.21748	Chchd10	1
GO:0042448	GO:0042448	progesterone metabolic process	1/385	18/23328	0.258927409	0.36193	0.21748	Egr1	1
GO:0042487	GO:0042487	regulation of odontogenesis of dentin-containing tooth	1/385	18/23328	0.258927409	0.36193	0.21748	Bmp4	1
GO:0043252	GO:0043252	sodium-independent organic anion transport	1/385	18/23328	0.258927409	0.36193	0.21748	Slco2a1	1
GO:0045342	GO:0045342	MHC class II biosynthetic process	1/385	18/23328	0.258927409	0.36193	0.21748	Il33	1
GO:0045779	GO:0045779	negative regulation of bone resorption	1/385	18/23328	0.258927409	0.36193	0.21748	Cd38	1
GO:0048268	GO:0048268	clathrin coat assembly	1/385	18/23328	0.258927409	0.36193	0.21748	Dab2	1
GO:0050872	GO:0050872	white fat cell differentiation	1/385	18/23328	0.258927409	0.36193	0.21748	Fabp4	1
GO:0051131	GO:0051131	chaperone-mediated protein complex assembly	1/385	18/23328	0.258927409	0.36193	0.21748	Clu	1
GO:0051580	GO:0051580	regulation of neurotransmitter uptake	1/385	18/23328	0.258927409	0.36193	0.21748	Itgb1	1
GO:0060192	GO:0060192	negative regulation of lipase activity	1/385	18/23328	0.258927409	0.36193	0.21748	Anxa1	1
GO:0060947	GO:0060947	cardiac vascular smooth muscle cell differentiation	1/385	18/23328	0.258927409	0.36193	0.21748	Pdgfb	1
GO:0061158	GO:0061158	3'-UTR-mediated mRNA destabilization	1/385	18/23328	0.258927409	0.36193	0.21748	Zfp36	1
GO:0071696	GO:0071696	ectodermal placode development	1/385	18/23328	0.258927409	0.36193	0.21748	Prox1	1
GO:0071801	GO:0071801	regulation of podosome assembly	1/385	18/23328	0.258927409	0.36193	0.21748	Fscn1	1
GO:0072077	GO:0072077	renal vesicle morphogenesis	1/385	18/23328	0.258927409	0.36193	0.21748	Bmp4	1
GO:0086011	GO:0086011	membrane repolarization during action potential	1/385	18/23328	0.258927409	0.36193	0.21748	Cav1	1
GO:0097091	GO:0097091	synaptic vesicle clustering	1/385	18/23328	0.258927409	0.36193	0.21748	Pcdh17	1
GO:0098856	GO:0098856	intestinal lipid absorption	1/385	18/23328	0.258927409	0.36193	0.21748	Cd36	1
GO:0099150	GO:0099150	regulation of postsynaptic specialization assembly	1/385	18/23328	0.258927409	0.36193	0.21748	Gap43	1
GO:0150052	GO:0150052	regulation of postsynapse assembly	1/385	18/23328	0.258927409	0.36193	0.21748	Gap43	1
GO:1901673	GO:1901673	regulation of mitotic spindle assembly	1/385	18/23328	0.258927409	0.36193	0.21748	Hspa1a	1
GO:2000846	GO:2000846	regulation of corticosteroid hormone secretion	1/385	18/23328	0.258927409	0.36193	0.21748	Dab2	1
GO:0090174	GO:0090174	organelle membrane fusion	3/385	107/2332	0.259582823	0.36272	0.21796	Tbc1d4/A	3
GO:0060324	GO:0060324	face development	2/385	60/23328	0.260594273	0.36389	0.21866	Tbx1/Col1	2
GO:2000134	GO:2000134	negative regulation of G1/S transition of mitotic cell cy	2/385	60/23328	0.260594273	0.36389	0.21866	Fhl1/Cdkn	2
GO:0021543	GO:0021543	pallium development	4/385	157/2332	0.26099797	0.36434	0.21893	Reln/Btg2	4
GO:0048704	GO:0048704	embryonic skeletal system morphogenesis	3/385	108/2332	0.264032966	0.36845	0.2214	Tbx1/Hox	3
GO:0034250	GO:0034250	positive regulation of cellular amide metabolic process	4/385	158/2332	0.26462596	0.36891	0.22167	Vim/Clu/T	4
GO:0055067	GO:0055067	monovalent inorganic cation homeostasis	4/385	158/2332	0.26462596	0.36891	0.22167	Car4/Sgk1	4
GO:1901988	GO:1901988	negative regulation of cell cycle phase transition	4/385	158/2332	0.26462596	0.36891	0.22167	Rgcc/Fhl1	4
GO:0032615	GO:0032615	interleukin-12 production	2/385	61/23328	0.266724269	0.37146	0.22321	Cd36/Thb	2
GO:0060119	GO:0060119	inner ear receptor cell development	2/385	61/23328	0.266724269	0.37146	0.22321	Cthrc1/Cli	2
GO:0060986	GO:0060986	endocrine hormone secretion	2/385	61/23328	0.266724269	0.37146	0.22321	Aqp1/Dab	2
GO:0021549	GO:0021549	cerebellum development	3/385	109/2332	0.268492482	0.37244	0.2238	Serpine2/	3
GO:0035249	GO:0035249	synaptic transmission, glutamatergic	3/385	109/2332	0.268492482	0.37244	0.2238	Reln/Serp	3
GO:1905477	GO:1905477	positive regulation of protein localization to membrane	3/385	109/2332	0.268492482	0.37244	0.2238	Prnp/Ptn/	3
GO:0002524	GO:0002524	hypersensitivity	1/385	19/23328	0.271167347	0.37244	0.2238	C3	1
GO:0002544	GO:0002544	chronic inflammatory response	1/385	19/23328	0.271167347	0.37244	0.2238	S100a9	1
GO:0002922	GO:0002922	positive regulation of humoral immune response	1/385	19/23328	0.271167347	0.37244	0.2238	C3	1
GO:0006309	GO:0006309	apoptotic DNA fragmentation	1/385	19/23328	0.271167347	0.37244	0.2238	Igfbp3	1
GO:0006590	GO:0006590	thyroid hormone generation	1/385	19/23328	0.271167347	0.37244	0.2238	Duoxa2	1
GO:0006656	GO:0006656	phosphatidylcholine biosynthetic process	1/385	19/23328	0.271167347	0.37244	0.2238	Fabp5	1
GO:0009200	GO:0009200	deoxyribonucleoside triphosphate metabolic process	1/385	19/23328	0.271167347	0.37244	0.2238	Cmpk2	1
GO:0010623	GO:0010623	programmed cell death involved in cell development	1/385	19/23328	0.271167347	0.37244	0.2238	Kitl	1
GO:0010766	GO:0010766	negative regulation of sodium ion transport	1/385	19/23328	0.271167347	0.37244	0.2238	Serpine2	1
GO:0014061	GO:0014061	regulation of norepinephrine secretion	1/385	19/23328	0.271167347	0.37244	0.2238	Ptgs1	1
GO:0016045	GO:0016045	detection of bacterium	1/385	19/23328	0.271167347	0.37244	0.2238	Pglyrp1	1
GO:0030050	GO:0030050	vesicle transport along actin filament	1/385	19/23328	0.271167347	0.37244	0.2238	Myo1b	1
GO:0032634	GO:0032634	interleukin-5 production	1/385	19/23328	0.271167347	0.37244	0.2238	Il33	1
GO:0032656	GO:0032656	regulation of interleukin-13 production	1/385	19/23328	0.271167347	0.37244	0.2238	Il33	1
GO:0032700	GO:0032700	negative regulation of interleukin-17 production	1/385	19/23328	0.271167347	0.37244	0.2238	Prnp	1
GO:0034138	GO:0034138	toll-like receptor 3 signaling pathway	1/385	19/23328	0.271167347	0.37244	0.2238	Cav1	1
GO:0035728	GO:0035728	response to hepatocyte growth factor	1/385	19/23328	0.271167347	0.37244	0.2238	Lgmn	1
GO:0035930	GO:0035930	corticosteroid hormone secretion	1/385	19/23328	0.271167347	0.37244	0.2238	Dab2	1
GO:0035988	GO:0035988	chondrocyte proliferation	1/385	19/23328	0.271167347	0.37244	0.2238	Comp	1
GO:0040037	GO:0040037	negative regulation of fibroblast growth factor receptor	1/385	19/23328	0.271167347	0.37244	0.2238	Thbs1	1
GO:0043652	GO:0043652	engulfment of apoptotic cell	1/385	19/23328	0.271167347	0.37244	0.2238	Thbs1	1
GO:0044783	GO:0044783	G1 DNA damage checkpoint	1/385	19/23328	0.271167347	0.37244	0.2238	Cdkn1a	1
GO:0045540	GO:0045540	regulation of cholesterol biosynthetic process	1/385	19/23328	0.271167347	0.37244	0.2238	Apoe	1
GO:0045837	GO:0045837	negative regulation of membrane potential	1/385	19/23328	0.271167347	0.37244	0.2238	Ptn	1
GO:0046068	GO:0046068	cGMP metabolic process	1/385	19/23328	0.271167347	0.37244	0.2238	Pde2a	1
GO:0046827	GO:0046827	positive regulation of protein export from nucleus	1/385	19/23328	0.271167347	0.37244	0.2238	Gas6	1
GO:0048243	GO:0048243	norepinephrine secretion	1/385	19/23328	0.271167347	0.37244	0.2238	Ptgs1	1
GO:0050774	GO:0050774	negative regulation of dendrite morphogenesis	1/385	19/23328	0.271167347	0.37244	0.2238	Id1	1
GO:0050884	GO:0050884	neuromuscular process controlling posture	1/385	19/23328	0.271167347	0.37244	0.2238	Tcf15	1
GO:0051238	GO:0051238	sequestering of metal ion	1/385	19/23328	0.271167347	0.37244	0.2238	Lcn2	1
GO:0051349	GO:0051349	positive regulation of lyase activity	1/385	19/23328	0.271167347	0.37244	0.2238	Nos3	1
GO:0071605	GO:0071605	monocyte chemotactic protein-1 production	1/385	19/23328	0.271167347	0.37244	0.2238	Cd24a	1
GO:0071637	GO:0071637	regulation of monocyte chemotactic protein-1 productio	1/385	19/23328	0.271167347	0.37244	0.2238	Cd24a	1
GO:0097623	GO:0097623	potassium ion export across plasma membrane	1/385	19/23328	0.271167347	0.37244	0.2238	Kcnn4	1
GO:0106118	GO:0106118	regulation of sterol biosynthetic process	1/385	19/23328	0.271167347	0.37244	0.2238	Apoe	1
GO:0150146	GO:0150146	cell junction disassembly	1/385	19/23328	0.271167347	0.37244	0.2238	C3	1
GO:1900451	GO:1900451	positive regulation of glutamate receptor signaling path	1/385	19/23328	0.271167347	0.37244	0.2238	Reln	1
GO:2000251	GO:2000251	positive regulation of actin cytoskeleton reorganization	1/385	19/23328	0.271167347	0.37244	0.2238	Tek	1
GO:2000641	GO:2000641	regulation of early endosome to late endosome transp	1/385	19/23328	0.271167347	0.37244	0.2238	Dab2	1
GO:0010171	GO:0010171	body morphogenesis	2/385	62/23328	0.272853844	0.37368	0.22454	Tbx1/Col1	2
GO:0019080	GO:0019080	viral gene expression	2/385	62/23328	0.272853844	0.37368	0.22454	Zfp36/Jun	2
GO:0050982	GO:0050982	detection of mechanical stimulus	2/385	62/23328	0.272853844	0.37368	0.22454	Cxcl12/Se	2
GO:0051148	GO:0051148	negative regulation of muscle cell differentiation	2/385	62/23328	0.272853844	0.37368	0.22454	Rgs4/Pdg	2
GO:1903078	GO:1903078	positive regulation of protein localization to plasma me	2/385	62/23328	0.272853844	0.37368	0.22454	Prnp/Itgb1	2
GO:0006275	GO:0006275	regulation of DNA replication	3/385	110/2332	0.272960614	0.37368	0.22454	Id3/Pdgfb	3
GO:0008344	GO:0008344	adult locomotory behavior	3/385	110/2332	0.272960614	0.37368	0.22454	Cxcl12/Hc	3
GO:0009410	GO:0009410	response to xenobiotic stimulus	3/385	110/2332	0.272960614	0.37368	0.22454	Cyp1a1/G	3
GO:0048709	GO:0048709	oligodendrocyte differentiation	3/385	110/2332	0.272960614	0.37368	0.22454	Clu/Ptn/Br	3
GO:0120034	GO:0120034	positive regulation of plasma membrane bounded cell	3/385	110/2332	0.272960614	0.37368	0.22454	Aqp1/Fsc	3
GO:0007005	GO:0007005	mitochondrion organization	10/385	481/2332	0.273255165	0.37396	0.22471	Higd1b/Kc	10
GO:0009755	GO:0009755	hormone-mediated signaling pathway	4/385	161/2332	0.275564683	0.377	0.22654	Sfrp1/Dab	4
GO:0016241	GO:0016241	regulation of macroautophagy	3/385	111/2332	0.277436614	0.37919	0.22785	Nupr1/Kdr	3
GO:0072009	GO:0072009	nephron epithelium development	3/385	111/2332	0.277436614	0.37919	0.22785	Pdgfb/Aqp	3
GO:1904659	GO:1904659	glucose transmembrane transport	3/385	111/2332	0.277436614	0.37919	0.22785	Fabp5/C3	3
GO:0044282	GO:0044282	small molecule catabolic process	7/385	320/2332	0.278269217	0.3802	0.22846	Adh7/Sca	7
GO:0001947	GO:0001947	heart looping	2/385	63/23328	0.278981345	0.38093	0.2289	Sox17/En	2
GO:0060999	GO:0060999	positive regulation of dendritic spine development	2/385	63/23328	0.278981345	0.38093	0.2289	Reln/Apoe	2
GO:0008645	GO:0008645	hexose transmembrane transport	3/385	112/2332	0.281919746	0.38171	0.22937	Fabp5/C3	3
GO:0007249	GO:0007249	I-kappaB kinase/NF-kappaB signaling	5/385	215/2332	0.282567846	0.38171	0.22937	Cd36/Hsp	5
GO:0140014	GO:0140014	mitotic nuclear division	6/385	268/2332	0.282568983	0.38171	0.22937	Rgcc/Cdk	6
GO:0002755	GO:0002755	MyD88-dependent toll-like receptor signaling pathway	1/385	20/23328	0.28320564	0.38171	0.22937	Irf7	1
GO:0006699	GO:0006699	bile acid biosynthetic process	1/385	20/23328	0.28320564	0.38171	0.22937	Prox1	1
GO:0007342	GO:0007342	fusion of sperm to egg plasma membrane involved in s	1/385	20/23328	0.28320564	0.38171	0.22937	Cd9	1
GO:0010528	GO:0010528	regulation of transposition	1/385	20/23328	0.28320564	0.38171	0.22937	Apobec3	1
GO:0010529	GO:0010529	negative regulation of transposition	1/385	20/23328	0.28320564	0.38171	0.22937	Apobec3	1
GO:0010818	GO:0010818	T cell chemotaxis	1/385	20/23328	0.28320564	0.38171	0.22937	Cxcl12	1
GO:0010888	GO:0010888	negative regulation of lipid storage	1/385	20/23328	0.28320564	0.38171	0.22937	Nfkbia	1

GO:0021535	GO:0021535	cell migration in hindbrain	1/385	20/23328	0.28320564	0.38171	0.22937	Itgb1	1
GO:0030002	GO:0030002	cellular anion homeostasis	1/385	20/23328	0.28320564	0.38171	0.22937	Ckb	1
GO:0030220	GO:0030220	platelet formation	1/385	20/23328	0.28320564	0.38171	0.22937	Actn1	1
GO:0030320	GO:0030320	cellular monovalent inorganic anion homeostasis	1/385	20/23328	0.28320564	0.38171	0.22937	Ckb	1
GO:0030810	GO:0030810	positive regulation of nucleotide biosynthetic process	1/385	20/23328	0.28320564	0.38171	0.22937	Nos3	1
GO:0030852	GO:0030852	regulation of granulocyte differentiation	1/385	20/23328	0.28320564	0.38171	0.22937	Rbp1	1
GO:0030889	GO:0030889	negative regulation of B cell proliferation	1/385	20/23328	0.28320564	0.38171	0.22937	Cd24a	1
GO:0032310	GO:0032310	prostaglandin secretion	1/385	20/23328	0.28320564	0.38171	0.22937	Edn1	1
GO:0032695	GO:0032695	negative regulation of interleukin-12 production	1/385	20/23328	0.28320564	0.38171	0.22937	Thbs1	1
GO:0033033	GO:0033033	negative regulation of myeloid cell apoptotic process	1/385	20/23328	0.28320564	0.38171	0.22937	Kitl	1
GO:0034143	GO:0034143	regulation of toll-like receptor 4 signaling pathway	1/385	20/23328	0.28320564	0.38171	0.22937	Lbp	1
GO:0035970	GO:0035970	peptidyl-threonine dephosphorylation	1/385	20/23328	0.28320564	0.38171	0.22937	Dusp1	1
GO:0044819	GO:0044819	mitotic G1/S transition checkpoint	1/385	20/23328	0.28320564	0.38171	0.22937	Cdkn1a	1
GO:0045217	GO:0045217	cell-cell junction maintenance	1/385	20/23328	0.28320564	0.38171	0.22937	F2r	1
GO:0045623	GO:0045623	negative regulation of T-helper cell differentiation	1/385	20/23328	0.28320564	0.38171	0.22937	Anxa1	1
GO:0045986	GO:0045986	negative regulation of smooth muscle contraction	1/385	20/23328	0.28320564	0.38171	0.22937	Calcrl	1
GO:0048245	GO:0048245	eosinophil chemotaxis	1/385	20/23328	0.28320564	0.38171	0.22937	Ccl21a	1
GO:0050965	GO:0050965	detection of temperature stimulus involved in sensory p	1/385	20/23328	0.28320564	0.38171	0.22937	Cxcl12	1
GO:0051608	GO:0051608	histamine transport	1/385	20/23328	0.28320564	0.38171	0.22937	Edn1	1
GO:0060252	GO:0060252	positive regulation of glial cell proliferation	1/385	20/23328	0.28320564	0.38171	0.22937	Vim	1
GO:0060307	GO:0060307	regulation of ventricular cardiac muscle cell membrane	1/385	20/23328	0.28320564	0.38171	0.22937	Gja5	1
GO:0070935	GO:0070935	3'-UTR-mediated mRNA stabilization	1/385	20/23328	0.28320564	0.38171	0.22937	Zfp36	1
GO:0072087	GO:0072087	renal vesicle development	1/385	20/23328	0.28320564	0.38171	0.22937	Bmp4	1
GO:0090030	GO:0090030	regulation of steroid hormone biosynthetic process	1/385	20/23328	0.28320564	0.38171	0.22937	Dab2	1
GO:1900373	GO:1900373	positive regulation of purine nucleotide biosynthetic pro	1/385	20/23328	0.28320564	0.38171	0.22937	Nos3	1
GO:1900746	GO:1900746	regulation of vascular endothelial growth factor signalin	1/385	20/23328	0.28320564	0.38171	0.22937	Dcn	1
GO:1903909	GO:1903909	regulation of receptor clustering	1/385	20/23328	0.28320564	0.38171	0.22937	Ptn	1
GO:1904385	GO:1904385	cellular response to angiotensin	1/385	20/23328	0.28320564	0.38171	0.22937	Cav1	1
GO:1990182	GO:1990182	exosomal secretion	1/385	20/23328	0.28320564	0.38171	0.22937	Sdc1	1
GO:2000773	GO:2000773	negative regulation of cellular senescence	1/385	20/23328	0.28320564	0.38171	0.22937	Plk2	1
GO:0009064	GO:0009064	glutamine family amino acid metabolic process	2/385	64/23328	0.285105177	0.38378	0.23061	Nos3/Ass	2
GO:0043331	GO:0043331	response to dsRNA	2/385	64/23328	0.285105177	0.38378	0.23061	Cav1/Nfkb	2
GO:0051784	GO:0051784	negative regulation of nuclear division	2/385	64/23328	0.285105177	0.38378	0.23061	Rgcc/Bmp	2
GO:1902807	GO:1902807	negative regulation of cell cycle G1/S phase transition	2/385	64/23328	0.285105177	0.38378	0.23061	Fhl1/Cdkn	2
GO:0035264	GO:0035264	multicellular organism growth	5/385	216/23328	0.285744295	0.38451	0.23105	Lgmn/Cdk	5
GO:0033135	GO:0033135	regulation of peptidyl-serine phosphorylation	4/385	164/23328	0.286576753	0.38551	0.23165	Cav1/Gas	4
GO:0016042	GO:0016042	lipid catabolic process	7/385	324/23328	0.288563974	0.38806	0.23318	Mgll/Scarb	7
GO:0007623	GO:0007623	circadian rhythm	5/385	217/23328	0.288927465	0.38842	0.2334	Id1/Id3/Eg	5
GO:0015749	GO:0015749	monosaccharide transmembrane transport	3/385	114/23328	0.290904505	0.39083	0.23485	Fabp5/C3	3
GO:1903076	GO:1903076	regulation of protein localization to plasma membrane	3/385	114/23328	0.290904505	0.39083	0.23485	Prnp/Dab2	3
GO:0015807	GO:0015807	L-amino acid transport	2/385	65/23328	0.291223794	0.39101	0.23495	Rgs4/Itgb1	2
GO:0044774	GO:0044774	mitotic DNA integrity checkpoint	2/385	65/23328	0.291223794	0.39101	0.23495	Cdkn1a/Ie	2
GO:0046631	GO:0046631	alpha-beta T cell activation	4/385	166/23328	0.293953379	0.39162	0.23532	Rsad2/Cd	4
GO:0001976	GO:0001976	nervous system process involved in regulation of syste	1/385	21/23328	0.295045602	0.39162	0.23532	Tnni3	1
GO:0007202	GO:0007202	activation of phospholipase C activity	1/385	21/23328	0.295045602	0.39162	0.23532	Htr2b	1
GO:0008210	GO:0008210	estrogen metabolic process	1/385	21/23328	0.295045602	0.39162	0.23532	Cyp1a1	1
GO:0009110	GO:0009110	vitamin biosynthetic process	1/385	21/23328	0.295045602	0.39162	0.23532	Pltp	1
GO:0010447	GO:0010447	response to acidic pH	1/385	21/23328	0.295045602	0.39162	0.23532	Lgmn	1
GO:0010869	GO:0010869	regulation of receptor biosynthetic process	1/385	21/23328	0.295045602	0.39162	0.23532	Edn1	1
GO:0014075	GO:0014075	response to amine	1/385	21/23328	0.295045602	0.39162	0.23532	Htr2b	1
GO:0032305	GO:0032305	positive regulation of icosanoid secretion	1/385	21/23328	0.295045602	0.39162	0.23532	Edn1	1
GO:0033605	GO:0033605	positive regulation of catecholamine secretion	1/385	21/23328	0.295045602	0.39162	0.23532	Cxcl12	1
GO:0034312	GO:0034312	diol biosynthetic process	1/385	21/23328	0.295045602	0.39162	0.23532	Acer2	1
GO:0034315	GO:0034315	regulation of Arp2/3 complex-mediated actin nucleatio	1/385	21/23328	0.295045602	0.39162	0.23532	Gmfg	1
GO:0036336	GO:0036336	dendritic cell migration	1/385	21/23328	0.295045602	0.39162	0.23532	Gas6	1
GO:0036344	GO:0036344	platelet morphogenesis	1/385	21/23328	0.295045602	0.39162	0.23532	Actn1	1
GO:0045063	GO:0045063	T-helper 1 cell differentiation	1/385	21/23328	0.295045602	0.39162	0.23532	Anxa1	1
GO:0045475	GO:0045475	locomotor rhythm	1/385	21/23328	0.295045602	0.39162	0.23532	Egr1	1
GO:0048486	GO:0048486	parasympathetic nervous system development	1/385	21/23328	0.295045602	0.39162	0.23532	Tbx1	1
GO:0050434	GO:0050434	positive regulation of viral transcription	1/385	21/23328	0.295045602	0.39162	0.23532	Jun	1
GO:0050650	GO:0050650	chondroitin sulfate proteoglycan biosynthetic process	1/385	21/23328	0.295045602	0.39162	0.23532	Cytl1	1
GO:0050966	GO:0050966	detection of mechanical stimulus involved in sensory p	1/385	21/23328	0.295045602	0.39162	0.23532	Cxcl12	1
GO:0051023	GO:0051023	regulation of immunoglobulin secretion	1/385	21/23328	0.295045602	0.39162	0.23532	Il33	1
GO:0051956	GO:0051956	negative regulation of amino acid transport	1/385	21/23328	0.295045602	0.39162	0.23532	Rgs4	1
GO:0055064	GO:0055064	chloride ion homeostasis	1/385	21/23328	0.295045602	0.39162	0.23532	Ckb	1
GO:0060055	GO:0060055	angiogenesis involved in wound healing	1/385	21/23328	0.295045602	0.39162	0.23532	Kdr	1
GO:0060088	GO:0060088	auditory receptor cell stereocilium organization	1/385	21/23328	0.295045602	0.39162	0.23532	Clic5	1
GO:0060456	GO:0060456	positive regulation of digestive system process	1/385	21/23328	0.295045602	0.39162	0.23532	Aqp1	1
GO:0070230	GO:0070230	positive regulation of lymphocyte apoptotic process	1/385	21/23328	0.295045602	0.39162	0.23532	Cd24a	1
GO:0072170	GO:0072170	metanephric tubule development	1/385	21/23328	0.295045602	0.39162	0.23532	Aqp1	1
GO:0090036	GO:0090036	regulation of protein kinase C signaling	1/385	21/23328	0.295045602	0.39162	0.23532	Flt4	1
GO:0097107	GO:0097107	postsynaptic density assembly	1/385	21/23328	0.295045602	0.39162	0.23532	Reln	1
GO:0098543	GO:0098543	detection of other organism	1/385	21/23328	0.295045602	0.39162	0.23532	Pglyrp1	1
GO:0099515	GO:0099515	actin filament-based transport	1/385	21/23328	0.295045602	0.39162	0.23532	Myo1b	1
GO:1901017	GO:1901017	negative regulation of potassium ion transmembrane tr	1/385	21/23328	0.295045602	0.39162	0.23532	Cav1	1
GO:1902254	GO:1902254	negative regulation of intrinsic apoptotic signaling path	1/385	21/23328	0.295045602	0.39162	0.23532	Cd74	1
GO:2000726	GO:2000726	negative regulation of cardiac muscle cell differentiatio	1/385	21/23328	0.295045602	0.39162	0.23532	Rgs4	1
GO:2001224	GO:2001224	positive regulation of neuron migration	1/385	21/23328	0.295045602	0.39162	0.23532	Reln	1
GO:0018958	GO:0018958	phenol-containing compound metabolic process	3/385	115/23328	0.295404707	0.39184	0.23546	Reln/Duox	3
GO:0034219	GO:0034219	carbohydrate transmembrane transport	3/385	115/23328	0.295404707	0.39184	0.23546	Fabp5/C3	3
GO:0001707	GO:0001707	mesoderm formation	2/385	66/23328	0.297335703	0.39403	0.23677	Bmp4/Sfrp	2
GO:0050433	GO:0050433	regulation of catecholamine secretion	2/385	66/23328	0.297335703	0.39403	0.23677	Cxcl12/Pt	2
GO:2000736	GO:2000736	regulation of stem cell differentiation	2/385	66/23328	0.297335703	0.39403	0.23677	Ptn/Bmp4	2
GO:0042472	GO:0042472	inner ear morphogenesis	3/385	116/23328	0.299909192	0.39732	0.23874	Tbx1/Cthr	3
GO:0010948	GO:0010948	negative regulation of cell cycle process	5/385	221/23328	0.301722065	0.39946	0.24004	Rgcc/Fhl1	5
GO:0098656	GO:0098656	anion transmembrane transport	5/385	221/23328	0.301722065	0.39946	0.24004	Rgs4/Prnp	5
GO:0033209	GO:0033209	tumor necrosis factor-mediated signaling pathway	2/385	67/23328	0.303439464	0.40059	0.24071	Gas6/Nfkb	2
GO:0086003	GO:0086003	cardiac muscle cell contraction	2/385	67/23328	0.303439464	0.40059	0.24071	Cav1/Gja5	2
GO:2000242	GO:2000242	negative regulation of reproductive process	2/385	67/23328	0.303439464	0.40059	0.24071	Arhgdib/B	2
GO:0000086	GO:0000086	G2/M transition of mitotic cell cycle	3/385	117/23328	0.304417271	0.40059	0.24071	Fhl1/Cdkn	3
GO:0002286	GO:0002286	T cell activation involved in immune response	3/385	117/23328	0.304417271	0.40059	0.24071	Fgl2/Cd74	3
GO:0006612	GO:0006612	protein targeting to membrane	3/385	117/23328	0.304417271	0.40059	0.24071	Prnp/Rtp4	3
GO:0032414	GO:0032414	positive regulation of ion transmembrane transporter a	3/385	117/23328	0.304417271	0.40059	0.24071	Reln/Bmp	3
GO:0045621	GO:0045621	positive regulation of lymphocyte differentiation	3/385	117/23328	0.304417271	0.40059	0.24071	Gas6/Cd7	3
GO:2001020	GO:2001020	regulation of response to DNA damage stimulus	5/385	222/23328	0.304934879	0.40059	0.24071	Cxcl12/Cl	5
GO:0032874	GO:0032874	positive regulation of stress-activated MAPK cascade	4/385	169/23328	0.305063083	0.40059	0.24071	Dab2/Trf/	4
GO:0000413	GO:0000413	protein peptidyl-prolyl isomerization	1/385	22/23328	0.306690492	0.40059	0.24071	Ppic	1
GO:0001573	GO:0001573	ganglioside metabolic process	1/385	22/23328	0.306690492	0.40059	0.24071	Gm2a	1
GO:0003323	GO:0003323	type B pancreatic cell development	1/385	22/23328	0.306690492	0.40059	0.24071	Bmp4	1
GO:0006144	GO:0006144	purine nucleobase metabolic process	1/385	22/23328	0.306690492	0.40059	0.24071	Gda	1
GO:0006221	GO:0006221	pyrimidine nucleotide biosynthetic process	1/385	22/23328	0.306690492	0.40059	0.24071	Cmpk2	1
GO:0009154	GO:0009154	purine ribonucleotide catabolic process	1/385	22/23328	0.306690492	0.40059	0.24071	Pde2a	1
GO:0009261	GO:0009261	ribonucleotide catabolic process	1/385	22/23328	0.306690492	0.40059	0.24071	Pde2a	1
GO:0014850	GO:0014850	response to muscle activity	1/385	22/23328	0.306690492	0.40059	0.24071	Metrnl	1
GO:0015813	GO:0015813	L-glutamate transmembrane transport	1/385	22/23328	0.306690492	0.40059	0.24071	Itgb1	1
GO:0018904	GO:0018904	ether metabolic process	1/385	22/23328	0.306690492	0.40059	0.24071	Ephx2	1
GO:0032366	GO:0032366	intracellular sterol transport	1/385	22/23328	0.306690492	0.40059	0.24071	Anxa2	1
GO:0032367	GO:0032367	intracellular cholesterol transport	1/385	22/23328	0.306690492	0.40059	0.24071	Anxa2	1
GO:0032616	GO:0032616	interleukin-13 production	1/385	22/23328	0.306690492	0.40059	0.24071	Il33	1
GO:0034377	GO:0034377	plasma lipoprotein particle assembly	1/385	22/23328	0.306690492	0.40059	0.24071	Apoe	1
GO:0043651	GO:0043651	linoleic acid metabolic process	1/385	22/23328	0.306690492	0.40059	0.24071	Ephx2	1
GO:0045624	GO:0045624	positive regulation of T-helper cell differentiation	1/385	22/23328	0.306690492	0.40059	0.24071	Anxa1	1
GO:0046641	GO:0046641	positive regulation of alpha-beta T cell proliferation	1/385	22/23328	0.306690492	0.40059	0.24071	Cd24a	1
GO:0048261	GO:0048261	negative regulation of receptor-mediated endocytosis	1/385	22/23328	0.306690492	0.40059	0.24071	Sh3gl3	1
GO:0048339	GO:0048339	paraxial mesoderm development	1/385	22/23328	0.306690492	0.40059	0.24071	Tcf15	1
GO:0048532	GO:0048532	anatomical structure arrangement	1/385	22/23328	0.306690492	0.40059	0.24071	Hspa5	1
GO:0051220	GO:0051220	cytoplasmic sequestering of protein	1/385	22/23328	0.306690492	0.40059	0.24071	Nfkbia	1
GO:0060438	GO:0060438	trachea development	1/385	22/23328	0.306690492	0.40059	0.24071	Bmp4	1
GO:0070242	GO:0070242	thymocyte apoptotic process	1/385	22/23328	0.306690492	0.40059	0.24071	Bmp4	1
GO:0071800	GO:0071800	podosome assembly	1/385	22/23328	0.306690492	0.40059	0.24071	Fscn1	1
GO:0072273	GO:0072273	metanephric nephron morphogenesis	1/385	22/23328	0.306690492	0.40059	0.24071	Bmp4	1
GO:0080111	GO:0080111	DNA demethylation	1/385	22/23328	0.306690492	0.40059	0.24071	Apobec3	1
GO:0099625	GO:0099625	ventricular cardiac muscle cell membrane repolarizatic	1/385	22/23328	0.306690492	0.40059	0.24071	Gja5	1
GO:1901223	GO:1901223	negative regulation of NIK/NF-kappaB signaling	1/385	22/23328	0.306690492	0.40059	0.24071	Adgrg3	1
GO:1902307	GO:1902307	positive regulation of sodium ion transmembrane trans	1/385	22/23328	0.306690492	0.40059	0.24071	Atp1b1	1
GO:1902547	GO:1902547	regulation of cellular response to vascular endothelial	1/385	22/23328	0.306690492	0.40059	0.24071	Dcn	1
GO:1903975	GO:1903975	regulation of glial cell migration	1/385	22/23328	0.306690492	0.40059	0.24071	Vim	1
GO:1904996	GO:1904996	positive regulation of leukocyte adhesion to vascular e	1/385	22/23328	0.306690492	0.40059	0.24071	Selp	1
GO:1990776	GO:1990776	response to angiotensin	1/385	22/23328	0.306690492	0.40059	0.24071	Cav1	1

GO:0018105	GO:0018105	peptidyl-serine phosphorylation	7/385	331/23328	0.30678574	0.40059	0.24071	Plk2/Pdgfl	7
GO:0051259	GO:0051259	protein complex oligomerization	5/385	223/23328	0.308152838	0.40225	0.24171	Steap4/Pr	5
GO:0008037	GO:0008037	cell recognition	6/385	277/23328	0.308252976	0.40226	0.24171	Cd36/Sca	6
GO:0009100	GO:0009100	glycoprotein metabolic process	7/385	332/23328	0.309407996	0.40267	0.24196	Bgn/Gxylt	7
GO:0002712	GO:0002712	regulation of B cell mediated immunity	2/385	68/23328	0.309533683	0.40267	0.24196	C3/Cd55	2
GO:0002889	GO:0002889	regulation of immunoglobulin mediated immune respor	2/385	68/23328	0.309533683	0.40267	0.24196	C3/Cd55	2
GO:0015800	GO:0015800	acidic amino acid transport	2/385	68/23328	0.309533683	0.40267	0.24196	Itgb1/Slc6	2
GO:0022029	GO:0022029	telencephalon cell migration	2/385	68/23328	0.309533683	0.40267	0.24196	Reln/Cxcl	2
GO:0030968	GO:0030968	endoplasmic reticulum unfolded protein response	2/385	68/23328	0.309533683	0.40267	0.24196	Atf3/Hspa	2
GO:0048641	GO:0048641	regulation of skeletal muscle tissue development	2/385	68/23328	0.309533683	0.40267	0.24196	Tbx1/Cyp	2
GO:0050771	GO:0050771	negative regulation of axonogenesis	2/385	68/23328	0.309533683	0.40267	0.24196	Thy1/Sem	2
GO:0071868	GO:0071868	cellular response to monoamine stimulus	2/385	68/23328	0.309533683	0.40267	0.24196	Rgs4/Htr2	2
GO:0071870	GO:0071870	cellular response to catecholamine stimulus	2/385	68/23328	0.309533683	0.40267	0.24196	Rgs4/Htr2	2
GO:0030902	GO:0030902	hindbrain development	4/385	171/23328	0.312494757	0.40627	0.24413	Serpine2/I	4
GO:0070304	GO:0070304	positive regulation of stress-activated protein kinase si	4/385	171/23328	0.312494757	0.40627	0.24413	Dab2/Trf/F	4
GO:0009880	GO:0009880	embryonic pattern specification	2/385	69/23328	0.315617015	0.40892	0.24571	Nrarp/Pcs	2
GO:0016239	GO:0016239	positive regulation of macroautophagy	2/385	69/23328	0.315617015	0.40892	0.24571	Kdr/Dcn	2
GO:0021536	GO:0021536	diencephalon development	2/385	69/23328	0.315617015	0.40892	0.24571	Ncam1/Br	2
GO:0034394	GO:0034394	protein localization to cell surface	2/385	69/23328	0.315617015	0.40892	0.24571	Gpihbp1/F	2
GO:0048168	GO:0048168	regulation of neuronal synaptic plasticity	2/385	69/23328	0.315617015	0.40892	0.24571	Kdr/Egr1	2
GO:0061371	GO:0061371	determination of heart left/right asymmetry	2/385	69/23328	0.315617015	0.40892	0.24571	Sox17/En	2
GO:0071867	GO:0071867	response to monoamine	2/385	69/23328	0.315617015	0.40892	0.24571	Rgs4/Htr2	2
GO:0071869	GO:0071869	response to catecholamine	2/385	69/23328	0.315617015	0.40892	0.24571	Rgs4/Htr2	2
GO:1904377	GO:1904377	positive regulation of protein localization to cell periphe	2/385	69/23328	0.315617015	0.40892	0.24571	Prnp/Itgb1	2
GO:0002320	GO:0002320	lymphoid progenitor cell differentiation	1/385	23/23328	0.318143515	0.40892	0.24571	Bmp4	1
GO:0002834	GO:0002834	regulation of response to tumor cell	1/385	23/23328	0.318143515	0.40892	0.24571	Cd24a	1
GO:0002837	GO:0002837	regulation of immune response to tumor cell	1/385	23/23328	0.318143515	0.40892	0.24571	Cd24a	1
GO:0003283	GO:0003283	atrial septum development	1/385	23/23328	0.318143515	0.40892	0.24571	Gja5	1
GO:0009065	GO:0009065	glutamine family amino acid catabolic process	1/385	23/23328	0.318143515	0.40892	0.24571	Nos3	1
GO:0010996	GO:0010996	response to auditory stimulus	1/385	23/23328	0.318143515	0.40892	0.24571	Ptn	1
GO:0015812	GO:0015812	gamma-aminobutyric acid transport	1/385	23/23328	0.318143515	0.40892	0.24571	Slc6a6	1
GO:0023019	GO:0023019	signal transduction involved in regulation of gene expr	1/385	23/23328	0.318143515	0.40892	0.24571	Sox17	1
GO:0032196	GO:0032196	transposition	1/385	23/23328	0.318143515	0.40892	0.24571	Apobec3	1
GO:0032727	GO:0032727	positive regulation of interferon-alpha production	1/385	23/23328	0.318143515	0.40892	0.24571	Irf7	1
GO:0034405	GO:0034405	response to fluid shear stress	1/385	23/23328	0.318143515	0.40892	0.24571	Ass1	1
GO:0035278	GO:0035278	miRNA mediated inhibition of translation	1/385	23/23328	0.318143515	0.40892	0.24571	Zfp36	1
GO:0036003	GO:0036003	positive regulation of transcription from RNA polymera	1/385	23/23328	0.318143515	0.40892	0.24571	Atf3	1
GO:0043371	GO:0043371	negative regulation of CD4-positive, alpha-beta T cell	1/385	23/23328	0.318143515	0.40892	0.24571	Anxa1	1
GO:0043501	GO:0043501	skeletal muscle adaptation	1/385	23/23328	0.318143515	0.40892	0.24571	Igfbp5	1
GO:0045026	GO:0045026	plasma membrane fusion	1/385	23/23328	0.318143515	0.40892	0.24571	Cd9	1
GO:0045606	GO:0045606	positive regulation of epidermal cell differentiation	1/385	23/23328	0.318143515	0.40892	0.24571	Bmp4	1
GO:0046599	GO:0046599	regulation of centriole replication	1/385	23/23328	0.318143515	0.40892	0.24571	Plk2	1
GO:0050857	GO:0050857	positive regulation of antigen receptor-mediated signal	1/385	23/23328	0.318143515	0.40892	0.24571	Kcnn4	1
GO:0050961	GO:0050961	detection of temperature stimulus involved in sensory	1/385	23/23328	0.318143515	0.40892	0.24571	Cxcl12	1
GO:0071359	GO:0071359	cellular response to dsRNA	1/385	23/23328	0.318143515	0.40892	0.24571	Cav1	1
GO:0090190	GO:0090190	positive regulation of branching involved in ureteric bu	1/385	23/23328	0.318143515	0.40892	0.24571	Bmp4	1
GO:0098703	GO:0098703	calcium ion import across plasma membrane	1/385	23/23328	0.318143515	0.40892	0.24571	Prnp	1
GO:0101023	GO:0101023	vascular endothelial cell proliferation	1/385	23/23328	0.318143515	0.40892	0.24571	Flt1	1
GO:1905562	GO:1905562	regulation of vascular endothelial cell proliferation	1/385	23/23328	0.318143515	0.40892	0.24571	Flt1	1
GO:2000345	GO:2000345	regulation of hepatocyte proliferation	1/385	23/23328	0.318143515	0.40892	0.24571	Ptn	1
GO:2000810	GO:2000810	regulation of bicellular tight junction assembly	1/385	23/23328	0.318143515	0.40892	0.24571	Cldn5	1
GO:2000831	GO:2000831	regulation of steroid hormone secretion	1/385	23/23328	0.318143515	0.40892	0.24571	Dab2	1
GO:0051260	GO:0051260	protein homooligomerization	4/385	173/23328	0.319942949	0.4111	0.24703	Steap4/Pr	4
GO:0002381	GO:0002381	immunoglobulin production involved in immunoglobulir	2/385	70/23328	0.321688162	0.41221	0.24769	Fgl2/H2-A	2
GO:0003143	GO:0003143	embryonic heart tube morphogenesis	2/385	70/23328	0.321688162	0.41221	0.24769	Sox17/En	2
GO:0007405	GO:0007405	neuroblast proliferation	2/385	70/23328	0.321688162	0.41221	0.24769	Cd24a/Ptr	2
GO:0019369	GO:0019369	arachidonic acid metabolic process	2/385	70/23328	0.321688162	0.41221	0.24769	Mgll/Ptgs1	2
GO:0048332	GO:0048332	mesoderm morphogenesis	2/385	70/23328	0.321688162	0.41221	0.24769	Bmp4/Sfrp	2
GO:0048488	GO:0048488	synaptic vesicle endocytosis	2/385	70/23328	0.321688162	0.41221	0.24769	Cd24a/Sh	2
GO:0050885	GO:0050885	neuromuscular process controlling balance	2/385	70/23328	0.321688162	0.41221	0.24769	Gm2a/Clic	2
GO:0140238	GO:0140238	presynaptic endocytosis	2/385	70/23328	0.321688162	0.41221	0.24769	Cd24a/Sh	2
GO:2000179	GO:2000179	positive regulation of neural precursor cell proliferation	2/385	70/23328	0.321688162	0.41221	0.24769	Prox1/Trf	2
GO:0022037	GO:0022037	metencephalon development	3/385	121/23328	0.322472159	0.41296	0.24814	Serpine2/I	3
GO:0042542	GO:0042542	response to hydrogen peroxide	3/385	121/23328	0.322472159	0.41296	0.24814	Lcn2/Aqp	3
GO:1901654	GO:1901654	response to ketone	4/385	174/23328	0.32367228	0.41437	0.24899	Adh1/Aqp	4
GO:0031929	GO:0031929	TOR signaling	3/385	122/23328	0.326988268	0.41838	0.2514	Reln/Kdr/	3
GO:0021885	GO:0021885	forebrain cell migration	2/385	71/23328	0.327745871	0.41838	0.2514	Reln/Cxcl	2
GO:0050909	GO:0050909	sensory perception of taste	2/385	71/23328	0.327745871	0.41838	0.2514	Cd36/Rtp	2
GO:0001829	GO:0001829	trophectodermal cell differentiation	1/385	24/23328	0.329407825	0.41838	0.2514	Junb	1
GO:0002864	GO:0002864	regulation of acute inflammatory response to antigenic	1/385	24/23328	0.329407825	0.41838	0.2514	C3	1
GO:0002903	GO:0002903	negative regulation of B cell apoptotic process	1/385	24/23328	0.329407825	0.41838	0.2514	Cd74	1
GO:0003272	GO:0003272	endocardial cushion formation	1/385	24/23328	0.329407825	0.41838	0.2514	Eng	1
GO:0022010	GO:0022010	central nervous system myelination	1/385	24/23328	0.329407825	0.41838	0.2514	Clu	1
GO:0030859	GO:0030859	polarized epithelial cell differentiation	1/385	24/23328	0.329407825	0.41838	0.2514	Tcf15	1
GO:0030903	GO:0030903	notochord development	1/385	24/23328	0.329407825	0.41838	0.2514	Id3	1
GO:0031643	GO:0031643	positive regulation of myelination	1/385	24/23328	0.329407825	0.41838	0.2514	Trf	1
GO:0032291	GO:0032291	axon ensheathment in central nervous system	1/385	24/23328	0.329407825	0.41838	0.2514	Clu	1
GO:0035510	GO:0035510	DNA dealkylation	1/385	24/23328	0.329407825	0.41838	0.2514	Apobec3	1
GO:0035743	GO:0035743	CD4-positive, alpha-beta T cell cytokine production	1/385	24/23328	0.329407825	0.41838	0.2514	Rsad2	1
GO:0040033	GO:0040033	negative regulation of translation, ncRNA-mediated	1/385	24/23328	0.329407825	0.41838	0.2514	Zfp36	1
GO:0045974	GO:0045974	regulation of translation, ncRNA-mediated	1/385	24/23328	0.329407825	0.41838	0.2514	Zfp36	1
GO:0046823	GO:0046823	negative regulation of nucleocytoplasmic transport	1/385	24/23328	0.329407825	0.41838	0.2514	Cd36	1
GO:0051457	GO:0051457	maintenance of protein location in nucleus	1/385	24/23328	0.329407825	0.41838	0.2514	Chchd10	1
GO:0062009	GO:0062009	secondary palate development	1/385	24/23328	0.329407825	0.41838	0.2514	Tbx1	1
GO:0070498	GO:0070498	interleukin-1-mediated signaling pathway	1/385	24/23328	0.329407825	0.41838	0.2514	Egr1	1
GO:0072677	GO:0072677	eosinophil migration	1/385	24/23328	0.329407825	0.41838	0.2514	Ccl21a	1
GO:0090335	GO:0090335	regulation of brown fat cell differentiation	1/385	24/23328	0.329407825	0.41838	0.2514	Metrnl	1
GO:0097150	GO:0097150	neuronal stem cell population maintenance	1/385	24/23328	0.329407825	0.41838	0.2514	Prox1	1
GO:0099623	GO:0099623	regulation of cardiac muscle cell membrane repolarizat	1/385	24/23328	0.329407825	0.41838	0.2514	Gja5	1
GO:1902656	GO:1902656	calcium ion import into cytosol	1/385	24/23328	0.329407825	0.41838	0.2514	Prnp	1
GO:1903077	GO:1903077	negative regulation of protein localization to plasma m	1/385	24/23328	0.329407825	0.41838	0.2514	Dab2	1
GO:2001257	GO:2001257	regulation of cation channel activity	4/385	176/23328	0.331139517	0.42041	0.25262	Reln/Cav1	4
GO:0072521	GO:0072521	purine-containing compound metabolic process	8/385	396/23328	0.331252339	0.42041	0.25262	Nupr1/Gd	8
GO:0002366	GO:0002366	leukocyte activation involved in immune response	6/385	285/23328	0.331407874	0.42041	0.25262	Fgl2/Il33/	6
GO:0030072	GO:0030072	peptide hormone secretion	6/385	285/23328	0.331407874	0.42041	0.25262	Sfrp1/Cpe	6
GO:1904888	GO:1904888	cranial skeletal system development	2/385	72/23328	0.333788935	0.42331	0.25436	Tbx1/Bmp	2
GO:0019318	GO:0019318	hexose metabolic process	5/385	231/23328	0.334050815	0.42351	0.25448	Atf3/Fabp	5
GO:0033157	GO:0033157	regulation of intracellular protein transport	5/385	232/23328	0.337303548	0.4275	0.25688	Cd36/Prnp	5
GO:0009988	GO:0009988	cell-cell recognition	2/385	73/23328	0.339816188	0.42797	0.25716	Cd9/Aldoa	2
GO:0021879	GO:0021879	forebrain neuron differentiation	2/385	73/23328	0.339816188	0.42797	0.25716	B2m/Prox	2
GO:0002093	GO:0002093	auditory receptor cell morphogenesis	1/385	25/23328	0.340486523	0.42797	0.25716	Clic5	1
GO:0002418	GO:0002418	immune response to tumor cell	1/385	25/23328	0.340486523	0.42797	0.25716	Cd24a	1
GO:0007274	GO:0007274	neuromuscular synaptic transmission	1/385	25/23328	0.340486523	0.42797	0.25716	Cd24a	1
GO:0007413	GO:0007413	axonal fasciculation	1/385	25/23328	0.340486523	0.42797	0.25716	Ncam1	1
GO:0009394	GO:0009394	2'-deoxyribonucleotide metabolic process	1/385	25/23328	0.340486523	0.42797	0.25716	Cmpk2	1
GO:0010640	GO:0010640	regulation of platelet-derived growth factor receptor sig	1/385	25/23328	0.340486523	0.42797	0.25716	Lox	1
GO:0010759	GO:0010759	positive regulation of macrophage chemotaxis	1/385	25/23328	0.340486523	0.42797	0.25716	Thbs1	1
GO:0010954	GO:0010954	positive regulation of protein processing	1/385	25/23328	0.340486523	0.42797	0.25716	Spon1	1
GO:0018195	GO:0018195	peptidyl-arginine modification	1/385	25/23328	0.340486523	0.42797	0.25716	Art3	1
GO:0019068	GO:0019068	virion assembly	1/385	25/23328	0.340486523	0.42797	0.25716	Apoe	1
GO:0019692	GO:0019692	deoxyribose phosphate metabolic process	1/385	25/23328	0.340486523	0.42797	0.25716	Cmpk2	1
GO:0032753	GO:0032753	positive regulation of interleukin-4 production	1/385	25/23328	0.340486523	0.42797	0.25716	Il33	1
GO:0034114	GO:0034114	regulation of heterotypic cell-cell adhesion	1/385	25/23328	0.340486523	0.42797	0.25716	Thy1	1
GO:0035813	GO:0035813	regulation of renal sodium excretion	1/385	25/23328	0.340486523	0.42797	0.25716	Edn1	1
GO:0044003	GO:0044003	modulation by symbiont of host process	1/385	25/23328	0.340486523	0.42797	0.25716	Fbln1	1
GO:0060004	GO:0060004	reflex	1/385	25/23328	0.340486523	0.42797	0.25716	Pmp22	1
GO:0060216	GO:0060216	definitive hemopoiesis	1/385	25/23328	0.340486523	0.42797	0.25716	Mfap5	1
GO:0061213	GO:0061213	positive regulation of mesonephros development	1/385	25/23328	0.340486523	0.42797	0.25716	Bmp4	1
GO:0072528	GO:0072528	pyrimidine-containing compound biosynthetic process	1/385	25/23328	0.340486523	0.42797	0.25716	Cmpk2	1
GO:0086019	GO:0086019	cell-cell signaling involved in cardiac conduction	1/385	25/23328	0.340486523	0.42797	0.25716	Gja5	1
GO:0090169	GO:0090169	regulation of spindle assembly	1/385	25/23328	0.340486523	0.42797	0.25716	Hspa1a	1
GO:0106030	GO:0106030	neuron projection fasciculation	1/385	25/23328	0.340486523	0.42797	0.25716	Ncam1	1
GO:1903798	GO:1903798	regulation of production of miRNAs involved in gene si	1/385	25/23328	0.340486523	0.42797	0.25716	Bmp4	1
GO:1905208	GO:1905208	negative regulation of cardiocyte differentiation	1/385	25/23328	0.340486523	0.42797	0.25716	Rgs4	1
GO:2000193	GO:2000193	positive regulation of fatty acid transport	1/385	25/23328	0.340486523	0.42797	0.25716	Edn1	1
GO:0043123	GO:0043123	positive regulation of I-kappaB kinase/NF-kappaB sign	3/385	125/23328	0.340532277	0.42797	0.25716	Cd36/Tgm	3
GO:0002263	GO:0002263	cell activation involved in immune response	6/385	289/23328	0.343069041	0.43102	0.259	Fgl2/Il33/	6
GO:0031341	GO:0031341	regulation of cell killing	3/385	126/23328	0.345043492	0.43338	0.26041	Cfh/B2m/	3
GO:0002292	GO:0002292	T cell differentiation involved in immune response	2/385	74/23328	0.345826508	0.43384	0.26069	Fgl2/Anxa	2

GO:0009060	GO:0009060	aerobic respiration	2/385	74/23328	0.345826508	0.43384	0.26069	Cox4i2/Cł	2
GO:0046824	GO:0046824	positive regulation of nucleocytoplasmic transport	2/385	74/23328	0.345826508	0.43384	0.26069	Gas6/Tek	2
GO:1901184	GO:1901184	regulation of ERBB signaling pathway	2/385	74/23328	0.345826508	0.43384	0.26069	Lgmn/Ptpı	2
GO:0000075	GO:0000075	cell cycle checkpoint	4/385	180/23328	0.346097932	0.43405	0.26082	Plk2/Cdkr	4
GO:0006470	GO:0006470	protein dephosphorylation	5/385	235/23328	0.347076422	0.43501	0.2614	Cdh5/Hsp	5
GO:0021953	GO:0021953	central nervous system neuron differentiation	5/385	235/23328	0.347076422	0.43501	0.2614	Sfrp1/Btg2	5
GO:0051101	GO:0051101	regulation of DNA binding	3/385	127/23328	0.349551997	0.43662	0.26236	Id1/Jun/Hı	3
GO:0060996	GO:0060996	dendritic spine development	3/385	127/23328	0.349551997	0.43662	0.26236	Reln/Plk2/	3
GO:0002455	GO:0002455	humoral immune response mediated by circulating imr	4/385	181/23328	0.349840405	0.43662	0.26236	Serping1/l	4
GO:0000737	GO:0000737	DNA catabolic process, endonucleolytic	1/385	26/23328	0.351382661	0.43662	0.26236	Igfbp3	1
GO:0006195	GO:0006195	purine nucleotide catabolic process	1/385	26/23328	0.351382661	0.43662	0.26236	Pde2a	1
GO:0007289	GO:0007289	spermatid nucleus differentiation	1/385	26/23328	0.351382661	0.43662	0.26236	Hmgb2	1
GO:0007620	GO:0007620	copulation	1/385	26/23328	0.351382661	0.43662	0.26236	Serpine2	1
GO:0010829	GO:0010829	negative regulation of glucose transmembrane transpc	1/385	26/23328	0.351382661	0.43662	0.26236	Fabp5	1
GO:0021801	GO:0021801	cerebral cortex radial glia guided migration	1/385	26/23328	0.351382661	0.43662	0.26236	Reln	1
GO:0022030	GO:0022030	telencephalon glial cell migration	1/385	26/23328	0.351382661	0.43662	0.26236	Reln	1
GO:0031100	GO:0031100	animal organ regeneration	1/385	26/23328	0.351382661	0.43662	0.26236	Cxcl12	1
GO:0032303	GO:0032303	regulation of icosanoid secretion	1/385	26/23328	0.351382661	0.43662	0.26236	Edn1	1
GO:0032800	GO:0032800	receptor biosynthetic process	1/385	26/23328	0.351382661	0.43662	0.26236	Edn1	1
GO:0035929	GO:0035929	steroid hormone secretion	1/385	26/23328	0.351382661	0.43662	0.26236	Dab2	1
GO:0042104	GO:0042104	positive regulation of activated T cell proliferation	1/385	26/23328	0.351382661	0.43662	0.26236	Cd24a	1
GO:0051788	GO:0051788	response to misfolded protein	1/385	26/23328	0.351382661	0.43662	0.26236	Clu	1
GO:0060065	GO:0060065	uterus development	1/385	26/23328	0.351382661	0.43662	0.26236	Cdkn1c	1
GO:0060338	GO:0060338	regulation of type I interferon-mediated signaling pathv	1/385	26/23328	0.351382661	0.43662	0.26236	Irf7	1
GO:0065005	GO:0065005	protein-lipid complex assembly	1/385	26/23328	0.351382661	0.43662	0.26236	Apoe	1
GO:0070233	GO:0070233	negative regulation of T cell apoptotic process	1/385	26/23328	0.351382661	0.43662	0.26236	Bmp4	1
GO:0070920	GO:0070920	regulation of production of small RNA involved in gene	1/385	26/23328	0.351382661	0.43662	0.26236	Bmp4	1
GO:0071474	GO:0071474	cellular hyperosmotic response	1/385	26/23328	0.351382661	0.43662	0.26236	Aqp1	1
GO:0090189	GO:0090189	regulation of branching involved in ureteric bud morph	1/385	26/23328	0.351382661	0.43662	0.26236	Bmp4	1
GO:0090344	GO:0090344	negative regulation of cell aging	1/385	26/23328	0.351382661	0.43662	0.26236	Plk2	1
GO:0120033	GO:0120033	negative regulation of plasma membrane bounded cell	1/385	26/23328	0.351382661	0.43662	0.26236	Kank1	1
GO:0140058	GO:0140058	neuron projection arborization	1/385	26/23328	0.351382661	0.43662	0.26236	Ptn	1
GO:1902175	GO:1902175	regulation of oxidative stress-induced intrinsic apoptoti	1/385	26/23328	0.351382661	0.43662	0.26236	Hspb1	1
GO:1904376	GO:1904376	negative regulation of protein localization to cell periph	1/385	26/23328	0.351382661	0.43662	0.26236	Dab2	1
GO:2000311	GO:2000311	regulation of AMPA receptor activity	1/385	26/23328	0.351382661	0.43662	0.26236	Reln	1
GO:0048645	GO:0048645	animal organ formation	2/385	75/23328	0.351818813	0.43703	0.26261	Tbx1/Bmp	2
GO:0061013	GO:0061013	regulation of mRNA catabolic process	3/385	128/23328	0.354057219	0.43968	0.2642	Vim/Btg2/	3
GO:0008543	GO:0008543	fibroblast growth factor receptor signaling pathway	2/385	76/23328	0.357792063	0.44406	0.26683	Pdgfb/Thb	2
GO:0045670	GO:0045670	regulation of osteoclast differentiation	2/385	76/23328	0.357792063	0.44406	0.26683	Sfrp1/Fos	2
GO:0002068	GO:0002068	glandular epithelial cell development	1/385	27/23328	0.362099238	0.44596	0.26798	Bmp4	1
GO:0003171	GO:0003171	atrioventricular valve development	1/385	27/23328	0.362099238	0.44596	0.26798	Gja5	1
GO:0003338	GO:0003338	metanephros morphogenesis	1/385	27/23328	0.362099238	0.44596	0.26798	Bmp4	1
GO:0006925	GO:0006925	inflammatory cell apoptotic process	1/385	27/23328	0.362099238	0.44596	0.26798	Anxa1	1
GO:0015874	GO:0015874	norepinephrine transport	1/385	27/23328	0.362099238	0.44596	0.26798	Ptgs1	1
GO:0016048	GO:0016048	detection of temperature stimulus	1/385	27/23328	0.362099238	0.44596	0.26798	Cxcl12	1
GO:0021511	GO:0021511	spinal cord patterning	1/385	27/23328	0.362099238	0.44596	0.26798	Reln	1
GO:0021871	GO:0021871	forebrain regionalization	1/385	27/23328	0.362099238	0.44596	0.26798	Bmp4	1
GO:0030212	GO:0030212	hyaluronan metabolic process	1/385	27/23328	0.362099238	0.44596	0.26798	Pdgfb	1
GO:0030970	GO:0030970	retrograde protein transport, ER to cytosol	1/385	27/23328	0.362099238	0.44596	0.26798	Hsp90b1	1
GO:0032069	GO:0032069	regulation of nuclease activity	1/385	27/23328	0.362099238	0.44596	0.26798	Hmgb2	1
GO:0035812	GO:0035812	renal sodium excretion	1/385	27/23328	0.362099238	0.44596	0.26798	Edn1	1
GO:0042059	GO:0042059	negative regulation of epidermal growth factor recepto	1/385	27/23328	0.362099238	0.44596	0.26798	Ptprf	1
GO:0042908	GO:0042908	xenobiotic transport	1/385	27/23328	0.362099238	0.44596	0.26798	Slc6a6	1
GO:0046639	GO:0046639	negative regulation of alpha-beta T cell differentiation	1/385	27/23328	0.362099238	0.44596	0.26798	Anxa1	1
GO:0048305	GO:0048305	immunoglobulin secretion	1/385	27/23328	0.362099238	0.44596	0.26798	Il33	1
GO:0048843	GO:0048843	negative regulation of axon extension involved in axon	1/385	27/23328	0.362099238	0.44596	0.26798	Sema3g	1
GO:0050849	GO:0050849	negative regulation of calcium-mediated signaling	1/385	27/23328	0.362099238	0.44596	0.26798	Prnp	1
GO:0060045	GO:0060045	positive regulation of cardiac muscle cell proliferation	1/385	27/23328	0.362099238	0.44596	0.26798	Ncam1	1
GO:0060716	GO:0060716	labyrinthine layer blood vessel development	1/385	27/23328	0.362099238	0.44596	0.26798	Junb	1
GO:0061003	GO:0061003	positive regulation of dendritic spine morphogenesis	1/385	27/23328	0.362099238	0.44596	0.26798	Reln	1
GO:0071472	GO:0071472	cellular response to salt stress	1/385	27/23328	0.362099238	0.44596	0.26798	Aqp1	1
GO:1901018	GO:1901018	positive regulation of potassium ion transmembrane tra	1/385	27/23328	0.362099238	0.44596	0.26798	Atp1b1	1
GO:1903319	GO:1903319	positive regulation of protein maturation	1/385	27/23328	0.362099238	0.44596	0.26798	Spon1	1
GO:1903513	GO:1903513	endoplasmic reticulum to cytosol transport	1/385	27/23328	0.362099238	0.44596	0.26798	Hsp90b1	1
GO:1905939	GO:1905939	regulation of gonad development	1/385	27/23328	0.362099238	0.44596	0.26798	Nupr1	1
GO:0033143	GO:0033143	regulation of intracellular steroid hormone receptor sig	2/385	77/23328	0.363745254	0.4476	0.26896	Sfrp1/Dab	2
GO:0045843	GO:0045843	negative regulation of striated muscle tissue developm	2/385	77/23328	0.363745254	0.4476	0.26896	Rgs4/Bmp	2
GO:2000573	GO:2000573	positive regulation of DNA biosynthetic process	2/385	77/23328	0.363745254	0.4476	0.26896	Pdgfb/Par	2
GO:0002705	GO:0002705	positive regulation of leukocyte mediated immunity	4/385	185/23328	0.364811182	0.44838	0.26943	Rsad2/Cd	4
GO:0009749	GO:0009749	response to glucose	4/385	185/23328	0.364811182	0.44838	0.26943	Vcam1/Th	4
GO:0043122	GO:0043122	regulation of I-kappaB kinase/NF-kappaB signaling	4/385	185/23328	0.364811182	0.44838	0.26943	Cd36/Hsp	4
GO:0071229	GO:0071229	cellular response to acid chemical	4/385	185/23328	0.364811182	0.44838	0.26943	Col5a2/Ar	4
GO:0006665	GO:0006665	sphingolipid metabolic process	3/385	131/23328	0.367547601	0.45148	0.27129	Plpp3/Gm	3
GO:0007093	GO:0007093	mitotic cell cycle checkpoint	3/385	131/23328	0.367547601	0.45148	0.27129	Plk2/Cdkr	3
GO:0006163	GO:0006163	purine nucleotide metabolic process	7/385	354/23328	0.367956269	0.45185	0.27151	Nupr1/Ald	7
GO:0019439	GO:0019439	aromatic compound catabolic process	8/385	411/23328	0.368408229	0.45227	0.27177	Vim/Btg2/	8
GO:0002066	GO:0002066	columnar/cuboidal epithelial cell development	2/385	78/23328	0.369677423	0.45356	0.27254	Bmp4/Clic	2
GO:0009142	GO:0009142	nucleoside triphosphate biosynthetic process	2/385	78/23328	0.369677423	0.45356	0.27254	Aldoa/Cm	2
GO:0002456	GO:0002456	T cell mediated immunity	3/385	132/23328	0.372034156	0.454	0.27281	Rsad2/Cd	3
GO:0050851	GO:0050851	antigen receptor-mediated signaling pathway	6/385	299/23328	0.372374326	0.454	0.27281	Thy1/Prnp	6
GO:0002347	GO:0002347	response to tumor cell	1/385	28/23328	0.372639206	0.454	0.27281	Cd24a	1
GO:0008340	GO:0008340	determination of adult lifespan	1/385	28/23328	0.372639206	0.454	0.27281	Cfh	1
GO:0010155	GO:0010155	regulation of proton transport	1/385	28/23328	0.372639206	0.454	0.27281	Slc25a4	1
GO:0030878	GO:0030878	thyroid gland development	1/385	28/23328	0.372639206	0.454	0.27281	Tbx1	1
GO:0032469	GO:0032469	endoplasmic reticulum calcium ion homeostasis	1/385	28/23328	0.372639206	0.454	0.27281	Tgm2	1
GO:0032647	GO:0032647	regulation of interferon-alpha production	1/385	28/23328	0.372639206	0.454	0.27281	Irf7	1
GO:0033233	GO:0033233	regulation of protein sumoylation	1/385	28/23328	0.372639206	0.454	0.27281	Egr1	1
GO:0034390	GO:0034390	smooth muscle cell apoptotic process	1/385	28/23328	0.372639206	0.454	0.27281	Edn1	1
GO:0034391	GO:0034391	regulation of smooth muscle cell apoptotic process	1/385	28/23328	0.372639206	0.454	0.27281	Edn1	1
GO:0045061	GO:0045061	thymic T cell selection	1/385	28/23328	0.372639206	0.454	0.27281	Cd74	1
GO:0045648	GO:0045648	positive regulation of erythrocyte differentiation	1/385	28/23328	0.372639206	0.454	0.27281	Hmgb2	1
GO:0048596	GO:0048596	embryonic camera-type eye morphogenesis	1/385	28/23328	0.372639206	0.454	0.27281	Prox1	1
GO:0051560	GO:0051560	mitochondrial calcium ion homeostasis	1/385	28/23328	0.372639206	0.454	0.27281	Tgm2	1
GO:0061217	GO:0061217	regulation of mesonephros development	1/385	28/23328	0.372639206	0.454	0.27281	Bmp4	1
GO:0072395	GO:0072395	signal transduction involved in cell cycle checkpoint	1/385	28/23328	0.372639206	0.454	0.27281	Cdkn1a	1
GO:0072401	GO:0072401	signal transduction involved in DNA integrity checkpoir	1/385	28/23328	0.372639206	0.454	0.27281	Cdkn1a	1
GO:0072422	GO:0072422	signal transduction involved in DNA damage checkpoi	1/385	28/23328	0.372639206	0.454	0.27281	Cdkn1a	1
GO:1902668	GO:1902668	negative regulation of axon guidance	1/385	28/23328	0.372639206	0.454	0.27281	Sema3g	1
GO:1903649	GO:1903649	regulation of cytoplasmic transport	1/385	28/23328	0.372639206	0.454	0.27281	Dab2	1
GO:1903671	GO:1903671	negative regulation of sprouting angiogenesis	1/385	28/23328	0.372639206	0.454	0.27281	Thbs1	1
GO:1904861	GO:1904861	excitatory synapse assembly	1/385	28/23328	0.372639206	0.454	0.27281	Reln	1
GO:2000310	GO:2000310	regulation of NMDA receptor activity	1/385	28/23328	0.372639206	0.454	0.27281	Reln	1
GO:0002260	GO:0002260	lymphocyte homeostasis	2/385	79/23328	0.375587643	0.4568	0.27449	Cd24a/Cd	2
GO:0034644	GO:0034644	cellular response to UV	2/385	79/23328	0.375587643	0.4568	0.27449	Aqp1/Cdk	2
GO:0045471	GO:0045471	response to ethanol	2/385	79/23328	0.375587643	0.4568	0.27449	Adh7/Adh	2
GO:0048635	GO:0048635	negative regulation of muscle organ development	2/385	79/23328	0.375587643	0.4568	0.27449	Rgs4/Bmp	2
GO:0048663	GO:0048663	neuron fate commitment	2/385	79/23328	0.375587643	0.4568	0.27449	Notch3/Bı	2
GO:0051966	GO:0051966	regulation of synaptic transmission, glutamatergic	2/385	79/23328	0.375587643	0.4568	0.27449	Reln/Serp	2
GO:0009746	GO:0009746	response to hexose	4/385	188/23328	0.37603063	0.4572	0.27473	Vcam1/Th	4
GO:0034284	GO:0034284	response to monosaccharide	4/385	189/23328	0.379767026	0.46161	0.27738	Vcam1/Th	4
GO:0009791	GO:0009791	post-embryonic development	3/385	134/23328	0.380988758	0.46261	0.27798	Flt1/Kdr/B	3
GO:0044839	GO:0044839	cell cycle G2/M phase transition	3/385	134/23328	0.380988758	0.46261	0.27798	Fhl1/Cdkn	3
GO:0036465	GO:0036465	synaptic vesicle recycling	2/385	80/23328	0.381475022	0.46261	0.27798	Cd24a/Sh	2
GO:0038034	GO:0038034	signal transduction in absence of ligand	2/385	80/23328	0.381475022	0.46261	0.27798	Lcn2/Kitl	2
GO:0060968	GO:0060968	regulation of gene silencing	2/385	80/23328	0.381475022	0.46261	0.27798	Zfp36/Bm	2
GO:0097192	GO:0097192	extrinsic apoptotic signaling pathway in absence of lig	2/385	80/23328	0.381475022	0.46261	0.27798	Lcn2/Kitl	2
GO:1901880	GO:1901880	negative regulation of protein depolymerization	2/385	80/23328	0.381475022	0.46261	0.27798	Stmn2/Tpı	2
GO:2001259	GO:2001259	positive regulation of cation channel activity	2/385	80/23328	0.381475022	0.46261	0.27798	Reln/Bmp	2
GO:0006220	GO:0006220	pyrimidine nucleotide metabolic process	1/385	29/23328	0.383005468	0.46286	0.27813	Cmpk2	1
GO:0010893	GO:0010893	positive regulation of steroid biosynthetic process	1/385	29/23328	0.383005468	0.46286	0.27813	Dab2	1
GO:0030318	GO:0030318	melanocyte differentiation	1/385	29/23328	0.383005468	0.46286	0.27813	Kitl	1
GO:0035094	GO:0035094	response to nicotine	1/385	29/23328	0.383005468	0.46286	0.27813	B2m	1
GO:0039694	GO:0039694	viral RNA genome replication	1/385	29/23328	0.383005468	0.46286	0.27813	Apobec3	1
GO:0045109	GO:0045109	intermediate filament organization	1/385	29/23328	0.383005468	0.46286	0.27813	Vim	1
GO:0045589	GO:0045589	regulation of regulatory T cell differentiation	1/385	29/23328	0.383005468	0.46286	0.27813	Ctla2a	1
GO:0070296	GO:0070296	sarcoplasmic reticulum calcium ion transport	1/385	29/23328	0.383005468	0.46286	0.27813	Sln	1
GO:0090314	GO:0090314	positive regulation of protein targeting to membrane	1/385	29/23328	0.383005468	0.46286	0.27813	Prnp	1
GO:0099622	GO:0099622	cardiac muscle cell membrane repolarization	1/385	29/23328	0.383005468	0.46286	0.27813	Gja5	1
GO:1900078	GO:1900078	positive regulation of cellular response to insulin stimu	1/385	29/23328	0.383005468	0.46286	0.27813	C1qtnf9	1

GO:1904292	GO:1904292	regulation of ERAD pathway	1/385	29/23328	0.383005468	0.46286	0.27813	Cav1	1
GO:0042113	GO:0042113	B cell activation	8/385	417/23328	0.38338365	0.46318	0.27832	Sfrp1/Cd3	8
GO:0046883	GO:0046883	regulation of hormone secretion	6/385	303/23328	0.384128224	0.46395	0.27878	Sfrp1/Cd3	6
GO:0000280	GO:0000280	nuclear division	8/385	418/23328	0.385883112	0.46593	0.27998	Rgcc/Pde	8
GO:0045682	GO:0045682	regulation of epidermis development	2/385	81/23328	0.387338706	0.46742	0.28087	Zfp36/Bm	2
GO:0051881	GO:0051881	regulation of mitochondrial membrane potential	2/385	81/23328	0.387338706	0.46742	0.28087	Kdr/Dcn	2
GO:0032479	GO:0032479	regulation of type I interferon production	2/385	82/23328	0.393177873	0.47245	0.28389	Irf7/Hmgb	2
GO:0001759	GO:0001759	organ induction	1/385	30/23328	0.393200879	0.47245	0.28389	Bmp4	1
GO:0003309	GO:0003309	type B pancreatic cell differentiation	1/385	30/23328	0.393200879	0.47245	0.28389	Bmp4	1
GO:0009262	GO:0009262	deoxyribonucleotide metabolic process	1/385	30/23328	0.393200879	0.47245	0.28389	Cmpk2	1
GO:0019433	GO:0019433	triglyceride catabolic process	1/385	30/23328	0.393200879	0.47245	0.28389	Lpl	1
GO:0021602	GO:0021602	cranial nerve morphogenesis	1/385	30/23328	0.393200879	0.47245	0.28389	Tbx1	1
GO:0032607	GO:0032607	interferon-alpha production	1/385	30/23328	0.393200879	0.47245	0.28389	Irf7	1
GO:0032816	GO:0032816	positive regulation of natural killer cell activation	1/385	30/23328	0.393200879	0.47245	0.28389	Gas6	1
GO:0043372	GO:0043372	positive regulation of CD4-positive, alpha-beta T cell d	1/385	30/23328	0.393200879	0.47245	0.28389	Anxa1	1
GO:0043457	GO:0043457	regulation of cellular respiration	1/385	30/23328	0.393200879	0.47245	0.28389	Cbfa2t3	1
GO:0045671	GO:0045671	negative regulation of osteoclast differentiation	1/385	30/23328	0.393200879	0.47245	0.28389	Sfrp1	1
GO:0048714	GO:0048714	positive regulation of oligodendrocyte differentiation	1/385	30/23328	0.393200879	0.47245	0.28389	Ptn	1
GO:0050951	GO:0050951	sensory perception of temperature stimulus	1/385	30/23328	0.393200879	0.47245	0.28389	Cxcl12	1
GO:0051957	GO:0051957	positive regulation of amino acid transport	1/385	30/23328	0.393200879	0.47245	0.28389	Itgb1	1
GO:0060317	GO:0060317	cardiac epithelial to mesenchymal transition	1/385	30/23328	0.393200879	0.47245	0.28389	Eng	1
GO:0048284	GO:0048284	organelle fusion	3/385	138/23328	0.398809915	0.479	0.28783	Tbc1d4/Ar	3
GO:0002532	GO:0002532	production of molecular mediator involved in inflamma	2/385	83/23328	0.398991735	0.479	0.28783	Lbp/Ephx2	2
GO:0021872	GO:0021872	forebrain generation of neurons	2/385	83/23328	0.398991735	0.479	0.28783	B2m/Prox	2
GO:1905475	GO:1905475	regulation of protein localization to membrane	4/385	195/23328	0.402127151	0.48011	0.28849	Prnp/Ptn/I	4
GO:0000132	GO:0000132	establishment of mitotic spindle orientation	1/385	31/23328	0.403228249	0.48011	0.28849	Itgb1	1
GO:0002092	GO:0002092	positive regulation of receptor internalization	1/385	31/23328	0.403228249	0.48011	0.28849	Dab2	1
GO:0007530	GO:0007530	sex determination	1/385	31/23328	0.403228249	0.48011	0.28849	Dhh	1
GO:0010803	GO:0010803	regulation of tumor necrosis factor-mediated signaling	1/385	31/23328	0.403228249	0.48011	0.28849	Gas6	1
GO:0021533	GO:0021533	cell differentiation in hindbrain	1/385	31/23328	0.403228249	0.48011	0.28849	Prox1	1
GO:0021904	GO:0021904	dorsal/ventral neural tube patterning	1/385	31/23328	0.403228249	0.48011	0.28849	Bmp4	1
GO:0032365	GO:0032365	intracellular lipid transport	1/385	31/23328	0.403228249	0.48011	0.28849	Anxa2	1
GO:0032786	GO:0032786	positive regulation of DNA-templated transcription, elo	1/385	31/23328	0.403228249	0.48011	0.28849	Dab2	1
GO:0033137	GO:0033137	negative regulation of peptidyl-serine phosphorylation	1/385	31/23328	0.403228249	0.48011	0.28849	Cav1	1
GO:0035640	GO:0035640	exploration behavior	1/385	31/23328	0.403228249	0.48011	0.28849	Apoe	1
GO:0044030	GO:0044030	regulation of DNA methylation	1/385	31/23328	0.403228249	0.48011	0.28849	H19	1
GO:0044062	GO:0044062	regulation of excretion	1/385	31/23328	0.403228249	0.48011	0.28849	Edn1	1
GO:0044319	GO:0044319	wound healing, spreading of cells	1/385	31/23328	0.403228249	0.48011	0.28849	Pdpn	1
GO:0044788	GO:0044788	modulation by host of viral process	1/385	31/23328	0.403228249	0.48011	0.28849	Apoe	1
GO:0045920	GO:0045920	negative regulation of exocytosis	1/385	31/23328	0.403228249	0.48011	0.28849	Anxa1	1
GO:0045932	GO:0045932	negative regulation of muscle contraction	1/385	31/23328	0.403228249	0.48011	0.28849	Calcrl	1
GO:0048011	GO:0048011	neurotrophin TRK receptor signaling pathway	1/385	31/23328	0.403228249	0.48011	0.28849	Ptprf	1
GO:0051125	GO:0051125	regulation of actin nucleation	1/385	31/23328	0.403228249	0.48011	0.28849	Gmfg	1
GO:0055075	GO:0055075	potassium ion homeostasis	1/385	31/23328	0.403228249	0.48011	0.28849	Atp1b1	1
GO:0060117	GO:0060117	auditory receptor cell development	1/385	31/23328	0.403228249	0.48011	0.28849	Clic5	1
GO:0061036	GO:0061036	positive regulation of cartilage development	1/385	31/23328	0.403228249	0.48011	0.28849	Bmp4	1
GO:0086010	GO:0086010	membrane depolarization during action potential	1/385	31/23328	0.403228249	0.48011	0.28849	Gja5	1
GO:0090505	GO:0090505	epiboly involved in wound healing	1/385	31/23328	0.403228249	0.48011	0.28849	Pdpn	1
GO:0098810	GO:0098810	neurotransmitter reuptake	1/385	31/23328	0.403228249	0.48011	0.28849	Itgb1	1
GO:1902253	GO:1902253	regulation of intrinsic apoptotic signaling pathway by p5	1/385	31/23328	0.403228249	0.48011	0.28849	Cd74	1
GO:1902932	GO:1902932	positive regulation of alcohol biosynthetic process	1/385	31/23328	0.403228249	0.48011	0.28849	Dab2	1
GO:1905523	GO:1905523	positive regulation of macrophage migration	1/385	31/23328	0.403228249	0.48011	0.28849	Thbs1	1
GO:1900180	GO:1900180	regulation of protein localization to nucleus	3/385	139/23328	0.40324406	0.48011	0.28849	Cd36/Tek/	3
GO:1904375	GO:1904375	regulation of protein localization to cell periphery	3/385	140/23328	0.407668825	0.48524	0.29158	Prnp/Dab2	3
GO:0009117	GO:0009117	nucleotide metabolic process	8/385	427/23328	0.408399222	0.48597	0.29201	Nupr1/Ald	8
GO:0006911	GO:0006911	phagocytosis, engulfment	4/385	197/23328	0.409550967	0.4872	0.29276	Cd36/Thb	4
GO:0043367	GO:0043367	CD4-positive, alpha-beta T cell differentiation	2/385	85/23328	0.410540553	0.48808	0.29328	Rsad2/An	2
GO:0001556	GO:0001556	oocyte maturation	1/385	32/23328	0.41309034	0.48808	0.29328	Pde3a	1
GO:0002438	GO:0002438	acute inflammatory response to antigenic stimulus	1/385	32/23328	0.41309034	0.48808	0.29328	C3	1
GO:0007007	GO:0007007	inner mitochondrial membrane organization	1/385	32/23328	0.41309034	0.48808	0.29328	Chchd10	1
GO:0008206	GO:0008206	bile acid metabolic process	1/385	32/23328	0.41309034	0.48808	0.29328	Prox1	1
GO:0010614	GO:0010614	negative regulation of cardiac muscle hypertrophy	1/385	32/23328	0.41309034	0.48808	0.29328	Rgs4	1
GO:0016242	GO:0016242	negative regulation of macroautophagy	1/385	32/23328	0.41309034	0.48808	0.29328	Nupr1	1
GO:0021697	GO:0021697	cerebellar cortex formation	1/385	32/23328	0.41309034	0.48808	0.29328	Prox1	1
GO:0032673	GO:0032673	regulation of interleukin-4 production	1/385	32/23328	0.41309034	0.48808	0.29328	Il33	1
GO:0032692	GO:0032692	negative regulation of interleukin-1 production	1/385	32/23328	0.41309034	0.48808	0.29328	Gas6	1
GO:0032743	GO:0032743	positive regulation of interleukin-2 production	1/385	32/23328	0.41309034	0.48808	0.29328	Anxa1	1
GO:0046475	GO:0046475	glycerophospholipid catabolic process	1/385	32/23328	0.41309034	0.48808	0.29328	Scarb1	1
GO:0050482	GO:0050482	arachidonic acid secretion	1/385	32/23328	0.41309034	0.48808	0.29328	Anxa1	1
GO:0051590	GO:0051590	positive regulation of neurotransmitter transport	1/385	32/23328	0.41309034	0.48808	0.29328	Itgb1	1
GO:0055022	GO:0055022	negative regulation of cardiac muscle tissue growth	1/385	32/23328	0.41309034	0.48808	0.29328	Rgs4	1
GO:0060977	GO:0060977	coronary vasculature morphogenesis	1/385	32/23328	0.41309034	0.48808	0.29328	Tbx1	1
GO:0061117	GO:0061117	negative regulation of heart growth	1/385	32/23328	0.41309034	0.48808	0.29328	Rgs4	1
GO:0062125	GO:0062125	regulation of mitochondrial gene expression	1/385	32/23328	0.41309034	0.48808	0.29328	Chchd10	1
GO:0071354	GO:0071354	cellular response to interleukin-6	1/385	32/23328	0.41309034	0.48808	0.29328	Selp	1
GO:0090504	GO:0090504	epiboly	1/385	32/23328	0.41309034	0.48808	0.29328	Pdpn	1
GO:1901797	GO:1901797	negative regulation of signal transduction by p53 class	1/385	32/23328	0.41309034	0.48808	0.29328	Cd74	1
GO:1903963	GO:1903963	arachidonate transport	1/385	32/23328	0.41309034	0.48808	0.29328	Anxa1	1
GO:1905476	GO:1905476	negative regulation of protein localization to membrane	1/385	32/23328	0.41309034	0.48808	0.29328	Dab2	1
GO:2000515	GO:2000515	negative regulation of CD4-positive, alpha-beta T cell a	1/385	32/23328	0.41309034	0.48808	0.29328	Anxa1	1
GO:0006970	GO:0006970	response to osmotic stress	2/385	86/23328	0.416274092	0.49154	0.29536	Aqp1/Mylk	2
GO:0032606	GO:0032606	type I interferon production	2/385	86/23328	0.416274092	0.49154	0.29536	Irf7/Hmgb	2
GO:0050906	GO:0050906	detection of stimulus involved in sensory perception	3/385	142/23328	0.416488451	0.49154	0.29536	Cxcl12/Rt	3
GO:1902850	GO:1902850	microtubule cytoskeleton organization involved in mito	3/385	142/23328	0.416488451	0.49154	0.29536	Plk2/Itgb1	3
GO:0016311	GO:0016311	dephosphorylation	7/385	374/23328	0.421796188	0.4976	0.299	Cdh5/Plpp	7
GO:0035304	GO:0035304	regulation of protein dephosphorylation	2/385	87/23328	0.421979492	0.4976	0.299	Cdh5/Hsp	2
GO:0044728	GO:0044728	DNA methylation or demethylation	2/385	87/23328	0.421979492	0.4976	0.299	H19/Apob	2
GO:0033081	GO:0033081	regulation of T cell differentiation in thymus	1/385	33/23328	0.422789868	0.49771	0.29907	Bmp4	1
GO:0034260	GO:0034260	negative regulation of GTPase activity	1/385	33/23328	0.422789868	0.49771	0.29907	Pecam1	1
GO:0045066	GO:0045066	regulatory T cell differentiation	1/385	33/23328	0.422789868	0.49771	0.29907	Ctla2a	1
GO:0045616	GO:0045616	regulation of keratinocyte differentiation	1/385	33/23328	0.422789868	0.49771	0.29907	Zfp36	1
GO:0045672	GO:0045672	positive regulation of osteoclast differentiation	1/385	33/23328	0.422789868	0.49771	0.29907	Fos	1
GO:0061157	GO:0061157	mRNA destabilization	1/385	33/23328	0.422789868	0.49771	0.29907	Zfp36	1
GO:0009566	GO:0009566	fertilization	4/385	201/23328	0.424340152	0.4994	0.30009	Cd9/Hoxd	4
GO:0010508	GO:0010508	positive regulation of autophagy	3/385	144/23328	0.425265359	0.50021	0.30057	Kdr/Plk2/I	3
GO:0090316	GO:0090316	positive regulation of intracellular protein transport	3/385	144/23328	0.425265359	0.50021	0.30057	Prnp/Gas6	3
GO:0060079	GO:0060079	excitatory postsynaptic potential	2/385	88/23328	0.427656119	0.50274	0.30209	Reln/Rgs4	2
GO:1904063	GO:1904063	negative regulation of cation transmembrane transport	2/385	88/23328	0.427656119	0.50274	0.30209	Rgs4/Cav	2
GO:0006006	GO:0006006	glucose metabolic process	4/385	202/23328	0.428023635	0.50303	0.30227	Atf3/Fabp	4
GO:0017148	GO:0017148	negative regulation of translation	3/385	145/23328	0.429636762	0.50478	0.30332	Btg2/Igfbp	3
GO:0006753	GO:0006753	nucleoside phosphate metabolic process	8/385	436/23328	0.43090143	0.50553	0.30377	Nupr1/Ald	8
GO:0002285	GO:0002285	lymphocyte activation involved in immune response	4/385	203/23328	0.431701048	0.50553	0.30377	Fgl2/Cd74	4
GO:0002703	GO:0002703	regulation of leukocyte mediated immunity	5/385	261/23328	0.431818261	0.50553	0.30377	Rsad2/Cd	5
GO:0005996	GO:0005996	monosaccharide metabolic process	5/385	261/23328	0.431818261	0.50553	0.30377	Atf3/Fabp	5
GO:0021591	GO:0021591	ventricular system development	1/385	34/23328	0.432329508	0.50553	0.30377	Aqp1	1
GO:0030808	GO:0030808	regulation of nucleotide biosynthetic process	1/385	34/23328	0.432329508	0.50553	0.30377	Nos3	1
GO:0032508	GO:0032508	DNA duplex unwinding	1/385	34/23328	0.432329508	0.50553	0.30377	Anxa1	1
GO:0034243	GO:0034243	regulation of transcription elongation from RNA polyme	1/385	34/23328	0.432329508	0.50553	0.30377	Dab2	1
GO:0035883	GO:0035883	enteroendocrine cell differentiation	1/385	34/23328	0.432329508	0.50553	0.30377	Bmp4	1
GO:0043950	GO:0043950	positive regulation of cAMP-mediated signaling	1/385	34/23328	0.432329508	0.50553	0.30377	Ece1	1
GO:0048710	GO:0048710	regulation of astrocyte differentiation	1/385	34/23328	0.432329508	0.50553	0.30377	Serpine2	1
GO:0051180	GO:0051180	vitamin transport	1/385	34/23328	0.432329508	0.50553	0.30377	Scarb1	1
GO:0062149	GO:0062149	detection of stimulus involved in sensory perception of	1/385	34/23328	0.432329508	0.50553	0.30377	Cxcl12	1
GO:0070741	GO:0070741	response to interleukin-6	1/385	34/23328	0.432329508	0.50553	0.30377	Selp	1
GO:0090162	GO:0090162	establishment of epithelial cell polarity	1/385	34/23328	0.432329508	0.50553	0.30377	Tcf15	1
GO:1900371	GO:1900371	regulation of purine nucleotide biosynthetic process	1/385	34/23328	0.432329508	0.50553	0.30377	Nos3	1
GO:1901976	GO:1901976	regulation of cell cycle checkpoint	1/385	34/23328	0.432329508	0.50553	0.30377	Prox1	1
GO:0051052	GO:0051052	regulation of DNA metabolic process	7/385	379/23328	0.435221926	0.50877	0.30572	Pdgfb/Igfb	7
GO:0009165	GO:0009165	nucleotide biosynthetic process	4/385	204/23328	0.435372125	0.50881	0.30574	Aldoa/Cm	4
GO:0051092	GO:0051092	positive regulation of NF-kappaB transcription factor a	3/385	147/23328	0.438343484	0.51193	0.30761	Cd36/Clu/	3
GO:0043242	GO:0043242	negative regulation of protein-containing complex disa	2/385	90/23328	0.438920658	0.51193	0.30761	Stmn2/Tp	2
GO:0001702	GO:0001702	gastrulation with mouth forming second	1/385	35/23328	0.441711887	0.51193	0.30761	Plpp3	1
GO:0002428	GO:0002428	antigen processing and presentation of peptide antigen	1/385	35/23328	0.441711887	0.51193	0.30761	B2m	1
GO:0006308	GO:0006308	DNA catabolic process	1/385	35/23328	0.441711887	0.51193	0.30761	Igfbp3	1
GO:0006471	GO:0006471	protein ADP-ribosylation	1/385	35/23328	0.441711887	0.51193	0.30761	Art3	1
GO:0006972	GO:0006972	hyperosmotic response	1/385	35/23328	0.441711887	0.51193	0.30761	Aqp1	1
GO:0007026	GO:0007026	negative regulation of microtubule depolymerization	1/385	35/23328	0.441711887	0.51193	0.30761	Stmn2	1
GO:0009187	GO:0009187	cyclic nucleotide metabolic process	1/385	35/23328	0.441711887	0.51193	0.30761	Pde2a	1
GO:0010758	GO:0010758	regulation of macrophage chemotaxis	1/385	35/23328	0.441711887	0.51193	0.30761	Thbs1	1

GO:0010837	GO:0010837	regulation of keratinocyte proliferation	1/385	35/23328	0.441711887	0.51193	0.30761	Zfp36	1
GO:0032660	GO:0032660	regulation of interleukin-17 production	1/385	35/23328	0.441711887	0.51193	0.30761	Prnp	1
GO:0033866	GO:0033866	nucleoside bisphosphate biosynthetic process	1/385	35/23328	0.441711887	0.51193	0.30761	Papss2	1
GO:0034030	GO:0034030	ribonucleoside bisphosphate biosynthetic process	1/385	35/23328	0.441711887	0.51193	0.30761	Papss2	1
GO:0034033	GO:0034033	purine nucleoside bisphosphate biosynthetic process	1/385	35/23328	0.441711887	0.51193	0.30761	Papss2	1
GO:0035116	GO:0035116	embryonic hindlimb morphogenesis	1/385	35/23328	0.441711887	0.51193	0.30761	Bmp4	1
GO:0040001	GO:0040001	establishment of mitotic spindle localization	1/385	35/23328	0.441711887	0.51193	0.30761	Itgb1	1
GO:0043516	GO:0043516	regulation of DNA damage response, signal transducti	1/385	35/23328	0.441711887	0.51193	0.30761	Cd74	1
GO:0045686	GO:0045686	negative regulation of glial cell differentiation	1/385	35/23328	0.441711887	0.51193	0.30761	Bmp4	1
GO:0045879	GO:0045879	negative regulation of smoothened signaling pathway	1/385	35/23328	0.441711887	0.51193	0.30761	Serpine2	1
GO:0046677	GO:0046677	response to antibiotic	1/385	35/23328	0.441711887	0.51193	0.30761	Id1	1
GO:0051973	GO:0051973	positive regulation of telomerase activity	1/385	35/23328	0.441711887	0.51193	0.30761	Parm1	1
GO:0060218	GO:0060218	hematopoietic stem cell differentiation	1/385	35/23328	0.441711887	0.51193	0.30761	Sfrp1	1
GO:0060236	GO:0060236	regulation of mitotic spindle organization	1/385	35/23328	0.441711887	0.51193	0.30761	Hspa1a	1
GO:0090313	GO:0090313	regulation of protein targeting to membrane	1/385	35/23328	0.441711887	0.51193	0.30761	Prnp	1
GO:0097106	GO:0097106	postsynaptic density organization	1/385	35/23328	0.441711887	0.51193	0.30761	Reln	1
GO:1900181	GO:1900181	negative regulation of protein localization to nucleus	1/385	35/23328	0.441711887	0.51193	0.30761	Cd36	1
GO:1905898	GO:1905898	positive regulation of response to endoplasmic reticulu	1/385	35/23328	0.441711887	0.51193	0.30761	Cav1	1
GO:1990089	GO:1990089	response to nerve growth factor	1/385	35/23328	0.441711887	0.51193	0.30761	Stmn2	1
GO:1990090	GO:1990090	cellular response to nerve growth factor stimulus	1/385	35/23328	0.441711887	0.51193	0.30761	Stmn2	1
GO:0099024	GO:0099024	plasma membrane invagination	4/385	206/23328	0.442694233	0.51292	0.30821	Cd36/Thb	4
GO:0040014	GO:0040014	regulation of multicellular organism growth	2/385	91/23328	0.444507443	0.51488	0.30939	Lgmn/H19	2
GO:0009150	GO:0009150	purine ribonucleotide metabolic process	6/385	324/23328	0.445632478	0.51604	0.31009	Nupr1/Ald	6
GO:0001101	GO:0001101	response to acid chemical	4/385	207/23328	0.446344753	0.51672	0.3105	Col5a2/Ar	4
GO:0002275	GO:0002275	myeloid cell activation involved in immune response	2/385	92/23328	0.450063196	0.52003	0.31248	Il33/Lbp	2
GO:0090630	GO:0090630	activation of GTPase activity	2/385	92/23328	0.450063196	0.52003	0.31248	Arhgef15/	2
GO:0002701	GO:0002701	negative regulation of production of molecular mediato	1/385	36/23328	0.450939591	0.52003	0.31248	Il33	1
GO:0008045	GO:0008045	motor neuron axon guidance	1/385	36/23328	0.450939591	0.52003	0.31248	Cxcl12	1
GO:0009112	GO:0009112	nucleobase metabolic process	1/385	36/23328	0.450939591	0.52003	0.31248	Gda	1
GO:0031069	GO:0031069	hair follicle morphogenesis	1/385	36/23328	0.450939591	0.52003	0.31248	Igfbp5	1
GO:0031424	GO:0031424	keratinization	1/385	36/23328	0.450939591	0.52003	0.31248	Cyp26b1	1
GO:0035115	GO:0035115	embryonic forelimb morphogenesis	1/385	36/23328	0.450939591	0.52003	0.31248	Hoxd9	1
GO:0042220	GO:0042220	response to cocaine	1/385	36/23328	0.450939591	0.52003	0.31248	Ncam1	1
GO:0042832	GO:0042832	defense response to protozoan	1/385	36/23328	0.450939591	0.52003	0.31248	Iigp1	1
GO:0050779	GO:0050779	RNA destabilization	1/385	36/23328	0.450939591	0.52003	0.31248	Zfp36	1
GO:1902116	GO:1902116	negative regulation of organelle assembly	1/385	36/23328	0.450939591	0.52003	0.31248	Nupr1	1
GO:2000516	GO:2000516	positive regulation of CD4-positive, alpha-beta T cell a	1/385	36/23328	0.450939591	0.52003	0.31248	Anxa1	1
GO:2000785	GO:2000785	regulation of autophagosome assembly	1/385	36/23328	0.450939591	0.52003	0.31248	Nupr1	1
GO:0002065	GO:0002065	columnar/cuboidal epithelial cell differentiation	3/385	150/23328	0.451307684	0.52031	0.31265	Bmp4/Pro	3
GO:1901293	GO:1901293	nucleoside phosphate biosynthetic process	4/385	209/23328	0.453623481	0.52284	0.31417	Aldoa/Cm	4
GO:0007215	GO:0007215	glutamate receptor signaling pathway	2/385	93/23328	0.45558742	0.52467	0.31527	Reln/Prnp	2
GO:0008589	GO:0008589	regulation of smoothened signaling pathway	2/385	93/23328	0.45558742	0.52467	0.31527	Sfrp1/Ser	2
GO:0009308	GO:0009308	amine metabolic process	2/385	93/23328	0.45558742	0.52467	0.31527	Cyp1a1/V	2
GO:0001782	GO:0001782	B cell homeostasis	1/385	37/23328	0.460015165	0.52802	0.31728	Cd74	1
GO:0002675	GO:0002675	positive regulation of acute inflammatory response	1/385	37/23328	0.460015165	0.52802	0.31728	C3	1
GO:0007099	GO:0007099	centriole replication	1/385	37/23328	0.460015165	0.52802	0.31728	Plk2	1
GO:0009066	GO:0009066	aspartate family amino acid metabolic process	1/385	37/23328	0.460015165	0.52802	0.31728	Ass1	1
GO:0014823	GO:0014823	response to activity	1/385	37/23328	0.460015165	0.52802	0.31728	Metrnl	1
GO:0021680	GO:0021680	cerebellar Purkinje cell layer development	1/385	37/23328	0.460015165	0.52802	0.31728	Hspa5	1
GO:0030574	GO:0030574	collagen catabolic process	1/385	37/23328	0.460015165	0.52802	0.31728	Itgb1	1
GO:0034314	GO:0034314	Arp2/3 complex-mediated actin nucleation	1/385	37/23328	0.460015165	0.52802	0.31728	Gmfg	1
GO:0040020	GO:0040020	regulation of meiotic nuclear division	1/385	37/23328	0.460015165	0.52802	0.31728	Pde3a	1
GO:0045047	GO:0045047	protein targeting to ER	1/385	37/23328	0.460015165	0.52802	0.31728	Hspa5	1
GO:0051294	GO:0051294	establishment of spindle orientation	1/385	37/23328	0.460015165	0.52802	0.31728	Itgb1	1
GO:0098751	GO:0098751	bone cell development	1/385	37/23328	0.460015165	0.52802	0.31728	Anxa2	1
GO:0050864	GO:0050864	regulation of B cell activation	5/385	270/23328	0.460742542	0.52871	0.3177	Sfrp1/Cd3	5
GO:1903313	GO:1903313	positive regulation of mRNA metabolic process	2/385	94/23328	0.46107964	0.52895	0.31784	Btg2/Zfp3	2
GO:0002429	GO:0002429	immune response-activating cell surface receptor sign	6/385	330/23328	0.463014997	0.53103	0.31909	Thy1/Prnp	6
GO:0002757	GO:0002757	immune response-activating signal transduction	6/385	331/23328	0.465899229	0.53419	0.32099	Thy1/Prnp	6
GO:0010324	GO:0010324	membrane invagination	4/385	213/23328	0.468084976	0.53533	0.32168	Cd36/Thb	4
GO:0007338	GO:0007338	single fertilization	3/385	154/23328	0.468399367	0.53533	0.32168	Cd9/Hoxd	3
GO:0009311	GO:0009311	oligosaccharide metabolic process	1/385	38/23328	0.468941109	0.53533	0.32168	Gm2a	1
GO:0018208	GO:0018208	peptidyl-proline modification	1/385	38/23328	0.468941109	0.53533	0.32168	Ppic	1
GO:0021799	GO:0021799	cerebral cortex radially oriented cell migration	1/385	38/23328	0.468941109	0.53533	0.32168	Reln	1
GO:0031114	GO:0031114	regulation of microtubule depolymerization	1/385	38/23328	0.468941109	0.53533	0.32168	Stmn2	1
GO:0032733	GO:0032733	positive regulation of interleukin-10 production	1/385	38/23328	0.468941109	0.53533	0.32168	Cd34	1
GO:0045622	GO:0045622	regulation of T-helper cell differentiation	1/385	38/23328	0.468941109	0.53533	0.32168	Anxa1	1
GO:0045684	GO:0045684	positive regulation of epidermis development	1/385	38/23328	0.468941109	0.53533	0.32168	Bmp4	1
GO:0046640	GO:0046640	regulation of alpha-beta T cell proliferation	1/385	38/23328	0.468941109	0.53533	0.32168	Cd24a	1
GO:0051968	GO:0051968	positive regulation of synaptic transmission, glutamate	1/385	38/23328	0.468941109	0.53533	0.32168	Reln	1
GO:0071391	GO:0071391	cellular response to estrogen stimulus	1/385	38/23328	0.468941109	0.53533	0.32168	Sfrp1	1
GO:0097028	GO:0097028	dendritic cell differentiation	1/385	38/23328	0.468941109	0.53533	0.32168	Tmem176	1
GO:0098534	GO:0098534	centriole assembly	1/385	38/23328	0.468941109	0.53533	0.32168	Plk2	1
GO:0110111	GO:0110111	negative regulation of animal organ morphogenesis	1/385	38/23328	0.468941109	0.53533	0.32168	Bmp4	1
GO:2000036	GO:2000036	regulation of stem cell population maintenance	1/385	38/23328	0.468941109	0.53533	0.32168	Ptn	1
GO:2000243	GO:2000243	positive regulation of reproductive process	2/385	96/23328	0.471966293	0.53864	0.32366	Pde3a/Pla	2
GO:0071322	GO:0071322	cellular response to carbohydrate stimulus	3/385	155/23328	0.472635435	0.53925	0.32403	Vcam1/Ga	3
GO:0007605	GO:0007605	sensory perception of sound	5/385	274/23328	0.473465071	0.54005	0.32451	Tbx1/Pkh	5
GO:0009259	GO:0009259	ribonucleotide metabolic process	6/385	334/23328	0.474527256	0.54112	0.32515	Nupr1/Ald	6
GO:0007224	GO:0007224	smoothened signaling pathway	3/385	156/23328	0.476856134	0.54239	0.32592	Sfrp1/Dhh	3
GO:0006885	GO:0006885	regulation of pH	2/385	97/23328	0.477359896	0.54239	0.32592	Car4/Edn1	2
GO:0002475	GO:0002475	antigen processing and presentation via MHC class Ib	1/385	39/23328	0.477719884	0.54239	0.32592	B2m	1
GO:0006778	GO:0006778	porphyrin-containing compound metabolic process	1/385	39/23328	0.477719884	0.54239	0.32592	Cyp1a1	1
GO:0007212	GO:0007212	dopamine receptor signaling pathway	1/385	39/23328	0.477719884	0.54239	0.32592	Rgs4	1
GO:0009651	GO:0009651	response to salt stress	1/385	39/23328	0.477719884	0.54239	0.32592	Aqp1	1
GO:0021983	GO:0021983	pituitary gland development	1/385	39/23328	0.477719884	0.54239	0.32592	Bmp4	1
GO:0031279	GO:0031279	regulation of cyclase activity	1/385	39/23328	0.477719884	0.54239	0.32592	Nos3	1
GO:0032735	GO:0032735	positive regulation of interleukin-12 production	1/385	39/23328	0.477719884	0.54239	0.32592	Cd36	1
GO:0032885	GO:0032885	regulation of polysaccharide biosynthetic process	1/385	39/23328	0.477719884	0.54239	0.32592	Pdgfb	1
GO:0042311	GO:0042311	vasodilation	1/385	39/23328	0.477719884	0.54239	0.32592	Apoe	1
GO:0042398	GO:0042398	cellular modified amino acid biosynthetic process	1/385	39/23328	0.477719884	0.54239	0.32592	Ckb	1
GO:0042554	GO:0042554	superoxide anion generation	1/385	39/23328	0.477719884	0.54239	0.32592	Edn1	1
GO:0050931	GO:0050931	pigment cell differentiation	1/385	39/23328	0.477719884	0.54239	0.32592	Kitl	1
GO:0090224	GO:0090224	regulation of spindle organization	1/385	39/23328	0.477719884	0.54239	0.32592	Hspa1a	1
GO:1900026	GO:1900026	positive regulation of substrate adhesion-dependent ce	1/385	39/23328	0.477719884	0.54239	0.32592	Dab2	1
GO:0071482	GO:0071482	cellular response to light stimulus	2/385	98/23328	0.482719834	0.54762	0.32906	Aqp1/Cdk	2
GO:0072080	GO:0072080	nephron tubule development	2/385	98/23328	0.482719834	0.54762	0.32906	Aqp1/Bmp	2
GO:0099565	GO:0099565	chemical synaptic transmission, postsynaptic	2/385	98/23328	0.482719834	0.54762	0.32906	Reln/Rgs4	2
GO:0046700	GO:0046700	heterocycle catabolic process	7/385	397/23328	0.483094439	0.5479	0.32923	Vim/Btg2/	7
GO:0001504	GO:0001504	neurotransmitter uptake	1/385	40/23328	0.486353911	0.54877	0.32975	Itgb1	1
GO:0002323	GO:0002323	natural killer cell activation involved in immune respon	1/385	40/23328	0.486353911	0.54877	0.32975	Pglyrp1	1
GO:0003009	GO:0003009	skeletal muscle contraction	1/385	40/23328	0.486353911	0.54877	0.32975	Tnni3	1
GO:0006984	GO:0006984	ER-nucleus signaling pathway	1/385	40/23328	0.486353911	0.54877	0.32975	Hspa5	1
GO:0009409	GO:0009409	response to cold	1/385	40/23328	0.486353911	0.54877	0.32975	Metrnl	1
GO:0014046	GO:0014046	dopamine secretion	1/385	40/23328	0.486353911	0.54877	0.32975	Cxcl12	1
GO:0014059	GO:0014059	regulation of dopamine secretion	1/385	40/23328	0.486353911	0.54877	0.32975	Cxcl12	1
GO:0031076	GO:0031076	embryonic camera-type eye development	1/385	40/23328	0.486353911	0.54877	0.32975	Prox1	1
GO:0032392	GO:0032392	DNA geometric change	1/385	40/23328	0.486353911	0.54877	0.32975	Anxa1	1
GO:0032633	GO:0032633	interleukin-4 production	1/385	40/23328	0.486353911	0.54877	0.32975	Il33	1
GO:0035307	GO:0035307	positive regulation of protein dephosphorylation	1/385	40/23328	0.486353911	0.54877	0.32975	Cdh5	1
GO:0038179	GO:0038179	neurotrophin signaling pathway	1/385	40/23328	0.486353911	0.54877	0.32975	Ptprf	1
GO:0043368	GO:0043368	positive T cell selection	1/385	40/23328	0.486353911	0.54877	0.32975	Cd74	1
GO:0045981	GO:0045981	positive regulation of nucleotide metabolic process	1/385	40/23328	0.486353911	0.54877	0.32975	Nos3	1
GO:0048741	GO:0048741	skeletal muscle fiber development	1/385	40/23328	0.486353911	0.54877	0.32975	Pmp22	1
GO:0060337	GO:0060337	type I interferon signaling pathway	1/385	40/23328	0.486353911	0.54877	0.32975	Irf7	1
GO:0070884	GO:0070884	regulation of calcineurin-NFAT signaling cascade	1/385	40/23328	0.486353911	0.54877	0.32975	Prnp	1
GO:0071357	GO:0071357	cellular response to type I interferon	1/385	40/23328	0.486353911	0.54877	0.32975	Irf7	1
GO:1900544	GO:1900544	positive regulation of purine nucleotide metabolic proc	1/385	40/23328	0.486353911	0.54877	0.32975	Nos3	1
GO:0006304	GO:0006304	DNA modification	2/385	100/23328	0.493337296	0.5564	0.33434	H19/Apob	2
GO:0006164	GO:0006164	purine nucleotide biosynthetic process	3/385	160/23328	0.493579361	0.5564	0.33434	Aldoa/Nos	3
GO:0002483	GO:0002483	antigen processing and presentation of endogenous pe	1/385	41/23328	0.494845569	0.5564	0.33434	B2m	1
GO:0019885	GO:0019885	antigen processing and presentation of endogenous pe	1/385	41/23328	0.494845569	0.5564	0.33434	B2m	1
GO:0030225	GO:0030225	macrophage differentiation	1/385	41/23328	0.494845569	0.5564	0.33434	Bmp4	1
GO:0045022	GO:0045022	early endosome to late endosome transport	1/385	41/23328	0.494845569	0.5564	0.33434	Dab2	1
GO:0048730	GO:0048730	epidermis morphogenesis	1/385	41/23328	0.494845569	0.5564	0.33434	Igfbp5	1
GO:0051693	GO:0051693	actin filament capping	1/385	41/23328	0.494845569	0.5564	0.33434	Tpm1	1
GO:0060603	GO:0060603	mammary gland duct morphogenesis	1/385	41/23328	0.494845569	0.5564	0.33434	Pthlh	1
GO:0072599	GO:0072599	establishment of protein localization to endoplasmic re	1/385	41/23328	0.494845569	0.5564	0.33434	Hspa5	1
GO:0106056	GO:0106056	regulation of calcineurin-mediated signaling	1/385	41/23328	0.494845569	0.5564	0.33434	Prnp	1

GO:1900744	GO:1900744	regulation of p38MAPK cascade	1/385	41/23328	0.494845569	0.5564	0.33434	Dusp1	1
GO:1905332	GO:1905332	positive regulation of morphogenesis of an epithelium	1/385	41/23328	0.494845569	0.5564	0.33434	Bmp4	1
GO:0002768	GO:0002768	immune response-regulating cell surface receptor sign	6/385	342/2332	0.497332322	0.55905	0.33593	Thy1/Prnp	6
GO:0007601	GO:0007601	visual perception	3/385	161/2332	0.49771888	0.55933	0.3361	Cfh/Col1a	3
GO:0006865	GO:0006865	amino acid transport	3/385	162/2332	0.501841354	0.56381	0.33879	Rgs4/Itgb	3
GO:0002823	GO:0002823	negative regulation of adaptive immune response base	1/385	42/23328	0.503197201	0.56428	0.33907	Il33	1
GO:0006687	GO:0006687	glycosphingolipid metabolic process	1/385	42/23328	0.503197201	0.56428	0.33907	Gm2a	1
GO:0010092	GO:0010092	specification of animal organ identity	1/385	42/23328	0.503197201	0.56428	0.33907	Bmp4	1
GO:0046633	GO:0046633	alpha-beta T cell proliferation	1/385	42/23328	0.503197201	0.56428	0.33907	Cd24a	1
GO:0046825	GO:0046825	regulation of protein export from nucleus	1/385	42/23328	0.503197201	0.56428	0.33907	Gas6	1
GO:0051154	GO:0051154	negative regulation of striated muscle cell differentiatio	1/385	42/23328	0.503197201	0.56428	0.33907	Rgs4	1
GO:0051339	GO:0051339	regulation of lyase activity	1/385	42/23328	0.503197201	0.56428	0.33907	Nos3	1
GO:0035418	GO:0035418	protein localization to synapse	2/385	102/2332	0.503816048	0.56467	0.3393	Reln/Hspb	2
GO:0045582	GO:0045582	positive regulation of T cell differentiation	2/385	102/2332	0.503816048	0.56467	0.3393	Cd74/Anx	2
GO:0002764	GO:0002764	immune response-regulating signaling pathway	6/385	345/2332	0.505798832	0.56659	0.34046	Thy1/Prnp	6
GO:0019693	GO:0019693	ribose phosphate metabolic process	6/385	345/2332	0.505798832	0.56659	0.34046	Nupr1/Ald	6
GO:0030004	GO:0030004	cellular monovalent inorganic cation homeostasis	2/385	103/2332	0.509002674	0.56987	0.34243	Sgk1/Atp1	2
GO:0048477	GO:0048477	oogenesis	2/385	103/2332	0.509002674	0.56987	0.34243	Pde3a/Ptr	2
GO:0046887	GO:0046887	positive regulation of hormone secretion	3/385	164/2332	0.510034166	0.57058	0.34286	Cd38/Dab	3
GO:0002753	GO:0002753	cytoplasmic pattern recognition receptor signaling path	1/385	43/23328	0.51141111	0.57058	0.34286	Nfkbia	1
GO:0014003	GO:0014003	oligodendrocyte development	1/385	43/23328	0.51141111	0.57058	0.34286	Clu	1
GO:0032620	GO:0032620	interleukin-17 production	1/385	43/23328	0.51141111	0.57058	0.34286	Prnp	1
GO:0045197	GO:0045197	establishment or maintenance of epithelial cell apical/b	1/385	43/23328	0.51141111	0.57058	0.34286	Tcf15	1
GO:0045880	GO:0045880	positive regulation of smoothened signaling pathway	1/385	43/23328	0.51141111	0.57058	0.34286	Sfrp1	1
GO:0046636	GO:0046636	negative regulation of alpha-beta T cell activation	1/385	43/23328	0.51141111	0.57058	0.34286	Anxa1	1
GO:0046854	GO:0046854	phosphatidylinositol phosphorylation	1/385	43/23328	0.51141111	0.57058	0.34286	Socs3	1
GO:0055026	GO:0055026	negative regulation of cardiac muscle tissue developm	1/385	43/23328	0.51141111	0.57058	0.34286	Rgs4	1
GO:0070232	GO:0070232	regulation of T cell apoptotic process	1/385	43/23328	0.51141111	0.57058	0.34286	Bmp4	1
GO:0098927	GO:0098927	vesicle-mediated transport between endosomal compa	1/385	43/23328	0.51141111	0.57058	0.34286	Dab2	1
GO:1901381	GO:1901381	positive regulation of potassium ion transmembrane tr	1/385	43/23328	0.51141111	0.57058	0.34286	Atp1b1	1
GO:2001258	GO:2001258	negative regulation of cation channel activity	1/385	43/23328	0.51141111	0.57058	0.34286	Cav1	1
GO:0050953	GO:0050953	sensory perception of light stimulus	3/385	165/2332	0.514104024	0.57257	0.34406	Cfh/Col1a	3
GO:0072522	GO:0072522	purine-containing compound biosynthetic process	3/385	165/2332	0.514104024	0.57257	0.34406	Aldoa/Nos	3
GO:0001657	GO:0001657	ureteric bud development	2/385	104/2332	0.514153779	0.57257	0.34406	Sfrp1/Bmp	2
GO:0043488	GO:0043488	regulation of mRNA stability	2/385	104/2332	0.514153779	0.57257	0.34406	Vim/Zfp36	2
GO:0061326	GO:0061326	renal tubule development	2/385	104/2332	0.514153779	0.57257	0.34406	Aqp1/Bmp	2
GO:0072163	GO:0072163	mesonephric epithelium development	2/385	104/2332	0.514153779	0.57257	0.34406	Sfrp1/Bmp	2
GO:0072164	GO:0072164	mesonephric tubule development	2/385	104/2332	0.514153779	0.57257	0.34406	Sfrp1/Bmp	2
GO:0010256	GO:0010256	endomembrane system organization	7/385	410/2332	0.516967419	0.57555	0.34585	Clu/Ndrg1	7
GO:0006958	GO:0006958	complement activation, classical pathway	3/385	166/2332	0.518155877	0.57652	0.34643	Serping1/	3
GO:0000077	GO:0000077	DNA damage checkpoint	2/385	105/2332	0.519269119	0.57652	0.34643	Cdkn1a/Ie	2
GO:0000959	GO:0000959	mitochondrial RNA metabolic process	1/385	44/23328	0.519489561	0.57652	0.34643	Chchd10	1
GO:0006024	GO:0006024	glycosaminoglycan biosynthetic process	1/385	44/23328	0.519489561	0.57652	0.34643	Pdgfb	1
GO:0006890	GO:0006890	retrograde vesicle-mediated transport, Golgi to endopla	1/385	44/23328	0.519489561	0.57652	0.34643	Plpp3	1
GO:0008631	GO:0008631	intrinsic apoptotic signaling pathway in response to oxi	1/385	44/23328	0.519489561	0.57652	0.34643	Hspb1	1
GO:0014904	GO:0014904	myotube cell development	1/385	44/23328	0.519489561	0.57652	0.34643	Pmp22	1
GO:0016925	GO:0016925	protein sumoylation	1/385	44/23328	0.519489561	0.57652	0.34643	Egr1	1
GO:0019883	GO:0019883	antigen processing and presentation of endogenous ar	1/385	44/23328	0.519489561	0.57652	0.34643	B2m	1
GO:0046621	GO:0046621	negative regulation of organ growth	1/385	44/23328	0.519489561	0.57652	0.34643	Rgs4	1
GO:1901998	GO:1901998	toxin transport	1/385	44/23328	0.519489561	0.57652	0.34643	Hspa5	1
GO:2000144	GO:2000144	positive regulation of DNA-templated transcription, initi	1/385	44/23328	0.519489561	0.57652	0.34643	Jun	1
GO:0035710	GO:0035710	CD4-positive, alpha-beta T cell activation	2/385	106/2332	0.524348471	0.58161	0.34948	Rsad2/An	2
GO:0043473	GO:0043473	pigmentation	2/385	106/2332	0.524348471	0.58161	0.34948	Sparc/Kitl	2
GO:0046425	GO:0046425	regulation of receptor signaling pathway via JAK-STAT	3/385	168/2332	0.526204685	0.58351	0.35063	Socs3/Pe	3
GO:0009166	GO:0009166	nucleotide catabolic process	1/385	45/23328	0.527434781	0.58364	0.3507	Pde2a	1
GO:0010824	GO:0010824	regulation of centrosome duplication	1/385	45/23328	0.527434781	0.58364	0.3507	Plk2	1
GO:0031128	GO:0031128	developmental induction	1/385	45/23328	0.527434781	0.58364	0.3507	Bmp4	1
GO:0032527	GO:0032527	protein exit from endoplasmic reticulum	1/385	45/23328	0.527434781	0.58364	0.3507	Hsp90b1	1
GO:0034340	GO:0034340	response to type I interferon	1/385	45/23328	0.527434781	0.58364	0.3507	Irf7	1
GO:0035136	GO:0035136	forelimb morphogenesis	1/385	45/23328	0.527434781	0.58364	0.3507	Hoxd9	1
GO:0042149	GO:0042149	cellular response to glucose starvation	1/385	45/23328	0.527434781	0.58364	0.3507	Hspa5	1
GO:0051293	GO:0051293	establishment of spindle localization	1/385	45/23328	0.527434781	0.58364	0.3507	Itgb1	1
GO:0044262	GO:0044262	cellular carbohydrate metabolic process	5/385	292/2332	0.529375491	0.58518	0.35163	Nupr1/Igf	5
GO:0001823	GO:0001823	mesonephros development	2/385	107/2332	0.529391626	0.58518	0.35163	Sfrp1/Bmp	2
GO:0030518	GO:0030518	intracellular steroid hormone receptor signaling pathwa	2/385	107/2332	0.529391626	0.58518	0.35163	Sfrp1/Dab	2
GO:0140053	GO:0140053	mitochondrial gene expression	2/385	107/2332	0.529391626	0.58518	0.35163	Cfh/Chch	2
GO:0006417	GO:0006417	regulation of translation	6/385	355/2332	0.533633271	0.58972	0.35436	Vim/Btg2/	6
GO:0000956	GO:0000956	nuclear-transcribed mRNA catabolic process	2/385	108/2332	0.534398394	0.59025	0.35468	Btg2/Zfp3	2
GO:0021987	GO:0021987	cerebral cortex development	2/385	108/2332	0.534398394	0.59025	0.35468	Reln/Col3	2
GO:0006904	GO:0006904	vesicle docking involved in exocytosis	1/385	46/23328	0.535248964	0.59041	0.35477	Ncam1	1
GO:0019228	GO:0019228	neuronal action potential	1/385	46/23328	0.535248964	0.59041	0.35477	Pmp22	1
GO:0030835	GO:0030835	negative regulation of actin filament depolymerization	1/385	46/23328	0.535248964	0.59041	0.35477	Tpm1	1
GO:0032881	GO:0032881	regulation of polysaccharide metabolic process	1/385	46/23328	0.535248964	0.59041	0.35477	Pdgfb	1
GO:0090317	GO:0090317	negative regulation of intracellular protein transport	1/385	46/23328	0.535248964	0.59041	0.35477	Cd36	1
GO:0090150	GO:0090150	establishment of protein localization to membrane	4/385	233/2332	0.538030599	0.59328	0.3565	Prnp/Rtp4	4
GO:0019827	GO:0019827	stem cell population maintenance	3/385	171/2332	0.538137552	0.59328	0.3565	Sfrp1/Ptn/	3
GO:0009141	GO:0009141	nucleoside triphosphate metabolic process	2/385	109/2332	0.539368598	0.59448	0.35722	Aldoa/Cm	2
GO:0002820	GO:0002820	negative regulation of adaptive immune response	1/385	47/23328	0.542934263	0.59731	0.35892	Il33	1
GO:0007628	GO:0007628	adult walking behavior	1/385	47/23328	0.542934263	0.59731	0.35892	Pmp22	1
GO:0030517	GO:0030517	negative regulation of axon extension	1/385	47/23328	0.542934263	0.59731	0.35892	Sema3g	1
GO:0033013	GO:0033013	tetrapyrrole metabolic process	1/385	47/23328	0.542934263	0.59731	0.35892	Cyp1a1	1
GO:0043029	GO:0043029	T cell homeostasis	1/385	47/23328	0.542934263	0.59731	0.35892	Cd24a	1
GO:0048246	GO:0048246	macrophage chemotaxis	1/385	47/23328	0.542934263	0.59731	0.35892	Thbs1	1
GO:0120163	GO:0120163	negative regulation of cold-induced thermogenesis	1/385	47/23328	0.542934263	0.59731	0.35892	Id1	1
GO:0071230	GO:0071230	cellular response to amino acid stimulus	3/385	173/2332	0.545996983	0.60037	0.36076	Col5a2/Co	3
GO:1904892	GO:1904892	regulation of receptor signaling pathway via STAT	3/385	173/2332	0.545996983	0.60037	0.36076	Socs3/Pe	3
GO:0009416	GO:0009416	response to light stimulus	5/385	298/2332	0.547430008	0.60178	0.36161	Nts/Aqp1/	5
GO:0006643	GO:0006643	membrane lipid metabolic process	3/385	174/2332	0.549897416	0.60404	0.36296	Plpp3/Gm	3
GO:0001580	GO:0001580	detection of chemical stimulus involved in sensory per	1/385	48/23328	0.550492801	0.60404	0.36296	Rtp4	1
GO:0007339	GO:0007339	binding of sperm to zona pellucida	1/385	48/23328	0.550492801	0.60404	0.36296	Aldoa	1
GO:0022602	GO:0022602	ovulation cycle process	1/385	48/23328	0.550492801	0.60404	0.36296	Nos3	1
GO:0046596	GO:0046596	regulation of viral entry into host cell	1/385	48/23328	0.550492801	0.60404	0.36296	Cd74	1
GO:0051972	GO:0051972	regulation of telomerase activity	1/385	48/23328	0.550492801	0.60404	0.36296	Parm1	1
GO:1903580	GO:1903580	positive regulation of ATP metabolic process	1/385	48/23328	0.550492801	0.60404	0.36296	Nupr1	1
GO:0098727	GO:0098727	maintenance of cell number	3/385	175/2332	0.553778105	0.60749	0.36503	Sfrp1/Ptn/	3
GO:0038127	GO:0038127	ERBB signaling pathway	2/385	112/2332	0.55405831	0.60763	0.36512	Lgmn/Ptp	2
GO:0006368	GO:0006368	transcription elongation from RNA polymerase II promo	1/385	49/23328	0.557926661	0.6106	0.3669	Dab2	1
GO:0014888	GO:0014888	striated muscle adaptation	1/385	49/23328	0.557926661	0.6106	0.3669	Igfbp5	1
GO:0032784	GO:0032784	regulation of DNA-templated transcription, elongation	1/385	49/23328	0.557926661	0.6106	0.3669	Dab2	1
GO:0033173	GO:0033173	calcineurin-NFAT signaling cascade	1/385	49/23328	0.557926661	0.6106	0.3669	Prnp	1
GO:0035196	GO:0035196	production of miRNAs involved in gene silencing by mi	1/385	49/23328	0.557926661	0.6106	0.3669	Bmp4	1
GO:0046638	GO:0046638	positive regulation of alpha-beta T cell differentiation	1/385	49/23328	0.557926661	0.6106	0.3669	Anxa1	1
GO:0048255	GO:0048255	mRNA stabilization	1/385	49/23328	0.557926661	0.6106	0.3669	Zfp36	1
GO:0090659	GO:0090659	walking behavior	1/385	49/23328	0.557926661	0.6106	0.3669	Pmp22	1
GO:0001942	GO:0001942	hair follicle development	2/385	113/2332	0.558880815	0.61132	0.36734	Igfbp5/Pd	2
GO:0031570	GO:0031570	DNA integrity checkpoint	2/385	113/2332	0.558880815	0.61132	0.36734	Cdkn1a/Ie	2
GO:0060078	GO:0060078	regulation of postsynaptic membrane potential	2/385	114/2332	0.563666106	0.61639	0.37039	Reln/Rgs4	2
GO:0002762	GO:0002762	negative regulation of myeloid leukocyte differentiation	1/385	50/23328	0.565237896	0.61682	0.37065	Sfrp1	1
GO:0007140	GO:0007140	male meiotic nuclear division	1/385	50/23328	0.565237896	0.61682	0.37065	Cyp26b1	1
GO:0021545	GO:0021545	cranial nerve development	1/385	50/23328	0.565237896	0.61682	0.37065	Tbx1	1
GO:0030166	GO:0030166	proteoglycan biosynthetic process	1/385	50/23328	0.565237896	0.61682	0.37065	Cytl1	1
GO:0034332	GO:0034332	adherens junction organization	1/385	50/23328	0.565237896	0.61682	0.37065	Cdh5	1
GO:0043370	GO:0043370	regulation of CD4-positive, alpha-beta T cell differentia	1/385	50/23328	0.565237896	0.61682	0.37065	Anxa1	1
GO:0051653	GO:0051653	spindle localization	1/385	50/23328	0.565237896	0.61682	0.37065	Itgb1	1
GO:1903573	GO:1903573	negative regulation of response to endoplasmic reticul	1/385	50/23328	0.565237896	0.61682	0.37065	Clu	1
GO:0019751	GO:0019751	polyol metabolic process	2/385	115/2332	0.568414096	0.61997	0.37253	Plpp3/Ace	2
GO:0031398	GO:0031398	positive regulation of protein ubiquitination	2/385	115/2332	0.568414096	0.61997	0.37253	Cav1/Hsp	2
GO:0001916	GO:0001916	positive regulation of T cell mediated cytotoxicity	1/385	51/23328	0.572428523	0.62235	0.37396	B2m	1
GO:0002474	GO:0002474	antigen processing and presentation of peptide antiger	1/385	51/23328	0.572428523	0.62235	0.37396	B2m	1
GO:0008089	GO:0008089	anterograde axonal transport	1/385	51/23328	0.572428523	0.62235	0.37396	Hspb1	1
GO:0031050	GO:0031050	dsRNA processing	1/385	51/23328	0.572428523	0.62235	0.37396	Bmp4	1
GO:0045010	GO:0045010	actin nucleation	1/385	51/23328	0.572428523	0.62235	0.37396	Gmfg	1
GO:0050913	GO:0050913	sensory perception of bitter taste	1/385	51/23328	0.572428523	0.62235	0.37396	Rtp4	1
GO:0060043	GO:0060043	regulation of cardiac muscle cell proliferation	1/385	51/23328	0.572428523	0.62235	0.37396	Ncam1	1
GO:0060428	GO:0060428	lung epithelium development	1/385	51/23328	0.572428523	0.62235	0.37396	Bmp4	1
GO:0070918	GO:0070918	production of small RNA involved in gene silencing by	1/385	51/23328	0.572428523	0.62235	0.37396	Bmp4	1
GO:0072527	GO:0072527	pyrimidine-containing compound metabolic process	1/385	51/23328	0.572428523	0.62235	0.37396	Cmpk2	1
GO:0099054	GO:0099054	presynapse assembly	1/385	51/23328	0.572428523	0.62235	0.37396	Pcdh17	1
GO:2000142	GO:2000142	regulation of DNA-templated transcription, initiation	1/385	51/23328	0.572428523	0.62235	0.37396	Jun	1

GO:0007052	GO:0007052	mitotic spindle organization	2/385	116/23328	0.573124708	0.62235	0.37396	Plk2/Hspa	2
GO:0022404	GO:0022404	molting cycle process	2/385	116/23328	0.573124708	0.62235	0.37396	Igfbp5/Pd	2
GO:0022405	GO:0022405	hair cycle process	2/385	116/23328	0.573124708	0.62235	0.37396	Igfbp5/Pd	2
GO:0043487	GO:0043487	regulation of RNA stability	2/385	116/23328	0.573124708	0.62235	0.37396	Vim/Zfp36	2
GO:0098773	GO:0098773	skin epidermis development	2/385	116/23328	0.573124708	0.62235	0.37396	Igfbp5/Pd	2
GO:0002639	GO:0002639	positive regulation of immunoglobulin production	1/385	52/23328	0.579500526	0.62797	0.37734	Il33	1
GO:0002888	GO:0002888	positive regulation of myeloid leukocyte mediated imm	1/385	52/23328	0.579500526	0.62797	0.37734	C3	1
GO:0010828	GO:0010828	positive regulation of glucose transmembrane transpor	1/385	52/23328	0.579500526	0.62797	0.37734	C3	1
GO:0042307	GO:0042307	positive regulation of protein import into nucleus	1/385	52/23328	0.579500526	0.62797	0.37734	Tek	1
GO:0042491	GO:0042491	inner ear auditory receptor cell differentiation	1/385	52/23328	0.579500526	0.62797	0.37734	Clic5	1
GO:0050912	GO:0050912	detection of chemical stimulus involved in sensory per	1/385	52/23328	0.579500526	0.62797	0.37734	Rtp4	1
GO:0065002	GO:0065002	intracellular protein transmembrane transport	1/385	52/23328	0.579500526	0.62797	0.37734	Hspa5	1
GO:1903202	GO:1903202	negative regulation of oxidative stress-induced cell de	1/385	52/23328	0.579500526	0.62797	0.37734	Hspb1	1
GO:0046632	GO:0046632	alpha-beta T cell differentiation	2/385	118/23328	0.582433564	0.63098	0.37915	Rsad2/An	2
GO:0001662	GO:0001662	behavioral fear response	1/385	53/23328	0.586455858	0.63354	0.38069	Apoe	1
GO:0002714	GO:0002714	positive regulation of B cell mediated immunity	1/385	53/23328	0.586455858	0.63354	0.38069	C3	1
GO:0002891	GO:0002891	positive regulation of immunoglobulin mediated immur	1/385	53/23328	0.586455858	0.63354	0.38069	C3	1
GO:0009395	GO:0009395	phospholipid catabolic process	1/385	53/23328	0.586455858	0.63354	0.38069	Scarb1	1
GO:0021795	GO:0021795	cerebral cortex cell migration	1/385	53/23328	0.586455858	0.63354	0.38069	Reln	1
GO:0043666	GO:0043666	regulation of phosphoprotein phosphatase activity	1/385	53/23328	0.586455858	0.63354	0.38069	Hsp90b1	1
GO:0044058	GO:0044058	regulation of digestive system process	1/385	53/23328	0.586455858	0.63354	0.38069	Aqp1	1
GO:0046676	GO:0046676	negative regulation of insulin secretion	1/385	53/23328	0.586455858	0.63354	0.38069	Sfrp1	1
GO:0048599	GO:0048599	oocyte development	1/385	53/23328	0.586455858	0.63354	0.38069	Pde3a	1
GO:0097720	GO:0097720	calcineurin-mediated signaling	1/385	53/23328	0.586455858	0.63354	0.38069	Prnp	1
GO:1900408	GO:1900408	negative regulation of cellular response to oxidative st	1/385	53/23328	0.586455858	0.63354	0.38069	Hspb1	1
GO:0002209	GO:0002209	behavioral defense response	1/385	54/23328	0.593296437	0.63993	0.38453	Apoe	1
GO:0010823	GO:0010823	negative regulation of mitochondrion organization	1/385	54/23328	0.593296437	0.63993	0.38453	Ier3	1
GO:0046834	GO:0046834	lipid phosphorylation	1/385	54/23328	0.593296437	0.63993	0.38453	Socs3	1
GO:0048066	GO:0048066	developmental pigmentation	1/385	54/23328	0.593296437	0.63993	0.38453	Kitl	1
GO:0051289	GO:0051289	protein homotetramerization	1/385	54/23328	0.593296437	0.63993	0.38453	B2m	1
GO:0071806	GO:0071806	protein transmembrane transport	1/385	54/23328	0.593296437	0.63993	0.38453	Hspa5	1
GO:0043200	GO:0043200	response to amino acid	3/385	187/23328	0.598754489	0.64566	0.38797	Col5a2/Co	3
GO:0002673	GO:0002673	regulation of acute inflammatory response	1/385	55/23328	0.600024154	0.64603	0.38819	C3	1
GO:0006754	GO:0006754	ATP biosynthetic process	1/385	55/23328	0.600024154	0.64603	0.38819	Aldoa	1
GO:0035176	GO:0035176	social behavior	1/385	55/23328	0.600024154	0.64603	0.38819	Tbx1	1
GO:0035306	GO:0035306	positive regulation of dephosphorylation	1/385	55/23328	0.600024154	0.64603	0.38819	Cdh5	1
GO:0042531	GO:0042531	positive regulation of tyrosine phosphorylation of STAT	1/385	55/23328	0.600024154	0.64603	0.38819	Pecam1	1
GO:0061001	GO:0061001	regulation of dendritic spine morphogenesis	1/385	55/23328	0.600024154	0.64603	0.38819	Reln	1
GO:0007098	GO:0007098	centrosome cycle	2/385	122/23328	0.600600797	0.64631	0.38837	Plk2/Pard	2
GO:1903828	GO:1903828	negative regulation of cellular protein localization	2/385	122/23328	0.600600797	0.64631	0.38837	Cd36/Dab	2
GO:1903829	GO:1903829	positive regulation of cellular protein localization	5/385	317/23328	0.602289462	0.64796	0.38936	Prnp/Ptn/	5
GO:0001914	GO:0001914	regulation of T cell mediated cytotoxicity	1/385	56/23328	0.606640864	0.65164	0.39157	B2m	1
GO:0006023	GO:0006023	aminoglycan biosynthetic process	1/385	56/23328	0.606640864	0.65164	0.39157	Pdgfb	1
GO:0015872	GO:0015872	dopamine transport	1/385	56/23328	0.606640864	0.65164	0.39157	Cxcl12	1
GO:0048538	GO:0048538	thymus development	1/385	56/23328	0.606640864	0.65164	0.39157	Tbx1	1
GO:1902883	GO:1902883	negative regulation of response to oxidative stress	1/385	56/23328	0.606640864	0.65164	0.39157	Hspb1	1
GO:1903533	GO:1903533	regulation of protein targeting	1/385	56/23328	0.606640864	0.65164	0.39157	Prnp	1
GO:0007259	GO:0007259	receptor signaling pathway via JAK-STAT	3/385	191/23328	0.613072562	0.65694	0.39475	Socs3/Pe	3
GO:0006584	GO:0006584	catecholamine metabolic process	1/385	57/23328	0.613148395	0.65694	0.39475	Ly6e	1
GO:0009712	GO:0009712	catechol-containing compound metabolic process	1/385	57/23328	0.613148395	0.65694	0.39475	Ly6e	1
GO:0009994	GO:0009994	oocyte differentiation	1/385	57/23328	0.613148395	0.65694	0.39475	Pde3a	1
GO:0042775	GO:0042775	mitochondrial ATP synthesis coupled electron transpor	1/385	57/23328	0.613148395	0.65694	0.39475	Cox4i2	1
GO:0043489	GO:0043489	RNA stabilization	1/385	57/23328	0.613148395	0.65694	0.39475	Zfp36	1
GO:0045058	GO:0045058	T cell selection	1/385	57/23328	0.613148395	0.65694	0.39475	Cd74	1
GO:1901292	GO:1901292	nucleoside phosphate catabolic process	1/385	57/23328	0.613148395	0.65694	0.39475	Pde2a	1
GO:1901607	GO:1901607	alpha-amino acid biosynthetic process	1/385	57/23328	0.613148395	0.65694	0.39475	Ass1	1
GO:1902373	GO:1902373	negative regulation of mRNA catabolic process	1/385	57/23328	0.613148395	0.65694	0.39475	Zfp36	1
GO:0051053	GO:0051053	negative regulation of DNA metabolic process	2/385	126/23328	0.618167088	0.66177	0.39765	Dusp1/Cd	2
GO:0006493	GO:0006493	protein O-linked glycosylation	1/385	58/23328	0.619548544	0.66177	0.39765	Gxylt2	1
GO:0032653	GO:0032653	regulation of interleukin-10 production	1/385	58/23328	0.619548544	0.66177	0.39765	Cd34	1
GO:0035019	GO:0035019	somatic stem cell population maintenance	1/385	58/23328	0.619548544	0.66177	0.39765	Sfrp1	1
GO:0035315	GO:0035315	hair cell differentiation	1/385	58/23328	0.619548544	0.66177	0.39765	Clic5	1
GO:0042267	GO:0042267	natural killer cell mediated cytotoxicity	1/385	58/23328	0.619548544	0.66177	0.39765	Prdx1	1
GO:0042596	GO:0042596	fear response	1/385	58/23328	0.619548544	0.66177	0.39765	Apoe	1
GO:0051445	GO:0051445	regulation of meiotic cell cycle	1/385	58/23328	0.619548544	0.66177	0.39765	Pde3a	1
GO:0051703	GO:0051703	intraspecies interaction between organisms	1/385	58/23328	0.619548544	0.66177	0.39765	Tbx1	1
GO:0099172	GO:0099172	presynapse organization	1/385	58/23328	0.619548544	0.66177	0.39765	Pcdh17	1
GO:1903902	GO:1903902	positive regulation of viral life cycle	1/385	58/23328	0.619548544	0.66177	0.39765	Cd74	1
GO:1904591	GO:1904591	positive regulation of protein import	1/385	58/23328	0.619548544	0.66177	0.39765	Tek	1
GO:0001836	GO:0001836	release of cytochrome c from mitochondria	1/385	59/23328	0.625843079	0.66815	0.40149	Jun	1
GO:0002011	GO:0002011	morphogenesis of an epithelial sheet	1/385	59/23328	0.625843079	0.66815	0.40149	Pdpn	1
GO:0010906	GO:0010906	regulation of glucose metabolic process	2/385	128/23328	0.626725369	0.66875	0.40185	Igfbp3/Igf	2
GO:0045727	GO:0045727	positive regulation of translation	2/385	128/23328	0.626725369	0.66875	0.40185	Vim/Thbs	2
GO:0044270	GO:0044270	cellular nitrogen compound catabolic process	6/385	391/23328	0.627663749	0.66958	0.40235	Vim/Btg2/	6
GO:0097696	GO:0097696	receptor signaling pathway via STAT	3/385	196/23328	0.63048274	0.67242	0.40405	Socs3/Pe	3
GO:0046434	GO:0046434	organophosphate catabolic process	2/385	129/23328	0.630948468	0.67274	0.40425	Scarb1/Pc	2
GO:0007595	GO:0007595	lactation	1/385	60/23328	0.632033736	0.67304	0.40443	Cav1	1
GO:0021517	GO:0021517	ventral spinal cord development	1/385	60/23328	0.632033736	0.67304	0.40443	Reln	1
GO:0046605	GO:0046605	regulation of centrosome cycle	1/385	60/23328	0.632033736	0.67304	0.40443	Plk2	1
GO:0050907	GO:0050907	detection of chemical stimulus involved in sensory per	1/385	60/23328	0.632033736	0.67304	0.40443	Rtp4	1
GO:0070231	GO:0070231	T cell apoptotic process	1/385	60/23328	0.632033736	0.67304	0.40443	Bmp4	1
GO:0007631	GO:0007631	feeding behavior	2/385	130/23328	0.635134294	0.676	0.4062	Cd36/Tcf1	2
GO:0090305	GO:0090305	nucleic acid phosphodiester bond hydrolysis	2/385	130/23328	0.635134294	0.676	0.4062	Igfbp3/Zfp	2
GO:0002228	GO:0002228	natural killer cell mediated immunity	1/385	61/23328	0.638122228	0.67849	0.4077	Prdx1	1
GO:0008652	GO:0008652	cellular amino acid biosynthetic process	1/385	61/23328	0.638122228	0.67849	0.4077	Ass1	1
GO:0042773	GO:0042773	ATP synthesis coupled electron transport	1/385	61/23328	0.638122228	0.67849	0.4077	Cox4i2	1
GO:0045824	GO:0045824	negative regulation of innate immune response	1/385	61/23328	0.638122228	0.67849	0.4077	Serping1	1
GO:0009582	GO:0009582	detection of abiotic stimulus	2/385	132/23328	0.643394445	0.68295	0.41038	Cxcl12/Se	2
GO:0010469	GO:0010469	regulation of signaling receptor activity	2/385	132/23328	0.643394445	0.68295	0.41038	Reln/Itgb1	2
GO:0031023	GO:0031023	microtubule organizing center organization	2/385	132/23328	0.643394445	0.68295	0.41038	Plk2/Pard	2
GO:0006839	GO:0006839	mitochondrial transport	3/385	200/23328	0.644016725	0.68295	0.41038	Slc25a4/C	3
GO:0006900	GO:0006900	vesicle budding from membrane	1/385	62/23328	0.644110234	0.68295	0.41038	Anxa2	1
GO:0030865	GO:0030865	cortical cytoskeleton organization	1/385	62/23328	0.644110234	0.68295	0.41038	Actn1	1
GO:0032387	GO:0032387	negative regulation of intracellular transport	1/385	62/23328	0.644110234	0.68295	0.41038	Cd36	1
GO:0032613	GO:0032613	interleukin-10 production	1/385	62/23328	0.644110234	0.68295	0.41038	Cd34	1
GO:0048512	GO:0048512	circadian behavior	1/385	62/23328	0.644110234	0.68295	0.41038	Egr1	1
GO:0051926	GO:0051926	negative regulation of calcium ion transport	1/385	62/23328	0.644110234	0.68295	0.41038	Nos3	1
GO:0098930	GO:0098930	axonal transport	1/385	62/23328	0.644110234	0.68295	0.41038	Hspb1	1
GO:0009581	GO:0009581	detection of external stimulus	2/385	133/23328	0.647468944	0.68599	0.41221	Cxcl12/Se	2
GO:0042303	GO:0042303	molting cycle	2/385	133/23328	0.647468944	0.68599	0.41221	Igfbp5/Pd	2
GO:0042633	GO:0042633	hair cycle	2/385	133/23328	0.647468944	0.68599	0.41221	Igfbp5/Pd	2
GO:0050994	GO:0050994	regulation of lipid catabolic process	1/385	63/23328	0.649999409	0.68815	0.4135	Scarb1	1
GO:0060038	GO:0060038	cardiac muscle cell proliferation	1/385	63/23328	0.649999409	0.68815	0.4135	Ncam1	1
GO:0097479	GO:0097479	synaptic vesicle localization	1/385	63/23328	0.649999409	0.68815	0.4135	Pcdh17	1
GO:1901655	GO:1901655	cellular response to ketone	2/385	135/23328	0.655507286	0.69305	0.41645	Aqp1/Bmp	2
GO:0001658	GO:0001658	branching involved in ureteric bud morphogenesis	1/385	64/23328	0.655791381	0.69305	0.41645	Bmp4	1
GO:0007622	GO:0007622	rhythmic behavior	1/385	64/23328	0.655791381	0.69305	0.41645	Egr1	1
GO:0043388	GO:0043388	positive regulation of DNA binding	1/385	64/23328	0.655791381	0.69305	0.41645	Hmgb2	1
GO:0046173	GO:0046173	polyol biosynthetic process	1/385	64/23328	0.655791381	0.69305	0.41645	Acer2	1
GO:0070988	GO:0070988	demethylation	1/385	64/23328	0.655791381	0.69305	0.41645	Apobec3	1
GO:0090278	GO:0090278	negative regulation of peptide hormone secretion	1/385	64/23328	0.655791381	0.69305	0.41645	Sfrp1	1
GO:0035195	GO:0035195	gene silencing by miRNA	2/385	136/23328	0.659471345	0.69677	0.41868	Zfp36/Bm	2
GO:0016236	GO:0016236	macroautophagy	3/385	205/23328	0.660437933	0.69761	0.41919	Nupr1/Kd	3
GO:0009206	GO:0009206	purine ribonucleoside triphosphate biosynthetic proces	1/385	65/23328	0.66148775	0.69766	0.41922	Aldoa	1
GO:0042093	GO:0042093	T-helper cell differentiation	1/385	65/23328	0.66148775	0.69766	0.41922	Anxa1	1
GO:0045454	GO:0045454	cell redox homeostasis	1/385	65/23328	0.66148775	0.69766	0.41922	Prdx1	1
GO:0098840	GO:0098840	protein transport along microtubule	1/385	65/23328	0.66148775	0.69766	0.41922	Hspb1	1
GO:0099118	GO:0099118	microtubule-based protein transport	1/385	65/23328	0.66148775	0.69766	0.41922	Hspb1	1
GO:2000514	GO:2000514	regulation of CD4-positive, alpha-beta T cell activation	1/385	65/23328	0.66148775	0.69766	0.41922	Anxa1	1
GO:0009101	GO:0009101	glycoprotein biosynthetic process	4/385	273/23328	0.662233197	0.69827	0.41959	Gxylt2/Cy	4
GO:0002294	GO:0002294	CD4-positive, alpha-beta T cell differentiation involved	1/385	66/23328	0.667090089	0.70269	0.42224	Anxa1	1
GO:0009145	GO:0009145	purine nucleoside triphosphate biosynthetic process	1/385	66/23328	0.667090089	0.70269	0.42224	Aldoa	1
GO:0048278	GO:0048278	vesicle docking	1/385	66/23328	0.667090089	0.70269	0.42224	Ncam1	1
GO:0070972	GO:0070972	protein localization to endoplasmic reticulum	1/385	66/23328	0.667090089	0.70269	0.42224	Hspa5	1
GO:0009411	GO:0009411	response to UV	2/385	138/23328	0.667289833	0.70272	0.42226	Aqp1/Cdk	2
GO:1903322	GO:1903322	positive regulation of protein modification by small pro	2/385	139/23328	0.671144515	0.7066	0.42459	Cav1/Hsp	2
GO:0002293	GO:0002293	alpha-beta T cell differentiation involved in immune re	1/385	67/23328	0.672599947	0.7076	0.42519	Anxa1	1
GO:0051298	GO:0051298	centrosome duplication	1/385	67/23328	0.672599947	0.7076	0.42519	Plk2	1
GO:0090307	GO:0090307	mitotic spindle assembly	1/385	67/23328	0.672599947	0.7076	0.42519	Hspa1a	1

GO:0002287	GO:0002287	alpha-beta T cell activation involved in immune respon	1/385	68/23328	0.678018847	0.71205	0.42786	Anxa1	1
GO:0006354	GO:0006354	DNA-templated transcription, elongation	1/385	68/23328	0.678018847	0.71205	0.42786	Dab2	1
GO:0009201	GO:0009201	ribonucleoside triphosphate biosynthetic process	1/385	68/23328	0.678018847	0.71205	0.42786	Aldoa	1
GO:0032413	GO:0032413	negative regulation of ion transmembrane transporter a	1/385	68/23328	0.678018847	0.71205	0.42786	Cav1	1
GO:0046637	GO:0046637	regulation of alpha-beta T cell differentiation	1/385	68/23328	0.678018847	0.71205	0.42786	Anxa1	1
GO:0099601	GO:0099601	regulation of neurotransmitter receptor activity	1/385	68/23328	0.678018847	0.71205	0.42786	Reln	1
GO:1902369	GO:1902369	negative regulation of RNA catabolic process	1/385	68/23328	0.678018847	0.71205	0.42786	Zfp36	1
GO:0001909	GO:0001909	leukocyte mediated cytotoxicity	2/385	141/23328	0.678745439	0.71245	0.42811	B2m/Prdx	2
GO:0009152	GO:0009152	purine ribonucleotide biosynthetic process	2/385	141/23328	0.678745439	0.71245	0.42811	Aldoa/Pap	2
GO:0035194	GO:0035194	post-transcriptional gene silencing by RNA	2/385	142/23328	0.682491967	0.71621	0.43036	Zfp36/Bm	2
GO:0001913	GO:0001913	T cell mediated cytotoxicity	1/385	69/23328	0.683348285	0.71675	0.43069	B2m	1
GO:0042058	GO:0042058	regulation of epidermal growth factor receptor signaling	1/385	69/23328	0.683348285	0.71675	0.43069	Ptprf	1
GO:0006305	GO:0006305	DNA alkylation	1/385	70/23328	0.688589736	0.7217	0.43366	H19	1
GO:0006306	GO:0006306	DNA methylation	1/385	70/23328	0.688589736	0.7217	0.43366	H19	1
GO:0017144	GO:0017144	drug metabolic process	1/385	70/23328	0.688589736	0.7217	0.43366	Cyp1a1	1
GO:0071333	GO:0071333	cellular response to glucose stimulus	2/385	144/23328	0.689877915	0.72287	0.43437	Vcam1/Ga	2
GO:0034655	GO:0034655	nucleobase-containing compound catabolic process	5/385	352/23328	0.692707665	0.72565	0.43604	Vim/Btg2/	5
GO:0060675	GO:0060675	ureteric bud morphogenesis	1/385	71/23328	0.693744649	0.72638	0.43647	Bmp4	1
GO:1901796	GO:1901796	regulation of signal transduction by p53 class mediator	1/385	71/23328	0.693744649	0.72638	0.43647	Cd74	1
GO:0016441	GO:0016441	posttranscriptional gene silencing	2/385	146/23328	0.697122115	0.72955	0.43838	Zfp36/Bm	2
GO:0071331	GO:0071331	cellular response to hexose stimulus	2/385	146/23328	0.697122115	0.72955	0.43838	Vcam1/Ga	2
GO:0006402	GO:0006402	mRNA catabolic process	3/385	217/23328	0.697593221	0.72967	0.43846	Vim/Btg2/	3
GO:0051054	GO:0051054	positive regulation of DNA metabolic process	3/385	217/23328	0.697593221	0.72967	0.43846	Pdgfb/Igft	3
GO:0072171	GO:0072171	mesonephric tubule morphogenesis	1/385	72/23328	0.698814447	0.73077	0.43911	Bmp4	1
GO:0071326	GO:0071326	cellular response to monosaccharide stimulus	2/385	147/23328	0.700691475	0.73255	0.44018	Vcam1/Ga	2
GO:0002090	GO:0002090	regulation of receptor internalization	1/385	73/23328	0.703800534	0.73506	0.4417	Dab2	1
GO:0002886	GO:0002886	regulation of myeloid leukocyte mediated immunity	1/385	73/23328	0.703800534	0.73506	0.4417	C3	1
GO:0060997	GO:0060997	dendritic spine morphogenesis	1/385	73/23328	0.703800534	0.73506	0.4417	Reln	1
GO:0097120	GO:0097120	receptor localization to synapse	1/385	73/23328	0.703800534	0.73506	0.4417	Reln	1
GO:0050871	GO:0050871	positive regulation of B cell activation	3/385	220/23328	0.706386429	0.73758	0.44321	Cd38/Cdk	3
GO:0072594	GO:0072594	establishment of protein localization to organelle	6/385	426/23328	0.707687746	0.73876	0.44391	Cd36/Cdk	6
GO:0034404	GO:0034404	nucleobase-containing small molecule biosynthetic pro	1/385	74/23328	0.708704288	0.73945	0.44433	Pde2a	1
GO:0099518	GO:0099518	vesicle cytoskeletal trafficking	1/385	74/23328	0.708704288	0.73945	0.44433	Myo1b	1
GO:0009260	GO:0009260	ribonucleotide biosynthetic process	2/385	150/23328	0.711190691	0.74186	0.44578	Aldoa/Pap	2
GO:0043279	GO:0043279	response to alkaloid	1/385	75/23328	0.713527065	0.74392	0.44702	Ncam1	1
GO:0098661	GO:0098661	inorganic anion transmembrane transport	1/385	75/23328	0.713527065	0.74392	0.44702	Prnp	1
GO:0006910	GO:0006910	phagocytosis, recognition	2/385	152/23328	0.718017931	0.74794	0.44943	Cd36/Sca	2
GO:0000271	GO:0000271	polysaccharide biosynthetic process	1/385	76/23328	0.718270198	0.74794	0.44943	Pdgfb	1
GO:0006094	GO:0006094	gluconeogenesis	1/385	76/23328	0.718270198	0.74794	0.44943	Atf3	1
GO:0045576	GO:0045576	mast cell activation	1/385	76/23328	0.718270198	0.74794	0.44943	Ndrg1	1
GO:1901606	GO:1901606	alpha-amino acid catabolic process	1/385	76/23328	0.718270198	0.74794	0.44943	Nos3	1
GO:0031397	GO:0031397	negative regulation of protein ubiquitination	1/385	77/23328	0.722935	0.75205	0.4519	Cav1	1
GO:0032410	GO:0032410	negative regulation of transporter activity	1/385	77/23328	0.722935	0.75205	0.4519	Cav1	1
GO:1901224	GO:1901224	positive regulation of NIK/NF-kappaB signaling	1/385	77/23328	0.722935	0.75205	0.4519	Edn1	1
GO:1903201	GO:1903201	regulation of oxidative stress-induced cell death	1/385	77/23328	0.722935	0.75205	0.4519	Hspb1	1
GO:0007286	GO:0007286	spermatid development	3/385	227/23328	0.726141612	0.75519	0.45379	Jam2/Dhh	3
GO:0014855	GO:0014855	striated muscle cell proliferation	1/385	78/23328	0.727522761	0.75607	0.45432	Ncam1	1
GO:0060563	GO:0060563	neuroepithelial cell differentiation	1/385	78/23328	0.727522761	0.75607	0.45432	Clic5	1
GO:0061180	GO:0061180	mammary gland epithelium development	1/385	78/23328	0.727522761	0.75607	0.45432	Pthlh	1
GO:0030073	GO:0030073	insulin secretion	3/385	229/23328	0.73159172	0.75981	0.45657	Sfrp1/Cd3	3
GO:0002637	GO:0002637	regulation of immunoglobulin production	1/385	79/23328	0.73203475	0.75981	0.45657	Il33	1
GO:0009205	GO:0009205	purine ribonucleoside triphosphate metabolic process	1/385	79/23328	0.73203475	0.75981	0.45657	Aldoa	1
GO:0019319	GO:0019319	hexose biosynthetic process	1/385	79/23328	0.73203475	0.75981	0.45657	Atf3	1
GO:0140056	GO:0140056	organelle localization by membrane tethering	1/385	79/23328	0.73203475	0.75981	0.45657	Ncam1	1
GO:0045333	GO:0045333	cellular respiration	2/385	157/23328	0.734494335	0.76199	0.45787	Cox4i2/Ct	2
GO:0046390	GO:0046390	ribose phosphate biosynthetic process	2/385	157/23328	0.734494335	0.76199	0.45787	Aldoa/Pap	2
GO:0008088	GO:0008088	axo-dendritic transport	1/385	80/23328	0.736472214	0.76328	0.45865	Hspb1	1
GO:0032465	GO:0032465	regulation of cytokinesis	1/385	80/23328	0.736472214	0.76328	0.45865	Plk2	1
GO:0032729	GO:0032729	positive regulation of interferon-gamma production	1/385	80/23328	0.736472214	0.76328	0.45865	Cd2	1
GO:0072078	GO:0072078	nephron tubule morphogenesis	1/385	80/23328	0.736472214	0.76328	0.45865	Bmp4	1
GO:0099504	GO:0099504	synaptic vesicle cycle	3/385	231/23328	0.736956355	0.7636	0.45884	Pcdh17/C	3
GO:0022904	GO:0022904	respiratory electron transport chain	1/385	81/23328	0.740836383	0.76743	0.46114	Cox4i2	1
GO:0000910	GO:0000910	cytokinesis	2/385	160/23328	0.743982444	0.77049	0.46298	Plk2/Dstn	2
GO:0001912	GO:0001912	positive regulation of leukocyte mediated cytotoxicity	1/385	82/23328	0.745128464	0.77111	0.46335	B2m	1
GO:0051262	GO:0051262	protein tetramerization	1/385	82/23328	0.745128464	0.77111	0.46335	B2m	1
GO:0072088	GO:0072088	nephron epithelium morphogenesis	1/385	82/23328	0.745128464	0.77111	0.46335	Bmp4	1
GO:0006664	GO:0006664	glycolipid metabolic process	1/385	83/23328	0.749349643	0.77509	0.46575	Gm2a	1
GO:0009199	GO:0009199	ribonucleoside triphosphate metabolic process	1/385	83/23328	0.749349643	0.77509	0.46575	Aldoa	1
GO:0048515	GO:0048515	spermatid differentiation	3/385	236/23328	0.74999748	0.77557	0.46604	Jam2/Dhh	3
GO:0001678	GO:0001678	cellular glucose homeostasis	2/385	163/23328	0.753179112	0.77785	0.4674	Vcam1/Ga	2
GO:0022406	GO:0022406	membrane docking	1/385	84/23328	0.75350109	0.77785	0.4674	Ncam1	1
GO:0022900	GO:0022900	electron transport chain	1/385	84/23328	0.75350109	0.77785	0.4674	Cox4i2	1
GO:0030148	GO:0030148	sphingolipid biosynthetic process	1/385	84/23328	0.75350109	0.77785	0.4674	Acer2	1
GO:0072028	GO:0072028	nephron morphogenesis	1/385	84/23328	0.75350109	0.77785	0.4674	Bmp4	1
GO:1903363	GO:1903363	negative regulation of cellular protein catabolic proces	1/385	84/23328	0.75350109	0.77785	0.4674	Anxa2	1
GO:1903509	GO:1903509	liposaccharide metabolic process	1/385	84/23328	0.75350109	0.77785	0.4674	Gm2a	1
GO:1990830	GO:1990830	cellular response to leukemia inhibitory factor	4/385	311/23328	0.757359294	0.7811	0.46936	Dtx1/Socs	4
GO:0006835	GO:0006835	dicarboxylic acid transport	1/385	85/23328	0.757583954	0.7811	0.46936	Itgb1	1
GO:0007218	GO:0007218	neuropeptide signaling pathway	1/385	85/23328	0.757583954	0.7811	0.46936	Nts	1
GO:0043648	GO:0043648	dicarboxylic acid metabolic process	1/385	85/23328	0.757583954	0.7811	0.46936	Ass1	1
GO:0046364	GO:0046364	monosaccharide biosynthetic process	1/385	85/23328	0.757583954	0.7811	0.46936	Atf3	1
GO:1990823	GO:1990823	response to leukemia inhibitory factor	4/385	312/23328	0.759555962	0.78294	0.47046	Dtx1/Socs	4
GO:1903320	GO:1903320	regulation of protein modification by small protein conj	3/385	240/23328	0.760054123	0.78326	0.47066	Cav1/Egr1	3
GO:0044272	GO:0044272	sulfur compound biosynthetic process	1/385	86/23328	0.761599364	0.78447	0.47138	Papss2	1
GO:0061333	GO:0061333	renal tubule morphogenesis	1/385	86/23328	0.761599364	0.78447	0.47138	Bmp4	1
GO:0006112	GO:0006112	energy reserve metabolic process	1/385	88/23328	0.769432252	0.79137	0.47553	Adgrf5	1
GO:0009144	GO:0009144	purine nucleoside triphosphate metabolic process	1/385	88/23328	0.769432252	0.79137	0.47553	Aldoa	1
GO:0045913	GO:0045913	positive regulation of carbohydrate metabolic process	1/385	88/23328	0.769432252	0.79137	0.47553	Pdgfb	1
GO:1900182	GO:1900182	positive regulation of protein localization to nucleus	1/385	88/23328	0.769432252	0.79137	0.47553	Tek	1
GO:1903312	GO:1903312	negative regulation of mRNA metabolic process	1/385	88/23328	0.769432252	0.79137	0.47553	Zfp36	1
GO:1903321	GO:1903321	negative regulation of protein modification by small pro	1/385	88/23328	0.769432252	0.79137	0.47553	Cav1	1
GO:0006260	GO:0006260	DNA replication	3/385	244/23328	0.76978179	0.79153	0.47563	Id3/Pdgfb/	3
GO:0031047	GO:0031047	gene silencing by RNA	2/385	169/23328	0.770721445	0.79211	0.47597	Zfp36/Bm	2
GO:0035303	GO:0035303	regulation of dephosphorylation	2/385	169/23328	0.770721445	0.79211	0.47597	Cdh5/Hsp	2
GO:0006887	GO:0006887	exocytosis	5/385	389/23328	0.771896308	0.79312	0.47658	Exoc3l2/N	5
GO:0009063	GO:0009063	cellular amino acid catabolic process	1/385	90/23328	0.777008429	0.79779	0.47938	Nos3	1
GO:0019226	GO:0019226	transmission of nerve impulse	1/385	90/23328	0.777008429	0.79779	0.47938	Pmp22	1
GO:0032024	GO:0032024	positive regulation of insulin secretion	1/385	90/23328	0.777008429	0.79779	0.47938	Cd38	1
GO:0015980	GO:0015980	energy derivation by oxidation of organic compounds	3/385	248/23328	0.779185951	0.79983	0.48061	Cox4i2/Ac	3
GO:0000045	GO:0000045	autophagosome assembly	1/385	92/23328	0.784336288	0.80469	0.48353	Nupr1	1
GO:0007033	GO:0007033	vacuole organization	2/385	174/23328	0.784501771	0.80469	0.48353	Nupr1/Anx	2
GO:0051648	GO:0051648	vesicle localization	2/385	174/23328	0.784501771	0.80469	0.48353	Pcdh17/M	2
GO:0006401	GO:0006401	RNA catabolic process	3/385	254/23328	0.792698397	0.8127	0.48835	Vim/Btg2/	3
GO:0017157	GO:0017157	regulation of exocytosis	3/385	254/23328	0.792698397	0.8127	0.48835	Ncam1/Sc	3
GO:0002251	GO:0002251	organ or tissue specific immune response	1/385	95/23328	0.794880165	0.81414	0.48921	Cfh	1
GO:0021954	GO:0021954	central nervous system neuron development	1/385	95/23328	0.794880165	0.81414	0.48921	Btg2	1
GO:0031343	GO:0031343	positive regulation of cell killing	1/385	95/23328	0.794880165	0.81414	0.48921	B2m	1
GO:1902600	GO:1902600	proton transmembrane transport	1/385	95/23328	0.794880165	0.81414	0.48921	Slc25a4	1
GO:0007051	GO:0007051	spindle organization	2/385	179/23328	0.797550219	0.81667	0.49073	Plk2/Hspa	2
GO:0030838	GO:0030838	positive regulation of actin filament polymerization	1/385	96/23328	0.798279258	0.81702	0.49094	Gmfg	1
GO:0060021	GO:0060021	roof of mouth development	1/385	96/23328	0.798279258	0.81702	0.49094	Tbx1	1
GO:1901605	GO:1901605	alpha-amino acid metabolic process	2/385	180/23328	0.800074603	0.81866	0.49193	Nos3/Ass	2
GO:1905037	GO:1905037	autophagosome organization	1/385	97/23328	0.801622168	0.82004	0.49276	Nupr1	1
GO:0050853	GO:0050853	B cell receptor signaling pathway	2/385	181/23328	0.802571089	0.82081	0.49322	Cd38/Bmx	2
GO:0031503	GO:0031503	protein-containing complex localization	3/385	259/23328	0.803427732	0.82149	0.49363	Reln/Exoc	3
GO:0006892	GO:0006892	post-Golgi vesicle-mediated transport	1/385	98/23328	0.804909821	0.8224	0.49417	Myo1b	1
GO:0036473	GO:0036473	cell death in response to oxidative stress	1/385	98/23328	0.804909821	0.8224	0.49417	Hspb1	1
GO:0140029	GO:0140029	exocytic process	1/385	98/23328	0.804909821	0.8224	0.49417	Ncam1	1
GO:0099003	GO:0099003	vesicle-mediated transport in synapse	3/385	260/23328	0.805516872	0.82282	0.49443	Pcdh17/C	3
GO:0033865	GO:0033865	nucleoside bisphosphate metabolic process	1/385	99/23328	0.808143128	0.8247	0.49555	Papss2	1
GO:0033875	GO:0033875	ribonucleoside bisphosphate metabolic process	1/385	99/23328	0.808143128	0.8247	0.49555	Papss2	1
GO:0034032	GO:0034032	purine nucleoside bisphosphate metabolic process	1/385	99/23328	0.808143128	0.8247	0.49555	Papss2	1
GO:0042158	GO:0042158	lipoprotein biosynthetic process	1/385	99/23328	0.808143128	0.8247	0.49555	Apoe	1
GO:0002377	GO:0002377	immunoglobulin production	3/385	262/23328	0.809639248	0.82602	0.49635	Fgl2/Il33/I	3
GO:0140013	GO:0140013	meiotic nuclear division	2/385	184/23328	0.80989546	0.82608	0.49639	Pde3a/Cy	2
GO:0006352	GO:0006352	DNA-templated transcription, initiation	1/385	101/23328	0.814450273	0.83012	0.49881	Jun	1
GO:0007041	GO:0007041	lysosomal transport	1/385	101/23328	0.814450273	0.83012	0.49881	Hspa1a	1
GO:0060993	GO:0060993	kidney morphogenesis	1/385	101/23328	0.814450273	0.83012	0.49881	Bmp4	1
GO:1903311	GO:1903311	regulation of mRNA metabolic process	3/385	266/23328	0.817663338	0.83319	0.50066	Vim/Btg2/	3

GO:0007173	GO:0007173	epidermal growth factor receptor signaling pathway	1/385	103/23328	0.820550597	0.83573	0.50218	Ptprf	1
GO:0046427	GO:0046427	positive regulation of receptor signaling pathway via JA	1/385	103/23328	0.820550597	0.83573	0.50218	Pecam1	1
GO:0050796	GO:0050796	regulation of insulin secretion	2/385	190/23328	0.823822017	0.83886	0.50406	Sfrp1/Cd3	2
GO:0001910	GO:0001910	regulation of leukocyte mediated cytotoxicity	1/385	105/23328	0.826450863	0.84092	0.5053	B2m	1
GO:0002444	GO:0002444	myeloid leukocyte mediated immunity	1/385	105/23328	0.826450863	0.84092	0.5053	C3	1
GO:0045921	GO:0045921	positive regulation of exocytosis	1/385	105/23328	0.826450863	0.84092	0.5053	Sdc1	1
GO:0006605	GO:0006605	protein targeting	3/385	271/23328	0.827288522	0.84157	0.50569	Prnp/Rtp4	3
GO:0001578	GO:0001578	microtubule bundle formation	1/385	106/23328	0.829328029	0.84323	0.50669	Tppp3	1
GO:0021510	GO:0021510	spinal cord development	1/385	106/23328	0.829328029	0.84323	0.50669	Reln	1
GO:0006486	GO:0006486	protein glycosylation	2/385	193/23328	0.830436582	0.84374	0.507	Gxylt2/Ac	2
GO:0015931	GO:0015931	nucleobase-containing compound transport	2/385	193/23328	0.830436582	0.84374	0.507	Slc25a4/Z	2
GO:0043413	GO:0043413	macromolecule glycosylation	2/385	193/23328	0.830436582	0.84374	0.507	Gxylt2/Ac	2
GO:1904894	GO:1904894	positive regulation of receptor signaling pathway via ST	1/385	107/23328	0.832157618	0.84529	0.50793	Pecam1	1
GO:0030705	GO:0030705	cytoskeleton-dependent intracellular transport	2/385	194/23328	0.832591295	0.84552	0.50807	Hspb1/My	2
GO:0043255	GO:0043255	regulation of carbohydrate biosynthetic process	1/385	108/23328	0.834940414	0.8477	0.50938	Pdgfb	1
GO:0051028	GO:0051028	mRNA transport	1/385	109/23328	0.83767719	0.85027	0.51092	Zfp36	1
GO:0016051	GO:0016051	carbohydrate biosynthetic process	2/385	197/23328	0.838908177	0.85132	0.51155	Atf3/Pdgft	2
GO:0051225	GO:0051225	spindle assembly	1/385	112/23328	0.845618925	0.85792	0.51552	Hspa1a	1
GO:1903046	GO:1903046	meiotic cell cycle process	2/385	202/23328	0.848958893	0.8611	0.51743	Pde3a/Cy	2
GO:0005976	GO:0005976	polysaccharide metabolic process	1/385	114/23328	0.850696901	0.86265	0.51836	Pdgfb	1
GO:0031396	GO:0031396	regulation of protein ubiquitination	2/385	206/23328	0.856585727	0.86841	0.52182	Cav1/Hsp	2
GO:0070085	GO:0070085	glycosylation	2/385	208/23328	0.860266031	0.87182	0.52387	Gxylt2/Ac	2
GO:0030317	GO:0030317	flagellated sperm motility	1/385	118/23328	0.860358482	0.87182	0.52387	Pltp	1
GO:0046467	GO:0046467	membrane lipid biosynthetic process	1/385	121/23328	0.867193127	0.87807	0.52763	Acer2	1
GO:0072329	GO:0072329	monocarboxylic acid catabolic process	1/385	121/23328	0.867193127	0.87807	0.52763	Cyp26b1	1
GO:0097722	GO:0097722	sperm motility	1/385	121/23328	0.867193127	0.87807	0.52763	Pltp	1
GO:0097305	GO:0097305	response to alcohol	2/385	212/23328	0.867368987	0.87807	0.52763	Adh7/Adh	2
GO:0000723	GO:0000723	telomere maintenance	1/385	123/23328	0.871563153	0.88189	0.52992	Hspa1a	1
GO:0010921	GO:0010921	regulation of phosphatase activity	1/385	123/23328	0.871563153	0.88189	0.52992	Hsp90b1	1
GO:0006997	GO:0006997	nucleus organization	1/385	124/23328	0.873694081	0.8834	0.53083	Hmgb2	1
GO:0051963	GO:0051963	regulation of synapse assembly	1/385	124/23328	0.873694081	0.8834	0.53083	Gap43	1
GO:0090277	GO:0090277	positive regulation of peptide hormone secretion	1/385	124/23328	0.873694081	0.8834	0.53083	Cd38	1
GO:0032200	GO:0032200	telomere organization	1/385	125/23328	0.875789745	0.88531	0.53198	Hspa1a	1
GO:0006611	GO:0006611	protein export from nucleus	1/385	127/23328	0.879877598	0.88901	0.5342	Gas6	1
GO:0042157	GO:0042157	lipoprotein metabolic process	1/385	127/23328	0.879877598	0.88901	0.5342	Apoe	1
GO:0006282	GO:0006282	regulation of DNA repair	1/385	128/23328	0.881870922	0.89081	0.53528	Ier3	1
GO:0060294	GO:0060294	cilium movement involved in cell motility	1/385	129/23328	0.883831254	0.89258	0.53634	Pltp	1
GO:0016054	GO:0016054	organic acid catabolic process	2/385	223/23328	0.885228181	0.89356	0.53693	Nos3/Cyp	2
GO:0046395	GO:0046395	carboxylic acid catabolic process	2/385	223/23328	0.885228181	0.89356	0.53693	Nos3/Cyp	2
GO:0042752	GO:0042752	regulation of circadian rhythm	1/385	130/23328	0.885759137	0.89388	0.53712	Prox1	1
GO:0001539	GO:0001539	cilium or flagellum-dependent cell motility	1/385	136/23328	0.896674415	0.90445	0.54348	Pltp	1
GO:0060285	GO:0060285	cilium-dependent cell motility	1/385	136/23328	0.896674415	0.90445	0.54348	Pltp	1
GO:0090276	GO:0090276	regulation of peptide hormone secretion	2/385	235/23328	0.902158497	0.90977	0.54667	Sfrp1/Cd3	2
GO:0007034	GO:0007034	vacuolar transport	1/385	140/23328	0.903366921	0.91077	0.54727	Hspa1a	1
GO:0009451	GO:0009451	RNA modification	1/385	143/23328	0.908100964	0.91532	0.55001	Apobec3	1
GO:0051168	GO:0051168	nuclear export	1/385	146/23328	0.91260366	0.91941	0.55247	Gas6	1
GO:0098693	GO:0098693	regulation of synaptic vesicle cycle	1/385	146/23328	0.91260366	0.91941	0.55247	Pcdh17	1
GO:0006836	GO:0006836	neurotransmitter transport	2/385	245/23328	0.914458817	0.92106	0.55346	Itgb1/Slc6	2
GO:0051588	GO:0051588	regulation of neurotransmitter transport	1/385	151/23328	0.919624429	0.92604	0.55645	Itgb1	1
GO:0006520	GO:0006520	cellular amino acid metabolic process	2/385	250/23328	0.920051184	0.92625	0.55658	Nos3/Ass	2
GO:0016482	GO:0016482	cytosolic transport	1/385	155/23328	0.924833724	0.93062	0.5592	Dab2	1
GO:0051650	GO:0051650	establishment of vesicle localization	1/385	155/23328	0.924833724	0.93062	0.5592	Myo1b	1
GO:0050657	GO:0050657	nucleic acid transport	1/385	157/23328	0.927310679	0.93266	0.56043	Zfp36	1
GO:0050658	GO:0050658	RNA transport	1/385	157/23328	0.927310679	0.93266	0.56043	Zfp36	1
GO:0051236	GO:0051236	establishment of RNA localization	1/385	160/23328	0.930874289	0.93602	0.56245	Zfp36	1
GO:0010970	GO:0010970	transport along microtubule	1/385	164/23328	0.935356156	0.9403	0.56502	Hspb1	1
GO:0048193	GO:0048193	Golgi vesicle transport	2/385	268/23328	0.937464512	0.94219	0.56616	Plpp3/Myo	2
GO:0006338	GO:0006338	chromatin remodeling	1/385	167/23328	0.938526277	0.94303	0.56666	Hmgb2	1
GO:0018205	GO:0018205	peptidyl-lysine modification	3/385	364/23328	0.941198651	0.94549	0.56814	Lox/Tgm2	3
GO:0016458	GO:0016458	gene silencing	2/385	279/23328	0.94626909	0.95036	0.57106	Zfp36/Bm	2
GO:0003341	GO:0003341	cilium movement	1/385	180/23328	0.950565892	0.95444	0.57352	Pltp	1
GO:0006403	GO:0006403	RNA localization	1/385	182/23328	0.952196639	0.95585	0.57436	Zfp36	1
GO:0006479	GO:0006479	protein methylation	1/385	183/23328	0.952991778	0.95619	0.57457	Btg2	1
GO:0008213	GO:0008213	protein alkylation	1/385	183/23328	0.952991778	0.95619	0.57457	Btg2	1
GO:0043414	GO:0043414	macromolecule methylation	2/385	297/23328	0.95818564	0.96117	0.57756	Btg2/H19	2
GO:0006473	GO:0006473	protein acetylation	1/385	192/23328	0.959579838	0.96234	0.57826	Nupr1	1
GO:0099111	GO:0099111	microtubule-based transport	1/385	195/23328	0.961564393	0.9641	0.57932	Hspb1	1
GO:0051321	GO:0051321	meiotic cell cycle	2/385	317/23328	0.968456372	0.97078	0.58333	Pde3a/Cy	2
GO:0032259	GO:0032259	methylation	2/385	349/23328	0.980034296	0.98215	0.59016	Btg2/H19	2
GO:0043543	GO:0043543	protein acylation	1/385	237/23328	0.981018399	0.9829	0.59062	Nupr1	1
GO:0071103	GO:0071103	DNA conformation change	1/385	242/23328	0.982549049	0.9842	0.5914	Anxa1	1
GO:0001505	GO:0001505	regulation of neurotransmitter levels	1/385	257/23328	0.986440925	0.98786	0.5936	Itgb1	1
GO:0051656	GO:0051656	establishment of organelle localization	2/385	382/23328	0.987631588	0.98881	0.59417	Itgb1/Myo	2
GO:0007018	GO:0007018	microtubule-based movement	2/385	385/23328	0.988162385	0.98911	0.59435	Hspb1/Plt	2
GO:0006310	GO:0006310	DNA recombination	1/385	288/23328	0.991955147	0.99267	0.59649	Hmgb2	1
GO:0060271	GO:0060271	cilium assembly	1/385	310/23328	0.994448132	0.99492	0.59784	Ablim3	1
GO:0044782	GO:0044782	cilium organization	1/385	343/23328	0.996819252	0.99706	0.59912	Ablim3	1
GO:0006281	GO:0006281	DNA repair	2/385	481/23328	0.997162364	0.99716	0.59919	Ier3/Hspa	2

EC 2 GO:BP

	ID	Description	GeneRatio	BgRatio	pvalue	p.adjust	qvalue	geneID	Count
GO:0009611	GO:0009611	response to wounding	42/374	492/23328	9.07E-19	2.32E-15	1.39E-15	Cfh/Pecar	42
GO:1901342	GO:1901342	regulation of vasculature development	36/374	356/23328	1.37E-18	2.32E-15	1.39E-15	Ptgis/Bmp	36
GO:0001667	GO:0001667	ameboidal-type cell migration	39/374	427/23328	1.67E-18	2.32E-15	1.39E-15	Pecam1/S	39
GO:0045765	GO:0045765	regulation of angiogenesis	34/374	320/23328	2.77E-18	2.89E-15	1.74E-15	Ptgis/Apln	34
GO:0050673	GO:0050673	epithelial cell proliferation	39/374	438/23328	4.02E-18	3.35E-15	2.02E-15	Sfrp1/Gkn	39
GO:0001935	GO:0001935	endothelial cell proliferation	24/374	149/23328	2.10E-17	1.46E-14	8.76E-15	Scarb1/Br	24
GO:2000146	GO:2000146	negative regulation of cell motility	31/374	291/23328	8.45E-17	5.03E-14	3.03E-14	Sfrp1/Gat	31
GO:0051271	GO:0051271	negative regulation of cellular component movement	32/374	326/23328	2.87E-16	1.28E-13	7.67E-14	Sfrp1/Gat	32
GO:0042060	GO:0042060	wound healing	33/374	349/23328	2.93E-16	1.28E-13	7.67E-14	Cfh/Pecar	33
GO:0001936	GO:0001936	regulation of endothelial cell proliferation	22/374	134/23328	3.06E-16	1.28E-13	7.67E-14	Bmp4/Apl	22
GO:0030336	GO:0030336	negative regulation of cell migration	29/374	278/23328	1.51E-15	5.71E-13	3.44E-13	Sfrp1/Igfb	29
GO:0050678	GO:0050678	regulation of epithelial cell proliferation	33/374	372/23328	1.91E-15	6.62E-13	3.98E-13	Sfrp1/Gkn	33
GO:0040013	GO:0040013	negative regulation of locomotion	31/374	326/23328	2.07E-15	6.63E-13	3.99E-13	Sfrp1/Gat	31
GO:0031589	GO:0031589	cell-substrate adhesion	32/374	351/23328	2.39E-15	7.11E-13	4.28E-13	Sfrp1/Vca	32
GO:0050900	GO:0050900	leukocyte migration	32/374	360/23328	4.89E-15	1.36E-12	8.18E-13	Vcam1/Pe	32
GO:0010631	GO:0010631	epithelial cell migration	29/374	295/23328	7.21E-15	1.88E-12	1.13E-12	Pecam1/S	29
GO:0043542	GO:0043542	endothelial cell migration	25/374	212/23328	8.36E-15	1.99E-12	1.20E-12	Pecam1/S	25
GO:0090132	GO:0090132	epithelium migration	29/374	297/23328	8.61E-15	1.99E-12	1.20E-12	Pecam1/S	29
GO:0090130	GO:0090130	tissue migration	29/374	299/23328	1.03E-14	2.25E-12	1.35E-12	Pecam1/S	29
GO:0030198	GO:0030198	extracellular matrix organization	28/374	302/23328	9.29E-14	1.94E-11	1.16E-11	Eln/Scx/L	28
GO:0043062	GO:0043062	extracellular structure organization	28/374	303/23328	1.01E-13	2.00E-11	1.20E-11	Eln/Scx/L	28
GO:0003018	GO:0003018	vascular process in circulatory system	24/374	224/23328	2.47E-13	4.68E-11	2.81E-11	Gja5/Mgll/	24
GO:1904018	GO:1904018	positive regulation of vasculature development	23/374	208/23328	4.02E-13	7.18E-11	4.32E-11	Ptgis/Apln	23
GO:0045766	GO:0045766	positive regulation of angiogenesis	22/374	188/23328	4.13E-13	7.18E-11	4.32E-11	Ptgis/Apln	22
GO:0050878	GO:0050878	regulation of body fluid levels	29/374	354/23328	7.73E-13	1.29E-10	7.75E-11	Cfh/Vwf/G	29
GO:0010811	GO:0010811	positive regulation of cell-substrate adhesion	18/374	128/23328	2.48E-12	3.98E-10	2.39E-10	Kdr/Cd36/	18
GO:1903034	GO:1903034	regulation of response to wounding	21/374	186/23328	2.87E-12	4.43E-10	2.67E-10	Klk8/Selp/	21
GO:0001666	GO:0001666	response to hypoxia	22/374	208/23328	3.24E-12	4.83E-10	2.90E-10	Ptgis/Kdr/	22
GO:0045785	GO:0045785	positive regulation of cell adhesion	31/374	435/23328	4.76E-12	6.84E-10	4.11E-10	Vcam1/Kc	31
GO:0010810	GO:0010810	regulation of cell-substrate adhesion	22/374	214/23328	5.74E-12	7.98E-10	4.80E-10	Kdr/Cd36/	22
GO:0019932	GO:0019932	second-messenger-mediated signaling	31/374	445/23328	8.53E-12	1.15E-09	6.90E-10	Vcam1/Nc	31
GO:0032103	GO:0032103	positive regulation of response to external stimulus	30/374	418/23328	8.93E-12	1.16E-09	7.00E-10	Fabp4/Kd	30
GO:0002040	GO:0002040	sprouting angiogenesis	17/374	123/23328	1.35E-11	1.71E-09	1.03E-09	Esm1/Bm	17
GO:0061041	GO:0061041	regulation of wound healing	18/374	146/23328	2.40E-11	2.89E-09	1.74E-09	Selp/Cd36	18
GO:0007596	GO:0007596	blood coagulation	19/374	166/23328	2.50E-11	2.89E-09	1.74E-09	Cfh/Vwf/S	19
GO:0010594	GO:0010594	regulation of endothelial cell migration	19/374	166/23328	2.50E-11	2.89E-09	1.74E-09	Bmp4/Kdr	19
GO:0007599	GO:0007599	hemostasis	19/374	168/23328	3.09E-11	3.48E-09	2.09E-09	Cfh/Vwf/S	19
GO:0036293	GO:0036293	response to decreased oxygen levels	22/374	234/23328	3.38E-11	3.66E-09	2.20E-09	Ptgis/Kdr/	22
GO:0050817	GO:0050817	coagulation	19/374	169/23328	3.43E-11	3.66E-09	2.20E-09	Cfh/Vwf/S	19
GO:0010632	GO:0010632	regulation of epithelial cell migration	22/374	235/23328	3.67E-11	3.83E-09	2.30E-09	Bmp4/Kdr	22
GO:0048608	GO:0048608	reproductive structure development	31/374	477/23328	4.97E-11	5.05E-09	3.04E-09	Sfrp1/Vca	31
GO:0061458	GO:0061458	reproductive system development	31/374	481/23328	6.12E-11	6.08E-09	3.66E-09	Sfrp1/Vca	31
GO:0050818	GO:0050818	regulation of coagulation	14/374	86/23328	9.29E-11	9.01E-09	5.42E-09	Selp/Cd36	14
GO:1901343	GO:1901343	negative regulation of vasculature development	17/374	139/23328	9.84E-11	9.32E-09	5.61E-09	Bmp4/Fbl	17
GO:0003158	GO:0003158	endothelium development	16/374	122/23328	1.22E-10	1.13E-08	6.79E-09	Pecam1/E	16
GO:0060326	GO:0060326	cell chemotaxis	24/374	303/23328	1.55E-10	1.40E-08	8.44E-09	Vcam1/Lo	24
GO:0048660	GO:0048660	regulation of smooth muscle cell proliferation	18/374	165/23328	1.87E-10	1.66E-08	9.99E-09	Bmp4/Igfb	18
GO:0010721	GO:0010721	negative regulation of cell development	27/374	387/23328	1.91E-10	1.66E-08	1.00E-08	Klk8/Bmp	27
GO:0001938	GO:0001938	positive regulation of endothelial cell proliferation	14/374	91/23328	2.03E-10	1.70E-08	1.02E-08	Bmp4/Apl	14

GO:0045446	GO:0045446	endothelial cell differentiation	15/374	108/2332ℓ	2.04E-10	1.70E-08	1.02E-08	Pecam1/E	15
GO:0070482	GO:0070482	response to oxygen levels	23/374	287/2332ℓ	2.99E-10	2.44E-08	1.47E-08	Ptgis/Kdr/	23
GO:0048659	GO:0048659	smooth muscle cell proliferation	18/374	170/2332ℓ	3.07E-10	2.46E-08	1.48E-08	Bmp4/Igfb	18
GO:1903036	GO:1903036	positive regulation of response to wounding	13/374	78/23328	3.23E-10	2.54E-08	1.53E-08	Selp/Cd36	13
GO:0018108	GO:0018108	peptidyl-tyrosine phosphorylation	24/374	322/2332ℓ	5.35E-10	4.13E-08	2.48E-08	Sfrp1/Pec	24
GO:0030193	GO:0030193	regulation of blood coagulation	13/374	82/23328	6.18E-10	4.68E-08	2.82E-08	Selp/Cd36	13
GO:0018212	GO:0018212	peptidyl-tyrosine modification	24/374	325/2332ℓ	6.45E-10	4.80E-08	2.89E-08	Sfrp1/Pec	24
GO:1900046	GO:1900046	regulation of hemostasis	13/374	83/23328	7.22E-10	5.28E-08	3.18E-08	Selp/Cd36	13
GO:0010595	GO:0010595	positive regulation of endothelial cell migration	14/374	103/2332ℓ	1.10E-09	7.92E-08	4.76E-08	Bmp4/Kdr	14
GO:0016525	GO:0016525	negative regulation of angiogenesis	15/374	122/2332ℓ	1.17E-09	8.26E-08	4.97E-08	Fbln5/Cd3	15
GO:0051961	GO:0051961	negative regulation of nervous system development	25/374	365/2332ℓ	1.34E-09	9.32E-08	5.61E-08	Cpe/Klk8/	25
GO:0001503	GO:0001503	ossification	26/374	394/2332ℓ	1.37E-09	9.39E-08	5.64E-08	Sfrp1/Mgp	26
GO:1903706	GO:1903706	regulation of hemopoiesis	27/374	424/2332ℓ	1.43E-09	9.60E-08	5.77E-08	Sfrp1/Rbp	27
GO:0042493	GO:0042493	response to drug	21/374	260/2332ℓ	1.56E-09	1.03E-07	6.21E-08	Sfrp1/Pam	21
GO:0033002	GO:0033002	muscle cell proliferation	20/374	236/2332ℓ	1.67E-09	1.09E-07	6.56E-08	Ncam1/Br	20
GO:2000181	GO:2000181	negative regulation of blood vessel morphogenesis	15/374	126/2332ℓ	1.84E-09	1.18E-07	7.11E-08	Fbln5/Cd3	15
GO:0050727	GO:0050727	regulation of inflammatory response	25/374	372/2332ℓ	1.98E-09	1.23E-07	7.42E-08	Ptgis/Mgll	25
GO:0007160	GO:0007160	cell-matrix adhesion	19/374	214/2332ℓ	1.98E-09	1.23E-07	7.42E-08	Vcam1/Pe	19
GO:0048661	GO:0048661	positive regulation of smooth muscle cell proliferation	14/374	108/2332ℓ	2.08E-09	1.28E-07	7.67E-08	Bmp4/Thb	14
GO:0090303	GO:0090303	positive regulation of wound healing	11/374	60/23328	2.64E-09	1.59E-07	9.58E-08	Selp/Cd36	11
GO:0010634	GO:0010634	positive regulation of epithelial cell migration	16/374	150/2332ℓ	2.70E-09	1.61E-07	9.67E-08	Bmp4/Kdr	16
GO:0030595	GO:0030595	leukocyte chemotaxis	19/374	219/2332ℓ	2.91E-09	1.71E-07	1.03E-07	Ednrb/Lbp	19
GO:0048771	GO:0048771	tissue remodeling	18/374	196/2332ℓ	3.10E-09	1.79E-07	1.08E-07	Sfrp1/Gja5	18
GO:0048010	GO:0048010	vascular endothelial growth factor receptor signaling pa	10/374	48/23328	3.79E-09	2.16E-07	1.30E-07	Bmp4/Kdr	10
GO:0050768	GO:0050768	negative regulation of neurogenesis	23/374	337/2332ℓ	6.65E-09	3.75E-07	2.25E-07	Klk8/Bmp4	23
GO:0050679	GO:0050679	positive regulation of epithelial cell proliferation	18/374	208/2332ℓ	7.95E-09	4.42E-07	2.66E-07	Sfrp1/Htra	18
GO:0045665	GO:0045665	negative regulation of neuron differentiation	20/374	259/2332ℓ	8.30E-09	4.55E-07	2.74E-07	Klk8/Ednr	20
GO:0043116	GO:0043116	negative regulation of vascular permeability	7/374	19/23328	1.10E-08	5.95E-07	3.58E-07	Apoe/Pde	7
GO:0001763	GO:0001763	morphogenesis of a branching structure	19/374	241/2332ℓ	1.41E-08	7.51E-07	4.52E-07	Cpe/Sfrp1	19
GO:0010712	GO:0010712	regulation of collagen metabolic process	10/374	55/23328	1.53E-08	8.09E-07	4.86E-07	Bmp4/Scx	10
GO:0032102	GO:0032102	negative regulation of response to external stimulus	24/374	384/2332ℓ	1.71E-08	8.93E-07	5.37E-07	Klk8/Htra1	24
GO:0097529	GO:0097529	myeloid leukocyte migration	18/374	219/2332ℓ	1.78E-08	9.16E-07	5.51E-07	Pecam1/E	18
GO:0060840	GO:0060840	artery development	13/374	108/2332ℓ	1.96E-08	9.96E-07	5.99E-07	Bmp4/Gja	13
GO:0061138	GO:0061138	morphogenesis of a branching epithelium	18/374	222/2332ℓ	2.20E-08	1.10E-06	6.64E-07	Sfrp1/Mgp	18
GO:0003007	GO:0003007	heart morphogenesis	20/374	277/2332ℓ	2.57E-08	1.28E-06	7.68E-07	Cpe/Bmp4	20
GO:0035296	GO:0035296	regulation of tube diameter	16/374	176/2332ℓ	2.71E-08	1.30E-06	7.80E-07	Gja5/Mgll/	16
GO:0097746	GO:0097746	regulation of blood vessel diameter	16/374	176/2332ℓ	2.71E-08	1.30E-06	7.80E-07	Gja5/Mgll/	16
GO:0031349	GO:0031349	positive regulation of defense response	20/374	278/2332ℓ	2.73E-08	1.30E-06	7.80E-07	Fabp4/Lbp	20
GO:0032963	GO:0032963	collagen metabolic process	13/374	111/2332ℓ	2.74E-08	1.30E-06	7.80E-07	Bmp4/Scx	13
GO:0035150	GO:0035150	regulation of tube size	16/374	177/2332ℓ	2.94E-08	1.37E-06	8.27E-07	Gja5/Mgll/	16
GO:0060485	GO:0060485	mesenchyme development	20/374	281/2332ℓ	3.27E-08	1.51E-06	9.10E-07	Sfrp1/Bmp	20
GO:1902903	GO:1902903	regulation of supramolecular fiber organization	23/374	370/2332ℓ	3.78E-08	1.73E-06	1.04E-06	Clu/Pecar	23
GO:0007229	GO:0007229	integrin-mediated signaling pathway	12/374	95/23328	4.00E-08	1.81E-06	1.09E-06	Itgbl1/Fn1	12
GO:0048844	GO:0048844	artery morphogenesis	11/374	77/23328	4.03E-08	1.81E-06	1.09E-06	Bmp4/Gja	11
GO:0007015	GO:0007015	actin filament organization	25/374	437/2332ℓ	4.87E-08	2.16E-06	1.30E-06	Pecam1/T	25
GO:0060537	GO:0060537	muscle tissue development	26/374	470/2332ℓ	5.11E-08	2.24E-06	1.35E-06	Ncam1/El	26
GO:0008217	GO:0008217	regulation of blood pressure	17/374	209/2332ℓ	5.18E-08	2.25E-06	1.35E-06	Gja5/Eph	17
GO:0007162	GO:0007162	negative regulation of cell adhesion	20/374	290/2332ℓ	5.50E-08	2.36E-06	1.42E-06	Bmp4/Thb	20
GO:0032970	GO:0032970	regulation of actin filament-based process	24/374	410/2332ℓ	5.92E-08	2.52E-06	1.52E-06	Pecam1/F	24
GO:0071900	GO:0071900	regulation of protein serine/threonine kinase activity	25/374	442/2332ℓ	6.07E-08	2.56E-06	1.54E-06	Sfrp1/Bmp	25
GO:0043534	GO:0043534	blood vessel endothelial cell migration	13/374	119/2332ℓ	6.34E-08	2.64E-06	1.59E-06	Scarb1/Kc	13
GO:0030278	GO:0030278	regulation of ossification	18/374	241/2332ℓ	7.74E-08	3.20E-06	1.92E-06	Sfrp1/Mgp	18
GO:0030168	GO:0030168	platelet activation	11/374	83/23328	8.93E-08	3.65E-06	2.20E-06	Cfh/Vwf/S	11
GO:0007517	GO:0007517	muscle organ development	24/374	420/2332ℓ	9.28E-08	3.76E-06	2.26E-06	Ncam1/El	24
GO:0003094	GO:0003094	glomerular filtration	7/374	25/23328	9.65E-08	3.87E-06	2.33E-06	Gja5/Adgr	7
GO:0032956	GO:0032956	regulation of actin cytoskeleton organization	22/374	361/2332ℓ	1.06E-07	4.21E-06	2.53E-06	Pecam1/F	22
GO:0003206	GO:0003206	cardiac chamber morphogenesis	14/374	147/2332ℓ	1.13E-07	4.44E-06	2.67E-06	Cpe/Bmp4	14
GO:0034329	GO:0034329	cell junction assembly	24/374	426/2332ℓ	1.21E-07	4.70E-06	2.83E-06	Pecam1/F	24
GO:0050680	GO:0050680	negative regulation of epithelial cell proliferation	14/374	148/2332ℓ	1.23E-07	4.74E-06	2.85E-06	Sfrp1/Gkn	14
GO:0007263	GO:0007263	nitric oxide mediated signal transduction	7/374	26/23328	1.30E-07	4.94E-06	2.97E-06	Cd36/Apo	7
GO:0097205	GO:0097205	renal filtration	7/374	26/23328	1.30E-07	4.94E-06	2.97E-06	Gja5/Adgr	7
GO:0019935	GO:0019935	cyclic-nucleotide-mediated signaling	17/374	223/2332ℓ	1.33E-07	4.99E-06	3.00E-06	Aplnr/Cd3	17
GO:0070372	GO:0070372	regulation of ERK1 and ERK2 cascade	20/374	308/2332ℓ	1.47E-07	5.47E-06	3.29E-06	Bmp4/Kdr	20
GO:0001890	GO:0001890	placenta development	15/374	174/2332ℓ	1.49E-07	5.50E-06	3.31E-06	Vcam1/Ht	15
GO:0014065	GO:0014065	phosphatidylinositol 3-kinase signaling	13/374	129/2332ℓ	1.65E-07	6.05E-06	3.64E-06	Kdr/Selp/C	13
GO:0009612	GO:0009612	response to mechanical stimulus	15/374	176/2332ℓ	1.73E-07	6.18E-06	3.72E-06	Scx/Cd36	15
GO:0030194	GO:0030194	positive regulation of blood coagulation	7/374	27/23328	1.73E-07	6.18E-06	3.72E-06	Selp/Cd36	7
GO:1900048	GO:1900048	positive regulation of hemostasis	7/374	27/23328	1.73E-07	6.18E-06	3.72E-06	Selp/Cd36	7
GO:0001558	GO:0001558	regulation of cell growth	24/374	438/2332ℓ	2.01E-07	7.10E-06	4.27E-06	Sfrp1/Mgll	24
GO:0042326	GO:0042326	negative regulation of phosphorylation	25/374	471/2332ℓ	2.03E-07	7.12E-06	4.28E-06	Sfrp1/Pec	25
GO:0007159	GO:0007159	leukocyte cell-cell adhesion	21/374	345/2332ℓ	2.12E-07	7.35E-06	4.42E-06	Vcam1/Pe	21
GO:0001649	GO:0001649	osteoblast differentiation	16/374	205/2332ℓ	2.26E-07	7.79E-06	4.68E-06	Sfrp1/Bmp	16
GO:0006979	GO:0006979	response to oxidative stress	23/374	409/2332ℓ	2.29E-07	7.84E-06	4.72E-06	Bmp4/Fbln	23
GO:0007266	GO:0007266	Rho protein signal transduction	15/374	181/2332ℓ	2.49E-07	8.45E-06	5.08E-06	Pecam1/A	15
GO:0031345	GO:0031345	negative regulation of cell projection organization	16/374	207/2332ℓ	2.58E-07	8.67E-06	5.22E-06	Klk8/Apoe	16
GO:0003014	GO:0003014	renal system process	12/374	113/2332ℓ	2.79E-07	9.32E-06	5.60E-06	Bmp4/Gja	12
GO:0050730	GO:0050730	regulation of peptidyl-tyrosine phosphorylation	18/374	263/2332ℓ	2.86E-07	9.48E-06	5.70E-06	Sfrp1/Pec	18
GO:0032964	GO:0032964	collagen biosynthetic process	9/374	57/23328	2.90E-07	9.52E-06	5.73E-06	Bmp4/Scx	9
GO:0050866	GO:0050866	negative regulation of cell activation	16/374	210/2332ℓ	3.14E-07	1.02E-05	6.14E-06	Sfrp1/Bmp	16
GO:1904035	GO:1904035	regulation of epithelial cell apoptotic process	11/374	94/23328	3.27E-07	1.06E-05	6.35E-06	Bmp4/Kdr	11
GO:0070371	GO:0070371	ERK1 and ERK2 cascade	20/374	325/2332ℓ	3.47E-07	1.11E-05	6.70E-06	Bmp4/Kdr	20
GO:0009636	GO:0009636	response to toxic substance	12/374	116/2332ℓ	3.72E-07	1.19E-05	7.13E-06	Fbln5/Soc	12
GO:0001945	GO:0001945	lymph vessel development	7/374	30/23328	3.81E-07	1.20E-05	7.19E-06	Kdr/Prox1	7
GO:0050820	GO:0050820	positive regulation of coagulation	7/374	30/23328	3.81E-07	1.20E-05	7.19E-06	Selp/Cd36	7
GO:0043114	GO:0043114	regulation of vascular permeability	8/374	44/23328	4.34E-07	1.35E-05	8.12E-06	Apoe/Pde	8
GO:0048015	GO:0048015	phosphatidylinositol-mediated signaling	14/374	164/2332ℓ	4.37E-07	1.35E-05	8.12E-06	Kdr/Selp/C	14
GO:1904019	GO:1904019	epithelial cell apoptotic process	12/374	118/2332ℓ	4.49E-07	1.38E-05	8.28E-06	Bmp4/Kdr	12
GO:0070374	GO:0070374	positive regulation of ERK1 and ERK2 cascade	16/374	216/2332ℓ	4.59E-07	1.40E-05	8.40E-06	Bmp4/Kdr	16
GO:0022407	GO:0022407	regulation of cell-cell adhesion	23/374	427/2332ℓ	4.88E-07	1.47E-05	8.87E-06	Vcam1/Br	23
GO:0002683	GO:0002683	negative regulation of immune system process	24/374	462/2332ℓ	5.27E-07	1.58E-05	9.51E-06	Sfrp1/Htra	24
GO:0048017	GO:0048017	inositol lipid-mediated signaling	14/374	167/2332ℓ	5.45E-07	1.62E-05	9.77E-06	Kdr/Selp/C	14
GO:0014066	GO:0014066	regulation of phosphatidylinositol 3-kinase signaling	11/374	99/23328	5.55E-07	1.64E-05	9.87E-06	Kdr/Selp/F	11
GO:0003205	GO:0003205	cardiac chamber development	15/374	194/2332ℓ	6.09E-07	1.79E-05	1.08E-05	Cpe/Bmp4	15
GO:0030324	GO:0030324	lung development	16/374	222/2332ℓ	6.62E-07	1.93E-05	1.16E-05	Mgp/Bmp4	16
GO:0048732	GO:0048732	gland development	24/374	471/2332ℓ	7.43E-07	2.14E-05	1.28E-05	Sfrp1/Vwf	24
GO:0090287	GO:0090287	regulation of cellular response to growth factor stimulu	18/374	281/2332ℓ	7.55E-07	2.14E-05	1.28E-05	Sfrp1/Htra	18
GO:0003231	GO:0003231	cardiac ventricle development	13/374	147/2332ℓ	7.55E-07	2.14E-05	1.28E-05	Cpe/Bmp4	13
GO:0007043	GO:0007043	cell-cell junction assembly	13/374	147/2332ℓ	7.55E-07	2.14E-05	1.28E-05	Pecam1/C	13
GO:0043254	GO:0043254	regulation of protein-containing complex assembly	23/374	438/2332ℓ	7.58E-07	2.14E-05	1.28E-05	Clu/Pecar	23
GO:0030323	GO:0030323	respiratory tube development	16/374	225/2332ℓ	7.92E-07	2.22E-05	1.33E-05	Mgp/Bmp4	16
GO:0032965	GO:0032965	regulation of collagen biosynthetic process	8/374	48/23328	8.73E-07	2.43E-05	1.46E-05	Bmp4/Scx	8
GO:0050921	GO:0050921	positive regulation of chemotaxis	13/374	149/2332ℓ	8.81E-07	2.43E-05	1.46E-05	Kdr/Lbp/T	13
GO:0031348	GO:0031348	negative regulation of defense response	16/374	228/2332ℓ	9.44E-07	2.59E-05	1.56E-05	Htra1/Ptgi	16
GO:0019934	GO:0019934	cGMP-mediated signaling	7/374	34/23328	9.54E-07	2.60E-05	1.56E-05	Cd36/Edn	7
GO:0010977	GO:0010977	negative regulation of neuron projection development	14/374	176/2332ℓ	1.03E-06	2.78E-05	1.67E-05	Klk8/Apoe	14
GO:0014706	GO:0014706	striated muscle tissue development	23/374	447/2332ℓ	1.07E-06	2.89E-05	1.74E-05	Ncam1/El	23
GO:0030099	GO:0030099	myeloid cell differentiation	22/374	414/2332ℓ	1.09E-06	2.90E-05	1.75E-05	Sfrp1/Rbp	22
GO:0050819	GO:0050819	negative regulation of coagulation	8/374	50/23328	1.21E-06	3.21E-05	1.93E-05	Apoe/Proc	8
GO:0045216	GO:0045216	cell-cell junction organization	15/374	205/2332ℓ	1.22E-06	3.23E-05	1.94E-05	Pecam1/F	15
GO:0097756	GO:0097756	negative regulation of blood vessel diameter	11/374	109/2332ℓ	1.46E-06	3.84E-05	2.31E-05	Gja5/Mgll/	11
GO:0001933	GO:0001933	negative regulation of protein phosphorylation	22/374	425/2332ℓ	1.67E-06	4.36E-05	2.62E-05	Sfrp1/Pec	22
GO:0010959	GO:0010959	regulation of metal ion transport	22/374	427/2332ℓ	1.81E-06	4.68E-05	2.81E-05	Bmp4/Apl	22
GO:1903035	GO:1903035	negative regulation of response to wounding	10/374	90/23328	1.82E-06	4.69E-05	2.82E-05	Klk8/Apoe	10
GO:0001885	GO:0001885	endothelial cell development	9/374	71/23328	1.97E-06	5.04E-05	3.03E-05	Pecam1/F	9
GO:0010543	GO:0010543	regulation of platelet activation	7/374	38/23328	2.12E-06	5.38E-05	3.24E-05	Selp/Apoe	7
GO:1904705	GO:1904705	regulation of vascular associated smooth muscle cell p	8/374	54/23328	2.21E-06	5.54E-05	3.33E-05	Mmp2/Cdl	8
GO:1990874	GO:1990874	vascular associated smooth muscle cell proliferation	8/374	54/23328	2.21E-06	5.54E-05	3.33E-05	Mmp2/Cdl	8
GO:0034368	GO:0034368	protein-lipid complex remodeling	6/374	25/23328	2.23E-06	5.54E-05	3.33E-05	Gpihbp1/S	6
GO:0034369	GO:0034369	plasma lipoprotein particle remodeling	6/374	25/23328	2.23E-06	5.54E-05	3.33E-05	Gpihbp1/S	6
GO:0010038	GO:0010038	response to metal ion	17/374	274/2332ℓ	2.37E-06	5.84E-05	3.51E-05	Pam/Ncar	17
GO:0007178	GO:0007178	transmembrane receptor protein serine/threonine kinas	20/374	368/2332ℓ	2.39E-06	5.87E-05	3.53E-05	Sfrp1/Htra	20
GO:1902904	GO:1902904	negative regulation of supramolecular fiber organizatio	13/374	163/2332ℓ	2.43E-06	5.95E-05	3.56E-05	Clu/Pecar	13
GO:0014068	GO:0014068	positive regulation of phosphatidylinositol 3-kinase sig	9/374	73/23328	2.50E-06	6.06E-05	3.64E-05	Kdr/Selp/F	9
GO:2000351	GO:2000351	regulation of endothelial cell apoptotic process	8/374	55/23328	2.56E-06	6.16E-05	3.70E-05	Bmp4/Kdr	8
GO:0043433	GO:0043433	negative regulation of DNA-binding transcription factor	13/374	164/2332ℓ	2.60E-06	6.22E-05	3.74E-05	Ptgis/Prox	13
GO:0061448	GO:0061448	connective tissue development	17/374	276/2332ℓ	2.61E-06	6.22E-05	3.74E-05	Cytl1/Mgp	17
GO:0033622	GO:0033622	integrin activation	6/374	26/23328	2.86E-06	6.74E-05	4.06E-05	Selp/Fblin	6
GO:0072012	GO:0072012	glomerulus vasculature development	6/374	26/23328	2.86E-06	6.74E-05	4.06E-05	Bmp4/Tek	6
GO:0035924	GO:0035924	cellular response to vascular endothelial growth factor	8/374	56/23328	2.94E-06	6.89E-05	4.14E-05	Kdr/Flt1/N	8

GO:0045444	GO:0045444	fat cell differentiation	16/374	252/23328	3.49E-06	8.12E-05	4.88E-05	Sfrp1/Fab	16
GO:0051017	GO:0051017	actin filament bundle assembly	13/374	169/23328	3.63E-06	8.19E-05	4.92E-05	Eln/Cgnl1/	13
GO:0001570	GO:0001570	vasculogenesis	10/374	97/23328	3.63E-06	8.19E-05	4.92E-05	Aplnr/Kdr/	10
GO:0018149	GO:0018149	peptide cross-linking	6/374	27/23328	3.63E-06	8.19E-05	4.92E-05	Bgn/Thbs1	6
GO:0034367	GO:0034367	protein-containing complex remodeling	6/374	27/23328	3.63E-06	8.19E-05	4.92E-05	Gpihbp1/S	6
GO:0061437	GO:0061437	renal system vasculature development	6/374	27/23328	3.63E-06	8.19E-05	4.92E-05	Bmp4/Tek	6
GO:0061440	GO:0061440	kidney vasculature development	6/374	27/23328	3.63E-06	8.19E-05	4.92E-05	Bmp4/Tek	6
GO:0072577	GO:0072577	endothelial cell apoptotic process	8/374	58/23328	3.86E-06	8.60E-05	5.17E-05	Bmp4/Kdr	8
GO:0060541	GO:0060541	respiratory system development	16/374	254/23328	3.86E-06	8.60E-05	5.17E-05	Mgp/Bmp4	16
GO:0048762	GO:0048762	mesenchymal cell differentiation	15/374	225/23328	3.89E-06	8.62E-05	5.18E-05	Sfrp1/Bmp	15
GO:0001819	GO:0001819	positive regulation of cytokine production	22/374	449/23328	4.07E-06	8.97E-05	5.40E-05	Clu/Gata3	22
GO:0051090	GO:0051090	regulation of DNA-binding transcription factor activity	21/374	415/23328	4.11E-06	9.01E-05	5.42E-05	Cytl1/Clu/	21
GO:0007265	GO:0007265	Ras protein signal transduction	19/374	350/23328	4.35E-06	9.35E-05	5.62E-05	Pecam1/A	19
GO:0030593	GO:0030593	neutrophil chemotaxis	10/374	99/23328	4.37E-06	9.35E-05	5.62E-05	Lbp/Cxcl1	10
GO:0070167	GO:0070167	regulation of biomineral tissue development	10/374	99/23328	4.37E-06	9.35E-05	5.62E-05	Mgp/Bmp4	10
GO:0110149	GO:0110149	regulation of biomineralization	10/374	99/23328	4.37E-06	9.35E-05	5.62E-05	Mgp/Bmp4	10
GO:0019229	GO:0019229	regulation of vasoconstriction	9/374	78/23328	4.37E-06	9.35E-05	5.62E-05	Gja5/Cd38	9
GO:0061572	GO:0061572	actin filament bundle organization	13/374	172/23328	4.40E-06	9.37E-05	5.63E-05	Eln/Cgnl1/	13
GO:0051216	GO:0051216	cartilage development	14/374	200/23328	4.65E-06	9.84E-05	5.92E-05	Cytl1/Mgp	14
GO:0060562	GO:0060562	epithelial tube morphogenesis	20/374	387/23328	5.10E-06	0.00011	6.45E-05	Sfrp1/Mgp	20
GO:0052547	GO:0052547	regulation of peptidase activity	21/374	421/23328	5.13E-06	0.00011	6.46E-05	Perp/Sfrp2	21
GO:1990266	GO:1990266	neutrophil migration	11/374	124/23328	5.21E-06	0.00011	6.54E-05	Pecam1/L	11
GO:0150063	GO:0150063	visual system development	21/374	422/23328	5.32E-06	0.00011	6.63E-05	Cfh/Bmp4	21
GO:0002237	GO:0002237	response to molecule of bacterial origin	20/374	389/23328	5.50E-06	0.00011	6.83E-05	Scarb1/Cc	20
GO:0072593	GO:0072593	reactive oxygen species metabolic process	17/374	292/23328	5.55E-06	0.00011	6.85E-05	Clu/Ptgis/	17
GO:0048286	GO:0048286	lung alveolus development	8/374	61/23328	5.68E-06	0.00012	6.99E-05	Bmp4/Kdr	8
GO:0071621	GO:0071621	granulocyte chemotaxis	11/374	126/23328	6.09E-06	0.00012	7.44E-05	Lbp/Cxcl1	11
GO:0048880	GO:0048880	sensory system development	21/374	426/23328	6.14E-06	0.00012	7.48E-05	Cfh/Bmp4	21
GO:0042310	GO:0042310	vasoconstriction	10/374	103/23328	6.25E-06	0.00013	7.57E-05	Gja5/Ednr	10
GO:0032835	GO:0032835	glomerulus development	8/374	62/23328	6.44E-06	0.00013	7.76E-05	Cfh/Bmp4	8
GO:0030500	GO:0030500	regulation of bone mineralization	9/374	82/23328	6.65E-06	0.00013	7.97E-05	Mgp/Bmp4	9
GO:0001569	GO:0001569	branching involved in blood vessel morphogenesis	7/374	45/23328	6.91E-06	0.00014	8.18E-05	Kdr/Sfrp2/	7
GO:0001937	GO:0001937	negative regulation of endothelial cell proliferation	7/374	45/23328	6.91E-06	0.00014	8.18E-05	Apoe/Thbs	7
GO:0006469	GO:0006469	negative regulation of protein kinase activity	14/374	207/23328	6.91E-06	0.00014	8.18E-05	Sfrp1/Bmp	14
GO:0019216	GO:0019216	regulation of lipid metabolic process	19/374	363/23328	7.33E-06	0.00014	8.62E-05	Scarb1/Dk	19
GO:0042692	GO:0042692	muscle cell differentiation	20/374	399/23328	8.00E-06	0.00016	9.37E-05	Tmsb4x/B	20
GO:0097530	GO:0097530	granulocyte migration	12/374	155/23328	8.03E-06	0.00016	9.37E-05	Pecam1/L	12
GO:0032967	GO:0032967	positive regulation of collagen biosynthetic process	6/374	31/23328	8.56E-06	0.00016	9.91E-05	Bmp4/Scx	6
GO:0050728	GO:0050728	negative regulation of inflammatory response	12/374	156/23328	8.58E-06	0.00016	9.91E-05	Ptgis/Gata	12
GO:0032496	GO:0032496	response to lipopolysaccharide	19/374	368/23328	8.89E-06	0.00017	0.0001	Scarb1/Cc	19
GO:0007264	GO:0007264	small GTPase mediated signal transduction	21/374	437/23328	9.05E-06	0.00017	0.0001	Pecam1/C	21
GO:0046890	GO:0046890	regulation of lipid biosynthetic process	13/374	184/23328	9.19E-06	0.00017	0.0001	Scarb1/Dk	13
GO:0043277	GO:0043277	apoptotic cell clearance	7/374	47/23328	9.32E-06	0.00018	0.00011	Scarb1/Cc	7
GO:0003281	GO:0003281	ventricular septum development	9/374	86/23328	9.86E-06	0.00018	0.00011	Bmp4/Gja	9
GO:0120192	GO:0120192	tight junction assembly	9/374	86/23328	9.86E-06	0.00018	0.00011	Pecam1/It	9
GO:0042063	GO:0042063	gliogenesis	18/374	338/23328	1.01E-05	0.00019	0.00011	Clu/Bmp4	18
GO:0002791	GO:0002791	regulation of peptide secretion	20/374	406/23328	1.03E-05	0.00019	0.00012	Sfrp1/Pan	20
GO:0030195	GO:0030195	negative regulation of blood coagulation	7/374	48/23328	1.08E-05	0.0002	0.00012	Apoe/Thbs	7
GO:0031346	GO:0031346	positive regulation of cell projection organization	22/374	478/23328	1.09E-05	0.0002	0.00012	Bmp4/Fez	22
GO:0002262	GO:0002262	myeloid cell homeostasis	13/374	187/23328	1.09E-05	0.0002	0.00012	Cfh/Bmp4	13
GO:0045995	GO:0045995	regulation of embryonic development	11/374	134/23328	1.10E-05	0.0002	0.00012	Sfrp1/Bmp	11
GO:0003012	GO:0003012	muscle system process	20/374	408/23328	1.11E-05	0.0002	0.00012	Gja5/Ednr	20
GO:0010466	GO:0010466	negative regulation of peptidase activity	15/374	246/23328	1.14E-05	0.00021	0.00012	Sfrp2/Thb	15
GO:0002685	GO:0002685	regulation of leukocyte migration	14/374	217/23328	1.18E-05	0.00021	0.00013	Pecam1/S	14
GO:0061028	GO:0061028	establishment of endothelial barrier	7/374	49/23328	1.24E-05	0.00022	0.00013	Pecam1/F	7
GO:1900047	GO:1900047	negative regulation of hemostasis	7/374	49/23328	1.24E-05	0.00022	0.00013	Apoe/Thbs	7
GO:1903522	GO:1903522	regulation of blood circulation	15/374	248/23328	1.26E-05	0.00022	0.00013	Gja5/Abcc	15
GO:0031214	GO:0031214	biomineral tissue development	12/374	162/23328	1.26E-05	0.00022	0.00013	Mgp/Bmp4	12
GO:0110148	GO:0110148	biomineralization	12/374	162/23328	1.26E-05	0.00022	0.00013	Mgp/Bmp4	12
GO:0010001	GO:0010001	glial cell differentiation	15/374	249/23328	1.32E-05	0.00023	0.00014	Clu/Bmp4	15
GO:0010876	GO:0010876	lipid localization	20/374	413/23328	1.32E-05	0.00023	0.00014	Gpihbp1/S	20
GO:0033627	GO:0033627	cell adhesion mediated by integrin	8/374	69/23328	1.44E-05	0.00025	0.00015	Itgbl1/Cd2	8
GO:0045600	GO:0045600	positive regulation of fat cell differentiation	8/374	69/23328	1.44E-05	0.00025	0.00015	Sfrp1/Sfrp	8
GO:0035023	GO:0035023	regulation of Rho protein signal transduction	11/374	138/23328	1.45E-05	0.00025	0.00015	Apoe/Mcf2	11
GO:0010714	GO:0010714	positive regulation of collagen metabolic process	6/374	34/23328	1.50E-05	0.00026	0.00015	Bmp4/Scx	6
GO:0001892	GO:0001892	embryonic placenta development	10/374	114/23328	1.54E-05	0.00026	0.00016	Vcam1/Sp	10
GO:0014033	GO:0014033	neural crest cell differentiation	9/374	91/23328	1.57E-05	0.00026	0.00016	Sfrp1/Bmp	9
GO:0120193	GO:0120193	tight junction organization	9/374	91/23328	1.57E-05	0.00026	0.00016	Pecam1/It	9
GO:1903524	GO:1903524	positive regulation of blood circulation	9/374	91/23328	1.57E-05	0.00026	0.00016	Gja5/Cd38	9
GO:0001654	GO:0001654	eye development	20/374	418/23328	1.57E-05	0.00026	0.00016	Cfh/Bmp4	20
GO:0007163	GO:0007163	establishment or maintenance of cell polarity	14/374	223/23328	1.61E-05	0.00027	0.00016	Fez1/Gata	14
GO:0060412	GO:0060412	ventricular septum morphogenesis	7/374	51/23328	1.62E-05	0.00027	0.00016	Bmp4/Gja	7
GO:0098754	GO:0098754	detoxification	7/374	51/23328	1.62E-05	0.00027	0.00016	Fbln5/Sod	7
GO:0050920	GO:0050920	regulation of chemotaxis	14/374	224/23328	1.69E-05	0.00028	0.00017	Kdr/Lbp/T	14
GO:0050731	GO:0050731	positive regulation of peptidyl-tyrosine phosphorylation	13/374	195/23328	1.71E-05	0.00028	0.00017	Pecam1/C	13
GO:0048483	GO:0048483	autonomic nervous system development	7/374	52/23328	1.85E-05	0.0003	0.00018	Vcam1/Ga	7
GO:0002064	GO:0002064	epithelial cell development	15/374	257/23328	1.91E-05	0.00031	0.00019	Pecam1/E	15
GO:2001214	GO:2001214	positive regulation of vasculogenesis	4/374	11/23328	1.96E-05	0.00032	0.00019	Kdr/Adm/F	4
GO:0033673	GO:0033673	negative regulation of kinase activity	14/374	227/23328	1.96E-05	0.00032	0.00019	Sfrp1/Bmp	14
GO:0061045	GO:0061045	negative regulation of wound healing	8/374	72/23328	1.98E-05	0.00032	0.00019	Apoe/Thbs	8
GO:0043535	GO:0043535	regulation of blood vessel endothelial cell migration	9/374	94/23328	2.04E-05	0.00033	0.0002	Kdr/Apoe/	9
GO:1904707	GO:1904707	positive regulation of vascular associated smooth mus	6/374	36/23328	2.12E-05	0.00034	0.0002	Mmp2/Pdg	6
GO:0002831	GO:0002831	regulation of response to biotic stimulus	16/374	291/23328	2.12E-05	0.00034	0.0002	Htra1/Lbp	16
GO:0051348	GO:0051348	negative regulation of transferase activity	15/374	260/23328	2.19E-05	0.00035	0.00021	Sfrp1/Bmp	15
GO:0001894	GO:0001894	tissue homeostasis	15/374	261/23328	2.29E-05	0.00036	0.00022	Cytl1/Scx/	15
GO:0034113	GO:0034113	heterotypic cell-cell adhesion	7/374	54/23328	2.38E-05	0.00037	0.00023	Vcam1/Pe	7
GO:0061077	GO:0061077	chaperone-mediated protein folding	7/374	54/23328	2.38E-05	0.00037	0.00023	Clu/Unc45	7
GO:0071214	GO:0071214	cellular response to abiotic stimulus	16/374	294/23328	2.40E-05	0.00037	0.00023	Sfrp1/Scx	16
GO:0104004	GO:0104004	cellular response to environmental stimulus	16/374	294/23328	2.40E-05	0.00037	0.00023	Sfrp1/Scx	16
GO:0021700	GO:0021700	developmental maturation	17/374	327/23328	2.41E-05	0.00037	0.00023	Kdr/Gata3	17
GO:0030282	GO:0030282	bone mineralization	10/374	120/23328	2.42E-05	0.00037	0.00023	Mgp/Bmp4	10
GO:0048736	GO:0048736	appendage development	13/374	202/23328	2.48E-05	0.00038	0.00023	Bmp4/Scx	13
GO:0060173	GO:0060173	limb development	13/374	202/23328	2.48E-05	0.00038	0.00023	Bmp4/Scx	13
GO:0120162	GO:0120162	positive regulation of cold-induced thermogenesis	9/374	97/23328	2.62E-05	0.0004	0.00024	Fabp4/Cd	9
GO:0034109	GO:0034109	homotypic cell-cell adhesion	8/374	75/23328	2.68E-05	0.00041	0.00025	Cfh/Ncam	8
GO:0051145	GO:0051145	smooth muscle cell differentiation	8/374	75/23328	2.68E-05	0.00041	0.00025	Tmsb4x/B	8
GO:0006869	GO:0006869	lipid transport	18/374	364/23328	2.71E-05	0.00041	0.00025	Gpihbp1/S	18
GO:0010232	GO:0010232	vascular transport	4/374	12/23328	2.91E-05	0.00044	0.00026	Cd36/Apo	4
GO:0038084	GO:0038084	vascular endothelial growth factor signaling pathway	6/374	38/23328	2.92E-05	0.00044	0.00026	Kdr/Nrp2/I	6
GO:0045601	GO:0045601	regulation of endothelial cell differentiation	6/374	38/23328	2.92E-05	0.00044	0.00026	Bmp4/Apc	6
GO:0007611	GO:0007611	learning or memory	16/374	299/23328	2.95E-05	0.00044	0.00026	Ncam1/Kl	16
GO:0000079	GO:0000079	regulation of cyclin-dependent protein serine/threonine	8/374	76/23328	2.95E-05	0.00044	0.00026	Prox1/Rgc	8
GO:0034101	GO:0034101	erythrocyte homeostasis	11/374	149/23328	2.98E-05	0.00044	0.00027	Bmp4/Lmo	11
GO:0008406	GO:0008406	gonad development	14/374	236/23328	3.02E-05	0.00045	0.00027	Sfrp1/Bmp	14
GO:0045620	GO:0045620	negative regulation of lymphocyte differentiation	7/374	56/23328	3.04E-05	0.00045	0.00027	Sfrp1/Bmp	7
GO:0034614	GO:0034614	cellular response to reactive oxygen species	11/374	150/23328	3.17E-05	0.00047	0.00028	Fbln5/Sod	11
GO:0035265	GO:0035265	organ growth	13/374	207/23328	3.21E-05	0.00047	0.00028	Ncam1/Kc	13
GO:0003073	GO:0003073	regulation of systemic arterial blood pressure	10/374	124/23328	3.21E-05	0.00047	0.00028	Gja5/Cd36	10
GO:0071560	GO:0071560	cellular response to transforming growth factor beta sti	13/374	208/23328	3.37E-05	0.00049	0.00029	Htra1/Scx	13
GO:0071827	GO:0071827	plasma lipoprotein particle organization	6/374	39/23328	3.40E-05	0.00049	0.0003	Gpihbp1/S	6
GO:0002523	GO:0002523	leukocyte migration involved in inflammatory response	5/374	24/23328	3.41E-05	0.00049	0.0003	Lbp/Cd24	5
GO:0003170	GO:0003170	heart valve development	7/374	57/23328	3.42E-05	0.00049	0.0003	Bmp4/Scx	7
GO:0050729	GO:0050729	positive regulation of inflammatory response	11/374	152/23328	3.58E-05	0.00051	0.00031	Fabp4/Lbp	11
GO:0045137	GO:0045137	development of primary sexual characteristics	14/374	240/23328	3.63E-05	0.00052	0.00031	Sfrp1/Bmp	14
GO:0071695	GO:0071695	anatomical structure maturation	15/374	273/23328	3.85E-05	0.00055	0.00033	Kdr/Gata3	15
GO:0110053	GO:0110053	regulation of actin filament organization	15/374	273/23328	3.85E-05	0.00055	0.00033	Pecam1/T	15
GO:0040019	GO:0040019	positive regulation of embryonic development	6/374	40/23328	3.95E-05	0.00056	0.00033	Scx/Gata3	6
GO:0085029	GO:0085029	extracellular matrix assembly	6/374	40/23328	3.95E-05	0.00056	0.00033	Lox/Fbln5	6
GO:0071559	GO:0071559	response to transforming growth factor beta	13/374	212/23328	4.11E-05	0.00058	0.00035	Htra1/Scx	13
GO:0003093	GO:0003093	regulation of glomerular filtration	4/374	13/23328	4.15E-05	0.00058	0.00035	Gja5/F2r/F	4
GO:0051924	GO:0051924	regulation of calcium ion transport	15/374	275/23328	4.19E-05	0.00058	0.00035	Bmp4/Apl	15
GO:0046688	GO:0046688	response to copper ion	5/374	25/23328	4.21E-05	0.00058	0.00035	Sod3/Mt1/	5
GO:0003279	GO:0003279	cardiac septum development	10/374	128/23328	4.22E-05	0.00058	0.00035	Bmp4/Gja	10
GO:1904029	GO:1904029	regulation of cyclin-dependent protein kinase activity	8/374	80/23328	4.30E-05	0.00059	0.00036	Prox1/Rgc	8
GO:0090257	GO:0090257	regulation of muscle system process	14/374	244/23328	4.35E-05	0.0006	0.00036	Gja5/Atp1	14
GO:0034599	GO:0034599	cellular response to oxidative stress	15/374	276/23328	4.36E-05	0.0006	0.00036	Bmp4/Fbln	15
GO:0002687	GO:0002687	positive regulation of leukocyte migration	11/374	156/23328	4.55E-05	0.00062	0.00037	Pecam1/S	11
GO:0030856	GO:0030856	regulation of epithelial cell differentiation	11/374	156/23328	4.55E-05	0.00062	0.00037	Bmp4/Gat	11
GO:0071248	GO:0071248	cellular response to metal ion	11/374	156/23328	4.55E-05	0.00062	0.00037	Fabp4/Fos	11

GO:0098801	GO:0098801	regulation of renal system process	6/374	41/23328	4.57E-05	0.00062	0.00037	Gja5/Ednr	6
GO:0072678	GO:0072678	T cell migration	7/374	60/23328	4.79E-05	0.00065	0.00039	Cxcl12/S1	7
GO:1903707	GO:1903707	negative regulation of hemopoiesis	11/374	157/23328	4.82E-05	0.00065	0.00039	Sfrp1/Bmp	11
GO:0071496	GO:0071496	cellular response to external stimulus	15/374	280/23328	5.14E-05	0.00068	0.00041	Vcam1/So	15
GO:0000303	GO:0000303	response to superoxide	5/374	26/23328	5.14E-05	0.00068	0.00041	Fbln5/Sod	5
GO:0050927	GO:0050927	positive regulation of positive chemotaxis	5/374	26/23328	5.14E-05	0.00068	0.00041	Kdr/Cxcl1	5
GO:0055094	GO:0055094	response to lipoprotein particle	5/374	26/23328	5.14E-05	0.00068	0.00041	Cd36/Apo	5
GO:0055074	GO:0055074	calcium ion homeostasis	21/374	493/23328	5.33E-05	0.00071	0.00042	Bmp4/Apl	21
GO:0006816	GO:0006816	calcium ion transport	19/374	420/23328	5.46E-05	0.00072	0.00043	Bmp4/Apl	19
GO:0051899	GO:0051899	membrane depolarization	8/374	83/23328	5.61E-05	0.00074	0.00044	Gja5/Kdr/I	8
GO:0045861	GO:0045861	negative regulation of proteolysis	17/374	350/23328	5.65E-05	0.00074	0.00045	Sfrp2/Thb	17
GO:0048145	GO:0048145	regulation of fibroblast proliferation	9/374	107/23328	5.72E-05	0.00075	0.00045	Sfrp1/Nup	9
GO:0071825	GO:0071825	protein-lipid complex subunit organization	6/374	43/23328	6.03E-05	0.00079	0.00047	Gpihbp1/S	6
GO:0003151	GO:0003151	outflow tract morphogenesis	8/374	84/23328	6.11E-05	0.00079	0.00048	Bmp4/Gja	8
GO:0070830	GO:0070830	bicellular tight junction assembly	8/374	84/23328	6.11E-05	0.00079	0.00048	Pecam1/It	8
GO:0000305	GO:0000305	response to oxygen radical	5/374	27/23328	6.23E-05	0.0008	0.00048	Fbln5/Sod	5
GO:0050926	GO:0050926	regulation of positive chemotaxis	5/374	27/23328	6.23E-05	0.0008	0.00048	Kdr/Cxcl1	5
GO:0071425	GO:0071425	hematopoietic stem cell proliferation	5/374	27/23328	6.23E-05	0.0008	0.00048	Sfrp2/Cxc	5
GO:0009615	GO:0009615	response to virus	15/374	286/23328	6.53E-05	0.00084	0.0005	Htra1/Abc	15
GO:0048144	GO:0048144	fibroblast proliferation	9/374	109/23328	6.62E-05	0.00084	0.00051	Sfrp1/Nup	9
GO:0051235	GO:0051235	maintenance of location	16/374	320/23328	6.62E-05	0.00084	0.00051	Tmsb4x/S	16
GO:0051098	GO:0051098	regulation of binding	18/374	390/23328	6.63E-05	0.00084	0.00051	Spon1/Bm	18
GO:0022408	GO:0022408	negative regulation of cell-cell adhesion	12/374	192/23328	6.73E-05	0.00085	0.00051	Bmp4/Cd2	12
GO:0051048	GO:0051048	negative regulation of secretion	13/374	223/23328	6.91E-05	0.00087	0.00052	Sfrp1/Igfb	13
GO:1903531	GO:1903531	negative regulation of secretion by cell	12/374	193/23328	7.08E-05	0.00089	0.00053	Sfrp1/Igfb	12
GO:0045637	GO:0045637	regulation of myeloid cell differentiation	13/374	224/23328	7.23E-05	0.0009	0.00054	Sfrp1/Rbp	13
GO:0060411	GO:0060411	cardiac septum morphogenesis	8/374	86/23328	7.23E-05	0.0009	0.00054	Bmp4/Gja	8
GO:0097581	GO:0097581	lamellipodium organization	8/374	86/23328	7.23E-05	0.0009	0.00054	Fscn1/Itgb	8
GO:0035904	GO:0035904	aorta development	7/374	64/23328	7.29E-05	0.0009	0.00054	Lox/Prox1	7
GO:0045992	GO:0045992	negative regulation of embryonic development	5/374	28/23328	7.48E-05	0.00092	0.00055	Bmp4/Col	5
GO:0060037	GO:0060037	pharyngeal system development	5/374	28/23328	7.48E-05	0.00092	0.00055	Bmp4/Gat	5
GO:0071402	GO:0071402	cellular response to lipoprotein particle stimulus	5/374	28/23328	7.48E-05	0.00092	0.00055	Cd36/Apo	5
GO:0051147	GO:0051147	regulation of muscle cell differentiation	11/374	165/23328	7.57E-05	0.00093	0.00056	Tmsb4x/B	11
GO:0043129	GO:0043129	surfactant homeostasis	4/374	15/23328	7.72E-05	0.00094	0.00056	Kdr/Adgrf5	4
GO:0071280	GO:0071280	cellular response to copper ion	4/374	15/23328	7.72E-05	0.00094	0.00056	Mt1/Prnp/	4
GO:0150105	GO:0150105	protein localization to cell-cell junction	4/374	15/23328	7.72E-05	0.00094	0.00056	Cdh5/Actb	4
GO:0019933	GO:0019933	cAMP-mediated signaling	12/374	195/23328	7.81E-05	0.00095	0.00057	Aplnr/Pde	12
GO:0034103	GO:0034103	regulation of tissue remodeling	8/374	87/23328	7.86E-05	0.00095	0.00057	Sfrp1/Cd3	8
GO:0071902	GO:0071902	positive regulation of protein serine/threonine kinase a	15/374	291/23328	7.93E-05	0.00095	0.00057	Bmp4/C1q	15
GO:0048738	GO:0048738	cardiac muscle tissue development	14/374	258/23328	7.93E-05	0.00095	0.00057	Ncam1/Br	14
GO:0030218	GO:0030218	erythrocyte differentiation	10/374	138/23328	7.99E-05	0.00095	0.00057	Bmp4/Lmo	10
GO:0007565	GO:0007565	female pregnancy	11/374	166/23328	7.99E-05	0.00095	0.00057	Ptgis/Mmp	11
GO:0042445	GO:0042445	hormone metabolic process	12/374	196/23328	8.20E-05	0.00098	0.00059	Cpe/Rbp1	12
GO:0032271	GO:0032271	regulation of protein polymerization	13/374	227/23328	8.27E-05	0.00098	0.00059	Pecam1/T	13
GO:0044706	GO:0044706	multi-multicellular organism process	12/374	197/23328	8.61E-05	0.00102	0.00061	Ptgis/Mmp	12
GO:0003254	GO:0003254	regulation of membrane depolarization	6/374	46/23328	8.89E-05	0.00104	0.00063	Gja5/Kdr/I	6
GO:0097006	GO:0097006	regulation of plasma lipoprotein particle levels	7/374	66/23328	8.89E-05	0.00104	0.00063	Gpihbp1/S	7
GO:0001516	GO:0001516	prostaglandin biosynthetic process	5/374	29/23328	8.92E-05	0.00104	0.00063	Ptgis/Ptgs	5
GO:0046457	GO:0046457	prostanoid biosynthetic process	5/374	29/23328	8.92E-05	0.00104	0.00063	Ptgis/Ptgs	5
GO:0050708	GO:0050708	regulation of protein secretion	17/374	365/23328	9.43E-05	0.0011	0.00066	Sfrp1/Pam	17
GO:0007204	GO:0007204	positive regulation of cytosolic calcium ion concentratio	16/374	330/23328	9.49E-05	0.0011	0.00066	Bmp4/Apl	16
GO:0048872	GO:0048872	homeostasis of number of cells	16/374	330/23328	9.49E-05	0.0011	0.00066	Cfh/Bmp4	16
GO:0006874	GO:0006874	cellular calcium ion homeostasis	20/374	476/23328	9.65E-05	0.00112	0.00067	Bmp4/Apl	20
GO:0007044	GO:0007044	cell-substrate junction assembly	8/374	90/23328	1.00E-04	0.00115	0.00069	Kdr/Tek/F	8
GO:0003179	GO:0003179	heart valve morphogenesis	6/374	47/23328	0.000100563	0.00115	0.00069	Bmp4/Scx	6
GO:0002697	GO:0002697	regulation of immune effector process	19/374	440/23328	0.000100817	0.00115	0.00069	Cfh/Htra1/	19
GO:0045860	GO:0045860	positive regulation of protein kinase activity	19/374	440/23328	0.000100817	0.00115	0.00069	Clu/Bmp4	19
GO:0045926	GO:0045926	negative regulation of growth	14/374	264/23328	0.000101248	0.00115	0.00069	Sfrp1/Bmp	14
GO:0001977	GO:0001977	renal system process involved in regulation of blood ve	4/374	16/23328	0.000101577	0.00115	0.00069	Gja5/F2r/F	4
GO:0071404	GO:0071404	cellular response to low-density lipoprotein particle stim	4/374	16/23328	0.000101577	0.00115	0.00069	Cd36/Lpl/	4
GO:1902430	GO:1902430	negative regulation of amyloid-beta formation	4/374	16/23328	0.000101577	0.00115	0.00069	Clu/Spon1	4
GO:2001212	GO:2001212	regulation of vasculogenesis	4/374	16/23328	0.000101577	0.00115	0.00069	Kdr/Adm/F	4
GO:0062197	GO:0062197	cellular response to chemical stress	16/374	332/23328	0.000101735	0.00115	0.00069	Bmp4/Fbln	16
GO:0051494	GO:0051494	negative regulation of cytoskeleton organization	11/374	171/23328	0.000104181	0.00117	0.0007	Pecam1/T	11
GO:0030947	GO:0030947	regulation of vascular endothelial growth factor recepto	5/374	30/23328	0.000105644	0.00118	0.00071	Bmp4/Kdr	5
GO:0051346	GO:0051346	negative regulation of hydrolase activity	18/374	405/23328	0.000106813	0.00119	0.00072	Pecam1/S	18
GO:0002792	GO:0002792	negative regulation of peptide secretion	9/374	116/23328	0.00010728	0.0012	0.00072	Sfrp1/Apo	9
GO:0007566	GO:0007566	embryo implantation	7/374	68/23328	0.000107693	0.0012	0.00072	Ptgis/Mmp	7
GO:0072091	GO:0072091	regulation of stem cell proliferation	8/374	91/23328	0.000108099	0.0012	0.00072	Bmp4/Cxc	8
GO:0043270	GO:0043270	positive regulation of ion transport	16/374	334/23328	0.000109053	0.00121	0.00072	Bmp4/Apl	16
GO:0000302	GO:0000302	response to reactive oxygen species	12/374	202/23328	0.000109258	0.00121	0.00072	Fbln5/Sod	12
GO:0043010	GO:0043010	camera-type eye development	17/374	370/23328	0.000111096	0.00122	0.00073	Cfh/Bmp4	17
GO:0045907	GO:0045907	positive regulation of vasoconstriction	6/374	48/23328	0.000113392	0.00124	0.00075	Gja5/Cd38	6
GO:0072089	GO:0072089	stem cell proliferation	10/374	144/23328	0.000113908	0.00124	0.00075	Bmp4/Sfrp	10
GO:0106106	GO:0106106	cold-induced thermogenesis	10/374	144/23328	0.000113908	0.00124	0.00075	Fabp4/Cd	10
GO:0120161	GO:0120161	regulation of cold-induced thermogenesis	10/374	144/23328	0.000113908	0.00124	0.00075	Fabp4/Cd	10
GO:0071901	GO:0071901	negative regulation of protein serine/threonine kinase a	9/374	117/23328	0.000114605	0.00124	0.00075	Sfrp1/Bmp	9
GO:0043405	GO:0043405	regulation of MAP kinase activity	15/374	301/23328	0.000115441	0.00125	0.00075	Sfrp1/Bmp	15
GO:0050890	GO:0050890	cognition	16/374	336/23328	0.000116828	0.00126	0.00076	Ncam1/Kl	16
GO:0006631	GO:0006631	fatty acid metabolic process	18/374	408/23328	0.000117135	0.00126	0.00076	Pam/Adh7	18
GO:0048146	GO:0048146	positive regulation of fibroblast proliferation	7/374	69/23328	0.000118222	0.00127	0.00076	Fn1/Pdgfa	7
GO:0010976	GO:0010976	positive regulation of neuron projection development	17/374	372/23328	0.000118529	0.00127	0.00076	Bmp4/Fez	17
GO:0048469	GO:0048469	cell maturation	12/374	205/23328	0.000125566	0.00134	0.00081	Kdr/Gata3	12
GO:0043297	GO:0043297	apical junction assembly	8/374	93/23328	0.000125995	0.00134	0.00081	Pecam1/It	8
GO:0150115	GO:0150115	cell-substrate junction organization	8/374	93/23328	0.000125995	0.00134	0.00081	Kdr/Tek/F	8
GO:0006692	GO:0006692	prostanoid metabolic process	6/374	49/23328	0.000127486	0.00134	0.00081	Ptgis/Ptgs	6
GO:0006693	GO:0006693	prostaglandin metabolic process	6/374	49/23328	0.000127486	0.00134	0.00081	Ptgis/Ptgs	6
GO:0019233	GO:0019233	sensory perception of pain	10/374	146/23328	0.00012769	0.00134	0.00081	Ncam1/Mc	10
GO:0045598	GO:0045598	regulation of fat cell differentiation	10/374	146/23328	0.00012769	0.00134	0.00081	Sfrp1/Gata	10
GO:0051480	GO:0051480	regulation of cytosolic calcium ion concentration	17/374	375/23328	0.000130495	0.00137	0.00082	Bmp4/Apl	17
GO:0033623	GO:0033623	regulation of integrin activation	4/374	17/23328	0.000131156	0.00137	0.00082	Selp/Fbln	4
GO:0042415	GO:0042415	norepinephrine metabolic process	4/374	17/23328	0.000131156	0.00137	0.00082	Gata3/Ly6	4
GO:0048875	GO:0048875	chemical homeostasis within a tissue	4/374	17/23328	0.000131156	0.00137	0.00082	Kdr/Adgrf5	4
GO:0051056	GO:0051056	regulation of small GTPase mediated signal transducti	14/374	271/23328	0.000133308	0.00139	0.00083	Cgnl1/Apo	14
GO:0007369	GO:0007369	gastrulation	11/374	176/23328	0.000134526	0.0014	0.00084	Sfrp1/Bmp	11
GO:0015908	GO:0015908	fatty acid transport	8/374	94/23328	0.00013582	0.00141	0.00085	Fabp4/Slc	8
GO:0051607	GO:0051607	defense response to virus	13/374	239/23328	0.000138519	0.00143	0.00086	Htra1/Abc	13
GO:0035051	GO:0035051	cardiocyte differentiation	11/374	177/23328	0.000141421	0.00146	0.00088	Vcam1/Br	11
GO:0001893	GO:0001893	maternal placenta development	5/374	32/23328	0.000145412	0.00148	0.00089	Ptgis/Nr2f	5
GO:0010043	GO:0010043	response to zinc ion	5/374	32/23328	0.000145412	0.00148	0.00089	Pam/Ass1	5
GO:0060669	GO:0060669	embryonic placenta morphogenesis	5/374	32/23328	0.000145412	0.00148	0.00089	Vcam1/Sp	5
GO:2000352	GO:2000352	negative regulation of endothelial cell apoptotic proces	5/374	32/23328	0.000145412	0.00148	0.00089	Kdr/Gata3	5
GO:0071772	GO:0071772	response to BMP	11/374	178/23328	0.000148615	0.00151	0.00091	Sfrp1/Htra	11
GO:0071773	GO:0071773	cellular response to BMP stimulus	11/374	178/23328	0.000148615	0.00151	0.00091	Sfrp1/Htra	11
GO:0098742	GO:0098742	cell-cell adhesion via plasma-membrane adhesion mol	12/374	209/23328	0.000150525	0.00152	0.00092	Vcam1/Pe	12
GO:1902105	GO:1902105	regulation of leukocyte differentiation	15/374	309/23328	0.000153914	0.00155	0.00093	Sfrp1/Rbp	15
GO:1903037	GO:1903037	regulation of leukocyte cell-cell adhesion	15/374	309/23328	0.000153914	0.00155	0.00093	Vcam1/Br	15
GO:0010633	GO:0010633	negative regulation of epithelial cell migration	7/374	72/23328	0.000154924	0.00155	0.00093	Apoe/Thbs	7
GO:0045123	GO:0045123	cellular extravasation	7/374	72/23328	0.000154924	0.00155	0.00093	Vcam1/Pe	7
GO:2000027	GO:2000027	regulation of animal organ morphogenesis	12/374	210/23328	0.000157389	0.00157	0.00095	Sfrp1/Bmp	12
GO:0031099	GO:0031099	regeneration	10/374	150/23328	0.00015951	0.00159	0.00096	Klk8/Cxcl1	10
GO:0052548	GO:0052548	regulation of endopeptidase activity	16/374	346/23328	0.000163417	0.00163	0.00098	Perp/Sfrp2	16
GO:0090596	GO:0090596	sensory organ morphogenesis	15/374	311/23328	0.000165115	0.00164	0.00099	Cfh/Bmp4	15
GO:0045603	GO:0045603	positive regulation of endothelial cell differentiation	4/374	18/23328	0.000166503	0.00164	0.00099	Bmp4/Cdh	4
GO:1902992	GO:1902992	negative regulation of amyloid precursor protein catabo	4/374	18/23328	0.000166503	0.00164	0.00099	Clu/Spon1	4
GO:1903019	GO:1903019	negative regulation of glycoprotein metabolic process	4/374	18/23328	0.000166503	0.00164	0.00099	Bace2/Cst	4
GO:0001659	GO:0001659	temperature homeostasis	11/374	181/23328	0.000172089	0.00169	0.00102	Fabp4/Cd	11
GO:1904036	GO:1904036	negative regulation of epithelial cell apoptotic process	6/374	52/23328	0.000178258	0.00175	0.00105	Kdr/Gata3	6
GO:0048592	GO:0048592	eye morphogenesis	11/374	182/23328	0.000180582	0.00177	0.00106	Cfh/Bmp4	11
GO:0030279	GO:0030279	negative regulation of ossification	8/374	98/23328	0.00018166	0.00177	0.00106	Sfrp1/Id1/	8
GO:0030510	GO:0030510	regulation of BMP signaling pathway	8/374	98/23328	0.00018166	0.00177	0.00106	Sfrp1/Htra	8
GO:0043583	GO:0043583	ear development	13/374	246/23328	0.000184159	0.00179	0.00108	Cytl1/Bmp	13
GO:0015711	GO:0015711	organic anion transport	19/374	463/23328	0.000193964	0.00188	0.00113	Scarb1/Fa	19
GO:0043502	GO:0043502	regulation of muscle adaptation	8/374	99/23328	0.000194912	0.00189	0.00113	Rgs4/Nos	8
GO:0002833	GO:0002833	positive regulation of response to biotic stimulus	10/374	154/23328	0.000197763	0.0019	0.00115	Lbp/Cxcl1	10
GO:0022612	GO:0022612	gland morphogenesis	10/374	154/23328	0.000197763	0.0019	0.00115	Sfrp1/Bmp	10
GO:0010596	GO:0010596	negative regulation of endothelial cell migration	6/374	53/23328	0.000198329	0.00191	0.00115	Apoe/Thbs	6
GO:0014743	GO:0014743	regulation of muscle hypertrophy	7/374	75/23328	0.000200349	0.00191	0.00115	Rgs4/Lmc	7
GO:0051057	GO:0051057	positive regulation of small GTPase mediated signal tr	7/374	75/23328	0.000200349	0.00191	0.00115	Kitl/Mcf2l/	7

GO:0097755	GO:0097755	positive regulation of blood vessel diameter	7/374	75/23328	0.000200349	0.00191	0.00115	Gja5/Kdr/I	7
GO:1903670	GO:1903670	regulation of sprouting angiogenesis	7/374	75/23328	0.000200349	0.00191	0.00115	Aplnr/Kdr/	7
GO:0001889	GO:0001889	liver development	9/374	126/23328	0.000201472	0.00191	0.00115	Vwf/Bmp4	9
GO:0015850	GO:0015850	organic hydroxy compound transport	13/374	249/23328	0.000207357	0.00196	0.00118	Scarb1/Co	13
GO:0010544	GO:0010544	negative regulation of platelet activation	4/374	19/23328	0.000208246	0.00197	0.00118	Apoe/Pdg	4
GO:0008277	GO:0008277	regulation of G protein-coupled receptor signaling path	9/374	127/23328	0.000213829	0.00202	0.00121	Mgll/Aplnr	9
GO:0070838	GO:0070838	divalent metal ion transport	19/374	467/23328	0.000216237	0.00203	0.00122	Bmp4/Apl	19
GO:0060070	GO:0060070	canonical Wnt signaling pathway	14/374	284/23328	0.000216588	0.00203	0.00122	Sfrp1/Dkk	14
GO:0046456	GO:0046456	icosanoid biosynthetic process	6/374	54/23328	0.00022014	0.00206	0.00124	Ptgis/Pla2	6
GO:0035025	GO:0035025	positive regulation of Rho protein signal transduction	5/374	35/23328	0.000225354	0.0021	0.00126	Mcf2l/F2r/	5
GO:0046686	GO:0046686	response to cadmium ion	5/374	35/23328	0.000225354	0.0021	0.00126	Fos/Mt1/P	5
GO:0072511	GO:0072511	divalent inorganic cation transport	19/374	470/23328	0.000234382	0.00218	0.00131	Bmp4/Apl	19
GO:0006809	GO:0006809	nitric oxide biosynthetic process	7/374	77/23328	0.000236195	0.00219	0.00132	Clu/Ptgis/	7
GO:0001704	GO:0001704	formation of primary germ layer	8/374	102/23328	0.000239513	0.00222	0.00133	Bmp4/Scx	8
GO:0006690	GO:0006690	icosanoid metabolic process	9/374	129/23328	0.000240438	0.00222	0.00133	Ptgis/Mgll	9
GO:0061008	GO:0061008	hepaticobiliary system development	9/374	129/23328	0.000240438	0.00222	0.00133	Vwf/Bmp4	9
GO:0002042	GO:0002042	cell migration involved in sprouting angiogenesis	6/374	55/23328	0.000243797	0.00224	0.00135	Kdr/Thbs1	6
GO:0045666	GO:0045666	positive regulation of neuron differentiation	19/374	472/23328	0.000247208	0.00227	0.00137	Bmp4/Fez	19
GO:0002695	GO:0002695	negative regulation of leukocyte activation	11/374	189/23328	0.000250587	0.0023	0.00138	Sfrp1/Bmp	11
GO:0003208	GO:0003208	cardiac ventricle morphogenesis	7/374	78/23328	0.00025596	0.00232	0.0014	Cpe/Gata	7
GO:0072676	GO:0072676	lymphocyte migration	8/374	103/23328	0.000256114	0.00232	0.0014	Gata3/Cxc	8
GO:1990845	GO:1990845	adaptive thermogenesis	10/374	159/23328	0.000256152	0.00232	0.0014	Fabp4/Cd	10
GO:0001946	GO:0001946	lymphangiogenesis	4/374	20/23328	0.00025703	0.00232	0.0014	Prox1/Flt4	4
GO:0035584	GO:0035584	calcium-mediated signaling using intracellular calcium	4/374	20/23328	0.00025703	0.00232	0.0014	Vcam1/Kc	4
GO:2000136	GO:2000136	regulation of cell proliferation involved in heart morpho	4/374	20/23328	0.00025703	0.00232	0.0014	Bmp4/Pim	4
GO:0035909	GO:0035909	aorta morphogenesis	5/374	36/23328	0.000258289	0.00233	0.0014	Prox1/Sox	5
GO:0001954	GO:0001954	positive regulation of cell-matrix adhesion	6/374	56/23328	0.000269408	0.00241	0.00145	Kdr/Cd36/	6
GO:0030199	GO:0030199	collagen fibril organization	6/374	56/23328	0.000269408	0.00241	0.00145	Scx/Lox/C	6
GO:0050873	GO:0050873	brown fat cell differentiation	6/374	56/23328	0.000269408	0.00241	0.00145	Fabp4/Se	6
GO:0051930	GO:0051930	regulation of sensory perception of pain	6/374	56/23328	0.000269408	0.00241	0.00145	Ncam1/M	6
GO:0050886	GO:0050886	endocrine process	8/374	104/23328	0.000273645	0.00244	0.00147	Gja5/Gata	8
GO:0045619	GO:0045619	regulation of lymphocyte differentiation	11/374	192/23328	0.000286944	0.00256	0.00154	Sfrp1/Bmp	11
GO:0045165	GO:0045165	cell fate commitment	14/374	292/23328	0.000287514	0.00256	0.00154	Sfrp1/Bmp	14
GO:1905954	GO:1905954	positive regulation of lipid localization	8/374	105/23328	0.000292145	0.00259	0.00156	Scarb1/Co	8
GO:1990748	GO:1990748	cellular detoxification	5/374	37/23328	0.000294753	0.00261	0.00157	Fbln5/Sod	5
GO:0043536	GO:0043536	positive regulation of blood vessel endothelial cell mig	6/374	57/23328	0.000297085	0.00262	0.00157	Kdr/Thbs1	6
GO:0051931	GO:0051931	regulation of sensory perception	6/374	57/23328	0.000297085	0.00262	0.00157	Ncam1/M	6
GO:0035282	GO:0035282	segmentation	8/374	106/23328	0.000311654	0.00273	0.00164	Sfrp1/Bmp	8
GO:0002043	GO:0002043	blood vessel endothelial cell proliferation involved in s	4/374	21/23328	0.000313511	0.00273	0.00164	Bmp4/Apl	4
GO:0019430	GO:0019430	removal of superoxide radicals	4/374	21/23328	0.000313511	0.00273	0.00164	Fbln5/Sod	4
GO:0046697	GO:0046697	decidualization	4/374	21/23328	0.000313511	0.00273	0.00164	Ptgis/Junb	4
GO:0061323	GO:0061323	cell proliferation involved in heart morphogenesis	4/374	21/23328	0.000313511	0.00273	0.00164	Bmp4/Pim	4
GO:1903038	GO:1903038	negative regulation of leukocyte cell-cell adhesion	9/374	134/23328	0.000319185	0.00278	0.00167	Bmp4/Cd2	9
GO:0071241	GO:0071241	cellular response to inorganic substance	12/374	227/23328	0.00032223	0.0028	0.00168	Fabp4/Fo	12
GO:0046209	GO:0046209	nitric oxide metabolic process	7/374	81/23328	0.000323363	0.0028	0.00169	Clu/Ptgis/	7
GO:0050709	GO:0050709	negative regulation of protein secretion	8/374	107/23328	0.000332213	0.00287	0.00172	Sfrp1/Apo	8
GO:0110020	GO:0110020	regulation of actomyosin structure organization	8/374	107/23328	0.000332213	0.00287	0.00172	Cgnl1/Pro	8
GO:2000249	GO:2000249	regulation of actin cytoskeleton reorganization	5/374	38/23328	0.00033499	0.00289	0.00174	Tek/Arhgc	5
GO:1905952	GO:1905952	regulation of lipid localization	10/374	165/23328	0.000344637	0.00296	0.00178	Scarb1/Co	10
GO:0019915	GO:0019915	lipid storage	7/374	82/23328	0.00034875	0.00299	0.0018	Scarb1/Co	7
GO:0016485	GO:0016485	protein processing	12/374	229/23328	0.000348876	0.00299	0.0018	Cpe/Perp/	12
GO:0034754	GO:0034754	cellular hormone metabolic process	8/374	108/23328	0.000353865	0.00302	0.00182	Rbp1/Adh	8
GO:0055123	GO:0055123	digestive system development	9/374	136/23328	0.000356138	0.00304	0.00183	Sfrp1/Bmp	9
GO:0048638	GO:0048638	regulation of developmental growth	17/374	409/23328	0.000360161	0.00306	0.00184	Sfrp1/Nca	17
GO:0032272	GO:0032272	negative regulation of protein polymerization	7/374	83/23328	0.000375708	0.00318	0.00191	Pecam1/T	7
GO:0002062	GO:0002062	chondrocyte differentiation	8/374	109/23328	0.000376652	0.00318	0.00191	Cytl1/Bmp	8
GO:0043393	GO:0043393	regulation of protein binding	12/374	231/23328	0.000377365	0.00318	0.00191	Spon1/Bm	12
GO:0022409	GO:0022409	positive regulation of cell-cell adhesion	13/374	265/23328	0.000378052	0.00318	0.00191	Vcam1/Se	13
GO:0002504	GO:0002504	antigen processing and presentation of peptide or poly	4/374	22/23328	0.00037836	0.00318	0.00191	Thbs1/H2	4
GO:2001026	GO:2001026	regulation of endothelial cell chemotaxis	4/374	22/23328	0.00037836	0.00318	0.00191	Kdr/Thbs1	4
GO:0030509	GO:0030509	BMP signaling pathway	10/374	167/23328	0.000379271	0.00318	0.00191	Sfrp1/Htra	10
GO:0030308	GO:0030308	negative regulation of cell growth	11/374	199/23328	0.000389396	0.00326	0.00196	Sfrp1/Fhl1	11
GO:0060711	GO:0060711	labyrinthine layer development	6/374	60/23328	0.000393684	0.00329	0.00198	Vcam1/Sp	6
GO:0045089	GO:0045089	positive regulation of innate immune response	8/374	110/23328	0.000400619	0.00334	0.00201	Lbp/Mmp2	8
GO:2001233	GO:2001233	regulation of apoptotic signaling pathway	17/374	413/23328	0.00040245	0.00335	0.00201	Sfrp1/Clu/	17
GO:0014032	GO:0014032	neural crest cell development	7/374	84/23328	0.000404308	0.00335	0.00202	Bmp4/Edr	7
GO:0051384	GO:0051384	response to glucocorticoid	7/374	84/23328	0.000404308	0.00335	0.00202	Bmp4/Ass	7
GO:0051099	GO:0051099	positive regulation of binding	11/374	200/23328	0.000406272	0.00336	0.00202	Spon1/Bm	11
GO:0097191	GO:0097191	extrinsic apoptotic signaling pathway	12/374	233/23328	0.000407797	0.00337	0.00202	Sfrp1/Bmp	12
GO:0007179	GO:0007179	transforming growth factor beta receptor signaling path	10/374	169/23328	0.000416756	0.00343	0.00207	Htra1/Lox	10
GO:0008584	GO:0008584	male gonad development	9/374	139/23328	0.000418131	0.00344	0.00207	Sfrp1/Kdr/	9
GO:0008630	GO:0008630	intrinsic apoptotic signaling pathway in response to DN	8/374	111/23328	0.000425812	0.00349	0.0021	Clu/Nupr1	8
GO:1904645	GO:1904645	response to amyloid-beta	5/374	40/23328	0.000427782	0.0035	0.00211	Vcam1/Co	5
GO:0046546	GO:0046546	development of primary male sexual characteristics	9/374	140/23328	0.000440668	0.0036	0.00217	Sfrp1/Kdr/	9
GO:0034370	GO:0034370	triglyceride-rich lipoprotein particle remodeling	3/374	10/23328	0.000451159	0.00361	0.00217	Gpihbp1/A	3
GO:0042421	GO:0042421	norepinephrine biosynthetic process	3/374	10/23328	0.000451159	0.00361	0.00217	Gata3/Epa	3
GO:0048251	GO:0048251	elastic fiber assembly	3/374	10/23328	0.000451159	0.00361	0.00217	Lox/Fbln5	3
GO:0061626	GO:0061626	pharyngeal arch artery morphogenesis	3/374	10/23328	0.000451159	0.00361	0.00217	Bmp4/Adc	3
GO:1902033	GO:1902033	regulation of hematopoietic stem cell proliferation	3/374	10/23328	0.000451159	0.00361	0.00217	Cxcl1/Kitl/	3
GO:0002320	GO:0002320	lymphoid progenitor cell differentiation	4/374	23/23328	0.000452256	0.00361	0.00217	Bmp4/Gat	4
GO:0010743	GO:0010743	regulation of macrophage derived foam cell differentiat	4/374	23/23328	0.000452256	0.00361	0.00217	Cd36/Lpl/	4
GO:0036303	GO:0036303	lymph vessel morphogenesis	4/374	23/23328	0.000452256	0.00361	0.00217	Prox1/Flt4	4
GO:0042572	GO:0042572	retinol metabolic process	4/374	23/23328	0.000452256	0.00361	0.00217	Rbp1/Adh	4
GO:0048485	GO:0048485	sympathetic nervous system development	4/374	23/23328	0.000452256	0.00361	0.00217	Gata3/Sox	4
GO:0071276	GO:0071276	cellular response to cadmium ion	4/374	23/23328	0.000452256	0.00361	0.00217	Fos/Mt1/M	4
GO:0071450	GO:0071450	cellular response to oxygen radical	4/374	23/23328	0.000452256	0.00361	0.00217	Fbln5/Sod	4
GO:0071451	GO:0071451	cellular response to superoxide	4/374	23/23328	0.000452256	0.00361	0.00217	Fbln5/Sod	4
GO:0048708	GO:0048708	astrocyte differentiation	7/374	86/23328	0.000466723	0.00371	0.00223	Vim/Plpp3	7
GO:2001057	GO:2001057	reactive nitrogen species metabolic process	7/374	86/23328	0.000466723	0.00371	0.00223	Clu/Ptgis/	7
GO:0031102	GO:0031102	neuron projection regeneration	6/374	62/23328	0.000470615	0.00373	0.00224	Klk8/Mmp	6
GO:1901615	GO:1901615	organic hydroxy compound metabolic process	19/374	498/23328	0.00047904	0.00379	0.00228	Rbp1/Adh	19
GO:0055001	GO:0055001	muscle cell development	11/374	205/23328	0.000500146	0.00394	0.00237	Bmp4/Lox	11
GO:0060420	GO:0060420	regulation of heart growth	7/374	87/23328	0.000500687	0.00394	0.00237	Ncam1/Pr	7
GO:0002699	GO:0002699	positive regulation of immune effector process	13/374	273/23328	0.000500904	0.00394	0.00237	Gata3/Cd	13
GO:2000379	GO:2000379	positive regulation of reactive oxygen species metabol	8/374	114/23328	0.000509211	0.004	0.0024	Clu/Cd36/	8
GO:0071260	GO:0071260	cellular response to mechanical stimulus	6/374	63/23328	0.000513225	0.00402	0.00242	Scx/Nos3/	6
GO:0060249	GO:0060249	anatomical structure homeostasis	17/374	422/23328	0.000513574	0.00402	0.00242	Cytl1/Scx	17
GO:0030010	GO:0030010	establishment of cell polarity	9/374	143/23328	0.000514351	0.00402	0.00242	Fez1/Gata	9
GO:0015718	GO:0015718	monocarboxylic acid transport	10/374	174/23328	0.000524142	0.00408	0.00246	Fabp4/Slc	10
GO:0046578	GO:0046578	regulation of Ras protein signal transduction	12/374	240/23328	0.000531155	0.00413	0.00248	Apoe/Kitl/	12
GO:0098869	GO:0098869	cellular oxidant detoxification	4/374	24/23328	0.000535887	0.00415	0.0025	Fbln5/Sod	4
GO:0031960	GO:0031960	response to corticosteroid	7/374	88/23328	0.00053659	0.00415	0.0025	Bmp4/Ass	7
GO:0046683	GO:0046683	response to organophosphorus	7/374	88/23328	0.00053659	0.00415	0.0025	Abcc9/Pd	7
GO:0031032	GO:0031032	actomyosin structure organization	11/374	207/23328	0.000542461	0.00418	0.00251	Eln/Cgnl1	11
GO:2000377	GO:2000377	regulation of reactive oxygen species metabolic proces	11/374	207/23328	0.000542461	0.00418	0.00251	Clu/Ptgis/	11
GO:0045927	GO:0045927	positive regulation of growth	14/374	312/23328	0.000557181	0.00429	0.00258	Sfrp1/Nca	14
GO:0015909	GO:0015909	long-chain fatty acid transport	6/374	64/23328	0.000558776	0.00429	0.00258	Fabp4/Cd	6
GO:0019882	GO:0019882	antigen processing and presentation	8/374	116/23328	0.000571817	0.00437	0.00263	Thbs1/H2	8
GO:0001656	GO:0001656	metanephros development	7/374	89/23328	0.000574513	0.00437	0.00263	Bmp4/Gat	7
GO:0014031	GO:0014031	mesenchymal cell development	7/374	89/23328	0.000574513	0.00437	0.00263	Bmp4/Edr	7
GO:0051149	GO:0051149	positive regulation of muscle cell differentiation	7/374	89/23328	0.000574513	0.00437	0.00263	Tmsb4x/B	7
GO:0071674	GO:0071674	mononuclear cell migration	7/374	89/23328	0.000574513	0.00437	0.00263	Pecam1/T	7
GO:0007548	GO:0007548	sex differentiation	14/374	315/23328	0.000612035	0.00461	0.00277	Sfrp1/Bmp	14
GO:0002468	GO:0002468	dendritic cell antigen processing and presentation	3/374	11/23328	0.000612983	0.00461	0.00277	Thbs1/Fgl	3
GO:0060346	GO:0060346	bone trabecula formation	3/374	11/23328	0.000612983	0.00461	0.00277	Sfrp1/Mm	3
GO:0061309	GO:0061309	cardiac neural crest cell development involved in outfl	3/374	11/23328	0.000612983	0.00461	0.00277	Bmp4/Hes	3
GO:0097084	GO:0097084	vascular associated smooth muscle cell development	3/374	11/23328	0.000612983	0.00461	0.00277	Adm/Hes1	3
GO:0150104	GO:0150104	transport across blood-brain barrier	3/374	11/23328	0.000612983	0.00461	0.00277	Cd36/Apo	3
GO:0048864	GO:0048864	stem cell development	7/374	90/23328	0.000614534	0.00462	0.00278	Bmp4/Edr	7
GO:0045667	GO:0045667	regulation of osteoblast differentiation	9/374	147/23328	0.000628031	0.0047	0.00283	Sfrp1/Bmp	9
GO:0010884	GO:0010884	positive regulation of lipid storage	4/374	25/23328	0.000629947	0.0047	0.00283	Scarb1/Co	4
GO:0032703	GO:0032703	negative regulation of interleukin-2 production	4/374	25/23328	0.000629947	0.0047	0.00283	Gata3/Prn	4
GO:0032878	GO:0032878	regulation of establishment or maintenance of cell pola	4/374	25/23328	0.000629947	0.0047	0.00283	Gata3/Tek	4
GO:0021782	GO:0021782	glial cell development	8/374	118/23328	0.000640503	0.00476	0.00286	Clu/Sox4/	8
GO:0060419	GO:0060419	heart growth	8/374	118/23328	0.000640503	0.00476	0.00286	Ncam1/Pr	8
GO:0002088	GO:0002088	lens development in camera-type eye	7/374	91/23328	0.000656737	0.00485	0.00292	Bmp4/Gat	7
GO:0042632	GO:0042632	cholesterol homeostasis	7/374	91/23328	0.000656737	0.00485	0.00292	Gpihbp1/S	7
GO:0048644	GO:0048644	muscle organ morphogenesis	7/374	91/23328	0.000656737	0.00485	0.00292	Prox1/Tcf	7
GO:0042982	GO:0042982	amyloid precursor protein metabolic process	6/374	66/23328	0.000659255	0.00486	0.00293	Clu/Spon1	6

GO:0071385	GO:0071385	cellular response to glucocorticoid stimulus	5/374	44/23328	0.000670004	0.00492	0.00296	Bmp4/Zfp	5
GO:0097237	GO:0097237	cellular response to toxic substance	5/374	44/23328	0.000670004	0.00492	0.00296	Fbln5/Sod	5
GO:1902895	GO:1902895	positive regulation of pri-miRNA transcription by RNA p	5/374	44/23328	0.000670004	0.00492	0.00296	Bmp4/Gat	5
GO:0001822	GO:0001822	kidney development	14/374	318/2332ɛ	0.000671406	0.00492	0.00296	Sfrp1/Cfh/	14
GO:0001952	GO:0001952	regulation of cell-matrix adhesion	8/374	119/2332ɛ	0.000677259	0.00494	0.00297	Kdr/Cd36/	8
GO:0042476	GO:0042476	odontogenesis	8/374	119/2332ɛ	0.000677259	0.00494	0.00297	Perp/Bmp	8
GO:0090092	GO:0090092	regulation of transmembrane receptor protein serine/th	12/374	247/2332ɛ	0.000684512	0.00499	0.003	Sfrp1/Htra	12
GO:0055092	GO:0055092	sterol homeostasis	7/374	92/23328	0.000701205	0.0051	0.00307	Gpihbp1/S	7
GO:0045088	GO:0045088	regulation of innate immune response	10/374	181/2332ɛ	0.000712148	0.00517	0.00311	Lbp/Apoe/	10
GO:1901570	GO:1901570	fatty acid derivative biosynthetic process	6/374	67/23328	0.000714466	0.00517	0.00311	Ptgis/Pla2	6
GO:0019722	GO:0019722	calcium-mediated signaling	11/374	214/2332ɛ	0.000714842	0.00517	0.00311	Vcam1/Nc	11
GO:0033559	GO:0033559	unsaturated fatty acid metabolic process	8/374	120/2332ɛ	0.000715697	0.00517	0.00311	Ptgis/Mgll.	8
GO:0042573	GO:0042573	retinoic acid metabolic process	4/374	26/23328	0.000735137	0.00527	0.00317	Rbp1/Adh	4
GO:0044342	GO:0044342	type B pancreatic cell proliferation	4/374	26/23328	0.000735137	0.00527	0.00317	Sfrp1/Igfb	4
GO:0070168	GO:0070168	negative regulation of biomineral tissue development	4/374	26/23328	0.000735137	0.00527	0.00317	Nos3/Ahs	4
GO:0110150	GO:0110150	negative regulation of biomineralization	4/374	26/23328	0.000735137	0.00527	0.00317	Nos3/Ahs	4
GO:1904030	GO:1904030	negative regulation of cyclin-dependent protein kinase	4/374	26/23328	0.000735137	0.00527	0.00317	Actb/Cdkr	4
GO:0010951	GO:0010951	negative regulation of endopeptidase activity	10/374	182/2332ɛ	0.000743053	0.00531	0.00319	Sfrp2/Thb	10
GO:0050918	GO:0050918	positive chemotaxis	5/374	45/23328	0.000743958	0.00531	0.00319	Kdr/Cxcl1	5
GO:0002028	GO:0002028	regulation of sodium ion transport	7/374	93/23328	0.000748024	0.00533	0.00321	Cxcl1/Atp	7
GO:0072006	GO:0072006	nephron development	9/374	151/2332ɛ	0.000761476	0.00542	0.00326	Cfh/Bmp4	9
GO:0030032	GO:0030032	lamellipodium assembly	6/374	68/23328	0.000773186	0.00548	0.0033	Fscn1/Itgt	6
GO:0046579	GO:0046579	positive regulation of Ras protein signal transduction	6/374	68/23328	0.000773186	0.00548	0.0033	Kitl/Mcf2l/	6
GO:0008016	GO:0008016	regulation of heart contraction	10/374	183/2332ɛ	0.000775055	0.00549	0.0033	Gja5/Abcc	10
GO:0010717	GO:0010717	regulation of epithelial to mesenchymal transition	7/374	94/23328	0.000797282	0.00562	0.00338	Sfrp1/Bmp	7
GO:0050808	GO:0050808	synapse organization	18/374	479/2332ɛ	0.000797289	0.00562	0.00338	Pcdh17/K	18
GO:0003263	GO:0003263	cardioblast proliferation	3/374	12/23328	0.000807622	0.00563	0.00339	Pim1/Hes	3
GO:0003264	GO:0003264	regulation of cardioblast proliferation	3/374	12/23328	0.000807622	0.00563	0.00339	Pim1/Hes	3
GO:0006957	GO:0006957	complement activation, alternative pathway	3/374	12/23328	0.000807622	0.00563	0.00339	Cfh/C3/Cf	3
GO:0030949	GO:0030949	positive regulation of vascular endothelial growth facto	3/374	12/23328	0.000807622	0.00563	0.00339	Bmp4/Kdr	3
GO:0034375	GO:0034375	high-density lipoprotein particle remodeling	3/374	12/23328	0.000807622	0.00563	0.00339	Scarb1/Ap	3
GO:1900272	GO:1900272	negative regulation of long-term synaptic potentiation	3/374	12/23328	0.000807622	0.00563	0.00339	Apoe/Prnp	3
GO:2001235	GO:2001235	positive regulation of apoptotic signaling pathway	10/374	184/2332ɛ	0.000808182	0.00563	0.00339	Sfrp1/Clu/	10
GO:0006636	GO:0006636	unsaturated fatty acid biosynthetic process	5/374	46/23328	0.000823842	0.00572	0.00344	Ptgis/Ptgs	5
GO:0071384	GO:0071384	cellular response to corticosteroid stimulus	5/374	46/23328	0.000823842	0.00572	0.00344	Bmp4/Zfp	5
GO:0043903	GO:0043903	regulation of symbiotic process	11/374	218/2332ɛ	0.000832291	0.00576	0.00347	Apoe/Prox	11
GO:0048545	GO:0048545	response to steroid hormone	11/374	218/2332ɛ	0.000832291	0.00576	0.00347	Sfrp1/Bmp	11
GO:0006801	GO:0006801	superoxide metabolic process	6/374	69/23328	0.000835564	0.00577	0.00347	Fbln5/Sod	6
GO:0045428	GO:0045428	regulation of nitric oxide biosynthetic process	6/374	69/23328	0.000835564	0.00577	0.00347	Clu/Ptgis/	6
GO:0048565	GO:0048565	digestive tract development	8/374	123/2332ɛ	0.000841662	0.00578	0.00347	Sfrp1/Bmp	8
GO:0031644	GO:0031644	regulation of nervous system process	10/374	185/2332ɛ	0.000842465	0.00578	0.00347	Ncam1/Kl	10
GO:0048754	GO:0048754	branching morphogenesis of an epithelial tube	10/374	185/2332ɛ	0.000842465	0.00578	0.00347	Mgp/Bmp4	10
GO:0002478	GO:0002478	antigen processing and presentation of exogenous pep	4/374	27/23328	0.00085216	0.00578	0.00347	H2-DMb1/	4
GO:0003071	GO:0003071	renal system process involved in regulation of systemi	4/374	27/23328	0.00085216	0.00578	0.00347	Gja5/F2r/F	4
GO:0010742	GO:0010742	macrophage derived foam cell differentiation	4/374	27/23328	0.00085216	0.00578	0.00347	Cd36/Lpl/	4
GO:0042044	GO:0042044	fluid transport	4/374	27/23328	0.00085216	0.00578	0.00347	Ednrb/Aqp	4
GO:0048745	GO:0048745	smooth muscle tissue development	4/374	27/23328	0.00085216	0.00578	0.00347	Bmp4/Pro	4
GO:0060343	GO:0060343	trabecula formation	4/374	27/23328	0.00085216	0.00578	0.00347	Sfrp1/Tek	4
GO:0060713	GO:0060713	labyrinthine layer morphogenesis	4/374	27/23328	0.00085216	0.00578	0.00347	Vcam1/Sp	4
GO:1905809	GO:1905809	negative regulation of synapse organization	4/374	27/23328	0.00085216	0.00578	0.00347	Apoe/Arhg	4
GO:1901568	GO:1901568	fatty acid derivative metabolic process	9/374	154/2332ɛ	0.000876018	0.00593	0.00357	Ptgis/Mgll	9
GO:0043500	GO:0043500	muscle adaptation	8/374	124/2332ɛ	0.000887395	0.00599	0.0036	Rgs4/Nos	8
GO:0051153	GO:0051153	regulation of striated muscle cell differentiation	8/374	124/2332ɛ	0.000887395	0.00599	0.0036	Bmp4/Pro	8
GO:0002573	GO:0002573	myeloid leukocyte differentiation	11/374	220/2332ɛ	0.00089676	0.00604	0.00363	Sfrp1/Rbp	11
GO:1990868	GO:1990868	response to chemokine	6/374	70/23328	0.00090175	0.00605	0.00364	Lox/Cxcl1	6
GO:1990869	GO:1990869	cellular response to chemokine	6/374	70/23328	0.00090175	0.00605	0.00364	Lox/Cxcl1	6
GO:0061053	GO:0061053	somite development	7/374	96/23328	0.000903471	0.00605	0.00364	Sfrp1/Scx	7
GO:0001990	GO:0001990	regulation of systemic arterial blood pressure by hormo	5/374	47/23328	0.000909946	0.00605	0.00364	Gja5/Nos3	5
GO:0045581	GO:0045581	negative regulation of T cell differentiation	5/374	47/23328	0.000909946	0.00605	0.00364	Bmp4/Nra	5
GO:0046460	GO:0046460	neutral lipid biosynthetic process	5/374	47/23328	0.000909946	0.00605	0.00364	Scarb1/Lp	5
GO:0046463	GO:0046463	acylglycerol biosynthetic process	5/374	47/23328	0.000909946	0.00605	0.00364	Scarb1/Lp	5
GO:0060135	GO:0060135	maternal process involved in female pregnancy	5/374	47/23328	0.000909946	0.00605	0.00364	Ptgis/Nr2f	5
GO:0006457	GO:0006457	protein folding	9/374	155/2332ɛ	0.00091717	0.00609	0.00366	Clu/Ppic/L	9
GO:0060047	GO:0060047	heart contraction	11/374	221/2332ɛ	0.000930512	0.00617	0.00371	Gja5/Abcc	11
GO:1903409	GO:1903409	reactive oxygen species biosynthetic process	8/374	125/2332ɛ	0.0009351	0.00619	0.00372	Clu/Ptgis/	8
GO:0051258	GO:0051258	protein polymerization	13/374	293/2332ɛ	0.000963487	0.00637	0.00383	Pecam1/T	13
GO:0060348	GO:0060348	bone development	11/374	222/2332ɛ	0.000965309	0.00637	0.00383	Bmp4/Scx	11
GO:0007492	GO:0007492	endoderm development	6/374	71/23328	0.000971901	0.00639	0.00384	Bmp4/Col	6
GO:0061035	GO:0061035	regulation of cartilage development	6/374	71/23328	0.000971901	0.00639	0.00384	Bmp4/Scx	6
GO:0045737	GO:0045737	positive regulation of cyclin-dependent protein serine/t	4/374	28/23328	0.00098172	0.00643	0.00386	Prox1/Rgc	4
GO:0090077	GO:0090077	foam cell differentiation	4/374	28/23328	0.00098172	0.00643	0.00386	Cd36/Lpl/	4
GO:2000178	GO:2000178	negative regulation of neural precursor cell proliferatio	4/374	28/23328	0.00098172	0.00643	0.00386	Btg2/Cd24	4
GO:0051604	GO:0051604	protein maturation	13/374	294/2332ɛ	0.000993808	0.00649	0.00391	Cpe/Perp/	13
GO:0010883	GO:0010883	regulation of lipid storage	5/374	48/23328	0.001002569	0.00652	0.00392	Scarb1/Cc	5
GO:0050850	GO:0050850	positive regulation of calcium-mediated signaling	5/374	48/23328	0.001002569	0.00652	0.00392	Ncam1/Kc	5
GO:0070527	GO:0070527	platelet aggregation	5/374	48/23328	0.001002569	0.00652	0.00392	Cfh/Fn1/P	5
GO:0034765	GO:0034765	regulation of ion transmembrane transport	18/374	489/2332ɛ	0.001007706	0.00654	0.00394	Bmp4/Gja	18
GO:0010744	GO:0010744	positive regulation of macrophage derived foam cell di	3/374	13/23328	0.001037472	0.00669	0.00402	Cd36/Lpl/	3
GO:0043589	GO:0043589	skin morphogenesis	3/374	13/23328	0.001037472	0.00669	0.00402	Itga6/Itgb4	3
GO:0048711	GO:0048711	positive regulation of astrocyte differentiation	3/374	13/23328	0.001037472	0.00669	0.00402	Hes1/Id2/	3
GO:0060670	GO:0060670	branching involved in labyrinthine layer morphogenesis	3/374	13/23328	0.001037472	0.00669	0.00402	Spint2/Ad	3
GO:1903708	GO:1903708	positive regulation of hemopoiesis	11/374	224/2332ɛ	0.001038134	0.00669	0.00402	Gata3/Cx	11
GO:0010611	GO:0010611	regulation of cardiac muscle hypertrophy	6/374	72/23328	0.001046171	0.00671	0.00404	Rgs4/Lmc	6
GO:0048002	GO:0048002	antigen processing and presentation of peptide antiger	6/374	72/23328	0.001046171	0.00671	0.00404	H2-Q6/H2	6
GO:0072001	GO:0072001	renal system development	14/374	333/2332ɛ	0.001046745	0.00671	0.00404	Sfrp1/Cfh/	14
GO:0051250	GO:0051250	negative regulation of lymphocyte activation	9/374	158/2332ɛ	0.001050136	0.00673	0.00404	Sfrp1/Bmp	9
GO:0051495	GO:0051495	positive regulation of cytoskeleton organization	11/374	225/2332ɛ	0.001076213	0.00688	0.00414	Prox1/Rgc	11
GO:0090207	GO:0090207	regulation of triglyceride metabolic process	5/374	49/23328	0.00110201	0.00702	0.00422	Scarb1/Cc	5
GO:1902041	GO:1902041	regulation of extrinsic apoptotic signaling pathway via	5/374	49/23328	0.00110201	0.00702	0.00422	Sfrp1/Sfrp	5
GO:0043406	GO:0043406	positive regulation of MAP kinase activity	11/374	226/2332ɛ	0.001115437	0.0071	0.00427	Bmp4/Thb	11
GO:0032535	GO:0032535	regulation of cellular component size	16/374	413/2332ɛ	0.001120592	0.00712	0.00428	Pecam1/T	16
GO:0002711	GO:0002711	positive regulation of T cell mediated immunity	6/374	73/23328	0.001124721	0.00713	0.00429	Gata3/Cd2	6
GO:0051155	GO:0051155	positive regulation of striated muscle cell differentiatio	6/374	73/23328	0.001124721	0.00713	0.00429	Bmp4/Pro	6
GO:0044403	GO:0044403	symbiotic process	15/374	374/2332ɛ	0.001127048	0.00713	0.00429	Vcam1/Sc	15
GO:0042116	GO:0042116	macrophage activation	7/374	100/2332ɛ	0.00114913	0.00726	0.00437	Clu/Lbp/T	7
GO:0046394	GO:0046394	carboxylic acid biosynthetic process	13/374	299/2332ɛ	0.001157665	0.0073	0.00439	Rbp1/Ptgi	13
GO:0001655	GO:0001655	urogenital system development	15/374	376/2332ɛ	0.001188242	0.00748	0.0045	Sfrp1/Cfh/	15
GO:0016053	GO:0016053	organic acid biosynthetic process	13/374	300/2332ɛ	0.001193003	0.0075	0.00451	Rbp1/Ptgi	13
GO:0070997	GO:0070997	neuron death	16/374	416/2332ɛ	0.001207683	0.00758	0.00456	Clu/Kdr/G	16
GO:0045646	GO:0045646	regulation of erythrocyte differentiation	5/374	50/23328	0.001208574	0.00758	0.00456	Lmo2/Hm	5
GO:0032092	GO:0032092	positive regulation of protein binding	7/374	101/2332ɛ	0.001218038	0.00761	0.00458	Spon1/Bn	7
GO:0033555	GO:0033555	multicellular organismal response to stress	7/374	101/2332ɛ	0.001218038	0.00761	0.00458	Ncam1/Ec	7
GO:1901214	GO:1901214	regulation of neuron death	15/374	377/2332ɛ	0.001219874	0.00761	0.00458	Clu/Kdr/G	15
GO:0048640	GO:0048640	negative regulation of developmental growth	8/374	131/2332ɛ	0.001266228	0.00789	0.00475	Sfrp1/Bmp	8
GO:0035162	GO:0035162	embryonic hemopoiesis	4/374	30/23328	0.001281273	0.00793	0.00477	Lmo2/Kdr	4
GO:0035767	GO:0035767	endothelial cell chemotaxis	4/374	30/23328	0.001281273	0.00793	0.00477	Kdr/Thbs1	4
GO:0051085	GO:0051085	chaperone cofactor-dependent protein refolding	4/374	30/23328	0.001281273	0.00793	0.00477	H2-DMb1/	4
GO:0071711	GO:0071711	basement membrane organization	4/374	30/23328	0.001281273	0.00793	0.00477	Itgb1/Spin	4
GO:1903579	GO:1903579	negative regulation of ATP metabolic process	4/374	30/23328	0.001281273	0.00793	0.00477	Nupr1/Cbt	4
GO:0003002	GO:0003002	regionalization	15/374	379/2332ɛ	0.001285268	0.00794	0.00477	Sfrp1/Bmp	15
GO:1903725	GO:1903725	regulation of phospholipid metabolic process	6/374	75/23328	0.00129531	0.00796	0.00479	Scarb1/Flt	6
GO:0043087	GO:0043087	regulation of GTPase activity	13/374	303/2332ɛ	0.001304459	0.00796	0.00479	Pecam1/F	13
GO:0014820	GO:0014820	tonic smooth muscle contraction	3/374	14/23328	0.00130479	0.00796	0.00479	Cd38/Mylk	3
GO:0035791	GO:0035791	platelet-derived growth factor receptor-beta signaling p	3/374	14/23328	0.00130479	0.00796	0.00479	Lox/Pdgfa	3
GO:0061308	GO:0061308	cardiac neural crest cell development involved in hear	3/374	14/23328	0.00130479	0.00796	0.00479	Bmp4/Hes	3
GO:0061450	GO:0061450	trophoblast cell migration	3/374	14/23328	0.00130479	0.00796	0.00479	Arhgdib/It	3
GO:0070365	GO:0070365	hepatocyte differentiation	3/374	14/23328	0.00130479	0.00796	0.00479	Bmp4/Pro	3
GO:0072330	GO:0072330	monocarboxylic acid biosynthetic process	10/374	196/2332ɛ	0.001304931	0.00796	0.00479	Rbp1/Ptgi	10
GO:0002244	GO:0002244	hematopoietic progenitor cell differentiation	9/374	163/2332ɛ	0.001306132	0.00796	0.00479	Sfrp1/Bmp	9
GO:0003015	GO:0003015	heart process	11/374	231/2332ɛ	0.001329656	0.00808	0.00486	Gja5/Abcc	11
GO:0009952	GO:0009952	anterior/posterior pattern specification	11/374	231/2332ɛ	0.001329656	0.00808	0.00486	Sfrp1/Bmp	11
GO:0010639	GO:0010639	negative regulation of organelle organization	16/374	420/2332ɛ	0.001332651	0.00809	0.00486	Pecam1/T	16
GO:0045017	GO:0045017	glycerolipid biosynthetic process	9/374	164/2332ɛ	0.001362908	0.00824	0.00496	Scarb1/Lp	9
GO:0051224	GO:0051224	negative regulation of protein transport	9/374	164/2332ɛ	0.001362908	0.00824	0.00496	Sfrp1/Cd3	9
GO:0015748	GO:0015748	organophosphate ester transport	7/374	103/2332ɛ	0.001365537	0.00824	0.00496	Scarb1/Kc	7
GO:0055024	GO:0055024	regulation of cardiac muscle tissue development	7/374	103/2332ɛ	0.001365537	0.00824	0.00496	Ncam1/Br	7
GO:0030307	GO:0030307	positive regulation of cell growth	10/374	198/2332ɛ	0.001407861	0.00848	0.0051	Sfrp1/Apo	10
GO:0045862	GO:0045862	positive regulation of proteolysis	14/374	344/2332ɛ	0.001422679	0.00856	0.00515	Clu/Perp/S	14
GO:0001974	GO:0001974	blood vessel remodeling	5/374	52/23328	0.001444304	0.00857	0.00515	Nos3/Flt4/	5

GO:0014074	GO:0014074	response to purine-containing compound	7/374	104/23328	0.001444334	0.00857	0.00515	Abcc9/Pde	7
GO:0046849	GO:0046849	bone remodeling	7/374	104/23328	0.001444334	0.00857	0.00515	Sfrp1/Cthr	7
GO:0048525	GO:0048525	negative regulation of viral process	7/374	104/23328	0.001444334	0.00857	0.00515	Prox1/Rsa	7
GO:1902106	GO:1902106	negative regulation of leukocyte differentiation	7/374	104/23328	0.001444334	0.00857	0.00515	Sfrp1/Bmp	7
GO:0001958	GO:0001958	endochondral ossification	4/374	31/23328	0.001452674	0.00857	0.00515	Bmp4/Scx	4
GO:0002828	GO:0002828	regulation of type 2 immune response	4/374	31/23328	0.001452674	0.00857	0.00515	Gata3/Rsa	4
GO:0033687	GO:0033687	osteoblast proliferation	4/374	31/23328	0.001452674	0.00857	0.00515	Sfrp1/Fbln	4
GO:0036075	GO:0036075	replacement ossification	4/374	31/23328	0.001452674	0.00857	0.00515	Bmp4/Scx	4
GO:0050901	GO:0050901	leukocyte tethering or rolling	4/374	31/23328	0.001452674	0.00857	0.00515	Vcam1/Se	4
GO:0051497	GO:0051497	negative regulation of stress fiber assembly	4/374	31/23328	0.001452674	0.00857	0.00515	Cgnl1/Kar	4
GO:0061037	GO:0061037	negative regulation of cartilage development	4/374	31/23328	0.001452674	0.00857	0.00515	Bmp4/Efe	4
GO:1904031	GO:1904031	positive regulation of cyclin-dependent protein kinase a	4/374	31/23328	0.001452674	0.00857	0.00515	Prox1/Rgc	4
GO:1905314	GO:1905314	semi-lunar valve development	4/374	31/23328	0.001452674	0.00857	0.00515	Bmp4/Gja	4
GO:0022898	GO:0022898	regulation of transmembrane transporter activity	12/374	270/23328	0.001472202	0.00867	0.00521	Bmp4/Fhl	12
GO:0060828	GO:0060828	regulation of canonical Wnt signaling pathway	11/374	234/23328	0.001473652	0.00867	0.00521	Sfrp1/Dkk	11
GO:0072331	GO:0072331	signal transduction by p53 class mediator	8/374	135/23328	0.001534542	0.00901	0.00542	Perp/Nupr	8
GO:0048662	GO:0048662	negative regulation of smooth muscle cell proliferation	5/374	53/23328	0.001574093	0.00919	0.00553	Igfbp3/No	5
GO:0050435	GO:0050435	amyloid-beta metabolic process	5/374	53/23328	0.001574093	0.00919	0.00553	Clu/Spon1	5
GO:0051055	GO:0051055	negative regulation of lipid biosynthetic process	5/374	53/23328	0.001574093	0.00919	0.00553	Dkk3/Apo	5
GO:0061383	GO:0061383	trabecula morphogenesis	5/374	53/23328	0.001574093	0.00919	0.00553	Sfrp1/Tek	5
GO:0072332	GO:0072332	intrinsic apoptotic signaling pathway by p53 class med	6/374	78/23328	0.001587405	0.00923	0.00555	Perp/Nupr	6
GO:0051928	GO:0051928	positive regulation of calcium ion transport	8/374	136/23328	0.001608192	0.00923	0.00555	Bmp4/Apl	8
GO:0010561	GO:0010561	negative regulation of glycoprotein biosynthetic proces	3/374	15/23328	0.001611699	0.00923	0.00555	Bace2/Itm	3
GO:0010885	GO:0010885	regulation of cholesterol storage	3/374	15/23328	0.001611699	0.00923	0.00555	Scarb1/Cc	3
GO:0019755	GO:0019755	one-carbon compound transport	3/374	15/23328	0.001611699	0.00923	0.00555	Car4/Aqp	3
GO:0030449	GO:0030449	regulation of complement activation	3/374	15/23328	0.001611699	0.00923	0.00555	Cfh/C3/Se	3
GO:0031115	GO:0031115	negative regulation of microtubule polymerization	3/374	15/23328	0.001611699	0.00923	0.00555	Cdh5/Stm	3
GO:0061307	GO:0061307	cardiac neural crest cell differentiation involved in hea	3/374	15/23328	0.001611699	0.00923	0.00555	Bmp4/Hes	3
GO:0061430	GO:0061430	bone trabecula morphogenesis	3/374	15/23328	0.001611699	0.00923	0.00555	Sfrp1/Mm	3
GO:1902043	GO:1902043	positive regulation of extrinsic apoptotic signaling path	3/374	15/23328	0.001611699	0.00923	0.00555	Sfrp1/Thb	3
GO:0014812	GO:0014812	muscle cell migration	7/374	106/23328	0.001612541	0.00923	0.00555	Tmsb4x/Ic	7
GO:0031532	GO:0031532	actin cytoskeleton reorganization	7/374	106/23328	0.001612541	0.00923	0.00555	Tek/Arhgc	7
GO:0034446	GO:0034446	substrate adhesion-dependent cell spreading	7/374	106/23328	0.001612541	0.00923	0.00555	Sfrp1/Tek	7
GO:0110110	GO:0110110	positive regulation of animal organ morphogenesis	7/374	106/23328	0.001612541	0.00923	0.00555	Bmp4/Gat	7
GO:0009306	GO:0009306	protein secretion	17/374	470/23328	0.001660525	0.0095	0.00571	Sfrp1/Pan	17
GO:1904950	GO:1904950	negative regulation of establishment of protein localiza	9/374	169/23328	0.00167735	0.00958	0.00576	Sfrp1/Cd3	9
GO:0055021	GO:0055021	regulation of cardiac muscle tissue growth	6/374	79/23328	0.001695107	0.00967	0.00581	Ncam1/Rc	6
GO:0035592	GO:0035592	establishment of protein localization to extracellular re	17/374	471/23328	0.001698093	0.00967	0.00582	Sfrp1/Pan	17
GO:0014897	GO:0014897	striated muscle hypertrophy	7/374	107/23328	0.001702164	0.00968	0.00582	Rgs4/Lmc	7
GO:0014009	GO:0014009	glial cell proliferation	5/374	54/23328	0.001712252	0.00973	0.00585	Clu/Sox4/	5
GO:0006639	GO:0006639	acylglycerol metabolic process	8/374	138/23328	0.001763879	0.01	0.00602	Scarb1/M	8
GO:0043266	GO:0043266	regulation of potassium ion transport	7/374	108/23328	0.00179561	0.01014	0.0061	Fhl1/Cxcl	7
GO:0045639	GO:0045639	positive regulation of myeloid cell differentiation	7/374	108/23328	0.00179561	0.01014	0.0061	Fos/Kitl/H	7
GO:0055017	GO:0055017	cardiac muscle tissue growth	7/374	108/23328	0.00179561	0.01014	0.0061	Ncam1/Rc	7
GO:0006813	GO:0006813	potassium ion transport	11/374	240/23328	0.001800063	0.01015	0.00611	Gja5/Kcnr	11
GO:0046661	GO:0046661	male sex differentiation	9/374	171/23328	0.001818343	0.01024	0.00616	Sfrp1/Kdr/	9
GO:0060914	GO:0060914	heart formation	4/374	33/23328	0.001842224	0.01036	0.00623	Bmp4/Pim	4
GO:0002720	GO:0002720	positive regulation of cytokine production involved in ir	5/374	55/23328	0.001859097	0.01043	0.00627	Gata3/Cd	5
GO:0003044	GO:0003044	regulation of systemic arterial blood pressure mediatec	5/374	55/23328	0.001859097	0.01043	0.00627	Gja5/Nos	5
GO:0014896	GO:0014896	muscle hypertrophy	7/374	109/23328	0.001892989	0.01059	0.00637	Rgs4/Lmc	7
GO:0051668	GO:0051668	localization within membrane	7/374	109/23328	0.001892989	0.01059	0.00637	Apoe/Cd2	7
GO:0006638	GO:0006638	neutral lipid metabolic process	8/374	140/23328	0.001931238	0.01071	0.00644	Scarb1/M	8
GO:0002830	GO:0002830	positive regulation of type 2 immune response	3/374	16/23328	0.001960189	0.01071	0.00644	Gata3/Rsa	3
GO:0007512	GO:0007512	adult heart development	3/374	16/23328	0.001960189	0.01071	0.00644	Aplnr/Han	3
GO:0010224	GO:0010224	response to UV-B	3/374	16/23328	0.001960189	0.01071	0.00644	Cdkn1a/C	3
GO:0015669	GO:0015669	gas transport	3/374	16/23328	0.001960189	0.01071	0.00644	Car4/Cygb	3
GO:0018119	GO:0018119	peptidyl-cysteine S-nitrosylation	3/374	16/23328	0.001960189	0.01071	0.00644	Gapdh/S1	3
GO:0019886	GO:0019886	antigen processing and presentation of exogenous pep	3/374	16/23328	0.001960189	0.01071	0.00644	H2-DMb1/	3
GO:0040034	GO:0040034	regulation of development, heterochronic	3/374	16/23328	0.001960189	0.01071	0.00644	Hmgn2/He	3
GO:0045820	GO:0045820	negative regulation of glycolytic process	3/374	16/23328	0.001960189	0.01071	0.00644	Nupr1/Cbf	3
GO:0048715	GO:0048715	negative regulation of oligodendrocyte differentiation	3/374	16/23328	0.001960189	0.01071	0.00644	Bmp4/Hes	3
GO:0060026	GO:0060026	convergent extension	3/374	16/23328	0.001960189	0.01071	0.00644	Sfrp1/Sfrp	3
GO:0060100	GO:0060100	positive regulation of phagocytosis, engulfment	3/374	16/23328	0.001960189	0.01071	0.00644	Cd36/Lbp	3
GO:0072537	GO:0072537	fibroblast activation	3/374	16/23328	0.001960189	0.01071	0.00644	Rgcc/Cyg	3
GO:0090177	GO:0090177	establishment of planar polarity involved in neural tube	3/374	16/23328	0.001960189	0.01071	0.00644	Sfrp1/Cthr	3
GO:1901550	GO:1901550	regulation of endothelial cell development	3/374	16/23328	0.001960189	0.01071	0.00644	Cdh5/Cldr	3
GO:1901722	GO:1901722	regulation of cell proliferation involved in kidney develo	3/374	16/23328	0.001960189	0.01071	0.00644	Bmp4/Gat	3
GO:1903140	GO:1903140	regulation of establishment of endothelial barrier	3/374	16/23328	0.001960189	0.01071	0.00644	Cdh5/Cldr	3
GO:1905155	GO:1905155	positive regulation of membrane invagination	3/374	16/23328	0.001960189	0.01071	0.00644	Cd36/Lbp	3
GO:0007389	GO:0007389	pattern specification process	17/374	478/23328	0.001981653	0.0108	0.0065	Sfrp1/Bmp	17
GO:0071692	GO:0071692	protein localization to extracellular region	17/374	478/23328	0.001981653	0.0108	0.0065	Sfrp1/Pan	17
GO:0002526	GO:0002526	acute inflammatory response	7/374	110/23328	0.001994413	0.01083	0.00651	Ednrb/Lbp	7
GO:0032231	GO:0032231	regulation of actin filament bundle assembly	7/374	110/23328	0.001994413	0.01083	0.00651	Cgnl1/Rgc	7
GO:0032409	GO:0032409	regulation of transporter activity	12/374	280/23328	0.001994998	0.01083	0.00651	Bmp4/Fhl	12
GO:0050732	GO:0050732	negative regulation of peptidyl-tyrosine phosphorylatio	5/374	56/23328	0.002014949	0.01091	0.00656	Sfrp1/Pec	5
GO:1902893	GO:1902893	regulation of pri-miRNA transcription by RNA polymera	5/374	56/23328	0.002014949	0.01091	0.00656	Bmp4/Gat	5
GO:0015914	GO:0015914	phospholipid transport	6/374	82/23328	0.002051662	0.01108	0.00666	Scarb1/Kc	6
GO:0019884	GO:0019884	antigen processing and presentation of exogenous ant	4/374	34/23328	0.002061758	0.01108	0.00666	H2-DMb1/	4
GO:0043537	GO:0043537	negative regulation of blood vessel endothelial cell mig	4/374	34/23328	0.002061758	0.01108	0.00666	Apoe/Thb	4
GO:0071634	GO:0071634	regulation of transforming growth factor beta productio	4/374	34/23328	0.002061758	0.01108	0.00666	Thbs1/Cd	4
GO:0098868	GO:0098868	bone growth	4/374	34/23328	0.002061758	0.01108	0.00666	Kdr/Fbln5	4
GO:1904646	GO:1904646	cellular response to amyloid-beta	4/374	34/23328	0.002061758	0.01108	0.00666	Vcam1/Cc	4
GO:0046486	GO:0046486	glycerolipid metabolic process	14/374	360/23328	0.002165861	0.01162	0.00699	Scarb1/M	14
GO:0050863	GO:0050863	regulation of T cell activation	13/374	321/23328	0.002170514	0.01163	0.007	Vcam1/Br	13
GO:0002548	GO:0002548	monocyte chemotaxis	5/374	57/23328	0.002180128	0.01163	0.007	Flt1/Cxcl1	5
GO:0010463	GO:0010463	mesenchymal cell proliferation	5/374	57/23328	0.002180128	0.01163	0.007	Bmp4/Kdr	5
GO:0043030	GO:0043030	regulation of macrophage activation	5/374	57/23328	0.002180128	0.01163	0.007	Lbp/Thbs	5
GO:0060415	GO:0060415	muscle tissue morphogenesis	6/374	83/23328	0.002182256	0.01163	0.007	Prox1/Ly6	6
GO:0030038	GO:0030038	contractile actin filament bundle assembly	7/374	112/23328	0.002209839	0.01175	0.00707	Eln/Cgnl1	7
GO:0043149	GO:0043149	stress fiber assembly	7/374	112/23328	0.002209839	0.01175	0.00707	Eln/Cgnl1	7
GO:1904062	GO:1904062	regulation of cation transmembrane transport	14/374	361/23328	0.00222135	0.0118	0.0071	Bmp4/Apl	14
GO:0006458	GO:0006458	'de novo' protein folding	4/374	35/23328	0.002298714	0.01209	0.00727	H2-DMb1/	4
GO:0016339	GO:0016339	calcium-dependent cell-cell adhesion via plasma mem	4/374	35/23328	0.002298714	0.01209	0.00727	Selp/Cdh1	4
GO:0034110	GO:0034110	regulation of homotypic cell-cell adhesion	4/374	35/23328	0.002298714	0.01209	0.00727	Cd24a/Pd	4
GO:0051084	GO:0051084	'de novo' posttranslational protein folding	4/374	35/23328	0.002298714	0.01209	0.00727	H2-DMb1/	4
GO:0055090	GO:0055090	acylglycerol homeostasis	4/374	35/23328	0.002298714	0.01209	0.00727	Gpihbp1/S	4
GO:0070328	GO:0070328	triglyceride homeostasis	4/374	35/23328	0.002298714	0.01209	0.00727	Gpihbp1/S	4
GO:1902003	GO:1902003	regulation of amyloid-beta formation	4/374	35/23328	0.002298714	0.01209	0.00727	Clu/Spon1	4
GO:0035148	GO:0035148	tube formation	9/374	177/23328	0.002299025	0.01209	0.00727	Sfrp1/Bmp	9
GO:0001837	GO:0001837	epithelial to mesenchymal transition	8/374	144/23328	0.002303384	0.01209	0.00727	Sfrp1/Bmp	8
GO:0050766	GO:0050766	positive regulation of phagocytosis	6/374	84/23328	0.002319021	0.01214	0.0073	Cd36/Lbp	6
GO:0050764	GO:0050764	regulation of phagocytosis	7/374	113/23328	0.00232407	0.01214	0.0073	Scarb1/Cc	7
GO:0001886	GO:0001886	endothelial cell morphogenesis	3/374	17/23328	0.002352124	0.01214	0.0073	Pecam1/C	3
GO:0003184	GO:0003184	pulmonary valve morphogenesis	3/374	17/23328	0.002352124	0.01214	0.0073	Bmp4/Gja	3
GO:0010878	GO:0010878	cholesterol storage	3/374	17/23328	0.002352124	0.01214	0.0073	Scarb1/Cc	3
GO:0017014	GO:0017014	protein nitrosylation	3/374	17/23328	0.002352124	0.01214	0.0073	Gapdh/S1	3
GO:0033194	GO:0033194	response to hydroperoxide	3/374	17/23328	0.002352124	0.01214	0.0073	Cd36/Cd3	3
GO:0071636	GO:0071636	positive regulation of transforming growth factor beta p	3/374	17/23328	0.002352124	0.01214	0.0073	Thbs1/Cd	3
GO:1900119	GO:1900119	positive regulation of execution phase of apoptosis	3/374	17/23328	0.002352124	0.01214	0.0073	Ptgis/Igfb	3
GO:2001028	GO:2001028	positive regulation of endothelial cell chemotaxis	3/374	17/23328	0.002352124	0.01214	0.0073	Kdr/Hspb	3
GO:0001755	GO:0001755	neural crest cell migration	5/374	58/23328	0.002354957	0.01214	0.0073	Bmp4/Edr	5
GO:0010656	GO:0010656	negative regulation of muscle cell apoptotic process	5/374	58/23328	0.002354957	0.01214	0.0073	Sfrp2/Nup	5
GO:0035690	GO:0035690	cellular response to drug	5/374	58/23328	0.002354957	0.01214	0.0073	Gstm1/Pr	5
GO:0045071	GO:0045071	negative regulation of viral genome replication	5/374	58/23328	0.002354957	0.01214	0.0073	Prox1/Rsa	5
GO:0061614	GO:0061614	pri-miRNA transcription by RNA polymerase II	5/374	58/23328	0.002354957	0.01214	0.0073	Bmp4/Gat	5
GO:0050821	GO:0050821	protein stabilization	9/374	178/23328	0.002388135	0.01229	0.00739	Clu/Gpihb	9
GO:0051651	GO:0051651	maintenance of location in cell	10/374	213/23328	0.002408766	0.01238	0.00745	Tmsb4x/A	10
GO:0006939	GO:0006939	smooth muscle contraction	7/374	114/23328	0.002442799	0.01253	0.00753	Ednrb/Cd	7
GO:1905330	GO:1905330	regulation of morphogenesis of an epithelium	7/374	114/23328	0.002442799	0.01253	0.00753	Sfrp1/Bmp	7
GO:0006959	GO:0006959	humoral immune response	15/374	406/23328	0.002498537	0.0128	0.0077	Cfh/Gata3	15
GO:0007568	GO:0007568	aging	11/374	251/23328	0.002550827	0.01295	0.00779	Cfh/Ncam	11
GO:0032232	GO:0032232	negative regulation of actin filament bundle assembly	4/374	36/23328	0.002553767	0.01295	0.00779	Cgnl1/Kar	4
GO:0034381	GO:0034381	plasma lipoprotein particle clearance	4/374	36/23328	0.002553767	0.01295	0.00779	Gpihbp1/S	4
GO:0035886	GO:0035886	vascular associated smooth muscle cell differentiation	4/374	36/23328	0.002553767	0.01295	0.00779	Adm/Hes1	4
GO:0070306	GO:0070306	lens fiber cell differentiation	4/374	36/23328	0.002553767	0.01295	0.00779	Maf/Prox1	4
GO:0071158	GO:0071158	positive regulation of cell cycle arrest	4/374	36/23328	0.002553767	0.01295	0.00779	Rgcc/Sox	4
GO:0071604	GO:0071604	transforming growth factor beta production	4/374	36/23328	0.002553767	0.01295	0.00779	Thbs1/Cd	4
GO:0097242	GO:0097242	amyloid-beta clearance	4/374	36/23328	0.002553767	0.01295	0.00779	Clu/Cd36/	4
GO:0008585	GO:0008585	female gonad development	7/374	115/23328	0.002566143	0.013	0.00782	Sfrp1/Bmp	7

GO:0007219	GO:0007219	Notch signaling pathway	9/374	180/2332{	0.002574582	0.01301	0.00782	Perp/Nrar	9
GO:1901861	GO:1901861	regulation of muscle tissue development	9/374	180/2332{	0.002574582	0.01301	0.00782	Ncam1/Br	9
GO:0048839	GO:0048839	inner ear development	10/374	215/2332{	0.002577118	0.01301	0.00782	Cytl1/Bmp	10
GO:0031333	GO:0031333	negative regulation of protein-containing complex asse	8/374	147/2332{	0.002617694	0.0132	0.00794	Clu/Pecar	8
GO:0042391	GO:0042391	regulation of membrane potential	16/374	450/2332{	0.002664707	0.01342	0.00807	Gja5/Kdr/I	16
GO:0030098	GO:0030098	lymphocyte differentiation	15/374	409/2332{	0.002678331	0.01347	0.0081	Sfrp1/Bmp	15
GO:2000045	GO:2000045	regulation of G1/S transition of mitotic cell cycle	7/374	116/2332{	0.002694219	0.01353	0.00814	Fhl1/Rgcc	7
GO:0006670	GO:0006670	sphingosine metabolic process	3/374	18/23328	0.002789248	0.01391	0.00837	Plpp3/Plp	3
GO:0021542	GO:0021542	dentate gyrus development	3/374	18/23328	0.002789248	0.01391	0.00837	Prox1/Btg	3
GO:0033631	GO:0033631	cell-cell adhesion mediated by integrin	3/374	18/23328	0.002789248	0.01391	0.00837	Cd24a/Plp	3
GO:0042249	GO:0042249	establishment of planar polarity of embryonic epitheliu	3/374	18/23328	0.002789248	0.01391	0.00837	Sfrp1/Cthr	3
GO:0072148	GO:0072148	epithelial cell fate commitment	3/374	18/23328	0.002789248	0.01391	0.00837	Prox1/Nr2	3
GO:1990000	GO:1990000	amyloid fibril formation	3/374	18/23328	0.002789248	0.01391	0.00837	Clu/Cd36/	3
GO:0002691	GO:0002691	regulation of cellular extravasation	4/374	37/23328	0.002827587	0.01407	0.00846	Pecam1/S	4
GO:0051482	GO:0051482	positive regulation of cytosolic calcium ion concentrati	4/374	37/23328	0.002827587	0.01407	0.00846	Tgm2/F2r/	4
GO:0007613	GO:0007613	memory	8/374	149/2332{	0.002845194	0.01414	0.0085	Klk8/Apoe	8
GO:0000082	GO:0000082	G1/S transition of mitotic cell cycle	9/374	183/2332{	0.002875697	0.01427	0.00858	Fhl1/Rgcc	9
GO:0007188	GO:0007188	adenylate cyclase-modulating G protein-coupled recep	10/374	219/2332{	0.002942085	0.01458	0.00877	Adgrl4/Ad	10
GO:0046545	GO:0046545	development of primary female sexual characteristics	7/374	118/2332{	0.002965042	0.01468	0.00883	Sfrp1/Bmp	7
GO:0046942	GO:0046942	carboxylic acid transport	13/374	333/2332{	0.002976492	0.01472	0.00885	Fabp4/Slc	13
GO:0097193	GO:0097193	intrinsic apoptotic signaling pathway	12/374	295/2332{	0.00305636	0.0151	0.00908	Clu/Perp/I	12
GO:0048593	GO:0048593	camera-type eye morphogenesis	8/374	151/2332{	0.003087828	0.01523	0.00916	Cfh/Bmp4	8
GO:0051702	GO:0051702	interaction with symbiont	6/374	89/23328	0.003101843	0.01529	0.00919	Apoe/Cxc	6
GO:0001706	GO:0001706	endoderm formation	4/374	38/23328	0.003120833	0.01529	0.0092	Col5a2/Fr	4
GO:0019432	GO:0019432	triglyceride biosynthetic process	4/374	38/23328	0.003120833	0.01529	0.0092	Scarb1/Lp	4
GO:0048265	GO:0048265	response to pain	4/374	38/23328	0.003120833	0.01529	0.0092	Ednrb/Thb	4
GO:0060674	GO:0060674	placenta blood vessel development	4/374	38/23328	0.003120833	0.01529	0.0092	Nr2f2/Jun	4
GO:0072210	GO:0072210	metanephric nephron development	4/374	38/23328	0.003120833	0.01529	0.0092	Bmp4/Hes	4
GO:0002253	GO:0002253	activation of immune response	15/374	416/2332{	0.003139759	0.01536	0.00924	Cfh/Bmx/I	15
GO:0046850	GO:0046850	regulation of bone remodeling	5/374	62/23328	0.003157237	0.01541	0.00927	Sfrp1/Cd3	5
GO:0060350	GO:0060350	endochondral bone morphogenesis	5/374	62/23328	0.003157237	0.01541	0.00927	Bmp4/Scx	5
GO:0090288	GO:0090288	negative regulation of cellular response to growth facto	8/374	152/2332{	0.003215029	0.01568	0.00943	Sfrp1/Htra	8
GO:0043547	GO:0043547	positive regulation of GTPase activity	10/374	222/2332{	0.003241986	0.01576	0.00948	Rgs4/Itga(	10
GO:0032680	GO:0032680	regulation of tumor necrosis factor production	7/374	120/2332{	0.003256226	0.01576	0.00948	Clu/Cd36/	7
GO:0003180	GO:0003180	aortic valve morphogenesis	3/374	19/23328	0.003273186	0.01576	0.00948	Bmp4/Gat	3
GO:0003215	GO:0003215	cardiac right ventricle morphogenesis	3/374	19/23328	0.003273186	0.01576	0.00948	Gata3/Sox	3
GO:0016486	GO:0016486	peptide hormone processing	3/374	19/23328	0.003273186	0.01576	0.00948	Cpe/Ece1	3
GO:0030502	GO:0030502	negative regulation of bone mineralization	3/374	19/23328	0.003273186	0.01576	0.00948	Ahsg/Srgr	3
GO:0035994	GO:0035994	response to muscle stretch	3/374	19/23328	0.003273186	0.01576	0.00948	Fos/Jun/N	3
GO:0060099	GO:0060099	regulation of phagocytosis, engulfment	3/374	19/23328	0.003273186	0.01576	0.00948	Cd36/Lbp/	3
GO:0060231	GO:0060231	mesenchymal to epithelial transition	3/374	19/23328	0.003273186	0.01576	0.00948	Bmp4/Gat	3
GO:0071071	GO:0071071	regulation of phospholipid biosynthetic process	3/374	19/23328	0.003273186	0.01576	0.00948	Pdgfa/Adc	3
GO:1905276	GO:1905276	regulation of epithelial tube formation	3/374	19/23328	0.003273186	0.01576	0.00948	Sfrp1/Gat	3
GO:0015849	GO:0015849	organic acid transport	13/374	337/2332{	0.003293976	0.01584	0.00953	Fabp4/Slc	13
GO:0006936	GO:0006936	muscle contraction	12/374	298/2332{	0.003315494	0.01592	0.00958	Gja5/Ednr	12
GO:0001818	GO:0001818	negative regulation of cytokine production	11/374	260/2332{	0.003337863	0.01601	0.00963	Gata3/Lbp	11
GO:0055088	GO:0055088	lipid homeostasis	8/374	153/2332{	0.003346265	0.01602	0.00963	Rbp1/Gpil	8
GO:0071478	GO:0071478	cellular response to radiation	8/374	153/2332{	0.003346265	0.01602	0.00963	Sfrp1/Gat	8
GO:0071479	GO:0071479	cellular response to ionizing radiation	5/374	63/23328	0.00338517	0.01618	0.00973	Sfrp1/Gat	5
GO:0002688	GO:0002688	regulation of leukocyte chemotaxis	7/374	121/2332{	0.003409756	0.01626	0.00978	Lbp/Thbs1	7
GO:0046620	GO:0046620	regulation of organ growth	7/374	121/2332{	0.003409756	0.01626	0.00978	Ncam1/Pr	7
GO:0030513	GO:0030513	positive regulation of BMP signaling pathway	4/374	39/23328	0.003434158	0.01632	0.00982	Bmp4/Kdr	4
GO:0050715	GO:0050715	positive regulation of cytokine secretion	4/374	39/23328	0.003434158	0.01632	0.00982	Cd36/Rgc	4
GO:0090049	GO:0090049	regulation of cell migration involved in sprouting angio	4/374	39/23328	0.003434158	0.01632	0.00982	Kdr/Thbs1	4
GO:0002821	GO:0002821	positive regulation of adaptive immune response	8/374	154/2332{	0.003481621	0.01651	0.00993	Gata3/Cd	8
GO:0008360	GO:0008360	regulation of cell shape	8/374	154/2332{	0.003481621	0.01651	0.00993	Kdr/Fblim	8
GO:0050868	GO:0050868	negative regulation of T cell activation	7/374	122/2332{	0.003568742	0.01689	0.01016	Bmp4/Cd2	7
GO:1903555	GO:1903555	regulation of tumor necrosis factor superfamily cytokin	7/374	122/2332{	0.003568742	0.01689	0.01016	Clu/Cd36/	7
GO:0001838	GO:0001838	embryonic epithelial tube formation	8/374	155/2332{	0.003621183	0.01707	0.01027	Sfrp1/Bmp	8
GO:0008347	GO:0008347	glial cell migration	5/374	64/23328	0.003624694	0.01707	0.01027	Fn1/Vim/L	5
GO:0045668	GO:0045668	negative regulation of osteoblast differentiation	5/374	64/23328	0.003624694	0.01707	0.01027	Sfrp1/Id1/	5
GO:0050707	GO:0050707	regulation of cytokine secretion	5/374	64/23328	0.003624694	0.01707	0.01027	Cd36/Rgc	5
GO:0014909	GO:0014909	smooth muscle cell migration	6/374	92/23328	0.003657443	0.01719	0.01034	Tmsb4x/Ig	6
GO:0043900	GO:0043900	regulation of multi-organism process	6/374	92/23328	0.003657443	0.01719	0.01034	Ednrb/Pde	6
GO:0032675	GO:0032675	regulation of interleukin-6 production	8/374	156/2332{	0.003765037	0.01765	0.01062	Cd36/Lbp/	8
GO:0042092	GO:0042092	type 2 immune response	4/374	40/23328	0.003768199	0.01765	0.01062	Gata3/Rsa	4
GO:1903959	GO:1903959	regulation of anion transmembrane transport	4/374	40/23328	0.003768199	0.01765	0.01062	Thbs1/Rg	4
GO:0002577	GO:0002577	regulation of antigen processing and presentation	3/374	20/23328	0.003805449	0.01767	0.01063	Thbs1/Fgl	3
GO:0010763	GO:0010763	positive regulation of fibroblast migration	3/374	20/23328	0.003805449	0.01767	0.01063	Thbs1/Itgt	3
GO:0010875	GO:0010875	positive regulation of cholesterol efflux	3/374	20/23328	0.003805449	0.01767	0.01063	Apoe/Nfkt	3
GO:0046519	GO:0046519	sphingoid metabolic process	3/374	20/23328	0.003805449	0.01767	0.01063	Plpp3/Plp	3
GO:0072243	GO:0072243	metanephric nephron epithelium development	3/374	20/23328	0.003805449	0.01767	0.01063	Hes1/Pdg	3
GO:0140448	GO:0140448	signaling receptor ligand precursor processing	3/374	20/23328	0.003805449	0.01767	0.01063	Cpe/Ece1	3
GO:1905153	GO:1905153	regulation of membrane invagination	3/374	20/23328	0.003805449	0.01767	0.01063	Cd36/Lbp/	3
GO:2000114	GO:2000114	regulation of establishment of cell polarity	3/374	20/23328	0.003805449	0.01767	0.01063	Gata3/Cdl	3
GO:0071774	GO:0071774	response to fibroblast growth factor	6/374	93/23328	0.003858092	0.01789	0.01076	Sfrp1/Gat	6
GO:0034308	GO:0034308	primary alcohol metabolic process	5/374	65/23328	0.003876135	0.01796	0.0108	Rbp1/Adh	5
GO:0030111	GO:0030111	regulation of Wnt signaling pathway	12/374	304/2332{	0.003887061	0.01799	0.01082	Sfrp1/Dkk	12
GO:0006953	GO:0006953	acute-phase response	4/374	41/23328	0.004123588	0.01897	0.01141	Ednrb/Lbp	4
GO:0010470	GO:0010470	regulation of gastrulation	4/374	41/23328	0.004123588	0.01897	0.01141	Scx/Col5a	4
GO:0034205	GO:0034205	amyloid-beta formation	4/374	41/23328	0.004123588	0.01897	0.01141	Clu/Spon1	4
GO:0046164	GO:0046164	alcohol catabolic process	4/374	41/23328	0.004123588	0.01897	0.01141	Adh7/Sca	4
GO:1902991	GO:1902991	regulation of amyloid precursor protein catabolic proce	4/374	41/23328	0.004123588	0.01897	0.01141	Clu/Spon1	4
GO:0090559	GO:0090559	regulation of membrane permeability	5/374	66/23328	0.004139814	0.01903	0.01144	Lbp/Rasip	5
GO:0002793	GO:0002793	positive regulation of peptide secretion	10/374	230/2332{	0.004161861	0.01911	0.01149	Kcnn4/Cd	10
GO:0045580	GO:0045580	regulation of T cell differentiation	8/374	159/2332{	0.004223227	0.01937	0.01165	Bmp4/Gat	8
GO:0016055	GO:0016055	Wnt signaling pathway	15/374	430/2332{	0.00425963	0.0195	0.01173	Cpe/Sfrp1	15
GO:0032640	GO:0032640	tumor necrosis factor production	7/374	126/2332{	0.004261721	0.0195	0.01173	Clu/Cd36/	7
GO:0051492	GO:0051492	regulation of stress fiber assembly	6/374	95/23328	0.004283708	0.01956	0.01176	Cgnl1/Rgc	6
GO:0055013	GO:0055013	cardiac muscle cell development	6/374	95/23328	0.004283708	0.01956	0.01176	Bmp4/Pro	6
GO:0002495	GO:0002495	antigen processing and presentation of peptide antiger	3/374	21/23328	0.004387439	0.01995	0.012	H2-DMb1/	3
GO:0003177	GO:0003177	pulmonary valve development	3/374	21/23328	0.004387439	0.01995	0.012	Bmp4/Gja	3
GO:0043117	GO:0043117	positive regulation of vascular permeability	3/374	21/23328	0.004387439	0.01995	0.012	Apoe/Pde	3
GO:0051900	GO:0051900	regulation of mitochondrial depolarization	3/374	21/23328	0.004387439	0.01995	0.012	Kdr/Dcn/B	3
GO:0046847	GO:0046847	filopodium assembly	5/374	67/23328	0.004416052	0.02006	0.01206	Dpysl3/Fs	5
GO:0198738	GO:0198738	cell-cell signaling by wnt	15/374	432/2332{	0.004443357	0.02014	0.01211	Cpe/Sfrp1	15
GO:0051101	GO:0051101	regulation of DNA binding	7/374	127/2332{	0.004449849	0.02014	0.01211	Gata3/Id1	7
GO:0090090	GO:0090090	negative regulation of canonical Wnt signaling pathwa	7/374	127/2332{	0.004449849	0.02014	0.01211	Sfrp1/Dkk	7
GO:0001709	GO:0001709	cell fate determination	4/374	42/23328	0.004500941	0.02031	0.01221	Bmp4/Gat	4
GO:0010762	GO:0010762	regulation of fibroblast migration	4/374	42/23328	0.004500941	0.02031	0.01221	Thbs1/Itgt	4
GO:1900077	GO:1900077	negative regulation of cellular response to insulin stim	4/374	42/23328	0.004500941	0.02031	0.01221	Lpl/Ahsg/S	4
GO:0002690	GO:0002690	positive regulation of leukocyte chemotaxis	6/374	96/23328	0.004509058	0.02032	0.01222	Lbp/Thbs1	6
GO:0071706	GO:0071706	tumor necrosis factor superfamily cytokine production	7/374	128/2332{	0.004644182	0.02091	0.01258	Clu/Cd36/	7
GO:2000116	GO:2000116	regulation of cysteine-type endopeptidase activity	10/374	234/2332{	0.004693245	0.02109	0.01269	Perp/Sfrp	10
GO:0002763	GO:0002763	positive regulation of myeloid leukocyte differentiation	5/374	68/23328	0.004705168	0.02109	0.01269	Fos/Kitl/C	5
GO:0032507	GO:0032507	maintenance of protein location in cell	5/374	68/23328	0.004705168	0.02109	0.01269	Tmsb4x/C	5
GO:2001244	GO:2001244	positive regulation of intrinsic apoptotic signaling pathw	5/374	68/23328	0.004705168	0.02109	0.01269	Clu/Nupr1	5
GO:0072175	GO:0072175	epithelial tube formation	8/374	162/2332{	0.004723211	0.02113	0.01271	Sfrp1/Bmp	8
GO:0030900	GO:0030900	forebrain development	14/374	393/2332{	0.004724125	0.02113	0.01271	Ncam1/Br	14
GO:0002709	GO:0002709	regulation of T cell mediated immunity	6/374	97/23328	0.004743023	0.02117	0.01273	Gata3/Cd	6
GO:0010660	GO:0010660	regulation of muscle cell apoptotic process	6/374	97/23328	0.004743023	0.02117	0.01273	Igfbp3/Sfr	6
GO:0031334	GO:0031334	positive regulation of protein-containing complex asse	10/374	235/2332{	0.004834083	0.02155	0.01296	Clu/Cd36/	10
GO:0061844	GO:0061844	antimicrobial humoral immune response mediated by a	7/374	129/2332{	0.004844847	0.02158	0.01298	Cxcl1/Cxc	7
GO:1903169	GO:1903169	regulation of calcium ion transmembrane transport	8/374	163/2332{	0.004899576	0.02176	0.01309	Bmp4/Apl	8
GO:0042771	GO:0042771	intrinsic apoptotic signaling pathway in response to DN	4/374	43/23328	0.004900864	0.02176	0.01309	Nupr1/Cdl	4
GO:1903053	GO:1903053	regulation of extracellular matrix organization	4/374	43/23328	0.004900864	0.02176	0.01309	Fscn1/Tcf	4
GO:0048678	GO:0048678	response to axon injury	5/374	69/23328	0.005007481	0.02213	0.01331	Klk8/Dpys	5
GO:0072111	GO:0072111	cell proliferation involved in kidney development	3/374	22/23328	0.005020451	0.02213	0.01331	Bmp4/Gat	3
GO:0072215	GO:0072215	regulation of metanephros development	3/374	22/23328	0.005020451	0.02213	0.01331	Bmp4/Pdc	3
GO:1900543	GO:1900543	negative regulation of purine nucleotide metabolic proc	3/374	22/23328	0.005020451	0.02213	0.01331	Nupr1/Cbt	3
GO:1901741	GO:1901741	positive regulation of myoblast fusion	3/374	22/23328	0.005020451	0.02213	0.01331	Cxcl12/Cx	3
GO:1903055	GO:1903055	positive regulation of extracellular matrix organization	3/374	22/23328	0.005020451	0.02213	0.01331	Fscn1/Rg	3
GO:2000353	GO:2000353	positive regulation of endothelial cell apoptotic process	3/374	22/23328	0.005020451	0.02213	0.01331	Bmp4/Thb	3
GO:0043588	GO:0043588	skin development	11/374	275/2332{	0.005072551	0.02231	0.01342	Col5a2/Tc	11
GO:0060560	GO:0060560	developmental growth involved in morphogenesis	11/374	275/2332{	0.005072551	0.02231	0.01342	Sfrp1/Bmp	11
GO:0034249	GO:0034249	negative regulation of cellular amide metabolic proces	8/374	164/2332{	0.005080944	0.02232	0.01342	Clu/Spon1	8
GO:0048863	GO:0048863	stem cell differentiation	10/374	237/2332{	0.005125711	0.02249	0.01353	Sfrp1/Bmp	10
GO:0001906	GO:0001906	cell killing	9/374	200/2332{	0.005145131	0.02253	0.01355	Cfh/Cxcl1	9
GO:1902905	GO:1902905	positive regulation of supramolecular fiber organizatior	9/374	200/2332{	0.005145131	0.02253	0.01355	Prox1/Rgc	9

GO:0007409	GO:0007409	axonogenesis	16/374	482/23328	0.005150973	0.02253	0.01355	Ncam1/M	16
GO:0045185	GO:0045185	maintenance of protein location	6/374	99/23328	0.005237569	0.02286	0.01375	Tmsb4x/C	6
GO:0062014	GO:0062014	negative regulation of small molecule metabolic proce	6/374	99/23328	0.005237569	0.02286	0.01375	Dkk3/Apo	6
GO:0051047	GO:0051047	positive regulation of secretion	15/374	440/23328	0.005243975	0.02287	0.01375	Kcnn4/Cd	15
GO:0002761	GO:0002761	regulation of myeloid leukocyte differentiation	7/374	131/23328	0.005265683	0.0229	0.01377	Sfrp1/Rbp	7
GO:0019730	GO:0019730	antimicrobial humoral response	8/374	165/23328	0.005267404	0.0229	0.01377	Cxcl1/Cxc	8
GO:2001236	GO:2001236	regulation of extrinsic apoptotic signaling pathway	8/374	165/23328	0.005267404	0.0229	0.01377	Sfrp1/Bmp	8
GO:0044843	GO:0044843	cell cycle G1/S phase transition	9/374	201/23328	0.005312501	0.02305	0.01386	Fhl1/Rgcc	9
GO:0050792	GO:0050792	regulation of viral process	9/374	201/23328	0.005312501	0.02305	0.01386	Apoe/Prox	9
GO:1903317	GO:1903317	regulation of protein maturation	5/374	70/23328	0.005323306	0.02305	0.01386	Spon1/Thl	5
GO:1903672	GO:1903672	positive regulation of sprouting angiogenesis	4/374	44/23328	0.005323951	0.02305	0.01386	Aplnr/Kdr/	4
GO:0050807	GO:0050807	regulation of synapse organization	11/374	277/23328	0.005349628	0.02314	0.01391	Klk8/Spar	11
GO:0051146	GO:0051146	striated muscle cell differentiation	12/374	317/23328	0.005398266	0.02332	0.01403	Bmp4/Lox	12
GO:0006909	GO:0006909	phagocytosis	13/374	358/23328	0.005443579	0.02349	0.01413	Pecam1/S	13
GO:1901652	GO:1901652	response to peptide	14/374	400/23328	0.005498858	0.02371	0.01426	Vcam1/Cc	14
GO:0006898	GO:0006898	receptor-mediated endocytosis	10/374	240/23328	0.005588826	0.02407	0.01448	Clu/Cd36/	10
GO:0009314	GO:0009314	response to radiation	14/374	401/23328	0.005617432	0.02417	0.01454	Sfrp1/Gat	14
GO:0042733	GO:0042733	embryonic digit morphogenesis	5/374	71/23328	0.005652958	0.02421	0.01456	Bmp4/Sfrp	5
GO:0043407	GO:0043407	negative regulation of MAP kinase activity	5/374	71/23328	0.005652958	0.02421	0.01456	Sfrp1/Bmp	5
GO:0032635	GO:0032635	interleukin-6 production	8/374	167/23328	0.005655969	0.02421	0.01456	Cd36/Lbp/	8
GO:0045834	GO:0045834	positive regulation of lipid metabolic process	8/374	167/23328	0.005655969	0.02421	0.01456	Scarb1/Cc	8
GO:2001242	GO:2001242	regulation of intrinsic apoptotic signaling pathway	8/374	167/23328	0.005655969	0.02421	0.01456	Clu/Nupr1	8
GO:0003176	GO:0003176	aortic valve development	3/374	23/23328	0.005705681	0.02427	0.0146	Bmp4/Gat	3
GO:0010866	GO:0010866	regulation of triglyceride biosynthetic process	3/374	23/23328	0.005705681	0.02427	0.0146	Scarb1/Th	3
GO:0032331	GO:0032331	negative regulation of chondrocyte differentiation	3/374	23/23328	0.005705681	0.02427	0.0146	Bmp4/Efe	3
GO:0045980	GO:0045980	negative regulation of nucleotide metabolic process	3/374	23/23328	0.005705681	0.02427	0.0146	Nupr1/Cbf	3
GO:0048333	GO:0048333	mesodermal cell differentiation	3/374	23/23328	0.005705681	0.02427	0.0146	Bmp4/Sfrp	3
GO:2000810	GO:2000810	regulation of bicellular tight junction assembly	3/374	23/23328	0.005705681	0.02427	0.0146	Cldn5/F11	3
GO:0001841	GO:0001841	neural tube formation	7/374	133/23328	0.005713378	0.02428	0.0146	Sfrp1/Bmp	7
GO:0046777	GO:0046777	protein autophosphorylation	10/374	241/23328	0.005750256	0.0243	0.01462	Bmx/Kdr/F	10
GO:0055006	GO:0055006	cardiac cell development	6/374	101/23328	0.00576888	0.0243	0.01462	Bmp4/Pro	6
GO:0003156	GO:0003156	regulation of animal organ formation	4/374	45/23328	0.005770779	0.0243	0.01462	Bmp4/Pim	4
GO:0010464	GO:0010464	regulation of mesenchymal cell proliferation	4/374	45/23328	0.005770779	0.0243	0.01462	Bmp4/Kdr	4
GO:0060421	GO:0060421	positive regulation of heart growth	4/374	45/23328	0.005770779	0.0243	0.01462	Ncam1/Pr	4
GO:0061756	GO:0061756	leukocyte adhesion to vascular endothelial cell	4/374	45/23328	0.005770779	0.0243	0.01462	Vcam1/Se	4
GO:0090184	GO:0090184	positive regulation of kidney development	4/374	45/23328	0.005770779	0.0243	0.01462	Bmp4/Gat	4
GO:1902743	GO:1902743	regulation of lamellipodium organization	4/374	45/23328	0.005770779	0.0243	0.01462	Fscn1/Pdp	4
GO:2000826	GO:2000826	regulation of heart morphogenesis	4/374	45/23328	0.005770779	0.0243	0.01462	Bmp4/Pim	4
GO:0071216	GO:0071216	cellular response to biotic stimulus	12/374	320/23328	0.005805904	0.02442	0.01469	Cd36/Lbp/	12
GO:0015672	GO:0015672	monovalent inorganic cation transport	16/374	489/23328	0.005891132	0.02476	0.01489	Gja5/Kcnr	16
GO:1902806	GO:1902806	regulation of cell cycle G1/S phase transition	7/374	134/23328	0.005947619	0.02497	0.01502	Fhl1/Rgcc	7
GO:0010657	GO:0010657	muscle cell apoptotic process	6/374	102/23328	0.006048801	0.02534	0.01524	Igfbp3/Sfr	6
GO:0050848	GO:0050848	regulation of calcium-mediated signaling	6/374	102/23328	0.006048801	0.02534	0.01524	Ncam1/Kc	6
GO:0006066	GO:0006066	alcohol metabolic process	12/374	323/23328	0.006237609	0.02605	0.01567	Rbp1/Adh	12
GO:0033344	GO:0033344	cholesterol efflux	4/374	46/23328	0.006241916	0.02605	0.01567	Scarb1/Ap	4
GO:0043618	GO:0043618	regulation of transcription from RNA polymerase II pro	4/374	46/23328	0.006241916	0.02605	0.01567	Cd36/Epa	4
GO:2000404	GO:2000404	regulation of T cell migration	4/374	46/23328	0.006241916	0.02605	0.01567	Cxcl12/Cc	4
GO:0003300	GO:0003300	cardiac muscle hypertrophy	6/374	103/23328	0.006338488	0.0264	0.01588	Rgs4/Lmc	6
GO:0090263	GO:0090263	positive regulation of canonical Wnt signaling pathway	6/374	103/23328	0.006338488	0.0264	0.01588	Sfrp1/Sfrp	6
GO:0031639	GO:0031639	plasminogen activation	3/374	24/23328	0.006444223	0.02668	0.01605	Thbs1/Ser	3
GO:0045736	GO:0045736	negative regulation of cyclin-dependent protein serine/	3/374	24/23328	0.006444223	0.02668	0.01605	Cdkn1a/N	3
GO:0050687	GO:0050687	negative regulation of defense response to virus	3/374	24/23328	0.006444223	0.02668	0.01605	Htra1/Fgl2	3
GO:0051882	GO:0051882	mitochondrial depolarization	3/374	24/23328	0.006444223	0.02668	0.01605	Kdr/Dcn/B	3
GO:0072207	GO:0072207	metanephric epithelium development	3/374	24/23328	0.006444223	0.02668	0.01605	Hes1/Pdg	3
GO:0090330	GO:0090330	regulation of platelet aggregation	3/374	24/23328	0.006444223	0.02668	0.01605	Pdpn/F11	3
GO:0030833	GO:0030833	regulation of actin filament polymerization	8/374	171/23328	0.006498245	0.02688	0.01616	Pecam1/T	8
GO:0071222	GO:0071222	cellular response to lipopolysaccharide	11/374	285/23328	0.006579623	0.02719	0.01635	Cd36/Lbp/	11
GO:0007187	GO:0007187	G protein-coupled receptor signaling pathway, coupled	10/374	246/23328	0.006612765	0.0273	0.01642	Adgrl4/Ad	10
GO:0060349	GO:0060349	bone morphogenesis	6/374	104/23328	0.00663813	0.02737	0.01646	Bmp4/Scx	6
GO:0010718	GO:0010718	positive regulation of epithelial to mesenchymal transit	4/374	47/23328	0.006737913	0.02776	0.01669	Bmp4/Rgc	4
GO:0043281	GO:0043281	regulation of cysteine-type endopeptidase activity invo	9/374	209/23328	0.006807979	0.02802	0.01685	Sfrp2/Thb	9
GO:0031647	GO:0031647	regulation of protein stability	11/374	287/23328	0.006919278	0.02845	0.01711	Clu/Gpihb	11
GO:0048562	GO:0048562	embryonic organ morphogenesis	12/374	328/23328	0.007013223	0.02881	0.01732	Bmp4/Gat	12
GO:0055007	GO:0055007	cardiac muscle cell differentiation	7/374	139/23328	0.007227893	0.02938	0.01767	Bmp4/Pro	7
GO:0010165	GO:0010165	response to X-ray	3/374	25/23328	0.007237079	0.02938	0.01767	Sfrp1/Gat	3
GO:0010894	GO:0010894	negative regulation of steroid biosynthetic process	3/374	25/23328	0.007237079	0.02938	0.01767	Dkk3/Apo	3
GO:0035024	GO:0035024	negative regulation of Rho protein signal transduction	3/374	25/23328	0.007237079	0.02938	0.01767	Itgb1/Rasi	3
GO:0043567	GO:0043567	regulation of insulin-like growth factor receptor signalin	3/374	25/23328	0.007237079	0.02938	0.01767	Igfbp3/Igf	3
GO:0045939	GO:0045939	negative regulation of steroid metabolic process	3/374	25/23328	0.007237079	0.02938	0.01767	Dkk3/Apo	3
GO:0051894	GO:0051894	positive regulation of focal adhesion assembly	3/374	25/23328	0.007237079	0.02938	0.01767	Kdr/Tek/T	3
GO:0070633	GO:0070633	transepithelial transport	3/374	25/23328	0.007237079	0.02938	0.01767	Ednrb/Aqp	3
GO:1901739	GO:1901739	regulation of myoblast fusion	3/374	25/23328	0.007237079	0.02938	0.01767	Cxcl12/Cx	3
GO:1902230	GO:1902230	negative regulation of intrinsic apoptotic signaling path	3/374	25/23328	0.007237079	0.02938	0.01767	Clu/Cxcl1	3
GO:1902993	GO:1902993	positive regulation of amyloid precursor protein catabo	3/374	25/23328	0.007237079	0.02938	0.01767	Clu/Spon1	3
GO:0003382	GO:0003382	epithelial cell morphogenesis	4/374	48/23328	0.007259308	0.02938	0.01767	Pecam1/C	4
GO:0045429	GO:0045429	positive regulation of nitric oxide biosynthetic process	4/374	48/23328	0.007259308	0.02938	0.01767	Clu/Cd36/	4
GO:0090279	GO:0090279	regulation of calcium ion import	4/374	48/23328	0.007259308	0.02938	0.01767	Cxcl12/He	4
GO:1903727	GO:1903727	positive regulation of phospholipid metabolic process	4/374	48/23328	0.007259308	0.02938	0.01767	Flt1/Tek/A	4
GO:0050803	GO:0050803	regulation of synapse structure or activity	11/374	289/23328	0.007272501	0.02941	0.01769	Klk8/Spar	11
GO:0030258	GO:0030258	lipid modification	10/374	250/23328	0.007372476	0.02978	0.01791	Adh7/Eph	10
GO:0007612	GO:0007612	learning	8/374	175/23328	0.007432317	0.02994	0.01801	Btg2/Ptgs	8
GO:0035107	GO:0035107	appendage morphogenesis	8/374	175/23328	0.007432317	0.02994	0.01801	Bmp4/Gja	8
GO:0035108	GO:0035108	limb morphogenesis	8/374	175/23328	0.007432317	0.02994	0.01801	Bmp4/Gja	8
GO:0007189	GO:0007189	adenylate cyclase-activating G protein-coupled recepto	7/374	140/23328	0.007506665	0.0302	0.01816	Adgrl4/Ad	7
GO:0006919	GO:0006919	activation of cysteine-type endopeptidase activity invol	5/374	76/23328	0.007519429	0.0302	0.01816	F2r/Acer2	5
GO:0050663	GO:0050663	cytokine secretion	5/374	76/23328	0.007519429	0.0302	0.01816	Cd36/Rgc	5
GO:1901215	GO:1901215	negative regulation of neuron death	10/374	251/23328	0.007572545	0.03038	0.01827	Kdr/Apoe/	10
GO:1903426	GO:1903426	regulation of reactive oxygen species biosynthetic proc	6/374	107/23328	0.007598686	0.03046	0.01832	Clu/Ptgis/	6
GO:0002819	GO:0002819	regulation of adaptive immune response	9/374	213/23328	0.007667302	0.03071	0.01847	Gata3/Cd	9
GO:0021537	GO:0021537	telencephalon development	10/374	252/23328	0.00777678	0.03108	0.0187	Bmp4/Pro	10
GO:0007157	GO:0007157	heterophilic cell-cell adhesion via plasma membrane c	4/374	49/23328	0.007806623	0.03108	0.0187	Vcam1/Se	4
GO:1903018	GO:1903018	regulation of glycoprotein metabolic process	4/374	49/23328	0.007806623	0.03108	0.0187	Bace2/Cs	4
GO:1904407	GO:1904407	positive regulation of nitric oxide metabolic process	4/374	49/23328	0.007806623	0.03108	0.0187	Clu/Cd36/	4
GO:1905953	GO:1905953	negative regulation of lipid localization	4/374	49/23328	0.007806623	0.03108	0.0187	Igfbp3/Ap	4
GO:2000725	GO:2000725	regulation of cardiac muscle cell differentiation	4/374	49/23328	0.007806623	0.03108	0.0187	Bmp4/Rgs	4
GO:0016202	GO:0016202	regulation of striated muscle tissue development	8/374	177/23328	0.007935638	0.03153	0.01896	Ncam1/Br	8
GO:0001756	GO:0001756	somitogenesis	5/374	77/23328	0.007938487	0.03153	0.01896	Sfrp1/Sfrp	5
GO:0006641	GO:0006641	triglyceride metabolic process	6/374	108/23328	0.007940044	0.03153	0.01896	Scarb1/Cc	6
GO:0048705	GO:0048705	skeletal system morphogenesis	10/374	253/23328	0.00798524	0.03168	0.01905	Sfrp1/Bmp	10
GO:0009713	GO:0009713	catechol-containing compound biosynthetic process	3/374	26/23328	0.008085158	0.03192	0.0192	Gata3/Epa	3
GO:0034104	GO:0034104	negative regulation of tissue remodeling	3/374	26/23328	0.008085158	0.03192	0.0192	Sfrp1/Cd3	3
GO:0042423	GO:0042423	catecholamine biosynthetic process	3/374	26/23328	0.008085158	0.03192	0.0192	Gata3/Epa	3
GO:0043171	GO:0043171	peptide catabolic process	3/374	26/23328	0.008085158	0.03192	0.0192	Cpe/Ece1	3
GO:0071549	GO:0071549	cellular response to dexamethasone stimulus	3/374	26/23328	0.008085158	0.03192	0.0192	Bmp4/Aqp	3
GO:0008154	GO:0008154	actin polymerization or depolymerization	9/374	215/23328	0.008126833	0.03205	0.01928	Pecam1/T	9
GO:0051897	GO:0051897	positive regulation of protein kinase B signaling	6/374	109/23328	0.008292299	0.03268	0.01965	Gata3/Thb	6
GO:0008625	GO:0008625	extrinsic apoptotic signaling pathway via death domain	5/374	78/23328	0.008373493	0.03278	0.01972	Sfrp1/Sfrp	5
GO:0046785	GO:0046785	microtubule polymerization	5/374	78/23328	0.008373493	0.03278	0.01972	Cdh5/Gda	5
GO:0050810	GO:0050810	regulation of steroid biosynthetic process	5/374	78/23328	0.008373493	0.03278	0.01972	Dkk3/Igfb	5
GO:0060976	GO:0060976	coronary vasculature development	5/374	78/23328	0.008373493	0.03278	0.01972	Bmp4/Apl	5
GO:0034332	GO:0034332	adherens junction organization	4/374	50/23328	0.008380367	0.03278	0.01972	Cdh11/Cd	4
GO:0043392	GO:0043392	negative regulation of DNA binding	4/374	50/23328	0.008380367	0.03278	0.01972	Id1/Jun/Id	4
GO:0061005	GO:0061005	cell differentiation involved in kidney development	4/374	50/23328	0.008380367	0.03278	0.01972	Bmp4/Gat	4
GO:0030326	GO:0030326	embryonic limb morphogenesis	7/374	143/23328	0.008390728	0.03278	0.01972	Bmp4/Gja	7
GO:0035113	GO:0035113	embryonic appendage morphogenesis	7/374	143/23328	0.008390728	0.03278	0.01972	Bmp4/Gja	7
GO:0071219	GO:0071219	cellular response to molecule of bacterial origin	11/374	295/23328	0.008417165	0.03283	0.01974	Cd36/Lbp/	11
GO:0009991	GO:0009991	response to extracellular stimulus	13/374	378/23328	0.008417549	0.03283	0.01974	Rbp1/Vca	13
GO:0031668	GO:0031668	cellular response to extracellular stimulus	9/374	217/23328	0.008607035	0.03354	0.02017	Vcam1/Sf	9
GO:0007156	GO:0007156	homophilic cell adhesion via plasma membrane adhes	6/374	110/23328	0.008655639	0.03369	0.02026	Pecam1/C	6
GO:0002708	GO:0002708	positive regulation of lymphocyte mediated immunity	7/374	144/23328	0.008701761	0.03384	0.02035	Gata3/Cd	7
GO:0032330	GO:0032330	regulation of chondrocyte differentiation	4/374	51/23328	0.00898103	0.03464	0.02083	Bmp4/Ma	4
GO:0042987	GO:0042987	amyloid precursor protein catabolic process	4/374	51/23328	0.00898103	0.03464	0.02083	Clu/Spon1	4
GO:0043620	GO:0043620	regulation of DNA-templated transcription in response	4/374	51/23328	0.00898103	0.03464	0.02083	Cd36/Epa	4
GO:0014829	GO:0014829	vascular associated smooth muscle contraction	3/374	27/23328	0.008989283	0.03464	0.02083	Ednrb/Cd	3
GO:0033688	GO:0033688	regulation of osteoblast proliferation	3/374	27/23328	0.008989283	0.03464	0.02083	Sfrp1/Fbl	3
GO:0035809	GO:0035809	regulation of urine volume	3/374	27/23328	0.008989283	0.03464	0.02083	Ednrb/Adr	3
GO:0045730	GO:0045730	respiratory burst	3/374	27/23328	0.008989283	0.03464	0.02083	Lbp/Cd24	3
GO:0051767	GO:0051767	nitric-oxide synthase biosynthetic process	3/374	27/23328	0.008989283	0.03464	0.02083	Kdr/Akap1	3
GO:0051769	GO:0051769	regulation of nitric-oxide synthase biosynthetic process	3/374	27/23328	0.008989283	0.03464	0.02083	Kdr/Akap1	3

GO:0090023	GO:0090023	positive regulation of neutrophil chemotaxis	3/374	27/23328	0.008989283	0.03464	0.02083	Lbp/Cd74/	3
GO:0048634	GO:0048634	regulation of muscle organ development	8/374	181/23328	0.00901859	0.03467	0.02085	Ncam1/Br	8
GO:0006633	GO:0006633	fatty acid biosynthetic process	7/374	145/23328	0.009021178	0.03467	0.02085	Ptgis/Mgll	7
GO:0001843	GO:0001843	neural tube closure	6/374	111/23328	0.009030247	0.03467	0.02085	Sfrp1/Bmp	6
GO:0019218	GO:0019218	regulation of steroid metabolic process	6/374	111/23328	0.009030247	0.03467	0.02085	Dkk3/Eph	6
GO:0051402	GO:0051402	neuron apoptotic process	11/374	298/23328	0.009039549	0.03467	0.02085	Kdr/Gata3	11
GO:1901653	GO:1901653	cellular response to peptide	11/374	299/23328	0.00925473	0.03546	0.02133	Vcam1/Cc	11
GO:1901862	GO:1901862	negative regulation of muscle tissue development	5/374	80/23328	0.0092925	0.03557	0.0214	Bmp4/Rgs	5
GO:0043524	GO:0043524	negative regulation of neuron apoptotic process	8/374	182/23328	0.009305772	0.03559	0.02141	Kdr/Apoe/	8
GO:0060606	GO:0060606	tube closure	6/374	112/23328	0.009416308	0.03598	0.02164	Sfrp1/Bmp	6
GO:0016331	GO:0016331	morphogenesis of embryonic epithelium	8/374	183/23328	0.009599718	0.03662	0.02202	Sfrp1/Bmp	8
GO:0032757	GO:0032757	positive regulation of interleukin-8 production	4/374	52/23328	0.009609091	0.03662	0.02202	Cd2/Lbp/F	4
GO:0045104	GO:0045104	intermediate filament cytoskeleton organization	4/374	52/23328	0.009609091	0.03662	0.02202	Krt18/Nes	4
GO:0002824	GO:0002824	positive regulation of adaptive immune response base	7/374	147/23328	0.00968568	0.03684	0.02216	Gata3/Cd	7
GO:0035270	GO:0035270	endocrine system development	7/374	147/23328	0.00968568	0.03684	0.02216	Clu/Bmp4	7
GO:0030100	GO:0030100	regulation of endocytosis	9/374	222/23328	0.00990173	0.03744	0.02252	Clu/Cd36/	9
GO:0002693	GO:0002693	positive regulation of cellular extravasation	3/374	28/23328	0.00995019	0.03744	0.02252	Pecam1/S	3
GO:0010460	GO:0010460	positive regulation of heart rate	3/374	28/23328	0.00995019	0.03744	0.02252	Rgs4/Adm	3
GO:0030224	GO:0030224	monocyte differentiation	3/374	28/23328	0.00995019	0.03744	0.02252	Bmp4/Cd7	3
GO:0034311	GO:0034311	diol metabolic process	3/374	28/23328	0.00995019	0.03744	0.02252	Plpp3/Plp	3
GO:0035987	GO:0035987	endodermal cell differentiation	3/374	28/23328	0.00995019	0.03744	0.02252	Col5a2/Fr	3
GO:0045648	GO:0045648	positive regulation of erythrocyte differentiation	3/374	28/23328	0.00995019	0.03744	0.02252	Hmgb2/Id	3
GO:0060039	GO:0060039	pericardium development	3/374	28/23328	0.00995019	0.03744	0.02252	Vcam1/Pc	3
GO:0060445	GO:0060445	branching involved in salivary gland morphogenesis	3/374	28/23328	0.00995019	0.03744	0.02252	Pdgfa/Tgf	3
GO:1901532	GO:1901532	regulation of hematopoietic progenitor cell differentiatio	3/374	28/23328	0.00995019	0.03744	0.02252	Kdr/Flt1/H	3
GO:1903131	GO:1903131	mononuclear cell differentiation	3/374	28/23328	0.00995019	0.03744	0.02252	Bmp4/Cd7	3
GO:1903671	GO:1903671	negative regulation of sprouting angiogenesis	3/374	28/23328	0.00995019	0.03744	0.02252	Thbs1/Mm	3
GO:0070661	GO:0070661	leukocyte proliferation	12/374	344/23328	0.010016002	0.03765	0.02265	Clu/Vcam	12
GO:0002705	GO:0002705	positive regulation of leukocyte mediated immunity	8/374	185/23328	0.010208278	0.03834	0.02306	Gata3/Cx	8
GO:0031103	GO:0031103	axon regeneration	4/374	53/23328	0.010265009	0.03842	0.0231	Klk8/Mmp	4
GO:0031113	GO:0031113	regulation of microtubule polymerization	4/374	53/23328	0.010265009	0.03842	0.0231	Cdh5/Gda	4
GO:0045103	GO:0045103	intermediate filament-based process	4/374	53/23328	0.010265009	0.03842	0.0231	Krt18/Nes	4
GO:0048713	GO:0048713	regulation of oligodendrocyte differentiation	4/374	53/23328	0.010265009	0.03842	0.0231	Bmp4/Hes	4
GO:0046660	GO:0046660	female sex differentiation	7/374	149/23328	0.010385248	0.03883	0.02335	Sfrp1/Bmp	7
GO:0003149	GO:0003149	membranous septum morphogenesis	2/374	10/23328	0.010594778	0.03909	0.02351	Bmp4/Id2	2
GO:0003211	GO:0003211	cardiac ventricle formation	2/374	10/23328	0.010594778	0.03909	0.02351	Sox4/Han	2
GO:0006707	GO:0006707	cholesterol catabolic process	2/374	10/23328	0.010594778	0.03909	0.02351	Scarb1/Ap	2
GO:0006977	GO:0006977	DNA damage response, signal transduction by p53 cla	2/374	10/23328	0.010594778	0.03909	0.02351	Sox4/Cdk	2
GO:0016127	GO:0016127	sterol catabolic process	2/374	10/23328	0.010594778	0.03909	0.02351	Scarb1/Ap	2
GO:0060297	GO:0060297	regulation of sarcomere organization	2/374	10/23328	0.010594778	0.03909	0.02351	Prox1/Edr	2
GO:0060907	GO:0060907	positive regulation of macrophage cytokine production	2/374	10/23328	0.010594778	0.03909	0.02351	Cd36/Cd7	2
GO:0070391	GO:0070391	response to lipoteichoic acid	2/374	10/23328	0.010594778	0.03909	0.02351	Cd36/Lbp	2
GO:0071223	GO:0071223	cellular response to lipoteichoic acid	2/374	10/23328	0.010594778	0.03909	0.02351	Cd36/Lbp	2
GO:0090091	GO:0090091	positive regulation of extracellular matrix disassembly	2/374	10/23328	0.010594778	0.03909	0.02351	Fscn1/Pdp	2
GO:0099541	GO:0099541	trans-synaptic signaling by lipid	2/374	10/23328	0.010594778	0.03909	0.02351	F2r/Fabp5	2
GO:0099542	GO:0099542	trans-synaptic signaling by endocannabinoid	2/374	10/23328	0.010594778	0.03909	0.02351	F2r/Fabp5	2
GO:2000489	GO:2000489	regulation of hepatic stellate cell activation	2/374	10/23328	0.010594778	0.03909	0.02351	Cygb/Pdg	2
GO:2000543	GO:2000543	positive regulation of gastrulation	2/374	10/23328	0.010594778	0.03909	0.02351	Scx/Gja1	2
GO:2000551	GO:2000551	regulation of T-helper 2 cell cytokine production	2/374	10/23328	0.010594778	0.03909	0.02351	Gata3/Rsa	2
GO:0090100	GO:0090100	positive regulation of transmembrane receptor protein	6/374	115/23328	0.010645022	0.03924	0.0236	Bmp4/Kdr	6
GO:0001738	GO:0001738	morphogenesis of a polarized epithelium	5/374	83/23328	0.010797722	0.03963	0.02383	Sfrp1/Cthr	5
GO:0045844	GO:0045844	positive regulation of striated muscle tissue developme	5/374	83/23328	0.010797722	0.03963	0.02383	Ncam1/Br	5
GO:0048636	GO:0048636	positive regulation of muscle organ development	5/374	83/23328	0.010797722	0.03963	0.02383	Ncam1/Br	5
GO:1903901	GO:1903901	negative regulation of viral life cycle	5/374	83/23328	0.010797722	0.03963	0.02383	Prox1/Rsa	5
GO:1904427	GO:1904427	positive regulation of calcium ion transmembrane trans	5/374	83/23328	0.010797722	0.03963	0.02383	Bmp4/Apl	5
GO:0030041	GO:0030041	actin filament polymerization	8/374	187/23328	0.010845015	0.03973	0.02389	Pecam1/T	8
GO:0034767	GO:0034767	positive regulation of ion transmembrane transport	8/374	187/23328	0.010845015	0.03973	0.02389	Bmp4/Apl	8
GO:0014014	GO:0014014	negative regulation of gliogenesis	4/374	54/23328	0.01094923	0.03983	0.02396	Bmp4/Hes	4
GO:0045687	GO:0045687	positive regulation of glial cell differentiation	4/374	54/23328	0.01094923	0.03983	0.02396	Hes1/Id2/	4
GO:0048546	GO:0048546	digestive tract morphogenesis	4/374	54/23328	0.01094923	0.03983	0.02396	Sfrp1/Bmp	4
GO:0055025	GO:0055025	positive regulation of cardiac muscle tissue developme	4/374	54/23328	0.01094923	0.03983	0.02396	Ncam1/Br	4
GO:1900271	GO:1900271	regulation of long-term synaptic potentiation	4/374	54/23328	0.01094923	0.03983	0.02396	Apoe/Reln	4
GO:0003416	GO:0003416	endochondral bone growth	3/374	29/23328	0.010968536	0.03983	0.02396	Kdr/Thbs1	3
GO:0061050	GO:0061050	regulation of cell growth involved in cardiac muscle cel	3/374	29/23328	0.010968536	0.03983	0.02396	Rgs4/Pi16	3
GO:0071548	GO:0071548	response to dexamethasone	3/374	29/23328	0.010968536	0.03983	0.02396	Bmp4/Aqp	3
GO:0071624	GO:0071624	positive regulation of granulocyte chemotaxis	3/374	29/23328	0.010968536	0.03983	0.02396	Lbp/Cd74	3
GO:0150117	GO:0150117	positive regulation of cell-substrate junction organizatio	3/374	29/23328	0.010968536	0.03983	0.02396	Kdr/Tek/T	3
GO:0008064	GO:0008064	regulation of actin polymerization or depolymerization	8/374	188/23328	0.011174181	0.04054	0.02438	Pecam1/T	8
GO:1901863	GO:1901863	positive regulation of muscle tissue development	5/374	84/23328	0.011334348	0.04109	0.02471	Ncam1/Br	5
GO:0070665	GO:0070665	positive regulation of leukocyte proliferation	7/374	152/23328	0.011502554	0.04166	0.02505	Vcam1/Cc	7
GO:0001764	GO:0001764	neuron migration	8/374	189/23328	0.011510671	0.04166	0.02505	Gata3/Cx	8
GO:0010769	GO:0010769	regulation of cell morphogenesis involved in differentia	12/374	351/23328	0.011607678	0.04197	0.02524	Mgll/Apoe	12
GO:0010665	GO:0010665	regulation of cardiac muscle cell apoptotic process	4/374	55/23328	0.011662181	0.04213	0.02534	Igfbp3/Sfr	4
GO:0055002	GO:0055002	striated muscle cell development	8/374	190/23328	0.011854575	0.04277	0.02572	Bmp4/Lox	8
GO:0002718	GO:0002718	regulation of cytokine production involved in immune r	5/374	85/23328	0.011888861	0.04277	0.02572	Gata3/Cd	5
GO:0014910	GO:0014910	regulation of smooth muscle cell migration	5/374	85/23328	0.011888861	0.04277	0.02572	Tmsb4x/Ic	5
GO:0030203	GO:0030203	glycosaminoglycan metabolic process	5/374	85/23328	0.011888861	0.04277	0.02572	Bgn/Itih5/	5
GO:0030301	GO:0030301	cholesterol transport	5/374	85/23328	0.011888861	0.04277	0.02572	Scarb1/Cc	5
GO:0014020	GO:0014020	primary neural tube formation	6/374	118/23328	0.011983287	0.04299	0.02586	Sfrp1/Bmp	6
GO:2000177	GO:2000177	regulation of neural precursor cell proliferation	6/374	118/23328	0.011983287	0.04299	0.02586	Prox1/Btg	6
GO:0006921	GO:0006921	cellular component disassembly involved in execution	3/374	30/23328	0.012044899	0.04299	0.02586	Igfbp3/Cd	3
GO:0010874	GO:0010874	regulation of cholesterol efflux	3/374	30/23328	0.012044899	0.04299	0.02586	Apoe/Nfkb	3
GO:0010955	GO:0010955	negative regulation of protein processing	3/374	30/23328	0.012044899	0.04299	0.02586	Thbs1/Ctl	3
GO:0043552	GO:0043552	positive regulation of phosphatidylinositol 3-kinase acti	3/374	30/23328	0.012044899	0.04299	0.02586	Flt1/Tek/F	3
GO:0043951	GO:0043951	negative regulation of cAMP-mediated signaling	3/374	30/23328	0.012044899	0.04299	0.02586	Aplnr/Pde	3
GO:0060143	GO:0060143	positive regulation of syncytium formation by plasma m	3/374	30/23328	0.012044899	0.04299	0.02586	Cxcl12/Cx	3
GO:1903318	GO:1903318	negative regulation of protein maturation	3/374	30/23328	0.012044899	0.04299	0.02586	Thbs1/Ctl	3
GO:0030832	GO:0030832	regulation of actin filament length	8/374	191/23328	0.012205986	0.04353	0.02618	Pecam1/T	8
GO:0043523	GO:0043523	regulation of neuron apoptotic process	10/374	270/23328	0.012229929	0.04358	0.02621	Kdr/Gata3	10
GO:0014013	GO:0014013	regulation of gliogenesis	7/374	154/23328	0.012294179	0.04377	0.02632	Bmp4/Vim	7
GO:0001523	GO:0001523	retinoid metabolic process	4/374	56/23328	0.012404276	0.04409	0.02651	Rbp1/Adh	4
GO:0045777	GO:0045777	positive regulation of blood pressure	4/374	56/23328	0.012404276	0.04409	0.02651	Ephx2/Nr2	4
GO:0015918	GO:0015918	sterol transport	5/374	86/23328	0.01246152	0.04421	0.02659	Scarb1/Cc	5
GO:1901379	GO:1901379	regulation of potassium ion transmembrane transport	5/374	86/23328	0.01246152	0.04421	0.02659	Fhl1/Atp1	5
GO:2001234	GO:2001234	negative regulation of apoptotic signaling pathway	9/374	231/23328	0.012595316	0.04455	0.02679	Clu/Bmp4	9
GO:0001867	GO:0001867	complement activation, lectin pathway	2/374	11/23328	0.012812955	0.04455	0.02679	Mfap4/Ser	2
GO:0002138	GO:0002138	retinoic acid biosynthetic process	2/374	11/23328	0.012812955	0.04455	0.02679	Rbp1/Cyp	2
GO:0003266	GO:0003266	regulation of secondary heart field cardioblast prolifera	2/374	11/23328	0.012812955	0.04455	0.02679	Hes1/Han	2
GO:0016102	GO:0016102	diterpenoid biosynthetic process	2/374	11/23328	0.012812955	0.04455	0.02679	Rbp1/Cyp	2
GO:0031077	GO:0031077	post-embryonic camera-type eye development	2/374	11/23328	0.012812955	0.04455	0.02679	Kdr/Flt1	2
GO:0033625	GO:0033625	positive regulation of integrin activation	2/374	11/23328	0.012812955	0.04455	0.02679	Cd24a/Ra	2
GO:0043568	GO:0043568	positive regulation of insulin-like growth factor receptor	2/374	11/23328	0.012812955	0.04455	0.02679	Igfbp3/Igf	2
GO:0043619	GO:0043619	regulation of transcription from RNA polymerase II pro	2/374	11/23328	0.012812955	0.04455	0.02679	Cd36/Epa	2
GO:0045647	GO:0045647	negative regulation of erythrocyte differentiation	2/374	11/23328	0.012812955	0.04455	0.02679	Lmo2/Zfp	2
GO:0046689	GO:0046689	response to mercury ion	2/374	11/23328	0.012812955	0.04455	0.02679	Fn1/Aqp1	2
GO:0048563	GO:0048563	post-embryonic animal organ morphogenesis	2/374	11/23328	0.012812955	0.04455	0.02679	Kdr/Flt1	2
GO:0051901	GO:0051901	positive regulation of mitochondrial depolarization	2/374	11/23328	0.012812955	0.04455	0.02679	Kdr/Dcn	2
GO:0060020	GO:0060020	Bergmann glial cell differentiation	2/374	11/23328	0.012812955	0.04455	0.02679	Vim/Plpp3	2
GO:0071732	GO:0071732	cellular response to nitric oxide	2/374	11/23328	0.012812955	0.04455	0.02679	Thbs1/Aq	2
GO:0072102	GO:0072102	glomerulus morphogenesis	2/374	11/23328	0.012812955	0.04455	0.02679	Bmp4/Not	2
GO:0072178	GO:0072178	nephric duct morphogenesis	2/374	11/23328	0.012812955	0.04455	0.02679	Bmp4/Gat	2
GO:0072431	GO:0072431	signal transduction involved in mitotic G1 DNA damage	2/374	11/23328	0.012812955	0.04455	0.02679	Sox4/Cdk	2
GO:0097278	GO:0097278	complement-dependent cytotoxicity	2/374	11/23328	0.012812955	0.04455	0.02679	Cfh/C3	2
GO:1901030	GO:1901030	positive regulation of mitochondrial outer membrane pe	2/374	11/23328	0.012812955	0.04455	0.02679	Chchd10/	2
GO:1902400	GO:1902400	intracellular signal transduction involved in G1 DNA da	2/374	11/23328	0.012812955	0.04455	0.02679	Sox4/Cdk	2
GO:1903365	GO:1903365	regulation of fear response	2/374	11/23328	0.012812955	0.04455	0.02679	Apoe/Gja1	2
GO:2000121	GO:2000121	regulation of removal of superoxide radicals	2/374	11/23328	0.012812955	0.04455	0.02679	Fbln5/Cd3	2
GO:2000822	GO:2000822	regulation of behavioral fear response	2/374	11/23328	0.012812955	0.04455	0.02679	Apoe/Gja1	2
GO:0006584	GO:0006584	catecholamine metabolic process	4/374	57/23328	0.01317591	0.04519	0.02718	Gata3/Ly6	4
GO:0006749	GO:0006749	glutathione metabolic process	4/374	57/23328	0.01317591	0.04519	0.02718	Gstm1/Mc	4
GO:0009712	GO:0009712	catechol-containing compound metabolic process	4/374	57/23328	0.01317591	0.04519	0.02718	Gata3/Ly6	4
GO:0035567	GO:0035567	non-canonical Wnt signaling pathway	4/374	57/23328	0.01317591	0.04519	0.02718	Sfrp1/Cthr	4
GO:0046622	GO:0046622	positive regulation of organ growth	4/374	57/23328	0.01317591	0.04519	0.02718	Ncam1/Pr	4
GO:0051591	GO:0051591	response to cAMP	4/374	57/23328	0.01317591	0.04519	0.02718	Aldh3a1/A	4
GO:0055078	GO:0055078	sodium ion homeostasis	4/374	57/23328	0.01317591	0.04519	0.02718	Ednrb/Atp	4
GO:0071622	GO:0071622	regulation of granulocyte chemotaxis	4/374	57/23328	0.01317591	0.04519	0.02718	Lbp/Thbs1	4
GO:2000648	GO:2000648	positive regulation of stem cell proliferation	4/374	57/23328	0.01317591	0.04519	0.02718	Bmp4/Cxc	4
GO:0002026	GO:0002026	regulation of the force of heart contraction	3/374	31/23328	0.01317978	0.04519	0.02718	Nos3/Adm	3
GO:0002063	GO:0002063	chondrocyte development	3/374	31/23328	0.01317978	0.04519	0.02718	Sfrp2/Ecm	3

GO:0032373	GO:0032373	positive regulation of sterol transport	3/374	31/23328	0.01317978	0.04519	0.02718	Apoe/Nfkb	3
GO:0032376	GO:0032376	positive regulation of cholesterol transport	3/374	31/23328	0.01317978	0.04519	0.02718	Apoe/Nfkb	3
GO:0042481	GO:0042481	regulation of odontogenesis	3/374	31/23328	0.01317978	0.04519	0.02718	Bmp4/Edr	3
GO:0048535	GO:0048535	lymph node development	3/374	31/23328	0.01317978	0.04519	0.02718	Pdpn/Id2/	3
GO:0095500	GO:0095500	acetylcholine receptor signaling pathway	3/374	31/23328	0.01317978	0.04519	0.02718	Ly6c1/Ly6	3
GO:1900117	GO:1900117	regulation of execution phase of apoptosis	3/374	31/23328	0.01317978	0.04519	0.02718	Ptgis/Igfb	3
GO:0021915	GO:0021915	neural tube development	8/374	194/23328	0.013306175	0.04558	0.02741	Sfrp1/Bm	8
GO:0062012	GO:0062012	regulation of small molecule metabolic process	12/374	358/23328	0.013387445	0.04582	0.02756	Dkk3/Eph	12
GO:0008593	GO:0008593	regulation of Notch signaling pathway	5/374	88/23328	0.013662283	0.04665	0.02806	Nrarp/Hes	5
GO:0032526	GO:0032526	response to retinoic acid	5/374	88/23328	0.013662283	0.04665	0.02806	Adh1/Tek/	5
GO:0062207	GO:0062207	regulation of pattern recognition receptor signaling patl	5/374	88/23328	0.013662283	0.04665	0.02806	Cd36/Lbp/	5
GO:2000241	GO:2000241	regulation of reproductive process	8/374	195/23328	0.013688527	0.0467	0.02809	Sfrp1/Bm	8
GO:0031109	GO:0031109	microtubule polymerization or depolymerization	6/374	122/23328	0.013946188	0.04734	0.02847	Cdh5/Gda	6
GO:0045807	GO:0045807	positive regulation of endocytosis	6/374	122/23328	0.013946188	0.04734	0.02847	Clu/Cd36/	6
GO:0001754	GO:0001754	eye photoreceptor cell differentiation	4/374	58/23328	0.013977462	0.04734	0.02847	Cfh/Thy1/	4
GO:0010662	GO:0010662	regulation of striated muscle cell apoptotic process	4/374	58/23328	0.013977462	0.04734	0.02847	Igfbp3/Sfr	4
GO:0010761	GO:0010761	fibroblast migration	4/374	58/23328	0.013977462	0.04734	0.02847	Thbs1/Itg	4
GO:0016101	GO:0016101	diterpenoid metabolic process	4/374	58/23328	0.013977462	0.04734	0.02847	Rbp1/Adh	4
GO:0032663	GO:0032663	regulation of interleukin-2 production	4/374	58/23328	0.013977462	0.04734	0.02847	Gata3/Prn	4
GO:0035456	GO:0035456	response to interferon-beta	4/374	58/23328	0.013977462	0.04734	0.02847	Ifi203/Mnd	4
GO:0048016	GO:0048016	inositol phosphate-mediated signaling	4/374	58/23328	0.013977462	0.04734	0.02847	Lmcd1/Pr	4
GO:0007519	GO:0007519	skeletal muscle tissue development	8/374	196/23328	0.01407884	0.0476	0.02863	Eln/Scx/N	8
GO:0043491	GO:0043491	protein kinase B signaling	8/374	196/23328	0.01407884	0.0476	0.02863	Lox/Kdr/G	8
GO:0040036	GO:0040036	regulation of fibroblast growth factor receptor signaling	3/374	32/23328	0.01437361	0.04836	0.02909	Gata3/Thb	3
GO:0043901	GO:0043901	negative regulation of multi-organism process	3/374	32/23328	0.01437361	0.04836	0.02909	Arhgdib/B	3
GO:0045577	GO:0045577	regulation of B cell differentiation	3/374	32/23328	0.01437361	0.04836	0.02909	Sfrp1/Cd2	3
GO:0051150	GO:0051150	regulation of smooth muscle cell differentiation	3/374	32/23328	0.01437361	0.04836	0.02909	Tmsb4x/B	3
GO:0055022	GO:0055022	negative regulation of cardiac muscle tissue growth	3/374	32/23328	0.01437361	0.04836	0.02909	Rgs4/Pi16	3
GO:0061117	GO:0061117	negative regulation of heart growth	3/374	32/23328	0.01437361	0.04836	0.02909	Rgs4/Pi16	3
GO:0050867	GO:0050867	positive regulation of cell activation	15/374	496/23328	0.01466343	0.0493	0.02965	Vcam1/Se	15
GO:0045786	GO:0045786	negative regulation of cell cycle	14/374	451/23328	0.014708482	0.04941	0.02972	Bmp4/Gat	14
GO:0010659	GO:0010659	cardiac muscle cell apoptotic process	4/374	59/23328	0.014809295	0.04963	0.02985	Igfbp3/Sfr	4
GO:0042551	GO:0042551	neuron maturation	4/374	59/23328	0.014809295	0.04963	0.02985	Ednrb/Pd	4
GO:0071156	GO:0071156	regulation of cell cycle arrest	4/374	59/23328	0.014809295	0.04963	0.02985	Rgcc/Sox	4
GO:0010950	GO:0010950	positive regulation of endopeptidase activity	7/374	160/23328	0.014904245	0.04991	0.03002	Perp/Sfrp	7
GO:0046888	GO:0046888	negative regulation of hormone secretion	5/374	90/23328	0.014938609	0.04994	0.03004	Sfrp1/Igfb	5
GO:1900076	GO:1900076	regulation of cellular response to insulin stimulus	5/374	90/23328	0.014938609	0.04994	0.03004	C1qtnf9/L	5
GO:0003157	GO:0003157	endocardium development	2/374	12/23328	0.015214022	0.04994	0.03004	Kdr/Prox1	2
GO:0003207	GO:0003207	cardiac chamber formation	2/374	12/23328	0.015214022	0.04994	0.03004	Sox4/Han	2
GO:0010749	GO:0010749	regulation of nitric oxide mediated signal transduction	2/374	12/23328	0.015214022	0.04994	0.03004	Thbs1/Pd	2
GO:0010755	GO:0010755	regulation of plasminogen activation	2/374	12/23328	0.015214022	0.04994	0.03004	Thbs1/Se	2
GO:0014745	GO:0014745	negative regulation of muscle adaptation	2/374	12/23328	0.015214022	0.04994	0.03004	Nos3/Igfb	2
GO:0014824	GO:0014824	artery smooth muscle contraction	2/374	12/23328	0.015214022	0.04994	0.03004	Cd38/Edn	2
GO:0031392	GO:0031392	regulation of prostaglandin biosynthetic process	2/374	12/23328	0.015214022	0.04994	0.03004	Fabp5/Cd	2
GO:0032490	GO:0032490	detection of molecule of bacterial origin	2/374	12/23328	0.015214022	0.04994	0.03004	Scarb1/Lb	2
GO:0034333	GO:0034333	adherens junction assembly	2/374	12/23328	0.015214022	0.04994	0.03004	Actb/Ram	2
GO:0034392	GO:0034392	negative regulation of smooth muscle cell apoptotic pro	2/374	12/23328	0.015214022	0.04994	0.03004	Gapdh/Ed	2
GO:0035376	GO:0035376	sterol import	2/374	12/23328	0.015214022	0.04994	0.03004	Scarb1/Co	2
GO:0035437	GO:0035437	maintenance of protein localization in endoplasmic reti	2/374	12/23328	0.015214022	0.04994	0.03004	Hspa5/Gja	2
GO:0035733	GO:0035733	hepatic stellate cell activation	2/374	12/23328	0.015214022	0.04994	0.03004	Cygb/Pdg	2
GO:0038063	GO:0038063	collagen-activated tyrosine kinase receptor signaling p	2/374	12/23328	0.015214022	0.04994	0.03004	Col4a2/Co	2
GO:0048241	GO:0048241	epinephrine transport	2/374	12/23328	0.015214022	0.04994	0.03004	Ly6e/Ptgs	2
GO:0048934	GO:0048934	peripheral nervous system neuron differentiation	2/374	12/23328	0.015214022	0.04994	0.03004	Hand2/Ho	2
GO:0048935	GO:0048935	peripheral nervous system neuron development	2/374	12/23328	0.015214022	0.04994	0.03004	Hand2/Ho	2
GO:0060213	GO:0060213	positive regulation of nuclear-transcribed mRNA poly(A	2/374	12/23328	0.015214022	0.04994	0.03004	Btg2/Zfp3	2
GO:0070508	GO:0070508	cholesterol import	2/374	12/23328	0.015214022	0.04994	0.03004	Scarb1/Co	2
GO:0072124	GO:0072124	regulation of glomerular mesangial cell proliferation	2/374	12/23328	0.015214022	0.04994	0.03004	Bmp4/Pd	2
GO:0072176	GO:0072176	nephric duct development	2/374	12/23328	0.015214022	0.04994	0.03004	Bmp4/Gat	2
GO:1902947	GO:1902947	regulation of tau-protein kinase activity	2/374	12/23328	0.015214022	0.04994	0.03004	Clu/Apoe	2
GO:2000343	GO:2000343	positive regulation of chemokine (C-X-C motif) ligand 2	2/374	12/23328	0.015214022	0.04994	0.03004	Lpl/Cd74	2
GO:0050714	GO:0050714	positive regulation of protein secretion	8/374	199/23328	0.015298449	0.05018	0.03018	Kcnn4/Cd	8
GO:0006694	GO:0006694	steroid biosynthetic process	7/374	161/23328	0.015374813	0.05035	0.03028	Scarb1/Dk	7
GO:0030178	GO:0030178	negative regulation of Wnt signaling pathway	7/374	161/23328	0.015374813	0.05035	0.03028	Sfrp1/Dkk	7
GO:1902414	GO:1902414	protein localization to cell junction	6/374	125/23328	0.015558165	0.05053	0.03039	Cdh5/Actb	6
GO:0014015	GO:0014015	positive regulation of gliogenesis	5/374	91/23328	0.0156057	0.05053	0.03039	Vim/Hes1/	5
GO:0031110	GO:0031110	regulation of microtubule polymerization or depolymeri:	5/374	91/23328	0.0156057	0.05053	0.03039	Cdh5/Gda	5
GO:0032611	GO:0032611	interleukin-1 beta production	5/374	91/23328	0.0156057	0.05053	0.03039	Cd36/Lpl/	5
GO:0044344	GO:0044344	cellular response to fibroblast growth factor stimulus	5/374	91/23328	0.0156057	0.05053	0.03039	Sfrp1/Gat	5
GO:0002486	GO:0002486	antigen processing and presentation of endogenous pe	3/374	33/23328	0.015626749	0.05053	0.03039	H2-Q6/H2	3
GO:0010667	GO:0010667	negative regulation of cardiac muscle cell apoptotic pro	3/374	33/23328	0.015626749	0.05053	0.03039	Sfrp2/Nup	3
GO:0032369	GO:0032369	negative regulation of lipid transport	3/374	33/23328	0.015626749	0.05053	0.03039	Igfbp3/Ap	3
GO:0050654	GO:0050654	chondroitin sulfate proteoglycan metabolic process	3/374	33/23328	0.015626749	0.05053	0.03039	Cytl1/Bgn/	3
GO:0060706	GO:0060706	cell differentiation involved in embryonic placenta deve	3/374	33/23328	0.015626749	0.05053	0.03039	Spint2/Nr	3
GO:0060740	GO:0060740	prostate gland epithelium morphogenesis	3/374	33/23328	0.015626749	0.05053	0.03039	Sfrp1/Bm	3
GO:1905144	GO:1905144	response to acetylcholine	3/374	33/23328	0.015626749	0.05053	0.03039	Ly6c1/Ly6	3
GO:1905145	GO:1905145	cellular response to acetylcholine	3/374	33/23328	0.015626749	0.05053	0.03039	Ly6c1/Ly6	3
GO:0010524	GO:0010524	positive regulation of calcium ion transport into cytosol	4/374	60/23328	0.015671755	0.05053	0.03039	Bmp4/Apl	4
GO:0021575	GO:0021575	hindbrain morphogenesis	4/374	60/23328	0.015671755	0.05053	0.03039	Prox1/Hes	4
GO:0070098	GO:0070098	chemokine-mediated signaling pathway	4/374	60/23328	0.015671755	0.05053	0.03039	Cxcl1/Cxc	4
GO:0090183	GO:0090183	regulation of kidney development	4/374	60/23328	0.015671755	0.05053	0.03039	Bmp4/Gat	4
GO:1901616	GO:1901616	organic hydroxy compound catabolic process	4/374	60/23328	0.015671755	0.05053	0.03039	Adh7/Sca	4
GO:2000401	GO:2000401	regulation of lymphocyte migration	4/374	60/23328	0.015671755	0.05053	0.03039	Cxcl12/Cc	4
GO:2001238	GO:2001238	positive regulation of extrinsic apoptotic signaling path	4/374	60/23328	0.015671755	0.05053	0.03039	Sfrp1/Thb	4
GO:0099173	GO:0099173	postsynapse organization	8/374	200/23328	0.015721504	0.05065	0.03046	Apoe/Nrp	8
GO:0030217	GO:0030217	T cell differentiation	10/374	282/23328	0.016122114	0.05182	0.03117	Bmp4/Gat	10
GO:0031341	GO:0031341	regulation of cell killing	6/374	126/23328	0.016122987	0.05182	0.03117	Cfh/Cxcl1	6
GO:1901216	GO:1901216	positive regulation of neuron death	6/374	126/23328	0.016122987	0.05182	0.03117	Clu/Apoe/	6
GO:0045685	GO:0045685	regulation of glial cell differentiation	5/374	92/23328	0.016292381	0.05233	0.03147	Bmp4/Hes	5
GO:0010812	GO:0010812	negative regulation of cell-substrate adhesion	4/374	61/23328	0.016565171	0.05316	0.03197	Thbs1/Col	4
GO:0019221	GO:0019221	cytokine-mediated signaling pathway	12/374	369/23328	0.016596415	0.05322	0.03201	Krt18/Cxc	12
GO:0030879	GO:0030879	mammary gland development	7/374	164/23328	0.01684981	0.05391	0.03242	Bmp4/Gat	7
GO:0002476	GO:0002476	antigen processing and presentation of endogenous pe	3/374	34/23328	0.016939489	0.05391	0.03242	H2-Q6/H2	3
GO:0002484	GO:0002484	antigen processing and presentation of endogenous pe	3/374	34/23328	0.016939489	0.05391	0.03242	H2-Q6/H2	3
GO:0003209	GO:0003209	cardiac atrium morphogenesis	3/374	34/23328	0.016939489	0.05391	0.03242	Gja5/Prox	3
GO:0014741	GO:0014741	negative regulation of muscle hypertrophy	3/374	34/23328	0.016939489	0.05391	0.03242	Rgs4/Pi16	3
GO:0048710	GO:0048710	regulation of astrocyte differentiation	3/374	34/23328	0.016939489	0.05391	0.03242	Hes1/Id2/	3
GO:0060512	GO:0060512	prostate gland morphogenesis	3/374	34/23328	0.016939489	0.05391	0.03242	Sfrp1/Bm	3
GO:0090022	GO:0090022	regulation of neutrophil chemotaxis	3/374	34/23328	0.016939489	0.05391	0.03242	Lbp/Cd74/	3
GO:0090162	GO:0090162	establishment of epithelial cell polarity	3/374	34/23328	0.016939489	0.05391	0.03242	Tcf15/Hes	3
GO:0090218	GO:0090218	positive regulation of lipid kinase activity	3/374	34/23328	0.016939489	0.05391	0.03242	Flt1/Tek/P	3
GO:0034764	GO:0034764	positive regulation of transmembrane transport	9/374	243/23328	0.016990727	0.05402	0.03249	Bmp4/Apl	9
GO:0045445	GO:0045445	myoblast differentiation	5/374	93/23328	0.016998875	0.05402	0.03249	Bmp4/Igfb	5
GO:0006575	GO:0006575	cellular modified amino acid metabolic process	7/374	165/23328	0.017362955	0.05513	0.03316	Gata3/Ass	7
GO:0010658	GO:0010658	striated muscle cell apoptotic process	4/374	62/23328	0.017489856	0.05524	0.03322	Igfbp3/Sfr	4
GO:0030330	GO:0030330	DNA damage response, signal transduction by p53 cla	4/374	62/23328	0.017489856	0.05524	0.03322	Sox4/Cdk	4
GO:0031018	GO:0031018	endocrine pancreas development	4/374	62/23328	0.017489856	0.05524	0.03322	Clu/Bmp4	4
GO:0031640	GO:0031640	killing of cells of other organism	4/374	62/23328	0.017489856	0.05524	0.03322	Cxcl1/Cxc	4
GO:0051058	GO:0051058	negative regulation of small GTPase mediated signal t	4/374	62/23328	0.017489856	0.05524	0.03322	Cgnl1/Itgb	4
GO:0051148	GO:0051148	negative regulation of muscle cell differentiation	4/374	62/23328	0.017489856	0.05524	0.03322	Rgs4/Pi16	4
GO:1905207	GO:1905207	regulation of cardiocyte differentiation	4/374	62/23328	0.017489856	0.05524	0.03322	Bmp4/Rgs	4
GO:0002328	GO:0002328	pro-B cell differentiation	2/374	13/23328	0.017791576	0.05531	0.03327	Sox4/Hes	2
GO:0003091	GO:0003091	renal water homeostasis	2/374	13/23328	0.017791576	0.05531	0.03327	Aqp7/Aqp	2
GO:0003174	GO:0003174	mitral valve development	2/374	13/23328	0.017791576	0.05531	0.03327	Gja5/Sox	2
GO:0009404	GO:0009404	toxin metabolic process	2/374	13/23328	0.017791576	0.05531	0.03327	Pam/Cyp1	2
GO:0021683	GO:0021683	cerebellar granular layer morphogenesis	2/374	13/23328	0.017791576	0.05531	0.03327	Prox1/Ser	2
GO:0034374	GO:0034374	low-density lipoprotein particle remodeling	2/374	13/23328	0.017791576	0.05531	0.03327	Apoe/Pla2	2
GO:0035627	GO:0035627	ceramide transport	2/374	13/23328	0.017791576	0.05531	0.03327	Abcb1b/P	2
GO:0035810	GO:0035810	positive regulation of urine volume	2/374	13/23328	0.017791576	0.05531	0.03327	Ednrb/Edr	2
GO:0042482	GO:0042482	positive regulation of odontogenesis	2/374	13/23328	0.017791576	0.05531	0.03327	Edn1/Cd3	2
GO:0043691	GO:0043691	reverse cholesterol transport	2/374	13/23328	0.017791576	0.05531	0.03327	Scarb1/Ap	2
GO:0045906	GO:0045906	negative regulation of vasoconstriction	2/374	13/23328	0.017791576	0.05531	0.03327	Mmp2/Adr	2
GO:0061052	GO:0061052	negative regulation of cell growth involved in cardiac m	2/374	13/23328	0.017791576	0.05531	0.03327	Rgs4/Pi16	2
GO:0070831	GO:0070831	basement membrane assembly	2/374	13/23328	0.017791576	0.05531	0.03327	Lamb1/Ra	2
GO:0071285	GO:0071285	cellular response to lithium ion	2/374	13/23328	0.017791576	0.05531	0.03327	Fabp4/Id2	2
GO:0071294	GO:0071294	cellular response to zinc ion	2/374	13/23328	0.017791576	0.05531	0.03327	Mt1/Mt2	2
GO:0072110	GO:0072110	glomerular mesangial cell proliferation	2/374	13/23328	0.017791576	0.05531	0.03327	Bmp4/Pd	2
GO:0072216	GO:0072216	positive regulation of metanephros development	2/374	13/23328	0.017791576	0.05531	0.03327	Pdgfa/Pd	2
GO:0097011	GO:0097011	cellular response to granulocyte macrophage colony-st	2/374	13/23328	0.017791576	0.05531	0.03327	Zfp36/Pde	2
GO:1901163	GO:1901163	regulation of trophoblast cell migration	2/374	13/23328	0.017791576	0.05531	0.03327	Arhgdib/G	2

GO:1902170	GO:1902170	cellular response to reactive nitrogen species	2/374	13/23328	0.017791576	0.05531	0.03327	Thbs1/Aq	2
GO:1904181	GO:1904181	positive regulation of membrane depolarization	2/374	13/23328	0.017791576	0.05531	0.03327	Kdr/Dcn	2
GO:0098739	GO:0098739	import across plasma membrane	7/374	166/23328	0.017887026	0.05557	0.03342	Abcc9/Atp	7
GO:0006644	GO:0006644	phospholipid metabolic process	12/374	374/23328	0.018233522	0.05642	0.03394	Scarb1/Flt	12
GO:0002428	GO:0002428	antigen processing and presentation of peptide antigen	3/374	35/23328	0.018312062	0.05642	0.03394	H2-Q6/H2	3
GO:0010719	GO:0010719	negative regulation of epithelial to mesenchymal transi	3/374	35/23328	0.018312062	0.05642	0.03394	Sfrp1/Gat	3
GO:0045686	GO:0045686	negative regulation of glial cell differentiation	3/374	35/23328	0.018312062	0.05642	0.03394	Bmp4/Hes	3
GO:0045746	GO:0045746	negative regulation of Notch signaling pathway	3/374	35/23328	0.018312062	0.05642	0.03394	Nrarp/Egfl	3
GO:0045987	GO:0045987	positive regulation of smooth muscle contraction	3/374	35/23328	0.018312062	0.05642	0.03394	Ptgs1/F2r	3
GO:0050832	GO:0050832	defense response to fungus	3/374	35/23328	0.018312062	0.05642	0.03394	Cxcl1/Pla	3
GO:0050869	GO:0050869	negative regulation of B cell activation	3/374	35/23328	0.018312062	0.05642	0.03394	Sfrp1/Cd2	3
GO:0060142	GO:0060142	regulation of syncytium formation by plasma membran	3/374	35/23328	0.018312062	0.05642	0.03394	Cxcl12/Cx	3
GO:0060292	GO:0060292	long-term synaptic depression	3/374	35/23328	0.018312062	0.05642	0.03394	Mgll/Cd38	3
GO:0071320	GO:0071320	cellular response to cAMP	3/374	35/23328	0.018312062	0.05642	0.03394	Aqp1/Pde	3
GO:0051100	GO:0051100	negative regulation of binding	7/374	167/23328	0.018422135	0.05659	0.03403	Actb/Id1/C	7
GO:0060538	GO:0060538	skeletal muscle organ development	8/374	206/23328	0.018439402	0.05659	0.03403	Eln/Scx/N	8
GO:1902115	GO:1902115	regulation of organelle assembly	8/374	206/23328	0.018439402	0.05659	0.03403	Fez1/Fscn	8
GO:0006721	GO:0006721	terpenoid metabolic process	4/374	63/23328	0.018446106	0.05659	0.03403	Rbp1/Adh	4
GO:0032760	GO:0032760	positive regulation of tumor necrosis factor production	4/374	63/23328	0.018446106	0.05659	0.03403	Clu/Cd36/	4
GO:1903428	GO:1903428	positive regulation of reactive oxygen species biosynth	4/374	63/23328	0.018446106	0.05659	0.03403	Clu/Cd36/	4
GO:0031343	GO:0031343	positive regulation of cell killing	5/374	95/23328	0.018472157	0.05663	0.03406	Cxcl1/H2-	5
GO:0032368	GO:0032368	regulation of lipid transport	6/374	130/23328	0.018523917	0.05674	0.03413	Igfbp3/Ap	6
GO:0031016	GO:0031016	pancreas development	5/374	96/23328	0.019239361	0.05889	0.03542	Clu/Bmp4	5
GO:0030858	GO:0030858	positive regulation of epithelial cell differentiation	4/374	64/23328	0.0194342	0.05927	0.03565	Bmp4/Cdh	4
GO:0043388	GO:0043388	positive regulation of DNA binding	4/374	64/23328	0.0194342	0.05927	0.03565	Gata3/Hes	4
GO:0048008	GO:0048008	platelet-derived growth factor receptor signaling pathw	4/374	64/23328	0.0194342	0.05927	0.03565	Lox/Pdgfa	4
GO:1903510	GO:1903510	mucopolysaccharide metabolic process	4/374	64/23328	0.0194342	0.05927	0.03565	Bgn/Itih5/	4
GO:1903557	GO:1903557	positive regulation of tumor necrosis factor superfamily	4/374	64/23328	0.0194342	0.05927	0.03565	Clu/Cd36/	4
GO:0003401	GO:0003401	axis elongation	3/374	36/23328	0.019744635	0.05995	0.03606	Sfrp1/Bmp	3
GO:0010453	GO:0010453	regulation of cell fate commitment	3/374	36/23328	0.019744635	0.05995	0.03606	Bmp4/Sfrp	3
GO:0010664	GO:0010664	negative regulation of striated muscle cell apoptotic pr	3/374	36/23328	0.019744635	0.05995	0.03606	Sfrp2/Nup	3
GO:0048009	GO:0048009	insulin-like growth factor receptor signaling pathway	3/374	36/23328	0.019744635	0.05995	0.03606	Igfbp3/Igf	3
GO:1902624	GO:1902624	positive regulation of neutrophil migration	3/374	36/23328	0.019744635	0.05995	0.03606	Lbp/Cd74	3
GO:2000171	GO:2000171	negative regulation of dendrite development	3/374	36/23328	0.019744635	0.05995	0.03606	Apoe/Plk2	3
GO:0002456	GO:0002456	T cell mediated immunity	6/374	132/23328	0.019811465	0.06007	0.03613	Gata3/Cd	6
GO:0099175	GO:0099175	regulation of postsynapse organization	6/374	132/23328	0.019811465	0.06007	0.03613	Apoe/Nrp	6
GO:0002027	GO:0002027	regulation of heart rate	5/374	97/23328	0.020027205	0.06063	0.03647	Gja5/Rgs	5
GO:0045069	GO:0045069	regulation of viral genome replication	5/374	97/23328	0.020027205	0.06063	0.03647	Prox1/Rsa	5
GO:0034121	GO:0034121	regulation of toll-like receptor signaling pathway	4/374	65/23328	0.020454401	0.06073	0.03652	Cd36/Lbp	4
GO:0042246	GO:0042246	tissue regeneration	4/374	65/23328	0.020454401	0.06073	0.03652	Cdkn1a/N	4
GO:0046503	GO:0046503	glycerolipid catabolic process	4/374	65/23328	0.020454401	0.06073	0.03652	Scarb1/M	4
GO:0044070	GO:0044070	regulation of anion transport	6/374	133/23328	0.020477516	0.06073	0.03652	Apoe/Thb	6
GO:0070663	GO:0070663	regulation of leukocyte proliferation	9/374	251/23328	0.02048529	0.06073	0.03652	Vcam1/Br	9
GO:0098657	GO:0098657	import into cell	9/374	251/23328	0.02048529	0.06073	0.03652	Cd36/Abc	9
GO:0010226	GO:0010226	response to lithium ion	2/374	14/23328	0.020539374	0.06073	0.03652	Fabp4/Id2	2
GO:0010644	GO:0010644	cell communication by electrical coupling	2/374	14/23328	0.020539374	0.06073	0.03652	Gja5/Gja1	2
GO:0034638	GO:0034638	phosphatidylcholine catabolic process	2/374	14/23328	0.020539374	0.06073	0.03652	Scarb1/Pl	2
GO:0035358	GO:0035358	regulation of peroxisome proliferator activated recepto	2/374	14/23328	0.020539374	0.06073	0.03652	Ptgis/Fab	2
GO:0035815	GO:0035815	positive regulation of renal sodium excretion	2/374	14/23328	0.020539374	0.06073	0.03652	Ednrb/Edr	2
GO:0038065	GO:0038065	collagen-activated signaling pathway	2/374	14/23328	0.020539374	0.06073	0.03652	Col4a2/Co	2
GO:0042983	GO:0042983	amyloid precursor protein biosynthetic process	2/374	14/23328	0.020539374	0.06073	0.03652	Bace2/Itm	2
GO:0042984	GO:0042984	regulation of amyloid precursor protein biosynthetic pro	2/374	14/23328	0.020539374	0.06073	0.03652	Bace2/Itm	2
GO:0043508	GO:0043508	negative regulation of JUN kinase activity	2/374	14/23328	0.020539374	0.06073	0.03652	Sfrp1/Sfrp	2
GO:0048505	GO:0048505	regulation of timing of cell differentiation	2/374	14/23328	0.020539374	0.06073	0.03652	Hes1/Serp	2
GO:0060211	GO:0060211	regulation of nuclear-transcribed mRNA poly(A) tail sh	2/374	14/23328	0.020539374	0.06073	0.03652	Btg2/Zfp3	2
GO:0060263	GO:0060263	regulation of respiratory burst	2/374	14/23328	0.020539374	0.06073	0.03652	Lbp/Jchai	2
GO:0061469	GO:0061469	regulation of type B pancreatic cell proliferation	2/374	14/23328	0.020539374	0.06073	0.03652	Sfrp1/Nup	2
GO:0071850	GO:0071850	mitotic cell cycle arrest	2/374	14/23328	0.020539374	0.06073	0.03652	Rgcc/Cdk	2
GO:0072224	GO:0072224	metanephric glomerulus development	2/374	14/23328	0.020539374	0.06073	0.03652	Pdgfb/Aq	2
GO:0072413	GO:0072413	signal transduction involved in mitotic cell cycle check	2/374	14/23328	0.020539374	0.06073	0.03652	Sox4/Cdk	2
GO:0090179	GO:0090179	planar cell polarity pathway involved in neural tube clo	2/374	14/23328	0.020539374	0.06073	0.03652	Sfrp1/Sfrp	2
GO:0097012	GO:0097012	response to granulocyte macrophage colony-stimulatin	2/374	14/23328	0.020539374	0.06073	0.03652	Zfp36/Pde	2
GO:0099550	GO:0099550	trans-synaptic signaling, modulating synaptic transmis	2/374	14/23328	0.020539374	0.06073	0.03652	F2r/Plat	2
GO:1902402	GO:1902402	signal transduction involved in mitotic DNA damage ch	2/374	14/23328	0.020539374	0.06073	0.03652	Sox4/Cdk	2
GO:1902403	GO:1902403	signal transduction involved in mitotic DNA integrity ch	2/374	14/23328	0.020539374	0.06073	0.03652	Sox4/Cdk	2
GO:1902902	GO:1902902	negative regulation of autophagosome assembly	2/374	14/23328	0.020539374	0.06073	0.03652	Fez1/Nup	2
GO:1902931	GO:1902931	negative regulation of alcohol biosynthetic process	2/374	14/23328	0.020539374	0.06073	0.03652	Dkk3/Apo	2
GO:1902950	GO:1902950	regulation of dendritic spine maintenance	2/374	14/23328	0.020539374	0.06073	0.03652	Apoe/Prn	2
GO:1903236	GO:1903236	regulation of leukocyte tethering or rolling	2/374	14/23328	0.020539374	0.06073	0.03652	Selp/Cxcl	2
GO:1904948	GO:1904948	midbrain dopaminergic neuron differentiation	2/374	14/23328	0.020539374	0.06073	0.03652	Sfrp1/Sfrp	2
GO:2001279	GO:2001279	regulation of unsaturated fatty acid biosynthetic proces	2/374	14/23328	0.020539374	0.06073	0.03652	Fabp5/Cd	2
GO:0010952	GO:0010952	positive regulation of peptidase activity	7/374	171/23328	0.020675113	0.06109	0.03674	Perp/Sfrp	7
GO:0045833	GO:0045833	negative regulation of lipid metabolic process	5/374	98/23328	0.020835883	0.06148	0.03697	Dkk3/Apo	5
GO:0060395	GO:0060395	SMAD protein signal transduction	5/374	98/23328	0.020835883	0.06148	0.03697	Bmp4/Fos	5
GO:0055076	GO:0055076	transition metal ion homeostasis	6/374	134/23328	0.021158605	0.06235	0.0375	Steap4/M	6
GO:0002920	GO:0002920	regulation of humoral immune response	3/374	37/23328	0.021237315	0.06235	0.0375	Cfh/C3/Se	3
GO:0007435	GO:0007435	salivary gland morphogenesis	3/374	37/23328	0.021237315	0.06235	0.0375	Pdgfa/Tgr	3
GO:0033363	GO:0033363	secretory granule organization	3/374	37/23328	0.021237315	0.06235	0.0375	Srgn/Aqp	3
GO:0060071	GO:0060071	Wnt signaling pathway, planar cell polarity pathway	3/374	37/23328	0.021237315	0.06235	0.0375	Sfrp1/Cth	3
GO:0071514	GO:0071514	genetic imprinting	3/374	37/23328	0.021237315	0.06235	0.0375	Meg3/Cdk	3
GO:1902229	GO:1902229	regulation of intrinsic apoptotic signaling pathway in re	3/374	37/23328	0.021237315	0.06235	0.0375	Clu/Cxcl1	3
GO:0072659	GO:0072659	protein localization to plasma membrane	10/374	295/23328	0.021303457	0.0625	0.03759	Krt18/Tme	10
GO:0046034	GO:0046034	ATP metabolic process	9/374	253/23328	0.021434404	0.06284	0.03779	Cfh/Nupr1	9
GO:0043112	GO:0043112	receptor metabolic process	8/374	212/23328	0.021478396	0.06288	0.03782	Cd36/Apo	8
GO:0022411	GO:0022411	cellular component disassembly	12/374	383/23328	0.021481657	0.06288	0.03782	Igfbp3/Mi	12
GO:0001707	GO:0001707	mesoderm formation	4/374	66/23328	0.021506955	0.06288	0.03782	Bmp4/Scx	4
GO:0032623	GO:0032623	interleukin-2 production	4/374	66/23328	0.021506955	0.06288	0.03782	Gata3/Prn	4
GO:0007200	GO:0007200	phospholipase C-activating G protein-coupled recepto	5/374	99/23328	0.021665581	0.0633	0.03807	Ednrb/Tgr	5
GO:0030177	GO:0030177	positive regulation of Wnt signaling pathway	6/374	135/23328	0.021854867	0.0638	0.03837	Sfrp1/Sfrp	6
GO:0008361	GO:0008361	regulation of cell size	8/374	213/23328	0.022017366	0.06423	0.03863	Mgll/Kcnn	8
GO:0006940	GO:0006940	regulation of smooth muscle contraction	4/374	67/23328	0.022592091	0.06573	0.03953	Ptgs1/F2r	4
GO:0030239	GO:0030239	myofibril assembly	4/374	67/23328	0.022592091	0.06573	0.03953	Prox1/Itgb	4
GO:0060688	GO:0060688	regulation of morphogenesis of a branching structure	4/374	67/23328	0.022592091	0.06573	0.03953	Cpe/Sfrp1	4
GO:0070613	GO:0070613	regulation of protein processing	4/374	67/23328	0.022592091	0.06573	0.03953	Spon1/Th	4
GO:0010831	GO:0010831	positive regulation of myotube differentiation	3/374	38/23328	0.022790156	0.06593	0.03966	Cxcl12/Cx	3
GO:0014002	GO:0014002	astrocyte development	3/374	38/23328	0.022790156	0.06593	0.03966	Vim/S100	3
GO:0035335	GO:0035335	peptidyl-tyrosine dephosphorylation	3/374	38/23328	0.022790156	0.06593	0.03966	Ptpn18/Pt	3
GO:0046627	GO:0046627	negative regulation of insulin receptor signaling pathw	3/374	38/23328	0.022790156	0.06593	0.03966	Ahsg/Soc	3
GO:0060219	GO:0060219	camera-type eye photoreceptor cell differentiation	3/374	38/23328	0.022790156	0.06593	0.03966	Cfh/Thy1/	3
GO:0070528	GO:0070528	protein kinase C signaling	3/374	38/23328	0.022790156	0.06593	0.03966	Flt4/Pdgf	3
GO:0090322	GO:0090322	regulation of superoxide metabolic process	3/374	38/23328	0.022790156	0.06593	0.03966	Fbln5/Cd	3
GO:1904037	GO:1904037	positive regulation of epithelial cell apoptotic process	3/374	38/23328	0.022790156	0.06593	0.03966	Bmp4/Thb	3
GO:0007050	GO:0007050	cell cycle arrest	6/374	137/23328	0.023293444	0.0666	0.04006	Thbs1/Rg	6
GO:0034341	GO:0034341	response to interferon-gamma	6/374	137/23328	0.023293444	0.0666	0.04006	Vim/H2-E	6
GO:0032755	GO:0032755	positive regulation of interleukin-6 production	5/374	101/23328	0.023388749	0.0666	0.04006	Cd36/Lbp	5
GO:0071456	GO:0071456	cellular response to hypoxia	5/374	101/23328	0.023388749	0.0666	0.04006	Ptgis/Rgc	5
GO:0003188	GO:0003188	heart valve formation	2/374	15/23328	0.023451324	0.0666	0.04006	Scx/Gja5	2
GO:0010715	GO:0010715	regulation of extracellular matrix disassembly	2/374	15/23328	0.023451324	0.0666	0.04006	Fscn1/Pd	2
GO:0010935	GO:0010935	regulation of macrophage cytokine production	2/374	15/23328	0.023451324	0.0666	0.04006	Cd36/Cd7	2
GO:0010958	GO:0010958	regulation of amino acid import across plasma membra	2/374	15/23328	0.023451324	0.0666	0.04006	Rgs4/Itgb	2
GO:0016114	GO:0016114	terpenoid biosynthetic process	2/374	15/23328	0.023451324	0.0666	0.04006	Rbp1/Cyp	2
GO:0023035	GO:0023035	CD40 signaling pathway	2/374	15/23328	0.023451324	0.0666	0.04006	Itgb1/Itga	2
GO:0033690	GO:0033690	positive regulation of osteoblast proliferation	2/374	15/23328	0.023451324	0.0666	0.04006	Fbln5/Cth	2
GO:0035745	GO:0035745	T-helper 2 cell cytokine production	2/374	15/23328	0.023451324	0.0666	0.04006	Gata3/Rsa	2
GO:0042178	GO:0042178	xenobiotic catabolic process	2/374	15/23328	0.023451324	0.0666	0.04006	Cyp1a1/G	2
GO:0042953	GO:0042953	lipoprotein transport	2/374	15/23328	0.023451324	0.0666	0.04006	Cd36/Lsr	2
GO:0043031	GO:0043031	negative regulation of macrophage activation	2/374	15/23328	0.023451324	0.0666	0.04006	Adgrf5/Cd	2
GO:0046541	GO:0046541	saliva secretion	2/374	15/23328	0.023451324	0.0666	0.04006	Kcnn4/Aq	2
GO:0050930	GO:0050930	induction of positive chemotaxis	2/374	15/23328	0.023451324	0.0666	0.04006	Cxcl12/Cc	2
GO:0051712	GO:0051712	positive regulation of killing of cells of other organism	2/374	15/23328	0.023451324	0.0666	0.04006	Cxcl1/Gap	2
GO:0051764	GO:0051764	actin crosslink formation	2/374	15/23328	0.023451324	0.0666	0.04006	Dpysl3/Ac	2
GO:0060442	GO:0060442	branching involved in prostate gland morphogenesis	2/374	15/23328	0.023451324	0.0666	0.04006	Sfrp1/Bmp	2
GO:0070307	GO:0070307	lens fiber cell development	2/374	15/23328	0.023451324	0.0666	0.04006	Prox1/Vim	2
GO:0090178	GO:0090178	regulation of establishment of planar polarity involved i	2/374	15/23328	0.023451324	0.0666	0.04006	Sfrp1/Sfrp	2
GO:0090594	GO:0090594	inflammatory response to wounding	2/374	15/23328	0.023451324	0.0666	0.04006	Cd24a/F2	2
GO:1903587	GO:1903587	regulation of blood vessel endothelial cell proliferation	2/374	15/23328	0.023451324	0.0666	0.04006	Aplnr/Thb	2
GO:1903789	GO:1903789	regulation of amino acid transmembrane transport	2/374	15/23328	0.023451324	0.0666	0.04006	Rgs4/Itgb	2
GO:2000095	GO:2000095	regulation of Wnt signaling pathway, planar cell polarit	2/374	15/23328	0.023451324	0.0666	0.04006	Sfrp1/Sfrp	2
GO:2000811	GO:2000811	negative regulation of anoikis	2/374	15/23328	0.023451324	0.0666	0.04006	Itgb1/Itga	2

GO:0030837	GO:0030837	negative regulation of actin filament polymerization	4/374	68/23328	0.023710024	0.06715	0.04039	Pecam1/T	4
GO:0043949	GO:0043949	regulation of cAMP-mediated signaling	4/374	68/23328	0.023710024	0.06715	0.04039	Aplnr/Pde	4
GO:0097194	GO:0097194	execution phase of apoptosis	4/374	68/23328	0.023710024	0.06715	0.04039	Ptgis/Igfbp	4
GO:1901264	GO:1901264	carbohydrate derivative transport	4/374	68/23328	0.023710024	0.06715	0.04039	Scarb1/Lb	4
GO:0048675	GO:0048675	axon extension	6/374	138/23328	0.024036016	0.06798	0.04089	Mgll/Apoe	6
GO:0050671	GO:0050671	positive regulation of lymphocyte proliferation	6/374	138/23328	0.024036016	0.06798	0.04089	Vcam1/Cc	6
GO:0017038	GO:0017038	protein import	8/374	217/23328	0.024268926	0.06855	0.04123	Clu/Gpihb	8
GO:0048639	GO:0048639	positive regulation of developmental growth	8/374	217/23328	0.024268926	0.06855	0.04123	Ncam1/Ap	8
GO:0002475	GO:0002475	antigen processing and presentation via MHC class Ib	3/374	39/23328	0.024403156	0.0686	0.04126	H2-Q6/H2	3
GO:0003230	GO:0003230	cardiac atrium development	3/374	39/23328	0.024403156	0.0686	0.04126	Gja5/Prox	3
GO:0010613	GO:0010613	positive regulation of cardiac muscle hypertrophy	3/374	39/23328	0.024403156	0.0686	0.04126	Ece1/Edn	3
GO:0035850	GO:0035850	epithelial cell differentiation involved in kidney develop	3/374	39/23328	0.024403156	0.0686	0.04126	Bmp4/Gat	3
GO:0050892	GO:0050892	intestinal absorption	3/374	39/23328	0.024403156	0.0686	0.04126	Scarb1/Cc	3
GO:0060251	GO:0060251	regulation of glial cell proliferation	3/374	39/23328	0.024403156	0.0686	0.04126	Vim/Hes1/	3
GO:0072523	GO:0072523	purine-containing compound catabolic process	3/374	39/23328	0.024403156	0.0686	0.04126	Gda/Nudt	3
GO:0032412	GO:0032412	regulation of ion transmembrane transporter activity	9/374	259/23328	0.024471135	0.06875	0.04135	Bmp4/Fhl	9
GO:0009880	GO:0009880	embryonic pattern specification	4/374	69/23328	0.02486095	0.06965	0.04189	Meox1/Nr	4
GO:0021536	GO:0021536	diencephalon development	4/374	69/23328	0.02486095	0.06965	0.04189	Ncam1/Br	4
GO:0031638	GO:0031638	zymogen activation	4/374	69/23328	0.02486095	0.06965	0.04189	Perp/Thbs	4
GO:0032677	GO:0032677	regulation of interleukin-8 production	4/374	69/23328	0.02486095	0.06965	0.04189	Cd2/Lbp/F	4
GO:0051091	GO:0051091	positive regulation of DNA-binding transcription factor	9/374	260/23328	0.025005531	0.07001	0.04211	Cytl1/Clu/	9
GO:0010522	GO:0010522	regulation of calcium ion transport into cytosol	5/374	103/23328	0.025198074	0.0705	0.0424	Bmp4/Apl	5
GO:0032946	GO:0032946	positive regulation of mononuclear cell proliferation	6/374	140/23328	0.025568349	0.07149	0.043	Vcam1/Cc	6
GO:0001541	GO:0001541	ovarian follicle development	4/374	70/23328	0.02604505	0.07219	0.04341	Bmp4/Kdr	4
GO:0030888	GO:0030888	regulation of B cell proliferation	4/374	70/23328	0.02604505	0.07219	0.04341	Cd38/Cd2	4
GO:0043954	GO:0043954	cellular component maintenance	4/374	70/23328	0.02604505	0.07219	0.04341	Apoe/F2r/	4
GO:0048332	GO:0048332	mesoderm morphogenesis	4/374	70/23328	0.02604505	0.07219	0.04341	Bmp4/Scx	4
GO:0055008	GO:0055008	cardiac muscle tissue morphogenesis	4/374	70/23328	0.02604505	0.07219	0.04341	Prox1/Ly6	4
GO:0150076	GO:0150076	neuroinflammatory response	4/374	70/23328	0.02604505	0.07219	0.04341	Clu/Nupr1	4
GO:2000107	GO:2000107	negative regulation of leukocyte apoptotic process	4/374	70/23328	0.02604505	0.07219	0.04341	Bmp4/Cxc	4
GO:0007616	GO:0007616	long-term memory	3/374	40/23328	0.026076261	0.07219	0.04341	Apoe/Relr	3
GO:0014742	GO:0014742	positive regulation of muscle hypertrophy	3/374	40/23328	0.026076261	0.07219	0.04341	Ece1/Edn	3
GO:0043551	GO:0043551	regulation of phosphatidylinositol 3-kinase activity	3/374	40/23328	0.026076261	0.07219	0.04341	Flt1/Tek/P	3
GO:0060325	GO:0060325	face morphogenesis	3/374	40/23328	0.026076261	0.07219	0.04341	Scx/Mmp2	3
GO:0070570	GO:0070570	regulation of neuron projection regeneration	3/374	40/23328	0.026076261	0.07219	0.04341	Klk8/Thy1	3
GO:0071542	GO:0071542	dopaminergic neuron differentiation	3/374	40/23328	0.026076261	0.07219	0.04341	Sfrp1/Sfrp	3
GO:0090175	GO:0090175	regulation of establishment of planar polarity	3/374	40/23328	0.026076261	0.07219	0.04341	Sfrp1/Cthr	3
GO:0097009	GO:0097009	energy homeostasis	3/374	40/23328	0.026076261	0.07219	0.04341	Cd36/C1q	3
GO:0045778	GO:0045778	positive regulation of ossification	5/374	104/23328	0.026135445	0.07222	0.04344	Bmp4/Cth	5
GO:0035966	GO:0035966	response to topologically incorrect protein	6/374	141/23328	0.026358351	0.07222	0.04344	Clu/Thbs1	6
GO:1902107	GO:1902107	positive regulation of leukocyte differentiation	7/374	180/23328	0.026430894	0.07222	0.04344	Gata3/Fos	7
GO:0003161	GO:0003161	cardiac conduction system development	2/374	16/23328	0.026521486	0.07222	0.04344	Gja5/Id2	2
GO:0010454	GO:0010454	negative regulation of cell fate commitment	2/374	16/23328	0.026521486	0.07222	0.04344	Sfrp2/Hes	2
GO:0014067	GO:0014067	negative regulation of phosphatidylinositol 3-kinase sig	2/374	16/23328	0.026521486	0.07222	0.04344	Slc9a3r2/	2
GO:0016322	GO:0016322	neuron remodeling	2/374	16/23328	0.026521486	0.07222	0.04344	Pdgfb/C3	2
GO:0030322	GO:0030322	stabilization of membrane potential	2/374	16/23328	0.026521486	0.07222	0.04344	Kcnn4/Ch	2
GO:0044872	GO:0044872	lipoprotein localization	2/374	16/23328	0.026521486	0.07222	0.04344	Cd36/Lsr	2
GO:0045655	GO:0045655	regulation of monocyte differentiation	2/374	16/23328	0.026521486	0.07222	0.04344	Cd74/Jun	2
GO:0051709	GO:0051709	regulation of killing of cells of other organism	2/374	16/23328	0.026521486	0.07222	0.04344	Cxcl1/Gap	2
GO:0060856	GO:0060856	establishment of blood-brain barrier	2/374	16/23328	0.026521486	0.07222	0.04344	Abcb1b/Ls	2
GO:0061000	GO:0061000	negative regulation of dendritic spine development	2/374	16/23328	0.026521486	0.07222	0.04344	Apoe/Plk2	2
GO:0070486	GO:0070486	leukocyte aggregation	2/374	16/23328	0.026521486	0.07222	0.04344	S100a8/S	2
GO:0071498	GO:0071498	cellular response to fluid shear stress	2/374	16/23328	0.026521486	0.07222	0.04344	Ass1/Klf2	2
GO:0071731	GO:0071731	response to nitric oxide	2/374	16/23328	0.026521486	0.07222	0.04344	Thbs1/Aq	2
GO:0072109	GO:0072109	glomerular mesangium development	2/374	16/23328	0.026521486	0.07222	0.04344	Bmp4/Pdg	2
GO:0072567	GO:0072567	chemokine (C-X-C motif) ligand 2 production	2/374	16/23328	0.026521486	0.07222	0.04344	Lpl/Cd74	2
GO:0072673	GO:0072673	lamellipodium morphogenesis	2/374	16/23328	0.026521486	0.07222	0.04344	Pdpn/Kan	2
GO:0090051	GO:0090051	negative regulation of cell migration involved in sprouti	2/374	16/23328	0.026521486	0.07222	0.04344	Thbs1/Mm	2
GO:0090083	GO:0090083	regulation of inclusion body assembly	2/374	16/23328	0.026521486	0.07222	0.04344	Clu/Apoe	2
GO:0090128	GO:0090128	regulation of synapse maturation	2/374	16/23328	0.026521486	0.07222	0.04344	Arhgef15/	2
GO:0090141	GO:0090141	positive regulation of mitochondrial fission	2/374	16/23328	0.026521486	0.07222	0.04344	Kdr/Dcn	2
GO:2000341	GO:2000341	regulation of chemokine (C-X-C motif) ligand 2 product	2/374	16/23328	0.026521486	0.07222	0.04344	Lpl/Cd74	2
GO:2000647	GO:2000647	negative regulation of stem cell proliferation	2/374	16/23328	0.026521486	0.07222	0.04344	Cd24a/Ptr	2
GO:1903039	GO:1903039	positive regulation of leukocyte cell-cell adhesion	8/374	221/23328	0.026677232	0.0726	0.04366	Vcam1/Se	8
GO:2001056	GO:2001056	positive regulation of cysteine-type endopeptidase acti	6/374	142/23328	0.027164398	0.07387	0.04443	Perp/F2r/	6
GO:0043113	GO:0043113	receptor clustering	4/374	71/23328	0.027262488	0.07409	0.04456	Apoe/Relr	4
GO:0002483	GO:0002483	antigen processing and presentation of endogenous pe	3/374	41/23328	0.027809367	0.07519	0.04522	H2-Q6/H2	3
GO:0002686	GO:0002686	negative regulation of leukocyte migration	3/374	41/23328	0.027809367	0.07519	0.04522	Cxcl12/Du	3
GO:0007431	GO:0007431	salivary gland development	3/374	41/23328	0.027809367	0.07519	0.04522	Pdgfa/Tgr	3
GO:0019885	GO:0019885	antigen processing and presentation of endogenous pe	3/374	41/23328	0.027809367	0.07519	0.04522	H2-Q6/H2	3
GO:0051281	GO:0051281	positive regulation of release of sequestered calcium i	3/374	41/23328	0.027809367	0.07519	0.04522	Aplnr/F2r/	3
GO:0072595	GO:0072595	maintenance of protein localization in organelle	3/374	41/23328	0.027809367	0.07519	0.04522	Chchd10/	3
GO:0086009	GO:0086009	membrane repolarization	3/374	41/23328	0.027809367	0.07519	0.04522	Gja5/Atp1	3
GO:1905332	GO:1905332	positive regulation of morphogenesis of an epithelium	3/374	41/23328	0.027809367	0.07519	0.04522	Bmp4/Gat	3
GO:0006022	GO:0006022	aminoglycan metabolic process	5/374	106/23328	0.028076345	0.07581	0.04559	Bgn/Itih5/	5
GO:0006165	GO:0006165	nucleoside diphosphate phosphorylation	5/374	106/23328	0.028076345	0.07581	0.04559	Nupr1/Cbf	5
GO:0016032	GO:0016032	viral process	10/374	309/23328	0.028138022	0.07593	0.04566	Vcam1/Ap	10
GO:0007193	GO:0007193	adenylate cyclase-inhibiting G protein-coupled recepto	4/374	72/23328	0.028513414	0.07689	0.04624	S1pr1/Pde	4
GO:0048588	GO:0048588	developmental cell growth	9/374	267/23328	0.028980345	0.0781	0.04697	Mgll/Apoe	9
GO:0002367	GO:0002367	cytokine production involved in immune response	5/374	107/23328	0.029080152	0.07832	0.0471	Gata3/Cd	5
GO:1901617	GO:1901617	organic hydroxy compound biosynthetic process	8/374	225/23328	0.029246961	0.07853	0.04723	Dkk3/Gata	8
GO:0006509	GO:0006509	membrane protein ectodomain proteolysis	3/374	42/23328	0.029602323	0.07853	0.04723	Apoe/Bac	3
GO:0010765	GO:0010765	positive regulation of sodium ion transport	3/374	42/23328	0.029602323	0.07853	0.04723	Cxcl1/Atp	3
GO:0030501	GO:0030501	positive regulation of bone mineralization	3/374	42/23328	0.029602323	0.07853	0.04723	Bmp4/Ptn	3
GO:0046839	GO:0046839	phospholipid dephosphorylation	3/374	42/23328	0.029602323	0.07853	0.04723	Ephx2/Plp	3
GO:0055023	GO:0055023	positive regulation of cardiac muscle tissue growth	3/374	42/23328	0.029602323	0.07853	0.04723	Ncam1/Pi	3
GO:2000279	GO:2000279	negative regulation of DNA biosynthetic process	3/374	42/23328	0.029602323	0.07853	0.04723	Dusp1/Cd	3
GO:0072073	GO:0072073	kidney epithelium development	6/374	145/23328	0.029679918	0.07853	0.04723	Sfrp1/Bmp	6
GO:1903364	GO:1903364	positive regulation of cellular protein catabolic process	6/374	145/23328	0.029679918	0.07853	0.04723	Clu/Apoe/	6
GO:0001710	GO:0001710	mesodermal cell fate commitment	2/374	17/23328	0.029744067	0.07853	0.04723	Bmp4/Sfrp	2
GO:0007096	GO:0007096	regulation of exit from mitosis	2/374	17/23328	0.029744067	0.07853	0.04723	Rgcc/Cdk	2
GO:0010713	GO:0010713	negative regulation of collagen metabolic process	2/374	17/23328	0.029744067	0.07853	0.04723	Fn1/Cygb	2
GO:0010752	GO:0010752	regulation of cGMP-mediated signaling	2/374	17/23328	0.029744067	0.07853	0.04723	Thbs1/Pd	2
GO:0010934	GO:0010934	macrophage cytokine production	2/374	17/23328	0.029744067	0.07853	0.04723	Cd36/Cd7	2
GO:0021681	GO:0021681	cerebellar granular layer development	2/374	17/23328	0.029744067	0.07853	0.04723	Prox1/Ser	2
GO:0032966	GO:0032966	negative regulation of collagen biosynthetic process	2/374	17/23328	0.029744067	0.07853	0.04723	Fn1/Cygb	2
GO:0060253	GO:0060253	negative regulation of glial cell proliferation	2/374	17/23328	0.029744067	0.07853	0.04723	Hes1/Ptn	2
GO:0060413	GO:0060413	atrial septum morphogenesis	2/374	17/23328	0.029744067	0.07853	0.04723	Gja5/Sox	2
GO:0060850	GO:0060850	regulation of transcription involved in cell fate commitn	2/374	17/23328	0.029744067	0.07853	0.04723	Prox1/Nr2	2
GO:0060973	GO:0060973	cell migration involved in heart development	2/374	17/23328	0.029744067	0.07853	0.04723	Bmp4/Har	2
GO:0070206	GO:0070206	protein trimerization	2/374	17/23328	0.029744067	0.07853	0.04723	Steap4/Cc	2
GO:0070293	GO:0070293	renal absorption	2/374	17/23328	0.029744067	0.07853	0.04723	Aqp7/Aqp	2
GO:0072010	GO:0072010	glomerular epithelium development	2/374	17/23328	0.029744067	0.07853	0.04723	Bmp4/Pdg	2
GO:0072234	GO:0072234	metanephric nephron tubule development	2/374	17/23328	0.029744067	0.07853	0.04723	Hes1/Aqp	2
GO:0090185	GO:0090185	negative regulation of kidney development	2/374	17/23328	0.029744067	0.07853	0.04723	Bmp4/Gat	2
GO:0090192	GO:0090192	regulation of glomerulus development	2/374	17/23328	0.029744067	0.07853	0.04723	Bmp4/Pdg	2
GO:0090336	GO:0090336	positive regulation of brown fat cell differentiation	2/374	17/23328	0.029744067	0.07853	0.04723	Metrnl/Pin	2
GO:1900221	GO:1900221	regulation of amyloid-beta clearance	2/374	17/23328	0.029744067	0.07853	0.04723	Clu/Apoe	2
GO:1902894	GO:1902894	negative regulation of pri-miRNA transcription by RNA	2/374	17/23328	0.029744067	0.07853	0.04723	Bmp4/Pdg	2
GO:1905331	GO:1905331	negative regulation of morphogenesis of an epithelium	2/374	17/23328	0.029744067	0.07853	0.04723	Bmp4/Gat	2
GO:2000380	GO:2000380	regulation of mesoderm development	2/374	17/23328	0.029744067	0.07853	0.04723	Sfrp2/Gja	2
GO:0043627	GO:0043627	response to estrogen	4/374	73/23328	0.02979796	0.07863	0.04729	Sfrp1/Cd2	4
GO:0002224	GO:0002224	toll-like receptor signaling pathway	5/374	108/23328	0.030106371	0.07929	0.04769	Cd36/Lbp/	5
GO:0008306	GO:0008306	associative learning	5/374	108/23328	0.030106371	0.07929	0.04769	Btg2/Itgb1	5
GO:0046939	GO:0046939	nucleotide phosphorylation	5/374	108/23328	0.030106371	0.07929	0.04769	Nupr1/Cbf	5
GO:0045787	GO:0045787	positive regulation of cell cycle	11/374	358/23328	0.030497019	0.08027	0.04827	Prox1/Nup	11
GO:0007422	GO:0007422	peripheral nervous system development	4/374	74/23328	0.031116245	0.08184	0.04922	Ednrb/Itgb	4
GO:0002702	GO:0002702	positive regulation of production of molecular mediator	5/374	109/23328	0.031155124	0.08184	0.04922	Gata3/Cd	5
GO:1901800	GO:1901800	positive regulation of proteasomal protein catabolic pro	5/374	109/23328	0.031155124	0.08184	0.04922	Clu/Nupr1	5
GO:0003298	GO:0003298	physiological muscle hypertrophy	3/374	43/23328	0.031454932	0.08237	0.04954	Rgs4/Pi16	3
GO:0003301	GO:0003301	physiological cardiac muscle hypertrophy	3/374	43/23328	0.031454932	0.08237	0.04954	Rgs4/Pi16	3
GO:0055026	GO:0055026	negative regulation of cardiac muscle tissue developm	3/374	43/23328	0.031454932	0.08237	0.04954	Rgs4/Pi16	3
GO:0061049	GO:0061049	cell growth involved in cardiac muscle cell developmer	3/374	43/23328	0.031454932	0.08237	0.04954	Rgs4/Pi16	3
GO:1903792	GO:1903792	negative regulation of anion transport	3/374	43/23328	0.031454932	0.08237	0.04954	Apoe/Thbs	3
GO:0017015	GO:0017015	regulation of transforming growth factor beta receptor s	5/374	110/23328	0.032226528	0.08413	0.0506	Htra1/Lox	5
GO:0022600	GO:0022600	digestive system process	5/374	110/23328	0.032226528	0.08413	0.0506	Scarb1/Kc	5
GO:0032612	GO:0032612	interleukin-1 production	5/374	110/23328	0.032226528	0.08413	0.0506	Cd36/Lpl/	5
GO:0048709	GO:0048709	oligodendrocyte differentiation	5/374	110/23328	0.032226528	0.08413	0.0506	Clu/Bmp4	5
GO:2000106	GO:2000106	regulation of leukocyte apoptotic process	5/374	110/23328	0.032226528	0.08413	0.0506	Bmp4/Cd2	5

GO:0034612	GO:0034612	response to tumor necrosis factor	7/374	188/23328	0.03239043	0.0845	0.05082	Sfrp1/Fab	7
GO:0032637	GO:0032637	interleukin-8 production	4/374	75/23328	0.03246837	0.08455	0.05085	Cd2/Lbp/F	4
GO:0032642	GO:0032642	regulation of chemokine production	4/374	75/23328	0.03246837	0.08455	0.05085	Lbp/Cd24	4
GO:0048645	GO:0048645	animal organ formation	4/374	75/23328	0.03246837	0.08455	0.05085	Bmp4/Pim	4
GO:0046651	GO:0046651	lymphocyte proliferation	10/374	317/23328	0.032679369	0.08504	0.05115	Vcam1/Br	10
GO:0001991	GO:0001991	regulation of systemic arterial blood pressure by circula	2/374	18/23328	0.03311342	0.08506	0.05116	Gja5/F2r	2
GO:0003214	GO:0003214	cardiac left ventricle morphogenesis	2/374	18/23328	0.03311342	0.08506	0.05116	Cpe/Sfrp2	2
GO:0006833	GO:0006833	water transport	2/374	18/23328	0.03311342	0.08506	0.05116	Aqp7/Aqp	2
GO:0007350	GO:0007350	blastoderm segmentation	2/374	18/23328	0.03311342	0.08506	0.05116	Nrp2/Pcsk	2
GO:0009886	GO:0009886	post-embryonic animal morphogenesis	2/374	18/23328	0.03311342	0.08506	0.05116	Kdr/Flt1	2
GO:0021783	GO:0021783	preganglionic parasympathetic fiber development	2/374	18/23328	0.03311342	0.08506	0.05116	Nrp2/Hes1	2
GO:0031571	GO:0031571	mitotic G1 DNA damage checkpoint	2/374	18/23328	0.03311342	0.08506	0.05116	Sox4/Cdk	2
GO:0032793	GO:0032793	positive regulation of CREB transcription factor activity	2/374	18/23328	0.03311342	0.08506	0.05116	Reln/Cd20	2
GO:0035633	GO:0035633	maintenance of blood-brain barrier	2/374	18/23328	0.03311342	0.08506	0.05116	Ptgs1/Cld	2
GO:0042026	GO:0042026	protein refolding	2/374	18/23328	0.03311342	0.08506	0.05116	Hspa5/Hs	2
GO:0044241	GO:0044241	lipid digestion	2/374	18/23328	0.03311342	0.08506	0.05116	Cd36/Aqp	2
GO:0045019	GO:0045019	negative regulation of nitric oxide biosynthetic process	2/374	18/23328	0.03311342	0.08506	0.05116	Ptgis/Cd3	2
GO:0046184	GO:0046184	aldehyde biosynthetic process	2/374	18/23328	0.03311342	0.08506	0.05116	Dkk3/Adh	2
GO:0050710	GO:0050710	negative regulation of cytokine secretion	2/374	18/23328	0.03311342	0.08506	0.05116	Rgcc/Srgr	2
GO:0051000	GO:0051000	positive regulation of nitric-oxide synthase activity	2/374	18/23328	0.03311342	0.08506	0.05116	Scarb1/Ap	2
GO:0060572	GO:0060572	morphogenesis of an epithelial bud	2/374	18/23328	0.03311342	0.08506	0.05116	Bmp4/Pth	2
GO:0070571	GO:0070571	negative regulation of neuron projection regeneration	2/374	18/23328	0.03311342	0.08506	0.05116	Klk8/Thy1	2
GO:1900153	GO:1900153	positive regulation of nuclear-transcribed mRNA catab	2/374	18/23328	0.03311342	0.08506	0.05116	Btg2/Zfp3	2
GO:1901881	GO:1901881	positive regulation of protein depolymerization	2/374	18/23328	0.03311342	0.08506	0.05116	Nes/Stmn	2
GO:1904406	GO:1904406	negative regulation of nitric oxide metabolic process	2/374	18/23328	0.03311342	0.08506	0.05116	Ptgis/Cd3	2
GO:1904706	GO:1904706	negative regulation of vascular associated smooth mus	2/374	18/23328	0.03311342	0.08506	0.05116	Cdkn1a/R	2
GO:1990778	GO:1990778	protein localization to cell periphery	11/374	363/23328	0.033224747	0.08518	0.05123	Krt18/Tme	11
GO:0002221	GO:0002221	pattern recognition receptor signaling pathway	6/374	149/23328	0.033265036	0.08518	0.05123	Cd36/Lbp	6
GO:0001676	GO:0001676	long-chain fatty acid metabolic process	5/374	111/23328	0.033320693	0.08518	0.05123	Pam/Mgll/	5
GO:0072009	GO:0072009	nephron epithelium development	5/374	111/23328	0.033320693	0.08518	0.05123	Bmp4/Gat	5
GO:0010559	GO:0010559	regulation of glycoprotein biosynthetic process	3/374	44/23328	0.033366955	0.08518	0.05123	Bace2/Itm	3
GO:0019883	GO:0019883	antigen processing and presentation of endogenous an	3/374	44/23328	0.033366955	0.08518	0.05123	H2-Q6/H2	3
GO:0042462	GO:0042462	eye photoreceptor cell development	3/374	44/23328	0.033366955	0.08518	0.05123	Cfh/Thy1/	3
GO:0045823	GO:0045823	positive regulation of heart contraction	3/374	44/23328	0.033366955	0.08518	0.05123	Rgs4/Adm	3
GO:0046621	GO:0046621	negative regulation of organ growth	3/374	44/23328	0.033366955	0.08518	0.05123	Rgs4/Pi16	3
GO:1903523	GO:1903523	negative regulation of blood circulation	3/374	44/23328	0.033366955	0.08518	0.05123	Mmp2/Adr	3
GO:0002274	GO:0002274	myeloid leukocyte activation	8/374	231/23328	0.033413821	0.0852	0.05124	Clu/Lbp/T	8
GO:0050670	GO:0050670	regulation of lymphocyte proliferation	8/374	231/23328	0.033413821	0.0852	0.05124	Vcam1/Br	8
GO:0046879	GO:0046879	hormone secretion	11/374	364/23328	0.033790388	0.08607	0.05177	Cpe/Sfrp1	11
GO:0034248	GO:0034248	regulation of cellular amide metabolic process	12/374	410/23328	0.033796643	0.08607	0.05177	Clu/Spon1	12
GO:1903844	GO:1903844	regulation of cellular response to transforming growth f	5/374	112/23328	0.03443772	0.08765	0.05272	Htra1/Lox	5
GO:0032943	GO:0032943	mononuclear cell proliferation	10/374	320/23328	0.034507999	0.08778	0.05279	Vcam1/Br	10
GO:0051783	GO:0051783	regulation of nuclear division	7/374	191/23328	0.034839231	0.08856	0.05327	Bmp4/Pde	7
GO:0010822	GO:0010822	positive regulation of mitochondrion organization	4/374	77/23328	0.035274474	0.08929	0.0537	Kdr/Dcn/C	4
GO:0045843	GO:0045843	negative regulation of striated muscle tissue developm	4/374	77/23328	0.035274474	0.08929	0.0537	Bmp4/Rgs	4
GO:0061097	GO:0061097	regulation of protein tyrosine kinase activity	4/374	77/23328	0.035274474	0.08929	0.0537	Reln/Prnp	4
GO:0071277	GO:0071277	cellular response to calcium ion	4/374	77/23328	0.035274474	0.08929	0.0537	Fos/Junb/	4
GO:0030890	GO:0030890	positive regulation of B cell proliferation	3/374	45/23328	0.03533811	0.08929	0.0537	Cd38/Cdk	3
GO:0032873	GO:0032873	negative regulation of stress-activated MAPK cascade	3/374	45/23328	0.03533811	0.08929	0.0537	Sfrp1/Sfrp	3
GO:0050856	GO:0050856	regulation of T cell receptor signaling pathway	3/374	45/23328	0.03533811	0.08929	0.0537	Kcnn4/Prr	3
GO:0070303	GO:0070303	negative regulation of stress-activated protein kinase s	3/374	45/23328	0.03533811	0.08929	0.0537	Sfrp1/Sfrp	3
GO:0071526	GO:0071526	semaphorin-plexin signaling pathway	3/374	45/23328	0.03533811	0.08929	0.0537	Ncam1/Nr	3
GO:0089718	GO:0089718	amino acid import across plasma membrane	3/374	45/23328	0.03533811	0.08929	0.0537	Rgs4/Itgb	3
GO:0006814	GO:0006814	sodium ion transport	8/374	234/23328	0.035641514	0.08995	0.0541	Cxcl1/Atp	8
GO:0032944	GO:0032944	regulation of mononuclear cell proliferation	8/374	234/23328	0.035641514	0.08995	0.0541	Vcam1/Br	8
GO:0021953	GO:0021953	central nervous system neuron differentiation	8/374	235/23328	0.036405851	0.09137	0.05496	Sfrp1/Cdh	8
GO:0002544	GO:0002544	chronic inflammatory response	2/374	19/23328	0.036624035	0.09137	0.05496	S100a9/G	2
GO:0002726	GO:0002726	positive regulation of T cell cytokine production	2/374	19/23328	0.036624035	0.09137	0.05496	Gata3/Rsa	2
GO:0006525	GO:0006525	arginine metabolic process	2/374	19/23328	0.036624035	0.09137	0.05496	Ass1/Nos	2
GO:0034111	GO:0034111	negative regulation of homotypic cell-cell adhesion	2/374	19/23328	0.036624035	0.09137	0.05496	Cd24a/Se	2
GO:0040037	GO:0040037	negative regulation of fibroblast growth factor receptor	2/374	19/23328	0.036624035	0.09137	0.05496	Gata3/Thb	2
GO:0042730	GO:0042730	fibrinolysis	2/374	19/23328	0.036624035	0.09137	0.05496	Thbs1/Ser	2
GO:0044539	GO:0044539	long-chain fatty acid import into cell	2/374	19/23328	0.036624035	0.09137	0.05496	Cd36/Thb	2
GO:0044783	GO:0044783	G1 DNA damage checkpoint	2/374	19/23328	0.036624035	0.09137	0.05496	Sox4/Cdk	2
GO:0055003	GO:0055003	cardiac myofibril assembly	2/374	19/23328	0.036624035	0.09137	0.05496	Prox1/Act	2
GO:0060033	GO:0060033	anatomical structure regression	2/374	19/23328	0.036624035	0.09137	0.05496	Flt1/Ninj1	2
GO:0060221	GO:0060221	retinal rod cell differentiation	2/374	19/23328	0.036624035	0.09137	0.05496	Cfh/Ptn	2
GO:0060707	GO:0060707	trophoblast giant cell differentiation	2/374	19/23328	0.036624035	0.09137	0.05496	Nr2f2/Soc	2
GO:0060712	GO:0060712	spongiotrophoblast layer development	2/374	19/23328	0.036624035	0.09137	0.05496	Adm/Socs	2
GO:0061081	GO:0061081	positive regulation of myeloid leukocyte cytokine produ	2/374	19/23328	0.036624035	0.09137	0.05496	Cd36/Cd7	2
GO:0072578	GO:0072578	neurotransmitter-gated ion channel clustering	2/374	19/23328	0.036624035	0.09137	0.05496	Apoe/Reln	2
GO:0140354	GO:0140354	lipid import into cell	2/374	19/23328	0.036624035	0.09137	0.05496	Cd36/Thb	2
GO:1903960	GO:1903960	negative regulation of anion transmembrane transport	2/374	19/23328	0.036624035	0.09137	0.05496	Thbs1/Rg	2
GO:1904754	GO:1904754	positive regulation of vascular associated smooth mus	2/374	19/23328	0.036624035	0.09137	0.05496	Pdgfb/Igfb	2
GO:0060042	GO:0060042	retina morphogenesis in camera-type eye	4/374	78/23328	0.036728581	0.0915	0.05503	Cfh/Prox1	4
GO:0007498	GO:0007498	mesoderm development	5/374	114/23328	0.036740743	0.0915	0.05503	Bmp4/Scx	5
GO:0010927	GO:0010927	cellular component assembly involved in morphogenes	5/374	114/23328	0.036740743	0.0915	0.05503	Prox1/Itgb	5
GO:0030183	GO:0030183	B cell differentiation	6/374	153/23328	0.037119774	0.09239	0.05557	Sfrp1/Cd2	6
GO:0045930	GO:0045930	negative regulation of mitotic cell cycle	8/374	236/23328	0.037181168	0.09249	0.05562	Bmp4/Fhl	8
GO:0031952	GO:0031952	regulation of protein autophosphorylation	3/374	46/23328	0.037368077	0.09262	0.0557	Pdgfa/Pdg	3
GO:0043616	GO:0043616	keratinocyte proliferation	3/374	46/23328	0.037368077	0.09262	0.0557	Klk8/Cdh1	3
GO:0045740	GO:0045740	positive regulation of DNA replication	3/374	46/23328	0.037368077	0.09262	0.0557	Cst3/Pdgf	3
GO:0060323	GO:0060323	head morphogenesis	3/374	46/23328	0.037368077	0.09262	0.0557	Scx/Mmp2	3
GO:0061098	GO:0061098	positive regulation of protein tyrosine kinase activity	3/374	46/23328	0.037368077	0.09262	0.0557	Reln/Prnp	3
GO:0098926	GO:0098926	postsynaptic signal transduction	3/374	46/23328	0.037368077	0.09262	0.0557	Ly6c1/Ly6	3
GO:0018958	GO:0018958	phenol-containing compound metabolic process	5/374	115/23328	0.037926912	0.09389	0.05647	Gata3/Ly6	5
GO:0019079	GO:0019079	viral genome replication	5/374	115/23328	0.037926912	0.09389	0.05647	Prox1/Rsa	5
GO:0032602	GO:0032602	chemokine production	4/374	79/23328	0.038216786	0.09439	0.05677	Lbp/Cd24	4
GO:0034644	GO:0034644	cellular response to UV	4/374	79/23328	0.038216786	0.09439	0.05677	Cdkn1a/A	4
GO:0048635	GO:0048635	negative regulation of muscle organ development	4/374	79/23328	0.038216786	0.09439	0.05677	Bmp4/Rgs	4
GO:1902117	GO:1902117	positive regulation of organelle assembly	4/374	79/23328	0.038216786	0.09439	0.05677	Fscn1/Prc	4
GO:0009914	GO:0009914	hormone transport	11/374	372/23328	0.038562754	0.09519	0.05725	Cpe/Sfrp1	11
GO:0002706	GO:0002706	regulation of lymphocyte mediated immunity	7/374	196/23328	0.039187802	0.09667	0.05814	Gata3/Cd	7
GO:0001941	GO:0001941	postsynaptic membrane organization	3/374	47/23328	0.039456498	0.09705	0.05837	Apoe/Reln	3
GO:0003197	GO:0003197	endocardial cushion development	3/374	47/23328	0.039456498	0.09705	0.05837	Bmp4/Thb	3
GO:0035458	GO:0035458	cellular response to interferon-beta	3/374	47/23328	0.039456498	0.09705	0.05837	Ifi203/Mnd	3
GO:0043902	GO:0043902	positive regulation of multi-organism process	3/374	47/23328	0.039456498	0.09705	0.05837	Ednrb/Pde	3
GO:0045214	GO:0045214	sarcomere organization	3/374	47/23328	0.039456498	0.09705	0.05837	Prox1/Itgb	3
GO:0032651	GO:0032651	regulation of interleukin-1 beta production	4/374	80/23328	0.039739117	0.09751	0.05865	Lpl/Mnda	4
GO:0048041	GO:0048041	focal adhesion assembly	4/374	80/23328	0.039739117	0.09751	0.05865	Kdr/Tek/A	4
GO:0050688	GO:0050688	regulation of defense response to virus	4/374	80/23328	0.039739117	0.09751	0.05865	Htra1/Fgl	4
GO:1900542	GO:1900542	regulation of purine nucleotide metabolic process	4/374	80/23328	0.039739117	0.09751	0.05865	Nupr1/Nos	4
GO:0002822	GO:0002822	regulation of adaptive immune response based on som	7/374	197/23328	0.040098192	0.09807	0.05898	Gata3/Cd	7
GO:0006937	GO:0006937	regulation of muscle contraction	6/374	156/23328	0.040190937	0.09807	0.05898	Gja5/Atp1	6
GO:0006883	GO:0006883	cellular sodium ion homeostasis	2/374	20/23328	0.040270545	0.09807	0.05898	Atp1b1/Sg	2
GO:0008209	GO:0008209	androgen metabolic process	2/374	20/23328	0.040270545	0.09807	0.05898	Scarb1/Ac	2
GO:0010818	GO:0010818	T cell chemotaxis	2/374	20/23328	0.040270545	0.09807	0.05898	Cxcl12/Cx	2
GO:0032495	GO:0032495	response to muramyl dipeptide	2/374	20/23328	0.040270545	0.09807	0.05898	Vim/Nfkbi	2
GO:0044819	GO:0044819	mitotic G1/S transition checkpoint	2/374	20/23328	0.040270545	0.09807	0.05898	Sox4/Cdk	2
GO:0046514	GO:0046514	ceramide catabolic process	2/374	20/23328	0.040270545	0.09807	0.05898	Gm2a/Ace	2
GO:0046851	GO:0046851	negative regulation of bone remodeling	2/374	20/23328	0.040270545	0.09807	0.05898	Sfrp1/Cd3	2
GO:0060307	GO:0060307	regulation of ventricular cardiac muscle cell membrane	2/374	20/23328	0.040270545	0.09807	0.05898	Gja5/Gja1	2
GO:0072189	GO:0072189	ureter development	2/374	20/23328	0.040270545	0.09807	0.05898	Bmp4/Gat	2
GO:1900151	GO:1900151	regulation of nuclear-transcribed mRNA catabolic proc	2/374	20/23328	0.040270545	0.09807	0.05898	Btg2/Zfp3	2
GO:1901028	GO:1901028	regulation of mitochondrial outer membrane permeabil	2/374	20/23328	0.040270545	0.09807	0.05898	Chchd10/	2
GO:0006986	GO:0006986	response to unfolded protein	5/374	117/23328	0.04036895	0.09825	0.05909	Thbs1/Hs	5
GO:0006140	GO:0006140	regulation of nucleotide metabolic process	4/374	81/23328	0.041295587	0.10033	0.06034	Nupr1/Nos	4
GO:0021675	GO:0021675	nerve development	4/374	81/23328	0.041295587	0.10033	0.06034	Vcam1/Nr	4
GO:0051881	GO:0051881	regulation of mitochondrial membrane potential	4/374	81/23328	0.041295587	0.10033	0.06034	Kdr/Ucp2/	4
GO:0006110	GO:0006110	regulation of glycolytic process	3/374	48/23328	0.041602977	0.10084	0.06065	Nupr1/Cbf	3
GO:0031111	GO:0031111	negative regulation of microtubule polymerization or de	3/374	48/23328	0.041602977	0.10084	0.06065	Cdh5/Stm	3
GO:0038066	GO:0038066	p38MAPK cascade	3/374	48/23328	0.041602977	0.10084	0.06065	Dusp1/Zfp	3
GO:1902622	GO:1902622	regulation of neutrophil migration	3/374	48/23328	0.041602977	0.10084	0.06065	Lbp/Cd74	3
GO:0009100	GO:0009100	glycoprotein metabolic process	10/374	332/23328	0.042536674	0.10304	0.06197	Cytl1/Gxyl	10
GO:0001912	GO:0001912	positive regulation of leukocyte mediated cytotoxicity	4/374	82/23328	0.042886196	0.10365	0.06234	Cxcl1/H2-	4
GO:0032370	GO:0032370	positive regulation of lipid transport	4/374	82/23328	0.042886196	0.10365	0.06234	Apoe/Nfkb	4
GO:0050805	GO:0050805	negative regulation of synaptic transmission	4/374	82/23328	0.042886196	0.10365	0.06234	Pcdh17/M	4
GO:0050870	GO:0050870	positive regulation of T cell activation	7/374	200/23328	0.042911791	0.10365	0.06234	Vcam1/Ga	7
GO:0071805	GO:0071805	potassium ion transmembrane transport	7/374	200/23328	0.042911791	0.10365	0.06234	Kcnn4/Fhl	7

GO:0071383	GO:0071383	cellular response to steroid hormone stimulus	6/374	159/23328	0.043418597	0.10475	0.063	Sfrp1/Bmp	6
GO:1903900	GO:1903900	regulation of viral life cycle	6/374	159/23328	0.043418597	0.10475	0.063	Prox1/Rsa	6
GO:0032941	GO:0032941	secretion by tissue	3/374	49/23328	0.043807087	0.10523	0.06329	Kcnn4/Aq	3
GO:0043090	GO:0043090	amino acid import	3/374	49/23328	0.043807087	0.10523	0.06329	Rgs4/Itgb	3
GO:0043550	GO:0043550	regulation of lipid kinase activity	3/374	49/23328	0.043807087	0.10523	0.06329	Flt1/Tek/P	3
GO:0090102	GO:0090102	cochlea development	3/374	49/23328	0.043807087	0.10523	0.06329	Cthrc1/He	3
GO:0070507	GO:0070507	regulation of microtubule cytoskeleton organization	7/374	201/23328	0.043877316	0.10523	0.06329	Plk2/Cdh5	7
GO:0002863	GO:0002863	positive regulation of inflammatory response to antiger	2/374	21/23328	0.044047714	0.10523	0.06329	Cd24a/C3	2
GO:0035089	GO:0035089	establishment of apical/basal cell polarity	2/374	21/23328	0.044047714	0.10523	0.06329	Fscn1/Tcf	2
GO:0035357	GO:0035357	peroxisome proliferator activated receptor signaling pa	2/374	21/23328	0.044047714	0.10523	0.06329	Ptgis/Fab	2
GO:0048486	GO:0048486	parasympathetic nervous system development	2/374	21/23328	0.044047714	0.10523	0.06329	Nrp2/Hes	2
GO:0048569	GO:0048569	post-embryonic animal organ development	2/374	21/23328	0.044047714	0.10523	0.06329	Kdr/Flt1	2
GO:0072170	GO:0072170	metanephric tubule development	2/374	21/23328	0.044047714	0.10523	0.06329	Hes1/Aqp	2
GO:0090036	GO:0090036	regulation of protein kinase C signaling	2/374	21/23328	0.044047714	0.10523	0.06329	Flt4/Akap	2
GO:0090050	GO:0090050	positive regulation of cell migration involved in sproutir	2/374	21/23328	0.044047714	0.10523	0.06329	Kdr/Plk2	2
GO:0097734	GO:0097734	extracellular exosome biogenesis	2/374	21/23328	0.044047714	0.10523	0.06329	Sdc1/Cd3	2
GO:1902004	GO:1902004	positive regulation of amyloid-beta formation	2/374	21/23328	0.044047714	0.10523	0.06329	Clu/Apoe	2
GO:2000209	GO:2000209	regulation of anoikis	2/374	21/23328	0.044047714	0.10523	0.06329	Itgb1/Itga5	2
GO:2000726	GO:2000726	negative regulation of cardiac muscle cell differentiatio	2/374	21/23328	0.044047714	0.10523	0.06329	Rgs4/Pi16	2
GO:0007088	GO:0007088	regulation of mitotic nuclear division	6/374	160/23328	0.044529546	0.10633	0.06395	Bmp4/Rg	6
GO:1901136	GO:1901136	carbohydrate derivative catabolic process	5/374	121/23328	0.045533566	0.10866	0.06535	Gm2a/Cst	5
GO:0007520	GO:0007520	myoblast fusion	3/374	50/23328	0.046068367	0.10955	0.06589	Cxcl12/Cx	3
GO:0010677	GO:0010677	negative regulation of cellular carbohydrate metabolic	3/374	50/23328	0.046068367	0.10955	0.06589	Nupr1/Cbf	3
GO:0018198	GO:0018198	peptidyl-cysteine modification	3/374	50/23328	0.046068367	0.10955	0.06589	Gapdh/S1	3
GO:0032715	GO:0032715	negative regulation of interleukin-6 production	3/374	50/23328	0.046068367	0.10955	0.06589	Cd24a/Cd	3
GO:0070169	GO:0070169	positive regulation of biomineral tissue development	3/374	50/23328	0.046068367	0.10955	0.06589	Bmp4/Ptn	3
GO:0110151	GO:0110151	positive regulation of biomineralization	3/374	50/23328	0.046068367	0.10955	0.06589	Bmp4/Ptn	3
GO:0006720	GO:0006720	isoprenoid metabolic process	4/374	84/23328	0.046169757	0.10955	0.06589	Rbp1/Adh	4
GO:0043154	GO:0043154	negative regulation of cysteine-type endopeptidase act	4/374	84/23328	0.046169757	0.10955	0.06589	Sfrp2/Thb	4
GO:0046530	GO:0046530	photoreceptor cell differentiation	4/374	84/23328	0.046169757	0.10955	0.06589	Cfh/Thy1/	4
GO:0051817	GO:0051817	modulation of process of other organism involved in sy	4/374	84/23328	0.046169757	0.10955	0.06589	Apoe/Gap	4
GO:0051937	GO:0051937	catecholamine transport	4/374	85/23328	0.047862641	0.11288	0.06789	Cxcl12/Ly	4
GO:0009154	GO:0009154	purine ribonucleotide catabolic process	2/374	22/23328	0.04795044	0.11288	0.06789	Nudt4/Pde	2
GO:0009261	GO:0009261	ribonucleotide catabolic process	2/374	22/23328	0.04795044	0.11288	0.06789	Nudt4/Pde	2
GO:0014912	GO:0014912	negative regulation of smooth muscle cell migration	2/374	22/23328	0.04795044	0.11288	0.06789	Igfbp3/Igf	2
GO:0018904	GO:0018904	ether metabolic process	2/374	22/23328	0.04795044	0.11288	0.06789	Ephx2/Pla	2
GO:0048532	GO:0048532	anatomical structure arrangement	2/374	22/23328	0.04795044	0.11288	0.06789	Nrp2/Hspa	2
GO:0050860	GO:0050860	negative regulation of T cell receptor signaling pathway	2/374	22/23328	0.04795044	0.11288	0.06789	Prnp/Thy1	2
GO:0051770	GO:0051770	positive regulation of nitric-oxide synthase biosynthetic	2/374	22/23328	0.04795044	0.11288	0.06789	Kdr/Akap1	2
GO:0051873	GO:0051873	killing by host of symbiont cells	2/374	22/23328	0.04795044	0.11288	0.06789	Cxcl1/Gap	2
GO:0072273	GO:0072273	metanephric nephron morphogenesis	2/374	22/23328	0.04795044	0.11288	0.06789	Bmp4/Hes	2
GO:0080111	GO:0080111	DNA demethylation	2/374	22/23328	0.04795044	0.11288	0.06789	Gata3/Apo	2
GO:0099625	GO:0099625	ventricular cardiac muscle cell membrane repolarizatio	2/374	22/23328	0.04795044	0.11288	0.06789	Gja5/Gja1	2
GO:1901223	GO:1901223	negative regulation of NIK/NF-kappaB signaling	2/374	22/23328	0.04795044	0.11288	0.06789	Adgrg3/Ts	2
GO:1903975	GO:1903975	regulation of glial cell migration	2/374	22/23328	0.04795044	0.11288	0.06789	Vim/Efem	2
GO:0009132	GO:0009132	nucleoside diphosphate metabolic process	5/374	123/23328	0.048256909	0.1132	0.06808	Nupr1/Cbf	5
GO:0051261	GO:0051261	protein depolymerization	5/374	123/23328	0.048256909	0.1132	0.06808	Mical2/Ne	5
GO:0001916	GO:0001916	positive regulation of T cell mediated cytotoxicity	3/374	51/23328	0.048386326	0.1132	0.06808	H2-Q6/H2	3
GO:0002474	GO:0002474	antigen processing and presentation of peptide antiger	3/374	51/23328	0.048386326	0.1132	0.06808	H2-Q6/H2	3
GO:0030901	GO:0030901	midbrain development	3/374	51/23328	0.048386326	0.1132	0.06808	Sfrp1/Sfrp	3
GO:0031663	GO:0031663	lipopolysaccharide-mediated signaling pathway	3/374	51/23328	0.048386326	0.1132	0.06808	Lbp/Nos3/	3
GO:0032722	GO:0032722	positive regulation of chemokine production	3/374	51/23328	0.048386326	0.1132	0.06808	Lbp/Lpl/C	3
GO:0046189	GO:0046189	phenol-containing compound biosynthetic process	3/374	51/23328	0.048386326	0.1132	0.06808	Gata3/Ep	3
GO:0046470	GO:0046470	phosphatidylcholine metabolic process	3/374	51/23328	0.048386326	0.1132	0.06808	Scarb1/Pl	3
GO:0051489	GO:0051489	regulation of filopodium assembly	3/374	51/23328	0.048386326	0.1132	0.06808	Dpysl3/Fs	3
GO:0060043	GO:0060043	regulation of cardiac muscle cell proliferation	3/374	51/23328	0.048386326	0.1132	0.06808	Ncam1/Pi	3
GO:1903532	GO:1903532	positive regulation of secretion by cell	11/374	388/23328	0.049479074	0.11569	0.06958	Kcnn4/Cd	11
GO:0035050	GO:0035050	embryonic heart tube development	4/374	86/23328	0.049589525	0.11588	0.0697	Gja5/Hes1	4
GO:1903052	GO:1903052	positive regulation of proteolysis involved in cellular pr	5/374	124/23328	0.049653956	0.11591	0.06971	Clu/Nupr1	5
GO:1905039	GO:1905039	carboxylic acid transmembrane transport	5/374	124/23328	0.049653956	0.11591	0.06971	Thbs1/Rg	5
GO:0030522	GO:0030522	intracellular receptor signaling pathway	7/374	207/23328	0.049964309	0.11656	0.07011	Sfrp1/Ptgi	7
GO:0009620	GO:0009620	response to fungus	3/374	52/23328	0.050760442	0.11796	0.07094	Cxcl1/Pla	3
GO:0030857	GO:0030857	negative regulation of epithelial cell differentiation	3/374	52/23328	0.050760442	0.11796	0.07094	Id1/Hes1/	3
GO:0032731	GO:0032731	positive regulation of interleukin-1 beta production	3/374	52/23328	0.050760442	0.11796	0.07094	Lpl/Mndal	3
GO:0043268	GO:0043268	positive regulation of potassium ion transport	3/374	52/23328	0.050760442	0.11796	0.07094	Fhl1/Cxcl	3
GO:0045124	GO:0045124	regulation of bone resorption	3/374	52/23328	0.050760442	0.11796	0.07094	Cd38/Ahs	3
GO:0045933	GO:0045933	positive regulation of muscle contraction	3/374	52/23328	0.050760442	0.11796	0.07094	Ptgs1/F2r	3
GO:0051353	GO:0051353	positive regulation of oxidoreductase activity	3/374	52/23328	0.050760442	0.11796	0.07094	Scarb1/Ap	3
GO:0010212	GO:0010212	response to ionizing radiation	5/374	125/23328	0.051074613	0.11849	0.07126	Sfrp1/Gat	5
GO:0043280	GO:0043280	positive regulation of cysteine-type endopeptidase acti	5/374	125/23328	0.051074613	0.11849	0.07126	F2r/Acer2	5
GO:1903825	GO:1903825	organic acid transmembrane transport	5/374	125/23328	0.051074613	0.11849	0.07126	Thbs1/Rg	5
GO:0044728	GO:0044728	DNA methylation or demethylation	4/374	87/23328	0.051350342	0.11907	0.07161	Gata3/Me	4
GO:0051896	GO:0051896	regulation of protein kinase B signaling	6/374	166/23328	0.051566886	0.1195	0.07187	Gata3/Thb	6
GO:0003283	GO:0003283	atrial septum development	2/374	23/23328	0.051973751	0.11978	0.07204	Gja5/Sox4	2
GO:0034383	GO:0034383	low-density lipoprotein particle clearance	2/374	23/23328	0.051973751	0.11978	0.07204	Scarb1/Co	2
GO:0034405	GO:0034405	response to fluid shear stress	2/374	23/23328	0.051973751	0.11978	0.07204	Ass1/Klf2	2
GO:0045056	GO:0045056	transcytosis	2/374	23/23328	0.051973751	0.11978	0.07204	Gpihbp1/C	2
GO:0051043	GO:0051043	regulation of membrane protein ectodomain proteolysis	2/374	23/23328	0.051973751	0.11978	0.07204	Apoe/Tim	2
GO:0061162	GO:0061162	establishment of monopolar cell polarity	2/374	23/23328	0.051973751	0.11978	0.07204	Fscn1/Tcf	2
GO:0072202	GO:0072202	cell differentiation involved in metanephros developme	2/374	23/23328	0.051973751	0.11978	0.07204	Bmp4/Pdg	2
GO:0097062	GO:0097062	dendritic spine maintenance	2/374	23/23328	0.051973751	0.11978	0.07204	Apoe/Prnp	2
GO:0140112	GO:0140112	extracellular vesicle biogenesis	2/374	23/23328	0.051973751	0.11978	0.07204	Sdc1/Cd3	2
GO:1901626	GO:1901626	regulation of postsynaptic membrane organization	2/374	23/23328	0.051973751	0.11978	0.07204	Apoe/Ptn	2
GO:0007586	GO:0007586	digestion	5/374	126/23328	0.052518896	0.1209	0.07271	Scarb1/Kc	5
GO:0036294	GO:0036294	cellular response to decreased oxygen levels	5/374	126/23328	0.052518896	0.1209	0.07271	Ptgis/Rgc	5
GO:0042552	GO:0042552	myelination	6/374	167/23328	0.052802094	0.12149	0.07307	Clu/Klk8/I	6
GO:0032436	GO:0032436	positive regulation of proteasomal ubiquitin-dependent	4/374	88/23328	0.053145011	0.12171	0.0732	Clu/Cbfa2	4
GO:0035914	GO:0035914	skeletal muscle cell differentiation	4/374	88/23328	0.053145011	0.12171	0.0732	Scx/Nupr1	4
GO:0042475	GO:0042475	odontogenesis of dentin-containing tooth	4/374	88/23328	0.053145011	0.12171	0.0732	Perp/Bmp	4
GO:0030514	GO:0030514	negative regulation of BMP signaling pathway	3/374	53/23328	0.053190168	0.12171	0.0732	Sfrp1/Htra	3
GO:0030850	GO:0030850	prostate gland development	3/374	53/23328	0.053190168	0.12171	0.0732	Sfrp1/Bmp	3
GO:0032371	GO:0032371	regulation of sterol transport	3/374	53/23328	0.053190168	0.12171	0.0732	Apoe/Nfkb	3
GO:0032374	GO:0032374	regulation of cholesterol transport	3/374	53/23328	0.053190168	0.12171	0.0732	Apoe/Nfkb	3
GO:0032720	GO:0032720	negative regulation of tumor necrosis factor production	3/374	53/23328	0.053190168	0.12171	0.0732	Lbp/Cd24	3
GO:0048247	GO:0048247	lymphocyte chemotaxis	3/374	53/23328	0.053190168	0.12171	0.0732	Cxcl12/Cc	3
GO:2000677	GO:2000677	regulation of transcription regulatory region DNA bindir	3/374	53/23328	0.053190168	0.12171	0.0732	Gata3/Id2	3
GO:0042770	GO:0042770	signal transduction in response to DNA damage	4/374	89/23328	0.054973441	0.12572	0.07561	Sox4/Cdk	4
GO:0051222	GO:0051222	positive regulation of protein transport	10/374	348/23328	0.055102927	0.12595	0.07575	Kcnn4/Cd	10
GO:0071356	GO:0071356	cellular response to tumor necrosis factor	6/374	169/23328	0.055326211	0.12639	0.07601	Sfrp1/Fab	6
GO:0051592	GO:0051592	response to calcium ion	5/374	128/23328	0.055478353	0.12666	0.07618	Fos/Thbs1	5
GO:0031331	GO:0031331	positive regulation of cellular catabolic process	11/374	396/23328	0.055653089	0.12672	0.07622	Clu/Kdr/Ig	11
GO:0035272	GO:0035272	exocrine system development	3/374	54/23328	0.055674929	0.12672	0.07622	Pdgfa/Tgr	3
GO:1900024	GO:1900024	regulation of substrate adhesion-dependent cell spread	3/374	54/23328	0.055674929	0.12672	0.07622	Pdpn/Kan	3
GO:1903556	GO:1903556	negative regulation of tumor necrosis factor superfamil	3/374	54/23328	0.055674929	0.12672	0.07622	Lbp/Cd24	3
GO:2000649	GO:2000649	regulation of sodium ion transmembrane transporter ac	3/374	54/23328	0.055674929	0.12672	0.07622	Atp1b1/Fx	3
GO:0006691	GO:0006691	leukotriene metabolic process	2/374	24/23328	0.056112799	0.12672	0.07622	Pla2g5/Dp	2
GO:0007205	GO:0007205	protein kinase C-activating G protein-coupled receptor	2/374	24/23328	0.056112799	0.12672	0.07622	F2r/Edn1	2
GO:0015732	GO:0015732	prostaglandin transport	2/374	24/23328	0.056112799	0.12672	0.07622	Slco2a1/E	2
GO:0030728	GO:0030728	ovulation	2/374	24/23328	0.056112799	0.12672	0.07622	Nos3/Plat	2
GO:0031670	GO:0031670	cellular response to nutrient	2/374	24/23328	0.056112799	0.12672	0.07622	Lpl/Pim1	2
GO:0031954	GO:0031954	positive regulation of protein autophosphorylation	2/374	24/23328	0.056112799	0.12672	0.07622	Pdgfa/Pdg	2
GO:0035455	GO:0035455	response to interferon-alpha	2/374	24/23328	0.056112799	0.12672	0.07622	Bst2/Ifit1	2
GO:0035510	GO:0035510	DNA dealkylation	2/374	24/23328	0.056112799	0.12672	0.07622	Gata3/Apo	2
GO:0035743	GO:0035743	CD4-positive, alpha-beta T cell cytokine production	2/374	24/23328	0.056112799	0.12672	0.07622	Gata3/Rsa	2
GO:0044331	GO:0044331	cell-cell adhesion mediated by cadherin	2/374	24/23328	0.056112799	0.12672	0.07622	Rgcc/Cdh	2
GO:0061339	GO:0061339	establishment or maintenance of monopolar cell polari	2/374	24/23328	0.056112799	0.12672	0.07622	Fscn1/Tcf	2
GO:0070841	GO:0070841	inclusion body assembly	2/374	24/23328	0.056112799	0.12672	0.07622	Clu/Apoe	2
GO:0090335	GO:0090335	regulation of brown fat cell differentiation	2/374	24/23328	0.056112799	0.12672	0.07622	Metrnl/Pin	2
GO:0097150	GO:0097150	neuronal stem cell population maintenance	2/374	24/23328	0.056112799	0.12672	0.07622	Prox1/Hes	2
GO:0099623	GO:0099623	regulation of cardiac muscle cell membrane repolarizat	2/374	24/23328	0.056112799	0.12672	0.07622	Gja5/Gja1	2
GO:0042098	GO:0042098	T cell proliferation	7/374	213/23328	0.056561382	0.12758	0.07673	Vcam1/Br	7
GO:0007272	GO:0007272	ensheathment of neurons	6/374	170/23328	0.056615166	0.12758	0.07673	Clu/Klk8/I	6
GO:0008366	GO:0008366	axon ensheathment	6/374	170/23328	0.056615166	0.12758	0.07673	Clu/Klk8/I	6
GO:1904064	GO:1904064	positive regulation of cation transmembrane transport	6/374	170/23328	0.056615166	0.12758	0.07673	Bmp4/Apl	6
GO:0006672	GO:0006672	ceramide metabolic process	4/374	90/23328	0.056835526	0.12794	0.07695	Gm2a/Plp	4
GO:0070509	GO:0070509	calcium ion import	4/374	90/23328	0.056835526	0.12794	0.07695	Cxcl12/He	4
GO:0032886	GO:0032886	regulation of microtubule-based process	8/374	258/23328	0.057113725	0.1285	0.07728	Fez1/Plk2	8
GO:0006826	GO:0006826	iron ion transport	3/374	55/23328	0.058214126	0.13041	0.07843	Steap4/M	3
GO:0009948	GO:0009948	anterior/posterior axis specification	3/374	55/23328	0.058214126	0.13041	0.07843	Bmp4/Nra	3

GO:0033619	GO:0033619	membrane protein proteolysis	3/374	55/23328	0.058214126	0.13041	0.07843	Apoe/Bac	3
GO:0045604	GO:0045604	regulation of epidermal cell differentiation	3/374	55/23328	0.058214126	0.13041	0.07843	Bmp4/Hes	3
GO:0045773	GO:0045773	positive regulation of axon extension	3/374	55/23328	0.058214126	0.13041	0.07843	Apoe/Cxc	3
GO:0045912	GO:0045912	negative regulation of carbohydrate metabolic process	3/374	55/23328	0.058214126	0.13041	0.07843	Nupr1/Cbf	3
GO:0046580	GO:0046580	negative regulation of Ras protein signal transduction	3/374	55/23328	0.058214126	0.13041	0.07843	Itgb1/Rasi	3
GO:0050891	GO:0050891	multicellular organismal water homeostasis	3/374	55/23328	0.058214126	0.13041	0.07843	Aqp7/Aqp	3
GO:0050852	GO:0050852	T cell receptor signaling pathway	5/374	130/23328	0.058532307	0.13105	0.07882	Kcnn4/Ga	5
GO:0032890	GO:0032890	regulation of organic acid transport	4/374	91/23328	0.05873115	0.13136	0.079	Thbs1/Rg	4
GO:2000117	GO:2000117	negative regulation of cysteine-type endopeptidase act	4/374	91/23328	0.05873115	0.13136	0.079	Sfrp2/Thb	4
GO:0060041	GO:0060041	retina development in camera-type eye	6/374	172/23328	0.059246958	0.13244	0.07965	Cfh/Prox1	6
GO:0031623	GO:0031623	receptor internalization	5/374	131/23328	0.060094688	0.13407	0.08064	Cd36/Itgb	5
GO:0002703	GO:0002703	regulation of leukocyte mediated immunity	8/374	261/23328	0.060270757	0.13407	0.08064	Gata3/Cx	8
GO:0006706	GO:0006706	steroid catabolic process	2/374	25/23328	0.060362861	0.13407	0.08064	Scarb1/Ap	2
GO:0032897	GO:0032897	negative regulation of viral transcription	2/374	25/23328	0.060362861	0.13407	0.08064	Zfp36/Jun	2
GO:0035813	GO:0035813	regulation of renal sodium excretion	2/374	25/23328	0.060362861	0.13407	0.08064	Ednrb/Edr	2
GO:0036342	GO:0036342	post-anal tail morphogenesis	2/374	25/23328	0.060362861	0.13407	0.08064	Sfrp2/Tcf1	2
GO:0048266	GO:0048266	behavioral response to pain	2/374	25/23328	0.060362861	0.13407	0.08064	Thbs1/Vw	2
GO:0051883	GO:0051883	killing of cells in other organism involved in symbiotic i	2/374	25/23328	0.060362861	0.13407	0.08064	Cxcl1/Gap	2
GO:0061213	GO:0061213	positive regulation of mesonephros development	2/374	25/23328	0.060362861	0.13407	0.08064	Bmp4/Gat	2
GO:1904738	GO:1904738	vascular associated smooth muscle cell migration	2/374	25/23328	0.060362861	0.13407	0.08064	Pdgfb/Igfb	2
GO:1904752	GO:1904752	regulation of vascular associated smooth muscle cell r	2/374	25/23328	0.060362861	0.13407	0.08064	Pdgfb/Igfb	2
GO:1905208	GO:1905208	negative regulation of cardiocyte differentiation	2/374	25/23328	0.060362861	0.13407	0.08064	Rgs4/Pi16	2
GO:0043409	GO:0043409	negative regulation of MAPK cascade	6/374	173/23328	0.060589821	0.13434	0.0808	Sfrp1/Bmp	6
GO:0002832	GO:0002832	negative regulation of response to biotic stimulus	4/374	92/23328	0.060660185	0.13434	0.0808	Htra1/Fgl2	4
GO:0006096	GO:0006096	glycolytic process	4/374	92/23328	0.060660185	0.13434	0.0808	Nupr1/Cbf	4
GO:0001914	GO:0001914	regulation of T cell mediated cytotoxicity	3/374	56/23328	0.060807137	0.13434	0.0808	H2-Q6/H2	3
GO:0021587	GO:0021587	cerebellum morphogenesis	3/374	56/23328	0.060807137	0.13434	0.0808	Prox1/Hsp	3
GO:0044060	GO:0044060	regulation of endocrine process	3/374	56/23328	0.060807137	0.13434	0.0808	Gja5/F2r/	3
GO:0048538	GO:0048538	thymus development	3/374	56/23328	0.060807137	0.13434	0.0808	Gata3/Hes	3
GO:0071675	GO:0071675	regulation of mononuclear cell migration	3/374	56/23328	0.060807137	0.13434	0.0808	Thbs1/Cx	3
GO:0071715	GO:0071715	icosanoid transport	3/374	56/23328	0.060807137	0.13434	0.0808	Slco2a1/F	3
GO:1901571	GO:1901571	fatty acid derivative transport	3/374	56/23328	0.060807137	0.13434	0.0808	Slco2a1/F	3
GO:0043271	GO:0043271	negative regulation of ion transport	6/374	174/23328	0.061950673	0.1368	0.08227	Apoe/Thb	6
GO:0008202	GO:0008202	steroid metabolic process	9/374	309/23328	0.062290875	0.13748	0.08268	Scarb1/Dh	9
GO:0006757	GO:0006757	ATP generation from ADP	4/374	93/23328	0.062622492	0.13806	0.08303	Nupr1/Cbf	4
GO:0009798	GO:0009798	axis specification	4/374	93/23328	0.062622492	0.13806	0.08303	Sfrp1/Bmp	4
GO:0002067	GO:0002067	glandular epithelial cell differentiation	3/374	57/23328	0.063453316	0.13967	0.084	Bmp4/Pro	3
GO:0007585	GO:0007585	respiratory gaseous exchange by respiratory system	3/374	57/23328	0.063453316	0.13967	0.084	Tcf15/Flt4	3
GO:0140115	GO:0140115	export across plasma membrane	3/374	57/23328	0.063453316	0.13967	0.084	Kcnn4/Atp	3
GO:0009895	GO:0009895	negative regulation of catabolic process	9/374	311/23328	0.064311117	0.14112	0.08487	Fez1/Nupr	9
GO:1901990	GO:1901990	regulation of mitotic cell cycle phase transition	8/374	265/23328	0.064647907	0.14112	0.08487	Fhl1/Rgcc	8
GO:0000289	GO:0000289	nuclear-transcribed mRNA poly(A) tail shortening	2/374	26/23328	0.064719337	0.14112	0.08487	Btg2/Zfp3	2
GO:0006195	GO:0006195	purine nucleotide catabolic process	2/374	26/23328	0.064719337	0.14112	0.08487	Nudt4/Pde	2
GO:0007620	GO:0007620	copulation	2/374	26/23328	0.064719337	0.14112	0.08487	Ednrb/Ser	2
GO:0010575	GO:0010575	positive regulation of vascular endothelial growth facto	2/374	26/23328	0.064719337	0.14112	0.08487	Flt4/C3	2
GO:0010592	GO:0010592	positive regulation of lamellipodium assembly	2/374	26/23328	0.064719337	0.14112	0.08487	Fscn1/Aq	2
GO:0021801	GO:0021801	cerebral cortex radial glia guided migration	2/374	26/23328	0.064719337	0.14112	0.08487	Lamb1/Re	2
GO:0022030	GO:0022030	telencephalon glial cell migration	2/374	26/23328	0.064719337	0.14112	0.08487	Lamb1/Re	2
GO:0030204	GO:0030204	chondroitin sulfate metabolic process	2/374	26/23328	0.064719337	0.14112	0.08487	Bgn/Dcn	2
GO:0030325	GO:0030325	adrenal gland development	2/374	26/23328	0.064719337	0.14112	0.08487	Ly6e/Cdk	2
GO:0034123	GO:0034123	positive regulation of toll-like receptor signaling pathwa	2/374	26/23328	0.064719337	0.14112	0.08487	Lbp/Rsad	2
GO:0060065	GO:0060065	uterus development	2/374	26/23328	0.064719337	0.14112	0.08487	Gata3/Cdl	2
GO:0090280	GO:0090280	positive regulation of calcium ion import	2/374	26/23328	0.064719337	0.14112	0.08487	Cxcl12/Pc	2
GO:2000050	GO:2000050	regulation of non-canonical Wnt signaling pathway	2/374	26/23328	0.064719337	0.14112	0.08487	Sfrp1/Sfrp	2
GO:2000637	GO:2000637	positive regulation of gene silencing by miRNA	2/374	26/23328	0.064719337	0.14112	0.08487	Bmp4/Zfp	2
GO:2000679	GO:2000679	positive regulation of transcription regulatory region DN	2/374	26/23328	0.064719337	0.14112	0.08487	Gata3/Ha	2
GO:2000738	GO:2000738	positive regulation of stem cell differentiation	2/374	26/23328	0.064719337	0.14112	0.08487	Bmp4/Ptn	2
GO:0009791	GO:0009791	post-embryonic development	5/374	134/23328	0.064923144	0.14149	0.08509	Bmp4/Kdr	5
GO:0001736	GO:0001736	establishment of planar polarity	3/374	58/23328	0.066151999	0.14381	0.08649	Sfrp1/Cth	3
GO:0001825	GO:0001825	blastocyst formation	3/374	58/23328	0.066151999	0.14381	0.08649	Tm4sf1/P	3
GO:0035019	GO:0035019	somatic stem cell population maintenance	3/374	58/23328	0.066151999	0.14381	0.08649	Sfrp1/Sox	3
GO:0043470	GO:0043470	regulation of carbohydrate catabolic process	3/374	58/23328	0.066151999	0.14381	0.08649	Nupr1/Cbf	3
GO:0019058	GO:0019058	viral life cycle	7/374	221/23328	0.066161969	0.14381	0.08649	Apoe/Prox	7
GO:2001243	GO:2001243	negative regulation of intrinsic apoptotic signaling path	4/374	95/23328	0.066646303	0.14479	0.08708	Clu/Cxcl1	4
GO:0045732	GO:0045732	positive regulation of protein catabolic process	7/374	222/23328	0.0674271	0.14641	0.08805	Clu/Apoe/	7
GO:0045638	GO:0045638	negative regulation of myeloid cell differentiation	4/374	96/23328	0.068707474	0.14835	0.08923	Sfrp1/Lmo	4
GO:1903578	GO:1903578	regulation of ATP metabolic process	4/374	96/23328	0.068707474	0.14835	0.08923	Nupr1/Cbf	4
GO:0007164	GO:0007164	establishment of tissue polarity	3/374	59/23328	0.068902501	0.14835	0.08923	Sfrp1/Cth	3
GO:0060193	GO:0060193	positive regulation of lipase activity	3/374	59/23328	0.068902501	0.14835	0.08923	Gpihbp1/F	3
GO:0060443	GO:0060443	mammary gland morphogenesis	3/374	59/23328	0.068902501	0.14835	0.08923	Bmp4/Igfb	3
GO:0003171	GO:0003171	atrioventricular valve development	2/374	27/23328	0.069177741	0.14835	0.08923	Gja5/Sox4	2
GO:0003338	GO:0003338	metanephros morphogenesis	2/374	27/23328	0.069177741	0.14835	0.08923	Bmp4/Hes	2
GO:0006882	GO:0006882	cellular zinc ion homeostasis	2/374	27/23328	0.069177741	0.14835	0.08923	Mt1/Mt2	2
GO:0014821	GO:0014821	phasic smooth muscle contraction	2/374	27/23328	0.069177741	0.14835	0.08923	Ednrb/Edr	2
GO:0015874	GO:0015874	norepinephrine transport	2/374	27/23328	0.069177741	0.14835	0.08923	Ptgs1/Actl	2
GO:0030212	GO:0030212	hyaluronan metabolic process	2/374	27/23328	0.069177741	0.14835	0.08923	Itih5/Pdgfl	2
GO:0032770	GO:0032770	positive regulation of monooxygenase activity	2/374	27/23328	0.069177741	0.14835	0.08923	Scarb1/Ap	2
GO:0035812	GO:0035812	renal sodium excretion	2/374	27/23328	0.069177741	0.14835	0.08923	Ednrb/Edr	2
GO:0060045	GO:0060045	positive regulation of cardiac muscle cell proliferation	2/374	27/23328	0.069177741	0.14835	0.08923	Ncam1/Pi	2
GO:0060148	GO:0060148	positive regulation of posttranscriptional gene silencing	2/374	27/23328	0.069177741	0.14835	0.08923	Bmp4/Zfp	2
GO:0060716	GO:0060716	labyrinthine layer blood vessel development	2/374	27/23328	0.069177741	0.14835	0.08923	Junb/Hes	2
GO:0061298	GO:0061298	retina vasculature development in camera-type eye	2/374	27/23328	0.069177741	0.14835	0.08923	Arhgef15/	2
GO:0090103	GO:0090103	cochlea morphogenesis	2/374	27/23328	0.069177741	0.14835	0.08923	Cthrc1/Fra	2
GO:0098581	GO:0098581	detection of external biotic stimulus	2/374	27/23328	0.069177741	0.14835	0.08923	Scarb1/Lb	2
GO:0150077	GO:0150077	regulation of neuroinflammatory response	2/374	27/23328	0.069177741	0.14835	0.08923	Nupr1/Cd	2
GO:1903010	GO:1903010	regulation of bone development	2/374	27/23328	0.069177741	0.14835	0.08923	Lox/Kdr	2
GO:1903319	GO:1903319	positive regulation of protein maturation	2/374	27/23328	0.069177741	0.14835	0.08923	Spon1/So	2
GO:1904994	GO:1904994	regulation of leukocyte adhesion to vascular endotheli	2/374	27/23328	0.069177741	0.14835	0.08923	Selp/Cxcl	2
GO:2000727	GO:2000727	positive regulation of cardiac muscle cell differentiation	2/374	27/23328	0.069177741	0.14835	0.08923	Bmp4/Edr	2
GO:1904951	GO:1904951	positive regulation of establishment of protein localizat	10/374	364/23328	0.069903549	0.14983	0.09012	Kcnn4/Cd	10
GO:0035821	GO:0035821	modulation of process of other organism	5/374	137/23328	0.069962768	0.14988	0.09015	Apoe/Gap	5
GO:0009896	GO:0009896	positive regulation of catabolic process	12/374	463/23328	0.071174648	0.1524	0.09166	Clu/Kdr/Ig	12
GO:0071887	GO:0071887	leukocyte apoptotic process	5/374	138/23328	0.071689338	0.15291	0.09196	Bmp4/Cd	5
GO:0045744	GO:0045744	negative regulation of G protein-coupled receptor signa	3/374	60/23328	0.071704119	0.15291	0.09196	Aplnr/Rgs	3
GO:0051893	GO:0051893	regulation of focal adhesion assembly	3/374	60/23328	0.071704119	0.15291	0.09196	Kdr/Tek/T	3
GO:0060324	GO:0060324	face development	3/374	60/23328	0.071704119	0.15291	0.09196	Scx/Mmp	3
GO:0090109	GO:0090109	regulation of cell-substrate junction assembly	3/374	60/23328	0.071704119	0.15291	0.09196	Kdr/Tek/T	3
GO:1901016	GO:1901016	regulation of potassium ion transmembrane transporte	3/374	60/23328	0.071704119	0.15291	0.09196	Fhl1/Atp1	3
GO:1905517	GO:1905517	macrophage migration	3/374	60/23328	0.071704119	0.15291	0.09196	Ednrb/Thb	3
GO:2000134	GO:2000134	negative regulation of G1/S transition of mitotic cell cy	3/374	60/23328	0.071704119	0.15291	0.09196	Fhl1/Sox4	3
GO:0032652	GO:0032652	regulation of interleukin-1 production	4/374	98/23328	0.072927435	0.15528	0.09339	Lpl/Mndal	4
GO:0071482	GO:0071482	cellular response to light stimulus	4/374	98/23328	0.072927435	0.15528	0.09339	Cdkn1a/A	4
GO:0072080	GO:0072080	nephron tubule development	4/374	98/23328	0.072927435	0.15528	0.09339	Bmp4/Gat	4
GO:0002089	GO:0002089	lens morphogenesis in camera-type eye	2/374	28/23328	0.073733706	0.1558	0.0937	Bmp4/Pro	2
GO:0008340	GO:0008340	determination of adult lifespan	2/374	28/23328	0.073733706	0.1558	0.0937	Cfh/Meg3	2
GO:0010458	GO:0010458	exit from mitosis	2/374	28/23328	0.073733706	0.1558	0.0937	Rgcc/Cdk	2
GO:0019835	GO:0019835	cytolysis	2/374	28/23328	0.073733706	0.1558	0.0937	Lbp/Fgl2	2
GO:0034390	GO:0034390	smooth muscle cell apoptotic process	2/374	28/23328	0.073733706	0.1558	0.0937	Gapdh/Ed	2
GO:0034391	GO:0034391	regulation of smooth muscle cell apoptotic process	2/374	28/23328	0.073733706	0.1558	0.0937	Gapdh/Ed	2
GO:0045061	GO:0045061	thymic T cell selection	2/374	28/23328	0.073733706	0.1558	0.0937	Gata3/Cd	2
GO:0045663	GO:0045663	positive regulation of myoblast differentiation	2/374	28/23328	0.073733706	0.1558	0.0937	Igfbp3/Cx	2
GO:0055069	GO:0055069	zinc ion homeostasis	2/374	28/23328	0.073733706	0.1558	0.0937	Mt1/Mt2	2
GO:0060384	GO:0060384	innervation	2/374	28/23328	0.073733706	0.1558	0.0937	Vcam1/Se	2
GO:0061217	GO:0061217	regulation of mesonephros development	2/374	28/23328	0.073733706	0.1558	0.0937	Bmp4/Gat	2
GO:0072395	GO:0072395	signal transduction involved in cell cycle checkpoint	2/374	28/23328	0.073733706	0.1558	0.0937	Sox4/Cdk	2
GO:0072401	GO:0072401	signal transduction involved in DNA integrity checkpoir	2/374	28/23328	0.073733706	0.1558	0.0937	Sox4/Cdk	2
GO:0072422	GO:0072422	signal transduction involved in DNA damage checkpoi	2/374	28/23328	0.073733706	0.1558	0.0937	Sox4/Cdk	2
GO:0090025	GO:0090025	regulation of monocyte chemotaxis	2/374	28/23328	0.073733706	0.1558	0.0937	Cxcl12/Du	2
GO:0042593	GO:0042593	glucose homeostasis	8/374	273/23328	0.073981179	0.15624	0.09397	Vcam1/Co	8
GO:0007584	GO:0007584	response to nutrient	3/374	61/23328	0.074556134	0.15706	0.09446	Rbp1/Lpl/	3
GO:0032732	GO:0032732	positive regulation of interleukin-1 production	3/374	61/23328	0.074556134	0.15706	0.09446	Lpl/Mndal	3
GO:0045776	GO:0045776	negative regulation of blood pressure	3/374	61/23328	0.074556134	0.15706	0.09446	Gja5/Cd3	3
GO:0051851	GO:0051851	modulation by host of symbiont process	3/374	61/23328	0.074556134	0.15706	0.09446	Apoe/Jun/	3
GO:0060986	GO:0060986	endocrine hormone secretion	3/374	61/23328	0.074556134	0.15706	0.09446	Gata3/Aq	3
GO:0021761	GO:0021761	limbic system development	4/374	99/23328	0.075085829	0.15802	0.09504	Prox1/Btg	4
GO:0046031	GO:0046031	ADP metabolic process	4/374	99/23328	0.075085829	0.15802	0.09504	Nupr1/Cbf	4
GO:0033500	GO:0033500	carbohydrate homeostasis	8/374	274/23328	0.075202308	0.15812	0.0951	Vcam1/Co	8
GO:0071453	GO:0071453	cellular response to oxygen levels	5/374	140/23328	0.075212146	0.15812	0.0951	Ptgis/Rgc	5
GO:0006304	GO:0006304	DNA modification	4/374	100/23328	0.077276219	0.16211	0.0975	Gata3/Me	4

GO:0046889	GO:0046889	positive regulation of lipid biosynthetic process	4/374	100/2332ε	0.077276219	0.16211	0.0975	Scarb1/Ap	4
GO:0003229	GO:0003229	ventricular cardiac muscle tissue development	3/374	62/23328	0.077457809	0.16211	0.0975	Prox1/Ly6	3
GO:0006081	GO:0006081	cellular aldehyde metabolic process	3/374	62/23328	0.077457809	0.16211	0.0975	Dkk3/Adh	3
GO:0010171	GO:0010171	body morphogenesis	3/374	62/23328	0.077457809	0.16211	0.0975	Scx/Mmp2	3
GO:0014911	GO:0014911	positive regulation of smooth muscle cell migration	3/374	62/23328	0.077457809	0.16211	0.0975	Tmsb4x/P	3
GO:0050854	GO:0050854	regulation of antigen receptor-mediated signaling pathw	3/374	62/23328	0.077457809	0.16211	0.0975	Kcnn4/Prr	3
GO:0061337	GO:0061337	cardiac conduction	3/374	62/23328	0.077457809	0.16211	0.0975	Gja5/Abcc	3
GO:0098930	GO:0098930	axonal transport	3/374	62/23328	0.077457809	0.16211	0.0975	Fez1/Hspl	3
GO:0002029	GO:0002029	desensitization of G protein-coupled receptor signaling	2/374	29/23328	0.078382978	0.1625	0.09773	Aplnr/Adm	2
GO:0003148	GO:0003148	outflow tract septum morphogenesis	2/374	29/23328	0.078382978	0.1625	0.09773	Bmp4/Nrp	2
GO:0022401	GO:0022401	negative adaptation of signaling pathway	2/374	29/23328	0.078382978	0.1625	0.09773	Aplnr/Adm	2
GO:0030262	GO:0030262	apoptotic nuclear changes	2/374	29/23328	0.078382978	0.1625	0.09773	Igfbp3/Cd	2
GO:0030318	GO:0030318	melanocyte differentiation	2/374	29/23328	0.078382978	0.1625	0.09773	Ednrb/Kitl	2
GO:0030511	GO:0030511	positive regulation of transforming growth factor beta r	2/374	29/23328	0.078382978	0.1625	0.09773	Thbs1/Lrg	2
GO:0033198	GO:0033198	response to ATP	2/374	29/23328	0.078382978	0.1625	0.09773	Abcc9/Hsp	2
GO:0033273	GO:0033273	response to vitamin	2/374	29/23328	0.078382978	0.1625	0.09773	Rbp1/Pim	2
GO:0042403	GO:0042403	thyroid hormone metabolic process	2/374	29/23328	0.078382978	0.1625	0.09773	Gata3/Rel	2
GO:0043032	GO:0043032	positive regulation of macrophage activation	2/374	29/23328	0.078382978	0.1625	0.09773	Lbp/Thbs1	2
GO:0050858	GO:0050858	negative regulation of antigen receptor-mediated signa	2/374	29/23328	0.078382978	0.1625	0.09773	Prnp/Thy1	2
GO:0060571	GO:0060571	morphogenesis of an epithelial fold	2/374	29/23328	0.078382978	0.1625	0.09773	Bmp4/Pth	2
GO:0061311	GO:0061311	cell surface receptor signaling pathway involved in hea	2/374	29/23328	0.078382978	0.1625	0.09773	Bmp4/Har	2
GO:0071353	GO:0071353	cellular response to interleukin-4	2/374	29/23328	0.078382978	0.1625	0.09773	Gata3/Hsp	2
GO:0090140	GO:0090140	regulation of mitochondrial fission	2/374	29/23328	0.078382978	0.1625	0.09773	Kdr/Dcn	2
GO:0090208	GO:0090208	positive regulation of triglyceride metabolic process	2/374	29/23328	0.078382978	0.1625	0.09773	Scarb1/Cc	2
GO:0097345	GO:0097345	mitochondrial outer membrane permeabilization	2/374	29/23328	0.078382978	0.1625	0.09773	Chchd10/I	2
GO:0099622	GO:0099622	cardiac muscle cell membrane repolarization	2/374	29/23328	0.078382978	0.1625	0.09773	Gja5/Gja1	2
GO:1903846	GO:1903846	positive regulation of cellular response to transforming	2/374	29/23328	0.078382978	0.1625	0.09773	Thbs1/Lrg	2
GO:0010821	GO:0010821	regulation of mitochondrion organization	5/374	142/2332ε	0.07882736	0.16325	0.09819	Kdr/Dcn/C	5
GO:0016052	GO:0016052	carbohydrate catabolic process	5/374	142/2332ε	0.07882736	0.16325	0.09819	Nupr1/Gm	5
GO:0060993	GO:0060993	kidney morphogenesis	4/374	101/2332ε	0.079498383	0.1644	0.09887	Bmp4/Gat	4
GO:2000060	GO:2000060	positive regulation of ubiquitin-dependent protein catal	4/374	101/2332ε	0.079498383	0.1644	0.09887	Clu/Cbfa2	4
GO:2001237	GO:2001237	negative regulation of extrinsic apoptotic signaling patl	4/374	101/2332ε	0.079498383	0.1644	0.09887	Bmp4/Sfrp	4
GO:0010506	GO:0010506	regulation of autophagy	8/374	278/2332ε	0.080208022	0.16578	0.09971	Kdr/Fez1/I	8
GO:0010830	GO:0010830	regulation of myotube differentiation	3/374	63/23328	0.080408394	0.16579	0.09971	Cxcl12/Cx	3
GO:0030104	GO:0030104	water homeostasis	3/374	63/23328	0.080408394	0.16579	0.09971	Aqp7/Aqp	3
GO:0045661	GO:0045661	regulation of myoblast differentiation	3/374	63/23328	0.080408394	0.16579	0.09971	Bmp4/Igfb	3
GO:0060038	GO:0060038	cardiac muscle cell proliferation	3/374	63/23328	0.080408394	0.16579	0.09971	Ncam1/Pi	3
GO:0150116	GO:0150116	regulation of cell-substrate junction organization	3/374	63/23328	0.080408394	0.16579	0.09971	Kdr/Tek/T	3
GO:0006956	GO:0006956	complement activation	6/374	187/2332ε	0.08127606	0.16749	0.10074	Cfh/Rgcc/	6
GO:0006914	GO:0006914	autophagy	11/374	425/2332ε	0.082238108	0.16931	0.10183	Kdr/Fez1/I	11
GO:0061919	GO:0061919	process utilizing autophagic mechanism	11/374	425/2332ε	0.082238108	0.16931	0.10183	Kdr/Fez1/I	11
GO:0044772	GO:0044772	mitotic cell cycle phase transition	10/374	376/2332ε	0.082515807	0.16975	0.10209	Fhl1/Rgcc	10
GO:0043624	GO:0043624	cellular protein complex disassembly	5/374	144/2332ε	0.082534321	0.16975	0.10209	Mical2/Ne	5
GO:0010770	GO:0010770	positive regulation of cell morphogenesis involved in d	6/374	188/2332ε	0.082887674	0.17039	0.10248	Apoe/Cxc	6
GO:0002724	GO:0002724	regulation of T cell cytokine production	2/374	30/23328	0.083121412	0.17045	0.10252	Gata3/Rsa	2
GO:0006929	GO:0006929	substrate-dependent cell migration	2/374	30/23328	0.083121412	0.17045	0.10252	Fn1/Pdgfb	2
GO:0030149	GO:0030149	sphingolipid catabolic process	2/374	30/23328	0.083121412	0.17045	0.10252	Gm2a/Ace	2
GO:0061082	GO:0061082	myeloid leukocyte cytokine production	2/374	30/23328	0.083121412	0.17045	0.10252	Cd36/Cd7	2
GO:0120178	GO:0120178	steroid hormone biosynthetic process	2/374	30/23328	0.083121412	0.17045	0.10252	Scarb1/Dk	2
GO:1902305	GO:1902305	regulation of sodium ion transmembrane transport	3/374	64/23328	0.083407125	0.17087	0.10277	Atp1b1/Fx	3
GO:1902807	GO:1902807	negative regulation of cell cycle G1/S phase transition	3/374	64/23328	0.083407125	0.17087	0.10277	Fhl1/Sox4	3
GO:0061136	GO:0061136	regulation of proteasomal protein catabolic process	6/374	189/2332ε	0.084516991	0.17306	0.10408	Clu/Apoe/	6
GO:0070588	GO:0070588	calcium ion transmembrane transport	8/374	282/2332ε	0.085407532	0.1748	0.10513	Bmp4/Apl	8
GO:0120032	GO:0120032	regulation of plasma membrane bounded cell projectio	6/374	190/2332ε	0.086163967	0.17573	0.10569	Dpysl3/Fs	6
GO:0001657	GO:0001657	ureteric bud development	4/374	104/2332ε	0.086353166	0.17573	0.10569	Sfrp1/Bmp	4
GO:0009135	GO:0009135	purine nucleoside diphosphate metabolic process	4/374	104/2332ε	0.086353166	0.17573	0.10569	Nupr1/Cbf	4
GO:0009179	GO:0009179	purine ribonucleoside diphosphate metabolic process	4/374	104/2332ε	0.086353166	0.17573	0.10569	Nupr1/Cbf	4
GO:0015844	GO:0015844	monoamine transport	4/374	104/2332ε	0.086353166	0.17573	0.10569	Cxcl12/Ly	4
GO:0046916	GO:0046916	cellular transition metal ion homeostasis	4/374	104/2332ε	0.086353166	0.17573	0.10569	Mt1/Prnp/	4
GO:0061326	GO:0061326	renal tubule development	4/374	104/2332ε	0.086353166	0.17573	0.10569	Bmp4/Gat	4
GO:0072163	GO:0072163	mesonephric epithelium development	4/374	104/2332ε	0.086353166	0.17573	0.10569	Sfrp1/Bmp	4
GO:0072164	GO:0072164	mesonephric tubule development	4/374	104/2332ε	0.086353166	0.17573	0.10569	Sfrp1/Bmp	4
GO:1901222	GO:1901222	regulation of NIK/NF-kappaB signaling	4/374	104/2332ε	0.086353166	0.17573	0.10569	Tek/Edn1/	4
GO:0032233	GO:0032233	positive regulation of actin filament bundle assembly	3/374	65/23328	0.086453224	0.17573	0.10569	Rgcc/Arhc	3
GO:0042461	GO:0042461	photoreceptor cell development	3/374	65/23328	0.086453224	0.17573	0.10569	Cfh/Thy1/	3
GO:0045840	GO:0045840	positive regulation of mitotic nuclear division	3/374	65/23328	0.086453224	0.17573	0.10569	Rgcc/Pdg	3
GO:0090398	GO:0090398	cellular senescence	3/374	65/23328	0.086453224	0.17573	0.10569	Plk2/Cdkr	3
GO:0061351	GO:0061351	neural precursor cell proliferation	6/374	191/2332ε	0.087828553	0.17764	0.10684	Prox1/Btg	6
GO:0000132	GO:0000132	establishment of mitotic spindle orientation	2/374	31/23328	0.087944973	0.17764	0.10684	Itgb1/Gja1	2
GO:0008299	GO:0008299	isoprenoid biosynthetic process	2/374	31/23328	0.087944973	0.17764	0.10684	Rbp1/Cyp	2
GO:0023058	GO:0023058	adaptation of signaling pathway	2/374	31/23328	0.087944973	0.17764	0.10684	Aplnr/Adm	2
GO:0043276	GO:0043276	anoikis	2/374	31/23328	0.087944973	0.17764	0.10684	Itgb1/Itga5	2
GO:0044062	GO:0044062	regulation of excretion	2/374	31/23328	0.087944973	0.17764	0.10684	Ednrb/Edr	2
GO:0060074	GO:0060074	synapse maturation	2/374	31/23328	0.087944973	0.17764	0.10684	Arhgef15/	2
GO:0060795	GO:0060795	cell fate commitment involved in formation of primary g	2/374	31/23328	0.087944973	0.17764	0.10684	Bmp4/Sfrp	2
GO:0061036	GO:0061036	positive regulation of cartilage development	2/374	31/23328	0.087944973	0.17764	0.10684	Bmp4/Scx	2
GO:0070670	GO:0070670	response to interleukin-4	2/374	31/23328	0.087944973	0.17764	0.10684	Gata3/Hsp	2
GO:0072574	GO:0072574	hepatocyte proliferation	2/374	31/23328	0.087944973	0.17764	0.10684	Prox1/Ptn	2
GO:0072575	GO:0072575	epithelial cell proliferation involved in liver morphogene	2/374	31/23328	0.087944973	0.17764	0.10684	Prox1/Ptn	2
GO:1902042	GO:1902042	negative regulation of extrinsic apoptotic signaling patl	2/374	31/23328	0.087944973	0.17764	0.10684	Sfrp2/Hm	2
GO:0001910	GO:0001910	regulation of leukocyte mediated cytotoxicity	4/374	105/2332ε	0.088700032	0.17908	0.1077	Cxcl1/H2-	4
GO:0030072	GO:0030072	peptide hormone secretion	8/374	285/2332ε	0.089434077	0.18047	0.10854	Cpe/Sfrp1	8
GO:0060491	GO:0060491	regulation of cell projection assembly	6/374	192/2332ε	0.089510698	0.18052	0.10857	Dpysl3/Fs	6
GO:2000736	GO:2000736	regulation of stem cell differentiation	3/374	66/23328	0.089545904	0.18052	0.10857	Bmp4/Hes	3
GO:0002449	GO:0002449	lymphocyte mediated immunity	11/374	432/2332ε	0.089666544	0.18068	0.10866	Gata3/Cd2	11
GO:0000768	GO:0000768	syncytium formation by plasma membrane fusion	3/374	67/23328	0.092684364	0.18574	0.11171	Cxcl12/Cx	3
GO:0021695	GO:0021695	cerebellar cortex development	3/374	67/23328	0.092684364	0.18574	0.11171	Prox1/Hsp	3
GO:0140253	GO:0140253	cell-cell fusion	3/374	67/23328	0.092684364	0.18574	0.11171	Cxcl12/Cx	3
GO:2000242	GO:2000242	negative regulation of reproductive process	3/374	67/23328	0.092684364	0.18574	0.11171	Bmp4/Arh	3
GO:0002902	GO:0002902	regulation of B cell apoptotic process	2/374	32/23328	0.092849731	0.18574	0.11171	Cd24a/Cd	2
GO:0010574	GO:0010574	regulation of vascular endothelial growth factor produc	2/374	32/23328	0.092849731	0.18574	0.11171	Flt4/C3	2
GO:0010614	GO:0010614	negative regulation of cardiac muscle hypertrophy	2/374	32/23328	0.092849731	0.18574	0.11171	Rgs4/Pi16	2
GO:0010939	GO:0010939	regulation of necrotic cell death	2/374	32/23328	0.092849731	0.18574	0.11171	Bok/Slc6a	2
GO:0016242	GO:0016242	negative regulation of macroautophagy	2/374	32/23328	0.092849731	0.18574	0.11171	Fez1/Nupr	2
GO:0046475	GO:0046475	glycerophospholipid catabolic process	2/374	32/23328	0.092849731	0.18574	0.11171	Scarb1/Pl	2
GO:0051491	GO:0051491	positive regulation of filopodium assembly	2/374	32/23328	0.092849731	0.18574	0.11171	Dpysl3/Fs	2
GO:0060306	GO:0060306	regulation of membrane repolarization	2/374	32/23328	0.092849731	0.18574	0.11171	Gja5/Gja1	2
GO:0090075	GO:0090075	relaxation of muscle	2/374	32/23328	0.092849731	0.18574	0.11171	Atp1b1/Sl	2
GO:0099560	GO:0099560	synaptic membrane adhesion	2/374	32/23328	0.092849731	0.18574	0.11171	Pcdh17/S	2
GO:1902110	GO:1902110	positive regulation of mitochondrial membrane permea	2/374	32/23328	0.092849731	0.18574	0.11171	Chchd10/I	2
GO:0001823	GO:0001823	mesonephros development	4/374	107/2332ε	0.093485092	0.18684	0.11237	Sfrp1/Bmp	4
GO:0019395	GO:0019395	fatty acid oxidation	4/374	107/2332ε	0.093485092	0.18684	0.11237	Adh7/Cd3	4
GO:0032147	GO:0032147	activation of protein kinase activity	7/374	242/2332ε	0.09576509	0.19107	0.11491	Bmp4/Thb	7
GO:0015800	GO:0015800	acidic amino acid transport	3/374	68/23328	0.095867795	0.19107	0.11491	Itgb1/Slc6	3
GO:0022029	GO:0022029	telencephalon cell migration	3/374	68/23328	0.095867795	0.19107	0.11491	Cxcl12/La	3
GO:0050432	GO:0050432	catecholamine secretion	3/374	68/23328	0.095867795	0.19107	0.11491	Cxcl12/Ly	3
GO:0006119	GO:0006119	oxidative phosphorylation	4/374	108/2332ε	0.095922736	0.19107	0.11491	Nupr1/Cox	4
GO:0009185	GO:0009185	ribonucleoside diphosphate metabolic process	4/374	108/2332ε	0.095922736	0.19107	0.11491	Nupr1/Cbf	4
GO:0032091	GO:0032091	negative regulation of protein binding	4/374	108/2332ε	0.095922736	0.19107	0.11491	Actb/Id1/C	4
GO:0008643	GO:0008643	carbohydrate transport	5/374	151/2332ε	0.096219783	0.19157	0.11521	Aqp7/Fab	5
GO:0000186	GO:0000186	activation of MAPKK activity	2/374	33/23328	0.097831859	0.19367	0.11648	Bmp4/F2r	2
GO:0003081	GO:0003081	regulation of systemic arterial blood pressure by renin-	2/374	33/23328	0.097831859	0.19367	0.11648	Gja5/F2r	2
GO:0022617	GO:0022617	extracellular matrix disassembly	2/374	33/23328	0.097831859	0.19367	0.11648	Fscn1/Pdp	2
GO:0031112	GO:0031112	positive regulation of microtubule polymerization or de	2/374	33/23328	0.097831859	0.19367	0.11648	Gda/Stmn	2
GO:0032891	GO:0032891	negative regulation of organic acid transport	2/374	33/23328	0.097831859	0.19367	0.11648	Thbs1/Rg	2
GO:0033598	GO:0033598	mammary gland epithelial cell proliferation	2/374	33/23328	0.097831859	0.19367	0.11648	Gata3/Id2	2
GO:0034260	GO:0034260	negative regulation of GTPase activity	2/374	33/23328	0.097831859	0.19367	0.11648	Pecam1/F	2
GO:0045652	GO:0045652	regulation of megakaryocyte differentiation	2/374	33/23328	0.097831859	0.19367	0.11648	Lox/Hmgb	2
GO:0045745	GO:0045745	positive regulation of G protein-coupled receptor signa	2/374	33/23328	0.097831859	0.19367	0.11648	Ece1/C3	2
GO:0050999	GO:0050999	regulation of nitric-oxide synthase activity	2/374	33/23328	0.097831859	0.19367	0.11648	Scarb1/Ap	2
GO:0072576	GO:0072576	liver morphogenesis	2/374	33/23328	0.097831859	0.19367	0.11648	Prox1/Ptn	2
GO:1902745	GO:1902745	positive regulation of lamellipodium organization	2/374	33/23328	0.097831859	0.19367	0.11648	Fscn1/Aq	2
GO:0050773	GO:0050773	regulation of dendrite development	6/374	197/2332ε	0.098182828	0.19427	0.11684	Apoe/Plk2	6
GO:0001959	GO:0001959	regulation of cytokine-mediated signaling pathway	4/374	109/2332ε	0.098390073	0.19449	0.11697	Cd24a/Irf	4
GO:1900006	GO:1900006	positive regulation of dendrite development	4/374	109/2332ε	0.098390073	0.19449	0.11697	Apoe/Reln	4
GO:0001913	GO:0001913	T cell mediated cytotoxicity	3/374	69/23328	0.099095379	0.19561	0.11765	H2-Q6/H2	3
GO:0006949	GO:0006949	syncytium formation	3/374	69/23328	0.099095379	0.19561	0.11765	Cxcl12/Cx	3
GO:0008542	GO:0008542	visual learning	3/374	69/23328	0.099095379	0.19561	0.11765	Itgb1/Nts/	3
GO:1990138	GO:1990138	neuron projection extension	6/374	198/2332ε	0.099969095	0.19724	0.11863	Mgll/Apoe	6

GO:0038061	GO:0038061	NIK/NF-kappaB signaling	4/374	110/23328	0.100886811	0.19886	0.1196	Tek/Edn1/	4
GO:0120034	GO:0120034	positive regulation of plasma membrane bounded cell	4/374	110/23328	0.100886811	0.19886	0.1196	Dpysl3/Fs	4
GO:1901361	GO:1901361	organic cyclic compound catabolic process	11/374	442/23328	0.100967174	0.19893	0.11964	Scarb1/Ep	11
GO:0003143	GO:0003143	embryonic heart tube morphogenesis	3/374	70/23328	0.102366289	0.20121	0.12101	Hes1/Han	3
GO:0017144	GO:0017144	drug metabolic process	3/374	70/23328	0.102366289	0.20121	0.12101	Cyp1a1/C	3
GO:0045669	GO:0045669	positive regulation of osteoblast differentiation	3/374	70/23328	0.102366289	0.20121	0.12101	Bmp4/Cth	3
GO:0048488	GO:0048488	synaptic vesicle endocytosis	3/374	70/23328	0.102366289	0.20121	0.12101	Cd24a/Ac	3
GO:0140238	GO:0140238	presynaptic endocytosis	3/374	70/23328	0.102366289	0.20121	0.12101	Cd24a/Ac	3
GO:0006884	GO:0006884	cell volume homeostasis	2/374	34/23328	0.102887632	0.20138	0.12112	Kcnn4/Aq	2
GO:0032689	GO:0032689	negative regulation of interferon-gamma production	2/374	34/23328	0.102887632	0.20138	0.12112	Gata3/Prn	2
GO:0032728	GO:0032728	positive regulation of interferon-beta production	2/374	34/23328	0.102887632	0.20138	0.12112	Irf7/Hmgb	2
GO:0035883	GO:0035883	enteroendocrine cell differentiation	2/374	34/23328	0.102887632	0.20138	0.12112	Bmp4/Hes	2
GO:0046466	GO:0046466	membrane lipid catabolic process	2/374	34/23328	0.102887632	0.20138	0.12112	Gm2a/Ace	2
GO:0048048	GO:0048048	embryonic eye morphogenesis	2/374	34/23328	0.102887632	0.20138	0.12112	Prox1/Mfa	2
GO:0090181	GO:0090181	regulation of cholesterol metabolic process	2/374	34/23328	0.102887632	0.20138	0.12112	Ephx2/Ap	2
GO:1902686	GO:1902686	mitochondrial outer membrane permeabilization involv	2/374	34/23328	0.102887632	0.20138	0.12112	Chchd10/	2
GO:2000406	GO:2000406	positive regulation of T cell migration	2/374	34/23328	0.102887632	0.20138	0.12112	Cxcl12/Cc	2
GO:0008637	GO:0008637	apoptotic mitochondrial changes	4/374	111/23328	0.103412647	0.20222	0.12162	Cd24a/Ch	4
GO:0016241	GO:0016241	regulation of macroautophagy	4/374	111/23328	0.103412647	0.20222	0.12162	Kdr/Fez1/	4
GO:0002460	GO:0002460	adaptive immune response based on somatic recombi	11/374	445/23328	0.104514975	0.20428	0.12286	Gata3/Cd2	11
GO:0042176	GO:0042176	regulation of protein catabolic process	10/374	395/23328	0.105164544	0.20545	0.12356	Clu/Apoe/	10
GO:0032869	GO:0032869	cellular response to insulin stimulus	6/374	201/23328	0.10543032	0.20587	0.12382	C1qtnf9/L	6
GO:0002437	GO:0002437	inflammatory response to antigenic stimulus	3/374	71/23328	0.105679692	0.20597	0.12388	Cd24a/Hn	3
GO:0021885	GO:0021885	forebrain cell migration	3/374	71/23328	0.105679692	0.20597	0.12388	Cxcl12/La	3
GO:0042509	GO:0042509	regulation of tyrosine phosphorylation of STAT protein	3/374	71/23328	0.105679692	0.20597	0.12388	Pecam1/H	3
GO:0060675	GO:0060675	ureteric bud morphogenesis	3/374	71/23328	0.105679692	0.20597	0.12388	Bmp4/Gat	3
GO:0007589	GO:0007589	body fluid secretion	4/374	112/23328	0.105967274	0.20644	0.12416	Kcnn4/Aq	4
GO:0031667	GO:0031667	response to nutrient levels	9/374	346/23328	0.106372438	0.20713	0.12457	Rbp1/Fstl	9
GO:1901987	GO:1901987	regulation of cell cycle phase transition	8/374	297/23328	0.106619476	0.20751	0.12481	Fhl1/Rgcc	8
GO:0034976	GO:0034976	response to endoplasmic reticulum stress	7/374	249/23328	0.107032439	0.20822	0.12523	Clu/Nupr1	7
GO:0002082	GO:0002082	regulation of oxidative phosphorylation	2/374	35/23328	0.108013424	0.20906	0.12573	Nupr1/Pde	2
GO:0002446	GO:0002446	neutrophil mediated immunity	2/374	35/23328	0.108013424	0.20906	0.12573	Cxcl1/Anx	2
GO:0003016	GO:0003016	respiratory system process	2/374	35/23328	0.108013424	0.20906	0.12573	Tcf15/Flt4	2
GO:0010591	GO:0010591	regulation of lamellipodium assembly	2/374	35/23328	0.108013424	0.20906	0.12573	Fscn1/Aq	2
GO:0034331	GO:0034331	cell junction maintenance	2/374	35/23328	0.108013424	0.20906	0.12573	F2r/Chchc	2
GO:0040001	GO:0040001	establishment of mitotic spindle localization	2/374	35/23328	0.108013424	0.20906	0.12573	Itgb1/Gja1	2
GO:0043243	GO:0043243	positive regulation of protein-containing complex disas	2/374	35/23328	0.108013424	0.20906	0.12573	Nes/Stmn	2
GO:0046677	GO:0046677	response to antibiotic	2/374	35/23328	0.108013424	0.20906	0.12573	Id1/Crip1	2
GO:0048147	GO:0048147	negative regulation of fibroblast proliferation	2/374	35/23328	0.108013424	0.20906	0.12573	Sfrp1/Nup	2
GO:1990089	GO:1990089	response to nerve growth factor	2/374	35/23328	0.108013424	0.20906	0.12573	Hes1/Stm	2
GO:1990090	GO:1990090	cellular response to nerve growth factor stimulus	2/374	35/23328	0.108013424	0.20906	0.12573	Hes1/Stm	2
GO:0009416	GO:0009416	response to light stimulus	8/374	298/23328	0.10812877	0.20918	0.12581	Itgb1/Cdk	8
GO:0030516	GO:0030516	regulation of axon extension	4/374	113/23328	0.108550377	0.2099	0.12624	Mgll/Apoe	4
GO:0021543	GO:0021543	pallium development	5/374	157/23328	0.108807364	0.2102	0.12642	Prox1/Btg	5
GO:0060402	GO:0060402	calcium ion transport into cytosol	5/374	157/23328	0.108807364	0.2102	0.12642	Bmp4/Apl	5
GO:0072171	GO:0072171	mesonephric tubule morphogenesis	3/374	72/23328	0.109034747	0.21054	0.12663	Bmp4/Gat	3
GO:0007411	GO:0007411	axon guidance	7/374	251/23328	0.110377187	0.21304	0.12813	Ncam1/Ga	7
GO:0034250	GO:0034250	positive regulation of cellular amide metabolic process	5/374	158/23328	0.110979983	0.214	0.12871	Clu/Apoe/	5
GO:0055067	GO:0055067	monovalent inorganic cation homeostasis	5/374	158/23328	0.110979983	0.214	0.12871	Ednrb/Atp	5
GO:0032649	GO:0032649	regulation of interferon-gamma production	4/374	114/23328	0.111161637	0.21406	0.12874	Gata3/Cd2	4
GO:0034440	GO:0034440	lipid oxidation	4/374	114/23328	0.111161637	0.21406	0.12874	Adh7/Cd3	4
GO:0042100	GO:0042100	B cell proliferation	4/374	114/23328	0.111161637	0.21406	0.12874	Cd38/Cd2	4
GO:0006091	GO:0006091	generation of precursor metabolites and energy	10/374	400/23328	0.111667763	0.21493	0.12927	Adh7/Adh	10
GO:0097485	GO:0097485	neuron projection guidance	7/374	252/23328	0.112070234	0.21561	0.12967	Ncam1/Ga	7
GO:0007632	GO:0007632	visual behavior	3/374	73/23328	0.11243061	0.2159	0.12985	Itgb1/Nts/	3
GO:0021879	GO:0021879	forebrain neuron differentiation	3/374	73/23328	0.11243061	0.2159	0.12985	Prox1/Nrp	3
GO:0042130	GO:0042130	negative regulation of T cell proliferation	3/374	73/23328	0.11243061	0.2159	0.12985	Bmp4/Cd2	3
GO:1903793	GO:1903793	positive regulation of anion transport	3/374	73/23328	0.11243061	0.2159	0.12985	Apoe/Itgb	3
GO:0002700	GO:0002700	regulation of production of molecular mediator of immu	5/374	159/23328	0.113173535	0.2161	0.12997	Gata3/Cd	5
GO:0009266	GO:0009266	response to temperature stimulus	5/374	159/23328	0.113173535	0.2161	0.12997	Cxcl12/Uc	5
GO:0006775	GO:0006775	fat-soluble vitamin metabolic process	2/374	36/23328	0.113205706	0.2161	0.12997	Rbp1/Pltp	2
GO:0010573	GO:0010573	vascular endothelial growth factor production	2/374	36/23328	0.113205706	0.2161	0.12997	Flt4/C3	2
GO:0034142	GO:0034142	toll-like receptor 4 signaling pathway	2/374	36/23328	0.113205706	0.2161	0.12997	Lbp/Nfkbi	2
GO:0048679	GO:0048679	regulation of axon regeneration	2/374	36/23328	0.113205706	0.2161	0.12997	Klk8/Ptn	2
GO:0060351	GO:0060351	cartilage development involved in endochondral bone	2/374	36/23328	0.113205706	0.2161	0.12997	Thbs1/Col	2
GO:0061384	GO:0061384	heart trabecula morphogenesis	2/374	36/23328	0.113205706	0.2161	0.12997	Tek/S1pr1	2
GO:1902116	GO:1902116	negative regulation of organelle assembly	2/374	36/23328	0.113205706	0.2161	0.12997	Fez1/Nupr	2
GO:1905209	GO:1905209	positive regulation of cardiocyte differentiation	2/374	36/23328	0.113205706	0.2161	0.12997	Bmp4/Edr	2
GO:2000191	GO:2000191	regulation of fatty acid transport	2/374	36/23328	0.113205706	0.2161	0.12997	Thbs1/Edr	2
GO:2000758	GO:2000758	positive regulation of peptidyl-lysine acetylation	2/374	36/23328	0.113205706	0.2161	0.12997	Gata3/Sox	2
GO:2000785	GO:2000785	regulation of autophagosome assembly	2/374	36/23328	0.113205706	0.2161	0.12997	Fez1/Nupr	2
GO:0001776	GO:0001776	leukocyte homeostasis	4/374	115/23328	0.113800726	0.21703	0.13053	Cfh/Cd24a	4
GO:0019751	GO:0019751	polyol metabolic process	4/374	115/23328	0.113800726	0.21703	0.13053	Plpp3/Plp	4
GO:0051302	GO:0051302	regulation of cell division	5/374	160/23328	0.115387857	0.21996	0.13229	Sfrp2/Pdg	5
GO:0090068	GO:0090068	positive regulation of cell cycle process	7/374	254/23328	0.115497407	0.22007	0.13236	Rgcc/Sox	7
GO:0006029	GO:0006029	proteoglycan metabolic process	3/374	74/23328	0.115866428	0.22047	0.1326	Cytl1/Bgn	3
GO:0021766	GO:0021766	hippocampus development	3/374	74/23328	0.115866428	0.22047	0.1326	Prox1/Btg	3
GO:2001021	GO:2001021	negative regulation of response to DNA damage stimu	3/374	74/23328	0.115866428	0.22047	0.1326	Clu/Cxcl1	3
GO:0051952	GO:0051952	regulation of amine transport	4/374	116/23328	0.116467313	0.22151	0.13322	Cxcl12/Rg	4
GO:0002053	GO:0002053	positive regulation of mesenchymal cell proliferation	2/374	37/23328	0.118461042	0.22418	0.13483	Kdr/Pdgfa	2
GO:0002218	GO:0002218	activation of innate immune response	2/374	37/23328	0.118461042	0.22418	0.13483	Ifi203/Mnc	2
GO:0009595	GO:0009595	detection of biotic stimulus	2/374	37/23328	0.118461042	0.22418	0.13483	Scarb1/Lb	2
GO:0030574	GO:0030574	collagen catabolic process	2/374	37/23328	0.118461042	0.22418	0.13483	Mmp2/Itgb	2
GO:0035794	GO:0035794	positive regulation of mitochondrial membrane permea	2/374	37/23328	0.118461042	0.22418	0.13483	Chchd10/	2
GO:0046329	GO:0046329	negative regulation of JNK cascade	2/374	37/23328	0.118461042	0.22418	0.13483	Sfrp1/Sfrp	2
GO:0048846	GO:0048846	axon extension involved in axon guidance	2/374	37/23328	0.118461042	0.22418	0.13483	Cxcl12/Nr	2
GO:0051294	GO:0051294	establishment of spindle orientation	2/374	37/23328	0.118461042	0.22418	0.13483	Itgb1/Gja1	2
GO:1902284	GO:1902284	neuron projection extension involved in neuron project	2/374	37/23328	0.118461042	0.22418	0.13483	Cxcl12/Nr	2
GO:1903427	GO:1903427	negative regulation of reactive oxygen species biosynt	2/374	37/23328	0.118461042	0.22418	0.13483	Ptgis/Cd3	2
GO:2000273	GO:2000273	positive regulation of signaling receptor activity	2/374	37/23328	0.118461042	0.22418	0.13483	Itgb1/Reln	2
GO:0050770	GO:0050770	regulation of axonogenesis	6/374	208/23328	0.118760257	0.22464	0.13511	Mgll/Apoe	6
GO:1901890	GO:1901890	positive regulation of cell junction assembly	4/374	117/23328	0.11916106	0.22523	0.13546	Kdr/Tek/C	4
GO:0007260	GO:0007260	tyrosine phosphorylation of STAT protein	3/374	75/23328	0.119341347	0.22523	0.13546	Pecam1/H	3
GO:0010507	GO:0010507	negative regulation of autophagy	3/374	75/23328	0.119341347	0.22523	0.13546	Fez1/Nupr	3
GO:0042306	GO:0042306	regulation of protein import into nucleus	3/374	75/23328	0.119341347	0.22523	0.13546	Bmp4/Cd3	3
GO:0046626	GO:0046626	regulation of insulin receptor signaling pathway	3/374	75/23328	0.119341347	0.22523	0.13546	Ahsg/Socs	3
GO:0006650	GO:0006650	glycerophospholipid metabolic process	7/374	257/23328	0.12073999	0.22766	0.13692	Scarb1/Pl	7
GO:0051403	GO:0051403	stress-activated MAPK cascade	7/374	257/23328	0.12073999	0.22766	0.13692	Sfrp1/Sfrp	7
GO:0008543	GO:0008543	fibroblast growth factor receptor signaling pathway	3/374	76/23328	0.122854508	0.23155	0.13926	Gata3/Thb	3
GO:0001953	GO:0001953	negative regulation of cell-matrix adhesion	2/374	38/23328	0.123776091	0.23234	0.13974	Thbs1/Acc	2
GO:0002861	GO:0002861	regulation of inflammatory response to antigenic stimu	2/374	38/23328	0.123776091	0.23234	0.13974	Cd24a/C3	2
GO:0021799	GO:0021799	cerebral cortex radially oriented cell migration	2/374	38/23328	0.123776091	0.23234	0.13974	Lamb1/Re	2
GO:0030851	GO:0030851	granulocyte differentiation	2/374	38/23328	0.123776091	0.23234	0.13974	Rbp1/Cbfa	2
GO:0043267	GO:0043267	negative regulation of potassium ion transport	2/374	38/23328	0.123776091	0.23234	0.13974	Rgs4/Nos	2
GO:0051953	GO:0051953	negative regulation of amine transport	2/374	38/23328	0.123776091	0.23234	0.13974	Rgs4/Ptgs	2
GO:0062208	GO:0062208	positive regulation of pattern recognition receptor signa	2/374	38/23328	0.123776091	0.23234	0.13974	Lbp/Rsad	2
GO:0110111	GO:0110111	negative regulation of animal organ morphogenesis	2/374	38/23328	0.123776091	0.23234	0.13974	Bmp4/Gat	2
GO:2000463	GO:2000463	positive regulation of excitatory postsynaptic potential	2/374	38/23328	0.123776091	0.23234	0.13974	Rgs4/Reln	2
GO:0060759	GO:0060759	regulation of response to cytokine stimulus	4/374	119/23328	0.124628661	0.23373	0.14057	Cd24a/Irf7	4
GO:2000278	GO:2000278	regulation of DNA biosynthetic process	4/374	119/23328	0.124628661	0.23373	0.14057	Dusp1/Cd	4
GO:0003407	GO:0003407	neural retina development	3/374	77/23328	0.12640505	0.23685	0.14245	Cfh/Thy1/	3
GO:0045453	GO:0045453	bone resorption	3/374	77/23328	0.12640505	0.23685	0.14245	Cd38/Ahs	3
GO:0006606	GO:0006606	protein import into nucleus	5/374	165/23328	0.126765115	0.23741	0.14279	Bmp4/Cd3	5
GO:0006090	GO:0006090	pyruvate metabolic process	4/374	120/23328	0.127401814	0.2385	0.14344	Nupr1/Cbf	4
GO:0021983	GO:0021983	pituitary gland development	2/374	39/23328	0.129147601	0.24079	0.14482	Bmp4/Hes	2
GO:0032781	GO:0032781	positive regulation of ATPase activity	2/374	39/23328	0.129147601	0.24079	0.14482	Atp1b1/M	2
GO:0042311	GO:0042311	vasodilation	2/374	39/23328	0.129147601	0.24079	0.14482	Ednrb/Apo	2
GO:0042554	GO:0042554	superoxide anion generation	2/374	39/23328	0.129147601	0.24079	0.14482	Cxcl1/Edr	2
GO:0050931	GO:0050931	pigment cell differentiation	2/374	39/23328	0.129147601	0.24079	0.14482	Ednrb/Kitl	2
GO:1900087	GO:1900087	positive regulation of G1/S transition of mitotic cell cyc	2/374	39/23328	0.129147601	0.24079	0.14482	Rgcc/Cyp	2
GO:1902108	GO:1902108	regulation of mitochondrial membrane permeability inv	2/374	39/23328	0.129147601	0.24079	0.14482	Chchd10/	2
GO:1905710	GO:1905710	positive regulation of membrane permeability	2/374	39/23328	0.129147601	0.24079	0.14482	Chchd10/	2
GO:2000403	GO:2000403	positive regulation of lymphocyte migration	2/374	39/23328	0.129147601	0.24079	0.14482	Cxcl12/Cc	2
GO:0014855	GO:0014855	striated muscle cell proliferation	3/374	78/23328	0.129992109	0.24194	0.14551	Ncam1/Pi	3
GO:0032088	GO:0032088	negative regulation of NF-kappaB transcription factor a	3/374	78/23328	0.129992109	0.24194	0.14551	Ptgis/Cd2	3
GO:0061180	GO:0061180	mammary gland epithelium development	3/374	78/23328	0.129992109	0.24194	0.14551	Gata3/Id2	3
GO:0070228	GO:0070228	regulation of lymphocyte apoptotic process	3/374	78/23328	0.129992109	0.24194	0.14551	Bmp4/Cd2	3
GO:0015837	GO:0015837	amine transport	4/374	121/23328	0.130200728	0.24211	0.14561	Cxcl12/Rg	4
GO:0042542	GO:0042542	response to hydrogen peroxide	4/374	121/23328	0.130200728	0.24211	0.14561	Mmp2/Aq	4

GO:0032984	GO:0032984	protein-containing complex disassembly	6/374	214/23328	0.130820959	0.24315	0.14624	Mical2/Ne	6
GO:0044770	GO:0044770	cell cycle phase transition	10/374	415/23328	0.13250775	0.24618	0.14806	Fhl1/Rgcc	10
GO:1901888	GO:1901888	regulation of cell junction assembly	6/374	215/23328	0.132886394	0.24677	0.14842	Kdr/Tek/C	6
GO:0031929	GO:0031929	TOR signaling	4/374	122/23328	0.133025043	0.24681	0.14844	Kdr/Gata3	4
GO:0090101	GO:0090101	negative regulation of transmembrane receptor protein	4/374	122/23328	0.133025043	0.24681	0.14844	Sfrp1/Htra	4
GO:0048663	GO:0048663	neuron fate commitment	3/374	79/23328	0.133614819	0.24746	0.14883	Bmp4/Not	3
GO:0051279	GO:0051279	regulation of release of sequestered calcium ion into c	3/374	79/23328	0.133614819	0.24746	0.14883	Aplnr/F2r/	3
GO:0051781	GO:0051781	positive regulation of cell division	3/374	79/23328	0.133614819	0.24746	0.14883	Pdgfa/Pdg	3
GO:0060191	GO:0060191	regulation of lipase activity	3/374	79/23328	0.133614819	0.24746	0.14883	Gpihbp1/F	3
GO:0048167	GO:0048167	regulation of synaptic plasticity	9/374	365/23328	0.134457875	0.24802	0.14917	Mgll/Kdr/A	9
GO:0000578	GO:0000578	embryonic axis specification	2/374	40/23328	0.134572409	0.24802	0.14917	Nrarp/Pcs	2
GO:0006730	GO:0006730	one-carbon metabolic process	2/374	40/23328	0.134572409	0.24802	0.14917	Car8/Car4	2
GO:0006984	GO:0006984	ER-nucleus signaling pathway	2/374	40/23328	0.134572409	0.24802	0.14917	Hspa5/Bo	2
GO:0009409	GO:0009409	response to cold	2/374	40/23328	0.134572409	0.24802	0.14917	Ucp2/Met	2
GO:0015804	GO:0015804	neutral amino acid transport	2/374	40/23328	0.134572409	0.24802	0.14917	Rgs4/Slc6	2
GO:0046006	GO:0046006	regulation of activated T cell proliferation	2/374	40/23328	0.134572409	0.24802	0.14917	Cd24a/Pr	2
GO:0051205	GO:0051205	protein insertion into membrane	2/374	40/23328	0.134572409	0.24802	0.14917	Mal/Rtp4	2
GO:0070884	GO:0070884	regulation of calcineurin-NFAT signaling cascade	2/374	40/23328	0.134572409	0.24802	0.14917	Lmcd1/Pr	2
GO:1901021	GO:1901021	positive regulation of calcium ion transmembrane trans	2/374	40/23328	0.134572409	0.24802	0.14917	Bmp4/Atp	2
GO:1902692	GO:1902692	regulation of neuroblast proliferation	2/374	40/23328	0.134572409	0.24802	0.14917	Cd24a/Ptr	2
GO:0046474	GO:0046474	glycerophospholipid biosynthetic process	4/374	123/23328	0.135874392	0.25031	0.15055	Pla2g5/Pc	4
GO:0051170	GO:0051170	import into nucleus	5/374	169/23328	0.136222985	0.25073	0.1508	Bmp4/Cd3	5
GO:0071897	GO:0071897	DNA biosynthetic process	5/374	169/23328	0.136222985	0.25073	0.1508	Lox/Dusp	5
GO:0008088	GO:0008088	axo-dendritic transport	3/374	80/23328	0.137272314	0.25211	0.15163	Fez1/Hspl	3
GO:0036465	GO:0036465	synaptic vesicle recycling	3/374	80/23328	0.137272314	0.25211	0.15163	Cd24a/Ac	3
GO:0038034	GO:0038034	signal transduction in absence of ligand	3/374	80/23328	0.137272314	0.25211	0.15163	Kitl/Bok/L	3
GO:0072078	GO:0072078	nephron tubule morphogenesis	3/374	80/23328	0.137272314	0.25211	0.15163	Bmp4/Gat	3
GO:0097192	GO:0097192	extrinsic apoptotic signaling pathway in absence of liga	3/374	80/23328	0.137272314	0.25211	0.15163	Kitl/Bok/L	3
GO:0002369	GO:0002369	T cell cytokine production	2/374	41/23328	0.140047437	0.25653	0.15428	Gata3/Rsa	2
GO:0030225	GO:0030225	macrophage differentiation	2/374	41/23328	0.140047437	0.25653	0.15428	Bmp4/Id2	2
GO:0046461	GO:0046461	neutral lipid catabolic process	2/374	41/23328	0.140047437	0.25653	0.15428	Mgll/Lpl	2
GO:0046464	GO:0046464	acylglycerol catabolic process	2/374	41/23328	0.140047437	0.25653	0.15428	Mgll/Lpl	2
GO:0106056	GO:0106056	regulation of calcineurin-mediated signaling	2/374	41/23328	0.140047437	0.25653	0.15428	Lmcd1/Pr	2
GO:1900744	GO:1900744	regulation of p38MAPK cascade	2/374	41/23328	0.140047437	0.25653	0.15428	Dusp1/Ha	2
GO:0045682	GO:0045682	regulation of epidermis development	3/374	81/23328	0.140963727	0.25772	0.155	Bmp4/Hes	3
GO:0051785	GO:0051785	positive regulation of nuclear division	3/374	81/23328	0.140963727	0.25772	0.155	Rgcc/Pdg	3
GO:1904589	GO:1904589	regulation of protein import	3/374	81/23328	0.140963727	0.25772	0.155	Bmp4/Cd3	3
GO:0019827	GO:0019827	stem cell population maintenance	5/374	171/23328	0.141066466	0.25772	0.155	Sfrp1/Pro	5
GO:0030902	GO:0030902	hindbrain development	5/374	171/23328	0.141066466	0.25772	0.155	Prox1/Itgb	5
GO:0045931	GO:0045931	positive regulation of mitotic cell cycle	5/374	171/23328	0.141066466	0.25772	0.155	Rgcc/Cyp	5
GO:0044242	GO:0044242	cellular lipid catabolic process	6/374	219/23328	0.141302048	0.25803	0.15519	Scarb1/M	6
GO:0008654	GO:0008654	phospholipid biosynthetic process	5/374	172/23328	0.143516152	0.26196	0.15755	Pla2g5/Pc	5
GO:0030048	GO:0030048	actin filament-based movement	4/374	126/23328	0.144568937	0.26257	0.15792	Gja5/Pdpr	4
GO:0051053	GO:0051053	negative regulation of DNA metabolic process	4/374	126/23328	0.144568937	0.26257	0.15792	Gata3/Du	4
GO:0120254	GO:0120254	olefinic compound metabolic process	4/374	126/23328	0.144568937	0.26257	0.15792	Mgll/Dkk3	4
GO:0060113	GO:0060113	inner ear receptor cell differentiation	3/374	82/23328	0.14468819	0.26257	0.15792	Bmp4/Cth	3
GO:0072088	GO:0072088	nephron epithelium morphogenesis	3/374	82/23328	0.14468819	0.26257	0.15792	Bmp4/Gat	3
GO:1901019	GO:1901019	regulation of calcium ion transmembrane transporter a	3/374	82/23328	0.14468819	0.26257	0.15792	Bmp4/Atp	3
GO:0009268	GO:0009268	response to pH	2/374	42/23328	0.145569692	0.26257	0.15792	Pam/Gja1	2
GO:0048873	GO:0048873	homeostasis of number of cells within a tissue	2/374	42/23328	0.145569692	0.26257	0.15792	Nos3/F2r	2
GO:0051154	GO:0051154	negative regulation of striated muscle cell differentiatio	2/374	42/23328	0.145569692	0.26257	0.15792	Rgs4/Pi16	2
GO:0072348	GO:0072348	sulfur compound transport	2/374	42/23328	0.145569692	0.26257	0.15792	Slc26a10/	2
GO:0098656	GO:0098656	anion transmembrane transport	6/374	221/23328	0.145600548	0.26257	0.15792	Thbs1/Rg	6
GO:1903050	GO:1903050	regulation of proteolysis involved in cellular protein cat	6/374	221/23328	0.145600548	0.26257	0.15792	Clu/Apoe/	6
GO:0060048	GO:0060048	cardiac muscle contraction	4/374	127/23328	0.147514697	0.26257	0.15792	Gja5/Atp1	4
GO:0003333	GO:0003333	amino acid transmembrane transport	3/374	83/23328	0.148444837	0.26257	0.15792	Rgs4/Itgb	3
GO:0021872	GO:0021872	forebrain generation of neurons	3/374	83/23328	0.148444837	0.26257	0.15792	Prox1/Nrp	3
GO:0031098	GO:0031098	stress-activated protein kinase signaling cascade	7/374	272/23328	0.148725714	0.26257	0.15792	Sfrp1/Sfrp	7
GO:0016311	GO:0016311	dephosphorylation	9/374	374/23328	0.148990836	0.26257	0.15792	Ephx2/Ptp	9
GO:0000050	GO:0000050	urea cycle	1/374	10/23328	0.149263526	0.26257	0.15792	Ass1	1
GO:0002934	GO:0002934	desmosome organization	1/374	10/23328	0.149263526	0.26257	0.15792	Perp	1
GO:0006527	GO:0006527	arginine catabolic process	1/374	10/23328	0.149263526	0.26257	0.15792	Nos3	1
GO:0006651	GO:0006651	diacylglycerol biosynthetic process	1/374	10/23328	0.149263526	0.26257	0.15792	Plpp1	1
GO:0009265	GO:0009265	2'-deoxyribonucleotide biosynthetic process	1/374	10/23328	0.149263526	0.26257	0.15792	Cmpk2	1
GO:0009650	GO:0009650	UV protection	1/374	10/23328	0.149263526	0.26257	0.15792	Mfap4	1
GO:0010745	GO:0010745	negative regulation of macrophage derived foam cell d	1/374	10/23328	0.149263526	0.26257	0.15792	Nfkbia	1
GO:0010940	GO:0010940	positive regulation of necrotic cell death	1/374	10/23328	0.149263526	0.26257	0.15792	Slc6a6	1
GO:0015865	GO:0015865	purine nucleotide transport	1/374	10/23328	0.149263526	0.26257	0.15792	Gja1	1
GO:0015911	GO:0015911	long-chain fatty acid import across plasma membrane	1/374	10/23328	0.149263526	0.26257	0.15792	Thbs1	1
GO:0021612	GO:0021612	facial nerve structural organization	1/374	10/23328	0.149263526	0.26257	0.15792	Nrp2	1
GO:0030952	GO:0030952	establishment or maintenance of cytoskeleton polarity	1/374	10/23328	0.149263526	0.26257	0.15792	Aqp1	1
GO:0031274	GO:0031274	positive regulation of pseudopodium assembly	1/374	10/23328	0.149263526	0.26257	0.15792	Cdc42ep3	1
GO:0031620	GO:0031620	regulation of fever generation	1/374	10/23328	0.149263526	0.26257	0.15792	Ednrb	1
GO:0032351	GO:0032351	negative regulation of hormone metabolic process	1/374	10/23328	0.149263526	0.26257	0.15792	Dkk3	1
GO:0032836	GO:0032836	glomerular basement membrane development	1/374	10/23328	0.149263526	0.26257	0.15792	Nid1	1
GO:0033085	GO:0033085	negative regulation of T cell differentiation in thymus	1/374	10/23328	0.149263526	0.26257	0.15792	Bmp4	1
GO:0033572	GO:0033572	transferrin transport	1/374	10/23328	0.149263526	0.26257	0.15792	Myo1b	1
GO:0033689	GO:0033689	negative regulation of osteoblast proliferation	1/374	10/23328	0.149263526	0.26257	0.15792	Sfrp1	1
GO:0034145	GO:0034145	positive regulation of toll-like receptor 4 signaling pathw	1/374	10/23328	0.149263526	0.26257	0.15792	Lbp	1
GO:0034380	GO:0034380	high-density lipoprotein particle assembly	1/374	10/23328	0.149263526	0.26257	0.15792	Apoe	1
GO:0039532	GO:0039532	negative regulation of viral-induced cytoplasmic patter	1/374	10/23328	0.149263526	0.26257	0.15792	Tspan6	1
GO:0045607	GO:0045607	regulation of inner ear auditory receptor cell differentia	1/374	10/23328	0.149263526	0.26257	0.15792	Hes1	1
GO:0045631	GO:0045631	regulation of mechanoreceptor differentiation	1/374	10/23328	0.149263526	0.26257	0.15792	Hes1	1
GO:0045654	GO:0045654	positive regulation of megakaryocyte differentiation	1/374	10/23328	0.149263526	0.26257	0.15792	Hmgb2	1
GO:0045869	GO:0045869	negative regulation of single stranded viral RNA replic	1/374	10/23328	0.149263526	0.26257	0.15792	Apobec3	1
GO:0046385	GO:0046385	deoxyribose phosphate biosynthetic process	1/374	10/23328	0.149263526	0.26257	0.15792	Cmpk2	1
GO:0046598	GO:0046598	positive regulation of viral entry into host cell	1/374	10/23328	0.149263526	0.26257	0.15792	Cd74	1
GO:0048739	GO:0048739	cardiac muscle fiber development	1/374	10/23328	0.149263526	0.26257	0.15792	Bmp4	1
GO:0051006	GO:0051006	positive regulation of lipoprotein lipase activity	1/374	10/23328	0.149263526	0.26257	0.15792	Gpihbp1	1
GO:0051917	GO:0051917	regulation of fibrinolysis	1/374	10/23328	0.149263526	0.26257	0.15792	Thbs1	1
GO:0060513	GO:0060513	prostatic bud formation	1/374	10/23328	0.149263526	0.26257	0.15792	Bmp4	1
GO:0060710	GO:0060710	chorio-allantoic fusion	1/374	10/23328	0.149263526	0.26257	0.15792	Vcam1	1
GO:0060982	GO:0060982	coronary artery morphogenesis	1/374	10/23328	0.149263526	0.26257	0.15792	Hand2	1
GO:0061517	GO:0061517	macrophage proliferation	1/374	10/23328	0.149263526	0.26257	0.15792	Clu	1
GO:0070244	GO:0070244	negative regulation of thymocyte apoptotic process	1/374	10/23328	0.149263526	0.26257	0.15792	Bmp4	1
GO:0070673	GO:0070673	response to interleukin-18	1/374	10/23328	0.149263526	0.26257	0.15792	Pdgfb	1
GO:0070857	GO:0070857	regulation of bile acid biosynthetic process	1/374	10/23328	0.149263526	0.26257	0.15792	Prox1	1
GO:0071257	GO:0071257	cellular response to electrical stimulus	1/374	10/23328	0.149263526	0.26257	0.15792	Actb	1
GO:0071305	GO:0071305	cellular response to vitamin D	1/374	10/23328	0.149263526	0.26257	0.15792	Pim1	1
GO:0071635	GO:0071635	negative regulation of transforming growth factor beta	1/374	10/23328	0.149263526	0.26257	0.15792	Cd24a	1
GO:0072070	GO:0072070	loop of Henle development	1/374	10/23328	0.149263526	0.26257	0.15792	Aqp1	1
GO:0072173	GO:0072173	metanephric tubule morphogenesis	1/374	10/23328	0.149263526	0.26257	0.15792	Hes1	1
GO:0072203	GO:0072203	cell proliferation involved in metanephros developmen	1/374	10/23328	0.149263526	0.26257	0.15792	Pdgfb	1
GO:0075294	GO:0075294	positive regulation by symbiont of entry into host	1/374	10/23328	0.149263526	0.26257	0.15792	Cd74	1
GO:0090129	GO:0090129	positive regulation of synapse maturation	1/374	10/23328	0.149263526	0.26257	0.15792	Reln	1
GO:0090269	GO:0090269	fibroblast growth factor production	1/374	10/23328	0.149263526	0.26257	0.15792	Rgcc	1
GO:0090270	GO:0090270	regulation of fibroblast growth factor production	1/374	10/23328	0.149263526	0.26257	0.15792	Rgcc	1
GO:0090385	GO:0090385	phagosome-lysosome fusion	1/374	10/23328	0.149263526	0.26257	0.15792	Pla2g5	1
GO:0090713	GO:0090713	immunological memory process	1/374	10/23328	0.149263526	0.26257	0.15792	Fgl2	1
GO:0097067	GO:0097067	cellular response to thyroid hormone stimulus	1/374	10/23328	0.149263526	0.26257	0.15792	Lmo2	1
GO:0097119	GO:0097119	postsynaptic density protein 95 clustering	1/374	10/23328	0.149263526	0.26257	0.15792	Reln	1
GO:0097384	GO:0097384	cellular lipid biosynthetic process	1/374	10/23328	0.149263526	0.26257	0.15792	Pla2g5	1
GO:0140052	GO:0140052	cellular response to oxidised low-density lipoprotein pa	1/374	10/23328	0.149263526	0.26257	0.15792	Cd36	1
GO:1900038	GO:1900038	negative regulation of cellular response to hypoxia	1/374	10/23328	0.149263526	0.26257	0.15792	Cd34	1
GO:1900103	GO:1900103	positive regulation of endoplasmic reticulum unfolded p	1/374	10/23328	0.149263526	0.26257	0.15792	Bok	1
GO:1901503	GO:1901503	ether biosynthetic process	1/374	10/23328	0.149263526	0.26257	0.15792	Pla2g5	1
GO:1901724	GO:1901724	positive regulation of cell proliferation involved in kidne	1/374	10/23328	0.149263526	0.26257	0.15792	Pdgfb	1
GO:1902746	GO:1902746	regulation of lens fiber cell differentiation	1/374	10/23328	0.149263526	0.26257	0.15792	Cdkn1c	1
GO:1903238	GO:1903238	positive regulation of leukocyte tethering or rolling	1/374	10/23328	0.149263526	0.26257	0.15792	Selp	1
GO:1905456	GO:1905456	regulation of lymphoid progenitor cell differentiation	1/374	10/23328	0.149263526	0.26257	0.15792	Hes1	1
GO:1905522	GO:1905522	negative regulation of macrophage migration	1/374	10/23328	0.149263526	0.26257	0.15792	Cd200	1
GO:1990264	GO:1990264	peptidyl-tyrosine dephosphorylation involved in inactiva	1/374	10/23328	0.149263526	0.26257	0.15792	Ptprb	1
GO:1990535	GO:1990535	neuron projection maintenance	1/374	10/23328	0.149263526	0.26257	0.15792	Prnp	1
GO:2000192	GO:2000192	negative regulation of fatty acid transport	1/374	10/23328	0.149263526	0.26257	0.15792	Thbs1	1
GO:2000807	GO:2000807	regulation of synaptic vesicle clustering	1/374	10/23328	0.149263526	0.26257	0.15792	Pcdh17	1
GO:2000980	GO:2000980	regulation of inner ear receptor cell differentiation	1/374	10/23328	0.149263526	0.26257	0.15792	Hes1	1
GO:2001138	GO:2001138	regulation of phospholipid transport	1/374	10/23328	0.149263526	0.26257	0.15792	Apoe	1
GO:2001140	GO:2001140	positive regulation of phospholipid transport	1/374	10/23328	0.149263526	0.26257	0.15792	Apoe	1
GO:0002696	GO:0002696	positive regulation of leukocyte activation	11/374	479/23328	0.149698522	0.26322	0.15831	Vcam1/Ga	11

GO:0032609	GO:0032609	interferon-gamma production	4/374	128/23328	0.150483612	0.26438	0.159	Gata3/Cd	4
GO:0061387	GO:0061387	regulation of extent of cell growth	4/374	128/23328	0.150483612	0.26438	0.159	Mgll/Apoe	4
GO:0042129	GO:0042129	regulation of T cell proliferation	5/374	175/23328	0.15097454	0.26441	0.15902	Vcam1/Br	5
GO:0098727	GO:0098727	maintenance of cell number	5/374	175/23328	0.15097454	0.26441	0.15902	Sfrp1/Pro	5
GO:0001783	GO:0001783	B cell apoptotic process	2/374	43/23328	0.151136266	0.26441	0.15902	Cd24a/Cd	2
GO:0002753	GO:0002753	cytoplasmic pattern recognition receptor signaling path	2/374	43/23328	0.151136266	0.26441	0.15902	Nfkbia/Ts	2
GO:0014003	GO:0014003	oligodendrocyte development	2/374	43/23328	0.151136266	0.26441	0.15902	Clu/Id2	2
GO:0032008	GO:0032008	positive regulation of TOR signaling	2/374	43/23328	0.151136266	0.26441	0.15902	Kdr/Reln	2
GO:0038083	GO:0038083	peptidyl-tyrosine autophosphorylation	2/374	43/23328	0.151136266	0.26441	0.15902	Bmx/Kdr	2
GO:0042304	GO:0042304	regulation of fatty acid biosynthetic process	2/374	43/23328	0.151136266	0.26441	0.15902	Fabp5/Cd	2
GO:0046782	GO:0046782	regulation of viral transcription	2/374	43/23328	0.151136266	0.26441	0.15902	Zfp36/Jun	2
GO:0060122	GO:0060122	inner ear receptor cell stereocilium organization	2/374	43/23328	0.151136266	0.26441	0.15902	Cthrc1/He	2
GO:0072028	GO:0072028	nephron morphogenesis	3/374	84/23328	0.152232803	0.26622	0.16011	Bmp4/Gat	3
GO:0032273	GO:0032273	positive regulation of protein polymerization	4/374	129/23328	0.153475295	0.26816	0.16128	Cdc42ep3	4
GO:0046434	GO:0046434	organophosphate catabolic process	4/374	129/23328	0.153475295	0.26816	0.16128	Scarb1/Pl	4
GO:0060401	GO:0060401	cytosolic calcium ion transport	5/374	177/23328	0.156035813	0.27244	0.16385	Bmp4/Apl	5
GO:0048524	GO:0048524	positive regulation of viral process	3/374	85/23328	0.156051224	0.27244	0.16385	Apoe/Cd7	3
GO:0010972	GO:0010972	negative regulation of G2/M transition of mitotic cell cy	2/374	44/23328	0.156744328	0.27296	0.16417	Fhl1/Cdkn	2
GO:0032350	GO:0032350	regulation of hormone metabolic process	2/374	44/23328	0.156744328	0.27296	0.16417	Dkk3/Gata	2
GO:0032768	GO:0032768	regulation of monooxygenase activity	2/374	44/23328	0.156744328	0.27296	0.16417	Scarb1/Ap	2
GO:0033628	GO:0033628	regulation of cell adhesion mediated by integrin	2/374	44/23328	0.156744328	0.27296	0.16417	Cd24a/Ac	2
GO:0048821	GO:0048821	erythrocyte development	2/374	44/23328	0.156744328	0.27296	0.16417	Adgrf5/Klf	2
GO:0050919	GO:0050919	negative chemotaxis	2/374	44/23328	0.156744328	0.27296	0.16417	Nrp2/Pdgf	2
GO:0042594	GO:0042594	response to starvation	5/374	178/23328	0.158592513	0.27606	0.16603	Fstl1/Cdkn	5
GO:0006665	GO:0006665	sphingolipid metabolic process	4/374	131/23328	0.15952541	0.27608	0.16605	Gm2a/Plp	4
GO:0032434	GO:0032434	regulation of proteasomal ubiquitin-dependent protein	4/374	131/23328	0.15952541	0.27608	0.16605	Clu/Cbfa2	4
GO:0061333	GO:0061333	renal tubule morphogenesis	3/374	86/23328	0.159899237	0.27608	0.16605	Bmp4/Gat	3
GO:1905897	GO:1905897	regulation of response to endoplasmic reticulum stress	3/374	86/23328	0.159899237	0.27608	0.16605	Clu/Nupr1	3
GO:0001961	GO:0001961	positive regulation of cytokine-mediated signaling path	2/374	45/23328	0.162391129	0.27608	0.16605	Irf7/Cd74	2
GO:0009166	GO:0009166	nucleotide catabolic process	2/374	45/23328	0.162391129	0.27608	0.16605	Nudt4/Pde	2
GO:0021696	GO:0021696	cerebellar cortex morphogenesis	2/374	45/23328	0.162391129	0.27608	0.16605	Prox1/Ser	2
GO:0034340	GO:0034340	response to type I interferon	2/374	45/23328	0.162391129	0.27608	0.16605	Irf7/Isg15	2
GO:0044088	GO:0044088	regulation of vacuole organization	2/374	45/23328	0.162391129	0.27608	0.16605	Fez1/Nupr	2
GO:0051293	GO:0051293	establishment of spindle localization	2/374	45/23328	0.162391129	0.27608	0.16605	Itgb1/Gja1	2
GO:1901031	GO:1901031	regulation of response to reactive oxygen species	2/374	45/23328	0.162391129	0.27608	0.16605	Fbln5/Cd3	2
GO:0006790	GO:0006790	sulfur compound metabolic process	7/374	279/23328	0.162747198	0.27608	0.16605	Bgn/Mical	7
GO:0002579	GO:0002579	positive regulation of antigen processing and presenta	1/374	11/23328	0.162908583	0.27608	0.16605	Cd74	1
GO:0002679	GO:0002679	respiratory burst involved in defense response	1/374	11/23328	0.162908583	0.27608	0.16605	Lbp	1
GO:0002921	GO:0002921	negative regulation of humoral immune response	1/374	11/23328	0.162908583	0.27608	0.16605	Serping1	1
GO:0003139	GO:0003139	secondary heart field specification	1/374	11/23328	0.162908583	0.27608	0.16605	Bmp4	1
GO:0009219	GO:0009219	pyrimidine deoxyribonucleotide metabolic process	1/374	11/23328	0.162908583	0.27608	0.16605	Cmpk2	1
GO:0009812	GO:0009812	flavonoid metabolic process	1/374	11/23328	0.162908583	0.27608	0.16605	Cyp1a1	1
GO:0019627	GO:0019627	urea metabolic process	1/374	11/23328	0.162908583	0.27608	0.16605	Ass1	1
GO:0021684	GO:0021684	cerebellar granular layer formation	1/374	11/23328	0.162908583	0.27608	0.16605	Prox1	1
GO:0021707	GO:0021707	cerebellar granule cell differentiation	1/374	11/23328	0.162908583	0.27608	0.16605	Prox1	1
GO:0031272	GO:0031272	regulation of pseudopodium assembly	1/374	11/23328	0.162908583	0.27608	0.16605	Cdc42ep3	1
GO:0031642	GO:0031642	negative regulation of myelination	1/374	11/23328	0.162908583	0.27608	0.16605	Klk8	1
GO:0031943	GO:0031943	regulation of glucocorticoid metabolic process	1/374	11/23328	0.162908583	0.27608	0.16605	Dkk3	1
GO:0032342	GO:0032342	aldosterone biosynthetic process	1/374	11/23328	0.162908583	0.27608	0.16605	Dkk3	1
GO:0033084	GO:0033084	regulation of immature T cell proliferation in thymus	1/374	11/23328	0.162908583	0.27608	0.16605	Bmp4	1
GO:0033327	GO:0033327	Leydig cell differentiation	1/374	11/23328	0.162908583	0.27608	0.16605	Dhh	1
GO:0033700	GO:0033700	phospholipid efflux	1/374	11/23328	0.162908583	0.27608	0.16605	Apoe	1
GO:0034650	GO:0034650	cortisol metabolic process	1/374	11/23328	0.162908583	0.27608	0.16605	Dkk3	1
GO:0036005	GO:0036005	response to macrophage colony-stimulating factor	1/374	11/23328	0.162908583	0.27608	0.16605	Pde2a	1
GO:0036006	GO:0036006	cellular response to macrophage colony-stimulating fa	1/374	11/23328	0.162908583	0.27608	0.16605	Pde2a	1
GO:0036499	GO:0036499	PERK-mediated unfolded protein response	1/374	11/23328	0.162908583	0.27608	0.16605	Bok	1
GO:0045602	GO:0045602	negative regulation of endothelial cell differentiation	1/374	11/23328	0.162908583	0.27608	0.16605	Id1	1
GO:0048149	GO:0048149	behavioral response to ethanol	1/374	11/23328	0.162908583	0.27608	0.16605	Adh1	1
GO:0048845	GO:0048845	venous blood vessel morphogenesis	1/374	11/23328	0.162908583	0.27608	0.16605	Prox1	1
GO:0051458	GO:0051458	corticotropin secretion	1/374	11/23328	0.162908583	0.27608	0.16605	Aqp1	1
GO:0051561	GO:0051561	positive regulation of mitochondrial calcium ion concer	1/374	11/23328	0.162908583	0.27608	0.16605	Tgm2	1
GO:0051601	GO:0051601	exocyst localization	1/374	11/23328	0.162908583	0.27608	0.16605	Tnfaip2	1
GO:0055015	GO:0055015	ventricular cardiac muscle cell development	1/374	11/23328	0.162908583	0.27608	0.16605	Prox1	1
GO:0060056	GO:0060056	mammary gland involution	1/374	11/23328	0.162908583	0.27608	0.16605	Igfbp5	1
GO:0060174	GO:0060174	limb bud formation	1/374	11/23328	0.162908583	0.27608	0.16605	Sox4	1
GO:0060405	GO:0060405	regulation of penile erection	1/374	11/23328	0.162908583	0.27608	0.16605	Ednrb	1
GO:0060693	GO:0060693	regulation of branching involved in salivary gland morp	1/374	11/23328	0.162908583	0.27608	0.16605	Pdgfa	1
GO:0060900	GO:0060900	embryonic camera-type eye formation	1/374	11/23328	0.162908583	0.27608	0.16605	Prox1	1
GO:0061299	GO:0061299	retina vasculature morphogenesis in camera-type eye	1/374	11/23328	0.162908583	0.27608	0.16605	Arhgef15	1
GO:0061365	GO:0061365	positive regulation of triglyceride lipase activity	1/374	11/23328	0.162908583	0.27608	0.16605	Gpihbp1	1
GO:0070493	GO:0070493	thrombin-activated receptor signaling pathway	1/374	11/23328	0.162908583	0.27608	0.16605	F2r	1
GO:0072172	GO:0072172	mesonephric tubule formation	1/374	11/23328	0.162908583	0.27608	0.16605	Gata3	1
GO:0072182	GO:0072182	regulation of nephron tubule epithelial cell differentiatio	1/374	11/23328	0.162908583	0.27608	0.16605	Gata3	1
GO:0086016	GO:0086016	AV node cell action potential	1/374	11/23328	0.162908583	0.27608	0.16605	Gja5	1
GO:0086027	GO:0086027	AV node cell to bundle of His cell signaling	1/374	11/23328	0.162908583	0.27608	0.16605	Gja5	1
GO:0090037	GO:0090037	positive regulation of protein kinase C signaling	1/374	11/23328	0.162908583	0.27608	0.16605	Flt4	1
GO:0090281	GO:0090281	negative regulation of calcium ion import	1/374	11/23328	0.162908583	0.27608	0.16605	Hes1	1
GO:0099612	GO:0099612	protein localization to axon	1/374	11/23328	0.162908583	0.27608	0.16605	Mal	1
GO:0140467	GO:0140467	integrated stress response signaling	1/374	11/23328	0.162908583	0.27608	0.16605	Bok	1
GO:1901142	GO:1901142	insulin metabolic process	1/374	11/23328	0.162908583	0.27608	0.16605	Cpe	1
GO:1901339	GO:1901339	regulation of store-operated calcium channel activity	1/374	11/23328	0.162908583	0.27608	0.16605	Bmp4	1
GO:1902166	GO:1902166	negative regulation of intrinsic apoptotic signaling path	1/374	11/23328	0.162908583	0.27608	0.16605	Cd74	1
GO:1902548	GO:1902548	negative regulation of cellular response to vascular en	1/374	11/23328	0.162908583	0.27608	0.16605	Dcn	1
GO:1903867	GO:1903867	extraembryonic membrane development	1/374	11/23328	0.162908583	0.27608	0.16605	Htra1	1
GO:1903911	GO:1903911	positive regulation of receptor clustering	1/374	11/23328	0.162908583	0.27608	0.16605	Ptn	1
GO:1905288	GO:1905288	vascular associated smooth muscle cell apoptotic proc	1/374	11/23328	0.162908583	0.27608	0.16605	Gapdh	1
GO:1905459	GO:1905459	regulation of vascular associated smooth muscle cell a	1/374	11/23328	0.162908583	0.27608	0.16605	Gapdh	1
GO:2000644	GO:2000644	regulation of receptor catabolic process	1/374	11/23328	0.162908583	0.27608	0.16605	Apoe	1
GO:2001280	GO:2001280	positive regulation of unsaturated fatty acid biosyntheti	1/374	11/23328	0.162908583	0.27608	0.16605	Cd74	1
GO:0010827	GO:0010827	regulation of glucose transmembrane transport	3/374	88/23328	0.167680606	0.28382	0.1707	Fabp5/C3	3
GO:0034620	GO:0034620	cellular response to unfolded protein	3/374	88/23328	0.167680606	0.28382	0.1707	Hspa5/Hs	3
GO:0050672	GO:0050672	negative regulation of lymphocyte proliferation	3/374	88/23328	0.167680606	0.28382	0.1707	Bmp4/Cd	3
GO:0010259	GO:0010259	multicellular organism aging	2/374	46/23328	0.168073995	0.28391	0.17076	Cfh/Meg3	2
GO:0010518	GO:0010518	positive regulation of phospholipase activity	2/374	46/23328	0.168073995	0.28391	0.17076	Flt1/Pla2g	2
GO:0019083	GO:0019083	viral transcription	2/374	46/23328	0.168073995	0.28391	0.17076	Zfp36/Jun	2
GO:1901985	GO:1901985	positive regulation of protein acetylation	2/374	46/23328	0.168073995	0.28391	0.17076	Gata3/Sox	2
GO:1905521	GO:1905521	regulation of macrophage migration	2/374	46/23328	0.168073995	0.28391	0.17076	Thbs1/Cd	2
GO:0002698	GO:0002698	negative regulation of immune effector process	4/374	134/23328	0.168761564	0.28496	0.17138	Htra1/Fgl	4
GO:0048813	GO:0048813	dendrite morphogenesis	5/374	182/23328	0.168987951	0.28523	0.17154	Itgb1/Id1/	5
GO:0032872	GO:0032872	regulation of stress-activated MAPK cascade	6/374	232/23328	0.17027075	0.28728	0.17278	Sfrp1/Sfrp	6
GO:0032945	GO:0032945	negative regulation of mononuclear cell proliferation	3/374	89/23328	0.171612251	0.28919	0.17393	Bmp4/Cd	3
GO:0042490	GO:0042490	mechanoreceptor differentiation	3/374	89/23328	0.171612251	0.28919	0.17393	Bmp4/Cth	3
GO:0070301	GO:0070301	cellular response to hydrogen peroxide	3/374	89/23328	0.171612251	0.28919	0.17393	Aqp1/Net1	3
GO:0043467	GO:0043467	regulation of generation of precursor metabolites and e	4/374	135/23328	0.171881623	0.28953	0.17413	Nupr1/Cbf	4
GO:0045332	GO:0045332	phospholipid translocation	2/374	47/23328	0.173790328	0.28977	0.17428	Kcnn4/Ab	2
GO:0048246	GO:0048246	macrophage chemotaxis	2/374	47/23328	0.173790328	0.28977	0.17428	Ednrb/Thb	2
GO:0050974	GO:0050974	detection of mechanical stimulus involved in sensory p	2/374	47/23328	0.173790328	0.28977	0.17428	Cxcl12/Se	2
GO:0060964	GO:0060964	regulation of gene silencing by miRNA	2/374	47/23328	0.173790328	0.28977	0.17428	Bmp4/Zfp	2
GO:0030534	GO:0030534	adult behavior	5/374	184/23328	0.174283719	0.28977	0.17428	Pcdh17/A	5
GO:0097553	GO:0097553	calcium ion transmembrane import into cytosol	4/374	136/23328	0.175021688	0.28977	0.17428	Aplnr/F2r/	4
GO:0009593	GO:0009593	detection of chemical stimulus	3/374	90/23328	0.175570069	0.28977	0.17428	Scarb1/Lb	3
GO:0032024	GO:0032024	positive regulation of insulin secretion	3/374	90/23328	0.175570069	0.28977	0.17428	Cd38/Sox	3
GO:0002329	GO:0002329	pre-B cell differentiation	1/374	12/23328	0.176335361	0.28977	0.17428	Cd24a	1
GO:0003129	GO:0003129	heart induction	1/374	12/23328	0.176335361	0.28977	0.17428	Bmp4	1
GO:0003183	GO:0003183	mitral valve morphogenesis	1/374	12/23328	0.176335361	0.28977	0.17428	Sox4	1
GO:0003337	GO:0003337	mesenchymal to epithelial transition involved in metan	1/374	12/23328	0.176335361	0.28977	0.17428	Bmp4	1
GO:0006123	GO:0006123	mitochondrial electron transport, cytochrome c to oxyg	1/374	12/23328	0.176335361	0.28977	0.17428	Cox4i2	1
GO:0006705	GO:0006705	mineralocorticoid biosynthetic process	1/374	12/23328	0.176335361	0.28977	0.17428	Dkk3	1
GO:0007501	GO:0007501	mesodermal cell fate specification	1/374	12/23328	0.176335361	0.28977	0.17428	Sfrp2	1
GO:0010870	GO:0010870	positive regulation of receptor biosynthetic process	1/374	12/23328	0.176335361	0.28977	0.17428	Edn1	1
GO:0010872	GO:0010872	regulation of cholesterol esterification	1/374	12/23328	0.176335361	0.28977	0.17428	Apoe	1
GO:0016264	GO:0016264	gap junction assembly	1/374	12/23328	0.176335361	0.28977	0.17428	Gja5	1
GO:0019646	GO:0019646	aerobic electron transport chain	1/374	12/23328	0.176335361	0.28977	0.17428	Cox4i2	1
GO:0021561	GO:0021561	facial nerve development	1/374	12/23328	0.176335361	0.28977	0.17428	Nrp2	1
GO:0021610	GO:0021610	facial nerve morphogenesis	1/374	12/23328	0.176335361	0.28977	0.17428	Nrp2	1
GO:0032341	GO:0032341	aldosterone metabolic process	1/374	12/23328	0.176335361	0.28977	0.17428	Dkk3	1
GO:0032754	GO:0032754	positive regulation of interleukin-5 production	1/374	12/23328	0.176335361	0.28977	0.17428	Gata3	1
GO:0033080	GO:0033080	immature T cell proliferation in thymus	1/374	12/23328	0.176335361	0.28977	0.17428	Bmp4	1
GO:0033240	GO:0033240	positive regulation of cellular amine metabolic process	1/374	12/23328	0.176335361	0.28977	0.17428	Epas1	1

GO:0033275	GO:0033275	actin-myosin filament sliding	1/374	12/23328	0.176335361	0.28977	0.17428	Actc1	1
GO:0033603	GO:0033603	positive regulation of dopamine secretion	1/374	12/23328	0.176335361	0.28977	0.17428	Cxcl12	1
GO:0033632	GO:0033632	regulation of cell-cell adhesion mediated by integrin	1/374	12/23328	0.176335361	0.28977	0.17428	Cd24a	1
GO:0035457	GO:0035457	cellular response to interferon-alpha	1/374	12/23328	0.176335361	0.28977	0.17428	Ifit1	1
GO:0042447	GO:0042447	hormone catabolic process	1/374	12/23328	0.176335361	0.28977	0.17428	Ece1	1
GO:0042989	GO:0042989	sequestering of actin monomers	1/374	12/23328	0.176335361	0.28977	0.17428	Tmsb4x	1
GO:0043922	GO:0043922	negative regulation by host of viral transcription	1/374	12/23328	0.176335361	0.28977	0.17428	Jun	1
GO:0044766	GO:0044766	multi-organism transport	1/374	12/23328	0.176335361	0.28977	0.17428	Bst2	1
GO:0045091	GO:0045091	regulation of single stranded viral RNA replication via (	1/374	12/23328	0.176335361	0.28977	0.17428	Apobec3	1
GO:0045586	GO:0045586	regulation of gamma-delta T cell differentiation	1/374	12/23328	0.176335361	0.28977	0.17428	Sox4	1
GO:0046462	GO:0046462	monoacylglycerol metabolic process	1/374	12/23328	0.176335361	0.28977	0.17428	Mgll	1
GO:0046643	GO:0046643	regulation of gamma-delta T cell activation	1/374	12/23328	0.176335361	0.28977	0.17428	Sox4	1
GO:0046794	GO:0046794	transport of virus	1/374	12/23328	0.176335361	0.28977	0.17428	Bst2	1
GO:0060159	GO:0060159	regulation of dopamine receptor signaling pathway	1/374	12/23328	0.176335361	0.28977	0.17428	Rgs4	1
GO:0060433	GO:0060433	bronchus development	1/374	12/23328	0.176335361	0.28977	0.17428	Bmp4	1
GO:0060439	GO:0060439	trachea morphogenesis	1/374	12/23328	0.176335361	0.28977	0.17428	Bmp4	1
GO:0060502	GO:0060502	epithelial cell proliferation involved in lung morphogene	1/374	12/23328	0.176335361	0.28977	0.17428	Bmp4	1
GO:0060525	GO:0060525	prostate glandular acinus development	1/374	12/23328	0.176335361	0.28977	0.17428	Sfrp1	1
GO:0060536	GO:0060536	cartilage morphogenesis	1/374	12/23328	0.176335361	0.28977	0.17428	Hand2	1
GO:0061085	GO:0061085	regulation of histone H3-K27 methylation	1/374	12/23328	0.176335361	0.28977	0.17428	Gata3	1
GO:0070278	GO:0070278	extracellular matrix constituent secretion	1/374	12/23328	0.176335361	0.28977	0.17428	Rgcc	1
GO:0070943	GO:0070943	neutrophil-mediated killing of symbiont cell	1/374	12/23328	0.176335361	0.28977	0.17428	Cxcl1	1
GO:0071236	GO:0071236	cellular response to antibiotic	1/374	12/23328	0.176335361	0.28977	0.17428	Crip1	1
GO:0071318	GO:0071318	cellular response to ATP	1/374	12/23328	0.176335361	0.28977	0.17428	Hsp90b1	1
GO:0071941	GO:0071941	nitrogen cycle metabolic process	1/374	12/23328	0.176335361	0.28977	0.17428	Ass1	1
GO:0072044	GO:0072044	collecting duct development	1/374	12/23328	0.176335361	0.28977	0.17428	Bmp4	1
GO:0090209	GO:0090209	negative regulation of triglyceride metabolic process	1/374	12/23328	0.176335361	0.28977	0.17428	Apoe	1
GO:0090557	GO:0090557	establishment of endothelial intestinal barrier	1/374	12/23328	0.176335361	0.28977	0.17428	F11r	1
GO:0097113	GO:0097113	AMPA glutamate receptor clustering	1/374	12/23328	0.176335361	0.28977	0.17428	Apoe	1
GO:0097709	GO:0097709	connective tissue replacement	1/374	12/23328	0.176335361	0.28977	0.17428	F2r	1
GO:0150079	GO:0150079	negative regulation of neuroinflammatory response	1/374	12/23328	0.176335361	0.28977	0.17428	Cd200	1
GO:1902510	GO:1902510	regulation of apoptotic DNA fragmentation	1/374	12/23328	0.176335361	0.28977	0.17428	Igfbp3	1
GO:1902513	GO:1902513	regulation of organelle transport along microtubule	1/374	12/23328	0.176335361	0.28977	0.17428	Fez1	1
GO:1902579	GO:1902579	multi-organism localization	1/374	12/23328	0.176335361	0.28977	0.17428	Bst2	1
GO:1903800	GO:1903800	positive regulation of production of miRNAs involved ir	1/374	12/23328	0.176335361	0.28977	0.17428	Bmp4	1
GO:2000402	GO:2000402	negative regulation of lymphocyte migration	1/374	12/23328	0.176335361	0.28977	0.17428	Cd200	1
GO:2000615	GO:2000615	regulation of histone H3-K9 acetylation	1/374	12/23328	0.176335361	0.28977	0.17428	Gata3	1
GO:0071229	GO:0071229	cellular response to acid chemical	5/374	185/23328	0.17695532	0.29067	0.17482	Col5a2/Mı	5
GO:0006470	GO:0006470	protein dephosphorylation	6/374	235/23328	0.177285668	0.29087	0.17494	Ptpn18/Pt	6
GO:0032868	GO:0032868	response to insulin	6/374	235/23328	0.177285668	0.29087	0.17494	C1qtnf9/L	6
GO:0070302	GO:0070302	regulation of stress-activated protein kinase signaling (	6/374	235/23328	0.177285668	0.29087	0.17494	Sfrp1/Sfrp	6
GO:0000266	GO:0000266	mitochondrial fission	2/374	48/23328	0.179537605	0.29378	0.17669	Kdr/Dcn	2
GO:0001774	GO:0001774	microglial cell activation	2/374	48/23328	0.179537605	0.29378	0.17669	Clu/Jun	2
GO:0002269	GO:0002269	leukocyte activation involved in inflammatory response	2/374	48/23328	0.179537605	0.29378	0.17669	Clu/Jun	2
GO:0050798	GO:0050798	activated T cell proliferation	2/374	48/23328	0.179537605	0.29378	0.17669	Cd24a/Prı	2
GO:1903115	GO:1903115	regulation of actin filament-based movement	2/374	48/23328	0.179537605	0.29378	0.17669	Gja5/Pdpı	2
GO:1990573	GO:1990573	potassium ion import across plasma membrane	2/374	48/23328	0.179537605	0.29378	0.17669	Abcc9/Atp	2
GO:1900407	GO:1900407	regulation of cellular response to oxidative stress	3/374	91/23328	0.179553214	0.29378	0.17669	Fbln5/Cd3	3
GO:0009411	GO:0009411	response to UV	4/374	138/23328	0.18136022	0.29651	0.17833	Cdkn1a/A	4
GO:0042471	GO:0042471	ear morphogenesis	4/374	138/23328	0.18136022	0.29651	0.17833	Gata3/Cth	4
GO:0055072	GO:0055072	iron ion homeostasis	3/374	92/23328	0.183560844	0.29999	0.18042	Steap4/Ep	3
GO:0010565	GO:0010565	regulation of cellular ketone metabolic process	4/374	139/23328	0.184557882	0.30081	0.18092	Dkk3/Prox	4
GO:0031669	GO:0031669	cellular response to nutrient levels	5/374	188/23328	0.185062203	0.30081	0.18092	Lpl/Pim1/(	5
GO:0021532	GO:0021532	neural tube patterning	2/374	49/23328	0.185313371	0.30081	0.18092	Bmp4/Hes	2
GO:0033173	GO:0033173	calcineurin-NFAT signaling cascade	2/374	49/23328	0.185313371	0.30081	0.18092	Lmcd1/Prı	2
GO:0034204	GO:0034204	lipid translocation	2/374	49/23328	0.185313371	0.30081	0.18092	Kcnn4/Ab	2
GO:0042755	GO:0042755	eating behavior	2/374	49/23328	0.185313371	0.30081	0.18092	Cd36/Tcf1	2
GO:1902808	GO:1902808	positive regulation of cell cycle G1/S phase transition	2/374	49/23328	0.185313371	0.30081	0.18092	Rgcc/Cyp	2
GO:0009308	GO:0009308	amine metabolic process	3/374	93/23328	0.187592122	0.30081	0.18092	Vcam1/Cy	3
GO:0033077	GO:0033077	T cell differentiation in thymus	3/374	93/23328	0.187592122	0.30081	0.18092	Bmp4/Gat	3
GO:0043506	GO:0043506	regulation of JUN kinase activity	3/374	93/23328	0.187592122	0.30081	0.18092	Sfrp1/Sfrp	3
GO:0001542	GO:0001542	ovulation from ovarian follicle	1/374	13/23328	0.189547343	0.30081	0.18092	Nos3	1
GO:0001660	GO:0001660	fever generation	1/374	13/23328	0.189547343	0.30081	0.18092	Ednrb	1
GO:0006983	GO:0006983	ER overload response	1/374	13/23328	0.189547343	0.30081	0.18092	Hspa5	1
GO:0007320	GO:0007320	insemination	1/374	13/23328	0.189547343	0.30081	0.18092	Serpine2	1
GO:0008212	GO:0008212	mineralocorticoid metabolic process	1/374	13/23328	0.189547343	0.30081	0.18092	Dkk3	1
GO:0009148	GO:0009148	pyrimidine nucleoside triphosphate biosynthetic proces	1/374	13/23328	0.189547343	0.30081	0.18092	Cmpk2	1
GO:0009263	GO:0009263	deoxyribonucleotide biosynthetic process	1/374	13/23328	0.189547343	0.30081	0.18092	Cmpk2	1
GO:0009950	GO:0009950	dorsal/ventral axis specification	1/374	13/23328	0.189547343	0.30081	0.18092	Sfrp1	1
GO:0010359	GO:0010359	regulation of anion channel activity	1/374	13/23328	0.189547343	0.30081	0.18092	Prnp	1
GO:0010739	GO:0010739	positive regulation of protein kinase A signaling	1/374	13/23328	0.189547343	0.30081	0.18092	Akap12	1
GO:0015816	GO:0015816	glycine transport	1/374	13/23328	0.189547343	0.30081	0.18092	Rgs4	1
GO:0021604	GO:0021604	cranial nerve structural organization	1/374	13/23328	0.189547343	0.30081	0.18092	Nrp2	1
GO:0021670	GO:0021670	lateral ventricle development	1/374	13/23328	0.189547343	0.30081	0.18092	Aqp1	1
GO:0021794	GO:0021794	thalamus development	1/374	13/23328	0.189547343	0.30081	0.18092	Ncam1	1
GO:0030948	GO:0030948	negative regulation of vascular endothelial growth facto	1/374	13/23328	0.189547343	0.30081	0.18092	Mmrn2	1
GO:0031269	GO:0031269	pseudopodium assembly	1/374	13/23328	0.189547343	0.30081	0.18092	Cdc42ep3	1
GO:0031953	GO:0031953	negative regulation of protein autophosphorylation	1/374	13/23328	0.189547343	0.30081	0.18092	Jun	1
GO:0032026	GO:0032026	response to magnesium ion	1/374	13/23328	0.189547343	0.30081	0.18092	Thbs1	1
GO:0032060	GO:0032060	bleb assembly	1/374	13/23328	0.189547343	0.30081	0.18092	Mylk	1
GO:0032372	GO:0032372	negative regulation of sterol transport	1/374	13/23328	0.189547343	0.30081	0.18092	Apoe	1
GO:0032375	GO:0032375	negative regulation of cholesterol transport	1/374	13/23328	0.189547343	0.30081	0.18092	Apoe	1
GO:0032486	GO:0032486	Rap protein signal transduction	1/374	13/23328	0.189547343	0.30081	0.18092	Plk2	1
GO:0032488	GO:0032488	Cdc42 protein signal transduction	1/374	13/23328	0.189547343	0.30081	0.18092	Apoe	1
GO:0032799	GO:0032799	low-density lipoprotein receptor particle metabolic proc	1/374	13/23328	0.189547343	0.30081	0.18092	Apoe	1
GO:0033083	GO:0033083	regulation of immature T cell proliferation	1/374	13/23328	0.189547343	0.30081	0.18092	Bmp4	1
GO:0033280	GO:0033280	response to vitamin D	1/374	13/23328	0.189547343	0.30081	0.18092	Pim1	1
GO:0034350	GO:0034350	regulation of glial cell apoptotic process	1/374	13/23328	0.189547343	0.30081	0.18092	Akap12	1
GO:0039692	GO:0039692	single stranded viral RNA replication via double strand	1/374	13/23328	0.189547343	0.30081	0.18092	Apobec3	1
GO:0042994	GO:0042994	cytoplasmic sequestering of transcription factor	1/374	13/23328	0.189547343	0.30081	0.18092	Nfkbia	1
GO:0043970	GO:0043970	histone H3-K9 acetylation	1/374	13/23328	0.189547343	0.30081	0.18092	Gata3	1
GO:0045176	GO:0045176	apical protein localization	1/374	13/23328	0.189547343	0.30081	0.18092	Actb	1
GO:0046007	GO:0046007	negative regulation of activated T cell proliferation	1/374	13/23328	0.189547343	0.30081	0.18092	Prnp	1
GO:0046512	GO:0046512	sphingosine biosynthetic process	1/374	13/23328	0.189547343	0.30081	0.18092	Acer2	1
GO:0046520	GO:0046520	sphingoid biosynthetic process	1/374	13/23328	0.189547343	0.30081	0.18092	Acer2	1
GO:0048012	GO:0048012	hepatocyte growth factor receptor signaling pathway	1/374	13/23328	0.189547343	0.30081	0.18092	Esm1	1
GO:0051639	GO:0051639	actin filament network formation	1/374	13/23328	0.189547343	0.30081	0.18092	Actn1	1
GO:0055062	GO:0055062	phosphate ion homeostasis	1/374	13/23328	0.189547343	0.30081	0.18092	Pthlh	1
GO:0055119	GO:0055119	relaxation of cardiac muscle	1/374	13/23328	0.189547343	0.30081	0.18092	Atp1b1	1
GO:0060272	GO:0060272	embryonic skeletal joint morphogenesis	1/374	13/23328	0.189547343	0.30081	0.18092	Bmp4	1
GO:0060340	GO:0060340	positive regulation of type I interferon-mediated signali	1/374	13/23328	0.189547343	0.30081	0.18092	Irf7	1
GO:0060601	GO:0060601	lateral sprouting from an epithelium	1/374	13/23328	0.189547343	0.30081	0.18092	Bmp4	1
GO:0070431	GO:0070431	nucleotide-binding oligomerization domain containing 2	1/374	13/23328	0.189547343	0.30081	0.18092	Nfkbia	1
GO:0071073	GO:0071073	positive regulation of phospholipid biosynthetic proces	1/374	13/23328	0.189547343	0.30081	0.18092	Adgrf5	1
GO:0071295	GO:0071295	cellular response to vitamin	1/374	13/23328	0.189547343	0.30081	0.18092	Pim1	1
GO:0071679	GO:0071679	commissural neuron axon guidance	1/374	13/23328	0.189547343	0.30081	0.18092	Ncam1	1
GO:0072160	GO:0072160	nephron tubule epithelial cell differentiation	1/374	13/23328	0.189547343	0.30081	0.18092	Gata3	1
GO:0072376	GO:0072376	protein activation cascade	1/374	13/23328	0.189547343	0.30081	0.18092	Fn1	1
GO:0072505	GO:0072505	divalent inorganic anion homeostasis	1/374	13/23328	0.189547343	0.30081	0.18092	Pthlh	1
GO:0072506	GO:0072506	trivalent inorganic anion homeostasis	1/374	13/23328	0.189547343	0.30081	0.18092	Pthlh	1
GO:0086067	GO:0086067	AV node cell to bundle of His cell communication	1/374	13/23328	0.189547343	0.30081	0.18092	Gja5	1
GO:0090193	GO:0090193	positive regulation of glomerulus development	1/374	13/23328	0.189547343	0.30081	0.18092	Pdgfb	1
GO:0097090	GO:0097090	presynaptic membrane organization	1/374	13/23328	0.189547343	0.30081	0.18092	Apoe	1
GO:0097201	GO:0097201	negative regulation of transcription from RNA polymera	1/374	13/23328	0.189547343	0.30081	0.18092	Jun	1
GO:0097577	GO:0097577	sequestering of iron ion	1/374	13/23328	0.189547343	0.30081	0.18092	Lcn2	1
GO:0097688	GO:0097688	glutamate receptor clustering	1/374	13/23328	0.189547343	0.30081	0.18092	Apoe	1
GO:0099558	GO:0099558	maintenance of synapse structure	1/374	13/23328	0.189547343	0.30081	0.18092	Chchd10	1
GO:0110154	GO:0110154	RNA decapping	1/374	13/23328	0.189547343	0.30081	0.18092	Zfp36	1
GO:0110156	GO:0110156	methylguanosine-cap decapping	1/374	13/23328	0.189547343	0.30081	0.18092	Zfp36	1
GO:1900025	GO:1900025	negative regulation of substrate adhesion-dependent c	1/374	13/23328	0.189547343	0.30081	0.18092	Kank1	1
GO:1901201	GO:1901201	regulation of extracellular matrix assembly	1/374	13/23328	0.189547343	0.30081	0.18092	Rgcc	1
GO:1901678	GO:1901678	iron coordination entity transport	1/374	13/23328	0.189547343	0.30081	0.18092	Lcn2	1
GO:1902285	GO:1902285	semaphorin-plexin signaling pathway involved in neuro	1/374	13/23328	0.189547343	0.30081	0.18092	Nrp2	1
GO:1903430	GO:1903430	negative regulation of cell maturation	1/374	13/23328	0.189547343	0.30081	0.18092	Ednrb	1
GO:1903862	GO:1903862	positive regulation of oxidative phosphorylation	1/374	13/23328	0.189547343	0.30081	0.18092	Nupr1	1
GO:1904251	GO:1904251	regulation of bile acid metabolic process	1/374	13/23328	0.189547343	0.30081	0.18092	Prox1	1
GO:2001223	GO:2001223	negative regulation of neuron migration	1/374	13/23328	0.189547343	0.30081	0.18092	Col3a1	1
GO:0050796	GO:0050796	regulation of insulin secretion	5/374	190/23328	0.190541179	0.30203	0.18165	Sfrp1/Cd3	5
GO:0001909	GO:0001909	leukocyte mediated cytotoxicity	4/374	141/23328	0.191007968	0.30203	0.18165	Cxcl1/H2-	4

GO:0009799	GO:0009799	specification of symmetry	4/374	141/23328	0.191007968	0.30203	0.18165	Bmp4/Pcs	4
GO:0008038	GO:0008038	neuron recognition	2/374	50/23328	0.191115246	0.30203	0.18165	Ncam1/En	2
GO:0021545	GO:0021545	cranial nerve development	2/374	50/23328	0.191115246	0.30203	0.18165	Nrp2/Hes1	2
GO:0032309	GO:0032309	icosanoid secretion	2/374	50/23328	0.191115246	0.30203	0.18165	Pla2g5/Ed	2
GO:0051653	GO:0051653	spindle localization	2/374	50/23328	0.191115246	0.30203	0.18165	Itgb1/Gja1	2
GO:0060147	GO:0060147	regulation of posttranscriptional gene silencing	2/374	50/23328	0.191115246	0.30203	0.18165	Bmp4/Zfp	2
GO:0060966	GO:0060966	regulation of gene silencing by RNA	2/374	50/23328	0.191115246	0.30203	0.18165	Bmp4/Zfp	2
GO:0070229	GO:0070229	negative regulation of lymphocyte apoptotic process	2/374	50/23328	0.191115246	0.30203	0.18165	Bmp4/Cd7	2
GO:1902475	GO:1902475	L-alpha-amino acid transmembrane transport	2/374	50/23328	0.191115246	0.30203	0.18165	Rgs4/Itgb	2
GO:0070252	GO:0070252	actin-mediated cell contraction	3/374	94/23328	0.191646217	0.30264	0.18202	Gja5/Pdpr	3
GO:0070664	GO:0070664	negative regulation of leukocyte proliferation	3/374	94/23328	0.191646217	0.30264	0.18202	Bmp4/Cd2	3
GO:0034504	GO:0034504	protein localization to nucleus	7/374	293/23328	0.192464909	0.30382	0.18273	Bmp4/Cd3	7
GO:0046488	GO:0046488	phosphatidylinositol metabolic process	4/374	142/23328	0.194259579	0.30619	0.18415	Pdgfa/Pitp	4
GO:0048706	GO:0048706	embryonic skeletal system development	4/374	142/23328	0.194259579	0.30619	0.18415	Bmp4/Scx	4
GO:1901991	GO:1901991	negative regulation of mitotic cell cycle phase transitio	4/374	142/23328	0.194259579	0.30619	0.18415	Fhl1/Rgcc	4
GO:1902850	GO:1902850	microtubule cytoskeleton organization involved in mito	4/374	142/23328	0.194259579	0.30619	0.18415	Itgb1/Plk2	4
GO:0021954	GO:0021954	central nervous system neuron development	3/374	95/23328	0.195722301	0.30826	0.1854	Cdh11/Btg	3
GO:0051341	GO:0051341	regulation of oxidoreductase activity	3/374	95/23328	0.195722301	0.30826	0.1854	Scarb1/Ap	3
GO:0000288	GO:0000288	nuclear-transcribed mRNA catabolic process, deadeny	2/374	51/23328	0.196940917	0.30913	0.18592	Btg2/Zfp3	2
GO:0008089	GO:0008089	anterograde axonal transport	2/374	51/23328	0.196940917	0.30913	0.18592	Fez1/Hspl	2
GO:0031648	GO:0031648	protein destabilization	2/374	51/23328	0.196940917	0.30913	0.18592	Id1/Prnp	2
GO:0035088	GO:0035088	establishment or maintenance of apical/basal cell pola	2/374	51/23328	0.196940917	0.30913	0.18592	Fscn1/Tcf	2
GO:0035137	GO:0035137	hindlimb morphogenesis	2/374	51/23328	0.196940917	0.30913	0.18592	Bmp4/Hox	2
GO:0060428	GO:0060428	lung epithelium development	2/374	51/23328	0.196940917	0.30913	0.18592	Bmp4/Klf2	2
GO:0060760	GO:0060760	positive regulation of response to cytokine stimulus	2/374	51/23328	0.196940917	0.30913	0.18592	Irf7/Cd74	2
GO:0061245	GO:0061245	establishment or maintenance of bipolar cell polarity	2/374	51/23328	0.196940917	0.30913	0.18592	Fscn1/Tcf	2
GO:0071470	GO:0071470	cellular response to osmotic stress	2/374	51/23328	0.196940917	0.30913	0.18592	Aqp1/Mylk	2
GO:0035725	GO:0035725	sodium ion transmembrane transport	4/374	143/23328	0.197528361	0.30993	0.1864	Atp1b1/Fx	4
GO:0060998	GO:0060998	regulation of dendritic spine development	3/374	96/23328	0.199819554	0.3107	0.18687	Apoe/Plk2	3
GO:2000243	GO:2000243	positive regulation of reproductive process	3/374	96/23328	0.199819554	0.3107	0.18687	Ednrb/Pde	3
GO:0002756	GO:0002756	MyD88-independent toll-like receptor signaling pathwa	1/374	14/23328	0.202547957	0.3107	0.18687	Irf7	1
GO:0002885	GO:0002885	positive regulation of hypersensitivity	1/374	14/23328	0.202547957	0.3107	0.18687	C3	1
GO:0007351	GO:0007351	tripartite regional subdivision	1/374	14/23328	0.202547957	0.3107	0.18687	Pcsk6	1
GO:0008298	GO:0008298	intracellular mRNA localization	1/374	14/23328	0.202547957	0.3107	0.18687	Zfp36	1
GO:0008595	GO:0008595	anterior/posterior axis specification, embryo	1/374	14/23328	0.202547957	0.3107	0.18687	Pcsk6	1
GO:0009084	GO:0009084	glutamine family amino acid biosynthetic process	1/374	14/23328	0.202547957	0.3107	0.18687	Ass1	1
GO:0010882	GO:0010882	regulation of cardiac muscle contraction by calcium ior	1/374	14/23328	0.202547957	0.3107	0.18687	Atp1b1	1
GO:0010889	GO:0010889	regulation of sequestering of triglyceride	1/374	14/23328	0.202547957	0.3107	0.18687	Lpl	1
GO:0018158	GO:0018158	protein oxidation	1/374	14/23328	0.202547957	0.3107	0.18687	Lox	1
GO:0019370	GO:0019370	leukotriene biosynthetic process	1/374	14/23328	0.202547957	0.3107	0.18687	Pla2g5	1
GO:0019896	GO:0019896	axonal transport of mitochondrion	1/374	14/23328	0.202547957	0.3107	0.18687	Fez1	1
GO:0021903	GO:0021903	rostrocaudal neural tube patterning	1/374	14/23328	0.202547957	0.3107	0.18687	Hes1	1
GO:0021984	GO:0021984	adenohypophysis development	1/374	14/23328	0.202547957	0.3107	0.18687	Hes1	1
GO:0030213	GO:0030213	hyaluronan biosynthetic process	1/374	14/23328	0.202547957	0.3107	0.18687	Pdgfb	1
GO:0032276	GO:0032276	regulation of gonadotropin secretion	1/374	14/23328	0.202547957	0.3107	0.18687	Gja1	1
GO:0032328	GO:0032328	alanine transport	1/374	14/23328	0.202547957	0.3107	0.18687	Slc6a6	1
GO:0032525	GO:0032525	somite rostral/caudal axis specification	1/374	14/23328	0.202547957	0.3107	0.18687	Nrarp	1
GO:0032736	GO:0032736	positive regulation of interleukin-13 production	1/374	14/23328	0.202547957	0.3107	0.18687	Gata3	1
GO:0033079	GO:0033079	immature T cell proliferation	1/374	14/23328	0.202547957	0.3107	0.18687	Bmp4	1
GO:0034116	GO:0034116	positive regulation of heterotypic cell-cell adhesion	1/374	14/23328	0.202547957	0.3107	0.18687	Thy1	1
GO:0034309	GO:0034309	primary alcohol biosynthetic process	1/374	14/23328	0.202547957	0.3107	0.18687	Dkk3	1
GO:0036296	GO:0036296	response to increased oxygen levels	1/374	14/23328	0.202547957	0.3107	0.18687	Pdpn	1
GO:0036376	GO:0036376	sodium ion export across plasma membrane	1/374	14/23328	0.202547957	0.3107	0.18687	Atp1b1	1
GO:0036498	GO:0036498	IRE1-mediated unfolded protein response	1/374	14/23328	0.202547957	0.3107	0.18687	Hspa5	1
GO:0042035	GO:0042035	regulation of cytokine biosynthetic process	1/374	14/23328	0.202547957	0.3107	0.18687	Gata3	1
GO:0042362	GO:0042362	fat-soluble vitamin biosynthetic process	1/374	14/23328	0.202547957	0.3107	0.18687	Pltp	1
GO:0042532	GO:0042532	negative regulation of tyrosine phosphorylation of STA	1/374	14/23328	0.202547957	0.3107	0.18687	Socs3	1
GO:0043312	GO:0043312	neutrophil degranulation	1/374	14/23328	0.202547957	0.3107	0.18687	Anxa3	1
GO:0045060	GO:0045060	negative thymic T cell selection	1/374	14/23328	0.202547957	0.3107	0.18687	Cd74	1
GO:0045651	GO:0045651	positive regulation of macrophage differentiation	1/374	14/23328	0.202547957	0.3107	0.18687	Id2	1
GO:0045792	GO:0045792	negative regulation of cell size	1/374	14/23328	0.202547957	0.3107	0.18687	C1qtnf9	1
GO:0046479	GO:0046479	glycosphingolipid catabolic process	1/374	14/23328	0.202547957	0.3107	0.18687	Gm2a	1
GO:0048537	GO:0048537	mucosa-associated lymphoid tissue development	1/374	14/23328	0.202547957	0.3107	0.18687	Id2	1
GO:0048541	GO:0048541	Peyer's patch development	1/374	14/23328	0.202547957	0.3107	0.18687	Id2	1
GO:0048755	GO:0048755	branching morphogenesis of a nerve	1/374	14/23328	0.202547957	0.3107	0.18687	Cpe	1
GO:0050667	GO:0050667	homocysteine metabolic process	1/374	14/23328	0.202547957	0.3107	0.18687	Dpep1	1
GO:0051152	GO:0051152	positive regulation of smooth muscle cell differentiatior	1/374	14/23328	0.202547957	0.3107	0.18687	Tmsb4x	1
GO:0051451	GO:0051451	myoblast migration	1/374	14/23328	0.202547957	0.3107	0.18687	Net1	1
GO:0060019	GO:0060019	radial glial cell differentiation	1/374	14/23328	0.202547957	0.3107	0.18687	Hes1	1
GO:0060281	GO:0060281	regulation of oocyte development	1/374	14/23328	0.202547957	0.3107	0.18687	Pde3a	1
GO:0070207	GO:0070207	protein homotrimerization	1/374	14/23328	0.202547957	0.3107	0.18687	Steap4	1
GO:0070423	GO:0070423	nucleotide-binding oligomerization domain containing s	1/374	14/23328	0.202547957	0.3107	0.18687	Nfkbia	1
GO:0070942	GO:0070942	neutrophil mediated cytotoxicity	1/374	14/23328	0.202547957	0.3107	0.18687	Cxcl1	1
GO:0071340	GO:0071340	skeletal muscle acetylcholine-gated channel clustering	1/374	14/23328	0.202547957	0.3107	0.18687	Ptn	1
GO:0071803	GO:0071803	positive regulation of podosome assembly	1/374	14/23328	0.202547957	0.3107	0.18687	Fscn1	1
GO:0072683	GO:0072683	T cell extravasation	1/374	14/23328	0.202547957	0.3107	0.18687	F11r	1
GO:0086069	GO:0086069	bundle of His cell to Purkinje myocyte communication	1/374	14/23328	0.202547957	0.3107	0.18687	Gja5	1
GO:0090331	GO:0090331	negative regulation of platelet aggregation	1/374	14/23328	0.202547957	0.3107	0.18687	Serpine2	1
GO:0097066	GO:0097066	response to thyroid hormone	1/374	14/23328	0.202547957	0.3107	0.18687	Lmo2	1
GO:0097202	GO:0097202	activation of cysteine-type endopeptidase activity	1/374	14/23328	0.202547957	0.3107	0.18687	Perp	1
GO:1902001	GO:1902001	fatty acid transmembrane transport	1/374	14/23328	0.202547957	0.3107	0.18687	Thbs1	1
GO:1903624	GO:1903624	regulation of DNA catabolic process	1/374	14/23328	0.202547957	0.3107	0.18687	Igfbp3	1
GO:1904396	GO:1904396	regulation of neuromuscular junction development	1/374	14/23328	0.202547957	0.3107	0.18687	Ptn	1
GO:1905063	GO:1905063	regulation of vascular associated smooth muscle cell c	1/374	14/23328	0.202547957	0.3107	0.18687	Pdgfb	1
GO:1905879	GO:1905879	regulation of oogenesis	1/374	14/23328	0.202547957	0.3107	0.18687	Pde3a	1
GO:2000035	GO:2000035	regulation of stem cell division	1/374	14/23328	0.202547957	0.3107	0.18687	Sfrp2	1
GO:2000052	GO:2000052	positive regulation of non-canonical Wnt signaling path	1/374	14/23328	0.202547957	0.3107	0.18687	Sfrp1	1
GO:2000347	GO:2000347	positive regulation of hepatocyte proliferation	1/374	14/23328	0.202547957	0.3107	0.18687	Ptn	1
GO:0002888	GO:0002888	positive regulation of myeloid leukocyte mediated imm	2/374	52/23328	0.202788136	0.3107	0.18687	Cxcl1/C3	2
GO:0032481	GO:0032481	positive regulation of type I interferon production	2/374	52/23328	0.202788136	0.3107	0.18687	Irf7/Hmgb	2
GO:0061014	GO:0061014	positive regulation of mRNA catabolic process	2/374	52/23328	0.202788136	0.3107	0.18687	Btg2/Zfp3	2
GO:0140353	GO:0140353	lipid export from cell	2/374	52/23328	0.202788136	0.3107	0.18687	Igfbp3/Ed	2
GO:0006885	GO:0006885	regulation of pH	3/374	97/23328	0.203937159	0.31235	0.18786	Ednrb/Car	3
GO:0017148	GO:0017148	negative regulation of translation	4/374	145/23328	0.204115804	0.31251	0.18795	Btg2/Zfp3	4
GO:0006109	GO:0006109	regulation of carbohydrate metabolic process	5/374	195/23328	0.204485062	0.31296	0.18822	Igfbp3/Nu	5
GO:0050851	GO:0050851	antigen receptor-mediated signaling pathway	7/374	299/23328	0.205828582	0.3149	0.18939	Bmx/Kcnr	7
GO:0051606	GO:0051606	detection of stimulus	6/374	247/23328	0.206458626	0.31575	0.1899	Scarb1/Lb	6
GO:0001662	GO:0001662	behavioral fear response	2/374	53/23328	0.208654723	0.31794	0.19122	Apoe/Gja1	2
GO:0009395	GO:0009395	phospholipid catabolic process	2/374	53/23328	0.208654723	0.31794	0.19122	Scarb1/Pl	2
GO:0021795	GO:0021795	cerebral cortex cell migration	2/374	53/23328	0.208654723	0.31794	0.19122	Lamb1/Re	2
GO:0032648	GO:0032648	regulation of interferon-beta production	2/374	53/23328	0.208654723	0.31794	0.19122	Irf7/Hmgb	2
GO:0045839	GO:0045839	negative regulation of mitotic nuclear division	2/374	53/23328	0.208654723	0.31794	0.19122	Bmp4/Rgc	2
GO:0046676	GO:0046676	negative regulation of insulin secretion	2/374	53/23328	0.208654723	0.31794	0.19122	Sfrp1/Ucp	2
GO:0046902	GO:0046902	regulation of mitochondrial membrane permeability	2/374	53/23328	0.208654723	0.31794	0.19122	Chchd10/	2
GO:0051955	GO:0051955	regulation of amino acid transport	2/374	53/23328	0.208654723	0.31794	0.19122	Rgs4/Itgb	2
GO:0055010	GO:0055010	ventricular cardiac muscle tissue morphogenesis	2/374	53/23328	0.208654723	0.31794	0.19122	Prox1/Ly6	2
GO:0097720	GO:0097720	calcineurin-mediated signaling	2/374	53/23328	0.208654723	0.31794	0.19122	Lmcd1/Pr	2
GO:0060291	GO:0060291	long-term synaptic potentiation	6/374	248/23328	0.208964687	0.3183	0.19143	Apoe/Plk2	6
GO:0006911	GO:0006911	phagocytosis, engulfment	5/374	197/23328	0.210156116	0.31999	0.19245	Cd36/Lbp	5
GO:0048511	GO:0048511	rhythmic process	7/374	301/23328	0.210360292	0.32017	0.19256	Prox1/Ass	7
GO:0006417	GO:0006417	regulation of translation	8/374	355/23328	0.212037293	0.32017	0.19256	Btg2/Thbs	8
GO:0097061	GO:0097061	dendritic spine organization	3/374	99/23328	0.212230198	0.32017	0.19256	Apoe/Reln	3
GO:0042742	GO:0042742	defense response to bacterium	10/374	464/23328	0.213408741	0.32017	0.19256	Cd36/Lbp	10
GO:0031330	GO:0031330	negative regulation of cellular catabolic process	6/374	250/23328	0.214008999	0.32017	0.19256	Fez1/Nupr	6
GO:0002209	GO:0002209	behavioral defense response	2/374	54/23328	0.214538561	0.32017	0.19256	Apoe/Gja1	2
GO:0007019	GO:0007019	microtubule depolymerization	2/374	54/23328	0.214538561	0.32017	0.19256	Stmn2/Sg	2
GO:0032355	GO:0032355	response to estradiol	2/374	54/23328	0.214538561	0.32017	0.19256	Mmp2/Nos	2
GO:0042743	GO:0042743	hydrogen peroxide metabolic process	2/374	54/23328	0.214538561	0.32017	0.19256	Cyp1a1/G	2
GO:0048066	GO:0048066	developmental pigmentation	2/374	54/23328	0.214538561	0.32017	0.19256	Ednrb/Kitl	2
GO:0051954	GO:0051954	positive regulation of amine transport	2/374	54/23328	0.214538561	0.32017	0.19256	Cxcl12/Itg	2
GO:0070542	GO:0070542	response to fatty acid	2/374	54/23328	0.214538561	0.32017	0.19256	Cd36/Lpl	2
GO:1902930	GO:1902930	regulation of alcohol biosynthetic process	2/374	54/23328	0.214538561	0.32017	0.19256	Dkk3/Apo	2
GO:2001222	GO:2001222	regulation of neuron migration	2/374	54/23328	0.214538561	0.32017	0.19256	Reln/Col3	2
GO:0046883	GO:0046883	regulation of hormone secretion	7/374	303/23328	0.21492903	0.32017	0.19256	Sfrp1/Igfb	7
GO:0001845	GO:0001845	phagolysosome assembly	1/374	15/23328	0.215340573	0.32017	0.19256	Pla2g5	1
GO:0006862	GO:0006862	nucleotide transport	1/374	15/23328	0.215340573	0.32017	0.19256	Gja1	1
GO:0006978	GO:0006978	DNA damage response, signal transduction by p53 cla	1/374	15/23328	0.215340573	0.32017	0.19256	Cdkn1a	1
GO:0007379	GO:0007379	segment specification	1/374	15/23328	0.215340573	0.32017	0.19256	Meox1	1
GO:0010960	GO:0010960	magnesium ion homeostasis	1/374	15/23328	0.215340573	0.32017	0.19256	Pthlh	1

GO:0016553	GO:0016553	base conversion or substitution editing	1/374	15/23328	0.215340573	0.32017	0.19256	Apobec3	1
GO:0019377	GO:0019377	glycolipid catabolic process	1/374	15/23328	0.215340573	0.32017	0.19256	Gm2a	1
GO:0021819	GO:0021819	layer formation in cerebral cortex	1/374	15/23328	0.215340573	0.32017	0.19256	Reln	1
GO:0021932	GO:0021932	hindbrain radial glia guided cell migration	1/374	15/23328	0.215340573	0.32017	0.19256	Itgb1	1
GO:0030007	GO:0030007	cellular potassium ion homeostasis	1/374	15/23328	0.215340573	0.32017	0.19256	Atp1b1	1
GO:0030299	GO:0030299	intestinal cholesterol absorption	1/374	15/23328	0.215340573	0.32017	0.19256	Cd36	1
GO:0031268	GO:0031268	pseudopodium organization	1/374	15/23328	0.215340573	0.32017	0.19256	Cdc42ep3	1
GO:0031650	GO:0031650	regulation of heat generation	1/374	15/23328	0.215340573	0.32017	0.19256	Ednrb	1
GO:0032308	GO:0032308	positive regulation of prostaglandin secretion	1/374	15/23328	0.215340573	0.32017	0.19256	Edn1	1
GO:0032530	GO:0032530	regulation of microvillus organization	1/374	15/23328	0.215340573	0.32017	0.19256	Fscn1	1
GO:0034162	GO:0034162	toll-like receptor 9 signaling pathway	1/374	15/23328	0.215340573	0.32017	0.19256	Rsad2	1
GO:0034433	GO:0034433	steroid esterification	1/374	15/23328	0.215340573	0.32017	0.19256	Apoe	1
GO:0034434	GO:0034434	sterol esterification	1/374	15/23328	0.215340573	0.32017	0.19256	Apoe	1
GO:0034435	GO:0034435	cholesterol esterification	1/374	15/23328	0.215340573	0.32017	0.19256	Apoe	1
GO:0035872	GO:0035872	nucleotide-binding domain, leucine rich repeat containi	1/374	15/23328	0.215340573	0.32017	0.19256	Nfkbia	1
GO:0042089	GO:0042089	cytokine biosynthetic process	1/374	15/23328	0.215340573	0.32017	0.19256	Gata3	1
GO:0042159	GO:0042159	lipoprotein catabolic process	1/374	15/23328	0.215340573	0.32017	0.19256	Apoe	1
GO:0042416	GO:0042416	dopamine biosynthetic process	1/374	15/23328	0.215340573	0.32017	0.19256	Epas1	1
GO:0043383	GO:0043383	negative T cell selection	1/374	15/23328	0.215340573	0.32017	0.19256	Cd74	1
GO:0045605	GO:0045605	negative regulation of epidermal cell differentiation	1/374	15/23328	0.215340573	0.32017	0.19256	Hes1	1
GO:0046548	GO:0046548	retinal rod cell development	1/374	15/23328	0.215340573	0.32017	0.19256	Cfh	1
GO:0048070	GO:0048070	regulation of developmental pigmentation	1/374	15/23328	0.215340573	0.32017	0.19256	Kitl	1
GO:0048680	GO:0048680	positive regulation of axon regeneration	1/374	15/23328	0.215340573	0.32017	0.19256	Ptn	1
GO:0060049	GO:0060049	regulation of protein glycosylation	1/374	15/23328	0.215340573	0.32017	0.19256	Acer2	1
GO:0060347	GO:0060347	heart trabecula formation	1/374	15/23328	0.215340573	0.32017	0.19256	Tek	1
GO:0060546	GO:0060546	negative regulation of necroptotic process	1/374	15/23328	0.215340573	0.32017	0.19256	Bok	1
GO:0061029	GO:0061029	eyelid development in camera-type eye	1/374	15/23328	0.215340573	0.32017	0.19256	Jun	1
GO:0061042	GO:0061042	vascular wound healing	1/374	15/23328	0.215340573	0.32017	0.19256	Kdr	1
GO:0061051	GO:0061051	positive regulation of cell growth involved in cardiac mu	1/374	15/23328	0.215340573	0.32017	0.19256	Edn1	1
GO:0061318	GO:0061318	renal filtration cell differentiation	1/374	15/23328	0.215340573	0.32017	0.19256	Bmp4	1
GO:0062099	GO:0062099	negative regulation of programmed necrotic cell death	1/374	15/23328	0.215340573	0.32017	0.19256	Bok	1
GO:0070593	GO:0070593	dendrite self-avoidance	1/374	15/23328	0.215340573	0.32017	0.19256	Emb	1
GO:0072074	GO:0072074	kidney mesenchyme development	1/374	15/23328	0.215340573	0.32017	0.19256	Bmp4	1
GO:0072112	GO:0072112	glomerular visceral epithelial cell differentiation	1/374	15/23328	0.215340573	0.32017	0.19256	Bmp4	1
GO:0072283	GO:0072283	metanephric renal vesicle morphogenesis	1/374	15/23328	0.215340573	0.32017	0.19256	Bmp4	1
GO:0072311	GO:0072311	glomerular epithelial cell differentiation	1/374	15/23328	0.215340573	0.32017	0.19256	Bmp4	1
GO:0072350	GO:0072350	tricarboxylic acid metabolic process	1/374	15/23328	0.215340573	0.32017	0.19256	Ass1	1
GO:0086014	GO:0086014	atrial cardiac muscle cell action potential	1/374	15/23328	0.215340573	0.32017	0.19256	Gja5	1
GO:0086026	GO:0086026	atrial cardiac muscle cell to AV node cell signaling	1/374	15/23328	0.215340573	0.32017	0.19256	Gja5	1
GO:0086066	GO:0086066	atrial cardiac muscle cell to AV node cell communicatio	1/374	15/23328	0.215340573	0.32017	0.19256	Gja5	1
GO:0098712	GO:0098712	L-glutamate import across plasma membrane	1/374	15/23328	0.215340573	0.32017	0.19256	Itgb1	1
GO:0098917	GO:0098917	retrograde trans-synaptic signaling	1/374	15/23328	0.215340573	0.32017	0.19256	Fabp5	1
GO:1900225	GO:1900225	regulation of NLRP3 inflammasome complex assembly	1/374	15/23328	0.215340573	0.32017	0.19256	Cd36	1
GO:1902165	GO:1902165	regulation of intrinsic apoptotic signaling pathway in re	1/374	15/23328	0.215340573	0.32017	0.19256	Cd74	1
GO:1903543	GO:1903543	positive regulation of exosomal secretion	1/374	15/23328	0.215340573	0.32017	0.19256	Sdc1	1
GO:1903961	GO:1903961	positive regulation of anion transmembrane transport	1/374	15/23328	0.215340573	0.32017	0.19256	Itgb1	1
GO:0019439	GO:0019439	aromatic compound catabolic process	9/374	411/23328	0.216197423	0.32072	0.19289	Ephx2/Igfl	9
GO:0018209	GO:0018209	peptidyl-serine modification	8/374	357/23328	0.216244421	0.32072	0.19289	Bgn/Sfrp2	8
GO:0010771	GO:0010771	negative regulation of cell morphogenesis involved in d	3/374	100/23328	0.216404033	0.32072	0.19289	Id1/Thy1/H	3
GO:0032006	GO:0032006	regulation of TOR signaling	3/374	100/23328	0.216404033	0.32072	0.19289	Kdr/Reln/I	3
GO:0042102	GO:0042102	positive regulation of T cell proliferation	3/374	100/23328	0.216404033	0.32072	0.19289	Vcam1/Cd	3
GO:0050795	GO:0050795	regulation of behavior	3/374	100/23328	0.216404033	0.32072	0.19289	Apoe/Reln	3
GO:0098659	GO:0098659	inorganic cation import across plasma membrane	3/374	100/23328	0.216404033	0.32072	0.19289	Abcc9/Atp	3
GO:0099587	GO:0099587	inorganic ion import across plasma membrane	3/374	100/23328	0.216404033	0.32072	0.19289	Abcc9/Atp	3
GO:1902882	GO:1902882	regulation of response to oxidative stress	3/374	100/23328	0.216404033	0.32072	0.19289	Fbln5/Cd3	3
GO:0002673	GO:0002673	regulation of acute inflammatory response	2/374	55/23328	0.220437596	0.32566	0.19586	Ednrb/C3	2
GO:0032608	GO:0032608	interferon-beta production	2/374	55/23328	0.220437596	0.32566	0.19586	Irf7/Hmgb	2
GO:0032892	GO:0032892	positive regulation of organic acid transport	2/374	55/23328	0.220437596	0.32566	0.19586	Itgb1/Edn	2
GO:0042531	GO:0042531	positive regulation of tyrosine phosphorylation of STAT	2/374	55/23328	0.220437596	0.32566	0.19586	Pecam1/H	2
GO:0050775	GO:0050775	positive regulation of dendrite morphogenesis	2/374	55/23328	0.220437596	0.32566	0.19586	Reln/Sgk1	2
GO:0050922	GO:0050922	negative regulation of chemotaxis	2/374	55/23328	0.220437596	0.32566	0.19586	Thbs1/Du	2
GO:0051496	GO:0051496	positive regulation of stress fiber assembly	2/374	55/23328	0.220437596	0.32566	0.19586	Rgcc/Arhg	2
GO:0097035	GO:0097035	regulation of membrane lipid distribution	2/374	55/23328	0.220437596	0.32566	0.19586	Kcnn4/Ab	2
GO:1902750	GO:1902750	negative regulation of cell cycle G2/M phase transition	2/374	55/23328	0.220437596	0.32566	0.19586	Fhl1/Cdkn	2
GO:0001824	GO:0001824	blastocyst development	4/374	150/23328	0.220856351	0.32604	0.19609	Tm4sf1/At	4
GO:0002065	GO:0002065	columnar/cuboidal epithelial cell differentiation	4/374	150/23328	0.220856351	0.32604	0.19609	Bmp4/Pro	4
GO:0071375	GO:0071375	cellular response to peptide hormone stimulus	6/374	253/23328	0.221653468	0.32711	0.19673	C1qtnf9/L	6
GO:0016358	GO:0016358	dendrite development	7/374	306/23328	0.221849488	0.32728	0.19684	Apoe/Itgb	7
GO:1903362	GO:1903362	regulation of cellular protein catabolic process	6/374	254/23328	0.22422176	0.32891	0.19782	Clu/Apoe/	6
GO:0050830	GO:0050830	defense response to Gram-positive bacterium	4/374	151/23328	0.224247822	0.32891	0.19782	Cd36/Lbp	4
GO:0045582	GO:0045582	positive regulation of T cell differentiation	3/374	102/23328	0.224802384	0.32891	0.19782	Gata3/Sox	3
GO:0070227	GO:0070227	lymphocyte apoptotic process	3/374	102/23328	0.224802384	0.32891	0.19782	Bmp4/Cd	3
GO:0051251	GO:0051251	positive regulation of lymphocyte activation	9/374	416/23328	0.226094657	0.32891	0.19782	Vcam1/Ga	9
GO:0007618	GO:0007618	mating	2/374	56/23328	0.226349835	0.32891	0.19782	Ednrb/Ser	2
GO:0030219	GO:0030219	megakaryocyte differentiation	2/374	56/23328	0.226349835	0.32891	0.19782	Lox/Hmgb	2
GO:0042446	GO:0042446	hormone biosynthetic process	2/374	56/23328	0.226349835	0.32891	0.19782	Scarb1/Dh	2
GO:0061900	GO:0061900	glial cell activation	2/374	56/23328	0.226349835	0.32891	0.19782	Clu/Jun	2
GO:0070265	GO:0070265	necrotic cell death	2/374	56/23328	0.226349835	0.32891	0.19782	Bok/Slc6a	2
GO:0098815	GO:0098815	modulation of excitatory postsynaptic potential	2/374	56/23328	0.226349835	0.32891	0.19782	Rgs4/Reln	2
GO:1900449	GO:1900449	regulation of glutamate receptor signaling pathway	2/374	56/23328	0.226349835	0.32891	0.19782	Reln/Prnp	2
GO:0009743	GO:0009743	response to carbohydrate	5/374	203/23328	0.227464367	0.32891	0.19782	Vcam1/Th	5
GO:0002070	GO:0002070	epithelial cell maturation	1/374	16/23328	0.227928512	0.32891	0.19782	Gja1	1
GO:0002866	GO:0002866	positive regulation of acute inflammatory response to a	1/374	16/23328	0.227928512	0.32891	0.19782	C3	1
GO:0006620	GO:0006620	posttranslational protein targeting to endoplasmic retic	1/374	16/23328	0.227928512	0.32891	0.19782	Hspa5	1
GO:0006825	GO:0006825	copper ion transport	1/374	16/23328	0.227928512	0.32891	0.19782	Steap4	1
GO:0006878	GO:0006878	cellular copper ion homeostasis	1/374	16/23328	0.227928512	0.32891	0.19782	Prnp	1
GO:0009147	GO:0009147	pyrimidine nucleoside triphosphate metabolic process	1/374	16/23328	0.227928512	0.32891	0.19782	Cmpk2	1
GO:0014733	GO:0014733	regulation of skeletal muscle adaptation	1/374	16/23328	0.227928512	0.32891	0.19782	Igfbp5	1
GO:0021978	GO:0021978	telencephalon regionalization	1/374	16/23328	0.227928512	0.32891	0.19782	Bmp4	1
GO:0030238	GO:0030238	male sex determination	1/374	16/23328	0.227928512	0.32891	0.19782	Dhh	1
GO:0032048	GO:0032048	cardiolipin metabolic process	1/374	16/23328	0.227928512	0.32891	0.19782	Pla2g5	1
GO:0035461	GO:0035461	vitamin transmembrane transport	1/374	16/23328	0.227928512	0.32891	0.19782	Scarb1	1
GO:0035641	GO:0035641	locomotory exploration behavior	1/374	16/23328	0.227928512	0.32891	0.19782	Apoe	1
GO:0042308	GO:0042308	negative regulation of protein import into nucleus	1/374	16/23328	0.227928512	0.32891	0.19782	Cd36	1
GO:0042492	GO:0042492	gamma-delta T cell differentiation	1/374	16/23328	0.227928512	0.32891	0.19782	Sox4	1
GO:0042772	GO:0042772	DNA damage response, signal transduction resulting in	1/374	16/23328	0.227928512	0.32891	0.19782	Cdkn1a	1
GO:0043084	GO:0043084	penile erection	1/374	16/23328	0.227928512	0.32891	0.19782	Ednrb	1
GO:0043217	GO:0043217	myelin maintenance	1/374	16/23328	0.227928512	0.32891	0.19782	Clu	1
GO:0043518	GO:0043518	negative regulation of DNA damage response, signal t	1/374	16/23328	0.227928512	0.32891	0.19782	Cd74	1
GO:0043923	GO:0043923	positive regulation by host of viral transcription	1/374	16/23328	0.227928512	0.32891	0.19782	Jun	1
GO:0044068	GO:0044068	modulation by symbiont of host cellular process	1/374	16/23328	0.227928512	0.32891	0.19782	Gapdh	1
GO:0044154	GO:0044154	histone H3-K14 acetylation	1/374	16/23328	0.227928512	0.32891	0.19782	Gata3	1
GO:0046485	GO:0046485	ether lipid metabolic process	1/374	16/23328	0.227928512	0.32891	0.19782	Pla2g5	1
GO:0046549	GO:0046549	retinal cone cell development	1/374	16/23328	0.227928512	0.32891	0.19782	Thy1	1
GO:0048385	GO:0048385	regulation of retinoic acid receptor signaling pathway	1/374	16/23328	0.227928512	0.32891	0.19782	Klf2	1
GO:0048681	GO:0048681	negative regulation of axon regeneration	1/374	16/23328	0.227928512	0.32891	0.19782	Klk8	1
GO:0050862	GO:0050862	positive regulation of T cell receptor signaling pathway	1/374	16/23328	0.227928512	0.32891	0.19782	Kcnn4	1
GO:0051044	GO:0051044	positive regulation of membrane protein ectodomain pr	1/374	16/23328	0.227928512	0.32891	0.19782	Apoe	1
GO:0051151	GO:0051151	negative regulation of smooth muscle cell differentiatic	1/374	16/23328	0.227928512	0.32891	0.19782	Pdgfb	1
GO:0051938	GO:0051938	L-glutamate import	1/374	16/23328	0.227928512	0.32891	0.19782	Itgb1	1
GO:0055089	GO:0055089	fatty acid homeostasis	1/374	16/23328	0.227928512	0.32891	0.19782	Apoe	1
GO:0060391	GO:0060391	positive regulation of SMAD protein signal transductior	1/374	16/23328	0.227928512	0.32891	0.19782	Bmp4	1
GO:0060766	GO:0060766	negative regulation of androgen receptor signaling patl	1/374	16/23328	0.227928512	0.32891	0.19782	Sfrp1	1
GO:0061548	GO:0061548	ganglion development	1/374	16/23328	0.227928512	0.32891	0.19782	Nrp2	1
GO:0070572	GO:0070572	positive regulation of neuron projection regeneration	1/374	16/23328	0.227928512	0.32891	0.19782	Ptn	1
GO:0070885	GO:0070885	negative regulation of calcineurin-NFAT signaling casc	1/374	16/23328	0.227928512	0.32891	0.19782	Prnp	1
GO:0071599	GO:0071599	otic vesicle development	1/374	16/23328	0.227928512	0.32891	0.19782	Gata3	1
GO:0072498	GO:0072498	embryonic skeletal joint development	1/374	16/23328	0.227928512	0.32891	0.19782	Bmp4	1
GO:0097094	GO:0097094	craniofacial suture morphogenesis	1/374	16/23328	0.227928512	0.32891	0.19782	Bmp4	1
GO:0097284	GO:0097284	hepatocyte apoptotic process	1/374	16/23328	0.227928512	0.32891	0.19782	Krt18	1
GO:0098974	GO:0098974	postsynaptic actin cytoskeleton organization	1/374	16/23328	0.227928512	0.32891	0.19782	Actb	1
GO:0099188	GO:0099188	postsynaptic cytoskeleton organization	1/374	16/23328	0.227928512	0.32891	0.19782	Actb	1
GO:0106057	GO:0106057	negative regulation of calcineurin-mediated signaling	1/374	16/23328	0.227928512	0.32891	0.19782	Prnp	1
GO:1900037	GO:1900037	regulation of cellular response to hypoxia	1/374	16/23328	0.227928512	0.32891	0.19782	Cd34	1
GO:1901077	GO:1901077	regulation of relaxation of muscle	1/374	16/23328	0.227928512	0.32891	0.19782	Sln	1
GO:1903541	GO:1903541	regulation of exosomal secretion	1/374	16/23328	0.227928512	0.32891	0.19782	Sdc1	1
GO:1903729	GO:1903729	regulation of plasma membrane organization	1/374	16/23328	0.227928512	0.32891	0.19782	S100a9	1
GO:1903779	GO:1903779	regulation of cardiac conduction	1/374	16/23328	0.227928512	0.32891	0.19782	Gja5	1
GO:1904590	GO:1904590	negative regulation of protein import	1/374	16/23328	0.227928512	0.32891	0.19782	Cd36	1

GO:2000194	GO:2000194	regulation of female gonad development	1/374	16/23328	0.227928512	0.32891	0.19782	Nupr1	1
GO:2000651	GO:2000651	positive regulation of sodium ion transmembrane trans	1/374	16/23328	0.227928512	0.32891	0.19782	Atp1b1	1
GO:0042113	GO:0042113	B cell activation	9/374	417/2332٤	0.228094514	0.32904	0.1979	Sfrp1/Cd3	9
GO:0050772	GO:0050772	positive regulation of axonogenesis	3/374	103/2332٤	0.229025342	0.33027	0.19863	Apoe/Cxc	3
GO:0019748	GO:0019748	secondary metabolic process	2/374	57/23328	0.232273345	0.33461	0.20124	Pam/Cyp1	2
GO:0045058	GO:0045058	T cell selection	2/374	57/23328	0.232273345	0.33461	0.20124	Gata3/Cd7	2
GO:1901292	GO:1901292	nucleoside phosphate catabolic process	2/374	57/23328	0.232273345	0.33461	0.20124	Nudt4/Pde	2
GO:0099024	GO:0099024	plasma membrane invagination	5/374	206/2332٤	0.236272232	0.3395	0.20419	Cd36/Lbp/	5
GO:0001708	GO:0001708	cell fate specification	3/374	105/2332٤	0.237514985	0.3395	0.20419	Sfrp2/Itgb	3
GO:0002444	GO:0002444	myeloid leukocyte mediated immunity	3/374	105/2332٤	0.237514985	0.3395	0.20419	Cxcl1/Anx	3
GO:0009953	GO:0009953	dorsal/ventral pattern formation	3/374	105/2332٤	0.237514985	0.3395	0.20419	Sfrp1/Bmp	3
GO:0007292	GO:0007292	female gamete generation	4/374	155/2332٤	0.237944735	0.3395	0.20419	Pde3a/No	4
GO:0071322	GO:0071322	cellular response to carbohydrate stimulus	4/374	155/2332٤	0.237944735	0.3395	0.20419	Vcam1/Sc	4
GO:0006493	GO:0006493	protein O-linked glycosylation	2/374	58/23328	0.23820625	0.3395	0.20419	Gxylt2/Ga	2
GO:0010517	GO:0010517	regulation of phospholipase activity	2/374	58/23328	0.23820625	0.3395	0.20419	Flt1/Pla2g	2
GO:0032655	GO:0032655	regulation of interleukin-12 production	2/374	58/23328	0.23820625	0.3395	0.20419	Cd36/Thb	2
GO:0042596	GO:0042596	fear response	2/374	58/23328	0.23820625	0.3395	0.20419	Apoe/Gja1	2
GO:0001101	GO:0001101	response to acid chemical	5/374	207/2332٤	0.239229306	0.3395	0.20419	Col5a2/Mı	5
GO:0001821	GO:0001821	histamine secretion	1/374	17/23328	0.24031504	0.3395	0.20419	Edn1	1
GO:0001921	GO:0001921	positive regulation of receptor recycling	1/374	17/23328	0.24031504	0.3395	0.20419	Ece1	1
GO:0001967	GO:0001967	suckling behavior	1/374	17/23328	0.24031504	0.3395	0.20419	Hand2	1
GO:0002327	GO:0002327	immature B cell differentiation	1/374	17/23328	0.24031504	0.3395	0.20419	Cd24a	1
GO:0002689	GO:0002689	negative regulation of leukocyte chemotaxis	1/374	17/23328	0.24031504	0.3395	0.20419	Dusp1	1
GO:0003159	GO:0003159	morphogenesis of an endothelium	1/374	17/23328	0.24031504	0.3395	0.20419	Bmp4	1
GO:0006704	GO:0006704	glucocorticoid biosynthetic process	1/374	17/23328	0.24031504	0.3395	0.20419	Dkk3	1
GO:0008272	GO:0008272	sulfate transport	1/374	17/23328	0.24031504	0.3395	0.20419	Slc26a10	1
GO:0015701	GO:0015701	bicarbonate transport	1/374	17/23328	0.24031504	0.3395	0.20419	Car4	1
GO:0021854	GO:0021854	hypothalamus development	1/374	17/23328	0.24031504	0.3395	0.20419	Nrp2	1
GO:0030730	GO:0030730	sequestering of triglyceride	1/374	17/23328	0.24031504	0.3395	0.20419	Lpl	1
GO:0032306	GO:0032306	regulation of prostaglandin secretion	1/374	17/23328	0.24031504	0.3395	0.20419	Edn1	1
GO:0032682	GO:0032682	negative regulation of chemokine production	1/374	17/23328	0.24031504	0.3395	0.20419	Cd24a	1
GO:0034349	GO:0034349	glial cell apoptotic process	1/374	17/23328	0.24031504	0.3395	0.20419	Akap12	1
GO:0034755	GO:0034755	iron ion transmembrane transport	1/374	17/23328	0.24031504	0.3395	0.20419	Steap4	1
GO:0040015	GO:0040015	negative regulation of multicellular organism growth	1/374	17/23328	0.24031504	0.3395	0.20419	H19	1
GO:0042670	GO:0042670	retinal cone cell differentiation	1/374	17/23328	0.24031504	0.3395	0.20419	Thy1	1
GO:0043249	GO:0043249	erythrocyte maturation	1/374	17/23328	0.24031504	0.3395	0.20419	Klf2	1
GO:0044546	GO:0044546	NLRP3 inflammasome complex assembly	1/374	17/23328	0.24031504	0.3395	0.20419	Cd36	1
GO:0044794	GO:0044794	positive regulation by host of viral process	1/374	17/23328	0.24031504	0.3395	0.20419	Apoe	1
GO:0045059	GO:0045059	positive thymic T cell selection	1/374	17/23328	0.24031504	0.3395	0.20419	Cd74	1
GO:0045064	GO:0045064	T-helper 2 cell differentiation	1/374	17/23328	0.24031504	0.3395	0.20419	Gata3	1
GO:0045198	GO:0045198	establishment of epithelial cell apical/basal polarity	1/374	17/23328	0.24031504	0.3395	0.20419	Tcf15	1
GO:0045793	GO:0045793	positive regulation of cell size	1/374	17/23328	0.24031504	0.3395	0.20419	Edn1	1
GO:0050665	GO:0050665	hydrogen peroxide biosynthetic process	1/374	17/23328	0.24031504	0.3395	0.20419	Cyp1a1	1
GO:0051004	GO:0051004	regulation of lipoprotein lipase activity	1/374	17/23328	0.24031504	0.3395	0.20419	Gpihbp1	1
GO:0051014	GO:0051014	actin filament severing	1/374	17/23328	0.24031504	0.3395	0.20419	Gmfg	1
GO:0055012	GO:0055012	ventricular cardiac muscle cell differentiation	1/374	17/23328	0.24031504	0.3395	0.20419	Prox1	1
GO:0055070	GO:0055070	copper ion homeostasis	1/374	17/23328	0.24031504	0.3395	0.20419	Prnp	1
GO:0060644	GO:0060644	mammary gland epithelial cell differentiation	1/374	17/23328	0.24031504	0.3395	0.20419	Id2	1
GO:0060788	GO:0060788	ectodermal placode formation	1/374	17/23328	0.24031504	0.3395	0.20419	Prox1	1
GO:0061154	GO:0061154	endothelial tube morphogenesis	1/374	17/23328	0.24031504	0.3395	0.20419	Bmp4	1
GO:0061179	GO:0061179	negative regulation of insulin secretion involved in cell	1/374	17/23328	0.24031504	0.3395	0.20419	Ucp2	1
GO:0070243	GO:0070243	regulation of thymocyte apoptotic process	1/374	17/23328	0.24031504	0.3395	0.20419	Bmp4	1
GO:0070262	GO:0070262	peptidyl-serine dephosphorylation	1/374	17/23328	0.24031504	0.3395	0.20419	Dusp1	1
GO:0070886	GO:0070886	positive regulation of calcineurin-NFAT signaling casca	1/374	17/23328	0.24031504	0.3395	0.20419	Lmcd1	1
GO:0071697	GO:0071697	ectodermal placode morphogenesis	1/374	17/23328	0.24031504	0.3395	0.20419	Prox1	1
GO:0072672	GO:0072672	neutrophil extravasation	1/374	17/23328	0.24031504	0.3395	0.20419	Pecam1	1
GO:0086012	GO:0086012	membrane depolarization during cardiac muscle cell ac	1/374	17/23328	0.24031504	0.3395	0.20419	Gja5	1
GO:0090493	GO:0090493	catecholamine uptake	1/374	17/23328	0.24031504	0.3395	0.20419	Actb	1
GO:0098814	GO:0098814	spontaneous synaptic transmission	1/374	17/23328	0.24031504	0.3395	0.20419	Itgb1	1
GO:0099640	GO:0099640	axo-dendritic protein transport	1/374	17/23328	0.24031504	0.3395	0.20419	Hspb1	1
GO:0106058	GO:0106058	positive regulation of calcineurin-mediated signaling	1/374	17/23328	0.24031504	0.3395	0.20419	Lmcd1	1
GO:1902176	GO:1902176	negative regulation of oxidative stress-induced intrinsic	1/374	17/23328	0.24031504	0.3395	0.20419	Hspb1	1
GO:2000047	GO:2000047	regulation of cell-cell adhesion mediated by cadherin	1/374	17/23328	0.24031504	0.3395	0.20419	Rgcc	1
GO:2000369	GO:2000369	regulation of clathrin-dependent endocytosis	1/374	17/23328	0.24031504	0.3395	0.20419	Sh3gl3	1
GO:0007224	GO:0007224	smoothened signaling pathway	4/374	156/2332٤	0.241399706	0.34092	0.20504	Sfrp1/Dhh	4
GO:0000041	GO:0000041	transition metal ion transport	3/374	106/2332٤	0.241780157	0.34111	0.20515	Steap4/M	3
GO:0043473	GO:0043473	pigmentation	3/374	106/2332٤	0.241780157	0.34111	0.20515	Ednrb/Kitl	3
GO:0106027	GO:0106027	neuron projection organization	3/374	106/2332٤	0.241780157	0.34111	0.20515	Apoe/Reln	3
GO:0001836	GO:0001836	release of cytochrome c from mitochondria	2/374	59/23328	0.244146733	0.34387	0.20681	Jun/Bok	2
GO:0007528	GO:0007528	neuromuscular junction development	2/374	59/23328	0.244146733	0.34387	0.20681	F2r/Ptn	2
GO:0007588	GO:0007588	excretion	2/374	59/23328	0.244146733	0.34387	0.20681	Ednrb/Edr	2
GO:0043462	GO:0043462	regulation of ATPase activity	2/374	59/23328	0.244146733	0.34387	0.20681	Atp1b1/M	2
GO:0044773	GO:0044773	mitotic DNA damage checkpoint	2/374	59/23328	0.244146733	0.34387	0.20681	Sox4/Cdk	2
GO:0007005	GO:0007005	mitochondrion organization	10/374	481/2332٤	0.245404447	0.34552	0.20781	Kdr/Fez1/	10
GO:1901988	GO:1901988	negative regulation of cell cycle phase transition	4/374	158/2332٤	0.248343736	0.34954	0.21023	Fhl1/Rgcc	4
GO:0021517	GO:0021517	ventral spinal cord development	2/374	60/23328	0.25009303	0.3502	0.21062	Sox4/Reln	2
GO:0048260	GO:0048260	positive regulation of receptor-mediated endocytosis	2/374	60/23328	0.25009303	0.3502	0.21062	Clu/C3	2
GO:0098900	GO:0098900	regulation of action potential	2/374	60/23328	0.25009303	0.3502	0.21062	Gja5/Cd3	2
GO:0021987	GO:0021987	cerebral cortex development	3/374	108/2332٤	0.250347475	0.3502	0.21062	Lamb1/Re	3
GO:2000058	GO:2000058	regulation of ubiquitin-dependent protein catabolic pro	4/374	159/2332٤	0.251831999	0.3502	0.21062	Clu/Cbfa2	4
GO:0000188	GO:0000188	inactivation of MAPK activity	1/374	18/23328	0.252503371	0.3502	0.21062	Dusp1	1
GO:0002076	GO:0002076	osteoblast development	1/374	18/23328	0.252503371	0.3502	0.21062	Pthlh	1
GO:0002281	GO:0002281	macrophage activation involved in immune response	1/374	18/23328	0.252503371	0.3502	0.21062	Lbp	1
GO:0002283	GO:0002283	neutrophil activation involved in immune response	1/374	18/23328	0.252503371	0.3502	0.21062	Anxa3	1
GO:0002836	GO:0002836	positive regulation of response to tumor cell	1/374	18/23328	0.252503371	0.3502	0.21062	Cd24a	1
GO:0002839	GO:0002839	positive regulation of immune response to tumor cell	1/374	18/23328	0.252503371	0.3502	0.21062	Cd24a	1
GO:0002883	GO:0002883	regulation of hypersensitivity	1/374	18/23328	0.252503371	0.3502	0.21062	C3	1
GO:0003128	GO:0003128	heart field specification	1/374	18/23328	0.252503371	0.3502	0.21062	Bmp4	1
GO:0006390	GO:0006390	mitochondrial transcription	1/374	18/23328	0.252503371	0.3502	0.21062	Chchd10	1
GO:0006474	GO:0006474	N-terminal protein amino acid acetylation	1/374	18/23328	0.252503371	0.3502	0.21062	Sox4	1
GO:0006662	GO:0006662	glycerol ether metabolic process	1/374	18/23328	0.252503371	0.3502	0.21062	Pla2g5	1
GO:0006700	GO:0006700	C21-steroid hormone biosynthetic process	1/374	18/23328	0.252503371	0.3502	0.21062	Dkk3	1
GO:0010801	GO:0010801	negative regulation of peptidyl-threonine phosphorylati	1/374	18/23328	0.252503371	0.3502	0.21062	Ddit4	1
GO:0010867	GO:0010867	positive regulation of triglyceride biosynthetic process	1/374	18/23328	0.252503371	0.3502	0.21062	Scarb1	1
GO:0014041	GO:0014041	regulation of neuron maturation	1/374	18/23328	0.252503371	0.3502	0.21062	Ednrb	1
GO:0030033	GO:0030033	microvillus assembly	1/374	18/23328	0.252503371	0.3502	0.21062	Fscn1	1
GO:0031281	GO:0031281	positive regulation of cyclase activity	1/374	18/23328	0.252503371	0.3502	0.21062	Nos3	1
GO:0032674	GO:0032674	regulation of interleukin-5 production	1/374	18/23328	0.252503371	0.3502	0.21062	Gata3	1
GO:0033574	GO:0033574	response to testosterone	1/374	18/23328	0.252503371	0.3502	0.21062	Adh1	1
GO:0033604	GO:0033604	negative regulation of catecholamine secretion	1/374	18/23328	0.252503371	0.3502	0.21062	Ptgs1	1
GO:0042407	GO:0042407	cristae formation	1/374	18/23328	0.252503371	0.3502	0.21062	Chchd10	1
GO:0042487	GO:0042487	regulation of odontogenesis of dentin-containing tooth	1/374	18/23328	0.252503371	0.3502	0.21062	Bmp4	1
GO:0042659	GO:0042659	regulation of cell fate specification	1/374	18/23328	0.252503371	0.3502	0.21062	Sfrp2	1
GO:0043252	GO:0043252	sodium-independent organic anion transport	1/374	18/23328	0.252503371	0.3502	0.21062	Slco2a1	1
GO:0044406	GO:0044406	adhesion of symbiont to host	1/374	18/23328	0.252503371	0.3502	0.21062	Scarb1	1
GO:0045779	GO:0045779	negative regulation of bone resorption	1/374	18/23328	0.252503371	0.3502	0.21062	Cd38	1
GO:0050872	GO:0050872	white fat cell differentiation	1/374	18/23328	0.252503371	0.3502	0.21062	Fabp4	1
GO:0051131	GO:0051131	chaperone-mediated protein complex assembly	1/374	18/23328	0.252503371	0.3502	0.21062	Clu	1
GO:0051580	GO:0051580	regulation of neurotransmitter uptake	1/374	18/23328	0.252503371	0.3502	0.21062	Itgb1	1
GO:0060841	GO:0060841	venous blood vessel development	1/374	18/23328	0.252503371	0.3502	0.21062	Prox1	1
GO:0060947	GO:0060947	cardiac vascular smooth muscle cell differentiation	1/374	18/23328	0.252503371	0.3502	0.21062	Pdgfb	1
GO:0061158	GO:0061158	3'-UTR-mediated mRNA destabilization	1/374	18/23328	0.252503371	0.3502	0.21062	Zfp36	1
GO:0071696	GO:0071696	ectodermal placode development	1/374	18/23328	0.252503371	0.3502	0.21062	Prox1	1
GO:0071801	GO:0071801	regulation of podosome assembly	1/374	18/23328	0.252503371	0.3502	0.21062	Fscn1	1
GO:0072077	GO:0072077	renal vesicle morphogenesis	1/374	18/23328	0.252503371	0.3502	0.21062	Bmp4	1
GO:0097091	GO:0097091	synaptic vesicle clustering	1/374	18/23328	0.252503371	0.3502	0.21062	Pcdh17	1
GO:0098856	GO:0098856	intestinal lipid absorption	1/374	18/23328	0.252503371	0.3502	0.21062	Cd36	1
GO:1901673	GO:1901673	regulation of mitotic spindle assembly	1/374	18/23328	0.252503371	0.3502	0.21062	Hspa1a	1
GO:2000696	GO:2000696	regulation of epithelial cell differentiation involved in ki	1/374	18/23328	0.252503371	0.3502	0.21062	Gata3	1
GO:0021549	GO:0021549	cerebellum development	3/374	109/2332٤	0.254648156	0.3527	0.21213	Prox1/Hsp	3
GO:0035249	GO:0035249	synaptic transmission, glutamatergic	3/374	109/2332٤	0.254648156	0.3527	0.21213	Reln/Serp	3
GO:0051209	GO:0051209	release of sequestered calcium ion into cytosol	3/374	109/2332٤	0.254648156	0.3527	0.21213	Aplnr/F2r/	3
GO:1905477	GO:1905477	positive regulation of protein localization to membrane	3/374	109/2332٤	0.254648156	0.3527	0.21213	Itgb1/Prnp	3
GO:0031641	GO:0031641	regulation of myelination	2/374	61/23328	0.256043433	0.35416	0.213	Klk8/Ptn	2
GO:0032615	GO:0032615	interleukin-12 production	2/374	61/23328	0.256043433	0.35416	0.213	Cd36/Thb	2
GO:0042698	GO:0042698	ovulation cycle	2/374	61/23328	0.256043433	0.35416	0.213	Nos3/Ptn	2
GO:0060119	GO:0060119	inner ear receptor cell development	2/374	61/23328	0.256043433	0.35416	0.213	Cthrc1/He	2
GO:0010324	GO:0010324	membrane invagination	5/374	213/2332٤	0.25717605	0.35561	0.21388	Cd36/Lbp/	5
GO:0006275	GO:0006275	regulation of DNA replication	3/374	110/2332٤	0.258959222	0.3576	0.21507	Cst3/Pdgf	3

GO:0008344	GO:0008344	adult locomotory behavior	3/374	110/23328	0.258959222	0.3576	0.21507	Cxcl12/Id2	3
GO:0035967	GO:0035967	cellular response to topologically incorrect protein	3/374	110/23328	0.258959222	0.3576	0.21507	Hspa5/Hs	3
GO:0051283	GO:0051283	negative regulation of sequestering of calcium ion	3/374	110/23328	0.258959222	0.3576	0.21507	Aplnr/F2r/	3
GO:0140014	GO:0140014	mitotic nuclear division	6/374	268/23328	0.261128458	0.35906	0.21595	Bmp4/Rgc	6
GO:0019080	GO:0019080	viral gene expression	2/374	62/23328	0.261996287	0.35906	0.21595	Zfp36/Jun	2
GO:0032613	GO:0032613	interleukin-10 production	2/374	62/23328	0.261996287	0.35906	0.21595	Isg15/Cd3	2
GO:0050982	GO:0050982	detection of mechanical stimulus	2/374	62/23328	0.261996287	0.35906	0.21595	Cxcl12/Se	2
GO:0051926	GO:0051926	negative regulation of calcium ion transport	2/374	62/23328	0.261996287	0.35906	0.21595	Nos3/Hes	2
GO:1903078	GO:1903078	positive regulation of protein localization to plasma me	2/374	62/23328	0.261996287	0.35906	0.21595	Itgb1/Prnp	2
GO:0006865	GO:0006865	amino acid transport	4/374	162/23328	0.262357043	0.35906	0.21595	Rgs4/Itgb	4
GO:0010675	GO:0010675	regulation of cellular carbohydrate metabolic process	4/374	162/23328	0.262357043	0.35906	0.21595	Igfbp3/Nu	4
GO:0007249	GO:0007249	I-kappaB kinase/NF-kappaB signaling	5/374	215/23328	0.263230149	0.35906	0.21595	Cd36/Hsp	5
GO:0030316	GO:0030316	osteoclast differentiation	3/374	111/23328	0.26327996	0.35906	0.21595	Sfrp1/Fos	3
GO:1904659	GO:1904659	glucose transmembrane transport	3/374	111/23328	0.26327996	0.35906	0.21595	Fabp5/C3	3
GO:0051052	GO:0051052	regulation of DNA metabolic process	8/374	379/23328	0.264444656	0.35906	0.21595	Gata3/Igfb	8
GO:0002031	GO:0002031	G protein-coupled receptor internalization	1/374	19/23328	0.264496667	0.35906	0.21595	Adm	1
GO:0002524	GO:0002524	hypersensitivity	1/374	19/23328	0.264496667	0.35906	0.21595	C3	1
GO:0002922	GO:0002922	positive regulation of humoral immune response	1/374	19/23328	0.264496667	0.35906	0.21595	C3	1
GO:0006027	GO:0006027	glycosaminoglycan catabolic process	1/374	19/23328	0.264496667	0.35906	0.21595	Lyve1	1
GO:0006309	GO:0006309	apoptotic DNA fragmentation	1/374	19/23328	0.264496667	0.35906	0.21595	Igfbp3	1
GO:0006590	GO:0006590	thyroid hormone generation	1/374	19/23328	0.264496667	0.35906	0.21595	Gata3	1
GO:0006656	GO:0006656	phosphatidylcholine biosynthetic process	1/374	19/23328	0.264496667	0.35906	0.21595	Fabp5	1
GO:0009200	GO:0009200	deoxyribonucleoside triphosphate metabolic process	1/374	19/23328	0.264496667	0.35906	0.21595	Cmpk2	1
GO:0010566	GO:0010566	regulation of ketone biosynthetic process	1/374	19/23328	0.264496667	0.35906	0.21595	Dkk3	1
GO:0010623	GO:0010623	programmed cell death involved in cell development	1/374	19/23328	0.264496667	0.35906	0.21595	Kitl	1
GO:0010766	GO:0010766	negative regulation of sodium ion transport	1/374	19/23328	0.264496667	0.35906	0.21595	Serpine2	1
GO:0014061	GO:0014061	regulation of norepinephrine secretion	1/374	19/23328	0.264496667	0.35906	0.21595	Ptgs1	1
GO:0030050	GO:0030050	vesicle transport along actin filament	1/374	19/23328	0.264496667	0.35906	0.21595	Myo1b	1
GO:0032274	GO:0032274	gonadotropin secretion	1/374	19/23328	0.264496667	0.35906	0.21595	Gja1	1
GO:0032352	GO:0032352	positive regulation of hormone metabolic process	1/374	19/23328	0.264496667	0.35906	0.21595	Gata3	1
GO:0032634	GO:0032634	interleukin-5 production	1/374	19/23328	0.264496667	0.35906	0.21595	Gata3	1
GO:0032656	GO:0032656	regulation of interleukin-13 production	1/374	19/23328	0.264496667	0.35906	0.21595	Gata3	1
GO:0032700	GO:0032700	negative regulation of interleukin-17 production	1/374	19/23328	0.264496667	0.35906	0.21595	Prnp	1
GO:0039531	GO:0039531	regulation of viral-induced cytoplasmic pattern recogni	1/374	19/23328	0.264496667	0.35906	0.21595	Tspan6	1
GO:0043652	GO:0043652	engulfment of apoptotic cell	1/374	19/23328	0.264496667	0.35906	0.21595	Thbs1	1
GO:0045540	GO:0045540	regulation of cholesterol biosynthetic process	1/374	19/23328	0.264496667	0.35906	0.21595	Apoe	1
GO:0045837	GO:0045837	negative regulation of membrane potential	1/374	19/23328	0.264496667	0.35906	0.21595	Ptn	1
GO:0046068	GO:0046068	cGMP metabolic process	1/374	19/23328	0.264496667	0.35906	0.21595	Pde2a	1
GO:0048243	GO:0048243	norepinephrine secretion	1/374	19/23328	0.264496667	0.35906	0.21595	Ptgs1	1
GO:0048484	GO:0048484	enteric nervous system development	1/374	19/23328	0.264496667	0.35906	0.21595	Ednrb	1
GO:0050774	GO:0050774	negative regulation of dendrite morphogenesis	1/374	19/23328	0.264496667	0.35906	0.21595	Id1	1
GO:0050884	GO:0050884	neuromuscular process controlling posture	1/374	19/23328	0.264496667	0.35906	0.21595	Tcf15	1
GO:0051238	GO:0051238	sequestering of metal ion	1/374	19/23328	0.264496667	0.35906	0.21595	Lcn2	1
GO:0051349	GO:0051349	positive regulation of lyase activity	1/374	19/23328	0.264496667	0.35906	0.21595	Nos3	1
GO:0060044	GO:0060044	negative regulation of cardiac muscle cell proliferation	1/374	19/23328	0.264496667	0.35906	0.21595	Gja1	1
GO:0060544	GO:0060544	regulation of necroptotic process	1/374	19/23328	0.264496667	0.35906	0.21595	Bok	1
GO:0062098	GO:0062098	regulation of programmed necrotic cell death	1/374	19/23328	0.264496667	0.35906	0.21595	Bok	1
GO:0071605	GO:0071605	monocyte chemotactic protein-1 production	1/374	19/23328	0.264496667	0.35906	0.21595	Cd24a	1
GO:0071637	GO:0071637	regulation of monocyte chemotactic protein-1 productic	1/374	19/23328	0.264496667	0.35906	0.21595	Cd24a	1
GO:0097623	GO:0097623	potassium ion export across plasma membrane	1/374	19/23328	0.264496667	0.35906	0.21595	Kcnn4	1
GO:0106118	GO:0106118	regulation of sterol biosynthetic process	1/374	19/23328	0.264496667	0.35906	0.21595	Apoe	1
GO:0150146	GO:0150146	cell junction disassembly	1/374	19/23328	0.264496667	0.35906	0.21595	C3	1
GO:1900451	GO:1900451	positive regulation of glutamate receptor signaling path	1/374	19/23328	0.264496667	0.35906	0.21595	Reln	1
GO:2000251	GO:2000251	positive regulation of actin cytoskeleton reorganization	1/374	19/23328	0.264496667	0.35906	0.21595	Tek	1
GO:2000269	GO:2000269	regulation of fibroblast apoptotic process	1/374	19/23328	0.264496667	0.35906	0.21595	Sfrp1	1
GO:0016042	GO:0016042	lipid catabolic process	7/374	324/23328	0.264892497	0.35948	0.21621	Scarb1/Mg	7
GO:0006941	GO:0006941	striated muscle contraction	4/374	163/23328	0.265884169	0.36071	0.21694	Gja5/Atp1	4
GO:0050864	GO:0050864	regulation of B cell activation	6/374	270/23328	0.26653019	0.36147	0.2174	Sfrp1/Cd3	6
GO:0008645	GO:0008645	hexose transmembrane transport	3/374	112/23328	0.267609667	0.36258	0.21807	Fabp5/C3	3
GO:0038127	GO:0038127	ERBB signaling pathway	3/374	112/23328	0.267609667	0.36258	0.21807	Ptprr/Cdh	3
GO:0051282	GO:0051282	regulation of sequestering of calcium ion	3/374	112/23328	0.267609667	0.36258	0.21807	Aplnr/F2r/	3
GO:0001947	GO:0001947	heart looping	2/374	63/23328	0.267949989	0.36269	0.21813	Hand2/Gja	2
GO:0006661	GO:0006661	phosphatidylinositol biosynthetic process	2/374	63/23328	0.267949989	0.36269	0.21813	Pdgfa/Pdg	2
GO:0060999	GO:0060999	positive regulation of dendritic spine development	2/374	63/23328	0.267949989	0.36269	0.21813	Apoe/Reln	2
GO:0007623	GO:0007623	circadian rhythm	5/374	217/23328	0.26931686	0.36442	0.21917	Prox1/Ass	5
GO:0046887	GO:0046887	positive regulation of hormone secretion	4/374	164/23328	0.269420036	0.36444	0.21919	Cd38/Sox	4
GO:0051208	GO:0051208	sequestering of calcium ion	3/374	113/23328	0.271947648	0.36774	0.22117	Aplnr/F2r/	3
GO:0009064	GO:0009064	glutamine family amino acid metabolic process	2/374	64/23328	0.273902984	0.36813	0.22141	Ass1/Nos	2
GO:0051784	GO:0051784	negative regulation of nuclear division	2/374	64/23328	0.273902984	0.36813	0.22141	Bmp4/Rgc	2
GO:0070988	GO:0070988	demethylation	2/374	64/23328	0.273902984	0.36813	0.22141	Gata3/Apc	2
GO:0090278	GO:0090278	negative regulation of peptide hormone secretion	2/374	64/23328	0.273902984	0.36813	0.22141	Sfrp1/Ucp	2
GO:0009101	GO:0009101	glycoprotein biosynthetic process	6/374	273/23328	0.274685235	0.36813	0.22141	Cytl1/Gxyl	6
GO:0015749	GO:0015749	monosaccharide transmembrane transport	3/374	114/23328	0.276293216	0.36813	0.22141	Fabp5/C3	3
GO:0043401	GO:0043401	steroid hormone mediated signaling pathway	3/374	114/23328	0.276293216	0.36813	0.22141	Sfrp1/Bmp	3
GO:0071346	GO:0071346	cellular response to interferon-gamma	3/374	114/23328	0.276293216	0.36813	0.22141	Vim/Gapd	3
GO:1903076	GO:1903076	regulation of protein localization to plasma membrane	3/374	114/23328	0.276293216	0.36813	0.22141	Itgb1/Actb	3
GO:0002755	GO:0002755	MyD88-dependent toll-like receptor signaling pathway	1/374	20/23328	0.276298042	0.36813	0.22141	Irf7	1
GO:0006699	GO:0006699	bile acid biosynthetic process	1/374	20/23328	0.276298042	0.36813	0.22141	Prox1	1
GO:0010528	GO:0010528	regulation of transposition	1/374	20/23328	0.276298042	0.36813	0.22141	Apobec3	1
GO:0010529	GO:0010529	negative regulation of transposition	1/374	20/23328	0.276298042	0.36813	0.22141	Apobec3	1
GO:0010888	GO:0010888	negative regulation of lipid storage	1/374	20/23328	0.276298042	0.36813	0.22141	Nfkbia	1
GO:0010984	GO:0010984	regulation of lipoprotein particle clearance	1/374	20/23328	0.276298042	0.36813	0.22141	Gpihbp1	1
GO:0021535	GO:0021535	cell migration in hindbrain	1/374	20/23328	0.276298042	0.36813	0.22141	Itgb1	1
GO:0030220	GO:0030220	platelet formation	1/374	20/23328	0.276298042	0.36813	0.22141	Actn1	1
GO:0030810	GO:0030810	positive regulation of nucleotide biosynthetic process	1/374	20/23328	0.276298042	0.36813	0.22141	Nos3	1
GO:0030852	GO:0030852	regulation of granulocyte differentiation	1/374	20/23328	0.276298042	0.36813	0.22141	Rbp1	1
GO:0030889	GO:0030889	negative regulation of B cell proliferation	1/374	20/23328	0.276298042	0.36813	0.22141	Cd24a	1
GO:0032310	GO:0032310	prostaglandin secretion	1/374	20/23328	0.276298042	0.36813	0.22141	Edn1	1
GO:0032695	GO:0032695	negative regulation of interleukin-12 production	1/374	20/23328	0.276298042	0.36813	0.22141	Thbs1	1
GO:0033033	GO:0033033	negative regulation of myeloid cell apoptotic process	1/374	20/23328	0.276298042	0.36813	0.22141	Kitl	1
GO:0033599	GO:0033599	regulation of mammary gland epithelial cell proliferatio	1/374	20/23328	0.276298042	0.36813	0.22141	Gata3	1
GO:0034143	GO:0034143	regulation of toll-like receptor 4 signaling pathway	1/374	20/23328	0.276298042	0.36813	0.22141	Lbp	1
GO:0035970	GO:0035970	peptidyl-threonine dephosphorylation	1/374	20/23328	0.276298042	0.36813	0.22141	Dusp1	1
GO:0045217	GO:0045217	cell-cell junction maintenance	1/374	20/23328	0.276298042	0.36813	0.22141	F2r	1
GO:0045649	GO:0045649	regulation of macrophage differentiation	1/374	20/23328	0.276298042	0.36813	0.22141	Id2	1
GO:0045986	GO:0045986	negative regulation of smooth muscle contraction	1/374	20/23328	0.276298042	0.36813	0.22141	Calcrl	1
GO:0046629	GO:0046629	gamma-delta T cell activation	1/374	20/23328	0.276298042	0.36813	0.22141	Sox4	1
GO:0048245	GO:0048245	eosinophil chemotaxis	1/374	20/23328	0.276298042	0.36813	0.22141	Ccl21a	1
GO:0048557	GO:0048557	embryonic digestive tract morphogenesis	1/374	20/23328	0.276298042	0.36813	0.22141	Id2	1
GO:0050965	GO:0050965	detection of temperature stimulus involved in sensory p	1/374	20/23328	0.276298042	0.36813	0.22141	Cxcl12	1
GO:0051608	GO:0051608	histamine transport	1/374	20/23328	0.276298042	0.36813	0.22141	Edn1	1
GO:0060252	GO:0060252	positive regulation of glial cell proliferation	1/374	20/23328	0.276298042	0.36813	0.22141	Vim	1
GO:0060547	GO:0060547	negative regulation of necrotic cell death	1/374	20/23328	0.276298042	0.36813	0.22141	Bok	1
GO:0070935	GO:0070935	3'-UTR-mediated mRNA stabilization	1/374	20/23328	0.276298042	0.36813	0.22141	Zfp36	1
GO:0072079	GO:0072079	nephron tubule formation	1/374	20/23328	0.276298042	0.36813	0.22141	Gata3	1
GO:0072087	GO:0072087	renal vesicle development	1/374	20/23328	0.276298042	0.36813	0.22141	Bmp4	1
GO:0090030	GO:0090030	regulation of steroid hormone biosynthetic process	1/374	20/23328	0.276298042	0.36813	0.22141	Dkk3	1
GO:1900373	GO:1900373	positive regulation of purine nucleotide biosynthetic pr	1/374	20/23328	0.276298042	0.36813	0.22141	Nos3	1
GO:1900746	GO:1900746	regulation of vascular endothelial growth factor signali	1/374	20/23328	0.276298042	0.36813	0.22141	Dcn	1
GO:1903909	GO:1903909	regulation of receptor clustering	1/374	20/23328	0.276298042	0.36813	0.22141	Ptn	1
GO:1990182	GO:1990182	exosomal secretion	1/374	20/23328	0.276298042	0.36813	0.22141	Sdc1	1
GO:2000678	GO:2000678	negative regulation of transcription regulatory region D	1/374	20/23328	0.276298042	0.36813	0.22141	Id2	1
GO:2000773	GO:2000773	negative regulation of cellular senescence	1/374	20/23328	0.276298042	0.36813	0.22141	Plk2	1
GO:0002429	GO:0002429	immune response-activating cell surface receptor sign	7/374	330/23328	0.279738074	0.37251	0.22404	Bmx/Kcnr	7
GO:0015807	GO:0015807	L-amino acid transport	2/374	65/23328	0.27985377	0.37251	0.22404	Rgs4/Itgb	2
GO:0044774	GO:0044774	mitotic DNA integrity checkpoint	2/374	65/23328	0.27985377	0.37251	0.22404	Sox4/Cdk	2
GO:0016050	GO:0016050	vesicle organization	6/374	275/23328	0.280154882	0.37279	0.22421	Pla2g5/Sc	6
GO:0034219	GO:0034219	carbohydrate transmembrane transport	3/374	115/23328	0.280645693	0.37321	0.22446	Fabp5/C3	3
GO:0048259	GO:0048259	regulation of receptor-mediated endocytosis	3/374	115/23328	0.280645693	0.37321	0.22446	Clu/Sh3gl	3
GO:0010948	GO:0010948	negative regulation of cell cycle process	5/374	221/23328	0.281580211	0.37433	0.22514	Bmp4/Fhl	5
GO:0002757	GO:0002757	immune response-activating signal transduction	7/374	331/23328	0.28223282	0.37508	0.22559	Bmx/Kcnr	7
GO:0042472	GO:0042472	inner ear morphogenesis	3/374	116/23328	0.285004411	0.37864	0.22773	Gata3/Cth	3
GO:0050433	GO:0050433	regulation of catecholamine secretion	2/374	66/23328	0.285800891	0.37864	0.22773	Cxcl12/Pt	2
GO:0070972	GO:0070972	protein localization to endoplasmic reticulum	2/374	66/23328	0.285800891	0.37864	0.22773	Hspa5/Gja	2
GO:0051259	GO:0051259	protein complex oligomerization	5/374	223/23328	0.287752917	0.37864	0.22773	Steap4/Eh	5
GO:0003417	GO:0003417	growth plate cartilage development	1/374	21/23328	0.287910558	0.37864	0.22773	Thbs1	1
GO:0008210	GO:0008210	estrogen metabolic process	1/374	21/23328	0.287910558	0.37864	0.22773	Cyp1a1	1
GO:0009110	GO:0009110	vitamin biosynthetic process	1/374	21/23328	0.287910558	0.37864	0.22773	Pltp	1
GO:0010869	GO:0010869	regulation of receptor biosynthetic process	1/374	21/23328	0.287910558	0.37864	0.22773	Edn1	1

GO:0032305	GO:0032305	positive regulation of icosanoid secretion	1/374	21/23328	0.287910558	0.37864	0.22773	Edn1	1
GO:0033605	GO:0033605	positive regulation of catecholamine secretion	1/374	21/23328	0.287910558	0.37864	0.22773	Cxcl12	1
GO:0033630	GO:0033630	positive regulation of cell adhesion mediated by integri	1/374	21/23328	0.287910558	0.37864	0.22773	Cd24a	1
GO:0034312	GO:0034312	diol biosynthetic process	1/374	21/23328	0.287910558	0.37864	0.22773	Acer2	1
GO:0034315	GO:0034315	regulation of Arp2/3 complex-mediated actin nucleatio	1/374	21/23328	0.287910558	0.37864	0.22773	Gmfg	1
GO:0034643	GO:0034643	establishment of mitochondrion localization, microtubu	1/374	21/23328	0.287910558	0.37864	0.22773	Fez1	1
GO:0036344	GO:0036344	platelet morphogenesis	1/374	21/23328	0.287910558	0.37864	0.22773	Actn1	1
GO:0042053	GO:0042053	regulation of dopamine metabolic process	1/374	21/23328	0.287910558	0.37864	0.22773	Epas1	1
GO:0042219	GO:0042219	cellular modified amino acid catabolic process	1/374	21/23328	0.287910558	0.37864	0.22773	Dpep1	1
GO:0044346	GO:0044346	fibroblast apoptotic process	1/374	21/23328	0.287910558	0.37864	0.22773	Sfrp1	1
GO:0045475	GO:0045475	locomotor rhythm	1/374	21/23328	0.287910558	0.37864	0.22773	Id2	1
GO:0046716	GO:0046716	muscle cell cellular homeostasis	1/374	21/23328	0.287910558	0.37864	0.22773	Lox	1
GO:0047497	GO:0047497	mitochondrion transport along microtubule	1/374	21/23328	0.287910558	0.37864	0.22773	Fez1	1
GO:0050434	GO:0050434	positive regulation of viral transcription	1/374	21/23328	0.287910558	0.37864	0.22773	Jun	1
GO:0050650	GO:0050650	chondroitin sulfate proteoglycan biosynthetic process	1/374	21/23328	0.287910558	0.37864	0.22773	Cytl1	1
GO:0050966	GO:0050966	detection of mechanical stimulus involved in sensory p	1/374	21/23328	0.287910558	0.37864	0.22773	Cxcl12	1
GO:0051956	GO:0051956	negative regulation of amino acid transport	1/374	21/23328	0.287910558	0.37864	0.22773	Rgs4	1
GO:0060055	GO:0060055	angiogenesis involved in wound healing	1/374	21/23328	0.287910558	0.37864	0.22773	Kdr	1
GO:0060456	GO:0060456	positive regulation of digestive system process	1/374	21/23328	0.287910558	0.37864	0.22773	Aqp1	1
GO:0070230	GO:0070230	positive regulation of lymphocyte apoptotic process	1/374	21/23328	0.287910558	0.37864	0.22773	Cd24a	1
GO:0090026	GO:0090026	positive regulation of monocyte chemotaxis	1/374	21/23328	0.287910558	0.37864	0.22773	Cxcl12	1
GO:0097107	GO:0097107	postsynaptic density assembly	1/374	21/23328	0.287910558	0.37864	0.22773	Reln	1
GO:0099515	GO:0099515	actin filament-based transport	1/374	21/23328	0.287910558	0.37864	0.22773	Myo1b	1
GO:1902254	GO:1902254	negative regulation of intrinsic apoptotic signaling path	1/374	21/23328	0.287910558	0.37864	0.22773	Cd74	1
GO:2001224	GO:2001224	positive regulation of neuron migration	1/374	21/23328	0.287910558	0.37864	0.22773	Reln	1
GO:0002286	GO:0002286	T cell activation involved in immune response	3/374	117/23328	0.28936871	0.37996	0.22852	Gata3/Fgl	3
GO:0006612	GO:0006612	protein targeting to membrane	3/374	117/23328	0.28936871	0.37996	0.22852	Prnp/Hspa	3
GO:0032414	GO:0032414	positive regulation of ion transmembrane transporter a	3/374	117/23328	0.28936871	0.37996	0.22852	Bmp4/Atp	3
GO:0045621	GO:0045621	positive regulation of lymphocyte differentiation	3/374	117/23328	0.28936871	0.37996	0.22852	Gata3/Sox	3
GO:0048814	GO:0048814	regulation of dendrite morphogenesis	3/374	117/23328	0.28936871	0.37996	0.22852	Id1/Reln/S	3
GO:0033209	GO:0033209	tumor necrosis factor-mediated signaling pathway	2/374	67/23328	0.291742938	0.38272	0.23018	Krt18/Nfkl	2
GO:0060425	GO:0060425	lung morphogenesis	2/374	67/23328	0.291742938	0.38272	0.23018	Bmp4/Id1	2
GO:2000756	GO:2000756	regulation of peptidyl-lysine acetylation	2/374	67/23328	0.291742938	0.38272	0.23018	Gata3/Sox	2
GO:0043434	GO:0043434	response to peptide hormone	7/374	336/23328	0.294786612	0.38659	0.23251	Ednrb/C1c	7
GO:0030968	GO:0030968	endoplasmic reticulum unfolded protein response	2/374	68/23328	0.297678549	0.38804	0.23338	Hspa5/Bo	2
GO:2000378	GO:2000378	negative regulation of reactive oxygen species metabo	2/374	68/23328	0.297678549	0.38804	0.23338	Ptgis/Cd3	2
GO:0000187	GO:0000187	activation of MAPK activity	3/374	119/23328	0.298111454	0.38804	0.23338	Thbs1/Igfb	3
GO:0000413	GO:0000413	protein peptidyl-prolyl isomerization	1/374	22/23328	0.299337231	0.38804	0.23338	Ppic	1
GO:0001573	GO:0001573	ganglioside metabolic process	1/374	22/23328	0.299337231	0.38804	0.23338	Gm2a	1
GO:0003323	GO:0003323	type B pancreatic cell development	1/374	22/23328	0.299337231	0.38804	0.23338	Bmp4	1
GO:0006144	GO:0006144	purine nucleobase metabolic process	1/374	22/23328	0.299337231	0.38804	0.23338	Gda	1
GO:0006221	GO:0006221	pyrimidine nucleotide biosynthetic process	1/374	22/23328	0.299337231	0.38804	0.23338	Cmpk2	1
GO:0006516	GO:0006516	glycoprotein catabolic process	1/374	22/23328	0.299337231	0.38804	0.23338	Cst3	1
GO:0010666	GO:0010666	positive regulation of cardiac muscle cell apoptotic pro	1/374	22/23328	0.299337231	0.38804	0.23338	Igfbp3	1
GO:0014850	GO:0014850	response to muscle activity	1/374	22/23328	0.299337231	0.38804	0.23338	Metrnl	1
GO:0015813	GO:0015813	L-glutamate transmembrane transport	1/374	22/23328	0.299337231	0.38804	0.23338	Itgb1	1
GO:0031649	GO:0031649	heat generation	1/374	22/23328	0.299337231	0.38804	0.23338	Ednrb	1
GO:0032148	GO:0032148	activation of protein kinase B activity	1/374	22/23328	0.299337231	0.38804	0.23338	Pdgfb	1
GO:0032616	GO:0032616	interleukin-13 production	1/374	22/23328	0.299337231	0.38804	0.23338	Gata3	1
GO:0034377	GO:0034377	plasma lipoprotein particle assembly	1/374	22/23328	0.299337231	0.38804	0.23338	Apoe	1
GO:0035634	GO:0035634	response to stilbenoid	1/374	22/23328	0.299337231	0.38804	0.23338	Cd36	1
GO:0042107	GO:0042107	cytokine metabolic process	1/374	22/23328	0.299337231	0.38804	0.23338	Gata3	1
GO:0043651	GO:0043651	linoleic acid metabolic process	1/374	22/23328	0.299337231	0.38804	0.23338	Ephx2	1
GO:0044273	GO:0044273	sulfur compound catabolic process	1/374	22/23328	0.299337231	0.38804	0.23338	Dpep1	1
GO:0045683	GO:0045683	negative regulation of epidermis development	1/374	22/23328	0.299337231	0.38804	0.23338	Hes1	1
GO:0046641	GO:0046641	positive regulation of alpha-beta T cell proliferation	1/374	22/23328	0.299337231	0.38804	0.23338	Cd24a	1
GO:0048261	GO:0048261	negative regulation of receptor-mediated endocytosis	1/374	22/23328	0.299337231	0.38804	0.23338	Sh3gl3	1
GO:0048339	GO:0048339	paraxial mesoderm development	1/374	22/23328	0.299337231	0.38804	0.23338	Tcf15	1
GO:0051220	GO:0051220	cytoplasmic sequestering of protein	1/374	22/23328	0.299337231	0.38804	0.23338	Nfkbia	1
GO:0051602	GO:0051602	response to electrical stimulus	1/374	22/23328	0.299337231	0.38804	0.23338	Actb	1
GO:0060438	GO:0060438	trachea development	1/374	22/23328	0.299337231	0.38804	0.23338	Bmp4	1
GO:0060602	GO:0060602	branch elongation of an epithelium	1/374	22/23328	0.299337231	0.38804	0.23338	Bmp4	1
GO:0060749	GO:0060749	mammary gland alveolus development	1/374	22/23328	0.299337231	0.38804	0.23338	Id2	1
GO:0061377	GO:0061377	mammary gland lobule development	1/374	22/23328	0.299337231	0.38804	0.23338	Id2	1
GO:0070242	GO:0070242	thymocyte apoptotic process	1/374	22/23328	0.299337231	0.38804	0.23338	Bmp4	1
GO:0071800	GO:0071800	podosome assembly	1/374	22/23328	0.299337231	0.38804	0.23338	Fscn1	1
GO:1900101	GO:1900101	regulation of endoplasmic reticulum unfolded protein re	1/374	22/23328	0.299337231	0.38804	0.23338	Bok	1
GO:1902307	GO:1902307	positive regulation of sodium ion transmembrane trans	1/374	22/23328	0.299337231	0.38804	0.23338	Atp1b1	1
GO:1902547	GO:1902547	regulation of cellular response to vascular endothelial	1/374	22/23328	0.299337231	0.38804	0.23338	Dcn	1
GO:1902644	GO:1902644	tertiary alcohol metabolic process	1/374	22/23328	0.299337231	0.38804	0.23338	Dkk3	1
GO:1904996	GO:1904996	positive regulation of leukocyte adhesion to vascular e	1/374	22/23328	0.299337231	0.38804	0.23338	Selp	1
GO:0071230	GO:0071230	cellular response to amino acid stimulus	4/374	173/23328	0.301573215	0.39082	0.23505	Col5a2/Mı	4
GO:0016239	GO:0016239	positive regulation of macroautophagy	2/374	69/23328	0.303606404	0.39292	0.23631	Kdr/Dcn	2
GO:0034394	GO:0034394	protein localization to cell surface	2/374	69/23328	0.303606404	0.39292	0.23631	Gpihbp1/F	2
GO:0061371	GO:0061371	determination of heart left/right asymmetry	2/374	69/23328	0.303606404	0.39292	0.23631	Hand2/Gja	2
GO:1904377	GO:1904377	positive regulation of protein localization to cell periphe	2/374	69/23328	0.303606404	0.39292	0.23631	Itgb1/Prnp	2
GO:0072521	GO:0072521	purine-containing compound metabolic process	8/374	396/23328	0.303666246	0.39292	0.23631	Nupr1/Nos	8
GO:0006643	GO:0006643	membrane lipid metabolic process	4/374	174/23328	0.305175635	0.39463	0.23734	Gm2a/Plp	4
GO:1901654	GO:1901654	response to ketone	4/374	174/23328	0.305175635	0.39463	0.23734	Bmp4/Adh	4
GO:0046700	GO:0046700	heterocycle catabolic process	8/374	397/23328	0.306015118	0.39559	0.23792	Igfbp3/Btg	8
GO:0030073	GO:0030073	insulin secretion	5/374	229/23328	0.306411791	0.39598	0.23815	Sfrp1/Cd3	5
GO:0022037	GO:0022037	metencephalon development	3/374	121/23328	0.306868825	0.39621	0.23829	Prox1/Hsp	3
GO:0050829	GO:0050829	defense response to Gram-negative bacterium	3/374	121/23328	0.306868825	0.39621	0.23829	Lbp/Adm/	3
GO:0002366	GO:0002366	leukocyte activation involved in immune response	6/374	285/23328	0.307846804	0.39621	0.23829	Gata3/Lbp	6
GO:0006305	GO:0006305	DNA alkylation	2/374	70/23328	0.309525231	0.39621	0.23829	Meg3/H19	2
GO:0006306	GO:0006306	DNA methylation	2/374	70/23328	0.309525231	0.39621	0.23829	Meg3/H19	2
GO:0006766	GO:0006766	vitamin metabolic process	2/374	70/23328	0.309525231	0.39621	0.23829	Rbp1/Pltp	2
GO:0007405	GO:0007405	neuroblast proliferation	2/374	70/23328	0.309525231	0.39621	0.23829	Cd24a/Ptr	2
GO:0019369	GO:0019369	arachidonic acid metabolic process	2/374	70/23328	0.309525231	0.39621	0.23829	Mgll/Ptgs1	2
GO:0030512	GO:0030512	negative regulation of transforming growth factor beta	2/374	70/23328	0.309525231	0.39621	0.23829	Htra1/Hsp	2
GO:0032922	GO:0032922	circadian regulation of gene expression	2/374	70/23328	0.309525231	0.39621	0.23829	Id1/Id2	2
GO:0055117	GO:0055117	regulation of cardiac muscle contraction	2/374	70/23328	0.309525231	0.39621	0.23829	Gja5/Atp1	2
GO:0002768	GO:0002768	immune response-regulating cell surface receptor sign	7/374	342/23328	0.310010271	0.39621	0.23829	Bmx/Kcnr	7
GO:0002313	GO:0002313	mature B cell differentiation involved in immune respo	1/374	23/23328	0.310581025	0.39621	0.23829	Itm2a	1
GO:0002834	GO:0002834	regulation of response to tumor cell	1/374	23/23328	0.310581025	0.39621	0.23829	Cd24a	1
GO:0002837	GO:0002837	regulation of immune response to tumor cell	1/374	23/23328	0.310581025	0.39621	0.23829	Cd24a	1
GO:0006907	GO:0006907	pinocytosis	1/374	23/23328	0.310581025	0.39621	0.23829	Ehd4	1
GO:0009065	GO:0009065	glutamine family amino acid catabolic process	1/374	23/23328	0.310581025	0.39621	0.23829	Nos3	1
GO:0010663	GO:0010663	positive regulation of striated muscle cell apoptotic pro	1/374	23/23328	0.310581025	0.39621	0.23829	Igfbp3	1
GO:0010996	GO:0010996	response to auditory stimulus	1/374	23/23328	0.310581025	0.39621	0.23829	Ptn	1
GO:0015812	GO:0015812	gamma-aminobutyric acid transport	1/374	23/23328	0.310581025	0.39621	0.23829	Slc6a6	1
GO:0032196	GO:0032196	transposition	1/374	23/23328	0.310581025	0.39621	0.23829	Apobec3	1
GO:0032288	GO:0032288	myelin assembly	1/374	23/23328	0.310581025	0.39621	0.23829	Itgb4	1
GO:0032727	GO:0032727	positive regulation of interferon-alpha production	1/374	23/23328	0.310581025	0.39621	0.23829	Irf7	1
GO:0032930	GO:0032930	positive regulation of superoxide anion generation	1/374	23/23328	0.310581025	0.39621	0.23829	Cxcl1	1
GO:0035278	GO:0035278	miRNA mediated inhibition of translation	1/374	23/23328	0.310581025	0.39621	0.23829	Zfp36	1
GO:0036003	GO:0036003	positive regulation of transcription from RNA polymera	1/374	23/23328	0.310581025	0.39621	0.23829	Klf2	1
GO:0042069	GO:0042069	regulation of catecholamine metabolic process	1/374	23/23328	0.310581025	0.39621	0.23829	Epas1	1
GO:0042537	GO:0042537	benzene-containing compound metabolic process	1/374	23/23328	0.310581025	0.39621	0.23829	Cyp4b1	1
GO:0043501	GO:0043501	skeletal muscle adaptation	1/374	23/23328	0.310581025	0.39621	0.23829	Igfbp5	1
GO:0045026	GO:0045026	plasma membrane fusion	1/374	23/23328	0.310581025	0.39621	0.23829	Tie1	1
GO:0045606	GO:0045606	positive regulation of epidermal cell differentiation	1/374	23/23328	0.310581025	0.39621	0.23829	Bmp4	1
GO:0046599	GO:0046599	regulation of centriole replication	1/374	23/23328	0.310581025	0.39621	0.23829	Plk2	1
GO:0050857	GO:0050857	positive regulation of antigen receptor-mediated signal	1/374	23/23328	0.310581025	0.39621	0.23829	Kcnn4	1
GO:0050961	GO:0050961	detection of temperature stimulus involved in sensory	1/374	23/23328	0.310581025	0.39621	0.23829	Cxcl12	1
GO:0070734	GO:0070734	histone H3-K27 methylation	1/374	23/23328	0.310581025	0.39621	0.23829	Gata3	1
GO:0071467	GO:0071467	cellular response to pH	1/374	23/23328	0.310581025	0.39621	0.23829	Gja1	1
GO:0090190	GO:0090190	positive regulation of branching involved in ureteric bu	1/374	23/23328	0.310581025	0.39621	0.23829	Bmp4	1
GO:0097186	GO:0097186	amelogenesis	1/374	23/23328	0.310581025	0.39621	0.23829	Perp	1
GO:0098703	GO:0098703	calcium ion import across plasma membrane	1/374	23/23328	0.310581025	0.39621	0.23829	Prnp	1
GO:0101023	GO:0101023	vascular endothelial cell proliferation	1/374	23/23328	0.310581025	0.39621	0.23829	Flt1	1
GO:1905562	GO:1905562	regulation of vascular endothelial cell proliferation	1/374	23/23328	0.310581025	0.39621	0.23829	Flt1	1
GO:2000345	GO:2000345	regulation of hepatocyte proliferation	1/374	23/23328	0.310581025	0.39621	0.23829	Ptn	1
GO:0050909	GO:0050909	sensory perception of taste	2/374	71/23328	0.315433798	0.40215	0.24187	Cd36/Rtp4	2
GO:0055081	GO:0055081	anion homeostasis	2/374	71/23328	0.315433798	0.40215	0.24187	Apoe/Pthl	2
GO:0008286	GO:0008286	insulin receptor signaling pathway	3/374	123/23328	0.315635868	0.40229	0.24195	Ahsg/Socs	3
GO:0002764	GO:0002764	immune response-regulating signaling pathway	7/374	345/23328	0.31767908	0.40477	0.24344	Bmx/Kcnr	7
GO:0090150	GO:0090150	establishment of protein localization to membrane	5/374	233/23328	0.318949137	0.40626	0.24434	Krt18/Prnp	5
GO:0002263	GO:0002263	cell activation involved in immune response	6/374	289/23328	0.319058379	0.40628	0.24435	Gata3/Lbp	6

GO:0032411	GO:0032411	positive regulation of transporter activity	3/374	124/23328	0.320021507	0.40684	0.24469	Bmp4/Atp	3
GO:0090277	GO:0090277	positive regulation of peptide hormone secretion	3/374	124/23328	0.320021507	0.40684	0.24469	Cd38/Sox	3
GO:1903845	GO:1903845	negative regulation of cellular response to transforming	2/374	72/23328	0.321330914	0.40684	0.24469	Htra1/Hsp	2
GO:0001829	GO:0001829	trophectodermal cell differentiation	1/374	24/23328	0.321644861	0.40684	0.24469	Junb	1
GO:0002864	GO:0002864	regulation of acute inflammatory response to antigenic	1/374	24/23328	0.321644861	0.40684	0.24469	C3	1
GO:0002903	GO:0002903	negative regulation of B cell apoptotic process	1/374	24/23328	0.321644861	0.40684	0.24469	Cd74	1
GO:0003181	GO:0003181	atrioventricular valve morphogenesis	1/374	24/23328	0.321644861	0.40684	0.24469	Sox4	1
GO:0008090	GO:0008090	retrograde axonal transport	1/374	24/23328	0.321644861	0.40684	0.24469	Dst	1
GO:0022010	GO:0022010	central nervous system myelination	1/374	24/23328	0.321644861	0.40684	0.24469	Clu	1
GO:0030859	GO:0030859	polarized epithelial cell differentiation	1/374	24/23328	0.321644861	0.40684	0.24469	Tcf15	1
GO:0032291	GO:0032291	axon ensheathment in central nervous system	1/374	24/23328	0.321644861	0.40684	0.24469	Clu	1
GO:0034505	GO:0034505	tooth mineralization	1/374	24/23328	0.321644861	0.40684	0.24469	Col1a1	1
GO:0040033	GO:0040033	negative regulation of translation, ncRNA-mediated	1/374	24/23328	0.321644861	0.40684	0.24469	Zfp36	1
GO:0042474	GO:0042474	middle ear morphogenesis	1/374	24/23328	0.321644861	0.40684	0.24469	Edn1	1
GO:0043586	GO:0043586	tongue development	1/374	24/23328	0.321644861	0.40684	0.24469	Hand2	1
GO:0045974	GO:0045974	regulation of translation, ncRNA-mediated	1/374	24/23328	0.321644861	0.40684	0.24469	Zfp36	1
GO:0046823	GO:0046823	negative regulation of nucleocytoplasmic transport	1/374	24/23328	0.321644861	0.40684	0.24469	Cd36	1
GO:0051457	GO:0051457	maintenance of protein location in nucleus	1/374	24/23328	0.321644861	0.40684	0.24469	Chchd10	1
GO:0051654	GO:0051654	establishment of mitochondrion localization	1/374	24/23328	0.321644861	0.40684	0.24469	Fez1	1
GO:0071392	GO:0071392	cellular response to estradiol stimulus	1/374	24/23328	0.321644861	0.40684	0.24469	Mmp2	1
GO:0072677	GO:0072677	eosinophil migration	1/374	24/23328	0.321644861	0.40684	0.24469	Ccl21a	1
GO:1902656	GO:1902656	calcium ion import into cytosol	1/374	24/23328	0.321644861	0.40684	0.24469	Prnp	1
GO:0043123	GO:0043123	positive regulation of I-kappaB kinase/NF-kappaB sign	3/374	125/23328	0.324407773	0.41021	0.24671	Cd36/Tgm	3
GO:0090276	GO:0090276	regulation of peptide hormone secretion	5/374	235/23328	0.325241563	0.41114	0.24727	Sfrp1/Cd3	5
GO:0000075	GO:0000075	cell cycle checkpoint	4/374	180/23328	0.326874801	0.41308	0.24844	Prox1/Sox	4
GO:0002886	GO:0002886	regulation of myeloid leukocyte mediated immunity	2/374	73/23328	0.327215429	0.41338	0.24862	Cxcl1/C3	2
GO:0046822	GO:0046822	regulation of nucleocytoplasmic transport	3/374	126/23328	0.328794086	0.41525	0.24974	Bmp4/Cd3	3
GO:0002418	GO:0002418	immune response to tumor cell	1/374	25/23328	0.332531611	0.41669	0.25061	Cd24a	1
GO:0007095	GO:0007095	mitotic G2 DNA damage checkpoint	1/374	25/23328	0.332531611	0.41669	0.25061	Cdkn1a	1
GO:0007274	GO:0007274	neuromuscular synaptic transmission	1/374	25/23328	0.332531611	0.41669	0.25061	Cd24a	1
GO:0007413	GO:0007413	axonal fasciculation	1/374	25/23328	0.332531611	0.41669	0.25061	Ncam1	1
GO:0008211	GO:0008211	glucocorticoid metabolic process	1/374	25/23328	0.332531611	0.41669	0.25061	Dkk3	1
GO:0009394	GO:0009394	2'-deoxyribonucleotide metabolic process	1/374	25/23328	0.332531611	0.41669	0.25061	Cmpk2	1
GO:0010640	GO:0010640	regulation of platelet-derived growth factor receptor sig	1/374	25/23328	0.332531611	0.41669	0.25061	Lox	1
GO:0010759	GO:0010759	positive regulation of macrophage chemotaxis	1/374	25/23328	0.332531611	0.41669	0.25061	Thbs1	1
GO:0010954	GO:0010954	positive regulation of protein processing	1/374	25/23328	0.332531611	0.41669	0.25061	Spon1	1
GO:0018195	GO:0018195	peptidyl-arginine modification	1/374	25/23328	0.332531611	0.41669	0.25061	Art3	1
GO:0019068	GO:0019068	virion assembly	1/374	25/23328	0.332531611	0.41669	0.25061	Apoe	1
GO:0019692	GO:0019692	deoxyribose phosphate metabolic process	1/374	25/23328	0.332531611	0.41669	0.25061	Cmpk2	1
GO:0032753	GO:0032753	positive regulation of interleukin-4 production	1/374	25/23328	0.332531611	0.41669	0.25061	Gata3	1
GO:0034114	GO:0034114	regulation of heterotypic cell-cell adhesion	1/374	25/23328	0.332531611	0.41669	0.25061	Thy1	1
GO:0044003	GO:0044003	modulation by symbiont of host process	1/374	25/23328	0.332531611	0.41669	0.25061	Gapdh	1
GO:0046426	GO:0046426	negative regulation of receptor signaling pathway via J	1/374	25/23328	0.332531611	0.41669	0.25061	Socs3	1
GO:0046471	GO:0046471	phosphatidylglycerol metabolic process	1/374	25/23328	0.332531611	0.41669	0.25061	Pla2g5	1
GO:0060004	GO:0060004	reflex	1/374	25/23328	0.332531611	0.41669	0.25061	Gja1	1
GO:0060216	GO:0060216	definitive hemopoiesis	1/374	25/23328	0.332531611	0.41669	0.25061	Mfap5	1
GO:0072528	GO:0072528	pyrimidine-containing compound biosynthetic process	1/374	25/23328	0.332531611	0.41669	0.25061	Cmpk2	1
GO:0086019	GO:0086019	cell-cell signaling involved in cardiac conduction	1/374	25/23328	0.332531611	0.41669	0.25061	Gja5	1
GO:0090169	GO:0090169	regulation of spindle assembly	1/374	25/23328	0.332531611	0.41669	0.25061	Hspa1a	1
GO:0106030	GO:0106030	neuron projection fasciculation	1/374	25/23328	0.332531611	0.41669	0.25061	Ncam1	1
GO:1903798	GO:1903798	regulation of production of miRNAs involved in gene si	1/374	25/23328	0.332531611	0.41669	0.25061	Bmp4	1
GO:2000193	GO:2000193	positive regulation of fatty acid transport	1/374	25/23328	0.332531611	0.41669	0.25061	Edn1	1
GO:2000737	GO:2000737	negative regulation of stem cell differentiation	1/374	25/23328	0.332531611	0.41669	0.25061	Hes1	1
GO:0002292	GO:0002292	T cell differentiation involved in immune response	2/374	74/23328	0.333086235	0.41687	0.25072	Gata3/Fgl	2
GO:0009060	GO:0009060	aerobic respiration	2/374	74/23328	0.333086235	0.41687	0.25072	Cox4i2/Cb	2
GO:0034404	GO:0034404	nucleobase-containing small molecule biosynthetic pro	2/374	74/23328	0.333086235	0.41687	0.25072	Nudt4/Pde	2
GO:0014902	GO:0014902	myotube differentiation	3/374	127/23328	0.333179877	0.41687	0.25072	Cxcl12/Cx	3
GO:0060996	GO:0060996	dendritic spine development	3/374	127/23328	0.333179877	0.41687	0.25072	Apoe/Plk2	3
GO:0022412	GO:0022412	cellular process involved in reproduction in multicellula	9/374	468/23328	0.337172192	0.42174	0.25365	Sfrp1/Bmp	9
GO:0045727	GO:0045727	positive regulation of translation	3/374	128/23328	0.337564588	0.42198	0.25379	Thbs1/Sox	3
GO:0061013	GO:0061013	regulation of mRNA catabolic process	3/374	128/23328	0.337564588	0.42198	0.25379	Btg2/Vim/	3
GO:0048747	GO:0048747	muscle fiber development	2/374	75/23328	0.338942259	0.42358	0.25475	Bmp4/Lox	2
GO:0006163	GO:0006163	purine nucleotide metabolic process	7/374	354/23328	0.340871962	0.425	0.25561	Nupr1/Nos	7
GO:0000737	GO:0000737	DNA catabolic process, endonucleolytic	1/374	26/23328	0.343244102	0.425	0.25561	Igfbp3	1
GO:0001780	GO:0001780	neutrophil homeostasis	1/374	26/23328	0.343244102	0.425	0.25561	Cfh	1
GO:0003084	GO:0003084	positive regulation of systemic arterial blood pressure	1/374	26/23328	0.343244102	0.425	0.25561	Nr2f2	1
GO:0007289	GO:0007289	spermatid nucleus differentiation	1/374	26/23328	0.343244102	0.425	0.25561	Hmgb2	1
GO:0010829	GO:0010829	negative regulation of glucose transmembrane transpo	1/374	26/23328	0.343244102	0.425	0.25561	Fabp5	1
GO:0031100	GO:0031100	animal organ regeneration	1/374	26/23328	0.343244102	0.425	0.25561	Cxcl12	1
GO:0032303	GO:0032303	regulation of icosanoid secretion	1/374	26/23328	0.343244102	0.425	0.25561	Edn1	1
GO:0032800	GO:0032800	receptor biosynthetic process	1/374	26/23328	0.343244102	0.425	0.25561	Edn1	1
GO:0032928	GO:0032928	regulation of superoxide anion generation	1/374	26/23328	0.343244102	0.425	0.25561	Cxcl1	1
GO:0042104	GO:0042104	positive regulation of activated T cell proliferation	1/374	26/23328	0.343244102	0.425	0.25561	Cd24a	1
GO:0045723	GO:0045723	positive regulation of fatty acid biosynthetic process	1/374	26/23328	0.343244102	0.425	0.25561	Cd74	1
GO:0046339	GO:0046339	diacylglycerol metabolic process	1/374	26/23328	0.343244102	0.425	0.25561	Plpp1	1
GO:0051788	GO:0051788	response to misfolded protein	1/374	26/23328	0.343244102	0.425	0.25561	Clu	1
GO:0060338	GO:0060338	regulation of type I interferon-mediated signaling pathw	1/374	26/23328	0.343244102	0.425	0.25561	Irf7	1
GO:0060765	GO:0060765	regulation of androgen receptor signaling pathway	1/374	26/23328	0.343244102	0.425	0.25561	Sfrp1	1
GO:0065005	GO:0065005	protein-lipid complex assembly	1/374	26/23328	0.343244102	0.425	0.25561	Apoe	1
GO:0070233	GO:0070233	negative regulation of T cell apoptotic process	1/374	26/23328	0.343244102	0.425	0.25561	Bmp4	1
GO:0070584	GO:0070584	mitochondrion morphogenesis	1/374	26/23328	0.343244102	0.425	0.25561	Fez1	1
GO:0070920	GO:0070920	regulation of production of small RNA involved in gene	1/374	26/23328	0.343244102	0.425	0.25561	Bmp4	1
GO:0071474	GO:0071474	cellular hyperosmotic response	1/374	26/23328	0.343244102	0.425	0.25561	Aqp1	1
GO:0090189	GO:0090189	regulation of branching involved in ureteric bud morpho	1/374	26/23328	0.343244102	0.425	0.25561	Bmp4	1
GO:0090344	GO:0090344	negative regulation of cell aging	1/374	26/23328	0.343244102	0.425	0.25561	Plk2	1
GO:0090382	GO:0090382	phagosome maturation	1/374	26/23328	0.343244102	0.425	0.25561	Pla2g5	1
GO:0098901	GO:0098901	regulation of cardiac muscle cell action potential	1/374	26/23328	0.343244102	0.425	0.25561	Gja5	1
GO:0120033	GO:0120033	negative regulation of plasma membrane bounded cell	1/374	26/23328	0.343244102	0.425	0.25561	Kank1	1
GO:0140058	GO:0140058	neuron projection arborization	1/374	26/23328	0.343244102	0.425	0.25561	Ptn	1
GO:1901380	GO:1901380	negative regulation of potassium ion transmembrane t	1/374	26/23328	0.343244102	0.425	0.25561	Rgs4	1
GO:1902175	GO:1902175	regulation of oxidative stress-induced intrinsic apoptoti	1/374	26/23328	0.343244102	0.425	0.25561	Hspb1	1
GO:1904893	GO:1904893	negative regulation of receptor signaling pathway via S	1/374	26/23328	0.343244102	0.425	0.25561	Socs3	1
GO:2000311	GO:2000311	regulation of AMPA receptor activity	1/374	26/23328	0.343244102	0.425	0.25561	Reln	1
GO:0050806	GO:0050806	positive regulation of synaptic transmission	7/374	355/23328	0.343463464	0.42515	0.2557	Apoe/Rgs	7
GO:0045670	GO:0045670	regulation of osteoclast differentiation	2/374	76/23328	0.344782468	0.42665	0.2566	Sfrp1/Fos	2
GO:0009749	GO:0009749	response to glucose	4/374	185/23328	0.345029983	0.42671	0.25664	Vcam1/Th	4
GO:0043122	GO:0043122	regulation of I-kappaB kinase/NF-kappaB signaling	4/374	185/23328	0.345029983	0.42671	0.25664	Cd36/Hsp	4
GO:0048285	GO:0048285	organelle fission	9/374	472/23328	0.346150339	0.42781	0.2573	Bmp4/Kdr	9
GO:0007631	GO:0007631	feeding behavior	3/374	130/23328	0.346328571	0.42781	0.2573	Cd36/Tcf1	3
GO:0008203	GO:0008203	cholesterol metabolic process	3/374	130/23328	0.346328571	0.42781	0.2573	Scarb1/Ep	3
GO:0042177	GO:0042177	negative regulation of protein catabolic process	3/374	130/23328	0.346328571	0.42781	0.2573	Timp4/Cst	3
GO:0046328	GO:0046328	regulation of JNK cascade	4/374	186/23328	0.348664874	0.43056	0.25895	Sfrp1/Sfrp	4
GO:0030433	GO:0030433	ubiquitin-dependent ERAD pathway	2/374	77/23328	0.350605866	0.43257	0.26016	Hspa5/Hs	2
GO:0031397	GO:0031397	negative regulation of protein ubiquitination	2/374	77/23328	0.350605866	0.43257	0.26016	Sox4/Isg1	2
GO:0007093	GO:0007093	mitotic cell cycle checkpoint	3/374	131/23328	0.35070677	0.43257	0.26016	Sox4/Plk2	3
GO:0007368	GO:0007368	determination of left/right symmetry	3/374	131/23328	0.35070677	0.43257	0.26016	Pcsk6/Ha	3
GO:0043200	GO:0043200	response to amino acid	4/374	187/23328	0.352300286	0.4335	0.26072	Col5a2/Mı	4
GO:0002068	GO:0002068	glandular epithelial cell development	1/374	27/23328	0.353785116	0.4335	0.26072	Bmp4	1
GO:0016048	GO:0016048	detection of temperature stimulus	1/374	27/23328	0.353785116	0.4335	0.26072	Cxcl12	1
GO:0021511	GO:0021511	spinal cord patterning	1/374	27/23328	0.353785116	0.4335	0.26072	Reln	1
GO:0021871	GO:0021871	forebrain regionalization	1/374	27/23328	0.353785116	0.4335	0.26072	Bmp4	1
GO:0030970	GO:0030970	retrograde protein transport, ER to cytosol	1/374	27/23328	0.353785116	0.4335	0.26072	Hsp90b1	1
GO:0032069	GO:0032069	regulation of nuclease activity	1/374	27/23328	0.353785116	0.4335	0.26072	Hmgb2	1
GO:0032528	GO:0032528	microvillus organization	1/374	27/23328	0.353785116	0.4335	0.26072	Fscn1	1
GO:0039528	GO:0039528	cytoplasmic pattern recognition receptor signaling path	1/374	27/23328	0.353785116	0.4335	0.26072	Tspan6	1
GO:0042908	GO:0042908	xenobiotic transport	1/374	27/23328	0.353785116	0.4335	0.26072	Slc6a6	1
GO:0045780	GO:0045780	positive regulation of bone resorption	1/374	27/23328	0.353785116	0.4335	0.26072	Ahsg	1
GO:0046852	GO:0046852	positive regulation of bone remodeling	1/374	27/23328	0.353785116	0.4335	0.26072	Ahsg	1
GO:0046885	GO:0046885	regulation of hormone biosynthetic process	1/374	27/23328	0.353785116	0.4335	0.26072	Dkk3	1
GO:0048799	GO:0048799	animal organ maturation	1/374	27/23328	0.353785116	0.4335	0.26072	Gata3	1
GO:0050849	GO:0050849	negative regulation of calcium-mediated signaling	1/374	27/23328	0.353785116	0.4335	0.26072	Prnp	1
GO:0061003	GO:0061003	positive regulation of dendritic spine morphogenesis	1/374	27/23328	0.353785116	0.4335	0.26072	Reln	1
GO:0071472	GO:0071472	cellular response to salt stress	1/374	27/23328	0.353785116	0.4335	0.26072	Aqp1	1
GO:0071677	GO:0071677	positive regulation of mononuclear cell migration	1/374	27/23328	0.353785116	0.4335	0.26072	Cxcl12	1
GO:1900120	GO:1900120	regulation of receptor binding	1/374	27/23328	0.353785116	0.4335	0.26072	Lox	1
GO:1901018	GO:1901018	positive regulation of potassium ion transmembrane tra	1/374	27/23328	0.353785116	0.4335	0.26072	Atp1b1	1
GO:1903513	GO:1903513	endoplasmic reticulum to cytosol transport	1/374	27/23328	0.353785116	0.4335	0.26072	Hsp90b1	1
GO:1905939	GO:1905939	regulation of gonad development	1/374	27/23328	0.353785116	0.4335	0.26072	Nupr1	1
GO:0007281	GO:0007281	germ cell development	7/374	359/23328	0.353852638	0.4335	0.26072	Bmp4/Pde	7

GO:0034763	GO:0034763	negative regulation of transmembrane transport	3/374	132/23328	0.35508174	0.43488	0.26155	Thbs1/Rg	3
GO:0009746	GO:0009746	response to hexose	4/374	188/23328	0.355935896	0.4358	0.2621	Vcam1/Th	4
GO:0060563	GO:0060563	neuroepithelial cell differentiation	2/374	78/23328	0.356411491	0.436	0.26222	Sox4/Hes	2
GO:0070373	GO:0070373	negative regulation of ERK1 and ERK2 cascade	2/374	78/23328	0.356411491	0.436	0.26222	Dusp1/Ptp	2
GO:1901983	GO:1901983	regulation of protein acetylation	2/374	78/23328	0.356411491	0.436	0.26222	Gata3/Sox	2
GO:0002440	GO:0002440	production of molecular mediator of immune response	7/374	361/23328	0.359059385	0.43911	0.26409	Gata3/Cd	7
GO:0034284	GO:0034284	response to monosaccharide	4/374	189/23328	0.359571388	0.43961	0.26439	Vcam1/Th	4
GO:0002260	GO:0002260	lymphocyte homeostasis	2/374	79/23328	0.362198418	0.44223	0.26597	Cd24a/Cd	2
GO:0045471	GO:0045471	response to ethanol	2/374	79/23328	0.362198418	0.44223	0.26597	Adh7/Adh	2
GO:0051966	GO:0051966	regulation of synaptic transmission, glutamatergic	2/374	79/23328	0.362198418	0.44223	0.26597	Reln/Serp	2
GO:0002347	GO:0002347	response to tumor cell	1/374	28/23328	0.36415739	0.44223	0.26597	Cd24a	1
GO:0006026	GO:0006026	aminoglycan catabolic process	1/374	28/23328	0.36415739	0.44223	0.26597	Lyve1	1
GO:0010738	GO:0010738	regulation of protein kinase A signaling	1/374	28/23328	0.36415739	0.44223	0.26597	Akap12	1
GO:0031365	GO:0031365	N-terminal protein amino acid modification	1/374	28/23328	0.36415739	0.44223	0.26597	Sox4	1
GO:0032469	GO:0032469	endoplasmic reticulum calcium ion homeostasis	1/374	28/23328	0.36415739	0.44223	0.26597	Tgm2	1
GO:0032647	GO:0032647	regulation of interferon-alpha production	1/374	28/23328	0.36415739	0.44223	0.26597	Irf7	1
GO:0032801	GO:0032801	receptor catabolic process	1/374	28/23328	0.36415739	0.44223	0.26597	Apoe	1
GO:0042744	GO:0042744	hydrogen peroxide catabolic process	1/374	28/23328	0.36415739	0.44223	0.26597	Gpx3	1
GO:0048596	GO:0048596	embryonic camera-type eye morphogenesis	1/374	28/23328	0.36415739	0.44223	0.26597	Prox1	1
GO:0051560	GO:0051560	mitochondrial calcium ion homeostasis	1/374	28/23328	0.36415739	0.44223	0.26597	Tgm2	1
GO:0060390	GO:0060390	regulation of SMAD protein signal transduction	1/374	28/23328	0.36415739	0.44223	0.26597	Bmp4	1
GO:0061099	GO:0061099	negative regulation of protein tyrosine kinase activity	1/374	28/23328	0.36415739	0.44223	0.26597	Thy1	1
GO:0061436	GO:0061436	establishment of skin barrier	1/374	28/23328	0.36415739	0.44223	0.26597	Lsr	1
GO:0071480	GO:0071480	cellular response to gamma radiation	1/374	28/23328	0.36415739	0.44223	0.26597	Cdkn1a	1
GO:0086005	GO:0086005	ventricular cardiac muscle cell action potential	1/374	28/23328	0.36415739	0.44223	0.26597	Gja5	1
GO:0098698	GO:0098698	postsynaptic specialization assembly	1/374	28/23328	0.36415739	0.44223	0.26597	Reln	1
GO:1900273	GO:1900273	positive regulation of long-term synaptic potentiation	1/374	28/23328	0.36415739	0.44223	0.26597	Reln	1
GO:1900745	GO:1900745	positive regulation of p38MAPK cascade	1/374	28/23328	0.36415739	0.44223	0.26597	Hand2	1
GO:1904861	GO:1904861	excitatory synapse assembly	1/374	28/23328	0.36415739	0.44223	0.26597	Reln	1
GO:2000310	GO:2000310	regulation of NMDA receptor activity	1/374	28/23328	0.36415739	0.44223	0.26597	Reln	1
GO:0060968	GO:0060968	regulation of gene silencing	2/374	80/23328	0.367965754	0.44659	0.2686	Bmp4/Zfp	2
GO:2001259	GO:2001259	positive regulation of cation channel activity	2/374	80/23328	0.367965754	0.44659	0.2686	Bmp4/Rel	2
GO:1901655	GO:1901655	cellular response to ketone	3/374	135/23328	0.36818218	0.44673	0.26868	Bmp4/Aqp	3
GO:0010389	GO:0010389	regulation of G2/M transition of mitotic cell cycle	2/374	81/23328	0.373712641	0.45173	0.27168	Fhl1/Cdkn	2
GO:0043525	GO:0043525	positive regulation of neuron apoptotic process	2/374	81/23328	0.373712641	0.45173	0.27168	Prnp/Jun	2
GO:0071347	GO:0071347	cellular response to interleukin-1	2/374	81/23328	0.373712641	0.45173	0.27168	Fn1/Ccl21	2
GO:0009913	GO:0009913	epidermal cell differentiation	4/374	193/23328	0.374105928	0.45173	0.27168	Bmp4/Ptg	4
GO:0001919	GO:0001919	regulation of receptor recycling	1/374	29/23328	0.37436362	0.45173	0.27168	Ece1	1
GO:0003085	GO:0003085	negative regulation of systemic arterial blood pressure	1/374	29/23328	0.37436362	0.45173	0.27168	Cd36	1
GO:0006220	GO:0006220	pyrimidine nucleotide metabolic process	1/374	29/23328	0.37436362	0.45173	0.27168	Cmpk2	1
GO:0021952	GO:0021952	central nervous system projection neuron axonogenes	1/374	29/23328	0.37436362	0.45173	0.27168	Cdh11	1
GO:0031116	GO:0031116	positive regulation of microtubule polymerization	1/374	29/23328	0.37436362	0.45173	0.27168	Gda	1
GO:0031295	GO:0031295	T cell costimulation	1/374	29/23328	0.37436362	0.45173	0.27168	Cd24a	1
GO:0034629	GO:0034629	cellular protein-containing complex localization	1/374	29/23328	0.37436362	0.45173	0.27168	Tnfaip2	1
GO:0039694	GO:0039694	viral RNA genome replication	1/374	29/23328	0.37436362	0.45173	0.27168	Apobec3	1
GO:0045109	GO:0045109	intermediate filament organization	1/374	29/23328	0.37436362	0.45173	0.27168	Vim	1
GO:0045589	GO:0045589	regulation of regulatory T cell differentiation	1/374	29/23328	0.37436362	0.45173	0.27168	Ctla2a	1
GO:0070296	GO:0070296	sarcoplasmic reticulum calcium ion transport	1/374	29/23328	0.37436362	0.45173	0.27168	Sln	1
GO:0090314	GO:0090314	positive regulation of protein targeting to membrane	1/374	29/23328	0.37436362	0.45173	0.27168	Prnp	1
GO:1900027	GO:1900027	regulation of ruffle assembly	1/374	29/23328	0.37436362	0.45173	0.27168	Kank1	1
GO:1900078	GO:1900078	positive regulation of cellular response to insulin stimu	1/374	29/23328	0.37436362	0.45173	0.27168	C1qtnf9	1
GO:1902235	GO:1902235	regulation of endoplasmic reticulum stress-induced intr	1/374	29/23328	0.37436362	0.45173	0.27168	Bok	1
GO:0030705	GO:0030705	cytoskeleton-dependent intracellular transport	4/374	194/23328	0.377736177	0.45567	0.27405	Fez1/Hspl	4
GO:0009117	GO:0009117	nucleotide metabolic process	8/374	427/23328	0.377887278	0.45572	0.27408	Nupr1/Nos	8
GO:0032479	GO:0032479	regulation of type I interferon production	2/374	82/23328	0.379438252	0.45745	0.27513	Irf7/Hmgb	2
GO:0046165	GO:0046165	alcohol biosynthetic process	3/374	138/23328	0.381235595	0.45938	0.27628	Dkk3/Apo	3
GO:0099111	GO:0099111	microtubule-based transport	4/374	195/23328	0.381364473	0.45938	0.27628	Fez1/Hspl	4
GO:1905475	GO:1905475	regulation of protein localization to membrane	4/374	195/23328	0.381364473	0.45938	0.27628	Itgb1/Actb	4
GO:0001759	GO:0001759	organ induction	1/374	30/23328	0.384406454	0.46118	0.27737	Bmp4	1
GO:0002335	GO:0002335	mature B cell differentiation	1/374	30/23328	0.384406454	0.46118	0.27737	Itm2a	1
GO:0003309	GO:0003309	type B pancreatic cell differentiation	1/374	30/23328	0.384406454	0.46118	0.27737	Bmp4	1
GO:0009262	GO:0009262	deoxyribonucleotide metabolic process	1/374	30/23328	0.384406454	0.46118	0.27737	Cmpk2	1
GO:0010863	GO:0010863	positive regulation of phospholipase C activity	1/374	30/23328	0.384406454	0.46118	0.27737	Flt1	1
GO:0019433	GO:0019433	triglyceride catabolic process	1/374	30/23328	0.384406454	0.46118	0.27737	Lpl	1
GO:0021602	GO:0021602	cranial nerve morphogenesis	1/374	30/23328	0.384406454	0.46118	0.27737	Nrp2	1
GO:0032607	GO:0032607	interferon-alpha production	1/374	30/23328	0.384406454	0.46118	0.27737	Irf7	1
GO:0043153	GO:0043153	entrainment of circadian clock by photoperiod	1/374	30/23328	0.384406454	0.46118	0.27737	Id2	1
GO:0043457	GO:0043457	regulation of cellular respiration	1/374	30/23328	0.384406454	0.46118	0.27737	Cbfa2t3	1
GO:0045671	GO:0045671	negative regulation of osteoclast differentiation	1/374	30/23328	0.384406454	0.46118	0.27737	Sfrp1	1
GO:0048714	GO:0048714	positive regulation of oligodendrocyte differentiation	1/374	30/23328	0.384406454	0.46118	0.27737	Ptn	1
GO:0050951	GO:0050951	sensory perception of temperature stimulus	1/374	30/23328	0.384406454	0.46118	0.27737	Cxcl12	1
GO:0051957	GO:0051957	positive regulation of amino acid transport	1/374	30/23328	0.384406454	0.46118	0.27737	Itgb1	1
GO:0002532	GO:0002532	production of molecular mediator involved in inflamma	2/374	83/23328	0.385141793	0.46193	0.27782	Ephx2/Lbp	2
GO:0016125	GO:0016125	sterol metabolic process	3/374	139/23328	0.385574074	0.46218	0.27797	Scarb1/Ep	3
GO:1900180	GO:1900180	regulation of protein localization to nucleus	3/374	139/23328	0.385574074	0.46218	0.27797	Bmp4/Cd3	3
GO:0008544	GO:0008544	epidermis development	6/374	313/23328	0.38723531	0.46404	0.27909	Bmp4/Pdc	6
GO:0009855	GO:0009855	determination of bilateral symmetry	3/374	140/23328	0.389905464	0.46683	0.28077	Pcsk6/Har	3
GO:0050905	GO:0050905	neuromuscular process	3/374	140/23328	0.389905464	0.46683	0.28077	Cfh/Tcf15	3
GO:1904375	GO:1904375	regulation of protein localization to cell periphery	3/374	140/23328	0.389905464	0.46683	0.28077	Itgb1/Actb	3
GO:0006942	GO:0006942	regulation of striated muscle contraction	2/374	84/23328	0.3908225	0.46766	0.28127	Gja5/Atp1	2
GO:0061515	GO:0061515	myeloid cell development	2/374	84/23328	0.3908225	0.46766	0.28127	Adgrf5/Klf	2
GO:1902652	GO:1902652	secondary alcohol metabolic process	3/374	141/23328	0.394229323	0.46872	0.2819	Scarb1/Ep	3
GO:0007530	GO:0007530	sex determination	1/374	31/23328	0.394288504	0.46872	0.2819	Dhh	1
GO:0009648	GO:0009648	photoperiodism	1/374	31/23328	0.394288504	0.46872	0.2819	Id2	1
GO:0021533	GO:0021533	cell differentiation in hindbrain	1/374	31/23328	0.394288504	0.46872	0.2819	Prox1	1
GO:0021904	GO:0021904	dorsal/ventral neural tube patterning	1/374	31/23328	0.394288504	0.46872	0.2819	Bmp4	1
GO:0022011	GO:0022011	myelination in peripheral nervous system	1/374	31/23328	0.394288504	0.46872	0.2819	Itgb4	1
GO:0031294	GO:0031294	lymphocyte costimulation	1/374	31/23328	0.394288504	0.46872	0.2819	Cd24a	1
GO:0032292	GO:0032292	peripheral nervous system axon ensheathment	1/374	31/23328	0.394288504	0.46872	0.2819	Itgb4	1
GO:0033137	GO:0033137	negative regulation of peptidyl-serine phosphorylation	1/374	31/23328	0.394288504	0.46872	0.2819	Ddit4	1
GO:0035640	GO:0035640	exploration behavior	1/374	31/23328	0.394288504	0.46872	0.2819	Apoe	1
GO:0044030	GO:0044030	regulation of DNA methylation	1/374	31/23328	0.394288504	0.46872	0.2819	H19	1
GO:0044319	GO:0044319	wound healing, spreading of cells	1/374	31/23328	0.394288504	0.46872	0.2819	Pdpn	1
GO:0044788	GO:0044788	modulation by host of viral process	1/374	31/23328	0.394288504	0.46872	0.2819	Apoe	1
GO:0045662	GO:0045662	negative regulation of myoblast differentiation	1/374	31/23328	0.394288504	0.46872	0.2819	Bmp4	1
GO:0045932	GO:0045932	negative regulation of muscle contraction	1/374	31/23328	0.394288504	0.46872	0.2819	Calcrl	1
GO:0048011	GO:0048011	neurotrophin TRK receptor signaling pathway	1/374	31/23328	0.394288504	0.46872	0.2819	Ddit4	1
GO:0051125	GO:0051125	regulation of actin nucleation	1/374	31/23328	0.394288504	0.46872	0.2819	Gmfg	1
GO:0055075	GO:0055075	potassium ion homeostasis	1/374	31/23328	0.394288504	0.46872	0.2819	Atp1b1	1
GO:0086010	GO:0086010	membrane depolarization during action potential	1/374	31/23328	0.394288504	0.46872	0.2819	Gja5	1
GO:0090505	GO:0090505	epiboly involved in wound healing	1/374	31/23328	0.394288504	0.46872	0.2819	Pdpn	1
GO:0098810	GO:0098810	neurotransmitter reuptake	1/374	31/23328	0.394288504	0.46872	0.2819	Itgb1	1
GO:1902253	GO:1902253	regulation of intrinsic apoptotic signaling pathway by p	1/374	31/23328	0.394288504	0.46872	0.2819	Cd74	1
GO:1905523	GO:1905523	positive regulation of macrophage migration	1/374	31/23328	0.394288504	0.46872	0.2819	Thbs1	1
GO:0006835	GO:0006835	dicarboxylic acid transport	2/374	85/23328	0.396479639	0.47105	0.28331	Itgb1/Gja1	2
GO:0043367	GO:0043367	CD4-positive, alpha-beta T cell differentiation	2/374	85/23328	0.396479639	0.47105	0.28331	Gata3/Rsa	2
GO:0007626	GO:0007626	locomotory behavior	5/374	258/23328	0.398116003	0.47286	0.28439	Apoe/Cxc	5
GO:0050906	GO:0050906	detection of stimulus involved in sensory perception	3/374	142/23328	0.398545217	0.47324	0.28462	Cxcl12/Se	3
GO:0007254	GO:0007254	JNK cascade	4/374	200/23328	0.399466495	0.4742	0.2852	Sfrp1/Sfrp	4
GO:0006753	GO:0006753	nucleoside phosphate metabolic process	8/374	436/23328	0.399747742	0.47439	0.28532	Nupr1/Nos	8
GO:0006970	GO:0006970	response to osmotic stress	2/374	86/23328	0.402112506	0.47634	0.28648	Aqp1/Mylk	2
GO:0031646	GO:0031646	positive regulation of nervous system process	2/374	86/23328	0.402112506	0.47634	0.28648	Rgs4/Reln	2
GO:0032606	GO:0032606	type I interferon production	2/374	86/23328	0.402112506	0.47634	0.28648	Irf7/Hmgb	2
GO:0001556	GO:0001556	oocyte maturation	1/374	32/23328	0.404012335	0.47634	0.28648	Pde3a	1
GO:0002438	GO:0002438	acute inflammatory response to antigenic stimulus	1/374	32/23328	0.404012335	0.47634	0.28648	C3	1
GO:0007007	GO:0007007	inner mitochondrial membrane organization	1/374	32/23328	0.404012335	0.47634	0.28648	Chchd10	1
GO:0008206	GO:0008206	bile acid metabolic process	1/374	32/23328	0.404012335	0.47634	0.28648	Prox1	1
GO:0009649	GO:0009649	entrainment of circadian clock	1/374	32/23328	0.404012335	0.47634	0.28648	Id2	1
GO:0021697	GO:0021697	cerebellar cortex formation	1/374	32/23328	0.404012335	0.47634	0.28648	Prox1	1
GO:0031645	GO:0031645	negative regulation of nervous system process	1/374	32/23328	0.404012335	0.47634	0.28648	Klk8	1
GO:0032673	GO:0032673	regulation of interleukin-4 production	1/374	32/23328	0.404012335	0.47634	0.28648	Gata3	1
GO:0035066	GO:0035066	positive regulation of histone acetylation	1/374	32/23328	0.404012335	0.47634	0.28648	Gata3	1
GO:0048384	GO:0048384	retinoic acid receptor signaling pathway	1/374	32/23328	0.404012335	0.47634	0.28648	Klf2	1
GO:0050482	GO:0050482	arachidonic acid secretion	1/374	32/23328	0.404012335	0.47634	0.28648	Pla2g5	1
GO:0051569	GO:0051569	regulation of histone H3-K4 methylation	1/374	32/23328	0.404012335	0.47634	0.28648	Gata3	1
GO:0051590	GO:0051590	positive regulation of neurotransmitter transport	1/374	32/23328	0.404012335	0.47634	0.28648	Itgb1	1
GO:0060561	GO:0060561	apoptotic process involved in morphogenesis	1/374	32/23328	0.404012335	0.47634	0.28648	Hand2	1
GO:0060977	GO:0060977	coronary vasculature morphogenesis	1/374	32/23328	0.404012335	0.47634	0.28648	Hand2	1
GO:0062125	GO:0062125	regulation of mitochondrial gene expression	1/374	32/23328	0.404012335	0.47634	0.28648	Chchd10	1

GO:0071354	GO:0071354	cellular response to interleukin-6	1/374	32/23328	0.404012335	0.47634	0.28648	Selp	1
GO:0090504	GO:0090504	epiboly	1/374	32/23328	0.404012335	0.47634	0.28648	Pdpn	1
GO:1901797	GO:1901797	negative regulation of signal transduction by p53 class	1/374	32/23328	0.404012335	0.47634	0.28648	Cd74	1
GO:1903963	GO:1903963	arachidonate transport	1/374	32/23328	0.404012335	0.47634	0.28648	Pla2g5	1
GO:0010508	GO:0010508	positive regulation of autophagy	3/374	144/23328	0.407151419	0.47957	0.28843	Kdr/Plk2/I	3
GO:0015698	GO:0015698	inorganic anion transport	3/374	144/23328	0.407151419	0.47957	0.28843	Slc26a10/	3
GO:0071333	GO:0071333	cellular response to glucose stimulus	3/374	144/23328	0.407151419	0.47957	0.28843	Vcam1/Sc	3
GO:0044282	GO:0044282	small molecule catabolic process	6/374	320/23328	0.407211421	0.47957	0.28843	Adh7/Sca	6
GO:0035304	GO:0035304	regulation of protein dephosphorylation	2/374	87/23328	0.407720424	0.48003	0.28871	Cdh5/Hsp	2
GO:0002285	GO:0002285	lymphocyte activation involved in immune response	4/374	203/23328	0.410286024	0.48291	0.29044	Gata3/Itm	4
GO:0060079	GO:0060079	excitatory postsynaptic potential	2/374	88/23328	0.413302746	0.48447	0.29137	Rgs4/Reln	2
GO:1901992	GO:1901992	positive regulation of mitotic cell cycle phase transition	2/374	88/23328	0.413302746	0.48447	0.29137	Rgcc/Cyp	2
GO:1903321	GO:1903321	negative regulation of protein modification by small pro	2/374	88/23328	0.413302746	0.48447	0.29137	Sox4/Isg1	2
GO:0001779	GO:0001779	natural killer cell differentiation	1/374	33/23328	0.413580475	0.48447	0.29137	Id2	1
GO:0021884	GO:0021884	forebrain neuron development	1/374	33/23328	0.413580475	0.48447	0.29137	Nrp2	1
GO:0033081	GO:0033081	regulation of T cell differentiation in thymus	1/374	33/23328	0.413580475	0.48447	0.29137	Bmp4	1
GO:0033561	GO:0033561	regulation of water loss via skin	1/374	33/23328	0.413580475	0.48447	0.29137	Lsr	1
GO:0042119	GO:0042119	neutrophil activation	1/374	33/23328	0.413580475	0.48447	0.29137	Anxa3	1
GO:0044818	GO:0044818	mitotic G2/M transition checkpoint	1/374	33/23328	0.413580475	0.48447	0.29137	Cdkn1a	1
GO:0045066	GO:0045066	regulatory T cell differentiation	1/374	33/23328	0.413580475	0.48447	0.29137	Ctla2a	1
GO:0045616	GO:0045616	regulation of keratinocyte differentiation	1/374	33/23328	0.413580475	0.48447	0.29137	Zfp36	1
GO:0045672	GO:0045672	positive regulation of osteoclast differentiation	1/374	33/23328	0.413580475	0.48447	0.29137	Fos	1
GO:0048566	GO:0048566	embryonic digestive tract development	1/374	33/23328	0.413580475	0.48447	0.29137	Id2	1
GO:0048841	GO:0048841	regulation of axon extension involved in axon guidance	1/374	33/23328	0.413580475	0.48447	0.29137	Cxcl12	1
GO:0060487	GO:0060487	lung epithelial cell differentiation	1/374	33/23328	0.413580475	0.48447	0.29137	Klf2	1
GO:0061157	GO:0061157	mRNA destabilization	1/374	33/23328	0.413580475	0.48447	0.29137	Zfp36	1
GO:0070266	GO:0070266	necroptotic process	1/374	33/23328	0.413580475	0.48447	0.29137	Bok	1
GO:0035637	GO:0035637	multicellular organismal signaling	3/374	146/23328	0.415720776	0.4867	0.29272	Gja5/Abcc	3
GO:0071331	GO:0071331	cellular response to hexose stimulus	3/374	146/23328	0.415720776	0.4867	0.29272	Vcam1/Sc	3
GO:0016236	GO:0016236	macroautophagy	4/374	205/23328	0.417477157	0.48862	0.29387	Kdr/Fez1/	4
GO:0009150	GO:0009150	purine ribonucleotide metabolic process	6/374	324/23328	0.418606012	0.4898	0.29458	Nupr1/Cbf	6
GO:0071326	GO:0071326	cellular response to monosaccharide stimulus	3/374	147/23328	0.419990646	0.49129	0.29547	Vcam1/Sc	3
GO:0016064	GO:0016064	immunoglobulin mediated immune response	5/374	265/23328	0.42024463	0.49144	0.29557	Irf7/Fgl2/C	5
GO:0000096	GO:0000096	sulfur amino acid metabolic process	1/374	34/23328	0.42299541	0.49218	0.29601	Dpep1	1
GO:0002021	GO:0002021	response to dietary excess	1/374	34/23328	0.42299541	0.49218	0.29601	Apoe	1
GO:0003203	GO:0003203	endocardial cushion morphogenesis	1/374	34/23328	0.42299541	0.49218	0.29601	Nos3	1
GO:0014044	GO:0014044	Schwann cell development	1/374	34/23328	0.42299541	0.49218	0.29601	Itgb4	1
GO:0021591	GO:0021591	ventricular system development	1/374	34/23328	0.42299541	0.49218	0.29601	Aqp1	1
GO:0030808	GO:0030808	regulation of nucleotide biosynthetic process	1/374	34/23328	0.42299541	0.49218	0.29601	Nos3	1
GO:0033032	GO:0033032	regulation of myeloid cell apoptotic process	1/374	34/23328	0.42299541	0.49218	0.29601	Kitl	1
GO:0043950	GO:0043950	positive regulation of cAMP-mediated signaling	1/374	34/23328	0.42299541	0.49218	0.29601	Ece1	1
GO:0051180	GO:0051180	vitamin transport	1/374	34/23328	0.42299541	0.49218	0.29601	Scarb1	1
GO:0060479	GO:0060479	lung cell differentiation	1/374	34/23328	0.42299541	0.49218	0.29601	Klf2	1
GO:0062149	GO:0062149	detection of stimulus involved in sensory perception of	1/374	34/23328	0.42299541	0.49218	0.29601	Cxcl12	1
GO:0070741	GO:0070741	response to interleukin-6	1/374	34/23328	0.42299541	0.49218	0.29601	Selp	1
GO:0099068	GO:0099068	postsynapse assembly	1/374	34/23328	0.42299541	0.49218	0.29601	Reln	1
GO:0120255	GO:0120255	olefinic compound biosynthetic process	1/374	34/23328	0.42299541	0.49218	0.29601	Dkk3	1
GO:1900274	GO:1900274	regulation of phospholipase C activity	1/374	34/23328	0.42299541	0.49218	0.29601	Flt1	1
GO:1900371	GO:1900371	regulation of purine nucleotide biosynthetic process	1/374	34/23328	0.42299541	0.49218	0.29601	Nos3	1
GO:1901976	GO:1901976	regulation of cell cycle checkpoint	1/374	34/23328	0.42299541	0.49218	0.29601	Prox1	1
GO:2000108	GO:2000108	positive regulation of leukocyte apoptotic process	1/374	34/23328	0.42299541	0.49218	0.29601	Cd24a	1
GO:0001702	GO:0001702	gastrulation with mouth forming second	1/374	35/23328	0.432259586	0.49975	0.30056	Plpp3	1
GO:0006308	GO:0006308	DNA catabolic process	1/374	35/23328	0.432259586	0.49975	0.30056	Igfbp3	1
GO:0006471	GO:0006471	protein ADP-ribosylation	1/374	35/23328	0.432259586	0.49975	0.30056	Art3	1
GO:0006972	GO:0006972	hyperosmotic response	1/374	35/23328	0.432259586	0.49975	0.30056	Aqp1	1
GO:0007026	GO:0007026	negative regulation of microtubule depolymerization	1/374	35/23328	0.432259586	0.49975	0.30056	Stmn2	1
GO:0009187	GO:0009187	cyclic nucleotide metabolic process	1/374	35/23328	0.432259586	0.49975	0.30056	Pde2a	1
GO:0010758	GO:0010758	regulation of macrophage chemotaxis	1/374	35/23328	0.432259586	0.49975	0.30056	Thbs1	1
GO:0010837	GO:0010837	regulation of keratinocyte proliferation	1/374	35/23328	0.432259586	0.49975	0.30056	Zfp36	1
GO:0014047	GO:0014047	glutamate secretion	1/374	35/23328	0.432259586	0.49975	0.30056	Gja1	1
GO:0032660	GO:0032660	regulation of interleukin-17 production	1/374	35/23328	0.432259586	0.49975	0.30056	Prnp	1
GO:0035116	GO:0035116	embryonic hindlimb morphogenesis	1/374	35/23328	0.432259586	0.49975	0.30056	Bmp4	1
GO:0043516	GO:0043516	regulation of DNA damage response, signal transducti	1/374	35/23328	0.432259586	0.49975	0.30056	Cd74	1
GO:0045879	GO:0045879	negative regulation of smoothened signaling pathway	1/374	35/23328	0.432259586	0.49975	0.30056	Serpine2	1
GO:0055083	GO:0055083	monovalent inorganic anion homeostasis	1/374	35/23328	0.432259586	0.49975	0.30056	Pthlh	1
GO:0060218	GO:0060218	hematopoietic stem cell differentiation	1/374	35/23328	0.432259586	0.49975	0.30056	Sfrp1	1
GO:0060236	GO:0060236	regulation of mitotic spindle organization	1/374	35/23328	0.432259586	0.49975	0.30056	Hspa1a	1
GO:0060441	GO:0060441	epithelial tube branching involved in lung morphogene	1/374	35/23328	0.432259586	0.49975	0.30056	Bmp4	1
GO:0086091	GO:0086091	regulation of heart rate by cardiac conduction	1/374	35/23328	0.432259586	0.49975	0.30056	Gja5	1
GO:0090313	GO:0090313	regulation of protein targeting to membrane	1/374	35/23328	0.432259586	0.49975	0.30056	Prnp	1
GO:0097106	GO:0097106	postsynaptic density organization	1/374	35/23328	0.432259586	0.49975	0.30056	Reln	1
GO:1900181	GO:1900181	negative regulation of protein localization to nucleus	1/374	35/23328	0.432259586	0.49975	0.30056	Cd36	1
GO:1903429	GO:1903429	regulation of cell maturation	1/374	35/23328	0.432259586	0.49975	0.30056	Ednrb	1
GO:1905898	GO:1905898	positive regulation of response to endoplasmic reticulu	1/374	35/23328	0.432259586	0.49975	0.30056	Bok	1
GO:0019724	GO:0019724	B cell mediated immunity	5/374	269/23328	0.432829905	0.50027	0.30088	Irf7/Fgl2/C	5
GO:0000045	GO:0000045	autophagosome assembly	2/374	92/23328	0.43536403	0.50292	0.30247	Fez1/Nupr	2
GO:0002275	GO:0002275	myeloid cell activation involved in immune response	2/374	92/23328	0.43536403	0.50292	0.30247	Lbp/Anxa3	2
GO:0044270	GO:0044270	cellular nitrogen compound catabolic process	7/374	391/23328	0.437443097	0.50518	0.30383	Igfbp3/Btg	7
GO:0018105	GO:0018105	peptidyl-serine phosphorylation	6/374	331/23328	0.43847843	0.50624	0.30447	Sfrp2/Tek	6
GO:0042180	GO:0042180	cellular ketone metabolic process	4/374	211/23328	0.438922909	0.50661	0.30469	Dkk3/Prox	4
GO:0007215	GO:0007215	glutamate receptor signaling pathway	2/374	93/23328	0.440809567	0.50775	0.30538	Reln/Prnp	2
GO:0008589	GO:0008589	regulation of smoothened signaling pathway	2/374	93/23328	0.440809567	0.50775	0.30538	Sfrp1/Serp	2
GO:0006910	GO:0006910	phagocytosis, recognition	3/374	152/23328	0.441176871	0.50775	0.30538	Scarb1/Cc	3
GO:0008045	GO:0008045	motor neuron axon guidance	1/374	36/23328	0.441375411	0.50775	0.30538	Cxcl12	1
GO:0009112	GO:0009112	nucleobase metabolic process	1/374	36/23328	0.441375411	0.50775	0.30538	Gda	1
GO:0031069	GO:0031069	hair follicle morphogenesis	1/374	36/23328	0.441375411	0.50775	0.30538	Igfbp5	1
GO:0031572	GO:0031572	G2 DNA damage checkpoint	1/374	36/23328	0.441375411	0.50775	0.30538	Cdkn1a	1
GO:0035115	GO:0035115	embryonic forelimb morphogenesis	1/374	36/23328	0.441375411	0.50775	0.30538	Hoxd9	1
GO:0042220	GO:0042220	response to cocaine	1/374	36/23328	0.441375411	0.50775	0.30538	Ncam1	1
GO:0048169	GO:0048169	regulation of long-term neuronal synaptic plasticity	1/374	36/23328	0.441375411	0.50775	0.30538	Kdr	1
GO:0050779	GO:0050779	RNA destabilization	1/374	36/23328	0.441375411	0.50775	0.30538	Zfp36	1
GO:0071398	GO:0071398	cellular response to fatty acid	1/374	36/23328	0.441375411	0.50775	0.30538	Lpl	1
GO:0009408	GO:0009408	response to heat	2/374	94/23328	0.446226161	0.51291	0.30848	Hspa1a/C	2
GO:1901879	GO:1901879	regulation of protein depolymerization	2/374	94/23328	0.446226161	0.51291	0.30848	Nes/Stmn	2
GO:1903313	GO:1903313	positive regulation of mRNA metabolic process	2/374	94/23328	0.446226161	0.51291	0.30848	Btg2/Zfp3	2
GO:0009259	GO:0009259	ribonucleotide metabolic process	6/374	334/23328	0.446959185	0.51361	0.3089	Nupr1/Cbf	6
GO:0001782	GO:0001782	B cell homeostasis	1/374	37/23328	0.450345254	0.51594	0.3103	Cd74	1
GO:0002675	GO:0002675	positive regulation of acute inflammatory response	1/374	37/23328	0.450345254	0.51594	0.3103	C3	1
GO:0007099	GO:0007099	centriole replication	1/374	37/23328	0.450345254	0.51594	0.3103	Plk2	1
GO:0009066	GO:0009066	aspartate family amino acid metabolic process	1/374	37/23328	0.450345254	0.51594	0.3103	Ass1	1
GO:0009954	GO:0009954	proximal/distal pattern formation	1/374	37/23328	0.450345254	0.51594	0.3103	Hoxd9	1
GO:0014823	GO:0014823	response to activity	1/374	37/23328	0.450345254	0.51594	0.3103	Metrnl	1
GO:0021680	GO:0021680	cerebellar Purkinje cell layer development	1/374	37/23328	0.450345254	0.51594	0.3103	Hspa5	1
GO:0033144	GO:0033144	negative regulation of intracellular steroid hormone rec	1/374	37/23328	0.450345254	0.51594	0.3103	Sfrp1	1
GO:0034314	GO:0034314	Arp2/3 complex-mediated actin nucleation	1/374	37/23328	0.450345254	0.51594	0.3103	Gmfg	1
GO:0040020	GO:0040020	regulation of meiotic nuclear division	1/374	37/23328	0.450345254	0.51594	0.3103	Pde3a	1
GO:0045047	GO:0045047	protein targeting to ER	1/374	37/23328	0.450345254	0.51594	0.3103	Hspa5	1
GO:0035264	GO:0035264	multicellular organism growth	4/374	216/23328	0.456619263	0.52298	0.31454	Meg3/Klf2	4
GO:0030838	GO:0030838	positive regulation of actin filament polymerization	2/374	96/23328	0.456970656	0.52309	0.31461	Cdc42ep3	2
GO:0070555	GO:0070555	response to interleukin-1	2/374	96/23328	0.456970656	0.52309	0.31461	Fn1/Ccl21	2
GO:0008037	GO:0008037	cell recognition	5/374	277/23328	0.45781632	0.52331	0.31474	Ncam1/Sc	5
GO:0009267	GO:0009267	cellular response to starvation	3/374	156/23328	0.457909035	0.52331	0.31474	Cdkn1a/H	3
GO:0009311	GO:0009311	oligosaccharide metabolic process	1/374	38/23328	0.459171448	0.52331	0.31474	Gm2a	1
GO:0010661	GO:0010661	positive regulation of muscle cell apoptotic process	1/374	38/23328	0.459171448	0.52331	0.31474	Igfbp3	1
GO:0018208	GO:0018208	peptidyl-proline modification	1/374	38/23328	0.459171448	0.52331	0.31474	Ppic	1
GO:0031114	GO:0031114	regulation of microtubule depolymerization	1/374	38/23328	0.459171448	0.52331	0.31474	Stmn2	1
GO:0032733	GO:0032733	positive regulation of interleukin-10 production	1/374	38/23328	0.459171448	0.52331	0.31474	Cd34	1
GO:0033238	GO:0033238	regulation of cellular amine metabolic process	1/374	38/23328	0.459171448	0.52331	0.31474	Epas1	1
GO:0045684	GO:0045684	positive regulation of epidermis development	1/374	38/23328	0.459171448	0.52331	0.31474	Bmp4	1
GO:0045940	GO:0045940	positive regulation of steroid metabolic process	1/374	38/23328	0.459171448	0.52331	0.31474	Apoe	1
GO:0046640	GO:0046640	regulation of alpha-beta T cell proliferation	1/374	38/23328	0.459171448	0.52331	0.31474	Cd24a	1
GO:0051968	GO:0051968	positive regulation of synaptic transmission, glutamate	1/374	38/23328	0.459171448	0.52331	0.31474	Reln	1
GO:0071391	GO:0071391	cellular response to estrogen stimulus	1/374	38/23328	0.459171448	0.52331	0.31474	Sfrp1	1
GO:0086065	GO:0086065	cell communication involved in cardiac conduction	1/374	38/23328	0.459171448	0.52331	0.31474	Gja5	1
GO:0098534	GO:0098534	centriole assembly	1/374	38/23328	0.459171448	0.52331	0.31474	Plk2	1
GO:2000036	GO:2000036	regulation of stem cell population maintenance	1/374	38/23328	0.459171448	0.52331	0.31474	Ptn	1
GO:0062013	GO:0062013	positive regulation of small molecule metabolic proces	3/374	157/23328	0.46205922	0.5263	0.31653	Nos3/Cd7	3
GO:0019217	GO:0019217	regulation of fatty acid metabolic process	2/374	97/23328	0.462297677	0.5263	0.31653	Fabp5/Cd	2
GO:1902749	GO:1902749	regulation of cell cycle G2/M phase transition	2/374	97/23328	0.462297677	0.5263	0.31653	Fhl1/Cdkn	2

GO:1905037	GO:1905037	autophagosome organization	2/374	97/23328	0.462297677	0.5263	0.31653	Fez1/Nupr	2
GO:0050777	GO:0050777	negative regulation of immune response	3/374	158/23328	0.466195649	0.5306	0.31912	Fgl2/Serpi	3
GO:0006892	GO:0006892	post-Golgi vesicle-mediated transport	2/374	98/23328	0.467593997	0.53075	0.31921	Krt18/Myo	2
GO:0099565	GO:0099565	chemical synaptic transmission, postsynaptic	2/374	98/23328	0.467593997	0.53075	0.31921	Rgs4/Reln	2
GO:0006778	GO:0006778	porphyrin-containing compound metabolic process	1/374	39/23328	0.467856286	0.53075	0.31921	Cyp1a1	1
GO:0007212	GO:0007212	dopamine receptor signaling pathway	1/374	39/23328	0.467856286	0.53075	0.31921	Rgs4	1
GO:0009651	GO:0009651	response to salt stress	1/374	39/23328	0.467856286	0.53075	0.31921	Aqp1	1
GO:0031279	GO:0031279	regulation of cyclase activity	1/374	39/23328	0.467856286	0.53075	0.31921	Nos3	1
GO:0032735	GO:0032735	positive regulation of interleukin-12 production	1/374	39/23328	0.467856286	0.53075	0.31921	Cd36	1
GO:0032885	GO:0032885	regulation of polysaccharide biosynthetic process	1/374	39/23328	0.467856286	0.53075	0.31921	Pdgfb	1
GO:0033028	GO:0033028	myeloid cell apoptotic process	1/374	39/23328	0.467856286	0.53075	0.31921	Kitl	1
GO:0036230	GO:0036230	granulocyte activation	1/374	39/23328	0.467856286	0.53075	0.31921	Anxa3	1
GO:0090224	GO:0090224	regulation of spindle organization	1/374	39/23328	0.467856286	0.53075	0.31921	Hspa1a	1
GO:1900026	GO:1900026	positive regulation of substrate adhesion-dependent ce	1/374	39/23328	0.467856286	0.53075	0.31921	Net1	1
GO:0007569	GO:0007569	cell aging	3/374	159/23328	0.470318027	0.53339	0.3208	Plk2/Cdkn	3
GO:0006805	GO:0006805	xenobiotic metabolic process	2/374	99/23328	0.47285923	0.53598	0.32236	Cyp1a1/G	2
GO:0036503	GO:0036503	ERAD pathway	2/374	99/23328	0.47285923	0.53598	0.32236	Hspa5/Hs	2
GO:0001504	GO:0001504	neurotransmitter uptake	1/374	40/23328	0.476402026	0.53737	0.32319	Itgb1	1
GO:0001881	GO:0001881	receptor recycling	1/374	40/23328	0.476402026	0.53737	0.32319	Ece1	1
GO:0014046	GO:0014046	dopamine secretion	1/374	40/23328	0.476402026	0.53737	0.32319	Cxcl12	1
GO:0014059	GO:0014059	regulation of dopamine secretion	1/374	40/23328	0.476402026	0.53737	0.32319	Cxcl12	1
GO:0030521	GO:0030521	androgen receptor signaling pathway	1/374	40/23328	0.476402026	0.53737	0.32319	Sfrp1	1
GO:0031076	GO:0031076	embryonic camera-type eye development	1/374	40/23328	0.476402026	0.53737	0.32319	Prox1	1
GO:0032633	GO:0032633	interleukin-4 production	1/374	40/23328	0.476402026	0.53737	0.32319	Gata3	1
GO:0034105	GO:0034105	positive regulation of tissue remodeling	1/374	40/23328	0.476402026	0.53737	0.32319	Ahsg	1
GO:0035307	GO:0035307	positive regulation of protein dephosphorylation	1/374	40/23328	0.476402026	0.53737	0.32319	Cdh5	1
GO:0038179	GO:0038179	neurotrophin signaling pathway	1/374	40/23328	0.476402026	0.53737	0.32319	Ddit4	1
GO:0043001	GO:0043001	Golgi to plasma membrane protein transport	1/374	40/23328	0.476402026	0.53737	0.32319	Krt18	1
GO:0043368	GO:0043368	positive T cell selection	1/374	40/23328	0.476402026	0.53737	0.32319	Cd74	1
GO:0045981	GO:0045981	positive regulation of nucleotide metabolic process	1/374	40/23328	0.476402026	0.53737	0.32319	Nos3	1
GO:0048520	GO:0048520	positive regulation of behavior	1/374	40/23328	0.476402026	0.53737	0.32319	Gja1	1
GO:0060337	GO:0060337	type I interferon signaling pathway	1/374	40/23328	0.476402026	0.53737	0.32319	Irf7	1
GO:0071357	GO:0071357	cellular response to type I interferon	1/374	40/23328	0.476402026	0.53737	0.32319	Irf7	1
GO:0086004	GO:0086004	regulation of cardiac muscle cell contraction	1/374	40/23328	0.476402026	0.53737	0.32319	Gja5	1
GO:1900544	GO:1900544	positive regulation of purine nucleotide metabolic proc	1/374	40/23328	0.476402026	0.53737	0.32319	Nos3	1
GO:0019693	GO:0019693	ribose phosphate metabolic process	6/374	345/23328	0.477801585	0.5388	0.32405	Nupr1/Cbf	6
GO:0007601	GO:0007601	visual perception	3/374	161/23328	0.478519492	0.53932	0.32436	Cfh/Epas1	3
GO:0009755	GO:0009755	hormone-mediated signaling pathway	3/374	161/23328	0.478519492	0.53932	0.32436	Sfrp1/Bmp	3
GO:0010498	GO:0010498	proteasomal protein catabolic process	8/374	470/23328	0.481913359	0.543	0.32658	Clu/Apoe/	8
GO:0008207	GO:0008207	C21-steroid hormone metabolic process	1/374	41/23328	0.484810891	0.54479	0.32765	Dkk3	1
GO:0010737	GO:0010737	protein kinase A signaling	1/374	41/23328	0.484810891	0.54479	0.32765	Akap12	1
GO:0014037	GO:0014037	Schwann cell differentiation	1/374	41/23328	0.484810891	0.54479	0.32765	Itgb4	1
GO:0045806	GO:0045806	negative regulation of endocytosis	1/374	41/23328	0.484810891	0.54479	0.32765	Sh3gl3	1
GO:0048730	GO:0048730	epidermis morphogenesis	1/374	41/23328	0.484810891	0.54479	0.32765	Igfbp5	1
GO:0060603	GO:0060603	mammary gland duct morphogenesis	1/374	41/23328	0.484810891	0.54479	0.32765	Pthlh	1
GO:0071364	GO:0071364	cellular response to epidermal growth factor stimulus	1/374	41/23328	0.484810891	0.54479	0.32765	Zfp36	1
GO:0072599	GO:0072599	establishment of protein localization to endoplasmic re	1/374	41/23328	0.484810891	0.54479	0.32765	Hspa5	1
GO:0097300	GO:0097300	programmed necrotic cell death	1/374	41/23328	0.484810891	0.54479	0.32765	Bok	1
GO:0099084	GO:0099084	postsynaptic specialization organization	1/374	41/23328	0.484810891	0.54479	0.32765	Reln	1
GO:0001678	GO:0001678	cellular glucose homeostasis	3/374	163/23328	0.486661413	0.54672	0.32882	Vcam1/Sc	3
GO:0007006	GO:0007006	mitochondrial membrane organization	2/374	102/23328	0.488464841	0.54831	0.32977	Chchd10/I	2
GO:0035418	GO:0035418	protein localization to synapse	2/374	102/23328	0.488464841	0.54831	0.32977	Hspb1/Re	2
GO:1901989	GO:1901989	positive regulation of cell cycle phase transition	2/374	102/23328	0.488464841	0.54831	0.32977	Rgcc/Cyp	2
GO:0010970	GO:0010970	transport along microtubule	3/374	164/23328	0.490709389	0.55053	0.33111	Fez1/Hspl	3
GO:0033135	GO:0033135	regulation of peptidyl-serine phosphorylation	3/374	164/23328	0.490709389	0.55053	0.33111	Sfrp2/Tek	3
GO:0006687	GO:0006687	glycosphingolipid metabolic process	1/374	42/23328	0.493085067	0.55155	0.33172	Gm2a	1
GO:0010092	GO:0010092	specification of animal organ identity	1/374	42/23328	0.493085067	0.55155	0.33172	Bmp4	1
GO:0016601	GO:0016601	Rac protein signal transduction	1/374	42/23328	0.493085067	0.55155	0.33172	Cdh13	1
GO:0021955	GO:0021955	central nervous system neuron axonogenesis	1/374	42/23328	0.493085067	0.55155	0.33172	Cdh11	1
GO:0042417	GO:0042417	dopamine metabolic process	1/374	42/23328	0.493085067	0.55155	0.33172	Epas1	1
GO:0046633	GO:0046633	alpha-beta T cell proliferation	1/374	42/23328	0.493085067	0.55155	0.33172	Cd24a	1
GO:0051339	GO:0051339	regulation of lyase activity	1/374	42/23328	0.493085067	0.55155	0.33172	Nos3	1
GO:0072583	GO:0072583	clathrin-dependent endocytosis	1/374	42/23328	0.493085067	0.55155	0.33172	Sh3gl3	1
GO:2000772	GO:2000772	regulation of cellular senescence	1/374	42/23328	0.493085067	0.55155	0.33172	Plk2	1
GO:0006821	GO:0006821	chloride transport	2/374	103/23328	0.493602257	0.55155	0.33172	Clca3a1/F	2
GO:0007173	GO:0007173	epidermal growth factor receptor signaling pathway	2/374	103/23328	0.493602257	0.55155	0.33172	Cdh13/Efe	2
GO:0030004	GO:0030004	cellular monovalent inorganic cation homeostasis	2/374	103/23328	0.493602257	0.55155	0.33172	Atp1b1/Sg	2
GO:0046427	GO:0046427	positive regulation of receptor signaling pathway via JA	2/374	103/23328	0.493602257	0.55155	0.33172	Pecam1/H	2
GO:0048477	GO:0048477	oogenesis	2/374	103/23328	0.493602257	0.55155	0.33172	Pde3a/Ptn	2
GO:0071466	GO:0071466	cellular response to xenobiotic stimulus	2/374	103/23328	0.493602257	0.55155	0.33172	Cyp1a1/G	2
GO:0050953	GO:0050953	sensory perception of light stimulus	3/374	165/23328	0.494741707	0.55267	0.3324	Cfh/Epas1	3
GO:0034655	GO:0034655	nucleobase-containing compound catabolic process	6/374	352/23328	0.497168311	0.55524	0.33394	Igfbp3/Btg	6
GO:0019731	GO:0019731	antibacterial humoral response	2/374	104/23328	0.498706946	0.55641	0.33465	Adm/Jcha	2
GO:0034766	GO:0034766	negative regulation of ion transmembrane transport	2/374	104/23328	0.498706946	0.55641	0.33465	Thbs1/Rg	2
GO:0043488	GO:0043488	regulation of mRNA stability	2/374	104/23328	0.498706946	0.55641	0.33465	Vim/Zfp36	2
GO:0046631	GO:0046631	alpha-beta T cell activation	3/374	166/23328	0.498758122	0.55641	0.33465	Gata3/Cd2	3
GO:0032007	GO:0032007	negative regulation of TOR signaling	1/374	43/23328	0.501226705	0.55797	0.33558	Ddit4	1
GO:0032620	GO:0032620	interleukin-17 production	1/374	43/23328	0.501226705	0.55797	0.33558	Prnp	1
GO:0045197	GO:0045197	establishment or maintenance of epithelial cell apical/b	1/374	43/23328	0.501226705	0.55797	0.33558	Tcf15	1
GO:0045880	GO:0045880	positive regulation of smoothened signaling pathway	1/374	43/23328	0.501226705	0.55797	0.33558	Sfrp1	1
GO:0046854	GO:0046854	phosphatidylinositol phosphorylation	1/374	43/23328	0.501226705	0.55797	0.33558	Socs3	1
GO:0070232	GO:0070232	regulation of T cell apoptotic process	1/374	43/23328	0.501226705	0.55797	0.33558	Bmp4	1
GO:1901381	GO:1901381	positive regulation of potassium ion transmembrane tra	1/374	43/23328	0.501226705	0.55797	0.33558	Atp1b1	1
GO:1902742	GO:1902742	apoptotic process involved in development	1/374	43/23328	0.501226705	0.55797	0.33558	Hand2	1
GO:0000077	GO:0000077	DNA damage checkpoint	2/374	105/23328	0.503778634	0.56052	0.33711	Sox4/Cdk	2
GO:0046634	GO:0046634	regulation of alpha-beta T cell activation	2/374	105/23328	0.503778634	0.56052	0.33711	Gata3/Cd2	2
GO:0046425	GO:0046425	regulation of receptor signaling pathway via JAK-STAT	3/374	168/23328	0.506742313	0.56355	0.33894	Pecam1/H	3
GO:0000280	GO:0000280	nuclear division	7/374	418/23328	0.506774005	0.56355	0.33894	Bmp4/Pde	7
GO:0099504	GO:0099504	synaptic vesicle cycle	4/374	231/23328	0.508471414	0.56403	0.33923	Pcdh17/C	4
GO:0021510	GO:0021510	spinal cord development	2/374	106/23328	0.508817066	0.56403	0.33923	Sox4/Reln	2
GO:0035710	GO:0035710	CD4-positive, alpha-beta T cell activation	2/374	106/23328	0.508817066	0.56403	0.33923	Gata3/Rsa	2
GO:0000959	GO:0000959	mitochondrial RNA metabolic process	1/374	44/23328	0.509237923	0.56403	0.33923	Chchd10	1
GO:0006024	GO:0006024	glycosaminoglycan biosynthetic process	1/374	44/23328	0.509237923	0.56403	0.33923	Pdgfb	1
GO:0006890	GO:0006890	retrograde vesicle-mediated transport, Golgi to endopla	1/374	44/23328	0.509237923	0.56403	0.33923	Plpp3	1
GO:0008631	GO:0008631	intrinsic apoptotic signaling pathway in response to oxi	1/374	44/23328	0.509237923	0.56403	0.33923	Hspb1	1
GO:0021522	GO:0021522	spinal cord motor neuron differentiation	1/374	44/23328	0.509237923	0.56403	0.33923	Sox4	1
GO:0034198	GO:0034198	cellular response to amino acid starvation	1/374	44/23328	0.509237923	0.56403	0.33923	Cdkn1a	1
GO:0043403	GO:0043403	skeletal muscle tissue regeneration	1/374	44/23328	0.509237923	0.56403	0.33923	Gja1	1
GO:0045747	GO:0045747	positive regulation of Notch signaling pathway	1/374	44/23328	0.509237923	0.56403	0.33923	Hes1	1
GO:0070849	GO:0070849	response to epidermal growth factor	1/374	44/23328	0.509237923	0.56403	0.33923	Zfp36	1
GO:0086002	GO:0086002	cardiac muscle cell action potential involved in contrac	1/374	44/23328	0.509237923	0.56403	0.33923	Gja5	1
GO:1901998	GO:1901998	toxin transport	1/374	44/23328	0.509237923	0.56403	0.33923	Hspa5	1
GO:2000144	GO:2000144	positive regulation of DNA-templated transcription, initi	1/374	44/23328	0.509237923	0.56403	0.33923	Jun	1
GO:0032874	GO:0032874	positive regulation of stress-activated MAPK cascade	3/374	169/23328	0.510709637	0.56551	0.34012	Flt4/Edn1/	3
GO:0033157	GO:0033157	regulation of intracellular protein transport	4/374	232/23328	0.511851465	0.56662	0.34079	Bmp4/Cd3	4
GO:0140053	GO:0140053	mitochondrial gene expression	2/374	107/23328	0.513822003	0.5685	0.34192	Cfh/Chchc	2
GO:1904894	GO:1904894	positive regulation of receptor signaling pathway via ST	2/374	107/23328	0.513822003	0.5685	0.34192	Pecam1/H	2
GO:0010824	GO:0010824	regulation of centrosome duplication	1/374	45/23328	0.517120805	0.57079	0.34329	Plk2	1
GO:0031128	GO:0031128	developmental induction	1/374	45/23328	0.517120805	0.57079	0.34329	Bmp4	1
GO:0032527	GO:0032527	protein exit from endoplasmic reticulum	1/374	45/23328	0.517120805	0.57079	0.34329	Hsp90b1	1
GO:0035136	GO:0035136	forelimb morphogenesis	1/374	45/23328	0.517120805	0.57079	0.34329	Hoxd9	1
GO:0042149	GO:0042149	cellular response to glucose starvation	1/374	45/23328	0.517120805	0.57079	0.34329	Hspa5	1
GO:0042181	GO:0042181	ketone biosynthetic process	1/374	45/23328	0.517120805	0.57079	0.34329	Dkk3	1
GO:0045923	GO:0045923	positive regulation of fatty acid metabolic process	1/374	45/23328	0.517120805	0.57079	0.34329	Cd74	1
GO:0071300	GO:0071300	cellular response to retinoic acid	1/374	45/23328	0.517120805	0.57079	0.34329	Aqp1	1
GO:0097178	GO:0097178	ruffle assembly	1/374	45/23328	0.517120805	0.57079	0.34329	Kank1	1
GO:0070304	GO:0070304	positive regulation of stress-activated protein kinase si	3/374	171/23328	0.518593666	0.57218	0.34413	Flt4/Edn1/	3
GO:0000956	GO:0000956	nuclear-transcribed mRNA catabolic process	2/374	108/23328	0.51879322	0.57218	0.34413	Btg2/Zfp3	2
GO:0048704	GO:0048704	embryonic skeletal system morphogenesis	2/374	108/23328	0.51879322	0.57218	0.34413	Bmp4/Hox	2
GO:0006904	GO:0006904	vesicle docking involved in exocytosis	1/374	46/23328	0.5248774	0.57798	0.34761	Ncam1	1
GO:0010799	GO:0010799	regulation of peptidyl-threonine phosphorylation	1/374	46/23328	0.5248774	0.57798	0.34761	Ddit4	1
GO:0032881	GO:0032881	regulation of polysaccharide metabolic process	1/374	46/23328	0.5248774	0.57798	0.34761	Pdgfb	1
GO:0061647	GO:0061647	histone H3-K9 modification	1/374	46/23328	0.5248774	0.57798	0.34761	Gata3	1
GO:0090317	GO:0090317	negative regulation of intracellular protein transport	1/374	46/23328	0.5248774	0.57798	0.34761	Cd36	1
GO:1902667	GO:1902667	regulation of axon guidance	1/374	46/23328	0.5248774	0.57798	0.34761	Cxcl12	1
GO:0051260	GO:0051260	protein homooligomerization	3/374	173/23328	0.526408844	0.57936	0.34844	Steap4/Eh	3
GO:1904892	GO:1904892	regulation of receptor signaling pathway via STAT	3/374	173/23328	0.526408844	0.57936	0.34844	Pecam1/H	3
GO:0009410	GO:0009410	response to xenobiotic stimulus	2/374	110/23328	0.528633683	0.58165	0.34982	Cyp1a1/G	2
GO:0090407	GO:0090407	organophosphate biosynthetic process	7/374	427/23328	0.529280392	0.58221	0.35016	Pla2g5/Nc	7

GO:0007033	GO:0007033	vacuole organization	3/374	174/23328	0.530290128	0.58317	0.35073	Fez1/Nupr	3
GO:0021772	GO:0021772	olfactory bulb development	1/374	47/23328	0.532509728	0.58483	0.35174	Id2	1
GO:0033013	GO:0033013	tetrapyrrole metabolic process	1/374	47/23328	0.532509728	0.58483	0.35174	Cyp1a1	1
GO:0043029	GO:0043029	T cell homeostasis	1/374	47/23328	0.532509728	0.58483	0.35174	Cd24a	1
GO:0051646	GO:0051646	mitochondrion localization	1/374	47/23328	0.532509728	0.58483	0.35174	Fez1	1
GO:0120163	GO:0120163	negative regulation of cold-induced thermogenesis	1/374	47/23328	0.532509728	0.58483	0.35174	Id1	1
GO:2001257	GO:2001257	regulation of cation channel activity	3/374	176/23328	0.537999173	0.59071	0.35527	Bmp4/Itgb	3
GO:0007009	GO:0007009	plasma membrane organization	2/374	112/23328	0.538336975	0.59092	0.3554	Clu/S100a	2
GO:0001580	GO:0001580	detection of chemical stimulus involved in sensory per	1/374	48/23328	0.540019773	0.59184	0.35595	Rtp4	1
GO:0010862	GO:0010862	positive regulation of pathway-restricted SMAD protein	1/374	48/23328	0.540019773	0.59184	0.35595	Bmp4	1
GO:0022602	GO:0022602	ovulation cycle process	1/374	48/23328	0.540019773	0.59184	0.35595	Nos3	1
GO:0046596	GO:0046596	regulation of viral entry into host cell	1/374	48/23328	0.540019773	0.59184	0.35595	Cd74	1
GO:1903580	GO:1903580	positive regulation of ATP metabolic process	1/374	48/23328	0.540019773	0.59184	0.35595	Nupr1	1
GO:1990928	GO:1990928	response to amino acid starvation	1/374	48/23328	0.540019773	0.59184	0.35595	Cdkn1a	1
GO:0001942	GO:0001942	hair follicle development	2/374	113/23328	0.543136783	0.59494	0.35782	Pdgfa/Igfb	2
GO:0031570	GO:0031570	DNA integrity checkpoint	2/374	113/23328	0.543136783	0.59494	0.35782	Sox4/Cdk	2
GO:0006695	GO:0006695	cholesterol biosynthetic process	1/374	49/23328	0.54740949	0.59852	0.35997	Apoe	1
GO:0010332	GO:0010332	response to gamma radiation	1/374	49/23328	0.54740949	0.59852	0.35997	Cdkn1a	1
GO:0014888	GO:0014888	striated muscle adaptation	1/374	49/23328	0.54740949	0.59852	0.35997	Igfbp5	1
GO:0035196	GO:0035196	production of miRNAs involved in gene silencing by mi	1/374	49/23328	0.54740949	0.59852	0.35997	Bmp4	1
GO:0048255	GO:0048255	mRNA stabilization	1/374	49/23328	0.54740949	0.59852	0.35997	Zfp36	1
GO:0090342	GO:0090342	regulation of cell aging	1/374	49/23328	0.54740949	0.59852	0.35997	Plk2	1
GO:1902653	GO:1902653	secondary alcohol biosynthetic process	1/374	49/23328	0.54740949	0.59852	0.35997	Apoe	1
GO:0060078	GO:0060078	regulation of postsynaptic membrane potential	2/374	114/23328	0.547901847	0.5989	0.3602	Rgs4/Reln	2
GO:0006913	GO:0006913	nucleocytoplasmic transport	5/374	308/23328	0.550993334	0.60196	0.36204	Bmp4/Cd3	5
GO:0051169	GO:0051169	nuclear transport	5/374	308/23328	0.550993334	0.60196	0.36204	Bmp4/Cd3	5
GO:0030216	GO:0030216	keratinocyte differentiation	2/374	115/23328	0.552632044	0.6036	0.36302	Ptgs1/Zfp	2
GO:1901605	GO:1901605	alpha-amino acid metabolic process	3/374	180/23328	0.553198432	0.60406	0.3633	Ass1/Nos	3
GO:0002762	GO:0002762	negative regulation of myeloid leukocyte differentiation	1/374	50/23328	0.554680801	0.60488	0.3638	Sfrp1	1
GO:0021988	GO:0021988	olfactory lobe development	1/374	50/23328	0.554680801	0.60488	0.3638	Id2	1
GO:0030166	GO:0030166	proteoglycan biosynthetic process	1/374	50/23328	0.554680801	0.60488	0.3638	Cytl1	1
GO:0043370	GO:0043370	regulation of CD4-positive, alpha-beta T cell differentia	1/374	50/23328	0.554680801	0.60488	0.3638	Gata3	1
GO:1903573	GO:1903573	negative regulation of response to endoplasmic reticul	1/374	50/23328	0.554680801	0.60488	0.3638	Clu	1
GO:0007052	GO:0007052	mitotic spindle organization	2/374	116/23328	0.557327266	0.60697	0.36505	Plk2/Hspa	2
GO:0022404	GO:0022404	molting cycle process	2/374	116/23328	0.557327266	0.60697	0.36505	Pdgfa/Igfb	2
GO:0022405	GO:0022405	hair cycle process	2/374	116/23328	0.557327266	0.60697	0.36505	Pdgfa/Igfb	2
GO:0043487	GO:0043487	regulation of RNA stability	2/374	116/23328	0.557327266	0.60697	0.36505	Vim/Zfp36	2
GO:0098773	GO:0098773	skin epidermis development	2/374	116/23328	0.557327266	0.60697	0.36505	Pdgfa/Igfb	2
GO:0031050	GO:0031050	dsRNA processing	1/374	51/23328	0.561835599	0.61045	0.36714	Bmp4	1
GO:0032456	GO:0032456	endocytic recycling	1/374	51/23328	0.561835599	0.61045	0.36714	Ehd4	1
GO:0045010	GO:0045010	actin nucleation	1/374	51/23328	0.561835599	0.61045	0.36714	Gmfg	1
GO:0050913	GO:0050913	sensory perception of bitter taste	1/374	51/23328	0.561835599	0.61045	0.36714	Rtp4	1
GO:0070918	GO:0070918	production of small RNA involved in gene silencing by	1/374	51/23328	0.561835599	0.61045	0.36714	Bmp4	1
GO:0072132	GO:0072132	mesenchyme morphogenesis	1/374	51/23328	0.561835599	0.61045	0.36714	Nos3	1
GO:0072527	GO:0072527	pyrimidine-containing compound metabolic process	1/374	51/23328	0.561835599	0.61045	0.36714	Cmpk2	1
GO:0099054	GO:0099054	presynapse assembly	1/374	51/23328	0.561835599	0.61045	0.36714	Pcdh17	1
GO:2000142	GO:2000142	regulation of DNA-templated transcription, initiation	1/374	51/23328	0.561835599	0.61045	0.36714	Jun	1
GO:0000086	GO:0000086	G2/M transition of mitotic cell cycle	2/374	117/23328	0.561987414	0.61045	0.36715	Fhl1/Cdkn	2
GO:0046632	GO:0046632	alpha-beta T cell differentiation	2/374	118/23328	0.566612405	0.61532	0.37007	Gata3/Rsa	2
GO:0032388	GO:0032388	positive regulation of intracellular transport	3/374	184/23328	0.568097764	0.61633	0.37068	Fez1/Tek/	3
GO:0010828	GO:0010828	positive regulation of glucose transmembrane transpor	1/374	52/23328	0.568875745	0.61633	0.37068	C3	1
GO:0042307	GO:0042307	positive regulation of protein import into nucleus	1/374	52/23328	0.568875745	0.61633	0.37068	Tek	1
GO:0042491	GO:0042491	inner ear auditory receptor cell differentiation	1/374	52/23328	0.568875745	0.61633	0.37068	Hes1	1
GO:0050912	GO:0050912	detection of chemical stimulus involved in sensory per	1/374	52/23328	0.568875745	0.61633	0.37068	Rtp4	1
GO:0060612	GO:0060612	adipose tissue development	1/374	52/23328	0.568875745	0.61633	0.37068	Id2	1
GO:0060632	GO:0060632	regulation of microtubule-based movement	1/374	52/23328	0.568875745	0.61633	0.37068	Fez1	1
GO:0065002	GO:0065002	intracellular protein transmembrane transport	1/374	52/23328	0.568875745	0.61633	0.37068	Hspa5	1
GO:1903202	GO:1903202	negative regulation of oxidative stress-induced cell dea	1/374	52/23328	0.568875745	0.61633	0.37068	Hspb1	1
GO:0001960	GO:0001960	negative regulation of cytokine-mediated signaling patl	1/374	53/23328	0.575803072	0.62206	0.37413	Ecm1	1
GO:0002714	GO:0002714	positive regulation of B cell mediated immunity	1/374	53/23328	0.575803072	0.62206	0.37413	C3	1
GO:0002891	GO:0002891	positive regulation of immunoglobulin mediated immur	1/374	53/23328	0.575803072	0.62206	0.37413	C3	1
GO:0034605	GO:0034605	cellular response to heat	1/374	53/23328	0.575803072	0.62206	0.37413	Cd34	1
GO:0042738	GO:0042738	exogenous drug catabolic process	1/374	53/23328	0.575803072	0.62206	0.37413	Cyp4b1	1
GO:0043666	GO:0043666	regulation of phosphoprotein phosphatase activity	1/374	53/23328	0.575803072	0.62206	0.37413	Hsp90b1	1
GO:0044058	GO:0044058	regulation of digestive system process	1/374	53/23328	0.575803072	0.62206	0.37413	Aqp1	1
GO:0048599	GO:0048599	oocyte development	1/374	53/23328	0.575803072	0.62206	0.37413	Pde3a	1
GO:0048701	GO:0048701	embryonic cranial skeleton morphogenesis	1/374	53/23328	0.575803072	0.62206	0.37413	Bmp4	1
GO:0052372	GO:0052372	modulation by symbiont of entry into host	1/374	53/23328	0.575803072	0.62206	0.37413	Cd74	1
GO:1900408	GO:1900408	negative regulation of cellular response to oxidative str	1/374	53/23328	0.575803072	0.62206	0.37413	Hspb1	1
GO:0010823	GO:0010823	negative regulation of mitochondrion organization	1/374	54/23328	0.582619384	0.62877	0.37816	Bok	1
GO:0042737	GO:0042737	drug catabolic process	1/374	54/23328	0.582619384	0.62877	0.37816	Cyp4b1	1
GO:0046834	GO:0046834	lipid phosphorylation	1/374	54/23328	0.582619384	0.62877	0.37816	Socs3	1
GO:0071806	GO:0071806	protein transmembrane transport	1/374	54/23328	0.582619384	0.62877	0.37816	Hspa5	1
GO:0007098	GO:0007098	centrosome cycle	2/374	122/23328	0.584759493	0.63092	0.37945	Plk2/Pard	2
GO:0016126	GO:0016126	sterol biosynthetic process	1/374	55/23328	0.589326454	0.63502	0.38192	Apoe	1
GO:0031529	GO:0031529	ruffle organization	1/374	55/23328	0.589326454	0.63502	0.38192	Kank1	1
GO:0035306	GO:0035306	positive regulation of dephosphorylation	1/374	55/23328	0.589326454	0.63502	0.38192	Cdh5	1
GO:0061001	GO:0061001	regulation of dendritic spine morphogenesis	1/374	55/23328	0.589326454	0.63502	0.38192	Reln	1
GO:0086001	GO:0086001	cardiac muscle cell action potential	1/374	55/23328	0.589326454	0.63502	0.38192	Gja5	1
GO:0007259	GO:0007259	receptor signaling pathway via JAK-STAT	3/374	191/23328	0.59342508	0.63911	0.38438	Pecam1/H	3
GO:0007416	GO:0007416	synapse assembly	3/374	191/23328	0.59342508	0.63911	0.38438	Pcdh17/R	3
GO:0006023	GO:0006023	aminoglycan biosynthetic process	1/374	56/23328	0.595926029	0.63998	0.38491	Pdgfb	1
GO:0015872	GO:0015872	dopamine transport	1/374	56/23328	0.595926029	0.63998	0.38491	Cxcl12	1
GO:0017145	GO:0017145	stem cell division	1/374	56/23328	0.595926029	0.63998	0.38491	Sfrp2	1
GO:0043647	GO:0043647	inositol phosphate metabolic process	1/374	56/23328	0.595926029	0.63998	0.38491	Nudt4	1
GO:0045599	GO:0045599	negative regulation of fat cell differentiation	1/374	56/23328	0.595926029	0.63998	0.38491	Gata3	1
GO:0051568	GO:0051568	histone H3-K4 methylation	1/374	56/23328	0.595926029	0.63998	0.38491	Gata3	1
GO:0098586	GO:0098586	cellular response to virus	1/374	56/23328	0.595926029	0.63998	0.38491	Tspan6	1
GO:1902883	GO:1902883	negative regulation of response to oxidative stress	1/374	56/23328	0.595926029	0.63998	0.38491	Hspb1	1
GO:1903350	GO:1903350	response to dopamine	1/374	56/23328	0.595926029	0.63998	0.38491	Rgs4	1
GO:1903351	GO:1903351	cellular response to dopamine	1/374	56/23328	0.595926029	0.63998	0.38491	Rgs4	1
GO:1903533	GO:1903533	regulation of protein targeting	1/374	56/23328	0.595926029	0.63998	0.38491	Prnp	1
GO:0006473	GO:0006473	protein acetylation	3/374	192/23328	0.596963985	0.64093	0.38548	Gata3/Nu	3
GO:0006486	GO:0006486	protein glycosylation	3/374	193/23328	0.600482797	0.64438	0.38755	Gxylt2/Ac	3
GO:0043413	GO:0043413	macromolecule glycosylation	3/374	193/23328	0.600482797	0.64438	0.38755	Gxylt2/Ac	3
GO:0099003	GO:0099003	vesicle-mediated transport in synapse	4/374	260/23328	0.60169722	0.6453	0.3881	Pcdh17/C	4
GO:0009994	GO:0009994	oocyte differentiation	1/374	57/23328	0.602419827	0.6453	0.3881	Pde3a	1
GO:0042775	GO:0042775	mitochondrial ATP synthesis coupled electron transpor	1/374	57/23328	0.602419827	0.6453	0.3881	Cox4i2	1
GO:0043489	GO:0043489	RNA stabilization	1/374	57/23328	0.602419827	0.6453	0.3881	Zfp36	1
GO:0060761	GO:0060761	negative regulation of response to cytokine stimulus	1/374	57/23328	0.602419827	0.6453	0.3881	Ecm1	1
GO:1901607	GO:1901607	alpha-amino acid biosynthetic process	1/374	57/23328	0.602419827	0.6453	0.3881	Ass1	1
GO:1902373	GO:1902373	negative regulation of mRNA catabolic process	1/374	57/23328	0.602419827	0.6453	0.3881	Zfp36	1
GO:0032653	GO:0032653	regulation of interleukin-10 production	1/374	58/23328	0.608809539	0.6508	0.39141	Cd34	1
GO:0035065	GO:0035065	regulation of histone acetylation	1/374	58/23328	0.608809539	0.6508	0.39141	Gata3	1
GO:0035315	GO:0035315	hair cell differentiation	1/374	58/23328	0.608809539	0.6508	0.39141	Hes1	1
GO:0043330	GO:0043330	response to exogenous dsRNA	1/374	58/23328	0.608809539	0.6508	0.39141	Nfkbia	1
GO:0051445	GO:0051445	regulation of meiotic cell cycle	1/374	58/23328	0.608809539	0.6508	0.39141	Pde3a	1
GO:0099172	GO:0099172	presynapse organization	1/374	58/23328	0.608809539	0.6508	0.39141	Pcdh17	1
GO:1903902	GO:1903902	positive regulation of viral life cycle	1/374	58/23328	0.608809539	0.6508	0.39141	Cd74	1
GO:1904591	GO:1904591	positive regulation of protein import	1/374	58/23328	0.608809539	0.6508	0.39141	Tek	1
GO:0032386	GO:0032386	regulation of intracellular transport	5/374	329/23328	0.609491984	0.65136	0.39175	Bmp4/Fez	5
GO:0043244	GO:0043244	regulation of protein-containing complex disassembly	2/374	128/23328	0.610917865	0.65255	0.39247	Nes/Stmn	2
GO:0097696	GO:0097696	receptor signaling pathway via STAT	3/374	196/23328	0.610917897	0.65255	0.39247	Pecam1/H	3
GO:0002011	GO:0002011	morphogenesis of an epithelial sheet	1/374	59/23328	0.615096828	0.65657	0.39488	Pdpn	1
GO:0030042	GO:0030042	actin filament depolymerization	1/374	59/23328	0.615096828	0.65657	0.39488	Mical2	1
GO:0033138	GO:0033138	positive regulation of peptidyl-serine phosphorylation	2/374	129/23328	0.615153616	0.65657	0.39488	Sfrp2/Tek	2
GO:0042752	GO:0042752	regulation of circadian rhythm	2/374	130/23328	0.619354007	0.66072	0.39738	Prox1/Id2	2
GO:0090305	GO:0090305	nucleic acid phosphodiester bond hydrolysis	2/374	130/23328	0.619354007	0.66072	0.39738	Igfbp3/Zfp	2
GO:0007595	GO:0007595	lactation	1/374	60/23328	0.621283333	0.6621	0.39821	Gja1	1
GO:0046605	GO:0046605	regulation of centrosome cycle	1/374	60/23328	0.621283333	0.6621	0.39821	Plk2	1
GO:0050907	GO:0050907	detection of chemical stimulus involved in sensory per	1/374	60/23328	0.621283333	0.6621	0.39821	Rtp4	1
GO:0070231	GO:0070231	T cell apoptotic process	1/374	60/23328	0.621283333	0.6621	0.39821	Bmp4	1
GO:0006839	GO:0006839	mitochondrial transport	3/374	200/23328	0.624545761	0.66541	0.4002	Ucp2/Chc	3
GO:0006893	GO:0006893	Golgi to plasma membrane transport	1/374	61/23328	0.627370665	0.66752	0.40147	Krt18	1
GO:0008652	GO:0008652	cellular amino acid biosynthetic process	1/374	61/23328	0.627370665	0.66752	0.40147	Ass1	1
GO:0042773	GO:0042773	ATP synthesis coupled electron transport	1/374	61/23328	0.627370665	0.66752	0.40147	Cox4i2	1
GO:0045824	GO:0045824	negative regulation of innate immune response	1/374	61/23328	0.627370665	0.66752	0.40147	Serping1	1
GO:0009582	GO:0009582	detection of abiotic stimulus	2/374	132/23328	0.627648886	0.66752	0.40147	Cxcl12/Se	2
GO:0010469	GO:0010469	regulation of signaling receptor activity	2/374	132/23328	0.627648886	0.66752	0.40147	Itgb1/Reln	2

GO:0031023	GO:0031023	microtubule organizing center organization	2/374	132/2332	0.627648886	0.66752	0.40147	Plk2/Pard	2
GO:0009566	GO:0009566	fertilization	3/374	201/2332	0.627901348	0.66762	0.40153	Mfge8/Nr2	3
GO:0006006	GO:0006006	glucose metabolic process	3/374	202/2332	0.631236289	0.67099	0.40355	Igfbp3/Fal	3
GO:0009581	GO:0009581	detection of external stimulus	2/374	133/2332	0.63174348	0.67102	0.40357	Cxcl12/Se	2
GO:0042303	GO:0042303	molting cycle	2/374	133/2332	0.63174348	0.67102	0.40357	Pdgfa/Igfb	2
GO:0042633	GO:0042633	hair cycle	2/374	133/2332	0.63174348	0.67102	0.40357	Pdgfa/Igfb	2
GO:0030865	GO:0030865	cortical cytoskeleton organization	1/374	62/23328	0.633360409	0.67188	0.40409	Actn1	1
GO:0032387	GO:0032387	negative regulation of intracellular transport	1/374	62/23328	0.633360409	0.67188	0.40409	Cd36	1
GO:0043966	GO:0043966	histone H3 acetylation	1/374	62/23328	0.633360409	0.67188	0.40409	Gata3	1
GO:0048512	GO:0048512	circadian behavior	1/374	62/23328	0.633360409	0.67188	0.40409	Id2	1
GO:0060393	GO:0060393	regulation of pathway-restricted SMAD protein phosph	1/374	62/23328	0.633360409	0.67188	0.40409	Bmp4	1
GO:0043161	GO:0043161	proteasome-mediated ubiquitin-dependent protein cata	6/374	405/2332	0.633606189	0.67197	0.40414	Clu/Cbfa2	6
GO:0006605	GO:0006605	protein targeting	4/374	271/2332	0.634150704	0.67237	0.40439	Prnp/Hspa	4
GO:0044839	GO:0044839	cell cycle G2/M phase transition	2/374	134/2332	0.635802928	0.67395	0.40534	Fhl1/Cdkn	2
GO:0050994	GO:0050994	regulation of lipid catabolic process	1/374	63/23328	0.639254125	0.67727	0.40733	Scarb1	1
GO:0097479	GO:0097479	synaptic vesicle localization	1/374	63/23328	0.639254125	0.67727	0.40733	Pcdh17	1
GO:0051701	GO:0051701	interaction with host	2/374	135/2332	0.6398273	0.6777	0.40759	Gapdh/Cd	2
GO:0035195	GO:0035195	gene silencing by miRNA	2/374	136/2332	0.643816675	0.68168	0.40998	Bmp4/Zfp	2
GO:0031396	GO:0031396	regulation of protein ubiquitination	3/374	206/2332	0.644368842	0.68168	0.40998	Sox4/Hsp	3
GO:0001658	GO:0001658	branching involved in ureteric bud morphogenesis	1/374	64/23328	0.64505335	0.68168	0.40998	Bmp4	1
GO:0006879	GO:0006879	cellular iron ion homeostasis	1/374	64/23328	0.64505335	0.68168	0.40998	Lcn2	1
GO:0007622	GO:0007622	rhythmic behavior	1/374	64/23328	0.64505335	0.68168	0.40998	Id2	1
GO:0043331	GO:0043331	response to dsRNA	1/374	64/23328	0.64505335	0.68168	0.40998	Nfkbia	1
GO:0046173	GO:0046173	polyol biosynthetic process	1/374	64/23328	0.64505335	0.68168	0.40998	Acer2	1
GO:0061951	GO:0061951	establishment of protein localization to plasma membr	1/374	64/23328	0.64505335	0.68168	0.40998	Krt18	1
GO:0070059	GO:0070059	intrinsic apoptotic signaling pathway in response to en	1/374	64/23328	0.64505335	0.68168	0.40998	Bok	1
GO:0021515	GO:0021515	cell differentiation in spinal cord	1/374	65/23328	0.650759593	0.68637	0.41281	Sox4	1
GO:0042093	GO:0042093	T-helper cell differentiation	1/374	65/23328	0.650759593	0.68637	0.41281	Gata3	1
GO:0045454	GO:0045454	cell redox homeostasis	1/374	65/23328	0.650759593	0.68637	0.41281	Txndc5	1
GO:0060389	GO:0060389	pathway-restricted SMAD protein phosphorylation	1/374	65/23328	0.650759593	0.68637	0.41281	Bmp4	1
GO:0098840	GO:0098840	protein transport along microtubule	1/374	65/23328	0.650759593	0.68637	0.41281	Hspb1	1
GO:0099118	GO:0099118	microtubule-based protein transport	1/374	65/23328	0.650759593	0.68637	0.41281	Hspb1	1
GO:2000514	GO:2000514	regulation of CD4-positive, alpha-beta T cell activation	1/374	65/23328	0.650759593	0.68637	0.41281	Gata3	1
GO:0070085	GO:0070085	glycosylation	3/374	208/2332	0.65081038	0.68637	0.41281	Gxylt2/Ac	3
GO:0001508	GO:0001508	action potential	2/374	138/2332	0.651690777	0.68713	0.41326	Gja5/Cd3	2
GO:0002294	GO:0002294	CD4-positive, alpha-beta T cell differentiation involved	1/374	66/23328	0.656374343	0.69172	0.41602	Gata3	1
GO:0048278	GO:0048278	vesicle docking	1/374	66/23328	0.656374343	0.69172	0.41602	Ncam1	1
GO:0046330	GO:0046330	positive regulation of JNK cascade	2/374	140/2332	0.659425986	0.69476	0.41785	Flt4/Edn1	2
GO:0002293	GO:0002293	alpha-beta T cell differentiation involved in immune res	1/374	67/23328	0.661899061	0.69666	0.41899	Gata3	1
GO:0051298	GO:0051298	centrosome duplication	1/374	67/23328	0.661899061	0.69666	0.41899	Plk2	1
GO:0086003	GO:0086003	cardiac muscle cell contraction	1/374	67/23328	0.661899061	0.69666	0.41899	Gja5	1
GO:0090307	GO:0090307	mitotic spindle assembly	1/374	67/23328	0.661899061	0.69666	0.41899	Hspa1a	1
GO:0035194	GO:0035194	post-transcriptional gene silencing by RNA	2/374	142/2332	0.667023144	0.70026	0.42116	Bmp4/Zfp	2
GO:0002287	GO:0002287	alpha-beta T cell activation involved in immune respon	1/374	68/23328	0.667335188	0.70026	0.42116	Gata3	1
GO:0002712	GO:0002712	regulation of B cell mediated immunity	1/374	68/23328	0.667335188	0.70026	0.42116	C3	1
GO:0002889	GO:0002889	regulation of immunoglobulin mediated immune respon	1/374	68/23328	0.667335188	0.70026	0.42116	C3	1
GO:0031122	GO:0031122	cytoplasmic microtubule organization	1/374	68/23328	0.667335188	0.70026	0.42116	Dst	1
GO:0046635	GO:0046635	positive regulation of alpha-beta T cell activation	1/374	68/23328	0.667335188	0.70026	0.42116	Cd24a	1
GO:0046637	GO:0046637	regulation of alpha-beta T cell differentiation	1/374	68/23328	0.667335188	0.70026	0.42116	Gata3	1
GO:0050771	GO:0050771	negative regulation of axonogenesis	1/374	68/23328	0.667335188	0.70026	0.42116	Thy1	1
GO:0071868	GO:0071868	cellular response to monoamine stimulus	1/374	68/23328	0.667335188	0.70026	0.42116	Rgs4	1
GO:0071870	GO:0071870	cellular response to catecholamine stimulus	1/374	68/23328	0.667335188	0.70026	0.42116	Rgs4	1
GO:0099601	GO:0099601	regulation of neurotransmitter receptor activity	1/374	68/23328	0.667335188	0.70026	0.42116	Reln	1
GO:1902369	GO:1902369	negative regulation of RNA catabolic process	1/374	68/23328	0.667335188	0.70026	0.42116	Zfp36	1
GO:0042058	GO:0042058	regulation of epidermal growth factor receptor signalin	1/374	69/23328	0.672684141	0.70516	0.42411	Cdh13	1
GO:0048168	GO:0048168	regulation of neuronal synaptic plasticity	1/374	69/23328	0.672684141	0.70516	0.42411	Kdr	1
GO:0071867	GO:0071867	response to monoamine	1/374	69/23328	0.672684141	0.70516	0.42411	Rgs4	1
GO:0071869	GO:0071869	response to catecholamine	1/374	69/23328	0.672684141	0.70516	0.42411	Rgs4	1
GO:0090316	GO:0090316	positive regulation of intracellular protein transport	2/374	144/2332	0.674483184	0.70687	0.42513	Tek/Prnp	2
GO:0002381	GO:0002381	immunoglobulin production involved in immunoglobulir	1/374	70/23328	0.677947313	0.70996	0.42699	Fgl2	1
GO:0050885	GO:0050885	neuromuscular process controlling balance	1/374	70/23328	0.677947313	0.70996	0.42699	Gm2a	1
GO:2000179	GO:2000179	positive regulation of neural precursor cell proliferation	1/374	70/23328	0.677947313	0.70996	0.42699	Prox1	1
GO:0006402	GO:0006402	mRNA catabolic process	3/374	217/2332	0.678764459	0.71046	0.42729	Btg2/Vim/	3
GO:0051054	GO:0051054	positive regulation of DNA metabolic process	3/374	217/2332	0.678764459	0.71046	0.42729	Igfbp3/An	3
GO:0072594	GO:0072594	establishment of protein localization to organelle	6/374	426/2332	0.681253506	0.71289	0.42875	Bmp4/Cd3	6
GO:0016441	GO:0016441	posttranscriptional gene silencing	2/374	146/2332	0.681807115	0.71329	0.42899	Bmp4/Zfp	2
GO:0007040	GO:0007040	lysosome organization	1/374	71/23328	0.683126078	0.71413	0.4295	Pla2g5	1
GO:0080171	GO:0080171	lytic vacuole organization	1/374	71/23328	0.683126078	0.71413	0.4295	Pla2g5	1
GO:1901796	GO:1901796	regulation of signal transduction by p53 class mediator	1/374	71/23328	0.683126078	0.71413	0.4295	Cd74	1
GO:0051092	GO:0051092	positive regulation of NF-kappaB transcription factor a	2/374	147/2332	0.685418377	0.71635	0.43083	Clu/Cd36	2
GO:0050871	GO:0050871	positive regulation of B cell activation	3/374	220/2332	0.687706901	0.71856	0.43216	Cd38/Cdk	3
GO:1904888	GO:1904888	cranial skeletal system development	1/374	72/23328	0.688221784	0.71892	0.43238	Bmp4	1
GO:0044262	GO:0044262	cellular carbohydrate metabolic process	4/374	292/2332	0.691206437	0.72185	0.43415	Igfbp3/Nu	4
GO:0060997	GO:0060997	dendritic spine morphogenesis	1/374	73/23328	0.693235761	0.72361	0.4352	Reln	1
GO:0097120	GO:0097120	receptor localization to synapse	1/374	73/23328	0.693235761	0.72361	0.4352	Reln	1
GO:2001020	GO:2001020	regulation of response to DNA damage stimulus	3/374	222/2332	0.693564485	0.72377	0.4353	Clu/Cxcl1	3
GO:0061025	GO:0061025	membrane fusion	2/374	150/2332	0.696051064	0.72619	0.43675	Pla2g5/Ti	2
GO:0043299	GO:0043299	leukocyte degranulation	1/374	74/23328	0.698169317	0.72767	0.43764	Anxa3	1
GO:0046824	GO:0046824	positive regulation of nucleocytoplasmic transport	1/374	74/23328	0.698169317	0.72767	0.43764	Tek	1
GO:0099518	GO:0099518	vesicle cytoskeletal trafficking	1/374	74/23328	0.698169317	0.72767	0.43764	Myo1b	1
GO:1901184	GO:1901184	regulation of ERBB signaling pathway	1/374	74/23328	0.698169317	0.72767	0.43764	Cdh13	1
GO:0043279	GO:0043279	response to alkaloid	1/374	75/23328	0.703023736	0.73236	0.44046	Ncam1	1
GO:0098661	GO:0098661	inorganic anion transmembrane transport	1/374	75/23328	0.703023736	0.73236	0.44046	Prnp	1
GO:0043414	GO:0043414	macromolecule methylation	4/374	297/2332	0.703820333	0.73301	0.44085	Gata3/Btg	4
GO:0000271	GO:0000271	polysaccharide biosynthetic process	1/374	76/23328	0.707800286	0.73642	0.4429	Pdgfb	1
GO:0006094	GO:0006094	gluconeogenesis	1/374	76/23328	0.707800286	0.73642	0.4429	Gapdh	1
GO:0031060	GO:0031060	regulation of histone methylation	1/374	76/23328	0.707800286	0.73642	0.4429	Gata3	1
GO:1901606	GO:1901606	alpha-amino acid catabolic process	1/374	76/23328	0.707800286	0.73642	0.4429	Nos3	1
GO:0007338	GO:0007338	single fertilization	2/374	154/2332	0.709764468	0.73828	0.44402	Mfge8/Ho	2
GO:0033143	GO:0033143	regulation of intracellular steroid hormone receptor sig	1/374	77/23328	0.712500213	0.7402	0.44518	Sfrp1	1
GO:0043507	GO:0043507	positive regulation of JUN kinase activity	1/374	77/23328	0.712500213	0.7402	0.44518	Edn1	1
GO:1901224	GO:1901224	positive regulation of NIK/NF-kappaB signaling	1/374	77/23328	0.712500213	0.7402	0.44518	Edn1	1
GO:1903201	GO:1903201	regulation of oxidative stress-induced cell death	1/374	77/23328	0.712500213	0.7402	0.44518	Hspb1	1
GO:2000573	GO:2000573	positive regulation of DNA biosynthetic process	1/374	77/23328	0.712500213	0.7402	0.44518	Pdgfb	1
GO:0002066	GO:0002066	columnar/cuboidal epithelial cell development	1/374	78/23328	0.717124742	0.74445	0.44773	Bmp4	1
GO:0009142	GO:0009142	nucleoside triphosphate biosynthetic process	1/374	78/23328	0.717124742	0.74445	0.44773	Cmpk2	1
GO:0046718	GO:0046718	viral entry into host cell	1/374	78/23328	0.717124742	0.74445	0.44773	Cd74	1
GO:0019318	GO:0019318	hexose metabolic process	3/374	231/2332	0.718900082	0.7461	0.44873	Igfbp3/Fal	3
GO:0045333	GO:0045333	cellular respiration	2/374	157/2332	0.71970758	0.74675	0.44912	Cox4i2/Cb	2
GO:0019319	GO:0019319	hexose biosynthetic process	1/374	79/23328	0.721675079	0.74842	0.45013	Gapdh	1
GO:0140056	GO:0140056	organelle localization by membrane tethering	1/374	79/23328	0.721675079	0.74842	0.45013	Ncam1	1
GO:0050954	GO:0050954	sensory perception of mechanical stimulus	4/374	305/2332	0.723222091	0.74984	0.45098	Cxcl12/Co	4
GO:0032465	GO:0032465	regulation of cytokinesis	1/374	80/23328	0.726152413	0.75232	0.45247	Plk2	1
GO:0032729	GO:0032729	positive regulation of interferon-gamma production	1/374	80/23328	0.726152413	0.75232	0.45247	Cd2	1
GO:1901880	GO:1901880	negative regulation of protein depolymerization	1/374	80/23328	0.726152413	0.75232	0.45247	Stmn2	1
GO:0022904	GO:0022904	respiratory electron transport chain	1/374	81/23328	0.73055791	0.75669	0.4551	Cox4i2	1
GO:0043543	GO:0043543	protein acylation	3/374	237/2332	0.734870252	0.76081	0.45757	Gata3/Nu	3
GO:0044409	GO:0044409	entry into host	1/374	82/23328	0.73489272	0.76081	0.45757	Cd74	1
GO:1990830	GO:1990830	cellular response to leukemia inhibitory factor	4/374	311/2332	0.737145707	0.76295	0.45886	Pcolce2/N	4
GO:0018394	GO:0018394	peptidyl-lysine acetylation	2/374	163/2332	0.738734971	0.76389	0.45943	Gata3/So	2
GO:0006664	GO:0006664	glycolipid metabolic process	1/374	83/23328	0.739157976	0.76389	0.45943	Gm2a	1
GO:0044106	GO:0044106	cellular amine metabolic process	1/374	83/23328	0.739157976	0.76389	0.45943	Epas1	1
GO:0061178	GO:0061178	regulation of insulin secretion involved in cellular resp	1/374	83/23328	0.739157976	0.76389	0.45943	Ucp2	1
GO:0072384	GO:0072384	organelle transport along microtubule	1/374	83/23328	0.739157976	0.76389	0.45943	Fez1	1
GO:1990542	GO:1990542	mitochondrial transmembrane transport	1/374	83/23328	0.739157976	0.76389	0.45943	Ucp2	1
GO:1990823	GO:1990823	response to leukemia inhibitory factor	4/374	312/2332	0.739414304	0.76397	0.45948	Pcolce2/N	4
GO:0007018	GO:0007018	microtubule-based movement	5/374	385/2332	0.741846612	0.76629	0.46087	Fez1/Hsp	5
GO:1903320	GO:1903320	regulation of protein modification by small protein conj	3/374	240/2332	0.742583402	0.76686	0.46122	Sox4/Hsp	3
GO:0022406	GO:0022406	membrane docking	1/374	84/23328	0.743354789	0.7669	0.46124	Ncam1	1
GO:0022900	GO:0022900	electron transport chain	1/374	84/23328	0.743354789	0.7669	0.46124	Cox4i2	1
GO:0030148	GO:0030148	sphingolipid biosynthetic process	1/374	84/23328	0.743354789	0.7669	0.46124	Acer2	1
GO:1903509	GO:1903509	liposaccharide metabolic process	1/374	84/23328	0.743354789	0.7669	0.46124	Gm2a	1
GO:0007218	GO:0007218	neuropeptide signaling pathway	1/374	85/23328	0.747484255	0.77059	0.46346	Nts	1
GO:0043648	GO:0043648	dicarboxylic acid metabolic process	1/374	85/23328	0.747484255	0.77059	0.46346	Ass1	1
GO:0046364	GO:0046364	monosaccharide biosynthetic process	1/374	85/23328	0.747484255	0.77059	0.46346	Gapdh	1
GO:0006958	GO:0006958	complement activation, classical pathway	2/374	166/2332	0.747829226	0.77075	0.46356	C3/Serpin	2
GO:1903829	GO:1903829	positive regulation of cellular protein localization	4/374	317/2332	0.750535842	0.77335	0.46512	Tek/Itgb1/	4
GO:0006338	GO:0006338	chromatin remodeling	2/374	167/2332	0.750799781	0.77343	0.46517	Gata3/Hm	2
GO:0006260	GO:0006260	DNA replication	3/374	244/2332	0.752589329	0.77509	0.46616	Cst3/Pdgf	3

GO:0031047	GO:0031047	gene silencing by RNA	2/374	169/23328	0.756650778	0.77888	0.46845	Bmp4/Zfp	2
GO:0035303	GO:0035303	regulation of dephosphorylation	2/374	169/23328	0.756650778	0.77888	0.46845	Cdh5/Hsp	2
GO:0002312	GO:0002312	B cell activation involved in immune response	1/374	88/23328	0.759479264	0.78064	0.4695	Itm2a	1
GO:0006112	GO:0006112	energy reserve metabolic process	1/374	88/23328	0.759479264	0.78064	0.4695	Adgrf5	1
GO:0045913	GO:0045913	positive regulation of carbohydrate metabolic process	1/374	88/23328	0.759479264	0.78064	0.4695	Pdgfb	1
GO:1900182	GO:1900182	positive regulation of protein localization to nucleus	1/374	88/23328	0.759479264	0.78064	0.4695	Tek	1
GO:1903312	GO:1903312	negative regulation of mRNA metabolic process	1/374	88/23328	0.759479264	0.78064	0.4695	Zfp36	1
GO:1904063	GO:1904063	negative regulation of cation transmembrane transport	1/374	88/23328	0.759479264	0.78064	0.4695	Rgs4	1
GO:0015980	GO:0015980	energy derivation by oxidation of organic compounds	3/374	248/23328	0.762281257	0.78333	0.47112	Cox4i2/Cb	3
GO:0006520	GO:0006520	cellular amino acid metabolic process	3/374	250/23328	0.767010939	0.78775	0.47378	Ass1/Nos	3
GO:0009063	GO:0009063	cellular amino acid catabolic process	1/374	90/23328	0.767158511	0.78775	0.47378	Nos3	1
GO:0043242	GO:0043242	negative regulation of protein-containing complex disa	1/374	90/23328	0.767158511	0.78775	0.47378	Stmn2	1
GO:0051648	GO:0051648	vesicle localization	2/374	174/23328	0.770761973	0.79121	0.47586	Pcdh17/M	2
GO:0040014	GO:0040014	regulation of multicellular organism growth	1/374	91/23328	0.770905938	0.79121	0.47586	H19	1
GO:0035773	GO:0035773	insulin secretion involved in cellular response to gluco	1/374	92/23328	0.774593212	0.79461	0.4779	Ucp2	1
GO:0090630	GO:0090630	activation of GTPase activity	1/374	92/23328	0.774593212	0.79461	0.4779	Arhgef15	1
GO:0006401	GO:0006401	RNA catabolic process	3/374	254/23328	0.776240865	0.7961	0.4788	Btg2/Vim/	3
GO:0007051	GO:0007051	spindle organization	2/374	179/23328	0.784156019	0.80402	0.48356	Plk2/Hspa	2
GO:0002251	GO:0002251	organ or tissue specific immune response	1/374	95/23328	0.785303665	0.805	0.48415	Cfh	1
GO:0060021	GO:0060021	roof of mouth development	1/374	96/23328	0.788759802	0.80812	0.48603	Hand2	1
GO:0098876	GO:0098876	vesicle-mediated transport to the plasma membrane	1/374	96/23328	0.788759802	0.80812	0.48603	Krt18	1
GO:0010256	GO:0010256	endomembrane system organization	5/374	410/23328	0.789126435	0.80812	0.48603	Clu/Srgn/	5
GO:0002455	GO:0002455	humoral immune response mediated by circulating imr	2/374	181/23328	0.789318638	0.80812	0.48603	C3/Serpin	2
GO:0050853	GO:0050853	B cell receptor signaling pathway	2/374	181/23328	0.789318638	0.80812	0.48603	Bmx/Cd38	2
GO:0005996	GO:0005996	monosaccharide metabolic process	3/374	261/23328	0.791670981	0.81033	0.48736	Igfbp3/Fal	3
GO:0006479	GO:0006479	protein methylation	2/374	183/23328	0.794372341	0.8127	0.48878	Gata3/Btg	2
GO:0008213	GO:0008213	protein alkylation	2/374	183/23328	0.794372341	0.8127	0.48878	Gata3/Btg	2
GO:0036473	GO:0036473	cell death in response to oxidative stress	1/374	98/23328	0.795506495	0.81346	0.48924	Hspb1	1
GO:0140029	GO:0140029	exocytic process	1/374	98/23328	0.795506495	0.81346	0.48924	Ncam1	1
GO:0030101	GO:0030101	natural killer cell activation	1/374	99/23328	0.798798814	0.81642	0.49102	Id2	1
GO:0042158	GO:0042158	lipoprotein biosynthetic process	1/374	99/23328	0.798798814	0.81642	0.49102	Apoe	1
GO:1903311	GO:1903311	regulation of mRNA metabolic process	3/374	266/23328	0.802143713	0.81964	0.49296	Btg2/Vim/	3
GO:0006352	GO:0006352	DNA-templated transcription, initiation	1/374	101/23328	0.805225699	0.82198	0.49437	Jun	1
GO:0006906	GO:0006906	vesicle fusion	1/374	101/23328	0.805225699	0.82198	0.49437	Pla2g5	1
GO:0007041	GO:0007041	lysosomal transport	1/374	101/23328	0.805225699	0.82198	0.49437	Hspa1a	1
GO:0031058	GO:0031058	positive regulation of histone modification	1/374	101/23328	0.805225699	0.82198	0.49437	Gata3	1
GO:0048193	GO:0048193	Golgi vesicle transport	3/374	268/23328	0.806207745	0.82279	0.49485	Krt18/Plpp	3
GO:0032259	GO:0032259	methylation	4/374	349/23328	0.81324226	0.82976	0.49905	Gata3/Btg	4
GO:0052126	GO:0052126	movement in host environment	1/374	104/23328	0.814484144	0.83083	0.49968	Cd74	1
GO:0045921	GO:0045921	positive regulation of exocytosis	1/374	105/23328	0.817471697	0.83367	0.50139	Sdc1	1
GO:0015931	GO:0015931	nucleobase-containing compound transport	2/374	193/23328	0.818066104	0.83407	0.50164	Zfp36/Gja	2
GO:0001578	GO:0001578	microtubule bundle formation	1/374	106/23328	0.820411264	0.83626	0.50295	Tppp3	1
GO:0030518	GO:0030518	intracellular steroid hormone receptor signaling pathwa	1/374	107/23328	0.823303616	0.8388	0.50448	Sfrp1	1
GO:0090174	GO:0090174	organelle membrane fusion	1/374	107/23328	0.823303616	0.8388	0.50448	Pla2g5	1
GO:0043255	GO:0043255	regulation of carbohydrate biosynthetic process	1/374	108/23328	0.826149508	0.84149	0.5061	Pdgfb	1
GO:0016051	GO:0016051	carbohydrate biosynthetic process	2/374	197/23328	0.826840191	0.84199	0.5064	Gapdh/Pd	2
GO:0009141	GO:0009141	nucleoside triphosphate metabolic process	1/374	109/23328	0.828949683	0.84372	0.50744	Cmpk2	1
GO:0051028	GO:0051028	mRNA transport	1/374	109/23328	0.828949683	0.84372	0.50744	Zfp36	1
GO:0051225	GO:0051225	spindle assembly	1/374	112/23328	0.837083183	0.85179	0.5123	Hspa1a	1
GO:0018205	GO:0018205	peptidyl-lysine modification	4/374	364/23328	0.837877954	0.85239	0.51266	Lox/Gata3	4
GO:0009165	GO:0009165	nucleotide biosynthetic process	2/374	204/23328	0.841284053	0.85565	0.51462	Nos3/Cmp	2
GO:0005976	GO:0005976	polysaccharide metabolic process	1/374	114/23328	0.842290057	0.85647	0.51511	Pdgfb	1
GO:0031398	GO:0031398	positive regulation of protein ubiquitination	1/374	115/23328	0.844830917	0.85884	0.51653	Hspa5	1
GO:0034968	GO:0034968	histone lysine methylation	1/374	116/23328	0.847330949	0.86117	0.51794	Gata3	1
GO:1905269	GO:1905269	positive regulation of chromatin organization	1/374	117/23328	0.849790807	0.86346	0.51931	Gata3	1
GO:1901293	GO:1901293	nucleoside phosphate biosynthetic process	2/374	209/23328	0.850924381	0.8644	0.51988	Nos3/Cmp	2
GO:0018107	GO:0018107	peptidyl-threonine phosphorylation	1/374	118/23328	0.852211135	0.86529	0.52041	Ddit4	1
GO:0030317	GO:0030317	flagellated sperm motility	1/374	118/23328	0.852211135	0.86529	0.52041	Pltp	1
GO:0097305	GO:0097305	response to alcohol	2/374	212/23328	0.856450066	0.86938	0.52287	Adh7/Adh	2
GO:0046467	GO:0046467	membrane lipid biosynthetic process	1/374	121/23328	0.859241226	0.87179	0.52432	Acer2	1
GO:0097722	GO:0097722	sperm motility	1/374	121/23328	0.859241226	0.87179	0.52432	Pltp	1
GO:1903828	GO:1903828	negative regulation of cellular protein localization	1/374	122/23328	0.86150967	0.87388	0.52558	Cd36	1
GO:0000723	GO:0000723	telomere maintenance	1/374	123/23328	0.863741652	0.87556	0.52659	Hspa1a	1
GO:0010921	GO:0010921	regulation of phosphatase activity	1/374	123/23328	0.863741652	0.87556	0.52659	Hsp90b1	1
GO:0051656	GO:0051656	establishment of organelle localization	4/374	382/23328	0.863798781	0.87556	0.52659	Fez1/Itgb1	4
GO:0006997	GO:0006997	nucleus organization	1/374	124/23328	0.865937758	0.87751	0.52776	Hmgb2	1
GO:0032200	GO:0032200	telomere organization	1/374	125/23328	0.868098561	0.87949	0.52895	Hspa1a	1
GO:0018210	GO:0018210	peptidyl-threonine modification	1/374	127/23328	0.872316516	0.88333	0.53127	Ddit4	1
GO:0042157	GO:0042157	lipoprotein metabolic process	1/374	127/23328	0.872316516	0.88333	0.53127	Apoe	1
GO:0006887	GO:0006887	exocytosis	4/374	389/23328	0.872879853	0.88369	0.53148	Ncam1/Ar	4
GO:0010906	GO:0010906	regulation of glucose metabolic process	1/374	128/23328	0.874374773	0.88499	0.53226	Igfbp3	1
GO:0060294	GO:0060294	cilium movement involved in cell motility	1/374	129/23328	0.876399939	0.88682	0.53336	Pltp	1
GO:0018022	GO:0018022	peptidyl-lysine methylation	1/374	131/23328	0.880353108	0.89061	0.53564	Gata3	1
GO:0007286	GO:0007286	spermatid development	2/374	227/23328	0.881373513	0.89142	0.53613	Dhh/Hmgb	2
GO:0001539	GO:0001539	cilium or flagellum-dependent cell motility	1/374	136/23328	0.889693046	0.8994	0.54093	Pltp	1
GO:0060285	GO:0060285	cilium-dependent cell motility	1/374	136/23328	0.889693046	0.8994	0.54093	Pltp	1
GO:0048284	GO:0048284	organelle fusion	1/374	138/23328	0.89322211	0.90275	0.54294	Pla2g5	1
GO:0048515	GO:0048515	spermatid differentiation	2/374	236/23328	0.894346525	0.90367	0.54349	Dhh/Hmgb	2
GO:1903322	GO:1903322	positive regulation of protein modification by small prot	1/374	139/23328	0.894944186	0.90405	0.54373	Hspa5	1
GO:0007034	GO:0007034	vacuolar transport	1/374	140/23328	0.896638562	0.90554	0.54462	Hspa1a	1
GO:0009451	GO:0009451	RNA modification	1/374	143/23328	0.901559897	0.91029	0.54748	Apobec3	1
GO:0016571	GO:0016571	histone methylation	1/374	144/23328	0.903147846	0.91168	0.54831	Gata3	1
GO:0006836	GO:0006836	neurotransmitter transport	2/374	245/23328	0.905992315	0.91414	0.54979	Itgb1/Slc6	2
GO:0016573	GO:0016573	histone acetylation	1/374	146/23328	0.90624751	0.91414	0.54979	Gata3	1
GO:0098693	GO:0098693	regulation of synaptic vesicle cycle	1/374	146/23328	0.90624751	0.91414	0.54979	Pcdh17	1
GO:0051588	GO:0051588	regulation of neurotransmitter transport	1/374	151/23328	0.913570649	0.92131	0.5541	Itgb1	1
GO:0006475	GO:0006475	internal protein amino acid acetylation	1/374	152/23328	0.914965332	0.92227	0.55468	Gata3	1
GO:0018393	GO:0018393	internal peptidyl-lysine acetylation	1/374	152/23328	0.914965332	0.92227	0.55468	Gata3	1
GO:0017157	GO:0017157	regulation of exocytosis	2/374	254/23328	0.916431351	0.92352	0.55543	Ncam1/Sc	2
GO:0051650	GO:0051650	establishment of vesicle localization	1/374	155/23328	0.91901614	0.9259	0.55687	Myo1b	1
GO:0050657	GO:0050657	nucleic acid transport	1/374	157/23328	0.921609174	0.92798	0.55812	Zfp36	1
GO:0050658	GO:0050658	RNA transport	1/374	157/23328	0.921609174	0.92798	0.55812	Zfp36	1
GO:0031503	GO:0031503	protein-containing complex localization	2/374	259/23328	0.921751041	0.92798	0.55812	Tnfaip2/R	2
GO:0002377	GO:0002377	immunoglobulin production	2/374	262/23328	0.924788365	0.9307	0.55975	Itm2a/Fgl2	2
GO:0000910	GO:0000910	cytokinesis	1/374	160/23328	0.925344281	0.9307	0.55975	Plk2	1
GO:0006164	GO:0006164	purine nucleotide biosynthetic process	1/374	160/23328	0.925344281	0.9307	0.55975	Nos3	1
GO:0051236	GO:0051236	establishment of RNA localization	1/374	160/23328	0.925344281	0.9307	0.55975	Zfp36	1
GO:0031056	GO:0031056	regulation of histone modification	1/374	163/23328	0.928901873	0.93383	0.56163	Gata3	1
GO:0040029	GO:0040029	regulation of gene expression, epigenetic	1/374	163/23328	0.928901873	0.93383	0.56163	Klf2	1
GO:0072522	GO:0072522	purine-containing compound biosynthetic process	1/374	165/23328	0.931179154	0.93589	0.56288	Nos3	1
GO:0007605	GO:0007605	sensory perception of sound	2/374	274/23328	0.935861862	0.94037	0.56557	Col1a1/Sl	2
GO:0016458	GO:0016458	gene silencing	2/374	279/23328	0.940004787	0.94431	0.56794	Bmp4/Zfp	2
GO:0003341	GO:0003341	cilium movement	1/374	180/23328	0.94609307	0.9502	0.57148	Pltp	1
GO:0006403	GO:0006403	RNA localization	1/374	182/23328	0.947820973	0.9517	0.57238	Zfp36	1
GO:0140013	GO:0140013	meiotic nuclear division	1/374	184/23328	0.949493634	0.95315	0.57326	Pde3a	1
GO:2001252	GO:2001252	positive regulation of chromosome organization	1/374	192/23328	0.95566626	0.95912	0.57684	Gata3	1
GO:0060271	GO:0060271	cilium assembly	2/374	310/23328	0.960540207	0.96378	0.57965	Ehd4/Ablii	2
GO:1903046	GO:1903046	meiotic cell cycle process	1/374	202/23328	0.962334657	0.96534	0.58059	Pde3a	1
GO:1902275	GO:1902275	regulation of chromatin organization	1/374	210/23328	0.966941303	0.96973	0.58323	Gata3	1
GO:0016054	GO:0016054	organic acid catabolic process	1/374	223/23328	0.973259023	0.9756	0.58676	Nos3	1
GO:0046395	GO:0046395	carboxylic acid catabolic process	1/374	223/23328	0.973259023	0.9756	0.58676	Nos3	1
GO:0044782	GO:0044782	cilium organization	2/374	343/23328	0.97495029	0.97706	0.58763	Ehd4/Ablii	2
GO:0016197	GO:0016197	endosomal transport	1/374	228/23328	0.975354535	0.97723	0.58774	Ehd4	1
GO:0001505	GO:0001505	regulation of neurotransmitter levels	1/374	257/23328	0.984652736	0.98631	0.5932	Itgb1	1
GO:0045055	GO:0045055	regulated exocytosis	1/374	261/23328	0.985624027	0.98704	0.59364	Anxa3	1
GO:0006310	GO:0006310	DNA recombination	1/374	288/23328	0.990756216	0.99195	0.59659	Hmgb2	1
GO:0051321	GO:0051321	meiotic cell cycle	1/374	317/23328	0.994250855	0.99521	0.59855	Pde3a	1
GO:0033044	GO:0033044	regulation of chromosome organization	1/374	369/23328	0.997550233	0.99827	0.60039	Gata3	1
GO:0016570	GO:0016570	histone modification	1/374	448/23328	0.999332178	0.99961	0.6012	Gata3	1
GO:0016569	GO:0016569	covalent chromatin modification	1/374	462/23328	0.999469819	0.99961	0.6012	Gata3	1
GO:0006281	GO:0006281	DNA repair	1/374	481/23328	0.999612484	0.99961	0.6012	Hspa1a	1

EC 3 GO:BP

	ID	Description	GeneRatio	BgRatio	pvalue	p.adjust	qvalue	geneID	Count
GO:0050673	GO:0050673	epithelial cell proliferation	42/389	438/23328	5.24E-20	2.14E-16	1.36E-16	Nr2f2/Igfb	42
GO:0001935	GO:0001935	endothelial cell proliferation	26/389	149/23328	3.10E-19	6.31E-16	4.01E-16	Nr2f2/Rgc	26
GO:0009611	GO:0009611	response to wounding	41/389	492/23328	2.36E-17	3.21E-14	2.04E-14	Mmrn1/Cc	41
GO:0001667	GO:0001667	ameboidal-type cell migration	38/389	427/23328	4.24E-17	4.31E-14	2.74E-14	Nr2f2/Rgc	38
GO:0001936	GO:0001936	regulation of endothelial cell proliferation	23/389	134/23328	5.41E-17	4.41E-14	2.80E-14	Nr2f2/Rgc	23
GO:1901342	GO:1901342	regulation of vasculature development	34/389	356/23328	2.47E-16	1.68E-13	1.07E-13	Cldn5/Rgc	34

GO:0050678	GO:0050678	regulation of epithelial cell proliferation	34/389	372/23328	9.30E-16	5.41E-13	3.44E-13	Nr2f2/Rgc	34
GO:0045765	GO:0045765	regulation of angiogenesis	31/389	320/23328	3.65E-15	1.86E-12	1.18E-12	Cldn5/Rgc	31
GO:0043542	GO:0043542	endothelial cell migration	25/389	212/23328	2.05E-14	9.29E-12	5.91E-12	Nr2f2/Rgc	25
GO:0050900	GO:0050900	leukocyte migration	31/389	360/23328	9.13E-14	3.72E-11	2.37E-11	Lbp/Thy1/	31
GO:0010631	GO:0010631	epithelial cell migration	28/389	295/23328	1.37E-13	5.07E-11	3.22E-11	Nr2f2/Rgc	28
GO:0090132	GO:0090132	epithelium migration	28/389	297/23328	1.62E-13	5.50E-11	3.49E-11	Nr2f2/Rgc	28
GO:0003158	GO:0003158	endothelium development	19/389	122/23328	1.83E-13	5.56E-11	3.54E-11	Cldn5/Nr2	19
GO:0090130	GO:0090130	tissue migration	28/389	299/23328	1.91E-13	5.56E-11	3.54E-11	Nr2f2/Rgc	28
GO:0045446	GO:0045446	endothelial cell differentiation	18/389	108/23328	2.38E-13	6.20E-11	3.94E-11	Cldn5/Nr2	18
GO:0042060	GO:0042060	wound healing	30/389	349/23328	2.43E-13	6.20E-11	3.94E-11	Mmrn1/Cc	30
GO:0030198	GO:0030198	extracellular matrix organization	27/389	302/23328	1.57E-12	3.62E-10	2.30E-10	Rgcc/Tcf1	27
GO:0051271	GO:0051271	negative regulation of cellular component movement	28/389	326/23328	1.60E-12	3.62E-10	2.30E-10	Thy1/Cldn	28
GO:0043062	GO:0043062	extracellular structure organization	27/389	303/23328	1.70E-12	3.65E-10	2.32E-10	Rgcc/Tcf1	27
GO:1903034	GO:1903034	regulation of response to wounding	21/389	186/23328	6.05E-12	1.23E-09	7.84E-10	Cd9/Ptn/C	21
GO:0001666	GO:0001666	response to hypoxia	22/389	208/23328	7.03E-12	1.32E-09	8.38E-10	Rgcc/Epa	22
GO:1900046	GO:1900046	regulation of hemostasis	15/389	83/23328	7.12E-12	1.32E-09	8.38E-10	Cd9/Cd34	15
GO:0030336	GO:0030336	negative regulation of cell migration	25/389	278/23328	9.47E-12	1.68E-09	1.07E-09	Thy1/Cldn	25
GO:1901343	GO:1901343	negative regulation of vasculature development	18/389	139/23328	1.98E-11	3.36E-09	2.14E-09	Cldn5/Rgc	18
GO:2000146	GO:2000146	negative regulation of cell motility	25/389	291/23328	2.56E-11	4.18E-09	2.66E-09	Thy1/Cldn	25
GO:0001938	GO:0001938	positive regulation of endothelial cell proliferation	15/389	91/23328	2.84E-11	4.45E-09	2.83E-09	Flt4/Cxcl1	15
GO:0003018	GO:0003018	vascular process in circulatory system	22/389	224/23328	3.06E-11	4.62E-09	2.94E-09	Cldn5/Nts	22
GO:0061041	GO:0061041	regulation of wound healing	18/389	146/23328	4.57E-11	6.65E-09	4.23E-09	Cd9/Cd34	18
GO:0007596	GO:0007596	blood coagulation	19/389	166/23328	4.90E-11	6.89E-09	4.38E-09	Mmrn1/Cc	19
GO:0040013	GO:0040013	negative regulation of locomotion	26/389	326/23328	5.34E-11	7.25E-09	4.61E-09	Thy1/Cldn	26
GO:0007599	GO:0007599	hemostasis	19/389	168/23328	6.05E-11	7.80E-09	4.96E-09	Mmrn1/Cc	19
GO:0060326	GO:0060326	cell chemotaxis	25/389	303/23328	6.13E-11	7.80E-09	4.96E-09	Lbp/Ccl21	25
GO:0050817	GO:0050817	coagulation	19/389	169/23328	6.72E-11	8.29E-09	5.27E-09	Mmrn1/Cc	19
GO:0036293	GO:0036293	response to decreased oxygen levels	22/389	234/23328	7.21E-11	8.55E-09	5.44E-09	Rgcc/Epa	22
GO:0032970	GO:0032970	regulation of actin filament-based process	29/389	410/23328	7.35E-11	8.55E-09	5.44E-09	Rgcc/Gmf	29
GO:0030193	GO:0030193	regulation of blood coagulation	14/389	82/23328	8.01E-11	9.07E-09	5.77E-09	Cd9/Cd34	14
GO:0007517	GO:0007517	muscle organ development	29/389	420/23328	1.30E-10	1.43E-08	9.11E-09	Nr2f2/Id3/	29
GO:0050818	GO:0050818	regulation of coagulation	14/389	86/23328	1.56E-10	1.67E-08	1.06E-08	Cd9/Cd34	14
GO:1902903	GO:1902903	regulation of supramolecular fiber organization	27/389	370/23328	1.69E-10	1.76E-08	1.12E-08	Rgcc/Gmf	27
GO:0016525	GO:0016525	negative regulation of angiogenesis	16/389	122/23328	2.17E-10	2.21E-08	1.40E-08	Cldn5/Rgc	16
GO:0002040	GO:0002040	sprouting angiogenesis	16/389	123/23328	2.46E-10	2.44E-08	1.55E-08	Flt4/Flt1/B	16
GO:0048010	GO:0048010	vascular endothelial growth factor receptor signaling pa	11/389	48/23328	3.13E-10	3.04E-08	1.93E-08	Flt4/Flt1/B	11
GO:0050878	GO:0050878	regulation of body fluid levels	26/389	354/23328	3.23E-10	3.06E-08	1.95E-08	Mmrn1/Cc	26
GO:2000181	GO:2000181	negative regulation of blood vessel morphogenesis	16/389	126/23328	3.54E-10	3.24E-08	2.06E-08	Cldn5/Rgc	16
GO:0050679	GO:0050679	positive regulation of epithelial cell proliferation	20/389	208/23328	3.58E-10	3.24E-08	2.06E-08	Ptn/Flt4/T	20
GO:0010594	GO:0010594	regulation of endothelial cell migration	18/389	166/23328	3.90E-10	3.45E-08	2.20E-08	Nr2f2/Rgc	18
GO:0060537	GO:0060537	muscle tissue development	30/389	470/23328	4.12E-10	3.57E-08	2.27E-08	Nr2f2/Igfb	30
GO:0010632	GO:0010632	regulation of epithelial cell migration	21/389	235/23328	5.02E-10	4.26E-08	2.71E-08	Nr2f2/Rgc	21
GO:0070482	GO:0070482	response to oxygen levels	23/389	287/23328	6.43E-10	5.35E-08	3.40E-08	Rgcc/Epa	23
GO:0045785	GO:0045785	positive regulation of cell adhesion	28/389	435/23328	1.33E-09	1.08E-07	6.84E-08	Thy1/Ptn/	28
GO:0031589	GO:0031589	cell-substrate adhesion	25/389	351/23328	1.35E-09	1.08E-07	6.84E-08	Thy1/Ptn/	25
GO:0010038	GO:0010038	response to metal ion	22/389	274/23328	1.47E-09	1.15E-07	7.33E-08	Cp/Fabp4	22
GO:0001885	GO:0001885	endothelial cell development	12/389	71/23328	2.06E-09	1.58E-07	1.01E-07	Cldn5/Col	12
GO:0051961	GO:0051961	negative regulation of nervous system development	25/389	365/23328	2.99E-09	2.26E-07	1.44E-07	Dtx1/Thy1	25
GO:0050680	GO:0050680	negative regulation of epithelial cell proliferation	16/389	148/23328	3.88E-09	2.88E-07	1.83E-07	Nr2f2/Rgc	16
GO:0031099	GO:0031099	regeneration	16/389	150/23328	4.72E-09	3.44E-07	2.19E-07	Thy1/Cd9/	16
GO:0007015	GO:0007015	actin filament organization	27/389	437/23328	6.36E-09	4.54E-07	2.89E-07	Rgcc/Gmf	27
GO:0019932	GO:0019932	second-messenger-mediated signaling	27/389	445/23328	9.33E-09	6.55E-07	4.17E-07	Cd36/Prnp	27
GO:0010721	GO:0010721	negative regulation of cell development	25/389	387/23328	9.72E-09	6.71E-07	4.27E-07	Dtx1/Thy1	25
GO:0014706	GO:0014706	striated muscle tissue development	27/389	447/23328	1.02E-08	6.96E-07	4.43E-07	Nr2f2/Hox	27
GO:0032956	GO:0032956	regulation of actin cytoskeleton organization	24/389	361/23328	1.11E-08	7.43E-07	4.72E-07	Rgcc/Gmf	24
GO:0031345	GO:0031345	negative regulation of cell projection organization	18/389	207/23328	1.35E-08	8.89E-07	5.65E-07	Thy1/Ptpr	18
GO:1904018	GO:1904018	positive regulation of vasculature development	18/389	208/23328	1.46E-08	9.43E-07	6.00E-07	Flt1/Cd34	18
GO:0045665	GO:0045665	negative regulation of neuron differentiation	20/389	259/23328	1.60E-08	1.02E-06	6.49E-07	Dtx1/Thy1	20
GO:0048660	GO:0048660	regulation of smooth muscle cell proliferation	16/389	165/23328	1.87E-08	1.17E-06	7.45E-07	Igfbp5/Bm	16
GO:0002685	GO:0002685	regulation of leukocyte migration	18/389	217/23328	2.82E-08	1.74E-06	1.11E-06	Lbp/Thy1/	18
GO:0048659	GO:0048659	smooth muscle cell proliferation	16/389	170/23328	2.86E-08	1.74E-06	1.11E-06	Igfbp5/Bm	16
GO:0048661	GO:0048661	positive regulation of smooth muscle cell proliferation	13/389	108/23328	3.11E-08	1.86E-06	1.18E-06	Igfbp5/Bm	13
GO:0010811	GO:0010811	positive regulation of cell-substrate adhesion	14/389	128/23328	3.17E-08	1.87E-06	1.19E-06	Thy1/Ptn/	14
GO:0030595	GO:0030595	leukocyte chemotaxis	18/389	219/23328	3.24E-08	1.89E-06	1.20E-06	Lbp/Ccl21	18
GO:0042445	GO:0042445	hormone metabolic process	17/389	196/23328	3.56E-08	2.01E-06	1.28E-06	Reln/Duox	17
GO:0048771	GO:0048771	tissue remodeling	17/389	196/23328	3.56E-08	2.01E-06	1.28E-06	Igfbp5/Ptn	17
GO:0032102	GO:0032102	negative regulation of response to external stimulus	24/389	384/23328	3.62E-08	2.02E-06	1.28E-06	Fgl2/Cd9/	24
GO:0010977	GO:0010977	negative regulation of neuron projection development	16/389	176/23328	4.67E-08	2.57E-06	1.63E-06	Thy1/Id1/	16
GO:0050768	GO:0050768	negative regulation of neurogenesis	22/389	337/23328	6.33E-08	3.44E-06	2.19E-06	Dtx1/Thy1	22
GO:0031102	GO:0031102	neuron projection regeneration	10/389	62/23328	7.36E-08	3.95E-06	2.51E-06	Thy1/Ptn/	10
GO:0008217	GO:0008217	regulation of blood pressure	17/389	209/23328	9.12E-08	4.83E-06	3.07E-06	Nr2f2/Eph	17
GO:0050866	GO:0050866	negative regulation of cell activation	17/389	210/23328	9.77E-08	5.10E-06	3.25E-06	Dtx1/Fgl2/	17
GO:1900047	GO:1900047	negative regulation of hemostasis	9/389	49/23328	1.04E-07	5.34E-06	3.40E-06	Cd9/Cd34	9
GO:0042326	GO:0042326	negative regulation of phosphorylation	26/389	471/23328	1.16E-07	5.89E-06	3.75E-06	Thy1/Nr2f	26
GO:0045766	GO:0045766	positive regulation of angiogenesis	16/389	188/23328	1.17E-07	5.89E-06	3.75E-06	Flt1/Cd34	16
GO:0046688	GO:0046688	response to copper ion	7/389	25/23328	1.26E-07	6.26E-06	3.98E-06	Cp/Prnp/L	7
GO:0010810	GO:0010810	regulation of cell-substrate adhesion	17/389	214/23328	1.28E-07	6.30E-06	4.01E-06	Thy1/Ptn/	17
GO:0001763	GO:0001763	morphogenesis of a branching structure	18/389	241/23328	1.39E-07	6.75E-06	4.29E-06	Flt1/Tbx1/	18
GO:0010595	GO:0010595	positive regulation of endothelial cell migration	12/389	103/23328	1.53E-07	7.31E-06	4.65E-06	Sparc/Flt4	12
GO:0010543	GO:0010543	regulation of platelet activation	8/389	38/23328	1.76E-07	8.32E-06	5.29E-06	Cd9/Serpi	8
GO:0097529	GO:0097529	myeloid leukocyte migration	17/389	219/23328	1.79E-07	8.38E-06	5.33E-06	Lbp/Ccl21	17
GO:0061138	GO:0061138	morphogenesis of a branching epithelium	17/389	222/23328	2.17E-07	1.01E-05	6.40E-06	Flt1/Tbx1/	17
GO:1903706	GO:1903706	regulation of hemopoiesis	24/389	424/23328	2.28E-07	1.04E-05	6.63E-06	Dtx1/Fgl2/	24
GO:0010634	GO:0010634	positive regulation of epithelial cell migration	14/389	150/23328	2.34E-07	1.04E-05	6.63E-06	Sparc/Flt4	14
GO:0003007	GO:0003007	heart morphogenesis	19/389	277/23328	2.37E-07	1.04E-05	6.63E-06	Tbx1/Sox1	19
GO:0001503	GO:0001503	ossification	23/389	394/23328	2.38E-07	1.04E-05	6.63E-06	Igfbp5/Id3	23
GO:0001933	GO:0001933	negative regulation of protein phosphorylation	24/389	425/23328	2.38E-07	1.04E-05	6.63E-06	Thy1/Nr2f	24
GO:0022407	GO:0022407	regulation of cell-cell adhesion	24/389	427/23328	2.59E-07	1.12E-05	7.15E-06	Dtx1/Thy1	24
GO:0035296	GO:0035296	regulation of tube diameter	15/389	176/23328	2.86E-07	1.21E-05	7.72E-06	Nts/Gja5/	15
GO:0097746	GO:0097746	regulation of blood vessel diameter	15/389	176/23328	2.86E-07	1.21E-05	7.72E-06	Nts/Gja5/	15
GO:0002683	GO:0002683	negative regulation of immune system process	25/389	462/23328	2.97E-07	1.23E-05	7.85E-06	Dtx1/Thy1	25
GO:0042692	GO:0042692	muscle cell differentiation	23/389	399/23328	2.97E-07	1.23E-05	7.85E-06	Cd9/Igfbp	23
GO:0035150	GO:0035150	regulation of tube size	15/389	177/23328	3.08E-07	1.27E-05	8.06E-06	Nts/Gja5/	15
GO:0035924	GO:0035924	cellular response to vascular endothelial growth factor	9/389	56/23328	3.45E-07	1.40E-05	8.93E-06	Flt4/Flt1/H	9
GO:0050727	GO:0050727	regulation of inflammatory response	22/389	372/23328	3.50E-07	1.41E-05	8.96E-06	Lbp/Fabp4	22
GO:0002064	GO:0002064	epithelial cell development	18/389	257/23328	3.62E-07	1.45E-05	9.20E-06	Cldn5/Col	18
GO:0003014	GO:0003014	renal system process	12/389	113/23328	4.24E-07	1.68E-05	1.07E-05	Gja5/Lgm	12
GO:0042493	GO:0042493	response to drug	18/389	260/23328	4.30E-07	1.68E-05	1.07E-05	Lcn2/Prnp	18
GO:0006979	GO:0006979	response to oxidative stress	23/389	409/23328	4.58E-07	1.78E-05	1.13E-05	Lcn2/Epas	23
GO:0043116	GO:0043116	negative regulation of vascular permeability	6/389	19/23328	4.67E-07	1.78E-05	1.13E-05	Cldn5/Apc	6
GO:0034109	GO:0034109	homotypic cell-cell adhesion	10/389	75/23328	4.68E-07	1.78E-05	1.13E-05	Cd9/Serpi	10
GO:0007162	GO:0007162	negative regulation of cell adhesion	19/389	290/23328	4.78E-07	1.80E-05	1.15E-05	Dtx1/Fgl2/	19
GO:0001945	GO:0001945	lymph vessel development	7/389	30/23328	4.97E-07	1.86E-05	1.18E-05	Nr2f2/Flt4	7
GO:0033002	GO:0033002	muscle cell proliferation	17/389	236/23328	5.17E-07	1.91E-05	1.22E-05	Igfbp5/Bm	17
GO:0072593	GO:0072593	reactive oxygen species metabolic process	19/389	292/23328	5.31E-07	1.95E-05	1.24E-05	Cd34/Ass	19
GO:1902904	GO:1902904	negative regulation of supramolecular fiber organizatio	14/389	163/23328	6.49E-07	2.36E-05	1.50E-05	Gmfg/Cdh	14
GO:0006898	GO:0006898	receptor-mediated endocytosis	17/389	240/23328	6.54E-07	2.36E-05	1.50E-05	Cd9/Cd36	17
GO:0030278	GO:0030278	regulation of ossification	17/389	241/23328	6.93E-07	2.48E-05	1.58E-05	Igfbp5/Id3	17
GO:0001937	GO:0001937	negative regulation of endothelial cell proliferation	8/389	45/23328	6.99E-07	2.48E-05	1.58E-05	Nr2f2/Rgc	8
GO:0051147	GO:0051147	regulation of muscle cell differentiation	14/389	165/23328	7.53E-07	2.64E-05	1.68E-05	Tbx1/Cxcl	14
GO:0110053	GO:0110053	regulation of actin filament organization	18/389	273/23328	8.74E-07	3.04E-05	1.94E-05	Rgcc/Gmf	18
GO:0051017	GO:0051017	actin filament bundle assembly	14/389	169/23328	1.00E-06	3.47E-05	2.21E-05	Rgcc/Id1/I	14
GO:0007178	GO:0007178	transmembrane receptor protein serine/threonine kinas	21/389	368/23328	1.15E-06	3.88E-05	2.47E-05	Cldn5/Id1/	21
GO:0003073	GO:0003073	regulation of systemic arterial blood pressure	12/389	124/23328	1.16E-06	3.88E-05	2.47E-05	Nr2f2/Cd3	12
GO:0003206	GO:0003206	cardiac chamber morphogenesis	13/389	147/23328	1.17E-06	3.88E-05	2.47E-05	Tbx1/Gja5	13
GO:0007043	GO:0007043	cell-cell junction assembly	13/389	147/23328	1.17E-06	3.88E-05	2.47E-05	Cldn5/Cd9	13
GO:0030195	GO:0030195	negative regulation of blood coagulation	8/389	48/23328	1.17E-06	3.88E-05	2.47E-05	Cd9/Cd34	8
GO:0030168	GO:0030168	platelet activation	10/389	83/23328	1.22E-06	4.01E-05	2.55E-05	Cd9/Serpi	10
GO:0061572	GO:0061572	actin filament bundle organization	14/389	172/23328	1.24E-06	4.04E-05	2.57E-05	Rgcc/Id1/I	14
GO:0090287	GO:0090287	regulation of cellular response to growth factor stimulu	18/389	281/23328	1.32E-06	4.28E-05	2.72E-05	Ptprf/Bmp	18
GO:0061028	GO:0061028	establishment of endothelial barrier	8/389	49/23328	1.38E-06	4.43E-05	2.82E-05	Cldn5/Cdh	8
GO:0001890	GO:0001890	placenta development	14/389	174/23328	1.43E-06	4.54E-05	2.88E-05	Nr2f2/Ptn/	14
GO:0001558	GO:0001558	regulation of cell growth	23/389	438/23328	1.49E-06	4.69E-05	2.98E-05	Igfbp5/Flt4	23
GO:0034110	GO:0034110	regulation of homotypic cell-cell adhesion	7/389	35/23328	1.53E-06	4.79E-05	3.04E-05	Cd9/Serpi	7
GO:0050819	GO:0050819	negative regulation of coagulation	8/389	50/23328	1.62E-06	5.04E-05	3.20E-05	Cd9/Cd34	8
GO:0003012	GO:0003012	muscle system process	22/389	408/23328	1.63E-06	5.04E-05	3.21E-05	Igfbp5/Gja	22
GO:0043393	GO:0043393	regulation of protein binding	16/389	231/23328	1.87E-06	5.73E-05	3.64E-05	Cldn5/Ptp	16
GO:0034754	GO:0034754	cellular hormone metabolic process	11/389	108/23328	1.96E-06	5.90E-05	3.75E-05	Spp1/Sca	11
GO:0060840	GO:0060840	artery development	11/389	108/23328	1.96E-06	5.90E-05	3.75E-05	Tbx1/Gja5	11

GO:0048678	GO:0048678	response to axon injury	9/389	69/23328	2.13E-06	6.37E-05	4.05E-05	Ptn/Gap43	9
GO:0097756	GO:0097756	negative regulation of blood vessel diameter	11/389	109/23328	2.14E-06	6.37E-05	4.05E-05	Gja5/Cav1	11
GO:0018108	GO:0018108	peptidyl-tyrosine phosphorylation	19/389	322/23328	2.29E-06	6.66E-05	4.23E-05	Reln/Thy1	19
GO:0002687	GO:0002687	positive regulation of leukocyte migration	13/389	156/23328	2.29E-06	6.66E-05	4.23E-05	Lbp/Thy1/	13
GO:0071248	GO:0071248	cellular response to metal ion	13/389	156/23328	2.29E-06	6.66E-05	4.23E-05	Fabp4/Prn	13
GO:0051149	GO:0051149	positive regulation of muscle cell differentiation	10/389	89/23328	2.34E-06	6.75E-05	4.29E-05	Tbx1/Cxcl	10
GO:0032103	GO:0032103	positive regulation of response to external stimulus	22/389	418/23328	2.42E-06	6.96E-05	4.42E-05	Lbp/Ccl21	22
GO:0031103	GO:0031103	axon regeneration	8/389	53/23328	2.56E-06	7.29E-05	4.64E-05	Ptn/Gap43	8
GO:1903035	GO:1903035	negative regulation of response to wounding	10/389	90/23328	2.59E-06	7.31E-05	4.65E-05	Cd9/Cd34	10
GO:0018212	GO:0018212	peptidyl-tyrosine modification	19/389	325/23328	2.62E-06	7.31E-05	4.65E-05	Reln/Thy1	19
GO:0070371	GO:0070371	ERK1 and ERK2 cascade	19/389	325/23328	2.62E-06	7.31E-05	4.65E-05	Ccl21a/Flt	19
GO:0003094	GO:0003094	glomerular filtration	6/389	25/23328	2.80E-06	7.76E-05	4.94E-05	Gja5/Adgr	6
GO:1903524	GO:1903524	positive regulation of blood circulation	10/389	91/23328	2.87E-06	7.89E-05	5.02E-05	Gja5/Adrb	10
GO:0061045	GO:0061045	negative regulation of wound healing	9/389	72/23328	3.06E-06	8.37E-05	5.32E-05	Cd9/Cd34	9
GO:0034329	GO:0034329	cell junction assembly	22/389	426/23328	3.29E-06	8.95E-05	5.69E-05	Reln/Thy1	22
GO:0031214	GO:0031214	biomineral tissue development	13/389	162/23328	3.48E-06	9.34E-05	5.94E-05	Srgn/Ptn/T	13
GO:0110148	GO:0110148	biomineralization	13/389	162/23328	3.48E-06	9.34E-05	5.94E-05	Srgn/Ptn/T	13
GO:0072012	GO:0072012	glomerulus vasculature development	6/389	26/23328	3.59E-06	9.50E-05	6.04E-05	Bmp4/Pdg	6
GO:0097205	GO:0097205	renal filtration	6/389	26/23328	3.59E-06	9.50E-05	6.04E-05	Gja5/Adgr	6
GO:0090257	GO:0090257	regulation of muscle system process	16/389	244/23328	3.80E-06	9.99E-05	6.35E-05	Igfbp5/Gja	16
GO:0045620	GO:0045620	negative regulation of lymphocyte differentiation	8/389	56/23328	3.93E-06	0.0001	6.50E-05	Dtx1/Fgl2/	8
GO:1904645	GO:1904645	response to amyloid-beta	7/389	40/23328	3.94E-06	0.0001	6.50E-05	Cd36/Prnp	7
GO:0061437	GO:0061437	renal system vasculature development	6/389	27/23328	4.55E-06	0.00012	7.42E-05	Bmp4/Pdg	6
GO:0061440	GO:0061440	kidney vasculature development	6/389	27/23328	4.55E-06	0.00012	7.42E-05	Bmp4/Pdg	6
GO:0042063	GO:0042063	gliogenesis	19/389	338/23328	4.63E-06	0.00012	7.49E-05	Dtx1/Reln	19
GO:1903522	GO:1903522	regulation of blood circulation	16/389	248/23328	4.68E-06	0.00012	7.53E-05	Epas1/Gja	16
GO:0070372	GO:0070372	regulation of ERK1 and ERK2 cascade	18/389	308/23328	4.82E-06	0.00012	7.71E-05	Ccl21a/Flt	18
GO:1902105	GO:1902105	regulation of leukocyte differentiation	18/389	309/23328	5.05E-06	0.00013	7.96E-05	Dtx1/Fgl2/	18
GO:0043534	GO:0043534	blood vessel endothelial cell migration	11/389	119/23328	5.07E-06	0.00013	7.96E-05	Rgcc/Hsp	11
GO:0003205	GO:0003205	cardiac chamber development	14/389	194/23328	5.12E-06	0.00013	7.96E-05	Tbx1/Gja5	14
GO:0001570	GO:0001570	vasculogenesis	10/389	97/23328	5.13E-06	0.00013	7.96E-05	Cd34/Sox	10
GO:0120162	GO:0120162	positive regulation of cold-induced thermogenesis	10/389	97/23328	5.13E-06	0.00013	7.96E-05	Lcn2/Epas	10
GO:0048844	GO:0048844	artery morphogenesis	9/389	77/23328	5.39E-06	0.00013	8.32E-05	Tbx1/Gja5	9
GO:0019935	GO:0019935	cyclic-nucleotide-mediated signaling	15/389	223/23328	5.60E-06	0.00014	8.59E-05	Cd36/Adrb	15
GO:0071900	GO:0071900	regulation of protein serine/threonine kinase activity	22/389	442/23328	5.93E-06	0.00014	9.04E-05	Thy1/Nr2f	22
GO:1903036	GO:1903036	positive regulation of response to wounding	9/389	78/23328	6.01E-06	0.00014	9.10E-05	Ptn/Cd36/	9
GO:0048608	GO:0048608	reproductive structure development	23/389	477/23328	6.14E-06	0.00015	9.24E-05	Nr2f2/Ptn/	23
GO:0007159	GO:0007159	leukocyte cell-cell adhesion	19/389	345/23328	6.21E-06	0.00015	9.30E-05	Dtx1/Thy1	19
GO:0001886	GO:0001886	endothelial cell morphogenesis	5/389	17/23328	6.59E-06	0.00015	9.81E-05	Col18a1/C	5
GO:0090303	GO:0090303	positive regulation of wound healing	8/389	60/23328	6.68E-06	0.00016	9.88E-05	Cd36/F11	8
GO:0061458	GO:0061458	reproductive system development	23/389	481/23328	7.03E-06	0.00016	0.0001	Nr2f2/Ptn/	23
GO:0030099	GO:0030099	myeloid cell differentiation	21/389	414/23328	7.25E-06	0.00017	0.00011	Epas1/Bm	21
GO:0031348	GO:0031348	negative regulation of defense response	15/389	228/23328	7.33E-06	0.00017	0.00011	Fgl2/Serpi	15
GO:0043114	GO:0043114	regulation of vascular permeability	7/389	44/23328	7.65E-06	0.00017	0.00011	Cldn5/Cdh	7
GO:0042310	GO:0042310	vasoconstriction	10/389	103/23328	8.81E-06	0.0002	0.00013	Gja5/Cav1	10
GO:0001569	GO:0001569	branching involved in blood vessel morphogenesis	7/389	45/23328	8.93E-06	0.0002	0.00013	Flt1/Tbx1/	7
GO:1990000	GO:1990000	amyloid fibril formation	5/389	18/23328	9.00E-06	0.0002	0.00013	Cd36/B2m	5
GO:0052547	GO:0052547	regulation of peptidase activity	21/389	421/23328	9.35E-06	0.00021	0.00013	Serpine2/S	21
GO:1902106	GO:1902106	negative regulation of leukocyte differentiation	10/389	104/23328	9.61E-06	0.00021	0.00014	Dtx1/Fgl2/	10
GO:0045926	GO:0045926	negative regulation of growth	16/389	264/23328	1.03E-05	0.00023	0.00014	Igfbp5/Ser	16
GO:0006469	GO:0006469	negative regulation of protein kinase activity	14/389	207/23328	1.08E-05	0.00024	0.00015	Thy1/Nr2f	14
GO:0070830	GO:0070830	bicellular tight junction assembly	9/389	84/23328	1.11E-05	0.00024	0.00015	Cldn5/Cld	9
GO:0031532	GO:0031532	actin cytoskeleton reorganization	10/389	106/23328	1.14E-05	0.00025	0.00016	Gmfg/Esa	10
GO:0010544	GO:0010544	negative regulation of platelet activation	5/389	19/23328	1.21E-05	0.00026	0.00017	Cd9/Serpi	5
GO:0001893	GO:0001893	maternal placenta development	6/389	32/23328	1.30E-05	0.00028	0.00018	Nr2f2/Ptn/	6
GO:0120192	GO:0120192	tight junction assembly	9/389	86/23328	1.35E-05	0.00029	0.00018	Cldn5/Cld	9
GO:1903707	GO:1903707	negative regulation of hemopoiesis	12/389	157/23328	1.36E-05	0.00029	0.00018	Dtx1/Fgl2/	12
GO:0048251	GO:0048251	elastic fiber assembly	4/389	10/23328	1.48E-05	0.00031	0.0002	Thsd4/Mfa	4
GO:1990845	GO:1990845	adaptive thermogenesis	12/389	159/23328	1.54E-05	0.00032	0.00021	Lcn2/Epas	12
GO:0048732	GO:0048732	gland development	22/389	471/23328	1.60E-05	0.00033	0.00021	Igfbp5/Ho	22
GO:0002262	GO:0002262	myeloid cell homeostasis	13/389	187/23328	1.66E-05	0.00034	0.00022	Epas1/Bm	13
GO:0043254	GO:0043254	regulation of protein-containing complex assembly	21/389	438/23328	1.69E-05	0.00035	0.00022	Gmfg/Cdc	21
GO:0019934	GO:0019934	cGMP-mediated signaling	6/389	34/23328	1.87E-05	0.00038	0.00024	Cd36/Apo	6
GO:1904646	GO:1904646	cellular response to amyloid-beta	6/389	34/23328	1.87E-05	0.00038	0.00024	Cd36/Prnp	6
GO:0046697	GO:0046697	decidualization	5/389	21/23328	2.05E-05	0.00042	0.00027	Ptn/Tppp3	5
GO:0050808	GO:0050808	synapse organization	22/389	479/23328	2.07E-05	0.00042	0.00027	Reln/Srgn	22
GO:0120193	GO:0120193	tight junction organization	9/389	91/23328	2.14E-05	0.00043	0.00027	Cldn5/Cld	9
GO:0060485	GO:0060485	mesenchyme development	16/389	281/23328	2.23E-05	0.00045	0.00028	Rgcc/Tcf1	16
GO:0007568	GO:0007568	aging	15/389	251/23328	2.29E-05	0.00046	0.00029	Prelp/Prnp	15
GO:0052548	GO:0052548	regulation of endopeptidase activity	18/389	346/23328	2.33E-05	0.00046	0.00029	Serpine2/S	18
GO:0001819	GO:0001819	positive regulation of cytokine production	21/389	449/23328	2.44E-05	0.00048	0.00031	Lbp/Rgcc/	21
GO:0009636	GO:0009636	response to toxic substance	10/389	116/23328	2.52E-05	0.0005	0.00031	Lcn2/Ephx	10
GO:0043297	GO:0043297	apical junction assembly	9/389	93/23328	2.54E-05	0.0005	0.00032	Cldn5/Cld	9
GO:0045123	GO:0045123	cellular extravasation	8/389	72/23328	2.62E-05	0.00051	0.00033	Thy1/Cxcl	8
GO:0051146	GO:0051146	striated muscle cell differentiation	17/389	317/23328	2.67E-05	0.00052	0.00033	Cd9/Igfbp	17
GO:0048762	GO:0048762	mesenchymal cell differentiation	14/389	225/23328	2.74E-05	0.00053	0.00034	Rgcc/Tbx1	14
GO:0051495	GO:0051495	positive regulation of cytoskeleton organization	14/389	225/23328	2.74E-05	0.00053	0.00034	Rgcc/Gmf	14
GO:1904035	GO:1904035	regulation of epithelial cell apoptotic process	9/389	94/23328	2.77E-05	0.00053	0.00034	Rgcc/Col1	9
GO:0051155	GO:0051155	positive regulation of striated muscle cell differentiatior	8/389	73/23328	2.91E-05	0.00055	0.00035	Tbx1/Cxcl	8
GO:0032271	GO:0032271	regulation of protein polymerization	14/389	227/23328	3.02E-05	0.00057	0.00036	Gmfg/Cdc	14
GO:0033673	GO:0033673	negative regulation of kinase activity	14/389	227/23328	3.02E-05	0.00057	0.00036	Thy1/Nr2f	14
GO:1904705	GO:1904705	regulation of vascular associated smooth muscle cell p	7/389	54/23328	3.06E-05	0.00057	0.00036	Igfbp5/Pd	7
GO:1990874	GO:1990874	vascular associated smooth muscle cell proliferation	7/389	54/23328	3.06E-05	0.00057	0.00036	Igfbp5/Pd	7
GO:0106106	GO:0106106	cold-induced thermogenesis	11/389	144/23328	3.11E-05	0.00058	0.00037	Lcn2/Epas	11
GO:0120161	GO:0120161	regulation of cold-induced thermogenesis	11/389	144/23328	3.11E-05	0.00058	0.00037	Lcn2/Epas	11
GO:0030282	GO:0030282	bone mineralization	10/389	120/23328	3.37E-05	0.00062	0.0004	Srgn/Ptn/	10
GO:0010232	GO:0010232	vascular transport	4/389	12/23328	3.39E-05	0.00062	0.0004	Cd36/Fab	4
GO:0051348	GO:0051348	negative regulation of transferase activity	15/389	260/23328	3.44E-05	0.00063	0.0004	Thy1/Nr2f	15
GO:2000351	GO:2000351	regulation of endothelial cell apoptotic process	7/389	55/23328	3.46E-05	0.00063	0.0004	Rgcc/Col1	7
GO:0038084	GO:0038084	vascular endothelial growth factor signaling pathway	6/389	38/23328	3.64E-05	0.00065	0.00042	Flt4/Hspb	6
GO:0045601	GO:0045601	regulation of endothelial cell differentiation	6/389	38/23328	3.64E-05	0.00065	0.00042	Cldn5/Id1/	6
GO:2000249	GO:2000249	regulation of actin cytoskeleton reorganization	6/389	38/23328	3.64E-05	0.00065	0.00042	Gmfg/Esa	6
GO:0010959	GO:0010959	regulation of metal ion transport	20/389	427/23328	3.73E-05	0.00066	0.00042	Thy1/Fxyd	20
GO:0000302	GO:0000302	response to reactive oxygen species	13/389	202/23328	3.73E-05	0.00066	0.00042	Lcn2/Cd3	13
GO:0003231	GO:0003231	cardiac ventricle development	11/389	147/23328	3.76E-05	0.00067	0.00042	Gja5/Bmp	11
GO:0045807	GO:0045807	positive regulation of endocytosis	10/389	122/23328	3.89E-05	0.00068	0.00043	Lbp/Cd36/	10
GO:0045777	GO:0045777	positive regulation of blood pressure	7/389	56/23328	3.90E-05	0.00068	0.00043	Nr2f2/Eph	7
GO:0050732	GO:0050732	negative regulation of peptidyl-tyrosine phosphorylatior	7/389	56/23328	3.90E-05	0.00068	0.00043	Thy1/Socs	7
GO:0050730	GO:0050730	regulation of peptidyl-tyrosine phosphorylation	15/389	263/23328	3.93E-05	0.00068	0.00044	Reln/Thy1	15
GO:0090330	GO:0090330	regulation of platelet aggregation	5/389	24/23328	4.11E-05	0.00071	0.00045	Cd9/Serpi	5
GO:0070167	GO:0070167	regulation of biomineral tissue development	9/389	99/23328	4.20E-05	0.00072	0.00046	Srgn/Ptn/	9
GO:0110149	GO:0110149	regulation of biomineralization	9/389	99/23328	4.20E-05	0.00072	0.00046	Srgn/Ptn/	9
GO:0034101	GO:0034101	erythrocyte homeostasis	11/389	149/23328	4.26E-05	0.00073	0.00046	Epas1/Bm	11
GO:0050921	GO:0050921	positive regulation of chemotaxis	11/389	149/23328	4.26E-05	0.00073	0.00046	Lbp/Ccl21	11
GO:0001649	GO:0001649	osteoblast differentiation	13/389	205/23328	4.34E-05	0.00073	0.00047	Igfbp5/Id3	13
GO:0045216	GO:0045216	cell-cell junction organization	13/389	205/23328	4.34E-05	0.00073	0.00047	Cldn5/Cd9	13
GO:0019216	GO:0019216	regulation of lipid metabolic process	18/389	363/23328	4.37E-05	0.00073	0.00047	Flt1/Ephx2	18
GO:0002548	GO:0002548	monocyte chemotaxis	7/389	57/23328	4.38E-05	0.00073	0.00047	Ccl21a/Flt	7
GO:0051153	GO:0051153	regulation of striated muscle cell differentiation	10/389	124/23328	4.47E-05	0.00075	0.00047	Tbx1/Cxcl	10
GO:0034614	GO:0034614	cellular response to reactive oxygen species	11/389	150/23328	4.53E-05	0.00075	0.00048	Lcn2/Cd3	11
GO:0019229	GO:0019229	regulation of vasoconstriction	8/389	78/23328	4.71E-05	0.00078	0.0005	Gja5/Cav1	8
GO:1903409	GO:1903409	reactive oxygen species biosynthetic process	10/389	125/23328	4.79E-05	0.00079	0.0005	Cd34/Ass	10
GO:2000377	GO:2000377	regulation of reactive oxygen species metabolic proces	13/389	207/23328	4.80E-05	0.00079	0.0005	Cd34/Ass	13
GO:0003093	GO:0003093	regulation of glomerular filtration	4/389	13/23328	4.83E-05	0.00079	0.0005	Gja5/Pdgf	4
GO:0072577	GO:0072577	endothelial cell apoptotic process	7/389	58/23328	4.91E-05	0.0008	0.00051	Rgcc/Col1	7
GO:0032092	GO:0032092	positive regulation of protein binding	9/389	101/23328	4.92E-05	0.0008	0.00051	Cldn5/Bm	9
GO:0034368	GO:0034368	protein-lipid complex remodeling	5/389	25/23328	5.07E-05	0.00082	0.00052	Pltp/Apoe/	5
GO:0034369	GO:0034369	plasma lipoprotein particle remodeling	5/389	25/23328	5.07E-05	0.00082	0.00052	Pltp/Apoe/	5
GO:0001659	GO:0001659	temperature homeostasis	12/389	181/23328	5.54E-05	0.00089	0.00057	Lcn2/Epas	12
GO:0006874	GO:0006874	cellular calcium ion homeostasis	21/389	476/23328	5.68E-05	0.00091	0.00058	Thy1/Cd3	21
GO:0022612	GO:0022612	gland morphogenesis	11/389	154/23328	5.75E-05	0.00092	0.00058	Igfbp5/Ptn	11
GO:0010951	GO:0010951	negative regulation of endopeptidase activity	12/389	182/23328	5.85E-05	0.00093	0.00059	Serpine2/S	12
GO:0002791	GO:0002791	regulation of peptide secretion	19/389	406/23328	5.88E-05	0.00093	0.00059	Rgcc/Srgn	19
GO:0008016	GO:0008016	regulation of heart contraction	12/389	183/23328	6.16E-05	0.00095	0.00061	Epas1/Gja	12
GO:0000303	GO:0000303	response to superoxide	5/389	26/23328	6.19E-05	0.00095	0.00061	Cd36/Prdx	5
GO:0007263	GO:0007263	nitric oxide mediated signal transduction	5/389	26/23328	6.19E-05	0.00095	0.00061	Cd36/Apo	5
GO:0042573	GO:0042573	retinoic acid metabolic process	5/389	26/23328	6.19E-05	0.00095	0.00061	Adh7/Rbp	5
GO:0044342	GO:0044342	type B pancreatic cell proliferation	5/389	26/23328	6.19E-05	0.00095	0.00061	Igfbp5/Nr4	5
GO:0050927	GO:0050927	positive regulation of positive chemotaxis	5/389	26/23328	6.19E-05	0.00095	0.00061	Ccl21a/Cx	5

GO:0055094	GO:0055094	response to lipoprotein particle	5/389	26/23328	6.19E-05	0.00095	0.00061	Cd9/Cd36	5
GO:0050728	GO:0050728	negative regulation of inflammatory response	11/389	156/23328	6.47E-05	0.00099	0.00063	Cd200/Ad	11
GO:0030098	GO:0030098	lymphocyte differentiation	19/389	409/23328	6.48E-05	0.00099	0.00063	Dtx1/Fgl2/	19
GO:0046890	GO:0046890	regulation of lipid biosynthetic process	12/389	184/23328	6.50E-05	0.00099	0.00063	Igfbp7/Fal	12
GO:1905954	GO:1905954	positive regulation of lipid localization	9/389	105/23328	6.67E-05	0.00101	0.00064	Cd36/Pltp	9
GO:0010644	GO:0010644	cell communication by electrical coupling	4/389	14/23328	6.68E-05	0.00101	0.00064	Gja5/Ank3	4
GO:0030500	GO:0030500	regulation of bone mineralization	8/389	82/23328	6.76E-05	0.00102	0.00065	Srgn/Ptn/	8
GO:0050807	GO:0050807	regulation of synapse organization	15/389	277/23328	7.08E-05	0.00106	0.00067	Reln/Srgn	15
GO:0048640	GO:0048640	negative regulation of developmental growth	10/389	131/23328	7.13E-05	0.00106	0.00068	Lgmn/Adr	10
GO:0010466	GO:0010466	negative regulation of peptidase activity	14/389	246/23328	7.24E-05	0.00108	0.00068	Serpine2/	14
GO:0051899	GO:0051899	membrane depolarization	8/389	83/23328	7.38E-05	0.00109	0.00069	Gja5/Ank3	8
GO:0000305	GO:0000305	response to oxygen radical	5/389	27/23328	7.50E-05	0.00109	0.00069	Cd36/Prdx	5
GO:0030194	GO:0030194	positive regulation of blood coagulation	5/389	27/23328	7.50E-05	0.00109	0.00069	Cd36/F11	5
GO:0034367	GO:0034367	protein-containing complex remodeling	5/389	27/23328	7.50E-05	0.00109	0.00069	Pltp/Apoe	5
GO:0050926	GO:0050926	regulation of positive chemotaxis	5/389	27/23328	7.50E-05	0.00109	0.00069	Ccl21a/Cx	5
GO:1900048	GO:1900048	positive regulation of hemostasis	5/389	27/23328	7.50E-05	0.00109	0.00069	Cd36/F11	5
GO:0050709	GO:0050709	negative regulation of protein secretion	9/389	107/23328	7.73E-05	0.00112	0.00071	Rgcc/Srgr	9
GO:0003151	GO:0003151	outflow tract morphogenesis	8/389	84/23328	8.04E-05	0.00116	0.00074	Tbx1/Sox	8
GO:0010001	GO:0010001	glial cell differentiation	14/389	249/23328	8.24E-05	0.00118	0.00075	Dtx1/Reln	14
GO:0015850	GO:0015850	organic hydroxy compound transport	14/389	249/23328	8.24E-05	0.00118	0.00075	Cd36/Cxc	14
GO:0002695	GO:0002695	negative regulation of leukocyte activation	12/389	189/23328	8.40E-05	0.0012	0.00076	Dtx1/Fgl2/	12
GO:0071280	GO:0071280	cellular response to copper ion	4/389	15/23328	8.98E-05	0.00128	0.00081	Prnp/Aqp1	4
GO:0071402	GO:0071402	cellular response to lipoprotein particle stimulus	5/389	28/23328	9.00E-05	0.00128	0.00081	Cd9/Cd36	5
GO:0055074	GO:0055074	calcium ion homeostasis	21/389	493/23328	9.31E-05	0.00132	0.00084	Thy1/Cd3	21
GO:0045444	GO:0045444	fat cell differentiation	14/389	252/23328	9.36E-05	0.00132	0.00084	Fabp4/Ad	14
GO:0022408	GO:0022408	negative regulation of cell-cell adhesion	12/389	192/23328	9.77E-05	0.00137	0.00087	Dtx1/Fgl2/	12
GO:0045619	GO:0045619	regulation of lymphocyte differentiation	12/389	192/23328	9.77E-05	0.00137	0.00087	Dtx1/Fgl2/	12
GO:0030100	GO:0030100	regulation of endocytosis	13/389	222/23328	9.79E-05	0.00137	0.00087	Lbp/Cd36/	13
GO:0048015	GO:0048015	phosphatidylinositol-mediated signaling	11/389	164/23328	0.000101453	0.00141	0.0009	Reln/Flt1/	11
GO:1903531	GO:1903531	negative regulation of secretion by cell	12/389	193/23328	0.00010259	0.00142	0.0009	Rgcc/Srgr	12
GO:0032963	GO:0032963	collagen metabolic process	9/389	111/23328	0.000102809	0.00142	0.0009	Rgcc/Id1/	9
GO:0042246	GO:0042246	tissue regeneration	7/389	65/23328	0.000102931	0.00142	0.0009	Cd9/Ptn/G	7
GO:0050920	GO:0050920	regulation of chemotaxis	13/389	224/23328	0.000107117	0.00147	0.00093	Lbp/Ccl21	13
GO:1901615	GO:1901615	organic hydroxy compound metabolic process	21/389	498/23328	0.000107165	0.00147	0.00093	Reln/Epas	21
GO:0051098	GO:0051098	regulation of binding	18/389	390/23328	0.000108983	0.00148	0.00094	Cldn5/Ptp	18
GO:0003254	GO:0003254	regulation of membrane depolarization	6/389	46/23328	0.000110286	0.00149	0.00095	Gja5/Ank3	6
GO:0030218	GO:0030218	erythrocyte differentiation	10/389	138/23328	0.00011032	0.00149	0.00095	Epas1/Bm	10
GO:0007565	GO:0007565	female pregnancy	11/389	166/23328	0.000113024	0.00152	0.00097	Nr2f2/Igfb	11
GO:0097006	GO:0097006	regulation of plasma lipoprotein particle levels	7/389	66/23328	0.000113514	0.00152	0.00097	Cd36/Pltp	7
GO:0050803	GO:0050803	regulation of synapse structure or activity	15/389	289/23328	0.000113538	0.00152	0.00097	Reln/Srgn	15
GO:0001977	GO:0001977	renal system process involved in regulation of blood vo	4/389	16/23328	0.000118216	0.00157	0.001	Gja5/Pdgf	4
GO:0071404	GO:0071404	cellular response to low-density lipoprotein particle stim	4/389	16/23328	0.000118216	0.00157	0.001	Cd9/Cd36	4
GO:1902430	GO:1902430	negative regulation of amyloid-beta formation	4/389	16/23328	0.000118216	0.00157	0.001	Prnp/Apoe	4
GO:0007519	GO:0007519	skeletal muscle tissue development	12/389	196/23328	0.000118717	0.00157	0.001	Nr2f2/Hox	12
GO:0048017	GO:0048017	inositol lipid-mediated signaling	11/389	167/23328	0.00011922	0.00157	0.001	Reln/Flt1/	11
GO:0048738	GO:0048738	cardiac muscle tissue development	14/389	258/23328	0.000119986	0.00158	0.001	Gja5/Bmp	14
GO:0071674	GO:0071674	mononuclear cell migration	8/389	89/23328	0.000121134	0.00159	0.00101	Ccl21a/Flt	8
GO:0071241	GO:0071241	cellular response to inorganic substance	13/389	227/23328	0.000122357	0.0016	0.00102	Fabp4/Prr	13
GO:0044706	GO:0044706	multi-multicellular organism process	12/389	197/23328	0.000124555	0.00161	0.00102	Nr2f2/Igfb	12
GO:0010718	GO:0010718	positive regulation of epithelial to mesenchymal transit	6/389	47/23328	0.000124666	0.00161	0.00102	Rgcc/Bmp	6
GO:0043277	GO:0043277	apoptotic cell clearance	6/389	47/23328	0.000124666	0.00161	0.00102	Cd36/Sca	6
GO:0060135	GO:0060135	maternal process involved in female pregnancy	6/389	47/23328	0.000124666	0.00161	0.00102	Nr2f2/Ptn/	6
GO:0030947	GO:0030947	regulation of vascular endothelial growth factor recepto	5/389	30/23328	0.000127012	0.00163	0.00103	Bmp4/Mm	5
GO:0050820	GO:0050820	positive regulation of coagulation	5/389	30/23328	0.000127012	0.00163	0.00103	Cd36/F11	5
GO:0001818	GO:0001818	negative regulation of cytokine production	14/389	260/23328	0.000130111	0.00166	0.00106	Lbp/Rgcc/	14
GO:0007179	GO:0007179	transforming growth factor beta receptor signaling path	11/389	169/23328	0.000132483	0.00169	0.00107	Cldn5/Lrg	11
GO:0030308	GO:0030308	negative regulation of cell growth	12/389	199/23328	0.000136973	0.00174	0.00111	Serpine2/	12
GO:0045907	GO:0045907	positive regulation of vasoconstriction	6/389	48/23328	0.000140493	0.00177	0.00113	Gja5/Cav1	6
GO:0070527	GO:0070527	platelet aggregation	6/389	48/23328	0.000140493	0.00177	0.00113	Cd9/Serpi	6
GO:0048644	GO:0048644	muscle organ morphogenesis	8/389	91/23328	0.00014162	0.00178	0.00113	Tcf15/Tbx	8
GO:0001906	GO:0001906	cell killing	12/389	200/23328	0.00014357	0.00179	0.00114	Nts/H2-Q6	12
GO:1902905	GO:1902905	positive regulation of supramolecular fiber organization	12/389	200/23328	0.00014357	0.00179	0.00114	Rgcc/Gmf	12
GO:0002792	GO:0002792	negative regulation of peptide secretion	9/389	116/23328	0.00014421	0.00179	0.00114	Rgcc/Srgr	9
GO:0019882	GO:0019882	antigen processing and presentation	9/389	116/23328	0.00014421	0.00179	0.00114	Fgl2/H2-Q	9
GO:0051494	GO:0051494	negative regulation of cytoskeleton organization	11/389	171/23328	0.000146982	0.00182	0.00116	Gmfg/Cdh	11
GO:0007204	GO:0007204	positive regulation of cytosolic calcium ion concentratio	16/389	330/23328	0.000149032	0.00183	0.00117	Thy1/Cd3	16
GO:1901652	GO:1901652	response to peptide	18/389	400/23328	0.000149326	0.00183	0.00117	Ptprf/Cd3	18
GO:0032967	GO:0032967	positive regulation of collagen biosynthetic process	5/389	31/23328	0.000149379	0.00183	0.00117	Rgcc/Bmp	5
GO:0045428	GO:0045428	regulation of nitric oxide biosynthetic process	7/389	69/23328	0.000150675	0.00184	0.00117	Cd34/Ass	7
GO:0050708	GO:0050708	regulation of protein secretion	17/389	365/23328	0.000151062	0.00184	0.00117	Rgcc/Srgr	17
GO:0071901	GO:0071901	negative regulation of protein serine/threonine kinase a	9/389	117/23328	0.000153969	0.00187	0.00119	Nr2f2/Bmp	9
GO:0006936	GO:0006936	muscle contraction	15/389	298/23328	0.000158943	0.00193	0.00123	Gja5/Adrb	15
GO:1904019	GO:1904019	epithelial cell apoptotic process	9/389	118/23328	0.000164273	0.00199	0.00126	Rgcc/Col1	9
GO:0007611	GO:0007611	learning or memory	15/389	299/23328	0.000164848	0.00199	0.00126	Reln/Ptn/	15
GO:0045860	GO:0045860	positive regulation of protein kinase activity	19/389	440/23328	0.000167506	0.00201	0.00128	Reln/Rgcc	19
GO:0019221	GO:0019221	cytokine-mediated signaling pathway	17/389	369/23328	0.0001719	0.00206	0.00131	Ccl21a/Pt	17
GO:0051346	GO:0051346	negative regulation of hydrolase activity	18/389	405/23328	0.000174022	0.00208	0.00132	Serpine2/	18
GO:0010043	GO:0010043	response to zinc ion	5/389	32/23328	0.000174636	0.00208	0.00132	Ass1/Crip	5
GO:0010717	GO:0010717	regulation of epithelial to mesenchymal transition	8/389	94/23328	0.000177638	0.00211	0.00134	Rgcc/Bmp	8
GO:0055001	GO:0055001	muscle cell development	12/389	205/23328	0.000180789	0.00214	0.00136	Bmp4/Rgs	12
GO:0060538	GO:0060538	skeletal muscle organ development	12/389	206/23328	0.000189146	0.00223	0.00142	Nr2f2/Hox	12
GO:0009612	GO:0009612	response to mechanical stimulus	11/389	176/23328	0.000189248	0.00223	0.00142	Ptn/Cd36/	11
GO:0045603	GO:0045603	positive regulation of endothelial cell differentiation	4/389	18/23328	0.000193579	0.00227	0.00144	Cldn5/Bm	4
GO:1902992	GO:1902992	negative regulation of amyloid precursor protein catabo	4/389	18/23328	0.000193579	0.00227	0.00144	Prnp/Apoe	4
GO:0010633	GO:0010633	negative regulation of epithelial cell migration	7/389	72/23328	0.000197124	0.0023	0.00146	Nr2f2/Rgc	7
GO:0098754	GO:0098754	detoxification	6/389	51/23328	0.000197701	0.0023	0.00146	Cd36/Prdx	6
GO:0031032	GO:0031032	actomyosin structure organization	12/389	207/23328	0.000197831	0.0023	0.00146	Rgcc/F11r	12
GO:0050999	GO:0050999	regulation of nitric-oxide synthase activity	5/389	33/23328	0.000203026	0.00235	0.00149	Cav1/Apo	5
GO:0071560	GO:0071560	cellular response to transforming growth factor beta sti	12/389	208/23328	0.000206853	0.00239	0.00152	Cldn5/Lrg	12
GO:0051480	GO:0051480	regulation of cytosolic calcium ion concentration	17/389	375/23328	0.00020786	0.00239	0.00152	Thy1/Cd3	17
GO:0098742	GO:0098742	cell-cell adhesion via plasma-membrane adhesion mol	12/389	209/23328	0.000216224	0.00248	0.00158	Ptprf/Spar	12
GO:1901861	GO:1901861	regulation of muscle tissue development	11/389	180/23328	0.000230093	0.00263	0.00167	Igfbp5/Tbx	11
GO:0051090	GO:0051090	regulation of DNA-binding transcription factor activity	18/389	415/23328	0.000234346	0.00267	0.0017	Reln/Rgcc	18
GO:0010714	GO:0010714	positive regulation of collagen metabolic process	5/389	34/23328	0.000234799	0.00267	0.0017	Rgcc/Bmp	5
GO:0030279	GO:0030279	negative regulation of ossification	8/389	98/23328	0.000237058	0.00268	0.00171	Igfbp5/Id3	8
GO:0060395	GO:0060395	SMAD protein signal transduction	8/389	98/23328	0.000237058	0.00268	0.00171	Bmp4/Vim	8
GO:0034599	GO:0034599	cellular response to oxidative stress	14/389	276/23328	0.000241379	0.00272	0.00173	Lcn2/Epas	14
GO:0010596	GO:0010596	negative regulation of endothelial cell migration	6/389	53/23328	0.000245062	0.00276	0.00175	Nr2f2/Rgc	6
GO:0043112	GO:0043112	receptor metabolic process	12/389	212/23328	0.000246537	0.00276	0.00175	Cd9/Cd36	12
GO:0071559	GO:0071559	response to transforming growth factor beta	12/389	212/23328	0.000246537	0.00276	0.00175	Cldn5/Lrg	12
GO:0014066	GO:0014066	regulation of phosphatidylinositol 3-kinase signaling	8/389	99/23328	0.00025421	0.00283	0.0018	Reln/Flt1/	8
GO:0014743	GO:0014743	regulation of muscle hypertrophy	7/389	75/23328	0.0002545	0.00283	0.0018	Igfbp5/Fbx	7
GO:0051145	GO:0051145	smooth muscle cell differentiation	7/389	75/23328	0.0002545	0.00283	0.0018	Bmp4/Pdg	7
GO:0001654	GO:0001654	eye development	18/389	418/23328	0.000255685	0.00283	0.0018	Maf/Thy1/	18
GO:0002428	GO:0002428	antigen processing and presentation of peptide antiger	5/389	35/23328	0.000270215	0.00297	0.00189	H2-Q6/B2	5
GO:0046686	GO:0046686	response to cadmium ion	5/389	35/23328	0.000270215	0.00297	0.00189	Prnp/Jun/	5
GO:0008360	GO:0008360	regulation of cell shape	10/389	154/23328	0.000270727	0.00297	0.00189	Ptn/Lst1/	10
GO:0042743	GO:0042743	hydrogen peroxide metabolic process	6/389	54/23328	0.000271866	0.00298	0.00189	Duoxa2/P	6
GO:0150063	GO:0150063	visual system development	18/389	422/23328	0.000286762	0.00313	0.00199	Maf/Thy1/	18
GO:0048754	GO:0048754	branching morphogenesis of an epithelial tube	11/389	185/23328	0.000291413	0.00317	0.00201	Flt1/Tbx1/	11
GO:0071456	GO:0071456	cellular response to hypoxia	8/389	101/23328	0.000291564	0.00317	0.00201	Rgcc/Epa	8
GO:0070374	GO:0070374	positive regulation of ERK1 and ERK2 cascade	12/389	216/23328	0.000292519	0.00317	0.00202	Ccl21a/Flt	12
GO:0001946	GO:0001946	lymphangiogenesis	4/389	20/23328	0.00029852	0.00323	0.00205	Flt4/Prox1	4
GO:0006809	GO:0006809	nitric oxide biosynthetic process	7/389	77/23328	0.000299703	0.00323	0.00205	Cd34/Ass	7
GO:0010712	GO:0010712	regulation of collagen metabolic process	6/389	55/23328	0.000300918	0.00323	0.00206	Rgcc/Bmp	6
GO:0034381	GO:0034381	plasma lipoprotein particle clearance	5/389	36/23328	0.000309542	0.00331	0.0021	Cd36/Anx	5
GO:1904707	GO:1904707	positive regulation of vascular associated smooth mus	5/389	36/23328	0.000309542	0.00331	0.0021	Igfbp5/Pd	5
GO:0048545	GO:0048545	response to steroid hormone	12/389	218/23328	0.000318114	0.00339	0.00216	Ass1/Bmp	12
GO:0014065	GO:0014065	phosphatidylinositol 3-kinase signaling	9/389	129/23328	0.000320837	0.00341	0.00217	Reln/Flt1/	9
GO:0048880	GO:0048880	sensory system development	18/389	426/23328	0.000321078	0.00341	0.00217	Maf/Thy1/	18
GO:0003208	GO:0003208	cardiac ventricle morphogenesis	7/389	78/23328	0.000324604	0.00343	0.00218	Sfrp2/Prox	7
GO:0030199	GO:0030199	collagen fibril organization	6/389	56/23328	0.000332349	0.0035	0.00223	Sfrp2/Anx	6
GO:0051250	GO:0051250	negative regulation of lymphocyte activation	10/389	158/23328	0.000332505	0.0035	0.00223	Dtx1/Fgl2/	10
GO:0072676	GO:0072676	lymphocyte migration	8/389	103/23328	0.000333285	0.0035	0.00223	Ccl21a/Cc	8
GO:0002691	GO:0002691	regulation of cellular extravasation	5/389	37/23328	0.000353055	0.0037	0.00235	Thy1/Cxcl	5
GO:0046849	GO:0046849	bone remodeling	8/389	104/23328	0.0003559	0.00372	0.00236	Ptn/Adrb2	8
GO:0060047	GO:0060047	heart contraction	12/389	221/23328	0.000360054	0.00375	0.00239	Epas1/Gja	12
GO:0019430	GO:0019430	removal of superoxide radicals	4/389	21/23328	0.000363932	0.00378	0.00241	Cd36/Prdx	4
GO:0030324	GO:0030324	lung development	12/389	222/23328	0.000375035	0.00389	0.00247	Igfbp5/Spa	12

GO:0042176	GO:0042176	regulation of protein catabolic process	17/389	395/23328	0.000379252	0.00392	0.00249	Serpine2/	17
GO:0009306	GO:0009306	protein secretion	19/389	470/23328	0.000382514	0.00395	0.00251	Rgcc/Srgr	19
GO:0051048	GO:0051048	negative regulation of secretion	12/389	223/23328	0.000390539	0.00402	0.00255	Rgcc/Srgr	12
GO:0035592	GO:0035592	establishment of protein localization to extracellular reg	19/389	471/23328	0.000392624	0.00403	0.00256	Rgcc/Srgr	19
GO:0010831	GO:0010831	positive regulation of myotube differentiation	5/389	38/23328	0.000401038	0.00411	0.00261	Tbx1/Cxcl	5
GO:0001755	GO:0001755	neural crest cell migration	6/389	58/23328	0.00040289	0.00411	0.00262	Tbx1/Bmp	6
GO:0035282	GO:0035282	segmentation	8/389	106/23328	0.000404882	0.00412	0.00262	Tcf15/Bmp	8
GO:0046209	GO:0046209	nitric oxide metabolic process	7/389	81/23328	0.000409406	0.00416	0.00264	Cd34/Ass	7
GO:0030323	GO:0030323	respiratory tube development	12/389	225/23328	0.000423178	0.00429	0.00273	Igfbp5/Sp	12
GO:0055076	GO:0055076	transition metal ion homeostasis	9/389	134/23328	0.000424717	0.00429	0.00273	Cp/Lcn2/E	9
GO:0045165	GO:0045165	cell fate commitment	14/389	292/23328	0.000426309	0.0043	0.00273	Nr2f2/Epa	14
GO:0014897	GO:0014897	striated muscle hypertrophy	8/389	107/23328	0.000431351	0.00432	0.00275	Igfbp5/Fb	8
GO:0110020	GO:0110020	regulation of actomyosin structure organization	8/389	107/23328	0.000431351	0.00432	0.00275	Rgcc/F11	8
GO:1903426	GO:1903426	regulation of reactive oxygen species biosynthetic proc	8/389	107/23328	0.000431351	0.00432	0.00275	Cd34/Ass	8
GO:2000353	GO:2000353	positive regulation of endothelial cell apoptotic process	4/389	22/23328	0.000438986	0.00438	0.00279	Rgcc/Col1	4
GO:0051258	GO:0051258	protein polymerization	14/389	293/23328	0.000441073	0.00439	0.00279	Gmfg/Cdc	14
GO:0051224	GO:0051224	negative regulation of protein transport	10/389	164/23328	0.000446925	0.00444	0.00282	Rgcc/Srgr	10
GO:0002475	GO:0002475	antigen processing and presentation via MHC class Ib	5/389	39/23328	0.000453781	0.00447	0.00284	H2-Q6/B2	5
GO:0032781	GO:0032781	positive regulation of ATPase activity	5/389	39/23328	0.000453781	0.00447	0.00284	Adrb2/Atp	5
GO:0050715	GO:0050715	positive regulation of cytokine secretion	5/389	39/23328	0.000453781	0.00447	0.00284	Rgcc/Cd3	5
GO:0071827	GO:0071827	plasma lipoprotein particle organization	5/389	39/23328	0.000453781	0.00447	0.00284	Pltp/Apoe	5
GO:0019933	GO:0019933	cAMP-mediated signaling	11/389	195/23328	0.000455933	0.00448	0.00285	Adrb2/Adc	11
GO:0032091	GO:0032091	negative regulation of protein binding	8/389	108/23328	0.000459208	0.0045	0.00286	Ptprf/Id1/	8
GO:0071692	GO:0071692	protein localization to extracellular region	19/389	478/23328	0.000470127	0.00458	0.00291	Rgcc/Srgr	19
GO:0002697	GO:0002697	regulation of immune effector process	18/389	440/23328	0.000470862	0.00458	0.00291	Lbp/Fgl2/	18
GO:0051928	GO:0051928	positive regulation of calcium ion transport	9/389	136/23328	0.000473355	0.00458	0.00291	Thy1/Adrb	9
GO:0032272	GO:0032272	negative regulation of protein polymerization	7/389	83/23328	0.000475156	0.00458	0.00291	Cdh5/Pec	7
GO:0045844	GO:0045844	positive regulation of striated muscle tissue developme	7/389	83/23328	0.000475156	0.00458	0.00291	Tbx1/Adrb	7
GO:0048636	GO:0048636	positive regulation of muscle organ development	7/389	83/23328	0.000475156	0.00458	0.00291	Tbx1/Adrb	7
GO:0060415	GO:0060415	muscle tissue morphogenesis	7/389	83/23328	0.000475156	0.00458	0.00291	Tbx1/Prox	7
GO:0072678	GO:0072678	T cell migration	6/389	60/23328	0.000484609	0.00466	0.00296	Ccl21a/Cc	6
GO:0014896	GO:0014896	muscle hypertrophy	8/389	109/23328	0.000488507	0.00468	0.00298	Igfbp5/Fb	8
GO:0062197	GO:0062197	cellular response to chemical stress	15/389	332/23328	0.000501357	0.00479	0.00305	Lcn2/Epas	15
GO:0034370	GO:0034370	triglyceride-rich lipoprotein particle remodeling	3/389	10/23328	0.000506083	0.00483	0.00307	Apoe/Gpil	3
GO:1901863	GO:1901863	positive regulation of muscle tissue development	7/389	84/23328	0.000511046	0.00486	0.00309	Tbx1/Adrb	7
GO:0085029	GO:0085029	extracellular matrix assembly	5/389	40/23328	0.000511584	0.00486	0.00309	Rgcc/Ths	5
GO:0030509	GO:0030509	BMP signaling pathway	10/389	167/23328	0.000515381	0.00486	0.00309	Id1/Bmp4	10
GO:0045834	GO:0045834	positive regulation of lipid metabolic process	10/389	167/23328	0.000515381	0.00486	0.00309	Flt1/Cd36	10
GO:0051100	GO:0051100	negative regulation of binding	10/389	167/23328	0.000515381	0.00486	0.00309	Ptprf/Id1/	10
GO:0032231	GO:0032231	regulation of actin filament bundle assembly	8/389	110/23328	0.000519302	0.00486	0.00309	Rgcc/Id1/	8
GO:0032612	GO:0032612	interleukin-1 production	8/389	110/23328	0.000519302	0.00486	0.00309	Cd36/Hsp	8
GO:0006907	GO:0006907	pinocytosis	4/389	23/23328	0.000524454	0.00486	0.00309	Cav1/Ehd	4
GO:0036303	GO:0036303	lymph vessel morphogenesis	4/389	23/23328	0.000524454	0.00486	0.00309	Flt4/Prox1	4
GO:0042572	GO:0042572	retinol metabolic process	4/389	23/23328	0.000524454	0.00486	0.00309	Adh7/Rbp	4
GO:0071276	GO:0071276	cellular response to cadmium ion	4/389	23/23328	0.000524454	0.00486	0.00309	Jun/Mt1/M	4
GO:0071450	GO:0071450	cellular response to oxygen radical	4/389	23/23328	0.000524454	0.00486	0.00309	Cd36/Prd	4
GO:0071451	GO:0071451	cellular response to superoxide	4/389	23/23328	0.000524454	0.00486	0.00309	Cd36/Prd	4
GO:0043010	GO:0043010	camera-type eye development	16/389	370/23328	0.000531023	0.00491	0.00312	Maf/Thy1/	16
GO:0003015	GO:0003015	heart process	12/389	231/23328	0.000535238	0.00492	0.00313	Epas1/Gja	12
GO:0009952	GO:0009952	anterior/posterior pattern specification	12/389	231/23328	0.000535238	0.00492	0.00313	Nr2f2/Hox	12
GO:1901653	GO:1901653	cellular response to peptide	14/389	299/23328	0.000539134	0.00495	0.00315	Ptprf/Cd3	14
GO:0022409	GO:0022409	positive regulation of cell-cell adhesion	13/389	265/23328	0.000547144	0.00501	0.00319	Thy1/Ank	13
GO:1904950	GO:1904950	negative regulation of establishment of protein localiza	10/389	169/23328	0.000565676	0.00517	0.00329	Rgcc/Srgr	10
GO:0050890	GO:0050890	cognition	15/389	336/23328	0.000567377	0.00517	0.00329	Reln/Ptn/	15
GO:0002483	GO:0002483	antigen processing and presentation of endogenous p	5/389	41/23328	0.00057475	0.00519	0.0033	H2-Q6/B2	5
GO:0019885	GO:0019885	antigen processing and presentation of endogenous p	5/389	41/23328	0.00057475	0.00519	0.0033	H2-Q6/B2	5
GO:0098801	GO:0098801	regulation of renal system process	5/389	41/23328	0.00057475	0.00519	0.0033	Gja5/Pdgf	5
GO:0043405	GO:0043405	regulation of MAP kinase activity	14/389	301/23328	0.00057569	0.00519	0.0033	Flt1/Bmp4	14
GO:0003229	GO:0003229	ventricular cardiac muscle tissue development	6/389	62/23328	0.000578683	0.00519	0.0033	Prox1/Eng	6
GO:0032835	GO:0032835	glomerulus development	6/389	62/23328	0.000578683	0.00519	0.0033	Bmp4/Pdg	6
GO:0046850	GO:0046850	regulation of bone remodeling	6/389	62/23328	0.000578683	0.00519	0.0033	Spp1/Gja	6
GO:0071453	GO:0071453	cellular response to oxygen levels	9/389	140/23328	0.000584392	0.00522	0.00332	Rgcc/Epa	9
GO:0030038	GO:0030038	contractile actin filament bundle assembly	8/389	112/23328	0.000585611	0.00522	0.00332	Rgcc/F11	8
GO:0043149	GO:0043149	stress fiber assembly	8/389	112/23328	0.000585611	0.00522	0.00332	Rgcc/F11	8
GO:0060411	GO:0060411	cardiac septum morphogenesis	7/389	86/23328	0.000589291	0.00523	0.00333	Tbx1/Gja5	7
GO:2001057	GO:2001057	reactive nitrogen species metabolic process	7/389	86/23328	0.000589291	0.00523	0.00333	Cd34/Ass	7
GO:0007272	GO:0007272	ensheathment of neurons	10/389	170/23328	0.000592308	0.00523	0.00333	Cldn5/Cd	10
GO:0008366	GO:0008366	axon ensheathment	10/389	170/23328	0.000592308	0.00523	0.00333	Cldn5/Cd	10
GO:0044403	GO:0044403	symbiotic process	16/389	374/23328	0.000596127	0.00526	0.00334	Rsad2/An	16
GO:0048736	GO:0048736	appendage development	11/389	202/23328	0.00061242	0.00538	0.00342	Nr2f2/Hox	11
GO:0060173	GO:0060173	limb development	11/389	202/23328	0.00061242	0.00538	0.00342	Nr2f2/Hox	11
GO:0002523	GO:0002523	leukocyte migration involved in inflammatory response	4/389	24/23328	0.000621119	0.00543	0.00345	Lbp/Ptn/S	4
GO:0098869	GO:0098869	cellular oxidant detoxification	4/389	24/23328	0.000621119	0.00543	0.00345	Cd36/Prd	4
GO:0071479	GO:0071479	cellular response to ionizing radiation	6/389	63/23328	0.000630737	0.0055	0.0035	Sfrp2/Net	6
GO:0034103	GO:0034103	regulation of tissue remodeling	7/389	87/23328	0.000631828	0.0055	0.0035	Flt4/Spp1/	7
GO:0001892	GO:0001892	embryonic placenta development	8/389	114/23328	0.000658599	0.00571	0.00363	Nr2f2/Epa	8
GO:0006939	GO:0006939	smooth muscle contraction	8/389	114/23328	0.000658599	0.00571	0.00363	Adrb2/Cav	8
GO:0015909	GO:0015909	long-chain fatty acid transport	6/389	64/23328	0.000686348	0.00589	0.00375	Fabp4/Cd	6
GO:0045668	GO:0045668	negative regulation of osteoblast differentiation	6/389	64/23328	0.000686348	0.00589	0.00375	Igfbp5/Id3	6
GO:0050707	GO:0050707	regulation of cytokine secretion	6/389	64/23328	0.000686348	0.00589	0.00375	Rgcc/Srgr	6
GO:0097084	GO:0097084	vascular associated smooth muscle cell development	3/389	11/23328	0.000687276	0.00589	0.00375	Eng/Comp	3
GO:0150104	GO:0150104	transport across blood-brain barrier	3/389	11/23328	0.000687276	0.00589	0.00375	Cd36/Fab	3
GO:0001837	GO:0001837	epithelial to mesenchymal transition	9/389	144/23328	0.000715902	0.00613	0.0039	Rgcc/Bmp	9
GO:0071825	GO:0071825	protein-lipid complex subunit organization	5/389	43/23328	0.000718423	0.00614	0.0039	Pltp/Apoe	5
GO:0001656	GO:0001656	metanephros development	7/389	89/23328	0.000724196	0.00617	0.00392	Id3/Sox17	7
GO:1903037	GO:1903037	regulation of leukocyte cell-cell adhesion	14/389	309/23328	0.000743721	0.00633	0.00402	Dtx1/Thy1	14
GO:0035265	GO:0035265	organ growth	11/389	207/23328	0.000749696	0.00636	0.00405	Rgs4/Stc1	11
GO:0051924	GO:0051924	regulation of calcium ion transport	13/389	275/23328	0.00077188	0.00654	0.00416	Thy1/Prnp	13
GO:0061448	GO:0061448	connective tissue development	13/389	276/23328	0.000798102	0.0067	0.00426	Maf/Bmp4	13
GO:0019883	GO:0019883	antigen processing and presentation of endogenous a	5/389	44/23328	0.000799569	0.0067	0.00426	H2-Q6/B2	5
GO:0032768	GO:0032768	regulation of monooxygenase activity	5/389	44/23328	0.000799569	0.0067	0.00426	Cav1/Apo	5
GO:0071385	GO:0071385	cellular response to glucocorticoid stimulus	5/389	44/23328	0.000799569	0.0067	0.00426	Bmp4/Fbx	5
GO:1902895	GO:1902895	positive regulation of pri-miRNA transcription by RNA p	5/389	44/23328	0.000799569	0.0067	0.00426	Bmp4/Pdg	5
GO:0016202	GO:0016202	regulation of striated muscle tissue development	10/389	177/23328	0.000809264	0.00677	0.0043	Tbx1/Adrb	10
GO:0002088	GO:0002088	lens development in camera-type eye	7/389	91/23328	0.000826938	0.00687	0.00437	Maf/Bmp4	7
GO:0014033	GO:0014033	neural crest cell differentiation	7/389	91/23328	0.000826938	0.00687	0.00437	Tbx1/Bmp	7
GO:0042632	GO:0042632	cholesterol homeostasis	7/389	91/23328	0.000826938	0.00687	0.00437	Ephx2/Fal	7
GO:0031333	GO:0031333	negative regulation of protein-containing complex asse	9/389	147/23328	0.000829602	0.00687	0.00437	Cdh5/Pec	9
GO:0045667	GO:0045667	regulation of osteoblast differentiation	9/389	147/23328	0.000829602	0.00687	0.00437	Igfbp5/Id3	9
GO:0071772	GO:0071772	response to BMP	10/389	178/23328	0.000845005	0.00697	0.00443	Id1/Bmp4	10
GO:0071773	GO:0071773	cellular response to BMP stimulus	10/389	178/23328	0.000845005	0.00697	0.00443	Id1/Bmp4	10
GO:0090280	GO:0090280	positive regulation of calcium ion import	4/389	26/23328	0.000851194	0.007	0.00445	Adrb2/Cx	4
GO:0031349	GO:0031349	positive regulation of defense response	13/389	278/23328	0.000852798	0.007	0.00445	Lbp/Fabp	13
GO:0045861	GO:0045861	negative regulation of proteolysis	15/389	350/23328	0.000860057	0.00705	0.00448	Serpine2/	15
GO:0055092	GO:0055092	sterol homeostasis	7/389	92/23328	0.000882448	0.00722	0.00459	Ephx2/Fal	7
GO:0050918	GO:0050918	positive chemotaxis	5/389	45/23328	0.000887359	0.00724	0.00461	Ccl21a/Cx	5
GO:0034375	GO:0034375	high-density lipoprotein particle remodeling	3/389	12/23328	0.000905068	0.00733	0.00466	Pltp/Apoe	3
GO:0072124	GO:0072124	regulation of glomerular mesangial cell proliferation	3/389	12/23328	0.000905068	0.00733	0.00466	Bmp4/Pdg	3
GO:1900272	GO:1900272	negative regulation of long-term synaptic potentiation	3/389	12/23328	0.000905068	0.00733	0.00466	Ptn/Prnp/	3
GO:1902947	GO:1902947	regulation of tau-protein kinase activity	3/389	12/23328	0.000905068	0.00733	0.00466	Apoe/Clu/	3
GO:0071774	GO:0071774	response to fibroblast growth factor	7/389	93/23328	0.000940854	0.0076	0.00483	Tbx1/Nr4a	7
GO:0002819	GO:0002819	regulation of adaptive immune response	11/389	213/23328	0.000947239	0.0076	0.00483	H2-Q6/Rs	11
GO:0051651	GO:0051651	maintenance of location in cell	11/389	213/23328	0.000947239	0.0076	0.00483	Thy1/Srgr	11
GO:0007566	GO:0007566	embryo implantation	6/389	68/23328	0.000947667	0.0076	0.00483	Tppp3/H2	6
GO:0032507	GO:0032507	maintenance of protein location in cell	6/389	68/23328	0.000947667	0.0076	0.00483	Srgn/Ank	6
GO:0007266	GO:0007266	Rho protein signal transduction	10/389	181/23328	0.000960088	0.00767	0.00488	Cdc42ep3	10
GO:0048634	GO:0048634	regulation of muscle organ development	10/389	181/23328	0.000960088	0.00767	0.00488	Tbx1/Adrb	10
GO:0090092	GO:0090092	regulation of transmembrane receptor protein serine/th	12/389	247/23328	0.000962017	0.00767	0.00488	Bmp4/Lrg	12
GO:0030217	GO:0030217	T cell differentiation	13/389	282/23328	0.000971666	0.00773	0.00492	Dtx1/Fgl2	13
GO:0043618	GO:0043618	regulation of transcription from RNA polymerase II pro	5/389	46/23328	0.000982124	0.00774	0.00492	Epas1/Cd	5
GO:0071384	GO:0071384	cellular response to corticosteroid stimulus	5/389	46/23328	0.000982124	0.00774	0.00492	Bmp4/Fbx	5
GO:0007160	GO:0007160	cell-matrix adhesion	11/389	214/23328	0.000984007	0.00774	0.00492	Thy1/Cd3	11
GO:0003071	GO:0003071	renal system process involved in regulation of systemi	4/389	27/23328	0.000986189	0.00774	0.00492	Gja5/Pdgf	4
GO:0018149	GO:0018149	peptide cross-linking	4/389	27/23328	0.000986189	0.00774	0.00492	Tgm2/Bgr	4
GO:1900120	GO:1900120	regulation of receptor binding	4/389	27/23328	0.000986189	0.00774	0.00492	Ptprf/B2m	4
GO:1905809	GO:1905809	negative regulation of synapse organization	4/389	27/23328	0.000986189	0.00774	0.00492	Ptn/Prnp/	4
GO:0043535	GO:0043535	regulation of blood vessel endothelial cell migration	7/389	94/23328	0.001002263	0.00785	0.00499	Rgcc/Hsp	7
GO:0048146	GO:0048146	positive regulation of fibroblast proliferation	6/389	69/23328	0.001023571	0.008	0.00509	Anxa2/Pd	6
GO:0051235	GO:0051235	maintenance of location	14/389	320/23328	0.001041097	0.00813	0.00517	Thy1/Srgr	14

GO:0051492	GO:0051492	regulation of stress fiber assembly	7/389	95/23328	0.001066779	0.00831	0.00528	Rgcc/F11r	7
GO:0045581	GO:0045581	negative regulation of T cell differentiation	5/389	47/23328	0.001084202	0.00843	0.00536	Dtx1/Fgl2/	5
GO:0071478	GO:0071478	cellular response to radiation	9/389	153/23328	0.001101122	0.0085	0.00541	Sfrp2/Mfa	9
GO:0055008	GO:0055008	cardiac muscle tissue morphogenesis	6/389	70/23328	0.001104057	0.0085	0.00541	Prox1/Eng	6
GO:1990868	GO:1990868	response to chemokine	6/389	70/23328	0.001104057	0.0085	0.00541	Ccl21a/Cx	6
GO:1990869	GO:1990869	cellular response to chemokine	6/389	70/23328	0.001104057	0.0085	0.00541	Ccl21a/Cx	6
GO:0009615	GO:0009615	response to virus	13/389	286/23328	0.001104108	0.0085	0.00541	Fgl2/Lcn2	13
GO:0002693	GO:0002693	positive regulation of cellular extravasation	4/389	28/23328	0.00113555	0.00871	0.00554	Thy1/Peca	4
GO:0060037	GO:0060037	pharyngeal system development	4/389	28/23328	0.00113555	0.00871	0.00554	Tbx1/Bmp	4
GO:1990266	GO:1990266	neutrophil migration	8/389	124/23328	0.001141394	0.00874	0.00556	Lbp/Ccl21	8
GO:0009404	GO:0009404	toxin metabolic process	3/389	13/23328	0.001162092	0.00884	0.00562	Pam/Cyp1	3
GO:0031953	GO:0031953	negative regulation of protein autophosphorylation	3/389	13/23328	0.001162092	0.00884	0.00562	Cav1/Eng	3
GO:0072110	GO:0072110	glomerular mesangial cell proliferation	3/389	13/23328	0.001162092	0.00884	0.00562	Bmp4/Pdg	3
GO:0007264	GO:0007264	small GTPase mediated signal transduction	17/389	437/23328	0.001163508	0.00884	0.00562	Reln/Cdc4	17
GO:0043407	GO:0043407	negative regulation of MAP kinase activity	6/389	71/23328	0.001189307	0.00897	0.00571	Bmp4/Sfrp	6
GO:0003382	GO:0003382	epithelial cell morphogenesis	5/389	48/23328	0.001193936	0.00897	0.00571	Col18a1/C	5
GO:0032965	GO:0032965	regulation of collagen biosynthetic process	5/389	48/23328	0.001193936	0.00897	0.00571	Rgcc/Bmp	5
GO:0090279	GO:0090279	regulation of calcium ion import	5/389	48/23328	0.001193936	0.00897	0.00571	Prnp/Adrb	5
GO:1903115	GO:1903115	regulation of actin filament-based movement	5/389	48/23328	0.001193936	0.00897	0.00571	Gja5/Cav1	5
GO:1903727	GO:1903727	positive regulation of phospholipid metabolic process	5/389	48/23328	0.001193936	0.00897	0.00571	Flt1/Adgrf	5
GO:0097530	GO:0097530	granulocyte migration	9/389	155/23328	0.001206138	0.00905	0.00575	Lbp/Ccl21	9
GO:0007389	GO:0007389	pattern specification process	18/389	478/23328	0.001214705	0.0091	0.00578	Reln/Nr2f2	18
GO:0060541	GO:0060541	respiratory system development	12/389	254/23328	0.001222514	0.00914	0.00581	Igfbp5/Sp	12
GO:0006937	GO:0006937	regulation of muscle contraction	9/389	156/23328	0.001261598	0.00935	0.00594	Gja5/Adrb	9
GO:0030856	GO:0030856	regulation of epithelial cell differentiation	9/389	156/23328	0.001261598	0.00935	0.00594	Cldn5/Id1/	9
GO:0030048	GO:0030048	actin filament-based movement	8/389	126/23328	0.001265329	0.00935	0.00594	Gja5/Cav1	8
GO:0031341	GO:0031341	regulation of cell killing	8/389	126/23328	0.001265329	0.00935	0.00594	H2-Q6/B2	8
GO:0036294	GO:0036294	cellular response to decreased oxygen levels	8/389	126/23328	0.001265329	0.00935	0.00594	Rgcc/Epa	8
GO:0071621	GO:0071621	granulocyte chemotaxis	8/389	126/23328	0.001265329	0.00935	0.00594	Lbp/Ccl21	8
GO:0006869	GO:0006869	lipid transport	15/389	364/23328	0.001271775	0.00935	0.00594	Lbp/Fabp4	15
GO:0021700	GO:0021700	developmental maturation	14/389	327/23328	0.001278062	0.00935	0.00594	Reln/Epas	14
GO:0008064	GO:0008064	regulation of actin polymerization or depolymerization	10/389	188/23328	0.001279147	0.00935	0.00594	Gmfg/Cdc	10
GO:0010611	GO:0010611	regulation of cardiac muscle hypertrophy	6/389	72/23328	0.001279506	0.00935	0.00594	Fbxo32/R	6
GO:0048002	GO:0048002	antigen processing and presentation of peptide antigen	6/389	72/23328	0.001279506	0.00935	0.00594	H2-Q6/B2	6
GO:0030510	GO:0030510	regulation of BMP signaling pathway	7/389	98/23328	0.001280076	0.00935	0.00594	Bmp4/Sfrp	7
GO:0032652	GO:0032652	regulation of interleukin-1 production	7/389	98/23328	0.001280076	0.00935	0.00594	Hspb1/Se	7
GO:0071902	GO:0071902	positive regulation of protein serine/threonine kinase a	13/389	291/23328	0.001290555	0.00937	0.00596	Rgcc/Flt1/	13
GO:0001516	GO:0001516	prostaglandin biosynthetic process	4/389	29/23328	0.001300069	0.00937	0.00596	Fabp5/Ptg	4
GO:0002029	GO:0002029	desensitization of G protein-coupled receptor signaling	4/389	29/23328	0.001300069	0.00937	0.00596	Adrb2/Apl	4
GO:0003148	GO:0003148	outflow tract septum morphogenesis	4/389	29/23328	0.001300069	0.00937	0.00596	Tbx1/Bmp	4
GO:0022401	GO:0022401	negative adaptation of signaling pathway	4/389	29/23328	0.001300069	0.00937	0.00596	Adrb2/Apl	4
GO:0042403	GO:0042403	thyroid hormone metabolic process	4/389	29/23328	0.001300069	0.00937	0.00596	Reln/Duox	4
GO:0046457	GO:0046457	prostanoid biosynthetic process	4/389	29/23328	0.001300069	0.00937	0.00596	Fabp5/Ptg	4
GO:0006692	GO:0006692	prostanoid metabolic process	5/389	49/23328	0.00131167	0.00941	0.00598	Fabp5/Ptg	5
GO:0006693	GO:0006693	prostaglandin metabolic process	5/389	49/23328	0.00131167	0.00941	0.00598	Fabp5/Ptg	5
GO:0007157	GO:0007157	heterophilic cell-cell adhesion via plasma membrane c	5/389	49/23328	0.00131167	0.00941	0.00598	Vcam1/Se	5
GO:0045732	GO:0045732	positive regulation of protein catabolic process	11/389	222/23328	0.00132277	0.00945	0.00601	Sox17/Ecs	11
GO:0060348	GO:0060348	bone development	11/389	222/23328	0.00132277	0.00945	0.00601	Sparc/Bm	11
GO:0008277	GO:0008277	regulation of G protein-coupled receptor signaling path	8/389	127/23328	0.00133119	0.00948	0.00603	Adrb2/Rgs	8
GO:0090090	GO:0090090	negative regulation of canonical Wnt signaling pathway	8/389	127/23328	0.00133119	0.00948	0.00603	Sox17/Tm	8
GO:0030593	GO:0030593	neutrophil chemotaxis	7/389	99/23328	0.001358131	0.00964	0.00613	Lbp/Ccl21	7
GO:0043502	GO:0043502	regulation of muscle adaptation	7/389	99/23328	0.001358131	0.00964	0.00613	Igfbp5/Fbx	7
GO:0002711	GO:0002711	positive regulation of T cell mediated immunity	6/389	73/23328	0.001374839	0.00972	0.00618	H2-Q6/Rs	6
GO:0014068	GO:0014068	positive regulation of phosphatidylinositol 3-kinase sig	6/389	73/23328	0.001374839	0.00972	0.00618	Reln/Flt1/	6
GO:0055002	GO:0055002	striated muscle cell development	10/389	190/23328	0.001384677	0.00978	0.00622	Bmp4/Rgs	10
GO:0003279	GO:0003279	cardiac septum development	8/389	128/23328	0.001399747	0.00987	0.00627	Tbx1/Gja5	8
GO:0006959	GO:0006959	humoral immune response	16/389	406/23328	0.00140927	0.00992	0.00631	Rgcc/Nts/	16
GO:0071214	GO:0071214	cellular response to abiotic stimulus	13/389	294/23328	0.001414505	0.00992	0.00631	Sfrp2/Mfa	13
GO:0104004	GO:0104004	cellular response to environmental stimulus	13/389	294/23328	0.001414505	0.00992	0.00631	Sfrp2/Mfa	13
GO:0030832	GO:0030832	regulation of actin filament length	10/389	191/23328	0.001440037	0.01008	0.00641	Gmfg/Cdc	10
GO:0070365	GO:0070365	hepatocyte differentiation	3/389	14/23328	0.001460819	0.01017	0.00647	Bmp4/Pro	3
GO:0072224	GO:0072224	metanephric glomerulus development	3/389	14/23328	0.001460819	0.01017	0.00647	Pdgfb/Aqp	3
GO:0090331	GO:0090331	negative regulation of platelet aggregation	3/389	14/23328	0.001460819	0.01017	0.00647	Cd9/Serpi	3
GO:0061008	GO:0061008	hepaticobiliary system development	8/389	129/23328	0.00147108	0.01021	0.00649	Ptn/Sox17	8
GO:0061844	GO:0061844	antimicrobial humoral immune response mediated by a	8/389	129/23328	0.00147108	0.01021	0.00649	Nts/Cxcl1	8
GO:0035767	GO:0035767	endothelial cell chemotaxis	4/389	30/23328	0.001480539	0.01026	0.00652	Lgmn/Hsp	4
GO:0048638	GO:0048638	regulation of developmental growth	16/389	409/23328	0.001519254	0.01051	0.00668	Lgmn/Adr	16
GO:0043270	GO:0043270	positive regulation of ion transport	14/389	334/23328	0.001558613	0.01076	0.00684	Reln/Thy1	14
GO:0034765	GO:0034765	regulation of ion transmembrane transport	18/389	489/23328	0.001562244	0.01077	0.00685	Reln/Thy1	18
GO:0001916	GO:0001916	positive regulation of T cell mediated cytotoxicity	5/389	51/23328	0.001572544	0.01079	0.00686	H2-Q6/B2	5
GO:0002474	GO:0002474	antigen processing and presentation of peptide antigen	5/389	51/23328	0.001572544	0.01079	0.00686	H2-Q6/B2	5
GO:0043620	GO:0043620	regulation of DNA-templated transcription in response	5/389	51/23328	0.001572544	0.01079	0.00686	Epas1/Cd	5
GO:0097755	GO:0097755	positive regulation of blood vessel diameter	6/389	75/23328	0.001581671	0.01081	0.00687	Gja5/Adrb	6
GO:1903725	GO:1903725	regulation of phospholipid metabolic process	6/389	75/23328	0.001581671	0.01081	0.00687	Flt1/Adgrf	6
GO:0031623	GO:0031623	receptor internalization	8/389	131/23328	0.001622389	0.01107	0.00704	Cd9/Cd36	8
GO:0010876	GO:0010876	lipid localization	16/389	413/23328	0.001677062	0.01132	0.0072	Lbp/Fabp4	16
GO:0032535	GO:0032535	regulation of cellular component size	16/389	413/23328	0.001677062	0.01132	0.0072	Gmfg/Cdc	16
GO:0002026	GO:0002026	regulation of the force of heart contraction	4/389	31/23328	0.001677748	0.01132	0.0072	Adrb2/Cav	4
GO:0023058	GO:0023058	adaptation of signaling pathway	4/389	31/23328	0.001677748	0.01132	0.0072	Adrb2/Apl	4
GO:0032373	GO:0032373	positive regulation of sterol transport	4/389	31/23328	0.001677748	0.01132	0.0072	Pltp/Anxa	4
GO:0032376	GO:0032376	positive regulation of cholesterol transport	4/389	31/23328	0.001677748	0.01132	0.0072	Pltp/Anxa	4
GO:0050901	GO:0050901	leukocyte tethering or rolling	4/389	31/23328	0.001677748	0.01132	0.0072	Cxcl12/Ja	4
GO:0016485	GO:0016485	protein processing	11/389	229/23328	0.001692797	0.01137	0.00723	Srgn/Serp	11
GO:0000079	GO:0000079	regulation of cyclin-dependent protein serine/threonine	6/389	76/23328	0.001693554	0.01137	0.00723	Nr2f2/Rgc	6
GO:0050663	GO:0050663	cytokine secretion	6/389	76/23328	0.001693554	0.01137	0.00723	Rgcc/Srgr	6
GO:0099175	GO:0099175	regulation of postsynapse organization	8/389	132/23328	0.001702526	0.01141	0.00725	Reln/Srgn	8
GO:0003300	GO:0003300	cardiac muscle hypertrophy	7/389	103/23328	0.001708226	0.01142	0.00726	Fbxo32/R	7
GO:0006941	GO:0006941	striated muscle contraction	9/389	163/23328	0.001710017	0.01142	0.00726	Gja5/Adrb	9
GO:0001974	GO:0001974	blood vessel remodeling	5/389	52/23328	0.001716391	0.01144	0.00728	Flt4/Epas1	5
GO:0001655	GO:0001655	urogenital system development	15/389	376/23328	0.001746183	0.01162	0.00739	Id3/Tnc/S	15
GO:0043433	GO:0043433	negative regulation of DNA-binding transcription factor	9/389	164/23328	0.001783358	0.0118	0.0075	Id3/Id1/Pr	9
GO:1901214	GO:1901214	regulation of neuron death	15/389	377/23328	0.001791633	0.0118	0.0075	Cd34/Ptpr	15
GO:0010885	GO:0010885	regulation of cholesterol storage	3/389	15/23328	0.001803563	0.0118	0.0075	Cd36/Sca	3
GO:0019755	GO:0019755	one-carbon compound transport	3/389	15/23328	0.001803563	0.0118	0.0075	Aqp1/Aqp	3
GO:0030449	GO:0030449	regulation of complement activation	3/389	15/23328	0.001803563	0.0118	0.0075	Serping1/	3
GO:0050930	GO:0050930	induction of positive chemotaxis	3/389	15/23328	0.001803563	0.0118	0.0075	Ccl21a/Cx	3
GO:0060546	GO:0060546	negative regulation of necroptotic process	3/389	15/23328	0.001803563	0.0118	0.0075	Cav1/Slc2	3
GO:0062099	GO:0062099	negative regulation of programmed necrotic cell death	3/389	15/23328	0.001803563	0.0118	0.0075	Cav1/Slc2	3
GO:0150105	GO:0150105	protein localization to cell-cell junction	3/389	15/23328	0.001803563	0.0118	0.0075	Cdh5/F11	3
GO:2000095	GO:2000095	regulation of Wnt signaling pathway, planar cell polarit	3/389	15/23328	0.001803563	0.0118	0.0075	Sfrp2/Dab	3
GO:0070997	GO:0070997	neuron death	16/389	416/23328	0.001804257	0.0118	0.0075	Cd34/Ptpr	16
GO:0061097	GO:0061097	regulation of protein tyrosine kinase activity	6/389	77/23328	0.001811345	0.01183	0.00752	Reln/Thy1	6
GO:0019730	GO:0019730	antimicrobial humoral response	9/389	165/23328	0.001859195	0.01207	0.00767	Nts/Cxcl1	9
GO:1905952	GO:1905952	regulation of lipid localization	9/389	165/23328	0.001859195	0.01207	0.00767	Cd36/Pltp	9
GO:0030850	GO:0030850	prostate gland development	5/389	53/23328	0.001869655	0.01207	0.00767	Tnc/Bmp4	5
GO:0050435	GO:0050435	amyloid-beta metabolic process	5/389	53/23328	0.001869655	0.01207	0.00767	Prnp/Apoe	5
GO:0055010	GO:0055010	ventricular cardiac muscle tissue morphogenesis	5/389	53/23328	0.001869655	0.01207	0.00767	Prox1/Eng	5
GO:0061383	GO:0061383	trabecula morphogenesis	5/389	53/23328	0.001869655	0.01207	0.00767	Eng/Mmp2	5
GO:0002698	GO:0002698	negative regulation of immune effector process	8/389	134/23328	0.001872175	0.01207	0.00767	Fgl2/Serp	8
GO:1903038	GO:1903038	negative regulation of leukocyte cell-cell adhesion	8/389	134/23328	0.001872175	0.01207	0.00767	Dtx1/Fgl2/	8
GO:0003002	GO:0003002	regionalization	15/389	379/23328	0.001885499	0.0121	0.0077	Reln/Nr2f2	15
GO:0010939	GO:0010939	regulation of necrotic cell death	4/389	32/23328	0.001892479	0.0121	0.0077	Cav1/Slc2	4
GO:0048384	GO:0048384	retinoic acid receptor signaling pathway	4/389	32/23328	0.001892479	0.0121	0.0077	Tbx1/Klf2/	4
GO:0051150	GO:0051150	regulation of smooth muscle cell differentiation	4/389	32/23328	0.001892479	0.0121	0.0077	Bmp4/Pdg	4
GO:0060669	GO:0060669	embryonic placenta morphogenesis	4/389	32/23328	0.001892479	0.0121	0.0077	Cdkn1c/S	4
GO:0097191	GO:0097191	extrinsic apoptotic signaling pathway	11/389	233/23328	0.001939636	0.01239	0.00788	Lcn2/Bmp	11
GO:0006816	GO:0006816	calcium ion transport	16/389	420/23328	0.001986346	0.01264	0.00804	Thy1/Prnp	16
GO:0010639	GO:0010639	negative regulation of organelle organization	16/389	420/23328	0.001986346	0.01264	0.00804	Rgcc/Gmf	16
GO:0042552	GO:0042552	myelination	9/389	167/23328	0.002018601	0.01283	0.00816	Cldn5/Cd9	9
GO:0099173	GO:0099173	postsynapse organization	10/389	200/23328	0.00202385	0.01284	0.00817	Reln/Srgn	10
GO:0048546	GO:0048546	digestive tract morphogenesis	5/389	54/23328	0.002032695	0.01286	0.00818	Sox17/Bm	5
GO:1900271	GO:1900271	regulation of long-term synaptic potentiation	5/389	54/23328	0.002032695	0.01286	0.00818	Reln/Ptn/F	5
GO:0045862	GO:0045862	positive regulation of proteolysis	14/389	344/23328	0.002047001	0.01293	0.00822	Ecscr/Lgn	14
GO:0048145	GO:0048145	regulation of fibroblast proliferation	7/389	107/23328	0.002124717	0.01336	0.0085	Anxa2/Pd	7
GO:0002486	GO:0002486	antigen processing and presentation of endogenous pe	4/389	33/23328	0.002125507	0.01336	0.0085	H2-Q6/H2	4
GO:0060740	GO:0060740	prostate gland epithelium morphogenesis	4/389	33/23328	0.002125507	0.01336	0.0085	Tnc/Bmp4	4
GO:0034341	GO:0034341	response to interferon-gamma	8/389	137/23328	0.002151356	0.0135	0.00859	Ccl21a/Mr	8
GO:0010224	GO:0010224	response to UV-B	3/389	16/23328	0.002192489	0.0136	0.00864	Mfap4/Cri	3
GO:0018119	GO:0018119	peptidyl-cysteine S-nitrosylation	3/389	16/23328	0.002192489	0.0136	0.00864	Gapdh/S1	3

GO:0070486	GO:0070486	leukocyte aggregation	3/389	16/23328	0.002192489	0.0136	0.00864	Jam2/S10	3
GO:0071498	GO:0071498	cellular response to fluid shear stress	3/389	16/23328	0.002192489	0.0136	0.00864	Ass1/Spp	3
GO:0072109	GO:0072109	glomerular mesangium development	3/389	16/23328	0.002192489	0.0136	0.00864	Bmp4/Pdg	3
GO:1901550	GO:1901550	regulation of endothelial cell development	3/389	16/23328	0.002192489	0.0136	0.00864	Cldn5/Cdh	3
GO:1901722	GO:1901722	regulation of cell proliferation involved in kidney develo	3/389	16/23328	0.002192489	0.0136	0.00864	Bmp4/Pdg	3
GO:1903140	GO:1903140	regulation of establishment of endothelial barrier	3/389	16/23328	0.002192489	0.0136	0.00864	Cldn5/Cdh	3
GO:1904029	GO:1904029	regulation of cyclin-dependent protein kinase activity	6/389	80/23328	0.002202148	0.01362	0.00866	Nr2f2/Rgc	6
GO:0002042	GO:0002042	cell migration involved in sprouting angiogenesis	5/389	55/23328	0.002205872	0.01362	0.00866	Nr4a1/Cle	5
GO:0006826	GO:0006826	iron ion transport	5/389	55/23328	0.002205872	0.01362	0.00866	Cp/Lcn2/E	5
GO:0071695	GO:0071695	anatomical structure maturation	12/389	273/23328	0.002237099	0.01379	0.00877	Epas1/Cd	12
GO:0043266	GO:0043266	regulation of potassium ion transport	7/389	108/23328	0.002240149	0.01379	0.00877	Prnp/Adrb	7
GO:0006639	GO:0006639	acylglycerol metabolic process	8/389	138/23328	0.002251348	0.01383	0.0088	Cd36/Thrs	8
GO:0060562	GO:0060562	epithelial tube morphogenesis	15/389	387/23328	0.002302997	0.01413	0.00898	Flt1/Tbx1/	15
GO:0002062	GO:0002062	chondrocyte differentiation	7/389	109/23328	0.00236036	0.01436	0.00913	Maf/Bmp4	7
GO:0048144	GO:0048144	fibroblast proliferation	7/389	109/23328	0.00236036	0.01436	0.00913	Anxa2/Pd	7
GO:1901800	GO:1901800	positive regulation of proteasomal protein catabolic pro	7/389	109/23328	0.00236036	0.01436	0.00913	Ecscr/Cav	7
GO:0030833	GO:0030833	regulation of actin filament polymerization	9/389	171/23328	0.002370079	0.01436	0.00913	Gmfg/Cdc	9
GO:0002476	GO:0002476	antigen processing and presentation of endogenous pe	4/389	34/23328	0.002377601	0.01436	0.00913	H2-Q6/H2	4
GO:0002484	GO:0002484	antigen processing and presentation of endogenous pe	4/389	34/23328	0.002377601	0.01436	0.00913	H2-Q6/H2	4
GO:0003209	GO:0003209	cardiac atrium morphogenesis	4/389	34/23328	0.002377601	0.01436	0.00913	Gja5/Prox	4
GO:0014741	GO:0014741	negative regulation of muscle hypertrophy	4/389	34/23328	0.002377601	0.01436	0.00913	Igfbp5/Fb	4
GO:0032689	GO:0032689	negative regulation of interferon-gamma production	4/389	34/23328	0.002377601	0.01436	0.00913	Prnp/Gas	4
GO:0060512	GO:0060512	prostate gland morphogenesis	4/389	34/23328	0.002377601	0.01436	0.00913	Tnc/Bmp4	4
GO:0001523	GO:0001523	retinoid metabolic process	5/389	56/23328	0.002389551	0.01436	0.00913	Adh7/Rbp	5
GO:0001914	GO:0001914	regulation of T cell mediated cytotoxicity	5/389	56/23328	0.002389551	0.01436	0.00913	H2-Q6/B2	5
GO:0001954	GO:0001954	positive regulation of cell-matrix adhesion	5/389	56/23328	0.002389551	0.01436	0.00913	Thy1/Cd3	5
GO:1902893	GO:1902893	regulation of pri-miRNA transcription by RNA polymera	5/389	56/23328	0.002389551	0.01436	0.00913	Bmp4/Pdg	5
GO:0007265	GO:0007265	Ras protein signal transduction	14/389	350/23328	0.00239657	0.01438	0.00914	Cdc42ep3	14
GO:0006638	GO:0006638	neutral lipid metabolic process	8/389	140/23328	0.00246226	0.01475	0.00938	Cd36/Thrs	8
GO:0017015	GO:0017015	regulation of transforming growth factor beta receptor s	7/389	110/23328	0.00248548	0.01487	0.00945	Lrg1/Cav1	7
GO:0032370	GO:0032370	positive regulation of lipid transport	6/389	82/23328	0.002495904	0.01491	0.00948	Pltp/Spp1	6
GO:0043409	GO:0043409	negative regulation of MAPK cascade	9/389	173/23328	0.002563172	0.01529	0.00972	Bmp4/Tim	9
GO:0032964	GO:0032964	collagen biosynthetic process	5/389	57/23328	0.002584095	0.01539	0.00979	Rgcc/Bmp	5
GO:0008630	GO:0008630	intrinsic apoptotic signaling pathway in response to DN	7/389	111/23328	0.002615642	0.01546	0.00983	Cxcl12/Ier	7
GO:0001921	GO:0001921	positive regulation of receptor recycling	3/389	17/23328	0.002629617	0.01546	0.00983	Anxa2/Da	3
GO:0010878	GO:0010878	cholesterol storage	3/389	17/23328	0.002629617	0.01546	0.00983	Cd36/Sca	3
GO:0017014	GO:0017014	protein nitrosylation	3/389	17/23328	0.002629617	0.01546	0.00983	Gapdh/S1	3
GO:0050665	GO:0050665	hydrogen peroxide biosynthetic process	3/389	17/23328	0.002629617	0.01546	0.00983	Duoxa2/D	3
GO:0051014	GO:0051014	actin filament severing	3/389	17/23328	0.002629617	0.01546	0.00983	Gmfg/Dstr	3
GO:0060850	GO:0060850	regulation of transcription involved in cell fate commitm	3/389	17/23328	0.002629617	0.01546	0.00983	Nr2f2/Sox	3
GO:0090192	GO:0090192	regulation of glomerulus development	3/389	17/23328	0.002629617	0.01546	0.00983	Bmp4/Pdg	3
GO:2001028	GO:2001028	positive regulation of endothelial cell chemotaxis	3/389	17/23328	0.002629617	0.01546	0.00983	Lgmn/Hsp	3
GO:0043243	GO:0043243	positive regulation of protein-containing complex disas	4/389	35/23328	0.002649522	0.01549	0.00985	Adrb2/Dst	4
GO:0055090	GO:0055090	acylglycerol homeostasis	4/389	35/23328	0.002649522	0.01549	0.00985	Apoe/Sca	4
GO:0070328	GO:0070328	triglyceride homeostasis	4/389	35/23328	0.002649522	0.01549	0.00985	Apoe/Sca	4
GO:1902003	GO:1902003	regulation of amyloid-beta formation	4/389	35/23328	0.002649522	0.01549	0.00985	Prnp/Apoe	4
GO:1903844	GO:1903844	regulation of cellular response to transforming growth f	7/389	112/23328	0.002750977	0.01604	0.0102	Lrg1/Cav1	7
GO:0071496	GO:0071496	cellular response to external stimulus	12/389	280/23328	0.002751675	0.01604	0.0102	Sfrp2/Cav	12
GO:0016101	GO:0016101	diterpenoid metabolic process	5/389	58/23328	0.002789869	0.01621	0.01031	Adh7/Rbp	5
GO:0061614	GO:0061614	pri-miRNA transcription by RNA polymerase II	5/389	58/23328	0.002789869	0.01621	0.01031	Bmp4/Pdg	5
GO:0014032	GO:0014032	neural crest cell development	6/389	84/23328	0.002818158	0.01633	0.01038	Tbx1/Bmp	6
GO:0051384	GO:0051384	response to glucocorticoid	6/389	84/23328	0.002818158	0.01633	0.01038	Ass1/Bmp	6
GO:0003401	GO:0003401	axis elongation	4/389	36/23328	0.002942019	0.01698	0.0108	Tnc/Bmp4	4
GO:0035886	GO:0035886	vascular associated smooth muscle cell differentiation	4/389	36/23328	0.002942019	0.01698	0.0108	Pdgfb/Eng	4
GO:0070306	GO:0070306	lens fiber cell differentiation	4/389	36/23328	0.002942019	0.01698	0.0108	Maf/Vim/C	4
GO:0043583	GO:0043583	ear development	11/389	246/23328	0.002951458	0.01701	0.01081	Maf/Tcf15	11
GO:0031346	GO:0031346	positive regulation of cell projection organization	17/389	478/23328	0.002971477	0.0171	0.01087	Reln/Lcn2	17
GO:0030301	GO:0030301	cholesterol transport	6/389	85/23328	0.002990495	0.01718	0.01093	Cd36/Pltp	6
GO:0043462	GO:0043462	regulation of ATPase activity	5/389	59/23328	0.003007238	0.01726	0.01097	Adrb2/Atp	5
GO:2000379	GO:2000379	positive regulation of reactive oxygen species metabol	7/389	114/23328	0.003037705	0.01741	0.01107	Ass1/Cd3	7
GO:0050863	GO:0050863	regulation of T cell activation	13/389	321/23328	0.003042124	0.01741	0.01107	Dtx1/Thy1	13
GO:1903364	GO:1903364	positive regulation of cellular protein catabolic process	8/389	145/23328	0.003057503	0.01747	0.01111	Ecscr/Cav	8
GO:0021542	GO:0021542	dentate gyrus development	3/389	18/23328	0.003116824	0.01764	0.01121	Reln/Prox	3
GO:0045019	GO:0045019	negative regulation of nitric oxide biosynthetic process	3/389	18/23328	0.003116824	0.01764	0.01121	Cd34/Cav	3
GO:0046184	GO:0046184	aldehyde biosynthetic process	3/389	18/23328	0.003116824	0.01764	0.01121	Aldoa/Dab	3
GO:0051000	GO:0051000	positive regulation of nitric-oxide synthase activity	3/389	18/23328	0.003116824	0.01764	0.01121	Apoe/Sca	3
GO:0072148	GO:0072148	epithelial cell fate commitment	3/389	18/23328	0.003116824	0.01764	0.01121	Nr2f2/Pro	3
GO:1901881	GO:1901881	positive regulation of protein depolymerization	3/389	18/23328	0.003116824	0.01764	0.01121	Dstn/Stmn	3
GO:1904406	GO:1904406	negative regulation of nitric oxide metabolic process	3/389	18/23328	0.003116824	0.01764	0.01121	Cd34/Cav	3
GO:1904062	GO:1904062	regulation of cation transmembrane transport	14/389	361/23328	0.003165225	0.01787	0.01136	Reln/Thy1	14
GO:0015918	GO:0015918	sterol transport	6/389	86/23328	0.003170585	0.01787	0.01136	Cd36/Pltp	6
GO:0048708	GO:0048708	astrocyte differentiation	6/389	86/23328	0.003170585	0.01787	0.01136	Serpine2/	6
GO:0019233	GO:0019233	sensory perception of pain	8/389	146/23328	0.003188942	0.01794	0.01141	Ephx2/Ad	8
GO:0010524	GO:0010524	positive regulation of calcium ion transport into cytosol	5/389	60/23328	0.003236568	0.01814	0.01153	Thy1/Adrb	5
GO:0045744	GO:0045744	negative regulation of G protein-coupled receptor signa	5/389	60/23328	0.003236568	0.01814	0.01153	Adrb2/Rgs	5
GO:0048260	GO:0048260	positive regulation of receptor-mediated endocytosis	5/389	60/23328	0.003236568	0.01814	0.01153	B2m/Anxa	5
GO:1990748	GO:1990748	cellular detoxification	4/389	37/23328	0.003255833	0.01822	0.01159	Cd36/Prdx	4
GO:0019722	GO:0019722	calcium-mediated signaling	10/389	214/23328	0.003297852	0.01843	0.01172	Prnp/Atp1	10
GO:0060420	GO:0060420	regulation of heart growth	6/389	87/23328	0.003358641	0.01874	0.01192	Rgs4/Slc2	6
GO:0008154	GO:0008154	actin polymerization or depolymerization	10/389	215/23328	0.003408871	0.019	0.01208	Gmfg/Cdc	10
GO:0048286	GO:0048286	lung alveolus development	5/389	61/23328	0.003478224	0.01936	0.01231	Igfbp5/Flt	5
GO:0031960	GO:0031960	response to corticosteroid	6/389	88/23328	0.003554876	0.01965	0.0125	Ass1/Bmp	6
GO:0032436	GO:0032436	positive regulation of proteasomal ubiquitin-dependent	6/389	88/23328	0.003554876	0.01965	0.0125	Cav1/Plk2	6
GO:0035914	GO:0035914	skeletal muscle cell differentiation	6/389	88/23328	0.003554876	0.01965	0.0125	Tbx1/Nr4a	6
GO:0046683	GO:0046683	response to organophosphorus	6/389	88/23328	0.003554876	0.01965	0.0125	Igfbp5/Aq	6
GO:0062207	GO:0062207	regulation of pattern recognition receptor signaling path	6/389	88/23328	0.003554876	0.01965	0.0125	Lbp/Cd36	6
GO:0035335	GO:0035335	peptidyl-tyrosine dephosphorylation	4/389	38/23328	0.003591691	0.01972	0.01254	Ptprb/Ptpr	4
GO:0043267	GO:0043267	negative regulation of potassium ion transport	4/389	38/23328	0.003591691	0.01972	0.01254	Rgs4/Ank	4
GO:0070528	GO:0070528	protein kinase C signaling	4/389	38/23328	0.003591691	0.01972	0.01254	Flt4/Pdgfb	4
GO:0072210	GO:0072210	metanephric nephron development	4/389	38/23328	0.003591691	0.01972	0.01254	Bmp4/Pdg	4
GO:1904037	GO:1904037	positive regulation of epithelial cell apoptotic process	4/389	38/23328	0.003591691	0.01972	0.01254	Rgcc/Col1	4
GO:0007613	GO:0007613	memory	8/389	149/23328	0.003609745	0.01979	0.01259	Reln/Ptn/L	8
GO:0016486	GO:0016486	peptide hormone processing	3/389	19/23328	0.003655857	0.01991	0.01266	Pcsk6/Ece	3
GO:0034111	GO:0034111	negative regulation of homotypic cell-cell adhesion	3/389	19/23328	0.003655857	0.01991	0.01266	Cd9/Serpi	3
GO:0060544	GO:0060544	regulation of necroptotic process	3/389	19/23328	0.003655857	0.01991	0.01266	Cav1/Slc2	3
GO:0062098	GO:0062098	regulation of programmed necrotic cell death	3/389	19/23328	0.003655857	0.01991	0.01266	Cav1/Slc2	3
GO:0071071	GO:0071071	regulation of phospholipid biosynthetic process	3/389	19/23328	0.003655857	0.01991	0.01266	Adgrf5/Pd	3
GO:0021782	GO:0021782	glial cell development	7/389	118/23328	0.003679204	0.01999	0.01271	Ndrg1/Vim	7
GO:0060419	GO:0060419	heart growth	7/389	118/23328	0.003679204	0.01999	0.01271	Rgs4/Slc2	7
GO:1905207	GO:1905207	regulation of cardiocyte differentiation	5/389	62/23328	0.00373257	0.02025	0.01288	Sox17/Bm	5
GO:0014031	GO:0014031	mesenchymal cell development	6/389	89/23328	0.003759503	0.02034	0.01293	Tbx1/Bmp	6
GO:0043903	GO:0043903	regulation of symbiotic process	10/389	218/23328	0.003759852	0.02034	0.01293	Rsad2/Oa	10
GO:0006631	GO:0006631	fatty acid metabolic process	15/389	408/23328	0.003776081	0.0204	0.01297	Ephx2/Fal	15
GO:0048872	GO:0048872	homeostasis of number of cells	13/389	330/23328	0.003841894	0.02073	0.01318	Epas1/Bm	13
GO:0003230	GO:0003230	cardiac atrium development	4/389	39/23328	0.00395031	0.02129	0.01354	Gja5/Prox	4
GO:0048864	GO:0048864	stem cell development	6/389	90/23328	0.003972738	0.02138	0.0136	Tbx1/Bmp	6
GO:0031644	GO:0031644	regulation of nervous system process	9/389	185/23328	0.003998485	0.02141	0.01362	Reln/Adrb	9
GO:0006721	GO:0006721	terpenoid metabolic process	5/389	63/23328	0.003999969	0.02141	0.01362	Adh7/Rbp	5
GO:0010830	GO:0010830	regulation of myotube differentiation	5/389	63/23328	0.003999969	0.02141	0.01362	Tbx1/Cxcl	5
GO:1903428	GO:1903428	positive regulation of reactive oxygen species biosynth	5/389	63/23328	0.003999969	0.02141	0.01362	Ass1/Cd3	5
GO:0002573	GO:0002573	myeloid leukocyte differentiation	10/389	220/23328	0.004009325	0.02144	0.01363	Bmp4/Anx	10
GO:0032680	GO:0032680	regulation of tumor necrosis factor production	7/389	120/23328	0.004036186	0.02152	0.01369	Lbp/Cd34	7
GO:0033559	GO:0033559	unsaturated fatty acid metabolic process	7/389	120/23328	0.004036186	0.02152	0.01369	Ephx2/Fal	7
GO:0051604	GO:0051604	protein maturation	12/389	294/23328	0.004069393	0.02163	0.01375	Srgn/Duo	12
GO:0050729	GO:0050729	positive regulation of inflammatory response	8/389	152/23328	0.00407231	0.02163	0.01375	Lbp/Fabp	8
GO:0090288	GO:0090288	negative regulation of cellular response to growth facto	8/389	152/23328	0.00407231	0.02163	0.01375	Ptprf/Sfrp	8
GO:0032611	GO:0032611	interleukin-1 beta production	6/389	91/23328	0.004194794	0.02222	0.01413	Cd36/Hsp	6
GO:0044344	GO:0044344	cellular response to fibroblast growth factor stimulus	6/389	91/23328	0.004194794	0.02222	0.01413	Tbx1/Nr4a	6
GO:2001233	GO:2001233	regulation of apoptotic signaling pathway	15/389	413/23328	0.004221834	0.02228	0.01416	Cxcl12/Br	15
GO:0002688	GO:0002688	regulation of leukocyte chemotaxis	7/389	121/23328	0.004224225	0.02228	0.01416	Lbp/Ptn/L	7
GO:0055088	GO:0055088	lipid homeostasis	8/389	153/23328	0.004236238	0.02228	0.01416	Ephx2/Fal	8
GO:0008209	GO:0008209	androgen metabolic process	3/389	20/23328	0.004248328	0.02228	0.01416	Spp1/Sca	3
GO:0010875	GO:0010875	positive regulation of cholesterol efflux	3/389	20/23328	0.004248328	0.02228	0.01416	Pltp/Cav1	3
GO:0035584	GO:0035584	calcium-mediated signaling using intracellular calcium	3/389	20/23328	0.004248328	0.02228	0.01416	Prnp/Vcar	3
GO:0060547	GO:0060547	negative regulation of necrotic cell death	3/389	20/23328	0.004248328	0.02228	0.01416	Cav1/Slc2	3
GO:0140448	GO:0140448	signaling receptor ligand precursor processing	3/389	20/23328	0.004248328	0.02228	0.01416	Pcsk6/Ece	3
GO:0043547	GO:0043547	positive regulation of GTPase activity	10/389	222/23328	0.004271711	0.02237	0.01422	Thy1/Ccl2	10
GO:0035904	GO:0035904	aorta development	5/389	64/23328	0.004280784	0.02237	0.01423	Tbx1/Prox	5
GO:0030041	GO:0030041	actin filament polymerization	9/389	187/23328	0.004289065	0.02237	0.01423	Gmfg/Cdc	9

GO:0034767	GO:0034767	positive regulation of ion transmembrane transport	9/389	187/23328	0.004289065	0.02237	0.01423	Reln/Thy1	9
GO:0070570	GO:0070570	regulation of neuron projection regeneration	4/389	40/23328	0.00433239	0.02257	0.01435	Thy1/Ptn/I	4
GO:0031109	GO:0031109	microtubule polymerization or depolymerization	7/389	122/23328	0.004418818	0.02296	0.0146	Tppp3/Ca	7
GO:1903555	GO:1903555	regulation of tumor necrosis factor superfamily cytokine	7/389	122/23328	0.004418818	0.02296	0.0146	Lbp/Cd34/	7
GO:0055072	GO:0055072	iron ion homeostasis	6/389	92/23328	0.004425888	0.02297	0.01461	Cp/Lcn2/E	6
GO:0045637	GO:0045637	regulation of myeloid cell differentiation	10/389	224/23328	0.004547464	0.02357	0.01499	B2m/Rbp1	10
GO:0032233	GO:0032233	positive regulation of actin filament bundle assembly	5/389	65/23328	0.004575377	0.0236	0.015	Rgcc/Id1/	5
GO:0034121	GO:0034121	regulation of toll-like receptor signaling pathway	5/389	65/23328	0.004575377	0.0236	0.015	Lbp/Cd36/	5
GO:0034308	GO:0034308	primary alcohol metabolic process	5/389	65/23328	0.004575377	0.0236	0.015	Adh7/Dab	5
GO:0090398	GO:0090398	cellular senescence	5/389	65/23328	0.004575377	0.0236	0.015	B2m/Cav1	5
GO:0001894	GO:0001894	tissue homeostasis	11/389	261/23328	0.004606564	0.02373	0.01509	Cldn5/Epa	11
GO:0002028	GO:0002028	regulation of sodium ion transport	6/389	93/23328	0.004666235	0.024	0.01526	Fxyd6/Ser	6
GO:0002686	GO:0002686	negative regulation of leukocyte migration	4/389	41/23328	0.004738622	0.0242	0.01539	Cd200/Cx	4
GO:0034205	GO:0034205	amyloid-beta formation	4/389	41/23328	0.004738622	0.0242	0.01539	Prnp/Apoe	4
GO:0046164	GO:0046164	alcohol catabolic process	4/389	41/23328	0.004738622	0.0242	0.01539	Apoe/Sca	4
GO:0086009	GO:0086009	membrane repolarization	4/389	41/23328	0.004738622	0.0242	0.01539	Gja5/Cav1	4
GO:1902991	GO:1902991	regulation of amyloid precursor protein catabolic proce	4/389	41/23328	0.004738622	0.0242	0.01539	Prnp/Apoe	4
GO:0009991	GO:0009991	response to extracellular stimulus	14/389	378/23328	0.00474099	0.0242	0.01539	Adrb2/Spp	14
GO:0043500	GO:0043500	muscle adaptation	7/389	124/23328	0.004828235	0.02459	0.01563	Igfbp5/Fbx	7
GO:1903052	GO:1903052	positive regulation of proteolysis involved in cellular pr	7/389	124/23328	0.004828235	0.02459	0.01563	Ecscr/Cav	7
GO:0042982	GO:0042982	amyloid precursor protein metabolic process	5/389	66/23328	0.004884105	0.02478	0.01575	Prnp/Apoe	5
GO:0090559	GO:0090559	regulation of membrane permeability	5/389	66/23328	0.004884105	0.02478	0.01575	Lbp/Ier3/F	5
GO:0002043	GO:0002043	blood vessel endothelial cell proliferation involved in sp	3/389	21/23328	0.004895727	0.02478	0.01575	Bmp4/Apl	3
GO:0035357	GO:0035357	peroxisome proliferator activated receptor signaling pa	3/389	21/23328	0.004895727	0.02478	0.01575	Fabp5/Act	3
GO:0043117	GO:0043117	positive regulation of vascular permeability	3/389	21/23328	0.004895727	0.02478	0.01575	Apoe/Pde	3
GO:0015908	GO:0015908	fatty acid transport	6/389	94/23328	0.004916052	0.02482	0.01578	Fabp4/Cd	6
GO:0070252	GO:0070252	actin-mediated cell contraction	6/389	94/23328	0.004916052	0.02482	0.01578	Gja5/Cav1	6
GO:0010212	GO:0010212	response to ionizing radiation	7/389	125/23328	0.005043345	0.02543	0.01617	Sfrp2/Net1	7
GO:0001709	GO:0001709	cell fate determination	4/389	42/23328	0.005169677	0.02597	0.01651	Sox17/Bm	4
GO:0006509	GO:0006509	membrane protein ectodomain proteolysis	4/389	42/23328	0.005169677	0.02597	0.01651	Timp3/Tim	4
GO:0007229	GO:0007229	integrin-mediated signaling pathway	6/389	95/23328	0.005175553	0.02597	0.01651	Thy1/Ptn/I	6
GO:0031343	GO:0031343	positive regulation of cell killing	6/389	95/23328	0.005175553	0.02597	0.01651	H2-Q6/B2	6
GO:0006940	GO:0006940	regulation of smooth muscle contraction	5/389	67/23328	0.005207325	0.02606	0.01657	Adrb2/Cav	5
GO:0046847	GO:0046847	filopodium assembly	5/389	67/23328	0.005207325	0.02606	0.01657	Gap43/Gp	5
GO:0001889	GO:0001889	liver development	7/389	126/23328	0.005265582	0.02626	0.0167	Ptn/Vwf/B	7
GO:0032640	GO:0032640	tumor necrosis factor production	7/389	126/23328	0.005265582	0.02626	0.0167	Lbp/Cd34/	7
GO:1901216	GO:1901216	positive regulation of neuron death	7/389	126/23328	0.005265582	0.02626	0.0167	Ptprf/Prnp	7
GO:0045580	GO:0045580	regulation of T cell differentiation	8/389	159/23328	0.005329076	0.02654	0.01688	Dtx1/Fgl2/	8
GO:0002690	GO:0002690	positive regulation of leukocyte chemotaxis	6/389	96/23328	0.005444956	0.02709	0.01722	Lbp/Ptn/Lg	6
GO:0002793	GO:0002793	positive regulation of peptide secretion	10/389	230/23328	0.005459577	0.02712	0.01725	Rgcc/Cd3	10
GO:0060048	GO:0060048	cardiac muscle contraction	7/389	127/23328	0.005495088	0.02727	0.01734	Gja5/Cav1	7
GO:0051302	GO:0051302	regulation of cell division	8/389	160/23328	0.005530376	0.02735	0.01739	Ptn/Sox17	8
GO:0070838	GO:0070838	divalent metal ion transport	16/389	467/23328	0.005539346	0.02735	0.01739	Thy1/Prnp	16
GO:0030837	GO:0030837	negative regulation of actin filament polymerization	5/389	68/23328	0.005545394	0.02735	0.01739	Pecam1/T	5
GO:1901264	GO:1901264	carbohydrate derivative transport	5/389	68/23328	0.005545394	0.02735	0.01739	Lbp/Pltp/S	5
GO:2001244	GO:2001244	positive regulation of intrinsic apoptotic signaling pathw	5/389	68/23328	0.005545394	0.02735	0.01739	Cav1/Clu/	5
GO:0072111	GO:0072111	cell proliferation involved in kidney development	3/389	22/23328	0.005599423	0.02748	0.01748	Bmp4/Pdg	3
GO:0072215	GO:0072215	regulation of metanephros development	3/389	22/23328	0.005599423	0.02748	0.01748	Bmp4/Pdg	3
GO:1901741	GO:1901741	positive regulation of myoblast fusion	3/389	22/23328	0.005599423	0.02748	0.01748	Cxcl12/Cx	3
GO:2001026	GO:2001026	regulation of endothelial cell chemotaxis	3/389	22/23328	0.005599423	0.02748	0.01748	Lgmn/Hsp	3
GO:0050731	GO:0050731	positive regulation of peptidyl-tyrosine phosphorylation	9/389	195/23328	0.005619643	0.02752	0.0175	Reln/Cd36	9
GO:2001234	GO:2001234	negative regulation of apoptotic signaling pathway	10/389	231/23328	0.005624589	0.02752	0.0175	Cxcl12/Bn	10
GO:1903053	GO:1903053	regulation of extracellular matrix organization	4/389	43/23328	0.005626216	0.02752	0.0175	Rgcc/Tcf1	4
GO:0002027	GO:0002027	regulation of heart rate	6/389	97/23328	0.005724475	0.02786	0.01772	Epas1/Gja	6
GO:0002709	GO:0002709	regulation of T cell mediated immunity	6/389	97/23328	0.005724475	0.02786	0.01772	H2-Q6/Rs	6
GO:0010660	GO:0010660	regulation of muscle cell apoptotic process	6/389	97/23328	0.005724475	0.02786	0.01772	Fndc1/Fbx	6
GO:0071706	GO:0071706	tumor necrosis factor superfamily cytokine production	7/389	128/23328	0.005732008	0.02786	0.01772	Lbp/Cd34/	7
GO:0006694	GO:0006694	steroid biosynthetic process	8/389	161/23328	0.005737424	0.02786	0.01772	Igfbp7/Cyl	8
GO:0030178	GO:0030178	negative regulation of Wnt signaling pathway	8/389	161/23328	0.005737424	0.02786	0.01772	Sox17/Tm	8
GO:0072330	GO:0072330	monocarboxylic acid biosynthetic process	9/389	196/23328	0.005806148	0.02816	0.01791	Fabp5/Mg	9
GO:0072511	GO:0072511	divalent inorganic cation transport	16/389	470/23328	0.005878787	0.02847	0.01811	Thy1/Prnp	16
GO:0001913	GO:0001913	T cell mediated cytotoxicity	5/389	69/23328	0.005898661	0.02847	0.01811	H2-Q6/B2	5
GO:0006801	GO:0006801	superoxide metabolic process	5/389	69/23328	0.005898661	0.02847	0.01811	Cd36/Prdx	5
GO:0045600	GO:0045600	positive regulation of fat cell differentiation	5/389	69/23328	0.005898661	0.02847	0.01811	Sfrp2/Zfp3	5
GO:0022898	GO:0022898	regulation of transmembrane transporter activity	11/389	270/23328	0.005908444	0.02849	0.01811	Reln/Fxyd	11
GO:0002822	GO:0002822	regulation of adaptive immune response based on som	9/389	197/23328	0.005997395	0.02886	0.01835	H2-Q6/Rs	9
GO:0008202	GO:0008202	steroid metabolic process	12/389	309/23328	0.006000247	0.02886	0.01835	Ephx2/Igfl	12
GO:0045823	GO:0045823	positive regulation of heart contraction	4/389	44/23328	0.006108881	0.02928	0.01862	Adrb2/Rgs	4
GO:0048821	GO:0048821	erythrocyte development	4/389	44/23328	0.006108881	0.02928	0.01862	Adgrf5/Klf	4
GO:0097237	GO:0097237	cellular response to toxic substance	4/389	44/23328	0.006108881	0.02928	0.01862	Cd36/Prdx	4
GO:2000116	GO:2000116	regulation of cysteine-type endopeptidase activity	10/389	234/23328	0.006143104	0.02941	0.0187	Lgmn/Sfrp	10
GO:0045185	GO:0045185	maintenance of protein location	6/389	99/23328	0.006314727	0.0302	0.0192	Srgn/Ank3	6
GO:0034383	GO:0034383	low-density lipoprotein particle clearance	3/389	23/23328	0.006360668	0.03024	0.01923	Cd36/Anx	3
GO:0034405	GO:0034405	response to fluid shear stress	3/389	23/23328	0.006360668	0.03024	0.01923	Ass1/Spp	3
GO:0048333	GO:0048333	mesodermal cell differentiation	3/389	23/23328	0.006360668	0.03024	0.01923	Bmp4/Sfrp	3
GO:0051043	GO:0051043	regulation of membrane protein ectodomain proteolysis	3/389	23/23328	0.006360668	0.03024	0.01923	Timp3/Tim	3
GO:2000810	GO:2000810	regulation of bicellular tight junction assembly	3/389	23/23328	0.006360668	0.03024	0.01923	Cldn5/F11	3
GO:0010769	GO:0010769	regulation of cell morphogenesis involved in differentia	13/389	351/23328	0.006378925	0.03029	0.01926	Reln/Thy1	13
GO:0034249	GO:0034249	negative regulation of cellular amide metabolic proces	8/389	164/23328	0.006394092	0.03033	0.01928	Igfbp5/Prr	8
GO:0002699	GO:0002699	positive regulation of immune effector process	11/389	273/23328	0.006401452	0.03033	0.01928	Lbp/Cd36/	11
GO:0051099	GO:0051099	positive regulation of binding	9/389	200/23328	0.006600372	0.03119	0.01984	Cldn5/Bm	9
GO:0051216	GO:0051216	cartilage development	9/389	200/23328	0.006600372	0.03119	0.01984	Maf/Bmp4	9
GO:0061756	GO:0061756	leukocyte adhesion to vascular endothelial cell	4/389	45/23328	0.006618298	0.03121	0.01984	Cxcl12/Ja	4
GO:0042116	GO:0042116	macrophage activation	6/389	100/23328	0.006625889	0.03121	0.01984	Lbp/Cd20	6
GO:0046889	GO:0046889	positive regulation of lipid biosynthetic process	6/389	100/23328	0.006625889	0.03121	0.01984	Adgrf5/Ap	6
GO:0048863	GO:0048863	stem cell differentiation	10/389	237/23328	0.006698112	0.03151	0.02004	Ptn/Tbx1/	10
GO:2000060	GO:2000060	positive regulation of ubiquitin-dependent protein catab	6/389	101/23328	0.006948027	0.03265	0.02076	Cav1/Plk2	6
GO:0051607	GO:0051607	defense response to virus	10/389	239/23328	0.007089213	0.03327	0.02116	Fgl2/Rsad	10
GO:2001242	GO:2001242	regulation of intrinsic apoptotic signaling pathway	8/389	167/23328	0.007106234	0.03332	0.02118	Cxcl12/Hs	8
GO:0006636	GO:0006636	unsaturated fatty acid biosynthetic process	4/389	46/23328	0.007155076	0.03335	0.02121	Fabp5/Ptg	4
GO:0031952	GO:0031952	regulation of protein autophosphorylation	4/389	46/23328	0.007155076	0.03335	0.02121	Cav1/Pdg	4
GO:0033344	GO:0033344	cholesterol efflux	4/389	46/23328	0.007155076	0.03335	0.02121	Pltp/Cav1	4
GO:0043616	GO:0043616	keratinocyte proliferation	4/389	46/23328	0.007155076	0.03335	0.02121	Cdh13/Klk	4
GO:0061098	GO:0061098	positive regulation of protein tyrosine kinase activity	4/389	46/23328	0.007155076	0.03335	0.02121	Reln/Prnp	4
GO:0045736	GO:0045736	negative regulation of cyclin-dependent protein serine/	3/389	24/23328	0.007180604	0.03339	0.02123	Nr2f2/Cdk	3
GO:0050687	GO:0050687	negative regulation of defense response to virus	3/389	24/23328	0.007180604	0.03339	0.02123	Fgl2/Tspa	3
GO:0010657	GO:0010657	muscle cell apoptotic process	6/389	102/23328	0.007281355	0.03382	0.02151	Fndc1/Fbx	6
GO:0045995	GO:0045995	regulation of embryonic development	7/389	134/23328	0.007317343	0.03395	0.02159	Sox17/Bm	7
GO:0051047	GO:0051047	positive regulation of secretion	15/389	440/23328	0.00742933	0.03443	0.0219	Rgcc/Cd3	15
GO:0001822	GO:0001822	kidney development	12/389	318/23328	0.007468717	0.03458	0.02199	Id3/Sox17	12
GO:0010522	GO:0010522	regulation of calcium ion transport into cytosol	6/389	103/23328	0.007626085	0.03519	0.02237	Thy1/Prnp	6
GO:0015748	GO:0015748	organophosphate ester transport	6/389	103/23328	0.007626085	0.03519	0.02237	Pltp/Apoe	6
GO:0055024	GO:0055024	regulation of cardiac muscle tissue development	6/389	103/23328	0.007626085	0.03519	0.02237	Bmp4/Rgs	6
GO:0032409	GO:0032409	regulation of transporter activity	11/389	280/23328	0.007677487	0.03538	0.0225	Reln/Fxyd	11
GO:0045214	GO:0045214	sarcomere organization	4/389	47/23328	0.007719809	0.03546	0.02255	Prox1/Csr	4
GO:0046460	GO:0046460	neutral lipid biosynthetic process	4/389	47/23328	0.007719809	0.03546	0.02255	Thrsp/Sca	4
GO:0046463	GO:0046463	acylglycerol biosynthetic process	4/389	47/23328	0.007719809	0.03546	0.02255	Thrsp/Sca	4
GO:1904064	GO:1904064	positive regulation of cation transmembrane transport	8/389	170/23328	0.00787668	0.03614	0.02298	Reln/Thy1	8
GO:0014074	GO:0014074	response to purine-containing compound	6/389	104/23328	0.007982428	0.0365	0.02321	Igfbp5/Aq	6
GO:0046916	GO:0046916	cellular transition metal ion homeostasis	6/389	104/23328	0.007982428	0.0365	0.02321	Cp/Lcn2/F	6
GO:0048525	GO:0048525	negative regulation of viral process	6/389	104/23328	0.007982428	0.0365	0.02321	Rsad2/Oa	6
GO:0006706	GO:0006706	steroid catabolic process	3/389	25/23328	0.008060263	0.03653	0.02323	Spp1/Apo	3
GO:0010165	GO:0010165	response to X-ray	3/389	25/23328	0.008060263	0.03653	0.02323	Sfrp2/Anx	3
GO:0010884	GO:0010884	positive regulation of lipid storage	3/389	25/23328	0.008060263	0.03653	0.02323	Cd36/Sca	3
GO:0032703	GO:0032703	negative regulation of interleukin-2 production	3/389	25/23328	0.008060263	0.03653	0.02323	Cd34/Prnp	3
GO:0043567	GO:0043567	regulation of insulin-like growth factor receptor signalin	3/389	25/23328	0.008060263	0.03653	0.02323	Igfbp5/Igfl	3
GO:1901739	GO:1901739	regulation of myoblast fusion	3/389	25/23328	0.008060263	0.03653	0.02323	Cxcl12/Cx	3
GO:1902230	GO:1902230	negative regulation of intrinsic apoptotic signaling path	3/389	25/23328	0.008060263	0.03653	0.02323	Cxcl12/Clu	3
GO:1902993	GO:1902993	positive regulation of amyloid precursor protein catabo	3/389	25/23328	0.008060263	0.03653	0.02323	Apoe/Clu/	3
GO:0030522	GO:0030522	intracellular receptor signaling pathway	9/389	207/23328	0.008188001	0.03706	0.02357	Tbx1/Fabp	9
GO:0010862	GO:0010862	positive regulation of pathway-restricted SMAD protein	4/389	48/23328	0.00831307	0.03755	0.02387	Bmp4/Eng	4
GO:0045429	GO:0045429	positive regulation of nitric oxide biosynthetic process	4/389	48/23328	0.00831307	0.03755	0.02387	Ass1/Cd3	4
GO:0051057	GO:0051057	positive regulation of small GTPase mediated signal tr	5/389	75/23328	0.008356759	0.03766	0.02395	Reln/F11r	5
GO:1903670	GO:1903670	regulation of sprouting angiogenesis	5/389	75/23328	0.008356759	0.03766	0.02395	Aplnr/Klf2	5
GO:0006066	GO:0006066	alcohol metabolic process	12/389	323/23328	0.008398703	0.03781	0.02404	Ephx2/Cy	12
GO:0007187	GO:0007187	G protein-coupled receptor signaling pathway, coupled	10/389	246/23328	0.008598824	0.03867	0.02459	Adrb2/Adc	10
GO:0014812	GO:0014812	muscle cell migration	6/389	106/23328	0.008730794	0.03917	0.02491	Igfbp5/Pd	6
GO:0034446	GO:0034446	substrate adhesion-dependent cell spreading	6/389	106/23328	0.008730794	0.03917	0.02491	Pdpn/Actr	6
GO:0090207	GO:0090207	regulation of triglyceride metabolic process	4/389	49/23328	0.008935417	0.03994	0.0254	Cd36/Thrs	4

GO:1904407	GO:1904407	positive regulation of nitric oxide metabolic process	4/389	49/23328	0.008935417	0.03994	0.0254	Ass1/Cd3	4
GO:2000725	GO:2000725	regulation of cardiac muscle cell differentiation	4/389	49/23328	0.008935417	0.03994	0.0254	Bmp4/Rgs	4
GO:0033622	GO:0033622	integrin activation	3/389	26/23328	0.009000577	0.03994	0.0254	Cxcl12/Se	3
GO:0034123	GO:0034123	positive regulation of toll-like receptor signaling pathwa	3/389	26/23328	0.009000577	0.03994	0.0254	Lbp/Rsad2	3
GO:0071549	GO:0071549	cellular response to dexamethasone stimulus	3/389	26/23328	0.009000577	0.03994	0.0254	Bmp4/Fbx	3
GO:0098901	GO:0098901	regulation of cardiac muscle cell action potential	3/389	26/23328	0.009000577	0.03994	0.0254	Gja5/Ank3	3
GO:1901380	GO:1901380	negative regulation of potassium ion transmembrane t	3/389	26/23328	0.009000577	0.03994	0.0254	Rgs4/Ank	3
GO:1904030	GO:1904030	negative regulation of cyclin-dependent protein kinase	3/389	26/23328	0.009000577	0.03994	0.0254	Nr2f2/Cdk	3
GO:2000050	GO:2000050	regulation of non-canonical Wnt signaling pathway	3/389	26/23328	0.009000577	0.03994	0.0254	Sfrp2/Dab	3
GO:0042391	GO:0042391	regulation of membrane potential	15/389	450/23328	0.009023254	0.04	0.02544	Reln/Ptn/C	15
GO:0032496	GO:0032496	response to lipopolysaccharide	13/389	368/23328	0.00928506	0.04111	0.02614	Lbp/Cd36/	13
GO:0007612	GO:0007612	learning	8/389	175/23328	0.009298039	0.04111	0.02614	Reln/Ptn/N	8
GO:0001756	GO:0001756	somitogenesis	5/389	77/23328	0.009313127	0.04111	0.02614	Tcf15/Sfrp	5
GO:0045453	GO:0045453	bone resorption	5/389	77/23328	0.009313127	0.04111	0.02614	Adrb2/Spp	5
GO:0006641	GO:0006641	triglyceride metabolic process	6/389	108/23328	0.009528122	0.04196	0.02668	Cd36/Thrs	6
GO:0055017	GO:0055017	cardiac muscle tissue growth	6/389	108/23328	0.009528122	0.04196	0.02668	Rgs4/Slc2	6
GO:0007520	GO:0007520	myoblast fusion	4/389	50/23328	0.009587387	0.04213	0.02679	Cd9/Cxcl1	4
GO:0045646	GO:0045646	regulation of erythrocyte differentiation	4/389	50/23328	0.009587387	0.04213	0.02679	B2m/Zfp3	4
GO:0007369	GO:0007369	gastrulation	8/389	176/23328	0.009603851	0.04216	0.02681	Sox17/Bm	8
GO:0046785	GO:0046785	microtubule polymerization	5/389	78/23328	0.009818462	0.04288	0.02727	Tppp3/Ca	5
GO:0050810	GO:0050810	regulation of steroid biosynthetic process	5/389	78/23328	0.009818462	0.04288	0.02727	Igfbp7/Ap	5
GO:0060976	GO:0060976	coronary vasculature development	5/389	78/23328	0.009818462	0.04288	0.02727	Tbx1/Bmp	5
GO:0070373	GO:0070373	negative regulation of ERK1 and ERK2 cascade	5/389	78/23328	0.009818462	0.04288	0.02727	Timp3/Ca	5
GO:1901215	GO:1901215	negative regulation of neuron death	10/389	251/23328	0.009820015	0.04288	0.02727	Cd34/Cd2	10
GO:0035051	GO:0035051	cardiocyte differentiation	8/389	177/23328	0.009917089	0.04326	0.02751	Sox17/Bm	8
GO:0032770	GO:0032770	positive regulation of monooxygenase activity	3/389	27/23328	0.010002375	0.04335	0.02757	Apoe/Sca	3
GO:0048745	GO:0048745	smooth muscle tissue development	3/389	27/23328	0.010002375	0.04335	0.02757	Bmp4/Pro	3
GO:0060343	GO:0060343	trabecula formation	3/389	27/23328	0.010002375	0.04335	0.02757	Mmp2/Tek	3
GO:0060713	GO:0060713	labyrinthine layer morphogenesis	3/389	27/23328	0.010002375	0.04335	0.02757	Socs3/Vca	3
GO:0071425	GO:0071425	hematopoietic stem cell proliferation	3/389	27/23328	0.010002375	0.04335	0.02757	Cd34/Sfrp	3
GO:0098581	GO:0098581	detection of external biotic stimulus	3/389	27/23328	0.010002375	0.04335	0.02757	Lbp/Scarb	3
GO:0002831	GO:0002831	regulation of response to biotic stimulus	11/389	291/23328	0.010073232	0.04361	0.02773	Lbp/Fgl2/S	11
GO:0050821	GO:0050821	protein stabilization	8/389	178/23328	0.010237858	0.04415	0.02808	Sox17/Tm	8
GO:0032330	GO:0032330	regulation of chondrocyte differentiation	4/389	51/23328	0.010269502	0.04415	0.02808	Maf/Bmp4	4
GO:0042987	GO:0042987	amyloid precursor protein catabolic process	4/389	51/23328	0.010269502	0.04415	0.02808	Prnp/Apoe	4
GO:0051489	GO:0051489	regulation of filopodium assembly	4/389	51/23328	0.010269502	0.04415	0.02808	Gap43/Gp	4
GO:0060412	GO:0060412	ventricular septum morphogenesis	4/389	51/23328	0.010269502	0.04415	0.02808	Gja5/Bmp	4
GO:0030326	GO:0030326	embryonic limb morphogenesis	7/389	143/23328	0.010274029	0.04415	0.02808	Hoxd9/Gja	7
GO:0035113	GO:0035113	embryonic appendage morphogenesis	7/389	143/23328	0.010274029	0.04415	0.02808	Hoxd9/Gja	7
GO:0048839	GO:0048839	inner ear development	9/389	215/23328	0.010340953	0.04427	0.02815	Maf/Igfbp7	9
GO:0051781	GO:0051781	positive regulation of cell division	5/389	79/23328	0.010342315	0.04427	0.02815	Ptn/Pdgfb	5
GO:0055021	GO:0055021	regulation of cardiac muscle tissue growth	5/389	79/23328	0.010342315	0.04427	0.02815	Rgs4/Slc2	5
GO:0048705	GO:0048705	skeletal system morphogenesis	10/389	253/23328	0.010343914	0.04427	0.02815	Hoxd9/Tcf	10
GO:0007156	GO:0007156	homophilic cell adhesion via plasma membrane adhes	6/389	110/23328	0.010376056	0.04436	0.02821	Ptprf/Esar	6
GO:0072001	GO:0072001	renal system development	12/389	333/23328	0.010528653	0.04496	0.02859	Id3/Sox17	12
GO:0072089	GO:0072089	stem cell proliferation	7/389	144/23328	0.010649255	0.04543	0.02889	Ptn/Cd34/	7
GO:0019218	GO:0019218	regulation of steroid metabolic process	6/389	111/23328	0.010819508	0.04611	0.02932	Ephx2/Igfl	6
GO:0032651	GO:0032651	regulation of interleukin-1 beta production	5/389	80/23328	0.010884993	0.04629	0.02943	Hspb1/Se	5
GO:1901862	GO:1901862	negative regulation of muscle tissue development	5/389	80/23328	0.010884993	0.04629	0.02943	Igfbp5/Bm	5
GO:0032731	GO:0032731	positive regulation of interleukin-1 beta production	4/389	52/23328	0.010982261	0.04661	0.02964	Hspb1/Mn	4
GO:0045124	GO:0045124	regulation of bone resorption	4/389	52/23328	0.010982261	0.04661	0.02964	Spp1/Cd3	4
GO:0010460	GO:0010460	positive regulation of heart rate	3/389	28/23328	0.011066394	0.04661	0.02964	Rgs4/Tpm	3
GO:0032801	GO:0032801	receptor catabolic process	3/389	28/23328	0.011066394	0.04661	0.02964	Lgmn/Anx	3
GO:0042744	GO:0042744	hydrogen peroxide catabolic process	3/389	28/23328	0.011066394	0.04661	0.02964	Prdx1/Hba	3
GO:0045737	GO:0045737	positive regulation of cyclin-dependent protein serine/t	3/389	28/23328	0.011066394	0.04661	0.02964	Rgcc/Pdg	3
GO:0045992	GO:0045992	negative regulation of embryonic development	3/389	28/23328	0.011066394	0.04661	0.02964	Bmp4/Sfrp	3
GO:0071480	GO:0071480	cellular response to gamma radiation	3/389	28/23328	0.011066394	0.04661	0.02964	Egr1/Crya	3
GO:0090025	GO:0090025	regulation of monocyte chemotaxis	3/389	28/23328	0.011066394	0.04661	0.02964	Lgmn/Cxc	3
GO:0097193	GO:0097193	intrinsic apoptotic signaling pathway	11/389	295/23328	0.011074091	0.04661	0.02964	Cxcl12/Hs	11
GO:0045088	GO:0045088	regulation of innate immune response	8/389	181/23328	0.011246397	0.04728	0.03007	Lbp/Serpi	8
GO:0003149	GO:0003149	membranous septum morphogenesis	2/389	10/23328	0.011423561	0.0473	0.03007	Bmp4/Id2	2
GO:0006707	GO:0006707	cholesterol catabolic process	2/389	10/23328	0.011423561	0.0473	0.03007	Apoe/Sca	2
GO:0015865	GO:0015865	purine nucleotide transport	2/389	10/23328	0.011423561	0.0473	0.03007	Slc25a4/G	2
GO:0016127	GO:0016127	sterol catabolic process	2/389	10/23328	0.011423561	0.0473	0.03007	Apoe/Sca	2
GO:0030049	GO:0030049	muscle filament sliding	2/389	10/23328	0.011423561	0.0473	0.03007	Tpm1/Tnn	2
GO:0051001	GO:0051001	negative regulation of nitric-oxide synthase activity	2/389	10/23328	0.011423561	0.0473	0.03007	Cav1/Eng	2
GO:0061517	GO:0061517	macrophage proliferation	2/389	10/23328	0.011423561	0.0473	0.03007	Clu/Il33	2
GO:0061626	GO:0061626	pharyngeal arch artery morphogenesis	2/389	10/23328	0.011423561	0.0473	0.03007	Bmp4/Adg	2
GO:0070391	GO:0070391	response to lipoteichoic acid	2/389	10/23328	0.011423561	0.0473	0.03007	Lbp/Cd36	2
GO:0071223	GO:0071223	cellular response to lipoteichoic acid	2/389	10/23328	0.011423561	0.0473	0.03007	Lbp/Cd36	2
GO:0072203	GO:0072203	cell proliferation involved in metanephros developmen	2/389	10/23328	0.011423561	0.0473	0.03007	Pdgfb/Egr	2
GO:0090269	GO:0090269	fibroblast growth factor production	2/389	10/23328	0.011423561	0.0473	0.03007	Rgcc/Cd5	2
GO:0090270	GO:0090270	regulation of fibroblast growth factor production	2/389	10/23328	0.011423561	0.0473	0.03007	Rgcc/Cd5	2
GO:1900122	GO:1900122	positive regulation of receptor binding	2/389	10/23328	0.011423561	0.0473	0.03007	B2m/Anxa	2
GO:1901724	GO:1901724	positive regulation of cell proliferation involved in kidne	2/389	10/23328	0.011423561	0.0473	0.03007	Pdgfb/Egr	2
GO:0051403	GO:0051403	stress-activated MAPK cascade	10/389	257/23328	0.011455254	0.04738	0.03013	Flt4/Prdx1	10
GO:0009896	GO:0009896	positive regulation of catabolic process	15/389	463/23328	0.01148828	0.04747	0.03018	Sox17/Ecs	15
GO:0048592	GO:0048592	eye morphogenesis	8/389	182/23328	0.011598335	0.04787	0.03044	Thy1/Ptn/	8
GO:0030514	GO:0030514	negative regulation of BMP signaling pathway	4/389	53/23328	0.011726147	0.04811	0.03059	Sfrp2/Cav	4
GO:0031113	GO:0031113	regulation of microtubule polymerization	4/389	53/23328	0.011726147	0.04811	0.03059	Cav1/Cdh	4
GO:0032371	GO:0032371	regulation of sterol transport	4/389	53/23328	0.011726147	0.04811	0.03059	Pltp/Anxa	4
GO:0032374	GO:0032374	regulation of cholesterol transport	4/389	53/23328	0.011726147	0.04811	0.03059	Pltp/Anxa	4
GO:0048247	GO:0048247	lymphocyte chemotaxis	4/389	53/23328	0.011726147	0.04811	0.03059	Ccl21a/Cx	4
GO:0048662	GO:0048662	negative regulation of smooth muscle cell proliferation	4/389	53/23328	0.011726147	0.04811	0.03059	Igfbp5/Ca	4
GO:0050764	GO:0050764	regulation of phagocytosis	6/389	113/23328	0.011746382	0.04814	0.03061	Lbp/Cd36/	6
GO:0002824	GO:0002824	positive regulation of adaptive immune response base	7/389	147/23328	0.011834672	0.04841	0.03078	H2-Q6/Rs	7
GO:0035270	GO:0035270	endocrine system development	7/389	147/23328	0.011834672	0.04841	0.03078	Tbx1/Bmp	7
GO:0060249	GO:0060249	anatomical structure homeostasis	14/389	422/23328	0.011867456	0.04847	0.03082	Cldn5/Epa	14
GO:0051402	GO:0051402	neuron apoptotic process	11/389	298/23328	0.011873408	0.04847	0.03082	Ptprf/Prnp	11
GO:0001912	GO:0001912	positive regulation of leukocyte mediated cytotoxicity	5/389	82/23328	0.012028024	0.049	0.03116	H2-Q6/B2	5
GO:0019915	GO:0019915	lipid storage	5/389	82/23328	0.012028024	0.049	0.03116	Cd36/Cav	5
GO:0046394	GO:0046394	carboxylic acid biosynthetic process	11/389	299/23328	0.012149398	0.04933	0.03137	Ass1/Fab	11
GO:0001919	GO:0001919	regulation of receptor recycling	3/389	29/23328	0.012193278	0.04933	0.03137	Anxa2/Da	3
GO:0003085	GO:0003085	negative regulation of systemic arterial blood pressure	3/389	29/23328	0.012193278	0.04933	0.03137	Cd36/Adrl	3
GO:0033273	GO:0033273	response to vitamin	3/389	29/23328	0.012193278	0.04933	0.03137	Spp1/Gas	3
GO:0048643	GO:0048643	positive regulation of skeletal muscle tissue developme	3/389	29/23328	0.012193278	0.04933	0.03137	Tbx1/Adrb	3
GO:0061050	GO:0061050	regulation of cell growth involved in cardiac muscle cel	3/389	29/23328	0.012193278	0.04933	0.03137	Rgs4/Slc2	3
GO:0071548	GO:0071548	response to dexamethasone	3/389	29/23328	0.012193278	0.04933	0.03137	Bmp4/Fbx	3
GO:0071346	GO:0071346	cellular response to interferon-gamma	6/389	114/23328	0.012230198	0.04943	0.03143	Ccl21a/Mr	6
GO:0051091	GO:0051091	positive regulation of DNA-binding transcription factor	10/389	260/23328	0.012346379	0.04985	0.0317	Reln/Rgcc	10
GO:0016053	GO:0016053	organic acid biosynthetic process	11/389	300/23328	0.012430251	0.05013	0.03188	Ass1/Fab	11
GO:0014009	GO:0014009	glial cell proliferation	4/389	54/23328	0.012501623	0.05013	0.03188	Ptn/Vim/C	4
GO:0034113	GO:0034113	heterotypic cell-cell adhesion	4/389	54/23328	0.012501623	0.05013	0.03188	Thy1/Cd2	4
GO:0046456	GO:0046456	icosanoid biosynthetic process	4/389	54/23328	0.012501623	0.05013	0.03188	Fabp5/Ptg	4
GO:0061077	GO:0061077	chaperone-mediated protein folding	4/389	54/23328	0.012501623	0.05013	0.03188	Hspb1/Un	4
GO:1900024	GO:1900024	regulation of substrate adhesion-dependent cell sprea	4/389	54/23328	0.012501623	0.05013	0.03188	Pdpn/Actr	4
GO:2000649	GO:2000649	regulation of sodium ion transmembrane transporter a	4/389	54/23328	0.012501623	0.05013	0.03188	Fxyd6/Ank	4
GO:1904427	GO:1904427	positive regulation of calcium ion transmembrane trans	5/389	83/23328	0.012628963	0.05059	0.03217	Thy1/Adrb	5
GO:0018958	GO:0018958	phenol-containing compound metabolic process	6/389	115/23328	0.012727858	0.05084	0.03233	Reln/Epas	6
GO:0048259	GO:0048259	regulation of receptor-mediated endocytosis	6/389	115/23328	0.012727858	0.05084	0.03233	Sh3gl3/B2	6
GO:0090100	GO:0090100	positive regulation of transmembrane receptor protein	6/389	115/23328	0.012727858	0.05084	0.03233	Bmp4/Lrg	6
GO:2000045	GO:2000045	regulation of G1/S transition of mitotic cell cycle	6/389	116/23328	0.013239555	0.05254	0.03341	Rgcc/Pdp	6
GO:0006720	GO:0006720	isoprenoid metabolic process	5/389	84/23328	0.013249901	0.05254	0.03341	Adh7/Rbp	5
GO:0043154	GO:0043154	negative regulation of cysteine-type endopeptidase ac	5/389	84/23328	0.013249901	0.05254	0.03341	Sfrp2/Nr4	5
GO:0050766	GO:0050766	positive regulation of phagocytosis	5/389	84/23328	0.013249901	0.05254	0.03341	Lbp/Cd36/	5
GO:0051817	GO:0051817	modulation of process of other organism involved in sy	5/389	84/23328	0.013249901	0.05254	0.03341	Anxa2/Ap	5
GO:0061515	GO:0061515	myeloid cell development	5/389	84/23328	0.013249901	0.05254	0.03341	Anxa2/Ad	5
GO:0043087	GO:0043087	regulation of GTPase activity	11/389	303/23328	0.013302512	0.05254	0.03341	Thy1/Ccl2	11
GO:0002720	GO:0002720	positive regulation of cytokine production involved in ir	4/389	55/23328	0.013309133	0.05254	0.03341	Cd36/Rsa	4
GO:0003044	GO:0003044	regulation of systemic arterial blood pressure mediatec	4/389	55/23328	0.013309133	0.05254	0.03341	Gja5/Adrb	4
GO:0010665	GO:0010665	regulation of cardiac muscle cell apoptotic process	4/389	55/23328	0.013309133	0.05254	0.03341	Fndc1/Fb	4
GO:0033619	GO:0033619	membrane protein proteolysis	4/389	55/23328	0.013309133	0.05254	0.03341	Timp3/Tim	4
GO:0051496	GO:0051496	positive regulation of stress fiber assembly	4/389	55/23328	0.013309133	0.05254	0.03341	Rgcc/Tpm	4
GO:0010874	GO:0010874	regulation of cholesterol efflux	3/389	30/23328	0.013383582	0.05258	0.03343	Pltp/Cav1	3
GO:0043552	GO:0043552	positive regulation of phosphatidylinositol 3-kinase acti	3/389	30/23328	0.013383582	0.05258	0.03343	Flt1/Pdgf	3
GO:0043951	GO:0043951	negative regulation of cAMP-mediated signaling	3/389	30/23328	0.013383582	0.05258	0.03343	Aplnr/Pde	3
GO:0060143	GO:0060143	positive regulation of syncytium formation by plasma m	3/389	30/23328	0.013383582	0.05258	0.03343	Cxcl12/Cx	3
GO:0120178	GO:0120178	steroid hormone biosynthetic process	3/389	30/23328	0.013383582	0.05258	0.03343	Scarb1/Da	3
GO:0072006	GO:0072006	nephron development	7/389	151/23328	0.013559809	0.05318	0.03381	Bmp4/Pdg	7

GO:0001765	GO:0001765	membrane raft assembly	2/389	11/23328	0.013809402	0.05318	0.03381	Anxa2/Ca	2
GO:0001867	GO:0001867	complement activation, lectin pathway	2/389	11/23328	0.013809402	0.05318	0.03381	Serping1/	2
GO:0002138	GO:0002138	retinoic acid biosynthetic process	2/389	11/23328	0.013809402	0.05318	0.03381	Rbp1/Cyp	2
GO:0002921	GO:0002921	negative regulation of humoral immune response	2/389	11/23328	0.013809402	0.05318	0.03381	Serping1/	2
GO:0016102	GO:0016102	diterpenoid biosynthetic process	2/389	11/23328	0.013809402	0.05318	0.03381	Rbp1/Cyp	2
GO:0031642	GO:0031642	negative regulation of myelination	2/389	11/23328	0.013809402	0.05318	0.03381	Jam2/Klk8	2
GO:0043568	GO:0043568	positive regulation of insulin-like growth factor receptor	2/389	11/23328	0.013809402	0.05318	0.03381	Igfbp5/Igf	2
GO:0043619	GO:0043619	regulation of transcription from RNA polymerase II pro	2/389	11/23328	0.013809402	0.05318	0.03381	Epas1/Cd	2
GO:0045647	GO:0045647	negative regulation of erythrocyte differentiation	2/389	11/23328	0.013809402	0.05318	0.03381	Zfp36/Lm	2
GO:0048845	GO:0048845	venous blood vessel morphogenesis	2/389	11/23328	0.013809402	0.05318	0.03381	Prox1/Eng	2
GO:0060020	GO:0060020	Bergmann glial cell differentiation	2/389	11/23328	0.013809402	0.05318	0.03381	Vim/Plpp3	2
GO:0060056	GO:0060056	mammary gland involution	2/389	11/23328	0.013809402	0.05318	0.03381	Igfbp5/Ca	2
GO:0060346	GO:0060346	bone trabecula formation	2/389	11/23328	0.013809402	0.05318	0.03381	Mmp2/Sfr	2
GO:0072102	GO:0072102	glomerulus morphogenesis	2/389	11/23328	0.013809402	0.05318	0.03381	Bmp4/Not	2
GO:0097278	GO:0097278	complement-dependent cytotoxicity	2/389	11/23328	0.013809402	0.05318	0.03381	Cd55/Cd5	2
GO:1903365	GO:1903365	regulation of fear response	2/389	11/23328	0.013809402	0.05318	0.03381	Apoe/Gja1	2
GO:2000121	GO:2000121	regulation of removal of superoxide radicals	2/389	11/23328	0.013809402	0.05318	0.03381	Cd36/Fbln	2
GO:2000644	GO:2000644	regulation of receptor catabolic process	2/389	11/23328	0.013809402	0.05318	0.03381	Anxa2/Ap	2
GO:2000822	GO:2000822	regulation of behavioral fear response	2/389	11/23328	0.013809402	0.05318	0.03381	Apoe/Gja1	2
GO:2001214	GO:2001214	positive regulation of vasculogenesis	2/389	11/23328	0.013809402	0.05318	0.03381	Cd34/Adm	2
GO:0034612	GO:0034612	response to tumor necrosis factor	8/389	188/23328	0.013882679	0.05334	0.03392	Ccl21a/Fa	8
GO:0030203	GO:0030203	glycosaminoglycan metabolic process	5/389	85/23328	0.013891113	0.05334	0.03392	Lyve1/Pdg	5
GO:0043367	GO:0043367	CD4-positive, alpha-beta T cell differentiation	5/389	85/23328	0.013891113	0.05334	0.03392	Rsad2/Ga	5
GO:0043406	GO:0043406	positive regulation of MAP kinase activity	9/389	226/23328	0.013963754	0.05357	0.03406	Flt1/Bmp4	9
GO:0050873	GO:0050873	brown fat cell differentiation	4/389	56/23328	0.014149101	0.05413	0.03442	Fabp4/Ad	4
GO:0051930	GO:0051930	regulation of sensory perception of pain	4/389	56/23328	0.014149101	0.05413	0.03442	Adrb2/Fab	4
GO:0070265	GO:0070265	necrotic cell death	4/389	56/23328	0.014149101	0.05413	0.03442	Cav1/Slc2	4
GO:0002237	GO:0002237	response to molecule of bacterial origin	13/389	389/23328	0.01420413	0.05428	0.03452	Lbp/Cd36	13
GO:0001764	GO:0001764	neuron migration	8/389	189/23328	0.014293145	0.05452	0.03467	Reln/Nr2f	8
GO:0061136	GO:0061136	regulation of proteasomal protein catabolic process	8/389	189/23328	0.014293145	0.05452	0.03467	Ecscr/Cav	8
GO:0051222	GO:0051222	positive regulation of protein transport	12/389	348/23328	0.014479694	0.05518	0.03509	Rgcc/Cd3	12
GO:0003281	GO:0003281	ventricular septum development	5/389	86/23328	0.014552874	0.05527	0.03514	Gja5/Bmp	5
GO:0097581	GO:0097581	lamellipodium organization	5/389	86/23328	0.014552874	0.05527	0.03514	Ablim3/Ac	5
GO:1901379	GO:1901379	regulation of potassium ion transmembrane transport	5/389	86/23328	0.014552874	0.05527	0.03514	Prnp/Rgs	5
GO:0002828	GO:0002828	regulation of type 2 immune response	3/389	31/23328	0.014637778	0.05527	0.03514	Rsad2/Il3	3
GO:0033687	GO:0033687	osteoblast proliferation	3/389	31/23328	0.014637778	0.05527	0.03514	Junb/Sfrp	3
GO:0048535	GO:0048535	lymph node development	3/389	31/23328	0.014637778	0.05527	0.03514	Ccl21a/Pc	3
GO:0051497	GO:0051497	negative regulation of stress fiber assembly	3/389	31/23328	0.014637778	0.05527	0.03514	F11r/S1pr	3
GO:0060795	GO:0060795	cell fate commitment involved in formation of primary g	3/389	31/23328	0.014637778	0.05527	0.03514	Sox17/Bm	3
GO:0095500	GO:0095500	acetylcholine receptor signaling pathway	3/389	31/23328	0.014637778	0.05527	0.03514	Ly6c1/Ly6	3
GO:1904031	GO:1904031	positive regulation of cyclin-dependent protein kinase a	3/389	31/23328	0.014637778	0.05527	0.03514	Rgcc/Pdg	3
GO:0042476	GO:0042476	odontogenesis	6/389	119/23328	0.014860748	0.05606	0.03565	Id3/Cd34/	6
GO:0002821	GO:0002821	positive regulation of adaptive immune response	7/389	154/23328	0.014966883	0.05635	0.03583	H2-Q6/Rs	7
GO:0014013	GO:0014013	regulation of gliogenesis	7/389	154/23328	0.014966883	0.05635	0.03583	Ptn/Serpir	7
GO:0043030	GO:0043030	regulation of macrophage activation	4/389	57/23328	0.015021932	0.05635	0.03583	Lbp/Cd20	4
GO:0043536	GO:0043536	positive regulation of blood vessel endothelial cell mig	4/389	57/23328	0.015021932	0.05635	0.03583	Hspb1/Pd	4
GO:0051591	GO:0051591	response to cAMP	4/389	57/23328	0.015021932	0.05635	0.03583	Igfbp5/Aq	4
GO:0051931	GO:0051931	regulation of sensory perception	4/389	57/23328	0.015021932	0.05635	0.03583	Adrb2/Fab	4
GO:0016032	GO:0016032	viral process	11/389	309/23328	0.015185419	0.05691	0.03619	Rsad2/Oa	11
GO:0030900	GO:0030900	forebrain development	13/389	393/23328	0.015333454	0.05742	0.03651	Reln/Nr2f	13
GO:0006457	GO:0006457	protein folding	7/389	155/23328	0.015458188	0.05783	0.03677	B2m/Hspb	7
GO:0043523	GO:0043523	regulation of neuron apoptotic process	10/389	270/23328	0.015697068	0.05867	0.03731	Ptprf/Prnp	10
GO:0090596	GO:0090596	sensory organ morphogenesis	11/389	311/23328	0.015855592	0.05874	0.03735	Thy1/Ptn/	11
GO:0001825	GO:0001825	blastocyst formation	4/389	58/23328	0.01592801	0.05874	0.03735	Sox17/Ptp	4
GO:0010656	GO:0010656	negative regulation of muscle cell apoptotic process	4/389	58/23328	0.01592801	0.05874	0.03735	Sfrp2/Cav	4
GO:0010662	GO:0010662	regulation of striated muscle cell apoptotic process	4/389	58/23328	0.01592801	0.05874	0.03735	Fndc1/Fbx	4
GO:0032663	GO:0032663	regulation of interleukin-2 production	4/389	58/23328	0.01592801	0.05874	0.03735	Cd34/Prnp	4
GO:0035456	GO:0035456	response to interferon-beta	4/389	58/23328	0.01592801	0.05874	0.03735	Iigp1/Mnd	4
GO:0045071	GO:0045071	negative regulation of viral genome replication	4/389	58/23328	0.01592801	0.05874	0.03735	Rsad2/Oa	4
GO:0032526	GO:0032526	response to retinoic acid	5/389	88/23328	0.015939099	0.05874	0.03735	Tbx1/Aqp	5
GO:0010614	GO:0010614	negative regulation of cardiac muscle hypertrophy	3/389	32/23328	0.015956258	0.05874	0.03735	Fbxo32/R	3
GO:0043901	GO:0043901	negative regulation of multi-organism process	3/389	32/23328	0.015956258	0.05874	0.03735	Gja1/Bst2	3
GO:0045577	GO:0045577	regulation of B cell differentiation	3/389	32/23328	0.015956258	0.05874	0.03735	Il2rg/Id2/S	3
GO:0055022	GO:0055022	negative regulation of cardiac muscle tissue growth	3/389	32/23328	0.015956258	0.05874	0.03735	Rgs4/Gja1	3
GO:0060306	GO:0060306	regulation of membrane repolarization	3/389	32/23328	0.015956258	0.05874	0.03735	Gja5/Cav1	3
GO:0061117	GO:0061117	negative regulation of heart growth	3/389	32/23328	0.015956258	0.05874	0.03735	Rgs4/Gja1	3
GO:0099560	GO:0099560	synaptic membrane adhesion	3/389	32/23328	0.015956258	0.05874	0.03735	Ptprf/Spar	3
GO:2000352	GO:2000352	negative regulation of endothelial cell apoptotic proces	3/389	32/23328	0.015956258	0.05874	0.03735	Cdh5/Gas	3
GO:0032675	GO:0032675	regulation of interleukin-6 production	7/389	156/23328	0.015960852	0.05874	0.03735	Lbp/Cd36	7
GO:0042542	GO:0042542	response to hydrogen peroxide	6/389	121/23328	0.016015135	0.05878	0.03738	Lcn2/Aqp	6
GO:0046620	GO:0046620	regulation of organ growth	6/389	121/23328	0.016015135	0.05878	0.03738	Rgs4/Slc2	6
GO:1901136	GO:1901136	carbohydrate derivative catabolic process	6/389	121/23328	0.016015135	0.05878	0.03738	Lyve1/Cts	6
GO:0051056	GO:0051056	regulation of small GTPase mediated signal transducti	10/389	271/23328	0.016065947	0.05891	0.03746	Reln/F11r	10
GO:0045927	GO:0045927	positive regulation of growth	11/389	312/23328	0.016198889	0.05909	0.03757	Flt4/Adrb2	11
GO:0003157	GO:0003157	endocardium development	2/389	12/23328	0.016390268	0.05909	0.03757	Sox17/Pro	2
GO:0006957	GO:0006957	complement activation, alternative pathway	2/389	12/23328	0.016390268	0.05909	0.03757	Cfd/Cfb	2
GO:0016264	GO:0016264	gap junction assembly	2/389	12/23328	0.016390268	0.05909	0.03757	Gja5/Cav1	2
GO:0030949	GO:0030949	positive regulation of vascular endothelial growth facto	2/389	12/23328	0.016390268	0.05909	0.03757	Bmp4/Tek	2
GO:0031392	GO:0031392	regulation of prostaglandin biosynthetic process	2/389	12/23328	0.016390268	0.05909	0.03757	Fabp5/An	2
GO:0032490	GO:0032490	detection of molecule of bacterial origin	2/389	12/23328	0.016390268	0.05909	0.03757	Lbp/Scarb	2
GO:0033275	GO:0033275	actin-myosin filament sliding	2/389	12/23328	0.016390268	0.05909	0.03757	Tpm1/Tnn	2
GO:0035376	GO:0035376	sterol import	2/389	12/23328	0.016390268	0.05909	0.03757	Cd36/Sca	2
GO:0042447	GO:0042447	hormone catabolic process	2/389	12/23328	0.016390268	0.05909	0.03757	Spp1/Ece	2
GO:0042989	GO:0042989	sequestering of actin monomers	2/389	12/23328	0.016390268	0.05909	0.03757	Tmsb10/T	2
GO:0043587	GO:0043587	tongue morphogenesis	2/389	12/23328	0.016390268	0.05909	0.03757	Tbx1/Cyp	2
GO:0044766	GO:0044766	multi-organism transport	2/389	12/23328	0.016390268	0.05909	0.03757	Cav1/Bst2	2
GO:0046794	GO:0046794	transport of virus	2/389	12/23328	0.016390268	0.05909	0.03757	Cav1/Bst2	2
GO:0048241	GO:0048241	epinephrine transport	2/389	12/23328	0.016390268	0.05909	0.03757	Ptgs1/Ly6	2
GO:0060213	GO:0060213	positive regulation of nuclear-transcribed mRNA poly(A	2/389	12/23328	0.016390268	0.05909	0.03757	Btg2/Zfp3	2
GO:0070278	GO:0070278	extracellular matrix constituent secretion	2/389	12/23328	0.016390268	0.05909	0.03757	Rgcc/Eng	2
GO:0070508	GO:0070508	cholesterol import	2/389	12/23328	0.016390268	0.05909	0.03757	Cd36/Sca	2
GO:1902579	GO:1902579	multi-organism localization	2/389	12/23328	0.016390268	0.05909	0.03757	Cav1/Bst2	2
GO:0031098	GO:0031098	stress-activated protein kinase signaling cascade	10/389	272/23328	0.016441225	0.05922	0.03766	Flt4/Prdx1	10
GO:0060402	GO:0060402	calcium ion transport into cytosol	7/389	157/23328	0.016475004	0.05929	0.0377	Thy1/Prnp	7
GO:0050868	GO:0050868	negative regulation of T cell activation	6/389	122/23328	0.016614944	0.05974	0.03799	Dtx1/Fgl2	6
GO:0051702	GO:0051702	interaction with symbiont	5/389	89/23328	0.016664075	0.05987	0.03807	Anxa2/Ap	5
GO:0010659	GO:0010659	cardiac muscle cell apoptotic process	4/389	59/23328	0.016867703	0.06055	0.0385	Fndc1/Fbx	4
GO:0050777	GO:0050777	negative regulation of immune response	7/389	158/23328	0.017000772	0.06097	0.03877	Fgl2/Serp	7
GO:0060828	GO:0060828	regulation of canonical Wnt signaling pathway	9/389	234/23328	0.017135008	0.0614	0.03904	Sox17/Tm	9
GO:0051261	GO:0051261	protein depolymerization	6/389	123/23328	0.017230064	0.06168	0.03922	Sh3gl3/Ds	6
GO:0001779	GO:0001779	natural killer cell differentiation	3/389	33/23328	0.017339333	0.06175	0.03926	Gas6/Id2/	3
GO:0031112	GO:0031112	positive regulation of microtubule polymerization or de	3/389	33/23328	0.017339333	0.06175	0.03926	Cav1/Stm	3
GO:0050654	GO:0050654	chondroitin sulfate proteoglycan metabolic process	3/389	33/23328	0.017339333	0.06175	0.03926	Cytl1/Bgn	3
GO:0070266	GO:0070266	necroptotic process	3/389	33/23328	0.017339333	0.06175	0.03926	Cav1/Slc2	3
GO:1905144	GO:1905144	response to acetylcholine	3/389	33/23328	0.017339333	0.06175	0.03926	Ly6c1/Ly6	3
GO:1905145	GO:1905145	cellular response to acetylcholine	3/389	33/23328	0.017339333	0.06175	0.03926	Ly6c1/Ly6	3
GO:0070509	GO:0070509	calcium ion import	5/389	90/23328	0.017410624	0.06195	0.03939	Prnp/Adrb	5
GO:0007569	GO:0007569	cell aging	7/389	159/23328	0.017538285	0.06229	0.03961	Prelp/B2m	7
GO:0071383	GO:0071383	cellular response to steroid hormone stimulus	7/389	159/23328	0.017538285	0.06229	0.03961	Bmp4/Fbx	7
GO:0060560	GO:0060560	developmental growth involved in morphogenesis	10/389	275/23328	0.017606082	0.06248	0.03973	Tnc/Cxcl1	10
GO:0009314	GO:0009314	response to radiation	13/389	401/23328	0.017796512	0.06299	0.04005	Nts/Sfrp2/	13
GO:0060711	GO:0060711	labyrinthine layer development	4/389	60/23328	0.017841355	0.06299	0.04005	Socs3/Vc	4
GO:0070098	GO:0070098	chemokine-mediated signaling pathway	4/389	60/23328	0.017841355	0.06299	0.04005	Ccl21a/Cx	4
GO:0098900	GO:0098900	regulation of action potential	4/389	60/23328	0.017841355	0.06299	0.04005	Cd36/Gja	4
GO:1901616	GO:1901616	organic hydroxy compound catabolic process	4/389	60/23328	0.017841355	0.06299	0.04005	Apoe/Sca	4
GO:2000401	GO:2000401	regulation of lymphocyte migration	4/389	60/23328	0.017841355	0.06299	0.04005	Ccl21a/Cc	4
GO:0032411	GO:0032411	positive regulation of transporter activity	6/389	124/23328	0.017860665	0.063	0.04006	Reln/Adrb	6
GO:0014015	GO:0014015	positive regulation of gliogenesis	5/389	91/23328	0.018178986	0.0639	0.04063	Ptn/Serpir	5
GO:0031110	GO:0031110	regulation of microtubule polymerization or depolymeri	5/389	91/23328	0.018178986	0.0639	0.04063	Cav1/Cdh	5
GO:0072091	GO:0072091	regulation of stem cell proliferation	5/389	91/23328	0.018178986	0.0639	0.04063	Ptn/Sox17	5
GO:2000117	GO:2000117	negative regulation of cysteine-type endopeptidase act	5/389	91/23328	0.018178986	0.0639	0.04063	Sfrp2/Nr4	5
GO:0046486	GO:0046486	glycerolipid metabolic process	12/389	360/23328	0.018381328	0.06454	0.04104	Cd36/Soc	12
GO:0030307	GO:0030307	positive regulation of cell growth	8/389	198/23328	0.018393847	0.06454	0.04104	Flt4/Cxcl1	8
GO:0002021	GO:0002021	response to dietary excess	3/389	34/23328	0.018787242	0.06526	0.0415	Adrb2/Ap	3
GO:0031338	GO:0031338	regulation of vesicle fusion	3/389	34/23328	0.018787242	0.06526	0.0415	Anxa2/An	3
GO:0043537	GO:0043537	negative regulation of blood vessel endothelial cell mi	3/389	34/23328	0.018787242	0.06526	0.0415	Rgcc/Apo	3
GO:0090218	GO:0090218	positive regulation of lipid kinase activity	3/389	34/23328	0.018787242	0.06526	0.0415	Flt1/Pdgf	3
GO:0098868	GO:0098868	bone growth	3/389	34/23328	0.018787242	0.06526	0.0415	Stc1/Fbln	3
GO:0007584	GO:0007584	response to nutrient	4/389	61/23328	0.018849294	0.06526	0.0415	Spp1/Gas	4

GO:0031641	GO:0031641	regulation of myelination	4/389	61/23328	0.018849294	0.06526	0.0415	Ptn/Jam2/	4
GO:0032732	GO:0032732	positive regulation of interleukin-1 production	4/389	61/23328	0.018849294	0.06526	0.0415	Hspb1/Mn	4
GO:0045776	GO:0045776	negative regulation of blood pressure	4/389	61/23328	0.018849294	0.06526	0.0415	Cd36/Gja5	4
GO:0051851	GO:0051851	modulation by host of symbiont process	4/389	61/23328	0.018849294	0.06526	0.0415	Anxa2/Ap	4
GO:0050714	GO:0050714	positive regulation of protein secretion	8/389	199/2332	0.018896441	0.06526	0.0415	Rgcc/Cd3	8
GO:0003091	GO:0003091	renal water homeostasis	2/389	13/23328	0.019159028	0.06526	0.0415	Aqp1/Aqp	2
GO:0010739	GO:0010739	positive regulation of protein kinase A signaling	2/389	13/23328	0.019159028	0.06526	0.0415	Adrb2/Aka	2
GO:0010744	GO:0010744	positive regulation of macrophage derived foam cell di	2/389	13/23328	0.019159028	0.06526	0.0415	Cd36/Lpl	2
GO:0021683	GO:0021683	cerebellar granular layer morphogenesis	2/389	13/23328	0.019159028	0.06526	0.0415	Serpine2/	2
GO:0030948	GO:0030948	negative regulation of vascular endothelial growth fact	2/389	13/23328	0.019159028	0.06526	0.0415	Mmrn2/Cc	2
GO:0032799	GO:0032799	low-density lipoprotein receptor particle metabolic proc	2/389	13/23328	0.019159028	0.06526	0.0415	Anxa2/Ap	2
GO:0034350	GO:0034350	regulation of glial cell apoptotic process	2/389	13/23328	0.019159028	0.06526	0.0415	Gas6/Aka	2
GO:0035627	GO:0035627	ceramide transport	2/389	13/23328	0.019159028	0.06526	0.0415	Pltp/Abcb	2
GO:0043691	GO:0043691	reverse cholesterol transport	2/389	13/23328	0.019159028	0.06526	0.0415	Apoe/Sca	2
GO:0045906	GO:0045906	negative regulation of vasoconstriction	2/389	13/23328	0.019159028	0.06526	0.0415	Mmp2/Adı	2
GO:0048548	GO:0048548	regulation of pinocytosis	2/389	13/23328	0.019159028	0.06526	0.0415	Cav1/Actr	2
GO:0048711	GO:0048711	positive regulation of astrocyte differentiation	2/389	13/23328	0.019159028	0.06526	0.0415	Serpine2/	2
GO:0060670	GO:0060670	branching involved in labyrinthine layer morphogenesis	2/389	13/23328	0.019159028	0.06526	0.0415	Socs3/Adı	2
GO:0061052	GO:0061052	negative regulation of cell growth involved in cardiac m	2/389	13/23328	0.019159028	0.06526	0.0415	Rgs4/Pi16	2
GO:0071073	GO:0071073	positive regulation of phospholipid biosynthetic proces	2/389	13/23328	0.019159028	0.06526	0.0415	Adgrf5/Htı	2
GO:0071281	GO:0071281	cellular response to iron ion	2/389	13/23328	0.019159028	0.06526	0.0415	B2m/Trf	2
GO:0071285	GO:0071285	cellular response to lithium ion	2/389	13/23328	0.019159028	0.06526	0.0415	Fabp4/Id2	2
GO:0071294	GO:0071294	cellular response to zinc ion	2/389	13/23328	0.019159028	0.06526	0.0415	Mt1/Mt2	2
GO:0072216	GO:0072216	positive regulation of metanephros development	2/389	13/23328	0.019159028	0.06526	0.0415	Pdgfb/Egr	2
GO:0090193	GO:0090193	positive regulation of glomerulus development	2/389	13/23328	0.019159028	0.06526	0.0415	Pdgfb/Egr	2
GO:0097011	GO:0097011	cellular response to granulocyte macrophage colony-st	2/389	13/23328	0.019159028	0.06526	0.0415	Zfp36/Pde	2
GO:1901163	GO:1901163	regulation of trophoblast cell migration	2/389	13/23328	0.019159028	0.06526	0.0415	Gja1/Arhg	2
GO:1903817	GO:1903817	negative regulation of voltage-gated potassium channe	2/389	13/23328	0.019159028	0.06526	0.0415	Ank3/Cav	2
GO:1904181	GO:1904181	positive regulation of membrane depolarization	2/389	13/23328	0.019159028	0.06526	0.0415	Ank3/Dcn	2
GO:0009798	GO:0009798	axis specification	5/389	93/23328	0.019782073	0.06714	0.04269	Bmp4/Pcs	5
GO:0045445	GO:0045445	myoblast differentiation	5/389	93/23328	0.019782073	0.06714	0.04269	Id3/Epas1	5
GO:1903169	GO:1903169	regulation of calcium ion transmembrane transport	7/389	163/2332	0.019808259	0.06714	0.04269	Thy1/Prnp	7
GO:0006813	GO:0006813	potassium ion transport	9/389	240/2332	0.019837266	0.06714	0.04269	Gja5/Prnp	9
GO:1904951	GO:1904951	positive regulation of establishment of protein localizat	12/389	364/2332	0.019842651	0.06714	0.04269	Rgcc/Cd3	12
GO:0014902	GO:0014902	myotube differentiation	6/389	127/2332	0.019847028	0.06714	0.04269	Cd9/Tbx1/	6
GO:0010658	GO:0010658	striated muscle cell apoptotic process	4/389	62/23328	0.019891827	0.06714	0.04269	Fndc1/Fb	4
GO:0014911	GO:0014911	positive regulation of smooth muscle cell migration	4/389	62/23328	0.019891827	0.06714	0.04269	Igfbp5/Pd	4
GO:0031640	GO:0031640	killing of cells of other organism	4/389	62/23328	0.019891827	0.06714	0.04269	Nts/Cxcl1	4
GO:0051148	GO:0051148	negative regulation of muscle cell differentiation	4/389	62/23328	0.019891827	0.06714	0.04269	Rgs4/Pdg	4
GO:0060393	GO:0060393	regulation of pathway-restricted SMAD protein phosph	4/389	62/23328	0.019891827	0.06714	0.04269	Bmp4/Enc	4
GO:0050792	GO:0050792	regulation of viral process	8/389	201/2332	0.019930949	0.06722	0.04274	Rsad2/Oa	8
GO:0050867	GO:0050867	positive regulation of cell activation	15/389	496/2332	0.020132932	0.06784	0.04314	Lbp/Thy1/	15
GO:0003016	GO:0003016	respiratory system process	3/389	35/23328	0.020300154	0.06801	0.04325	Flt4/Tcf15	3
GO:0016339	GO:0016339	calcium-dependent cell-cell adhesion via plasma mem	3/389	35/23328	0.020300154	0.06801	0.04325	Cdh5/Cdh	3
GO:0035025	GO:0035025	positive regulation of Rho protein signal transduction	3/389	35/23328	0.020300154	0.06801	0.04325	F11r/Mcf2	3
GO:0050869	GO:0050869	negative regulation of B cell activation	3/389	35/23328	0.020300154	0.06801	0.04325	Samsn1/Id	3
GO:0060142	GO:0060142	regulation of syncytium formation by plasma membran	3/389	35/23328	0.020300154	0.06801	0.04325	Cxcl12/Cx	3
GO:0060292	GO:0060292	long-term synaptic depression	3/389	35/23328	0.020300154	0.06801	0.04325	Mgll/Plk2/	3
GO:0071320	GO:0071320	cellular response to cAMP	3/389	35/23328	0.020300154	0.06801	0.04325	Igfbp5/Aq	3
GO:0045017	GO:0045017	glycerolipid biosynthetic process	7/389	164/2332	0.020406343	0.06831	0.04344	Fabp5/Thı	7
GO:0032609	GO:0032609	interferon-gamma production	6/389	128/2332	0.020541209	0.06859	0.04362	Prnp/Cd2/	6
GO:0043244	GO:0043244	regulation of protein-containing complex disassembly	6/389	128/2332	0.020541209	0.06859	0.04362	Adrb2/Dst	6
GO:0051592	GO:0051592	response to calcium ion	6/389	128/2332	0.020541209	0.06859	0.04362	Lgmn/Cav	6
GO:1901879	GO:1901879	regulation of protein depolymerization	5/389	94/23328	0.020617243	0.06879	0.04374	Dstn/Stmr	5
GO:0032760	GO:0032760	positive regulation of tumor necrosis factor production	4/389	63/23328	0.020969241	0.06985	0.04442	Lbp/Cd36/	4
GO:0071260	GO:0071260	cellular response to mechanical stimulus	4/389	63/23328	0.020969241	0.06985	0.04442	Cav1/F11	4
GO:2001236	GO:2001236	regulation of extrinsic apoptotic signaling pathway	7/389	165/2332	0.021016901	0.06995	0.04448	Bmp4/Tim	7
GO:0006690	GO:0006690	icosanoid metabolic process	6/389	129/2332	0.021251683	0.07068	0.04494	Fabp5/Mg	6
GO:0034764	GO:0034764	positive regulation of transmembrane transport	9/389	243/2332	0.021298259	0.07077	0.045	Reln/Thy1	9
GO:0060070	GO:0060070	canonical Wnt signaling pathway	10/389	284/2332	0.021466263	0.0709	0.04508	Sox17/Tm	10
GO:0051341	GO:0051341	regulation of oxidoreductase activity	5/389	95/23328	0.021475115	0.0709	0.04508	Cav1/Apo	5
GO:0055013	GO:0055013	cardiac muscle cell development	5/389	95/23328	0.021475115	0.0709	0.04508	Bmp4/Rgs	5
GO:0006775	GO:0006775	fat-soluble vitamin metabolic process	3/389	36/23328	0.021878166	0.0709	0.04508	Pltp/Rbp1	3
GO:0010453	GO:0010453	regulation of cell fate commitment	3/389	36/23328	0.021878166	0.0709	0.04508	Sox17/Bm	3
GO:0032232	GO:0032232	negative regulation of actin filament bundle assembly	3/389	36/23328	0.021878166	0.0709	0.04508	F11r/S1pr	3
GO:0035909	GO:0035909	aorta morphogenesis	3/389	36/23328	0.021878166	0.0709	0.04508	Tbx1/Prox	3
GO:0048009	GO:0048009	insulin-like growth factor receptor signaling pathway	3/389	36/23328	0.021878166	0.0709	0.04508	Igfbp5/Igf	3
GO:0048679	GO:0048679	regulation of axon regeneration	3/389	36/23328	0.021878166	0.0709	0.04508	Ptn/Ptprf/	3
GO:0061384	GO:0061384	heart trabecula morphogenesis	3/389	36/23328	0.021878166	0.0709	0.04508	Eng/Tek/S	3
GO:0071158	GO:0071158	positive regulation of cell cycle arrest	3/389	36/23328	0.021878166	0.0709	0.04508	Rgcc/Id2/	3
GO:0097242	GO:0097242	amyloid-beta clearance	3/389	36/23328	0.021878166	0.0709	0.04508	Cd36/Apo	3
GO:2000171	GO:2000171	negative regulation of dendrite development	3/389	36/23328	0.021878166	0.0709	0.04508	Id1/Apoe/	3
GO:0032368	GO:0032368	regulation of lipid transport	6/389	130/2332	0.021978603	0.0709	0.04508	Pltp/Spp1/	6
GO:0008347	GO:0008347	glial cell migration	4/389	64/23328	0.022081805	0.0709	0.04508	Reln/Cd9/	4
GO:0030858	GO:0030858	positive regulation of epithelial cell differentiation	4/389	64/23328	0.022081805	0.0709	0.04508	Cldn5/Bm	4
GO:1902305	GO:1902305	regulation of sodium ion transmembrane transport	4/389	64/23328	0.022081805	0.0709	0.04508	Fxyd6/Ank	4
GO:1903557	GO:1903557	positive regulation of tumor necrosis factor superfamily	4/389	64/23328	0.022081805	0.0709	0.04508	Lbp/Cd36/	4
GO:0010226	GO:0010226	response to lithium ion	2/389	14/23328	0.022108729	0.0709	0.04508	Fabp4/Id2	2
GO:0018158	GO:0018158	protein oxidation	2/389	14/23328	0.022108729	0.0709	0.04508	Loxl2/Lox	2
GO:0032769	GO:0032769	negative regulation of monooxygenase activity	2/389	14/23328	0.022108729	0.0709	0.04508	Cav1/Eng	2
GO:0035358	GO:0035358	regulation of peroxisome proliferator activated recepto	2/389	14/23328	0.022108729	0.0709	0.04508	Fabp5/Ptg	2
GO:0035791	GO:0035791	platelet-derived growth factor receptor-beta signaling p	2/389	14/23328	0.022108729	0.0709	0.04508	Pdgfb/Lox	2
GO:0036296	GO:0036296	response to increased oxygen levels	2/389	14/23328	0.022108729	0.0709	0.04508	Cav1/Pdp	2
GO:0042532	GO:0042532	negative regulation of tyrosine phosphorylation of STA	2/389	14/23328	0.022108729	0.0709	0.04508	Socs3/Ca	2
GO:0043508	GO:0043508	negative regulation of JUN kinase activity	2/389	14/23328	0.022108729	0.0709	0.04508	Sfrp2/Sfrp	2
GO:0044764	GO:0044764	multi-organism cellular process	2/389	14/23328	0.022108729	0.0709	0.04508	Cav1/Gas	2
GO:0046886	GO:0046886	positive regulation of hormone biosynthetic process	2/389	14/23328	0.022108729	0.0709	0.04508	Dab2/Egr	2
GO:0051152	GO:0051152	positive regulation of smooth muscle cell differentiatio	2/389	14/23328	0.022108729	0.0709	0.04508	Eng/Tmsb	2
GO:0051451	GO:0051451	myoblast migration	2/389	14/23328	0.022108729	0.0709	0.04508	Net1/Anxa	2
GO:0060211	GO:0060211	regulation of nuclear-transcribed mRNA poly(A) tail sh	2/389	14/23328	0.022108729	0.0709	0.04508	Btg2/Zfp3	2
GO:0061450	GO:0061450	trophoblast cell migration	2/389	14/23328	0.022108729	0.0709	0.04508	Gja1/Arhg	2
GO:0061469	GO:0061469	regulation of type B pancreatic cell proliferation	2/389	14/23328	0.022108729	0.0709	0.04508	Nr4a1/Sfr	2
GO:0071850	GO:0071850	mitotic cell cycle arrest	2/389	14/23328	0.022108729	0.0709	0.04508	Rgcc/Cdk	2
GO:0090179	GO:0090179	planar cell polarity pathway involved in neural tube clo	2/389	14/23328	0.022108729	0.0709	0.04508	Sfrp2/Sfrp	2
GO:0097012	GO:0097012	response to granulocyte macrophage colony-stimulatir	2/389	14/23328	0.022108729	0.0709	0.04508	Zfp36/Pde	2
GO:0097066	GO:0097066	response to thyroid hormone	2/389	14/23328	0.022108729	0.0709	0.04508	Cfb/Lmo2	2
GO:1902950	GO:1902950	regulation of dendritic spine maintenance	2/389	14/23328	0.022108729	0.0709	0.04508	Prnp/Apoe	2
GO:1903236	GO:1903236	regulation of leukocyte tethering or rolling	2/389	14/23328	0.022108729	0.0709	0.04508	Cxcl12/Se	2
GO:1904948	GO:1904948	midbrain dopaminergic neuron differentiation	2/389	14/23328	0.022108729	0.0709	0.04508	Sfrp2/Sfrp	2
GO:1905063	GO:1905063	regulation of vascular associated smooth muscle cell c	2/389	14/23328	0.022108729	0.0709	0.04508	Pdgfb/Eng	2
GO:2000035	GO:2000035	regulation of stem cell division	2/389	14/23328	0.022108729	0.0709	0.04508	Sox17/Sfr	2
GO:2000052	GO:2000052	positive regulation of non-canonical Wnt signaling path	2/389	14/23328	0.022108729	0.0709	0.04508	Dab2/Sfrp	2
GO:2001279	GO:2001279	regulation of unsaturated fatty acid biosynthetic proces	2/389	14/23328	0.022108729	0.0709	0.04508	Fabp5/An	2
GO:0048469	GO:0048469	cell maturation	8/389	205/2332	0.022119834	0.0709	0.04508	Epas1/Cd	8
GO:0032635	GO:0032635	interleukin-6 production	7/389	167/2332	0.022275904	0.07135	0.04537	Lbp/Cd36/	7
GO:0061053	GO:0061053	somite development	5/389	96/23328	0.022355893	0.07155	0.04549	Tcf15/Sfrp	5
GO:0002761	GO:0002761	regulation of myeloid leukocyte differentiation	6/389	131/2332	0.022722119	0.0726	0.04617	Rbp1/Id2/	6
GO:0032434	GO:0032434	regulation of proteasomal ubiquitin-dependent protein	6/389	131/2332	0.022722119	0.0726	0.04617	Cav1/Plk2	6
GO:0031647	GO:0031647	regulation of protein stability	10/389	287/2332	0.022880298	0.07305	0.04645	Sox17/Id1	10
GO:0032386	GO:0032386	regulation of intracellular transport	11/389	329/2332	0.022924715	0.07314	0.04651	Cd36/Prn	11
GO:0010976	GO:0010976	positive regulation of neuron projection development	12/389	372/2332	0.023022953	0.07339	0.04667	Reln/Ptn/	12
GO:0060389	GO:0060389	pathway-restricted SMAD protein phosphorylation	4/389	65/23328	0.023229768	0.07399	0.04705	Bmp4/Eng	4
GO:0002456	GO:0002456	T cell mediated immunity	6/389	132/2332	0.023482375	0.07434	0.04727	H2-Q6/Rs	6
GO:0002920	GO:0002920	regulation of humoral immune response	3/389	37/23328	0.023521313	0.07434	0.04727	Serping1/	3
GO:0009595	GO:0009595	detection of biotic stimulus	3/389	37/23328	0.023521313	0.07434	0.04727	Lbp/Scarb	3
GO:0033363	GO:0033363	secretory granule organization	3/389	37/23328	0.023521313	0.07434	0.04727	Srgn/Serp	3
GO:0048846	GO:0048846	axon extension involved in axon guidance	3/389	37/23328	0.023521313	0.07434	0.04727	Cxcl12/Nr	3
GO:0060071	GO:0060071	Wnt signaling pathway, planar cell polarity pathway	3/389	37/23328	0.023521313	0.07434	0.04727	Sfrp2/Dab	3
GO:0071709	GO:0071709	membrane assembly	3/389	37/23328	0.023521313	0.07434	0.04727	Ank3/Anxa	3
GO:1902229	GO:1902229	regulation of intrinsic apoptotic signaling pathway in re	3/389	37/23328	0.023521313	0.07434	0.04727	Cxcl12/Cl	3
GO:1902284	GO:1902284	neuron projection extension involved in neuron project	3/389	37/23328	0.023521313	0.07434	0.04727	Cxcl12/Nr	3
GO:1903427	GO:1903427	negative regulation of reactive oxygen species biosynt	3/389	37/23328	0.023521313	0.07434	0.04727	Cd34/Cav	3
GO:0071356	GO:0071356	cellular response to tumor necrosis factor	7/389	169/2332	0.023586179	0.07449	0.04736	Ccl21a/Fa	7
GO:0015711	GO:0015711	organic anion transport	14/389	463/2332	0.024264723	0.07657	0.04869	Fabp4/Cd	14
GO:0032623	GO:0032623	interleukin-2 production	4/389	66/23328	0.024413359	0.07698	0.04895	Cd34/Prn	4
GO:0043281	GO:0043281	regulation of cysteine-type endopeptidase activity invo	8/389	209/2332	0.024473324	0.07711	0.04903	Sfrp2/Nr4	8
GO:0030258	GO:0030258	lipid modification	9/389	250/2332	0.02500563	0.07823	0.04975	Ephx2/Cd	9
GO:1902806	GO:1902806	regulation of cell cycle G1/S phase transition	6/389	134/2332	0.02505368	0.07823	0.04975	Rgcc/Pdp	6
GO:2000027	GO:2000027	regulation of animal organ morphogenesis	8/389	210/2332	0.025088068	0.07823	0.04975	Ptn/Cd34/	8

GO:0014002	GO:0014002	astrocyte development	3/389	38/23328	0.025229566	0.07823	0.04975	Vim/S100a	3
GO:0019432	GO:0019432	triglyceride biosynthetic process	3/389	38/23328	0.025229566	0.07823	0.04975	Thrsp/Sca	3
GO:0060674	GO:0060674	placenta blood vessel development	3/389	38/23328	0.025229566	0.07823	0.04975	Nr2f2/Soc	3
GO:0062208	GO:0062208	positive regulation of pattern recognition receptor signa	3/389	38/23328	0.025229566	0.07823	0.04975	Lbp/Rsad2	3
GO:2000463	GO:2000463	positive regulation of excitatory postsynaptic potential	3/389	38/23328	0.025229566	0.07823	0.04975	Reln/Adrb	3
GO:0006862	GO:0006862	nucleotide transport	2/389	15/23328	0.025232601	0.07823	0.04975	Slc25a4/G	2
GO:0016114	GO:0016114	terpenoid biosynthetic process	2/389	15/23328	0.025232601	0.07823	0.04975	Rbp1/Cyp	2
GO:0031115	GO:0031115	negative regulation of microtubule polymerization	2/389	15/23328	0.025232601	0.07823	0.04975	Cdh5/Stm	2
GO:0031340	GO:0031340	positive regulation of vesicle fusion	2/389	15/23328	0.025232601	0.07823	0.04975	Anxa2/Anx	2
GO:0043031	GO:0043031	negative regulation of macrophage activation	2/389	15/23328	0.025232601	0.07823	0.04975	Cd200/Ad	2
GO:0043129	GO:0043129	surfactant homeostasis	2/389	15/23328	0.025232601	0.07823	0.04975	Epas1/Ad	2
GO:0051764	GO:0051764	actin crosslink formation	2/389	15/23328	0.025232601	0.07823	0.04975	Dpysl3/Tn	2
GO:0060442	GO:0060442	branching involved in prostate gland morphogenesis	2/389	15/23328	0.025232601	0.07823	0.04975	Bmp4/Sfrp	2
GO:0061430	GO:0061430	bone trabecula morphogenesis	2/389	15/23328	0.025232601	0.07823	0.04975	Mmp2/Sfr	2
GO:0070307	GO:0070307	lens fiber cell development	2/389	15/23328	0.025232601	0.07823	0.04975	Vim/Prox1	2
GO:0090178	GO:0090178	regulation of establishment of planar polarity involved i	2/389	15/23328	0.025232601	0.07823	0.04975	Sfrp2/Sfrp	2
GO:1900121	GO:1900121	negative regulation of receptor binding	2/389	15/23328	0.025232601	0.07823	0.04975	Ptprf/B2m	2
GO:1902043	GO:1902043	positive regulation of extrinsic apoptotic signaling path	2/389	15/23328	0.025232601	0.07823	0.04975	Timp3/Sfr	2
GO:0007411	GO:0007411	axon guidance	9/389	251/23328	0.025570385	0.07911	0.0503	Reln/Gap4	9
GO:0000768	GO:0000768	syncytium formation by plasma membrane fusion	4/389	67/23328	0.025632789	0.07911	0.0503	Cd9/Cxcl1	4
GO:0030239	GO:0030239	myofibril assembly	4/389	67/23328	0.025632789	0.07911	0.0503	Prox1/Csr	4
GO:0060688	GO:0060688	regulation of morphogenesis of a branching structure	4/389	67/23328	0.025632789	0.07911	0.0503	Bmp4/Pgf	4
GO:0140253	GO:0140253	cell-cell fusion	4/389	67/23328	0.025632789	0.07911	0.0503	Cd9/Cxcl1	4
GO:1901570	GO:1901570	fatty acid derivative biosynthetic process	4/389	67/23328	0.025632789	0.07911	0.0503	Fabp5/Ptg	4
GO:0097485	GO:0097485	neuron projection guidance	9/389	252/23328	0.026144126	0.08063	0.05127	Reln/Gap4	9
GO:0055123	GO:0055123	digestive system development	6/389	136/23328	0.026693619	0.08226	0.05231	Sox17/Bm	6
GO:0002763	GO:0002763	positive regulation of myeloid leukocyte differentiation	4/389	68/23328	0.026888252	0.08234	0.05236	Id2/Kitl/Ju	4
GO:0030032	GO:0030032	lamellipodium assembly	4/389	68/23328	0.026888252	0.08234	0.05236	Ablim3/Ac	4
GO:0043949	GO:0043949	regulation of cAMP-mediated signaling	4/389	68/23328	0.026888252	0.08234	0.05236	Aplnr/Pde	4
GO:0046579	GO:0046579	positive regulation of Ras protein signal transduction	4/389	68/23328	0.026888252	0.08234	0.05236	F11r/Mcf2	4
GO:0050432	GO:0050432	catecholamine secretion	4/389	68/23328	0.026888252	0.08234	0.05236	Cxcl12/Sr	4
GO:2000378	GO:2000378	negative regulation of reactive oxygen species metabo	4/389	68/23328	0.026888252	0.08234	0.05236	Cd34/Cav	4
GO:0072659	GO:0072659	protein localization to plasma membrane	10/389	295/23328	0.026979388	0.08234	0.05236	Prnp/Tme	10
GO:0030513	GO:0030513	positive regulation of BMP signaling pathway	3/389	39/23328	0.027002838	0.08234	0.05236	Bmp4/Cdh	3
GO:0050892	GO:0050892	intestinal absorption	3/389	39/23328	0.027002838	0.08234	0.05236	Cd36/F11	3
GO:0072523	GO:0072523	purine-containing compound catabolic process	3/389	39/23328	0.027002838	0.08234	0.05236	Gda/Nudt	3
GO:0090049	GO:0090049	regulation of cell migration involved in sprouting angio	3/389	39/23328	0.027002838	0.08234	0.05236	Plk2/Mmr	3
GO:1900087	GO:1900087	positive regulation of G1/S transition of mitotic cell cyc	3/389	39/23328	0.027002838	0.08234	0.05236	Rgcc/Anx	3
GO:1902108	GO:1902108	regulation of mitochondrial membrane permeability inv	3/389	39/23328	0.027002838	0.08234	0.05236	Ier3/Slc25	3
GO:2000403	GO:2000403	positive regulation of lymphocyte migration	3/389	39/23328	0.027002838	0.08234	0.05236	Ccl21a/Cx	3
GO:0015718	GO:0015718	monocarboxylic acid transport	7/389	174/23328	0.027091896	0.08242	0.05241	Fabp4/Cd	7
GO:0043271	GO:0043271	negative regulation of ion transport	7/389	174/23328	0.027091896	0.08242	0.05241	Serpine2/	7
GO:0033555	GO:0033555	multicellular organismal response to stress	5/389	101/23328	0.027110018	0.08242	0.05241	Reln/Apoe	5
GO:0055006	GO:0055006	cardiac cell development	5/389	101/23328	0.027110018	0.08242	0.05241	Bmp4/Rgs	5
GO:1903362	GO:1903362	regulation of cellular protein catabolic process	9/389	254/23328	0.027318834	0.083	0.05277	Ecscr/Anx	9
GO:0035821	GO:0035821	modulation of process of other organism	6/389	137/23328	0.027539655	0.0836	0.05316	Anxa2/Ap	6
GO:0035107	GO:0035107	appendage morphogenesis	7/389	175/23328	0.027833307	0.08437	0.05365	Hoxd9/Gja	7
GO:0035108	GO:0035108	limb morphogenesis	7/389	175/23328	0.027833307	0.08437	0.05365	Hoxd9/Gja	7
GO:0045666	GO:0045666	positive regulation of neuron differentiation	14/389	472/23328	0.027954227	0.08458	0.05378	Reln/Ptn/F	14
GO:0001704	GO:0001704	formation of primary germ layer	5/389	102/23328	0.028132114	0.08458	0.05378	Sox17/Bm	5
GO:0006949	GO:0006949	syncytium formation	4/389	69/23328	0.028179919	0.08458	0.05378	Cd9/Cxcl1	4
GO:0009880	GO:0009880	embryonic pattern specification	4/389	69/23328	0.028179919	0.08458	0.05378	Pcsk6/Nrp	4
GO:0021536	GO:0021536	diencephalon development	4/389	69/23328	0.028179919	0.08458	0.05378	Bmp4/Nrp	4
GO:1901888	GO:1901888	regulation of cell junction assembly	8/389	215/23328	0.028324179	0.08458	0.05378	Thy1/Cldn	8
GO:0035023	GO:0035023	regulation of Rho protein signal transduction	6/389	138/23328	0.028403236	0.08458	0.05378	F11r/Apoe	6
GO:0002830	GO:0002830	positive regulation of type 2 immune response	2/389	16/23328	0.028524045	0.08458	0.05378	Rsad2/Il33	2
GO:0003161	GO:0003161	cardiac conduction system development	2/389	16/23328	0.028524045	0.08458	0.05378	Gja5/Id2	2
GO:0006825	GO:0006825	copper ion transport	2/389	16/23328	0.028524045	0.08458	0.05378	Cp/Steap4	2
GO:0007512	GO:0007512	adult heart development	2/389	16/23328	0.028524045	0.08458	0.05378	Aplnr/Gja1	2
GO:0014067	GO:0014067	negative regulation of phosphatidylinositol 3-kinase sig	2/389	16/23328	0.028524045	0.08458	0.05378	Serpine2/	2
GO:0015669	GO:0015669	gas transport	2/389	16/23328	0.028524045	0.08458	0.05378	Aqp1/Car4	2
GO:0043217	GO:0043217	myelin maintenance	2/389	16/23328	0.028524045	0.08458	0.05378	Ndrg1/Clu	2
GO:0048385	GO:0048385	regulation of retinoic acid receptor signaling pathway	2/389	16/23328	0.028524045	0.08458	0.05378	Klf2/Cyp2	2
GO:0048715	GO:0048715	negative regulation of oligodendrocyte differentiation	2/389	16/23328	0.028524045	0.08458	0.05378	Bmp4/Id2	2
GO:0060026	GO:0060026	convergent extension	2/389	16/23328	0.028524045	0.08458	0.05378	Sfrp2/Sfrp	2
GO:0060100	GO:0060100	positive regulation of phagocytosis, engulfment	2/389	16/23328	0.028524045	0.08458	0.05378	Lbp/Cd36	2
GO:0060391	GO:0060391	positive regulation of SMAD protein signal transductior	2/389	16/23328	0.028524045	0.08458	0.05378	Bmp4/Dab	2
GO:0060766	GO:0060766	negative regulation of androgen receptor signaling patl	2/389	16/23328	0.028524045	0.08458	0.05378	Dab2/Sfrp	2
GO:0060856	GO:0060856	establishment of blood-brain barrier	2/389	16/23328	0.028524045	0.08458	0.05378	Eng/Abcb	2
GO:0061000	GO:0061000	negative regulation of dendritic spine development	2/389	16/23328	0.028524045	0.08458	0.05378	Apoe/Plk2	2
GO:0072537	GO:0072537	fibroblast activation	2/389	16/23328	0.028524045	0.08458	0.05378	Rgcc/Pdg	2
GO:0090083	GO:0090083	regulation of inclusion body assembly	2/389	16/23328	0.028524045	0.08458	0.05378	Apoe/Clu	2
GO:0090128	GO:0090128	regulation of synapse maturation	2/389	16/23328	0.028524045	0.08458	0.05378	Reln/Arhg	2
GO:0090177	GO:0090177	establishment of planar polarity involved in neural tube	2/389	16/23328	0.028524045	0.08458	0.05378	Sfrp2/Sfrp	2
GO:1903779	GO:1903779	regulation of cardiac conduction	2/389	16/23328	0.028524045	0.08458	0.05378	Gja5/Cav1	2
GO:1905155	GO:1905155	positive regulation of membrane invagination	2/389	16/23328	0.028524045	0.08458	0.05378	Lbp/Cd36	2
GO:2000651	GO:2000651	positive regulation of sodium ion transmembrane trans	2/389	16/23328	0.028524045	0.08458	0.05378	Ank3/Atp1	2
GO:2001212	GO:2001212	regulation of vasculogenesis	2/389	16/23328	0.028524045	0.08458	0.05378	Cd34/Adm	2
GO:0001881	GO:0001881	receptor recycling	3/389	40/23328	0.028840982	0.08502	0.05406	Anxa2/Da	3
GO:0006730	GO:0006730	one-carbon metabolic process	3/389	40/23328	0.028840982	0.08502	0.05406	Car4/Car8	3
GO:0007616	GO:0007616	long-term memory	3/389	40/23328	0.028840982	0.08502	0.05406	Reln/Apoe	3
GO:0042092	GO:0042092	type 2 immune response	3/389	40/23328	0.028840982	0.08502	0.05406	Rsad2/Il33	3
GO:0043551	GO:0043551	regulation of phosphatidylinositol 3-kinase activity	3/389	40/23328	0.028840982	0.08502	0.05406	Flt1/Pdgfb	3
GO:0071542	GO:0071542	dopaminergic neuron differentiation	3/389	40/23328	0.028840982	0.08502	0.05406	Cxcl12/Sf	3
GO:0086004	GO:0086004	regulation of cardiac muscle cell contraction	3/389	40/23328	0.028840982	0.08502	0.05406	Gja5/Cav1	3
GO:0090175	GO:0090175	regulation of establishment of planar polarity	3/389	40/23328	0.028840982	0.08502	0.05406	Sfrp2/Dab	3
GO:0010565	GO:0010565	regulation of cellular ketone metabolic process	6/389	139/23328	0.029284483	0.08627	0.05485	Fabp5/Ca	6
GO:0060401	GO:0060401	cytosolic calcium ion transport	7/389	177/23328	0.02935719	0.08642	0.05495	Thy1/Prnp	7
GO:0032922	GO:0032922	circadian regulation of gene expression	4/389	70/23328	0.029507946	0.08661	0.05507	Id3/Id1/Id2	4
GO:0055117	GO:0055117	regulation of cardiac muscle contraction	4/389	70/23328	0.029507946	0.08661	0.05507	Gja5/Cav1	4
GO:0150076	GO:0150076	neuroinflammatory response	4/389	70/23328	0.029507946	0.08661	0.05507	Cd200/Clu	4
GO:2000107	GO:2000107	negative regulation of leukocyte apoptotic process	4/389	70/23328	0.029507946	0.08661	0.05507	Cxcl12/Bm	4
GO:0031668	GO:0031668	cellular response to extracellular stimulus	8/389	217/23328	0.029695896	0.0871	0.05538	Sfrp2/Vca	8
GO:0007189	GO:0007189	adenylate cyclase-activating G protein-coupled recepto	6/389	140/23328	0.030183514	0.0884	0.05621	Adrb2/Adc	6
GO:0045778	GO:0045778	positive regulation of ossification	5/389	104/23328	0.03024884	0.0884	0.05621	Ptn/Adrb2	5
GO:0050886	GO:0050886	endocrine process	5/389	104/23328	0.03024884	0.0884	0.05621	Gja5/Aqp1	5
GO:0060349	GO:0060349	bone morphogenesis	5/389	104/23328	0.03024884	0.0884	0.05621	Bmp4/Sfrp	5
GO:1901222	GO:1901222	regulation of NIK/NF-kappaB signaling	5/389	104/23328	0.03024884	0.0884	0.05621	Adgrg3/Pr	5
GO:0070661	GO:0070661	leukocyte proliferation	11/389	344/23328	0.030385872	0.08874	0.05643	Prnp/Cxcl	11
GO:0032412	GO:0032412	regulation of ion transmembrane transporter activity	9/389	259/23328	0.030417439	0.08877	0.05644	Reln/Fxyd	9
GO:1903532	GO:1903532	positive regulation of secretion by cell	12/389	388/23328	0.03048842	0.08891	0.05654	Rgcc/Cd3	12
GO:0097300	GO:0097300	programmed necrotic cell death	3/389	41/23328	0.0307438	0.08959	0.05697	Cav1/Slc2	3
GO:0007492	GO:0007492	endoderm development	4/389	71/23328	0.030872468	0.08971	0.05704	Sox17/Bm	4
GO:0042733	GO:0042733	embryonic digit morphogenesis	4/389	71/23328	0.030872468	0.08971	0.05704	Bmp4/Sfrp	4
GO:0043113	GO:0043113	receptor clustering	4/389	71/23328	0.030872468	0.08971	0.05704	Reln/Thy1	4
GO:0061035	GO:0061035	regulation of cartilage development	4/389	71/23328	0.030872468	0.08971	0.05704	Maf/Bmp4	4
GO:0001909	GO:0001909	leukocyte mediated cytotoxicity	6/389	141/23328	0.031100444	0.09022	0.05737	H2-Q6/Pro	6
GO:0009799	GO:0009799	specification of symmetry	6/389	141/23328	0.031100444	0.09022	0.05737	Tbx1/Sox1	6
GO:0007188	GO:0007188	adenylate cyclase-modulating G protein-coupled recep	8/389	219/23328	0.031112688	0.09022	0.05737	Adrb2/Adc	8
GO:0001910	GO:0001910	regulation of leukocyte mediated cytotoxicity	5/389	105/23328	0.031343753	0.09082	0.05775	H2-Q6/B2	5
GO:0007219	GO:0007219	Notch signaling pathway	7/389	180/23328	0.031747079	0.09104	0.05789	Dtx1/Enhc	7
GO:1902107	GO:1902107	positive regulation of leukocyte differentiation	7/389	180/23328	0.031747079	0.09104	0.05789	Gas6/Il2rg	7
GO:0001710	GO:0001710	mesodermal cell fate commitment	2/389	17/23328	0.031976636	0.09104	0.05789	Bmp4/Sfrp	2
GO:0002024	GO:0002024	diet induced thermogenesis	2/389	17/23328	0.031976636	0.09104	0.05789	Adrb2/Clic	2
GO:0003184	GO:0003184	pulmonary valve morphogenesis	2/389	17/23328	0.031976636	0.09104	0.05789	Gja5/Bmp	2
GO:0006968	GO:0006968	cellular defense response	2/389	17/23328	0.031976636	0.09104	0.05789	B2m/Il33	2
GO:0007096	GO:0007096	regulation of exit from mitosis	2/389	17/23328	0.031976636	0.09104	0.05789	Rgcc/Cdk	2
GO:0010934	GO:0010934	macrophage cytokine production	2/389	17/23328	0.031976636	0.09104	0.05789	Cd36/Gas	2
GO:0021681	GO:0021681	cerebellar granular layer development	2/389	17/23328	0.031976636	0.09104	0.05789	Serpine2/	2
GO:0033194	GO:0033194	response to hydroperoxide	2/389	17/23328	0.031976636	0.09104	0.05789	Cd36/Cd3	2
GO:0033623	GO:0033623	regulation of integrin activation	2/389	17/23328	0.031976636	0.09104	0.05789	Selp/Fblim	2
GO:0034349	GO:0034349	glial cell apoptotic process	2/389	17/23328	0.031976636	0.09104	0.05789	Gas6/Aka	2
GO:0040015	GO:0040015	negative regulation of multicellular organism growth	2/389	17/23328	0.031976636	0.09104	0.05789	Lgmn/Adr	2
GO:0042415	GO:0042415	norepinephrine metabolic process	2/389	17/23328	0.031976636	0.09104	0.05789	Epas1/Ly6	2
GO:0048875	GO:0048875	chemical homeostasis within a tissue	2/389	17/23328	0.031976636	0.09104	0.05789	Epas1/Ad	2
GO:0060973	GO:0060973	cell migration involved in heart development	2/389	17/23328	0.031976636	0.09104	0.05789	Bmp4/Eng	2
GO:0070293	GO:0070293	renal absorption	2/389	17/23328	0.031976636	0.09104	0.05789	Aqp1/Aqp	2
GO:0071636	GO:0071636	positive regulation of transforming growth factor beta p	2/389	17/23328	0.031976636	0.09104	0.05789	Cd34/Cd2	2
GO:0072010	GO:0072010	glomerular epithelium development	2/389	17/23328	0.031976636	0.09104	0.05789	Bmp4/Pdg	2

GO:0086012	GO:0086012	membrane depolarization during cardiac muscle cell ac	2/389	17/23328	0.031976636	0.09104	0.05789	Gja5/Ank3	2
GO:1900119	GO:1900119	positive regulation of execution phase of apoptosis	2/389	17/23328	0.031976636	0.09104	0.05789	Ptgis/Bok	2
GO:1900221	GO:1900221	regulation of amyloid-beta clearance	2/389	17/23328	0.031976636	0.09104	0.05789	Apoe/Clu	2
GO:1902894	GO:1902894	negative regulation of pri-miRNA transcription by RNA	2/389	17/23328	0.031976636	0.09104	0.05789	Bmp4/Pdg	2
GO:2000369	GO:2000369	regulation of clathrin-dependent endocytosis	2/389	17/23328	0.031976636	0.09104	0.05789	Sh3gl3/Da	2
GO:2000380	GO:2000380	regulation of mesoderm development	2/389	17/23328	0.031976636	0.09104	0.05789	Sfrp2/Gja1	2
GO:0030111	GO:0030111	regulation of Wnt signaling pathway	10/389	304/23328	0.032189919	0.09158	0.05823	Sox17/Tm	10
GO:0000041	GO:0000041	transition metal ion transport	5/389	106/23328	0.032463206	0.09216	0.0586	Cp/Lcn2/E	5
GO:0006022	GO:0006022	aminoglycan metabolic process	5/389	106/23328	0.032463206	0.09216	0.0586	Lyve1/Pdg	5
GO:0035710	GO:0035710	CD4-positive, alpha-beta T cell activation	5/389	106/23328	0.032463206	0.09216	0.0586	Rsad2/Ga	5
GO:0007409	GO:0007409	axonogenesis	14/389	482/23328	0.032521405	0.09226	0.05867	Reln/Thy1	14
GO:0019058	GO:0019058	viral life cycle	8/389	221/23328	0.032575144	0.09229	0.05868	Rsad2/Oa	8
GO:1903050	GO:1903050	regulation of proteolysis involved in cellular protein cat	8/389	221/23328	0.032575144	0.09229	0.05868	Ecscr/Cav	8
GO:0009268	GO:0009268	response to pH	3/389	42/23328	0.03271104	0.09229	0.05868	Lgmn/Gja	3
GO:0010765	GO:0010765	positive regulation of sodium ion transport	3/389	42/23328	0.03271104	0.09229	0.05868	Adrb2/Ank	3
GO:0030501	GO:0030501	positive regulation of bone mineralization	3/389	42/23328	0.03271104	0.09229	0.05868	Ptn/Adrb2	3
GO:0046839	GO:0046839	phospholipid dephosphorylation	3/389	42/23328	0.03271104	0.09229	0.05868	Ephx2/Plp	3
GO:1900077	GO:1900077	negative regulation of cellular response to insulin stimu	3/389	42/23328	0.03271104	0.09229	0.05868	Ptprf/Socs	3
GO:2000279	GO:2000279	negative regulation of DNA biosynthetic process	3/389	42/23328	0.03271104	0.09229	0.05868	Gja1/Dusp	3
GO:0043524	GO:0043524	negative regulation of neuron apoptotic process	7/389	182/23328	0.033410758	0.0942	0.0599	Lgmn/Apo	7
GO:0002367	GO:0002367	cytokine production involved in immune response	5/389	107/23328	0.033607322	0.09469	0.06021	Cd36/Rsa	5
GO:0043627	GO:0043627	response to estrogen	4/389	73/23328	0.033711458	0.09491	0.06035	Cav1/Mmp	4
GO:0002708	GO:0002708	positive regulation of lymphocyte mediated immunity	6/389	144/23328	0.033959702	0.09548	0.06071	H2-Q6/Rs	6
GO:0043624	GO:0043624	cellular protein complex disassembly	6/389	144/23328	0.033959702	0.09548	0.06071	Sh3gl3/Ds	6
GO:0000082	GO:0000082	G1/S transition of mitotic cell cycle	7/389	183/23328	0.03426398	0.09627	0.06121	Rgcc/Plk2	7
GO:0003298	GO:0003298	physiological muscle hypertrophy	3/389	43/23328	0.034742402	0.09731	0.06187	Rgs4/Slc2	3
GO:0003301	GO:0003301	physiological cardiac muscle hypertrophy	3/389	43/23328	0.034742402	0.09731	0.06187	Rgs4/Slc2	3
GO:0055026	GO:0055026	negative regulation of cardiac muscle tissue developm	3/389	43/23328	0.034742402	0.09731	0.06187	Rgs4/Gja1	3
GO:0061049	GO:0061049	cell growth involved in cardiac muscle cell developmer	3/389	43/23328	0.034742402	0.09731	0.06187	Rgs4/Slc2	3
GO:0002224	GO:0002224	toll-like receptor signaling pathway	5/389	108/23328	0.034776215	0.09731	0.06187	Lbp/Cd36/	5
GO:0008306	GO:0008306	associative learning	5/389	108/23328	0.034776215	0.09731	0.06187	Reln/Nts/L	5
GO:0031331	GO:0031331	positive regulation of cellular catabolic process	12/389	396/23328	0.03481401	0.09735	0.0619	Ecscr/Adr	12
GO:1903708	GO:1903708	positive regulation of hemopoiesis	8/389	224/23328	0.034855678	0.0974	0.06193	Flt1/Gas6/	8
GO:0006633	GO:0006633	fatty acid biosynthetic process	6/389	145/23328	0.034949286	0.09759	0.06205	Fabp5/Mg	6
GO:2001235	GO:2001235	positive regulation of apoptotic signaling pathway	7/389	184/23328	0.035131565	0.09782	0.0622	Timp3/Ca	7
GO:0000188	GO:0000188	inactivation of MAPK activity	2/389	18/23328	0.035584112	0.09782	0.0622	Cav1/Dus	2
GO:0002281	GO:0002281	macrophage activation involved in immune response	2/389	18/23328	0.035584112	0.09782	0.0622	Lbp/Il33	2
GO:0003214	GO:0003214	cardiac left ventricle morphogenesis	2/389	18/23328	0.035584112	0.09782	0.0622	Sfrp2/Cpe	2
GO:0006670	GO:0006670	sphingosine metabolic process	2/389	18/23328	0.035584112	0.09782	0.0622	Plpp3/Plp	2
GO:0006700	GO:0006700	C21-steroid hormone biosynthetic process	2/389	18/23328	0.035584112	0.09782	0.0622	Dab2/Egr1	2
GO:0006833	GO:0006833	water transport	2/389	18/23328	0.035584112	0.09782	0.0622	Aqp1/Aqp	2
GO:0007350	GO:0007350	blastoderm segmentation	2/389	18/23328	0.035584112	0.09782	0.0622	Pcsk6/Nrp	2
GO:0021783	GO:0021783	preganglionic parasympathetic fiber development	2/389	18/23328	0.035584112	0.09782	0.0622	Tbx1/Nrp2	2
GO:0032793	GO:0032793	positive regulation of CREB transcription factor activity	2/389	18/23328	0.035584112	0.09782	0.0622	Reln/Cd20	2
GO:0033574	GO:0033574	response to testosterone	2/389	18/23328	0.035584112	0.09782	0.0622	Spp1/Adh	2
GO:0035633	GO:0035633	maintenance of blood-brain barrier	2/389	18/23328	0.035584112	0.09782	0.0622	Cldn5/Ptg	2
GO:0042026	GO:0042026	protein refolding	2/389	18/23328	0.035584112	0.09782	0.0622	B2m/Hspa	2
GO:0042249	GO:0042249	establishment of planar polarity of embryonic epitheliu	2/389	18/23328	0.035584112	0.09782	0.0622	Sfrp2/Sfrp	2
GO:0042659	GO:0042659	regulation of cell fate specification	2/389	18/23328	0.035584112	0.09782	0.0622	Sox17/Sfr	2
GO:0044241	GO:0044241	lipid digestion	2/389	18/23328	0.035584112	0.09782	0.0622	Cd36/Aqp	2
GO:0044406	GO:0044406	adhesion of symbiont to host	2/389	18/23328	0.035584112	0.09782	0.0622	Scarb1/Ga	2
GO:0050710	GO:0050710	negative regulation of cytokine secretion	2/389	18/23328	0.035584112	0.09782	0.0622	Rgcc/Srgr	2
GO:0060841	GO:0060841	venous blood vessel development	2/389	18/23328	0.035584112	0.09782	0.0622	Prox1/Eng	2
GO:0070571	GO:0070571	negative regulation of neuron projection regeneration	2/389	18/23328	0.035584112	0.09782	0.0622	Thy1/Klk8	2
GO:1900153	GO:1900153	positive regulation of nuclear-transcribed mRNA catab	2/389	18/23328	0.035584112	0.09782	0.0622	Btg2/Zfp3	2
GO:1903019	GO:1903019	negative regulation of glycoprotein metabolic process	2/389	18/23328	0.035584112	0.09782	0.0622	Bace2/Cst	2
GO:1904706	GO:1904706	negative regulation of vascular associated smooth mus	2/389	18/23328	0.035584112	0.09782	0.0622	Rgs5/Cdk	2
GO:0002702	GO:0002702	positive regulation of production of molecular mediator	5/389	109/23328	0.035969992	0.09875	0.06279	Cd36/Rsa	5
GO:0051668	GO:0051668	localization within membrane	5/389	109/23328	0.035969992	0.09875	0.06279	Reln/Thy1	5
GO:0015672	GO:0015672	monovalent inorganic cation transport	14/389	489/23328	0.036026329	0.09884	0.06285	Fxyd6/Ser	14
GO:0032350	GO:0032350	regulation of hormone metabolic process	3/389	44/23328	0.036837537	0.10072	0.06405	Duoxa2/D	3
GO:0043403	GO:0043403	skeletal muscle tissue regeneration	3/389	44/23328	0.036837537	0.10072	0.06405	Cd9/Gja1/	3
GO:0044091	GO:0044091	membrane biogenesis	3/389	44/23328	0.036837537	0.10072	0.06405	Ank3/Anx	3
GO:0046621	GO:0046621	negative regulation of organ growth	3/389	44/23328	0.036837537	0.10072	0.06405	Rgs4/Gja1	3
GO:1903672	GO:1903672	positive regulation of sprouting angiogenesis	3/389	44/23328	0.036837537	0.10072	0.06405	Aplnr/Plk2	3
GO:0002460	GO:0002460	adaptive immune response based on somatic recombi	13/389	445/23328	0.036992816	0.10108	0.06427	Fgl2/H2-Q	13
GO:0038061	GO:0038061	NIK/NF-kappaB signaling	5/389	110/23328	0.037188751	0.10141	0.06448	Adgrg3/Pr	5
GO:0045089	GO:0045089	positive regulation of innate immune response	5/389	110/23328	0.037188751	0.10141	0.06448	Lbp/Irf7/M	5
GO:2000106	GO:2000106	regulation of leukocyte apoptotic process	5/389	110/23328	0.037188751	0.10141	0.06448	Cxcl12/Br	5
GO:0006956	GO:0006956	complement activation	7/389	187/23328	0.037821275	0.10307	0.06554	Rgcc/Serp	7
GO:0001676	GO:0001676	long-chain fatty acid metabolic process	5/389	111/23328	0.038432583	0.10459	0.06651	Ephx2/Cd	5
GO:0030316	GO:0030316	osteoclast differentiation	5/389	111/23328	0.038432583	0.10459	0.06651	Anxa2/Jur	5
GO:0062012	GO:0062012	regulation of small molecule metabolic process	11/389	358/23328	0.038784666	0.10482	0.06665	Ephx2/Fal	11
GO:0010464	GO:0010464	regulation of mesenchymal cell proliferation	3/389	45/23328	0.038996055	0.10482	0.06665	Ptn/Tbx1/I	3
GO:0032873	GO:0032873	negative regulation of stress-activated MAPK cascade	3/389	45/23328	0.038996055	0.10482	0.06665	Sfrp2/Sfrp	3
GO:0042181	GO:0042181	ketone biosynthetic process	3/389	45/23328	0.038996055	0.10482	0.06665	Aldoa/Dat	3
GO:0060421	GO:0060421	positive regulation of heart growth	3/389	45/23328	0.038996055	0.10482	0.06665	Slc25a4/F	3
GO:0070303	GO:0070303	negative regulation of stress-activated protein kinase s	3/389	45/23328	0.038996055	0.10482	0.06665	Sfrp2/Sfrp	3
GO:0071300	GO:0071300	cellular response to retinoic acid	3/389	45/23328	0.038996055	0.10482	0.06665	Tbx1/Aqp1	3
GO:0071526	GO:0071526	semaphorin-plexin signaling pathway	3/389	45/23328	0.038996055	0.10482	0.06665	Nrp2/Ncar	3
GO:0090184	GO:0090184	positive regulation of kidney development	3/389	45/23328	0.038996055	0.10482	0.06665	Bmp4/Pdg	3
GO:2000826	GO:2000826	regulation of heart morphogenesis	3/389	45/23328	0.038996055	0.10482	0.06665	Tbx1/Bmp	3
GO:0002221	GO:0002221	pattern recognition receptor signaling pathway	6/389	149/23328	0.039092605	0.10482	0.06665	Lbp/Cd36/	6
GO:0002031	GO:0002031	G protein-coupled receptor internalization	2/389	19/23328	0.039340378	0.10482	0.06665	Htr2b/Adr	2
GO:0002544	GO:0002544	chronic inflammatory response	2/389	19/23328	0.039340378	0.10482	0.06665	Gja1/S100	2
GO:0002726	GO:0002726	positive regulation of T cell cytokine production	2/389	19/23328	0.039340378	0.10482	0.06665	Rsad2/B2	2
GO:0006027	GO:0006027	glycosaminoglycan catabolic process	2/389	19/23328	0.039340378	0.10482	0.06665	Lyve1/Pgl	2
GO:0006590	GO:0006590	thyroid hormone generation	2/389	19/23328	0.039340378	0.10482	0.06665	Duoxa2/C	2
GO:0010566	GO:0010566	regulation of ketone biosynthetic process	2/389	19/23328	0.039340378	0.10482	0.06665	Dab2/Egr1	2
GO:0032352	GO:0032352	positive regulation of hormone metabolic process	2/389	19/23328	0.039340378	0.10482	0.06665	Dab2/Egr1	2
GO:0032823	GO:0032823	regulation of natural killer cell differentiation	2/389	19/23328	0.039340378	0.10482	0.06665	Gas6/Pgly	2
GO:0035988	GO:0035988	chondrocyte proliferation	2/389	19/23328	0.039340378	0.10482	0.06665	Stc1/Com	2
GO:0035994	GO:0035994	response to muscle stretch	2/389	19/23328	0.039340378	0.10482	0.06665	Jun/Fos	2
GO:0042730	GO:0042730	fibrinolysis	2/389	19/23328	0.039340378	0.10482	0.06665	Serping1/	2
GO:0060033	GO:0060033	anatomical structure regression	2/389	19/23328	0.039340378	0.10482	0.06665	Flt1/Ninj1	2
GO:0060099	GO:0060099	regulation of phagocytosis, engulfment	2/389	19/23328	0.039340378	0.10482	0.06665	Lbp/Cd36	2
GO:0060231	GO:0060231	mesenchymal to epithelial transition	2/389	19/23328	0.039340378	0.10482	0.06665	Tcf15/Bm	2
GO:0060707	GO:0060707	trophoblast giant cell differentiation	2/389	19/23328	0.039340378	0.10482	0.06665	Nr2f2/Soc	2
GO:0060712	GO:0060712	spongiotrophoblast layer development	2/389	19/23328	0.039340378	0.10482	0.06665	Socs3/Adı	2
GO:0072578	GO:0072578	neurotransmitter-gated ion channel clustering	2/389	19/23328	0.039340378	0.10482	0.06665	Reln/Apoe	2
GO:1900451	GO:1900451	positive regulation of glutamate receptor signaling path	2/389	19/23328	0.039340378	0.10482	0.06665	Reln/Adrb	2
GO:1904754	GO:1904754	positive regulation of vascular associated smooth mus	2/389	19/23328	0.039340378	0.10482	0.06665	Igfbp5/Pd	2
GO:1905276	GO:1905276	regulation of epithelial tube formation	2/389	19/23328	0.039340378	0.10482	0.06665	Sfrp2/Sfrp	2
GO:2000269	GO:2000269	regulation of fibroblast apoptotic process	2/389	19/23328	0.039340378	0.10482	0.06665	Gas6/Sfrp	2
GO:0007009	GO:0007009	plasma membrane organization	5/389	112/23328	0.039701572	0.10565	0.06718	Ndrg1/Ank	5
GO:0038127	GO:0038127	ERBB signaling pathway	5/389	112/23328	0.039701572	0.10565	0.06718	Ptprf/Lgm	5
GO:0045843	GO:0045843	negative regulation of striated muscle tissue developm	4/389	77/23328	0.039831409	0.10585	0.06731	Bmp4/Rgs	4
GO:0071277	GO:0071277	cellular response to calcium ion	4/389	77/23328	0.039831409	0.10585	0.06731	Lgmn/Jun	4
GO:0042593	GO:0042593	glucose homeostasis	9/389	273/23328	0.04037941	0.10724	0.06819	Igfbp5/Cd	9
GO:0002274	GO:0002274	myeloid leukocyte activation	8/389	231/23328	0.040591399	0.10773	0.0685	Lbp/Cd20	8
GO:0120032	GO:0120032	regulation of plasma membrane bounded cell projectio	7/389	190/23328	0.04064295	0.1078	0.06855	Gap43/Cd	7
GO:0033500	GO:0033500	carbohydrate homeostasis	9/389	274/23328	0.041166254	0.10904	0.06933	Igfbp5/Cd	9
GO:0045740	GO:0045740	positive regulation of DNA replication	3/389	46/23328	0.041217518	0.10904	0.06933	Pdgfb/Cst	3
GO:0098926	GO:0098926	postsynaptic signal transduction	3/389	46/23328	0.041217518	0.10904	0.06933	Ly6c1/Ly6	3
GO:2000404	GO:2000404	regulation of T cell migration	3/389	46/23328	0.041217518	0.10904	0.06933	Ccl21a/Cc	3
GO:0048593	GO:0048593	camera-type eye morphogenesis	6/389	151/23328	0.041276427	0.10912	0.06939	Thy1/Ptn/I	6
GO:0032872	GO:0032872	regulation of stress-activated MAPK cascade	8/389	232/23328	0.041459051	0.10954	0.06965	Flt4/Prdx1	8
GO:0043588	GO:0043588	skin development	9/389	275/23328	0.041963403	0.1108	0.07045	Igfbp5/Tcf	9
GO:0007498	GO:0007498	mesoderm development	5/389	114/23328	0.042315317	0.11144	0.07086	Tcf15/Tbx	5
GO:0010927	GO:0010927	cellular component assembly involved in morphogenes	5/389	114/23328	0.042315317	0.11144	0.07086	Cd9/Prox1	5
GO:0032649	GO:0032649	regulation of interferon-gamma production	5/389	114/23328	0.042315317	0.11144	0.07086	Prnp/Cd2/	5
GO:1905330	GO:1905330	regulation of morphogenesis of an epithelium	5/389	114/23328	0.042315317	0.11144	0.07086	Bmp4/Sfrp	5
GO:0060491	GO:0060491	regulation of cell projection assembly	7/389	192/23328	0.042598283	0.11211	0.07129	Gap43/Cd	7
GO:0046879	GO:0046879	hormone secretion	11/389	364/23328	0.042837196	0.11263	0.07162	Spp1/Nmt	11
GO:0034644	GO:0034644	cellular response to UV	4/389	79/23328	0.043113006	0.11263	0.07162	Mfap4/Aq	4
GO:0048635	GO:0048635	negative regulation of muscle organ development	4/389	79/23328	0.043113006	0.11263	0.07162	Bmp4/Rgs	4
GO:0060191	GO:0060191	regulation of lipase activity	4/389	79/23328	0.043113006	0.11263	0.07162	Flt1/Gpihb	4
GO:0010818	GO:0010818	T cell chemotaxis	2/389	20/23328	0.043239494	0.11263	0.07162	Cxcl12/Cx	2
GO:0010984	GO:0010984	regulation of lipoprotein particle clearance	2/389	20/23328	0.043239494	0.11263	0.07162	Anxa2/Gp	2

GO:0045986	GO:0045986	negative regulation of smooth muscle contraction	2/389	20/23328	0.043239494	0.11263	0.07162	Adrb2/Cal	2
GO:0046519	GO:0046519	sphingoid metabolic process	2/389	20/23328	0.043239494	0.11263	0.07162	Plpp3/Plp	2
GO:0046851	GO:0046851	negative regulation of bone remodeling	2/389	20/23328	0.043239494	0.11263	0.07162	Cd38/Sfrp	2
GO:0060307	GO:0060307	regulation of ventricular cardiac muscle cell membrane	2/389	20/23328	0.043239494	0.11263	0.07162	Gja5/Gja1	2
GO:0072189	GO:0072189	ureter development	2/389	20/23328	0.043239494	0.11263	0.07162	Sox17/Bm	2
GO:0072243	GO:0072243	metanephric nephron epithelium development	2/389	20/23328	0.043239494	0.11263	0.07162	Pdgfb/Aqp	2
GO:1900151	GO:1900151	regulation of nuclear-transcribed mRNA catabolic proc	2/389	20/23328	0.043239494	0.11263	0.07162	Btg2/Zfp3	2
GO:1901028	GO:1901028	regulation of mitochondrial outer membrane permeabil	2/389	20/23328	0.043239494	0.11263	0.07162	Ier3/Bok	2
GO:1905153	GO:1905153	regulation of membrane invagination	2/389	20/23328	0.043239494	0.11263	0.07162	Lbp/Cd36	2
GO:2000136	GO:2000136	regulation of cell proliferation involved in heart morpho	2/389	20/23328	0.043239494	0.11263	0.07162	Tbx1/Bmp	2
GO:0001941	GO:0001941	postsynaptic membrane organization	3/389	47/23328	0.043501453	0.1131	0.07192	Reln/Ptn/	3
GO:0035458	GO:0035458	cellular response to interferon-beta	3/389	47/23328	0.043501453	0.1131	0.07192	Iigp1/Mnd	3
GO:0042088	GO:0042088	T-helper 1 type immune response	3/389	47/23328	0.043501453	0.1131	0.07192	Gadd45g/	3
GO:0019079	GO:0019079	viral genome replication	5/389	115/23328	0.043660201	0.11344	0.07213	Rsad2/Oa	5
GO:0031334	GO:0031334	positive regulation of protein-containing complex asse	8/389	235/23328	0.044135625	0.11453	0.07282	Gmfg/Cdc	8
GO:0070302	GO:0070302	regulation of stress-activated protein kinase signaling	8/389	235/23328	0.044135625	0.11453	0.07282	Flt4/Prdx1	8
GO:1901568	GO:1901568	fatty acid derivative metabolic process	6/389	154/23328	0.044694021	0.1159	0.0737	Fabp5/Mg	6
GO:2001259	GO:2001259	positive regulation of cation channel activity	4/389	80/23328	0.044809218	0.11613	0.07384	Reln/Adrb	4
GO:0001774	GO:0001774	microglial cell activation	3/389	48/23328	0.045847345	0.11844	0.07531	Clu/Il33/Ju	3
GO:0002269	GO:0002269	leukocyte activation involved in inflammatory response	3/389	48/23328	0.045847345	0.11844	0.07531	Clu/Il33/Ju	3
GO:0010883	GO:0010883	regulation of lipid storage	3/389	48/23328	0.045847345	0.11844	0.07531	Cd36/Sca	3
GO:0038066	GO:0038066	p38MAPK cascade	3/389	48/23328	0.045847345	0.11844	0.07531	Gadd45g/	3
GO:0050850	GO:0050850	positive regulation of calcium-mediated signaling	3/389	48/23328	0.045847345	0.11844	0.07531	Cdh13/Nc	3
GO:0032414	GO:0032414	positive regulation of ion transmembrane transporter a	5/389	117/23328	0.04642626	0.11979	0.07617	Reln/Adrb	5
GO:1901890	GO:1901890	positive regulation of cell junction assembly	5/389	117/23328	0.04642626	0.11979	0.07617	Thy1/Cldn	5
GO:0021675	GO:0021675	nerve development	4/389	81/23328	0.04654233	0.11993	0.07626	Tbx1/Serp	4
GO:0043525	GO:0043525	positive regulation of neuron apoptotic process	4/389	81/23328	0.04654233	0.11993	0.07626	Ptprf/Prnp	4
GO:0002706	GO:0002706	regulation of lymphocyte mediated immunity	7/389	196/23328	0.046689251	0.12024	0.07645	H2-Q6/Rs	7
GO:0003177	GO:0003177	pulmonary valve development	2/389	21/23328	0.047275681	0.12053	0.07664	Gja5/Bmp	2
GO:0003417	GO:0003417	growth plate cartilage development	2/389	21/23328	0.047275681	0.12053	0.07664	Stc1/Com	2
GO:0010039	GO:0010039	response to iron ion	2/389	21/23328	0.047275681	0.12053	0.07664	B2m/Trf	2
GO:0044346	GO:0044346	fibroblast apoptotic process	2/389	21/23328	0.047275681	0.12053	0.07664	Gas6/Sfrp	2
GO:0045063	GO:0045063	T-helper 1 cell differentiation	2/389	21/23328	0.047275681	0.12053	0.07664	Gadd45g/	2
GO:0045475	GO:0045475	locomotor rhythm	2/389	21/23328	0.047275681	0.12053	0.07664	Id2/Egr1	2
GO:0046716	GO:0046716	muscle cell cellular homeostasis	2/389	21/23328	0.047275681	0.12053	0.07664	Aldoa/Lox	2
GO:0048486	GO:0048486	parasympathetic nervous system development	2/389	21/23328	0.047275681	0.12053	0.07664	Tbx1/Nrp2	2
GO:0051900	GO:0051900	regulation of mitochondrial depolarization	2/389	21/23328	0.047275681	0.12053	0.07664	Bok/Dcn	2
GO:0061323	GO:0061323	cell proliferation involved in heart morphogenesis	2/389	21/23328	0.047275681	0.12053	0.07664	Tbx1/Bmp	2
GO:0090026	GO:0090026	positive regulation of monocyte chemotaxis	2/389	21/23328	0.047275681	0.12053	0.07664	Lgmn/Cxc	2
GO:0090036	GO:0090036	regulation of protein kinase C signaling	2/389	21/23328	0.047275681	0.12053	0.07664	Flt4/Akap	2
GO:0090050	GO:0090050	positive regulation of cell migration involved in sprouti	2/389	21/23328	0.047275681	0.12053	0.07664	Plk2/Anxa	2
GO:1901017	GO:1901017	negative regulation of potassium ion transmembrane t	2/389	21/23328	0.047275681	0.12053	0.07664	Ank3/Cav	2
GO:1902004	GO:1902004	positive regulation of amyloid-beta formation	2/389	21/23328	0.047275681	0.12053	0.07664	Apoe/Clu	2
GO:2000726	GO:2000726	negative regulation of cardiac muscle cell differentiatic	2/389	21/23328	0.047275681	0.12053	0.07664	Rgs4/Pi16	2
GO:0002253	GO:0002253	activation of immune response	12/389	416/23328	0.047503873	0.12103	0.07696	Thy1/Rgc	12
GO:0070588	GO:0070588	calcium ion transmembrane transport	9/389	282/23328	0.047835716	0.12168	0.07737	Thy1/Prnp	9
GO:0046632	GO:0046632	alpha-beta T cell differentiation	5/389	118/23328	0.047847519	0.12168	0.07737	Rsad2/Ga	5
GO:2000177	GO:2000177	regulation of neural precursor cell proliferation	5/389	118/23328	0.047847519	0.12168	0.07737	Ptn/Prox1	5
GO:0010332	GO:0010332	response to gamma radiation	3/389	49/23328	0.048254645	0.1224	0.07783	Egr1/Crya	3
GO:0043550	GO:0043550	regulation of lipid kinase activity	3/389	49/23328	0.048254645	0.1224	0.07783	Flt1/Pdgfb	3
GO:1902041	GO:1902041	regulation of extrinsic apoptotic signaling pathway via	3/389	49/23328	0.048254645	0.1224	0.07783	Timp3/Sfr	3
GO:1902808	GO:1902808	positive regulation of cell cycle G1/S phase transition	3/389	49/23328	0.048254645	0.1224	0.07783	Rgcc/Anx	3
GO:0015914	GO:0015914	phospholipid transport	4/389	82/23328	0.048312295	0.1224	0.07783	Pltp/Apoe	4
GO:0050805	GO:0050805	negative regulation of synaptic transmission	4/389	82/23328	0.048312295	0.1224	0.07783	Mgll/Plk2/	4
GO:0009914	GO:0009914	hormone transport	11/389	372/23328	0.048682324	0.12326	0.07838	Spp1/Nmb	11
GO:0046578	GO:0046578	regulation of Ras protein signal transduction	8/389	240/23328	0.048844965	0.1236	0.07859	F11r/Apoe	8
GO:0001952	GO:0001952	regulation of cell-matrix adhesion	5/389	119/23328	0.049294307	0.12466	0.07927	Thy1/Cd3	5
GO:0048562	GO:0048562	embryonic organ morphogenesis	10/389	328/23328	0.049452224	0.12498	0.07947	Hoxd9/Tb	10
GO:0046777	GO:0046777	protein autophosphorylation	8/389	241/23328	0.049824502	0.12584	0.08002	Thy1/Flt4/	8
GO:0001738	GO:0001738	morphogenesis of a polarized epithelium	4/389	83/23328	0.050119046	0.12643	0.08039	Tcf15/Sfrp	4
GO:1903901	GO:1903901	negative regulation of viral life cycle	4/389	83/23328	0.050119046	0.12643	0.08039	Rsad2/Oa	4
GO:0071222	GO:0071222	cellular response to lipopolysaccharide	9/389	285/23328	0.050511266	0.12734	0.08097	Lbp/Cd36	9
GO:0018198	GO:0018198	peptidyl-cysteine modification	3/389	50/23328	0.050722767	0.12745	0.08104	Gapdh/S1	3
GO:0032715	GO:0032715	negative regulation of interleukin-6 production	3/389	50/23328	0.050722767	0.12745	0.08104	Cd200/Klf	3
GO:0043392	GO:0043392	negative regulation of DNA binding	3/389	50/23328	0.050722767	0.12745	0.08104	Id1/Id2/Ju	3
GO:0070169	GO:0070169	positive regulation of biomineral tissue development	3/389	50/23328	0.050722767	0.12745	0.08104	Ptn/Adrb2	3
GO:0110151	GO:0110151	positive regulation of biomineralization	3/389	50/23328	0.050722767	0.12745	0.08104	Ptn/Adrb2	3
GO:0009266	GO:0009266	response to temperature stimulus	6/389	159/23328	0.050772276	0.12745	0.08104	Cd34/Adr	6
GO:2000058	GO:2000058	regulation of ubiquitin-dependent protein catabolic pro	6/389	159/23328	0.050772276	0.12745	0.08104	Cav1/Plk2	6
GO:0032147	GO:0032147	activation of protein kinase activity	8/389	242/23328	0.050816692	0.12748	0.08106	Prnp/Bmp	8
GO:0006516	GO:0006516	glycoprotein catabolic process	2/389	22/23328	0.051443306	0.12787	0.08131	Ctsl/Cst3	2
GO:0009154	GO:0009154	purine ribonucleotide catabolic process	2/389	22/23328	0.051443306	0.12787	0.08131	Nudt4/Pde	2
GO:0009261	GO:0009261	ribonucleotide catabolic process	2/389	22/23328	0.051443306	0.12787	0.08131	Nudt4/Pde	2
GO:0010499	GO:0010499	proteasomal ubiquitin-independent protein catabolic pr	2/389	22/23328	0.051443306	0.12787	0.08131	Psmb9/Ps	2
GO:0010666	GO:0010666	positive regulation of cardiac muscle cell apoptotic pro	2/389	22/23328	0.051443306	0.12787	0.08131	Fndc1/Fb	2
GO:0032148	GO:0032148	activation of protein kinase B activity	2/389	22/23328	0.051443306	0.12787	0.08131	Pdgfb/Gas	2
GO:0035634	GO:0035634	response to stilbenoid	2/389	22/23328	0.051443306	0.12787	0.08131	Cd36/Hba	2
GO:0050860	GO:0050860	negative regulation of T cell receptor signaling pathwa	2/389	22/23328	0.051443306	0.12787	0.08131	Thy1/Prnp	2
GO:0051770	GO:0051770	positive regulation of nitric-oxide synthase biosynthetic	2/389	22/23328	0.051443306	0.12787	0.08131	Akap12/Il	2
GO:0060602	GO:0060602	branch elongation of an epithelium	2/389	22/23328	0.051443306	0.12787	0.08131	Tnc/Bmp4	2
GO:0099625	GO:0099625	ventricular cardiac muscle cell membrane repolarizatic	2/389	22/23328	0.051443306	0.12787	0.08131	Gja5/Gja1	2
GO:1901223	GO:1901223	negative regulation of NIK/NF-kappaB signaling	2/389	22/23328	0.051443306	0.12787	0.08131	Adgrg3/Ts	2
GO:1902307	GO:1902307	positive regulation of sodium ion transmembrane trans	2/389	22/23328	0.051443306	0.12787	0.08131	Ank3/Atp1	2
GO:1903055	GO:1903055	positive regulation of extracellular matrix organization	2/389	22/23328	0.051443306	0.12787	0.08131	Rgcc/Pdp	2
GO:1903975	GO:1903975	regulation of glial cell migration	2/389	22/23328	0.051443306	0.12787	0.08131	Vim/Efem	2
GO:0006942	GO:0006942	regulation of striated muscle contraction	4/389	84/23328	0.051962503	0.12908	0.08208	Gja5/Cav	4
GO:0044843	GO:0044843	cell cycle G1/S phase transition	7/389	201/23328	0.05214533	0.12938	0.08227	Rgcc/Plk2	7
GO:0070507	GO:0070507	regulation of microtubule cytoskeleton organization	7/389	201/23328	0.05214533	0.12938	0.08227	Cav1/Cdh	7
GO:0031648	GO:0031648	protein destabilization	3/389	51/23328	0.053251097	0.13204	0.08396	Sox17/Id1	3
GO:0090101	GO:0090101	negative regulation of transmembrane receptor protein	5/389	122/23328	0.053788046	0.13318	0.08469	Sfrp2/Cav	5
GO:0002718	GO:0002718	regulation of cytokine production involved in immune r	4/389	85/23328	0.053842572	0.13318	0.08469	Cd36/Rsa	4
GO:0014910	GO:0014910	regulation of smooth muscle cell migration	4/389	85/23328	0.053842572	0.13318	0.08469	Igfbp5/Pd	4
GO:0051937	GO:0051937	catecholamine transport	4/389	85/23328	0.053842572	0.13318	0.08469	Cxcl12/Sr	4
GO:0048565	GO:0048565	digestive tract development	5/389	123/23328	0.055337113	0.13671	0.08693	Sox17/Bm	5
GO:0010663	GO:0010663	positive regulation of striated muscle cell apoptotic pro	2/389	23/23328	0.055736891	0.13671	0.08693	Fndc1/Fb	2
GO:0010743	GO:0010743	regulation of macrophage derived foam cell differentiat	2/389	23/23328	0.055736891	0.13671	0.08693	Cd36/Lpl	2
GO:0010866	GO:0010866	regulation of triglyceride biosynthetic process	2/389	23/23328	0.055736891	0.13671	0.08693	Thrsp/Sca	2
GO:0031579	GO:0031579	membrane raft organization	2/389	23/23328	0.055736891	0.13671	0.08693	Anxa2/Ca	2
GO:0032331	GO:0032331	negative regulation of chondrocyte differentiation	2/389	23/23328	0.055736891	0.13671	0.08693	Bmp4/Efe	2
GO:0045056	GO:0045056	transcytosis	2/389	23/23328	0.055736891	0.13671	0.08693	Cd300lg/C	2
GO:0072202	GO:0072202	cell differentiation involved in metanephros developme	2/389	23/23328	0.055736891	0.13671	0.08693	Bmp4/Pdg	2
GO:0097062	GO:0097062	dendritic spine maintenance	2/389	23/23328	0.055736891	0.13671	0.08693	Prnp/Apoe	2
GO:0098703	GO:0098703	calcium ion import across plasma membrane	2/389	23/23328	0.055736891	0.13671	0.08693	Prnp/Adrb	2
GO:1901626	GO:1901626	regulation of postsynaptic membrane organization	2/389	23/23328	0.055736891	0.13671	0.08693	Ptn/Apoe	2
GO:0031646	GO:0031646	positive regulation of nervous system process	4/389	86/23328	0.055759142	0.13671	0.08693	Reln/Adrb	4
GO:0035050	GO:0035050	embryonic heart tube development	4/389	86/23328	0.055759142	0.13671	0.08693	Sox17/Gja	4
GO:0045104	GO:0045104	intermediate filament cytoskeleton organization	3/389	52/23328	0.055838984	0.13671	0.08693	Vim/Dst/N	3
GO:0048483	GO:0048483	autonomic nervous system development	3/389	52/23328	0.055838984	0.13671	0.08693	Tbx1/Nrp2	3
GO:0051353	GO:0051353	positive regulation of oxidoreductase activity	3/389	52/23328	0.055838984	0.13671	0.08693	Apoe/Sca	3
GO:1904036	GO:1904036	negative regulation of epithelial cell apoptotic process	3/389	52/23328	0.055838984	0.13671	0.08693	Cdh5/Gas	3
GO:0043434	GO:0043434	response to peptide hormone	10/389	336/23328	0.056366942	0.13792	0.0877	Ptprf/Socs	10
GO:0030879	GO:0030879	mammary gland development	6/389	164/23328	0.057333165	0.1402	0.08915	Igfbp5/Ho	6
GO:0001960	GO:0001960	negative regulation of cytokine-mediated signaling path	3/389	53/23328	0.058485755	0.14249	0.0906	Ptprf/Cav	3
GO:0032720	GO:0032720	negative regulation of tumor necrosis factor production	3/389	53/23328	0.058485755	0.14249	0.0906	Lbp/Cd34	3
GO:0045103	GO:0045103	intermediate filament-based process	3/389	53/23328	0.058485755	0.14249	0.0906	Vim/Dst/N	3
GO:0046902	GO:0046902	regulation of mitochondrial membrane permeability	3/389	53/23328	0.058485755	0.14249	0.0906	Ier3/Slc25	3
GO:0048713	GO:0048713	regulation of oligodendrocyte differentiation	3/389	53/23328	0.058485755	0.14249	0.0906	Ptn/Bmp4	3
GO:0051055	GO:0051055	negative regulation of lipid biosynthetic process	3/389	53/23328	0.058485755	0.14249	0.0906	Pdgfb/Apo	3
GO:1902414	GO:1902414	protein localization to cell junction	5/389	125/23328	0.058511956	0.14249	0.0906	Reln/Hspb	5
GO:0006575	GO:0006575	cellular modified amino acid metabolic process	6/389	165/23328	0.05870359	0.14287	0.09084	Reln/Ass1	6
GO:0008593	GO:0008593	regulation of Notch signaling pathway	4/389	88/23328	0.059701281	0.14521	0.09233	Dtx1/Enhc	4
GO:0071219	GO:0071219	cellular response to molecule of bacterial origin	9/389	295/23328	0.060133476	0.14526	0.09237	Lbp/Cd36	9
GO:0046822	GO:0046822	regulation of nucleocytoplasmic transport	5/389	126/23328	0.060137698	0.14526	0.09237	Cd36/Bmp	5
GO:0120254	GO:0120254	olefinic compound metabolic process	5/389	126/23328	0.060137698	0.14526	0.09237	Ephx2/Mg	5
GO:0031670	GO:0031670	cellular response to nutrient	2/389	24/23328	0.060151099	0.14526	0.09237	Gas6/Lpl	2
GO:0035455	GO:0035455	response to interferon-alpha	2/389	24/23328	0.060151099	0.14526	0.09237	Gas6/Bst2	2
GO:0043586	GO:0043586	tongue development	2/389	24/23328	0.060151099	0.14526	0.09237	Tbx1/Cyp	2
GO:0044331	GO:0044331	cell-cell adhesion mediated by cadherin	2/389	24/23328	0.060151099	0.14526	0.09237	Rgcc/Cdh	2
GO:0051882	GO:0051882	mitochondrial depolarization	2/389	24/23328	0.060151099	0.14526	0.09237	Bok/Dcn	2

GO:0070841	GO:0070841	inclusion body assembly	2/389	24/23328	0.060151099	0.14526	0.09237	Apoe/Clu	2
GO:0072207	GO:0072207	metanephric epithelium development	2/389	24/23328	0.060151099	0.14526	0.09237	Pdgfb/Aqp	2
GO:0099623	GO:0099623	regulation of cardiac muscle cell membrane repolariza	2/389	24/23328	0.060151099	0.14526	0.09237	Gja5/Gja1	2
GO:1902656	GO:1902656	calcium ion import into cytosol	2/389	24/23328	0.060151099	0.14526	0.09237	Prnp/Adrb	2
GO:0070663	GO:0070663	regulation of leukocyte proliferation	8/389	251/23328	0.060321638	0.14559	0.09257	Prnp/Bmp	8
GO:0014014	GO:0014014	negative regulation of gliogenesis	3/389	54/23328	0.061190704	0.14725	0.09363	Ptn/Bmp4	3
GO:0045687	GO:0045687	positive regulation of glial cell differentiation	3/389	54/23328	0.061190704	0.14725	0.09363	Ptn/Serpir	3
GO:0055025	GO:0055025	positive regulation of cardiac muscle tissue developme	3/389	54/23328	0.061190704	0.14725	0.09363	Bmp4/Slc	3
GO:0070542	GO:0070542	response to fatty acid	3/389	54/23328	0.061190704	0.14725	0.09363	Id3/Cd36/	3
GO:1903556	GO:1903556	negative regulation of tumor necrosis factor superfamil	3/389	54/23328	0.061190704	0.14725	0.09363	Lbp/Cd34	3
GO:0070301	GO:0070301	cellular response to hydrogen peroxide	4/389	89/23328	0.061726562	0.14845	0.09439	Lcn2/Aqp	4
GO:0009593	GO:0009593	detection of chemical stimulus	4/389	90/23328	0.063787768	0.15314	0.09737	Lbp/Rtp4/	4
GO:0046888	GO:0046888	negative regulation of hormone secretion	4/389	90/23328	0.063787768	0.15314	0.09737	Nmb/Ucp2	4
GO:1900076	GO:1900076	regulation of cellular response to insulin stimulus	4/389	90/23328	0.063787768	0.15314	0.09737	Ptprf/Socs	4
GO:0009948	GO:0009948	anterior/posterior axis specification	3/389	55/23328	0.063953102	0.15317	0.0974	Bmp4/Pcs	3
GO:0030834	GO:0030834	regulation of actin filament depolymerization	3/389	55/23328	0.063953102	0.15317	0.0974	Dstn/Tpm	3
GO:0050891	GO:0050891	multicellular organismal water homeostasis	3/389	55/23328	0.063953102	0.15317	0.0974	Aqp1/Aqp	3
GO:0086001	GO:0086001	cardiac muscle cell action potential	3/389	55/23328	0.063953102	0.15317	0.0974	Gja5/Ank3	3
GO:0042180	GO:0042180	cellular ketone metabolic process	7/389	211/23328	0.064217327	0.15371	0.09774	Fabp5/Ca	7
GO:0007095	GO:0007095	mitotic G2 DNA damage checkpoint	2/389	25/23328	0.064680737	0.15383	0.09782	Ier3/Cdkn	2
GO:0010894	GO:0010894	negative regulation of steroid biosynthetic process	2/389	25/23328	0.064680737	0.15383	0.09782	Apoe/Prox	2
GO:0032878	GO:0032878	regulation of establishment or maintenance of cell pola	2/389	25/23328	0.064680737	0.15383	0.09782	Cdh5/Tek	2
GO:0032897	GO:0032897	negative regulation of viral transcription	2/389	25/23328	0.064680737	0.15383	0.09782	Zfp36/Jun	2
GO:0036342	GO:0036342	post-anal tail morphogenesis	2/389	25/23328	0.064680737	0.15383	0.09782	Tcf15/Sfrp	2
GO:0045939	GO:0045939	negative regulation of steroid metabolic process	2/389	25/23328	0.064680737	0.15383	0.09782	Apoe/Prox	2
GO:0046426	GO:0046426	negative regulation of receptor signaling pathway via J	2/389	25/23328	0.064680737	0.15383	0.09782	Socs3/Ca	2
GO:0051894	GO:0051894	positive regulation of focal adhesion assembly	2/389	25/23328	0.064680737	0.15383	0.09782	Thy1/Tek	2
GO:1904738	GO:1904738	vascular associated smooth muscle cell migration	2/389	25/23328	0.064680737	0.15383	0.09782	Igfbp5/Pd	2
GO:1904752	GO:1904752	regulation of vascular associated smooth muscle cell r	2/389	25/23328	0.064680737	0.15383	0.09782	Igfbp5/Pd	2
GO:1905208	GO:1905208	negative regulation of cardiocyte differentiation	2/389	25/23328	0.064680737	0.15383	0.09782	Rgs4/Pi16	2
GO:0032273	GO:0032273	positive regulation of protein polymerization	5/389	129/23328	0.065167871	0.1549	0.09849	Gmfg/Cdc	5
GO:0031667	GO:0031667	response to nutrient levels	10/389	346/23328	0.065866164	0.15647	0.09949	Adrb2/Spp	10
GO:0048511	GO:0048511	rhythmic process	9/389	301/23328	0.066434984	0.15773	0.10029	Id3/Ptn/As	9
GO:0042446	GO:0042446	hormone biosynthetic process	3/389	56/23328	0.066772196	0.15797	0.10045	Scarb1/Da	3
GO:0044060	GO:0044060	regulation of endocrine process	3/389	56/23328	0.066772196	0.15797	0.10045	Gja5/Dab2	3
GO:0061900	GO:0061900	glial cell activation	3/389	56/23328	0.066772196	0.15797	0.10045	Clu/Il33/Ju	3
GO:0071675	GO:0071675	regulation of mononuclear cell migration	3/389	56/23328	0.066772196	0.15797	0.10045	Lgmn/Cxc	3
GO:0098815	GO:0098815	modulation of excitatory postsynaptic potential	3/389	56/23328	0.066772196	0.15797	0.10045	Reln/Adrb	3
GO:1900449	GO:1900449	regulation of glutamate receptor signaling pathway	3/389	56/23328	0.066772196	0.15797	0.10045	Reln/Prnp	3
GO:0042177	GO:0042177	negative regulation of protein catabolic process	5/389	130/23328	0.066895441	0.15817	0.10058	Serpine2/	5
GO:0002832	GO:0002832	negative regulation of response to biotic stimulus	4/389	92/23328	0.068017239	0.16045	0.10203	Fgl2/Serp	4
GO:0014909	GO:0014909	smooth muscle cell migration	4/389	92/23328	0.068017239	0.16045	0.10203	Igfbp5/Pd	4
GO:0043900	GO:0043900	regulation of multi-organism process	4/389	92/23328	0.068017239	0.16045	0.10203	Gja1/Pde	4
GO:0045685	GO:0045685	regulation of glial cell differentiation	4/389	92/23328	0.068017239	0.16045	0.10203	Ptn/Serpir	4
GO:0032984	GO:0032984	protein-containing complex disassembly	7/389	214/23328	0.068143461	0.16066	0.10216	Sh3gl3/Ac	7
GO:0007368	GO:0007368	determination of left/right symmetry	5/389	131/23328	0.068648351	0.16175	0.10285	Tbx1/Sox	5
GO:0060041	GO:0060041	retina development in camera-type eye	6/389	172/23328	0.068841657	0.16193	0.10297	Thy1/Ptn/	6
GO:0000289	GO:0000289	nuclear-transcribed mRNA poly(A) tail shortening	2/389	26/23328	0.06932075	0.16193	0.10297	Btg2/Zfp3	2
GO:0003084	GO:0003084	positive regulation of systemic arterial blood pressure	2/389	26/23328	0.06932075	0.16193	0.10297	Nr2f2/Eng	2
GO:0006195	GO:0006195	purine nucleotide catabolic process	2/389	26/23328	0.06932075	0.16193	0.10297	Nudt4/Pde	2
GO:0030204	GO:0030204	chondroitin sulfate metabolic process	2/389	26/23328	0.06932075	0.16193	0.10297	Bgn/Dcn	2
GO:0030325	GO:0030325	adrenal gland development	2/389	26/23328	0.06932075	0.16193	0.10297	Cdkn1c/Ly	2
GO:0034104	GO:0034104	negative regulation of tissue remodeling	2/389	26/23328	0.06932075	0.16193	0.10297	Cd38/Sfrp	2
GO:0043171	GO:0043171	peptide catabolic process	2/389	26/23328	0.06932075	0.16193	0.10297	Ece1/Cpe	2
GO:0060765	GO:0060765	regulation of androgen receptor signaling pathway	2/389	26/23328	0.06932075	0.16193	0.10297	Dab2/Sfrp	2
GO:0070168	GO:0070168	negative regulation of biomineral tissue development	2/389	26/23328	0.06932075	0.16193	0.10297	Srgn/Gas	2
GO:0110150	GO:0110150	negative regulation of biomineralization	2/389	26/23328	0.06932075	0.16193	0.10297	Srgn/Gas	2
GO:1904893	GO:1904893	negative regulation of receptor signaling pathway via S	2/389	26/23328	0.06932075	0.16193	0.10297	Socs3/Ca	2
GO:2000311	GO:2000311	regulation of AMPA receptor activity	2/389	26/23328	0.06932075	0.16193	0.10297	Reln/Adrb	2
GO:2000637	GO:2000637	positive regulation of gene silencing by miRNA	2/389	26/23328	0.06932075	0.16193	0.10297	Bmp4/Zfp	2
GO:2000738	GO:2000738	positive regulation of stem cell differentiation	2/389	26/23328	0.06932075	0.16193	0.10297	Ptn/Bmp4	2
GO:0003170	GO:0003170	heart valve development	3/389	57/23328	0.069647211	0.16195	0.10298	Gja5/Bmp	3
GO:0007585	GO:0007585	respiratory gaseous exchange by respiratory system	3/389	57/23328	0.069647211	0.16195	0.10298	Flt4/Tcf15	3
GO:0010463	GO:0010463	mesenchymal cell proliferation	3/389	57/23328	0.069647211	0.16195	0.10298	Ptn/Tbx1/	3
GO:0019748	GO:0019748	secondary metabolic process	3/389	57/23328	0.069647211	0.16195	0.10298	Pam/Cyp1	3
GO:0035567	GO:0035567	non-canonical Wnt signaling pathway	3/389	57/23328	0.069647211	0.16195	0.10298	Sfrp2/Dab	3
GO:0046622	GO:0046622	positive regulation of organ growth	3/389	57/23328	0.069647211	0.16195	0.10298	Slc25a4/F	3
GO:0060761	GO:0060761	negative regulation of response to cytokine stimulus	3/389	57/23328	0.069647211	0.16195	0.10298	Ptprf/Cav	3
GO:2000648	GO:2000648	positive regulation of stem cell proliferation	3/389	57/23328	0.069647211	0.16195	0.10298	Bmp4/Gja	3
GO:0043506	GO:0043506	regulation of JUN kinase activity	4/389	93/23328	0.070185111	0.16311	0.10372	Sfrp2/Dab	4
GO:0035264	GO:0035264	multicellular organism growth	7/389	216/23328	0.070839292	0.16454	0.10462	Lgmn/Adr	7
GO:1901654	GO:1901654	response to ketone	6/389	174/23328	0.071913367	0.16694	0.10615	Bmp4/Fbx	6
GO:0007623	GO:0007623	circadian rhythm	7/389	217/23328	0.072210739	0.16744	0.10647	Id3/Ass1/	7
GO:0017038	GO:0017038	protein import	7/389	217/23328	0.072210739	0.16744	0.10647	Cd36/Bmp	7
GO:0001736	GO:0001736	establishment of planar polarity	3/389	58/23328	0.072577349	0.16781	0.1067	Sfrp2/Dab	3
GO:0001754	GO:0001754	eye photoreceptor cell differentiation	3/389	58/23328	0.072577349	0.16781	0.1067	Thy1/Ptn/	3
GO:0010517	GO:0010517	regulation of phospholipase activity	3/389	58/23328	0.072577349	0.16781	0.1067	Flt1/Anxa	3
GO:0035690	GO:0035690	cellular response to drug	3/389	58/23328	0.072577349	0.16781	0.1067	Prnp/Gas	3
GO:0048016	GO:0048016	inositol phosphate-mediated signaling	3/389	58/23328	0.072577349	0.16781	0.1067	Prnp/Pdgf	3
GO:0002478	GO:0002478	antigen processing and presentation of exogenous pep	2/389	27/23328	0.07406622	0.16962	0.10785	B2m/H2-K	2
GO:0006882	GO:0006882	cellular zinc ion homeostasis	2/389	27/23328	0.07406622	0.16962	0.10785	Mt1/Mt2	2
GO:0010742	GO:0010742	macrophage derived foam cell differentiation	2/389	27/23328	0.07406622	0.16962	0.10785	Cd36/Lpl	2
GO:0014829	GO:0014829	vascular associated smooth muscle contraction	2/389	27/23328	0.07406622	0.16962	0.10785	Cd38/Con	2
GO:0033688	GO:0033688	regulation of osteoblast proliferation	2/389	27/23328	0.07406622	0.16962	0.10785	Sfrp1/Fbln	2
GO:0035809	GO:0035809	regulation of urine volume	2/389	27/23328	0.07406622	0.16962	0.10785	Adrb2/Adr	2
GO:0042044	GO:0042044	fluid transport	2/389	27/23328	0.07406622	0.16962	0.10785	Aqp1/Aqp	2
GO:0045780	GO:0045780	positive regulation of bone resorption	2/389	27/23328	0.07406622	0.16962	0.10785	Spp1/Trf	2
GO:0046852	GO:0046852	positive regulation of bone remodeling	2/389	27/23328	0.07406622	0.16962	0.10785	Spp1/Trf	2
GO:0046885	GO:0046885	regulation of hormone biosynthetic process	2/389	27/23328	0.07406622	0.16962	0.10785	Dab2/Egr	2
GO:0051767	GO:0051767	nitric-oxide synthase biosynthetic process	2/389	27/23328	0.07406622	0.16962	0.10785	Akap12/Il3	2
GO:0051769	GO:0051769	regulation of nitric-oxide synthase biosynthetic process	2/389	27/23328	0.07406622	0.16962	0.10785	Akap12/Il3	2
GO:0060148	GO:0060148	positive regulation of posttranscriptional gene silencing	2/389	27/23328	0.07406622	0.16962	0.10785	Bmp4/Zfp	2
GO:0061298	GO:0061298	retina vasculature development in camera-type eye	2/389	27/23328	0.07406622	0.16962	0.10785	Pdgfb/Arh	2
GO:0071677	GO:0071677	positive regulation of mononuclear cell migration	2/389	27/23328	0.07406622	0.16962	0.10785	Lgmn/Cxc	2
GO:1904994	GO:1904994	regulation of leukocyte adhesion to vascular endothelia	2/389	27/23328	0.07406622	0.16962	0.10785	Cxcl12/Se	2
GO:2000727	GO:2000727	positive regulation of cardiac muscle cell differentiatio	2/389	27/23328	0.07406622	0.16962	0.10785	Bmp4/Slc	2
GO:2001243	GO:2001243	negative regulation of intrinsic apoptotic signaling path	4/389	95/23328	0.074626062	0.1708	0.10861	Cxcl12/Hs	4
GO:0007164	GO:0007164	establishment of tissue polarity	3/389	59/23328	0.075561795	0.17236	0.1096	Sfrp2/Dab	3
GO:0030042	GO:0030042	actin filament depolymerization	3/389	59/23328	0.075561795	0.17236	0.1096	Dstn/Tpm	3
GO:0042551	GO:0042551	neuron maturation	3/389	59/23328	0.075561795	0.17236	0.1096	Cdkn1c/A	3
GO:0060193	GO:0060193	positive regulation of lipase activity	3/389	59/23328	0.075561795	0.17236	0.1096	Flt1/Gpihb	3
GO:0060443	GO:0060443	mammary gland morphogenesis	3/389	59/23328	0.075561795	0.17236	0.1096	Igfbp5/Bm	3
GO:0071156	GO:0071156	regulation of cell cycle arrest	3/389	59/23328	0.075561795	0.17236	0.1096	Rgcc/Id2/	3
GO:0030177	GO:0030177	positive regulation of Wnt signaling pathway	5/389	135/23328	0.075912071	0.17306	0.11005	Sfrp2/Cav	5
GO:0031016	GO:0031016	pancreas development	4/389	96/23328	0.076898678	0.17522	0.11141	Bmp4/Pro	4
GO:1901990	GO:1901990	regulation of mitotic cell cycle phase transition	8/389	265/23328	0.07719708	0.1758	0.11178	Rgcc/Cdk	8
GO:0097553	GO:0097553	calcium ion transmembrane import into cytosol	5/389	136/23328	0.077790637	0.17703	0.11257	Thy1/Prnp	5
GO:1903039	GO:1903039	positive regulation of leukocyte cell-cell adhesion	7/389	221/23328	0.07785332	0.17703	0.11257	Thy1/Cav	7
GO:0006909	GO:0006909	phagocytosis	10/389	358/23328	0.078547089	0.17703	0.11257	Lbp/Cd36	10
GO:0090183	GO:0090183	regulation of kidney development	3/389	60/23328	0.078599712	0.17703	0.11257	Bmp4/Pd	3
GO:1901016	GO:1901016	regulation of potassium ion transmembrane transporte	3/389	60/23328	0.078599712	0.17703	0.11257	Ank3/Cav	3
GO:2001238	GO:2001238	positive regulation of extrinsic apoptotic signaling path	3/389	60/23328	0.078599712	0.17703	0.11257	Timp3/Ca	3
GO:0002089	GO:0002089	lens morphogenesis in camera-type eye	2/389	28/23328	0.07891236	0.17703	0.11257	Bmp4/Pro	2
GO:0006026	GO:0006026	aminoglycan catabolic process	2/389	28/23328	0.07891236	0.17703	0.11257	Lyve1/Pgl	2
GO:0010458	GO:0010458	exit from mitosis	2/389	28/23328	0.07891236	0.17703	0.11257	Rgcc/Cdk	2
GO:0010738	GO:0010738	regulation of protein kinase A signaling	2/389	28/23328	0.07891236	0.17703	0.11257	Adrb2/Aka	2
GO:0019835	GO:0019835	cytolysis	2/389	28/23328	0.07891236	0.17703	0.11257	Lbp/Fgl2	2
GO:0030224	GO:0030224	monocyte differentiation	2/389	28/23328	0.07891236	0.17703	0.11257	Bmp4/Jun	2
GO:0030878	GO:0030878	thyroid gland development	2/389	28/23328	0.07891236	0.17703	0.11257	Tbx1/Duo	2
GO:0034311	GO:0034311	diol metabolic process	2/389	28/23328	0.07891236	0.17703	0.11257	Plpp3/Plp	2
GO:0055069	GO:0055069	zinc ion homeostasis	2/389	28/23328	0.07891236	0.17703	0.11257	Mt1/Mt2	2
GO:0060039	GO:0060039	pericardium development	2/389	28/23328	0.07891236	0.17703	0.11257	Pdpn/Vca	2
GO:0060384	GO:0060384	innervation	2/389	28/23328	0.07891236	0.17703	0.11257	Serpine2/	2
GO:0060390	GO:0060390	regulation of SMAD protein signal transduction	2/389	28/23328	0.07891236	0.17703	0.11257	Bmp4/Dal	2
GO:0060445	GO:0060445	branching involved in salivary gland morphogenesis	2/389	28/23328	0.07891236	0.17703	0.11257	Pdgfb/Tgr	2
GO:0061099	GO:0061099	negative regulation of protein tyrosine kinase activity	2/389	28/23328	0.07891236	0.17703	0.11257	Thy1/Cav	2
GO:0086005	GO:0086005	ventricular cardiac muscle cell action potential	2/389	28/23328	0.07891236	0.17703	0.11257	Gja5/Cav	2
GO:0090077	GO:0090077	foam cell differentiation	2/389	28/23328	0.07891236	0.17703	0.11257	Cd36/Lpl	2
GO:0098698	GO:0098698	postsynaptic specialization assembly	2/389	28/23328	0.07891236	0.17703	0.11257	Reln/Gap	2

GO:1900273	GO:1900273	positive regulation of long-term synaptic potentiation	2/389	28/23328	0.07891236	0.17703	0.11257	Reln/Lgm	2
GO:1903131	GO:1903131	mononuclear cell differentiation	2/389	28/23328	0.07891236	0.17703	0.11257	Bmp4/Jun	2
GO:1903671	GO:1903671	negative regulation of sprouting angiogenesis	2/389	28/23328	0.07891236	0.17703	0.11257	Klf2/Mmrn	2
GO:2000178	GO:2000178	negative regulation of neural precursor cell proliferatio	2/389	28/23328	0.07891236	0.17703	0.11257	Ptn/Btg2	2
GO:0045069	GO:0045069	regulation of viral genome replication	4/389	97/23328	0.079205728	0.17759	0.11292	Rsad2/Oa	4
GO:0048588	GO:0048588	developmental cell growth	8/389	267/23328	0.079817396	0.17886	0.11373	Cxcl12/Rg	8
GO:0007163	GO:0007163	establishment or maintenance of cell polarity	7/389	223/23328	0.080768563	0.1809	0.11503	Pard6g/Tc	7
GO:0071482	GO:0071482	cellular response to light stimulus	4/389	98/23328	0.081546953	0.18246	0.11602	Mfap4/Aq	4
GO:0048675	GO:0048675	axon extension	5/389	138/23328	0.081622311	0.18246	0.11602	Cxcl12/Nr	5
GO:0071887	GO:0071887	leukocyte apoptotic process	5/389	138/23328	0.081622311	0.18246	0.11602	Cxcl12/Br	5
GO:0042698	GO:0042698	ovulation cycle	3/389	61/23328	0.081690251	0.18246	0.11602	Ptn/Egr1/	3
GO:0060986	GO:0060986	endocrine hormone secretion	3/389	61/23328	0.081690251	0.18246	0.11602	Aqp1/Dab	3
GO:0016125	GO:0016125	sterol metabolic process	5/389	139/23328	0.083575215	0.18531	0.11783	Ephx2/Cy	5
GO:0055007	GO:0055007	cardiac muscle cell differentiation	5/389	139/23328	0.083575215	0.18531	0.11783	Bmp4/Rgs	5
GO:0003416	GO:0003416	endochondral bone growth	2/389	29/23328	0.083854515	0.18531	0.11783	Stc1/Com	2
GO:0030511	GO:0030511	positive regulation of transforming growth factor beta r	2/389	29/23328	0.083854515	0.18531	0.11783	Lrg1/Dab2	2
GO:0031116	GO:0031116	positive regulation of microtubule polymerization	2/389	29/23328	0.083854515	0.18531	0.11783	Cav1/Gda	2
GO:0031295	GO:0031295	T cell costimulation	2/389	29/23328	0.083854515	0.18531	0.11783	Cav1/Lgal	2
GO:0043032	GO:0043032	positive regulation of macrophage activation	2/389	29/23328	0.083854515	0.18531	0.11783	Lbp/Il33	2
GO:0045589	GO:0045589	regulation of regulatory T cell differentiation	2/389	29/23328	0.083854515	0.18531	0.11783	Il2rg/Ctla2	2
GO:0050858	GO:0050858	negative regulation of antigen receptor-mediated signa	2/389	29/23328	0.083854515	0.18531	0.11783	Thy1/Prnp	2
GO:0061311	GO:0061311	cell surface receptor signaling pathway involved in hea	2/389	29/23328	0.083854515	0.18531	0.11783	Sox17/Bm	2
GO:0071353	GO:0071353	cellular response to interleukin-4	2/389	29/23328	0.083854515	0.18531	0.11783	Mrc1/Tuba	2
GO:0090208	GO:0090208	positive regulation of triglyceride metabolic process	2/389	29/23328	0.083854515	0.18531	0.11783	Cd36/Sca	2
GO:0090314	GO:0090314	positive regulation of protein targeting to membrane	2/389	29/23328	0.083854515	0.18531	0.11783	Prnp/Ank3	2
GO:0097345	GO:0097345	mitochondrial outer membrane permeabilization	2/389	29/23328	0.083854515	0.18531	0.11783	Ier3/Bok	2
GO:0099622	GO:0099622	cardiac muscle cell membrane repolarization	2/389	29/23328	0.083854515	0.18531	0.11783	Gja5/Gja1	2
GO:0150117	GO:0150117	positive regulation of cell-substrate junction organizati	2/389	29/23328	0.083854515	0.18531	0.11783	Thy1/Tek	2
GO:1901185	GO:1901185	negative regulation of ERBB signaling pathway	2/389	29/23328	0.083854515	0.18531	0.11783	Ptprf/Lgm	2
GO:1903846	GO:1903846	positive regulation of cellular response to transforming	2/389	29/23328	0.083854515	0.18531	0.11783	Lrg1/Dab2	2
GO:0021761	GO:0021761	limbic system development	4/389	99/23328	0.083922083	0.18531	0.11783	Reln/Nrp2	4
GO:0030101	GO:0030101	natural killer cell activation	4/389	99/23328	0.083922083	0.18531	0.11783	Prdx1/Gas	4
GO:0097061	GO:0097061	dendritic spine organization	4/389	99/23328	0.083922083	0.18531	0.11783	Reln/Prnp	4
GO:1990778	GO:1990778	protein localization to cell periphery	10/389	363/23328	0.084248244	0.18593	0.11823	Prnp/Tme	10
GO:0006081	GO:0006081	cellular aldehyde metabolic process	3/389	62/23328	0.084832544	0.18682	0.11879	Aldoa/Dab	3
GO:0031018	GO:0031018	endocrine pancreas development	3/389	62/23328	0.084832544	0.18682	0.11879	Bmp4/Clu	3
GO:0060350	GO:0060350	endochondral bone morphogenesis	3/389	62/23328	0.084832544	0.18682	0.11879	Bmp4/Stc	3
GO:0061337	GO:0061337	cardiac conduction	3/389	62/23328	0.084832544	0.18682	0.11879	Gja5/Cav1	3
GO:0009855	GO:0009855	determination of bilateral symmetry	5/389	140/23328	0.085552686	0.1883	0.11973	Tbx1/Sox1	5
GO:0010771	GO:0010771	negative regulation of cell morphogenesis involved in	4/389	100/23328	0.086330841	0.18981	0.12069	Thy1/Id1/	4
GO:0050795	GO:0050795	regulation of behavior	4/389	100/23328	0.086330841	0.18981	0.12069	Reln/Apoe	4
GO:0048167	GO:0048167	regulation of synaptic plasticity	10/389	365/23328	0.086597713	0.19029	0.121	Reln/Ptn/S	10
GO:0035966	GO:0035966	response to topologically incorrect protein	5/389	141/23328	0.087554606	0.19229	0.12227	Hspb1/Clu	5
GO:0001947	GO:0001947	heart looping	3/389	63/23328	0.088025709	0.19301	0.12273	Sox17/En	3
GO:0030104	GO:0030104	water homeostasis	3/389	63/23328	0.088025709	0.19301	0.12273	Aqp1/Aqp	3
GO:0045661	GO:0045661	regulation of myoblast differentiation	3/389	63/23328	0.088025709	0.19301	0.12273	Id3/Bmp4/	3
GO:0030534	GO:0030534	adult behavior	6/389	184/23328	0.088430868	0.19334	0.12294	Hoxd9/Cx	6
GO:0032388	GO:0032388	positive regulation of intracellular transport	6/389	184/23328	0.088430868	0.19334	0.12294	Prnp/Ank3	6
GO:0032755	GO:0032755	positive regulation of interleukin-6 production	4/389	101/23328	0.088772937	0.19334	0.12294	Lbp/Cd36/	4
GO:2001237	GO:2001237	negative regulation of extrinsic apoptotic signaling path	4/389	101/23328	0.088772937	0.19334	0.12294	Bmp4/Sfrp	4
GO:0002724	GO:0002724	regulation of T cell cytokine production	2/389	30/23328	0.088888153	0.19334	0.12294	Rsad2/B2	2
GO:0002825	GO:0002825	regulation of T-helper 1 type immune response	2/389	30/23328	0.088888153	0.19334	0.12294	Il33/Anxa1	2
GO:0010863	GO:0010863	positive regulation of phospholipase C activity	2/389	30/23328	0.088888153	0.19334	0.12294	Flt1/Htr2b	2
GO:0010955	GO:0010955	negative regulation of protein processing	2/389	30/23328	0.088888153	0.19334	0.12294	Serpine2/	2
GO:0021602	GO:0021602	cranial nerve morphogenesis	2/389	30/23328	0.088888153	0.19334	0.12294	Tbx1/Nrp2	2
GO:0035162	GO:0035162	embryonic hemopoiesis	2/389	30/23328	0.088888153	0.19334	0.12294	Kitl/Lmo2	2
GO:0043372	GO:0043372	positive regulation of CD4-positive, alpha-beta T cell d	2/389	30/23328	0.088888153	0.19334	0.12294	Il2rg/Anxa	2
GO:0061082	GO:0061082	myeloid leukocyte cytokine production	2/389	30/23328	0.088888153	0.19334	0.12294	Cd36/Gas	2
GO:0071711	GO:0071711	basement membrane organization	2/389	30/23328	0.088888153	0.19334	0.12294	Cav1/Flrt2	2
GO:1903318	GO:1903318	negative regulation of protein maturation	2/389	30/23328	0.088888153	0.19334	0.12294	Serpine2/	2
GO:1903579	GO:1903579	negative regulation of ATP metabolic process	2/389	30/23328	0.088888153	0.19334	0.12294	Ier3/Pde2	2
GO:0071216	GO:0071216	cellular response to biotic stimulus	9/389	320/23328	0.089049393	0.19359	0.1231	Lbp/Cd36/	9
GO:0002705	GO:0002705	positive regulation of leukocyte mediated immunity	6/389	185/23328	0.090187872	0.19596	0.1246	H2-Q6/Rs	6
GO:0050848	GO:0050848	regulation of calcium-mediated signaling	4/389	102/23328	0.091248072	0.19799	0.1259	Prnp/Cdh1	4
GO:0006879	GO:0006879	cellular iron ion homeostasis	3/389	64/23328	0.091268852	0.19799	0.1259	Cp/Lcn2/T	3
GO:1903510	GO:1903510	mucopolysaccharide metabolic process	3/389	64/23328	0.091268852	0.19799	0.1259	Pdgfb/Bgr	3
GO:0035725	GO:0035725	sodium ion transmembrane transport	5/389	143/23328	0.091631305	0.19867	0.12633	Fxyd6/Ank	5
GO:0046328	GO:0046328	regulation of JNK cascade	6/389	186/23328	0.091963796	0.19929	0.12672	Flt4/Sfrp2	6
GO:0090263	GO:0090263	positive regulation of canonical Wnt signaling pathway	4/389	103/23328	0.09375594	0.20189	0.12838	Sfrp2/Cav	4
GO:0002063	GO:0002063	chondrocyte development	2/389	31/23328	0.094008868	0.20189	0.12838	Sfrp2/Con	2
GO:0008299	GO:0008299	isoprenoid biosynthetic process	2/389	31/23328	0.094008868	0.20189	0.12838	Rbp1/Cyp	2
GO:0031294	GO:0031294	lymphocyte costimulation	2/389	31/23328	0.094008868	0.20189	0.12838	Cav1/Lgal	2
GO:0042481	GO:0042481	regulation of odontogenesis	2/389	31/23328	0.094008868	0.20189	0.12838	Cd34/Bmp	2
GO:0045662	GO:0045662	negative regulation of myoblast differentiation	2/389	31/23328	0.094008868	0.20189	0.12838	Id3/Bmp4	2
GO:0045932	GO:0045932	negative regulation of muscle contraction	2/389	31/23328	0.094008868	0.20189	0.12838	Adrb2/Cal	2
GO:0060074	GO:0060074	synapse maturation	2/389	31/23328	0.094008868	0.20189	0.12838	Reln/Arhg	2
GO:0061036	GO:0061036	positive regulation of cartilage development	2/389	31/23328	0.094008868	0.20189	0.12838	Bmp4/Lox	2
GO:0061037	GO:0061037	negative regulation of cartilage development	2/389	31/23328	0.094008868	0.20189	0.12838	Bmp4/Efe	2
GO:0070670	GO:0070670	response to interleukin-4	2/389	31/23328	0.094008868	0.20189	0.12838	Mrc1/Tuba	2
GO:0072574	GO:0072574	hepatocyte proliferation	2/389	31/23328	0.094008868	0.20189	0.12838	Ptn/Prox1	2
GO:0072575	GO:0072575	epithelial cell proliferation involved in liver morphogene	2/389	31/23328	0.094008868	0.20189	0.12838	Ptn/Prox1	2
GO:0086010	GO:0086010	membrane depolarization during action potential	2/389	31/23328	0.094008868	0.20189	0.12838	Gja5/Ank3	2
GO:1900117	GO:1900117	regulation of execution phase of apoptosis	2/389	31/23328	0.094008868	0.20189	0.12838	Ptgis/Bok	2
GO:1905314	GO:1905314	semi-lunar valve development	2/389	31/23328	0.094008868	0.20189	0.12838	Gja5/Bmp	2
GO:2001014	GO:2001014	regulation of skeletal muscle cell differentiation	2/389	31/23328	0.094008868	0.20189	0.12838	Tbx1/Cyp2	2
GO:0046503	GO:0046503	glycerolipid catabolic process	3/389	65/23328	0.094561066	0.20297	0.12906	Mgll/Scarb	3
GO:0010770	GO:0010770	positive regulation of cell morphogenesis involved in d	6/389	188/23328	0.095572142	0.20503	0.13038	Reln/Ptprf	6
GO:0072073	GO:0072073	kidney epithelium development	5/389	145/23328	0.095804271	0.20542	0.13062	Bmp4/Pdg	5
GO:0015844	GO:0015844	monoamine transport	4/389	104/23328	0.096296224	0.20637	0.13122	Cxcl12/Sr	4
GO:0006644	GO:0006644	phospholipid metabolic process	10/389	374/23328	0.097658071	0.20907	0.13294	Flt1/Socs3	10
GO:0016311	GO:0016311	dephosphorylation	10/389	374/23328	0.097658071	0.20907	0.13294	Ptprb/Eph	10
GO:0001707	GO:0001707	mesoderm formation	3/389	66/23328	0.097901433	0.2092	0.13303	Bmp4/Sfrp	3
GO:0050433	GO:0050433	regulation of catecholamine secretion	3/389	66/23328	0.097901433	0.2092	0.13303	Cxcl12/Sr	3
GO:0006814	GO:0006814	sodium ion transport	7/389	234/23328	0.097913934	0.2092	0.13303	Fxyd6/Ser	7
GO:0045598	GO:0045598	regulation of fat cell differentiation	5/389	146/23328	0.097926505	0.2092	0.13303	Sfrp2/Zfp3	5
GO:0031645	GO:0031645	negative regulation of nervous system process	2/389	32/23328	0.099212376	0.21129	0.13435	Jam2/Klk8	2
GO:0032692	GO:0032692	negative regulation of interleukin-1 production	2/389	32/23328	0.099212376	0.21129	0.13435	Serpinb1a	2
GO:0050482	GO:0050482	arachidonic acid secretion	2/389	32/23328	0.099212376	0.21129	0.13435	Nmb/Anxa	2
GO:0051491	GO:0051491	positive regulation of filopodium assembly	2/389	32/23328	0.099212376	0.21129	0.13435	Gpm6a/Dp	2
GO:1902110	GO:1902110	positive regulation of mitochondrial membrane permea	2/389	32/23328	0.099212376	0.21129	0.13435	Ier3/Bok	2
GO:1903963	GO:1903963	arachidonate transport	2/389	32/23328	0.099212376	0.21129	0.13435	Nmb/Anxa	2
GO:0006470	GO:0006470	protein dephosphorylation	7/389	235/23328	0.099565023	0.21193	0.13476	Ptprb/Ptpr	7
GO:0007416	GO:0007416	synapse assembly	6/389	191/23328	0.101124815	0.2147	0.13652	Reln/Gap4	6
GO:0051783	GO:0051783	regulation of nuclear division	6/389	191/23328	0.101124815	0.2147	0.13652	Rgcc/Bmp	6
GO:0045930	GO:0045930	negative regulation of mitotic cell cycle	7/389	236/23328	0.101231344	0.2147	0.13652	Rgcc/Bmp	7
GO:0033209	GO:0033209	tumor necrosis factor-mediated signaling pathway	3/389	67/23328	0.101289025	0.2147	0.13652	Actn4/Gas	3
GO:0060425	GO:0060425	lung morphogenesis	3/389	67/23328	0.101289025	0.2147	0.13652	Tnc/Id1/Br	3
GO:0070613	GO:0070613	regulation of protein processing	3/389	67/23328	0.101289025	0.2147	0.13652	Serpine2/	3
GO:0086003	GO:0086003	cardiac muscle cell contraction	3/389	67/23328	0.101289025	0.2147	0.13652	Gja5/Cav1	3
GO:2000242	GO:2000242	negative regulation of reproductive process	3/389	67/23328	0.101289025	0.2147	0.13652	Bmp4/Gja	3
GO:0106027	GO:0106027	neuron projection organization	4/389	106/23328	0.101472733	0.21486	0.13663	Reln/Prnp	4
GO:0110110	GO:0110110	positive regulation of animal organ morphogenesis	4/389	106/23328	0.101472733	0.21486	0.13663	Ptn/Cd34/	4
GO:0000186	GO:0000186	activation of MAPKK activity	2/389	33/23328	0.104494507	0.21989	0.13982	Bmp4/Gad	2
GO:0010667	GO:0010667	negative regulation of cardiac muscle cell apoptotic pr	2/389	33/23328	0.104494507	0.21989	0.13982	Sfrp2/Slc2	2
GO:0022617	GO:0022617	extracellular matrix disassembly	2/389	33/23328	0.104494507	0.21989	0.13982	Eng/Pdpn	2
GO:0033081	GO:0033081	regulation of T cell differentiation in thymus	2/389	33/23328	0.104494507	0.21989	0.13982	Bmp4/Il2r	2
GO:0034260	GO:0034260	negative regulation of GTPase activity	2/389	33/23328	0.104494507	0.21989	0.13982	F11r/Peca	2
GO:0044818	GO:0044818	mitotic G2/M transition checkpoint	2/389	33/23328	0.104494507	0.21989	0.13982	Ier3/Cdkn	2
GO:0045066	GO:0045066	regulatory T cell differentiation	2/389	33/23328	0.104494507	0.21989	0.13982	Il2rg/Ctla2	2
GO:0048841	GO:0048841	regulation of axon extension involved in axon guidance	2/389	33/23328	0.104494507	0.21989	0.13982	Cxcl12/Se	2
GO:0051354	GO:0051354	negative regulation of oxidoreductase activity	2/389	33/23328	0.104494507	0.21989	0.13982	Cav1/Eng	2
GO:0060706	GO:0060706	cell differentiation involved in embryonic placenta deve	2/389	33/23328	0.104494507	0.21989	0.13982	Nr2f2/Soc	2
GO:0060914	GO:0060914	heart formation	2/389	33/23328	0.104494507	0.21989	0.13982	Tbx1/Bmp	2
GO:0072576	GO:0072576	liver morphogenesis	2/389	33/23328	0.104494507	0.21989	0.13982	Ptn/Prox1	2
GO:0048641	GO:0048641	regulation of skeletal muscle tissue development	3/389	68/23328	0.104722907	0.22026	0.14006	Tbx1/Adrb	3
GO:0009913	GO:0009913	epidermal cell differentiation	6/389	193/23328	0.104919111	0.22056	0.14025	Bmp4/Ptg	6
GO:0009100	GO:0009100	glycoprotein metabolic process	9/389	332/23328	0.105420422	0.2215	0.14084	Pcsk6/Cyt	9
GO:0002366	GO:0002366	leukocyte activation involved in immune response	8/389	285/23328	0.105748447	0.22207	0.14121	Lbp/Fgl2/	8
GO:0001824	GO:0001824	blastocyst development	5/389	150/23328	0.106650246	0.22373	0.14227	Sox17/Ptp	5

GO:0061025	GO:0061025	membrane fusion	5/389	150/23328	0.106650246	0.22373	0.14227	Cd9/Anxa	5
GO:0045639	GO:0045639	positive regulation of myeloid cell differentiation	4/389	108/23328	0.1067749	0.22388	0.14236	Id2/Kitl/Ju	4
GO:0046942	GO:0046942	carboxylic acid transport	9/389	333/23328	0.106857005	0.22394	0.1424	Fabp4/Cd	9
GO:0016055	GO:0016055	Wnt signaling pathway	11/389	430/23328	0.10722932	0.2246	0.14282	Sox17/Tm	11
GO:0032677	GO:0032677	regulation of interleukin-8 production	3/389	69/23328	0.108202134	0.22641	0.14397	Lbp/Cd2/A	3
GO:0061371	GO:0061371	determination of heart left/right asymmetry	3/389	69/23328	0.108202134	0.22641	0.14397	Sox17/En	3
GO:0050830	GO:0050830	defense response to Gram-positive bacterium	5/389	151/23328	0.108889075	0.22764	0.14475	Lbp/Cd36/	5
GO:0001959	GO:0001959	regulation of cytokine-mediated signaling pathway	4/389	109/23328	0.10947223	0.22764	0.14475	Ptprf/Cav1	4
GO:0051897	GO:0051897	positive regulation of protein kinase B signaling	4/389	109/23328	0.10947223	0.22764	0.14475	Igfbp5/En	4
GO:1900006	GO:1900006	positive regulation of dendrite development	4/389	109/23328	0.10947223	0.22764	0.14475	Reln/Ptn/F	4
GO:0022411	GO:0022411	cellular component disassembly	10/389	383/23328	0.109512978	0.22764	0.14475	Sh3gl3/Ac	10
GO:0002449	GO:0002449	lymphocyte mediated immunity	11/389	432/23328	0.109769851	0.22764	0.14475	Fgl2/H2-Q	11
GO:0198738	GO:0198738	cell-cell signaling by wnt	11/389	432/23328	0.109769851	0.22764	0.14475	Sox17/Tm	11
GO:0019884	GO:0019884	antigen processing and presentation of exogenous ant	2/389	34/23328	0.109851212	0.22764	0.14475	B2m/H2-K	2
GO:0033032	GO:0033032	regulation of myeloid cell apoptotic process	2/389	34/23328	0.109851212	0.22764	0.14475	Kitl/Anxa1	2
GO:0048048	GO:0048048	embryonic eye morphogenesis	2/389	34/23328	0.109851212	0.22764	0.14475	Prox1/Mfa	2
GO:0048710	GO:0048710	regulation of astrocyte differentiation	2/389	34/23328	0.109851212	0.22764	0.14475	Serpine2/I	2
GO:0071634	GO:0071634	regulation of transforming growth factor beta productio	2/389	34/23328	0.109851212	0.22764	0.14475	Cd34/Cd2	2
GO:0090162	GO:0090162	establishment of epithelial cell polarity	2/389	34/23328	0.109851212	0.22764	0.14475	Tcf15/Gja	2
GO:0090181	GO:0090181	regulation of cholesterol metabolic process	2/389	34/23328	0.109851212	0.22764	0.14475	Ephx2/Ap	2
GO:0099068	GO:0099068	postsynapse assembly	2/389	34/23328	0.109851212	0.22764	0.14475	Reln/Gap4	2
GO:0120255	GO:0120255	olefinic compound biosynthetic process	2/389	34/23328	0.109851212	0.22764	0.14475	Dab2/Egr1	2
GO:1900274	GO:1900274	regulation of phospholipase C activity	2/389	34/23328	0.109851212	0.22764	0.14475	Flt1/Htr2b	2
GO:1902686	GO:1902686	mitochondrial outer membrane permeabilization involv	2/389	34/23328	0.109851212	0.22764	0.14475	Ier3/Bok	2
GO:2000406	GO:2000406	positive regulation of T cell migration	2/389	34/23328	0.109851212	0.22764	0.14475	Ccl21a/Cx	2
GO:0070665	GO:0070665	positive regulation of leukocyte proliferation	5/389	152/23328	0.111150722	0.23021	0.14638	Vcam1/Cc	5
GO:0001541	GO:0001541	ovarian follicle development	3/389	70/23328	0.111725754	0.23058	0.14662	Bmp4/Mm	3
GO:0003143	GO:0003143	embryonic heart tube morphogenesis	3/389	70/23328	0.111725754	0.23058	0.14662	Sox17/En	3
GO:0006766	GO:0006766	vitamin metabolic process	3/389	70/23328	0.111725754	0.23058	0.14662	Pltp/Rbp1	3
GO:0017144	GO:0017144	drug metabolic process	3/389	70/23328	0.111725754	0.23058	0.14662	Cyp4b1/C	3
GO:0045669	GO:0045669	positive regulation of osteoblast differentiation	3/389	70/23328	0.111725754	0.23058	0.14662	Bmp4/Sfrp	3
GO:0048332	GO:0048332	mesoderm morphogenesis	3/389	70/23328	0.111725754	0.23058	0.14662	Bmp4/Sfrp	3
GO:1903317	GO:1903317	regulation of protein maturation	3/389	70/23328	0.111725754	0.23058	0.14662	Serpine2/	3
GO:0002263	GO:0002263	cell activation involved in immune response	8/389	289/23328	0.112077755	0.23086	0.1468	Lbp/Fgl2/	8
GO:0006275	GO:0006275	regulation of DNA replication	4/389	110/23328	0.112199909	0.23086	0.1468	Id3/Pdgfb/	4
GO:0008344	GO:0008344	adult locomotory behavior	4/389	110/23328	0.112199909	0.23086	0.1468	Hoxd9/Cx	4
GO:0022600	GO:0022600	digestive system process	4/389	110/23328	0.112199909	0.23086	0.1468	Cd36/F11	4
GO:0048709	GO:0048709	oligodendrocyte differentiation	4/389	110/23328	0.112199909	0.23086	0.1468	Ptn/Bmp4	4
GO:0120034	GO:0120034	positive regulation of plasma membrane bounded cell	4/389	110/23328	0.112199909	0.23086	0.1468	Cdc42ep3	4
GO:0015849	GO:0015849	organic acid transport	9/389	337/23328	0.112713469	0.23171	0.14734	Fabp4/Cd	9
GO:0050773	GO:0050773	regulation of dendrite development	6/389	197/23328	0.112726566	0.23171	0.14734	Reln/Ptn/F	6
GO:0030183	GO:0030183	B cell differentiation	5/389	153/23328	0.11343501	0.23305	0.14819	Adgrg3/Il2	5
GO:0001843	GO:0001843	neural tube closure	4/389	111/23328	0.114957566	0.23532	0.14963	Bmp4/Sfrp	4
GO:0008637	GO:0008637	apoptotic mitochondrial changes	4/389	111/23328	0.114957566	0.23532	0.14963	Ier3/Slc25	4
GO:0072009	GO:0072009	nephron epithelium development	4/389	111/23328	0.114957566	0.23532	0.14963	Bmp4/Pdc	4
GO:0010719	GO:0010719	negative regulation of epithelial to mesenchymal transi	2/389	35/23328	0.115278548	0.23532	0.14963	Sfrp2/Sfrp	2
GO:0010837	GO:0010837	regulation of keratinocyte proliferation	2/389	35/23328	0.115278548	0.23532	0.14963	Ctsl/Zfp36	2
GO:0045686	GO:0045686	negative regulation of glial cell differentiation	2/389	35/23328	0.115278548	0.23532	0.14963	Bmp4/Id2	2
GO:0045746	GO:0045746	negative regulation of Notch signaling pathway	2/389	35/23328	0.115278548	0.23532	0.14963	Egfl7/Nrar	2
GO:0046677	GO:0046677	response to antibiotic	2/389	35/23328	0.115278548	0.23532	0.14963	Id1/Crip1	2
GO:0060441	GO:0060441	epithelial tube branching involved in lung morphogene	2/389	35/23328	0.115278548	0.23532	0.14963	Tnc/Bmp4	2
GO:0086091	GO:0086091	regulation of heart rate by cardiac conduction	2/389	35/23328	0.115278548	0.23532	0.14963	Gja5/Cav1	2
GO:0090313	GO:0090313	regulation of protein targeting to membrane	2/389	35/23328	0.115278548	0.23532	0.14963	Prnp/Ank3	2
GO:1905898	GO:1905898	positive regulation of response to endoplasmic reticulu	2/389	35/23328	0.115278548	0.23532	0.14963	Cav1/Bok	2
GO:0042509	GO:0042509	regulation of tyrosine phosphorylation of STAT protein	3/389	71/23328	0.115292811	0.23532	0.14963	Socs3/Ca	3
GO:0002833	GO:0002833	positive regulation of response to biotic stimulus	5/389	154/23328	0.115741754	0.23612	0.15014	Lbp/Irf7/M	5
GO:0007589	GO:0007589	body fluid secretion	4/389	112/23328	0.117744825	0.23997	0.15259	Anxa2/Ca	4
GO:0060606	GO:0060606	tube closure	4/389	112/23328	0.117744825	0.23997	0.15259	Bmp4/Sfrp	4
GO:0007254	GO:0007254	JNK cascade	6/389	200/23328	0.118770592	0.24181	0.15376	Flt4/Sfrp2	6
GO:0050870	GO:0050870	positive regulation of T cell activation	6/389	200/23328	0.118770592	0.24181	0.15376	Thy1/Cav1	6
GO:0007193	GO:0007193	adenylate cyclase-inhibiting G protein-coupled recepto	3/389	72/23328	0.118902342	0.24196	0.15386	Akap12/S	3
GO:0051606	GO:0051606	detection of stimulus	7/389	247/23328	0.120551089	0.24452	0.15548	Lbp/Serpi	7
GO:0030516	GO:0030516	regulation of axon extension	4/389	113/23328	0.120561303	0.24452	0.15548	Cxcl12/Mc	4
GO:0010664	GO:0010664	negative regulation of striated muscle cell apoptotic pr	2/389	36/23328	0.120772688	0.24452	0.15548	Sfrp2/Slc2	2
GO:0031069	GO:0031069	hair follicle morphogenesis	2/389	36/23328	0.120772688	0.24452	0.15548	Igfbp5/Cts	2
GO:0031572	GO:0031572	G2 DNA damage checkpoint	2/389	36/23328	0.120772688	0.24452	0.15548	Ier3/Cdkn	2
GO:0060351	GO:0060351	cartilage development involved in endochondral bone	2/389	36/23328	0.120772688	0.24452	0.15548	Stc1/Com	2
GO:0071398	GO:0071398	cellular response to fatty acid	2/389	36/23328	0.120772688	0.24452	0.15548	Id3/Lpl	2
GO:0071604	GO:0071604	transforming growth factor beta production	2/389	36/23328	0.120772688	0.24452	0.15548	Cd34/Cd2	2
GO:1905209	GO:1905209	positive regulation of cardiocyte differentiation	2/389	36/23328	0.120772688	0.24452	0.15548	Bmp4/Slc	2
GO:2000516	GO:2000516	positive regulation of CD4-positive, alpha-beta T cell a	2/389	36/23328	0.120772688	0.24452	0.15548	Il2rg/Anxa	2
GO:0009566	GO:0009566	fertilization	6/389	201/23328	0.1208206	0.24452	0.15548	Cd9/Nr2f2	6
GO:0060291	GO:0060291	long-term synaptic potentiation	7/389	248/23328	0.122395879	0.24759	0.15743	Reln/Ptn/S	7
GO:0021879	GO:0021879	forebrain neuron differentiation	3/389	73/23328	0.12255338	0.24778	0.15756	B2m/Nrp2	3
GO:1901361	GO:1901361	organic cyclic compound catabolic process	11/389	442/23328	0.122989954	0.24854	0.15804	Ephx2/Vir	11
GO:1901987	GO:1901987	regulation of cell cycle phase transition	8/389	297/23328	0.125338812	0.25291	0.16082	Rgcc/Cdk	8
GO:0002292	GO:0002292	T cell differentiation involved in immune response	3/389	74/23328	0.126244955	0.25291	0.16082	Fgl2/Gadc	3
GO:0006029	GO:0006029	proteoglycan metabolic process	3/389	74/23328	0.126244955	0.25291	0.16082	Cytl1/Bgn	3
GO:0021766	GO:0021766	hippocampus development	3/389	74/23328	0.126244955	0.25291	0.16082	Reln/Prox	3
GO:1901184	GO:1901184	regulation of ERBB signaling pathway	3/389	74/23328	0.126244955	0.25291	0.16082	Ptprf/Lgm	3
GO:2001021	GO:2001021	negative regulation of response to DNA damage stimu	3/389	74/23328	0.126244955	0.25291	0.16082	Cxcl12/Cl	3
GO:0008585	GO:0008585	female gonad development	4/389	115/23328	0.126280356	0.25291	0.16082	Bmp4/Mm	4
GO:0030216	GO:0030216	keratinocyte differentiation	4/389	115/23328	0.126280356	0.25291	0.16082	Ptgs1/Zfp	4
GO:0002218	GO:0002218	activation of innate immune response	2/389	37/23328	0.12632991	0.25291	0.16082	Mndal/Ifi2	2
GO:0002931	GO:0002931	response to ischemia	2/389	37/23328	0.12632991	0.25291	0.16082	Cav1/Egr1	2
GO:0007435	GO:0007435	salivary gland morphogenesis	2/389	37/23328	0.12632991	0.25291	0.16082	Pdgfb/Tgr	2
GO:0009954	GO:0009954	proximal/distal pattern formation	2/389	37/23328	0.12632991	0.25291	0.16082	Hoxd9/Cy	2
GO:0030574	GO:0030574	collagen catabolic process	2/389	37/23328	0.12632991	0.25291	0.16082	Mmp2/Cts	2
GO:0033144	GO:0033144	negative regulation of intracellular steroid hormone rec	2/389	37/23328	0.12632991	0.25291	0.16082	Dab2/Sfrp	2
GO:0035794	GO:0035794	positive regulation of mitochondrial membrane permea	2/389	37/23328	0.12632991	0.25291	0.16082	Ier3/Bok	2
GO:0046329	GO:0046329	negative regulation of JNK cascade	2/389	37/23328	0.12632991	0.25291	0.16082	Sfrp2/Sfrp	2
GO:0051482	GO:0051482	positive regulation of cytosolic calcium ion concentrati	2/389	37/23328	0.12632991	0.25291	0.16082	Tgm2/S1p	2
GO:0071514	GO:0071514	genetic imprinting	2/389	37/23328	0.12632991	0.25291	0.16082	Cdkn1c/M	2
GO:2000273	GO:2000273	positive regulation of signaling receptor activity	2/389	37/23328	0.12632991	0.25291	0.16082	Reln/Adrb	2
GO:0002700	GO:0002700	regulation of production of molecular mediator of immu	5/389	159/23328	0.127605648	0.25521	0.16228	Cd36/Rsa	5
GO:1903900	GO:1903900	regulation of viral life cycle	5/389	159/23328	0.127605648	0.25521	0.16228	Rsad2/Oa	5
GO:0051952	GO:0051952	regulation of amine transport	4/389	116/23328	0.129182137	0.25824	0.1642	Cxcl12/Rc	4
GO:0021537	GO:0021537	telencephalon development	7/389	252/23328	0.129918764	0.25907	0.16473	Reln/Cxcl	7
GO:0007260	GO:0007260	tyrosine phosphorylation of STAT protein	3/389	75/23328	0.129976093	0.25907	0.16473	Socs3/Ca	3
GO:0032637	GO:0032637	interleukin-8 production	3/389	75/23328	0.129976093	0.25907	0.16473	Lbp/Cd2/A	3
GO:0042306	GO:0042306	regulation of protein import into nucleus	3/389	75/23328	0.129976093	0.25907	0.16473	Cd36/Bmp	3
GO:0048747	GO:0048747	muscle fiber development	3/389	75/23328	0.129976093	0.25907	0.16473	Bmp4/Lox	3
GO:0007088	GO:0007088	regulation of mitotic nuclear division	5/389	160/23328	0.130043066	0.25907	0.16473	Rgcc/Bmp	5
GO:0010950	GO:0010950	positive regulation of endopeptidase activity	5/389	160/23328	0.130043066	0.25907	0.16473	Lgmn/Sfrp	5
GO:0071375	GO:0071375	cellular response to peptide hormone stimulus	7/389	253/23328	0.131835028	0.26133	0.16617	Ptprf/Socs	7
GO:0001706	GO:0001706	endoderm formation	2/389	38/23328	0.131946596	0.26133	0.16617	Sox17/Du	2
GO:0010661	GO:0010661	positive regulation of muscle cell apoptotic process	2/389	38/23328	0.131946596	0.26133	0.16617	Fndc1/Fbx	2
GO:0045940	GO:0045940	positive regulation of steroid metabolic process	2/389	38/23328	0.131946596	0.26133	0.16617	Apoe/Dab	2
GO:0046627	GO:0046627	negative regulation of insulin receptor signaling pathwa	2/389	38/23328	0.131946596	0.26133	0.16617	Ptprf/Socs	2
GO:0048265	GO:0048265	response to pain	2/389	38/23328	0.131946596	0.26133	0.16617	Reln/Vwa1	2
GO:0051953	GO:0051953	negative regulation of amine transport	2/389	38/23328	0.131946596	0.26133	0.16617	Rgs4/Ptgs	2
GO:0060219	GO:0060219	camera-type eye photoreceptor cell differentiation	2/389	38/23328	0.131946596	0.26133	0.16617	Thy1/Ptn	2
GO:0086065	GO:0086065	cell communication involved in cardiac conduction	2/389	38/23328	0.131946596	0.26133	0.16617	Gja5/Cav1	2
GO:0090322	GO:0090322	regulation of superoxide metabolic process	2/389	38/23328	0.131946596	0.26133	0.16617	Cd36/Fblr	2
GO:0097028	GO:0097028	dendritic cell differentiation	2/389	38/23328	0.131946596	0.26133	0.16617	Tmem176	2
GO:2000036	GO:2000036	regulation of stem cell population maintenance	2/389	38/23328	0.131946596	0.26133	0.16617	Ptn/Loxl2	2
GO:0006986	GO:0006986	response to unfolded protein	4/389	117/23328	0.132111548	0.26153	0.1663	Hspb1/Bo	4
GO:0006919	GO:0006919	activation of cysteine-type endopeptidase activity invol	3/389	76/23328	0.133745821	0.26454	0.16821	Bok/S100	3
GO:0090068	GO:0090068	positive regulation of cell cycle process	7/389	254/23328	0.133765347	0.26454	0.16821	Rgcc/Pdg	7
GO:0014020	GO:0014020	primary neural tube formation	4/389	118/23328	0.135068181	0.26686	0.16969	Bmp4/Sfrp	4
GO:0046545	GO:0046545	development of primary female sexual characteristics	4/389	118/23328	0.135068181	0.26686	0.16969	Bmp4/Mm	4
GO:0045786	GO:0045786	negative regulation of cell cycle	11/389	451/23328	0.13561511	0.26776	0.17026	Nr2f2/Rgc	11
GO:0050770	GO:0050770	regulation of axonogenesis	6/389	208/23328	0.135653436	0.26776	0.17026	Thy1/Cxcl	6
GO:0046883	GO:0046883	regulation of hormone secretion	8/389	303/23328	0.135799881	0.26792	0.17036	Spp1/Nmb	8
GO:0003407	GO:0003407	neural retina development	3/389	77/23328	0.137553163	0.2702	0.17181	Thy1/Ptn/	3
GO:0007257	GO:0007257	activation of JUN kinase activity	2/389	39/23328	0.137619231	0.2702	0.17181	Dab2/Trf	2
GO:0010613	GO:0010613	positive regulation of cardiac muscle hypertrophy	2/389	39/23328	0.137619231	0.2702	0.17181	Slc25a4/E	2
GO:0021983	GO:0021983	pituitary gland development	2/389	39/23328	0.137619231	0.2702	0.17181	Bmp4/Duc	2
GO:0033028	GO:0033028	myeloid cell apoptotic process	2/389	39/23328	0.137619231	0.2702	0.17181	Kitl/Anxa1	2

GO:0035850	GO:0035850	epithelial cell differentiation involved in kidney develop	2/389	39/23328	0.137619231	0.2702	0.17181	Bmp4/Pdg	2
GO:0042311	GO:0042311	vasodilation	2/389	39/23328	0.137619231	0.2702	0.17181	Adrb2/Apo	2
GO:0060251	GO:0060251	regulation of glial cell proliferation	2/389	39/23328	0.137619231	0.2702	0.17181	Ptn/Vim	2
GO:1900026	GO:1900026	positive regulation of substrate adhesion-dependent ce	2/389	39/23328	0.137619231	0.2702	0.17181	Dab2/Net1	2
GO:1905710	GO:1905710	positive regulation of membrane permeability	2/389	39/23328	0.137619231	0.2702	0.17181	Ier3/Bok	2
GO:0060759	GO:0060759	regulation of response to cytokine stimulus	4/389	119/23328	0.13805162	0.27079	0.17218	Ptprf/Cav1	4
GO:2000278	GO:2000278	regulation of DNA biosynthetic process	4/389	119/23328	0.13805162	0.27079	0.17218	Pdgfb/Gja	4
GO:0046887	GO:0046887	positive regulation of hormone secretion	5/389	164/23328	0.140001201	0.27448	0.17453	Spp1/Nmb	5
GO:0050806	GO:0050806	positive regulation of synaptic transmission	9/389	355/23328	0.141202104	0.27668	0.17593	Reln/Ptn/S	9
GO:0008625	GO:0008625	extrinsic apoptotic signaling pathway via death domain	3/389	78/23328	0.14139714	0.27668	0.17593	Timp3/Sfr	3
GO:0046718	GO:0046718	viral entry into host cell	3/389	78/23328	0.14139714	0.27668	0.17593	Cav1/Gas	3
GO:0060042	GO:0060042	retina morphogenesis in camera-type eye	3/389	78/23328	0.14139714	0.27668	0.17593	Thy1/Ptn/	3
GO:0007626	GO:0007626	locomotory behavior	7/389	258/23328	0.141625462	0.27686	0.17605	Reln/Hoxc	7
GO:0032886	GO:0032886	regulation of microtubule-based process	7/389	258/23328	0.141625462	0.27686	0.17605	Cav1/Cdh	7
GO:0000578	GO:0000578	embryonic axis specification	2/389	40/23328	0.143344402	0.27822	0.17691	Pcsk6/Nra	2
GO:0001562	GO:0001562	response to protozoan	2/389	40/23328	0.143344402	0.27822	0.17691	Iigp1/Ier3	2
GO:0009409	GO:0009409	response to cold	2/389	40/23328	0.143344402	0.27822	0.17691	Adrb2/Ucp	2
GO:0014046	GO:0014046	dopamine secretion	2/389	40/23328	0.143344402	0.27822	0.17691	Cxcl12/Sn	2
GO:0014059	GO:0014059	regulation of dopamine secretion	2/389	40/23328	0.143344402	0.27822	0.17691	Cxcl12/Sn	2
GO:0014742	GO:0014742	positive regulation of muscle hypertrophy	2/389	40/23328	0.143344402	0.27822	0.17691	Slc25a4/E	2
GO:0015804	GO:0015804	neutral amino acid transport	2/389	40/23328	0.143344402	0.27822	0.17691	Rgs4/Slc6	2
GO:0030521	GO:0030521	androgen receptor signaling pathway	2/389	40/23328	0.143344402	0.27822	0.17691	Dab2/Sfrp	2
GO:0034105	GO:0034105	positive regulation of tissue remodeling	2/389	40/23328	0.143344402	0.27822	0.17691	Spp1/Trf	2
GO:0040019	GO:0040019	positive regulation of embryonic development	2/389	40/23328	0.143344402	0.27822	0.17691	Pdgfb/Gja	2
GO:0060325	GO:0060325	face morphogenesis	2/389	40/23328	0.143344402	0.27822	0.17691	Tbx1/Mmp	2
GO:0070884	GO:0070884	regulation of calcineurin-NFAT signaling cascade	2/389	40/23328	0.143344402	0.27822	0.17691	Prnp/Lmc	2
GO:0097009	GO:0097009	energy homeostasis	2/389	40/23328	0.143344402	0.27822	0.17691	Cd36/C1q	2
GO:1901021	GO:1901021	positive regulation of calcium ion transmembrane trans	2/389	40/23328	0.143344402	0.27822	0.17691	Bmp4/Atp	2
GO:1903959	GO:1903959	regulation of anion transmembrane transport	2/389	40/23328	0.143344402	0.27822	0.17691	Prnp/Rgs4	2
GO:0015837	GO:0015837	amine transport	4/389	121/23328	0.144097237	0.27942	0.17767	Cxcl12/Rg	4
GO:0050829	GO:0050829	defense response to Gram-negative bacterium	4/389	121/23328	0.144097237	0.27942	0.17767	Lbp/B2m/	4
GO:0046631	GO:0046631	alpha-beta T cell activation	5/389	166/23328	0.145102257	0.2811	0.17874	Rsad2/Ga	5
GO:0098739	GO:0098739	import across plasma membrane	5/389	166/23328	0.145102257	0.2811	0.17874	Prnp/Adrb	5
GO:0048663	GO:0048663	neuron fate commitment	3/389	79/23328	0.145276777	0.2813	0.17887	Bmp4/Not	3
GO:0045787	GO:0045787	positive regulation of cell cycle	9/389	358/23328	0.146279926	0.28311	0.18002	Rgcc/Lgm	9
GO:0008361	GO:0008361	regulation of cell size	6/389	213/23328	0.146749158	0.28375	0.18043	Cxcl12/C1	6
GO:0042098	GO:0042098	T cell proliferation	6/389	213/23328	0.146749158	0.28375	0.18043	Prnp/Cxcl	6
GO:0002703	GO:0002703	regulation of leukocyte mediated immunity	7/389	261/23328	0.147663818	0.28535	0.18145	H2-Q6/Rs	7
GO:0002369	GO:0002369	T cell cytokine production	2/389	41/23328	0.149118792	0.28535	0.18145	Rsad2/B2	2
GO:0006953	GO:0006953	acute-phase response	2/389	41/23328	0.149118792	0.28535	0.18145	Lbp/Serpi	2
GO:0007431	GO:0007431	salivary gland development	2/389	41/23328	0.149118792	0.28535	0.18145	Pdgfb/Tgr	2
GO:0008207	GO:0008207	C21-steroid hormone metabolic process	2/389	41/23328	0.149118792	0.28535	0.18145	Dab2/Egr1	2
GO:0010470	GO:0010470	regulation of gastrulation	2/389	41/23328	0.149118792	0.28535	0.18145	Sfrp2/Gja1	2
GO:0010737	GO:0010737	protein kinase A signaling	2/389	41/23328	0.149118792	0.28535	0.18145	Adrb2/Aka	2
GO:0030225	GO:0030225	macrophage differentiation	2/389	41/23328	0.149118792	0.28535	0.18145	Bmp4/Id2	2
GO:0045806	GO:0045806	negative regulation of endocytosis	2/389	41/23328	0.149118792	0.28535	0.18145	Sh3gl3/Ca	2
GO:0046461	GO:0046461	neutral lipid catabolic process	2/389	41/23328	0.149118792	0.28535	0.18145	Mgll/Lpl	2
GO:0046464	GO:0046464	acylglycerol catabolic process	2/389	41/23328	0.149118792	0.28535	0.18145	Mgll/Lpl	2
GO:0048730	GO:0048730	epidermis morphogenesis	2/389	41/23328	0.149118792	0.28535	0.18145	Igfbp5/Cts	2
GO:0051281	GO:0051281	positive regulation of release of sequestered calcium io	2/389	41/23328	0.149118792	0.28535	0.18145	Thy1/Apln	2
GO:0051693	GO:0051693	actin filament capping	2/389	41/23328	0.149118792	0.28535	0.18145	Tpm1/Cap	2
GO:0071364	GO:0071364	cellular response to epidermal growth factor stimulus	2/389	41/23328	0.149118792	0.28535	0.18145	Dab2/Zfp3	2
GO:0099084	GO:0099084	postsynaptic specialization organization	2/389	41/23328	0.149118792	0.28535	0.18145	Reln/Gap4	2
GO:0106056	GO:0106056	regulation of calcineurin-mediated signaling	2/389	41/23328	0.149118792	0.28535	0.18145	Prnp/Lmc	2
GO:1900744	GO:1900744	regulation of p38MAPK cascade	2/389	41/23328	0.149118792	0.28535	0.18145	Gadd45g/	2
GO:1905332	GO:1905332	positive regulation of morphogenesis of an epithelium	2/389	41/23328	0.149118792	0.28535	0.18145	Bmp4/Gja	2
GO:0038034	GO:0038034	signal transduction in absence of ligand	3/389	80/23328	0.149191097	0.28535	0.18145	Lcn2/Kitl/	3
GO:0050688	GO:0050688	regulation of defense response to virus	3/389	80/23328	0.149191097	0.28535	0.18145	Fgl2/Tspa	3
GO:0097192	GO:0097192	extrinsic apoptotic signaling pathway in absence of liga	3/389	80/23328	0.149191097	0.28535	0.18145	Lcn2/Kitl/	3
GO:1901880	GO:1901880	negative regulation of protein depolymerization	3/389	80/23328	0.149191097	0.28535	0.18145	Stmn2/Tp	3
GO:0034248	GO:0034248	regulation of cellular amide metabolic process	10/389	410/23328	0.149718453	0.28623	0.182	Igfbp5/Prr	10
GO:0009895	GO:0009895	negative regulation of catabolic process	8/389	311/23328	0.150411851	0.28679	0.18236	Serpine2/	8
GO:0071897	GO:0071897	DNA biosynthetic process	5/389	169/23328	0.152901176	0.28679	0.18236	Pdgfb/Gja	5
GO:0051881	GO:0051881	regulation of mitochondrial membrane potential	3/389	81/23328	0.153139127	0.28679	0.18236	Ucp2/Bok	3
GO:1904589	GO:1904589	regulation of protein import	3/389	81/23328	0.153139127	0.28679	0.18236	Cd36/Bmp	3
GO:0008544	GO:0008544	epidermis development	8/389	313/23328	0.154179952	0.28679	0.18236	Igfbp5/Bm	8
GO:0000050	GO:0000050	urea cycle	1/389	10/23328	0.15480769	0.28679	0.18236	Ass1	1
GO:0000185	GO:0000185	activation of MAPKKK activity	1/389	10/23328	0.15480769	0.28679	0.18236	Gadd45g	1
GO:0003307	GO:0003307	regulation of Wnt signaling pathway involved in heart d	1/389	10/23328	0.15480769	0.28679	0.18236	Sox17	1
GO:0006000	GO:0006000	fructose metabolic process	1/389	10/23328	0.15480769	0.28679	0.18236	Aldoa	1
GO:0006651	GO:0006651	diacylglycerol biosynthetic process	1/389	10/23328	0.15480769	0.28679	0.18236	Plpp1	1
GO:0006977	GO:0006977	DNA damage response, signal transduction by p53 cla	1/389	10/23328	0.15480769	0.28679	0.18236	Cdkn1a	1
GO:0009650	GO:0009650	UV protection	1/389	10/23328	0.15480769	0.28679	0.18236	Mfap4	1
GO:0010940	GO:0010940	positive regulation of necrotic cell death	1/389	10/23328	0.15480769	0.28679	0.18236	Slc6a6	1
GO:0021612	GO:0021612	facial nerve structural organization	1/389	10/23328	0.15480769	0.28679	0.18236	Nrp2	1
GO:0030952	GO:0030952	establishment or maintenance of cytoskeleton polarity	1/389	10/23328	0.15480769	0.28679	0.18236	Aqp1	1
GO:0031274	GO:0031274	positive regulation of pseudopodium assembly	1/389	10/23328	0.15480769	0.28679	0.18236	Cdc42ep3	1
GO:0032815	GO:0032815	negative regulation of natural killer cell activation	1/389	10/23328	0.15480769	0.28679	0.18236	Pglyrp1	1
GO:0033029	GO:0033029	regulation of neutrophil apoptotic process	1/389	10/23328	0.15480769	0.28679	0.18236	Anxa1	1
GO:0033085	GO:0033085	negative regulation of T cell differentiation in thymus	1/389	10/23328	0.15480769	0.28679	0.18236	Bmp4	1
GO:0033689	GO:0033689	negative regulation of osteoblast proliferation	1/389	10/23328	0.15480769	0.28679	0.18236	Sfrp1	1
GO:0034145	GO:0034145	positive regulation of toll-like receptor 4 signaling path	1/389	10/23328	0.15480769	0.28679	0.18236	Lbp	1
GO:0034380	GO:0034380	high-density lipoprotein particle assembly	1/389	10/23328	0.15480769	0.28679	0.18236	Apoe	1
GO:0035811	GO:0035811	negative regulation of urine volume	1/389	10/23328	0.15480769	0.28679	0.18236	Adrb2	1
GO:0035907	GO:0035907	dorsal aorta development	1/389	10/23328	0.15480769	0.28679	0.18236	Eng	1
GO:0036462	GO:0036462	TRAIL-activated apoptotic signaling pathway	1/389	10/23328	0.15480769	0.28679	0.18236	Timp3	1
GO:0039532	GO:0039532	negative regulation of viral-induced cytoplasmic patter	1/389	10/23328	0.15480769	0.28679	0.18236	Tspan6	1
GO:0042421	GO:0042421	norepinephrine biosynthetic process	1/389	10/23328	0.15480769	0.28679	0.18236	Epas1	1
GO:0046598	GO:0046598	positive regulation of viral entry into host cell	1/389	10/23328	0.15480769	0.28679	0.18236	Lgals1	1
GO:0048703	GO:0048703	embryonic viscerocranium morphogenesis	1/389	10/23328	0.15480769	0.28679	0.18236	Tbx1	1
GO:0048739	GO:0048739	cardiac muscle fiber development	1/389	10/23328	0.15480769	0.28679	0.18236	Bmp4	1
GO:0051006	GO:0051006	positive regulation of lipoprotein lipase activity	1/389	10/23328	0.15480769	0.28679	0.18236	Gpihbp1	1
GO:0060297	GO:0060297	regulation of sarcomere organization	1/389	10/23328	0.15480769	0.28679	0.18236	Prox1	1
GO:0060513	GO:0060513	prostatic bud formation	1/389	10/23328	0.15480769	0.28679	0.18236	Bmp4	1
GO:0060710	GO:0060710	chorio-allantoic fusion	1/389	10/23328	0.15480769	0.28679	0.18236	Vcam1	1
GO:0060907	GO:0060907	positive regulation of macrophage cytokine production	1/389	10/23328	0.15480769	0.28679	0.18236	Cd36	1
GO:0060982	GO:0060982	coronary artery morphogenesis	1/389	10/23328	0.15480769	0.28679	0.18236	Tbx1	1
GO:0070244	GO:0070244	negative regulation of thymocyte apoptotic process	1/389	10/23328	0.15480769	0.28679	0.18236	Bmp4	1
GO:0070673	GO:0070673	response to interleukin-18	1/389	10/23328	0.15480769	0.28679	0.18236	Pdgfb	1
GO:0070857	GO:0070857	regulation of bile acid biosynthetic process	1/389	10/23328	0.15480769	0.28679	0.18236	Prox1	1
GO:0072070	GO:0072070	loop of Henle development	1/389	10/23328	0.15480769	0.28679	0.18236	Aqp1	1
GO:0075294	GO:0075294	positive regulation by symbiont of entry into host	1/389	10/23328	0.15480769	0.28679	0.18236	Lgals1	1
GO:0090091	GO:0090091	positive regulation of extracellular matrix disassembly	1/389	10/23328	0.15480769	0.28679	0.18236	Pdpn	1
GO:0090129	GO:0090129	positive regulation of synapse maturation	1/389	10/23328	0.15480769	0.28679	0.18236	Reln	1
GO:0090713	GO:0090713	immunological memory process	1/389	10/23328	0.15480769	0.28679	0.18236	Fgl2	1
GO:0097067	GO:0097067	cellular response to thyroid hormone stimulus	1/389	10/23328	0.15480769	0.28679	0.18236	Lmo2	1
GO:0097119	GO:0097119	postsynaptic density protein 95 clustering	1/389	10/23328	0.15480769	0.28679	0.18236	Reln	1
GO:0097264	GO:0097264	self proteolysis	1/389	10/23328	0.15480769	0.28679	0.18236	Lgmn	1
GO:0099541	GO:0099541	trans-synaptic signaling by lipid	1/389	10/23328	0.15480769	0.28679	0.18236	Fabp5	1
GO:0099542	GO:0099542	trans-synaptic signaling by endocannabinoid	1/389	10/23328	0.15480769	0.28679	0.18236	Fabp5	1
GO:0140052	GO:0140052	cellular response to oxidised low-density lipoprotein pa	1/389	10/23328	0.15480769	0.28679	0.18236	Cd36	1
GO:1900038	GO:1900038	negative regulation of cellular response to hypoxia	1/389	10/23328	0.15480769	0.28679	0.18236	Cd34	1
GO:1900103	GO:1900103	positive regulation of endoplasmic reticulum unfolded p	1/389	10/23328	0.15480769	0.28679	0.18236	Bok	1
GO:1902033	GO:1902033	regulation of hematopoietic stem cell proliferation	1/389	10/23328	0.15480769	0.28679	0.18236	Kitl	1
GO:1902746	GO:1902746	regulation of lens fiber cell differentiation	1/389	10/23328	0.15480769	0.28679	0.18236	Cdkn1c	1
GO:1903238	GO:1903238	positive regulation of leukocyte tethering or rolling	1/389	10/23328	0.15480769	0.28679	0.18236	Selp	1
GO:1905522	GO:1905522	negative regulation of macrophage migration	1/389	10/23328	0.15480769	0.28679	0.18236	Cd200	1
GO:1990264	GO:1990264	peptidyl-tyrosine dephosphorylation involved in inactiva	1/389	10/23328	0.15480769	0.28679	0.18236	Ptprb	1
GO:1990535	GO:1990535	neuron projection maintenance	1/389	10/23328	0.15480769	0.28679	0.18236	Prnp	1
GO:2000489	GO:2000489	regulation of hepatic stellate cell activation	1/389	10/23328	0.15480769	0.28679	0.18236	Pdgfb	1
GO:2000543	GO:2000543	positive regulation of gastrulation	1/389	10/23328	0.15480769	0.28679	0.18236	Gja1	1
GO:2000551	GO:2000551	regulation of T-helper 2 cell cytokine production	1/389	10/23328	0.15480769	0.28679	0.18236	Rsad2	1
GO:2000774	GO:2000774	positive regulation of cellular senescence	1/389	10/23328	0.15480769	0.28679	0.18236	B2m	1
GO:2000807	GO:2000807	regulation of synaptic vesicle clustering	1/389	10/23328	0.15480769	0.28679	0.18236	Pcdh17	1
GO:2001138	GO:2001138	regulation of phospholipid transport	1/389	10/23328	0.15480769	0.28679	0.18236	Apoe	1
GO:2001140	GO:2001140	positive regulation of phospholipid transport	1/389	10/23328	0.15480769	0.28679	0.18236	Apoe	1
GO:0051154	GO:0051154	negative regulation of striated muscle cell differentiatic	2/389	42/23328	0.15493918	0.28679	0.18236	Rgs4/Pi16	2
GO:0055023	GO:0055023	positive regulation of cardiac muscle tissue growth	2/389	42/23328	0.15493918	0.28679	0.18236	Slc25a4/N	2
GO:0072348	GO:0072348	sulfur compound transport	2/389	42/23328	0.15493918	0.28679	0.18236	Slc6a6/Sl	2

GO:0072583	GO:0072583	clathrin-dependent endocytosis	2/389	42/23328	0.15493918	0.28679	0.18236	Sh3gl3/Da	2
GO:2000772	GO:2000772	regulation of cellular senescence	2/389	42/23328	0.15493918	0.28679	0.18236	B2m/Plk2	2
GO:0042742	GO:0042742	defense response to bacterium	11/389	464/23328	0.15503225	0.28683	0.18239	Lbp/Lcn2/	11
GO:0048639	GO:0048639	positive regulation of developmental growth	6/389	217/23328	0.155913102	0.28833	0.18334	Adrb2/Cx	6
GO:0044409	GO:0044409	entry into host	3/389	82/23328	0.157119892	0.29043	0.18467	Cav1/Gas	3
GO:0010952	GO:0010952	positive regulation of peptidase activity	5/389	171/23328	0.158195913	0.29215	0.18577	Lgmn/Sfrp	5
GO:0045931	GO:0045931	positive regulation of mitotic cell cycle	5/389	171/23328	0.158195913	0.29215	0.18577	Rgcc/Lgm	5
GO:0007586	GO:0007586	digestion	4/389	126/23328	0.159650593	0.29471	0.18739	Cd36/F11	4
GO:0014003	GO:0014003	oligodendrocyte development	2/389	43/23328	0.160802437	0.29603	0.18823	Clu/Id2	2
GO:0032008	GO:0032008	positive regulation of TOR signaling	2/389	43/23328	0.160802437	0.29603	0.18823	Reln/Gas6	2
GO:0042304	GO:0042304	regulation of fatty acid biosynthetic process	2/389	43/23328	0.160802437	0.29603	0.18823	Fabp5/Anx	2
GO:0046782	GO:0046782	regulation of viral transcription	2/389	43/23328	0.160802437	0.29603	0.18823	Zfp36/Jun	2
GO:1903792	GO:1903792	negative regulation of anion transport	2/389	43/23328	0.160802437	0.29603	0.18823	Rgs4/Apo	2
GO:2001258	GO:2001258	negative regulation of cation channel activity	2/389	43/23328	0.160802437	0.29603	0.18823	Ank3/Cav	2
GO:0021872	GO:0021872	forebrain generation of neurons	3/389	83/23328	0.161132424	0.2965	0.18854	B2m/Nrp2	3
GO:0046651	GO:0046651	lymphocyte proliferation	8/389	317/23328	0.161850312	0.29769	0.18929	Prnp/Cxcl	8
GO:0051260	GO:0051260	protein homooligomerization	5/389	173/23328	0.163564743	0.30071	0.19121	Prnp/B2m	5
GO:0010498	GO:0010498	proteasomal protein catabolic process	11/389	470/23328	0.164449028	0.30102	0.19141	Ecscr/Cav	11
GO:0046530	GO:0046530	photoreceptor cell differentiation	3/389	84/23328	0.165175755	0.30102	0.19141	Thy1/Ptn/	3
GO:0061387	GO:0061387	regulation of extent of cell growth	4/389	128/23328	0.166038485	0.30102	0.19141	Cxcl12/Mc	4
GO:0010972	GO:0010972	negative regulation of G2/M transition of mitotic cell cy	2/389	44/23328	0.166705528	0.30102	0.19141	Ier3/Cdkn	2
GO:0042462	GO:0042462	eye photoreceptor cell development	2/389	44/23328	0.166705528	0.30102	0.19141	Thy1/Pdgf	2
GO:0050919	GO:0050919	negative chemotaxis	2/389	44/23328	0.166705528	0.30102	0.19141	Nrp2/Sem	2
GO:0070849	GO:0070849	response to epidermal growth factor	2/389	44/23328	0.166705528	0.30102	0.19141	Dab2/Zfp3	2
GO:0086002	GO:0086002	cardiac muscle cell action potential involved in contrac	2/389	44/23328	0.166705528	0.30102	0.19141	Gja5/Cav1	2
GO:1903523	GO:1903523	negative regulation of blood circulation	2/389	44/23328	0.166705528	0.30102	0.19141	Mmp2/Adr	2
GO:0032943	GO:0032943	mononuclear cell proliferation	8/389	320/23328	0.167718077	0.30102	0.19141	Prnp/Cxcl	8
GO:0001767	GO:0001767	establishment of lymphocyte polarity	1/389	11/23328	0.168907519	0.30102	0.19141	Cyp26b1	1
GO:0001768	GO:0001768	establishment of T cell polarity	1/389	11/23328	0.168907519	0.30102	0.19141	Cyp26b1	1
GO:0002468	GO:0002468	dendritic cell antigen processing and presentation	1/389	11/23328	0.168907519	0.30102	0.19141	Fgl2	1
GO:0002679	GO:0002679	respiratory burst involved in defense response	1/389	11/23328	0.168907519	0.30102	0.19141	Lbp	1
GO:0002923	GO:0002923	regulation of humoral immune response mediated by c	1/389	11/23328	0.168907519	0.30102	0.19141	Cd55	1
GO:0003139	GO:0003139	secondary heart field specification	1/389	11/23328	0.168907519	0.30102	0.19141	Bmp4	1
GO:0003266	GO:0003266	regulation of secondary heart field cardioblast prolifera	1/389	11/23328	0.168907519	0.30102	0.19141	Tbx1	1
GO:0008300	GO:0008300	isoprenoid catabolic process	1/389	11/23328	0.168907519	0.30102	0.19141	Cyp26b1	1
GO:0009812	GO:0009812	flavonoid metabolic process	1/389	11/23328	0.168907519	0.30102	0.19141	Cyp1a1	1
GO:0014889	GO:0014889	muscle atrophy	1/389	11/23328	0.168907519	0.30102	0.19141	Fbxo32	1
GO:0019627	GO:0019627	urea metabolic process	1/389	11/23328	0.168907519	0.30102	0.19141	Ass1	1
GO:0021684	GO:0021684	cerebellar granular layer formation	1/389	11/23328	0.168907519	0.30102	0.19141	Prox1	1
GO:0021707	GO:0021707	cerebellar granule cell differentiation	1/389	11/23328	0.168907519	0.30102	0.19141	Prox1	1
GO:0031077	GO:0031077	post-embryonic camera-type eye development	1/389	11/23328	0.168907519	0.30102	0.19141	Flt1	1
GO:0031272	GO:0031272	regulation of pseudopodium assembly	1/389	11/23328	0.168907519	0.30102	0.19141	Cdc42ep3	1
GO:0032342	GO:0032342	aldosterone biosynthetic process	1/389	11/23328	0.168907519	0.30102	0.19141	Dab2	1
GO:0032429	GO:0032429	regulation of phospholipase A2 activity	1/389	11/23328	0.168907519	0.30102	0.19141	Anxa1	1
GO:0033084	GO:0033084	regulation of immature T cell proliferation in thymus	1/389	11/23328	0.168907519	0.30102	0.19141	Bmp4	1
GO:0033700	GO:0033700	phospholipid efflux	1/389	11/23328	0.168907519	0.30102	0.19141	Apoe	1
GO:0036005	GO:0036005	response to macrophage colony-stimulating factor	1/389	11/23328	0.168907519	0.30102	0.19141	Pde2a	1
GO:0036006	GO:0036006	cellular response to macrophage colony-stimulating fa	1/389	11/23328	0.168907519	0.30102	0.19141	Pde2a	1
GO:0036499	GO:0036499	PERK-mediated unfolded protein response	1/389	11/23328	0.168907519	0.30102	0.19141	Bok	1
GO:0045602	GO:0045602	negative regulation of endothelial cell differentiation	1/389	11/23328	0.168907519	0.30102	0.19141	Id1	1
GO:0046689	GO:0046689	response to mercury ion	1/389	11/23328	0.168907519	0.30102	0.19141	Aqp1	1
GO:0048149	GO:0048149	behavioral response to ethanol	1/389	11/23328	0.168907519	0.30102	0.19141	Adh1	1
GO:0048563	GO:0048563	post-embryonic animal organ morphogenesis	1/389	11/23328	0.168907519	0.30102	0.19141	Flt1	1
GO:0048617	GO:0048617	embryonic foregut morphogenesis	1/389	11/23328	0.168907519	0.30102	0.19141	Sox17	1
GO:0048630	GO:0048630	skeletal muscle tissue growth	1/389	11/23328	0.168907519	0.30102	0.19141	Adrb2	1
GO:0051458	GO:0051458	corticotropin secretion	1/389	11/23328	0.168907519	0.30102	0.19141	Aqp1	1
GO:0051561	GO:0051561	positive regulation of mitochondrial calcium ion concer	1/389	11/23328	0.168907519	0.30102	0.19141	Tgm2	1
GO:0051601	GO:0051601	exocyst localization	1/389	11/23328	0.168907519	0.30102	0.19141	Exoc3l2	1
GO:0051901	GO:0051901	positive regulation of mitochondrial depolarization	1/389	11/23328	0.168907519	0.30102	0.19141	Dcn	1
GO:0055015	GO:0055015	ventricular cardiac muscle cell development	1/389	11/23328	0.168907519	0.30102	0.19141	Prox1	1
GO:0060353	GO:0060353	regulation of cell adhesion molecule production	1/389	11/23328	0.168907519	0.30102	0.19141	Cav1	1
GO:0060900	GO:0060900	embryonic camera-type eye formation	1/389	11/23328	0.168907519	0.30102	0.19141	Prox1	1
GO:0061299	GO:0061299	retina vasculature morphogenesis in camera-type eye	1/389	11/23328	0.168907519	0.30102	0.19141	Arhgef15	1
GO:0061309	GO:0061309	cardiac neural crest cell development involved in outflo	1/389	11/23328	0.168907519	0.30102	0.19141	Bmp4	1
GO:0061365	GO:0061365	positive regulation of triglyceride lipase activity	1/389	11/23328	0.168907519	0.30102	0.19141	Gpihbp1	1
GO:0071600	GO:0071600	otic vesicle morphogenesis	1/389	11/23328	0.168907519	0.30102	0.19141	Tbx1	1
GO:0071732	GO:0071732	cellular response to nitric oxide	1/389	11/23328	0.168907519	0.30102	0.19141	Aqp1	1
GO:0072178	GO:0072178	nephric duct morphogenesis	1/389	11/23328	0.168907519	0.30102	0.19141	Bmp4	1
GO:0072431	GO:0072431	signal transduction involved in mitotic G1 DNA damag	1/389	11/23328	0.168907519	0.30102	0.19141	Cdkn1a	1
GO:0086016	GO:0086016	AV node cell action potential	1/389	11/23328	0.168907519	0.30102	0.19141	Gja5	1
GO:0086027	GO:0086027	AV node cell to bundle of His cell signaling	1/389	11/23328	0.168907519	0.30102	0.19141	Gja5	1
GO:0086103	GO:0086103	G protein-coupled receptor signaling pathway involved	1/389	11/23328	0.168907519	0.30102	0.19141	Cav1	1
GO:0090037	GO:0090037	positive regulation of protein kinase C signaling	1/389	11/23328	0.168907519	0.30102	0.19141	Flt4	1
GO:0098664	GO:0098664	G protein-coupled serotonin receptor signaling pathwa	1/389	11/23328	0.168907519	0.30102	0.19141	Htr2b	1
GO:0099612	GO:0099612	protein localization to axon	1/389	11/23328	0.168907519	0.30102	0.19141	Ank3	1
GO:0140467	GO:0140467	integrated stress response signaling	1/389	11/23328	0.168907519	0.30102	0.19141	Bok	1
GO:1901030	GO:1901030	positive regulation of mitochondrial outer membrane pe	1/389	11/23328	0.168907519	0.30102	0.19141	Bok	1
GO:1901142	GO:1901142	insulin metabolic process	1/389	11/23328	0.168907519	0.30102	0.19141	Cpe	1
GO:1901339	GO:1901339	regulation of store-operated calcium channel activity	1/389	11/23328	0.168907519	0.30102	0.19141	Bmp4	1
GO:1902260	GO:1902260	negative regulation of delayed rectifier potassium char	1/389	11/23328	0.168907519	0.30102	0.19141	Ank3	1
GO:1902400	GO:1902400	intracellular signal transduction involved in G1 DNA da	1/389	11/23328	0.168907519	0.30102	0.19141	Cdkn1a	1
GO:1902548	GO:1902548	negative regulation of cellular response to vascular en	1/389	11/23328	0.168907519	0.30102	0.19141	Dcn	1
GO:1903867	GO:1903867	extraembryonic membrane development	1/389	11/23328	0.168907519	0.30102	0.19141	Htra1	1
GO:1903911	GO:1903911	positive regulation of receptor clustering	1/389	11/23328	0.168907519	0.30102	0.19141	Ptn	1
GO:1905288	GO:1905288	vascular associated smooth muscle cell apoptotic proc	1/389	11/23328	0.168907519	0.30102	0.19141	Gapdh	1
GO:1905459	GO:1905459	regulation of vascular associated smooth muscle cell a	1/389	11/23328	0.168907519	0.30102	0.19141	Gapdh	1
GO:2001280	GO:2001280	positive regulation of unsaturated fatty acid biosyntheti	1/389	11/23328	0.168907519	0.30102	0.19141	Anxa1	1
GO:0048524	GO:0048524	positive regulation of viral process	3/389	85/23328	0.16924892	0.30139	0.19165	Apoe/Jun/	3
GO:0033138	GO:0033138	positive regulation of peptidyl-serine phosphorylation	4/389	129/23328	0.169266345	0.30139	0.19165	Sfrp2/Cav	4
GO:0051259	GO:0051259	protein complex oligomerization	6/389	223/23328	0.170115586	0.30277	0.19252	Prnp/B2m	6
GO:2001257	GO:2001257	regulation of cation channel activity	5/389	176/23328	0.171752546	0.30555	0.19429	Reln/Adrb	5
GO:0008203	GO:0008203	cholesterol metabolic process	4/389	130/23328	0.172516215	0.30581	0.19445	Ephx2/Cy	4
GO:0001961	GO:0001961	positive regulation of cytokine-mediated signaling path	2/389	45/23328	0.172645503	0.30581	0.19445	Irf7/Gas6	2
GO:0003156	GO:0003156	regulation of animal organ formation	2/389	45/23328	0.172645503	0.30581	0.19445	Tbx1/Bmp	2
GO:0009166	GO:0009166	nucleotide catabolic process	2/389	45/23328	0.172645503	0.30581	0.19445	Nudt4/Pde	2
GO:0021696	GO:0021696	cerebellar cortex morphogenesis	2/389	45/23328	0.172645503	0.30581	0.19445	Serpine2/I	2
GO:0030890	GO:0030890	positive regulation of B cell proliferation	2/389	45/23328	0.172645503	0.30581	0.19445	Cd38/Cdk	2
GO:0050856	GO:0050856	regulation of T cell receptor signaling pathway	2/389	45/23328	0.172645503	0.30581	0.19445	Thy1/Prnp	2
GO:0089718	GO:0089718	amino acid import across plasma membrane	2/389	45/23328	0.172645503	0.30581	0.19445	Rgs4/Slc6	2
GO:1901031	GO:1901031	regulation of response to reactive oxygen species	2/389	45/23328	0.172645503	0.30581	0.19445	Cd36/Fbln	2
GO:1902743	GO:1902743	regulation of lamellipodium organization	2/389	45/23328	0.172645503	0.30581	0.19445	Aqp1/Pdp	2
GO:1905897	GO:1905897	regulation of response to endoplasmic reticulum stress	3/389	86/23328	0.17335096	0.30692	0.19516	Cav1/Clu/	3
GO:0035148	GO:0035148	tube formation	5/389	177/23328	0.174516678	0.30885	0.19639	Bmp4/Sfrp	5
GO:1901617	GO:1901617	organic hydroxy compound biosynthetic process	6/389	225/23328	0.174966453	0.30952	0.19681	Epas1/Plt	6
GO:0016050	GO:0016050	vesicle organization	7/389	275/23328	0.177384255	0.31234	0.19861	Srgn/Cd34	7
GO:0044772	GO:0044772	mitotic cell cycle phase transition	9/389	376/23328	0.178605248	0.31234	0.19861	Rgcc/Cdk	9
GO:0010259	GO:0010259	multicellular organism aging	2/389	46/23328	0.178619503	0.31234	0.19861	Comp/Me	2
GO:0010518	GO:0010518	positive regulation of phospholipase activity	2/389	46/23328	0.178619503	0.31234	0.19861	Flt1/Htr2b	2
GO:0019083	GO:0019083	viral transcription	2/389	46/23328	0.178619503	0.31234	0.19861	Zfp36/Jun	2
GO:0030835	GO:0030835	negative regulation of actin filament depolymerization	2/389	46/23328	0.178619503	0.31234	0.19861	Tpm1/Cap	2
GO:0060323	GO:0060323	head morphogenesis	2/389	46/23328	0.178619503	0.31234	0.19861	Tbx1/Mmp	2
GO:1902667	GO:1902667	regulation of axon guidance	2/389	46/23328	0.178619503	0.31234	0.19861	Cxcl12/Se	2
GO:1905521	GO:1905521	regulation of macrophage migration	2/389	46/23328	0.178619503	0.31234	0.19861	Cd9/Cd20	2
GO:0034763	GO:0034763	negative regulation of transmembrane transport	4/389	132/23328	0.179080178	0.31234	0.19861	Rgs4/Ank	4
GO:0002696	GO:0002696	positive regulation of leukocyte activation	11/389	479/23328	0.179090843	0.31234	0.19861	Lbp/Thy1/	11
GO:0042475	GO:0042475	odontogenesis of dentin-containing tooth	3/389	88/23328	0.181637848	0.31234	0.19861	Tbx1/Bmp	3
GO:0060079	GO:0060079	excitatory postsynaptic potential	3/389	88/23328	0.181637848	0.31234	0.19861	Reln/Adrb	3
GO:1901992	GO:1901992	positive regulation of mitotic cell cycle phase transition	3/389	88/23328	0.181637848	0.31234	0.19861	Rgcc/Anx	3
GO:1904063	GO:1904063	negative regulation of cation transmembrane transport	3/389	88/23328	0.181637848	0.31234	0.19861	Rgs4/Ank	3
GO:0008037	GO:0008037	cell recognition	7/389	277/23328	0.181826383	0.31234	0.19861	Cd9/Gap4	7
GO:0001841	GO:0001841	neural tube formation	4/389	133/23328	0.182393364	0.31234	0.19861	Bmp4/Sfrp	4
GO:0044070	GO:0044070	regulation of anion transport	4/389	133/23328	0.182393364	0.31234	0.19861	Prnp/Rgs4	4
GO:0003129	GO:0003129	heart induction	1/389	12/23328	0.182772723	0.31234	0.19861	Bmp4	1
GO:0003263	GO:0003263	cardioblast proliferation	1/389	12/23328	0.182772723	0.31234	0.19861	Tbx1	1
GO:0003264	GO:0003264	regulation of cardioblast proliferation	1/389	12/23328	0.182772723	0.31234	0.19861	Tbx1	1
GO:0003337	GO:0003337	mesenchymal to epithelial transition involved in metan	1/389	12/23328	0.182772723	0.31234	0.19861	Bmp4	1
GO:0006047	GO:0006047	UDP-N-acetylglucosamine metabolic process	1/389	12/23328	0.182772723	0.31234	0.19861	Uap1	1
GO:0006123	GO:0006123	mitochondrial electron transport, cytochrome c to oxyg	1/389	12/23328	0.182772723	0.31234	0.19861	Cox4i2	1
GO:0006705	GO:0006705	mineralocorticoid biosynthetic process	1/389	12/23328	0.182772723	0.31234	0.19861	Dab2	1

GO:0007210	GO:0007210	serotonin receptor signaling pathway	1/389	12/23328	0.182772723	0.31234	0.19861	Htr2b	1
GO:0007440	GO:0007440	foregut morphogenesis	1/389	12/23328	0.182772723	0.31234	0.19861	Sox17	1
GO:0007501	GO:0007501	mesodermal cell fate specification	1/389	12/23328	0.182772723	0.31234	0.19861	Sfrp2	1
GO:0010749	GO:0010749	regulation of nitric oxide mediated signal transduction	1/389	12/23328	0.182772723	0.31234	0.19861	Pde2a	1
GO:0010755	GO:0010755	regulation of plasminogen activation	1/389	12/23328	0.182772723	0.31234	0.19861	Serpine2	1
GO:0010872	GO:0010872	regulation of cholesterol esterification	1/389	12/23328	0.182772723	0.31234	0.19861	Apoe	1
GO:0014745	GO:0014745	negative regulation of muscle adaptation	1/389	12/23328	0.182772723	0.31234	0.19861	Igfbp5	1
GO:0014824	GO:0014824	artery smooth muscle contraction	1/389	12/23328	0.182772723	0.31234	0.19861	Cd38	1
GO:0014874	GO:0014874	response to stimulus involved in regulation of muscle a	1/389	12/23328	0.182772723	0.31234	0.19861	Fbxo32	1
GO:0019646	GO:0019646	aerobic electron transport chain	1/389	12/23328	0.182772723	0.31234	0.19861	Cox4i2	1
GO:0021561	GO:0021561	facial nerve development	1/389	12/23328	0.182772723	0.31234	0.19861	Nrp2	1
GO:0021610	GO:0021610	facial nerve morphogenesis	1/389	12/23328	0.182772723	0.31234	0.19861	Nrp2	1
GO:0030388	GO:0030388	fructose 1,6-bisphosphate metabolic process	1/389	12/23328	0.182772723	0.31234	0.19861	Aldoa	1
GO:0032341	GO:0032341	aldosterone metabolic process	1/389	12/23328	0.182772723	0.31234	0.19861	Dab2	1
GO:0032754	GO:0032754	positive regulation of interleukin-5 production	1/389	12/23328	0.182772723	0.31234	0.19861	Il33	1
GO:0032825	GO:0032825	positive regulation of natural killer cell differentiation	1/389	12/23328	0.182772723	0.31234	0.19861	Gas6	1
GO:0033080	GO:0033080	immature T cell proliferation in thymus	1/389	12/23328	0.182772723	0.31234	0.19861	Bmp4	1
GO:0033240	GO:0033240	positive regulation of cellular amine metabolic process	1/389	12/23328	0.182772723	0.31234	0.19861	Epas1	1
GO:0033603	GO:0033603	positive regulation of dopamine secretion	1/389	12/23328	0.182772723	0.31234	0.19861	Cxcl12	1
GO:0034392	GO:0034392	negative regulation of smooth muscle cell apoptotic pro	1/389	12/23328	0.182772723	0.31234	0.19861	Gapdh	1
GO:0035437	GO:0035437	maintenance of protein localization in endoplasmic reti	1/389	12/23328	0.182772723	0.31234	0.19861	Gja1	1
GO:0035457	GO:0035457	cellular response to interferon-alpha	1/389	12/23328	0.182772723	0.31234	0.19861	Gas6	1
GO:0035733	GO:0035733	hepatic stellate cell activation	1/389	12/23328	0.182772723	0.31234	0.19861	Pdgfb	1
GO:0036295	GO:0036295	cellular response to increased oxygen levels	1/389	12/23328	0.182772723	0.31234	0.19861	Cav1	1
GO:0038063	GO:0038063	collagen-activated tyrosine kinase receptor signaling p	1/389	12/23328	0.182772723	0.31234	0.19861	Col4a2	1
GO:0042363	GO:0042363	fat-soluble vitamin catabolic process	1/389	12/23328	0.182772723	0.31234	0.19861	Cyp26b1	1
GO:0043922	GO:0043922	negative regulation by host of viral transcription	1/389	12/23328	0.182772723	0.31234	0.19861	Jun	1
GO:0045348	GO:0045348	positive regulation of MHC class II biosynthetic proces	1/389	12/23328	0.182772723	0.31234	0.19861	Il33	1
GO:0045625	GO:0045625	regulation of T-helper 1 cell differentiation	1/389	12/23328	0.182772723	0.31234	0.19861	Anxa1	1
GO:0046462	GO:0046462	monoacylglycerol metabolic process	1/389	12/23328	0.182772723	0.31234	0.19861	Mgll	1
GO:0048934	GO:0048934	peripheral nervous system neuron differentiation	1/389	12/23328	0.182772723	0.31234	0.19861	Hoxd9	1
GO:0048935	GO:0048935	peripheral nervous system neuron development	1/389	12/23328	0.182772723	0.31234	0.19861	Hoxd9	1
GO:0051024	GO:0051024	positive regulation of immunoglobulin secretion	1/389	12/23328	0.182772723	0.31234	0.19861	Il33	1
GO:0060159	GO:0060159	regulation of dopamine receptor signaling pathway	1/389	12/23328	0.182772723	0.31234	0.19861	Rgs4	1
GO:0060433	GO:0060433	bronchus development	1/389	12/23328	0.182772723	0.31234	0.19861	Bmp4	1
GO:0060439	GO:0060439	trachea morphogenesis	1/389	12/23328	0.182772723	0.31234	0.19861	Bmp4	1
GO:0060502	GO:0060502	epithelial cell proliferation involved in lung morphogene	1/389	12/23328	0.182772723	0.31234	0.19861	Bmp4	1
GO:0060525	GO:0060525	prostate glandular acinus development	1/389	12/23328	0.182772723	0.31234	0.19861	Sfrp1	1
GO:0060536	GO:0060536	cartilage morphogenesis	1/389	12/23328	0.182772723	0.31234	0.19861	Stc1	1
GO:0060872	GO:0060872	semicircular canal development	1/389	12/23328	0.182772723	0.31234	0.19861	Tbx1	1
GO:0070444	GO:0070444	oligodendrocyte progenitor proliferation	1/389	12/23328	0.182772723	0.31234	0.19861	Trf	1
GO:0070445	GO:0070445	regulation of oligodendrocyte progenitor proliferation	1/389	12/23328	0.182772723	0.31234	0.19861	Trf	1
GO:0071236	GO:0071236	cellular response to antibiotic	1/389	12/23328	0.182772723	0.31234	0.19861	Crip1	1
GO:0071318	GO:0071318	cellular response to ATP	1/389	12/23328	0.182772723	0.31234	0.19861	Hsp90b1	1
GO:0071941	GO:0071941	nitrogen cycle metabolic process	1/389	12/23328	0.182772723	0.31234	0.19861	Ass1	1
GO:0072044	GO:0072044	collecting duct development	1/389	12/23328	0.182772723	0.31234	0.19861	Bmp4	1
GO:0072176	GO:0072176	nephric duct development	1/389	12/23328	0.182772723	0.31234	0.19861	Bmp4	1
GO:0072584	GO:0072584	caveolin-mediated endocytosis	1/389	12/23328	0.182772723	0.31234	0.19861	Cav1	1
GO:0090209	GO:0090209	negative regulation of triglyceride metabolic process	1/389	12/23328	0.182772723	0.31234	0.19861	Apoe	1
GO:0090343	GO:0090343	positive regulation of cell aging	1/389	12/23328	0.182772723	0.31234	0.19861	B2m	1
GO:0090557	GO:0090557	establishment of endothelial intestinal barrier	1/389	12/23328	0.182772723	0.31234	0.19861	F11r	1
GO:0097113	GO:0097113	AMPA glutamate receptor clustering	1/389	12/23328	0.182772723	0.31234	0.19861	Apoe	1
GO:0150079	GO:0150079	negative regulation of neuroinflammatory response	1/389	12/23328	0.182772723	0.31234	0.19861	Cd200	1
GO:1903800	GO:1903800	positive regulation of production of miRNAs involved ir	1/389	12/23328	0.182772723	0.31234	0.19861	Bmp4	1
GO:2000343	GO:2000343	positive regulation of chemokine (C-X-C motif) ligand 2	1/389	12/23328	0.182772723	0.31234	0.19861	Lpl	1
GO:2000402	GO:2000402	negative regulation of lymphocyte migration	1/389	12/23328	0.182772723	0.31234	0.19861	Cd200	1
GO:0001990	GO:0001990	regulation of systemic arterial blood pressure by hormo	2/389	47/23328	0.184624749	0.31458	0.20003	Gja5/Ece1	2
GO:0002820	GO:0002820	negative regulation of adaptive immune response	2/389	47/23328	0.184624749	0.31458	0.20003	Samsn1/Il	2
GO:0003179	GO:0003179	heart valve morphogenesis	2/389	47/23328	0.184624749	0.31458	0.20003	Gja5/Bmp	2
GO:0003197	GO:0003197	endocardial cushion development	2/389	47/23328	0.184624749	0.31458	0.20003	Bmp4/Eng	2
GO:0032814	GO:0032814	regulation of natural killer cell activation	2/389	47/23328	0.184624749	0.31458	0.20003	Gas6/Pgly	2
GO:0050974	GO:0050974	detection of mechanical stimulus involved in sensory p	2/389	47/23328	0.184624749	0.31458	0.20003	Serpine2/(	2
GO:0060964	GO:0060964	regulation of gene silencing by miRNA	2/389	47/23328	0.184624749	0.31458	0.20003	Bmp4/Zfp	2
GO:0051701	GO:0051701	interaction with host	4/389	135/2332	0.189079865	0.3219	0.20469	Cav1/Gas	4
GO:1901655	GO:1901655	cellular response to ketone	4/389	135/2332	0.189079865	0.3219	0.20469	Bmp4/Fbx	4
GO:0050670	GO:0050670	regulation of lymphocyte proliferation	6/389	231/2332	0.189850622	0.32308	0.20544	Prnp/Bmp	6
GO:0007044	GO:0007044	cell-substrate junction assembly	3/389	90/23328	0.190028835	0.32311	0.20546	Thy1/Tek/	3
GO:0043242	GO:0043242	negative regulation of protein-containing complex disa	3/389	90/23328	0.190028835	0.32311	0.20546	Stmn2/Tp	3
GO:0031111	GO:0031111	negative regulation of microtubule polymerization or de	2/389	48/23328	0.190658549	0.32405	0.20605	Cdh5/Stm	2
GO:0033157	GO:0033157	regulation of intracellular protein transport	6/389	232/2332	0.192377869	0.32447	0.20632	Cd36/Prnp	6
GO:1900407	GO:1900407	regulation of cellular response to oxidative stress	3/389	91/23328	0.194261021	0.32447	0.20632	Cd36/Hsp	3
GO:0007050	GO:0007050	cell cycle arrest	4/389	137/2332	0.195843489	0.32447	0.20632	Rgcc/Cdk	4
GO:0001781	GO:0001781	neutrophil apoptotic process	1/389	13/23328	0.196407198	0.32447	0.20632	Anxa1	1
GO:0002551	GO:0002551	mast cell chemotaxis	1/389	13/23328	0.196407198	0.32447	0.20632	Pgf	1
GO:0003174	GO:0003174	mitral valve development	1/389	13/23328	0.196407198	0.32447	0.20632	Gja5	1
GO:0003306	GO:0003306	Wnt signaling pathway involved in heart development	1/389	13/23328	0.196407198	0.32447	0.20632	Sox17	1
GO:0007320	GO:0007320	insemination	1/389	13/23328	0.196407198	0.32447	0.20632	Serpine2	1
GO:0008212	GO:0008212	mineralocorticoid metabolic process	1/389	13/23328	0.196407198	0.32447	0.20632	Dab2	1
GO:0009950	GO:0009950	dorsal/ventral axis specification	1/389	13/23328	0.196407198	0.32447	0.20632	Sfrp1	1
GO:0010359	GO:0010359	regulation of anion channel activity	1/389	13/23328	0.196407198	0.32447	0.20632	Prnp	1
GO:0010988	GO:0010988	regulation of low-density lipoprotein particle clearance	1/389	13/23328	0.196407198	0.32447	0.20632	Anxa2	1
GO:0015816	GO:0015816	glycine transport	1/389	13/23328	0.196407198	0.32447	0.20632	Rgs4	1
GO:0021604	GO:0021604	cranial nerve structural organization	1/389	13/23328	0.196407198	0.32447	0.20632	Nrp2	1
GO:0021670	GO:0021670	lateral ventricle development	1/389	13/23328	0.196407198	0.32447	0.20632	Aqp1	1
GO:0021794	GO:0021794	thalamus development	1/389	13/23328	0.196407198	0.32447	0.20632	Ncam1	1
GO:0030836	GO:0030836	positive regulation of actin filament depolymerization	1/389	13/23328	0.196407198	0.32447	0.20632	Dstn	1
GO:0031269	GO:0031269	pseudopodium assembly	1/389	13/23328	0.196407198	0.32447	0.20632	Cdc42ep3	1
GO:0032026	GO:0032026	response to magnesium ion	1/389	13/23328	0.196407198	0.32447	0.20632	Ank3	1
GO:0032060	GO:0032060	bleb assembly	1/389	13/23328	0.196407198	0.32447	0.20632	Emp1	1
GO:0032372	GO:0032372	negative regulation of sterol transport	1/389	13/23328	0.196407198	0.32447	0.20632	Apoe	1
GO:0032375	GO:0032375	negative regulation of cholesterol transport	1/389	13/23328	0.196407198	0.32447	0.20632	Apoe	1
GO:0032486	GO:0032486	Rap protein signal transduction	1/389	13/23328	0.196407198	0.32447	0.20632	Plk2	1
GO:0032488	GO:0032488	Cdc42 protein signal transduction	1/389	13/23328	0.196407198	0.32447	0.20632	Apoe	1
GO:0033083	GO:0033083	regulation of immature T cell proliferation	1/389	13/23328	0.196407198	0.32447	0.20632	Bmp4	1
GO:0033280	GO:0033280	response to vitamin D	1/389	13/23328	0.196407198	0.32447	0.20632	Spp1	1
GO:0034374	GO:0034374	low-density lipoprotein particle remodeling	1/389	13/23328	0.196407198	0.32447	0.20632	Apoe	1
GO:0038166	GO:0038166	angiotensin-activated signaling pathway	1/389	13/23328	0.196407198	0.32447	0.20632	Cav1	1
GO:0042482	GO:0042482	positive regulation of odontogenesis	1/389	13/23328	0.196407198	0.32447	0.20632	Cd34	1
GO:0042693	GO:0042693	muscle cell fate commitment	1/389	13/23328	0.196407198	0.32447	0.20632	Tbx1	1
GO:0043589	GO:0043589	skin morphogenesis	1/389	13/23328	0.196407198	0.32447	0.20632	Itga6	1
GO:0044090	GO:0044090	positive regulation of vacuole organization	1/389	13/23328	0.196407198	0.32447	0.20632	Anxa2	1
GO:0045161	GO:0045161	neuronal ion channel clustering	1/389	13/23328	0.196407198	0.32447	0.20632	Ank3	1
GO:0045628	GO:0045628	regulation of T-helper 2 cell differentiation	1/389	13/23328	0.196407198	0.32447	0.20632	Anxa1	1
GO:0046007	GO:0046007	negative regulation of activated T cell proliferation	1/389	13/23328	0.196407198	0.32447	0.20632	Prnp	1
GO:0048012	GO:0048012	hepatocyte growth factor receptor signaling pathway	1/389	13/23328	0.196407198	0.32447	0.20632	Esm1	1
GO:0051639	GO:0051639	actin filament network formation	1/389	13/23328	0.196407198	0.32447	0.20632	Pls3	1
GO:0055119	GO:0055119	relaxation of cardiac muscle	1/389	13/23328	0.196407198	0.32447	0.20632	Atp1b1	1
GO:0060272	GO:0060272	embryonic skeletal joint morphogenesis	1/389	13/23328	0.196407198	0.32447	0.20632	Bmp4	1
GO:0060340	GO:0060340	positive regulation of type I interferon-mediated signali	1/389	13/23328	0.196407198	0.32447	0.20632	Irf7	1
GO:0060352	GO:0060352	cell adhesion molecule production	1/389	13/23328	0.196407198	0.32447	0.20632	Cav1	1
GO:0060394	GO:0060394	negative regulation of pathway-restricted SMAD protei	1/389	13/23328	0.196407198	0.32447	0.20632	Eng	1
GO:0060601	GO:0060601	lateral sprouting from an epithelium	1/389	13/23328	0.196407198	0.32447	0.20632	Bmp4	1
GO:0061418	GO:0061418	regulation of transcription from RNA polymerase II pro	1/389	13/23328	0.196407198	0.32447	0.20632	Egr1	1
GO:0071295	GO:0071295	cellular response to vitamin	1/389	13/23328	0.196407198	0.32447	0.20632	Gas6	1
GO:0071679	GO:0071679	commissural neuron axon guidance	1/389	13/23328	0.196407198	0.32447	0.20632	Ncam1	1
GO:0086067	GO:0086067	AV node cell to bundle of His cell communication	1/389	13/23328	0.196407198	0.32447	0.20632	Gja5	1
GO:0097090	GO:0097090	presynaptic membrane organization	1/389	13/23328	0.196407198	0.32447	0.20632	Apoe	1
GO:0097201	GO:0097201	negative regulation of transcription from RNA polymera	1/389	13/23328	0.196407198	0.32447	0.20632	Jun	1
GO:0097577	GO:0097577	sequestering of iron ion	1/389	13/23328	0.196407198	0.32447	0.20632	Lcn2	1
GO:0097688	GO:0097688	glutamate receptor clustering	1/389	13/23328	0.196407198	0.32447	0.20632	Apoe	1
GO:0110154	GO:0110154	RNA decapping	1/389	13/23328	0.196407198	0.32447	0.20632	Zfp36	1
GO:0110156	GO:0110156	methylguanosine-cap decapping	1/389	13/23328	0.196407198	0.32447	0.20632	Zfp36	1
GO:1900025	GO:1900025	negative regulation of substrate adhesion-dependent c	1/389	13/23328	0.196407198	0.32447	0.20632	Actn4	1
GO:1901096	GO:1901096	regulation of autophagosome maturation	1/389	13/23328	0.196407198	0.32447	0.20632	Adrb2	1
GO:1901201	GO:1901201	regulation of extracellular matrix assembly	1/389	13/23328	0.196407198	0.32447	0.20632	Rgcc	1
GO:1901678	GO:1901678	iron coordination entity transport	1/389	13/23328	0.196407198	0.32447	0.20632	Lcn2	1
GO:1902170	GO:1902170	cellular response to reactive nitrogen species	1/389	13/23328	0.196407198	0.32447	0.20632	Aqp1	1
GO:1902285	GO:1902285	semaphorin-plexin signaling pathway involved in neuro	1/389	13/23328	0.196407198	0.32447	0.20632	Nrp2	1
GO:1903651	GO:1903651	positive regulation of cytoplasmic transport	1/389	13/23328	0.196407198	0.32447	0.20632	Dab2	1

GO:1904251	GO:1904251	regulation of bile acid metabolic process	1/389	13/23328	0.196407198	0.32447	0.20632	Prox1	1
GO:2000833	GO:2000833	positive regulation of steroid hormone secretion	1/389	13/23328	0.196407198	0.32447	0.20632	Dab2	1
GO:2001054	GO:2001054	negative regulation of mesenchymal cell apoptotic proc	1/389	13/23328	0.196407198	0.32447	0.20632	Tbx1	1
GO:0006695	GO:0006695	cholesterol biosynthetic process	2/389	49/23328	0.19671829	0.32447	0.20632	Cyb5r3/Ap	2
GO:0021532	GO:0021532	neural tube patterning	2/389	49/23328	0.19671829	0.32447	0.20632	Sox17/Bm	2
GO:0032941	GO:0032941	secretion by tissue	2/389	49/23328	0.19671829	0.32447	0.20632	Aqp1/Gja1	2
GO:0033173	GO:0033173	calcineurin-NFAT signaling cascade	2/389	49/23328	0.19671829	0.32447	0.20632	Prnp/Lmc	2
GO:0042755	GO:0042755	eating behavior	2/389	49/23328	0.19671829	0.32447	0.20632	Tcf15/Cd3	2
GO:0043090	GO:0043090	amino acid import	2/389	49/23328	0.19671829	0.32447	0.20632	Rgs4/Slc6	2
GO:0046638	GO:0046638	positive regulation of alpha-beta T cell differentiation	2/389	49/23328	0.19671829	0.32447	0.20632	Il2rg/Anxa	2
GO:0090342	GO:0090342	regulation of cell aging	2/389	49/23328	0.19671829	0.32447	0.20632	B2m/Plk2	2
GO:1902653	GO:1902653	secondary alcohol biosynthetic process	2/389	49/23328	0.19671829	0.32447	0.20632	Cyb5r3/Ap	2
GO:1903018	GO:1903018	regulation of glycoprotein metabolic process	2/389	49/23328	0.19671829	0.32447	0.20632	Bace2/Cs	2
GO:0032944	GO:0032944	regulation of mononuclear cell proliferation	6/389	234/23328	0.197470688	0.32557	0.20702	Prnp/Bmp	6
GO:0002275	GO:0002275	myeloid cell activation involved in immune response	3/389	92/23328	0.198516429	0.32703	0.20795	Lbp/Anxa	3
GO:0006096	GO:0006096	glycolytic process	3/389	92/23328	0.198516429	0.32703	0.20795	Ier3/Aldoa	3
GO:0001508	GO:0001508	action potential	4/389	138/23328	0.199253076	0.32785	0.20847	Cd36/Gja5	4
GO:0009411	GO:0009411	response to UV	4/389	138/23328	0.199253076	0.32785	0.20847	Mfap4/Aqp	4
GO:0050671	GO:0050671	positive regulation of lymphocyte proliferation	4/389	138/23328	0.199253076	0.32785	0.20847	Vcam1/Cc	4
GO:0021953	GO:0021953	central nervous system neuron differentiation	6/389	235/23328	0.200035902	0.32876	0.20905	Sfrp2/B2m	6
GO:0032868	GO:0032868	response to insulin	6/389	235/23328	0.200035902	0.32876	0.20905	Ptprf/Socs	6
GO:0030072	GO:0030072	peptide hormone secretion	7/389	285/23328	0.200048419	0.32876	0.20905	Ucp2/Aqp	7
GO:0008406	GO:0008406	gonad development	6/389	236/23328	0.202613412	0.33155	0.21082	Bmp4/Sfrp	6
GO:0006757	GO:0006757	ATP generation from ADP	3/389	93/23328	0.20279414	0.33155	0.21082	Ier3/Aldoa	3
GO:0007215	GO:0007215	glutamate receptor signaling pathway	3/389	93/23328	0.20279414	0.33155	0.21082	Reln/Prnp	3
GO:0009308	GO:0009308	amine metabolic process	3/389	93/23328	0.20279414	0.33155	0.21082	Epas1/Vc	3
GO:0033077	GO:0033077	T cell differentiation in thymus	3/389	93/23328	0.20279414	0.33155	0.21082	Bmp4/B2r	3
GO:0150115	GO:0150115	cell-substrate junction organization	3/389	93/23328	0.20279414	0.33155	0.21082	Thy1/Tek/	3
GO:0008038	GO:0008038	neuron recognition	2/389	50/23328	0.202801437	0.33155	0.21082	Gap43/Nc	2
GO:0021545	GO:0021545	cranial nerve development	2/389	50/23328	0.202801437	0.33155	0.21082	Tbx1/Nrp2	2
GO:0032309	GO:0032309	icosanoid secretion	2/389	50/23328	0.202801437	0.33155	0.21082	Nmb/Anxa	2
GO:0043370	GO:0043370	regulation of CD4-positive, alpha-beta T cell differentia	2/389	50/23328	0.202801437	0.33155	0.21082	Il2rg/Anxa	2
GO:0060147	GO:0060147	regulation of posttranscriptional gene silencing	2/389	50/23328	0.202801437	0.33155	0.21082	Bmp4/Zfp	2
GO:0060966	GO:0060966	regulation of gene silencing by RNA	2/389	50/23328	0.202801437	0.33155	0.21082	Bmp4/Zfp	2
GO:0061005	GO:0061005	cell differentiation involved in kidney development	2/389	50/23328	0.202801437	0.33155	0.21082	Bmp4/Pdg	2
GO:0032946	GO:0032946	positive regulation of mononuclear cell proliferation	4/389	140/23328	0.206125506	0.3339	0.21232	Vcam1/Cc	4
GO:0046330	GO:0046330	positive regulation of JNK cascade	4/389	140/23328	0.206125506	0.3339	0.21232	Flt4/Gadd	4
GO:0070664	GO:0070664	negative regulation of leukocyte proliferation	3/389	94/23328	0.207093239	0.3339	0.21232	Prnp/Bmp	3
GO:0000288	GO:0000288	nuclear-transcribed mRNA catabolic process, deadeny	2/389	51/23328	0.208905532	0.3339	0.21232	Btg2/Zfp3	2
GO:0030901	GO:0030901	midbrain development	2/389	51/23328	0.208905532	0.3339	0.21232	Sfrp2/Sfrp	2
GO:0032722	GO:0032722	positive regulation of chemokine production	2/389	51/23328	0.208905532	0.3339	0.21232	Lbp/Lpl	2
GO:0035137	GO:0035137	hindlimb morphogenesis	2/389	51/23328	0.208905532	0.3339	0.21232	Hoxd9/Bm	2
GO:0046470	GO:0046470	phosphatidylcholine metabolic process	2/389	51/23328	0.208905532	0.3339	0.21232	Fabp5/Sc	2
GO:0060043	GO:0060043	regulation of cardiac muscle cell proliferation	2/389	51/23328	0.208905532	0.3339	0.21232	Ncam1/Gj	2
GO:0060428	GO:0060428	lung epithelium development	2/389	51/23328	0.208905532	0.3339	0.21232	Bmp4/Klf2	2
GO:0060760	GO:0060760	positive regulation of response to cytokine stimulus	2/389	51/23328	0.208905532	0.3339	0.21232	Irf7/Gas6	2
GO:1902652	GO:1902652	secondary alcohol metabolic process	4/389	141/23328	0.20958743	0.3339	0.21232	Ephx2/Cy	4
GO:0002638	GO:0002638	negative regulation of immunoglobulin production	1/389	14/23328	0.209814773	0.3339	0.21232	Il33	1
GO:0002756	GO:0002756	MyD88-independent toll-like receptor signaling pathwa	1/389	14/23328	0.209814773	0.3339	0.21232	Irf7	1
GO:0002829	GO:0002829	negative regulation of type 2 immune response	1/389	14/23328	0.209814773	0.3339	0.21232	Anxa1	1
GO:0007351	GO:0007351	tripartite regional subdivision	1/389	14/23328	0.209814773	0.3339	0.21232	Pcsk6	1
GO:0008298	GO:0008298	intracellular mRNA localization	1/389	14/23328	0.209814773	0.3339	0.21232	Zfp36	1
GO:0008595	GO:0008595	anterior/posterior axis specification, embryo	1/389	14/23328	0.209814773	0.3339	0.21232	Pcsk6	1
GO:0009084	GO:0009084	glutamine family amino acid biosynthetic process	1/389	14/23328	0.209814773	0.3339	0.21232	Ass1	1
GO:0009111	GO:0009111	vitamin catabolic process	1/389	14/23328	0.209814773	0.3339	0.21232	Cyp26b1	1
GO:0009226	GO:0009226	nucleotide-sugar biosynthetic process	1/389	14/23328	0.209814773	0.3339	0.21232	Uap1	1
GO:0010804	GO:0010804	negative regulation of tumor necrosis factor-mediated	1/389	14/23328	0.209814773	0.3339	0.21232	Gas6	1
GO:0010882	GO:0010882	regulation of cardiac muscle contraction by calcium ior	1/389	14/23328	0.209814773	0.3339	0.21232	Atp1b1	1
GO:0010889	GO:0010889	regulation of sequestering of triglyceride	1/389	14/23328	0.209814773	0.3339	0.21232	Lpl	1
GO:0014820	GO:0014820	tonic smooth muscle contraction	1/389	14/23328	0.209814773	0.3339	0.21232	Cd38	1
GO:0014831	GO:0014831	gastro-intestinal system smooth muscle contraction	1/389	14/23328	0.209814773	0.3339	0.21232	Htr2b	1
GO:0021903	GO:0021903	rostrocaudal neural tube patterning	1/389	14/23328	0.209814773	0.3339	0.21232	Sox17	1
GO:0021984	GO:0021984	adenohypophysis development	1/389	14/23328	0.209814773	0.3339	0.21232	Duox2	1
GO:0030213	GO:0030213	hyaluronan biosynthetic process	1/389	14/23328	0.209814773	0.3339	0.21232	Pdgfb	1
GO:0032276	GO:0032276	regulation of gonadotropin secretion	1/389	14/23328	0.209814773	0.3339	0.21232	Gja1	1
GO:0032328	GO:0032328	alanine transport	1/389	14/23328	0.209814773	0.3339	0.21232	Slc6a6	1
GO:0032525	GO:0032525	somite rostral/caudal axis specification	1/389	14/23328	0.209814773	0.3339	0.21232	Nrarp	1
GO:0032736	GO:0032736	positive regulation of interleukin-13 production	1/389	14/23328	0.209814773	0.3339	0.21232	Il33	1
GO:0033079	GO:0033079	immature T cell proliferation	1/389	14/23328	0.209814773	0.3339	0.21232	Bmp4	1
GO:0034116	GO:0034116	positive regulation of heterotypic cell-cell adhesion	1/389	14/23328	0.209814773	0.3339	0.21232	Thy1	1
GO:0034309	GO:0034309	primary alcohol biosynthetic process	1/389	14/23328	0.209814773	0.3339	0.21232	Dab2	1
GO:0034638	GO:0034638	phosphatidylcholine catabolic process	1/389	14/23328	0.209814773	0.3339	0.21232	Scarb1	1
GO:0036376	GO:0036376	sodium ion export across plasma membrane	1/389	14/23328	0.209814773	0.3339	0.21232	Atp1b1	1
GO:0038065	GO:0038065	collagen-activated signaling pathway	1/389	14/23328	0.209814773	0.3339	0.21232	Col4a2	1
GO:0042362	GO:0042362	fat-soluble vitamin biosynthetic process	1/389	14/23328	0.209814773	0.3339	0.21232	Pltp	1
GO:0042983	GO:0042983	amyloid precursor protein biosynthetic process	1/389	14/23328	0.209814773	0.3339	0.21232	Bace2	1
GO:0042984	GO:0042984	regulation of amyloid precursor protein biosynthetic pro	1/389	14/23328	0.209814773	0.3339	0.21232	Bace2	1
GO:0043312	GO:0043312	neutrophil degranulation	1/389	14/23328	0.209814773	0.3339	0.21232	Anxa3	1
GO:0045651	GO:0045651	positive regulation of macrophage differentiation	1/389	14/23328	0.209814773	0.3339	0.21232	Id2	1
GO:0045792	GO:0045792	negative regulation of cell size	1/389	14/23328	0.209814773	0.3339	0.21232	C1qtnf9	1
GO:0048505	GO:0048505	regulation of timing of cell differentiation	1/389	14/23328	0.209814773	0.3339	0.21232	Serpine2	1
GO:0048537	GO:0048537	mucosa-associated lymphoid tissue development	1/389	14/23328	0.209814773	0.3339	0.21232	Id2	1
GO:0048541	GO:0048541	Peyer's patch development	1/389	14/23328	0.209814773	0.3339	0.21232	Id2	1
GO:0048755	GO:0048755	branching morphogenesis of a nerve	1/389	14/23328	0.209814773	0.3339	0.21232	Cpe	1
GO:0060263	GO:0060263	regulation of respiratory burst	1/389	14/23328	0.209814773	0.3339	0.21232	Lbp	1
GO:0060281	GO:0060281	regulation of oocyte development	1/389	14/23328	0.209814773	0.3339	0.21232	Pde3a	1
GO:0060911	GO:0060911	cardiac cell fate commitment	1/389	14/23328	0.209814773	0.3339	0.21232	Sox17	1
GO:0061308	GO:0061308	cardiac neural crest cell development involved in hear	1/389	14/23328	0.209814773	0.3339	0.21232	Bmp4	1
GO:0061615	GO:0061615	glycolytic process through fructose-6-phosphate	1/389	14/23328	0.209814773	0.3339	0.21232	Aldoa	1
GO:0070207	GO:0070207	protein homotrimerization	1/389	14/23328	0.209814773	0.3339	0.21232	Steap4	1
GO:0071340	GO:0071340	skeletal muscle acetylcholine-gated channel clustering	1/389	14/23328	0.209814773	0.3339	0.21232	Ptn	1
GO:0072413	GO:0072413	signal transduction involved in mitotic cell cycle check	1/389	14/23328	0.209814773	0.3339	0.21232	Cdkn1a	1
GO:0072683	GO:0072683	T cell extravasation	1/389	14/23328	0.209814773	0.3339	0.21232	F11r	1
GO:0086069	GO:0086069	bundle of His cell to Purkinje myocyte communication	1/389	14/23328	0.209814773	0.3339	0.21232	Gja5	1
GO:0097202	GO:0097202	activation of cysteine-type endopeptidase activity	1/389	14/23328	0.209814773	0.3339	0.21232	Lgmn	1
GO:0097531	GO:0097531	mast cell migration	1/389	14/23328	0.209814773	0.3339	0.21232	Pgf	1
GO:0098911	GO:0098911	regulation of ventricular cardiac muscle cell action pote	1/389	14/23328	0.209814773	0.3339	0.21232	Cav1	1
GO:1902402	GO:1902402	signal transduction involved in mitotic DNA damage ch	1/389	14/23328	0.209814773	0.3339	0.21232	Cdkn1a	1
GO:1902403	GO:1902403	signal transduction involved in mitotic DNA integrity ch	1/389	14/23328	0.209814773	0.3339	0.21232	Cdkn1a	1
GO:1902931	GO:1902931	negative regulation of alcohol biosynthetic process	1/389	14/23328	0.209814773	0.3339	0.21232	Apoe	1
GO:1904396	GO:1904396	regulation of neuromuscular junction development	1/389	14/23328	0.209814773	0.3339	0.21232	Ptn	1
GO:1905879	GO:1905879	regulation of oogenesis	1/389	14/23328	0.209814773	0.3339	0.21232	Pde3a	1
GO:2000347	GO:2000347	positive regulation of hepatocyte proliferation	1/389	14/23328	0.209814773	0.3339	0.21232	Ptn	1
GO:0045137	GO:0045137	development of primary sexual characteristics	6/389	240/23328	0.21304277	0.33868	0.21535	Bmp4/Sfrp	6
GO:0010821	GO:0010821	regulation of mitochondrion organization	4/389	142/23328	0.213065882	0.33868	0.21535	Ier3/Pde2	4
GO:2001056	GO:2001056	positive regulation of cysteine-type endopeptidase acti	4/389	142/23328	0.213065882	0.33868	0.21535	Lgmn/Bok	4
GO:0061351	GO:0061351	neural precursor cell proliferation	5/389	191/23328	0.214878879	0.341	0.21683	Ptn/Prox1	5
GO:0030857	GO:0030857	negative regulation of epithelial cell differentiation	2/389	52/23328	0.215028194	0.341	0.21683	Id1/Cav1	2
GO:0032757	GO:0032757	positive regulation of interleukin-8 production	2/389	52/23328	0.215028194	0.341	0.21683	Lbp/Cd2	2
GO:0043268	GO:0043268	positive regulation of potassium ion transport	2/389	52/23328	0.215028194	0.341	0.21683	Adrb2/Atp	2
GO:0061014	GO:0061014	positive regulation of mRNA catabolic process	2/389	52/23328	0.215028194	0.341	0.21683	Btg2/Zfp3	2
GO:0140353	GO:0140353	lipid export from cell	2/389	52/23328	0.215028194	0.341	0.21683	Spp1/Dab	2
GO:0045638	GO:0045638	negative regulation of myeloid cell differentiation	3/389	96/23328	0.215751987	0.34175	0.21731	Zfp36/Sfrp	3
GO:0060998	GO:0060998	regulation of dendritic spine development	3/389	96/23328	0.215751987	0.34175	0.21731	Reln/Apoe	3
GO:0070555	GO:0070555	response to interleukin-1	3/389	96/23328	0.215751987	0.34175	0.21731	Ccl21a/Eg	3
GO:0030010	GO:0030010	establishment of cell polarity	4/389	143/23328	0.216560402	0.34289	0.21804	Tcf15/Cdh	4
GO:0015698	GO:0015698	inorganic anion transport	4/389	144/23328	0.220070533	0.34504	0.2194	Prnp/Clca	4
GO:0090316	GO:0090316	positive regulation of intracellular protein transport	4/389	144/23328	0.220070533	0.34504	0.2194	Prnp/Ank3	4
GO:0019217	GO:0019217	regulation of fatty acid metabolic process	3/389	97/23328	0.220109846	0.34504	0.2194	Fabp5/Ca	3
GO:0001662	GO:0001662	behavioral fear response	2/389	53/23328	0.221167113	0.34504	0.2194	Apoe/Gja1	2
GO:0045839	GO:0045839	negative regulation of mitotic nuclear division	2/389	53/23328	0.221167113	0.34504	0.2194	Rgcc/Bmp	2
GO:0046676	GO:0046676	negative regulation of insulin secretion	2/389	53/23328	0.221167113	0.34504	0.2194	Ucp2/Sfrp	2
GO:0048701	GO:0048701	embryonic cranial skeleton morphogenesis	2/389	53/23328	0.221167113	0.34504	0.2194	Tbx1/Bmp	2
GO:0052372	GO:0052372	modulation by symbiont of entry into host	2/389	53/23328	0.221167113	0.34504	0.2194	Cav1/Lgal	2
GO:0097720	GO:0097720	calcineurin-mediated signaling	2/389	53/23328	0.221167113	0.34504	0.2194	Prnp/Lmc	2
GO:0001711	GO:0001711	endodermal cell fate commitment	1/389	15/23328	0.222999213	0.34504	0.2194	Sox17	1
GO:0003188	GO:0003188	heart valve formation	1/389	15/23328	0.222999213	0.34504	0.2194	Gja5	1
GO:0006978	GO:0006978	DNA damage response, signal transduction by p53 cla	1/389	15/23328	0.222999213	0.34504	0.2194	Cdkn1a	1
GO:0007379	GO:0007379	segment specification	1/389	15/23328	0.222999213	0.34504	0.2194	Meox1	1

GO:0010561	GO:0010561	negative regulation of glycoprotein biosynthetic proces	1/389	15/23328	0.222999213	0.34504	0.2194	Bace2	1
GO:0010715	GO:0010715	regulation of extracellular matrix disassembly	1/389	15/23328	0.222999213	0.34504	0.2194	Pdpn	1
GO:0010839	GO:0010839	negative regulation of keratinocyte proliferation	1/389	15/23328	0.222999213	0.34504	0.2194	Ctsl	1
GO:0010935	GO:0010935	regulation of macrophage cytokine production	1/389	15/23328	0.222999213	0.34504	0.2194	Cd36	1
GO:0010958	GO:0010958	regulation of amino acid import across plasma membra	1/389	15/23328	0.222999213	0.34504	0.2194	Rgs4	1
GO:0010960	GO:0010960	magnesium ion homeostasis	1/389	15/23328	0.222999213	0.34504	0.2194	Ank3	1
GO:0021819	GO:0021819	layer formation in cerebral cortex	1/389	15/23328	0.222999213	0.34504	0.2194	Reln	1
GO:0030007	GO:0030007	cellular potassium ion homeostasis	1/389	15/23328	0.222999213	0.34504	0.2194	Atp1b1	1
GO:0030299	GO:0030299	intestinal cholesterol absorption	1/389	15/23328	0.222999213	0.34504	0.2194	Cd36	1
GO:0031268	GO:0031268	pseudopodium organization	1/389	15/23328	0.222999213	0.34504	0.2194	Cdc42ep3	1
GO:0032717	GO:0032717	negative regulation of interleukin-8 production	1/389	15/23328	0.222999213	0.34504	0.2194	Anxa1	1
GO:0033690	GO:0033690	positive regulation of osteoblast proliferation	1/389	15/23328	0.222999213	0.34504	0.2194	Fbln5	1
GO:0034162	GO:0034162	toll-like receptor 9 signaling pathway	1/389	15/23328	0.222999213	0.34504	0.2194	Rsad2	1
GO:0034433	GO:0034433	steroid esterification	1/389	15/23328	0.222999213	0.34504	0.2194	Apoe	1
GO:0034434	GO:0034434	sterol esterification	1/389	15/23328	0.222999213	0.34504	0.2194	Apoe	1
GO:0034435	GO:0034435	cholesterol esterification	1/389	15/23328	0.222999213	0.34504	0.2194	Apoe	1
GO:0035745	GO:0035745	T-helper 2 cell cytokine production	1/389	15/23328	0.222999213	0.34504	0.2194	Rsad2	1
GO:0042159	GO:0042159	lipoprotein catabolic process	1/389	15/23328	0.222999213	0.34504	0.2194	Apoe	1
GO:0042178	GO:0042178	xenobiotic catabolic process	1/389	15/23328	0.222999213	0.34504	0.2194	Cyp1a1	1
GO:0042416	GO:0042416	dopamine biosynthetic process	1/389	15/23328	0.222999213	0.34504	0.2194	Epas1	1
GO:0042953	GO:0042953	lipoprotein transport	1/389	15/23328	0.222999213	0.34504	0.2194	Cd36	1
GO:0045591	GO:0045591	positive regulation of regulatory T cell differentiation	1/389	15/23328	0.222999213	0.34504	0.2194	Il2rg	1
GO:0045760	GO:0045760	positive regulation of action potential	1/389	15/23328	0.222999213	0.34504	0.2194	Ank3	1
GO:0046541	GO:0046541	saliva secretion	1/389	15/23328	0.222999213	0.34504	0.2194	Aqp1	1
GO:0048070	GO:0048070	regulation of developmental pigmentation	1/389	15/23328	0.222999213	0.34504	0.2194	Kitl	1
GO:0048680	GO:0048680	positive regulation of axon regeneration	1/389	15/23328	0.222999213	0.34504	0.2194	Ptn	1
GO:0051712	GO:0051712	positive regulation of killing of cells of other organism	1/389	15/23328	0.222999213	0.34504	0.2194	Gapdh	1
GO:0060347	GO:0060347	heart trabecula formation	1/389	15/23328	0.222999213	0.34504	0.2194	Tek	1
GO:0060700	GO:0060700	regulation of ribonuclease activity	1/389	15/23328	0.222999213	0.34504	0.2194	Oasl2	1
GO:0061029	GO:0061029	eyelid development in camera-type eye	1/389	15/23328	0.222999213	0.34504	0.2194	Jun	1
GO:0061051	GO:0061051	positive regulation of cell growth involved in cardiac m	1/389	15/23328	0.222999213	0.34504	0.2194	Slc25a4	1
GO:0061307	GO:0061307	cardiac neural crest cell differentiation involved in hea	1/389	15/23328	0.222999213	0.34504	0.2194	Bmp4	1
GO:0061318	GO:0061318	renal filtration cell differentiation	1/389	15/23328	0.222999213	0.34504	0.2194	Bmp4	1
GO:0072074	GO:0072074	kidney mesenchyme development	1/389	15/23328	0.222999213	0.34504	0.2194	Bmp4	1
GO:0072112	GO:0072112	glomerular visceral epithelial cell differentiation	1/389	15/23328	0.222999213	0.34504	0.2194	Bmp4	1
GO:0072283	GO:0072283	metanephric renal vesicle morphogenesis	1/389	15/23328	0.222999213	0.34504	0.2194	Bmp4	1
GO:0072311	GO:0072311	glomerular epithelial cell differentiation	1/389	15/23328	0.222999213	0.34504	0.2194	Bmp4	1
GO:0072350	GO:0072350	tricarboxylic acid metabolic process	1/389	15/23328	0.222999213	0.34504	0.2194	Ass1	1
GO:0086014	GO:0086014	atrial cardiac muscle cell action potential	1/389	15/23328	0.222999213	0.34504	0.2194	Gja5	1
GO:0086026	GO:0086026	atrial cardiac muscle cell to AV node cell signaling	1/389	15/23328	0.222999213	0.34504	0.2194	Gja5	1
GO:0086066	GO:0086066	atrial cardiac muscle cell to AV node cell communicati	1/389	15/23328	0.222999213	0.34504	0.2194	Gja5	1
GO:0098917	GO:0098917	retrograde trans-synaptic signaling	1/389	15/23328	0.222999213	0.34504	0.2194	Fabp5	1
GO:1900225	GO:1900225	regulation of NLRP3 inflammasome complex assembl	1/389	15/23328	0.222999213	0.34504	0.2194	Cd36	1
GO:1903587	GO:1903587	regulation of blood vessel endothelial cell proliferation	1/389	15/23328	0.222999213	0.34504	0.2194	Aplnr	1
GO:1903789	GO:1903789	regulation of amino acid transmembrane transport	1/389	15/23328	0.222999213	0.34504	0.2194	Rgs4	1
GO:1904294	GO:1904294	positive regulation of ERAD pathway	1/389	15/23328	0.222999213	0.34504	0.2194	Cav1	1
GO:2000811	GO:2000811	negative regulation of anoikis	1/389	15/23328	0.222999213	0.34504	0.2194	Cav1	1
GO:0017148	GO:0017148	negative regulation of translation	4/389	145/23328	0.223595817	0.34583	0.21991	Igfbp5/Btg	4
GO:0021915	GO:0021915	neural tube development	5/389	194/23328	0.223890635	0.34616	0.22011	Sox17/Bm	5
GO:0045833	GO:0045833	negative regulation of lipid metabolic process	3/389	98/23328	0.224485517	0.34668	0.22045	Pdgfb/Apo	3
GO:0072080	GO:0072080	nephron tubule development	3/389	98/23328	0.224485517	0.34668	0.22045	Bmp4/Aqp	3
GO:0099565	GO:0099565	chemical synaptic transmission, postsynaptic	3/389	98/23328	0.224485517	0.34668	0.22045	Reln/Adrb	3
GO:2000241	GO:2000241	regulation of reproductive process	5/389	195/23328	0.226919688	0.35	0.22255	Bmp4/Gja	5
GO:0035637	GO:0035637	multicellular organismal signaling	4/389	146/23328	0.227135798	0.35	0.22255	Gja5/Ank3	4
GO:0002209	GO:0002209	behavioral defense response	2/389	54/23328	0.227320052	0.35	0.22255	Apoe/Gja1	2
GO:0010823	GO:0010823	negative regulation of mitochondrion organization	2/389	54/23328	0.227320052	0.35	0.22255	Ier3/Bok	2
GO:0032355	GO:0032355	response to estradiol	2/389	54/23328	0.227320052	0.35	0.22255	Mmp2/Anx	2
GO:0035272	GO:0035272	exocrine system development	2/389	54/23328	0.227320052	0.35	0.22255	Pdgfb/Tgr	2
GO:1902930	GO:1902930	regulation of alcohol biosynthetic process	2/389	54/23328	0.227320052	0.35	0.22255	Apoe/Dab	2
GO:2001222	GO:2001222	regulation of neuron migration	2/389	54/23328	0.227320052	0.35	0.22255	Reln/Flrt2	2
GO:0006805	GO:0006805	xenobiotic metabolic process	3/389	99/23328	0.228878124	0.35187	0.22374	Cyp26b1/	3
GO:0007200	GO:0007200	phospholipase C-activating G protein-coupled recepto	3/389	99/23328	0.228878124	0.35187	0.22374	Tgm2/S1p	3
GO:0046031	GO:0046031	ADP metabolic process	3/389	99/23328	0.228878124	0.35187	0.22374	Ier3/Aldoa	3
GO:0062014	GO:0062014	negative regulation of small molecule metabolic proce	3/389	99/23328	0.228878124	0.35187	0.22374	Ier3/Apoe	3
GO:0043491	GO:0043491	protein kinase B signaling	5/389	196/23328	0.229960843	0.3534	0.22472	Igfbp5/En	5
GO:0051092	GO:0051092	positive regulation of NF-kappaB transcription factor a	4/389	147/23328	0.230690019	0.35439	0.22534	Cd36/Cav	4
GO:0098659	GO:0098659	inorganic cation import across plasma membrane	3/389	100/23328	0.233286803	0.35486	0.22564	Prnp/Adrb	3
GO:0099587	GO:0099587	inorganic ion import across plasma membrane	3/389	100/23328	0.233286803	0.35486	0.22564	Prnp/Adrb	3
GO:1902882	GO:1902882	regulation of response to oxidative stress	3/389	100/23328	0.233286803	0.35486	0.22564	Cd36/Hsp	3
GO:0016126	GO:0016126	sterol biosynthetic process	2/389	55/23328	0.233484843	0.35486	0.22564	Cyb5r3/Ap	2
GO:0031529	GO:0031529	ruffle organization	2/389	55/23328	0.233484843	0.35486	0.22564	Cav1/Tpm	2
GO:0035036	GO:0035036	sperm-egg recognition	2/389	55/23328	0.233484843	0.35486	0.22564	Cd9/Aldoa	2
GO:0045604	GO:0045604	regulation of epidermal cell differentiation	2/389	55/23328	0.233484843	0.35486	0.22564	Bmp4/Zfp	2
GO:0045773	GO:0045773	positive regulation of axon extension	2/389	55/23328	0.233484843	0.35486	0.22564	Cxcl12/Ap	2
GO:0050775	GO:0050775	positive regulation of dendrite morphogenesis	2/389	55/23328	0.233484843	0.35486	0.22564	Reln/Ptprf	2
GO:0050922	GO:0050922	negative regulation of chemotaxis	2/389	55/23328	0.233484843	0.35486	0.22564	Dusp1/Se	2
GO:1902750	GO:1902750	negative regulation of cell cycle G2/M phase transition	2/389	55/23328	0.233484843	0.35486	0.22564	Ier3/Cdkn	2
GO:0002070	GO:0002070	epithelial cell maturation	1/389	16/23328	0.235964225	0.35486	0.22564	Gja1	1
GO:0002407	GO:0002407	dendritic cell chemotaxis	1/389	16/23328	0.235964225	0.35486	0.22564	Gas6	1
GO:0003222	GO:0003222	ventricular trabecula myocardium morphogenesis	1/389	16/23328	0.235964225	0.35486	0.22564	Eng	1
GO:0006878	GO:0006878	cellular copper ion homeostasis	1/389	16/23328	0.235964225	0.35486	0.22564	Prnp	1
GO:0010454	GO:0010454	negative regulation of cell fate commitment	1/389	16/23328	0.235964225	0.35486	0.22564	Sfrp2	1
GO:0014733	GO:0014733	regulation of skeletal muscle adaptation	1/389	16/23328	0.235964225	0.35486	0.22564	Igfbp5	1
GO:0016322	GO:0016322	neuron remodeling	1/389	16/23328	0.235964225	0.35486	0.22564	Pdgfb	1
GO:0021978	GO:0021978	telencephalon regionalization	1/389	16/23328	0.235964225	0.35486	0.22564	Bmp4	1
GO:0033089	GO:0033089	positive regulation of T cell differentiation in thymus	1/389	16/23328	0.235964225	0.35486	0.22564	Il2rg	1
GO:0035461	GO:0035461	vitamin transmembrane transport	1/389	16/23328	0.235964225	0.35486	0.22564	Scarb1	1
GO:0035641	GO:0035641	locomotory exploration behavior	1/389	16/23328	0.235964225	0.35486	0.22564	Apoe	1
GO:0040034	GO:0040034	regulation of development, heterochronic	1/389	16/23328	0.235964225	0.35486	0.22564	Serpine2	1
GO:0042308	GO:0042308	negative regulation of protein import into nucleus	1/389	16/23328	0.235964225	0.35486	0.22564	Cd36	1
GO:0042772	GO:0042772	DNA damage response, signal transduction resulting in	1/389	16/23328	0.235964225	0.35486	0.22564	Cdkn1a	1
GO:0043923	GO:0043923	positive regulation by host of viral transcription	1/389	16/23328	0.235964225	0.35486	0.22564	Jun	1
GO:0044068	GO:0044068	modulation by symbiont of host cellular process	1/389	16/23328	0.235964225	0.35486	0.22564	Gapdh	1
GO:0044872	GO:0044872	lipoprotein localization	1/389	16/23328	0.235964225	0.35486	0.22564	Cd36	1
GO:0045655	GO:0045655	regulation of monocyte differentiation	1/389	16/23328	0.235964225	0.35486	0.22564	Jun	1
GO:0045820	GO:0045820	negative regulation of glycolytic process	1/389	16/23328	0.235964225	0.35486	0.22564	Ier3	1
GO:0046549	GO:0046549	retinal cone cell development	1/389	16/23328	0.235964225	0.35486	0.22564	Thy1	1
GO:0048681	GO:0048681	negative regulation of axon regeneration	1/389	16/23328	0.235964225	0.35486	0.22564	Klk8	1
GO:0051044	GO:0051044	positive regulation of membrane protein ectodomain p	1/389	16/23328	0.235964225	0.35486	0.22564	Apoe	1
GO:0051151	GO:0051151	negative regulation of smooth muscle cell differentiatic	1/389	16/23328	0.235964225	0.35486	0.22564	Pdgfb	1
GO:0051386	GO:0051386	regulation of neurotrophin TRK receptor signaling path	1/389	16/23328	0.235964225	0.35486	0.22564	Ptprf	1
GO:0051709	GO:0051709	regulation of killing of cells of other organism	1/389	16/23328	0.235964225	0.35486	0.22564	Gapdh	1
GO:0055089	GO:0055089	fatty acid homeostasis	1/389	16/23328	0.235964225	0.35486	0.22564	Apoe	1
GO:0061548	GO:0061548	ganglion development	1/389	16/23328	0.235964225	0.35486	0.22564	Nrp2	1
GO:0070166	GO:0070166	enamel mineralization	1/389	16/23328	0.235964225	0.35486	0.22564	Tbx1	1
GO:0070572	GO:0070572	positive regulation of neuron projection regeneration	1/389	16/23328	0.235964225	0.35486	0.22564	Ptn	1
GO:0070885	GO:0070885	negative regulation of calcineurin-NFAT signaling casc	1/389	16/23328	0.235964225	0.35486	0.22564	Prnp	1
GO:0071599	GO:0071599	otic vesicle development	1/389	16/23328	0.235964225	0.35486	0.22564	Tbx1	1
GO:0071731	GO:0071731	response to nitric oxide	1/389	16/23328	0.235964225	0.35486	0.22564	Aqp1	1
GO:0072498	GO:0072498	embryonic skeletal joint development	1/389	16/23328	0.235964225	0.35486	0.22564	Bmp4	1
GO:0072567	GO:0072567	chemokine (C-X-C motif) ligand 2 production	1/389	16/23328	0.235964225	0.35486	0.22564	Lpl	1
GO:0072673	GO:0072673	lamellipodium morphogenesis	1/389	16/23328	0.235964225	0.35486	0.22564	Pdpn	1
GO:0090051	GO:0090051	negative regulation of cell migration involved in sprouti	1/389	16/23328	0.235964225	0.35486	0.22564	Mmrn2	1
GO:0090141	GO:0090141	positive regulation of mitochondrial fission	1/389	16/23328	0.235964225	0.35486	0.22564	Dcn	1
GO:0097094	GO:0097094	craniofacial suture morphogenesis	1/389	16/23328	0.235964225	0.35486	0.22564	Bmp4	1
GO:0106057	GO:0106057	negative regulation of calcineurin-mediated signaling	1/389	16/23328	0.235964225	0.35486	0.22564	Prnp	1
GO:1900037	GO:1900037	regulation of cellular response to hypoxia	1/389	16/23328	0.235964225	0.35486	0.22564	Cd34	1
GO:1903729	GO:1903729	regulation of plasma membrane organization	1/389	16/23328	0.235964225	0.35486	0.22564	S100a9	1
GO:1904590	GO:1904590	negative regulation of protein import	1/389	16/23328	0.235964225	0.35486	0.22564	Cd36	1
GO:1905048	GO:1905048	regulation of metallopeptidase activity	1/389	16/23328	0.235964225	0.35486	0.22564	Timp3	1
GO:2000341	GO:2000341	regulation of chemokine (C-X-C motif) ligand 2 product	1/389	16/23328	0.235964225	0.35486	0.22564	Lpl	1
GO:2000647	GO:2000647	negative regulation of stem cell proliferation	1/389	16/23328	0.235964225	0.35486	0.22564	Ptn	1
GO:2001053	GO:2001053	regulation of mesenchymal cell apoptotic process	1/389	16/23328	0.235964225	0.35486	0.22564	Tbx1	1
GO:1990138	GO:1990138	neuron projection extension	5/389	198/23328	0.236078336	0.3549	0.22567	Cxcl12/Nr	5
GO:0043161	GO:0043161	proteasome-mediated ubiquitin-dependent protein cata	9/389	405/23328	0.236657861	0.35564	0.22614	Cav1/Psm	9
GO:0006906	GO:0006906	vesicle fusion	3/389	101/23328	0.237710697	0.35709	0.22706	Anxa2/An	3
GO:0046660	GO:0046660	female sex differentiation	4/389	149/23328	0.237839368	0.35715	0.2271	Bmp4/Mm	4
GO:0015872	GO:0015872	dopamine transport	2/389	56/23328	0.239659385	0.35883	0.22817	Cxcl12/Sr	2
GO:0017145	GO:0017145	stem cell division	2/389	56/23328	0.239659385	0.35883	0.22817	Sox17/Sfr	2

GO:0021587	GO:0021587	cerebellum morphogenesis	2/389	56/23328	0.239659385	0.35883	0.22817	Serpine2/I	2
GO:0071715	GO:0071715	icosanoid transport	2/389	56/23328	0.239659385	0.35883	0.22817	Nmb/Anxa	2
GO:1901571	GO:1901571	fatty acid derivative transport	2/389	56/23328	0.239659385	0.35883	0.22817	Nmb/Anxa	2
GO:1903350	GO:1903350	response to dopamine	2/389	56/23328	0.239659385	0.35883	0.22817	Rgs4/Htr2	2
GO:1903351	GO:1903351	cellular response to dopamine	2/389	56/23328	0.239659385	0.35883	0.22817	Rgs4/Htr2	2
GO:1903533	GO:1903533	regulation of protein targeting	2/389	56/23328	0.239659385	0.35883	0.22817	Prnp/Ank3	2
GO:0031330	GO:0031330	negative regulation of cellular catabolic process	6/389	250/23328	0.239886299	0.35904	0.2283	Timp3/Anx	6
GO:0007006	GO:0007006	mitochondrial membrane organization	3/389	102/23328	0.242148958	0.36216	0.23028	Ier3/Slc25	3
GO:1901989	GO:1901989	positive regulation of cell cycle phase transition	3/389	102/23328	0.242148958	0.36216	0.23028	Rgcc/Anxa	3
GO:0071805	GO:0071805	potassium ion transmembrane transport	5/389	200/23328	0.24224087	0.36216	0.23029	Prnp/Rgs4	5
GO:0098657	GO:0098657	import into cell	6/389	251/23328	0.242625964	0.3626	0.23057	Cd36/Prnp	6
GO:0032869	GO:0032869	cellular response to insulin stimulus	5/389	201/23328	0.245338326	0.36344	0.2311	Ptprf/Socs	5
GO:0002067	GO:0002067	glandular epithelial cell differentiation	2/389	57/23328	0.245841646	0.36344	0.2311	Bmp4/Pro	2
GO:0006584	GO:0006584	catecholamine metabolic process	2/389	57/23328	0.245841646	0.36344	0.2311	Epas1/Ly6	2
GO:0009712	GO:0009712	catechol-containing compound metabolic process	2/389	57/23328	0.245841646	0.36344	0.2311	Epas1/Ly6	2
GO:0050879	GO:0050879	multicellular organismal movement	2/389	57/23328	0.245841646	0.36344	0.2311	Adrb2/Cor	2
GO:0050881	GO:0050881	musculoskeletal movement	2/389	57/23328	0.245841646	0.36344	0.2311	Adrb2/Cor	2
GO:0140115	GO:0140115	export across plasma membrane	2/389	57/23328	0.245841646	0.36344	0.2311	Atp1b1/Gj	2
GO:1901292	GO:1901292	nucleoside phosphate catabolic process	2/389	57/23328	0.245841646	0.36344	0.2311	Nudt4/Pde	2
GO:0006821	GO:0006821	chloride transport	3/389	103/23328	0.246600749	0.36344	0.2311	Prnp/Clca	3
GO:0007173	GO:0007173	epidermal growth factor receptor signaling pathway	3/389	103/23328	0.246600749	0.36344	0.2311	Ptprf/Efen	3
GO:0071466	GO:0071466	cellular response to xenobiotic stimulus	3/389	103/23328	0.246600749	0.36344	0.2311	Cyp26b1/	3
GO:0018209	GO:0018209	peptidyl-serine modification	8/389	357/23328	0.247240081	0.36344	0.2311	Sfrp2/Cav	8
GO:0046034	GO:0046034	ATP metabolic process	6/389	253/23328	0.248132974	0.36344	0.2311	Cox4i2/At	6
GO:0002689	GO:0002689	negative regulation of leukocyte chemotaxis	1/389	17/23328	0.248713449	0.36344	0.2311	Dusp1	1
GO:0003159	GO:0003159	morphogenesis of an endothelium	1/389	17/23328	0.248713449	0.36344	0.2311	Bmp4	1
GO:0003198	GO:0003198	epithelial to mesenchymal transition involved in endoca	1/389	17/23328	0.248713449	0.36344	0.2311	Eng	1
GO:0008272	GO:0008272	sulfate transport	1/389	17/23328	0.248713449	0.36344	0.2311	Slc26a10	1
GO:0010310	GO:0010310	regulation of hydrogen peroxide metabolic process	1/389	17/23328	0.248713449	0.36344	0.2311	Duoxa2	1
GO:0010752	GO:0010752	regulation of cGMP-mediated signaling	1/389	17/23328	0.248713449	0.36344	0.2311	Pde2a	1
GO:0015701	GO:0015701	bicarbonate transport	1/389	17/23328	0.248713449	0.36344	0.2311	Car4	1
GO:0021854	GO:0021854	hypothalamus development	1/389	17/23328	0.248713449	0.36344	0.2311	Nrp2	1
GO:0030730	GO:0030730	sequestering of triglyceride	1/389	17/23328	0.248713449	0.36344	0.2311	Lpl	1
GO:0032570	GO:0032570	response to progesterone	1/389	17/23328	0.248713449	0.36344	0.2311	Cav1	1
GO:0032780	GO:0032780	negative regulation of ATPase activity	1/389	17/23328	0.248713449	0.36344	0.2311	Tnnt2	1
GO:0034755	GO:0034755	iron ion transmembrane transport	1/389	17/23328	0.248713449	0.36344	0.2311	Steap4	1
GO:0035729	GO:0035729	cellular response to hepatocyte growth factor stimulus	1/389	17/23328	0.248713449	0.36344	0.2311	Lgmn	1
GO:0036035	GO:0036035	osteoclast development	1/389	17/23328	0.248713449	0.36344	0.2311	Anxa2	1
GO:0042074	GO:0042074	cell migration involved in gastrulation	1/389	17/23328	0.248713449	0.36344	0.2311	Sox17	1
GO:0042670	GO:0042670	retinal cone cell differentiation	1/389	17/23328	0.248713449	0.36344	0.2311	Thy1	1
GO:0043249	GO:0043249	erythrocyte maturation	1/389	17/23328	0.248713449	0.36344	0.2311	Klf2	1
GO:0044546	GO:0044546	NLRP3 inflammasome complex assembly	1/389	17/23328	0.248713449	0.36344	0.2311	Cd36	1
GO:0044794	GO:0044794	positive regulation by host of viral process	1/389	17/23328	0.248713449	0.36344	0.2311	Apoe	1
GO:0045064	GO:0045064	T-helper 2 cell differentiation	1/389	17/23328	0.248713449	0.36344	0.2311	Anxa1	1
GO:0045198	GO:0045198	establishment of epithelial cell apical/basal polarity	1/389	17/23328	0.248713449	0.36344	0.2311	Tcf15	1
GO:0045346	GO:0045346	regulation of MHC class II biosynthetic process	1/389	17/23328	0.248713449	0.36344	0.2311	Il33	1
GO:0045579	GO:0045579	positive regulation of B cell differentiation	1/389	17/23328	0.248713449	0.36344	0.2311	Il2rg	1
GO:0048853	GO:0048853	forebrain morphogenesis	1/389	17/23328	0.248713449	0.36344	0.2311	Duox2	1
GO:0051004	GO:0051004	regulation of lipoprotein lipase activity	1/389	17/23328	0.248713449	0.36344	0.2311	Gpihbp1	1
GO:0055012	GO:0055012	ventricular cardiac muscle cell differentiation	1/389	17/23328	0.248713449	0.36344	0.2311	Prox1	1
GO:0055070	GO:0055070	copper ion homeostasis	1/389	17/23328	0.248713449	0.36344	0.2311	Prnp	1
GO:0060253	GO:0060253	negative regulation of glial cell proliferation	1/389	17/23328	0.248713449	0.36344	0.2311	Ptn	1
GO:0060413	GO:0060413	atrial septum morphogenesis	1/389	17/23328	0.248713449	0.36344	0.2311	Gja5	1
GO:0060644	GO:0060644	mammary gland epithelial cell differentiation	1/389	17/23328	0.248713449	0.36344	0.2311	Id2	1
GO:0060788	GO:0060788	ectodermal placode formation	1/389	17/23328	0.248713449	0.36344	0.2311	Prox1	1
GO:0061154	GO:0061154	endothelial tube morphogenesis	1/389	17/23328	0.248713449	0.36344	0.2311	Bmp4	1
GO:0061179	GO:0061179	negative regulation of insulin secretion involved in cell	1/389	17/23328	0.248713449	0.36344	0.2311	Ucp2	1
GO:0070206	GO:0070206	protein trimerization	1/389	17/23328	0.248713449	0.36344	0.2311	Steap4	1
GO:0070243	GO:0070243	regulation of thymocyte apoptotic process	1/389	17/23328	0.248713449	0.36344	0.2311	Bmp4	1
GO:0070262	GO:0070262	peptidyl-serine dephosphorylation	1/389	17/23328	0.248713449	0.36344	0.2311	Dusp1	1
GO:0070886	GO:0070886	positive regulation of calcineurin-NFAT signaling casca	1/389	17/23328	0.248713449	0.36344	0.2311	Lmcd1	1
GO:0071360	GO:0071360	cellular response to exogenous dsRNA	1/389	17/23328	0.248713449	0.36344	0.2311	Cav1	1
GO:0071697	GO:0071697	ectodermal placode morphogenesis	1/389	17/23328	0.248713449	0.36344	0.2311	Prox1	1
GO:0072234	GO:0072234	metanephric nephron tubule development	1/389	17/23328	0.248713449	0.36344	0.2311	Aqp1	1
GO:0072672	GO:0072672	neutrophil extravasation	1/389	17/23328	0.248713449	0.36344	0.2311	Pecam1	1
GO:0090185	GO:0090185	negative regulation of kidney development	1/389	17/23328	0.248713449	0.36344	0.2311	Bmp4	1
GO:0097152	GO:0097152	mesenchymal cell apoptotic process	1/389	17/23328	0.248713449	0.36344	0.2311	Tbx1	1
GO:0099640	GO:0099640	axo-dendritic protein transport	1/389	17/23328	0.248713449	0.36344	0.2311	Hspb1	1
GO:0106058	GO:0106058	positive regulation of calcineurin-mediated signaling	1/389	17/23328	0.248713449	0.36344	0.2311	Lmcd1	1
GO:1902176	GO:1902176	negative regulation of oxidative stress-induced intrinsi	1/389	17/23328	0.248713449	0.36344	0.2311	Hspb1	1
GO:1905331	GO:1905331	negative regulation of morphogenesis of an epithelium	1/389	17/23328	0.248713449	0.36344	0.2311	Bmp4	1
GO:2000047	GO:2000047	regulation of cell-cell adhesion mediated by cadherin	1/389	17/23328	0.248713449	0.36344	0.2311	Rgcc	1
GO:2001044	GO:2001044	regulation of integrin-mediated signaling pathway	1/389	17/23328	0.248713449	0.36344	0.2311	Dab2	1
GO:0016358	GO:0016358	dendrite development	7/389	306/23328	0.250928269	0.36556	0.23245	Reln/Ptn/L	7
GO:0001657	GO:0001657	ureteric bud development	3/389	104/23328	0.251065239	0.36556	0.23245	Bmp4/Pgf	3
GO:0009135	GO:0009135	purine nucleoside diphosphate metabolic process	3/389	104/23328	0.251065239	0.36556	0.23245	Ier3/Aldoa	3
GO:0009179	GO:0009179	purine ribonucleoside diphosphate metabolic process	3/389	104/23328	0.251065239	0.36556	0.23245	Ier3/Aldoa	3
GO:0019731	GO:0019731	antibacterial humoral response	3/389	104/23328	0.251065239	0.36556	0.23245	B2m/Trf/A	3
GO:0034766	GO:0034766	negative regulation of ion transmembrane transport	3/389	104/23328	0.251065239	0.36556	0.23245	Rgs4/Ank	3
GO:0052126	GO:0052126	movement in host environment	3/389	104/23328	0.251065239	0.36556	0.23245	Cav1/Gas	3
GO:0061326	GO:0061326	renal tubule development	3/389	104/23328	0.251065239	0.36556	0.23245	Bmp4/Aqp	3
GO:0072163	GO:0072163	mesonephric epithelium development	3/389	104/23328	0.251065239	0.36556	0.23245	Bmp4/Pgf	3
GO:0072164	GO:0072164	mesonephric tubule development	3/389	104/23328	0.251065239	0.36556	0.23245	Bmp4/Pgf	3
GO:0002285	GO:0002285	lymphocyte activation involved in immune response	5/389	203/23328	0.251564212	0.36603	0.23274	Fgl2/Gadd	5
GO:0009743	GO:0009743	response to carbohydrate	5/389	203/23328	0.251564212	0.36603	0.23274	Ucp2/Vca	5
GO:0006493	GO:0006493	protein O-linked glycosylation	2/389	58/23328	0.252029657	0.36644	0.23301	Galnt18/G	2
GO:0042596	GO:0042596	fear response	2/389	58/23328	0.252029657	0.36644	0.23301	Apoe/Gja1	2
GO:0001708	GO:0001708	cell fate specification	3/389	105/23328	0.255541611	0.37142	0.23617	Tbx1/Sox1	3
GO:0007338	GO:0007338	single fertilization	4/389	154/23328	0.255930312	0.37185	0.23645	Cd9/Hoxd	4
GO:0044770	GO:0044770	cell cycle phase transition	9/389	415/23328	0.258107318	0.37424	0.23797	Rgcc/Cdk	9
GO:0001836	GO:0001836	release of cytochrome c from mitochondria	2/389	59/23328	0.258221512	0.37424	0.23797	Jun/Bok	2
GO:0007528	GO:0007528	neuromuscular junction development	2/389	59/23328	0.258221512	0.37424	0.23797	Ptn/Tnc	2
GO:0007588	GO:0007588	excretion	2/389	59/23328	0.258221512	0.37424	0.23797	Stc1/Dab2	2
GO:0044773	GO:0044773	mitotic DNA damage checkpoint	2/389	59/23328	0.258221512	0.37424	0.23797	Ier3/Cdkn	2
GO:0001838	GO:0001838	embryonic epithelial tube formation	4/389	155/23328	0.259582221	0.37424	0.23797	Bmp4/Sfrp	4
GO:0071322	GO:0071322	cellular response to carbohydrate stimulus	4/389	155/23328	0.259582221	0.37424	0.23797	Ucp2/Vca	4
GO:0006165	GO:0006165	nucleoside diphosphate phosphorylation	3/389	106/23328	0.260029054	0.37424	0.23797	Ier3/Aldoa	3
GO:0051251	GO:0051251	positive regulation of lymphocyte activation	9/389	416/23328	0.260286524	0.37424	0.23797	Thy1/Cav1	9
GO:0001991	GO:0001991	regulation of systemic arterial blood pressure by circula	1/389	18/23328	0.261250469	0.37424	0.23797	Gja5	1
GO:0002283	GO:0002283	neutrophil activation involved in immune response	1/389	18/23328	0.261250469	0.37424	0.23797	Anxa3	1
GO:0003128	GO:0003128	heart field specification	1/389	18/23328	0.261250469	0.37424	0.23797	Bmp4	1
GO:0009886	GO:0009886	post-embryonic animal morphogenesis	1/389	18/23328	0.261250469	0.37424	0.23797	Flt1	1
GO:0010867	GO:0010867	positive regulation of triglyceride biosynthetic process	1/389	18/23328	0.261250469	0.37424	0.23797	Scarb1	1
GO:0016540	GO:0016540	protein autoprocessing	1/389	18/23328	0.261250469	0.37424	0.23797	Ctsl	1
GO:0031571	GO:0031571	mitotic G1 DNA damage checkpoint	1/389	18/23328	0.261250469	0.37424	0.23797	Cdkn1a	1
GO:0032674	GO:0032674	regulation of interleukin-5 production	1/389	18/23328	0.261250469	0.37424	0.23797	Il33	1
GO:0032968	GO:0032968	positive regulation of transcription elongation from RN	1/389	18/23328	0.261250469	0.37424	0.23797	Dab2	1
GO:0033604	GO:0033604	negative regulation of catecholamine secretion	1/389	18/23328	0.261250469	0.37424	0.23797	Ptgs1	1
GO:0033631	GO:0033631	cell-cell adhesion mediated by integrin	1/389	18/23328	0.261250469	0.37424	0.23797	Plpp3	1
GO:0042448	GO:0042448	progesterone metabolic process	1/389	18/23328	0.261250469	0.37424	0.23797	Egr1	1
GO:0042487	GO:0042487	regulation of odontogenesis of dentin-containing tooth	1/389	18/23328	0.261250469	0.37424	0.23797	Bmp4	1
GO:0045342	GO:0045342	MHC class II biosynthetic process	1/389	18/23328	0.261250469	0.37424	0.23797	Il33	1
GO:0045779	GO:0045779	negative regulation of bone resorption	1/389	18/23328	0.261250469	0.37424	0.23797	Cd38	1
GO:0048268	GO:0048268	clathrin coat assembly	1/389	18/23328	0.261250469	0.37424	0.23797	Dab2	1
GO:0050872	GO:0050872	white fat cell differentiation	1/389	18/23328	0.261250469	0.37424	0.23797	Fabp4	1
GO:0051131	GO:0051131	chaperone-mediated protein complex assembly	1/389	18/23328	0.261250469	0.37424	0.23797	Clu	1
GO:0060192	GO:0060192	negative regulation of lipase activity	1/389	18/23328	0.261250469	0.37424	0.23797	Anxa1	1
GO:0060572	GO:0060572	morphogenesis of an epithelial bud	1/389	18/23328	0.261250469	0.37424	0.23797	Bmp4	1
GO:0060947	GO:0060947	cardiac vascular smooth muscle cell differentiation	1/389	18/23328	0.261250469	0.37424	0.23797	Pdgfb	1
GO:0061158	GO:0061158	3'-UTR-mediated mRNA destabilization	1/389	18/23328	0.261250469	0.37424	0.23797	Zfp36	1
GO:0071696	GO:0071696	ectodermal placode development	1/389	18/23328	0.261250469	0.37424	0.23797	Prox1	1
GO:0072077	GO:0072077	renal vesicle morphogenesis	1/389	18/23328	0.261250469	0.37424	0.23797	Bmp4	1
GO:0086011	GO:0086011	membrane repolarization during action potential	1/389	18/23328	0.261250469	0.37424	0.23797	Cav1	1
GO:0097091	GO:0097091	synaptic vesicle clustering	1/389	18/23328	0.261250469	0.37424	0.23797	Pcdh17	1
GO:0098856	GO:0098856	intestinal lipid absorption	1/389	18/23328	0.261250469	0.37424	0.23797	Cd36	1
GO:0099150	GO:0099150	regulation of postsynaptic specialization assembly	1/389	18/23328	0.261250469	0.37424	0.23797	Gap43	1
GO:0150052	GO:0150052	regulation of postsynapse assembly	1/389	18/23328	0.261250469	0.37424	0.23797	Gap43	1
GO:1901673	GO:1901673	regulation of mitotic spindle assembly	1/389	18/23328	0.261250469	0.37424	0.23797	Hspa1a	1
GO:2000846	GO:2000846	regulation of corticosteroid hormone secretion	1/389	18/23328	0.261250469	0.37424	0.23797	Dab2	1

GO:0007595	GO:0007595	lactation	2/389	60/23328	0.264415367	0.37774	0.24019	Cav1/Gja1	2
GO:0021575	GO:0021575	hindbrain morphogenesis	2/389	60/23328	0.264415367	0.37774	0.24019	Serpine2/I	2
GO:0051893	GO:0051893	regulation of focal adhesion assembly	2/389	60/23328	0.264415367	0.37774	0.24019	Thy1/Tek	2
GO:0060324	GO:0060324	face development	2/389	60/23328	0.264415367	0.37774	0.24019	Tbx1/Mmp	2
GO:0090109	GO:0090109	regulation of cell-substrate junction assembly	2/389	60/23328	0.264415367	0.37774	0.24019	Thy1/Tek	2
GO:1905517	GO:1905517	macrophage migration	2/389	60/23328	0.264415367	0.37774	0.24019	Cd9/Cd20	2
GO:0001823	GO:0001823	mesonephros development	3/389	107/23328	0.264526769	0.37774	0.24019	Bmp4/Pgf	3
GO:0019395	GO:0019395	fatty acid oxidation	3/389	107/23328	0.264526769	0.37774	0.24019	Cd36/C1q	3
GO:0090174	GO:0090174	organelle membrane fusion	3/389	107/23328	0.264526769	0.37774	0.24019	Anxa2/An	3
GO:0009185	GO:0009185	ribonucleoside diphosphate metabolic process	3/389	108/23328	0.269033965	0.38377	0.24403	Ier3/Aldoa	3
GO:0046939	GO:0046939	nucleotide phosphorylation	3/389	108/23328	0.269033965	0.38377	0.24403	Ier3/Aldoa	3
GO:0048704	GO:0048704	embryonic skeletal system morphogenesis	3/389	108/23328	0.269033965	0.38377	0.24403	Hoxd9/Tb	3
GO:0010812	GO:0010812	negative regulation of cell-substrate adhesion	2/389	61/23328	0.270609438	0.3846	0.24455	Actn4/Lga	2
GO:0035249	GO:0035249	synaptic transmission, glutamatergic	3/389	109/23328	0.273549863	0.3846	0.24455	Reln/Serp	3
GO:0051209	GO:0051209	release of sequestered calcium ion into cytosol	3/389	109/23328	0.273549863	0.3846	0.24455	Thy1/Apln	3
GO:1905477	GO:1905477	positive regulation of protein localization to membrane	3/389	109/23328	0.273549863	0.3846	0.24455	Ptn/Prnp/	3
GO:0003180	GO:0003180	aortic valve morphogenesis	1/389	19/23328	0.273578807	0.3846	0.24455	Bmp4	1
GO:0006525	GO:0006525	arginine metabolic process	1/389	19/23328	0.273578807	0.3846	0.24455	Ass1	1
GO:0006656	GO:0006656	phosphatidylcholine biosynthetic process	1/389	19/23328	0.273578807	0.3846	0.24455	Fabp5	1
GO:0010623	GO:0010623	programmed cell death involved in cell development	1/389	19/23328	0.273578807	0.3846	0.24455	Kitl	1
GO:0010766	GO:0010766	negative regulation of sodium ion transport	1/389	19/23328	0.273578807	0.3846	0.24455	Serpine2	1
GO:0014061	GO:0014061	regulation of norepinephrine secretion	1/389	19/23328	0.273578807	0.3846	0.24455	Ptgs1	1
GO:0016045	GO:0016045	detection of bacterium	1/389	19/23328	0.273578807	0.3846	0.24455	Pglyrp1	1
GO:0030050	GO:0030050	vesicle transport along actin filament	1/389	19/23328	0.273578807	0.3846	0.24455	Actn4	1
GO:0030502	GO:0030502	negative regulation of bone mineralization	1/389	19/23328	0.273578807	0.3846	0.24455	Srgn	1
GO:0032274	GO:0032274	gonadotropin secretion	1/389	19/23328	0.273578807	0.3846	0.24455	Gja1	1
GO:0032634	GO:0032634	interleukin-5 production	1/389	19/23328	0.273578807	0.3846	0.24455	Il33	1
GO:0032656	GO:0032656	regulation of interleukin-13 production	1/389	19/23328	0.273578807	0.3846	0.24455	Il33	1
GO:0032700	GO:0032700	negative regulation of interleukin-17 production	1/389	19/23328	0.273578807	0.3846	0.24455	Prnp	1
GO:0034138	GO:0034138	toll-like receptor 3 signaling pathway	1/389	19/23328	0.273578807	0.3846	0.24455	Cav1	1
GO:0035728	GO:0035728	response to hepatocyte growth factor	1/389	19/23328	0.273578807	0.3846	0.24455	Lgmn	1
GO:0035930	GO:0035930	corticosteroid hormone secretion	1/389	19/23328	0.273578807	0.3846	0.24455	Dab2	1
GO:0039531	GO:0039531	regulation of viral-induced cytoplasmic pattern recogni	1/389	19/23328	0.273578807	0.3846	0.24455	Tspan6	1
GO:0043373	GO:0043373	CD4-positive, alpha-beta T cell lineage commitment	1/389	19/23328	0.273578807	0.3846	0.24455	Ctsl	1
GO:0044539	GO:0044539	long-chain fatty acid import into cell	1/389	19/23328	0.273578807	0.3846	0.24455	Cd36	1
GO:0044783	GO:0044783	G1 DNA damage checkpoint	1/389	19/23328	0.273578807	0.3846	0.24455	Cdkn1a	1
GO:0045540	GO:0045540	regulation of cholesterol biosynthetic process	1/389	19/23328	0.273578807	0.3846	0.24455	Apoe	1
GO:0045837	GO:0045837	negative regulation of membrane potential	1/389	19/23328	0.273578807	0.3846	0.24455	Ptn	1
GO:0046068	GO:0046068	cGMP metabolic process	1/389	19/23328	0.273578807	0.3846	0.24455	Pde2a	1
GO:0046827	GO:0046827	positive regulation of protein export from nucleus	1/389	19/23328	0.273578807	0.3846	0.24455	Gas6	1
GO:0048243	GO:0048243	norepinephrine secretion	1/389	19/23328	0.273578807	0.3846	0.24455	Ptgs1	1
GO:0050774	GO:0050774	negative regulation of dendrite morphogenesis	1/389	19/23328	0.273578807	0.3846	0.24455	Id1	1
GO:0050884	GO:0050884	neuromuscular process controlling posture	1/389	19/23328	0.273578807	0.3846	0.24455	Tcf15	1
GO:0051238	GO:0051238	sequestering of metal ion	1/389	19/23328	0.273578807	0.3846	0.24455	Lcn2	1
GO:0055003	GO:0055003	cardiac myofibril assembly	1/389	19/23328	0.273578807	0.3846	0.24455	Prox1	1
GO:0060044	GO:0060044	negative regulation of cardiac muscle cell proliferation	1/389	19/23328	0.273578807	0.3846	0.24455	Gja1	1
GO:0060221	GO:0060221	retinal rod cell differentiation	1/389	19/23328	0.273578807	0.3846	0.24455	Ptn	1
GO:0061081	GO:0061081	positive regulation of myeloid leukocyte cytokine produ	1/389	19/23328	0.273578807	0.3846	0.24455	Cd36	1
GO:0106118	GO:0106118	regulation of sterol biosynthetic process	1/389	19/23328	0.273578807	0.3846	0.24455	Apoe	1
GO:0140354	GO:0140354	lipid import into cell	1/389	19/23328	0.273578807	0.3846	0.24455	Cd36	1
GO:1902259	GO:1902259	regulation of delayed rectifier potassium channel activi	1/389	19/23328	0.273578807	0.3846	0.24455	Ank3	1
GO:1903960	GO:1903960	negative regulation of anion transmembrane transport	1/389	19/23328	0.273578807	0.3846	0.24455	Rgs4	1
GO:2000251	GO:2000251	positive regulation of actin cytoskeleton reorganization	1/389	19/23328	0.273578807	0.3846	0.24455	Tek	1
GO:2000641	GO:2000641	regulation of early endosome to late endosome transp	1/389	19/23328	0.273578807	0.3846	0.24455	Dab2	1
GO:0010171	GO:0010171	body morphogenesis	2/389	62/23328	0.276802002	0.38819	0.24684	Tbx1/Mmp	2
GO:0019080	GO:0019080	viral gene expression	2/389	62/23328	0.276802002	0.38819	0.24684	Zfp36/Jun	2
GO:0032387	GO:0032387	negative regulation of intracellular transport	2/389	62/23328	0.276802002	0.38819	0.24684	Cd36/Crya	2
GO:0048512	GO:0048512	circadian behavior	2/389	62/23328	0.276802002	0.38819	0.24684	Id2/Egr1	2
GO:0050854	GO:0050854	regulation of antigen receptor-mediated signaling path	2/389	62/23328	0.276802002	0.38819	0.24684	Thy1/Prnp	2
GO:0050982	GO:0050982	detection of mechanical stimulus	2/389	62/23328	0.276802002	0.38819	0.24684	Serpine2/	2
GO:0098930	GO:0098930	axonal transport	2/389	62/23328	0.276802002	0.38819	0.24684	Hspb1/Ds	2
GO:0000910	GO:0000910	cytokinesis	4/389	160/23328	0.277986118	0.38957	0.24772	Ank3/Dstr	4
GO:0009410	GO:0009410	response to xenobiotic stimulus	3/389	110/23328	0.278073694	0.38957	0.24772	Cyp26b1/	3
GO:0051283	GO:0051283	negative regulation of sequestering of calcium ion	3/389	110/23328	0.278073694	0.38957	0.24772	Thy1/Apln	3
GO:0006661	GO:0006661	phosphatidylinositol biosynthetic process	2/389	63/23328	0.282991388	0.39563	0.25157	Pdgfb/Htr	2
GO:0060038	GO:0060038	cardiac muscle cell proliferation	2/389	63/23328	0.282991388	0.39563	0.25157	Ncam1/Gj	2
GO:0060999	GO:0060999	positive regulation of dendritic spine development	2/389	63/23328	0.282991388	0.39563	0.25157	Reln/Apoe	2
GO:0150116	GO:0150116	regulation of cell-substrate junction organization	2/389	63/23328	0.282991388	0.39563	0.25157	Thy1/Tek	2
GO:0072175	GO:0072175	epithelial tube formation	4/389	162/23328	0.285406955	0.39563	0.25157	Bmp4/Sfrp	4
GO:0002577	GO:0002577	regulation of antigen processing and presentation	1/389	20/23328	0.285701929	0.39563	0.25157	Fgl2	1
GO:0002755	GO:0002755	MyD88-dependent toll-like receptor signaling pathway	1/389	20/23328	0.285701929	0.39563	0.25157	Irf7	1
GO:0006699	GO:0006699	bile acid biosynthetic process	1/389	20/23328	0.285701929	0.39563	0.25157	Prox1	1
GO:0006883	GO:0006883	cellular sodium ion homeostasis	1/389	20/23328	0.285701929	0.39563	0.25157	Atp1b1	1
GO:0007342	GO:0007342	fusion of sperm to egg plasma membrane involved in s	1/389	20/23328	0.285701929	0.39563	0.25157	Cd9	1
GO:0010763	GO:0010763	positive regulation of fibroblast migration	1/389	20/23328	0.285701929	0.39563	0.25157	Akap12	1
GO:0030002	GO:0030002	cellular anion homeostasis	1/389	20/23328	0.285701929	0.39563	0.25157	Stc1	1
GO:0030320	GO:0030320	cellular monovalent inorganic anion homeostasis	1/389	20/23328	0.285701929	0.39563	0.25157	Stc1	1
GO:0030852	GO:0030852	regulation of granulocyte differentiation	1/389	20/23328	0.285701929	0.39563	0.25157	Rbp1	1
GO:0032495	GO:0032495	response to muramyl dipeptide	1/389	20/23328	0.285701929	0.39563	0.25157	Vim	1
GO:0033033	GO:0033033	negative regulation of myeloid cell apoptotic process	1/389	20/23328	0.285701929	0.39563	0.25157	Kitl	1
GO:0034143	GO:0034143	regulation of toll-like receptor 4 signaling pathway	1/389	20/23328	0.285701929	0.39563	0.25157	Lbp	1
GO:0035970	GO:0035970	peptidyl-threonine dephosphorylation	1/389	20/23328	0.285701929	0.39563	0.25157	Dusp1	1
GO:0044819	GO:0044819	mitotic G1/S transition checkpoint	1/389	20/23328	0.285701929	0.39563	0.25157	Cdkn1a	1
GO:0045623	GO:0045623	negative regulation of T-helper cell differentiation	1/389	20/23328	0.285701929	0.39563	0.25157	Anxa1	1
GO:0045649	GO:0045649	regulation of macrophage differentiation	1/389	20/23328	0.285701929	0.39563	0.25157	Id2	1
GO:0048245	GO:0048245	eosinophil chemotaxis	1/389	20/23328	0.285701929	0.39563	0.25157	Ccl21a	1
GO:0048557	GO:0048557	embryonic digestive tract morphogenesis	1/389	20/23328	0.285701929	0.39563	0.25157	Id2	1
GO:0050965	GO:0050965	detection of temperature stimulus involved in sensory	1/389	20/23328	0.285701929	0.39563	0.25157	Cxcl12	1
GO:0060252	GO:0060252	positive regulation of glial cell proliferation	1/389	20/23328	0.285701929	0.39563	0.25157	Vim	1
GO:0070935	GO:0070935	3'-UTR-mediated mRNA stabilization	1/389	20/23328	0.285701929	0.39563	0.25157	Zfp36	1
GO:0072087	GO:0072087	renal vesicle development	1/389	20/23328	0.285701929	0.39563	0.25157	Bmp4	1
GO:0090030	GO:0090030	regulation of steroid hormone biosynthetic process	1/389	20/23328	0.285701929	0.39563	0.25157	Dab2	1
GO:1900746	GO:1900746	regulation of vascular endothelial growth factor signali	1/389	20/23328	0.285701929	0.39563	0.25157	Dcn	1
GO:1903909	GO:1903909	regulation of receptor clustering	1/389	20/23328	0.285701929	0.39563	0.25157	Ptn	1
GO:1904385	GO:1904385	cellular response to angiotensin	1/389	20/23328	0.285701929	0.39563	0.25157	Cav1	1
GO:2000114	GO:2000114	regulation of establishment of cell polarity	1/389	20/23328	0.285701929	0.39563	0.25157	Cdh5	1
GO:2000678	GO:2000678	negative regulation of transcription regulatory region D	1/389	20/23328	0.285701929	0.39563	0.25157	Id2	1
GO:2000773	GO:2000773	negative regulation of cellular senescence	1/389	20/23328	0.285701929	0.39563	0.25157	Plk2	1
GO:0051282	GO:0051282	regulation of sequestering of calcium ion	3/389	112/23328	0.287142125	0.39749	0.25275	Thy1/Apln	3
GO:0002244	GO:0002244	hematopoietic progenitor cell differentiation	4/389	163/23328	0.289128367	0.39949	0.25402	Flt4/Flt1/B	4
GO:0001658	GO:0001658	branching involved in ureteric bud morphogenesis	2/389	64/23328	0.289175986	0.39949	0.25402	Bmp4/Pgf	2
GO:0007622	GO:0007622	rhythmic behavior	2/389	64/23328	0.289175986	0.39949	0.25402	Id2/Egr1	2
GO:0048008	GO:0048008	platelet-derived growth factor receptor signaling pathw	2/389	64/23328	0.289175986	0.39949	0.25402	Pdgfb/Lox	2
GO:0051784	GO:0051784	negative regulation of nuclear division	2/389	64/23328	0.289175986	0.39949	0.25402	Rgcc/Bmp	2
GO:0090278	GO:0090278	negative regulation of peptide hormone secretion	2/389	64/23328	0.289175986	0.39949	0.25402	Ucp2/Sfrp	2
GO:0051208	GO:0051208	sequestering of calcium ion	3/389	113/23328	0.291685238	0.40282	0.25614	Thy1/Apln	3
GO:0033135	GO:0033135	regulation of peptidyl-serine phosphorylation	4/389	164/23328	0.292856546	0.4043	0.25708	Sfrp2/Cav	4
GO:0042093	GO:0042093	T-helper cell differentiation	2/389	65/23328	0.295354238	0.40566	0.25795	Gadd45g/	2
GO:0042461	GO:0042461	photoreceptor cell development	2/389	65/23328	0.295354238	0.40566	0.25795	Thy1/Pdgf	2
GO:0044774	GO:0044774	mitotic DNA integrity checkpoint	2/389	65/23328	0.295354238	0.40566	0.25795	Ier3/Cdkn	2
GO:0045840	GO:0045840	positive regulation of mitotic nuclear division	2/389	65/23328	0.295354238	0.40566	0.25795	Rgcc/Pdg	2
GO:2000514	GO:2000514	regulation of CD4-positive, alpha-beta T cell activation	2/389	65/23328	0.295354238	0.40566	0.25795	Il2rg/Anxa	2
GO:0050864	GO:0050864	regulation of B cell activation	6/389	270/23328	0.296223885	0.40566	0.25795	Samsn1/Il	6
GO:0034440	GO:0034440	lipid oxidation	3/389	114/23328	0.296233309	0.40566	0.25795	Cd36/C1q	3
GO:0043401	GO:0043401	steroid hormone mediated signaling pathway	3/389	114/23328	0.296233309	0.40566	0.25795	Bmp4/Dal	3
GO:0060078	GO:0060078	regulation of postsynaptic membrane potential	3/389	114/23328	0.296233309	0.40566	0.25795	Reln/Adrb	3
GO:0006606	GO:0006606	protein import into nucleus	4/389	165/23328	0.296591076	0.40566	0.25795	Cd36/Bmp	4
GO:0002363	GO:0002363	alpha-beta T cell lineage commitment	1/389	21/23328	0.297623241	0.40566	0.25795	Ctsl	1
GO:0007190	GO:0007190	activation of adenylate cyclase activity	1/389	21/23328	0.297623241	0.40566	0.25795	Adrb2	1
GO:0007202	GO:0007202	activation of phospholipase C activity	1/389	21/23328	0.297623241	0.40566	0.25795	Htr2b	1
GO:0008210	GO:0008210	estrogen metabolic process	1/389	21/23328	0.297623241	0.40566	0.25795	Cyp1a1	1
GO:0009110	GO:0009110	vitamin biosynthetic process	1/389	21/23328	0.297623241	0.40566	0.25795	Pltp	1
GO:0010447	GO:0010447	response to acidic pH	1/389	21/23328	0.297623241	0.40566	0.25795	Lgmn	1
GO:0014075	GO:0014075	response to amine	1/389	21/23328	0.297623241	0.40566	0.25795	Htr2b	1
GO:0032332	GO:0032332	positive regulation of chondrocyte differentiation	1/389	21/23328	0.297623241	0.40566	0.25795	Loxl2	1
GO:0033605	GO:0033605	positive regulation of catecholamine secretion	1/389	21/23328	0.297623241	0.40566	0.25795	Cxcl12	1
GO:0034315	GO:0034315	regulation of Arp2/3 complex-mediated actin nucleatio	1/389	21/23328	0.297623241	0.40566	0.25795	Gmfg	1
GO:0035089	GO:0035089	establishment of apical/basal cell polarity	1/389	21/23328	0.297623241	0.40566	0.25795	Tcf15	1
GO:0036336	GO:0036336	dendritic cell migration	1/389	21/23328	0.297623241	0.40566	0.25795	Gas6	1

GO:0042053	GO:0042053	regulation of dopamine metabolic process	1/389	21/23328	0.297623241	0.40566	0.25795	Epas1	1
GO:0048569	GO:0048569	post-embryonic animal organ development	1/389	21/23328	0.297623241	0.40566	0.25795	Flt1	1
GO:0050434	GO:0050434	positive regulation of viral transcription	1/389	21/23328	0.297623241	0.40566	0.25795	Jun	1
GO:0050650	GO:0050650	chondroitin sulfate proteoglycan biosynthetic process	1/389	21/23328	0.297623241	0.40566	0.25795	Cytl1	1
GO:0050966	GO:0050966	detection of mechanical stimulus involved in sensory p	1/389	21/23328	0.297623241	0.40566	0.25795	Cxcl12	1
GO:0051023	GO:0051023	regulation of immunoglobulin secretion	1/389	21/23328	0.297623241	0.40566	0.25795	Il33	1
GO:0051956	GO:0051956	negative regulation of amino acid transport	1/389	21/23328	0.297623241	0.40566	0.25795	Rgs4	1
GO:0060088	GO:0060088	auditory receptor cell stereocilium organization	1/389	21/23328	0.297623241	0.40566	0.25795	Clic5	1
GO:0060456	GO:0060456	positive regulation of digestive system process	1/389	21/23328	0.297623241	0.40566	0.25795	Aqp1	1
GO:0072170	GO:0072170	metanephric tubule development	1/389	21/23328	0.297623241	0.40566	0.25795	Aqp1	1
GO:0097107	GO:0097107	postsynaptic density assembly	1/389	21/23328	0.297623241	0.40566	0.25795	Reln	1
GO:0097734	GO:0097734	extracellular exosome biogenesis	1/389	21/23328	0.297623241	0.40566	0.25795	Cd34	1
GO:0098543	GO:0098543	detection of other organism	1/389	21/23328	0.297623241	0.40566	0.25795	Pglyrp1	1
GO:0099515	GO:0099515	actin filament-based transport	1/389	21/23328	0.297623241	0.40566	0.25795	Actn4	1
GO:2000209	GO:2000209	regulation of anoikis	1/389	21/23328	0.297623241	0.40566	0.25795	Cav1	1
GO:2001224	GO:2001224	positive regulation of neuron migration	1/389	21/23328	0.297623241	0.40566	0.25795	Reln	1
GO:0051896	GO:0051896	regulation of protein kinase B signaling	4/389	166/23328	0.30033154	0.40921	0.26021	Igfbp5/Eng	4
GO:0019751	GO:0019751	polyol metabolic process	3/389	115/23328	0.30078562	0.4097	0.26051	Plpp3/Plpp	3
GO:0002294	GO:0002294	CD4-positive, alpha-beta T cell differentiation involved	2/389	66/23328	0.301524638	0.41043	0.26098	Gadd45g/	2
GO:2000736	GO:2000736	regulation of stem cell differentiation	2/389	66/23328	0.301524638	0.41043	0.26098	Ptn/Bmp4	2
GO:0002293	GO:0002293	alpha-beta T cell differentiation involved in immune res	2/389	67/23328	0.307685734	0.4169	0.26509	Gadd45g/	2
GO:0021695	GO:0021695	cerebellar cortex development	2/389	67/23328	0.307685734	0.4169	0.26509	Serpine2/I	2
GO:0003323	GO:0003323	type B pancreatic cell development	1/389	22/23328	0.309346095	0.4169	0.26509	Bmp4	1
GO:0006144	GO:0006144	purine nucleobase metabolic process	1/389	22/23328	0.309346095	0.4169	0.26509	Gda	1
GO:0014912	GO:0014912	negative regulation of smooth muscle cell migration	1/389	22/23328	0.309346095	0.4169	0.26509	Igfbp5	1
GO:0018904	GO:0018904	ether metabolic process	1/389	22/23328	0.309346095	0.4169	0.26509	Ephx2	1
GO:0031649	GO:0031649	heat generation	1/389	22/23328	0.309346095	0.4169	0.26509	Adrb2	1
GO:0032366	GO:0032366	intracellular sterol transport	1/389	22/23328	0.309346095	0.4169	0.26509	Anxa2	1
GO:0032367	GO:0032367	intracellular cholesterol transport	1/389	22/23328	0.309346095	0.4169	0.26509	Anxa2	1
GO:0032616	GO:0032616	interleukin-13 production	1/389	22/23328	0.309346095	0.4169	0.26509	Il33	1
GO:0034377	GO:0034377	plasma lipoprotein particle assembly	1/389	22/23328	0.309346095	0.4169	0.26509	Apoe	1
GO:0043651	GO:0043651	linoleic acid metabolic process	1/389	22/23328	0.309346095	0.4169	0.26509	Ephx2	1
GO:0044851	GO:0044851	hair cycle phase	1/389	22/23328	0.309346095	0.4169	0.26509	Ctsl	1
GO:0045624	GO:0045624	positive regulation of T-helper cell differentiation	1/389	22/23328	0.309346095	0.4169	0.26509	Anxa1	1
GO:0045838	GO:0045838	positive regulation of membrane potential	1/389	22/23328	0.309346095	0.4169	0.26509	Ank3	1
GO:0048261	GO:0048261	negative regulation of receptor-mediated endocytosis	1/389	22/23328	0.309346095	0.4169	0.26509	Sh3gl3	1
GO:0048339	GO:0048339	paraxial mesoderm development	1/389	22/23328	0.309346095	0.4169	0.26509	Tcf15	1
GO:0048532	GO:0048532	anatomical structure arrangement	1/389	22/23328	0.309346095	0.4169	0.26509	Nrp2	1
GO:0051602	GO:0051602	response to electrical stimulus	1/389	22/23328	0.309346095	0.4169	0.26509	Fbxo32	1
GO:0051873	GO:0051873	killing by host of symbiont cells	1/389	22/23328	0.309346095	0.4169	0.26509	Gapdh	1
GO:0060438	GO:0060438	trachea development	1/389	22/23328	0.309346095	0.4169	0.26509	Bmp4	1
GO:0060749	GO:0060749	mammary gland alveolus development	1/389	22/23328	0.309346095	0.4169	0.26509	Id2	1
GO:0061377	GO:0061377	mammary gland lobule development	1/389	22/23328	0.309346095	0.4169	0.26509	Id2	1
GO:0070242	GO:0070242	thymocyte apoptotic process	1/389	22/23328	0.309346095	0.4169	0.26509	Bmp4	1
GO:0072273	GO:0072273	metanephric nephron morphogenesis	1/389	22/23328	0.309346095	0.4169	0.26509	Bmp4	1
GO:1900101	GO:1900101	regulation of endoplasmic reticulum unfolded protein re	1/389	22/23328	0.309346095	0.4169	0.26509	Bok	1
GO:1900543	GO:1900543	negative regulation of purine nucleotide metabolic proc	1/389	22/23328	0.309346095	0.4169	0.26509	Ier3	1
GO:1902547	GO:1902547	regulation of cellular response to vascular endothelial	1/389	22/23328	0.309346095	0.4169	0.26509	Dcn	1
GO:1904996	GO:1904996	positive regulation of leukocyte adhesion to vascular e	1/389	22/23328	0.309346095	0.4169	0.26509	Selp	1
GO:1990776	GO:1990776	response to angiotensin	1/389	22/23328	0.309346095	0.4169	0.26509	Cav1	1
GO:0002286	GO:0002286	T cell activation involved in immune response	3/389	117/23328	0.309900147	0.41709	0.26521	Fgl2/Gadd	3
GO:0006612	GO:0006612	protein targeting to membrane	3/389	117/23328	0.309900147	0.41709	0.26521	Prnp/Rtp4	3
GO:0045621	GO:0045621	positive regulation of lymphocyte differentiation	3/389	117/23328	0.309900147	0.41709	0.26521	Gas6/Il2rg	3
GO:0048814	GO:0048814	regulation of dendrite morphogenesis	3/389	117/23328	0.309900147	0.41709	0.26521	Reln/Ptprf	3
GO:0032874	GO:0032874	positive regulation of stress-activated MAPK cascade	4/389	169/23328	0.311584452	0.41908	0.26648	Flt4/Gadd	4
GO:0051170	GO:0051170	import into nucleus	4/389	169/23328	0.311584452	0.41908	0.26648	Cd36/Bmp	4
GO:0002287	GO:0002287	alpha-beta T cell activation involved in immune respon	2/389	68/23328	0.313836122	0.42058	0.26743	Gadd45g/	2
GO:0015800	GO:0015800	acidic amino acid transport	2/389	68/23328	0.313836122	0.42058	0.26743	Slc6a6/Gj	2
GO:0022029	GO:0022029	telencephalon cell migration	2/389	68/23328	0.313836122	0.42058	0.26743	Reln/Cxcl	2
GO:0032413	GO:0032413	negative regulation of ion transmembrane transporter a	2/389	68/23328	0.313836122	0.42058	0.26743	Ank3/Cav	2
GO:0046635	GO:0046635	positive regulation of alpha-beta T cell activation	2/389	68/23328	0.313836122	0.42058	0.26743	Il2rg/Anxa	2
GO:0046637	GO:0046637	regulation of alpha-beta T cell differentiation	2/389	68/23328	0.313836122	0.42058	0.26743	Il2rg/Anxa	2
GO:0050771	GO:0050771	negative regulation of axonogenesis	2/389	68/23328	0.313836122	0.42058	0.26743	Thy1/Sem	2
GO:0071868	GO:0071868	cellular response to monoamine stimulus	2/389	68/23328	0.313836122	0.42058	0.26743	Rgs4/Htr2	2
GO:0071870	GO:0071870	cellular response to catecholamine stimulus	2/389	68/23328	0.313836122	0.42058	0.26743	Rgs4/Htr2	2
GO:0097194	GO:0097194	execution phase of apoptosis	2/389	68/23328	0.313836122	0.42058	0.26743	Ptgis/Bok	2
GO:0099601	GO:0099601	regulation of neurotransmitter receptor activity	2/389	68/23328	0.313836122	0.42058	0.26743	Reln/Adrb	2
GO:0000187	GO:0000187	activation of MAPK activity	3/389	119/23328	0.319023292	0.42388	0.26953	Dab2/Igfb	3
GO:0019827	GO:0019827	stem cell population maintenance	4/389	171/23328	0.319108619	0.42388	0.26953	Ptn/Loxl2/	4
GO:0070304	GO:0070304	positive regulation of stress-activated protein kinase si	4/389	171/23328	0.319108619	0.42388	0.26953	Flt4/Gadd	4
GO:0010506	GO:0010506	regulation of autophagy	6/389	278/23328	0.319473727	0.42388	0.26953	Adrb2/Iigp	6
GO:0016239	GO:0016239	positive regulation of macroautophagy	2/389	69/23328	0.31997445	0.42388	0.26953	Adrb2/Dcn	2
GO:0031638	GO:0031638	zymogen activation	2/389	69/23328	0.31997445	0.42388	0.26953	Serpine2/I	2
GO:0033627	GO:0033627	cell adhesion mediated by integrin	2/389	69/23328	0.31997445	0.42388	0.26953	Plpp3/Itga	2
GO:0034394	GO:0034394	protein localization to cell surface	2/389	69/23328	0.31997445	0.42388	0.26953	Gpihbp1/F	2
GO:0042058	GO:0042058	regulation of epidermal growth factor receptor signaling	2/389	69/23328	0.31997445	0.42388	0.26953	Ptprf/Cdh1	2
GO:0071867	GO:0071867	response to monoamine	2/389	69/23328	0.31997445	0.42388	0.26953	Rgs4/Htr2	2
GO:0071869	GO:0071869	response to catecholamine	2/389	69/23328	0.31997445	0.42388	0.26953	Rgs4/Htr2	2
GO:0002313	GO:0002313	mature B cell differentiation involved in immune respor	1/389	23/23328	0.320873786	0.42388	0.26953	Lgals1	1
GO:0002320	GO:0002320	lymphoid progenitor cell differentiation	1/389	23/23328	0.320873786	0.42388	0.26953	Bmp4	1
GO:0002827	GO:0002827	positive regulation of T-helper 1 type immune response	1/389	23/23328	0.320873786	0.42388	0.26953	Anxa1	1
GO:0003176	GO:0003176	aortic valve development	1/389	23/23328	0.320873786	0.42388	0.26953	Bmp4	1
GO:0003283	GO:0003283	atrial septum development	1/389	23/23328	0.320873786	0.42388	0.26953	Gja5	1
GO:0010996	GO:0010996	response to auditory stimulus	1/389	23/23328	0.320873786	0.42388	0.26953	Ptn	1
GO:0015812	GO:0015812	gamma-aminobutyric acid transport	1/389	23/23328	0.320873786	0.42388	0.26953	Slc6a6	1
GO:0023019	GO:0023019	signal transduction involved in regulation of gene expr	1/389	23/23328	0.320873786	0.42388	0.26953	Sox17	1
GO:0032288	GO:0032288	myelin assembly	1/389	23/23328	0.320873786	0.42388	0.26953	Cd9	1
GO:0032727	GO:0032727	positive regulation of interferon-alpha production	1/389	23/23328	0.320873786	0.42388	0.26953	Irf7	1
GO:0035278	GO:0035278	miRNA mediated inhibition of translation	1/389	23/23328	0.320873786	0.42388	0.26953	Zfp36	1
GO:0036003	GO:0036003	positive regulation of transcription from RNA polymera	1/389	23/23328	0.320873786	0.42388	0.26953	Klf2	1
GO:0042069	GO:0042069	regulation of catecholamine metabolic process	1/389	23/23328	0.320873786	0.42388	0.26953	Epas1	1
GO:0042537	GO:0042537	benzene-containing compound metabolic process	1/389	23/23328	0.320873786	0.42388	0.26953	Cyp4b1	1
GO:0043369	GO:0043369	CD4-positive or CD8-positive, alpha-beta T cell lineage	1/389	23/23328	0.320873786	0.42388	0.26953	Ctsl	1
GO:0043371	GO:0043371	negative regulation of CD4-positive, alpha-beta T cell (	1/389	23/23328	0.320873786	0.42388	0.26953	Anxa1	1
GO:0043501	GO:0043501	skeletal muscle adaptation	1/389	23/23328	0.320873786	0.42388	0.26953	Igfbp5	1
GO:0045026	GO:0045026	plasma membrane fusion	1/389	23/23328	0.320873786	0.42388	0.26953	Cd9	1
GO:0045606	GO:0045606	positive regulation of epidermal cell differentiation	1/389	23/23328	0.320873786	0.42388	0.26953	Bmp4	1
GO:0045980	GO:0045980	negative regulation of nucleotide metabolic process	1/389	23/23328	0.320873786	0.42388	0.26953	Ier3	1
GO:0046599	GO:0046599	regulation of centriole replication	1/389	23/23328	0.320873786	0.42388	0.26953	Plk2	1
GO:0048485	GO:0048485	sympathetic nervous system development	1/389	23/23328	0.320873786	0.42388	0.26953	Nrp2	1
GO:0050961	GO:0050961	detection of temperature stimulus involved in sensory p	1/389	23/23328	0.320873786	0.42388	0.26953	Cxcl12	1
GO:0061162	GO:0061162	establishment of monopolar cell polarity	1/389	23/23328	0.320873786	0.42388	0.26953	Tcf15	1
GO:0071359	GO:0071359	cellular response to dsRNA	1/389	23/23328	0.320873786	0.42388	0.26953	Cav1	1
GO:0071467	GO:0071467	cellular response to pH	1/389	23/23328	0.320873786	0.42388	0.26953	Gja1	1
GO:0090190	GO:0090190	positive regulation of branching involved in ureteric bu	1/389	23/23328	0.320873786	0.42388	0.26953	Bmp4	1
GO:0097186	GO:0097186	amelogenesis	1/389	23/23328	0.320873786	0.42388	0.26953	Tbx1	1
GO:0101023	GO:0101023	vascular endothelial cell proliferation	1/389	23/23328	0.320873786	0.42388	0.26953	Flt1	1
GO:0140112	GO:0140112	extracellular vesicle biogenesis	1/389	23/23328	0.320873786	0.42388	0.26953	Cd34	1
GO:1905562	GO:1905562	regulation of vascular endothelial cell proliferation	1/389	23/23328	0.320873786	0.42388	0.26953	Flt1	1
GO:2000345	GO:2000345	regulation of hepatocyte proliferation	1/389	23/23328	0.320873786	0.42388	0.26953	Ptn	1
GO:2000831	GO:2000831	regulation of steroid hormone secretion	1/389	23/23328	0.320873786	0.42388	0.26953	Dab2	1
GO:0008654	GO:0008654	phospholipid biosynthetic process	4/389	172/23328	0.322876177	0.42638	0.27112	Fabp5/Adc	4
GO:0006090	GO:0006090	pyruvate metabolic process	3/389	120/23328	0.323586416	0.42718	0.27163	Ier3/Aldoa	3
GO:0019369	GO:0019369	arachidonic acid metabolic process	2/389	70/23328	0.326099412	0.42953	0.27312	Mgll/Ptgs1	2
GO:0030512	GO:0030512	negative regulation of transforming growth factor beta r	2/389	70/23328	0.326099412	0.42953	0.27312	Cav1/Htra	2
GO:0030888	GO:0030888	regulation of B cell proliferation	2/389	70/23328	0.326099412	0.42953	0.27312	Cd38/Cdk	2
GO:0043954	GO:0043954	cellular component maintenance	2/389	70/23328	0.326099412	0.42953	0.27312	Prnp/Apoe	2
GO:0048488	GO:0048488	synaptic vesicle endocytosis	2/389	70/23328	0.326099412	0.42953	0.27312	Sh3gl3/Sn	2
GO:0140238	GO:0140238	presynaptic endocytosis	2/389	70/23328	0.326099412	0.42953	0.27312	Sh3gl3/Sn	2
GO:2000179	GO:2000179	positive regulation of neural precursor cell proliferation	2/389	70/23328	0.326099412	0.42953	0.27312	Prox1/Trf	2
GO:0001829	GO:0001829	trophectodermal cell differentiation	1/389	24/23328	0.332209555	0.43351	0.27566	Junb	1
GO:0003272	GO:0003272	endocardial cushion formation	1/389	24/23328	0.332209555	0.43351	0.27566	Eng	1
GO:0007205	GO:0007205	protein kinase C-activating G protein-coupled receptor	1/389	24/23328	0.332209555	0.43351	0.27566	Htr2b	1
GO:0008090	GO:0008090	retrograde axonal transport	1/389	24/23328	0.332209555	0.43351	0.27566	Dst	1
GO:0022010	GO:0022010	central nervous system myelination	1/389	24/23328	0.332209555	0.43351	0.27566	Clu	1
GO:0030859	GO:0030859	polarized epithelial cell differentiation	1/389	24/23328	0.332209555	0.43351	0.27566	Tcf15	1
GO:0030903	GO:0030903	notochord development	1/389	24/23328	0.332209555	0.43351	0.27566	Id3	1
GO:0031639	GO:0031639	plasminogen activation	1/389	24/23328	0.332209555	0.43351	0.27566	Serpine2	1
GO:0031643	GO:0031643	positive regulation of myelination	1/389	24/23328	0.332209555	0.43351	0.27566	Trf	1

GO:0031954	GO:0031954	positive regulation of protein autophosphorylation	1/389	24/23328	0.332209555	0.43351	0.27566	Pdgfb	1
GO:0032291	GO:0032291	axon ensheathment in central nervous system	1/389	24/23328	0.332209555	0.43351	0.27566	Clu	1
GO:0032691	GO:0032691	negative regulation of interleukin-1 beta production	1/389	24/23328	0.332209555	0.43351	0.27566	Serpinb1a	1
GO:0034505	GO:0034505	tooth mineralization	1/389	24/23328	0.332209555	0.43351	0.27566	Tbx1	1
GO:0035743	GO:0035743	CD4-positive, alpha-beta T cell cytokine production	1/389	24/23328	0.332209555	0.43351	0.27566	Rsad2	1
GO:0040033	GO:0040033	negative regulation of translation, ncRNA-mediated	1/389	24/23328	0.332209555	0.43351	0.27566	Zfp36	1
GO:0042474	GO:0042474	middle ear morphogenesis	1/389	24/23328	0.332209555	0.43351	0.27566	Tbx1	1
GO:0045974	GO:0045974	regulation of translation, ncRNA-mediated	1/389	24/23328	0.332209555	0.43351	0.27566	Zfp36	1
GO:0046823	GO:0046823	negative regulation of nucleocytoplasmic transport	1/389	24/23328	0.332209555	0.43351	0.27566	Cd36	1
GO:0061339	GO:0061339	establishment or maintenance of monopolar cell polari	1/389	24/23328	0.332209555	0.43351	0.27566	Tcf15	1
GO:0062009	GO:0062009	secondary palate development	1/389	24/23328	0.332209555	0.43351	0.27566	Tbx1	1
GO:0070498	GO:0070498	interleukin-1-mediated signaling pathway	1/389	24/23328	0.332209555	0.43351	0.27566	Egr1	1
GO:0071392	GO:0071392	cellular response to estradiol stimulus	1/389	24/23328	0.332209555	0.43351	0.27566	Mmp2	1
GO:0072677	GO:0072677	eosinophil migration	1/389	24/23328	0.332209555	0.43351	0.27566	Ccl21a	1
GO:0097150	GO:0097150	neuronal stem cell population maintenance	1/389	24/23328	0.332209555	0.43351	0.27566	Prox1	1
GO:1903077	GO:1903077	negative regulation of protein localization to plasma me	1/389	24/23328	0.332209555	0.43351	0.27566	Dab2	1
GO:0021885	GO:0021885	forebrain cell migration	2/389	71/23328	0.332209748	0.43351	0.27566	Reln/Cxcl	2
GO:0050909	GO:0050909	sensory perception of taste	2/389	71/23328	0.332209748	0.43351	0.27566	Cd36/Rtp4	2
GO:0055081	GO:0055081	anion homeostasis	2/389	71/23328	0.332209748	0.43351	0.27566	Stc1/Apoe	2
GO:0060675	GO:0060675	ureteric bud morphogenesis	2/389	71/23328	0.332209748	0.43351	0.27566	Bmp4/Pgf	2
GO:0042129	GO:0042129	regulation of T cell proliferation	4/389	175/23328	0.334196067	0.43582	0.27713	Prnp/Bmp	4
GO:0098727	GO:0098727	maintenance of cell number	4/389	175/23328	0.334196067	0.43582	0.27713	Ptn/Loxl2/	4
GO:0030073	GO:0030073	insulin secretion	5/389	229/23328	0.335282655	0.4371	0.27794	Ucp2/Gja1	5
GO:0009132	GO:0009132	nucleoside diphosphate metabolic process	3/389	123/23328	0.33727419	0.43942	0.27941	Ier3/Aldoa	3
GO:0046474	GO:0046474	glycerophospholipid biosynthetic process	3/389	123/23328	0.33727419	0.43942	0.27941	Fabp5/Pd	3
GO:0072171	GO:0072171	mesonephric tubule morphogenesis	2/389	72/23328	0.338304245	0.44034	0.28	Bmp4/Pgf	2
GO:1903845	GO:1903845	negative regulation of cellular response to transforming	2/389	72/23328	0.338304245	0.44034	0.28	Cav1/Htra	2
GO:1904888	GO:1904888	cranial skeletal system development	2/389	72/23328	0.338304245	0.44034	0.28	Tbx1/Bmp	2
GO:0072521	GO:0072521	purine-containing compound metabolic process	8/389	396/23328	0.341390096	0.44379	0.28219	Oasl2/Ier3	8
GO:0099504	GO:0099504	synaptic vesicle cycle	5/389	231/23328	0.34186061	0.44379	0.28219	Sh3gl3/Ac	5
GO:0002093	GO:0002093	auditory receptor cell morphogenesis	1/389	25/23328	0.343356589	0.44379	0.28219	Clic5	1
GO:0007413	GO:0007413	axonal fasciculation	1/389	25/23328	0.343356589	0.44379	0.28219	Ncam1	1
GO:0010640	GO:0010640	regulation of platelet-derived growth factor receptor sig	1/389	25/23328	0.343356589	0.44379	0.28219	Lox	1
GO:0010954	GO:0010954	positive regulation of protein processing	1/389	25/23328	0.343356589	0.44379	0.28219	Spon1	1
GO:0018195	GO:0018195	peptidyl-arginine modification	1/389	25/23328	0.343356589	0.44379	0.28219	Art3	1
GO:0019068	GO:0019068	virion assembly	1/389	25/23328	0.343356589	0.44379	0.28219	Apoe	1
GO:0032753	GO:0032753	positive regulation of interleukin-4 production	1/389	25/23328	0.343356589	0.44379	0.28219	Il33	1
GO:0034114	GO:0034114	regulation of heterotypic cell-cell adhesion	1/389	25/23328	0.343356589	0.44379	0.28219	Thy1	1
GO:0044003	GO:0044003	modulation by symbiont of host process	1/389	25/23328	0.343356589	0.44379	0.28219	Gapdh	1
GO:0048266	GO:0048266	behavioral response to pain	1/389	25/23328	0.343356589	0.44379	0.28219	Vwa1	1
GO:0051883	GO:0051883	killing of cells in other organism involved in symbiotic i	1/389	25/23328	0.343356589	0.44379	0.28219	Gapdh	1
GO:0060004	GO:0060004	reflex	1/389	25/23328	0.343356589	0.44379	0.28219	Gja1	1
GO:0060216	GO:0060216	definitive hemopoiesis	1/389	25/23328	0.343356589	0.44379	0.28219	Mfap5	1
GO:0061213	GO:0061213	positive regulation of mesonephros development	1/389	25/23328	0.343356589	0.44379	0.28219	Bmp4	1
GO:0070633	GO:0070633	transepithelial transport	1/389	25/23328	0.343356589	0.44379	0.28219	Aqp1	1
GO:0071880	GO:0071880	adenylate cyclase-activating adrenergic receptor signa	1/389	25/23328	0.343356589	0.44379	0.28219	Adrb2	1
GO:0086019	GO:0086019	cell-cell signaling involved in cardiac conduction	1/389	25/23328	0.343356589	0.44379	0.28219	Gja5	1
GO:0090169	GO:0090169	regulation of spindle assembly	1/389	25/23328	0.343356589	0.44379	0.28219	Hspa1a	1
GO:0106030	GO:0106030	neuron projection fasciculation	1/389	25/23328	0.343356589	0.44379	0.28219	Ncam1	1
GO:1903798	GO:1903798	regulation of production of miRNAs involved in gene si	1/389	25/23328	0.343356589	0.44379	0.28219	Bmp4	1
GO:0009988	GO:0009988	cell-cell recognition	2/389	73/23328	0.344381735	0.44484	0.28286	Cd9/Aldoa	2
GO:0042130	GO:0042130	negative regulation of T cell proliferation	2/389	73/23328	0.344381735	0.44484	0.28286	Prnp/Bmp	2
GO:0042594	GO:0042594	response to starvation	4/389	178/23328	0.345533762	0.44618	0.28371	Gas6/Zfp3	4
GO:0043280	GO:0043280	positive regulation of cysteine-type endopeptidase acti	3/389	125/23328	0.346391749	0.44715	0.28433	Bok/S100a	3
GO:0007422	GO:0007422	peripheral nervous system development	2/389	74/23328	0.35044109	0.45195	0.28738	Hoxd9/Nd	2
GO:0034404	GO:0034404	nucleobase-containing small molecule biosynthetic pro	2/389	74/23328	0.35044109	0.45195	0.28738	Nudt4/Pde	2
GO:0046824	GO:0046824	positive regulation of nucleocytoplasmic transport	2/389	74/23328	0.35044109	0.45195	0.28738	Gas6/Tek	2
GO:0051053	GO:0051053	negative regulation of DNA metabolic process	3/389	126/23328	0.350946412	0.45245	0.2877	Gja1/Dusp	3
GO:0006091	GO:0006091	generation of precursor metabolites and energy	8/389	400/23328	0.351392134	0.45289	0.28798	Cox4i2/Ac	8
GO:0000075	GO:0000075	cell cycle checkpoint	4/389	180/23328	0.353097139	0.45293	0.288	Ier3/Prox1	4
GO:0001780	GO:0001780	neutrophil homeostasis	1/389	26/23328	0.354318023	0.45293	0.288	Anxa1	1
GO:0007620	GO:0007620	copulation	1/389	26/23328	0.354318023	0.45293	0.288	Serpine2	1
GO:0009713	GO:0009713	catechol-containing compound biosynthetic process	1/389	26/23328	0.354318023	0.45293	0.288	Epas1	1
GO:0010575	GO:0010575	positive regulation of vascular endothelial growth facto	1/389	26/23328	0.354318023	0.45293	0.288	Flt4	1
GO:0010592	GO:0010592	positive regulation of lamellipodium assembly	1/389	26/23328	0.354318023	0.45293	0.288	Aqp1	1
GO:0010829	GO:0010829	negative regulation of glucose transmembrane transpo	1/389	26/23328	0.354318023	0.45293	0.288	Fabp5	1
GO:0021801	GO:0021801	cerebral cortex radial glia guided migration	1/389	26/23328	0.354318023	0.45293	0.288	Reln	1
GO:0022030	GO:0022030	telencephalon glial cell migration	1/389	26/23328	0.354318023	0.45293	0.288	Reln	1
GO:0031100	GO:0031100	animal organ regeneration	1/389	26/23328	0.354318023	0.45293	0.288	Cxcl12	1
GO:0035929	GO:0035929	steroid hormone secretion	1/389	26/23328	0.354318023	0.45293	0.288	Dab2	1
GO:0042423	GO:0042423	catecholamine biosynthetic process	1/389	26/23328	0.354318023	0.45293	0.288	Epas1	1
GO:0045723	GO:0045723	positive regulation of fatty acid biosynthetic process	1/389	26/23328	0.354318023	0.45293	0.288	Anxa1	1
GO:0046339	GO:0046339	diacylglycerol metabolic process	1/389	26/23328	0.354318023	0.45293	0.288	Plpp1	1
GO:0051788	GO:0051788	response to misfolded protein	1/389	26/23328	0.354318023	0.45293	0.288	Clu	1
GO:0060065	GO:0060065	uterus development	1/389	26/23328	0.354318023	0.45293	0.288	Cdkn1c	1
GO:0060338	GO:0060338	regulation of type I interferon-mediated signaling pathw	1/389	26/23328	0.354318023	0.45293	0.288	Irf7	1
GO:0065005	GO:0065005	protein-lipid complex assembly	1/389	26/23328	0.354318023	0.45293	0.288	Apoe	1
GO:0070233	GO:0070233	negative regulation of T cell apoptotic process	1/389	26/23328	0.354318023	0.45293	0.288	Bmp4	1
GO:0070920	GO:0070920	regulation of production of small RNA involved in gene	1/389	26/23328	0.354318023	0.45293	0.288	Bmp4	1
GO:0071474	GO:0071474	cellular hyperosmotic response	1/389	26/23328	0.354318023	0.45293	0.288	Aqp1	1
GO:0090189	GO:0090189	regulation of branching involved in ureteric bud morpho	1/389	26/23328	0.354318023	0.45293	0.288	Bmp4	1
GO:0090344	GO:0090344	negative regulation of cell aging	1/389	26/23328	0.354318023	0.45293	0.288	Plk2	1
GO:0140058	GO:0140058	neuron projection arborization	1/389	26/23328	0.354318023	0.45293	0.288	Ptn	1
GO:1902175	GO:1902175	regulation of oxidative stress-induced intrinsic apoptoti	1/389	26/23328	0.354318023	0.45293	0.288	Hspb1	1
GO:1904376	GO:1904376	negative regulation of protein localization to cell periph	1/389	26/23328	0.354318023	0.45293	0.288	Dab2	1
GO:0051101	GO:0051101	regulation of DNA binding	3/389	127/23328	0.355497541	0.45415	0.28878	Id1/Id2/Ju	3
GO:0060996	GO:0060996	dendritic spine development	3/389	127/23328	0.355497541	0.45415	0.28878	Reln/Apoe	3
GO:0032642	GO:0032642	regulation of chemokine production	2/389	75/23328	0.356481227	0.45498	0.28931	Lbp/Lpl	2
GO:0046626	GO:0046626	regulation of insulin receptor signaling pathway	2/389	75/23328	0.356481227	0.45498	0.28931	Ptprf/Socs	2
GO:0048645	GO:0048645	animal organ formation	2/389	75/23328	0.356481227	0.45498	0.28931	Tbx1/Bmp	2
GO:0061013	GO:0061013	regulation of mRNA catabolic process	3/389	128/23328	0.360044558	0.45939	0.29211	Vim/Btg2/	3
GO:0048813	GO:0048813	dendrite morphogenesis	4/389	182/23328	0.360661052	0.46003	0.29252	Reln/Ptn/F	4
GO:0008543	GO:0008543	fibroblast growth factor receptor signaling pathway	2/389	76/23328	0.362501102	0.46064	0.29291	Pdgfb/Flrt	2
GO:0045670	GO:0045670	regulation of osteoclast differentiation	2/389	76/23328	0.362501102	0.46064	0.29291	Sfrp1/Fos	2
GO:0016331	GO:0016331	morphogenesis of embryonic epithelium	4/389	183/23328	0.36444232	0.46064	0.29291	Bmp4/Sfrp	4
GO:0046434	GO:0046434	organophosphate catabolic process	3/389	129/23328	0.364586896	0.46064	0.29291	Scarb1/Nu	3
GO:0002068	GO:0002068	glandular epithelial cell development	1/389	27/23328	0.365096939	0.46064	0.29291	Bmp4	1
GO:0003171	GO:0003171	atrioventricular valve development	1/389	27/23328	0.365096939	0.46064	0.29291	Gja5	1
GO:0003338	GO:0003338	metanephros morphogenesis	1/389	27/23328	0.365096939	0.46064	0.29291	Bmp4	1
GO:0006925	GO:0006925	inflammatory cell apoptotic process	1/389	27/23328	0.365096939	0.46064	0.29291	Anxa1	1
GO:0014821	GO:0014821	phasic smooth muscle contraction	1/389	27/23328	0.365096939	0.46064	0.29291	Htr2b	1
GO:0015874	GO:0015874	norepinephrine transport	1/389	27/23328	0.365096939	0.46064	0.29291	Ptgs1	1
GO:0016048	GO:0016048	detection of temperature stimulus	1/389	27/23328	0.365096939	0.46064	0.29291	Cxcl12	1
GO:0021511	GO:0021511	spinal cord patterning	1/389	27/23328	0.365096939	0.46064	0.29291	Reln	1
GO:0021871	GO:0021871	forebrain regionalization	1/389	27/23328	0.365096939	0.46064	0.29291	Bmp4	1
GO:0030212	GO:0030212	hyaluronan metabolic process	1/389	27/23328	0.365096939	0.46064	0.29291	Pdgfb	1
GO:0030970	GO:0030970	retrograde protein transport, ER to cytosol	1/389	27/23328	0.365096939	0.46064	0.29291	Hsp90b1	1
GO:0032069	GO:0032069	regulation of nuclease activity	1/389	27/23328	0.365096939	0.46064	0.29291	Oasl2	1
GO:0039528	GO:0039528	cytoplasmic pattern recognition receptor signaling path	1/389	27/23328	0.365096939	0.46064	0.29291	Tspan6	1
GO:0042059	GO:0042059	negative regulation of epidermal growth factor recepto	1/389	27/23328	0.365096939	0.46064	0.29291	Ptprf	1
GO:0042908	GO:0042908	xenobiotic transport	1/389	27/23328	0.365096939	0.46064	0.29291	Slc6a6	1
GO:0045730	GO:0045730	respiratory burst	1/389	27/23328	0.365096939	0.46064	0.29291	Lbp	1
GO:0046639	GO:0046639	negative regulation of alpha-beta T cell differentiation	1/389	27/23328	0.365096939	0.46064	0.29291	Anxa1	1
GO:0048305	GO:0048305	immunoglobulin secretion	1/389	27/23328	0.365096939	0.46064	0.29291	Il33	1
GO:0048843	GO:0048843	negative regulation of axon extension involved in axon	1/389	27/23328	0.365096939	0.46064	0.29291	Sema3g	1
GO:0050849	GO:0050849	negative regulation of calcium-mediated signaling	1/389	27/23328	0.365096939	0.46064	0.29291	Prnp	1
GO:0060045	GO:0060045	positive regulation of cardiac muscle cell proliferation	1/389	27/23328	0.365096939	0.46064	0.29291	Ncam1	1
GO:0060716	GO:0060716	labyrinthine layer blood vessel development	1/389	27/23328	0.365096939	0.46064	0.29291	Junb	1
GO:0061003	GO:0061003	positive regulation of dendritic spine morphogenesis	1/389	27/23328	0.365096939	0.46064	0.29291	Reln	1
GO:0071472	GO:0071472	cellular response to salt stress	1/389	27/23328	0.365096939	0.46064	0.29291	Aqp1	1
GO:0090023	GO:0090023	positive regulation of neutrophil chemotaxis	1/389	27/23328	0.365096939	0.46064	0.29291	Lbp	1
GO:0090103	GO:0090103	cochlea morphogenesis	1/389	27/23328	0.365096939	0.46064	0.29291	Tbx1	1
GO:0150077	GO:0150077	regulation of neuroinflammatory response	1/389	27/23328	0.365096939	0.46064	0.29291	Cd200	1
GO:1901018	GO:1901018	positive regulation of potassium ion transmembrane tra	1/389	27/23328	0.365096939	0.46064	0.29291	Atp1b1	1
GO:1903010	GO:1903010	regulation of bone development	1/389	27/23328	0.365096939	0.46064	0.29291	Lox	1
GO:1903319	GO:1903319	positive regulation of protein maturation	1/389	27/23328	0.365096939	0.46064	0.29291	Spon1	1
GO:1903513	GO:1903513	endoplasmic reticulum to cytosol transport	1/389	27/23328	0.365096939	0.46064	0.29291	Hsp90b1	1
GO:0010822	GO:0010822	positive regulation of mitochondrion organization	2/389	77/23328	0.368499714	0.46421	0.29518	Bok/Dcn	2
GO:0030433	GO:0030433	ubiquitin-dependent ERAD pathway	2/389	77/23328	0.368499714	0.46421	0.29518	Cav1/Hsp	2

GO:0032410	GO:0032410	negative regulation of transporter activity	2/389	77/23328	0.368499714	0.46421	0.29518	Ank3/Cav	2
GO:0033143	GO:0033143	regulation of intracellular steroid hormone receptor sigr	2/389	77/23328	0.368499714	0.46421	0.29518	Dab2/Sfrp	2
GO:0043507	GO:0043507	positive regulation of JUN kinase activity	2/389	77/23328	0.368499714	0.46421	0.29518	Dab2/Trf	2
GO:0009749	GO:0009749	response to glucose	4/389	185/23328	0.372001732	0.46848	0.29789	Ucp2/Vca	4
GO:0007093	GO:0007093	mitotic cell cycle checkpoint	3/389	131/23328	0.37365532	0.46864	0.29799	Ier3/Plk2/(	3
GO:0002066	GO:0002066	columnar/cuboidal epithelial cell development	2/389	78/23328	0.374476098	0.46864	0.29799	Bmp4/Clic	2
GO:0014855	GO:0014855	striated muscle cell proliferation	2/389	78/23328	0.374476098	0.46864	0.29799	Ncam1/Gj	2
GO:0032088	GO:0032088	negative regulation of NF-kappaB transcription factor a	2/389	78/23328	0.374476098	0.46864	0.29799	Cd200/Ptc	2
GO:0072332	GO:0072332	intrinsic apoptotic signaling pathway by p53 class med	2/389	78/23328	0.374476098	0.46864	0.29799	Bok/Cdkn	2
GO:0008340	GO:0008340	determination of adult lifespan	1/389	28/23328	0.375696367	0.46864	0.29799	Meg3	1
GO:0010155	GO:0010155	regulation of proton transport	1/389	28/23328	0.375696367	0.46864	0.29799	Slc25a4	1
GO:0032469	GO:0032469	endoplasmic reticulum calcium ion homeostasis	1/389	28/23328	0.375696367	0.46864	0.29799	Tgm2	1
GO:0032647	GO:0032647	regulation of interferon-alpha production	1/389	28/23328	0.375696367	0.46864	0.29799	Irf7	1
GO:0033233	GO:0033233	regulation of protein sumoylation	1/389	28/23328	0.375696367	0.46864	0.29799	Egr1	1
GO:0034390	GO:0034390	smooth muscle cell apoptotic process	1/389	28/23328	0.375696367	0.46864	0.29799	Gapdh	1
GO:0034391	GO:0034391	regulation of smooth muscle cell apoptotic process	1/389	28/23328	0.375696367	0.46864	0.29799	Gapdh	1
GO:0035987	GO:0035987	endodermal cell differentiation	1/389	28/23328	0.375696367	0.46864	0.29799	Sox17	1
GO:0045648	GO:0045648	positive regulation of erythrocyte differentiation	1/389	28/23328	0.375696367	0.46864	0.29799	Id2	1
GO:0045663	GO:0045663	positive regulation of myoblast differentiation	1/389	28/23328	0.375696367	0.46864	0.29799	Cxcl9	1
GO:0048596	GO:0048596	embryonic camera-type eye morphogenesis	1/389	28/23328	0.375696367	0.46864	0.29799	Prox1	1
GO:0048820	GO:0048820	hair follicle maturation	1/389	28/23328	0.375696367	0.46864	0.29799	Ctsl	1
GO:0051016	GO:0051016	barbed-end actin filament capping	1/389	28/23328	0.375696367	0.46864	0.29799	Capg	1
GO:0051560	GO:0051560	mitochondrial calcium ion homeostasis	1/389	28/23328	0.375696367	0.46864	0.29799	Tgm2	1
GO:0061217	GO:0061217	regulation of mesonephros development	1/389	28/23328	0.375696367	0.46864	0.29799	Bmp4	1
GO:0061436	GO:0061436	establishment of skin barrier	1/389	28/23328	0.375696367	0.46864	0.29799	Cyp26b1	1
GO:0071875	GO:0071875	adrenergic receptor signaling pathway	1/389	28/23328	0.375696367	0.46864	0.29799	Adrb2	1
GO:0072395	GO:0072395	signal transduction involved in cell cycle checkpoint	1/389	28/23328	0.375696367	0.46864	0.29799	Cdkn1a	1
GO:0072401	GO:0072401	signal transduction involved in DNA integrity checkpoir	1/389	28/23328	0.375696367	0.46864	0.29799	Cdkn1a	1
GO:0072422	GO:0072422	signal transduction involved in DNA damage checkpoi	1/389	28/23328	0.375696367	0.46864	0.29799	Cdkn1a	1
GO:1900745	GO:1900745	positive regulation of p38MAPK cascade	1/389	28/23328	0.375696367	0.46864	0.29799	Gadd45g	1
GO:1901532	GO:1901532	regulation of hematopoietic progenitor cell differentiatic	1/389	28/23328	0.375696367	0.46864	0.29799	Flt1	1
GO:1902668	GO:1902668	negative regulation of axon guidance	1/389	28/23328	0.375696367	0.46864	0.29799	Sema3g	1
GO:1903649	GO:1903649	regulation of cytoplasmic transport	1/389	28/23328	0.375696367	0.46864	0.29799	Dab2	1
GO:1904861	GO:1904861	excitatory synapse assembly	1/389	28/23328	0.375696367	0.46864	0.29799	Reln	1
GO:2000310	GO:2000310	regulation of NMDA receptor activity	1/389	28/23328	0.375696367	0.46864	0.29799	Reln	1
GO:0010256	GO:0010256	endomembrane system organization	8/389	410/23328	0.376539256	0.46955	0.29857	Srgn/Serp	8
GO:0006163	GO:0006163	purine nucleotide metabolic process	7/389	354/23328	0.377847006	0.47104	0.29952	Oasl2/Ier3	7
GO:0009416	GO:0009416	response to light stimulus	6/389	298/23328	0.378532631	0.47175	0.29997	Nts/Mfap4	6
GO:0019439	GO:0019439	aromatic compound catabolic process	8/389	411/23328	0.379062098	0.47226	0.3003	Ephx2/Vin	8
GO:0022412	GO:0022412	cellular process involved in reproduction in multicellula	9/389	468/23328	0.379574994	0.47276	0.30061	Cd9/Ptn/C	9
GO:0032602	GO:0032602	chemokine production	2/389	79/23328	0.380429327	0.47324	0.30092	Lbp/Lpl	2
GO:0045471	GO:0045471	response to ethanol	2/389	79/23328	0.380429327	0.47324	0.30092	Adh7/Adh	2
GO:0051279	GO:0051279	regulation of release of sequestered calcium ion into c	2/389	79/23328	0.380429327	0.47324	0.30092	Thy1/Apln	2
GO:0051966	GO:0051966	regulation of synaptic transmission, glutamatergic	2/389	79/23328	0.380429327	0.47324	0.30092	Reln/Serp	2
GO:0009746	GO:0009746	response to hexose	4/389	188/23328	0.383327935	0.47656	0.30303	Ucp2/Vca	4
GO:0031669	GO:0031669	cellular response to nutrient levels	4/389	188/23328	0.383327935	0.47656	0.30303	Gas6/Jun/	4
GO:0010893	GO:0010893	positive regulation of steroid biosynthetic process	1/389	29/23328	0.386119291	0.47756	0.30366	Dab2	1
GO:0030318	GO:0030318	melanocyte differentiation	1/389	29/23328	0.386119291	0.47756	0.30366	Kitl	1
GO:0033198	GO:0033198	response to ATP	1/389	29/23328	0.386119291	0.47756	0.30366	Hsp90b1	1
GO:0034629	GO:0034629	cellular protein-containing complex localization	1/389	29/23328	0.386119291	0.47756	0.30366	Exoc3l2	1
GO:0035094	GO:0035094	response to nicotine	1/389	29/23328	0.386119291	0.47756	0.30366	B2m	1
GO:0045109	GO:0045109	intermediate filament organization	1/389	29/23328	0.386119291	0.47756	0.30366	Vim	1
GO:0060571	GO:0060571	morphogenesis of an epithelial fold	1/389	29/23328	0.386119291	0.47756	0.30366	Bmp4	1
GO:0071624	GO:0071624	positive regulation of granulocyte chemotaxis	1/389	29/23328	0.386119291	0.47756	0.30366	Lbp	1
GO:0090140	GO:0090140	regulation of mitochondrial fission	1/389	29/23328	0.386119291	0.47756	0.30366	Dcn	1
GO:1900027	GO:1900027	regulation of ruffle assembly	1/389	29/23328	0.386119291	0.47756	0.30366	Cav1	1
GO:1900078	GO:1900078	positive regulation of cellular response to insulin stimu	1/389	29/23328	0.386119291	0.47756	0.30366	C1qtnf9	1
GO:1902235	GO:1902235	regulation of endoplasmic reticulum stress-induced intr	1/389	29/23328	0.386119291	0.47756	0.30366	Bok	1
GO:1904292	GO:1904292	regulation of ERAD pathway	1/389	29/23328	0.386119291	0.47756	0.30366	Cav1	1
GO:0008088	GO:0008088	axo-dendritic transport	2/389	80/23328	0.386358512	0.47756	0.30366	Hspb1/Ds	2
GO:0032465	GO:0032465	regulation of cytokinesis	2/389	80/23328	0.386358512	0.47756	0.30366	Plk2/Pkp4	2
GO:0036465	GO:0036465	synaptic vesicle recycling	2/389	80/23328	0.386358512	0.47756	0.30366	Sh3gl3/Sr	2
GO:0048041	GO:0048041	focal adhesion assembly	2/389	80/23328	0.386358512	0.47756	0.30366	Thy1/Tek	2
GO:0060968	GO:0060968	regulation of gene silencing	2/389	80/23328	0.386358512	0.47756	0.30366	Bmp4/Zfp	2
GO:0072078	GO:0072078	nephron tubule morphogenesis	2/389	80/23328	0.386358512	0.47756	0.30366	Bmp4/Pgf	2
GO:0034284	GO:0034284	response to monosaccharide	4/389	189/23328	0.387098557	0.47832	0.30415	Ucp2/Vca	4
GO:0009791	GO:0009791	post-embryonic development	3/389	134/23328	0.387209287	0.47832	0.30415	Flt1/Bmp4	3
GO:0050796	GO:0050796	regulation of insulin secretion	4/389	190/23328	0.390866246	0.48269	0.30693	Ucp2/Gja1	4
GO:0010389	GO:0010389	regulation of G2/M transition of mitotic cell cycle	2/389	81/23328	0.3922628	0.48383	0.30765	Ier3/Cdkn	2
GO:0045682	GO:0045682	regulation of epidermis development	2/389	81/23328	0.3922628	0.48383	0.30765	Bmp4/Zfp	2
GO:0051785	GO:0051785	positive regulation of nuclear division	2/389	81/23328	0.3922628	0.48383	0.30765	Rgcc/Pdg	2
GO:0071347	GO:0071347	cellular response to interleukin-1	2/389	81/23328	0.3922628	0.48383	0.30765	Ccl21a/Eg	2
GO:0042113	GO:0042113	B cell activation	8/389	417/23328	0.394217309	0.48609	0.30909	Adgrg3/Sa	8
GO:0001759	GO:0001759	organ induction	1/389	30/23328	0.39636864	0.48653	0.30937	Bmp4	1
GO:0002335	GO:0002335	mature B cell differentiation	1/389	30/23328	0.39636864	0.48653	0.30937	Lgals1	1
GO:0002360	GO:0002360	T cell lineage commitment	1/389	30/23328	0.39636864	0.48653	0.30937	Ctsl	1
[truncated: 2,750,302 more chars]
